# Identification of endoplasmic reticulum stress-associated lncRNAs influencing inflammation and VSMC function in abdominal aortic aneurysm

**DOI:** 10.1042/CS20242476

**Published:** 2025-03-25

**Authors:** Rafael Almendra-Pegueros, Cristina Rodriguez, Mercedes Camacho, David Sánchez-Infantes, J. Luis Sánchez-Quesada, Susana Cáncer, Elvira Pérez-Marlasca, Gema Medina-Gómez, José Martinez-González, Ana B. García-Redondo, María Galán

**Affiliations:** 1 Institut de Recerca Sant Pau (IR Sant Pau), Barcelona, Spain; 2 Centro de Investigación Biomédica en Red de Enfermedades Cardiovasculares (CIBERCV), Instituto de Salud Carlos III (ISCIII), Madrid, Spain; 3 Departamento de Ciencias Básicas de la Salud, Facultad de Ciencias de la Salud, Universidad Rey Juan Carlos, Alcorcón, Madrid, Spain; 4 Centro de Investigación Biomédica en Red de Fisiopatología de la Obesidad y Nutrición (CIBERobn), Instituto de Salud Carlos III (ISCIII), Madrid, Spain; 5 Centro de Investigación Biomédica en Red de Diabetes y Enfermedades Metabólicas (CIBERDEM), Instituto de Salud Carlos III (ISCIII), Madrid, Spain; 6 Unidad de Angiología y Cirugía Vascular, Hospital Universitario Fundación de Alcorcón, Alcorcón, Madrid, Spain; 7 Instituto de Investigaciones Biomédicas de Barcelona-Consejo Superior de Investigaciones Científicas (IIBB-CSIC), Barcelona, Spain; 8 Departamento de Fisiología, Facultad de Medicina, Universidad Autónoma de Madrid, Madrid, Spain

**Keywords:** aneurysm, aorta, endoplasmic reticulum stress, long non-coding RNA

## Abstract

Endoplasmic reticulum (ER) stress plays a critical role in the abdominal aortic aneurysm (AAA), a life-threatening disease characterized by inflammation, destructive remodeling, and vascular smooth muscle cells (VSMCs) dysfunction. The current therapy relies on surgical repair, but no effective pharmacological strategies are available to limit aneurysm progression. Long non-coding RNAs (lncRNAs) are essential factors in health and disease; however, their specific contribution to AAA development and its relationship with ER stress remain unexplored. Here, we have performed a whole-genome transcriptomic analysis characterizing the expression profile of lncRNAs in AAA. RNA sequencing was carried out in abdominal aorta from patients with AAA and healthy donors. We identified 6576 differentially expressed (DE)-mRNAs and 1283 DE-lncRNAs. Interestingly, bioinformatic analysis revealed a set of 368 DE-lncRNAs related to ER stress. The differential expression of the most induced lncRNAs (IL-21-AS1, ITPKB-IT, PCED1B-AS1, TCL-6, LINC00494, LINC00582, LINC00626, LINC00861, and LINC00892) was validated in a large cohort of patients with AAA. The ability of these selected lncRNAs to discriminate patients with AAA from healthy subjects was established by receiveroperating characteristic curves and logistic regression analysis. In human aortic VSMC and Jurkat T-cells, tunicamycin-induced ER stress triggered the expression of IL21-AS1, LINC00626, LINC00494, LINC00892, PCED1B-AS1, ITPKB-IT, and TCL-6, while tauroursodeoxycholic acid counteracted these effects. Finally, an integrated analysis of mRNA-lncRNA co-expression revealed the correlation between the selected lncRNAs and the DE-mRNAs involved in immune response and muscle contraction. Therefore, these DE-lncRNAs potentially implicated in the ER stress response, a pathological process in AAA, could be considered as potential therapeutic target to handle AAA.

## Introduction

Abdominal aortic aneurysm (AAA) is an age-related degenerative disorder with high morbidity and mortality and is commonly asymptomatic [1–3]. It is defined as a severe pathological condition in which the abdominal aorta enlarges reaching aortic diameter values >3.0 cm. In subjects aged over 65 years, the prevalence of AAAs is estimated between 2% and 6%, depending on the population under study, and AAA rupture is the most severe consequence of this disease, being a worldwide leading cause of death [3,4]. Although the risk of rupture increases exponentially as the aortic diameter grows, aneurysm size is not always a good predictor since small aneurysms occasionally rupture and a proportion of large AAA remains stable over time [4–6]. In this context, the management of these patients consists of watchful observation, being aneurysm diameter as the principal surrogate marker for disease progression and rupture, and the main determinant for surgical repair, the only therapeutic option for this disorder [7]. Therefore, effective therapeutic approaches able to halt the expansion of AAA are urgently needed. Potential drug candidates previously assessed in clinical trials did not show a clear therapeutic effect, and even in some cases, they could lead to a paradoxical worse progression with faster AAA growth [8–10]. Consequently, the development of therapeutic strategies for slowing AAA expansion and providing better clinical outcomes requires a deep understanding of AAA pathogenesis.

AAA is a complex disease involving various risk factors, such as smoking, aging, male sex, history of AAA in first-degree relatives, atherosclerotic disease, and hypertension [3–5]. The pathogenic mechanisms underlying the destructive vascular remodeling characteristic of this disease entail protease activation, extracellular matrix (ECM) degradation, vascular smooth muscle cells (VSMCs) phenotypic switch and death, and extensive medial and adventitial degeneration combined with an exacerbated inflammatory response [11–13]. Interestingly, we and others have reported the critical role of endoplasmic reticulum (ER) stress in the pathogenesis of several cardiovascular diseases including hypertension, atherosclerosis, heart failure, and AAA [14–16]. Environmental factors related to aneurysmal disease, such as hypoxia, reactive oxygen species, aging, and genetic factors, disrupt ER function leading to an accumulation of misfolded and unfolded proteins in the ER lumen, which promotes ER stress. When ER stress occurs, the unfolding protein response (UPR) is activated, initiating adaptive responses for maintaining cellular homeostasis. However, UPR is a double-edged sword as temporary activation can restore ER homeostasis, but, if the ER stress is prolonged, the homeostatic capacity of the adaptive UPR becomes saturated and the UPR response is dominated by the maladaptive process leading to apoptosis [17–19].

Noncoding RNAs have been gaining much attention as epigenetic regulators in cardiovascular diseases. Among them, long noncoding RNAs (lncRNAs) stand out as essential factors in health and disease with a significant, but not yet clearly defined, role in vascular physiology. Accumulating evidence supports the influence of lncRNAs in the regulation of vascular function and inflammation [20–23]. However, studies focused on lncRNAs and AAA are still scarce, and their specific contribution to aneurysm disease development and progression remains largely unexplored [23,24], particularly concerning ER stress. In the present study, we have performed a whole-genome transcriptomic analysis characterizing the expression profile of lncRNAs in AAA, identifying a signature of ER-stress-associated lncRNAs, most of which are also associated with mitochondrial function, that may be useful for the development of new pharmacological interventions in AAA.

## Materials and methods

### Human samples

Human abdominal aneurysmal aortas were obtained from patients diagnosed with infrarenal AAA and undergoing open surgical repair at the Hospital de la Santa Creu i Sant Pau (HSCSP; Barcelona, Spain). The diagnosis of AAA was confirmed by computed tomography scan. Patients with AAA and with negative histories of rheumatological, immunological diseases, aortitis, or genetic syndromes such as Marfan disease were included in the study. Other exclusion criteria were juxtarenal aneurysms and mycotic aneurysms. Healthy abdominal aortas came from multiorgan donors. Approval to use the discarded human tissue was given by the Ethics Committee of the HSCSP, and the participation in the study of patients and controls was based on the informed consent of patients or legal representatives. Research was conducted in accordance with the Declaration of Helsinki of 1975. Abdominal aorta segments were obtained according to standard operating procedures and ethical guidelines. Samples of control subjects had no postmortem evidence of AAA. For RNA studies, parts of the tissue samples were collected, snap-frozen, and stored at −80°C for subsequent RNA extraction.

### Cell culture

Human VSMCs were isolated from the aorta of multiorgan donors by an explant procedure as previously described [16]. Endothelium-denuded medial tissue was cut into 2–4-mm cubes that were transferred to a 23-cm^2^ culture flask containing 5 ml of prewarmed culture medium M199 (Gibco, Carslbad, Cam U.S.A.) supplemented with 10% fetal calf serum (FCS; Biological Industries, Kibbutz Beit-Haemek, Israel) and antibiotics (100 U/ml penicillin and 0.1 mg/ml streptomycin). VSMCs migrate out from the explants within two to three weeks. Then, after removing the explants from the flask surface, cells were trypsinized, used as P1 stage cells, and routinely subcultured. Human VSMCs were cultured in M199 supplemented with 20% FCS, 2% human serum, 2 mmol/l L-glutamine (Invitrogen), and antibiotics incubated at 37°C in a 5% CO_2_-humidified incubator.

For experimental procedures, cells between passages 3 and 6 were seeded in six-multiwell plates. Subconfluent cells were serum-starved in a medium supplemented with 1% FCS for 24 h prior to the addition of 1 µg/ml of tunicamycin (Sigma-Aldrich) or vehicle (dimethyl sulfoxide, DMSO, Sigma-Aldrich) for 8 h. In some experiments, cells were pretreated with the ER stress inhibitor tauroursodeoxycholic acid (TUDCA; 500 µg/ml), 1 h prior to exposure to tunicamycin [25].

The human T-acute lymphoblastic leukemia Jurkat cell line was purchased from American Type Culture Collection (ATCC, Manassas, VA), maintained in suspension, propagated in RPMI 1640 medium, GlutaMAX (Gibco, Carslbad, Cam U.S.A.) supplemented with 20% FCS and 1% antibiotics (100 U/ml penicillin and 0.1 mg/ml streptomycin), and incubated at 37°C in a 5% CO_2_-humidified incubator. For experimental procedures, Jurkat cells were seeded at a density of 5 × 10^5^ per well, pretreated or not for 8 h with 100 µg/ml of TUDCA [26], and then incubated with 1 µg/ml of tunicamycin for 48 h.

### Total mRNA isolation from tissue and cells

The RNeasy Fibrous Mini Kit (Qiagen, Venlo, Netherlands) was used to isolate total RNA from human aortic samples following the manufacturer’s recommendation. Total RNA isolation from human VSMCs and Jurkat cell was performed using the TRI Reagent (Zymo, Research, U.S.A.) following the manufacturer’s instructions. Total RNA was determined and quantified by a NanoDrop 1000 Spectrophotometer (Thermo Scientific). For RNA sequencing (RNA-seq), RNA integrity and quality was assessed on Agilent 2100 Bioanalyzer (Aligent Technologies, Santa Clara, CA, U.S.A.).

### RNA-sequencing analysis

To study up-regulated and down-regulated genes in different patient groups, a whole-genome analysis was performed using RNA-seq for whole-genome transcriptome using RNA-seq at the Genomics/Transcriptomics Core of the CNAG (Barcelona). Total RNA was used as starting material to synthesize cDNA libraries using the Illumina Stranded mRNA Prep kit based on the poly-A selection method, according to the manufacturer’s protocol (Illumina, San Diego, CA, U.S.A.). Libraries were run simultaneously on an Illumina NextSeq 500 sequencing system by the NextSeq 500/550 High-Output reagent kit v2.5 of 150 cycles (2 × 74 bp paired end) (Illumina).

Paired sequence files in FASTQ format were analyzed on the BaseSpace Sequence Hub platform with the Dragen RNA Pipeline v3.7.5 (Illumina). The reference genome used was UCSC hg19. Differential expression analyses were performed with the Dragen Differential Expression application (Illumina). The compared subset of samples were AAA and healthy donor samples. Significantly differentially expressed genes (DEGs) were defined as those with adjusted *P*-values of <0.05 and visualized by EnhancedVolcano tool from R Studio (EnhancedVolcano tool from Bioconductor package.https://bioconductor.org/packages/devel/bioc/vignettes/EnhancedVolcano/inst/doc/EnhancedVolcano.html), and DEGs were ranked by log2 fold change.

### Gene enrichment analysis

Functional enrichment studies were carried out using String Database (version 12.0) (https://string-db.org/) and Reactome Pathways and visualized using SRplot [27]. The complete list of enriched terms can be found in Table S1. LncRNA enrichment analysis was performed by introducing GO terms related to ER stress and mitochondria into the lncRNA Ontology (http://bio-bigdata.hrbmu.edu.cn/lncrnaontology/). The complete list of lncRNAs associated with ER stress and mitochondria can be found in Tables S3 and S4.

For DE mRNAs-lncRNA co-expression matrix, we evaluate whether the normalized expression value of the selected lncRNA and the DE mRNA is linearly correlated by calculating the Person correlation coefficient. We considered correlating the pairs with a Pearson correlation coefficient greater than |0.9|. The complete list of values can be found in Table S2. All DE-mRNA correlated to LINC00494, PCED1B-AS1, TCL6, and LINC00892 were used to perform a functional enrichment study as described above. Reactome pathways selected were used to construct protein–protein interaction networks according to the String database and visualized using Cytoscape software (http://www.cytoscape.org).

### Quantitative real-time PCR

DNase I-treated total RNA (1 µg) was reverse-transcribed into cDNA using the High-Capacity cDNA Archive Kit (Applied Biosystems, Foster City, CA, U.S.A.). The mRNA levels of DE-lncRNAs and ER stress markers were quantified by using specific probes provided by the assay-on-demand system (Applied Biosystems): ATF-4 (Hs00909569_g1), ATF-6 (Hs00232586_m1), CHOP or DDIT3 (Hs99999172_m1), cysteine-rich with EGF-like domains 2 (CRELD2, Hs00360923_g1), heat shock protein 5 (HSPA5), or GRP78 (Hs99999174_m1), IRE1 or ERN1 (Hs00176385_m1), Suppressor/Enhancer of Lin-12-like (SEL1L, Hs01071406_m1), and XBP1 (Hs00231936_m1); and the lncRNAs: CCDC26 (Hs00540885_m1), ITGB2-AS1 (Hs04274319_m1), IL21-AS1 (Hs04976181_s1), ITPKB-IT1 (Hs05032319_s1), LY86-AS1 (Hs00543584_m1), MIR137HG (Hs01939875_s1), PCED1B-AS1 (Hs05027356_m1), TCL6 (Hs00220956_m1), WASIR2 (Hs00415846_m1), LINC00158 (Hs00365202_m1), LINC00381 (Hs01381499_g1), LINC00426 (Hs03680869_m1), LINC00494 (Hs01052916_s1), LINC00544 (Hs00417034_m1), LINC00582 (Hs04274021_m1), LINC00626 (Hs00365491_m1), LINC00861 (HS04274413_m1), LINC00892 (Hs00866930_s1), and LINC00926 (Hs03680805_m1). As housekeeping genes, glyceraldehyde 3-phosphate dehydrogenase (Hs02758991_g1) and β-actin (Hs99999903_m1) or r18SRNA (Hs.PT.39a.22214856.q) were used. Each sample was amplified in duplicate by quantitative RT-PCR in an ABI PRISM 7900HT Sequence Detection System (Applied Biosystems). The same results were obtained after the normalization to both housekeeping genes. Relative mRNA levels were determined using the 2−∆∆Ct method.

### Statistical analysis

Results are expressed as mean ± SEM of the number (*n*) of samples indicated in the figure legends. Demographic and clinical characteristics of the human individuals were expressed as mean ± SD. The normal distribution of the variables was determined by the Shapiro–Wilk test. When data followed a normal distribution, differences between the two groups were assessed using the Student’s *t*-test (two-tailed), and when normality failed, the Mann–Whitney rank sum test was applied. To compare more than two groups, the one-way ANOVA or the Kruskal–Wallis ANOVA test was used and corrected for multiple comparisons by controlling the false discovery rate with the two-stage step-up method of Benjamini, Krieger, and Yekutieli. Pearson product–moment correlation coefficient was used to study the correlation between variables following a normal distribution, whereas Spearman’s rank-order correlation was applied to determine the association between variables when data failed the normality test. Receiver-operating characteristic (ROC) curve analysis was used to evaluate the ability of each lncRNA to discriminate patients from healthy controls. Data analysis was carried out using GraphPad Prism version 9.2.20 software (La Jolla, CA, U.S.A.) and the R Software version 4.4.0 (R Foundation for Statistical Computing, Vienna, Austria. https://www.r-project.org/foundation/). Values of *P*≤0.05 were considered significant.

## Results

### Transcriptional profiling in human AAA

**Figure 1:**
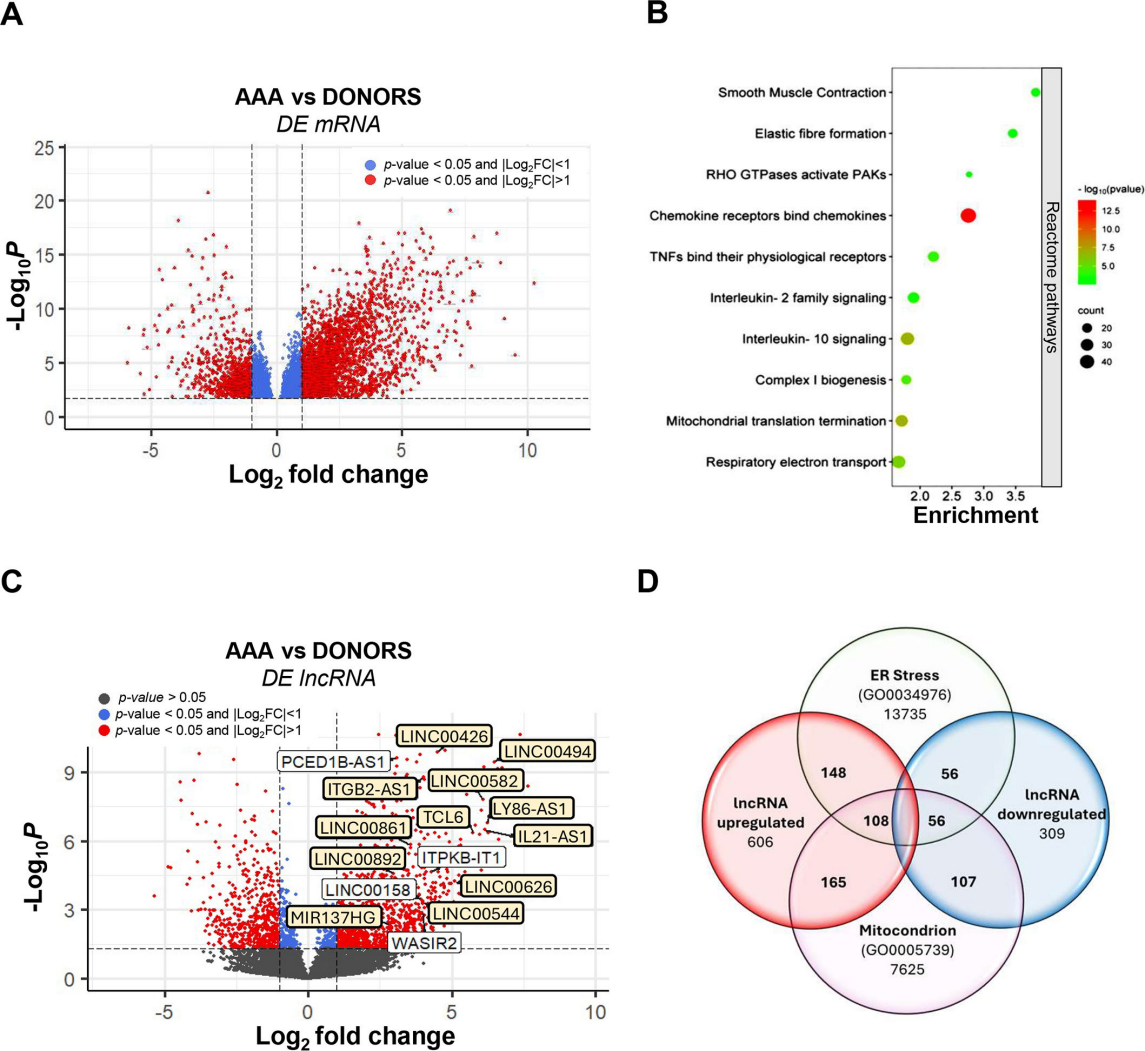
Differentially expressed mRNA and lncRNA based on RNA-seq analysis of abdominal aortic samples from patients (AAA) and healthy donors. (**A**) Volcano plot showing DE-mRNAs expression represented by dots found in AAA tissue samples versus donors (CT). Dots in blue denote DE-mRNAs with a *P* value <0.05 and |Log_2_FC| < 1, and dots in red denote those significantly regulated with |Log_2_FC| ≥ 1. (**B**) Graphic scheme representing the most enriched processes by Reactome pathways of differentially expressed mRNAs (DE-mRNAs). (**C**) Volcano plot of DE-lncRNAs represented by dots found in AAA tissue samples versus donors (CT). Relevant lncRNAs for the study have been highlighted. Dots in blue denote DE-lncRNAs with a *P* value <0.05 and |Log_2_FC| < 1; dots in red denote those significantly regulated with |Log_2_FC| ≥ 1; and dots in gray denote nonsignificant changes of expression. Highlighted in yellow those DE-lncRNAs common with mitochondrion. (**D**) Venn diagram showing the number of up-regulated and down-regulated lncRNAs found in AAA tissue overlapping with those included in the term GO0034976 (ER stress regulation) and those DE-lncRNAs (273 up-regulated and 163 down-regulated) associated with the GO term mitochondrion (GO0005739). A total of 164 of these (108 up-regulated and 56 down-regulated) are DE-lncRNAs common to both processes. AAA, abdominal aortic aneurysm; lncRNA, long non-coding RNA; RNA-seq, RNA sequencing.

A transcriptomic analysis of abdominal aorta samples from patients and controls was performed by RNA-seq (*n* = 12 AAA patients and *n* = 7 donors). Demographics and clinical characteristics of patients and donors included in the RNA-seq closely matched for age, sex, and smoking habit as depicted in Table 1. All the patients included in the study had an abdominal aorta diameter greater than 55 mm, which is the current threshold size indicated for AAA surgical repair [1,2]. Most AAA patients were male, active smokers or ex-smokers, and suffered from hypertension and dyslipidemia (Table 1). The unsupervised hierarchical clustering for gene expression data revealed a distinctive profile associated with aneurysmal disease (Supplementary Figure 1A). A total of 6572 genes were differentially expressed in human AAA in comparison with donors, with 4035 genes up-regulated and 2538 down-regulated (Figure 1A). Functional enrichment analysis of these DEGs indicates an affectation of processes such as smooth muscle contraction, elastic fiber formation, immune system signaling, and mitochondrial biology (Figure 1B). Notably, besides mRNA transcripts, our analysis also identified 1283 DE-lncRNAs in AAA (Supplementary Figure S1B; Figure 1C).

**Table 1:** Demographics and clinical data of abdominal aortic aneurysm patients and controls included in the RNA sequencing analysis.

Characteristics	Controls (*n* = 7)	AAA patients (*n* = 12)
Age (years)	64.6 ± 10.2	65.1 ± 8.4
Sex Male Female	100% 0%	100% 0%
Smoke Currently active Ex-smokers	42.8% 57.2%	50% 50%
Hypertension Yes No	71.4% 28.6%	91.6% 8.4%
Diabetes Yes No	28.6% 71.4%	25.0% 75.0%
Dyslipidemia Yes No	28.6% 71.4%	58.3% 41.7%
Aortic diameter (mm)	-	72.6 ± 15.1

Continuous variables are presented as mean ± SD. Nominal variables are presented as %. No significant differences were found between patients and donors.

### Enrichment of lncRNAs involved in the response to ER stress pathway in human AAA

**Figure 2:**
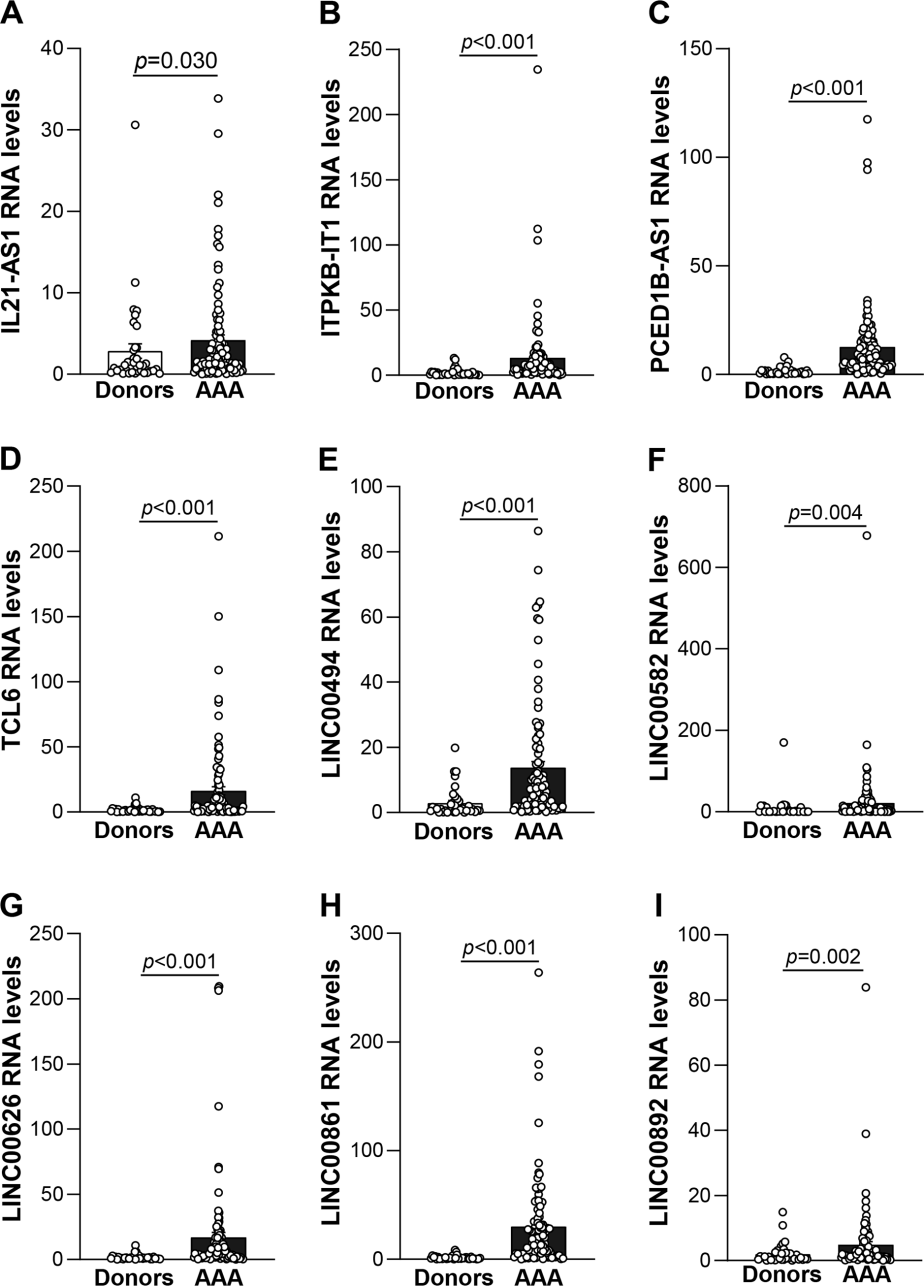
Validation of differentially up-regulated lncRNAs in a large cohort of patients with AAA. (**A–I**) LncRNA up-regulated in the aorta from AAA patients (*n* = 101) in comparison with donors (*n* = 39) was confirmed by real-time PCR (qRT-PCR). The expression of lnRNAs was normalized to β-actin. Data are expressed as mean ± SEM; the *t-*test was used to evaluate differences between both groups. *P*-value <0.05 was considered as statistically significant. AAA, abdominal aortic aneurysm; lncRNA, long non-coding RNA.

Our previous research highlighted the importance of ER stress in the pathophysiology of human and experimental AAA [16,28]. Of note, as shown in the Venn diagram (Figure 1D), functional enrichment analysis in the LncRNA Ontology database revealed that a set of 368 DE-lncRNAs (256 up-regulated and 112 down-regulated) were members of the gene ontology category GO0034976, corresponding to the response to ER stress. Since ER and mitochondrial stress are closely related processes, we also aimed to explore whether these DE-lncRNAs could be related to mitochondrial dysfunction. As shown in Figure 1D, 436 DE-lncRNAs (273 up-regulated and 163 down-regulated) were associated with the GO term mitochondrion (GO0005739). It should be noted that 164 of these (108 up-regulated and 56 down-regulated) are DE-lncRNAs common to both processes (Figure 1D). We specifically focused on the most significantly increased lncRNAs of those up-regulated 256 lncRNAs that include, ITGB2-AS1, IL21-AS1, ITPKB-IT1, LY86-AS1, MIR137HG, PCED1B-AS1, TCL6, WASIR2, LINC00158, LINC00426, LINC00494, LINC00582, LINC00626, LINC00861, LINC00892, and LINC00926 (Figure 1C; highlighted in yellow those DE-lncRNAs common with mitochondrion). The up-regulation in human AAA of these lncRNAs was ratified by real-time PCR in the same set of aneurysmal and donor samples used for the transcriptomic profiling, as shown in Supplementary Figure S2. The expression analysis was also carried out in a large cohort of AAA patients (*n* = 100) and healthy donors (*n* = 39) (Table 2), showing similar characteristics than those described above, that is, about a quarter of AAA patients were diabetics, had chronic kidney disease, and/or suffered from ischemic heart disease. Roughly 60% of the patients were being treated with cholesterol-lowering drugs, antiplatelets, and anticoagulants and 33% with ACE inhibitors (Table 2). Interestingly, the induction of those nine lncRNAs exhibiting the greatest expression in abdominal aorta (IL21-AS1, ITPKB-IT1, PCED1B-AS1, TCL6, LINC00494, LINC00582, LINC00626, LINC00861, and LINC00892) was further confirmed in this large cohort of aneurysmal samples (Figure 2).

**Table 2:** Demographics and clinical data of abdominal aortic aneurysm patients and controls included in the study

Characteristics	All (*n* = 139) N (%)	Controls (*n* = 39) N (%)	AAA patients (*n* = 100) N (%)	*P*-value	Missing values N (%)
Age (years)	70 (65.0–76.0)	60.8 (53.0–52.5)	71.2 (66.8–76.0)	<0.001	0 (0)
Sex Male Female	110 (80.9%) 26 (19.1%)	15 (41.7%) 21 (58.3%)	95 (95.0%) 5 (5.0%)	<0.001	3 (2.2%)
Smoking Active Nonactive	34 (25.0%) 102 (75.0%)	8 (22.2%) 28 (77.8%)	26 (26.0%) 74 (74.0%)	0.654	3 (2.2%)
Hypertension Yes No	85 (69.7%) 37 (30.3%)	13 (59.1%) 9 (40.9%)	72 (72.0%) 28 (28.0%)	0.233	17 (12.2%)
Diabetes Yes No	27 (19.9%) 109 (80.1%)	4 (11.1%) 32 (88.9%)	23 (23.0%) 77 (77. 0%)	0.125	3 (2.2%)
Dyslipidemia Yes No	68 (50.0%) 68 (50.0%)	9 (25.0%) 27 (75.0%)	59 (59.0%) 41 (41.0%)	<0.001	3 (2.2%)
Chronic kidney disease Yes No	25 (18.4%) 111 (81.6%)	1 (2.8%) 35 (97.2%)	24 (24.0%) 76 (76.0%)	0.005	3 (2.2%)
Ischemic heart disease Yes No	25 (22.1%) 88 (77.9%)	1 (7.7%) 12 (92.3%)	24 (24.0%) 76 (76.0%)	0.291	26 (18.7%)
Peripheral artery disease Yes No	47 (45.2%) 57 (54.8%)	1 (25.0%) 3 (75.0%)	46 (46.0%) 54 (54.0%)	0.625	35 (25.2%)
Antiaggregant use Yes No	62 (45.9%) 73 (54.1%)	3 (8.3%) 33 (91.7%)	59 (59.6%) 40 (40.4%)	<0.001	4 (2.9%)
Statins use Yes No	74 (54.4%) 65 (45.6%)	6 (16.7%) 30 (83.3%)	68 (68.0%) 32 (32.0%)	<0.001	3 (2.2%)
IECA use Yes No	35 (26.1%) 99 (73.9%)	2 (5.6%) 34 (94.4%)	33 (33.7%) 65 (65.3%)	0.001	5 (3.6%)
Corticosteroids use Yes No	8 (5.9%) 128 (94.1%)	1 (2.8%) 35 (97.2%)	7 (7.0%) 93 (93.0%)	0.681	3 (2.2%)
AINEs use Yes No	4 (2.9%) 132 (97.1%)	0 (0.0%) 36 (100.0%)	4 (4.0%) 96 (96.0%)	0.573	3 (2.2%)
Immunosuppressants use Yes No	5 (3.7%) 131 (96.3%)	1 (2.8%) 35 (97.2%)	4 (4.0%) 96 (96.0%)	1.000	3 (2.2%)
Aortic diameter (mm)		-	63.0 (56.2–75.2)	…	0 (0)

Because of the participation of the selected lncRNAs in the response to ER stress cluster, we sought to investigate whether there was any correlation between their expression and that of ER stress and ER-associated degradation (ERAD) markers in this large cohort. As depicted in Table 3, significant correlations were found between HSPA5, ATF-6, CHOP, and IRE-1/ERN1 and some of the selected lncRNAs, being XBP1 the most outstanding, since it significantly correlated with ITPKB-IT1, TCL6, and LINC00582. No correlations were detected for ATF-4. More remarkable were the co-expression detected with the ERAD markers SEL1L and CRELD2, the latter correlating with IL21-AS1, ITPKB-IT1, LINC00626, LINC00861, and LINC00892 (Table 3).

**Table 3:** Correlation of endoplasmic reticulum stress markers gene expression with long non-coding RNAs.

	IL21-AS1	ITPKB-IT	PCED1B-AS1	TCL6	LINC00494	LINC00582	LINC00626	LINC00861	LINC00892
	Rho (*P*- value)	Rho (*P*- value)	Rho (*P-*value)	Rho (*P-*value)	Rho (*P*- value)	Rho (*P-*value)	Rho (*P-*value)	Rho (*P-*value)	Rho (*P-*value)
ATF4	−0.060 (0.565)	−0.126 (0.228)	−0.109 (0.300)	0.040 (0.705)	−0.088 (0.399)	−0.105 (0.314)	−0.029 (0.781)	0.012 (0.905)	−0.025 (0.815)
HSPA5	0.009 (0.942)	0.069 (0.560)	0.183 (0.560)	0.072 (0.540)	0.074 (0.528)	0.301 (0.009)	0.035 (0.764)	0.126 (0.284)	0.116 (0.326)
ATF6	−0.027 (0.833)	0.081 (0.520)	0.235 (0.059)	0.273 (0.028)	0.198 (0.113)	0.253 (0.042)	−0.005 (0.971)	0.219 (0.079)	0.224 (0.073)
CHOP	0.123 (0.348)	0.079 (0.548)	0.291 (0.024)	0.233 (0.073)	0.217 (0.096)	0.389 (0.002)	0.154 (0.239)	0.193 (0.140)	0.233 (0.073)
IRE-1	0.027 (0.823)	0.099 (0.423)	0.281 (0.020)	0.195 (0.112)	0.111 (0.367)	0.246 (0.043)	0.137 (0.266)	0.181 (0.140)	0.156 (0.203)
XBP1	0.071 (0.571)	0.304 (0.013)	0.222 (0.074)	0.234 (0.049)	0.085 (0.497)	0.417 (0.001)	0.101 (0.420)	0.148 (0.235)	0.217 (0.081)
SEL1L	0.086 (0.418)	0.137 (0.199)	0.127 (0.234)	0.214 (0.043)	0.019 (0.856)	−0.011 (0.918)	0.112 (0.295)	0.075 (0.482)	0.108 (0.312)
CRELD2	0.233 (0.041)	0.262 (0.017)	0.166 (0.121)	0.132 (0.262)	0.076 (0.520)	0.169 (0.138)	0.217 (0.050)	0.230 (0.040)	0.251 (0.029)

*P*<0.05 in bold indicates a significant *P*-value.

**Figure 3:**
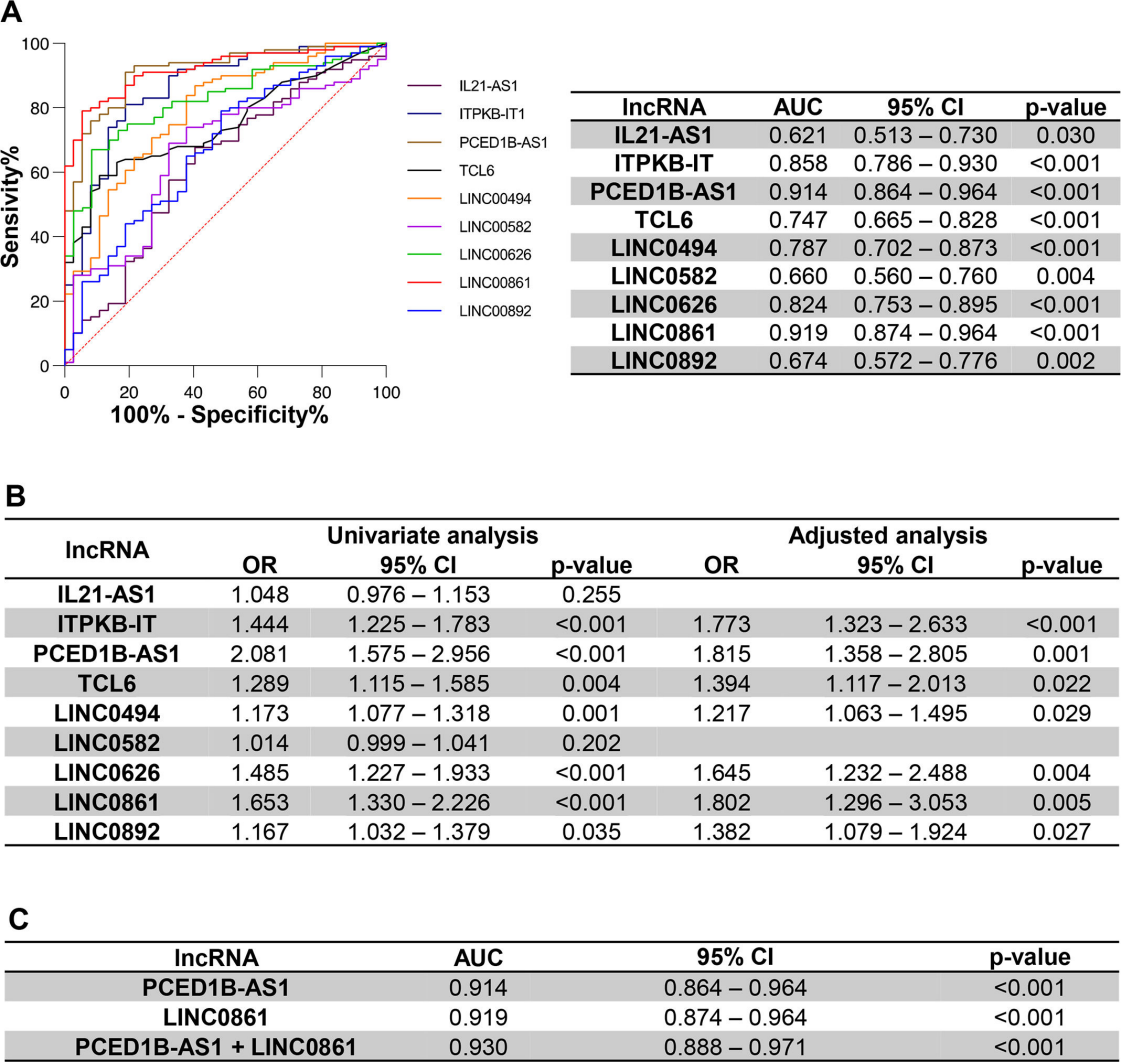
Diagnostic capacity assessment of selected lncRNAs. Receiver-operating characteristic (ROC) curve analysis evidences that the selected LncRNAs discriminate AAA patients (*n* = 101) from the healthy controls (*n* = 39). (**A**) ROC curves for the nine selected lncRNAs are shown. The table below shows the area under curve (AUC) with the 95% confidence interval and *P-*value for each lncRNA. (**B**) Univariate logistic regression analysis for the selected lncRNAs. The model was adjusted to sex, age, and smoking history. (**C**) Table showing the AUC with the 95% confidence interval and *P-*value of the lncRNAs with the highest OR. *P*-value <0.05 is considered as statistically significant. AAA, abdominal aortic aneurysm; lncRNA, long non-coding RNA.

Interestingly, the ROC curve analyses of the nine lncRNAs showed that all lncRNAs discriminated AAA patients from donor subjects. LINC0861 and PCED1B-AS1 displayed the highest performance. The area under the curve (AUC) was 0.919 (95% CI 0.87–0.96) for LINC0861, and 0.914 (95% CI 0.86–0.96) for PCED1B-AS1, indicating that these two lncRNAs show the best sensitivity and specificity and supporting the reliability of each lncRNA in distinguishing aneurysmal from donor samples (Figure 3A). Additionally, we performed a logistic regression analysis to figure out the association of each lncRNA to AAA diagnosis (Figure 3B). In the univariate analysis, the lncRNAs IL21-AS1 and LINC0582 were excluded due to the loss of the association with AAA. After adjusting for age, gender, and smoking, the rest of the lncRNAs increased their association with AAA. PCED1B-AS1 and LINC0861 exhibited the best-adjusted OR values and were selected for further analysis in ROC curves. Combining these lncRNAs in a single model did not result in a significantly superior discriminatory capability compared with each lncRNA alone with an AUC of 0.930 (95% IC 0.888–0.971). Furthermore, we extended this analysis by performing a backward stepwise regression analysis resulting in a combination of four lncRNAs (ITPKB-IT + LINC0626 + LINC0861 + LINC0892), but the discriminatory performance of this combination analyzed by ROC curves resulted in a non-significant AUC improvement of 0.933 (95% IC 0.893–0.974), compared with each lncRNA alone (data not shown).

### Regulation of lncRNAs by ER stress in VSMC and Jurkat cells in culture

**Figure 4:**
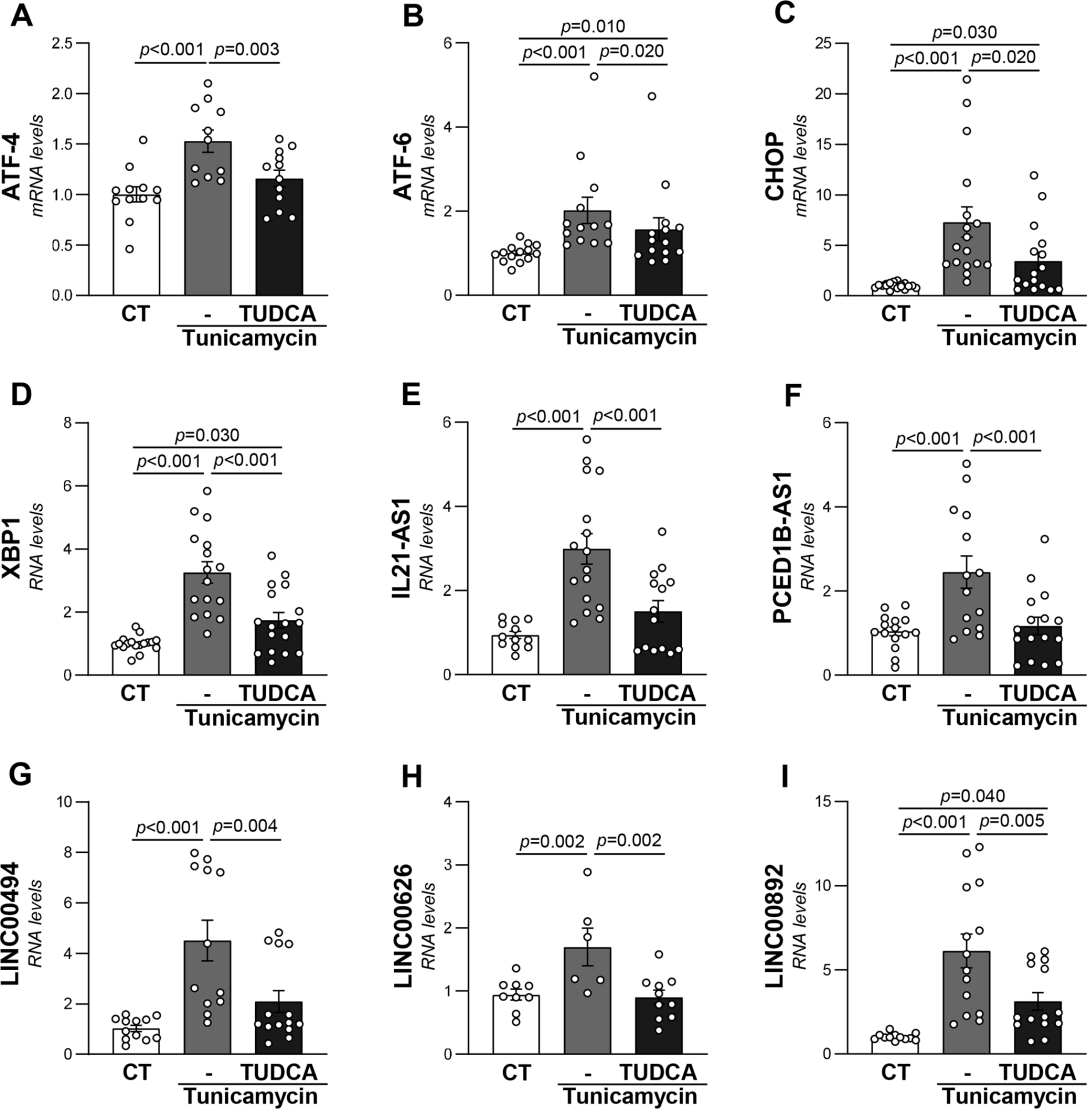
The induction of ER stress in human VSMCs triggered the expression of specific lncRNAs. VSMCs were treated with by tunicamycin (1 μg/ml) in the presence or absence of TUDCA (500 μg/ml). The transcript levels of ATF4 (**A**), ATF6 (**B**), CHOP (**C**)**,** XBP1 (**D**), and those of the lncRNAs: IL21-AS1 (**E**), PCED1B-AS1 (**F**), LINC00494 (**G**), LINC00626 (**H**), and LINC00892 (**I**) were assessed by real-time PCR and normalized to glyceraldehyde 3-phosphate dehydrogenase. Results are expressed as mean ± SEM. The one-way ANOVA corrected for multiple comparisons by controlling the false discovery rate (two-stage step-up method of Benjamini, Krieger, and Yekutieli) was used to evaluate differences between conditions. *P-*value <0.05 was considered statistically significant. ER, endoplasmic reticulum; lncRNA, long non-coding RNA; TUDCA, tauroursodeoxycholic acid; VSMCs, vascular smooth muscle cells.

**Figure 5:**
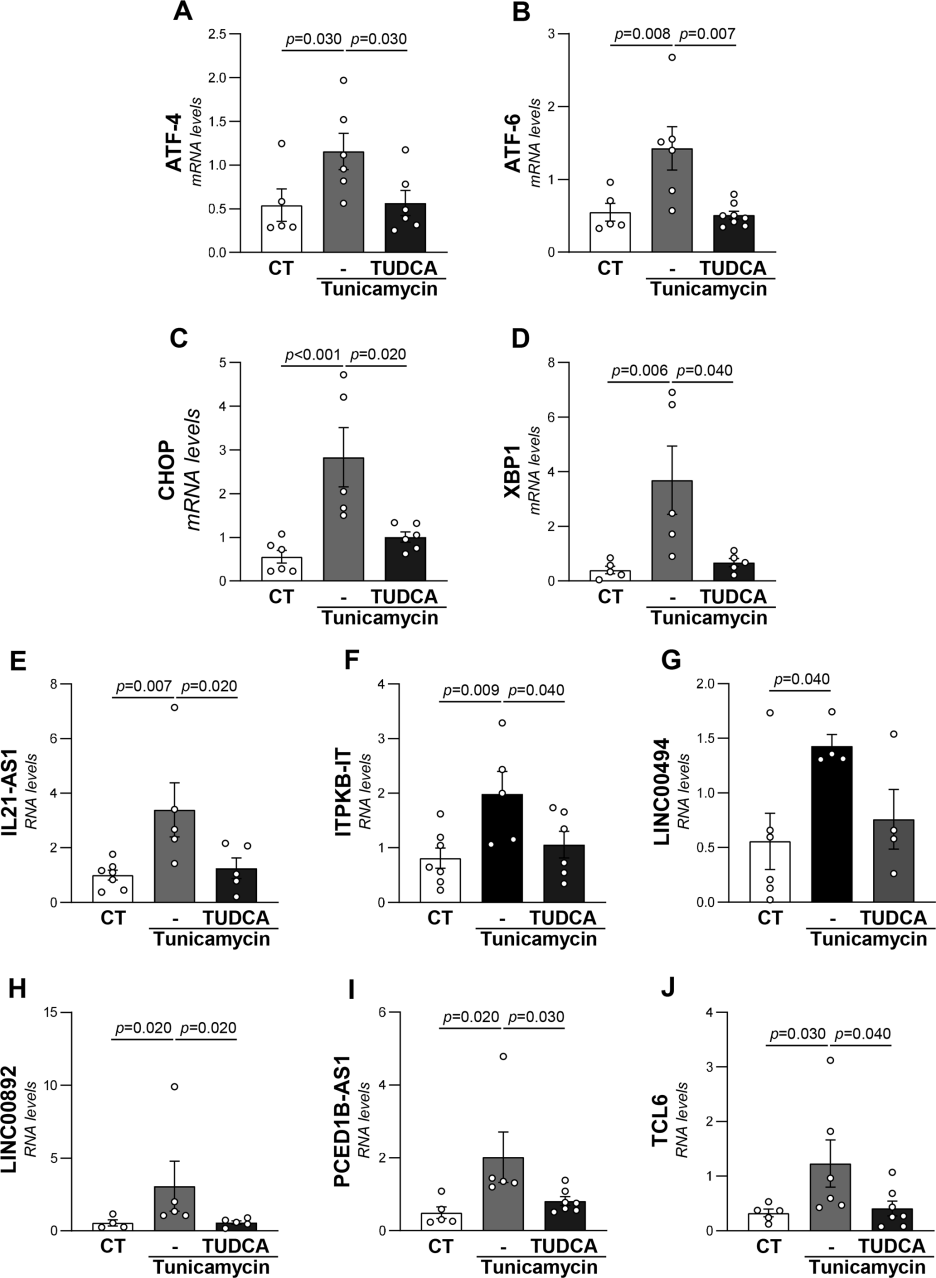
The induction of ER stress in Jurkat T cells up-regulated the expression of specific lncRNAs. Jurkat T cells were treated with by tunicamycin (1 μg/ml) in the presence or absence of TUDCA (100 μg/ml). The transcript levels of ATF4 (**A**), ATF6 (**B**), CHOP (**C**), XBP1 (**D**), and those of the lncRNAs: IL21-AS1 (**E**), ITPKB-IT (**F**), LINC00494 (**G**), LINC00892 (**H**), PCED1B-AS1 (**I**), and TCL6 (**J**) were assessed by real-time PCR and normalized to r18s. Results are expressed as mean ± SEM. The one-way ANOVA corrected for multiple comparisons by controlling the false discovery rate (two-stage step-up method of Benjamini, Krieger, and Yekutieli) was used to evaluate differences between conditions. *P-*value <0.05 is considered statistically significant. ER, endoplasmic reticulum; lncRNA, long non-coding RNA; TUDCA, tauroursodeoxycholic acid; VSMCs, vascular smooth muscle cells.

Next, we aimed to characterize the ER stress-dependent regulation of the identified lncRNAs in human VSMC and Jurkat cells, a human immortalized T-lymphocyte cell line. The treatment with tunicamycin, which is extensively used as an experimental tool to induce ER stress, significantly increased the expression of the ER stress markers ATF-4, ATF-6, CHOP, and XBP-1 in both cell types (Figures 4 and 5). Concurrently, tunicamycin up-regulated the levels of five of the selected lncRNAs (IL21-AS1, PCED1B-AS1, LINC00494, LINC00626, and LINC00892) in VSMCs (Figure 4), while in Jurkat, it enhanced those of IL21-AS1, ITPKB-IT1, LINC00494, LINC00892, PCED1B-AS1, and TCL6 (Figure 5). The treatment with TUDCA, a classic ER stress inhibitor that significantly abolished the tunicamycin-mediated increase in ER stress markers in both cell types (Figures 4 and 5), normalized IL21-AS1, PCED1B-AS1, LINC00494, and LINC00626 in VSMC (Figure 4), while it attenuated the up-regulation of all lncRNAs induced by tunicamycin in Jurkat cells, except that of LINC00494 (Figures 4 and 5).

### Integrated analysis of mRNA-lncRNA co-expression

**Figure 6:**
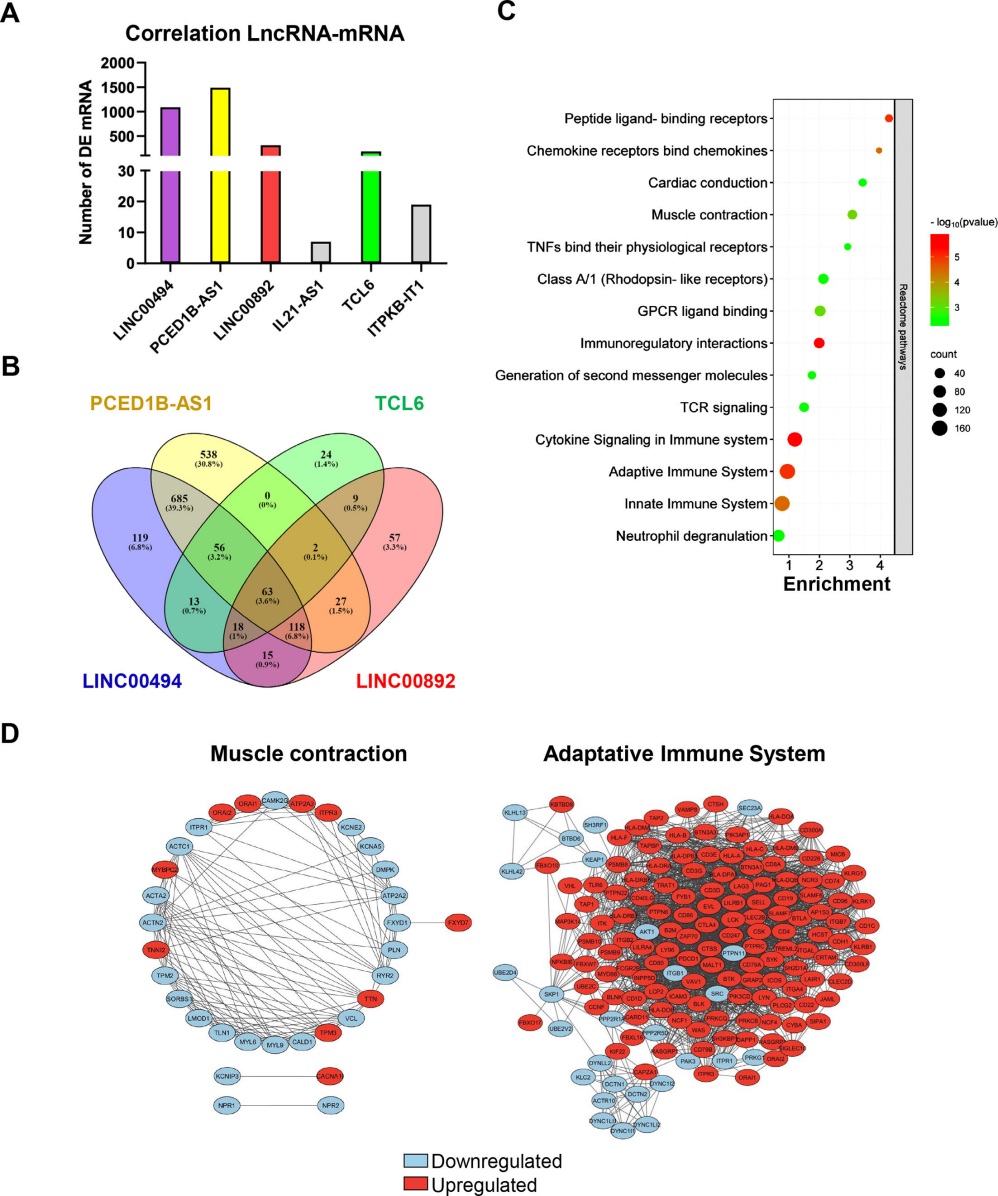
Comprehensive analysis of mRNA-lncRNA co-expression profile in AAA. (**A**) Number of differentially expressed mRNAs whose expression correlates with that of those lncRNAs related to ER stress and validated in the study. (**B**) Venn diagram showing the relationship between DEGs whose expression correlates with the lncRNAs validated in the study. (**C**) Reactome pathways enrichment of DEGs whose expression correlates with that of lncRNAs validated in the study. (**D**) Protein–protein interaction network of the DEGs identified in (**A**) and involved in muscle contraction and adaptive immune system. Down-regulated DEGs in aorta from AAA were indicated in blue and up-regulated in red. AAA, abdominal aortic aneurysm; DEGs, differentially expressed genes; ER, endoplasmic reticulum; lncRNA, long non-coding RNA.

To establish the possible functional relationship between the selected lncRNAs and the DE-mRNAs identified in human AAA, we generated a co-expression matrix. Both LINC00494 and PCED1B-AS1 correlate with more than 1000 DE-mRNAs (Figure 6A), most of which are common (Figure 6B). In turn, we observed that TCL6 and LINC00892 correlate with 185 and 310 DE-mRNAs, respectively (Figure 6A), and found that these genes are largely shared with those that correlate with LINC00494 and PCED1B-AS1 (Figure 6B). The ontological analysis of the mRNA transcripts differentially regulated in AAA that correlate with LINC00494, PCED1B-AS1, LINC00892, and TCL6 evidenced the participation of these DE-mRNAs in muscle contraction and in different aspects of the immune response (immunoregulatory interactions, TCR signaling, cytokine signaling in immune system, adaptive immune system, innate immune system, and neutrophil degranulation in 336 different genes among different categories) (Figure 6C). Protein interactions among the DE-mRNAs included in the clusters ‘muscle contraction’ and ‘adaptive immune system’ were analyzed by STRING as depicted in Figure 6D, where it can be observed how most of the DE-mRNAs involved in muscle contraction were down-regulated in AAA tissue (blue bubbles), while those related to the adaptive immune system were up-regulated (red bubbles).

## Discussion

Although remarkable progress has been made in AAA research over the last few decades, the development of new pharmacological strategies to fight against this disease has been a challenge which is still unsolved [10]. Notably, accumulating evidence supports that epigenetics is critical in aneurysmal disease and that its control may hold potential for therapeutic intervention. Certainly, epigenetic mechanisms involving histone posttranscriptional modifications, DNA methylation, and non-coding RNAs seem to play an essential role in the control of fundamental processes in aneurysmal disease, such as VSMC function, inflammation, and ECM remodeling, and emerge as potential targets for pharmacological interventions and biomarkers [10,29]. However, among them, the regulation of gene expression through lncRNAs is less understood and has been poorly characterized. Here, our transcriptome profiling in human abdominal aorta samples has revealed a more complete picture of lncRNAs in this disease and our bioinformatic analysis has provided a comprehensive analysis of relevant lncRNAs related to ER stress in the pathogenesis of AAA.

Similar to previous studies using RNA-seq in human AAA and healthy aortic tissue samples [30–32], our high-throughput sequencing and functional enrichment analysis revealed the disturbance of genes involved in the control of vascular function and inflammation in this disease. Patients and donors included in our transcriptional profiling study were matched for age, sex, and smoking habit, allowing to minimize biased observations. Interestingly, our analysis identified a bulk of transcripts corresponding to lncRNAs whose expression was differentially regulated in aneurysmal samples. Previous high-throughput analysis in human AAA samples focused on the study of lncRNAs was limited, and few lncRNAs were confirmed as differentially regulated in human disease [29–31,33].

Of note, a huge number of the differentially regulated lncRNAs found in our study are involved in the response to ER stress, which plays a critical role in AAA development. In fact, we have previously reported the induction of ER stress in human AAA, its relationship with oxidative stress, and that ER stress inhibition abrogates aneurysm development in a preclinical model [16,28]. Specifically, our validation in a large cohort of patients with AAA and donors identified nine ER stress-related lncRNAs significantly up-regulated in aneurysmal tissue. Moreover, a significant correlation with the expression of ER stress and ERAD markers was detected for most of these lncRNAs. Further, PCED1B-AS1, LINC0861, IPTKB-1, LINC0626, LINC0494, and TCL6 exhibited the highest AUC values in ROC curves, indicating a high sensitivity and specificity regarding their ability to distinguish those individuals with AAA. The adjusted analysis by confounding factors showed that most of the selected lncRNAs kept their association with AAA excepting IL21-AS1 and LINC0582.

Recent works have focused on the mechanisms linking specific lncRNAs to ER stress and their contribution to high-impact diseases such as heart disease, cancer, and diabetes. These studies have unraveled how the downstream components of the UPR regulate transcription and translation reprogramming to ensure selective gene and lncRNAs expression in response to pathological stimulus [34–36]. It should be noted that some of the lncRNAs investigated in the present study, such as TCL6, LINC0892, and IL21-AS1, were reported to be involved in metastasis and tumor progression [37,38] and in other pathological conditions such as preeclampsia [39,40]; however, this is the first study involving these selected lncRNAs in AAA.

The contribution of lncRNAs to human AAA has been scarcely addressed, although recent studies have revealed that specific lncRNAs affect some of the mechanisms underlying AAA growth [29–33]. A recent single-cell RNA sequencing analysis revealed the down-regulation of the lncRNA CARMN in human aneurysmal VSMC associated with the altered contractile function of these cells [41]. Accordingly, in our study, CARMN expression was found down-regulated in AAA samples, and it is also one of the lncRNA related to ER stress (data not shown). The up-regulation of the lncRNA H19 was confirmed in a small cohort of tissue samples from donors and AAA patients, and studies in preclinical models support its role in the progression of AAA through the control of VSMC survival and inflammation [29,42,43]. Similarly, GAS5 and PVT1 have been involved in human AAA development by inducing VSMC apoptosis among other mechanisms [44,45]. In accordance, our RNA-seq analysis detected the up-regulation of H19 and PVT1, but not that of GAS5, in AAA samples (data not shown); however, neither H19 nor PVT1 was among the ER stress-related lncRNAs identified in our study.

Both medial VSMC paucity and vascular inflammation are critical contributors to vascular degeneration and weakening in AAA [12,46,47] and are closely associated with ER stress and mitochondrial dysfunction [13,16,28]. Although our data in human samples could not ascertain whether the response of these lncRNAs i a cause or a consequence of the response related to ER stress, studies on VSMC and T cells in culture indicate that, in both cell types, the activation of ER stress significantly up-regulated the expression of most of the DE-lncRNAs found in human aneurysmal lesions, an effect abolished by the ER stress inhibitor TUDCA. Thus, these data support the direct relationship between these transcripts and ER stress in vascular and inflammatory cells. Further, we have established the lncRNA-mRNA network in human AAA. Based on the functional enrichment analysis of the DEGs and from the ontological analysis of the correlation between the DE-mRNA and DE-lncRNAs, the identified lncRNAs signature has been related to both vascular inflammation and VSMC contractile dysfunction, and similar results were found by analyzing the protein interaction profile. Although some of the lncRNAs investigated in our work have been described as biomarkers in other disorders and lncRNA H19 shows evidence to be a diagnostic biomarker for cardiovascular diseases, no specific data have been reported in the setting of AAA [48]. The differential profile of other lncRNAs rather than those described here has been recently reported in peripheral blood mononuclear cells and serum from AAA patients [49,50], and their interest as biomarkers deserves further investigation.

In summary, based on our data, lncRNAs might be considered as key regulatory molecules involved in the response to ER stress in AAA, affecting both vascular inflammation and VSMC function. Although the identified lncRNAs have no murine homologous counterparts, hampering *in vivo* studies, further research is warranted to determine whether the lncRNAs signature described in this work is involved in the progression of the disease and might become the basis for the development of new pharmacologic interventions in AAA.

**Clinical perspectives**

- No effective pharmacological strategies are available to limit abdominal aortic aneurysm (AAA) progression; therefore, there is an urgent clinical need to identify new therapeutic targets. Long non-coding RNAs (lncRNAs) contribute to the regulation of vascular function and inflammation, but their specific contribution to AAA development remains unknown.
- We determined the lncRNAs expression profile in AAA by transcriptomic analysis, identifying a signature of endoplasmic reticulum (ER) stress-associated lncRNAs that discriminate patients with AAA from healthy subjects. Interestingly, an integrated analysis of differentially expressed mRNAs and selected lncRNAs co-expression revealed their role in immune response and muscle contraction.
- The identified lncRNAs emerge as critical regulatory molecules of ER stress in AAA and may be considered as therapeutic targets for the management of this disease.

## Supplementary material

Supplementary Figure S1

Supplementary Table S1

Supplementary Table S2

Supplementary Table S3

Supplementary Table S4

## Data Availability

The data that support the findings of this study are included as supplementary files (Tables S1, S2, and S3) in Excel format and may be asked of the corresponding authors upon reasonable request.

## Open Access

This article has been published open access under our Subscribe to Open programme, made possible through the support of our subscribing institutions, learn more here: https://portlandpress.com/pages/open_access_options_and_prices#conditional

## Abbreviations

AAA: abdominal aortic aneurysm
DEGs: differentially expressed genes
ER: endoplasmic reticulum
RNA-seq: RNA sequencing
VSMCs: vascular smooth muscle cells
lncRNA: long non-coding RNA
