# Supplementary material for: Identification of endoplasmic reticulum stress-associated lncRNAs influencing inflammation and VSMC function in abdominal aortic aneurysm
Source: Clin Sci (Lond). 2025 Mar 25;139(6):357–72. doi: 10.1042/CS20242476 (PMC12204013; doi:10.1042/CS20242476)
Supplement: Supplementary Figure S1 [file CS-139-06-CS20242476-s001.docx]

SUPPLEMENTARY MATERIAL

**Identification of endoplasmic reticulum stress-associated lncRNAs influencing inflammation and VSMC function in abdominal aortic aneurysm**

Rafael Almendra-Pegueros^1^, Cristina Rodriguez^1,2^, Mercedes Camacho^1,2^, David Sánchez-Infantes^3^, J. Luis Sánchez-Quesada^1^, Susana Cáncer^4^, Elvira Pérez-Marlasca^3^, Gema Medina-Gómez^3^, José Martinez-González^2,5^, Ana B. García-Redondo^2,6*^, María Galán^2,3*^.

^1^ Institut de Recerca Sant Pau (IR Sant Pau). Barcelona, Spain.

^2^ Centro de Investigación Biomédica en Red de Enfermedades Cardiovasculares (CIBERCV), Instituto Carlos III (ISCIII). Madrid, España.

^3^ Facultad de Ciencias Básicas de la Salud, Universidad Rey Juan Carlos. Alcorcón, Madrid, España.

^4^Unidad de Angiología y Cirugía Vascular. Hospital Universitario Fundación de Alcorcón, Alcorcón, Madrid.

^5^Instituto de Investigaciones Biomédicas de Barcelona-Consejo Superior de Investigaciones Científicas (IIBB-CSIC),Barcelona, España.

^6^ Departamento de Fisiología, Facultad de Medicina, Universidad Autónoma de Madrid, Madrid, Spain.

*Co-corresponding authors.

**Figure S1**

**
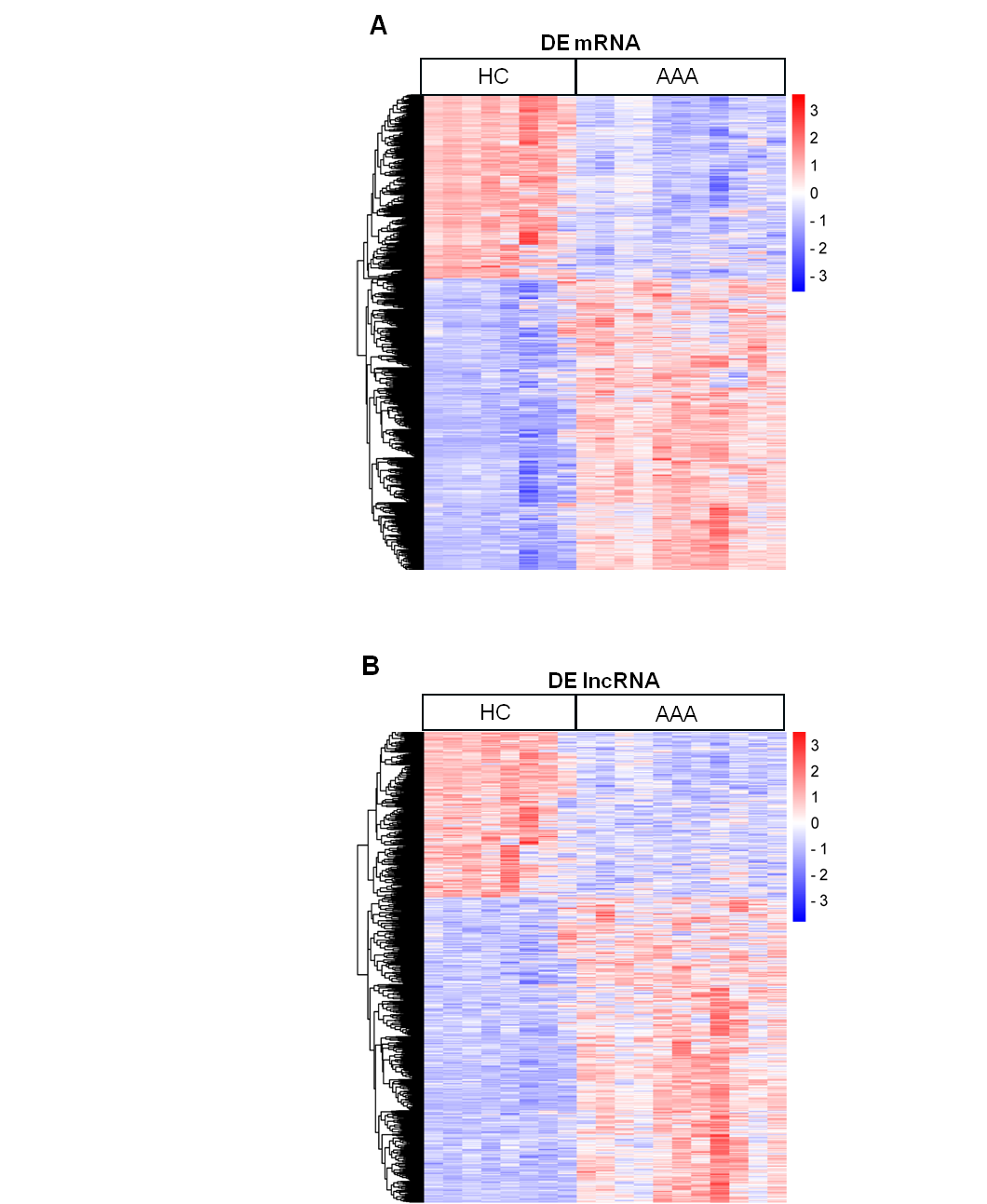
**

**Figure Supplementary 1.** **Profiles of differentially expressed mRNA and lncRNA based on RNAseq analysis of abdominal aortic samples from patients (AAA) or healthy donors (controls).** **(A-B)** Heatmaps showing differentially expressed mRNA (DE mRNAs) and differentially expressed LncRNA (DE LncRNAs) profiles in healthy donors (HC) and AAA patients.

**
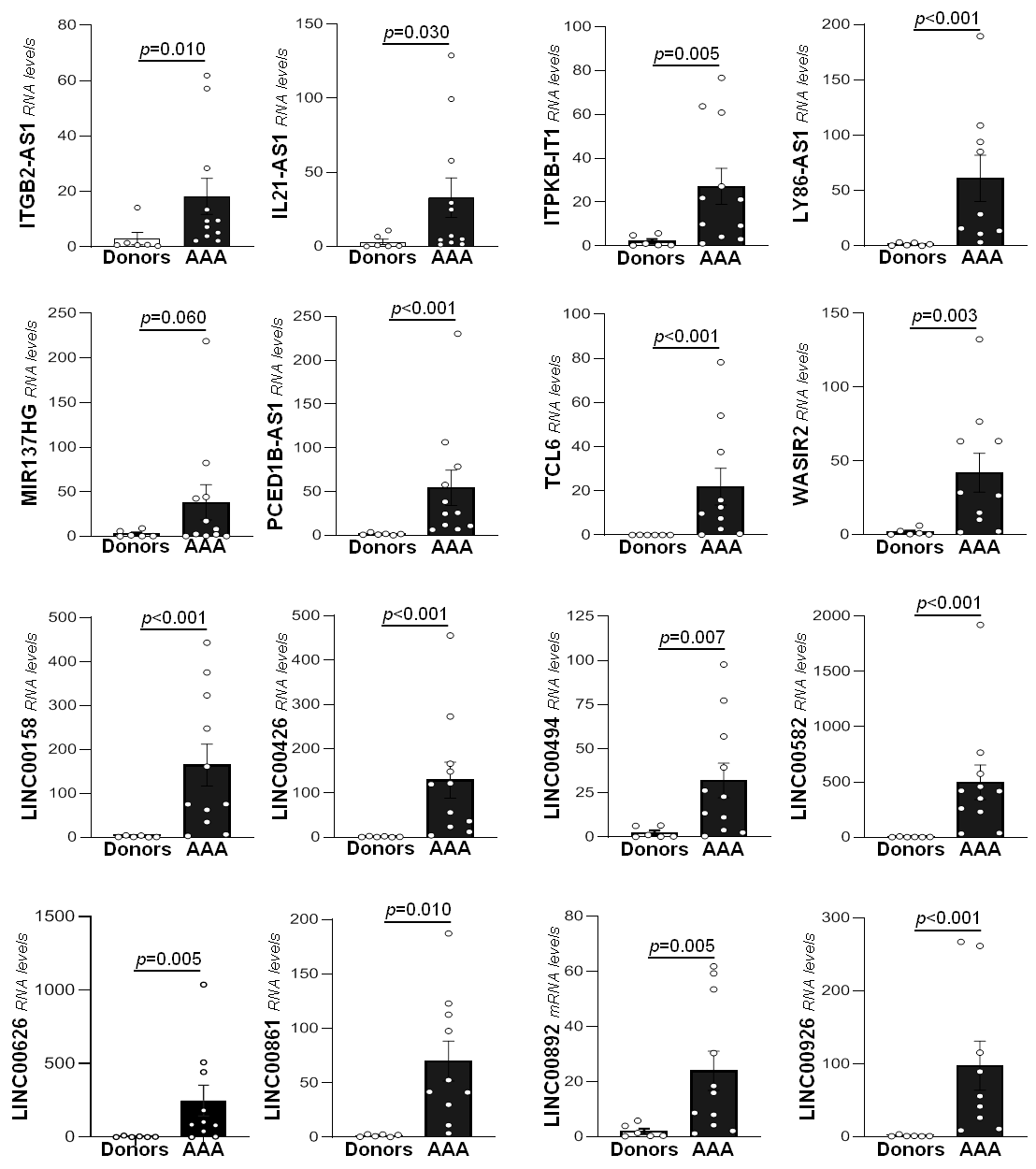
Figure S2**

**Figure Supplementary 2. The overexpression of the most significantly upregulated lncRNA in the aortic wall from AAA patients was confirmed by qRT-PCR.** The overexpression of 16 LncRNA in the aortic wall from AAA patients (n=11) was statistically significant (p <0.05) in comparison to healthy donors (n=6). The expression was normalized to ß-Actin*.*
