# Supplementary material for: Identification of endoplasmic reticulum stress-associated lncRNAs influencing inflammation and VSMC function in abdominal aortic aneurysm
Source: Clin Sci (Lond). 2025 Mar 25;139(6):357–72. doi: 10.1042/CS20242476 (PMC12204013; doi:10.1042/CS20242476)
Supplement: Supplementary Table S1 [file CS-139-06-CS20242476-s002.pdf]

Table S1. RNAseq analysis of aorta from AAA patients and donors. lncRNA expressed in aortic samples inclu

ID	
GO0034976 (13733 elements)	lncRNA upregulated in AAA (256 elements)
linc-BLK	RP11-360F5.3
RP11-560A15.3	RP11-322E11.5
RP11-27P7.1	RP11-87G24.6
RP11-783L4.1	RP11-862L9.3
linc-RBFOX2-2	LINC00924
RP11-798K23.4	RP11-624L4.1
RP11-354K4.2	RP11-109A6.3
RP6-114E22.1	CTC-378H22.2
RP11-309E23.2	RP11-142C4.6
linc-ZNF99-5	AC104024.1
CTC-504A5.1	CTD-2047H16.2
RP1-177I10.1	AC007750.5
RP11-100L22.4	RP11-945C19.4
RP11-201E8.1	GS1-72M22.1
linc-DCC-1	RP1-30M3.5
DMD-AS1	CTC-523E23.1
linc-PHACTR2-2	RP4-555D20.2
RP11-388N2.1	RP13-580F15.2
RP11-614F17.2	RP11-423H2.3
linc-TAOK3-1	CTC-265F19.1
RP11-50B3.2	CACNA1C-AS1
RP11-690I21.2	FAM225A
linc-OAF-4	RP11-203E8.1
linc-TMEM179	RP11-61F12.1
RP11-341G5.3	RP11-439L18.1
RP11-384E22.1	RP11-446N19.1
linc-LOC100132288-1	CTB-114C7.4
RP11-386M24.3	RP11-489O18.1
RP11-335E6.2	RP11-705C15.3
AC003051.1	RP5-991G20.2
RP11-6O2.3	AP001434.2
RP11-567G11.1	RP11-261P9.4
RP11-794G24.1	CTD-2547L24.4
RP11-395P17.11	DIO3OS
RP11-10N16.2	RP11-16C1.2
linc-TP53TG3B-6	RP11-834C11.10
RP11-95P13.2	RP1-8B1.4
AC019068.2	RP11-203B7.2
linc-MFAP4-2	CTB-41I6.2
LINC00864	ITPKB-IT1
RP1-273G13.3	LINC00626
AC010729.2	RP11-1143G9.4
linc-DHRS4-2	RP11-147L13.8
RP11-463J10.3	RP11-325K4.2
linc-AKR1E2-13	RP11-1036E20.9
linc-DYRK1A-1	AC004947.2

RP11-335O4.3
RP11-96B2.1
RP11-45L9.1
RP11-267A15.3
linc-C20orf71
linc-PION-1
linc-ALG10
RP4-754E20__A.5
linc-ZNF479-1
RP11-460N16.1
LINC00683
AC012065.4
RP11-70F11.11
RP11-70F11.8
linc-USP12-1
RP11-116N8.2
linc-HEXDC-3
RP11-432I5.6
RP11-295M18.2
RP1-10C16.1
linc-MAOA-1
CTC-551A13.1
linc-TRIML2-6
AC005306.3
RP11-82C23.2
linc-CCRN4L-10
RP11-472B18.2
FAM183CP
AC007036.6
RP11-981G7.1
AC004535.2
RP11-608B3.1
RP11-1079K10.2
RP11-465K16.1
CTB-104H12.4
RP11-869B15.1
RP11-261C10.2
RP11-523O18.7
RP11-638I2.2
RP11-594C13.2
linc-TSPAN5-1
linc-PCDH9-4
CTA-747E2.10
RP11-513D5.5
AC019118.2
RP11-14I17.2
linc-SALL3-1
AC067961.1
linc-FGFR1OP-9
RP11-554D15.4
LINC00158
RP11-284F21.10
LINC00861
CTD-2583A14.8
RP11-92K15.3
RP11-44F14.2
LINC00544
RP11-138I18.2
LINC00926
MIR600HG
RP11-536O18.1
RP5-1011O1.3
ITGB2-AS1
RP11-809N8.4
RP11-266O8.1
RP1-68D18.2
RP11-598F7.6
RP11-473M20.9
TCL6
RP11-799O21.2
CTD-2006K23.1
WASIR2
AP003774.1
CTB-102L5.7
CTD-2509G16.5
AC027601.1
RP11-1151B14.4
LINC00174
RP11-493L12.4
AC009784.3
RP11-1149M10.2
RP11-344B5.2
RP11-542K23.7
RP11-119J18.1
LINC00299
RP11-996F15.2
U62631.5
RP11-815J21.1
KB-173C10.1
AC008982.2
CTC-281F24.1
RP11-445F6.2
LINC00562
RP11-33A14.1
RP11-327F22.6
CTC-523E23.11
RP11-125O18.1
CCDC26
RP11-328J6.1
RP11-16K12.1

RP11-598C10.2	BX255923.3
RP11-140M13.1	RP11-526I2.5
RP11-433J22.2	RP11-283G6.4
AC108448.3	HMGA1P4
RSF1-IT1	RP11-60A8.1
RP5-1100I6.2	RP11-284N8.3
RP13-20L14.4	ZBTB20-AS1
EHHADH-AS1	RP11-439E19.9
CTC-348L5.1	RP11-768B22.2
RP11-53O19.2	RP11-111A22.1
linc-LPHN2-2	RP11-21C4.1
RP11-242F11.2	RP11-16E12.2
CTC-268N12.3	RP11-594N15.3
LINC00210	RP11-492E3.2
LINC00698	RP1-212P9.3
RP11-685M7.5	AC159540.1
RP1-276E15.1	RP1-29C18.9
linc-PLEKHA5-1	RP11-960L18.1
linc-HIST1H2AI-1	RP11-404F10.2
RP11-542K23.9	CACNA1C-AS2
linc-C13orf28-4	RP11-415F23.3
CTD-2049O4.1	RP11-693J15.4
CTD-2515C13.2	PCED1B-AS1
RP11-83M16.5	RP13-638C3.4
RP11-292B1.2	LINC00841
RP11-686D16.1	RP1-29C18.8
RP11-470L19.2	PCAT1
RP11-798G7.6	AP001056.1
RP11-17M24.1	RP11-667K14.3
AC006037.2	RP11-412P11.1
AC114752.1	RP11-873E20.1
linc-GJA1-2	RP11-35G9.5
AC106875.1	RP11-624C23.1
RP11-637O11.2	TRBV11-2
RP11-352D13.5	RP11-1094M14.5
linc-PROP1-2	RP11-11N9.4
RP11-142G1.3	LINC00494
AC005197.2	RP11-649A18.4
linc-TGS1	DBH-AS1
AC005019.3	RP11-13P5.1
RP11-164C12.2	RP11-333A23.4
LINC00460	RP11-96K19.4
linc-ALDH6A1	RFPL1S
AC093690.1	AC092484.1
RP11-295M3.2	RP11-44F14.8
linc-FMN1-4	RP11-497H17.1
AC004448.5	RP11-354E11.2
linc-TMEM132C-8	LINC00243
linc-INSIG2-1	AC137932.5
linc-C3orf67-1	RP11-863P13.3

RP11-5K23.5
linc-TBX20-4
AC104389.28
AP000705.7
SC22CB-1D7.1
linc-SLITRK1-4
CTD-2335O3.3
linc-GAPT-1
RP1-267L14.3
linc-CNKSR2
HNF1A-AS1
CTD-2162K18.3
AC016909.1
Metazoa_SRP
RP1-159G19.1
linc-APCDD1
RP11-532F6.5
RP11-343N15.2
linc-NDN-2
FAM170B-AS1
RP11-5N23.3
linc-NADSYN1-2
linc-TRIM43B-2
RP11-61D3.1
RP11-196G11.2
linc-PRR20A-2
RP11-527L4.6
RP11-439M11.1
linc-C2orf60-2
CTD-2161F6.2
RP5-1047A19.4
RP11-387H17.6
CTA-357J21.1
RP11-168L7.1
linc-ATL2-1
RP11-148K1.12
linc-CD9-3
RP4-781K5.9
RP11-719J20.1
AC093375.1
linc-ZC3H12A-1
RP11-142A23.1
CTD-2270F17.1
RP11-584P21.2
RP11-245J9.4
linc-RORB-7
RP11-104D21.3
RP1-239B22.5
linc-SLC38A11
RP11-15M15.1
RP11-380I10.4
CTD-2576D5.4
RP11-223C24.1
RP4-647J21.1
AC013463.2
CTD-2342N23.3
AC079807.4
IL21-AS1
RP11-43F13.3
FAM13A-AS1
AC009133.17
AC004538.3
AC093642.4
AC006460.2
CTD-2035E11.4
LINC00525
RP11-495K9.6
AE000661.37
RP11-219E7.1
H19
CHRM3-AS2
RP11-598F7.5
RP11-519G16.3
RP11-419I17.1
RP11-138A9.1
RP13-452N2.1
RP11-713C5.1
LINC00582
RP11-303E16.2
LINC00937
RP11-164H13.1
RP11-96K19.2
RP11-325K4.3
AP001057.1
RP11-68I3.11
RP11-680C21.1
LINC00880
LINC00578
ANKRD44-IT1
RP11-415F23.2
Z84812.4
RP5-968J1.1
RP11-297B17.3
AC006129.2
LINC00656
RP11-598F7.3
LINC00381
WT1-AS
RP5-1092A3.4
AC132217.4

RP11-780O17.1	RP11-44N11.1
AC004381.7	RP11-23P13.6
RP11-373J21.1	LINC00639
RP11-342D11.2	RP11-1084E5.1
linc-HAS2-1	RP11-373L24.1
RP11-388M20.1	RP11-482H16.1
AC005304.1	RP11-161M6.3
RP11-401P9.6	AF127936.3
linc-SALL1-1	LINC00892
linc-ADRA1D-3	CTC-510F12.4
AP002856.4	RP11-645C24.5
linc-SEP15-6	LINC00402
RP11-521O16.2	IGF2-AS
CTC-479C5.10	LINC00189
RP11-483P21.3	RP11-96D1.11
linc-DTL-1	RP11-408O19.5
RP11-395B7.7	LINC00877
RP11-666E17.1	RP11-334C17.5
RP11-369E15.4	AJ006998.2
RP11-446H18.5	RP11-611O2.5
RP11-210K20.2	AC131056.3
CTB-25J19.9	LINC00968
RP11-867G2.5	LINC00944
RP11-165H23.1	LY86-AS1
linc-RGPD4-8	RAMP2-AS1
RP4-735C1.4	RP11-585P4.5
GS1-251I9.2	RP11-865I6.2
AP4B1-AS1	MIR137HG
linc-TMEM215-1	RP11-1023P17.2
RP11-48B3.3	RP13-297E16.4
linc-ARID1B-5	AC007278.3
RP11-558A11.3	IFNG-AS1
linc-FOXF1-5	RP1-153P14.5
linc-GJB5	RP11-491F9.1
RP5-1085F17.3	RP11-588H23.3
linc-C13orf23-3	RP3-395M20.9
AC011193.1	RP5-1091N2.9
WI2-87327B8.1	RP11-326C3.15
RP11-158A14.1	RP11-325F22.2
linc-FAM174A-1	RP11-214O1.3
RP11-478H13.1	CTD-2020K17.3
linc-GAS6-2	RP11-318K15.2
NLGN1-AS1	RP4-622L5.2
AC010982.2	RP11-981P6.1
RP11-145E17.2	RP11-527N22.2
RP11-69H14.6	AC006369.2
HCCAT5	AC022182.3
A2ML1-AS1	RP11-430C7.5
RP11-380L11.3	CTD-2583P5.3
RP11-810B23.1	AC008697.1

AC012494.1
RP11-463P17.1
RP11-845C23.2
RP11-16D22.2
linc-NCKAP1-2
linc-CD99-3
AC012507.4
RP11-150O12.2
linc-DLX6-2
linc-RAB40A-1
AC005592.1
RP11-16L9.3
linc-FAM169A
linc-ABCA5-7
RP11-29B9.1
RP11-385J1.2
DOCK9-AS1
AC015987.1
RP1-232L24.3
RP1-148H17.1
AC096732.2
SAA2-SAA4
RP5-896L10.1
RP11-261C10.5
LINC00690
RP11-384C4.2
RP11-659E9.4
linc-POTEB-1
MIMT1
linc-ADAMTSL3
SLC9A9-AS2
RP1-12G14.5
RP11-510M2.9
RP13-726E6.1
AP000235.3
RP11-883A18.3
RP11-522B15.4
RP11-123O22.1
RP11-797A18.3
linc-HIST1H2AG-4
linc-IRX3-2
RP11-96B5.4
RP11-99H20.1
RP11-242P2.2
linc-NUDT15-3
AC007308.6
AC011243.1
RP4-647C14.3
RP11-151A6.4
RP11-347P5.1
AC012314.8
RP11-415F23.4
RP11-760H22.2
CTD-2555A7.3
RP11-13P5.2
CHL1-AS2
LINC00426
RP11-557H15.4
RP3-439F8.1
RP4-758J18.7

RP11-77K12.3
RP5-1048B16.1
linc-LRFN5-2
RP11-142G1.1
linc-NGLY1-1
linc-P4HA1
ACAP2-IT1
RP11-1002K11.1
GS1-256O22.5
linc-KLF12-1
RP11-282O18.6
RP11-669C19.1
AP000402.3
CTC-246B18.8
RP11-506K6.4
AC068535.2
RP11-459J23.1
RP11-576E20.1
RP11-19N8.7
RP4-723E3.1
linc-CD1D-2
linc-ALDH1A1-2
RP11-380M21.4
RP11-403N16.3
linc-OCM-3
RP11-932O9.4
RP11-174G17
RP11-831F12.2
linc-LOC150786
RP11-703G6.1
RP11-139K4.2
RP11-321M21.1
RP11-305A4.3
RP11-522D2.1
linc-CNOT6-1
RP11-618M23.2
AC002331.1
linc-RGS8-1
RP11-552E20.1
RP11-429E11.2
linc-GALNTL6-1
linc-SMC1B-2
RP11-48F14.1
RP11-20I23.13
TMEM212-AS1
RP11-98D18.3
RP11-762H8.4
linc-XPA-2
AC005943.4
TDRG1

RP4-631H13.2	
linc-FBXL5-2	
RP11-388G22.1	
RP11-563P16.1	
CTD-2026D20.2	
LINC00619	
RP11-885N19.6	
RP11-588P8.1	
CTD-2506J14.1	
RP11-485G7.6	
GPHB5	
AC104782.3	
linc-TLE3-2	
linc-PKIA-2	
linc-POTEC-3	
linc-RARRES3	
RP11-109P11.1	
RP3-473B4.3	
RP11-103H7.3	
linc-TMEM169-7	
linc-NTM-6	
RP11-491F9.8	
LINC00272	GO0034976.txt
RP11-73G16.1	
linc-RAB19	
RP11-1085N6.2	
RP11-905K4.1	
linc-ARHGAP28-7	
RP4-614O4.11	
MORC1-AS1	
RP11-75C23.1	
linc-MDM2	
RP11-14N9.2	
LINC00675	
RP11-166A12.1	
RP1-23E21.2	
RP11-2E17.1	
KB-1930G5.3	
linc-ST3GAL5-2	
AC018470.4	
CTD-2021H9.1	
RP11-450H5.1	
SEC62-AS1	
RP11-375H17.1	
AC005013.5	
RP11-775H9.3	
linc-TSSK3-2	
linc-SSTR4-5	
RP11-84C10.2	
AC012445.1	

CTD-3118D11.2
RP11-486B10.3
AC092669.2
linc-ASCC3-1
linc-SMS
LINC00305
linc-CADM2-6
KB-1608C10.2
RP11-568A19.1
linc-POLR3F
AP001439.2
AP006216.11
RP11-272D20.2
linc-SOX3-2
RP11-685G9.4
linc-C2CD4B-7
AC104781.1
RP11-862G15.1
linc-ALDH1L1-3
RP11-442N1.1
RP11-765K14.1
linc-ALG2-1
RP11-205M3.3
CTD-2081K17.2
CTC-229L21.1
linc-LRFN2-1
RP11-238K6.2
linc-RGL4-1
CTC-360P9.5
RERG-IT1
linc-TRMT112-3
RP11-171I2.1
RP11-667F9.2
RP11-554D14.6
RP11-553P9.3
LINC00486
AC002115.5
RP5-1185K9.1
linc-C15orf41-1
RP5-1139I1.1
linc-RSPO3
linc-GLDC-1
CTD-2016O11.1
RP4-678D15.1
RP11-63E5.6
linc-C18orf62-3
RP11-28F1.2
RP11-1084A12.2
RP11-90E5.1
linc-NUDT9-1

RP4-706G24.1
linc-IVNS1ABP
RP11-159J2.2
RP11-391M7.3
RP11-132E11.2
AC093627.8
RP11-128P17.1
AC005162.4
CTD-2145A24.3
CTB-184G21.1
linc-NOTCH2-3
RP11-20F24.4
RP11-278H7.3
RP11-514A9.1
RP1-64K7.4
LINC00475
RP11-702L15.4
RP11-25K21.1
RP5-827O9.1
RP11-569A11.1
CHL1-AS1
RP1-111C20.4
RP11-85O21.2
RP11-263K4.5
RP11-672L10.3
CTD-2196E14.4
RP5-1022P6.4
RP11-439C8.2
RP11-453E2.2
RP11-431D12.1
RP11-127O4.3
CTC-304I17.3
AC009487.4
linc-SOX9-4
RP11-579E24.2
RP1-50O24.6
AC011526.1
RP11-180D21.3
RP11-706F1.1
RP1-90J20.2
GRIK1-AS1
RP11-148I19.1
RP11-716O23.1
RP3-495K2.2
RP11-417F21.1
RP11-351I21.11
RP11-416O18.2
RP1-225E12.2
CTB-180A7.6
RP11-53M11.3

AC007386.2
SMIM2-IT1
RP11-309M7.1
WI2-2373I1.2
LINC00517
AC118754.4
MLIP-AS1
RP11-93K22.13
linc-EPHA6-2
CTD-2078B5.2
LA16c-306A4.1
RP11-368P15.3
linc-KIAA1755-8
AC008079.9
AC007463.2
CTD-2297D10.1
AC004237.1
RP11-879F14.3
linc-TTC23
RP11-781M16.2
linc-KIAA0649-5
AC007566.10
linc-OR2AG1-2
RP11-466A19.8
RP11-626P14.2
linc-GGTLC1-1
CYP17A1-AS1
SIDT1-AS1
RP11-216C10.1
AC073333.8
ADAMTS9-AS1
RP11-447M4.1
RP11-114B7.6
linc-GPD2-3
AC018766.4
AC000370.2
RP11-700H13.1
RP11-298O21.5
CTC-265N9.1
U66059.58
L34079.3
RP11-141J13.5
RP11-264K23.1
linc-FOXB2-3
CTD-2587H19.3
AC008060.5
RP11-203H2.2
CTD-2313J17.5
AL162151.4
linc-TMC5

linc-ZNF449-2
linc-FAM84B-9
AC007682.1
RP11-369K17.1
RP11-502N13.2
linc-HABP4-3
RP11-415C15.2
RP6-102O10.1
RP11-43F13.4
linc-XRCC2-2
linc-PDCD2-1
linc-CPB2
linc-LOC642587-7
linc-IQCA1-6
AC010744.1
linc-TSEN2
RP1-124C6.1
linc-GALNT1
RP11-338N10.3
LINC00577
linc-PRDM8-3
CTD-2184D3.3
RP11-210M15.1
LA16c-366D1.3
PCDH9-AS4
RP11-55L3.1
RP11-706C16.5
RP11-335K5.2
RP11-12D16.2
XXyac-YM21GA2.4
linc-RALGPS2-2
linc-FLJ44606-4
NKX2-2-AS1
ADAMTS19-AS1
linc-ARFGEF2-7
CTC-367F4.1
CTD-2530N21.4
RP11-136C24.2
RP11-252P19.2
RP3-405J10.3
RP11-64B16.3
linc-CDH10-1
RP11-146G7.2
RP11-488C13.6
linc-CDH11-2
RP11-266K22.2
linc-IARS2-2
RP11-1070N10.5
RP11-488L4.1
RP5-944M2.1

linc-TMEM90A
RP11-1070N10.7
RP11-64J4.2
RP11-556H2.2
linc-SKIL
RP11-779O18.1
linc-VPS33B-3
RP11-458K10.3
RP11-1064E11.1
RP11-669M16.2
linc-ACTL7A-2
LINC00348
AC009120.6
linc-SPTLC3-1
RP11-167H9.6
RP11-864J10.4
AC016700.3
LINC00901
RP5-845O24.8
linc-ARHGAP32-2
linc-STEAP1-2
linc-GLTSCR1-2
linc-USP14-1
LBX1-AS1
RP11-923I11.6
linc-HIST1H2AI-2
RP11-142O6.1
AC003092.2
MIR1587
LINC00555
AL157359.4
AC113167.1
linc-GPR141-1
RP11-616M22.7
linc-ATP13A4-2
RP11-454C18.2
RP4-784A16.1
RP11-586D19.2
RP11-486O13.2
AC011518.1
AP000345.4
RP11-196D18.1
linc-ADCY2-8
AC090044.1
LINC00709
linc-AHR-1
RP11-515E23.1
RP11-122L4.1
RP11-64D24.4
linc-FAHD2B-1

CTD-3138F19.1
linc-RCBTB1
linc-RPL38-3
RP11-64C12.4
AC115522.3
AP000946.2
RP11-894P9.1
KB-431C1.5
RP11-398M15.1
FAM41C
AC007193.8
PSMD5-AS1
LINC00516
linc-KCNMB2-3
RP11-166N6.2
RP11-18A15.1
linc-KIAA0564-3
RP3-395M20.7
FAM157A
RP11-116G8.5
linc-CD180-8
LINC00868
RP3-448I9.2
RP11-475A13.1
RP4-614N24.1
linc-MTOR-2
RP11-710F7.2
RP11-74C13.3
RP11-100L22.2
linc-CARD11-5
RP11-537A6.9
linc-ZNF516-3
AP000469.2
linc-WDR81-3
RP11-439H8.4
FAM25D
RP11-462G22.1
RP11-521J24.1
CTD-2194L12.2
RP11-386M24.8
RP6-91H8.5
RP11-577N1.1
AC103881.1
CTB-57H20.1
RP11-383C5.7
RP4-665J23.4
linc-USPL1-2
AC020743.4
AP000432.2
linc-C8orf46

RP11-304L19.11
RP11-69E11.8
RP11-91K9.1
linc-GPR157-2
RP11-889D3.2
linc-ARFGEF2-14
RP11-1137G4.3
linc-ANKRD50-3
linc-CHD9-6
RP11-473E2.2
RP11-156K13.2
RP11-310H4.2
RP11-439K3.3
CACNA2D3-AS1
RP11-44L9.3
linc-HMX1-3
ALG13-AS1
AC018730.3
CTD-2516F10.4
linc-YOD1-5
AC017074.2
RP11-88I21.2
RP11-5P22.3
linc-ABLIM2
RP11-834C11.8
RP1-20C7.6
RP11-805F19.1
RP1-65J11.5
LINC00575
RP11-476K15.1
RP11-756K15.2
linc-DUSP22-2
PLCB1-IT1
RP11-63N8.3
RP11-679B19.2
linc-DPP6-2
linc-LDOC1L-1
RP11-281P23.2
RP11-142A12.1
linc-RHOBTB2-2
CTD-3096M3.2
RP4-794H19.1
SNORD116-21
AC000120.7
PABPC5-AS1
linc-ERG-11
LPP-AS1
RP11-481J2.2
linc-NRIP1-1
RP11-676F20.1

AC139887.4
AC007795.1
AF131215.3
AC017076.5
RP11-290F20.3
LINC00641
RP11-91I8.1
linc-C22orf24-2
linc-PTPRD-2
RP11-155L15.1
RP11-152C17.1
RP11-21A7A.2
linc-C3orf30-6
CTD-2376I20.1
LEMD1-AS1
AB015752.4
RP3-398G3.5
RP13-30A9.1
linc-FMOD-3
linc-RNF170-1
RP11-61E11.1
linc-CDH5-10
RP11-705O24.3
AP000431.1
RP4-791M13.3
CTB-109A12.1
GRM7-AS2
RP11-180I22.2
AC108066.1
RP13-379O24.2
LINC00622
RP11-299P2.1
RP11-403D15.2
linc-SLC25A48-2
RP5-839B4.8
RP1-60O19.1
linc-SVIL-1
linc-SULF2-6
RP11-223C24.2
linc-KIF16B-4
RP11-431J24.2
RP11-643G5.6
RP11-328C8.5
GS1-18A18.1
RP11-778H2.1
RP11-527D7.1
RP11-71E19.5
RP11-511P7.2
RP11-265N7.2
linc-EFHA1-3

RP11-89B16.1
RP11-253D19.1
RP11-299D14.2
linc-ADAMTS14
linc-FGFRL1
linc-PLGLB2
LINC00226
RRM1-AS1
RP11-355I22.6
RP11-271F18.4
CTD-2582M21.1
RP11-274M17.3
RP11-423H2.5
linc-MAP1LC3B2-6
RP11-439H13.2
RP5-1057J7.6
RP11-834C11.3
linc-CYP7B1-1
linc-NRSN1-4
RP11-142G7.2
linc-RYR3
RP11-363D24.1
linc-STX2-3
AC145110.1
RP11-863H1.1
linc-NTSR2-4
RP11-202G11.2
RP5-1195D24.1
linc-PCDH20-4
linc-ANKRA2-5
linc-N4BP3
RP11-167N24.3
RP11-159H3.2
RP11-281J9.2
RP11-449D8.5
RP11-348B17.1
RP11-867G23.4
CTD-2066L21.3
linc-SPAM1
RP11-766N7.3
RP11-220I1.2
RP11-84A1.3
linc-CLDN24-3
RP11-71G7.1
RP11-256L11.1
RP11-61J19.2
linc-SLC35F5-2
RP11-445F12.2
RP11-4O3.2
RP11-436H11.3

AC011523.2
linc-VWA5A
AC091969.1
RP11-49G10.3
linc-SH3BP2
RP11-544A12.8
RP11-65L3.4
RP11-124D2.3
AC027119.1
RP11-304M2.5
linc-URB2-3
PCDH9-AS2
RP11-256I23.1
RP11-348F1.3
AC010524.2
linc-RPL19-3
linc-ZNF91-1
RP11-382E9.1
AC097495.3
RP11-380D23.2
linc-SLC9A8
RP11-353N14.1
RP11-73G16.2
RP11-1437A8.5
RP11-192C21.2
PTCSC3
RP11-513M1.1
linc-GATAD1
AC016745.3
AC140912.1
linc-LRP12-2
RP11-1145L24.1
RP11-109D24.1
linc-RBM12B-2
CTD-2296D1.1
RP11-98J9.3
linc-CES2-2
linc-DUXA-2
RP11-246A10.1
linc-DDX18
RP11-79H23.3
RP11-342C20.2
RP4-738P15.1
RP11-532E4.2
linc-PIGF-2
MORC2-AS1
linc-C18orf55-6
KB-1107E3.1
RP11-642D21.2
RP11-749H20.4

linc-MAP1LC3B2-7
linc-HNF4G
CTB-46B19.2
RP11-454K7.1
RP11-162G9.1
RP11-169E6.4
LL0XNC01-250H12.3
RP11-517C16.4
linc-AP3S1-2
CTD-2210P24.6
CTD-2308G16.1
CTC-297N7.1
AC013401.2
linc-KIAA1462
linc-UNC5D-1
LINC00161
RP11-1030E3.1
AC013271.3
RP11-323I1.1
linc-STARD3NL
RP11-395E19.7
RP11-404K5.4
linc-PIK3C3-4
RP11-302J23.1
ZNF571-AS1
CTD-2135D7.5
RP11-134D3.2
linc-CDH9-3
RP11-831A10.1
CTD-2008A1.1
linc-FBXL14
RP11-1078H9.5
RP11-133K1.7
RP11-65J21.1
CTC-503J8.6
linc-AGA-10
RP11-838N2.4
linc-CHAC2-2
linc-RAB17-2
CTD-2270L9.5
RP11-37N22.1
RP11-567N4.3
ARHGEF19-AS1
LINC00604
SCARNA22
RP11-413M3.4
CTD-3179P9.1
GS1-251I9.3
RP11-177F11.1
RP4-597A16.2

RP5-991C6.3
linc-LIG4-1
linc-CARD11-3
RP11-405K6.1
RP11-78J21.4
linc-TSPO2-1
linc-MFHAS1-2
RP11-25C19.3
LA16c-385E7.1
RP11-883G14.2
RP11-571M6.17
linc-RPRM-2
CTD-3099C6.11
linc-MOCS2-1
FAM215B
RP11-704J17.5
linc-CADM1-4
HCG23
RP11-520D19.2
RP4-644L1.2
AC073636.1
RP11-409K20.6
linc-SDCBP
RP11-229P13.20
linc-C3orf30-4
RP11-240D10.4
linc-HMBOX1
linc-GARS-3
RP11-415A20.1
CTD-2561B21.3
AC116035.2
RP11-372K14.2
RP11-486O13.4
linc-NUDCD2-5
RP11-671C19.2
RP11-945A11.2
RP11-248J18.2
AC007099.2
CTD-2154H6.1
RP11-252M18.3
RP11-720L2.3
RP11-359N11.2
RP11-628E19.2
AC006227.1
linc-COX7A2L-2
RP11-173A16.2
linc-EDAR
RP11-848D3.5
AP005135.2
RP11-909B2.1

RP11-703M24.5
RP11-91P24.6
RP11-655C2.3
AC096579.7
RP1-17K7.2
RP1-207H1.3
CTB-39G8.3
RP11-360D2.1
RP11-647P12.1
RP11-727M10.2
RP11-456O19.4
linc-OTUD4-1
XX-CR54.1
RP11-509A17.3
RP5-983L19.2
RP11-334A14.8
RP11-250B2.3
RP4-555L14.4
linc-MUSK-4
linc-TAF13
linc-DLGAP2-1
linc-PTCHD2-1
linc-CBLB-12
LINC00320
linc-GCNT2-5
linc-CX3CR1-3
RP4-779E11.3
linc-CDH2-2
linc-ANKRD20A1-4
CACNA1C-AS4
RP3-393K13.1
RP11-96A1.5
RP11-806L2.2
RP11-325L7.2
linc-RHOB-5
linc-VPS33B-5
RP11-22P4.2
RP11-371I1.2
RP11-774I5.1
RP11-638I2.9
RP11-57C13.4
AC007271.3
AC009276.4
AC008691.1
RP11-138A23.2
linc-DAOA-2
linc-FER-2
LINC00595
RP11-342K6.3
RP11-69H7.3

RP11-109P6.2
CTD-2227I18.1
RP11-927P21.2
RP11-484D2.4
linc-E2F7-3
RP1-59M18.2
RP4-564M11.2
CTD-2568P8.1
RP11-110H1.8
linc-LRIG1-1
PPEF1-AS1
RP11-399F2.2
AC004160.4
RP11-103J17.2
linc-DUSP26-3
RP11-457D2.3
RP11-793B23.1
AC068580.6
linc-KLHL25-1
RP3-348I23.2
RP11-794P6.1
RP1-256G22.2
RP11-36N20.1
LL0XNC01-237H1.3
linc-C18orf62-7
CTD-2134P3.1
linc-OR4M2-5
CTC-523E23.14
linc-CENPW-2
AC114813.1
RP11-76E17.2
RP11-508O18.1
RP11-114O8.1
RP11-232L2.2
RP13-653N12.1
RP11-24N18.1
LINC00566
AC009965.2
CTD-2325P2.4
linc-CALCOCO1-6
linc-ERICH1-8
CTA-342B11.2
RP1-45I4.3
linc-NUDT12-4
LINC00334
linc-THPO-2
AC018832.1
AC107622.1
RP11-281A20.1
linc-ZNF521

RP11-706D8.3
AC010987.6
RP11-181B11.2
RP11-240A16.1
RP11-36B6.1
linc-AJAP1-4
linc-MUC20-5
RP11-12K6.2
RP11-799B12.2
linc-ZNF567
RP11-58B17.2
linc-GTF2I
CTC-268N12.2
RP11-319G9.5
CTB-33O18.2
RP11-261N11.8
RP1-136J15.3
linc-LRCH1-4
RP11-700F16.3
linc-DYDC1-3
RP1-193H18.2
PCGEM1
CTC-548K16.2
RP5-834N19.1
linc-DNAH6-5
RP3-410C9.2
linc-MT1B
CTB-12O2.1
linc-PARP4
AC013402.4
linc-MELK
RP11-52L5.6
RP11-64B16.4
RP11-1033H12.1
AC016716.2
RP13-635I23.3
linc-ZNF726-7
RP11-713N11.5
linc-MMADHC-3
RP11-168O10.6
NARF-IT1
RP11-444A22.1
CTD-3247F14.2
CTD-3110H11.1
linc-AKAP4
RP11-81F13.1
linc-EGR4-3
RP11-619A14.3
RP11-395L14.3
RP11-441F2.2

linc-HMGCS1
linc-RGMA-6
RP11-727F15.13
linc-G2E3-2
linc-IKZF2-2
linc-STX18-2
RP11-190I17.2
RP11-45M22.3
linc-FRG1-2
linc-LOC642587-5
AC098973.2
CTD-2193G5.1
RP11-370F5.4
linc-CDH9-5
RP11-365O16.6
RP11-536C10.4
RP11-782C8.4
RP11-907D1.1
AC114803.3
linc-ZIC2
linc-RAD21-3
linc-TNKS2-2
CTC-429L19.3
RP11-80B9.1
linc-THBS1-2
RP11-3L21.2
RP11-168C9.1
RP11-194G10.3
linc-IL1F9
RP11-548L20.1
CTC-441N14.1
linc-ALDH1B1-2
RP11-2N1.1
CTD-2523D13.1
CTC-366B18.4
RP3-425P12.5
AP001063.1
CADM2-AS1
linc-KIAA1712-3
linc-CDK17-3
linc-ATPBD4-2
RP11-281H11.1
RP11-15B24.1
AC008694.3
RP11-701B16.2
AC012456.3
RP6-206I17.3
AC009508.1
RP11-161D15.2
RP11-697B24.1

RP11-197N18.7
linc-PSMB2
RP11-316M21.7
RP5-828K20.2
RP11-169D4.2
AC079610.1
AC104113.3
AC092660.1
RP11-540D4.3
linc-ARF5-4
linc-DLGAP2-2
linc-ZNF236-4
linc-C15orf2-7
linc-C16orf78-3
linc-SESN3-2
linc-FGF9-1
RP11-254I22.1
RP13-444H2.1
RP11-38J22.6
RP11-243A14.1
LINC00685
RP11-697E22.3
RP11-433J8.2
RP11-337L12.1
RP11-254F7.1
linc-RWDD4
RP1-309F20.4
AC064875.2
RP11-386O9.2
RP11-567M16.2
RP11-430H10.3
CTA-992D9.7
RP11-255M2.1
RP11-340E6.1
RP11-133L19.2
AC020595.1
AC002519.8
RP11-116A1.1
linc-LINGO2-2
RP11-313F23.3
CTB-60B18.12
AC002401.1
RP11-304C12.3
CTD-2517M22.9
linc-HARS-1
RP11-936I5.1
linc-RBM26-1
linc-COL4A2-1
RP11-318G21.4
RP11-12K11.2

linc-SLC25A15-1
RP11-749H17.2
RP1-5O6.6
CTC-369A16.3
RP11-176F3.7
RP11-433M22.1
RP4-668G5.1
linc-ST8SIA3-1
RP13-497K6.1
RP1-249H1.2
RP11-429H9.4
linc-CA8-1
RP11-463J7.2
linc-COL6A5-4
linc-MAP1LC3B-4
RP11-542A14.1
RP1-122K4.3
linc-ELK3-1
RP11-422P22.1
RP11-526P5.1
linc-STXBP6-2
RP11-265F19.1
linc-COPG-1
RP11-364B14.1
RP11-164C1.2
linc-IGFBP7-12
linc-LPPR5-1
AC010127.5
RP11-809C18.3
linc-PSD2-1
RP11-804L24.2
RP3-323P13.2
RP11-104H15.8
RP11-893F2.6
RP11-475B2.1
linc-CCL23
linc-GPR45-3
linc-LRRC8D-2
linc-API5-2
AC007557.2
RP11-354I13.2
RP11-121L11.1
RP11-193P11.3
RP4-684O24.5
linc-MYCN-2
CTB-58E17.9
linc-CCBE1
BACH1-IT2
RP4-755D9.1
linc-ODZ3-4

RP11-528N21.1
RP11-315A17.1
CTA-339C12.1
CTC-499B15.8
RP11-261P24.2
RP11-168P8.3
RP11-167H9.4
FAM182A
linc-CREBBP
WI2-80269A6.1
RP11-1072C15.2
LINC00421
RP11-100F15.1
linc-SLC25A32-2
KB-1183D5.14
RP11-13J8.1
AGBL1-AS1
RP11-485M7.3
RP11-687D19.1
RP11-1376P16.1
linc-MPHOSPH8-3
RP4-693M11.3
RP11-186F10.2
RP11-356J5.12
RP1-288M22.2
RP11-213G2.2
RP11-212I21.4
linc-MGAT5B-3
RP13-39P12.3
RP11-475O23.2
RP11-713P17.3
CTC-137K3.1
linc-NKX2-2-1
linc-CSTB-1
RP11-90P5.4
RP11-803D5.4
CTB-35F21.1
RP11-400K9.3
CTD-2535I10.1
linc-NOL4-2
RP11-638L3.3
RP11-101E14.2
RP11-81H14.2
linc-UROD
RP1-90L6.3
RP11-85I21.1
RP11-82O18.2
RP11-501O2.5
RP11-401O9.3
RP3-388E23.2

RP11-98F14.11
linc-RTL1-2
RP11-672A2.4
RP11-390K5.3
RP11-445N18.7
AL589743.2
RP11-757G1.5
RP5-1160K1.8
linc-MAPK13
RP11-297L17.2
AC010145.3
RP1-296L11.1
CTD-2525I3.6
linc-GPM6B
linc-BAI3-4
linc-MFSD9-6
RP11-256L6.3
RP11-928F19.5
RP1-228H13.5
RP11-108M12.3
linc-DYNC1LI1-2
RP11-338K17.8
linc-MAD2L2
linc-LCP1-2
linc-AMD1-2
AC010907.2
CSNK1G2-AS1
AC144525.1
linc-PCDH8-9
RP11-47G4.2
linc-VWC2-3
RP4-584D14.5
linc-TNFAIP8L1-1
linc-LINS-4
RP3-414A15.2
linc-TTC7A-1
RP5-837M10.2
RP5-837J1.4
linc-FBXW4-3
RP11-744K17.2
linc-LARGE-6
LINC00176
AC018799.1
RP11-540O11.4
RP11-697E14.2
AP000282.2
RP11-252A24.5
linc-FREM2
linc-MTFR1-1
AC010148.1

FAM27L
RP11-946L20.2
RP11-77A13.1
RP11-276M12.1
linc-HEXB-2
RP11-720L2.4
linc-ARFGEF2-4
linc-PTBP2-3
ITCH-IT1
AC005559.2
RP5-1065P14.2
CTD-3113P16.5
linc-NLRP1-1
AC007277.3
RP11-515O17.2
KCNAB1-AS2
RP11-256P1.1
TNRC6C-AS1
linc-NR3C2-2
RP4-591L5.1
RP11-129I19.2
RP11-42A4.1
RP11-622A1.2
linc-GABRA5-2
CTD-2329C7.2
linc-IL1R1
AC013460.1
KB-1043D8.8
RP11-107I14.2
AC004790.3
CTD-2339L15.3
RP11-734K21.5
AC005029.1
linc-PAXIP1-1
linc-SSTR4-3
RP11-90B9.2
RP13-494C23.1
RP1-140K8.1
AP002954.3
CTD-3126B10.1
RP11-789F5.1
linc-KLRC1
RP11-359D14.2
CTD-3128G10.6
LINC00312
AC005550.3
AC005356.1
RP5-827L5.2
LL22NC03-2H8.5
RP11-218M11.1

linc-PAH-1
linc-CCDC99
AP001476.4
linc-MFSD2A
linc-ANKRD55-4
linc-DCAF4L1
RP11-85M11.2
linc-ASB7-4
RP11-959I15.4
AC145123.2
linc-DYRK4
RP11-353N4.5
RP11-565F19.5
linc-CCDC146-2
linc-LPHN3-5
AC074212.5
linc-RELN
RP4-646N3.1
RP11-54O7.2
AC012457.2
linc-CCDC37-4
linc-SRD5A2-3
RP11-429J17.4
RP11-204N11.2
linc-ARFGEF2-5
CTD-3193O13.8
RP11-88I18.2
RP11-227D2.3
CTD-2034I21.2
linc-SLC35B4-2
RP11-457M11.2
AC142119.1
CTD-2089N3.1
CTD-2081C10.1
linc-CYP24A1-1
RP11-436F9.1
RP11-513G11.2
linc-ADRA2C-2
RP11-164J13.1
CTD-2535L24.3
MIR4500HG
CTD-2145A24.4
RP11-244N9.6
linc-MTHFSD-1
RP5-1184F4.5
linc-IQSEC2
CTD-2369P2.2
AC015969.3
linc-PLCH2
AC009227.2

linc-ZNF337-5
linc-MCM2-1
RP11-991C1.1
RP11-482E14.2
AC078852.2
RP11-724M22.1
AC105393.1
RP11-359E8.3
MIR4321
LA16c-329F2.1
RP11-1079H9.1
linc-CALCB-1
linc-TPCN2
linc-GPR65-2
RAI1-AS1
linc-HSPB8
linc-AGGF1-2
linc-FUBP1
KB-1639H6.4
linc-SLITRK5-15
CTB-70G10.1
linc-GPR85
RP11-372M18.1
CTD-2353F22.1
CTC-498J12.3
RP11-173A16.1
linc-GALC-3
RP11-649A18.5
RP11-336A10.2
CTD-2316B1.1
CNTN4-AS1
SNORD62A
VIPR1-AS1
AC083900.1
RP11-2K6.2
RP11-112L7.1
CTD-2006H14.2
RP11-141J10.1
AC113617.1
RP11-102N11.1
CTC-559E9.8
linc-HRH1
linc-MCTP2-6
RP11-109D20.1
linc-MARK1-2
RP11-600L4.1
linc-GPRC5C
CTC-505O3.2
TPTE2P6
RP11-669N7.3

RP11-359E19.2
AC017006.2
RP11-930O11.2
DLGAP1-AS3
AC104134.2
AC139100.3
linc-PLAC1L-2
RP11-528A4.2
CTB-32P11.1
RP4-798A10.4
RP11-111M22.4
RP11-4N23.1
RP4-559A3.6
linc-ARFIP1-7
linc-MAN1A2-2
AC097532.2
linc-C15orf42-1
RP11-1007O24.2
RP3-326I13.1
linc-ALDH3B1-2
RP11-797E24.3
RP11-20I23.7
RP1-102G20.5
linc-SYNC-1
CTD-2562J15.4
linc-G2E3-4
CTD-2267D19.6
linc-SNTG1-3
RP11-480D4.6
RP11-648K4.2
RP11-295P9.8
RP11-357K6.2
RP11-323C15.2
RP11-10A14.3
CTD-3064M3.7
linc-GADD45G-2
CTB-180C19.1
RP11-340I6.7
CTD-2547G23.2
hsa-mir-7515
BCYRN1
RP11-375I20.6
AC006128.2
RP11-328K2.1
linc-ADCY2-1
RP4-816N1.7
RP11-445O16.3
linc-AGRP-AS
RP11-395I6.2
linc-ERG-8

linc-CXorf49B-1
RP11-195B3.1
KCNMB2-IT1
AC133106.2
RP11-382A20.6
RP5-890E16.4
linc-C10orf113-2
linc-DACT2-5
linc-ENPP4-2
linc-IRX4-2
RP11-565N2.1
KB-1460A1.5
CLYBL-AS2
RP11-467I17.1
RAPGEF4-AS1
CTD-2132N18.4
AC002398.11
RP5-1021I20.6
linc-ADD3-1
RP11-58K22.1
CTD-2308B18.2
RP11-279O17.1
RP11-16N2.1
GS1-124K5.11
RP3-403L10.3
RP11-567L7.6
RP11-752D24.3
CTB-30L5.1
NRG3-AS1
RP11-495K9.5
linc-AGTR1-5
RP11-19O2.2
RP11-435B5.6
RP11-779P15.2
RP11-57A1.1
CTA-363E6.2
linc-VAT1L
RP11-190D6.2
linc-EGFL6-2
linc-JAG1-2
RP11-410C4.4
RP11-58E21.4
RP11-388E23.2
RP11-770E5.1
RP1-104O17.1
KB-1991G8.1
RP11-246K15.1
RP11-169N13.4
RP11-324E6.6
CTD-2035E11.5

linc-ACSM5-5
linc-EXOC7
linc-GXYLT2-2
VWC2L-IT1
linc-MTIF3
RP1-136B1.1
RP11-78B10.2
CTD-3088G3.6
AC005041.17
RP11-205K6.2
CTD-2568A17.5
RP11-127L21.1
linc-OR4F16-5
linc-C4orf45-1
linc-PRKAA2-5
linc-AIDA
RP11-229P13.15
RP11-384J4.1
LINC00404
XXbac-BPG181B23.7
AP000688.8
linc-SGCG-7
RP11-109E10.1
linc-RABL3-2
RP11-73M18.8
CTA-481E9.3
RP5-859D4.3
linc-LMO2-1
linc-NETO1-5
linc-PLD5-4
linc-CELF4-9
RP5-1029F21.4
linc-LRRC33
RP11-25I11.1
linc-EHD3-1
FAM230B
linc-SAP30
linc-PHACTR1
RP11-267C16.1
RP11-57H14.3
linc-CDH5-4
AP001055.6
linc-IGFBP7-6
RP11-305B6.3
RP11-442O18.2
RP11-1C1.6
linc-FAM120B-7
CLDN10-AS1
RP11-571O6.1
RP11-125K10.5

CTD-2049J23.2
RP11-436D23.1
linc-C4orf41
RP11-622O11.4
LINC00444
RP11-13N13.6
RP4-773N10.4
linc-FPGT-11
RP11-478H13.3
AC107081.5
RP11-474D1.2
RP11-124I4.2
RP11-344A16.2
CTC-347C20.2
linc-NAA16-1
linc-ZFP42-7
linc-KCNQ2
linc-TSPAN2
linc-ZNF507-3
RP11-187C18.2
RP11-332M4.1
RP5-1057J7.7
CTB-111H14.1
RP11-219G17.4
linc-GRIP1-4
AF064858.6
RP11-268G12.1
MYCBP2-AS1
RP11-508N22.9
linc-BCL6-5
RP11-143K11.5
RP3-355L5.4
RP11-537P22.2
RP11-737O24.1
LA16c-316G12.2
linc-NDST3-4
AC078882.1
CTC-523E23.6
AC004125.3
linc-JAKMIP1
ERI3-IT1
RP11-791O21.5
CTB-120L21.1
RP11-689P11.2
RP11-173P15.7
RP11-380P13.1
RP11-182J1.5
linc-LZTS1-1
linc-VWDE-2
CTC-471F3.6

RP11-208K4.1
RP11-274H2.3
RP11-503E24.3
CARS-AS1
RMDN2-AS1
AC017104.6
RP4-799D16.1
RP11-338L22.2
RP11-488L18.10
RP11-754N21.1
CTD-3149D2.3
AC006042.6
AC078842.3
linc-BSPRY
linc-FRG1-11
CTD-2251F13.1
RP11-219A15.2
CTB-191K22.5
CTA-256D12.11
linc-HAAO-5
linc-SEL1L-5
linc-PPIAL4A-5
RP11-342K6.2
CTD-2520I13.1
AC007403.1
CTD-2281E23.3
linc-LOC388946
CTD-2586B10.1
linc-PLCE1-1
RP11-149I23.3
RP1-302D9.4
AC017048.1
MYO16-AS2
RP11-546O6.4
linc-PCDH20-7
RP11-1029J19.4
LINC00269
RP11-627D16.1
RP11-171N4.1
LINC00366
linc-C13orf34-7
AC106801.1
linc-SLC1A4-1
linc-NBPF3-1
RP11-97O12.7
RP11-277B15.2
RP11-428J1.5
RP5-912I13.1
RP5-837I24.2
RP11-650L12.2

LA16c-425C2.1
RP11-423O2.7
AC007040.7
linc-TRA2A-3
EHMT2-AS1
CTB-83J4.1
RP11-328M4.3
LINC00645
AC009505.4
Z83307.3
FAM66D
RP11-735B13.2
LINC00355
RP1-77H15.1
AC093063.3
linc-SLC30A5-2
LINC00866
linc-ASNSD1
RP11-93B14.4
CDRT7
RP11-421N8.2
ASH1L-IT1
linc-LRP1B-2
ATG10-IT1
RP11-10J21.6
RP11-113I22.1
RP5-1142J19.1
AC113144.2
ENOX1-AS2
RP11-510C10.2
LINC00583
RP11-398H6.1
RP11-1141N12.1
linc-LRRTM3-5
RP11-173B14.4
CTA-221G9.7
AC112518.3
CTC-359M8.3
RP11-65L3.2
CTD-2196E14.6
linc-SLITRK5-10
RP11-923I11.5
linc-MIXL1-1
linc-NR1I3
RP11-2A4.3
AC004490.1
LINC00434
RP11-38C18.2
RP3-399L15.1
RP5-905H7.3

RP11-249C24.11
RP11-1348G14.4
linc-NTM-8
RP11-2E11.5
linc-ARHGAP26-5
GPC5-AS2
RP11-498C9.2
RP11-177N22.3
linc-IRX2-2
AC005943.5
linc-FAM84A-4
linc-GALNTL6-4
linc-OR51F2-2
linc-CTDSP2-3
SCARNA9
linc-TMEM154
RP11-612B6.1
RP11-438C19.1
linc-TMEM132C-6
RP11-71G12.1
linc-NTN1-4
linc-FLJ44606-2
RP11-447M12.2
CTD-2066L21.1
RP11-429B14.3
linc-ZC3H12B-2
linc-WDR7-5
CTD-2529P6.3
RP11-217E13.1
AL589986.2
RP11-662M24.2
RP3-380B8.4
AC010468.2
RP3-414A15.11
linc-LRRC6
linc-OR2D2
CTD-2247C11.4
RP11-84C10.4
ALMS1-IT1
linc-P2RY2-2
linc-RNASEH1-1
AC006150.1
linc-ARHGAP28-1
RP13-560N11.1
RP11-376M2.2
linc-PRSS3-1
linc-PZP-3
linc-RELL1-4
RP11-411B10.2
PLSCR5-AS1

GS1-24F4.2
CTB-104H12.6
RP11-321E8.3
linc-ZFP42-1
RP11-760N9.1
RP11-120C12.3
AC013727.1
RP11-262K1.1
RP11-805I24.1
linc-BRAP-1
linc-MARCKS-4
CTD-2542C24.8
CTD-2314G24.2
RP11-1101H11.1
linc-PROM2-1
RP11-203J24.8
RP13-991F5.2
linc-ACTB
linc-ITGA4-2
RP11-85O21.4
linc-C12orf40-3
LINC00453
AP000479.1
linc-VCAN
RP11-426L16.3
RP1-79C4.1
RP11-717I24.1
RP11-351M8.2
RP11-663P9.1
RP11-370K11.1
linc-NCKAP5
RP11-85B7.2
linc-GRM1
AC114788.2
NHS-AS1
ERVMER61-1
RP11-59O6.3
RP11-157P23.2
RP11-408E5.5
LINC00303
IDI2-AS1
linc-DLC1
linc-C10orf57-2
PHKA2-AS1
RP11-344G13.1
CTD-2541J13.2
RP11-386G11.3
linc-CLVS2
linc-MYO3A-2
CTD-2378E21.1

RP11-57H12.2
LINC00222
RP11-245J24.1
RP11-61G23.1
RP11-650J17.1
RP11-517B11.7
RP11-461K13.1
RP1-290I10.7
linc-FAM75A6-4
RP11-2A4.4
RP11-467C18.1
RP11-223A3.1
RP11-553E24.2
linc-C16orf68-2
CTD-2349P21.11
RP5-903G2.2
AC022816.2
CTD-2374C24.1
RP11-80F22.15
RP11-134J21.1
linc-C1orf43-2
RP11-473J6.1
linc-IFNK
AC012370.2
RP11-39M21.1
RP11-3B12.3
linc-WHSC2-1
AC007381.2
linc-ABHD6
RP11-566H8.3
KB-1047C11.1
RP11-143J24.1
BIRC6-AS1
linc-FABP5-2
RP11-802O23.3
RP11-96D1.3
linc-POM121L12-2
CTB-134F13.1
ESRG
RP3-511B24.6
linc-EBF3-4
linc-PTP4A3-1
RP11-849F2.9
LINC00448
linc-IRS2-2
linc-AKT3-4
RP4-657D16.6
linc-CSMD1-4
AC006129.4
RP11-344F13.1

linc-HDAC4-2
RP11-37C7.2
RP1-102D24.5
CTC-436K13.1
RP11-212D3.3
RP11-707A18.1
linc-DHX37-12
CTD-2283N19.1
RP11-550H2.1
SNORA40
RP11-417O11.5
AC074389.9
linc-SCRG1
RP11-30L8.1
SNRK-AS1
RP5-1125A11.4
SLC6A1-AS1
RP11-574H6.1
linc-NAV3-1
RP11-379B8.1
RP11-318L16.6
ERICH1-AS1
linc-IRX3-1
RP11-333I13.1
RP11-561O23.8
AC007036.4
AY269186.2
linc-CDR2L
RP11-30J20.1
RP4-776F14.3
RP11-555J4.4
RP3-340B19.3
AP001062.8
LINC00958
linc-GRIA4-2
RP5-947P14.1
AF241725.6
AC009499.1
RP11-229P13.25
CTD-2252P21.1
linc-COL9A2-2
linc-C9orf123-2
linc-CYR61-1
RP11-325K19.1
linc-C9orf66-5
CTD-2533K21.4
RP11-725D20.1
AP000281.2
CTD-2529O21.2
CTB-43E15.2

RP11-227G15.9
RP11-103C3.1
linc-ZNF382-3
CTD-3092A11.2
linc-TMEM189-2
CTD-2247C11.3
RP11-554A11.8
RP1-251I12.1
linc-ZNF467-1
RP11-443B20.1
linc-HS6ST1-5
RP5-1121A15.3
RP3-454B23.1
linc-PRR20A-4
RP5-1087E8.3
RP5-836N10.1
RP11-360I2.1
linc-SLC22A15-1
linc-ZBTB32
RP11-204P2.3
linc-MBOAT1-5
ITIH4-AS1
CTC-339F2.2
RP11-10O22.1
RP11-5L12.1
AC004744.3
linc-C17orf97-3
CTB-164N12.1
CTB-33G10.11
CTD-2517M22.14
AGAP1-IT1
RP4-735C1.6
linc-SIK1-2
linc-DHRS7B-1
RP5-1160K1.3
linc-FOXC1-3
CCDC13-AS1
RP11-445O3.3
RP1-272J12.1
AC107057.2
DPYD-AS2
CTC-444N24.11
RP11-245J9.6
NPPA-AS1
AC079354.5
CTC-465D4.1
AC010149.4
GS1-179L18.1
linc-DTHD1-8
RP11-328K4.1

RP11-508N12.2
linc-FAM113B-2
linc-FAM19A5-2
RP11-324J13.2
linc-ODZ4-3
RP11-673C5.4
linc-APPL2-2
AC009495.3
linc-RXRA-2
RP11-728G15.1
LINC00862
RP11-594N15.2
LINC00350
linc-OBSL1-1
linc-C13orf34-14
RP11-12M5.4
RP11-282O18.3
XX-C283C717.1
RP11-193H5.1
linc-ARID1B-3
RP11-168L7.3
RP1-137D17.1
RP11-486L19.2
RP11-576D8.4
RP11-440I14.2
RP11-20J15.3
IGBP1-AS2
RP11-573J24.1
RP11-13K12.5
linc-CTDSP2-4
AC096579.13
linc-RPL38-1
RP11-218I7.2
RP11-815J21.4
CTD-2560E9.5
linc-DPYD-1
AC016751.2
RP11-383B4.4
linc-ACTBL2-2
RP11-356I2.1
RP11-30O15.1
AC092171.4
AC011747.4
RP11-288L9.1
RP11-346D19.1
RP11-360A18.1
AC019100.3
linc-WFDC2-1
AC005754.8
RP11-46A10.4

RP11-498C9.16
linc-ASB5-1
linc-TP53TG3B-8
linc-LCTL-2
LA16c-390E6.4
linc-ADAMTS9-3
linc-DYRK2-2
linc-HERC1
RP11-536N17.1
RP11-101P17.11
RP11-514D23.1
RP11-109P14.9
HS6ST2-AS1
linc-NIPBL
PRSS29P
RP11-577G20.2
RP11-442J21.1
RP11-106E15.1
RP11-239E10.3
linc-CD276
linc-DNAH6-1
linc-C6orf120-8
RP1-104O17.2
RP11-834C11.5
linc-LCLAT1-2
AC000123.4
RP5-1021I20.2
AP001626.1
RP11-111A21.1
RP11-158M2.4
GYG2-AS1
RP5-1069C8.3
RP11-10H3.1
linc-DEFA4
CTD-2340E1.2
RP1-86D1.3
RP11-340A13.2
GS1-204I12.1
RP1-257A7.4
RP11-727A23.11
RP11-120M18.5
RP4-704D23.1
linc-PTPRD-4
RP11-357N13.3
linc-ODF2L
RP11-299H22.7
AC144521.1
RP11-159D8.1
RP11-24P14.1
RP11-98J9.1

RP11-151N17.1
RP11-435B5.7
RP11-185E8.1
RP1-86D1.5
RP11-394O9.1
CTD-2246P4.1
CTC-428G20.2
linc-BLCAP-2
RP11-495P10.5
linc-FAM43A-5
linc-CDH23
linc-DUSP22-4
RP11-309H21.2
RP11-737F9.1
linc-FAM110B-6
RP5-921G16.1
RP11-38L15.8
RP11-284A20.1
linc-RBM15
linc-ROPN1B-2
RP11-543C4.3
RP11-461F11.1
CTB-25B13.9
linc-MAP1LC3B2-11
SPON1
AP000345.2
linc-HNRNPA2B1-3
RP11-542G1.2
linc-SERTAD2-2
linc-GZMK
linc-RASD2
RP4-562J12.2
linc-CBWD6-3
RP11-1049H7.2
linc-ITGB2-4
linc-ZNF354A
AC009473.1
AC016629.3
CTD-3023J11.2
RP11-234K19.1
linc-PLCL2-2
linc-PAX9-3
linc-ZNF311
KB-1410C5.2
CTD-2307P3.1
RP11-6E9.4
OPCML-IT2
AC003077.1
CTD-2308N23.2
RP11-127I20.7

RP11-38P6.1
CTD-2116N24.1
AC005863.2
linc-VRK2-1
linc-GCNT2-2
RP4-791M13.5
linc-CITED4
RP11-778O17.4
CTD-3046C4.1
RP5-1112D6.8
RP11-181F12.1
linc-GNAZ
CTD-2296D1.3
CTD-3185P2.1
XKR5
RP11-256I23.3
RP11-195M16.1
RP1-161P9.5
linc-IRF2-1
linc-NOVA1-4
RP11-1437A8.3
linc-ZCCHC17-7
TSSC1-IT1
linc-ZNF507-1
RP11-431N15.2
RP11-563D10.1
CTA-796E4.3
CTC-503J8.4
CTA-360L10.1
RP11-6N17.1
CELF2-AS1
linc-ANXA8L2-1
linc-KRTAP5-11
RP11-297C4.2
LA16c-380H5.3
NKX2-1-AS1
RP11-65D17.1
linc-HDDC2-3
RP11-58O3.2
RP13-895J2.3
RP11-454K7.3
RP11-526J3.3
RP11-65M17.3
RP11-157D23.2
RP11-276A18.2
DLX6-AS2
linc-GALNT5-1
SMYD3-IT1
RP11-175I6.5
RP11-630D6.5

FAM155A-IT1
RP11-686D22.9
XXbac-BPG308J9.3
KB-1836B5.1
RP11-317O24.2
RP11-799D4.3
RP11-446J8.1
RP11-20G13.2
AC010649.1
RP11-138P22.1
RP11-527H14.3
RP11-183E24.2
RP11-452H21.2
AC007161.5
linc-OR4M2-2
linc-ZNF99-3
RP11-702H23.6
RP11-397O4.1
RP11-240M16.1
LINC00700
linc-XRCC4-2
PWRN1
RP4-782G3.1
RP11-1C8.4
AC007680.2
AC114877.3
linc-DLGAP2-8
RP11-199B17.1
RP11-193M21.1
RP11-255C15.3
RP11-305E6.4
RP11-856M7.1
linc-DHRS2-1
RP4-718D20.3
RP11-25G10.2
AC009518.4
RP11-927P21.11
linc-API5-3
RP11-256L11.3
LAMTOR5-AS1
LINC00561
RP11-395E19.5
LINC00411
RP11-377G16.2
AC010525.7
MIR4454
AC006003.3
linc-C18orf21
RP11-369F10.2
linc-RCBTB2-3

LINC00570
RP11-219E7.4
RP11-322I2.1
CTC-205M6.5
RP1-288H2.2
AC011891.5
linc-CDYL-1
AC037445.1
RP11-324F11.1
RP11-1M18.1
ZNF32-AS1
RP1-167G20.1
LINC00473
GNAS-AS1
linc-CD93-2
RP11-597M17.1
RP11-605F22.1
AC067960.1
RP11-95M15.1
RP11-461L13.5
linc-SCAMP1-2
RP11-1094M14.14
RP11-538D16.2
FAM226A
linc-C12orf74-1
linc-TCP1-1
RP11-711C17.2
AC006041.1
CTD-2061E19.6
linc-GOLGA6L1-1
linc-SAMD11-8
linc-BCL2L10
RP11-129B22.1
linc-C10orf107-1
AC008069.1
RP11-729I10.2
RP11-337C18.9
linc-PARK2-3
AP000696.2
RERG-AS1
RP1-257A15.1
linc-ZNFX1
linc-VSTM2B-4
CTD-2560K21.6
linc-EVI2A
RP11-415J8.3
linc-SAMD12-2
RP11-143N13.2
linc-HNRNPA2B1-1
RP11-13A1.1

RP11-206M11.7
linc-PDE10A-1
linc-GAGE10
RP5-842K16.1
RP11-780O24.2
RP11-320G24.1
RP11-384P14.1
RP6-74O6.3
RP11-863P13.6
RP11-791M20.1
linc-MYEOV-1
linc-GPR173-2
linc-GLA-1
RP11-150D5.2
CTD-2002H8.2
RP11-646E18.2
RP11-341A22.2
F10-AS1
ATP6V0E2-AS1
RP11-436H11.5
RP11-211N11.5
RP1-213J1P__B.2
linc-TMEM132D-4
RP1-171K16.5
RP11-120K24.5
RP11-298O21.7
RP11-407A16.7
RP11-115I9.1
RP11-785H20.1
AC104135.3
CTD-2527I21.14
linc-TNFSF11
RP11-706C16.7
linc-IRS2-1
CTD-2306M10.1
PLCE1-AS1
LINC00462
linc-PTPN1-1
linc-C13orf31-1
CTC-548H10.2
linc-SEC22C-1
RP11-255A11.2
RP11-17E2.2
AC007950.1
RP11-116N8.4
linc-C15orf2-11
RP11-782C8.1
linc-TPBG-1
CTB-43E15.3
linc-PVALB-1

RP11-740P5.3
FAM157C
RP11-77G23.5
RP11-301G19.1
linc-NUDT10
linc-ZNF236-6
CTD-3193O13.13
linc-HIST3H3
linc-FAM38B-3
RP11-196I18.4
linc-GCNT2-9
linc-TCF7L2-3
linc-GPHN-3
linc-USP12-3
RP11-616M22.2
RP11-483E7.1
CTD-2201E9.2
linc-SPAG16-1
RP11-420B22.1
RP11-260E18.1
RP11-108P20.2
PLCB2-AS1
RP11-133N21.10
CTD-2013N17.4
RP11-383G10.5
AC091962.3
linc-IFT74-4
RP11-742B18.1
ALG9-IT1
linc-BASP1-3
linc-UGT2B15
KLHL6-AS1
RP11-79P5.5
CTD-2319I12.1
RP11-712P20.2
AC018730.1
RP11-123M6.2
linc-NFIA-1
RP11-546K22.3
KIRREL3-AS3
linc-RASL10A
RP11-406A20.1
linc-NGFR-4
RP11-543D5.1
RP11-463D19.1
RP11-60L3.1
RP11-1O2.1
RP11-505E24.2
RP11-217L21.1
RP11-361H10.3

linc-SPRY1-2
linc-PCDH7-7
linc-ARFGEF2-13
RP11-193F5.1
AC018693.6
SNORA11
RP11-138H8.6
AC066593.1
RP11-168F9.2
RP11-344B5.3
RP11-11C20.3
linc-SLC35B4-3
CTB-78F1.2
RP11-496N12.6
AP001065.2
RP11-134K1.3
AC078941.1
RP5-826L7.1
AC096669.3
linc-NKX2-5-2
RP11-298A8.2
linc-CNTLN-5
AC008937.2
AC025918.2
RP4-777D9.2
linc-GAS1-3
AC018359.3
NEBL-AS1
RP4-782L23.2
linc-PSMD11
CTD-2218K11.2
LINC00870
AC114730.11
RP11-429O1.1
linc-SHISA3
linc-GLI2-2
AC009411.2
AC083843.1
linc-VPS8-1
LINC00703
CTA-407F11.7
RP11-526I2.1
RP11-111M22.5
RP11-452C8.1
linc-CDH11-4
linc-ZNF727-1
RP11-830F9.7
RP11-499P20.2
linc-PCDH18-1
linc-POTEB-3

RP11-56L13.7
RP11-18J9.3
linc-ACTL7A-6
CTD-3220F14.2
RP11-16O9.2
KCNQ1DN
CTD-3074O7.2
linc-LIPJ-2
AC020743.2
RP11-523O18.5
LINC00519
RP11-97C16.1
RP11-277J6.2
RP11-63D14.1
CTD-2620I22.3
CTB-25B13.12
AC096559.2
linc-PPP1R3B-2
RP4-799P18.2
linc-IP6K2
RP11-121J20.1
linc-ERCC4-1
RP5-943J3.1
IQCF5-AS1
MKNK1-AS1
RP1-288L1.5
LINC00951
RP11-407H12.8
linc-DEFB104B
linc-DKK3-1
LINC00967
C9orf106
RP5-1077I2.3
RP11-49K24.8
RP11-366M4.13
linc-JARID2-1
AC051649.12
RP11-68L1.2
linc-MAEA-4
RP11-978I15.10
linc-CCRN4L-8
LINC00319
RP11-164N3.1
RP11-661P17.1
linc-C14orf101-3
RP11-15K2.2
linc-SGCG-3
RP11-46A10.2
RP13-1039J1.2
linc-IDI1

RP11-254F19.2
RP11-445H22.4
PRICKLE2-AS1
RP11-847H18.2
RP11-412B14.1
AC002550.5
RP13-638C3.2
ELOVL2-AS1
RP11-512N4.2
linc-NTN1-2
linc-FAM75A7-8
RP11-964E11.2
linc-C9orf79-1
linc-MKI67-4
RP11-415D17.4
RP11-529H20.6
RP11-524P6.1
AC092597.3
RP11-429B14.1
UBXN8
KB-1592A4.15
AC022431.3
RP11-731F5.1
linc-AMY1A-4
linc-DMGDH-2
CTB-73N10.1
RP11-31L22.3
RP11-9L18.3
RP1-37J18.1
linc-TMEM132D-6
linc-SLC16A7-2
linc-RPS23-2
RP11-110L15.1
RP11-534L6.2
EGFLAM-AS3
linc-CEP110-8
RP11-468D11.1
AC091133.1
AC005895.4
LINC00624
RP11-700E23.1
RP11-671J11.7
AC073628.1
RP11-327F22.4
RP11-144O23.18
linc-EN2-2
RP1-292B18.3
RP1-28O10.1
RP11-706J10.1
RP11-434D2.2

AP005530.1
RP11-663N22.1
RP4-564F22.5
linc-PDE3A-3
RP11-275I4.1
RP3-329E20.2
linc-NAA35-1
RP11-71J2.1
RP11-90P5.7
RP11-498B4.5
linc-DCAF17-1
linc-ANO1-1
RP11-283C24.1
linc-RGMA-16
RP11-632K5.3
linc-PPARGC1A
linc-COL1A1-1
RP11-300J18.3
CTD-2311B13.5
AC011625.1
linc-KCNK13
linc-SLC30A4-1
linc-FICD
RP11-348J24.2
RP11-875H7.5
AP000797.3
linc-FAM72B-3
linc-TCERG1L-3
RP11-589N15.2
linc-TFAP2C-3
AC018755.17
RP11-1082L8.3
AC005538.5
RP11-782C8.2
linc-ASCL1-2
linc-GRIN3A-3
RP11-356B19.11
linc-EXOC4-4
linc-ADRA1B-2
RP11-439L18.3
RP11-5N11.2
RP11-137H2.6
RP1-63G5.7
RP11-84A19.2
RP11-386B13.3
linc-ODZ3-6
RP11-1114I9.1
AP001605.4
RP11-432I5.2
RP11-432J24.2

CTD-2292M16.8
linc-ARHGEF38-2
linc-STK39-1
RP11-580I1.2
KRBOX1-AS1
linc-CPXM2-1
RP11-770E5.3
LARGE-IT1
RP5-1103B4.3
linc-CEP110-7
RP11-73M18.6
RP11-826N14.4
linc-DNAJC19-5
KIAA0196-AS1
linc-FAM20A-1
linc-OXNAD1
linc-TRPM5
RP11-541G9.2
RP11-141J13.3
linc-RBM7
RP11-230C9.4
LINC00446
RP11-543A18.1
CTA-125H2.1
RP11-701H24.2
RP11-662B19.2
linc-FAM84A-3
RP11-809C18.1
AC093642.3
linc-SLC12A7-1
RP11-239L20.6
AC091153.4
CTC-235G5.3
RP11-148B6.2
linc-NOX3-1
RP11-1217F2.15
CTC-499B15.6
RP11-473L15.2
RP4-734G22.3
MED4-AS1
RP11-993B23.3
linc-ATP13A4-8
RP11-84D1.1
linc-BMP7
CTD-2144E22.6
RP1-65P5.3
RP11-463I20.3
linc-TMEM132C-10
AL928742.12
linc-PTGR2

RP11-733C7.1
RP5-1114G22.2
linc-GTPBP4-1
linc-PPP2R2B-2
linc-TMC3-3
linc-FTMT-3
CTD-3194G12.1
RP11-215H22.1
RP1-105O18.1
AC027269.2
RP11-326L2.1
RP11-44F14.11
RP11-20B24.7
RP11-485O10.2
RP1-269M15.3
RP11-36D19.8
linc-CDKN3-1
RP11-318G21.3
RP11-1000B6.2
CTC-436P18.3
AC124944.3
RP11-642C5.1
CTB-35F21.3
RP13-143G15.4
AC005498.4
AC064852.4
linc-TAGAP-1
RP11-292F9.2
linc-PMFBP1-2
AC005392.13
linc-ETV3-1
CTB-102L5.8
RP11-255G12.2
RP11-543H23.2
linc-DPPA3
RP11-669I1.1
RP11-780K2.1
RP4-594L9.2
RP4-800F24.1
linc-PTPRG-1
HPN-AS1
RP11-570H19.2
RP11-242F24.1
RP11-420L9.4
AC131056.5
AC018462.2
Z83001.1
RP11-767C1.2
RP11-125P18.1
AP000438.2

RP4-657D16.3
RP11-715J22.3
RP11-48O20.4
RP4-543J13.1
RP11-263K4.3
RP11-513N24.1
RP11-413P11.1
CTD-2135D7.3
RP11-157E21.1
CTC-273B12.8
RP11-345P4.7
RP1-293L6.1
RP11-402P6.9
linc-CD300C
NAV2-AS5
RP11-1060G2.1
RP11-665J16.1
linc-CBLB-10
CTD-2207P18.1
RP11-812E19.3
linc-CELF4-4
RP11-304L19.4
RP11-202K23.1
RP11-218E20.2
linc-OLIG2-2
LINC00388
RP11-566E18.3
linc-EMB-3
RP11-418I22.2
RP11-165M1.3
RP4-544H6.2
linc-IL4R-1
AC002398.5
RP11-142J21.2
RP13-122B23.8
RP3-500L14.2
AC004951.6
CTB-187M2.2
RP11-252K23.1
RP11-345M22.1
RP4-614C15.3
RP11-562A8.4
AC097713.4
RP11-471M2.2
RP11-214K3.24
linc-MAP1LC3B2-13
U6
RP4-583K8.1
linc-TCTE3-3
RP11-296O14.3

linc-EFHA1-5
RP11-184A2.2
CTD-2260A17.1
RP1-5O6.4
linc-TMEM41B
linc-CARD11-1
RP3-467L1.4
RP11-84E24.3
linc-DHX37-6
AP006285.6
linc-CEP290
linc-GJA1-1
RP11-293E1.1
RP11-528G1.2
PRKX-AS1
RP11-301L7.1
RP11-558A11.2
RP11-148O21.2
linc-RHD-2
RP11-118M9.3
ITPK1-AS1
linc-BMP4-1
RP13-329D4.3
RP11-348P10.2
linc-LRGUK-2
RP11-672A2.6
RP11-100K18.1
RP11-25E2.1
RP11-340B18.1
linc-SULF2-10
RP11-804A23.1
RP11-665C14.2
AC005597.1
RP11-358B23.5
linc-C19orf12-1
linc-ISLR2-2
AC135050.5
AC067969.2
RP11-538C21.1
RP4-760C5.5
RP4-610C12.1
linc-LYRM7-2
linc-EXOSC9-6
LINC00838
LINC00458
linc-ANKRD10-4
RP11-51J9.4
RP11-981G7.2
RP11-27M9.1
AC091729.7

linc-WDR5
CTD-2531D15.4
linc-ATP10A-1
RP11-358L4.1
CTC-261N6.3
RP11-431K24.3
linc-PRR20A-1
RP13-884E18.2
linc-CDH6-9
SAPCD1-AS1
linc-NDUFV2
RP11-565F19.3
RP11-138M12.1
linc-RTN4
RP5-1166F10.1
linc-CPEB2-13
RP11-88H12.2
AC093850.2
linc-ERI1-1
RP11-711K1.8
linc-ANO5-2
FAM74A2
RP11-1058N17.1
CTD-2373J6.1
CTD-2210P24.2
RP11-687M24.7
linc-CELF2-3
RP11-139B1.1
CTB-92J24.3
RP11-429J17.2
SNORD45
RP11-74K11.2
RP11-659E9.2
linc-KLRD1
IL21R-AS1
LINC00610
RP11-51B13.1
LINC00052
RP11-292D4.2
CTD-2008L17.2
CTD-2007L18.5
FER1L6-AS2
linc-CLRN2-3
RP11-1149O23.4
CTB-111F10.1
linc-FBXO32
RP11-732A19.6
RP11-495K9.3
AC005682.8
AF127577.10

linc-USP47-2
RP11-708L7.6
CTD-2012I17.1
RP11-424D14.1
AC097382.5
RP11-122C5.1
AC020594.5
RP4-784A16.3
AC004878.8
RP11-519M16.1
RP11-680F20.10
AC007228.9
STAU2-AS1
linc-ERICH1-6
RP11-219F10.1
RP11-275F13.1
linc-VRK2-3
RP11-91J3.2
RP11-13E5.2
linc-TLL1-2
RP11-799B12.4
AC007285.7
RP11-962G15.1
RP11-566H8.2
RP11-20D14.3
RP11-476B1.1
linc-ADAM29-1
ZBTB20-AS3
RP11-476M19.3
CTD-2031P19.5
linc-MRPS18A-4
RP11-124O11.2
RP11-444D13.1
RP1-69D17.4
linc-TAOK3-5
RP11-349J5.2
CTD-3239E11.2
RP11-526A4.1
CTC-265F19.3
RP11-534N16.1
AC004920.3
TEX26-AS1
LINC00202-2
linc-YWHAQ-4
LINC00336
RP13-507P19.1
LINC00840
RP3-514A23.2
RP11-572M11.3
RP11-187A9.3

RP4-635E18.8
RP11-279F6.2
AC012485.2
AC011752.1
linc-AGBL4-2
linc-IGFBP3-2
RP6-65G23.1
AC008268.1
EMCN-IT3
linc-CHAC2-5
RP11-102F4.2
linc-ANO5-1
linc-VCAM1
linc-RREB1-7
RP11-181B11.1
CTC-436K13.3
AC090587.2
RP11-39H13.1
RP4-753D10.3
KB-1460A1.3
linc-SALL1-4
linc-ZNF673-3
linc-PRKCQ-1
LINC00501
RP11-473I1.9
linc-FOXP2-5
RP11-570L15.1
Z69720.2
RP11-20G6.2
RP11-756H20.1
linc-ERG-3
RP11-317N12.1
RP5-881L22.4
RP5-994D16.9
linc-PECI-4
AC005546.2
linc-FRG2C-4
linc-CDH5-2
linc-ZNF354B-1
RP11-328J2.1
linc-C1orf57-2
RP11-459E5.1
CTD-2240H23.2
RP11-26M5.3
CTA-503F6.2
AC006116.15
linc-LAMA1-7
RP11-254I22.3
SNORD56
linc-PHF20L1

linc-RTL1-7
RP11-95M5.1
AC004112.4
linc-NUCB2-1
RP11-44D19.1
RP1-15D23.2
AC105253.1
RP11-366O17.4
RP11-104J23.2
RP11-325D5.3
linc-CEP110-15
RP11-152L7.1
RP11-697H9.2
RP1-310O13.12
AF015720.3
linc-KIRREL3
RP11-285G1.2
linc-RNF11
RP11-169K17.3
RP11-307P5.2
RP11-319E16.2
AC005592.3
AC011893.3
RP4-550H1.5
LINC00028
XXyac-YX155B6.5
RP11-219E7.2
linc-IRX1-2
RP11-332E3.2
RP11-166D19.1
linc-CETN1-1
RP11-643A5.2
RP11-452J13.1
RP11-547C5.1
RP11-65M17.1
CTD-2561B21.4
AP000593.7
RP11-150O12.5
linc-C17orf108-1
linc-SLITRK1-2
RP11-113E21.1
RP11-649A18.7
RP11-49K4.2
linc-FGGY-4
AC122136.2
LINC00668
AC004069.2
RP11-120E13.1
CTC-537E7.1
linc-MUC2-1

linc-SHPRH-4
linc-POU3F1-1
KB-1184D12.1
RP11-434I12.2
RP11-66N24.4
RP1-178F10.3
CTD-2528A14.5
linc-COX7C-1
linc-PAX1-1
RP11-335O13.8
RP11-759A9.1
RP11-178L8.3
RP11-433J20.1
RP11-326A13.1
AC008163.4
APCDD1L-AS1
AC144836.1
RP11-449D8.2
RP11-399F4.4
linc-STAP1-7
RP11-329E24.6
RP11-356J5.4
RP11-58K22.4
RP11-10A14.5
linc-CMPK2-5
AC079354.3
linc-SYNPO2-1
linc-PARP8-2
AC005757.6
RP11-710C12.1
linc-TRIML2-2
RP11-430L3.1
RP5-1039K5.13
linc-SNX20-1
RP11-640A1.3
POU6F2-AS2
AC009303.1
linc-C6orf170-1
linc-AADAT-2
RP11-723D22.3
linc-NRSN1-2
RP11-15I11.3
linc-ZNF726-5
RP11-58G13.1
linc-GUCA2B
linc-PRM1-2
RP11-78A18.2
linc-CCDC39-1
RP11-137J7.2
AC104809.4

RP11-438F14.3
AL109763.1
GS1-465N13.1
linc-TNFRSF19-2
linc-IGFL4
RP11-616L12.1
linc-TTC15-2
linc-PFDN4-3
linc-PDE5A-2
RP11-774D14.1
GHc-362H12.3
linc-RAG2-2
RP11-455B3.1
RP11-258F22.2
RP4-676J13.2
RP11-787D18.2
RP11-702F3.1
linc-DHX37-14
AC116609.1
linc-APIP-2
linc-DUSP4-4
linc-NT5DC1-2
RP11-304F15.6
linc-TNC-2
linc-SORBS2-3
linc-TMEM188
RP11-395P17.3
linc-ALCAM-1
CTD-2308B18.4
RP11-153K16.1
CTC-451A6.4
RP11-79P5.7
RP11-99H8.1
RP11-358H18.2
RP4-718J7.4
CTC-542B22.2
AC073257.1
RP11-272L13.3
RP11-231G15.3
RP11-274H24.1
RP11-70P17.1
LncRNA
AGBL5-IT1
AL592494.5
AC092657.2
linc-IQCJ
CTC-548K16.5
linc-FAM120B-5
RP11-779P15.1
linc-HAS2-6

RP11-19D2.1
LINC00661
linc-CST7-1
CTD-2553C6.1
RP11-521C20.2
linc-CHCHD6-1
AC091814.2
RP11-323N12.5
RP5-1092A11.5
linc-PTK2-1
linc-LYZL1-2
RP11-730G20.1
RP11-1136G11.8
AC021021.2
linc-SGPP1
LINC00911
RP4-764O22.2
RP11-482D24.3
linc-CRH-1
CTD-2534J5.1
RP11-614O9.1
linc-C20orf196-1
AP001171.1
GPC6-AS1
RP11-133F8.2
CTD-2313J23.1
RP3-527G5.1
RP11-1020A11.2
RP11-171L9.1
AC096570.1
RP11-3D4.3
linc-ANKRD20A4-4
RP11-116D2.1
AC020601.1
CTD-3023L14.3
RP11-170N11.1
linc-ADAM18-2
AC095067.1
linc-KHDRBS3-4
AC096669.1
linc-DHX37-21
RP11-659F24.1
RP11-713P17.5
GS1-594A7.3
RP1-111B22.3
CSAG2
CTD-2538G9.6
RP11-275I14.4
RP11-495P10.3
linc-C20orf166

RP11-116O18.3
linc-SECTM1-3
DCUN1D2-AS
AC092669.3
CTD-2528L19.6
RP11-305L7.3
RP11-488I20.8
LINC00238
RP1-228P16.4
linc-C2CD4A-7
linc-CORO1C
RP4-724E16.2
linc-CHM-1
RP11-20I23.6
ANKRD62P1-PARP4P3
CTD-2194D22.2
AC003984.1
RP11-129J12.1
RP11-501O2.3
RP11-429D19.1
RP4-669P10.16
RP11-91H12.3
RP11-132N15.2
linc-DCLK2-5
linc-MTRNR2L1-4
RP11-338C15.5
linc-PLEKHH2-4
CTD-2349P21.10
linc-MTHFSD-3
linc-SLC16A7-1
RP11-338E21.3
RP11-97E7.2
RP11-445P17.8
CTD-2008P7.10
INHBA-AS1
linc-FBXW4-1
RP11-16B9.1
RP11-495K9.7
RP11-742D12.2
RP11-129K12.1
RP11-395I6.3
RP5-1142A6.9
TTN-AS1
RP11-815I9.4
CTD-2337I7.1
RP11-434D2.3
RP11-136I14.2
RP11-395I14.2
CTD-2611O12.8
RP11-723O4.2

RP11-405F3.4
linc-ZNF107-1
RP1-67K17.4
RP11-973N13.3
RP1-80N2.2
AC010524.4
RP11-394J1.2
RP4-604K5.2
RP11-431I8.1
linc-ANKRD55-2
GS1-5L10.1
RP11-152O14.4
linc-TMEFF2-2
CTC-558O2.2
RP11-365P13.3
MTUS2-AS2
RP11-1055B8.2
linc-CPSF7-2
RP11-802H3.2
RP11-293P20.2
AP006621.9
XXbac-B476C20.17
linc-BSND
RP11-374M1.3
RP11-372E1.6
RP11-134K13.4
RP11-24B19.4
AC006262.5
LINC00293
linc-ATOH8
RP11-73B2.2
RP3-370M22.8
linc-USP24-4
AC008592.4
RP11-219G10.3
RP11-14C22.6
linc-VPS36-1
AC093662.5
ZIC4-AS1
linc-C15orf2-9
AC069155.1
RP11-236L14.1
linc-LRRC16A-2
RP11-118F19.1
RP11-153K11.3
AC005235.1
linc-ATG12-1
linc-PIK3C2G-2
linc-PPDPF-1
RP11-461L13.3

CTD-2621I17.6
RP11-396B14.2
LINC00687
linc-GBE1-1
AC114730.2
CNTFR-AS1
AC005550.5
RP11-372H2.1
RP11-221N13.4
linc-CYP24A1-3
RP11-829H16.3
CTC-529L17.2
RP11-96H17.3
linc-PUM2-3
RP11-354M20.3
RP5-1028L10.2
AC053503.11
CTB-7E3.1
linc-ZNF831
RP11-232M24.1
RP11-353B9.1
RP11-288I21.1
CTC-338M12.7
RP11-290K4.1
RP11-163F15.1
RP11-23B15.1
RP11-138H8.3
RP11-284F21.9
RP11-167N4.2
CTD-2050I18.1
AC096574.4
RP11-347K2.1
linc-FPGT-5
linc-C10orf96
linc-CCDC37-2
linc-WDTC1
linc-RNASEH2B-1
LINC00593
RP11-107K17.1
CTB-131B5.2
linc-RGS18-6
RP11-809N8.2
HOTTIP
linc-S100B-1
AC009498.1
linc-SLC17A2
AF064860.7
CTD-2281E23.1
linc-ZNF492-2
RP11-627G18.4

linc-SLC10A5
AC087073.1
RP11-540N6.1
RP11-807H7.1
RP11-118G23.1
RP11-631F7.2
CTD-2572N17.1
AC092580.3
RP11-359E8.5
RP11-297H3.3
linc-CCND1-1
RP11-753D20.1
linc-ID3-1
ERVH48-1
linc-CLLU1-4
CTD-2530H12.7
RP11-155I9.1
AC019330.1
AC025016.1
AC013402.2
RP11-734K21.3
RP11-523L1.2
RP5-820B18.3
linc-CBLB-2
linc-BMPER
AC005740.6
AC012668.3
AC114814.4
RP11-202D1.2
RP11-703C10.1
CTC-255N20.1
RP11-355N15.1
RP11-785G17.1
RP11-91I20.3
RP11-505K9.4
RP5-1077H22.1
RP1-300G12.2
AC016582.2
CTD-2287O16.4
linc-KCND3-2
linc-SPTLC2
TRMT2B-AS1
AC069277.2
RP11-927P21.1
RP11-92B11.3
AC016292.1
RP4-749H3.2
RP11-218M11.6
linc-ZNF32-5
AC007131.1

ITGB5-AS1
RP5-945F2.3
RP11-108P20.4
LA16c-2F2.8
RP11-172F10.1
CTD-2265O21.3
AC009950.2
linc-ANKFN1
RP11-631F7.1
linc-PARP11-1
BCAR4
CTD-2537I9.5
linc-USP38
AC011306.2
linc-RPP25-1
RP11-841O20.2
ARAP1-AS2
linc-CCR8-1
linc-NDUFA5-2
linc-NR2F2-4
RP11-606D9.1
RP11-360A18.2
linc-CDYL-4
RP11-644L4.1
CTD-2313F11.3
RP11-76E12.1
AC074019.1
RP11-336K24.4
CTB-147C22.9
RP11-776H12.1
linc-CTSO-2
CTC-276P9.2
CTC-459F4.1
RP11-125D12.1
RP11-626H12.3
linc-MYO3B-2
linc-ADRA2A-2
RP11-214K3.20
linc-SSTR1-3
linc-MTRNR2L1-2
AF001548.6
RP11-1260E13.4
RP11-464C19.2
TAB3-AS1
AC079630.2
AC013733.3
LA16c-361A3.3
linc-AKR1E2-4
linc-SFMBT1
RP1-81D8.3

linc-RTL1-6
RP11-49C24.1
linc-CD207-1
AC016903.2
AC008060.8
linc-IQCA1-2
linc-C22orf26
ARPP21-AS1
AC005160.3
RP11-1028N23.3
ZNF346-IT1
RP11-215G15.5
RP11-136H19.1
linc-MEST-2
RP3-368A4.5
RP11-252M21.6
linc-GRAMD4-1
GS1-410F4.4
linc-USP24-1
PLS1-AS1
RP11-107I14.5
RP11-428G5.5
linc-SOX1-2
RP1-249H1.4
RP11-519G16.2
RP11-26L20.4
linc-C17orf103
RP11-510J16.5
RP11-240L7.4
linc-TTC24-1
AC020571.3
NTRK3-AS1
CTC-327F10.4
RP5-1013A22.2
RP11-76C10.5
AC113607.1
BACH1-IT1
AC079584.2
E2F3-IT1
RP11-125B21.2
RP11-264B17.4
linc-CERK-4
linc-C5orf38-4
linc-TUBA1A-3
RP11-124G5.3
linc-SYT4-3
CTC-525D6.2
COL4A2-AS2
linc-PNO1-2
RP11-145M9.4

RP1-28O17.1
linc-LGALS12
linc-STK32B-2
AC005740.5
RP11-314P15.2
linc-STAG3L2
CTD-2194D22.1
RP11-275O4.3
RP11-229P13.23
linc-CCDC122-2
RP11-587H10.2
RP11-68I18.10
RP5-956O18.3
CTC-806A22.1
RP11-47P18.1
linc-DNAJB11-3
linc-MMD-2
AC096574.5
linc-OLIG2-5
RP1-35C21.2
RP11-78O7.2
RP11-251M1.1
LINC00594
linc-C20orf46
RP11-380G5.2
RP11-305O6.3
linc-GCNT7-1
RP11-118H17.1
AC023481.1
linc-BTN1A1-1
linc-RHOBTB1
AD001527.7
linc-ATXN3L
RP11-431M3.2
RP11-142J21.1
AP000473.5
AC099684.1
RP11-536C5.7
CTD-2639E6.4
RP11-374M1.4
MIR302B
linc-GPR27-3
AP001625.6
RP11-413H22.2
RP3-337D23.3
RP11-701H16.4
RP11-16E23.3
linc-CYP11B2
MIR151A
linc-OPRM1-1

HCG21
linc-RAN-2
RP11-336A10.4
PRICKLE2-AS2
TMEM191C
RP11-94A24.1
RP11-548O1.3
linc-WDR48
RP11-44L9.2
RP11-303E16.8
linc-UTP23-1
RP11-478K15.1
CTD-2015A6.1
CASC8
RP11-547D24.1
linc-SLMO2-3
AC011524.2
RP11-110A12.2
linc-ZBTB37
RP11-59J16.2
linc-PCSK2
AC096559.1
AC079779.5
RP5-1029F21.2
AP001422.3
RP11-1148L6.5
linc-XRCC2-5
linc-WDR11-2
RP11-123K19.1
linc-TNRC6C-2
linc-MFSD9-11
DLX6-AS1
AC113167.2
AC009229.5
RP11-588K22.2
linc-ADRA2C-4
RP11-443N24.3
RP11-583F2.2
RP11-531H8.2
RP11-59N23.1
AC068489.1
RP11-48G14.2
linc-VAX1-2
RP11-168O22.1
RP11-357K6.3
RP11-1007I13.4
linc-IDH3B-1
linc-SCUBE1
RBMS3-AS1
RP11-641A6.2

RP11-272J7.4
RP11-301N24.3
linc-CDKN2C-5
linc-SCN2A-3
RP11-354K1.2
RP11-698F20.3
AC020956.3
CTD-2530H12.2
RP11-527H14.1
linc-RAP1GAP2-2
linc-CNTNAP5-5
XXbac-BPG248L24.13
AP000688.14
linc-HTATIP2
RP5-1022J11.2
RP11-372M18.2
RP11-425M5.5
RP11-1E6.1
linc-PECI-2
RP11-106M3.3
RP11-717D12.1
linc-ZC3H12B-4
RP11-265N6.2
linc-BCHE-2
RP11-416N4.1
linc-NR2F1-2
RP11-305P14.1
linc-PRKX-2
AC016722.3
linc-CHST11-2
CTD-3060P21.1
RP11-318G21.2
RP11-677I18.3
RP11-104H15.7
linc-FAHD2B-5
linc-CDH7-2
linc-VWDE-1
RP5-1164C1.2
RP11-932O9.8
RP11-119H12.6
RP13-20L14.1
RP5-894D12.3
linc-DCTN1
RP11-241G9.3
CTB-174D11.2
AC034187.2
RP4-715N11.2
RP11-343B18.2
RP11-111K18.2
RP11-154H12.2

AC007038.7
RP11-299H22.1
CTC-479C5.6
RP11-771D21.2
RP11-531P20.1
AC011995.1
RP1-276N6.2
linc-C3orf79-4
CTC-479C5.17
RP11-99A1.2
linc-CDH6-1
RP11-258F22.1
RP1-102E24.8
RP11-385E5.5
RP1-153P14.8
CTA-392C11.1
RP11-501O2.1
AC012531.23
linc-U2AF1-3
RP11-247A12.1
RP11-573D15.8
linc-MPP7-2
RP11-66D17.5
linc-SHB
LINC00330
linc-UBE2U
RP11-203F8.1
linc-C6orf35
RP13-137A17.4
LA16c-23H5.4
RP11-448G4.4
linc-KLK3-2
RP4-740C4.6
RP1-34H18.1
RP5-881L22.6
LINC00564
AC084125.4
EPHA1-AS1
RP11-145H9.3
RP11-1018N14.4
RP11-184E9.2
AC017074.1
CTD-2296D1.4
RP11-353N14.5
RP5-857K21.3
LINC00572
RP11-379F12.4
linc-TMEM100-1
RP11-23E10.2
AC004070.1

MEG8
XXyac-YX65C7_A.3
RP11-138E16.1
linc-SGCD
linc-SERPINB3-1
RP11-527D15.1
RP11-245P10.6
linc-TBX3-4
RP11-361I14.2
linc-SLC48A1-3
RP11-247L20.4
RP11-121G22.3
AP001429.1
RP11-909N17.2
RP11-552F3.13
CTC-457E21.1
AC007091.1
AC100830.5
RP11-778J16.3
linc-C6orf120-5
linc-GATA4-1
CTC-490G23.4
LINC00943
RP11-429A20.4
RP11-923I11.3
RP11-65J3.15
linc-MGLL
RP11-333J10.2
CXorf51B
RP5-1068B5.3
linc-CDH9-7
SRD5A2
AP002856.5
RP5-887A10.1
RP1-137K2.2
linc-PARK2-4
linc-GNA13
RP11-349A8.3
RP11-1082A3.1
RP11-344B2.3
RP11-286N3.1
RP11-304M2.2
LINC00904
RP11-498C9.17
AC068718.1
KB-1836B5.4
AP006285.7
RP3-333A15.2
AC078883.3
RP1-102H19.6

RP11-989F5.1
AC104389.16
linc-RRP15-1
AC003986.5
AC092755.4
AC073257.2
CTB-35F21.4
RP11-678G14.2
linc-SGCG-4
linc-ZNF479-7
RP1-40E16.11
RP11-56N19.5
RP11-536P6.3
CTD-2012M11.3
RP4-710M3.2
RP11-453A12.1
RP11-62L18.3
RP11-158D2.2
linc-MACF1-2
linc-CD33
RP11-617I14.1
linc-PLCZ1-4
RP11-280O1.2
linc-PCDH8-5
RP11-563E2.2
linc-STAT4-2
RP4-697K14.12
linc-COL1A2-1
RP1-290I10.5
linc-DTNBP1-3
linc-WDR60-3
RP11-709B3.2
linc-CACNA2D1
RP11-307L3.4
RP11-335I12.2
RP11-52J3.2
linc-DLK1-1
RP11-14P20.1
linc-STS-5
AC009232.2
RP11-561E1.1
AC073626.2
AF003625.3
RP11-324C10.1
RP11-421L21.2
AP001271.5
RP4-704D21.2
RP11-346D6.6
linc-BMP10
linc-NETO1-3

RP5-1173A5.1
AC124997.1
RP11-412H8.2
CTD-2537O9.1
linc-TRHR
linc-PRMT6-5
linc-SLC30A5-5
RP11-459K23.2
RP11-93H12.2
RP11-3B12.5
RP5-916O11.2
linc-GUF1-2
RP5-894A10.6
AC016730.1
RP11-939C17.4
linc-GPR26-2
CTD-3187F8.11
RP11-445O3.2
RP11-1151B14.2
RP11-347K2.2
RP11-362L22.1
RP11-806K15.1
RP11-313P18.2
RP11-114F10.3
RP11-736E3.1
linc-ZNF99-1
RP11-495K9.9
RP11-135D11.2
linc-DLGAP5-1
AC113608.1
RP11-94C24.11
linc-FAM55D
linc-NOB1
linc-MEGF10
FAM66A
linc-GOLIM4
linc-C7orf65-6
RP11-707G14.7
RP11-801I18.1
RP11-856M7.6
RP11-77K12.8
RP5-867C24.5
RP11-545G3.1
RP11-269G24.2
linc-MPPED2-2
RP11-93B14.5
RP11-38P22.2
RP11-264L1.4
RP11-190P13.2
NAV2-IT1

linc-ELMOD1
AC090505.1
AC004540.4
LINC00613
AC083843.3
linc-DCLK2-4
RP11-53B2.1
HPYR1
RP5-955M13.4
RP11-1E3.1
linc-PCSK6-1
RP11-863P13.4
linc-CADM2-4
linc-HIVEP1-2
RP11-61A14.1
RP5-1010E17.2
AC108051.2
RP11-65L3.1
linc-APOB-3
AC114730.7
RP11-867G2.2
RP11-798G7.8
RP4-781K5.4
RP11-235E17.3
CTC-429P9.3
linc-GUCY1A2
RP4-806M20.5
LINC00636
RP11-346L1.2
CTD-2281E23.2
RP11-141M1.3
linc-MRGPRF-1
linc-WDR72
RP11-54O7.14
RP11-8L8.2
AC022819.3
AC074389.7
linc-ZFP42-3
RP11-816J8.1
AC009158.1
linc-TSHZ1-2
CTB-31O20.3
RP11-417L19.2
RP11-324O2.3
CTA-833B7.2
linc-ADAMTS8-1
linc-PAQR8-2
LINC00459
AF241725.4
linc-COBL-7

RP3-404F18.5
linc-GLT25D2-1
RP11-390K5.6
linc-NOX3-5
RP11-401I19.1
RP11-354I10.1
LINC00352
CTB-49A3.5
linc-HUS1B-4
RP1-125N5.2
RP11-800A3.4
RNF185-AS1
AC005789.9
AC092316.1
RP11-417J8.1
linc-CDC16-2
RP5-986I17.2
RP11-1391J7.1
RP11-634B7.5
GPC5-AS1
RP11-439L8.3
RP11-451H23.3
RP11-401N18.1
RP11-122F24.1
linc-ZNF33A-4
RP13-36G14.4
CTD-2545M3.2
RP11-548B3.3
AC008103.5
CTD-2541M15.4
RP11-53B2.5
RP11-208N20.1
RP11-428C6.2
RP11-966I7.2
RP11-129J12.2
AC013448.2
RP11-114H24.7
linc-PRKAR2B
linc-FSTL5-1
XXbac-BPG55C20.7
linc-RGPD4-4
AC004019.13
linc-MUC20-9
RP5-1119A7.10
linc-FABP3-2
RP3-468B3.2
AC104809.2
RP11-1021N1.2
RP1-127D3.4
CTD-2554C21.3

RP3-417O22.3
LL22NC01-81G9.3
RP11-89C3.4
linc-HERC6-1
RP11-500B12.1
linc-C6orf223
AC005625.1
RP11-531A24.7
RP11-582J16.5
RP11-297P16.3
CYP4F35P
RP11-227G15.3
CTD-2620I22.1
RP11-572M11.1
RP11-98D18.17
RP13-216E22.4
RP11-325I22.2
RP11-488I20.9
CTC-527H23.3
AC007040.5
RP11-386G11.8
RP11-449L23.2
linc-FAM92B-4
linc-TP53TG3B-1
CTA-250D10.23
CTD-2210P24.1
CTD-2023N9.3
linc-NUDT15-2
NEFL
RP11-118A3.1
linc-OLR1-2
RP5-1172N10.3
AC008753.4
linc-CCKAR-4
HOXD-AS1
RP11-678G14.3
RP11-646J21.3
linc-CDK9-1
linc-E2F7-4
RP1-13P20.6
linc-CR2-2
RP1-155D22.1
RP11-310P5.2
RP11-252I13.2
RP11-432J24.6
linc-RORB-3
linc-FAM20A-2
RP11-557J10.5
RP11-397D12.4
RP11-462G12.2

RP11-510C10.4
RP3-477O4.16
RP5-930J4.4
linc-TECTB-2
AL928768.3
RP11-403B2.5
RP11-738G5.1
LINC00507
RP11-394A14.4
linc-FBRSL1-3
RP11-354O24.1
RP11-165I9.6
linc-EHBP1
RP11-250B2.6
linc-COL18A1-2
EMCN-IT1
RP11-531A24.5
RP11-236J17.5
CTD-2528A14.3
linc-RTTN-4
linc-PDE1A
RP11-690J15.1
RP11-445J9.1
RP11-557H15.5
CTC-523E23.5
RP11-274H2.5
linc-LRRTM4-5
RP11-274B21.8
RP11-707P17.1
linc-SLC25A27-4
linc-GPR31-2
linc-TACR2
RP11-889D3.1
linc-ADAM11
AC108462.1
linc-FBXO33-1
RP11-696D21.2
linc-ALDH1A1-5
RP11-357N13.1
CTD-2515H24.3
LINC00694
RP11-404P21.5
RP11-268G12.3
RP11-60A24.3
RP11-97E7.1
RP11-481G8.2
linc-MARCKS-2
linc-SLC10A2-4
RP11-467L24.1
LINC00846

RP11-403N16.2
RP11-399H11.3
linc-ATG2B-4
RP11-398B16.2
linc-CXorf23
RP11-526F3.1
RP11-717H13.1
RP11-529E10.7
linc-ZNF608-5
OSTN-AS1
RP11-471L13.2
SNORA58
EAF1-AS1
linc-OBSL1-3
RP11-1029J19.2
linc-TMEM88B-1
C20orf166-AS1
linc-SMAD2
RP11-627G18.2
RP11-499E18.1
RP11-524D16__A.3
RP1-146A15.1
LINC00423
RP11-156E6.1
RP11-19E11.1
linc-SLC35F3
linc-HOXB9
AC109309.4
RP11-434I12.3
LINC00341
LINC00387
RP11-322F10.2
linc-MGAT4C-1
RP11-654G14.1
RP11-428L21.1
RP11-805I24.4
RP11-15N24.4
CTD-2582D11.1
RP11-820I16.3
RP11-761I4.4
RP4-745E8.2
linc-FAT3-1
RP11-298D21.3
AP000462.1
CTD-2349P21.9
linc-ZNF536
linc-RECK-3
RP11-101E5.1
RP4-728D4.2
RP11-27G13.1

AC004257.3
RP1-40E16.9
AC078842.4
RP11-379F4.6
linc-C20orf196-2
RP11-281A20.2
AP000857.3
RP11-116D17.2
TMPRSS4-AS1
RP11-509E16.1
AP000439.1
RP11-314O13.1
linc-FASTKD3-1
linc-KIAA1383-2
RP11-185E12.2
RP11-616K22.2
RP11-94I2.4
RP11-16L14.2
AC011899.9
AC010090.1
RP11-335E6.3
RP11-192H23.7
RP11-430N14.4
RP11-593F5.2
RP3-523K23.2
LINC00391
CTC-518B2.9
AP003039.3
linc-C11orf82-3
CTD-2331C18.5
RP11-554E23.2
linc-MAST4-1
BRWD1-IT1
linc-SMARCAL1-2
RP11-173E2.2
CTC-264O10.1
AC010127.3
RP11-673E1.4
CTD-2200A16.1
RP11-553K8.2
RP11-360L9.7
linc-GTF2H2-5
linc-TTLL7-7
linc-ZCCHC17-5
linc-SMC3
RP11-497G19.1
DAPK1-IT1
linc-LOC100132247
LINC00672
RP4-668J24.2

RP11-197M22.2
linc-ZNF467-2
RP11-74H8.1
linc-POGK-1
RP11-453M23.1
RP11-217C7.1
RP11-243E13.1
linc-BIRC6
RP11-274B18.4
RP11-141A19.1
RP1-45C12.1
RP11-1096D5.2
linc-LARS2-1
DNM3-IT1
linc-C15orf2-10
RP11-403I13.9
RP11-249M12.2
linc-NMT2
linc-STK39-3
linc-MUM1L1
linc-SETBP1
linc-RAI14-1
KIRREL3-AS2
LINC00621
linc-TMEM132C-4
RP11-96C23.10
CTD-2085J24.4
AC084809.3
RP13-608F4.8
RP11-351C8.1
RP11-61L19.1
linc-LEP
linc-DYNLL2-2
RP11-307L14.1
RP11-805J14.3
RP11-445K13.2
CTD-2333K2.1
MMP12
AC068610.3
RP11-469N6.1
RP11-1017G21.4
RP5-827L5.1
CTD-2373H9.5
RP11-254F7.2
RP5-1027O11.1
LINC00478
RP11-3N2.3
RP1-41C23.1
linc-OAF-1
CTC-497E21.5

AC123023.1
RP11-374A4.1
AC008278.2
linc-FLJ44606-1
linc-ACTRT2-1
linc-ARSJ
AC138655.4
linc-FAM118B
RP11-458D21.2
RP11-846F4.12
RP11-805I24.3
XX-C2158C12.2
RP11-567I13.1
linc-ABCA1-10
linc-SEMG1
RP11-382D12.1
KCNH1-IT1
RP13-131K19.1
RP11-539E19.2
RP1-40E16.2
RP11-769O8.1
RP5-857K21.1
AC093732.1
CTC-552D5.1
RP11-168G22.1
linc-PELI2
RP11-551L14.6
RP11-659O3.1
AC005104.3
linc-C6
linc-MSX1-1
RP11-29P20.1
linc-BIRC7-3
RP11-981G7.6
RP11-242C19.2
CTC-329D1.3
RP5-902P8.12
linc-PITRM1-4
linc-FBLN1-AS
LNX1-AS1
AC114776.3
AC073321.4
SMAD9-AS1
CTC-360P9.1
RP11-325D8.1
linc-CPEB4-7
RP11-619L19.2
RP11-94B19.1
CTD-2517M14.5
linc-CDK20-1

RP11-14O19.2
RP11-114G22.1
RP11-144G6.4
RP11-169K16.4
linc-TBC1D20
RP11-81H3.2
linc-ANP32A-2
linc-ITGA6-1
RP11-161I2.1
AC007461.2
AC005339.2
linc-TYRP1-1
RP11-566J3.2
RP11-274B18.2
linc-NLRP1-2
RP11-696P8.2
RP11-317J10.4
linc-LGALS13
AC024028.1
RP11-670N15.1
linc-USP24-3
linc-CTSD-2
linc-ZMYM6
RP3-430N8.10
linc-ARMC3-2
CTD-2022H16.1
linc-GBE1-3
linc-CDC42BPB-2
RP11-116O11.1
KCND3-AS1
CTD-3023L14.1
CTD-2510F5.4
linc-ASAP1-1
RP11-546K22.2
EVX1-AS
linc-MSR1-1
TET2-AS1
RP11-405F3.5
RASA3-IT1
AC007383.4
RP11-402P6.11
RP1-310O13.7
RP5-951N9.1
RP11-189B4.6
CTB-41I6.1
NAALADL2-AS2
RP11-69G7.1
RP11-95I16.4
RP11-362K14.5
RP11-982M15.7

RP11-367B6.2
RP3-395M20.3
linc-NEUROD6-2
RP11-211C9.1
RP5-828H9.1
RP11-680F20.5
RP11-63B19.1
CTC-459F4.5
CTB-108O6.2
RP11-649E7.7
LINC00276
linc-MPHOSPH6-2
CTC-228N24.1
linc-SLC25A15-3
RP11-1152H15.1
AC130469.2
RP11-622J8.1
RP11-634B22.4
ISM1-AS1
AC007098.1
RP4-598G3.1
RP11-64K12.8
RP11-817J15.2
linc-RGPD1
RP11-31L23.3
RP11-540E16.2
linc-VPS8-2
RP11-101P17.10
linc-ENPP6-3
linc-DTNBP1-1
AC007948.1
RP11-884K10.7
CTC-344H19.4
linc-ARFGEF2-11
linc-ALOX12-1
CTD-3049M7.1
AC006026.13
RP11-77H9.5
RP4-545L17.7
AC006129.1
RP11-73M18.10
linc-B4GALNT4-2
CTB-175P5.4
DSCAM-AS1
CACNA1C-IT3
linc-ZFP42-4
RP11-1055B8.8
linc-TPK1-2
RP11-73O6.3
linc-PARP10-2

AL121578.2
linc-POU4F2-1
linc-TES-1
RP1-302D9.2
RP3-410B11.1
RP11-479I1.4
linc-PLEKHH2-2
RP11-239A17.1
DMD-AS2
AC007879.6
linc-SCTR-7
RP4-680D5.8
CTD-2286N8.2
RP11-474D1.3
RP11-286B14.2
linc-ONECUT1
RP11-439E19.10
RP11-244F12.2
RP5-991O23.1
RP11-136I14.5
linc-CASP10-2
linc-SALL1-6
linc-CNOT2
RP11-350G24.1
linc-ATP4B-1
RP11-483F11.7
linc-NLRP8
linc-CNTNAP2-1
RP11-14D22.1
GHRLOS
linc-MPPE1-3
RP11-427H3.1
linc-TYRP1-6
RP11-563M4.1
AF121898.3
linc-ATP2B2-3
RP11-307O13.1
linc-ZNF280D
linc-DIRAS2-5
linc-OR7C2-2
RP11-31I22.2
CTB-33O18.3
RP11-359J6.1
RP11-128I7.1
linc-ZNF703-1
RP11-1406H17.1
linc-SEL1L-7
linc-PTBP2-6
ZBTB40-IT1
RP11-54O7.16

RP11-70C1.3
LINC00290
RP5-842K16.2
RP11-284F21.8
AC007228.5
AP000343.2
RP1-92C4.2
RP11-83C7.1
linc-NID2-4
ADORA2A-AS1
RP11-547I7.1
linc-DYDC1-4
RP11-541F9.2
linc-ITIH2-13
CTA-797E19.1
linc-UQCRFS1-2
AC000111.6
RP11-1J7.1
RP11-1007G5.2
RP5-865N13.2
RP11-781P6.1
RP11-1259L22.1
IGSF11-AS1
CTA-150C2.13
linc-UCHL3-5
RP11-111D3.2
AC005062.2
AC074183.4
linc-ADCY2-5
linc-SLC44A1
RP11-67L3.2
RP11-451G4.1
linc-LRRC38-7
linc-RAB6C-5
RP11-646J21.2
RP11-738E22.3
AC104654.2
RP11-286N22.10
linc-CPA5
linc-PGS1-3
CTC-276P9.3
AC016912.3
RP11-781A6.1
RP11-221N13.2
linc-C1orf83-2
linc-C15orf2-2
RP5-884C9.2
linc-FZD10-2
RP11-655G22.1
CTD-2619J13.17

CTA-929C8.5
AC007679.3
RP11-423J7.1
AC011899.10
linc-PSMD7-5
UBE2Q1-AS1
linc-ASB4-2
CTD-2309H9.3
RP11-511I2.2
RP3-461P17.10
linc-DOK6
GS1-166A23.2
linc-PARK2-6
RP11-244B22.11
ZNRF3-IT1
linc-EXOC6B-1
RP11-30G8.1
RP11-35J10.4
RP1-138B7.5
linc-PDE10A-4
RP11-75C9.1
linc-NUDT6-1
linc-ZNF625
LINC00523
RP11-388C12.8
AF011889.5
CTC-365E16.1
linc-JARID2-3
linc-CTNNA1-1
SLC38A3
AC007078.4
CTC-215O4.4
CTC-400I9.2
linc-MMP2
CTD-3065B20.3
RP11-462G12.4
RP11-108K3.4
linc-SULF2-2
RP5-1160K1.6
linc-SLITRK5-11
AC068286.1
RP11-1026M7.3
LINC00858
linc-CDH5-8
AC124944.5
RP11-3D4.2
RP5-1178H5.2
AP001271.3
LINC00102
AC090505.6

RP11-66A2.1
RP11-100N20.1
RP11-414H23.2
DIAPH2-AS1
RP11-393K12.2
linc-KIAA1257
RP11-449P1.1
AC114765.2
OPA1-AS1
AC034228.4
linc-FBXO17
RP1-117P20.3
RP11-259A24.1
RP11-364C11.3
AL132709.1
RP11-401P9.4
linc-COL5A1-4
RP11-247I13.7
CTC-563A5.2
RP11-7N14.1
AC007349.5
linc-C11orf82-4
ATP2B2-IT2
linc-GPATCH4
RP11-136O12.2
linc-SEPP1-2
linc-SLITRK1-7
linc-ATXN7L3B-2
LINC00483
C10orf43
RP11-108O10.2
AC010096.1
HTR2C-IT1
CTB-167B5.2
linc-PFAS-1
RP11-989E6.3
RP11-95K23.3
RP11-54H7.2
linc-SSBP3-1
RP11-746B8.1
RP11-165I9.4
RP4-536B24.2
AC005253.4
RP11-231K24.2
AC007392.4
AC003090.1
linc-RIMBP2
linc-SCGB1D4-1
linc-MYOM1-2
linc-LARGE-3

linc-PRR5-4
RP11-1D12.2
linc-CXorf30
linc-PPARA
RP11-6N13.1
RP11-232C2.2
linc-ARL15
RP11-66B24.5
RP11-64I17.1
BACE2-IT1
linc-CDH5-11
linc-NEURL1B-1
LINC00307
linc-PRICKLE2-2
linc-CCDC40-3
linc-FOXP2-6
linc-LYZL1-4
RP11-506O24.2
linc-WRB-4
RP11-308N19.1
linc-SPTBN1-2
linc-RXFP2-2
RP11-524H19.2
TRIM36-IT1
linc-LAMA1-2
RP11-733D4.1
RP11-365N19.2
RP11-32D16.1
CTA-125H2.2
RP11-248E9.6
RP11-143O10.1
RP4-712E4.2
linc-API5-4
AP000346.2
RP11-355E10.1
CTD-2538G9.5
RP4-764O22.1
RP11-218C14.8
RP11-311H10.4
RP11-737F9.2
CTD-2568A17.1
linc-ZIC1-6
RP13-753N3.1
RP11-13N12.1
AC092071.1
AC084149.2
RP11-523L20.1
RP11-157P1.5
linc-HSD17B12-1
RP1-3J17.3

linc-MUC20-2
RP11-738B7.1
RP11-598F7.4
RP11-561B11.6
linc-CTNNB1-1
linc-EGFLAM-2
AC007251.2
RP11-697N18.4
RP11-168A11.1
linc-ZNF366-5
CTD-2245F17.9
CTC-425F1.2
RP11-4K16.2
linc-NFAM1
AP001059.5
linc-PSMG4
RP1-116K23.1
linc-RNF152
linc-GRIP1-3
RP11-152P23.2
linc-ZNF107-3
linc-CPSF7-1
XXbac-BCX196D17.5
RP11-91P17.1
linc-PHB-2
AC105461.1
RP11-94L15.2
linc-UGT8-2
linc-CACNA1A-1
RP13-192B19.2
RP11-437L7.1
linc-DNAH6-2
linc-COL4A2-3
AC013472.3
linc-FAM69C-2
RP11-513O17.2
AL022344.4
linc-ALOX5-1
RP11-688G15.3
linc-GCNT2-3
RP4-672N11.1
RP11-514F3.4
RP11-298E9.6
AC008269.2
RP1-34M23.5
AC073115.7
linc-CCDC140-2
RP11-324I22.3
linc-CTSO-1
LINC00466

RP11-366M4.3
AP000233.4
linc-SECTM1-2
RP11-355I22.7
linc-RHOB-7
linc-ARHGAP28-2
linc-ZNF257-4
CTA-363E6.5
RP11-91J19.4
linc-DSG1-3
LINC00083
RP11-406O16.1
linc-ERG-9
RP11-90J7.4
CACNA1C-IT1
RP3-471C18.2
CTC-218H9.1
RP11-876F14.1
RP11-523O18.1
linc-FOXF1-1
linc-SMC6
RP11-46H11.3
RP1-68D18.3
MIR296
RP11-400D2.3
AC055764.1
linc-APOB-12
linc-WFDC2-2
CTD-2008P7.1
linc-FAT4-1
linc-C16orf72
linc-TGM3-1
AC004152.6
linc-C6orf105-3
linc-ZNF726-3
C4A-AS1
linc-VTA1-2
AC073987.1
RP11-504P24.4
AC006160.8
linc-H3F3C-2
RP11-571M6.13
RP11-259K15.2
linc-XPO7-4
AP001619.3
RP11-566K19.3
RP11-6B19.1
CTD-2331D11.3
RP11-533N14.3
linc-SBDS-1

RP11-552M11.4
RP11-715C4.1
C20orf203
linc-PHOX2B-2
DSCR9
CTC-264K15.6
AC012668.1
linc-NBPF6-2
RP11-350N15.3
linc-CCRN4L-17
RP4-586O15.1
RP11-343K8.3
linc-PPAP2A-1
RP11-429B14.4
linc-SMARCA2-2
LINC00843
RP11-61I13.3
RP5-867C24.4
RP11-800A3.2
RP11-145M4.1
RP11-592B15.9
RP11-37C7.1
RP11-264J4.5
RP11-647F2.2
RP11-673P17.2
linc-GBP6-4
linc-CLIC6
RP11-38M15.3
RP11-350O14.18
RP11-736I10.1
AC098828.2
AC068858.1
linc-GRHL2-4
linc-IGFBP2-3
RPS6KA2-AS1
RP11-793A3.2
RP11-674P19.2
linc-DEPDC1-1
linc-TRIO-1
RP11-19P22.5
CTC-508F8.1
linc-FLI1-6
linc-MTRNR2L6-3
AC092168.3
linc-PDHX-2
LA16c-312E8.2
RP5-898J17.1
LSAMP-AS1
linc-SLC25A46
RP11-73B2.4

AC024592.9
CTB-187M2.1
linc-SUMF1-2
linc-MS4A6E
linc-LEPROTL1-6
U91324.1
RP11-158I23.1
RP11-118B22.4
linc-PRKACG-2
linc-FRG1-5
linc-FBXL7-1
linc-TMEM132C-13
AC084117.3
RP11-680G24.5
RP11-287F9.2
linc-CEP110-5
linc-GCM1
CTB-193M12.1
RP11-340I6.1
MIR4313
RP11-343J18.1
XXyac-YM21GA2.7
RP11-96G10.1
RP11-466A17.1
CTD-3007L5.1
RP5-897D18.1
linc-ANKRD56-1
linc-BMPR2
linc-ETAA1-4
CTD-2296D1.5
AC006272.1
RP1-93H18.7
RP11-572C15.5
linc-TMEM132C-12
AC003009.1
linc-ZNF133
AC008154.5
RP11-146I2.1
RP6-27P15.2
KLF7-IT1
RP11-171A24.2
RP1-232P20.1
CTA-397C4.2
RP11-256I9.2
AC013448.1
RP11-716O23.2
linc-APH1A-5
AC006050.2
RP11-161M6.2
RP11-769O8.3

linc-THBS4-2
CTD-2134A5.4
RP13-270P17.2
RP11-396O20.1
RP11-128D14.1
linc-ASCC3-3
linc-MYT1L-4
EGFLAM-AS1
RP11-389C8.2
AC002127.4
RP11-300J18.2
CTD-2207O23.11
linc-SUSD4
RP11-159D12.9
RP11-66N11.7
RP11-589M4.3
linc-CPEB2-11
RP1-283E3.8
linc-MC3R-3
linc-RPRM-6
RP11-550P17.5
RP11-337N6.1
RP11-686F15.3
OOEP-AS1
RP11-304L19.2
CTD-2012K14.3
linc-CRABP2
RP11-143E21.6
linc-PTBP2-1
AC015933.2
RP11-38J22.1
RP1-23K20.2
AC015923.1
RP11-115J16.1
RP5-1061H20.5
RP4-549F15.1
RP1-14D6.7
linc-MAP3K8-3
RP3-430N8.11
linc-SRGN
AP001469.9
AC007064.24
RP11-148O21.4
RP11-669E14.4
CTD-3195I5.4
linc-ITPRIP-4
AF127936.5
RP11-783K16.10
AC007327.5
linc-UCHL3-2

MIR3179-3
linc-PXN
RP11-314D7.3
RP11-283I3.2
RP11-550H2.2
AC015922.6
linc-NRG1-2
linc-PCTP
RP11-398J10.2
RP11-255H23.4
RP11-854K16.3
RP13-297E16.5
linc-AKIRIN1-4
linc-DCTD
RP11-178F10.2
RP11-7F17.3
AC068580.7
RP11-182J23.1
RP5-1100I6.1
RP3-433F14.3
RP11-98J9.2
RP11-619J20.1
RP11-201A3.1
RP11-114M1.1
LINC00114
RP11-2N5.2
AC004012.1
linc-TRIM36
RP11-770G2.4
linc-SLC15A3
linc-AGAP1-1
RP11-211F2.1
MCCC1-AS1
RP11-466A19.1
RP11-1038A11.3
AC097713.3
CTB-61M7.2
linc-HRASLS-2
linc-SEP15-1
CTC-499J9.1
RP11-479J7.2
RP1-56K13.2
RP4-669H2.1
linc-CLLU1-6
MRGPRG-AS1
AC005703.3
RMST
RP11-536O18.2
RP11-215N21.1
RP3-416H24.4

CTC-518B2.12
LINC00638
linc-IRX3-6
linc-ACTL7A-11
RP11-375D13.2
CTC-325H20.2
RP11-226P1.2
RP11-280K24.4
linc-MAX-1
RP11-507B12.2
RP11-678P16.1
RP11-114G1.2
RP11-687M24.8
XXbac-BPGBPG55C20.3
linc-PEX14
RP11-23P13.7
RP11-61O1.1
linc-SPACA3
linc-C7orf27-3
linc-AKR1E2-17
LINC00599
LA16c-429E7.1
AC010967.3
AC009499.2
CTB-178M22.1
linc-SORCS3-3
CTD-2544M6.2
CTC-207P7.1
RP11-490D19.6
RP11-133K1.6
linc-TMEM55A-1
RP11-276H19.2
RP11-398J5.1
RP11-238K6.1
linc-TCERG1L-1
RP11-161H23.5
RP11-145F16.2
CTC-510F12.2
U1
RP5-1112D6.7
RP11-32F11.2
THRB-IT1
C1QTNF9-AS1
RP11-113K21.4
linc-SERHL2-1
Z82214.2
linc-UBE3C-3
RP11-523H24.3
RP11-65L19.4
RP11-189E14.4

RP11-556I14.2
AC007249.3
RP11-305P22.5
RP11-804A23.4
RP3-475N16.1
RP11-44I10.3
linc-ADCYAP1R1
RP11-875H7.2
RP11-253E3.3
linc-BAHD1
FARP1-AS1
GS1-120K12.4
linc-MTRNR2L9-3
RP11-168O16.2
AC106870.1
AC159540.3
AC022311.1
RP5-1077H22.2
RP11-640N11.2
AC012506.1
CTC-558O19.1
linc-ABI1-1
linc-AQPEP
RP11-386B13.4
RP5-1042I8.7
linc-ADCY6-1
RP11-503L23.1
CTD-2384A14.2
linc-BRD9-1
RP11-503C24.3
RP11-420N3.2
linc-SLCO2A1-2
linc-CDC25A
RP11-587P21.2
RP11-753D20.3
LINC00207
linc-AK2-1
PEX5L-AS1
AC004870.3
linc-C8orf4-1
RP3-508I15.18
linc-GRK7
linc-C5orf38-5
linc-LOC100288255-1
RP11-494I9.2
linc-AZIN1-1
RP11-242O24.5
linc-GUCY1A3-2
RP11-789C1.2
RP11-568A7.2

PCCA-AS1
linc-SEMA4D-1
LINC00403
RP11-319E12.1
AC079163.1
CTD-2076M15.1
LINC00364
linc-C1orf198
RP11-571M6.18
LINC00317
RP11-481E4.2
RP11-318M2.3
RP11-1114A5.4
linc-CNTNAP5-3
RP11-231E19.1
AP000697.6
RP11-712C7.1
linc-PATE4
linc-LAMA2
RP11-540B6.6
MYB-AS1
AF064858.11
RP3-329A5.8
linc-RFPL4A
RP11-720L8.1
RP11-395P13.4
linc-POTEB-2
linc-ANO1-3
linc-KCTD5
AF129075.5
RP1-62O9.3
RP11-428C19.4
linc-KCNMB1-4
RP11-378I13.1
linc-CALB2-1
RP11-434C1.2
linc-GPR78-1
RP11-443N24.4
AP001092.4
LINC00035
linc-TMEM90B-3
RP11-392M18.5
AP001626.2
CTD-2545H1.1
RP11-729M20.1
linc-MARCH11-2
linc-COTL1-2
BX322557.13
linc-SHOX-3
RP11-69C17.3

RP11-384P7.7
AC006947.1
CTC-546K23.1
CTD-2124B20.2
RP11-510I5.4
linc-GLI2-3
CTD-2311B13.2
ZNF883
AC068535.4
linc-NCAM2-1
RP11-540K16.1
RP11-27G22.1
linc-GOLGA8B-2
RP11-756P10.4
RP11-552D4.1
RP11-789C17.3
linc-DLEU7-2
RP11-381I15.1
linc-BET1-1
CITF22-24E5.1
CTD-2647L4.4
RP11-279L11.1
linc-PDE3A-5
RP11-201M22.1
linc-FANCL-5
RP4-575N6.5
linc-DNAJB11-1
linc-FAM18B2
RP11-176D17.3
linc-TOX2
linc-DOPEY1-2
linc-MARCH6
RP11-94B19.5
RP11-308D13.3
RP11-213G6.2
LL22NC03-13G6.2
RP11-325F22.5
CTD-2037L6.2
CTD-2555O16.1
FAM99A
AP001442.2
linc-C11orf58-4
RP11-164N3.3
linc-UTP23-2
RP11-20J15.2
RP11-253D19.2
RP11-301G7.1
RP11-351M16.3
linc-ZNF676-2
CTD-2275D24.1

RP11-658F2.8
CTD-2143L24.1
KCP
linc-GLRA3-2
CTD-2382H12.1
linc-RBM26-5
RP11-45F15.1
RP11-556G22.3
MACC1-AS1
RP11-95H11.1
linc-ASTN2-3
AC139099.6
NRG1-IT2
linc-ODZ3-1
linc-GSX1
RP11-774O3.3
linc-XPO7-2
linc-MANEA-7
RP11-322J23.1
RP11-159F24.6
CTD-2552K11.2
RP11-417J8.6
linc-C13orf34-9
RP11-96C23.9
RP11-90C1.1
RP11-503N18.4
linc-FLI1-3
RP11-327J17.2
RP11-231E6.1
linc-HELT-2
AC007250.4
IL20RB-AS1
linc-GYPC-3
RP11-390E23.3
linc-GPC5-8
linc-HMCN1-1
RP11-522M21.2
RP11-352M15.1
CTD-2349P21.6
linc-HDGFL1-1
linc-ATG2B-3
linc-TNFRSF9
RP11-440L14.3
linc-RGPD4-6
linc-RPS6KA3-2
RP4-655J12.5
linc-PCLO
RP11-167N24.5
RP11-165F24.3
LINC00332

linc-MBNL1
RP11-495P10.1
BX322559.3
linc-FER-1
linc-BRD3-5
linc-UFM1-4
RP3-331H24.4
RP11-708H21.4
AC108676.1
RP11-38G5.2
linc-ATP6V0D2-2
LINC00643
LINC00445
RP4-671G15.2
linc-SRSF2-3
linc-SSTR1-1
AP000487.4
CTD-3203P2.1
RP11-56B16.4
RP11-59J16.1
CTD-2528A14.1
linc-ANKRD20A1-10
RP11-129K12.3
RP11-360O19.4
CTA-363E6.1
RP13-977J11.2
CTD-2026K11.2
linc-CARD11-7
linc-GRID1
linc-GDF10-1
RP11-203H2.1
RP11-18D7.3
RP11-283I3.6
RP11-1112J20.2
AC073218.2
RP11-1055B8.4
RP11-3P17.4
CTC-325H20.4
RP3-445N2.1
linc-SELK-2
RP11-270M14.4
RP11-397C18.2
linc-PRKAA2-4
linc-TXLNB-1
CTD-2653D5.1
linc-SPACA1
RP11-313C15.1
RP11-1085N6.4
RP4-591N18.2
KCNQ5-IT1

RP11-164O23.7
RP11-436K8.1
RP11-473A10.2
linc-ELFN2
AL132709.8
RP11-44B19.1
AC013269.3
RP1-241P17.1
linc-IRX5
linc-ELTD1-1
DIAPH3-AS1
RP11-160E2.21
AC005355.2
RP1-302G2.5
RP11-495P10.8
RP11-563N6.6
RP1-91G5.3
RP4-737A23.2
RP11-13N13.5
RP11-302L19.1
RP11-58E21.5
RP11-843P14.1
AC093911.1
RP5-1024C24.1
RP11-382F24.2
CTD-2517M22.17
RP1-16A9.1
RP5-1185I7.1
linc-VCX3B-1
RP11-146E13.4
RP11-103H7.1
RP11-384F7.2
RP4-796I8.1
RP11-893F2.15
RP11-135J2.3
linc-U2AF1-1
AC010091.1
RP11-116O18.1
RP11-400D2.2
linc-FITM2
RP11-473E2.4
linc-FAM196B
linc-CDKN2C-3
KB-7G2.9
linc-C9orf16
RP11-700N1.1
linc-MTOR-1
CTB-138E5.1
AC107072.2
RP11-407A16.4

FGF12-AS2
CTD-2380F24.1
linc-EXOSC9-2
RP11-38F22.1
RP11-153F1.2
RP11-411K7.1
linc-IGFL1-1
CTD-2244C20.2
linc-RANBP3L-5
linc-RTL1-4
linc-COIL-4
linc-SGCG-2
linc-THBS1-1
linc-FAM75D4-2
RP11-115K3.1
AC004869.3
RP11-223P11.3
CTC-441N14.2
linc-VANGL1-1
linc-P2RY6
RP11-353P15.1
CTC-304I17.5
AC062020.2
RP11-4O3.1
CTC-305H11.1
RP1-127L4.7
LINC00492
RP11-593F23.1
CTC-444N24.8
RP11-886P16.6
RP11-308N19.3
RP11-1069G10.1
CTC-338M12.9
LINC00086
RP11-245P10.8
7SK
linc-ZC3H15
CTD-2291D10.2
RP11-529H2.2
RP11-675F6.4
RP11-438E5.1
RP11-354H21.1
linc-CNTN1
RP11-400F19.18
RP1-251M9.3
RP11-863P13.2
linc-LRRC10B
linc-CARTPT
RP11-661D19.3
RP11-960D24.1

RP11-209K10.2
CTD-2385L22.1
linc-DIO3-7
RP11-418J17.3
linc-C3orf30-5
RP11-269F21.2
RP11-644C3.1
CTC-429P9.5
RP1-20N2.6
linc-TMPO-1
linc-THTPA
XX-FW83128A1.2
linc-U2AF1-5
RP11-122A21.2
linc-MRPS30-2
CTD-2531D15.5
KB-1299A7.2
HLA-DQB1-AS1
RP11-702F3.4
RP11-222A5.1
CTD-2072I24.1
XXbac-BPG13B8.10
linc-CADM2-11
RP11-2G1.1
AC007003.1
RP11-300G22.2
AC005154.5
linc-SAA1
LINC00484
RP11-452D21.2
linc-C13orf34-10
CHODL-AS1
RP11-567J20.1
linc-CLDN10-1
RP5-892K4.1
KB-1568E2.1
RP11-125M16.1
RP3-449M8.6
RP11-131K5.1
linc-BCHE-4
RP11-183I6.2
RP11-296E23.1
LINC00906
linc-MFSD9-13
linc-CTNNBL1-2
AC010745.3
RP11-572O6.1
RP11-864I4.3
RP11-21A7A.4
RP11-932O9.10

linc-GPR132
KCNQ5-AS1
AC091132.1
WDFY3-AS1
CTC-493L21.1
RP5-1177I5.3
RP11-518I13.1
RP11-108B14.5
LINC00929
RP11-48D4.2
RP11-454C18.1
RP11-171N4.4
linc-SCGB1D4-2
linc-C6orf120-3
linc-FAM110B-2
RP11-123O1.1
CTB-4E7.1
linc-SEC11A-1
linc-SULF2-8
linc-INADL
RP1-278O22.2
RP4-601K24.1
CTC-461F20.1
RP11-429J17.5
RP11-431M3.1
linc-TYRP1-5
RP11-64K12.10
PCDH9-AS3
linc-FIGNL1
MAGI2-AS1
linc-EPHA3
linc-C13orf34-19
RP11-465B22.5
RP11-475D8.1
RP11-71H9.2
RP11-4G2.1
linc-GNAL-3
RP11-545P6.2
RP11-324O2.6
linc-MCCC2
RP11-63A11.1
linc-TMEFF2-3
RP11-685B13.2
linc-MBL2-2
RP11-22D3.1
linc-PICK1
linc-TMEM72-2
linc-CBWD3-5
FNDC1-IT1
AP001631.9

RP11-474B16.1
RP11-96D24.1
linc-NBPF15-2
LINC00515
RP11-296K13.4
RP11-165P7.1
linc-CLVS1
linc-ZIC4-4
CTC-295J13.3
CTB-105L4.2
CTD-2227C6.3
linc-LRRC1-2
linc-ZNF32-3
RP11-303E16.6
AE000658.31
linc-ISX-1
CTD-3025N20.2
RP1-71H24.1
AOAH-IT1
RP11-488C13.4
linc-AKAP17A-1
RP11-476H20.1
RP11-109I13.2
CTC-467M3.1
RP11-120M18.2
CTD-2532K18.2
linc-SH2D7-2
CTD-2256P15.4
RP11-418B12.1
linc-GGTLC1-7
CTD-2609K8.3
linc-IFIT2
RP5-1121H13.3
linc-CD93-3
RP11-484O2.1
RP11-353M9.1
RP11-465I4.2
RP11-18H21.3
RP11-953B20.1
RP11-380B22.1
AC012451.1
linc-ANKRD10-6
BACH1-IT3
linc-PRPS1L1
RP3-359N14.2
linc-CA5A-1
RP11-317G6.1
AC005307.1
SYP-AS1
RP11-436F21.1

linc-CDK10
RP11-1094H24.3
RP11-625L16.3
RP11-517O13.1
CTB-22K21.2
RP11-498E2.8
linc-SERPINB3-4
RP11-513I15.6
CTD-2185K10.1
AC007292.6
RP11-320P7.1
RP11-755E23.3
AC092620.3
LINC00987
linc-PELO-1
RP11-71J4.2
RP11-666F17.1
AC005009.1
RP11-30L15.6
RP11-839G9.1
linc-ADPRH
RP1-206D15.3
RP11-97O12.6
AP000439.5
linc-SULF1-1
RP11-327I22.6
CTC-431G16.2
RP11-165M1.2
RP11-215P8.3
ZNF385D-AS1
AC012513.6
RP11-158A8.1
RP11-108K3.1
RP5-1014C4.3
linc-CCM2-1
linc-ATP6AP2-5
RP11-8P11.3
RP5-981O7.2
MIR3179-1
RP11-1E22.1
linc-C20orf202
linc-ZNF167
RP11-3G20.2
linc-CBLB-11
RP11-26J3.3
AC009227.3
RP11-806O11.1
RP11-14O22.1
linc-ANKRD1-2
RNU5A-3P

LINC00879
RP11-50B3.4
linc-PDE7A
linc-TAF1L-2
RP11-484P15.1
CTD-2061E9.1
linc-RAD21-4
RP11-142L1.1
linc-PPP1R1B
CTD-2538C1.3
RP11-316M20.1
linc-MRPS33-2
RP11-552C15.1
KB-1958F4.2
RP11-638F5.1
AC113607.3
STX18-IT1
RP11-395P13.2
AP000998.2
RP11-167N5.5
RP11-13A1.3
RP11-120I21.2
RP11-486F17.1
linc-IER5L-1
linc-PRDM4-3
CTD-2113L7.1
linc-SDCCAG8
RP11-473I1.10
RP11-1C8.7
linc-MKI67IP-2
AC079779.7
RP11-798K3.3
RP11-391J2.3
RP11-887P2.5
RP11-90C4.2
linc-TMEM99-2
RP11-536K17.1
RP11-673F18.1
AP001891.1
linc-SPR-2
linc-CHRNB4
linc-KIF2B-2
RP11-74M11.2
linc-TYR-5
RP11-96D1.10
RP11-458K10.2
linc-AKAP9-3
linc-RNF2-1
HHIP-AS1
RP11-138H10.2

linc-NIPAL2-1
RP11-436H22.1
AC138430.4
linc-GPR123-2
RNF219-AS1
linc-PET112L-3
linc-C1orf124-1
RP11-45A16.4
RP11-375D13.4
RP11-64C12.6
RP11-174J11.1
RP11-452J21.2
linc-PRSS3-3
RP11-552E20.4
AC105760.3
linc-UNC13C-4
RP11-2B6.2
linc-CHD9-8
RP11-369E15.2
linc-C16orf58-1
RP11-410K21.2
RP1-71H19.2
AC026188.1
linc-HMGB1-2
RP11-49O14.3
RP11-1144P22.1
RP11-180K7.1
AP000475.2
AP000462.3
linc-IL1RAPL1
CTD-2195M18.1
RP11-279O17.2
AC099344.2
AC011516.1
RP11-2E11.9
RP11-420J11.2
RP11-486P11.1
RP11-608O21.1
linc-RGMA-8
CTD-2278I10.1
linc-NANOS1-2
linc-WDR91
linc-FOXA1-2
XXbac-BPG27H4.8
linc-DHX30
AC099344.3
RP11-745L13.2
RP11-501J20.5
RP1-12G14.6
linc-LRRTM1-6

CTC-487M23.5
linc-MYCN-3
linc-ARHGEF10
CTC-254B4.1
RP11-365O16.3
RP11-428L9.1
AC003958.2
CTD-2524L6.2
AP000855.4
RP11-1140I5.1
linc-AZI2-2
CTD-3032J10.3
RP11-242J7.1
GS1-279B7.1
RP11-404P21.3
RP11-883G14.3
linc-CYP7B1-3
linc-CLDN5-2
CTC-296K1.3
RP11-705O24.1
RP11-500K19.1
RP11-469L4.1
AF011889.2
RP11-16C1.3
RP11-1038A11.1
MAPT-AS1
RP11-815J4.1
RP11-77G23.2
RP11-1124B17.1
linc-BNC2-2
RP11-521B24.5
linc-SCN11A-2
RP11-383C6.2
CTD-2369P2.4
RP11-43D2.2
AC068491.2
RP11-147O5.1
linc-DNM1L
CTD-2196E14.3
RP11-119F19.2
RP11-257A22.1
AC063976.3
linc-RGS8-3
RP11-12K22.1
linc-TBX3-2
LINC00974
linc-BRI3BP-2
CTD-2544M6.1
RP11-524C21.2
linc-EPB42

RP11-728F11.4
linc-EPHB2
RP11-402L1.4
AC104623.2
RP11-177F15.1
linc-EIF2C4-2
RP11-7I15.3
AL133247.2
RP1-90G24.10
CTC-471C19.2
RP11-168K11.2
RP11-379K22.2
RP11-339A11.2
linc-HAAO-4
linc-SATB1-2
AC019186.1
RP11-569A11.2
RP11-297M9.2
linc-FABP1
RP4-705O1.1
RP11-30K9.4
RP11-415J8.7
linc-FAM114A1-1
RP11-353N14.3
linc-ARL1-1
linc-ALK-2
linc-TMEM132B-2
RP11-143G3.1
RP4-671G15.3
RP11-152K4.2
RP11-162D16.2
linc-SEL1L-9
linc-ST6GAL2-3
RP11-507J18.2
AC069363.1
linc-THSD4
RP4-794H19.4
linc-RAB2A-1
LINC00954
linc-PRH2-1
CTD-2501E16.2
CTD-2323K18.1
linc-ZNF74-1
RP11-350F16.1
linc-AKR1E2-12
RP11-179A10.1
RP11-216M21.7
linc-KCNMB2-10
AP003774.5
RP11-20F24.2

MLK7-AS1
CTC-360P9.3
linc-RDH10
RP11-253M7.6
linc-GRPEL1-2
linc-MKLN1-1
RP5-837M10.4
RP1-46F2.2
AC011525.2
AC068039.4
linc-RAB6C-3
RP11-91K8.1
RP11-359E19.1
RP11-1101K5.1
RP5-1050E16.2
HOXB-AS4
linc-ATXN10
AC002511.2
ATG10-AS1
RP11-381P6.1
RP11-556N4.1
AC067956.1
linc-COG6-2
RP11-139K4.1
RP3-337H4.8
RP11-351A20.1
AC004593.3
linc-XRCC6
RP11-91P24.5
linc-PCDH20-2
linc-C1orf151-2
RP4-778K6.3
RP11-255B23.4
RP11-552E10.1
TTLL11-IT1
AC007193.10
AC114730.5
CTD-2086O20.3
RP11-317M11.1
RP11-119D18.1
LINC00393
CTD-2105E13.13
RP1-266L20.9
linc-ACP6
RP11-102F4.3
linc-IRX1-7
linc-PRDM11-1
RP11-79N23.1
linc-AGA-8
linc-RHOB-9

RP11-420A6.2
linc-ZRANB1
RP11-686D22.4
linc-TTC7A-2
RP11-707M3.3
RP11-174G17.2
RP11-21L19.1
RP11-555M1.3
RP3-395P12.2
RP11-554I8.1
AC104532.4
linc-TMEM90B-6
RP11-140H17.1
linc-CHL1-1
RP11-660M5.1
RP11-764E7.1
RP11-293P20.4
RP11-114H21.2
CTD-2384A14.1
CTC-458G6.2
linc-P2RX5
RP11-977G19.14
AC002306.1
linc-ANKRD50-5
linc-ZNF337-8
CTD-2308B18.1
linc-FOXP3
RP11-442J17.2
linc-NCAM1-2
RP11-760D2.1
HAR1A
AC068196.1
CTC-436K13.6
linc-TFAP2A-2
RP11-678G15.2
linc-VSNL1-2
RP11-615J4.3
RP11-1079K10.4
RP11-531A24.3
RP11-290L1.5
linc-NMNAT2-2
CTD-2616J11.9
RP11-701I24.3
RP11-160E2.16
RP11-989F5.3
RP11-124N3.3
RP3-416H24.1
linc-TCF7L2-1
CTD-3222D19.10
linc-ANKRD10-8

RP11-454P21.1
RP11-375O18.2
linc-CADM2-2
AC004543.2
linc-RPGRIP1L
RP11-509E10.1
RP11-46D6.1
RP11-379F4.8
RP11-654D12.2
AC144833.1
linc-ITIH2-8
KCNJ6-IT1
RP11-119K6.6
RP11-395P13.5
AC093609.1
linc-APBA2-3
RP11-617D20.2
LINC00689
linc-CLEC2D-5
RP5-1030M6.3
AC097499.1
linc-HRCT1
linc-WRN-1
RP3-462E2.5
RP11-231E4.3
RP5-1070A16.1
RP11-96H17.2
RP11-281N10.1
RP11-394A14.2
RP11-561I11.3
AC005152.2
RP1-111D6.3
RP11-300M6.1
LINC00354
RP11-420K8.1
RP11-145G20.1
RP11-167P11.2
LINC00635
MTOR-AS1
RP11-105N14.2
linc-NPVF-1
RP11-760L24.1
AC004775.5
RP11-267A15.1
RP11-849F2.5
linc-ERI1-3
linc-C9orf135-1
RP1-56L9.7
RP11-61A14.3
RP11-6O2.4

RP11-21B23.2
CTC-250I14.3
RP1-149A16.17
AL035610.2
RP11-42O15.3
linc-PRKACB-2
linc-GPC5-1
linc-METTL13-2
RP3-332B22.1
RP11-432I5.4
RP11-379L18.1
RP3-477O4.14
RP11-178L8.5
RP11-567L7.5
RP5-1051D14.1
RP11-730N24.1
linc-RALGAPA1-3
RP11-736N17.8
FEZF1-AS1
linc-PCDH8-3
RP11-230L22.4
linc-SIK1-1
RP1-85F18.6
linc-MTDH
linc-ANKRD30A-2
RP11-522B15.7
linc-SLC6A19-1
AC007317.1
LINC00457
RP1-166H4.2
RP1-54A3.1
RP11-161I10.1
linc-ZNF479-3
linc-STIL-4
RP11-288L9.4
RP11-21L23.2
U95743.1
RP11-325D15.2
RP11-105C19.2
linc-EOMES-2
linc-DET1-2
linc-COX5B-3
RP11-57C13.6
RP11-470L19.5
RP11-557L19.1
RP11-775H9.1
RP4-803J11.2
LINC00500
linc-CLK4
RP4-604K5.3

ZNF503-AS1
SRGAP3-AS4
RP11-451G4.3
linc-MGAT5B-2
CTD-2023N9.1
RP3-467K16.7
RP11-141O11.1
linc-GREB1-1
RP11-362A1.1
LINC00615
AC100848.1
AC113610.1
linc-CILP
RP11-61L14.6
RP11-11K13.1
RP11-165I9.8
RP11-415L24.1
RP11-341G23.3
linc-KRTAP11-1
linc-INO80
RP11-462G2.1
RP11-820I16.1
RP11-116N8.1
RP11-87N24.3
RP11-744J10.3
RP11-486M23.1
RP11-242P2.1
CTD-3224K15.3
RP11-354I13.1
RP11-227F19.5
linc-SMPD1
linc-KCNAB2-1
linc-KLHL7-2
linc-HLCS
linc-OTUD7A-1
LINC00922
linc-ZNF716-5
RP11-796I2.1
RP11-171I2.4
TRIM31-AS1
AF127577.8
RP11-413E1.4
RP11-763B22.6
AC103563.9
LHFPL3-AS1
CTD-2125J1.1
AC023115.1
linc-OPN4-5
linc-IQCG-1
AC013248.2

linc-NRF1
linc-HSCB-4
linc-DTHD1-1
linc-ZFAT-4
RP11-304F15.3
linc-IGFBP3-4
CTD-2184D3.7
CTD-2341M24.1
RP11-360I20.2
RP11-266J6.2
CTD-2206N4.2
RP11-214C8.5
CTB-1H10.1
AC104777.1
linc-LRRTM4-3
RP5-1101C3.1
RP11-375B1.2
linc-FAM92B-2
linc-XRCC4-3
RP1-90L6.2
MIR744
CTD-3037G24.3
linc-PSORS1C1-2
linc-CBX7
PLCH1-AS2
RP11-214K3.22
AC012613.2
RP11-798G7.5
TRPC5OS
linc-PRDM4-1
AC007743.1
RP11-610J23.1
RP11-296I10.3
linc-PROM1
linc-CFH-1
linc-ACTL7A-9
RP11-350D17.2
linc-SH3BP4-1
RP11-455F5.4
linc-MEF2D
CTB-147C13.1
AC004862.6
AC002128.5
linc-SRSF7
linc-MARCO
linc-VIM-2
linc-LOC100129335-4
AC003682.16
linc-HS3ST1-6
RP11-752L20.5

RP11-231I13.2
linc-ITIH2-1
RP11-53B2.3
RP11-83J21.3
RP11-13E1.5
WARS2-IT1
RP11-20B24.5
HLCS-IT1
GRTP1-AS1
RP4-781K5.7
RP4-647C14.2
SSPO
RP11-179A16.2
linc-BAT2L1
RP11-13K12.1
RP11-321G12.1
AC011897.2
RP11-449O16.2
U3
RP11-461C13.1
linc-HMX1-5
LINC00521
linc-SOD1-2
linc-TP53
RP3-471M13.2
AGBL4-IT1
RP3-326L13.2
RP11-98D18.16
RP11-188C12.3
RP11-95K23.5
RP1-261G23.5
RP11-625L16.1
RP11-434D2.7
AC093702.1
RP11-690G19.3
AC013480.2
RP11-87C12.5
linc-LAMA1-4
RP11-557C18.3
RP11-94P11.4
AC090286.4
CTD-2083E4.7
RP11-136K14.2
AC130710.1
AC005534.9
linc-TTC14
RP11-392P7.7
RP11-567M16.5
AC005281.2
AC023128.1

CTD-2336H13.2
LINC00202-1
CTC-261N6.1
AC005042.4
RP1-130H16.16
linc-HNF1B-2
AL122127.25
RP11-57P19.1
linc-SLC6A9
RP11-95L3.2
RP11-111H3.3
RP11-47J17.2
CTC-419K13.1
linc-LYZL1-1
linc-INHBB
linc-HTR2A-4
U51244.2
RP11-331K21.1
linc-COL5A1-2
RP11-236B18.2
RP11-656D10.5
linc-CD5L-1
RP4-724E13.2
AP000997.1
RP11-247I13.8
RP3-449M8.9
linc-PPWD1
AC087393.1
linc-SCPEP1
RP11-547C13.1
AIRN
RP11-572C21.1
RP1-163G9.2
AC004869.2
AC007163.3
RP4-740C4.9
ADAM6
AC013727.2
AC053503.6
linc-PPIL5-3
AC007349.7
AC068134.8
linc-C8orf79-1
RP5-856G1.1
LINC00297
CTD-3116E22.7
RP11-357D18.1
linc-UBE2E3-2
RP11-762I7.4
RP11-158I9.7

RP11-505P4.6
linc-CRH-2
RP11-313E19.2
LINC00609
linc-PRSS12-1
RP11-213G21.1
linc-LYZL1-6
linc-ST8SIA4
linc-KLHL29-8
linc-KIAA1524-1
linc-DACT2-7
linc-TNFAIP3-4
RP11-20A20.2
CTA-249B10.1
linc-C13orf34-15
linc-CD9-1
RP11-366O17.3
AC034228.2
RP11-167B3.1
RP11-252M21.7
GPR50-AS1
CTD-2526M8.3
RP11-797H7.5
RP11-369C8.1
linc-ZNF726-1
RP11-413G15.1
linc-PHF21A-1
RP11-354P11.4
linc-IGSF10-1
RP11-384P7.5
linc-TSPO2-4
CAMTA1-IT1
linc-SSTR4-2
RP11-380L11.4
linc-ZNF716-8
linc-CDH5-6
linc-TMCC3
XXbac-B476C20.14
LINC00200
AC002465.2
linc-MAP1LC3B-6
RP11-319E16.1
RP11-347D21.2
RP11-321N4.3
linc-ADARB2-5
BSN-AS1
AC007278.2
RP11-119D9.1
RP11-794A8.1
linc-EHD3-2

LINC00474
RP11-815J21.3
AC099754.1
CTB-77H17.1
CTD-2616J11.2
linc-BTC-7
AC007563.3
RP11-8L2.1
AB015752.3
RP11-309L24.4
RP11-87E22.1
linc-ZNF707
linc-CXorf27-1
AC011518.2
AC000099.1
linc-CACNA1A-3
linc-C15orf2-4
CTC-756D1.3
RP11-51M18.1
CTD-2017C7.1
RP11-973H7.1
RP11-111F5.3
RP11-550A5.2
RP11-328K15.1
RP11-5N11.4
CTD-2363C16.2
AC010729.1
RP11-10N23.4
RP11-460I19.2
linc-TBPL2-2
RP11-762E8.1
linc-C11orf82-6
AC007040.8
CTD-2201E9.4
linc-CENPQ-2
RP1-118J21.5
RP11-1166P10.8
linc-SERPINA12
RP11-5P4.3
linc-IRAK1BP1-2
RP11-521D12.2
RP11-521M14.1
linc-TTC7A-3
RP1-60O19.2
RP11-435D7.3
RP11-1C1.4
RP11-503C24.6
AC003973.5
RP11-159D23.2
RP11-117D22.1

linc-ATP13A4-3
RP11-521L9.2
FAM66E
linc-NEU3
RP11-49L2.1
linc-OTOS-1
LINC00664
ARHGEF38-IT1
CTC-251D13.1
linc-C13orf28-2
AL133168.3
RP4-669L17.2
RP11-473I1.5
linc-SAMSN1-5
RP11-571M6.7
linc-HSF2-2
linc-SLC25A32-3
RP4-568F9.3
RP11-53B5.1
RP11-59N23.3
linc-RNF121
RP11-77B22.2
RP5-991C6.4
linc-SLITRK1-5
AC073635.5
RP11-419C23.1
linc-UBASH3B-3
linc-ZNF107-5
RP11-407G23.3
CTD-2024D23.1
linc-CPEB4-2
RP11-545I10.2
linc-TMEM5
RP11-576N17.5
linc-WDR26-1
RP11-216P16.2
RP11-802E16.3
linc-MSR1-3
linc-LRRN3-1
RP11-540O11.6
RP11-141J10.2
CTD-2074D8.1
RP11-100M12.2
linc-UTP23-4
linc-CHD9-11
CTB-174O21.2
RP11-70C1.1
CTD-2006K23.2
AC007966.1
RP6-91H8.1

CTD-2377D24.8
linc-NUDT12-1
AC127904.2
linc-SLC9A3-2
RP11-192P3.5
RP11-672L10.2
AL845154.2
linc-RPP30-5
linc-S100G
RP11-691H4.4
LINC00654
RP11-321E2.4
LINC00852
AL157359.3
RP11-680N20.1
RP11-222K16.1
RP11-433J22.3
RP11-297L17.4
RP11-219J21.2
RP11-464D20.6
linc-P2RY1-1
linc-SP110-3
CTD-2532D12.5
RP11-402F9.3
RP11-554K11.2
RP11-196H14.3
RP11-149P24.1
RP11-509J21.2
AP001255.2
linc-GLI3-3
linc-LHFPL4-2
LINC00461
LINC00491
RP11-709D24.5
RP11-134F2.2
RP11-264I13.2
RP11-692C24.1
RP11-168K9.1
CTD-2291D10.1
AC006372.6
RP11-698N11.2
AC009411.1
CTD-2044J15.2
linc-SNRPB2-1
CTD-2187J20.1
GS1-122H1.2
CTC-503K11.2
CTD-2034I4.2
RP11-78O9.1
LINC00617

linc-ATG10
CTD-2566J3.1
RP11-446E9.2
linc-NDUFB4-4
linc-DLK1-3
GRM5-AS1
RP11-502H18.2
RP11-146N18.1
DLEU7-AS1
RP11-308B5.2
RP11-543N12.1
RP11-1078H9.1
linc-NDST3-8
linc-PTPRM
ATXN8OS
linc-LZTS1-3
AC009410.1
linc-PELI1-5
RP11-62F24.1
linc-CCDC140-10
linc-ING2
TBX5-AS1
AC009492.1
RP11-89N17.3
FOXP1-AS1
RP11-28G8.1
AP001597.1
AC012442.5
RP5-850O15.3
RP11-955H22.3
RP11-508P1.2
NCKAP5-IT1
linc-ST6GAL2-9
RP11-245G13.2
linc-VEGFC-2
linc-SLC35B3-BP
RP11-526H11.1
CTC-304I17.2
AF240627.2
RP11-267N12.1
AC109828.1
AC007193.6
SIX3-AS1
AC068490.1
CTD-2215E18.2
RP11-469M7.1
AC018766.6
linc-QSOX1
RP11-403I13.4
RP11-320H14.1

RP11-543F8.2
RP11-290F5.1
RP11-87H9.3
linc-LARGE-4
linc-NUBPL-1
linc-PBX1
AP000221.1
linc-FAM102A
RP11-538D16.3
linc-CLEC2D-6
RP13-977J11.8
RP11-107I14.4
RP11-286E11.1
linc-PKN2-2
linc-CYP4B1-1
RP11-90P5.2
CTD-2147F2.2
AC024084.1
AC018742.1
TRAPPC12-AS1
RP11-789C17.1
C9orf135-AS1
RP11-370P15.2
linc-CNTNAP2-3
RP11-80H5.6
linc-GPR39-2
linc-NKX2-2-4
CTC-559E9.5
RP11-513H8.1
RP11-575F12.1
AF127936.7
CCDC39-AS1
RP11-175E9.1
RP1-249I4.2
RP11-62I21.1
RP11-521O16.1
CTD-2228A4.1
RP11-302F12.3
linc-TRPC4-1
RP11-547C5.2
RP11-690D19.3
linc-CCDC140-9
linc-FAM5C-4
CTC-453G23.5
RP4-734C18.1
linc-SEPSECS-2
AC006126.4
linc-FSIP2
RP11-67C2.2
CTD-2176I21.2

KB-1254G8.1
FLNB-AS1
RP11-160H12.2
linc-C17orf108-4
linc-NID2-2
RP11-403A21.1
RP11-85G21.3
AC138655.6
linc-MAF-5
CTD-2287N17.1
RP11-416O18.1
linc-PAPPA-2
LINC00359
linc-DHX37-22
RP1-97J1.2
linc-LRRC4-2
linc-DYDC1-6
linc-PDSS1-3
RP11-379K17.11
GS1-39E22.1
RP11-159D12.2
AC007389.3
RP11-753A21.1
RP11-36B15.1
MACROD2-AS1
RP11-707F2.1
RP11-44F21.2
AC079767.4
CTB-26E19.1
RP1-135L22.1
linc-VIPR2-5
RP11-932A10.1
linc-SLITRK5-8
AC005262.2
RP5-1059M17.1
linc-UBXN2B
RP13-126C7.1
linc-AGA-3
CTD-2130O13.1
linc-AKR1C2-2
RP11-272D12.2
AP006621.6
RP11-553N19.1
AC004840.8
RP11-329N22.1
AC090939.1
linc-SHISA6-2
RP11-382A20.2
RP11-574O16.1
linc-PROK2-1

RP11-456O19.3
RP11-831H9.3
linc-C1QL2
RP11-174M13.2
RP4-662A9.2
CTC-457E21.6
linc-FRS2-2
AC012361.1
RP11-430C7.4
RP1-92O14.6
linc-OTUD4-2
RP11-49I4.3
linc-MKRN3-2
AC114730.3
linc-IARS2-3
LINC00989
linc-SPRY2-1
RP1-59D14.1
linc-TMC3-4
AC006116.24
linc-CHST9-2
linc-C18orf62-4
RP11-1112C15.1
linc-ZNF462-3
AE000658.22
linc-ECHDC3-1
linc-PPARGC1B
RP11-687F6.1
XXbac-BPG154L12.4
RP11-432J9.5
CTD-2275D24.3
LINC00558
RP11-756J15.3
AC090505.4
RP11-23N2.4
RP5-1052I5.1
linc-ZNF16
linc-FOXO4
linc-PAFAH2-2
linc-CRP
CTB-113P19.3
RP11-307C19.3
AC010731.3
RP11-666N19.1
THOC7-AS1
linc-MAP1LC3B2-8
RP11-460B17.2
RP11-345M22.3
RP11-554D15.1
AC069257.8

RP11-543H12.1
CTD-3076O17.1
linc-JPH1-1
RP11-18B16.2
RP5-837J1.2
RP11-92K15.1
RP4-798A10.2
linc-BDH1-3
RP11-94B19.3
RP11-728E14.2
AC005307.3
linc-C14orf101-4
MIR194-2
RP11-299G20.3
RP11-930P14.1
RP11-321C24.1
MEG3
AC016735.3
linc-ZNF479-11
linc-WNT3
RP11-424I19.1
linc-CNTNAP4
AC008992.1
linc-CDH6-3
RP11-68L1.1
CTD-2340D6.2
RP11-40C11.2
linc-CDR2-1
CTD-2050E21.2
LL22NC03-121E8.3
linc-PANK1-5
RP11-35J23.1
RP11-185P18.2
RP11-80K21.3
CTD-2023J5.1
linc-SHOX-1
RP3-495K2.1
RP11-366H4.3
LA16c-349E10.1
AC018464.3
linc-FRG2B
RP11-16L9.2
RP11-470E16.1
AC124861.1
CLRN1-AS1
AC019172.2
linc-C8orf83-2
linc-CADM1-1
linc-SLC46A2
linc-CNTNAP5-1

linc-ALDH1A1-4
LINC00343
LINC00283
linc-ZMAT4-1
RP11-108M21.1
RP1-170O19.14
RP1-27K12.4
linc-PAPPA-5
CTD-2008P7.3
linc-BCL3
CTD-2015G9.1
RP11-718B12.2
RP11-449P15.1
AP000472.2
linc-GLI2-1
linc-PCDH15
XXbac-BPG254F23.7
RP11-46O21.2
RP11-288G11.3
RP11-429J17.7
linc-MLPH-3
AC006369.3
linc-C5orf43-1
RP11-481J2.1
linc-DLGAP2-4
RP11-794P6.3
RP11-15B24.5
RP11-582E3.2
linc-SLC28A3
linc-TRPS1-1
AC007106.1
CTC-293G12.1
ACTA2-AS1
RP11-1084I9.1
linc-FLRT3
linc-PTPN20B-1
RP4-594A5.1
RP11-818F20.4
CTD-2024P10.2
RP5-1154L15.2
linc-JPH4-2
linc-PGLYRP2-2
linc-CELF2-9
linc-ULBP3-2
linc-ELTD1-3
RP11-624G17.3
RP11-727M10.1
linc-MAP3K8-6
linc-ROPN1B-1
RP5-1177M21.1

CTD-2050B12.1
linc-MRPL22
RP11-774O3.1
AC004051.2
AC092168.4
AC005606.14
RP11-82L20.1
CTD-2555A7.2
RP11-464O2.2
RP11-437J19.1
linc-EIF5AL1-1
CTC-1337H24.1
linc-MASTL
CTD-2547H18.1
linc-C20orf72
RP11-120A1.1
linc-ZFAND3-2
RP11-436M15.3
RP11-756P10.2
GPC6-AS2
linc-COX4NB-1
RP11-469J4.3
linc-SPRY2-3
linc-HEATR2-3
RP11-155O18.6
RP5-1132H15.1
RP11-122K13.12
linc-CCDC140-6
SPATA8-AS1
RP11-434B12.1
RP4-753D4.2
RP11-542C10.1
KB-1458E12.1
AC016682.1
RP11-855O10.3
CTD-2151A2.1
linc-FAM82A1-4
linc-PAX9-2
linc-KCNMB1-2
RP13-379L11.2
CTD-2071N1.1
RP11-466P24.6
RP11-288H12.4
AF212831.2
CTD-2194A8.2
RP11-228B15.4
RP11-231G15.2
RP11-518L10.5
RP11-101E7.2
RP11-286H14.8

LINC00264
RP11-46C24.6
C4B-AS1
RP11-1379J22.2
linc-PPFIA2
AL353997.3
RP11-481J13.1
linc-PPP2R2D-4
AC114808.2
RP11-941H19.3
linc-KIAA1024
RP11-692C24.2
AC006547.8
RP11-1100L3.8
RP13-895J2.7
RP11-435O5.7
linc-LINS-2
RP11-148B3.1
RP11-498D10.5
linc-FAM120B-8
linc-ZBTB44
RP11-770G2.2
AP000290.7
RP11-212I21.2
RP11-346J10.1
linc-CX3CR1-1
CTD-2043I16.1
linc-FAM75C2-2
AC073842.19
RP11-543B16.3
CTD-2313F11.1
AP001610.9
LINC00443
RP11-80G7.1
linc-GPC5-6
linc-KIAA1383-3
RP11-636O21.1
RP11-429K17.1
CTD-2066L21.2
RP1-69M21.2
RP11-85L21.4
RP4-784A16.2
linc-TRIM38
CTA-342B11.1
CTD-2587H19.2
AC002398.13
AC091177.1
linc-SH3RF3
linc-C7orf27-1
AC092159.3

linc-LMAN1-1
LINC00315
AC090627.1
CTD-3187F8.2
RP11-1085N6.6
LARGE-AS1
RP11-588L15.2
RP11-218F4.1
RP11-379J5.5
RP11-7O14.1
linc-SNRK
RP11-798K3.4
linc-FOXF1-6
LINC00633
RP11-388K12.2
RP11-148O21.6
RP1-67M12.2
linc-NHLH2-6
RP11-108P20.1
RP11-309N17.4
CTD-2537I9.13
AC092570.2
LINC00845
RP11-259O2.2
AC019117.2
CTD-3253I12.1
snoU13
RP11-570K4.1
linc-GNPDA1
RP11-673P17.4
SYNPR-AS1
linc-UTS2D-2
RP11-619A14.2
RP11-809M12.1
AC109826.1
RP11-489G11.3
linc-CENPP-9
HAO2-IT1
linc-CHRM3
RP3-508I15.14
AC005394.1
RP11-359G22.2
RP11-332K15.1
RP11-662J14.2
linc-ZNF33A-5
linc-TCTE3-7
RP11-150O12.1
linc-FAT1-9
RP11-573E11.2
RP11-701P16.4

AP001469.7
linc-FIGNL2-1
linc-FAR2-1
GRIFIN
CTD-2540B15.12
AC005330.2
RP11-304L19.12
AC006547.13
RP11-568J23.5
linc-MC3R-1
CTD-2576F9.2
RP11-115E19.1
linc-PDE3A-1
RP11-353N4.1
RP11-433M22.2
RP11-812E19.7
RP11-363E7.4
RP11-106M7.1
CTC-471J1.9
AC138472.6
RP11-711C17.1
RP11-94H18.1
linc-STIM2-3
RP11-315H15.1
CTA-941F9.9
linc-SYK-2
RP4-594I10.3
RP13-297E16.3
RP11-386G11.10
linc-STIM2-1
CTC-459M5.2
RP11-419C19.2
RP11-417E7.1
linc-SNTG2-6
linc-CYLC2-2
RP11-490N5.2
AC008277.1
AC005324.7
linc-CCDC111-4
RP11-45A12.1
linc-TMEM132C-15
linc-ERGIC1-1
RP11-17A19.2
LA16c-395F10.2
RP11-545A16.1
RP11-879D6.1
RP11-536K7.5
linc-TSN-3
AC017053.1
RP5-1033H22.2

RP11-59H7.3
RP4-565E6.1
RP11-285A1.1
AC011754.1
linc-C17orf72
RP11-74C3.1
linc-TMEM132D-3
RP11-84O12.4
linc-ADAM2-2
RP11-67K19.3
CTD-2521M24.6
LINC00242
linc-MNAT1
CTD-2139B15.5
AC004448.2
linc-TMCC1-1
linc-GGTLC1-2
RP11-1090M7.1
AC012354.6
linc-FAM92B-1
RP11-555H7.2
RP11-519C12.1
linc-SEZ6-1
linc-MPRIP-1
linc-CLDN24-1
linc-TP53I11-2
RP11-718G2.5
linc-CARD11-10
RP11-165J3.6
CTC-490E21.10
RP11-143K11.1
linc-TRIML2-5
CTB-113P19.5
AC079150.3
RP11-216N14.8
RP5-836J3.1
RP11-499O7.7
ADIPOQ-AS1
RP11-662G23.1
AP000476.1
RP11-342H21.2
RP11-15K3.1
RP11-54O7.1
RP11-775D22.3
RP11-121C2.2
RP11-58O15.1
RP11-38J22.3
RP11-504P24.6
RP11-104J23.1
RP5-1154L15.1

linc-NTRK2-3
RP5-963E22.4
CTD-2128A3.3
linc-CEP110-14
CTD-2004A9.1
CTD-3135A9.3
RP11-21C17.1
CTD-2636A23.2
RP5-984P4.4
RP11-770E5.2
linc-C10orf137-3
RP11-334A14.5
linc-C18orf62-6
linc-DUSP26-4
SMCR2
linc-KIAA1712-4
AC109333.10
AC087501.1
LL0XNC01-237H1.2
RP11-140I24.1
RP11-1057B8.2
AC011286.1
RP11-108P20.3
linc-CDH6-4
linc-ISLR2-3
linc-ZFHX4-2
RP11-307C12.11
RP11-284H18.1
RP11-493P1.2
RP11-12A2.3
RP11-277L2.5
RP11-301J7.8
RP11-439L18.2
LA16c-395F10.1
RP11-341G23.2
AP000477.3
linc-TIMP4
linc-EPHB3-2
CTB-60E11.4
RP11-76E17.3
linc-ZSCAN2-2
RP11-11N5.1
RP11-450K4.1
RP11-543E8.1
RP11-234B24.4
linc-SERPINI1-5
RP11-866E20.3
RP11-687M24.4
RP11-813F20.2
RP1-39J2.1

RP11-439C15.4
linc-FTH1-3
RP11-621L6.2
RP11-384C4.7
AC107399.2
RP11-258O13.1
RP11-243M5.1
RP3-513G18.2
linc-MRPL19-2
linc-ZBED1-2
RP5-858B6.1
RP11-493K23.1
linc-MACROD1
RP11-7K24.3
RP5-1028L10.1
RP11-39E3.3
linc-SMC1B-6
RP11-490O24.2
CT60
linc-LOC642587-4
RP11-1338A24.1
linc-AMN1
AC079135.1
CTB-92J24.2
FER1L6-AS1
RP11-795F19.1
AC034110.1
linc-ODZ3-3
AC068538.4
RP11-1060G2.2
RP11-83A16.1
RP11-298P3.4
RP11-122K13.14
RP11-574O7.1
AC015849.16
RP11-517B11.4
AC073094.4
RP11-476M19.2
RP11-565F19.4
PRSS51
linc-GLRB-2
RP11-648L3.2
AC010987.5
RP11-403H13.1
linc-HOXA13-1
RP11-101E14.3
RP11-91K11.2
RP1-29C18.10
RP11-533E19.6
RP11-632K5.2

linc-ZNF726-8
linc-ZNF492-3
CNTN4-AS2
RP11-181G12.2
CTC-448D22.1
RP5-1121A15.1
RP1-142L7.5
linc-SYK-1
RP11-292E2.1
RP11-53L24.1
RP1-257I9.2
linc-FOXG1-8
RP11-461O7.1
AC073928.2
RP5-907D15.3
LINC00919
linc-SULF2-5
RP11-35P15.1
linc-DCAF17-3
CTD-3126B10.4
AC011537.3
RP11-26J3.1
RP11-378E13.3
RP11-152P17.3
RP11-355O1.11
RP11-162D9.3
CTD-2325M2.1
linc-NFIL3
HCG24
RP13-60M5.2
RP11-617F23.1
AF011889.6
RP11-301H24.3
RP11-299H22.6
RP11-472K22.1
AC004063.1
linc-ENKUR-2
AC116035.1
RP11-586K12.11
RP11-348J24.1
GRM7-AS1
WWC3-AS1
RP11-426J5.2
RP11-562A8.5
AC005329.7
CTC-353G13.1
linc-PRSS3-5
linc-RNFT2-1
GCSAML-AS1
RP11-214K3.23

AC023469.2
AP005530.2
RP11-676J12.6
RP11-767C1.1
CTA-984G1.5
RP11-108M9.2
linc-SLC35F5-1
linc-CCNG1
ST8SIA6-AS1
RP13-631K18.2
linc-XRCC2-1
CTD-2647E9.3
RP11-471M2.3
RP11-332J15.4
linc-CDH2-1
CTC-265F19.2
RP11-716H6.1
RP11-715G15.1
CACNA1C-AS3
RP11-720L2.2
AC068057.2
RP11-638I2.8
AC007099.1
RP11-198M11.2
RP11-415I12.3
linc-RBFOX2-1
RP11-28O3.2
RP5-1170K4.7
CTD-2192J16.21
LINC00159
RP11-527N12.1
linc-TMC7-2
RP11-403A21.3
AC000067.1
TLR8-AS1
linc-C1orf14
RP11-285F7.2
linc-ARID1B-1
RP11-22P4.1
AC008984.2
RP11-266E6.3
RP11-91P24.7
RP11-102C16.3
AC093838.7
linc-ANAPC13
linc-RYR2-2
AC093171.1
linc-SLC30A4-2
CTD-2366F13.2
linc-AGAP9-2

RP4-610C12.3
RP11-567G24.3
RP11-1007I4.1
NR2E3
RP1-17K7.3
RP11-597A11.6
RP11-731N10.1
AC079613.1
RP11-706C16.8
RP11-66H6.3
FAM74A3
RP11-983C2.2
CTC-369A16.2
RP5-1180C10.2
RP11-255M2.2
CTA-384D8.31
RP11-25J23.3
RP11-1024P17.1
RP11-342K6.4
linc-CTNNB1-3
linc-DHX37-7
CTD-3080F16.3
AC016768.1
RP5-899B16.2
linc-GTPBP8
CTD-2231H16.1
AC002511.3
CTD-2139B15.2
RP11-318K12.2
linc-ALK-1
CTD-2521M24.5
linc-TMEM207-1
LINC00605
RP11-132N15.3
RP11-432J9.3
RP11-463I20.2
RP11-21B23.3
linc-SNTG2-3
linc-LOC100130274
linc-FAM91A1-3
linc-OR5AC2-1
RP11-542A14.2
CTD-2540F13.2
linc-MPHOSPH8-6
linc-NR4A3
RP11-37E23.5
linc-CSMD2
RP11-696F12.1
linc-DUSP4-5
RP11-24I21.1

RP11-1109M24.16
RP11-358H18.3
linc-EN2-1
CTC-428H11.2
linc-HFM1-2
AC102953.6
RP5-894D12.5
linc-C9orf172
CTA-31J9.2
RP11-580I1.1
MIR1255B1
RP11-536C5.2
RP5-931K24.3
RP5-940J5.6
linc-SESN3-1
RP11-497H16.9
AC092431.3
RP11-54A4.2
RP11-420M1.2
AC078852.1
TTLL10-AS1
linc-MED1
linc-ULBP2-2
linc-RBKS-5
AC018804.3
linc-CITED2-5
RP4-640H8.2
AC009542.2
linc-RAB8B
LINC00345
FAM138A
RP11-556H2.4
RP11-855C21.1
linc-CTTNBP2-6
AC005772.2
RP11-133L14.5
RP11-546M21.6
RP11-121L11.2
RP11-284P20.3
RP11-144L1.4
RP11-23E19.1
ADAM20P1
RP11-342C24.8
CTC-340D7.1
AC107079.1
linc-CELF4-8
RP11-470F18.1
IMMP2L-IT1
linc-YWHAQ-1
RP11-84D1.2

RP11-386M24.9
RP13-577H12.2
RP4-809F18.1
RP11-149B9.2
KB-1507C5.3
linc-ZNF367-2
RP11-701H24.7
linc-APPL2-1
RP11-410N8.3
RP11-598D14.1
RP11-6B4.1
RP11-13B9.5
linc-C8orf86-3
OTX2-AS1
RP11-81F13.2
linc-GALNT9-1
linc-TCF24
RP11-697M17.2
RP11-883G14.1
RP1-168P16.2
AP000797.4
RP11-148L24.1
linc-NDRG1
RP11-656A15.1
RP11-429P3.5
LZTS1-AS1
RP11-75C9.2
RP11-35G22.1
linc-CDYL-2
linc-ANKRD1-1
AC108056.1
RP11-1006G14.1
AC097468.4
RP11-177B4.2
RP1-79C4.4
LINC00162
linc-MAP3K8-1
RP11-337A23.6
linc-HUS1B-1
RP11-706J10.3
AC098973.1
RP11-400N13.3
HTR3E-AS1
RP11-158I3.3
CTD-2015G9.2
RP11-983P16.2
LINC00851
RP11-45F15.2
AC104532.3
RP11-263K4.4

RP11-469H8.8
linc-FNDC1-2
RP11-169D4.1
RP11-255G21.1
RP11-661C8.2
LINC00557
RP11-520D19.1
CTD-2653B5.1
linc-ADAR
CTD-2562J17.9
linc-TSHZ3-2
RP11-503C24.4
linc-DAOA-1
linc-FLJ43860
RP11-691H4.3
RP11-259P1.1
linc-C1orf174-1
RP11-390B4.3
MAST4-IT1
RP11-227H15.5
RP11-162K11.4
linc-MDGA1-1
linc-SMCHD1
RP5-872K7.7
RP1-59D14.3
RP11-613F22.8
RP5-1139I1.2
RP11-408B11.2
RP11-277P12.9
RP11-672A2.5
linc-FRG2C-5
RP11-472G21.2
MYO16-AS1
AC147651.1
CTD-2050E21.1
RP13-614K11.2
RP11-731K22.1
RP11-2C24.6
RP11-496D24.2
RP11-3K16.2
RP11-132M7.3
RP11-684B21.1
RP13-507P19.2
RP11-307C19.1
FAM225B
RP11-278I4.2
linc-C16orf78-2
linc-S1PR1
linc-NFE2L3-2
linc-TMC7-1

AC084193.1
linc-MKI67-3
CTC-325J23.2
AC005042.5
RP11-476H24.1
linc-SLC9A3-1
linc-WFDC12
linc-API5-5
RP11-110I1.6
RP11-478P10.1
AC004485.3
NEGR1-IT1
RP11-511B23.3
RP11-61G19.1
linc-PABPC4L-7
RP11-159M11.2
RP11-15B24.4
linc-ATP10A-2
RP11-412H9.2
AL157871.2
linc-ZMAT4-2
linc-HTR2C
RP11-378I6.1
CTD-2308B18.3
RP11-61K9.2
CTD-2199O4.1
linc-RBM26-2
RP11-403P17.3
CLYBL-AS1
RP11-158L12.5
AF124730.4
AC010132.10
RP5-893G23.1
linc-ZCCHC17-9
CTB-85P21.2
RP11-501E14.1
RP11-794M8.1
CTA-445C9.15
AC064834.1
RP11-483H20.6
linc-TCTE3-2
LINC00682
AC005484.5
linc-NR3C2-3
CTD-2540B15.6
RP11-542M13.3
RP4-719C8.1
RP11-161D15.1
AF064858.10
RP3-330M21.5

AC079586.1
TCF7L1-IT1
linc-JAG1-3
linc-STMN2-1
linc-PTGER4-1
linc-ANO5-3
KCNAB1-AS1
linc-CPPED1-3
RP11-513G11.3
Z99756.1
linc-PTBP2-2
CTD-2373N4.5
linc-ARFGEF2-8
AC005336.1
RP11-77H9.2
AC105398.3
RP1-58B11.1
RP4-651E10.4
CTA-929C8.7
CTC-782O7.1
AP000688.29
linc-TNPO1-2
RP11-124O11.1
linc-PLAC1L-1
RP13-16H11.5
CTA-398F10.2
AP002954.4
CTD-3118D11.3
linc-TMEM169-1
linc-BTBD6-2
linc-CCRN4L-5
RP11-69C17.4
RP11-524K22.1
RP11-930O11.1
AC017006.3
ELMO1-AS1
AP000474.1
RP11-498M15.1
linc-SLITRK5-5
RP11-82L7.4
RP11-176N18.2
RP11-122K13.7
RP4-753M9.1
CTD-2147F2.1
RP11-473O4.4
RP11-809H16.5
AC105053.3
CADM2-AS2
RP11-397H6.1
linc-RREB1-4

linc-WAS
RP11-567O16.1
RP4-545K15.5
CTA-126B4.7
UNC5B-AS1
RP11-171G2.1
RP11-257P3.3
RP11-118G23.2
linc-PAM-1
KB-1205A7.2
linc-PEX13
linc-RNASE1
linc-TMSL3
linc-NOP14-1
RP11-7M10.2
RP3-521E19.2
RP11-359J14.2
linc-COX4NB-7
WI2-85898F10.1
RP11-545N8.3
RP11-438N16.1
NAV2-AS4
AC073071.1
linc-GOLGA7B
RP5-1121A15.4
DLGAP1-AS4
linc-DIAPH3-1
RP4-568F9.6
RP11-406D1.2
ALDH1L1-AS1
RP11-356K23.2
linc-NOS1AP
AC093110.3
RP11-326E22.1
linc-TCF7-3
RP4-665N4.8
LYPD8
linc-NAV2
RP11-818C3.1
linc-LOC389493-5
linc-GPR65-3
linc-TYRP1-2
RP11-797A18.4
RP11-899L11.3
CTD-2089N3.2
RP11-322D14.2
RP1-232N11.2
linc-CPEB4-5
AC016738.3
linc-APLN-1

AC092155.4
linc-CCDC93-1
linc-AGBL4-1
RP11-881L2.1
linc-LHX1-2
RP11-22A3.2
linc-HACE1-2
RP11-417J1.1
RP4-604G5.1
RP11-846C15.2
CTD-2525I3.3
linc-TCP11-4
JRK
LL22NC03-23C6.13
RP5-1185H19.2
CTD-2382E5.4
RP11-944L7.4
RP5-881L22.5
linc-FAHD2B-4
EMCN-IT2
AC019048.1
CTD-2647L4.1
RP4-617C6.1
CTD-2001E22.1
AP001630.5
linc-PIK3C2G-1
linc-MYL2-3
linc-DDX26B-1
AC002539.1
RP11-353N4.4
RP11-1157N2__B.2
RP11-136B18.2
RP11-64K12.4
LRCOL1
RP11-315F22.1
linc-PET112L-5
RP11-394O4.3
CTB-43E15.1
RP11-314N13.3
RP11-315E17.1
AC006372.4
RP11-757G1.6
AC012462.3
AP000282.3
CTC-505O3.3
linc-PLXNA2-4
RP11-259F4.1
RP11-635N19.3
RP11-364P22.2
GS1-204I12.4

RP11-24B19.3
RP11-307P5.1
RP11-47J17.3
CTC-1337H24.2
RP1-90L14.1
RP11-367J7.3
linc-DCUN1D5-1
linc-CBLB-8
RP11-327L3.3
RP11-501O2.4
linc-RPL24-1
linc-MCTP2-7
RP11-438B23.2
CTB-181H17.1
RP11-95D17.1
XXcos-LUCA11.4
RP11-667M19.10
RP11-165D7.3
linc-CETN1-2
linc-SV2C-2
RP11-404I7.2
CTC-458I2.2
RP11-498D10.6
linc-STAP1-8
RP11-1H15.2
RP4-655J12.4
AC016710.1
STARD13-IT1
linc-MAP3K2-1
RP11-85B7.4
linc-C13orf33-1
RP11-768G7.3
RP11-664I21.5
linc-BTNL8-1
AL592528.1
CTD-2509G16.2
TUSC7
RP11-272K23.3
linc-CSNK1A1
RP11-651P23.5
AC023672.2
RP11-46E17.6
AC010145.4
linc-DLGAP2-3
linc-TAS2R38
linc-RGS5-2
RP11-34N19.1
RP11-344B23.2
RP11-33I11.2
RP11-354P11.2

AL109763.2
linc-CRH-4
linc-RAD23B-2
linc-SEMA3B
CTD-2050N2.1
AC012065.7
AC009236.1
RP11-489D6.2
linc-ADD3-2
AP000459.4
RP11-493E12.1
RP11-271K21.11
AC226118.1
RP11-283I3.4
RP11-30L3.2
RP11-38H17.1
RP13-735L24.1
AP000770.1
linc-APIP-1
RP11-461L13.2
linc-NT5DC1-1
RP1-91J24.3
linc-PDRG1
linc-COL15A1
RP11-334E6.12
linc-KILLIN
XXyac-YR29IB3.1
RP11-374A22.1
linc-NRSN1-1
RP11-554A11.7
RP11-177G23.2
linc-SGTB-4
linc-GYPA-2
linc-PDE3A-4
RP11-317J19.1
RP11-896J10.3
AC004528.4
RP11-557J10.4
RP11-203M5.7
linc-TTC15-5
RP11-542B15.1
AC005606.15
RP11-435B5.5
linc-MNX1-1
linc-CHFR-2
linc-NOX3-2
linc-PPP4R1-1
RP11-1060J15.7
RP11-665C16.5
RP11-226L15.5

RP11-316I3.2
linc-CDC42BPB-4
AC010136.2
linc-HYI-1
CTD-2503O16.4
CTD-3064C13.1
RP4-739H11.3
RP5-1110E20.1
RP11-373D23.3
linc-HIST3H2A-2
RP11-350F4.2
linc-COG2-2
RP11-461L18.1
linc-ISL1-2
SHANK2-AS1
RP11-54O7.3
LINC00567
RP11-524G24.2
RP11-624D11.2
linc-CLP1
CTD-3179P9.2
DCTN1-AS1
linc-FAT1-2
RP11-474D1.1
RP11-433A10.2
RP11-73M18.7
AC007563.1
linc-NDC80-1
RP11-354M1.2
RP11-152H18.3
linc-C6orf170-2
RP11-401E14.2
linc-FAM154B-1
linc-FAM120B-6
RP11-319G6.3
RP11-565A3.1
RP5-1031J8.1
AF064858.7
FAM230C
RP1-56J10.8
DSCAM-IT1
linc-GATA3-2
RP11-579D7.2
RP11-214L13.1
RP5-1092A11.2
RP3-429O6.1
AC144450.1
linc-CCDC80-1
RP11-57H14.4
AC073133.1

linc-ADAMTSL4
RP11-394B5.2
RP11-482M8.3
RP11-770J1.7
linc-C4orf40
RP11-1C1.7
AC009336.24
linc-CCDC59-1
RP5-1029K10.2
AC006296.1
CTD-2337J16.1
BZRAP1-AS1
RP11-552M11.8
CTB-3M24.3
linc-CXorf49B-2
CTD-2218G20.2
linc-FCHSD2-3
RP11-510D19.1
RP11-144G16.1
SNORD62B
AC012360.6
AC123886.2
RP11-630C16.1
linc-G2E3-3
linc-C20orf85
linc-C9orf170-4
linc-KIAA1614-2
linc-MRGPRF-4
RP11-565P22.2
NICN1-AS1
linc-TPK1-3
linc-C10orf119-4
RP11-129K12.4
RP5-1099E6.3
RP11-669N7.2
RP11-498P14.3
RP11-352D13.6
RP11-25B7.1
RP11-890B15.2
RP11-680F20.9
AP000487.6
RP11-588P7.1
RP11-521C20.3
RP11-324I22.2
linc-VKORC1L1
RP11-4C20.4
linc-STXBP6-1
AC006378.2
RP11-565N2.2
linc-ARHGAP28-6

RP11-15M15.2
RP11-244H18.3
RP11-382A20.5
RP11-787I22.3
linc-CSTB-9
TNR-IT1
RP5-935K16.1
CTD-2643I7.1
RP11-56F10.3
FZD10-AS1
RP4-593C16.3
linc-GRIP1-7
RP11-284G10.1
AF131217.1
RP11-301H24.4
linc-SLC6A6
linc-SEH1L
linc-PPP2R2D-3
RP11-630C16.2
RP11-127I20.5
CTD-2245F17.2
RP11-1049A21.2
RP11-706J10.2
RP11-332H18.3
linc-MARK1-1
RP11-325E5.1
RP5-1119D9.4
RP11-553L6.5
linc-OTUD4-3
RP5-1039K5.16
linc-WNT4
RP11-334N17.1
CTD-2561B21.7
RP11-778J15.1
RP11-856F16.2
linc-GBP5-1
RP11-782C8.3
RP1-200K18.1
RP11-691G17.1
AC009955.8
RP11-521D12.1
linc-VIPR2-2
AC015936.3
RP11-30G8.2
RP11-85L21.6
SYN2
CTC-507E2.1
AC011901.2
RP11-154C3.2
linc-SORCS3-2

linc-DAB2
RP11-433J20.2
RP11-752D24.2
RP11-458N5.1
RP11-727A23.7
RP11-140I16.3
linc-KITLG-1
RP11-152O14.1
CTD-2130F23.2
RP11-236J17.3
RP11-440I14.3
AP001628.6
MCF2L-AS1
RP11-739N10.1
RP11-166D18.1
RP4-683M8.2
linc-CDH20-1
RP11-343N15.1
LINC00505
MIRLET7DHG
linc-ADRA1D-2
RP4-777O23.2
linc-COMT
RP11-255A11.21
RP1-317E23.7
RP11-350J20.5
RP11-1085N6.5
linc-HS3ST1-1
AC064853.2
RP11-867G2.6
linc-SEP15-5
RP11-27G24.3
RP11-348F1.2
RP11-65D24.1
linc-SDSL
linc-MMP21
linc-SLC25A45-5
linc-CD9-4
RP11-328N19.1
BACH1-AS1
linc-DMRTB1-1
RP11-335O13.7
AC104777.4
CTD-2161F6.3
linc-PRR20A-3
RP1-154K9.2
RP1-86C11.7
linc-AMZ2-2
RP1-249F5.3
RP4-697K14.3

RP11-349O10.1
RP11-6N17.9
linc-ABHD3-1
AP000654.4
RP11-308B16.2
linc-LOX-3
linc-MMAA
linc-OBFC2A
linc-ABCC4-1
RP11-393M11.2
RP11-215P8.4
linc-FAM57A-1
RP11-342D11.3
RP11-624L4.2
linc-SBDS-5
linc-PAXIP1-6
linc-CSMD3
linc-FAM19A5-8
linc-CDH11-5
RP11-227F19.1
RP4-714D9.5
linc-RCC2-1
AC005082.12
linc-ACTL7A-7
linc-PHF14-1
RP11-231N3.1
CTD-2545M3.8
RP4-601P9.1
RP11-112J1.2
linc-SGTB-2
RP11-186N15.3
RP11-785F11.1
linc-NLRP3-3
RP11-10J5.1
RP11-522B15.5
AC012309.5
RP11-6J21.2
linc-ARID1B-4
CTC-575I10.1
RP11-150O12.3
linc-EBF3-2
linc-C13orf23-2
AC002064.4
RP11-966I7.1
AC012358.7
RP1-167G20.2
RP11-245J9.5
linc-FOXF1-4
linc-PLD5-5
RP11-462G12.1

linc-GSDMD-2
linc-ATP1B3
RP11-263C24.1
SRGAP3-AS2
LINC00948
linc-MALT1-2
linc-GRB14
RP11-501G6.1
linc-DAAM1-2
linc-RALGPS2-1
linc-ZNRF2
RP11-16L9.4
linc-MAGI3
linc-DARS
linc-IL28RA-1
AC006960.7
linc-C20orf70
RP11-619L12.3
RP1-32B1.4
CTD-2319I12.5
linc-LOC100132288-2
RP3-365O12.2
linc-TAOK3-2
RP11-348N5.7
RP11-834C11.11
RP11-129B22.2
RP11-510D21.1
RP1-41P2.7
RP11-670E13.5
linc-COBL-3
linc-DCC-2
AC004447.2
RP3-507I15.2
RP11-445L13__B.3
RP13-317D12.3
RP11-466A19.5
linc-FAM103A1
RP11-436H11.4
RP11-849F2.4
RP11-431M7.2
linc-ALG2-2
AC008174.3
RP11-466L17.1
linc-SALL3-2
RP11-639B1.1
RP11-15G16.1
RP11-102J14.1
linc-TRIP10-6
RP1-158P9.1
RP11-247A12.7

RP5-1085F17.4
linc-SH3BP4-3
SIK3-IT1
linc-PDIK1L-2
RP13-392I16.1
RP11-259P20.1
RP11-472K17.1
linc-PLEKHA5-2
PCAT2
CTD-3137H5.1
RP11-138B4.1
RP11-903H12.3
RP11-148B18.3
AC007620.3
RP11-311F12.2
RP11-442N1.2
linc-INF2
linc-RFX8
AC019055.1
linc-SMC2-1
RP11-227G15.8
RP1-30M3.6
LINC00415
linc-CCKAR-8
RP11-573G6.8
RP11-383M4.6
MRPL23-AS1
RP11-440G9.1
RP11-298J23.8
linc-FAT3-4
LA16c-380H5.2
linc-OAF-3
RP11-304F15.7
linc-SSPN-1
RP11-295D4.1
linc-MRPL32-2
linc-SMAD7
RP11-560A15.4
linc-LRP8-3
RP11-145P16.3
RSF1-IT2
RP11-508N22.8
RP3-368A4.6
AC007036.5
RP11-139I14.2
RP5-977B1.12
linc-PPAPDC3
RP5-1086L22.1
AC019118.3
RP11-53O19.3

RP4-530I15.9
RP11-478K15.6
CTD-2514K5.4
RP11-439C15.5
LINC00684
RP11-545H22.1
RP5-905H7.8
RP11-373E16.3
RP11-5P4.1
linc-LEPROTL1-2
RP11-3B12.2
RP5-1096J16.1
EGFR-AS1
linc-AMD1-1
LINC00589
AC005757.7
FRMPD3-AS1
RP11-366H4.1
CTD-2536I1.1
RP11-421E14.2
CELF2-AS2
AP001476.3
RP11-508M1.7
linc-EML6-2
RP11-175B12.2
RP11-402G3.5
RP11-1041F24.1
RP11-862G15.2
linc-C4orf27-2
RP11-364B6.1
RP13-238F13.5
CTC-281M20.4
RP3-414A15.10
linc-ALDH1L1-2
CTD-2196E14.5
CTD-2235C13.3
RP11-308N19.4
AC007283.5
linc-STEAP1-3
RP11-438C19.2
RP11-84C10.3
AC104655.2
AC011753.5
RP11-115D19.1
AC002480.5
RP11-569G13.2
RP11-157E16.1
RP11-152H18.4
RP11-89M22.3
RP11-956J14.1

LINC00427
linc-SORCS1-2
linc-C18orf62-2
AKT3-IT1
RP11-215E13.1
RP11-298O21.6
AC006262.10
linc-TWIST2-2
AC068535.3
linc-KCNIP1
LINC00304
AMMECR1-IT1
LINC00211
RP11-334E15.2
CTB-75G16.3
LINC00365
AC018495.3
linc-LIPT2
RP11-342M3.5
RP11-363J20.1
RP11-397O8.7
RP11-871F6.3
LINC00087
RP11-320N21.2
AC017060.1
linc-TLE3-1
AP003025.2
linc-GP9
RP11-315L6.1
SNAP47-AS1
RP11-184A2.3
linc-TCP11L2-2
RP5-1126H10.2
TSPAN9-IT1
RP11-171I2.2
CTC-360P9.4
RP11-178F10.3
RP11-734K21.4
linc-ANXA6
RP11-278H7.4
AC083867.4
LINC00485
AC079799.2
CTD-2021H9.2
RP11-290D2.3
CTD-2014E2.2
RP3-416J7.5
RP3-434O14.8
RP11-297J22.1
RP11-96B5.3

RP11-6N13.4
LINC00676
CTD-3199J23.4
RP11-2E17.2
RP11-312J18.6
RP11-1C8.5
RP11-157D23.1
LINC00970
linc-FMR1
AC021016.8
RP11-128P17.2
AC006277.2
linc-PMEPA1-1
RP11-83M16.6
RP11-62H20.1
RP4-655C5.4
linc-BICC1
AC007970.1
LINC00452
linc-PHYHIPL
linc-UBR2-1
linc-ANKRD10-3
linc-BTG1-2
linc-GNAQ-4
RP11-686D22.5
RP11-736K20.5
AC108463.1
RP11-305P22.9
AC092619.1
linc-FXR1
AC073325.1
FTCD-AS1
RP11-77K12.4
linc-CD180-12
AC087430.1
RP11-248N22.1
linc-BAI3-3
RP11-434D12.1
linc-TMEM30B
A2ML1-AS2
linc-ZNF366-6
RP11-367F23.2
RP11-850P15.1
AC067945.2
RP11-402L5.1
RP13-820C6.2
linc-TMED7
LINC00598
RP11-95H3.1
linc-HIST1H2AG-5

linc-OPRK1-3
RP11-704M14.1
RP11-113O24.3
RP11-166B2.5
RP11-420A23.1
linc-NCKAP1-1
RP5-1112D6.4
linc-KCNB2-4
RP11-96L7.2
RP11-149F8.5
XIRP2-AS1
CTB-89H12.4
RP11-370A5.1
RP11-482E14.1
linc-DYNC2H1-4
RP11-29B9.2
RP11-384C4.6
RP11-849N15.4
RP11-35J1.1
LL22NC03-N64E9.1
linc-IL1A
RP11-956E11.1
RP11-845M18.6
RP11-553P9.2
RP11-148E17.1
RP11-5N11.7
linc-ULBP2-1
CTD-2527I21.4
linc-SDK2-1
RP11-142G1.2
linc-ZNF273-1
CTD-2589M5.4
linc-ABCA5-6
AC018685.1
AC078942.1
RP11-471B22.2
RP11-7C6.1
RP11-268I9.3
RP11-366K1.1
RP11-21I10.2
linc-OR8D4-1
RP5-936J12.1
AC009133.21
AC133633.2
RP11-419J16.1
linc-CHRD
linc-ANKRD30A-3
linc-CMC1-2
RP11-41O4.2
linc-GABRA5-1

AC005324.6
MAGI1-AS1
RP11-235E17.6
linc-C13orf34-6
linc-SAMD11-4
RP4-752I6.1
RP11-10J21.5
RP11-561O23.7
RP11-49K24.4
LINC00640
RP11-248E9.5
RP11-386M24.6
RP1-37N7.4
RP11-556H2.3
RP11-845C23.3
CTD-2626G11.2
linc-C14orf180-2
linc-TTC35-1
RP11-294O2.2
AC002044.4
linc-ANKRD10-5
linc-SOX1-1
linc-P2RY2-1
RP13-870H17.3
CTC-543D15.3
CTD-2009A10.1
RP11-383D22.1
MYLK-AS2
CXADRP3
linc-NKX2-5-1
RP11-488C13.7
RP11-626H12.2
RP5-944M2.2
linc-ANKRD20A1-2
RP11-60L3.2
CTC-482H14.5
RP11-428J1.4
AC009495.4
linc-C6orf203-1
RP11-486M23.2
linc-RLN2
linc-CHD9-7
RP11-410E4.1
RP11-465K1.2
linc-STAC
linc-ELTD1-11
RP5-1125M8.2
GS1-18A18.2
NTM-IT
CTD-3051D23.4

linc-SCUBE3
linc-IGSF1-3
RP3-393E18.2
RP11-1136J12.1
CHRM3-AS1
linc-ROPN1B-3
RP11-567C2.1
RP11-3P22.2
linc-PPAPDC1A
linc-C5orf32-2
RP11-589M4.1
RP11-61J19.3
RP11-525K10.2
RP11-411H5.1
RP11-863P13.1
linc-SULF2-7
FGF12-AS1
LINC-ROR
RP11-720D4.3
RP11-484P15.2
RP11-474B12.1
RP11-1136G4.1
CTB-158E9.1
RP11-549J18.1
linc-DUSP22-1
RP11-115A15.1
RP11-699A5.2
RP11-1080G15.2
linc-ALX4-1
RP13-395E19.3
linc-C2CD4B-5
RP11-534L20.5
RP1-37N7.1
linc-C1orf124-2
RP11-292F22.5
linc-ACP1
RP11-749H17.1
AL121656.5
RP11-661C3.2
SCEL-AS1
linc-TUSC3
RP11-529E15.1
LINC00184
RP11-473M10.3
RP11-94H6.1
RP11-156K13.3
linc-LRPPRC-1
RP11-894P9.2
RP11-148M9.1
RP11-62C7.2

linc-PKNOX1
linc-CTU1-1
AC000032.2
linc-BAI1-2
linc-BEX4
RP11-93O14.2
RP11-4O1.2
linc-TMEM232
RP11-356N1.2
LINC00607
linc-SEC24B-3
RP11-543D5.4
ARHGEF3-AS1
RP11-637O19.2
FAM157B
RP11-666I19.2
RP11-361D14.2
RP4-663N10.1
linc-G2E3-1
RP6-191P20.4
PCDH9-AS1
RP11-475A13.2
linc-ITIH2-5
linc-RRP1B-6
AC009120.5
CTD-2140G10.1
RP1-97G4.1
RP11-98L5.2
linc-FAM84B-8
linc-C1orf174-3
RP11-510M2.1
linc-SNX10
RP11-78A19.2
RP11-498C9.15
AJ239322.3
AY269186.1
CTC-550B14.7
RP11-128M1.1
RP11-713N11.4
RP11-160E2.17
RP11-563N12.2
RP11-575L7.2
linc-ZBTB16
CTB-31N19.3
RP11-127O4.2
LA16c-306A4.2
linc-C1QTNF9B
LINC00545
RP11-545P7.4
RP11-1070A24.2

linc-TLR3-1
AC008834.1
linc-DOCK10-3
CTD-2583P5.1
RP11-235G24.1
RP5-1042K10.10
CTC-448F2.4
linc-APOB-1
SH3BP5-AS1
AC100802.3
RP11-534L6.5
IQCJ-SCHIP1-AS1
RP11-59E19.1
linc-PAX8-3
COL5A1-AS1
linc-KIAA0649-4
linc-SLC6A19-2
RP3-448I9.1
RP11-458K10.1
ZFAT-AS1
RP11-499F3.1
linc-ZZEF1
KCND3-IT1
RP5-997D16.2
RP11-1096D5.1
linc-CCDC146-3
RP11-838N2.5
linc-RNF2-2
RP11-184D12.1
linc-LCLAT1-1
linc-SLC39A10-10
RP11-344E13.4
RP11-453O5.1
RP11-78H24.1
RP1-149A16.12
RP11-207C16.4
AC002386.1
AF131215.9
linc-KIAA2018
RP11-361K17.2
linc-CDH22
CTD-2544H17.1
CTC-297N7.9
RP11-153N17.1
RP11-1191J2.2
linc-OR7C2-1
AC092667.2
RP11-552O4.1
RP11-67H24.2
linc-MRPS33-3

linc-LMOD2
RP11-58A18.1
RP3-512E2.2
RP11-103H7.2
CTD-3193O13.14
linc-MPPE1-1
RP11-231L11.3
RP11-159K7.2
RP11-2H8.2
RP11-430H10.2
RP11-157N3.1
LINC00587
linc-C11orf90-2
RP3-462C17.1
AC108025.2
AP000439.3
RP1-35C21.1
linc-FAM186A-2
SLC9A9-AS1
STPG2-AS1
linc-DTNB
RP1-90G24.6
linc-IFT88-1
linc-CIDEA-1
RP11-50D9.3
AC087499.10
linc-LSG1-2
RP11-574K11.8
linc-DIRAS2-2
RP11-218E20.3
RP11-230G5.2
CTD-3224I3.3
linc-SUCLG1
RP11-867O8.5
RP11-147N17.1
RP11-223P11.2
RP1-225E12.3
RP11-766N7.4
linc-ALS2-2
ZDHHC20-IT1
RP11-382A18.2
RP11-98O2.1
RP11-465L8.1
RP11-25L3.3
CTD-2201E9.1
AC118653.2
AC005532.5
RP11-35O7.1
RP11-731D1.4
RP11-439C8.1

linc-FAM84B-3
RP11-34A14.3
NAV2-AS1
RP11-456O19.5
RP5-921G16.2
AC093627.7
RP11-91J3.3
linc-DENND3-1
RP11-267D19.1
RP11-789C2.1
RP11-716D16.1
RP1-90G24.8
RP11-380D23.1
AP001469.5
linc-ANXA8L2-2
linc-OSBPL1A-1
linc-KLF3-5
RP11-234B24.2
LINC00554
RP1-121G13.2
CTD-2561J22.5
linc-HDDC2-4
linc-C7orf28B
RP11-60I3.4
RP11-519G16.5
LINC00907
linc-INSIG1-2
linc-ZCCHC17-8
linc-TNFAIP3-2
AC012594.1
RP11-160N1.9
AP001189.4
RP1-74B13.2
RP11-219O3.2
linc-RPL7
SNORA71B
linc-NUPL1-2
HTR2A-AS1
RP13-259N13.2
linc-NPBWR2
RP11-465L10.7
RP11-189E14.5
AC011288.2
RP11-222N13.1
CTC-498M16.2
linc-PRKAA2-7
linc-MAP3K7-2
CTC-490G23.2
RP11-654A16.1
AF127577.11

RP11-586K12.8
linc-MAP3K5
TBC1D3P1-DHX40P1
linc-ZMIZ1-1
AC106706.1
linc-ITGA11
LINC00616
RP11-362I1.1
linc-LYRM4
RP11-1078H9.6
AF186192.6
linc-BRD9-2
RP11-566K11.7
RP11-290M5.2
MAGI2-AS2
AC079896.1
RP11-506M13.3
linc-LRP12-1
RP11-761N21.1
FOXP1-IT1
RP11-391M1.4
RP11-194N12.2
linc-ARAP3
AC010975.1
linc-FGD3-1
RP11-527H14.6
RP11-977P2.1
RP11-642M2.1
RP11-397A16.3
RP11-134D3.1
RP11-421P23.2
linc-RAP1B-4
RP11-252P19.1
RP11-443C10.1
LINC00347
RP11-181E10.3
GPC5-IT1
RP11-43N5.1
RP3-495K2.3
AP001601.2
linc-POC1A-1
AC003973.4
linc-E2F2-2
RP11-524K14.1
RP11-219E7.3
AC009518.5
linc-ZC3H12B-1
RP11-357N13.2
CTB-186G2.1
RP11-44M6.1

AC006373.1
VENTXP1
RP11-242F4.2
linc-FAM75A1-2
RP11-195E2.4
AC064865.1
linc-PFN2-1
linc-ZNF673-2
AF131215.4
linc-CPT2-1
LL0XNC01-250H12.2
RP5-837I24.4
AP000695.4
RP5-916L7.2
RP11-496I9.1
RP11-4F22.2
HHATL-AS1
LINC00498
linc-PRKCE-1
RP11-164P12.3
CTD-2544N14.3
AC024132.1
RP1-191L6.2
AC007292.3
RP11-482D24.2
RP11-543G18.1
RP11-44K6.3
linc-FOXG1-6
AC009134.1
RP11-203F10.5
linc-NUDT6-4
linc-GOT2-5
RP11-140A10.3
linc-PARP11-5
linc-ADAMTS9-4
RP11-579O24.3
CTB-50L17.5
RP11-955H22.1
linc-CTCFL-1
linc-CCDC140-4
AC068492.1
FREM2-AS1
AC005863.1
RP13-137A17.6
RP11-544M22.1
linc-MLPH-5
linc-RASL12
RP11-23D5.1
linc-ACBD5-3
RP11-349K16.1

RP11-264A11.1
RP11-722P11.4
RP11-529H20.5
AL035610.1
RP11-276H7.3
RP11-595B24.1
linc-NKAIN2-2
RP11-669M2.1
linc-GNAQ-7
RP11-752G15.8
CTA-250D10.19
linc-MKI67IP-6
RP3-325F22.3
RP6-24A23.3
CTD-3096M3.1
RP11-562F9.2
linc-SIM2
RP11-431J17.1
RP11-310H4.3
RP11-521I2.3
linc-C22orf9
RP11-442J17.3
AC012368.1
RP11-1B20.1
RP11-64D22.5
linc-CHST11-1
RP11-323I15.5
RP11-961A15.1
AP000344.3
RP11-302F12.10
RP11-455O6.2
linc-TACC2-4
RP5-888M10.2
HECW1-IT1
XXYLT1-AS1
CTD-2643K12.2
PAPPA-AS2
RP4-564O4.1
RP11-807G9.2
linc-COX7A2L-1
AC010525.6
AP000431.2
AC006378.3
linc-ZFP42-10
RP13-30A9.2
RP11-46A10.5
linc-GLRB-4
AP001257.1
linc-TSPAN5-2
LINC00560

linc-KY-1
RP11-76I14.1
RP11-556E13.1
RP11-497D6.5
AP000260.4
linc-TG
linc-REG3G-1
RP11-567M16.1
linc-ARHGAP11B-2
RP11-470P21.2
RP4-797C5.2
RP1-274L7.1
AC007631.1
MDC1-AS1
CTC-564N23.2
CTD-2332E11.2
RP5-1104E15.6
RP11-1103G16.1
linc-C10orf93-1
linc-SDHC
CTA-109P11.4
linc-NBEA-1
CTD-2170G1.2
RP4-745K6.1
linc-SMEK2
linc-SPNS3
linc-C10orf11-1
LINC00839
RP11-420O16.1
AC026150.5
CTC-428G20.3
RP11-252E2.2
RP11-885B4.2
linc-EIF2AK3-7
AC098872.3
RP11-93O7.5
LA16c-325D7.1
CTD-2555A7.1
RP11-430H10.4
linc-TRUB2-2
AC018878.3
RP11-680F20.11
RP11-128B16.3
linc-F13A1-1
linc-ZNF236-5
RP11-580I1.3
linc-MINPP1
RP11-654A16.3
LINC00308
RP11-132N15.1

RP11-714L20.1
RP11-762H8.1
XXyac-YX60D10.1
CTC-325J23.3
RP4-541C22.5
RP11-1113L8.1
linc-ORC6-7
linc-CREB3L1-1
CTC-501O10.1
linc-ZNF322A-4
RP11-611O2.1
linc-SEMA6A-3
CTC-471J1.2
RP11-5N11.5
linc-IGSF3
AC005276.1
NAALADL2-AS1
RP11-686D22.8
RP11-356C4.3
RP11-91N2.3
linc-OSBPL2
AC010894.5
AC004878.7
ST7-AS2
linc-GPC6
linc-CLLU1-2
linc-PMFBP1-1
RP11-480G7.1
linc-TMEM56-1
AC011747.6
FAM95B1
RP11-410N8.1
RP11-31I22.4
linc-ZNF540
linc-MAP3K9-2
RP11-876N24.2
linc-SORBS3
RP11-66D17.3
RP11-362F19.1
linc-HIATL1-3
AC004603.4
RP11-784B15.1
RP4-614O4.12
AC092301.3
linc-MBOAT1-4
RP11-536C10.7
AGAP11
linc-GPR125-1
RP11-286M16.1
linc-TCERG1L-4

RP11-114H23.2
linc-STX18-3
CTA-714B7.5
AC091770.3
linc-GPRIN3-2
RP5-951N9.2
RP13-638C3.3
AC004702.2
CTD-2231E14.8
RP11-16L21.7
CDH23-AS1
RP11-494H4.3
RP11-274B21.9
AC108868.6
RP11-698N11.4
RP11-148O21.3
AL050303.7
linc-ANKRD33B-1
RP11-739G5.1
SCARNA10
linc-YIPF5-4
linc-C14orf101-2
AC093822.1
linc-NDUFV3-3
linc-NLRP3-1
RP4-799P18.3
SLC39A12-AS1
CTC-366B18.2
LINC00160
AD000091.2
CTD-2046I8.1
RP11-295K2.3
KB-1000E4.2
RP1-90J20.12
RP1-55C23.7
linc-H1FNT
AC006293.3
linc-ZNF337-10
linc-BMP2-2
RP11-611E13.3
linc-HIVEP1-3
AC092535.3
linc-DMGDH-1
linc-RHOBTB2-1
RP11-69H7.2
RP11-907D1.2
CTC-506B8.1
CTC-454I21.4
linc-C8orf86-1
RP11-145M4.3

linc-GNG8
linc-CNTNAP5-7
RP11-262A12.1
RP11-439C15.2
RP11-6F6.1
RP1-59D14.5
linc-WNK1
linc-PDE10A-2
linc-ATP4B-2
LINC00543
KB-1083B1.1
AC091705.1
linc-ASCL1-1
RP11-847H18.3
linc-ZBTB40
CTD-2562J17.7
linc-SOX17-1
KB-1592A4.14
linc-EPHA7-5
linc-TBC1D8-1
RP11-389K14.3
MAGI2-IT1
RP11-700E23.2
linc-SHOX-4
RP11-469N6.3
RP11-680F20.12
linc-PRSS3-6
RP11-315H15.3
CTD-2587H24.5
linc-GPR45-2
RP11-1082L8.2
AC097461.4
CEACAM20
VCAN-AS1
RP5-1063M23.2
RP5-850O15.4
CTC-575N7.1
RP11-1293J14.1
linc-HUS1B-7
RP11-649A16.1
linc-CERK-2
RP11-535A19.1
linc-UBE2E1
KRTAP7-1
RP13-653N12.2
RP11-17A4.3
AC087491.2
linc-AJAP1-5
RP11-399E6.4
RP11-756P10.5

RP11-732A19.5
AC009478.1
RP3-454G6.2
linc-RHOU
linc-EXOSC9-5
linc-CDH1
LINC00596
RP11-459C13.1
AC104024.2
RP11-292D4.3
linc-IRAK4-2
RP11-341G5.1
linc-BTG1-1
linc-ODZ2-2
linc-PML
RP11-477H21.2
LINC00925
PLCL2-AS1
linc-PARM1-2
linc-AKR1E2-6
CTC-510F12.6
CTD-2201G16.1
RP11-492A10.1
RP11-474I16.1
RP1-257A7.5
RP11-586D19.1
MIR1-2
CTD-2131I18.1
AC091729.8
linc-NETO1-1
AC005301.8
ATP11A-AS1
CTC-497E21.3
CERS6-AS1
RP3-453P22.2
CTD-2258A20.5
RP11-333E1.2
RP11-981G7.3
RP11-245C17.2
linc-POTED-3
FAM83A-AS1
linc-POLR3A-4
RP11-484D2.5
linc-FLI1-1
AC008592.7
LINC00708
RP11-628E19.3
CTD-2555O16.2
RP11-211G23.2
linc-GBP5-6

RP11-439I14.2
RP11-813F20.4
RP11-525J21.1
AC133680.1
RP11-227G15.2
linc-RBM11-1
CTC-430J12.2
RP11-167P20.1
linc-C9orf170-2
RP11-753H16.3
RP11-53I6.4
RP11-268P4.5
RP5-1198O20.4
linc-MUC20-10
linc-GSDMD-1
linc-TMEM196-1
linc-PCDH9-2
RP1-45I4.2
LINC00701
ABO
linc-SUZ12-4
RP11-118K6.2
RP11-61O1.2
AC011525.4
MFI2-AS1
RP11-90P13.1
GLYCTK-AS1
RP3-522J7.6
SNAP25-AS1
linc-NDN-4
CTD-3096P4.1
linc-NUP35
RP13-143G15.3
NREP-AS1
RP11-395F4.1
DPYD-AS1
linc-KCNIP4-2
linc-CDH6-8
RP11-20O12.1
PWRN2
AC011718.2
RP1-90K10.4
RP11-114J13.1
AP001610.5
RP11-340F14.5
linc-ZBED5-3
RP11-121L11.3
RP11-818O24.3
AC026806.2
RP4-635E18.7

RP6-91H8.2
CTD-2272D18.1
RP1-69D17.3
AC093326.1
RP11-65J3.2
RP11-1078H9.2
linc-ZNF492-1
linc-PRDM2
RP11-124N19.3
RP11-793A3.1
linc-USP28-2
MACROD2-IT1
RP11-1223D19.1
CTB-49A3.2
linc-HAS2-3
RP11-2N5.1
RP11-366F6.2
AC004692.4
CTC-523E23.15
RP11-509J21.4
RP11-669E14.6
linc-PDE6H-2
linc-C5orf38-2
linc-AMY1A-3
linc-FBN1
RP11-1042B17.3
AC008746.3
RP11-770G2.5
linc-MTMR9
RP1-17K7.1
B4GALT4-AS1
linc-SPTBN1-3
RP11-340I6.3
linc-JARID2-2
linc-SERHL2-3
RP11-330M19.1
RP11-329J18.3
linc-LRIG1-2
RP11-115H15.2
RP5-1050D4.2
RP11-548M13.1
AC074366.3
linc-CELF4-3
RP11-94C24.8
linc-TP53I11-1
RP11-231C18.1
RP11-408H20.1
SMCR5
linc-TCTE3-4
RP11-671C19.1

RP13-455A7.1
RP1-228P16.3
CTD-2234B20.1
linc-GALNT5-2
CTD-2587M2.1
NPSR1-AS1
MME-AS1
linc-SLC7A11-1
AC093590.1
RP11-107N7.1
CTC-340I23.2
RP11-173M1.8
linc-SUMF1-9
AC006116.12
CTC-467M3.2
RP11-174G6.1
linc-RGS5-1
AF096876.1
PITRM1-AS1
NBPF5P
RP11-192H6.2
LINC00917
linc-COLEC10-1
AC144831.1
linc-XKR4
linc-MUDENG
CTC-339D2.1
RP11-110H1.9
AC132807.1
AC109589.1
linc-ZFC3H1-2
linc-MSI2
linc-EFEMP1-4
RP11-433J8.1
linc-TMPRSS15-9
AC106869.2
RP11-356O9.1
RP3-460G2.2
RP11-279F6.3
RP11-415C15.3
linc-RUNX2-3
linc-ZPBP-1
RP11-558A11.1
linc-SPAST-2
RP11-443O13.3
RP11-135A1.2
RP11-84E24.2
AC000036.4
RP11-300A12.2
LINC00686

RP4-550H1.4
RP11-368D24__A.1
AC012075.2
CTD-2521M24.11
linc-DAO-1
linc-C15orf2-6
CTC-308K20.2
CTD-2280E9.1
linc-WWC3
linc-PTCHD2-2
RP11-179B15.5
RP11-381K20.5
AC091199.1
linc-C2orf86-3
CTA-276F8.2
RP4-742J24.2
RP11-103J17.1
linc-C1orf227-2
RP11-169K17.4
linc-CSGALNACT1-2
RP5-919F19.5
RP11-657O9.1
RP11-1376P16.2
RP11-736P16.1
linc-SNRPN
linc-WDHD1-2
RP1-117O3.2
ARHGAP22-IT1
GRM7-AS3
RP11-676J12.8
CTB-187M2.3
RP1-86D1.4
linc-GXYLT2-3
RP11-478K7.2
AC140542.2
RP11-159N11.3
RP11-589B3.6
linc-KCTD4
linc-LYSMD3-1
RP11-554A11.9
RP5-905N1.2
RP11-15I11.2
linc-TMEM90B-2
linc-SEC24D
CTC-347C20.1
RP4-630C24.3
RP11-675M1.2
CTD-2619J13.9
linc-SETD7-1
AC012462.2

MIR663A
RP11-793H13.3
linc-C9orf102-3
linc-INPP4B-2
linc-CLLU1-11
linc-AGTR1-6
RP11-118E18.4
AC004791.2
RP11-334J6.6
CTD-2199O4.3
RP11-427M20.1
CTD-3023L14.2
RP11-278A23.2
linc-DDX51
RP11-31K23.2
RP6-1O2.1
AC005162.5
AC096558.1
AC011196.3
AC018643.4
linc-DCLK1
linc-IL21R
RP11-16C18.3
linc-TNFRSF19-1
AC016907.3
linc-AP3S1-3
linc-DTNBP1-2
RP11-402G3.3
linc-OLA1
RP11-257I14.1
linc-ALCAM-2
linc-CMPK2-11
RP11-357K6.1
AC007557.3
AC008703.1
RP11-441F2.5
linc-ALDH1A1-7
linc-CTBP1-3
linc-ADARB2-6
RP11-314D7.4
CTD-2008P7.8
CTD-2530H12.8
linc-PM20D2
RP11-463O12.3
linc-RILPL1-2
RP11-347D21.4
RP11-158G18.1
RP4-583P15.11
RP11-115J23.1
RP11-536I6.1

linc-KIF18A
RP11-339B21.9
linc-MBNL2
linc-PPP4R1-3
RP11-209E8.1
RP11-88I18.3
CTC-558O2.1
AC019064.1
RP11-429A20.3
RP11-401F2.4
RP11-491F9.5
RP11-612B6.2
RP11-115N4.1
linc-COL4A2-2
linc-ESR2
RP11-628O18.1
RP11-385F5.4
RP11-560I19.1
MIR2117
MYCNOS
PRC1-AS1
AC004837.5
CTC-450M9.1
linc-MAN2B2-2
LINC00092
RP11-517A5.4
AC021224.1
linc-RGMA-7
CTC-548K16.6
RP11-343D24.2
AC105393.2
RP11-498M5.2
linc-RNF34
CTB-31O20.6
RP11-517C16.2
linc-MANEA-3
AC006115.3
CTA-481E9.4
AC012363.4
AC016644.1
RP11-849H4.4
RP6-159A1.3
AC016751.3
AC139099.4
linc-MPHOSPH8-4
CTA-134P22.2
RP11-400N13.1
RP11-383M4.2
AP002856.7
RP11-362K14.7

RP11-363L24.3
linc-PLCZ1-3
RP11-991C1.2
RP11-426A6.5
CTC-537E7.2
CTC-391G2.1
BTBD9-AS1
linc-TLK1
linc-DACT2-4
RP11-555J4.3
linc-SCEL-1
RP11-160A10.2
RP11-46I1.1
RP13-578N3.3
RP11-317P15.5
RP11-439E19.7
RP11-203B7.1
AC000111.3
CTD-3131K8.3
AC015977.6
AC025165.8
linc-LY75
OCIAD1-AS1
RP11-1112C15.2
RP11-61G23.2
RP11-310I9.1
linc-PLEKHH2-5
RP11-744K17.1
RP11-95I16.2
linc-NETO1-4
linc-CLN5-1
LINC00502
RP11-646I6.6
MLIP-IT1
HOXC-AS5
RP1-102K2.6
AL450992.2
RP11-6N17.10
linc-IGFBP3-3
linc-TMEM132E-3
linc-SALL1-3
RP11-55K22.5
linc-RREB1-6
FAM222A-AS1
RP11-840I19.5
AC091814.3
RP5-994D16.3
RP4-784A16.4
linc-SPATA3
linc-C13orf34-11

AC003084.2
AC005253.2
CTB-96E2.7
RP11-497G19.2
linc-KLF12-2
LINC00029
XXyac-YX155B6.6
linc-IL1R2-1
RP11-276H1.2
RP11-373J16.1
RP11-332J15.1
CTD-2561B21.5
RP11-138I17.1
AC010096.2
RP11-463J10.4
linc-ITIH2-3
linc-WTAP-2
AC007126.1
RP11-340I6.8
RP11-553K8.5
SNORD57
RP11-181B18.1
RP11-158J3.2
RP11-697H9.3
linc-YIPF1
RP11-6L6.7
RP11-574K11.29
ARHGEF7-IT1
linc-C1orf57-1
linc-UBE3C-4
RP11-568J23.4
RP11-503C24.2
linc-TOR1AIP2-1
linc-ADD3-7
linc-ENPP4-1
RP4-712E4.1
RP11-269F20.1
linc-C18orf55-8
linc-DNAH14
RP11-402N8.1
RP11-449D8.1
CTA-929C8.8
RP11-822E23.2
CTC-228N24.2
linc-PIF1
RP11-269C4.2
RP11-14I17.3
linc-PPDPF-4
linc-WRB-5
RP11-653B10.1

RP11-231G3.1
RP11-478B9.1
linc-DSG1-8
RP11-526P6.1
LINC00667
linc-CERK-7
CTD-2324F15.2
linc-DHX37-10
RP11-69E9.1
RP11-93I21.3
RP11-483K5.3
AC091736.10
RP11-973H7.5
RP11-357F12.1
linc-C10orf57-3
linc-NR2F2-2
linc-SVIL-2
RP11-69L16.4
CTB-27N1.1
linc-SYNPO2-2
RP11-166N17.3
AC005562.1
RP11-722E23.2
CTD-3162L10.1
RP11-923I11.4
linc-PEX26-1
RP11-746E8.1
RP11-298J20.3
RP11-488P3.1
TTC3-AS1
linc-NAA35-2
RP11-511B23.1
CTD-2034I21.1
AC005550.4
XIAP-AS1
linc-ASB7-3
linc-BPIL2
RP11-111E14.1
CTC-338M12.6
RP11-24O6.2
RP11-16E18.3
linc-CST11
AC073834.3
GS1-122H1.1
RP11-945A11.1
linc-SCCPDH
RP11-178G16.5
LATS2-AS1
RP11-274B21.10
linc-KCNB2-6

RP11-161D15.3
linc-SLITRK6-10
RP5-866L20.1
linc-TAGAP-6
RP1-37J18.2
RP11-479O16.1
linc-AKAP3-1
linc-DMRT2
linc-CBLB-1
RP11-791G15.2
RP11-419L4.1
RP11-807H7.2
linc-MKI67-5
linc-C16orf78-6
AC007403.2
AC114814.3
RP11-84C13.2
linc-ARFGEF2-6
RP11-1018N14.1
linc-LYZL1-3
RP11-56I23.1
RP11-182L7.1
LINC00488
linc-TNK2-1
LINC00311
AC005522.7
CTD-3074O7.12
RP11-95O2.1
RP11-515O17.3
linc-KIAA1737-3
RP1-80N2.3
RP11-138H8.2
AC005954.3
linc-BTRC-2
linc-TNFAIP2
RP11-723D22.2
linc-ADRA2C-1
linc-CYP24A1-4
CTB-33O18.1
RP11-457P14.5
RP11-266K4.9
linc-DBX2-1
CTD-2644I21.1
AC013275.2
linc-KLHL13-1
RP11-349P19.1
NADK2-AS1
linc-PHACTR2-1
AC008067.2
linc-DIRAS2-1

RP11-973F15.2
linc-GRAMD3-4
RP11-64B16.5
RP11-435M3.2
linc-CCDC93-3
linc-CCND1-4
RP11-437F6.1
RP11-23I7.1
AC011513.4
MIR371B
linc-ZNF814
AC018866.1
RP5-1142A6.8
AC096669.2
RP11-286B14.1
RP11-478C1.7
linc-KCNA5
RP3-425P12.1
linc-TCN2-2
RP11-611L7.1
linc-PIK3R1-1
linc-KCNE2-1
linc-CD5L-2
RP11-95O2.5
linc-GADD45G-1
AC011738.4
CTB-181F24.1
linc-MTHFSD-4
RP11-15F12.3
RP3-453D15.1
AC073316.2
AP001595.1
CTD-2316B1.2
RP11-264E18.1
RP11-183M13.1
RP5-998H6.2
RP13-57D9.3
RP11-723O4.9
linc-PZP-4
linc-RTL1-8
linc-GGTLC1-3
LINC00266-1
CTD-3194G12.2
CTC-542B22.1
RP11-385D13.3
RP11-184M15.1
linc-NSDHL
linc-LOC389493-3
LINC00237
RP11-100M12.1

CECR3
CTD-2325A15.5
RP11-24D15.1
RP11-385N23.1
AC018717.1
AC006296.2
MYCBP2-AS2
RP11-290K4.2
RP5-1011O1.2
linc-ZDHHC11
RP11-63N3.1
RP11-90P5.5
CTD-2611O12.7
C6orf3
RP11-162N7.1
linc-UCHL3-3
RP11-264E20.2
linc-TMEM206-3
linc-CPEB2-15
linc-C8orf4-2
RP11-536C10.16
linc-MIB2
RP11-109M19.1
RP11-94B19.7
RP4-639J15.1
linc-FAM84A-5
CTD-3098H1.2
linc-LOC100500938-1
AP000459.7
RP11-265O12.1
linc-RUNX1
RP11-607P23.1
RP11-445P19.1
RP11-53A1.2
linc-WFDC8
linc-C2CD4A-6
TSGA10IP
SORCS3-AS1
RP11-1250I15.3
linc-ZNF727-5
RP11-202I11.2
AC007879.4
RP11-50C13.1
CTA-363E19.2
RP11-24J23.2
linc-PRKACG-1
linc-CDH11-1
RP11-744D14.2
linc-NUBPL-2
linc-LGALS9B-2

RP11-755O11.2
RP11-147G16.1
CTD-3105H18.13
CTA-280A3__B.2
RP11-153M7.5
RP11-380F14.2
RP11-899L11.1
linc-GPR65-5
RP11-56H2.2
linc-ZP2
linc-ADCY1-4
RP4-539M6.14
AC004231.2
RP5-1004I9.1
linc-SLC25A38
linc-AADAT-1
RP11-680F20.4
linc-ARFGEF2-3
AC010880.1
linc-P2RX7
AC007952.4
RP11-124K4.1
LINC00433
RP3-399L15.2
SPATA42
RP11-295P9.6
KCNQ1-AS1
AC017116.11
AC000364.1
TGFA-IT1
RP5-1187J4.11
RP11-3P22.1
AC092580.4
RP11-973H7.3
linc-LRRC3B
RP11-3L23.2
RP11-758M4.4
UPK1A-AS1
RP11-290F24.3
CTD-3064H18.4
linc-RGMA-5
RP11-383C5.3
RP11-26E5.1
RP11-719K4.6
DAQB-12N14.5
linc-RELL1-3
RP11-408J6.1
linc-LAMA4-1
RP11-521E5.1
RP11-359M6.1

RP11-666A8.9
AC068057.1
RP11-285M22.1
CTD-2036A18.2
RP11-374M1.2
RP11-18F14.2
linc-KLF3-2
linc-HDAC4-1
SLC8A1-AS1
RP11-10A14.6
RP11-447B18.1
RP11-162I7.1
linc-LRRTM4-7
linc-NTN1-3
AC114812.8
RP11-164N3.2
AC010971.1
linc-TARP
linc-GATA5-1
AP000304.2
linc-CDCA4-2
linc-PLEKHO2-1
RP11-292D4.1
AC136896.1
linc-RASGRP1-1
linc-AADACL4
RP11-96C21.1
LINC00302
LINC00977
linc-RXRA-1
RPS6KA2-IT1
linc-FAM113B-1
RP11-503D12.1
AC023115.4
RP11-187O7.3
RP4-630A11.3
RP5-940F7.2
RP11-227D13.4
linc-CDH3-1
RP11-10O17.3
AC010745.4
RP11-203J24.9
linc-COL4A2-7
RP4-758J24.5
linc-EEF1B2-2
ZNF32-AS2
RP11-138H11.1
RP11-753B14.1
RP11-22M7.2
RP11-565F19.2

linc-RORB-8
RP11-58A17.4
SNX29P2
linc-MARCKS-3
RP11-795H16.3
linc-ADD3-9
linc-TTPA-1
RP11-19D2.2
LINC00710
linc-ABCA5-1
RP11-802F5.1
CTD-2247C11.5
linc-KCMF1
RP11-274H2.2
LINC00395
RP11-689P11.3
AC009312.1
AC009480.3
RP5-1044H5.1
LA16c-358B7.3
RP11-95P13.1
linc-AQP11
linc-MLLT4-1
linc-TMEM8A-1
CTD-2329K10.1
linc-OPRK1-5
LINC00358
linc-CD1D-1
RP11-114H24.3
RP11-538I12.3
linc-BAI3-1
RP11-713M15.1
RP11-344J7.2
linc-KCNG1
RP11-196P2.1
RP3-467K16.2
linc-PCDH8-6
AC092652.1
CTC-384G19.1
CTC-244M17.1
RP11-551L14.4
linc-ZNF479-9
RP11-318C24.2
linc-RNPC3-5
RP11-314D7.2
CTC-487M23.7
linc-MALT1-1
RP11-190J1.3
linc-FAM75A6-3
CTD-2240J17.1

CTD-2527I21.9
linc-OLIG3-3
linc-TECTB-1
OSBPL10-AS1
linc-HAAO-6
linc-BCL11B
linc-AUTS2-5
RP11-545L5.1
RP11-436F23.1
linc-FRG1-4
RP4-705F19.1
linc-DOCK3
linc-SEL1L-4
linc-ZFP42-9
linc-MAN1A1
RP11-393I23.4
RP11-324D17.2
TRPC7-AS1
RP11-89M16.1
linc-MBOAT4-1
linc-SLC30A5-3
linc-CISD1
RP11-99L13.2
RP11-70F11.7
linc-ODF3B
RP11-467D18.2
RP11-402J6.1
CTB-32H22.1
RP11-755B10.2
AL109761.5
AC060834.3
RP11-575I8.1
RP11-21L1.1
RP11-932O9.9
linc-ADRA2A-1
RP3-417L20.4
linc-KLK15
linc-ST3GAL5-3
RP11-544L8__B.2
linc-GAS1-2
LINC00867
RP11-48F14.2
RP11-650L12.1
RP11-423P10.2
TMEM212-IT1
CTD-3149D2.4
RP4-533D7.4
RP11-476F14.1
linc-COX4NB-3
CTD-3234P18.2

CTD-2015H3.1
linc-SCAMP1-1
linc-ZNF257-1
RP11-17A4.4
linc-CHST8
RP11-178L8.9
RP11-339D23.1
AC003003.5
GS1-278J22.1
CTD-2318H23.1
RP11-513D5.2
linc-DLX6-1
linc-PAK1-2
linc-ABCA5-8
RP11-57A19.5
linc-PCDH20-6
RP11-345F18.1
RP11-235E17.4
AC019118.4
linc-ARID1B-2
linc-GRIA3
linc-DNAJC24-1
linc-ARHGAP32-1
RP11-3N13.2
RP11-12L8.1
RP11-390D11.1
linc-JAKMIP3-1
linc-PRR20A-5
AC008940.1
RP11-430H10.1
AC011998.1
AF131216.6
RP11-11N5.3
AP000345.1
C16orf82
RP11-118B18.1
AF038458.5
linc-TRIM43B-3
RP3-341D10.4
RP11-344L13.2
linc-HS6ST1-4
AC079154.1
CTC-297N7.7
RP11-57J16.1
RP11-462G8.3
AC009095.4
RP13-216E22.5
RP11-462B18.2
RP11-100G15.10
RP11-624J12.1

linc-CHMP4C-2
RP11-481C4.1
RP3-388N13.3
linc-NPHS1
RP11-17M24.2
RP11-490E15.2
RP11-199F6.4
linc-IRX1-3
RP11-672F9.1
RP6-201G10.2
RP11-202H2.1
linc-DTHD1-5
RP11-20G13.3
RP3-380E11.2
RP11-68I3.2
ST6GAL2-IT1
RP11-757O6.1
RP11-950C14.3
linc-GAS7-2
PCAT4
CTB-25B13.6
RP4-683L5.1
AC004158.3
CTC-457E21.2
linc-RAB3GAP2-3
RP11-399O19.8
linc-SALL1-2
RP1-309H15.2
linc-APOC3-3
RABGAP1L-IT1
linc-GPR27-2
RP11-347H15.4
RP11-54O15.3
linc-FRMD3
linc-OPRM1-2
linc-UBQLN2
linc-GNAI1-1
RP11-182J1.12
RP5-1107A17.4
RP11-725M22.1
linc-ODZ4-2
RP11-13K12.2
RP11-503L19.1
RP5-1172N10.4
RP13-439H18.4
AC002551.1
RP11-208N14.4
RP11-130F10.1
AC007092.1
RP11-351N6.1

AC011747.3
linc-NPAS2-1
SGOL1-AS1
RP4-806M20.3
LINC00942
RP13-401N8.1
linc-TRA2A-2
linc-MRPS31
LINC00342
RP11-262I2.2
AC116366.6
EIF2B5-IT1
RP3-340N1.6
RP11-475O23.3
RP11-661G16.2
RP11-1149O23.2
RP11-960B9.2
linc-ALCAM-4
AC004054.1
TEX41
LINC00221
linc-SLC19A1-4
linc-QRFPR
linc-C18orf55-3
RP11-430C1.1
RP13-81N3.2
RP11-17G12.2
RP5-952N6.1
linc-HABP4-2
CTD-2007A10.1
RP11-650J17.2
linc-GATA5-4
RP11-586K12.10
RP11-264B14.1
AC099552.2
HCCAT3
AC017048.2
COL18A1-AS1
RP11-384O8.1
RP11-98L5.5
RP11-720N19.2
RP1-156L9.1
RP1-278O22.1
RP11-734K21.2
linc-TMEM14A-2
RP11-798G7.7
RP11-544A12.4
RP11-574F11.3
AC073321.3
RP11-324P9.1

linc-FAM120B-4
linc-RTTN-2
RP11-470M17.2
RP11-34C15.2
linc-TMEM99-1
LINC00930
LINC00957
RP5-1057I20.2
linc-CABP2
RP11-436H11.6
linc-MYO3A-1
linc-MTERFD1
RP11-122G18.5
linc-C4orf37
RP11-430B1.1
linc-OAF-5
RP11-562L8.1
RP11-641D5.2
RP11-840I19.3
AC012370.3
CTD-2007H18.1
RP11-207N4.3
linc-BAIAP2L1
linc-NUDT15-4
linc-ACTL7A-5
XXbac-BPGBPG55C20.1
linc-NHLH2-3
RP11-893F2.9
AC004014.3
RP11-142M10.2
AP000289.6
CXorf49B
CTB-59C6.3
linc-C3orf57-2
RP11-150O12.4
RHOA-IT1
CTD-2503I6.1
RP11-310I24.1
linc-HNRNPA1-3
RP11-279N8.1
CTD-2062F14.3
NALCN-AS1
RP11-136K7.3
linc-FBXL7-2
CTC-436K13.2
RP11-143M1.3
LINC00556
RP11-6N17.2
linc-SLITRK1-1
RP11-282K24.3

linc-CLMN-2
AC007422.1
RP11-96D1.9
linc-C16orf68-1
RP11-598C10.1
RP11-80F22.14
RP11-298D21.1
AF067845.1
linc-MID2
linc-RPL11-1
RP11-1072N2.3
RP11-1151B14.3
linc-ARHGAP26-6
hsa-mir-335
RP11-554L12.1
linc-MON1B-2
linc-SYNGR1-2
CTC-529G1.1
linc-STX2-7
CTB-25J19.1
RP11-256I23.2
RP11-269G24.3
AC061961.2
CTC-573N18.1
RP11-63K6.7
linc-C15orf59-1
AC024560.2
linc-HTR2A-1
RP11-328C8.4
linc-SPATA16-2
RP11-554D15.3
RP11-275I4.2
RP5-857K21.15
RP11-299M14.2
CTC-565M22.1
RP11-173D3.1
CTD-2044J15.1
LINC00349
RP11-143A12.3
RP11-60A8.2
LINC00618
AP004550.1
linc-POM121L12-4
RP3-323N1.2
RP11-80B9.4
linc-TUSC1
RP11-265P11.2
RP11-25I15.2
linc-TFAP2D-1
AC010525.4

RP11-392B6.1
ABCA9-AS1
AC113331.9
RP11-133I21.2
LA16c-83F12.6
linc-SFSWAP-2
RP5-945I17.2
-
RP11-490G2.2
RP11-167H9.5
RNF157-AS1
AC093159.1
CTD-2659N19.2
CTD-2296D1.2
RP11-282I1.2
RP11-293E1.2
CTA-796E4.4
linc-NAV3-2
CTD-2554C21.2
RP11-260A9.6
linc-SLC35F5-3
CTD-2210P24.3
RP11-527H14.4
ZBTB20-AS2
linc-APPL2-3
linc-FOXK1
linc-MARK1-3
LINC00487
RP5-1021I20.5
RP11-554D20.1
linc-PTPRU-2
RP11-256L6.2
linc-NOM1-2
RP11-856M7.2
RP11-799P8.1
RP11-505E24.3
CTC-277H1.7
AC007787.2
linc-TSHZ2-1
RP11-297C4.3
linc-KLHL29-2
linc-CSTB-6
RP11-844P9.2
linc-RABL3-1
RP11-21G20.3
RP11-401P9.1
linc-HNRNPA2B1-2
HTR4-IT1
linc-MACF1-1
CTD-2135D7.4

RP11-577G20.1
linc-LRGUK-1
RP11-769N22.1
LINC00927
RP11-368I23.2
AC015849.2
RP11-678G14.4
RP11-317N8.3
AC096570.2
XX-C2158C12.1
RP11-317O24.1
linc-ERICH1-7
RP1-128O3.4
GS1-433O24.1
linc-VLDLR-2
linc-GABRA6-2
RP11-566K11.5
linc-LEPROTL1-7
linc-GHR
ANO1-AS1
linc-ELTD1-6
RP11-121P10.1
CTD-2058B24.3
CTC-523E23.4
RP3-518E13.2
RP11-398E10.1
RP11-383H17.4
RP11-1437A8.4
RP11-265N7.1
RP5-997D24.3
CTD-3080P12.3
CTB-49A3.4
linc-CCDC67
RP11-788H18.1
AF131215.2
RP5-837I24.6
linc-SLAMF9
linc-NPHP1-1
RP1-104O17.3
RP11-24J19.1
EGFLAM-AS4
linc-PRMT6-2
LA16c-390E6.5
RP11-298H24.1
RP11-119F7.5
RP11-428G2.1
linc-PTPRD-3
LA16c-360H6.3
RP11-526N18.1
IGBP1-AS1

AC034228.3
linc-MGMT-4
LINC00518
RP4-681L3.2
RP11-351O2.1
AC104984.4
linc-LYPD6
RP11-44K6.4
RP11-386P4.1
CTD-2501M5.1
RP11-284A20.2
linc-RBL2
RP1-86D1.2
RP11-18F14.1
linc-TP53BP2-4
linc-UMODL1-5
ARHGEF7-AS1
CCDC148-AS1
RP11-18H21.1
RP11-806J6.1
linc-HS3ST5-2
RP11-677I18.4
linc-STAP1-3
RP11-390N6.1
RP11-90J7.2
EFCAB6-AS1
GS1-204I12.2
AC011524.1
RP11-173L6.1
RP11-158M2.3
RP11-627J17.1
RP11-460H9.1
AC093585.6
linc-CNTLN-4
linc-WDR17
RP11-274J7.2
RP11-88L24.4
linc-YIPF5-3
RP11-737O24.2
RP4-773N10.6
RP11-355I22.5
RP11-159L20.2
RP11-202A13.1
RP11-95G17.2
RP11-2O17.2
CTD-2555C10.3
RP11-775C24.3
SNORA59B
RP4-791M13.4
linc-ATP6V1C2-3

RP11-340A13.1
AC100830.4
linc-UBE2F
RP11-144G6.10
ATP13A4-AS1
CNOT10-AS1
linc-CBLN4-1
RP11-260M19.2
RP5-1051H14.2
RP11-338N10.2
UPP2-IT1
linc-SOX6
RP11-662M24.1
RP11-52J3.3
RP11-431K24.4
AC018731.3
ARMC2-AS1
RP11-118H15.1
RP11-35L17.3
FRMD6-AS2
RP11-188P20.3
linc-B3GALT2-1
linc-TGFBR2-2
RP11-461L13.4
RP11-73M14.1
CTD-3010D24.3
RP11-120K24.4
RP4-802A10.1
FLG-AS1
RP4-660H19.1
RP11-409I10.2
AC093484.4
MIR3150B
linc-NGFR-3
RP5-858B6.3
linc-GDF10-3
linc-ZNF599-1
linc-PARP11-3
AC124057.5
linc-FAM92A1-3
RP11-362A9.3
RP11-381N20.2
RP11-510C10.3
RP11-94M14.3
CTD-2015A6.2
RP11-740P5.2
linc-NFIA-2
RP11-348J12.2
linc-KLKB1
RP11-723G8.1

CTD-3185P2.2
RP11-2L8.1
RP11-612A1.1
RP11-1006G14.4
RP11-809F4.3
CTA-972D3.2
AC105339.1
RP11-379I19.3
LL0XNC01-7P3.1
linc-KIN-7
RP11-444P10.1
linc-CPXM2-2
RP3-377H17.2
linc-CPO-1
RP11-237D3.1
CTD-3187F8.14
RP5-956O18.2
RP11-448G15.3
AC090044.2
LA16c-366D3.1
RP11-626E13.1
RP11-542G1.1
linc-RUFY4-3
AC104654.1
linc-SPRY1-1
linc-CCDC60
linc-FAHD2B-2
U91319.1
CTB-114C7.3
RP11-1191J2.4
linc-GPHN-2
linc-CD180-7
RP11-383J24.6
linc-KIAA0564-2
HULC
linc-TMEM132C-7
UHRF1
CTD-2515A14.1
linc-DOCK10-1
RP11-805I24.2
RP11-831A10.2
RP11-74K19.1
RP4-813D12.2
linc-CLCN6
CTD-2116F7.1
linc-ZNF727-2
LINC00284
AC020743.3
RP11-1018N14.2
linc-PIGX

CTD-2309O5.3
RP11-661A12.14
RP11-63P12.6
RP11-810M2.2
RP11-91I20.1
CTD-2270L9.2
RP3-436N22.3
linc-FAM75A7-15
RP11-541G9.1
CTB-47B11.3
AC133633.1
RP11-179A7.2
AC013401.1
linc-KIF26B
linc-LRRTM4-2
AC092675.4
RP11-91I20.4
AC003092.1
RP11-4F5.2
linc-C15orf41-5
RP11-327I22.4
linc-HLF
RP11-616M22.1
linc-SERPINB3-2
linc-C14orf102-1
ARHGEF9-IT1
linc-CHST2-2
RP11-597D13.8
CTC-321K16.1
ZRANB2-AS1
CTD-2006O16.2
CTD-2236F14.1
AC002368.4
linc-CDC42SE2
RP11-626G11.3
RP11-138H8.7
AC096670.3
CSAG3
CTA-992D9.6
LINC00871
linc-DCT-1
linc-FAM75A7-9
RP11-203L2.4
RP11-73G16.3
AC012499.1
RP11-371M22.1
RP11-673E11.2
RP11-452N4.1
RP11-20L19.1
RP13-923O23.6

RP11-923I11.1
RP11-19O2.1
linc-CPEB4-8
AC007386.4
RP11-420K14.8
AC018730.4
linc-KIAA0649-2
CTD-2189E23.1
RP11-576M8.2
RP11-70O5.2
RP11-20I20.2
RP11-322N21.2
AC012363.13
RP11-298O21.2
RP11-285E9.5
linc-FNBP1-2
AC106874.1
RP11-632F7.3
linc-CSNK1E
RP11-64C1.1
RP11-259O18.4
linc-NCOA1-1
RP11-644F5.16
RP11-838N2.3
RP11-297P16.4
HCG20
RP11-192H23.8
AC093381.2
XX-FW81066F1.2
RP11-450H6.3
linc-NPVF-3
AC096649.3
RP11-134N1.2
linc-GABRB2-1
RP11-379P15.1
KB-318B8.7
AC107218.3
AC012506.3
AP001505.9
RP11-843A23.1
linc-PRPF18-3
PINK1-AS
RP11-799D4.2
RP11-248J18.3
linc-MYEOV-2
AC016994.2
CTB-43E15.4
linc-FAT3-2
RP11-438D14.2
AC004741.3

linc-DSP
RP11-94M14.2
RP11-701H24.3
AC006547.15
linc-NXT1
RP11-367G18.1
STEAP3-AS1
RP11-196O2.1
RP11-277P12.10
RP11-310E22.5
RP11-13L2.2
LA16c-380H5.4
RP11-388P9.2
LINC00470
RP11-530N7.3
CTD-2297D10.2
CTD-2081C10.7
linc-PARK2-2
SERHL
AC104135.4
RP11-1152H14.1
linc-SMOC1
RP11-231I16.1
linc-PEX3
TINCR
linc-ENOSF1
RP11-767N6.7
linc-WDR7-6
linc-DGKH-3
RP11-473M14.3
linc-IFT74-5
linc-CPEB2-4
linc-FAHD2B-7
RP11-1197K16.2
RP6-74O6.2
linc-CPNE5
linc-PELO-2
linc-HUS1B-5
RP11-323H21.3
linc-COMMD6-2
linc-DAAM1-1
RP11-609L23.1
AC092625.1
CTD-2193P3.2
RP11-91H12.4
AC002056.5
AC133785.1
linc-ZNF131-6
linc-RPP30-4
RP11-506O24.1

RP11-337N6.2
CTD-2632K10.1
linc-PTPN3
RP5-1031D4.2
linc-AJAP1-1
ANO1-AS2
RP11-811P12.3
PHEX-AS1
linc-CPEB2-12
RP11-867G23.3
AC115115.2
RP11-187C18.5
linc-MAFB-1
RP1-20B11.2
AC006196.1
RP1-170O19.17
RP11-403I13.7
RP11-313F23.4
ZNF205-AS1
AC092162.1
LINC00651
CTD-2396E7.9
linc-CDH4-1
RP11-124N3.2
AC009970.1
RP11-127B16.1
RP3-368B9.2
RP11-388K2.1
CTD-2010I22.2
RP11-693J15.5
RP11-103J8.2
RP11-31I22.3
AC006273.7
AC113618.1
RP1-5O6.5
AC073115.6
RP5-1010E17.1
AC078851.1
linc-SERPINI1-2
RP11-727A23.4
CTA-204B4.2
RP11-395B7.2
linc-OR7C2-3
linc-TYRP1-7
CTD-2314B22.1
AF178030.2
linc-CD247
RP11-99C10.1
EGFLAM-AS2
LINC00520

linc-WRB-1
linc-CLLU1-5
CTD-2213F21.4
AC006076.1
RP11-123M21.1
SAMSN1-AS1
AC025811.3
linc-CNN1
RP11-352G18.2
RP11-360L9.8
RP11-321E2.3
RP1-272L16.1
AC004041.2
RP11-85G21.2
linc-QSOX2
RP11-107M16.2
RP11-605F22.2
RP13-210D15.4
RP11-665N17.4
AC006552.1
CTD-2619J13.16
LINC00693
linc-EPHA7-3
linc-SCYL1-3
RP11-44F21.3
linc-PSMD7-4
RP11-180N14.1
linc-SLITRK5-6
CTC-360G5.6
RP11-314C16.1
RP3-470B24.5
RP11-753H16.5
linc-ZNF423-1
RP1-288L1.4
CTD-2235H24.2
RP11-291B21.2
linc-ANKRD28
ABCC5-AS1
linc-ATP2B1-2
linc-ANKRD9-1
linc-C11orf66
AC008746.12
AC073236.3
linc-REPIN1
linc-SLITRK5-9
linc-FAM120B-2
AF064860.5
RP11-445H22.3
LINC00705
linc-MORF4-3

RP11-385M4.1
PTPRG-AS1
RP11-254F19.3
linc-C7orf65-11
RP3-470L22.1
AC097468.7
AC018737.3
AC108868.5
GS1-166A23.1
linc-FAM9C-1
RP11-2C15.1
RP11-855A2.3
linc-MAN2A1-2
RP11-661G16.1
RP11-57H12.5
linc-EMP2
AC007879.2
linc-C13orf23-1
CTC-359M8.1
AC114808.3
linc-NCOA2
AC005944.2
RP11-111I12.1
RP11-648F7.1
RP11-99J16__A.2
RP11-343J3.2
linc-SOCS5
RP11-567N4.2
RP11-625I7.1
linc-ELF5
AC004840.9
linc-MECR-5
CASC16
linc-HSPH1
linc-NEK2
RP11-26P13.2
ZNF32-AS3
linc-TMPRSS15-7
GS1-519E5.1
RP11-414J4.2
RP11-84A14.5
LINC00208
linc-IQCG-5
RP13-514E23.1
RP11-680F20.6
AL109767.1
RP11-362K14.6
RP11-381K20.4
linc-C13orf34-16
linc-DEGS2

AC074389.5
NAALADL2-AS3
linc-ACTL7A-4
linc-ISOC2-1
RP11-689K5.3
RP11-96H17.1
RP11-338H14.1
AC005703.2
RP11-501G7.1
RP11-434C1.1
LINC00608
RP11-14C22.3
linc-ODZ4-1
RP11-498P14.4
AC074011.2
linc-XPC-4
linc-UNC13C-2
RP11-342M3.1
linc-SUPT3H
linc-PITRM1-5
RP11-94B19.6
RP11-89N17.4
XXbac-B33L19.4
AD000684.2
linc-PUM2-2
RP11-397C12.1
RP1-292B18.4
CTB-193M12.3
RP11-94B19.2
linc-ARFGEF2-10
linc-ATF7IP2-1
CTD-2560E9.3
AC007349.4
RP11-730N24.2
RP11-523L20.2
RP11-517A5.5
AC141930.2
RP11-221J22.2
RP11-82L18.2
RP11-525K10.1
CTD-2245F17.6
RP11-131J3.1
AC005387.3
linc-ZWINT-2
RP11-1081L13.4
LINC00440
KB-1460A1.2
linc-USP16-11
XXbac-B461K10.4
linc-GALNT13-2

linc-DSG1-2
linc-NRSN1-5
RP11-284A20.3
linc-LOC389493-1
LINC00323
RP11-317B7.3
linc-ASTN2-2
AC003102.3
linc-DNAJC19-4
CTD-2371O3.2
CTD-2568A17.8
linc-IGFBP7-4
RP11-13N12.2
RP11-472N19.3
CTA-363E6.6
linc-MKI67IP-4
EIF2B5-AS1
linc-ANO1-2
CTC-400I9.3
RP11-497K21.1
linc-STXBP1-1
linc-PTPRS-1
linc-GCNT2-4
linc-UGT8-1
linc-YTHDF3
linc-PKN2-1
linc-NUMB
RP11-989E6.8
RP3-335N17.2
LINC00837
RP11-14D22.2
linc-SNX9
RP1-60N8.1
RP11-220C2.1
linc-CREM-4
AC084149.1
RP11-495P10.7
RP11-236J17.6
RP6-24A23.7
AC090696.2
RP11-311C24.1
RP11-243A14.3
linc-NKX2-2-5
XXbac-B476C20.13
RP11-755F10.3
RP1-253P7.1
HOXD-AS2
RP11-98D18.1
RP11-114M1.2
AC004988.1

RP11-351J23.1
linc-VRK2-2
RP11-577B7.1
RP11-805L22.3
RP11-133L19.1
AC079790.2
RP1-137H15.2
RP11-21M24.3
ARHGAP26-AS1
RP5-865N13.1
RP11-554D13.1
DNAJB8-AS1
AC098820.4
RP11-124B13.1
RP11-214O1.1
RP11-313P18.1
RP11-260M19.1
AC013402.5
linc-FAM5C-1
RP11-451G4.2
RP11-600K15.1
CTD-2012K14.2
RP11-66N24.3
RP11-463O9.6
AC079117.1
linc-CITED2-4
linc-EGLN3
linc-TGM3-2
CTD-2354A18.1
RP11-343N15.5
RP11-787D18.1
RP11-403B2.6
AP000233.3
RP11-51M24.1
CTD-2555I5.1
RP1-68D18.4
linc-AGA-2
RP11-655H13.2
linc-SALL1-5
RP11-428O18.4
linc-GALNT13-3
linc-DHX37-23
RP11-794P6.6
AC093326.3
CTD-3064M3.1
linc-FAM92B-5
RP11-415D17.3
linc-CIB1-2
linc-GBP5-5
linc-CCDC90A-7

RP11-109D20.2
linc-MANEA-8
RP11-35J10.5
RP11-557C18.4
RP11-439E19.1
RP11-575L7.4
AC007556.3
RP11-122M14.3
ANKRD10-IT1
RP11-214L19.1
RP11-152L7.2
GK-IT1
linc-RBMS1-4
AC005281.1
AF131216.5
CTD-2168K21.1
linc-BBS12-1
AC002480.2
RP11-164D18.2
RP11-108K3.3
RP11-688I9.4
linc-ADC
RP11-258C19.7
linc-ICA1
RP11-445P17.3
linc-TBC1D21
CTD-2292M14.1
RP11-763E3.1
AC023347.1
RP11-149I9.2
RP11-10A14.7
RP11-219J21.1
RP11-232C2.1
FGF14-AS1
linc-NDST1-2
RP11-488L18.8
RP11-316I3.1
CTD-2553L13.4
RP5-1119A7.17
RP11-203M5.2
AC069278.4
RP11-514D23.2
RP11-509J21.3
RP13-884E18.4
RP11-371F15.3
linc-CYBRD1-2
AC005534.8
RP11-165E7.1
RP11-118E18.2
linc-DENND1A

RP11-544D21.1
RP11-498J9.2
linc-ASXL1
linc-MC3R-2
ADARB2-AS1
linc-APOB-4
RP11-370I10.2
linc-LCP1-1
RP11-702L6.4
RP5-905G11.3
RP5-988G17.1
linc-CDH5-12
linc-TSC22D1-1
ETV5-AS1
linc-SLCO4A1-2
linc-CDH3-2
RP1-144F13.3
AC010976.2
RP11-313I2.11
AC140481.4
RP1-20B21.4
RP1-149A16.3
linc-PKP2
AC079612.2
RP11-229P13.19
linc-ATAD1
AC067969.1
RP11-75N6.3
linc-KIAA1524-2
CTD-2666L21.1
RP11-139J15.5
PCA3
CTB-31O20.4
linc-TNRC6A-1
RP11-474N8.5
RP11-790J24.1
linc-CASP10-1
linc-HIVEP1-1
CTD-2302E22.2
linc-LAMA1-1
RP11-178G16.4
RP11-347D21.3
CTC-529L17.1
RP11-884K10.6
RP11-707P20.1
linc-PRKAA2-2
LINC00524
RP11-467I20.6
PSORS1C3
RP11-136K7.2

RP11-875H7.1
linc-RBPJ
CTD-2306M5.1
linc-CCDC90A-2
RP11-144A16.5
RP1-178F10.1
linc-NEURL1B-2
RP11-63A1.2
linc-PRR5-3
RP11-138J23.1
linc-RXFP2-1
RP11-24P4.1
RP11-583F2.5
AC008440.5
linc-ALCAM-3
linc-DHCR7-2
RP11-69C17.2
linc-IER3IP1
linc-PRICKLE2-1
RP11-300J18.1
RC3H1-IT1
RP4-694A7.2
PSMD6-AS2
linc-LRIT1-1
linc-C15orf2-8
RP13-895J2.2
RP11-158M2.2
RP5-968D22.1
RP11-79E3.2
RP11-1018N14.3
linc-GGTLC1-8
RP1-66N13.1
RP11-115J16.2
SNORA70D
RP11-567C20.2
LINC00601
RP11-404O13.1
Z83851.4
linc-TACC3
RP11-304L19.1
AC012322.1
AC064834.3
AC008991.1
RP11-93K22.6
linc-NTRK2-6
RP11-292F9.1
AC073128.10
linc-RAB11FIP5
RP11-968A15.2
linc-SNTG2-7

AC007381.3
linc-C14orf159-2
WWC2-AS1
RP11-909N17.3
RP3-395M20.2
RP1-38C16.2
RP11-16P20.3
RP11-473C19.1
RP11-530I17.1
linc-PCSK6-2
linc-ATP1B1-2
linc-GABRR3-2
linc-TMEM132D-2
linc-IQCA1-1
linc-MRPS18A-2
AP006748.1
RP11-153K16.2
AC147651.5
USP12-AS1
LINC00113
RP11-457M11.5
AC073409.1
RP11-809H16.4
RP11-389O22.1
RP11-161M6.5
linc-C1QTNF3-1
RP11-76K13.3
linc-N6AMT1
XXbac-BPGBPG55C20.2
LMCD1-AS1
RP11-166N17.1
linc-ALDH1A2-1
linc-SEP15-2
linc-FRMD4B-2
CTD-2197I11.1
RP1-56K13.3
linc-COX19
RP11-327J17.1
RP11-464F9.21
DSCR10
RP11-125I23.3
RP11-619L12.4
CTD-2207A17.1
RP11-542F9.1
RP13-16H11.7
TRIL
RP1-137K24.1
linc-FCGR1A-3
linc-MPRIP-2
linc-BCL2A1-3

RP11-168L22.2
AC105402.4
RP11-479J7.1
linc-CCDC140-3
RP11-532M24.1
RP11-736G13.1
RP1-283K11.2
linc-LOC388630-3
linc-CEP110-10
RP11-586L23.1
linc-CCDC82-4
RP11-258B16.1
linc-PDE3A-2
RP4-537K23.4
linc-NAALADL1
RP11-463H12.2
RP11-326A19.4
RP11-76E17.4
linc-ATP6AP2-4
RP11-316A16.1
AC083864.3
RP5-1113E3.3
linc-RNF19A
RP4-763G1.2
linc-SMARCA2-3
AC010082.2
RP11-285G1.9
DISC1FP1
RP11-535C7.1
linc-TTLL7-6
linc-ACTRT2-2
AC008154.4
RP11-266A24.1
KDM5C-IT1
RP11-20G6.3
RP11-123B3.2
linc-NAV3-3
AP000356.2
RP11-408H20.3
AFF2-IT1
linc-NBAS-9
RP11-19P22.8
linc-CSMD1-5
RP11-685N10.1
LINC00887
PEX5L-AS2
CTD-2199O4.6
RP3-508I15.10
NAV2-AS2
DGCR5

linc-GRIK2-1
CTD-2033A16.1
linc-GRHL2-3
RP11-550C4.6
RP11-219B17.2
AC018705.5
AC010731.2
linc-CLRN2-1
RP11-748C4.1
MTUS2-AS1
RP11-628E19.4
RP11-74C13.4
RP5-1007G16.1
RP11-506D12.5
LINC00229
linc-ECHDC3-2
linc-GPATCH2-9
linc-ASCL1-3
CTD-2231E14.5
linc-GOT2-3
RP11-392O17.1
linc-MMD-1
CTD-2227C6.2
RP11-800A3.3
RP11-700H6.4
RP13-379L11.1
LL0XNC01-37G1.1
LINC00928
RP11-268G13.1
RP11-417B4.2
RP11-632B21.1
KB-1507C5.4
linc-ENPP6-4
RP11-264E20.1
RP11-10N23.2
RP11-75C10.7
RP11-293G6__A.2
linc-TMEM100-2
RP11-982M15.8
RP11-349E4.1
RP1-67K17.3
linc-CYLC2-3
linc-PCDH7-5
RP11-178F10.1
linc-CCT5-9
linc-CCT4
RP11-162J8.2
AP001615.9
linc-TRIM9-2
AC019117.1

RP11-804F13.1
linc-DLEU7-1
CTD-2562J17.4
linc-ADRA1A
linc-HIST1H2AG-3
NRG1-IT1
CTB-147C22.8
linc-ADCY1-2
U82695.10
RP11-435O5.6
RP11-1042B17.5
AJ006995.3
RP11-69E11.4
linc-SUMF1-3
CTC-524C5.2
CTB-58E17.2
RP11-384F7.1
linc-GNPDA2
AC009236.2
RP11-65J3.3
RP11-163M18.1
RP11-331F9.3
linc-KYNU-3
RP11-626I20.3
linc-CXorf59
linc-C13orf34-1
linc-HAO2-3
GIMD1
RP11-490N5.1
RP11-180M15.3
RP11-456H18.1
RP11-963H4.5
linc-HES1-3
linc-FLI1-4
AC026202.5
RP11-38C18.3
RP3-463P15.1
RP11-136F16.1
linc-SYNE1-2
RP11-540K16.2
CTD-2545H1.2
RP11-283I3.1
RP11-120K24.3
linc-C17orf102
RP11-774O3.2
CTD-2126E3.3
RP1-297M16.2
RP11-89M20.2
RP11-507B12.1
AC092687.3

linc-MLLT4-2
linc-NQO2-2
AC008992.2
RP1-63G5.5
linc-ITGB2-1
AC068542.1
linc-MLPH-2
RP4-788L20.3
linc-PPP2R5C
CTD-2013M15.1
RP11-806L2.6
RP1-65P5.5
linc-FAM19A5-1
linc-GLRB-6
linc-VTA1-1
AP001136.2
linc-FDX1
linc-ATG5-2
linc-RIPK4
AC007364.1
RP5-875H18.4
linc-KARS-3
RP11-354P11.3
RP11-429G19.3
RP11-110L15.2
linc-GPRIN3-1
CTC-459M5.1
RP11-78F17.1
RP11-328D5.1
AC137723.5
linc-PCDH20-9
RP4-529N6.2
linc-ZNF227
RP4-697P8.3
RP11-361L15.4
RP11-24H2.2
linc-GUSB-4
linc-KCNN2
AC114765.1
RP11-275N1.1
RP11-429J17.8
AC008073.9
CTA-331P3.1
linc-ARL6IP5
AC002429.5
linc-SGMS2-1
RP11-44F14.5
RP11-434D2.12
RP11-719N22.2
linc-STAB2-1

MIR519A2
RP5-1024G6.8
RP11-864G5.3
linc-C6orf120-6
linc-MYH10
WASF3-AS1
CTB-11I22.2
RP11-216N14.9
RP11-456O19.2
PCYT1B-AS1
AC093642.6
CTD-2015H3.2
RP11-522M21.3
RP1-166H1.2
RP11-298E2.2
linc-GOLGA8B-1
AC093639.1
CTD-2029E14.1
linc-THBS2-3
linc-FHL2-2
linc-FAM71F2-3
AC062021.1
RP11-380P13.2
RP11-46C24.3
LA16c-321D4.2
linc-SEPSECS-1
linc-ALDH1A1-3
RP11-288D9.1
AC011306.1
linc-KRBA1-1
AC106870.2
linc-GPSM1
RP11-231C18.2
linc-TPRA1
AL022476.2
linc-ADSS
RP11-862P13.1
linc-TCERG1L-2
RP11-465M18.1
RP11-264M12.2
RP11-461A8.4
RP11-285B24.1
RP11-363G2.4
RP11-171I2.5
linc-PTP4A3-3
CTB-35F21.5
linc-GBP6-5
AC006007.1
CTD-2277K2.1
RP11-78C3.1

linc-ORC6-2
RP11-396J6.1
linc-HOXC13-3
AC019349.5
EFCAB14-AS1
RP11-5N11.3
RP11-351E7.1
linc-XCL1
KIRREL-IT1
linc-PAFAH1B1
RP11-333J10.3
RP11-72L22.1
RP11-47P18.2
RP11-19C24.1
AC012506.2
AC159540.2
RP11-342K6.1
RP11-609N14.1
RP11-6N17.3
AC006262.4
linc-ZMIZ1-2
RP11-343J18.2
linc-SYT12
linc-PLEKHO2-2
AC006458.3
linc-SHOX-2
linc-ANKRD55-3
AC073130.1
linc-RASGRP1-2
CTD-2396E7.10
RP4-669B10.3
RP11-704C2.1
AP001042.1
RP11-452H21.4
RP4-742C19.8
RP11-316M1.12
CTD-2171N6.1
RP11-299L17.3
linc-PPP4R1-5
RP11-460B17.3
RP11-156L14.1
linc-CSTB-7
linc-NRG1-1
linc-HDAC11-2
linc-TMEM132C-14
RP11-347E10.1
TMEM5-AS1
RP11-64P12.8
WDR7-UA1
CTD-2152M20.2

FRMPD4-AS1
linc-ATP11B-2
linc-MKRN3-3
linc-CNTNAP5-4
AC011343.1
RP11-613M10.6
RP3-503A6.2
AC006272.2
LINC00316
LINC00379
RP11-115F18.1
RP11-703I16.1
RP11-753D20.4
linc-FAM96B
RP11-79E3.3
AC004691.5
RP11-339N8.1
RP11-154F14.2
GS1-421I3.4
linc-TUBA1A-4
RP11-329B9.1
AC010974.3
RP11-749H20.1
RP11-398G24.2
L29074.3
linc-KRTAP13-1-1
RP11-798K3.2
linc-ALX4-2
RP11-157I4.4
RP11-353N14.4
RP11-395P13.3
AC093326.2
RP5-827C21.2
linc-GLDC-2
RP11-406A20.4
RP11-180O5.2
CTD-3195I5.1
WWTR1-IT1
RP11-379I19.1
RP11-88H10.3
CASC7
linc-IPCEF1
AD000685.1
linc-JAKMIP3-3
RP11-316E14.6
RP11-671P2.1
RP1-286D6.5
linc-GLRA3-3
CTC-297N7.10
AC068490.2

RP11-769N19.2
CTD-2050I18.2
RP11-405O10.2
AC016735.1
RP11-79M19.2
AC004623.2
linc-HTR2A-3
linc-IGSF5-2
RP11-752G15.3
linc-STK32B-1
linc-C2CD4B-1
RP11-379C10.1
linc-OR5H6
RP11-372E1.4
RP11-511B23.2
RP11-199O14.1
AC012668.2
AC008278.3
ARHGAP26-IT1
RP11-22D3.2
RP11-496I2.2
linc-EVX1-1
AC007131.2
RP5-1107A17.3
linc-ASB2
linc-ATG2B-2
LINC00032
linc-RIMS1-1
RP11-317J10.2
RP11-452H21.1
linc-EFR3A-1
LINC00536
RP4-753D10.5
AC092620.2
CTB-1I21.1
RP11-989E6.10
AC004053.1
RP11-424M22.3
linc-EPB41L4A
linc-TEAD1
AC073316.1
TSIX
RP11-229E13.2
linc-DYNC1I1-1
RP11-313A24.1
linc-MOBP
RP11-885B4.1
linc-LRRTM1-2
linc-ADCY1-3
RP11-573N10.1

RP3-333A15.1
linc-PDE10A-3
RP11-298J23.5
RP11-448P19.1
linc-PGA3
linc-NFIA-4
AP003419.16
RP11-789C17.5
RP11-33N16.2
RP11-587H10.1
RP11-56L13.1
CTC-467M3.3
CTC-304I17.4
linc-ZNF479-8
RP11-615I2.1
RP11-233E12.1
RP11-443N24.2
linc-LOC100129636-3
WDR52-AS1
RP11-1012E15.1
HCG22
AC018359.1
linc-GALNT10
RP3-443C4.2
RP11-92C4.3
RP11-498E2.7
RP3-510O8.4
RP11-17A4.2
BPESC1
RP13-225O21.2
RP11-945F5.1
linc-RPP25-2
AC107070.1
AP001043.1
RP11-537H15.3
linc-B3GALT2-3
RP11-314N14.1
linc-LRRC38-3
RP1-93H18.6
AC007179.1
linc-PLP1
CT49
linc-ADRB1-2
RP11-104E19.1
RP11-264B17.2
RP11-587P21.3
CTD-2055G21.1
linc-MBL2-1
CTC-340A15.2
RP11-343L5.2

CTB-178M22.2
RP11-434D9.1
RP11-39M21.2
linc-B3GAT2-1
RP11-197P3.5
RP11-374M1.5
RP11-1L12.3
linc-ANG
linc-NR2F2-3
RP11-261C10.1
RP11-394G3.2
AP001062.7
linc-DHFRL1-4
AF131215.8
RP11-800A18.4
RP11-706F1.2
RP11-356O9.2
linc-ST6GAL2-8
RP11-1028N23.2
RP4-749H3.1
RP11-4M23.3
linc-NPVF-5
CTC-436K13.5
RP11-244H18.1
linc-FTH1-2
linc-CDH9-6
RP11-347C18.5
RP11-886D15.1
AC079776.1
RP11-71H9.1
linc-ROR2
linc-MGMT-1
AC013733.4
RP11-18H21.2
RP1-153G14.4
CTD-3099C6.9
linc-RTL1-5
linc-CAPRIN2
linc-MYCT1-1
RP11-395P13.6
linc-HNRNPA3-2
linc-ZNF676-1
RP11-461F11.3
linc-CDYL-3
RP11-504I13.3
RP11-327I22.5
linc-HMCN1-2
RP11-699L21.2
RP11-728K20.2
linc-ASCC3-2

linc-USP24-2
RP11-542G1.3
linc-NKIRAS1-1
RP1-187B23.1
RP11-26L20.3
CTD-3025N20.3
ARID4B-IT1
RP11-286N3.2
linc-KHDRBS3-5
RP11-402L1.11
RP11-492L8.2
GS1-600G8.3
linc-TPBG-3
RP11-347J14.8
RP13-221M14.2
CTD-2245E15.3
RP11-243J18.2
AL773572.7
RP11-179B15.6
RP11-357H3.1
RP11-58B2.1
ENOX1-AS1
AC004870.4
linc-CPEB2-6
RP11-126O1.4
RP11-1017G21.6
RP11-465B22.8
RP4-713A8.1
CTD-2140G10.4
RP11-673C5.2
RP11-508M8.1
AL133249.1
RP11-122C5.3
linc-TRRAP
linc-SCNN1G-2
RP3-522P13.3
RP11-396O20.2
linc-TSHZ2-4
RP5-1111A8.3
CTD-3138B18.6
linc-SAA2
AC008074.4
RP11-319E12.2
RP11-406H4.1
LINC00571
RP11-1018N14.5
RP5-1024N4.4
CTD-2128A3.2
RP11-686F15.2
RP11-104L21.2

RP11-10C8.2
linc-ATP6AP2-3
CTC-550B14.6
MPRIP-AS1
RP11-763K15.1
CTD-2105E13.15
linc-RNF32-1
RP11-265D19.6
RP11-101P17.9
AC007557.4
linc-CYP2J2
RP11-18F14.4
linc-CCT5-7
RP4-813D12.3
RP11-416N2.3
RP11-540H22.2
AC012507.3
TSPEAR-AS1
RP11-196E1.3
RP11-80H8.3
CTD-2135D7.2
RP11-328K22.1
CTB-107G13.1
RP11-127L20.5
AC104135.2
RP11-619F23.2
RP11-27M24.3
RP11-361F15.2
linc-MLXIP-1
linc-C2orf51
LINC00903
linc-IMPG1-2
RP11-856M7.4
linc-C1orf21-2
AC092839.3
linc-WDR60-2
RP11-375D13.3
linc-XPA-1
RP11-958J22.3
RP5-857K21.4
RP11-680F8.4
linc-PDLIM3
RP11-33B1.4
linc-ANP32D-1
RP11-850F7.7
linc-KLF7-2
AC005754.7
RP11-277J6.3
linc-RAPH1-2
AP002856.6

linc-RPS6KA3-1
CTD-2534I21.9
CTD-2526M8.2
linc-OTUD7B-1
RP11-166P13.4
AP001048.4
RP11-38G5.4
RP11-389C8.3
LINC00563
linc-CHRAC1-2
linc-YES1-1
linc-METTL14-3
linc-DPYD-2
RP11-285M22.3
RP11-344B2.2
RP11-767N15.1
RP11-190J23.1
RP11-269F21.3
linc-TNRC6C-3
linc-FBXO36-2
linc-LOC647589-1
linc-FAM114A1-2
AC011718.3
linc-ARSI
RP3-404K8.2
AC090957.2
linc-SPR-1
RP11-123K19.2
linc-SERP2-4
RP4-614C15.2
RP11-89K22.1
AC010761.10
linc-SOX11-1
linc-LPA-3
RP11-44N12.2
AC004009.3
RP11-84A19.4
RP11-530C5.2
linc-PCDH7-1
FOXI3
FGF12-AS3
SLC7A11-AS1
RP11-517I3.1
RP11-71E19.2
linc-PRMT6-4
RP1-81P11.1
RP11-98G13.1
RP1-12G14.7
RP11-264B17.5
ARSD-AS1

RBMS3-AS2
linc-IPO5
U66061.31
linc-GDF10-2
linc-KY-2
CTD-2006C1.12
CTA-85E5.10
linc-BCHE-1
RP11-59E19.4
linc-ART3
RP5-1029F21.3
linc-LHX2-2
RP11-396N11.1
linc-HNRNPA3-1
linc-CERK-1
AC116614.1
RP11-40F8.2
RP11-7F17.4
CTB-54I1.1
linc-PPAP2A-2
linc-ALG2-3
linc-FBRSL1-2
RP11-666A20.4
RP11-93N20.1
linc-CLMN-1
linc-GOT2-1
linc-UGGT2
linc-BHLHE23-1
RP11-269M20.3
RP11-227F8.2
linc-GAS2-1
RP11-21B21.4
RP11-48G14.1
RP11-63C8.1
linc-SDC2
AP000472.3
linc-SCN2A-2
linc-CCDC140-8
RP11-834C11.4
RP4-737E23.2
RP5-894D12.4
RP11-524F11.1
RP11-64D24.2
RP11-215A19.1
RP11-369E15.1
CTD-2223O18.1
RP11-63M22.1
AC104653.1
C10orf71-AS1
linc-VN1R2

linc-CD5
RP11-462P6.1
linc-FBXO33-2
RP11-172E9.2
linc-FAM78A-2
RP11-354H21.2
RP5-1069C8.2
RP4-809F18.2
TPRG1-AS2
CTD-2377D24.6
RP11-79C6.3
RP11-386D6.1
RP13-726E6.2
RP11-958F21.1
RP11-23E19.2
linc-CLINT1-2
RP11-505C13.1
CTD-2124B20.3
RP11-265N6.1
RP11-340A13.3
RP11-77M5.1
linc-SSTR1-4
RP11-697E2.4
LA16c-444G7.2
RP11-120K9.2
RP11-120K18.2
RP11-556I14.1
RP11-535M15.1
RP11-373I8.1
linc-HSPC159
linc-IRX2-1
LINC00344
LINC00331
linc-SCUBE2
RP11-848P1.4
linc-EML4
PKD1L3
linc-CPA6-5
RP11-3P17.5
RP11-594C13.1
CTD-2659N19.10
GPR158-AS1
linc-MYO3B-1
AC010729.3
NDP-AS1
RP11-552M14.1
RP1-122K4.2
RP11-573G6.9
AC003986.7
RP11-281O15.8

RP11-58K22.5
linc-TNP1-3
AC009531.2
RP11-8L21.1
RP3-412A9.10
RP11-567E21.3
AC005022.1
RP11-62C3.6
RP11-586K12.4
KB-1517D11.4
linc-NKX2-2-3
RP11-195B17.1
RP11-513O17.3
RP11-634B7.4
linc-CTTNBP2NL
AC005387.2
RP11-12M5.3
RP11-53B2.4
RP11-3J1.1
AC007464.1
RP11-477N3.1
RP11-40A13.1
RP11-14I4.3
AC013264.2
AC011284.3
AB019440.50
AC013472.4
RP5-1022P6.7
RP11-20G13.1
RP11-411B10.3
AC103563.8
linc-PRDM1-1
RP11-582J16.4
linc-PLCB4
RP11-76C10.2
linc-FOXB2-2
linc-GPR137B-3
RP11-317P15.3
RP11-285C1.2
CTD-2260A17.3
linc-ZNF716-4
RP4-765H13.1
RP11-417J8.2
linc-FAM69C-1
linc-THBD
linc-USP12-4
linc-FGGY-2
linc-GREB1-2
CTC-527H23.4
RP11-439L8.4

RP4-792G4.2
RP1-140J1.1
RP11-282C5.1
RP3-425P12.2
linc-SLITRK1-8
SRGAP3-AS3
linc-MEF2A
MIR4720
RP11-2L8.2
RP11-293A10.3
linc-PLTP
RP11-211G23.1
RP11-316P17.2
RP1-29G21.1
RP11-998D10.7
RP11-143M1.4
ARAP1-AS1
RP11-12A16.3
RP11-384M15.3
RP11-69I8.2
linc-FGFR1OP-6
RP4-765C7.1
AF038458.3
linc-RAB23-3
linc-CCKAR-3
linc-PLXDC2-1
RP11-263E1.1
CTD-2089N3.3
RP11-331K15.1
CDRT8
RP11-67M9.1
RP11-714G18.1
RP11-449L23.3
CTD-2023N9.2
linc-PCBD1-4
AC103828.1
RP11-776A13.1
RP5-905H7.9
linc-ZNF33A-3
RP5-1067M6.3
linc-RAB11A
linc-PRLR
RP11-170M17.1
linc-PCDH7-8
RP11-87N24.2
CTB-51A17.1
linc-C6orf145-2
RP5-974N19.1
linc-CHD9-5
AP001596.6

RP11-247I13.11
GS1-278J22.2
RP11-219C24.10
RP11-304F15.4
RP11-214C8.2
linc-PRKDC-2
CTC-321K16.4
RP11-196H14.4
linc-ZFP42-2
RP4-530I15.6
RP11-403P17.2
linc-DHRS2-2
RP5-857K21.2
RP11-521D12.5
RP11-344P13.4
RP11-96P7.1
linc-SPTBN1-1
linc-CBWD3-2
RP11-264L1.3
APOBEC3B-AS1
linc-DPP6-1
RP11-96D1.7
RP11-469A15.2
linc-KBTBD3
linc-KAZ
RP4-584D14.6
AP000959.2
RP11-293F5.1
linc-KIAA0182-2
RP5-965F6.2
CTD-3064H18.2
linc-PCGF5-1
CTD-2189E23.2
RP11-297L17.3
CTD-3037G24.4
SPATA13-AS1
RP3-413H6.2
RP11-421F16.3
RP4-652L8.1
linc-ZNF608-4
linc-PITX2-1
RP11-445O3.1
AC068483.1
linc-SUZ12-2
linc-SNX19
KB-1047C11.2
CTD-2011F17.2
LINC00603
linc-ZSCAN1-2
RP11-751H17.1

LA16c-390H2.4
RP1-202O8.2
RP11-60H5.1
linc-KIAA1217
RP11-173P15.3
RP11-403I13.8
RP11-348M17.2
RP5-1042K10.13
RP11-561P12.5
LINC00424
RP11-244O19.1
RP11-212E8.1
linc-RREB1-3
RP11-2L4.1
RP11-809N15.2
linc-GINS2
RP1-283K11.3
RP11-1081M5.1
AC004906.3
linc-ADAMTS8-2
linc-TMEM72-1
RP11-680B3.2
RP5-1121E10.2
RP4-620F22.2
RP11-513G11.4
RP11-514F3.5
AC003088.1
CTD-2311B13.7
linc-SLAIN1
linc-RAI14-2
RP11-587D21.4
GAS6-AS1
AC099342.1
linc-CACNA1S
CTC-429P9.2
linc-SPIN1-1
RP1-224A6.9
RP5-1006K12.1
linc-GLT25D2-2
AP000330.8
RP11-945C19.1
RP11-473O4.5
RP11-192H23.6
RP3-526F5.2
linc-CCRN4L-14
LINC00945
RP1-85F18.5
CTB-127C13.1
AC005265.1
RP11-61A14.2

RP11-848P1.3
CTD-2014E2.6
linc-CADM2-3
linc-EFCAB9-2
linc-LSR
linc-CHD1-3
RP11-114F10.2
RP11-52A20.2
AC002463.3
RP11-551M15.1
AC012593.1
CTC-327F10.5
CTA-14H9.5
AL133493.2
linc-RALGAPA2-3
RP11-309H21.3
RP11-501C14.6
linc-C11orf82-5
linc-FAM19A5-4
RP11-85G21.1
RP11-514D23.3
RP11-461A8.1
RP11-110I1.11
RP11-966I7.3
CTD-2196E14.9
linc-PFKFB3
RP3-446N13.1
RP11-715F3.2
RP11-789A21.1
RP11-128P10.1
linc-TSKU-1
RP11-779O18.3
linc-KCNA4-2
RP11-389J22.1
linc-BRI3BP-1
AC097500.2
RP11-10J21.4
AC079466.1
RP1-63M2.6
RP11-276G3.1
RP11-650K20.2
RP1-92C8.3
RP11-168J19.2
RP11-284J1.1
RP11-350F16.2
RP3-428L16.1
RP11-150O12.6
linc-NANOS1-5
RP11-449J1.1
RP13-436F16.1

KIRREL3-AS1
AC108004.3
CTB-52I2.5
CTD-2541M15.3
RP3-416J7.4
linc-CDH18-3
RP11-588P7.2
linc-GRIA4-3
RP11-529E10.6
RP11-37L2.1
RP11-1D12.1
RP11-44I10.6
RP11-85O21.5
RP11-529H2.1
RP11-132A1.3
linc-COL18A1-1
AC004878.2
RP11-315A16.1
RP11-430L16.1
CTD-3118D7.1
linc-AGA-5
RP11-70J12.1
RP11-1277A3.1
linc-PREX1-3
LINC00489
RP11-227F19.2
AC106053.1
RP11-264B14.2
LINC00508
linc-C10orf119-1
RP11-897M7.1
linc-IRX1-4
CTA-797E19.2
RP11-86A5.1
RP11-746M1.1
MIR4732
RP5-937E21.8
RP1-155D22.2
linc-RORB-2
CTD-3032H12.1
linc-SEL1L-6
linc-PITRM1-3
RP1-18D14.7
CTB-118P15.2
linc-ADRA1D-1
RP11-665G4.1
RP11-510M2.5
AC002519.5
RP11-641A6.3
RP11-25I15.3

PROSER2-AS1
linc-FAM116B-1
RP11-109M17.2
RP11-423O2.5
RP11-244K5.1
linc-P2RX4
RP11-544L8__B.4
AC005487.2
linc-WASL
RP1-84O15.2
linc-CNTNAP5-8
AC010890.1
linc-HIST1H2AG-1
RP4-633I8.4
RP11-4B14.3
CTB-176F20.3
linc-IRX1-6
RP11-432J24.3
linc-PAXIP1-4
linc-C15orf2-3
RP11-366L20.4
RP11-451B8.1
RP11-510M2.6
RP11-259O2.1
RP11-676J12.4
AC011298.2
RP4-610C12.4
linc-FRG2C-6
RP11-830F9.5
AC010745.2
RP1-302D9.3
RP11-87G24.3
linc-ZNF366-2
LINC00644
RP11-711G10.1
CTD-2653M23.3
KB-1410C5.3
RP11-136K7.1
RP11-861E21.1
linc-ITGB1
RP11-542K23.10
linc-MDGA2-3
CTC-457E21.9
RP11-77C3.3
RP5-964N17.1
RP11-380J14.1
RP11-538I12.2
AL163953.3
AC003985.1
AC083884.8

linc-SAMD12-1
RP11-118B18.2
CTC-218B8.3
LINC00939
linc-C4orf45-2
DIP2A-IT1
RP11-541N10.3
linc-SCAND3-2
CTD-2515H24.4
linc-UBE2QL1
AC010105.1
AL022344.5
RP11-267M23.3
CTD-2643K12.3
AP000302.58
linc-KIAA1383-1
linc-RGL4-4
RP4-740C4.8
CTD-2005H7.2
RP11-973H7.4
RP11-332E19.2
linc-LRRTM4-6
RP11-214K3.21
CTD-2199O4.7
RP3-406A7.7
RP11-491H19.1
linc-OXCT1-1
linc-TBX20-2
linc-IGF1R
RP11-512N21.3
RP1-172N19.1
RP11-661A12.5
linc-ATP5G3-1
RP11-627G18.1
AC009505.2
linc-C2orf27A-2
RP11-17L5.4
linc-FTMT-1
CTC-484P3.3
CTD-2192J16.26
CTD-2126E3.4
RP11-844P9.1
AC079779.6
RP11-324L3.1
RP11-575B7.3
CTD-3001H11.1
RP11-105C19.1
AF001548.5
CTD-2313P7.1
linc-BTC-2

RP11-501J20.2
RP11-217E22.5
HNRNPU-AS1
linc-SLC10A2-1
AC011524.3
RP11-297H3.4
linc-RYBP-3
RP11-493L12.5
U82670.4
linc-TBX3-5
linc-ZDHHC17-2
RP11-703H8.9
RP11-819C21.1
linc-CRYBB1-7
RP11-672A2.1
UBOX5-AS1
CTC-471C19.1
linc-KLHL29-9
RP11-25D3.1
RP11-375B1.1
RP11-626K17.3
RP11-116D17.3
LINC00392
RP11-3M1.1
RP3-523E19.2
linc-FAM154B-3
linc-ZIC4-1
linc-ARHGAP20-1
linc-SLC35C1-3
linc-CXXC1
RP11-434I12.4
RP11-326N17.1
AL132796.1
AP000462.2
RP5-1142A6.7
linc-FAM75A6-1
linc-CXCR4-1
linc-C9orf131
RP1-150O5.3
linc-PTDSS1-1
RP11-335E6.4
linc-KCNG2
FANK1-AS1
linc-IRF6
RP11-90D11.1
RP11-661O13.1
RP11-347D21.1
RP3-471C18.1
CTC-264O10.2
RP11-13J10.1

LINC00614
linc-PTBP2-7
GS1-259H13.2
LINC00326
linc-CCDC59-2
linc-UCHL3-6
KLHL22-IT1
linc-CPEB4-1
RP1-182D15.2
RP11-6I2.3
linc-C2orf69-1
CTC-273B12.10
linc-TSHR-1
linc-PDSS1-1
RP11-87E22.2
RP11-346D14.1
RP11-15F12.1
AP000692.9
linc-FAM183A
RP11-492I21.1
AC008592.8
linc-TMEM18-3
AP000265.1
linc-LOC100129520
RP11-204C23.1
SNORD64
linc-MLPH-4
linc-ACSM5-6
RP11-354P11.8
linc-SYNCRIP
AC024619.2
RP11-427H3.2
RP11-820L6.1
linc-CSTB-2
RP11-268F1.3
RNF216-IT1
CTD-2542L18.1
RP11-88H10.2
linc-NUDT6-2
RP11-301J16.7
linc-UQCRFS1-3
CTB-118N6.3
linc-FOXQ1-1
linc-APOH
CTD-2363C16.1
GLIS3-AS1
RP11-307O10.1
RP11-245A18.1
linc-LRRC8D-3
RP11-855O10.2

RP11-1O10.1
MIR1470
AC018647.3
RP11-642D21.1
RP11-204E9.1
CTD-3157E16.1
linc-ZNF543
AC004449.6
linc-ZNF107-6
linc-TAF5L-3
RP11-314M24.1
RP5-1029K10.4
RP11-432J9.6
linc-C10orf25
GS1-115G20.1
RP11-343D2.11
linc-C16orf82
RP11-364C11.2
RP11-435B5.3
POU6F2-AS1
CTC-400I9.1
RP11-439E19.3
linc-EGFL7-2
AC096772.6
MIR381HG
linc-ST8SIA3-2
RP11-379K17.12
AC010983.1
linc-SOD1-1
linc-SBDS-4
RP11-117D22.2
linc-CITED2-2
RP4-761J14.10
RP11-158M2.5
linc-C17orf108-5
RP11-277K23.1
RP11-380D15.3
linc-C13orf28-3
RP11-91J19.3
RP11-95H8.5
CTD-2293H3.1
RP11-363E6.4
linc-MANEA-6
AC062031.1
RP11-238I10.1
linc-RNPC3-4
linc-ARRDC4-1
AC006130.3
linc-SLITRK1-6
linc-FANCB

EPN2-AS1
linc-TREM1-2
RP11-148B3.2
RP11-290F24.6
RP11-384J4.2
RP11-222K16.2
linc-ENOPH1-1
CTC-210G5.1
SNORA71A
RP11-675P14.1
RP11-452B18.2
LINC00469
linc-UBE2E3-3
linc-ERG-10
MIR7-3HG
RP11-243J16.7
RP11-269C23.3
RP11-47J17.1
RP11-1109M24.5
linc-UBN2
RP11-130L8.1
linc-CBLB-4
RP3-326L13.3
linc-WISP3-3
RP11-69C13.1
RP11-342A1.1
RP11-417E7.2
linc-IMPG1-1
AC006159.5
RP11-95K23.6
RP11-50E11.3
RP11-351A20.2
CTD-2132N18.2
RP13-487K5.1
CTC-518B2.10
RP11-10J18.3
RP11-625H11.2
RP11-205K6.1
AC092635.1
RP11-170L3.8
linc-ZEB1-1
RP11-203M5.8
linc-HMX1-6
AC118345.1
RP11-232C2.3
RP11-98D18.15
linc-API5-1
RP11-107D24.2
RP5-1112F19.2
CTD-2588J1.1

RP11-179A16.1
RP11-81K13.1
FTO-IT1
RP4-591L5.2
linc-SLC22A16
RP11-807H22.7
RP11-281P23.1
RP11-64K12.9
LINC00499
RP11-689C9.1
RP11-809C9.2
RP11-233G1.4
LINC00442
RP11-338E21.2
linc-STIM2-2
GS1-24F4.3
RP11-80H5.7
linc-EMP1-1
RP11-180I4.2
linc-C21orf2-2
LINC00514
linc-CES5A-1
RP3-510L9.1
DAOA-AS1
RP11-615I2.7
linc-POLG
ITCH-AS1
RP11-782C8.5
RP11-62G11.2
linc-TNFAIP3-3
linc-RABL2A-2
RP11-359N5.1
RP11-298E9.5
RP11-213G21.2
CTD-2012K14.4
linc-ENPEP-1
RP4-785G19.5
RP1-293L8.2
CTD-2195M15.3
RP11-491F9.6
RP11-327F22.5
RP11-165F24.5
RP11-554A11.5
RP11-340F16.1
linc-GJA8-1
AC012317.1
RP11-378A13.1
AP001604.3
RP4-583P15.10
linc-LACTB

RP1-184J9.2
AJ009632.3
RP11-747D18.1
AP001465.5
AC018766.5
RP11-97N19.2
AC004893.11
CTC-313D10.1
RP11-421L10.1
RP11-561I11.2
CTD-2105E13.14
linc-ANKRD13C-1
XXbac-B33L19.3
CTD-2616J11.3
RP11-31I22.1
linc-RUNX1T1-2
CTC-548K16.1
RP11-63E9.1
RP11-402L6.1
RP11-547I7.2
RP11-473L15.3
RP11-313C4.1
DSCR4-IT1
RP3-437I16.1
linc-CPEB2-10
RP11-502M1.2
RP11-395D3.1
RP11-168P8.5
RP11-202D1.3
AC005616.2
RP11-403I13.5
RP11-890B15.3
linc-DLX2-3
AC005262.3
CTD-3214K23.1
RP11-468H14.2
AC093382.1
linc-ALOX15
RP11-525K10.3
AC005786.7
AC009365.4
XXyac-YM21GA2.3
WI2-1959D15.1
RP11-292E2.4
linc-DIRAS2-4
linc-PDIA6
linc-PCDH10-1
linc-C15orf2-5
linc-BTBD3-1
DMD-AS3

AC051649.16
linc-NOP14-3
RP11-416I2.1
linc-CNNM1
RP11-381K7.1
RP11-521L9.1
linc-RANBP3L-1
linc-LSMD1-1
RP11-318I4.1
RP11-25H12.1
RP11-252C15.1
RP11-510H23.3
RP11-807H17.1
linc-GLRX3-2
AC007319.1
RP5-843L14.1
RP11-215E13.2
linc-CKS2
RP11-693L9.2
AC125421.1
RP11-20B7.1
RP11-794M8.2
linc-ERGIC2
linc-GADD45A
CTD-2281M20.1
RP11-1082L8.4
AP003774.6
RP11-364P22.1
P4HA2-AS1
AC073254.1
linc-ZNF674
RP4-710M16.2
RP11-138C9.1
CDC42-IT1
RP11-253A20.1
RP11-51G5.1
linc-GPR65-1
RP11-504A18.1
linc-ZNF726-2
RP11-560G2.1
RP11-474D1.4
AC096554.1
RP11-272D12.1
RP3-333B15.4
linc-RAB6C-4
linc-GNB4-2
RP11-294K24.4
linc-KIF2A-2
LINC00116
RP1-140C12.2

RP11-321A17.5
linc-NRG2-1
RP11-225H22.5
linc-FAM75A1-4
linc-CCDC37-5
RP11-175O19.4
linc-ATPBD4-3
RP1-46F2.3
linc-NSUN2-1
RP3-476K8.3
AP001628.7
CTD-2008N3.1
RP1-287H17.1
linc-ODZ3-2
RP11-540O11.7
linc-TBX3-1
DPYD-IT1
linc-PGK2
RP11-488I20.3
RP11-168K9.2
linc-SULF2-3
RP5-890O3.9
RP11-6I2.4
linc-UGT2B10
linc-OR10Q1
RP11-1020M18.10
MIR4458
RP1-122O8.7
CTD-2194L12.3
AC005780.1
linc-GPR39-1
RP11-4C20.3
AC013436.6
LL09NC01-254D11.1
RP11-544D21.2
RP11-977G19.12
linc-LYZL2-4
JAG1
linc-NR2F2-6
AC114752.3
linc-PELI1-4
RP11-360F5.1
CYP4F8
RP11-619L19.1
NCOA7-AS1
RP11-13N13.2
RP11-34F13.3
AC005954.4
linc-ARMC3-1
CTA-929C8.6

LINC00565
linc-SH3BGRL2-3
RP11-672L10.1
RP11-798M19.3
RP11-542M13.2
linc-GJA5
RP11-82L18.4
linc-MAP3K8-2
RP11-725G5.2
RP1-212P9.2
AC139712.4
linc-CLN5-4
RP11-456I15.2
RP11-709D24.6
linc-CCDC82-2
RP11-218M22.2
RP11-706O15.7
AC108051.3
CTA-398F10.1
RP11-379K22.3
RP11-20I23.11
RP11-1105G2.4
RP11-1129I3.1
linc-MYLK4
linc-ANKRD20A1-1
RP11-686G23.2
RP11-443A13.5
RP11-685A21.1
CTD-2369P2.5
RP11-382B18.4
RP11-375A5.1
LRRC3-AS1
AC012442.6
AC005594.3
CTD-2034I4.1
linc-EPHA7-2
AP006547.3
RP11-89N17.2
linc-FAM134A
linc-COL5A1-1
RP11-629G13.1
RP11-253I19.3
LINC00351
RP5-1002M8.4
CTD-2325B11.1
linc-GLIPR1L1-3
LINC00529
linc-SAMSN1-4
CTD-2215E18.3
AC005258.3

linc-NSMCE4A-2
linc-FTSJD1
RP5-1119O21.2
linc-COX10
RP11-244B22.6
RP11-359D24.1
RP11-483P21.6
linc-LDB2-3
RP11-14J7.6
ATP2B2-IT1
RP11-626P14.1
AC106900.6
CTC-498M16.4
RP5-1139B12.4
RP1-90J4.1
linc-MRPS18C
AP000962.2
LA16c-380A1.1
linc-DCAF4L2
linc-TSHZ3-1
XX-C2158C6.1
RP11-916L7.1
SHANK2-AS3
JRKL-AS1
RP11-702F3.3
RP11-463P17.3
RP11-112J3.16
RP11-338O1.2
AC010761.13
RP13-348B13.2
AC008073.7
RP11-354A14.1
linc-JPH4-1
RP5-1180C18.1
RP11-526P5.2
CTD-2311B13.1
linc-AIM1-2
LINC00658
RP11-109J4.1
RP11-166O4.5
RP11-685G9.2
RP11-184I16.4
linc-ANKRD20A2-2
RP11-305L7.1
linc-RHOXF2
RP11-352B15.2
RP11-434E6.2
RP11-1090M7.2
linc-PPP1R3B-1
linc-ADAMTS19

linc-FANCL-6
CTD-3193K9.3
AC015849.19
linc-C9orf106-3
LINC00314
linc-MAP1LC3B-5
CTD-2050B12.2
AC023669.1
RP11-310J24.3
DHRSX-IT1
linc-PPP1R1A
RP11-516J2.1
RP11-556G22.2
CTA-520D8.2
linc-LRCH2-2
RP11-12C17.2
RP11-501C14.8
RP11-236P13.1
CTD-2126E3.1
linc-TCP10-2
AC005009.2
linc-SLC2A13-4
XX-C2158C6.3
linc-NXPH2-1
linc-EML6-3
FAM138D
RP11-1022B3.1
RP11-359D14.3
RP11-685F15.1
CTD-2139B15.4
RP11-231G15.1
linc-ADRA1B-1
linc-KCNS2
RP11-381O7.3
RP4-650F12.2
DLGAP1-AS5
linc-XPO7-1
RP11-579E24.1
AF230666.2
LINC00051
GS1-39E22.2
CTD-2562J17.2
linc-NIPAL2-2
RP11-143P4.2
linc-RYK
CTD-2647L4.5
RP11-466P24.7
linc-COX4NB-4
linc-HABP2
linc-KCNMB1-1

linc-WFIKKN2
FAM99B
RP11-44N21.4
RP11-383G6.3
RP11-350A1.2
RP11-308K2.1
linc-CLPB
AC109642.1
RP11-319G9.3
AC005518.2
RP11-384P7.6
CTD-2243E23.1
AC090952.5
RP11-732M18.3
Z82214.3
RP11-568A7.3
linc-IL9-2
AL163953.2
RP11-424M21.1
RP11-367G6.3
RP11-973F15.1
RP11-753A21.2
linc-POLE4
linc-NTRK2-4
linc-CDR2-2
AP000997.2
linc-NFE2L3-1
RP11-138E9.2
RP11-379J13.2
CTD-3076O17.2
AC005616.1
RP11-20I23.8
linc-TAF1B-3
RP11-659G9.3
RP11-307C19.2
RP1-142L7.9
CTD-2201I18.1
linc-ALDH1A1-1
linc-CDCA4-1
linc-ERICH1-4
RP11-711D18.2
RP11-395N3.1
CTB-113P19.4
linc-TEKT1-3
RP3-486I3.5
AC079988.3
SOS1-IT1
linc-ACSL3
RP11-126K1.8
RP11-375H19.2

RP5-1027O15.1
AP000688.15
RP11-822E23.7
LINC00398
RP5-1142A6.2
RP11-1252I4.2
RP11-527H14.2
linc-MUC20-8
linc-SEP15-7
AC114763.1
RP11-527F13.1
RP11-682N22.1
DIO2-AS1
RP5-908M14.5
RP11-555G19.1
AC005237.4
CTD-2275D24.4
linc-LAMA4-5
linc-OXGR1-1
AL132709.5
CTB-131B5.5
linc-SPRY2-2
RP11-154H12.3
linc-FAM150B-2
RP11-13G14.4
linc-CCRN4L-7
CTD-2256P15.5
linc-SRD5A2-2
RP11-242O24.3
linc-FAM174B
linc-PAPPA-4
RP11-805J14.5
linc-IL1F7
linc-PHF17-1
RP11-728E14.3
RP11-128P17.3
AC107057.1
AC016831.7
LINC00400
AC133528.2
RP11-388K12.1
RP11-71N10.1
RP11-568J23.6
RP11-25O3.1
RP11-26N15.2
RP11-363J20.2
LA16c-OS12.2
RP11-181K3.4
RP11-344A5.1
RP11-123K3.4

linc-ZHX2-1
linc-DGCR6-1
AL162759.1
AC073641.2
RP11-469H8.6
CTC-260E6.6
RP11-701P16.5
linc-GRHL2-8
linc-SLITRK5-14
RP3-508I15.19
RP11-817O13.6
FMR1-IT1
CTA-109P11.1
RP11-712L6.7
linc-TSPYL5
RP11-380D11.2
RP11-20J1.1
AC096655.2
RP11-66B24.1
linc-CCDC90A-4
RP3-434P1.6
RP11-672A2.3
linc-PTK2B
RP3-426I6.6
CTD-2019O4.1
RP11-5P4.2
linc-MSX2-5
RP11-121E16.1
linc-APOL5
GACAT1
RP11-303E16.5
RP11-66A2.2
RP11-716H6.2
RP11-466I1.1
RP3-449H6.1
linc-DCUN1D1
linc-CECR2-3
RP13-539F13.3
LINC00559
LINC00982
linc-KALRN-2
linc-BCL2A1-1
linc-THBS2-2
AC144835.1
RP11-152L20.3
C12orf80
linc-FAR2-2
RP11-92G19.2
linc-NUDT12-5
SLIT2-IT1

AC010967.2
CTC-518P12.6
CTC-458A3.8
linc-AKIRIN1-3
RP11-108E14.1
RP11-1260E13.1
RP1-231P7P.1
RP11-2C7.1
RP11-94H18.2
linc-SRP9-3
RP11-168G22.3
STARD13-AS
linc-RTL1-1
RP5-1024G6.2
RP11-44H4.1
AC092684.1
RP1-149C7.1
RP11-102N12.2
linc-LMAN1-2
AC002064.5
RP11-798L4.1
linc-ATP6AP2-6
CTD-3012A18.1
linc-ZNF98-1
RP11-182L21.5
RP11-665I14.1
linc-EVX2-2
RP5-968D22.3
linc-NTRK2-1
NRG1-IT3
linc-MLLT10-2
RP11-338N10.1
linc-EFHA2
RP11-23D24.2
RP1-251M9.2
RP4-529N6.1
RP11-141O15.1
RP11-83C7.2
linc-FLI1-5
linc-TANC2-1
ASMTL-AS1
RP5-963E22.5
linc-ADAM2-1
RP11-80F22.4
RP11-599B13.3
linc-CLDN24-2
RP11-809H16.2
RP5-1142A6.5
RP11-145M4.2
CTD-2382H12.2

linc-SSTR1-2
linc-DIMT1L
AC104699.1
AP000355.2
RP11-298J23.9
RP11-480O10.1
linc-ACTL7A-10
RP11-772C9.1
RP11-266L9.2
linc-CLLU1-7
SIGLECP3
AC006019.4
AC002472.11
linc-STIM2-4
LL22NC03-N27C7.1
linc-GPATCH2-3
linc-OR4C12-2
linc-CCDC140-1
CTD-2233C11.2
AC013402.3
linc-PSMG2
AC005789.11
RP5-978I12.1
linc-RSPH1-2
RP11-826N14.1
RP11-47L3.1
RP5-1096D14.3
RP11-171A24.3
linc-GRHPR
AC015849.13
CTB-37A13.1
RP11-88H9.2
RP5-899B16.1
CTC-273B12.6
linc-UTS2D-1
RP11-285A18.2
RP11-67A1.2
AP006216.5
RP11-128L5.1
RP11-1100L3.7
RP11-143A22.1
RP4-799P18.4
LINC00106
RP11-350N15.4
linc-SMARCA2-1
C21orf91-OT1
RP11-556O15.1
AC008281.1
AC084219.3
RP11-814P5.1

CTB-61M7.1
RP11-136K14.3
RP11-946L20.4
linc-GRHL2-1
RP4-714D9.2
linc-ZNF507-6
RP11-111J6.2
CTD-2278I10.4
RP11-80K6.2
AC092669.6
linc-ZNF322B-3
linc-POTED-6
RP11-414H23.3
linc-RNF219-1
TBC1D4-AS1
RP11-722M1.1
CTB-99A3.1
linc-MGAT5B-1
RP3-461P17.9
RP11-158K1.3
RP11-664H17.1
RP11-460H14.1
CTD-2382E5.2
FAM74A5
linc-RAB7A-2
linc-SSTR4-4
linc-CXorf64-2
RP1-149L1.1
RP11-6B19.2
RP11-1084I9.2
RP11-495O10.1
linc-TIMM22-1
AC004066.3
RP11-355F22.1
YEATS2-AS1
RP11-472K22.2
RP1-56K13.5
CTD-2134A5.3
linc-NOTCH1-1
RP11-467L20.10
RP11-662J14.1
RP11-677O4.2
RP11-358M11.3
linc-RBBP9-2
linc-FGD6-3
AC010731.4
CTA-392E5.1
linc-EXOSC9-1
RP11-769O8.2
RP11-34P13.14

RP11-632L2.1
RP4-788P17.1
SZT2-AS1
RP11-359N11.1
AF001550.7
LINC00606
RP11-332H17.1
RP11-216F19.2
AP004372.1
RP11-259O2.3
linc-TSPO2-3
RP11-711K1.7
RP11-307E17.8
linc-RPRM-7
RP11-397G17.1
LL22NC03-86D4.1
RP1-305B16.2
RP11-46C24.5
linc-TCTE3-8
linc-MIER3
linc-SEC24B-2
RP11-255P5.2
linc-PCDH8-10
XXbac-B33L19.6
RP11-223F20.2
CTD-2340D6.1
LINC00844
RP5-1172A22.1
RP11-708B6.2
GS1-393G12.12
LL22NC03-102D1.18
RP11-269G24.6
RP11-243E13.2
RP11-89H19.1
RP11-27M24.1
CTD-3051D23.1
linc-FBXO36-3
linc-KLF4-1
LINC00620
linc-ZBED4-3
RP11-45A12.2
linc-PTAR1-5
linc-ALDH1A1-6
KB-1930G5.4
RP11-235G24.3
RP11-476D10.1
AC064871.3
RP11-561O23.5
AC073343.13
RP11-168E17.1

linc-PFKP-2
linc-SLK-1
linc-MGAT4C-2
AC092661.1
LINC00163
AC092687.5
RP11-789C1.1
LINC00890
RP13-614K11.1
RP5-1097F14.3
RP11-834C11.6
CTD-2140G10.2
linc-ERICH1-1
AC093843.1
RP11-109A6.2
linc-C15orf41-2
RP4-771M4.3
linc-SULF2-4
CTD-2291D10.3
RP11-218C14.5
RP11-958J22.1
RP11-503G7.1
AC097467.2
linc-PPP1R3A-2
PPP2R2B-IT1
CTD-3229J4.1
RP11-44K6.2
RP13-259F12.2
linc-PRDM10
RP11-445F12.1
XXbac-BPG181B23.4
AC092415.1
RP11-151E14.1
RP11-149A7.2
RP11-1260E13.3
RP11-87M18.2
RP11-234O6.2
RP11-522N14.2
RP11-570L15.2
linc-GPR123-1
linc-KIF2B-1
RP11-810P12.5
LINC00856
AC009229.6
RP11-798K23.1
RP11-734I18.1
RP11-575L7.8
RP11-21A7A.3
linc-MARCKS-5
linc-IRX1-8

RP11-456D7.1
AC009487.5
RP11-477D19.2
linc-GTF2H2-4
linc-TYR-4
RP11-254F7.3
linc-E2F3
MIR145
RP11-755F10.1
linc-AGGF1-1
LINC00266-3
RP11-472G23.10
AP000146.2
RP11-6E9.5
RP11-10C24.2
LINC00905
RP5-1109J22.2
HMBOX1-IT1
linc-TNRC6C-1
RP11-272B17.1
RP11-282I1.1
RP11-117L5.4
RP11-165J3.5
RP11-298O21.3
RP11-273B19.2
ITPKB-AS1
linc-GAP43-1
RP11-44L9.1
linc-TMEM207-3
CTD-2275D24.2
CTD-2571L23.6
RP11-321E8.4
RP11-131K5.2
RP11-422J15.1
RP11-469N6.2
GS1-421I3.2
RP11-269F21.1
RP11-887P2.6
RP11-1072C15.4
RP11-216M21.5
HAR1B
linc-C6orf89
RP11-407A16.3
AC009120.10
RP11-413B19.2
linc-KLF6-7
RP11-189E14.3
RP11-475C16.2
RP11-676J15.1
RP11-489N22.3

RP11-386D6.2
linc-ACBD5-1
linc-POM121L12-1
RP11-442O18.1
RP11-531H8.1
RP11-867G23.1
RP11-44F21.5
RP5-1121H13.4
linc-TGDS-1
LINC00885
CTC-527H23.2
linc-DHX37-18
linc-KBTBD12-1
RP11-893F2.14
AC005519.4
RP11-31E13.2
XX-FW80269A6.1
RPS10P7
AC012074.2
RP11-75N4.2
RP11-304M2.3
RP11-489E7.1
RP11-168G16.2
RP11-261C10.4
linc-C9orf66-1
RP11-91A18.5
CTD-2377D24.4
RP11-255I10.2
CTD-2587H19.1
RP11-713M6.2
DIAPH3-AS2
CTD-2313F11.2
linc-TBX3-3
RP11-87P13.2
RP11-293N14.1
RP11-503P10.1
linc-SAAL1
linc-NEURL1B-3
KB-1208A12.3
linc-HERPUD2-2
RP11-958F21.3
LINC00551
LINC00966
RP5-906A24.2
RP11-18D7.2
linc-TCP10-4
RP11-26L16.1
AC073284.4
linc-ELFN1
CTC-301O7.4

RP11-56B16.5
RP11-159N11.4
RP11-164P12.4
RP11-355I22.2
RP11-451L19.1
AC062020.1
AP005273.1
linc-MMRN1-2
linc-JAM2-6
AC006445.8
RP11-410C4.5
RP11-279O17.3
RP11-344N17.8
linc-SERPINI1-4
AC016722.4
AC105344.2
RP11-860B13.3
RP11-843P14.2
linc-IRX4-1
linc-CEP120-1
RP11-404O13.5
SHANK2-AS2
RP13-379O24.3
RP11-788A4.1
RP11-498C9.13
RP11-494M8.4
RP11-282O18.7
CTC-525D6.1
LINC00333
MIR3179-2
CTB-174D11.1
AC007292.4
linc-CHRNA4
RP1-78O14.1
linc-PDE6B
linc-NRXN1-3
linc-CD180-4
RP11-404J23.1
RP11-597A11.2
linc-ANKRD10-7
linc-C14orf102-2
AC005523.2
RP11-400L8.2
AC010969.1
RP11-167N24.4
CTA-254O6.1
RP11-135L13.4
CTA-392C11.2
AC016903.1
CTB-191D16.1

AC007740.1
U47924.30
linc-TFPI-1
KB-1615E4.3
linc-WSCD2
MEG9
linc-RAB2A-2
linc-CDH6-11
linc-WDFY4
linc-CDH3-4
RP11-395P13.1
CTD-2196P11.2
RP5-894A10.2
RP11-353N14.2
RP11-356K23.1
RP11-654C22.2
RP5-860P4.2
RP11-728F11.3
RP11-108K3.2
RP11-218E20.5
XXbac-BPG254F23.6
RP11-229P13.22
RP11-646E18.4
RP11-280H21.1
RP11-589P10.5
RP11-325B23.2
RP11-653G8.2
RP11-17M15.1
linc-CCDC122-3
linc-NOVA1-7
ATP13A5-AS1
TAB3-AS2
AC009518.8
RP11-126O1.2
linc-RNASE3
RP11-6C14.1
RP5-1022P6.5
RP11-6L6.3
linc-KIN-2
linc-C3orf39
linc-HAUS5
AC004941.3
RP11-1C8.6
AATK-AS1
RP11-428C19.5
RP11-271F18.1
linc-FRG1-7
RP11-273B19.1
linc-ZDHHC6-2
linc-LYPLAL1

CTB-36H16.2
AC104534.2
RP4-742N3.1
RP11-527L4.5
linc-PROC-4
linc-GBE1-7
linc-FOXC1-1
linc-FOXG1-7
RP11-488C13.5
RP11-63P12.7
RP11-108M9.1
linc-CPEB4-4
RP11-388M20.9
linc-ISX-2
linc-ZCCHC17-1
RP11-796E10.1
AC005358.3
RP1-45N11.1
linc-CTNND2-3
RP11-616M22.5
linc-WASF3
ZMYM4-AS1
CTD-3073N11.9
linc-OR11A1
RP11-3G20.1
RP11-733O18.1
linc-MRPS33-1
AP000266.7
RP11-732M18.2
RP11-309M23.1
linc-CARD11-9
RP11-161I6.2
RP11-415C15.1
RP11-114L10.2
CTD-3105H18.7
RP11-76G10.1
AC026150.8
CTD-2201G3.1
AC073218.3
RP11-265D17.2
linc-AKR1E2-14
linc-PCSK5-2
AC113607.2
RP11-327I22.8
linc-RFTN1
RP11-1096G20.5
AC099668.5
linc-UNC13C-5
PDX1-AS1
CTD-2033D15.1

RP11-766F14.1
linc-TXLNB-2
CTD-2168K21.2
RP11-1038A11.2
RP11-777N19.1
RP11-617B3.2
RP11-378A12.1
linc-PRELP
AP001625.5
linc-VWC2-1
linc-RGSL1-3
AC007879.1
AC023115.2
linc-REST
linc-EPHA6-1
RP11-507K2.2
linc-SLCO3A1-1
ATP1B3-AS1
RP11-31F19.1
RP11-510M2.10
linc-DNAH8-2
RP3-337H4.10
linc-TRAPPC1
linc-GATA6
linc-C17orf97-2
RP11-754B17.1
RP5-1086K13.1
RP11-330A16.1
AC079776.3
linc-ELAC2-2
CTC-550B14.8
RP11-467I20.2
AC006481.1
linc-ARFGEF2-2
AC026471.6
RP11-157E14.1
linc-FAM155B
LA16c-4G1.3
RP13-137A17.5
RP11-1094M14.12
CTD-2524L6.3
RP11-302I18.3
RP11-167P22.3
linc-COL5A1-5
RP11-16P6.1
RP11-506B6.6
AC072062.3
RP11-983C2.3
linc-ZP4-2
AC062032.1

CTD-3051D23.3
RP11-443G13.2
RP11-382A20.4
RP11-496N12.9
RP11-445L6.3
RP11-125D12.2
RP5-1125N11.1
RP11-506N2.1
RP13-212L9.1
AC135178.7
SOX21-AS1
CXorf51A
RP11-374P20.4
RP11-515E23.2
RP11-286E11.2
RP5-928E24.2
RP11-336K24.5
RP11-473M20.5
RP11-29B2.5
CTD-2532K18.1
CTC-286N12.1
DISC1-IT1
RP11-328J14.1
linc-YPEL5-1
GS1-542M4.4
RP11-231L11.1
RP11-401H2.1
CTD-3099C6.7
AC093627.11
RP1-118J21.25
linc-NOL4-3
RP11-102L12.2
RP11-270M14.5
linc-CNTNAP5-6
RP11-154B12.3
AP000477.2
RP11-19J5.2
RP11-301L8.2
linc-VWA5B1-2
RP11-171N4.3
CTD-2620I22.7
linc-CCND2
RP11-355F16.1
RP11-324J3.1
linc-NR2F2-5
WI2-81516E3.1
RP11-543D10.2
RP11-96C23.14
linc-SYT4-2
RP11-882G5.1

linc-SAFB-2
linc-DYX1C1
RP11-172E10.1
linc-ZNF681-7
linc-FBXO33-5
linc-DBT-2
linc-UBLCP1-3
linc-OPCML
AC006000.5
RP11-605F20.1
RP11-109E24.1
RP11-21J7.1
RP11-45I20.1
RP11-597M12.1
RP11-705O24.2
RP11-123H22.1
linc-LHFP
RP11-532N4.2
RP3-468B3.3
linc-CCR8-2
linc-C9orf66-6
AC013269.4
linc-LIN28B-2
RP11-1028N23.4
LMLN-AS1
RP3-327A19.5
RP11-154H17.1
CTC-493L21.2
CTD-3020H12.4
RP11-349I1.2
RP13-612N21.1
linc-TYRP1-4
AC007652.1
FBXO36-IT1
RP11-151D14.1
linc-AKR1E2-5
REV3L-IT1
RP11-498E2.9
RP11-22A3.1
AC009299.2
linc-CAP2
COL4A2-AS1
RP11-465L10.10
CTC-439O9.3
AF131215.6
RP1-67A8.3
AC004562.1
AC006380.3
linc-CGNL1-1
linc-NTS

AC002511.1
RP11-1008C21.1
RP11-389J22.3
RP5-973N23.4
RP11-406O23.2
LINC00301
RP11-810P8.1
RP11-526K17.2
linc-SHPRH-9
RP11-7M8.2
linc-NEIL3-3
RP11-813I20.2
linc-PRH2-2
RP11-757F18.5
LINC00972
RP11-849I19.1
LL22NC03-104C7.1
RP11-30K9.5
LYST-AS1
RP11-493L12.3
linc-FGFR1OP-1
HOXB-AS5
linc-TMEM132B-1
RP11-218M11.3
OSTM1-AS1
LINC00671
RP4-740C4.7
RP1-111C20.3
CTC-535M15.2
CTD-2350J17.1
RP11-122D10.1
linc-SOX8
RP11-23E10.3
RP13-46H24.1
linc-GREB1L-1
linc-ZFHX3-3
linc-VWC2-4
CTD-2118P12.1
RP11-436H11.2
DTX2P1-UPK3BP1-PMS2P11
RP11-1080G15.1
RP11-423G4.7
RP11-1149M10.1
linc-RAB6C-2
linc-RAD21-5
linc-MEOX1
RP11-554D14.7
linc-BAI3-5
RP11-75L1.1
RP11-406H23.2

RP5-1024N4.2
linc-XPC-2
RP11-309N24.1
CTC-573M9.1
RP11-533E19.2
RP11-265P11.1
linc-MFAP3L
XX-DJ76P10__A.2
AC007193.9
CTC-360G5.9
NUCB1-AS1
CACNA1C-IT2
linc-CDH5-9
LINC00362
RP5-1050E16.1
RP11-425E13.1
RP11-557H15.3
RP11-86H7.6
RP11-646J21.4
RASA2-IT1
RP11-264C15.2
AP001065.15
RP11-506E9.3
linc-KIAA1712-5
RP11-3G21.1
linc-TMEM132C-3
linc-TRIM52
linc-KIAA0427-3
LINC00678
PADI6
RP1-240B8.3
linc-ZNF717-1
RP11-556O9.2
linc-CXADR-5
AC010641.1
RP11-192P3.4
CTC-453G23.4
RP11-89C3.3
linc-RAD23B-1
RP11-495O11.1
RP11-473E2.3
linc-C18orf55-7
KB-1958F4.1
linc-CHL1-2
linc-SULF2-9
RP11-552F3.4
linc-EFCAB4B
linc-HEG1
AC069394.1
RP11-446F17.3

linc-TMEM90B-5
RP11-100M12.3
AC104655.3
RP11-436I24.1
RP11-227B21.2
RP1-151B14.6
linc-CLEC2D-4
RP11-157B13.7
RP11-100L22.1
CTD-2574D22.4
CTD-2247C11.1
LINC00378
RP11-227H15.4
RP11-266E14.1
RP11-196H14.2
AC026167.1
CTC-479C5.16
RP11-368N21.4
RP11-1186N24.5
CTD-3032J10.4
RP11-289F5.1
linc-TCEAL2
RP4-781K5.6
RP11-748L13.6
RP3-340N1.2
linc-CBWD3-4
RP11-376O6.2
linc-PSMA8-4
linc-DNAH6-4
CTD-3032J10.2
RP13-238F13.3
RP11-26O3.1
linc-ARHGEF11
RP11-428L9.2
RP1-302D9.1
linc-UNC5B-1
RP11-14O19.1
CTD-2154B17.1
linc-SEMA6C
linc-KIAA0232
linc-LRRC4-1
linc-PMFBP1-3
RP11-100F15.2
CTB-91J4.1
RP11-963H4.3
RP11-78O22.1
RP11-19N8.4
RP11-307L14.2
linc-SLC10A2-3
RP11-881M11.8

linc-CRISPLD2
RP11-879F14.1
linc-RGL4-6
RP11-618K13.2
RP11-492E3.51
LINC00849
RP11-863P13.5
linc-PITRM1-1
CACNA1G-AS1
CTC-425O23.2
RP4-655L22.2
RP11-536I6.2
linc-GNB2
CTC-379B2.4
linc-NTM-4
LINC00642
RP11-344N10.2
CTD-2532D12.4
TCEAL3-AS1
linc-IL6-2
FOXN3-AS2
linc-STOX2
ZNF630-AS1
AC024704.2
AC005152.3
RP11-141M1.1
RP11-909M7.3
RP11-43A14.1
AC108051.1
linc-CD99-9
RP11-25I9.2
ZNF385D-AS2
RP1-209A6.1
linc-OBSL1-4
RP11-690C23.2
AC140481.8
RP4-736H5.3
CTD-2091N23.1
RP3-355L5.5
RP1-213J1P__B.1
RP11-298D21.2
RP11-775H9.2
Z69666.2
RP11-99L13.1
5S_rRNA
AC006372.5
linc-OR14J1-2
RP11-1070N10.4
RP11-638I2.6
RP11-96O20.2

linc-ZNF366-4
linc-MYOT-4
RP11-883G14.4
RP11-1105O14.1
RP11-122C21.1
linc-SATB1-1
CTD-2561B21.10
RP3-380B4.1
SRGAP3-AS1
RP11-583F24.8
CTD-2383I20.1
RP11-296A18.6
RP11-675F6.3
CTD-2339F6.1
linc-CD9-2
RP11-167J8.3
AC003664.1
RP11-334E6.3
RP4-782L23.1
RP11-12M5.1
RP11-463O9.9
RP11-669M16.1
linc-NTSR2-3
linc-EIF5
RP11-624A4.1
linc-CR2-1
CTD-2090I13.1
AC009878.2
RP11-209D14.4
linc-KIAA0146-1
RP11-618G20.1
linc-KIF13B
RP11-559N14.5
RP11-89K21.1
RP1-4G17.2
RP5-955M13.3
AC022201.4
CTD-2540M10.1
RP11-90K17.2
linc-FRG2C-1
AC016723.4
linc-NR2E3-2
RP11-388M20.2
RP11-178L8.8
RP11-90C4.3
RP11-103C16.2
RP11-710F7.3
PLCH1-AS1
linc-GNE
AC104777.2

AC009262.2
RP11-248N22.2
linc-HEATR2-5
RP11-356M20.3
linc-PROKR2-4
CTC-462L7.1
CTD-2085J24.3
linc-FPR1
linc-API5-7
CTB-167B5.1
linc-NPVF-2
TBL1XR1-AS1
GS1-21A4.1
RP11-202D18.2
RP11-692P14.1
RP11-300M24.1
linc-ACTL7A-8
linc-AUTS2-1
NCAM1-AS1
AC104820.2
linc-OAF-2
AE000661.50
linc-CCL25-1
linc-CDKN2C-4
RP11-776A13.3
RP11-645N11.3
RP3-400B16.3
RP11-375B1.3
linc-P2RY1-2
AC007405.8
RP13-152O15.5
CTD-2232E5.2
LINC00477
linc-BASP1-1
RP11-214F16.4
linc-ZFAT-3
AP001464.4
AC142293.3
AC019185.4
linc-UBASH3B-4
RP11-166B2.3
linc-MYL2-4
CTC-360P9.2
RP11-555F9.2
linc-TMEM156
linc-C1orf192
linc-RCC2-2
linc-FGFR1OP-8
linc-ZNF99-7
RP11-63E5.8

RP11-449J10.1
linc-SYT1-1
RP11-379F4.7
RP4-718N17.2
CTD-3222D19.11
RP11-48B3.4
linc-PCBD1-2
RP11-250B2.5
linc-HSF2-1
RP11-320P7.2
RP1-127C7.6
RP11-541F9.1
linc-GMPS-3
CTD-2298J14.2
RP3-462D8.2
AC005537.2
LINC00681
RP4-612J11.1
CTD-2140B24.6
linc-SIP1-1
linc-C1orf151-1
linc-SEC61G-3
AC018685.2
LINC00534
linc-GMPR
RP11-655M14.13
RP11-296E23.2
AC006159.3
RP11-260O18.1
linc-COL6A6
CTD-2350C19.1
RP4-726F1.1
RP11-73C9.1
linc-MPPED2-1
AC009404.2
RP11-173P15.5
RP11-618M23.4
AC017002.4
linc-MAF-3
RP11-335O4.1
RP11-64P14.7
AC092198.1
RP11-358D14.2
RP11-22H5.2
AC098823.3
linc-DTHD1-9
linc-OSBPL8
RP11-146N23.4
linc-ANKRD20A1-11
linc-CIDEA-3

RP11-231E4.2
RP4-601P9.2
linc-UFM1-3
RP11-478C19.2
linc-TMEM132E-2
AC007395.3
AJ239322.1
AP000783.2
PHKA1-AS1
linc-ARHGAP20-3
linc-FGF9-4
RP11-53B2.2
RP11-126K15.1
linc-IBTK
linc-CD300LB
RP11-670E13.2
RP11-296L22.8
CTD-2357A8.3
LINC00410
AC005224.2
RP11-280K24.1
MIR202
linc-ADCY2-4
RP5-940J5.8
RP11-206L10.5
linc-UMOD
RP11-320M16.2
RP11-1070N10.6
AC091493.2
linc-MORF4L1-2
linc-RHAG
RP11-500M8.4
linc-FYN
linc-PCDH8-2
RP11-364L4.3
AC000124.1
RP11-575A19.2
RP11-744I24.3
RP11-573G6.6
RP1-111D6.2
RP11-565A3.2
CTD-2130F23.1
SCGB1B2P
CTD-2589H19.6
RP11-1079K10.5
linc-COL13A1
RP11-174I12.2
linc-ARNT2
CTD-2333M24.1
RP11-678G15.1

RP1-230L10.1
RP3-462E2.3
RP11-96D1.5
linc-PPIL5-2
GS1-174L6.4
linc-GALNTL4
CTA-363E6.7
linc-PCGF5-3
linc-OLIG2-6
RP11-115J16.3
GS1-600G8.5
linc-SALL3-3
RP11-203P2.2
SPTY2D1-AS1
AC018890.6
RP11-895K13.2
linc-EFHA1-1
CTD-2184D3.6
RP11-139E24.1
RP11-342D14.1
CTD-2507G9.1
AC012358.8
LINC00353
linc-NETO1-2
CASK-AS1
GRIP2
AP001627.1
RP11-257I8.2
AC073133.2
RP11-5P18.5
AC073869.19
AC004901.1
AC009014.3
linc-MGST2
linc-VSNL1-1
CTD-2587M23.1
RP11-909M7.5
LINC01685
PRKAG2-AS1
LINC02390
RP11-553L6.2
PCAT19
TMC3-AS1
RP3-416J7.6
RP11-664I21.6
SCAT1
LINC01163
C2CD4D-AS1
RP3-528L19.1
LINC01679

RUNDC3A-AS1
LINC01270
DNAH17-AS1
MIR3945HG
RP11-615I2.2
FMNL1-DT
CNNM3-DT
RP11-705C15.5
RP11-667K14.4
DDN-AS1
RP1-261G23.7
RP11-109G23.3
RP11-419K12.2
LINC01206
CORO1A-AS1
LINC02754
RP11-80I3.1
RP11-796E2.4
RP11-155G14.6
RP1-134E15.3
RP11-390F4.3
HEXD-IT1
RP3-359N14.3
RP3-395M20.12
RP11-617F23.2
NABP1-OT1
RP11-1105G2.3
KIF1C-AS1
RP11-134G8.7
NAPA-AS1
LINC01393
RP11-174M15.1
ZBTB44-DT
LINC02273
LINC01094
SBNO1-AS1
LINC01480
RP11-253D19.4
LEMD1-DT
KB-836E9.1
RP11-734K23.9
TAT-AS1
LINC02408
FLICR
RP11-342I1.3
CTD-2396E7.11
RP11-758N13.1
MDS2
LINC01281
AATBC

RP11-137H2.4
LINC01801
EML2-AS1
RP5-1180E21.5
RP11-750H9.5
LINC01882
HCP5
AC009495.2
RP11-1070N10.3
LINC02732
LINC01150
RP11-57K16.1
DUXAP8
LINC01127
RP11-543C4.1
FILNC1
RP1-313I6.12
RP11-284F21.7
RP11-622O11.6
LINC02257
LNCDAT
PDXP-DT
RP11-532F6.3
RP11-1149O23.3
RP6-91H8.3
GS1-57L11.3
VPS9D1-AS1
RP11-345F18.2
RP1-46C2.1
ITGA6-AS1
LINC02158
RP11-681B3.4
IL6R-AS1
RP11-549D23.1
FUT8-AS1
RP11-83N9.6
LINC01506
GTSCR1
RP5-906C1.1
RP11-1191J2.5
CTD-2284J15.1
LINC02091
RP4-568C11.4
CH17-112M04.1
RP5-1171I10.5
CTD-2360A17.3
LINC02427
RP11-118B22.2
UBR5-AS1
RP11-689B22.2

LINC00528
NARF-AS2
LINC02481
RP13-714J12.1
NUP153-AS1
RP11-496H1.4
LINC02694
LLNLR-470E3.1
COA6-AS1
LINC02132
FAM153CP
RP11-326C3.2
NR2F1-AS1
CTA-384D8.34
RP11-426C22.4
RP11-534K14.5
LINC01637
CTD-2035E11.3
RP11-357H14.17
LL22NC03-N95F10.1
CTC-273B12.13
LINC01010
MMP25-AS1
RP11-1136L8.3
LINC01891
RP11-104L21.3
LHFPL3-AS2
LINC01426
RP11-394G3.3
RP11-153P14.1
RP11-495F22.1
PRKCQ-AS1
LINC02126
CH507-528H12.1
LY6E-DT
RP11-80H8.4
LINC02397
RP11-202G18.1
RP11-385M4.5
AC104530.1
LINC01857
RP11-111G13.1
XXbac-BPG181B23.9
SLC5A4-AS1
RP11-160E2.19
RP11-157L14.6
LINC02325
AC021188.4
LINC01237
RP4-620F22.3

RP13-126P21.2
RP11-622I12.1
KLRK1-AS1
GABPB1-AS1
ATP2B1-AS1
CTD-2561B21.11
LINC00324
LINC01226
XX-C00717C00720L.1
CFLAR-AS1
C3orf36
CITF22-49E9.3
LINC00824
RP11-1399P15.1
AC022182.1
RP3-508I15.21
RP11-196B3.5
LINC02362
PDE2A-AS2
RALGPS2-AS1
AC006538.8
RP11-495L19.1
RP11-102L12.3
RP1-147M19.2
FLJ16779
SEMA6A-AS2
C12orf77
PEG13
RP11-517H2.6
ECE1-AS1
RP11-545E17.3
CTA-246H3.13
LINC01724
LINC01871
RP11-438D8.6
RP11-512C24.4
C22orf24
RP11-96H19.1
RP11-50I19.2
RP11-416N2.4
CTD-3203P2.3
LINC02642
LINC02798
SFTA1P
RP11-33I11.4
RP5-1166H10.4
RP11-345F18.3
RP11-29A19.3
RP11-214O1.2
RP11-520A21.2

LINC00540
AC000082.4
CLLU1-AS1
CCDC183-AS1
RP11-339H12.1
RP11-197N18.2
RERE-AS1
AF196972.9
WAKMAR2
ELFN1-AS1
LINC01484
RP5-820A21.2
AFAP1-AS1
C6orf223
LUCAT1
DLGAP4-AS1
CCL3-AS1
TNFRSF14-AS1
RP11-390P2.4
RP11-121A8.1
RP11-66B24.2
LINC02413
LINC00649
RP11-744N12.3
KLHDC7B-DT
RP11-326K13.5
SLC25A25-AS1
XXYLT1-AS2
RP11-358B23.7
C6orf99
RP11-484K9.4
RP11-348J12.6
LINC02207
TTC39C-AS1
MIR3150BHG
RP11-452K12.4
LIX1-AS1
RP11-15A1.3
MIR3142HG
RP11-517H2.8
LINC01781
HOXA11-AS
SUGCT-AS1
RP1-97D16.9
LINC02577
RP11-542M13.4
RP1-236J16.3
TSPOAP1-AS1
RP11-68I3.4
PRR34

L3MBTL2-AS1
RP11-730K11.1
HECW2-AS1
RP11-413N10.3
RP11-63G10.4
LINC01422
RP5-1184F4.7
RP11-946P6.6
LINC00707
LACTB2-AS1
RP11-363D14.1
LINC02805
MIR223HG
KB-1440D3.16
RP11-27J8.3
CTA-373H7.7
SPAG5-AS1
LINC01705
RP11-567P19.1
LINC01735
RP11-323P17.2
LINC02446
RUNX3-AS1
CTD-2587H24.14
CH17-67K23.2
TSBP1-AS1
RP11-726G1.2
LINC02829
SATB1-AS1
RP4-673M15.1
CTD-2313J17.6
LINC01539
RP11-329B9.4
RP11-568J23.8
FLJ40194
AC058791.1
LINC00706
RP11-482G13.1
RP11-861A13.2
RP11-572B2.1
LINC02870
SPINT1-AS1
RP11-422P24.13
C9orf139
TMPO-AS1
USP7-AS1
AC003104.1
NRIR
PRR29-AS1
RFX5-AS1

LL22NC03-N14H11.1
RP11-256B12.2
LINC02457
AC005083.1
ZMIZ1-AS1
LINC01055
AC074289.1
CTC-428G20.6
DOCK8-AS1
LINC00239
LINC01727
RP11-81H14.1
PSMB8-AS1
CTA-414D7.1
RP11-643E20.1
CTD-2201E18.8
LINC01800
CEP250-AS1
RP11-483P21.2
LINC01914
RP11-293M10.6
ANKRD44-AS1
LINC01638
LINC01353
MAP3K14-AS1
LINC00900
RP5-1142A6.10
RP11-567J20.3
RP11-245D16.4
LINC02391
RP3-413H6.3
RP11-316M20.3
FAM226B
DPP4-DT
LINC01684
LINC01678
RP11-429E11.3
CTC-378H22.1
RP4-644F6.4
LINC02812
DRAIC
GRAPLDR
RP11-114N1.1
CTD-2540L5.6
AC004540.5
LINC01962
RP5-841K13.1
CTD-3214H19.6
PRNCR1
AC112721.2

RP11-216B13.1	
DARS1-AS1	
RP5-1086D14.6	
LINC00539	
RP11-363K21.1	
RP11-281P23.3	UP.TXT
RP3-383B8.1	
RP11-1223D19.3	
CTD-2325P2.5	
LINC01013	
CTD-2369P2.8	
RP4-671O14.6	
LINC01772	
OBSCN-AS1	
CTD-2536I1.3	
RP3-333H23.10	
LL21NC02-1C16.2	
RP11-655C2.4	
RP11-321P16.3	
RP11-127I20.8	
RP13-1016M1.2	
LINC01116	
LINC01136	
TRG-AS1	
LINC01614	
EWSAT1	
RP11-25K19.1	
LINC01176	
STXBP5-AS1	
AP001059.7	
LINC02785	
NCK1-DT	
LLNLR-263F3.1	
RP11-1143G9.5	
SENCR	
RP11-323N12.6	
CH507-513H4.1	
FAM30A	
CH17-264L24.2	
UICLM	
LINCR-0001	
LINC01588	
LINC02014	
LINC01060	
LINC02604	
RP11-116G8.8	
LINC01991	
LINC02723	
HSD11B1-AS1	
LINC02804	

CCDC18-AS1
MIR9-1HG
LINC00920
HELLPAR
LINC02328
RP11-379K17.4
LINC01215
RP11-44M6.7
LINC02009
RP11-902B17.1
AC093818.1
LBX2-AS1
CARD8-AS1
ADAMTSL4-AS1
LINC02611
C22orf34
MIR646HG
RP11-706O15.1
RP11-236P2.2
XXyac-YR21IF11.3
RP11-667K14.20
MIR4432HG
RP11-191L9.4
RP11-16E23.5
RP3-470L22.2
RP11-830F9.6
RP11-102N12.3
RP11-231C14.7
LNCARSR
CH507-254M2.2
RP11-206F17.3
LINC02541
SOCAR
RP1-167A14.3
BISPR
LINC01876
RP11-413G22.3
LINC01410
RPH3AL-AS1
RBM26-AS1
CTD-3222D19.5
FENDRR
RP11-496H1.3
LINC01579
CTA-445C9.17
GP6-AS1
TNK2-AS1
RP11-345P4.12
AC009133.12
LEF1-AS1

RP3-431P23.5
AC002456.2
CTD-2301A4.6
TFAP2E-AS1
LINC02605
RP11-537P24.1
RP11-499F19.3
LMNTD2-AS1
CTD-2003C8.2
LINC02422
MIR222HG
RP11-761I4.6
WDR86-AS1
KIAA0087
MIATNB
RP1-240K6.4
FLJ31104
LERFS
LINC02416
LINC00921
PTPRN2-AS1
RP3-455J7.4
RP11-125O18.2
RP11-328C17.2
KB-1471A8.1
LINC01694
LINC01126
RP11-115D10.1
CTD-2154I11.2
LINC01012
RBM38-AS1
AC025442.4
RP11-785D18.3
CHMP1B-AS1
CTB-85C5.1
RP11-283G6.5
LINC00467
CERNA2
LINC02728
AC096579.15
RP5-1028K7.2
CSTF3-DT
RP3-467K16.4
LINC02384
CTD-2547L24.3
RP11-474P2.6
MIR210HG
SLFN12L
RP11-388C12.1
RP11-637A17.2

RP11-686F15.4
CATIP-AS1
PIK3CD-AS1
FAM201A
IL6-AS1
LINC01624
RP11-506H21.6
C7orf69
AC129492.6
LINC01934
RP11-231C14.10
LINC00173
AC007255.8
LINC02648
RP11-340F14.6
MATN1-AS1
LINC02086
CDKN2B-AS1
LINC02361
RP11-596C23.2
RP1-244F24.1
LINC01670
RP11-262H14.14
RP11-405M12.4
LINC01572
MMP2-AS1
LINC02576
LNCAROD
AC026202.3
PGM5P4-AS1
USP30-AS1
RP11-93B14.10
RP11-360L9.9
LINC02452
RP11-399B17.2
RP11-247C2.2
HID1-AS1
RP11-134L10.2
RP11-80K21.4
RP11-27J8.4
RP11-147L13.14
RP11-339H12.2
MIAT
EML4-AS1
RP11-48G14.3
RP11-426C22.5
LRRC8D-DT
DTNB-AS1
LINC01036
KB-1615E4.2

PDCD4-AS1
PTCSC1
MROCKI
RP1-118J21.27
LINC01305
LINC00996
WI2-87327B8.2
PARP11-AS1
MHENCR
RP11-683I7.1
RP11-335J9.1
RP11-9N12.2
LINC01096
SLC12A5-AS1
MIR155HG
AC124789.1
LINC02345
RP11-53I6.2
PYCARD-AS1
RP11-888D10.4
RP11-383J24.1
AC000068.5
AC023590.1
LINC02245
KB-1572B10.1
RP11-568N6.1
ADPGK-AS1
LINC01811
RP11-81A1.10
THBS3-AS1
RP11-433N2.1
RP11-93B14.9
RP5-1120P11.3
PVT1
GAPLINC
C20orf197
COPDA1
LINC01238
LINC01943
LINC02324
RNF32-AS1
LINC01806
BOLA3-AS1
RP11-77P6.2
CSGALNACT2-DT
RP11-471B22.3
RP11-104L21.4
PACERR
RP11-230C9.2
RP11-10K16.1

PRECSIT
CYP2U1-AS1
DACT3-AS1
RP11-318N11.1
LINC01600
RP11-403A3.3
RNF217-AS1
RP13-631K18.5
RP11-9G1.3
RP11-11M20.4
RP11-427E2.1
RP11-566K19.6
SCN1A-AS1
CTD-2013N24.2
DLG5-AS1
RP11-284H19.1
RP11-346C20.4
RP11-125C10.1
RP11-1275H24.1
MBNL1-AS1
LINC01354
RP11-125E1.2
MAST4-AS1
PPP1R9A-AS1
CTA-212D2.2
USP27X-DT
LINC01990
RP1-158P9.2
SH3RF3-AS1
KRT7-AS
RP4-785P20.3
RP11-435J9.2
RP1-206D15.6
RP11-49N14.10
RP11-59C5.3
MIR100HG
AC137932.6
RP5-855D21.2
LINC01844
SCTR-AS1
LL21NC02-21A1.1
AC004637.1
RP11-325L12.6
MIR193BHG
RP11-96C23.13
MIR325HG
RP11-116G8.7
CARMAL
RP11-92A5.2
TWSG1-DT

AC093673.5
SRD5A3-AS1
AC005301.9
MIR1-1HG
NQO1-DT
TMEM92-AS1
RP11-316O14.1
PRDM16-DT
RP1-137D17.3
RP11-495L18.2
NRAV
TBC1D8-AS1
KB-2042G5.1
BVES-AS1
LINC01515
RP4-613B23.8
AP000318.2
KCNK15-AS1
U47924.31
LINC01936
LINC02102
SLC25A34-AS1
FGF14-AS2
FAM198B-AS1
ZNRF3-AS1
ZFPM2-AS1
LLNLR-304G9.1
RP11-973D8.5
RP11-650K20.3
LINC00652
C1orf229
LNC-LBCS
RP11-622C24.2
LINC02716
RUFY1-AS1
SPRY4-AS1
RP11-983P16.4
RP11-195F19.30
LINC00165
AC007563.5
LLNLR-271E8.1
RP4-781B1.5
SMIM2-AS1
RP11-924A14.2
HCG15
RP11-173C1.1
DIRC3-AS1
AC007952.6
PICART1
ATP6V1B1-AS1

CTD-2012K14.8
CTD-2523D13.2
NAV2-AS6
RP11-421J10.1
WI2-89031B12.1
ARMCX3-AS1
SLC2A1-AS1
CTD-2506P8.6
RP11-1212A22.9
LGALSL-DT
MAP2K5-DT
RP11-472K17.4
LINC00857
RP11-899L11.4
RP3-454M7.3
LINC02104
LINC01123
RP11-114G11.5
GAS6-DT
AC016738.4
LINC01852
LINC00327
RP11-156G14.7
RP11-332H18.5
RP11-696N14.1
RP4-680D5.2
RP11-401P9.5
RP11-272L14.3
LINC01016
RP11-412D9.4
RP11-304C12.5
LINC01748
LINC02762
ZNF516-AS1
SAMMSON
XXbac-BPGBPG34I8.5
C8orf34-AS1
RP11-109D24.2
RP11-778D9.13
RP11-702N8.3
FZD4-DT
ARHGEF26-AS1
MPPED2-AS1
LINC01186
RP11-800A3.7
RP5-1116C7.2
FOXN3-AS1
CTD-2207P18.2
LINC02696
ITPR1-DT

RP5-875H18.9
AC007405.6
DPH6-DT
CRNDE
MSC-AS1
TTC23L-AS1
CNN3-DT
MRPS30-DT
LINC02725
PLS3-AS1
LINC01752
JAZF1-AS1
EDIL3-DT
PRRT3-AS1
ADAMTS9-AS2
RP11-177H13.2
LASTR
LINC00472
FHAD1-AS1
LINC01106
RP11-107N15.1
LINC02872
UNC5C-AS1
RP11-420L9.5
MEIS1-AS2
SLC25A21-AS1
RP11-758H9.2
RP11-479O17.12
KIRREL1-IT1
AC007405.4
LINC01715
RP1-150O5.4
RP4-785P20.2
RP11-539G18.4
RP11-744A16.4
B4GALT1-AS1
RP11-315H12.3
APCDD1L-DT
RP11-400K9.4
LINC01151
RP11-221G19.1
LINC01449
RP13-349O20.2
RP11-69M11.1
RP11-446H18.6
RP3-495O10.5
LINC01278
RP11-403A3.2
LINC02649
RP11-669I1.2

KB-1410C5.5
RP11-680F8.3
MIR497HG
RP4-737E23.7
RP11-59H7.4
RP11-879F14.2
RP11-976B16.1
LINC02701
RP11-755H23.1
LINC02664
RP11-371C18.3
RP1-140K8.5
LINC02767
RP11-346M10.3
FRMD6-AS1
USP3-AS1
RP11-16N11.2
TBX2-AS1
RP11-489G11.4
LINC02861
RP11-58A18.2
RP11-111J6.4
RP11-285J16.1
CMB9-94B1.2
RP11-529J17.6
LINC01738
CTD-2292N2.1
RP11-1275H24.2
LINC01615
LOXL1-AS1
RP11-115H13.1
RP11-107G24.4
LINC01285
RP11-145A3.1
RP11-701H24.4
RBPMS-AS1
LINC01447
RP3-509L4.5
CARMN
RP11-686O6.2
RP11-111N20.1
RP11-135M8.1
RP11-571L19.8
RP11-21L23.3
PCAT7
ZNF503-AS2
LINC02351
RP11-279O9.4
RP11-1167A19.16
CH17-55D9.1

BCDIN3D-AS1
CACTIN-AS1
CTD-2530H12.4
RP11-483C6.1
CTC-435M10.13
C5orf38
RP11-399K21.14
LL0XNC01-246D9.1
CASC18
HRAT92
CCND2-AS1
RP11-205A6.1
LINC01266
RP4-535H23.3
PRKCZ-AS1
RP11-96C23.5
NFIA-AS2
LINC02188
RP11-29A19.2
CTA-521C9.1
RP11-230F18.5
THRB-AS1
RP3-347H13.12
RP11-190A12.10
CTC-308K20.1
LINC00632
LINC02600
CTB-58E17.5
DNAH10OS
RP11-105N14.1
CH507-145C22.4
BX470102.3
LINC02875
RP3-325F22.5
RP11-536K17.2
RP11-893F2.5
PRR34-AS1
XXbac-BPG252P9.9
RP11-383I23.2
LINC02849
CERS3-AS1
RP11-816J6.3
RP11-56L13.8
ZNF295-AS1
AC084219.4
GATA6-AS1
CEROX1
RP11-77K12.9
LINC02269
RP11-635F12.1

CTB-140J7.3

DOWN.txt

ıding in GO term response to ER estress (GO:0034976).

lncRNA downregulated in AAA (112 elements)
AC097724.3
RP11-311F12.1
ARHGEF7-AS2
RP11-1134I14.8
RP11-281O15.4
RP11-51J9.6
CTD-2310F14.1
CTD-2026K11.1
AC079779.4
AC068831.15
RP11-252K23.2
SACS-AS1
AF186192.1
RP11-626H12.1
RP11-861E21.2
RP11-1336O20.2
RP11-307B6.3
FGF13-AS1
RP11-305F18.1
RP11-71H17.7
RP11-795J1.1
RP11-731J8.2
RP11-405A12.2
SLC26A4-AS1
ALDH1L1-AS2
LINC00310
RP11-22L13.1
RP11-71E19.1
LINC00670
RP11-867G23.10
RP11-63M22.2
AC003986.6
SPANXA2-OT1
RP11-1081M5.2
RP4-584D14.7
FAM181A-AS1
KB-1448A5.1
RP11-545A16.4
RP3-467L1.6
RBMS3-AS3
RP11-982M15.2
RP11-527N22.1
LINC00592
RP11-857B24.5
RP11-592N21.2
CTB-140J7.2

FOXD2-AS1
RP11-47I22.1
RP11-434H14.1
RP11-554A11.4
AC006538.1
RP11-344B5.4
MAMDC2-AS1
RP11-403B2.7
TTLL7-IT1
RP11-32B5.7
AC093390.1
AC067959.1
RP11-389G6.3
RP11-197K6.1
RP11-263K19.6
LINC00504
RP11-707P17.2
RP11-483L5.1
CTD-2334D19.1
LINC00454
LINC00865
HAND2-AS1
RP11-999E24.3
LDLRAD4-AS1
RP11-680F8.1
RP11-1109F11.3
RP11-158I9.5
CTB-12A17.3
RP11-154D6.1
CTD-3064M3.3
RP11-65J21.4
AC005618.6
AC106786.1
RP11-157J24.2
AC006262.6
RP11-92C4.6
CTD-2541J13.1
RP11-304L19.3
RP11-543D5.2
CTB-78F1.1
SNHG14
RP11-735G4.1
AC009228.1
AC010907.5
RP11-445N18.5
RP11-760D2.5
CTD-2005H7.1
RP11-613D13.8
RP1-223B1.1
VAC14-AS1

RP11-259K5.1
SSTR5-AS1
RP11-341G23.4
AC073130.3
CTC-296K1.4
AL022344.7
RP1-30E17.2
RP11-747H7.3
AC103564.7
RP11-80I15.4
CALML3-AS1
RP11-416N4.4
RP11-367F23.1
LINC00691
SERTAD4-AS1
LINC00940

13733
