# Supplementary material for: Identification of endoplasmic reticulum stress-associated lncRNAs influencing inflammation and VSMC function in abdominal aortic aneurysm
Source: Clin Sci (Lond). 2025 Mar 25;139(6):357–72. doi: 10.1042/CS20242476 (PMC12204013; doi:10.1042/CS20242476)
Supplement: Supplementary Table S2 [file CS-139-06-CS20242476-s003.pdf]

List of DE mRNA whose expression correlates with IL21-AS1

DE mRNA	Pearson	p-value
FA2H	0.90247568	1.2633E-07
TOR3A	0.92070402	2.3187E-08
EIF2AK3	0.91321641	4.8643E-08
KISS1R	0.90062871	1.4721E-07
TOP1	0.909024	7.1588E-08
AMER2	0.90242728	1.2684E-07
SMC1B	0.93325154	5.6002E-09

List of DE mRNA whose expression correlates with LINC00494

DE mRNA	Pearson	p-value
GATA3	0.93194749	6.5717E-09
IRF4	0.95660178	1.5624E-10
CXCL13	0.97696693	7.6799E-13
ICOS	0.92129175	2.181E-08
PIM2	0.93359773	5.3645E-09
MMP12	0.90323085	1.1856E-07
CCR7	0.95543866	1.9485E-10
CCR6	0.96838526	1.101E-11
SLAMF1	0.94553784	1.0366E-09
SDC1	0.92643558	1.25E-08
KLHL42	-0.90854464	7.4733E-08
CCDC88C	0.92533185	1.4132E-08
UBD	0.95039301	4.7661E-10
RUNX3	0.9521953	3.5013E-10
TAS1R3	0.91933299	2.6696E-08
RHOH	0.96993595	7.2178E-12
CXCR5	0.96994482	7.2E-12
RALGPS2	0.95581241	1.8162E-10
CD69	0.93253615	6.1163E-09
DERL3	0.94355935	1.3944E-09
RASSF6	0.90931138	6.9758E-08
BHLHA15	0.93857374	2.8147E-09
CXCR4	0.96267331	4.4303E-11
IGLL5	0.9564489	1.6089E-10
ENSG0000027	0.90134202	1.3882E-07
CR2	0.98036399	2.0013E-13
LTB	0.97694232	7.7493E-13
CD79A	0.96224896	4.8701E-11
SLA2	0.9131573	4.8916E-08
IL21R	0.93201058	6.5215E-09
CPNE5	0.93644057	3.7347E-09
GZMB	0.91100629	5.9775E-08
ALPK2	0.93017386	8.1282E-09
TIGIT	0.9574625	1.3216E-10
CYTIP	0.93708539	3.4323E-09
P2RY10	0.95425703	2.4241E-10
LTF	0.92468009	1.5182E-08
MEI1	0.95941001	8.933E-11
CD5	0.92739812	1.1213E-08
RASSF5	0.92100125	2.2481E-08
CD27	0.95839231	1.0988E-10
HSH2D	0.98332562	5.0365E-14
HLA-B	0.93378736	5.2391E-09

PYHIN1	0.93806485	3.0139E-09
FCRL3	0.9813763	1.2806E-13
NIBAN3	0.98944453	1.055E-15
GPR183	0.90040032	1.4999E-07
PRDM1	0.91018484	6.4445E-08
SHISA4	-0.94190513	1.7724E-09
BCL11B	0.9253482	1.4107E-08
SPIB	0.9835865	4.4086E-14
CD19	0.99196049	1.0515E-16
BCL2L11	0.90459855	1.0555E-07
FCRL2	0.9869884	6.1928E-15
KLRB1	0.90753412	8.1762E-08
TCF7	0.96080674	6.6651E-11
TAGAP	0.950586	4.6138E-10
BCL11A	0.98698778	6.1953E-15
MMP9	0.93749691	3.2507E-09
MZB1	0.94074026	2.0899E-09
PNOC	0.95092119	4.3595E-10
FGFRL1	-0.90597061	9.3773E-08
PLD4	0.97033184	6.4574E-12
SPOCK2	0.9551352	2.062E-10
SLAMF7	0.93816887	2.9722E-09
NUGGC	0.94804153	7.0085E-10
JSRP1	0.94248655	1.6304E-09
HLA-DOB	0.97041946	6.299E-12
P2RY8	0.96328644	3.8566E-11
SH2D3A	0.98502169	2.0353E-14
LY9	0.96392128	3.3324E-11
SH2D1A	0.94101724	2.0102E-09
RASGRP1	0.93414915	5.0069E-09
FCRLA	0.95737369	1.3449E-10
FDCSP	0.96240617	4.7029E-11
FCMR	0.97777664	5.6812E-13
LAG3	0.90365799	1.1436E-07
AGAP2	0.94694838	8.3341E-10
ISG20	0.9519531	3.6519E-10
FAAH2	0.92709852	1.1601E-08
CD3E	0.95860179	1.0534E-10
TRABD2A	0.97464498	1.7238E-12
ITK	0.94072729	2.0937E-09
PTPRCAP	0.97114837	5.1079E-12
CARMIL2	0.98115297	1.4161E-13
ZBP1	0.96029555	7.4282E-11
MS4A1	0.98998752	6.7464E-16
SIDT1	0.97566637	1.2196E-12
CCR4	0.90022673	1.5214E-07
LAX1	0.94911106	5.8944E-10

LCK	0.95558451	1.8959E-10
ZNF831	0.95326041	2.9016E-10
ACAP1	0.97905784	3.4445E-13
LAMP3	0.93023594	8.0687E-09
TNFRSF13B	0.98458573	2.5936E-14
GPR18	0.97243488	3.4814E-12
CLEC17A	0.96479981	2.7105E-11
POU2AF1	0.96063991	6.9062E-11
CD247	0.96667191	1.7142E-11
RTKN2	0.94563448	1.0214E-09
SYTL1	0.97569038	1.2095E-12
BTG1	0.90111054	1.4149E-07
CIBAR2	0.90692597	8.6264E-08
CD3D	0.96214234	4.9865E-11
ARHGAP15	0.92118172	2.2062E-08
GP1BA	0.94356513	1.3933E-09
STAP1	0.98190402	1.0048E-13
TJP3	0.94146573	1.8868E-09
NUP210	0.9566557	1.5462E-10
BLK	0.98593256	1.1977E-14
RAB11FIP4	0.96612794	1.9635E-11
NUAK2	0.94822733	6.8027E-10
IL7R	0.92954527	8.7525E-09
ENSG0000027	0.92444279	1.558E-08
SP140	0.98008753	2.2517E-13
IL2RG	0.96432503	3.0325E-11
PLA2G2D	0.96053264	7.0652E-11
ERMN	0.95586221	1.7991E-10
CBFA2T3	0.96105972	6.3136E-11
HSPB1	-0.92874705	9.6056E-09
TESPA1	0.96138288	5.8883E-11
GZMK	0.92104057	2.2389E-08
LTK	0.93042858	7.8865E-09
PLCG2	0.9552864	2.0047E-10
CD226	0.93653925	3.687E-09
FAM177B	0.96851992	1.0622E-11
CD52	0.97082901	5.6028E-12
CXCR3	0.95964785	8.5047E-11
CCL5	0.90231189	1.2807E-07
TBC1D10C	0.97387108	2.2199E-12
ITGB7	0.97146068	4.6615E-12
SEMA7A	0.95209093	3.5655E-10
TNFRSF13C	0.98240452	7.9298E-14
FCRL1	0.98459971	2.5738E-14
MTMR11	-0.92177115	2.0741E-08
LEF1	0.93921795	2.5791E-09
PDCD1	0.92651399	1.239E-08

ADGRG5	0.9764821	9.1525E-13
CD1C	0.96283302	4.2741E-11
LTA	0.95606981	1.7297E-10
IKZF3	0.9665929	1.7486E-11
GPR132	0.90243515	1.2676E-07
NPTXR	-0.9304776	7.8407E-09
ZAP70	0.96596396	2.0447E-11
C16orf54	0.92773446	1.0792E-08
CD48	0.9579766	1.194E-10
NLRC3	0.96137168	5.9026E-11
CD96	0.93519946	4.3832E-09
LAD1	0.94788231	7.1893E-10
TP73	0.9461902	9.378E-10
FLT3	0.93913171	2.6096E-09
PRKCH	0.90356996	1.1521E-07
MC4R	0.90279614	1.2298E-07
SPAG4	0.91086938	6.0532E-08
SEPTIN1	0.97798044	5.2569E-13
FCRL5	0.95727897	1.37E-10
TNFRSF9	0.95831954	1.1149E-10
SMPDL3B	0.91699891	3.3746E-08
DLGAP5	0.92502416	1.462E-08
CAMK4	0.91265828	5.1268E-08
PATL2	0.96812351	1.1799E-11
ANO9	0.97767947	5.8939E-13
RCSD1	0.96042183	7.2329E-11
BANK1	0.96982988	7.4345E-12
SEL1L3	0.94515848	1.0982E-09
RPS6KA6	-0.9052437	9.9863E-08
CD6	0.95431335	2.3993E-10
HLA-C	0.91402997	4.5028E-08
BTLA	0.95090316	4.3728E-10
SHISA8	0.9658029	2.1272E-11
ICOSLG	0.95489851	2.1546E-10
CD40LG	0.96884413	9.7383E-12
TRAT1	0.95086796	4.399E-10
SYNGR3	0.9001738	1.528E-07
MYBL2	0.94745912	7.6897E-10
CD24	0.92976419	8.5305E-09
CD7	0.94857227	6.4345E-10
ARAP2	0.91438686	4.3518E-08
JCHAIN	0.93594607	3.9823E-09
MAP4K1	0.98570164	1.3744E-14
CCL22	0.91801093	3.0512E-08
CD38	0.9381853	2.9657E-09
MYB	0.93017899	8.1233E-09
PVRIG	0.95858058	1.0579E-10

PARP15	0.96958687	7.9526E-12
P2RX5	0.98750742	4.3891E-15
RAC2	0.9570304	1.4381E-10
FCER2	0.94906641	5.9376E-10
HLA-A	0.91475817	4.1994E-08
FOXP3	0.90590768	9.4287E-08
SIRPG	0.96870846	1.01E-11
IL2RB	0.90726667	8.3716E-08
CTLA4	0.94185075	1.7863E-09
HS3ST3B1	0.93433642	4.8903E-09
PRKCQ	0.9482205	6.8102E-10
IPCEF1	0.93461643	4.7204E-09
STIM2	0.94707761	8.1667E-10
TIFAB	0.96021742	7.5514E-11
LARGE2	0.98181697	1.0463E-13
NUSAP1	0.92705263	1.1661E-08
GRAP2	0.93698525	3.4778E-09
TOX	0.92172876	2.0834E-08
DEXI	-0.93514871	4.4117E-09
PTK2B	0.97315748	2.7845E-12
VPREB3	0.97365519	2.379E-12
XCR1	0.94911156	5.8939E-10
MYO1G	0.95161273	3.8733E-10
CARD11	0.97186114	4.1395E-12
SEMA4A	0.92459942	1.5316E-08
IL16	0.94751874	7.6174E-10
ATP10B	0.90193656	1.3214E-07
TLR10	0.96632169	1.8713E-11
TMEM156	0.97137652	4.7783E-12
SRMS	0.95927177	9.1906E-11
WNT10A	0.96207861	5.0572E-11
CD3G	0.93195409	6.5664E-09
SKAP1	0.9482177	6.8132E-10
CIITA	0.96888933	9.6203E-12
GMFG	0.94782612	7.254E-10
GPR160	0.94673104	8.6224E-10
BEND4	0.95982706	8.1939E-11
ICAM3	0.95017196	4.9459E-10
POU2F2	0.96593102	2.0613E-11
RELT	0.91159146	5.663E-08
B2M	0.90385778	1.1244E-07
ADH5	-0.95642819	1.6153E-10
KLHL6	0.97136631	4.7926E-12
ZBTB32	0.93724388	3.3613E-09
FCHO1	0.97117648	5.0662E-12
TRAF3IP3	0.96526573	2.4241E-11
CD2	0.93625952	3.8238E-09

BIK	0.92637484	1.2585E-08
RBP5	0.94745	7.7008E-10
ADAM8	0.91002377	6.5398E-08
PPP1R16B	0.95106693	4.2528E-10
MPZL3	0.90950956	6.8521E-08
RRM2	0.90393531	1.117E-07
ESR2	0.96537953	2.3583E-11
DEF6	0.96786898	1.2613E-11
KIF21B	0.96471654	2.7647E-11
S1PR4	0.94379575	1.3467E-09
CD83	0.90817354	7.7251E-08
CYTH1	0.96282876	4.2782E-11
GPR174	0.94166797	1.8334E-09
KIF1C	-0.96064415	6.8999E-11
TMEM178B	0.94472325	1.1728E-09
AIM2	0.97443404	1.8483E-12
SEMA4D	0.96702529	1.5676E-11
CCNE2	0.91855795	2.8879E-08
LIMD2	0.97411692	2.0502E-12
CLNK	0.98788046	3.3964E-15
CD74	0.94557498	1.0308E-09
RASAL3	0.96711296	1.533E-11
PDE7A	0.96799774	1.2195E-11
CENPF	0.90461673	1.0539E-07
MKI67	0.93103375	7.3374E-09
CRTAM	0.9187813	2.8235E-08
ACADL	-0.9027465	1.2349E-07
APBA2	0.91461163	4.2589E-08
BIRC3	0.92292081	1.8361E-08
LGALS2	0.95313595	2.9666E-10
TSHR	0.9195619	2.608E-08
PACSIN1	0.94973468	5.3193E-10
LIMS4	-0.93677517	3.575E-09
RIPOR2	0.9599302	8.0196E-11
DAPP1	0.96087276	6.5717E-11
UBASH3A	0.93316719	5.659E-09
CD37	0.97331895	2.6468E-12
LIME1	0.95600141	1.7523E-10
APOBEC3B	0.90527862	9.9562E-08
ADORA2A	0.94050371	2.1601E-09
C16orf74	0.93805013	3.0198E-09
MYBPC2	0.94200573	1.7471E-09
CDC14A	0.91126866	5.8346E-08
TOP2A	0.92531636	1.4157E-08
TIAM1	0.90161164	1.3575E-07
IDO1	0.93204571	6.4937E-09
IL5RA	0.90201608	1.3127E-07

CD22	0.96676445	1.6747E-11
ARID3A	0.90566785	9.6269E-08
TNFRSF4	0.92161827	2.1077E-08
CD1E	0.93499085	4.5014E-09
ASF1B	0.92759721	1.0962E-08
RCAN3	0.9380441	3.0223E-09
SLAMF6	0.95735105	1.3508E-10
PSMB10	0.92503494	1.4602E-08
SNX22	0.96564413	2.2114E-11
RHOF	0.95046872	4.7058E-10
CETP	0.90324239	1.1845E-07
CELSR1	0.9298575	8.4373E-09
FMNL1	0.92593018	1.3226E-08
TMC8	0.96210418	5.0287E-11
AMPD1	0.92349644	1.7262E-08
KCNJ10	0.93446726	4.8103E-09
SCML4	0.97790111	5.4186E-13
SHISAL2A	0.97855128	4.2131E-13
HLA-DQA1	0.9031097	1.1978E-07
ITPR3	0.94500684	1.1237E-09
OSBPL3	0.9312558	7.1445E-09
SMAP2	0.95426869	2.4189E-10
FYB1	0.92639895	1.2551E-08
KLRG1	0.93431401	4.9042E-09
BCL2A1	0.90913743	7.0861E-08
PLCH2	0.96487641	2.6615E-11
INPP5D	0.96710276	1.537E-11
ITGAL	0.94959018	5.448E-10
CORO7	0.95451015	2.3144E-10
HLA-F	0.94693293	8.3543E-10
CNTNAP2	0.91626031	3.6293E-08
PAX5	0.97775326	5.7317E-13
TBX21	0.93800511	3.0381E-09
GPSM3	0.94707032	8.1761E-10
TBC1D4	0.90856001	7.4631E-08
DENND1C	0.96561045	2.2297E-11
GLCCI1	0.94880186	6.1993E-10
HMMR	0.91541572	3.9408E-08
PSD4	0.95381001	2.629E-10
ARHGAP25	0.96679469	1.662E-11
FBXL16	0.92603735	1.3069E-08
ABI3	0.91578784	3.8008E-08
RASGRF1	0.94352565	1.4014E-09
CHD7	0.95188619	3.6946E-10
TTN	0.91812573	3.0163E-08
KLHL14	0.96402852	3.2503E-11
AKNA	0.9665712	1.7582E-11

SSTR3	0.93784571	3.1034E-09
KNTC1	0.95979523	8.2484E-11
EPHA1	0.94999803	5.0916E-10
GPA33	0.92998575	8.3108E-09
IKZF1	0.9200518	2.4802E-08
PLEKHG7	0.9649093	2.6407E-11
SELL	0.92172675	2.0838E-08
WAS	0.94281979	1.5536E-09
SEPTIN6	0.97088125	5.5191E-12
IL27RA	0.9519401	3.6602E-10
ATAD5	0.90617889	9.2089E-08
DOCK8	0.92674035	1.2079E-08
CLEC2D	0.96440799	2.974E-11
BLM	0.94355392	1.3956E-09
ADAM28	0.94478825	1.1614E-09
TMIGD2	0.91305709	4.938E-08
UBE2C	0.91203199	5.4359E-08
CD79B	0.95104501	4.2687E-10
PTPN7	0.95185029	3.7176E-10
EVI2B	0.92734251	1.1284E-08
BUB1	0.91530282	3.9842E-08
STK10	0.95456638	2.2907E-10
UBALD2	0.90635805	9.0662E-08
KEL	0.9394113	2.5119E-09
TCL1A	0.96993754	7.2146E-12
EZH2	0.91687941	3.4148E-08
PRDM6	-0.90100986	1.4267E-07
AURKB	0.92157569	2.1171E-08
MAPK13	0.90483949	1.034E-07
CNR2	0.98535542	1.6824E-14
PBX4	0.91019092	6.441E-08
PTPRC	0.90054824	1.4819E-07
HLA-DRA	0.91423606	4.415E-08
CCDC88B	0.9649874	2.5918E-11
MAN2B1	0.92118524	2.2054E-08
SLC37A1	0.95554786	1.909E-10
ACOXL	0.94271665	1.5771E-09
KIFC1	0.93627226	3.8174E-09
GCSAM	0.97138161	4.7711E-12
BIN2	0.93185937	6.6423E-09
CSF2RA	0.93098498	7.3804E-09
NLRC5	0.97068	5.8478E-12
ARHGAP9	0.93888943	2.697E-09
RAB37	0.92746289	1.1131E-08
GIPR	0.92588098	1.3298E-08
CDK5R1	0.96237877	4.7317E-11
RGL4	0.90931184	6.9755E-08

ARHGAP45	0.96720523	1.4973E-11
CCDC18	0.94935764	5.6608E-10
FAM181B	-0.91890903	2.7872E-08
TCEAL5	-0.91027166	6.3937E-08
SFMBT2	0.91851115	2.9016E-08
SNX20	0.92896602	9.3646E-09
KLRK1	0.90119805	1.4048E-07
PASK	0.96052431	7.0777E-11
LPXN	0.94763784	7.4747E-10
TSPAN13	0.94410474	1.2864E-09
STAT4	0.93150523	6.9331E-09
IRAG2	0.94994052	5.1406E-10
RNASET2	0.94338548	1.4305E-09
IL17REL	0.93585551	4.0291E-09
ARHGAP27	0.96314223	3.9853E-11
JAML	0.92882793	9.5159E-09
GRAMD1B	0.90406075	1.1051E-07
TIGD3	0.93255203	6.1044E-09
P2RX5-TAX1B	0.97793036	5.3585E-13
IRF8	0.9640097	3.2646E-11
BTK	0.94346197	1.4145E-09
CENPM	0.94263932	1.5948E-09
HLA-DMA	0.92187377	2.0518E-08
NCAPH	0.90118116	1.4067E-07
CRYBG1	0.90952107	6.8449E-08
FAM214A	0.93086601	7.4861E-09
STK17B	0.92634486	1.2627E-08
NCKAP1L	0.93311738	5.694E-09
COTL1	0.91184804	5.5298E-08
SYK	0.91650882	3.5418E-08
HLA-DPB1	0.92498602	1.4681E-08
ARHGAP30	0.9290587	9.2642E-09
PDCD4	0.90562323	9.6641E-08
VAV1	0.91837205	2.9425E-08
PIK3CD	0.9637038	3.5044E-11
TNFRSF10A	0.92015577	2.4538E-08
FCRL4	0.9432257	1.4644E-09
SGO1	0.97093499	5.4341E-12
TMEM163	0.92003983	2.4833E-08
ZC3H12D	0.96154026	5.6905E-11
GAPT	0.9341532	5.0044E-09
HOOK1	0.91553977	3.8937E-08
CYBA	0.93257421	6.0878E-09
SPN	0.93062291	7.7064E-09
ABCA7	0.9623182	4.7958E-11
TNFSF11	0.91165957	5.6274E-08
TNFRSF18	0.91793275	3.0752E-08

CD72	0.9669257	1.6078E-11
CACNA1I	0.91839567	2.9355E-08
ESPL1	0.9327354	5.9685E-09
GPR55	0.95823558	1.1338E-10
SAPCD2	0.93739003	3.297E-09
LCP2	0.90966536	6.7561E-08
REEP2	-0.93760685	3.2036E-09
DOCK2	0.91405389	4.4925E-08
ZNF217	0.92333521	1.7564E-08
CLMN	0.90492798	1.0261E-07
TLR9	0.9461633	9.4171E-10
GRAP	0.96089305	6.5433E-11
ALOX5	0.91366132	4.6636E-08
TAP1	0.93233519	6.2685E-09
TACC3	0.95812619	1.1589E-10
LSP1	0.93841836	2.8743E-09
TTC22	0.95075426	4.4846E-10
SLFN12L	0.92449122	1.5498E-08
PTPN22	0.91691524	3.4027E-08
HLA-DOA	0.95271773	3.1948E-10
EXOC3L4	0.93081057	7.5359E-09
TMC6	0.9587271	1.027E-10
FGD3	0.9386117	2.8003E-09
SH3KBP1	0.90962972	6.7779E-08
DTHD1	0.90609496	9.2764E-08
IGFLR1	0.94175729	1.8102E-09
ZNF296	0.95167711	3.8305E-10
CORO1A	0.93865065	2.7856E-09
CBX4	0.91517566	4.0336E-08
PLAC8	0.92889703	9.44E-09
LINGO3	0.96186313	5.3028E-11
JAK3	0.91935272	2.6643E-08
CFLAR	0.93400613	5.0976E-09
TMEM154	0.90671621	8.7866E-08
LAPTM5	0.90851592	7.4926E-08
KNL1	0.90518905	1.0033E-07
HLA-DMB	0.92188972	2.0484E-08
HJURP	0.91582112	3.7885E-08
CTSH	0.96291727	4.1936E-11
CDC42SE2	0.91237598	5.2642E-08
ICA1	0.90841254	7.5621E-08
ANGPTL6	0.92968181	8.6135E-09
SH3BP1	0.90500731	1.0192E-07
PCDHB6	-0.91902044	2.7559E-08
NFKBIE	0.93434262	4.8865E-09
LSR	0.93894018	2.6785E-09
ARHGAP4	0.95205827	3.5858E-10

TXK	0.96356759	3.6161E-11
UCP2	0.93614049	3.8833E-09
CLEC9A	0.92786383	1.0633E-08
NPHS1	0.9219219	2.0414E-08
CDCA2	0.91284288	5.0386E-08
NDC80	0.93801818	3.0328E-09
PTPN6	0.94586047	9.8667E-10
CCNB2	0.93133731	7.0748E-09
RASGRP3	0.92942006	8.8818E-09
GTSE1	0.90635784	9.0663E-08
SPR	-0.92479459	1.4993E-08
IRF7	0.93062578	7.7037E-09
NCF1	0.94383908	1.3381E-09
PLCB2	0.92890782	9.4281E-09
STK4	0.92765799	1.0886E-08
TROAP	0.91058639	6.2124E-08
SASH3	0.9226554	1.8888E-08
NEK2	0.92795771	1.052E-08
ADM2	0.94481974	1.1559E-09
GHRL	0.92461756	1.5286E-08
FERMT3	0.91006617	6.5146E-08
NFE2L3	0.92357057	1.7125E-08
RNF166	0.94049464	2.1628E-09
GMIP	0.94900247	5.9999E-10
PSMB9	0.93243731	6.1908E-09
HCLS1	0.93848189	2.8498E-09
ATP2A3	0.94622637	9.3258E-10
MAP3K9	0.90372851	1.1368E-07
DOK3	0.91435263	4.3661E-08
LRRK1	0.93925355	2.5666E-09
PRKCZ	0.93123295	7.1641E-09
RNF44	0.97210648	3.8458E-12
KMO	0.93495807	4.5202E-09
STAMBPL1	0.90305308	1.2035E-07
WDFY4	0.93743638	3.2768E-09
LPAR2	0.94100558	2.0135E-09
PAN3	0.90003132	1.5458E-07
TNFRSF17	0.9500855	5.0179E-10
SP110	0.93567634	4.1232E-09
PAG1	0.94730042	7.8851E-10
WNT16	0.92396851	1.6404E-08
TASL	0.90569226	9.6065E-08
ANXA2R	0.94389688	1.3267E-09
ARRDC5	0.94140572	1.9029E-09
LGALS9	0.90970154	6.734E-08
FANCD2	0.94558794	1.0287E-09
SH3TC1	0.92147627	2.1393E-08

LST1	0.90891987	7.2261E-08
PLAG1	0.95897988	9.7561E-11
TTC24	0.95764406	1.2752E-10
BLNK	0.94285848	1.5449E-09
PRKX	0.93064353	7.6875E-09
CALHM6	0.90095769	1.4329E-07
RPS6KA1	0.91338999	4.7851E-08
CNTRL	0.94515712	1.0984E-09
DPEP2	0.93832974	2.9087E-09
PARVG	0.94179954	1.7994E-09
CDH1	0.92287078	1.846E-08
DRAM2	0.92383781	1.6638E-08
ARMH1	0.9123356	5.2841E-08
HCST	0.90469385	1.047E-07
OXNAD1	0.92263245	1.8935E-08
HS3ST3A1	0.91819195	2.9963E-08
LMNTD2	0.94761799	7.4984E-10
MAP3K14	0.93094132	7.419E-09
ILDR1	0.97380205	2.2697E-12
SLC25A23	-0.91650814	3.542E-08
UNC13D	0.94172975	1.8173E-09
E2F2	0.92662953	1.2231E-08
FBXW7	0.90029947	1.5124E-07
PSMB8	0.92173947	2.081E-08
SIT1	0.94790857	7.1592E-10
TNFSF8	0.90043123	1.4962E-07
TDP1	0.92955307	8.7445E-09
SIGLEC10	0.92775508	1.0766E-08
MALT1	0.9234356	1.7376E-08
ZBTB46	0.93276997	5.9432E-09
GFI1	0.94101237	2.0116E-09
RGS14	0.90214372	1.2988E-07
ZNF814	0.94757371	7.5513E-10
ANKRD44	0.9511382	4.2014E-10
TPD52	0.92720831	1.1457E-08
CMTM7	0.90670069	8.7985E-08
DENND2D	0.91669111	3.4788E-08
WDR62	0.90444667	1.0693E-07
PCNX2	0.90388552	1.1217E-07
MCM5	0.95023362	4.8952E-10
VSIG1	0.9460143	9.636E-10
CASP10	0.90268459	1.2414E-07
TMPRSS13	0.95012152	4.9878E-10
RND2	-0.9017028	1.3473E-07
SPNS3	0.9363486	3.7797E-09
KCNN4	0.93266737	6.0186E-09
STXBP2	0.91004858	6.525E-08

ISL2	0.91237323	5.2655E-08
MTMR14	0.96200399	5.1411E-11
JARID2	0.93312438	5.6891E-09
NME8	0.91037262	6.335E-08
ASPM	0.91667189	3.4854E-08
TLR6	0.92304691	1.8116E-08
KBTBD8	0.92387193	1.6577E-08
IFNLR1	0.94602611	9.6185E-10
MUC3A	0.92475221	1.5062E-08
CIAO2A	0.91114833	5.8998E-08
PDE6G	0.92191538	2.0428E-08
FXYD7	0.90816879	7.7283E-08
MOB3A	0.94918531	5.8232E-10
ANKRD35	-0.92723092	1.1428E-08
KLK1	0.9472832	7.9065E-10
TGIF2	0.93189661	6.6124E-09
CD1D	0.93671767	3.602E-09
IL23A	0.90404465	1.1066E-07
GRIK5	-0.93601972	3.9445E-09
NR2F6	-0.92177647	2.0729E-08
TREML2	0.92584514	1.3351E-08
IL12RB1	0.91219917	5.3519E-08
MFNG	0.90997269	6.5702E-08
RAP2C	0.90684193	8.6902E-08
FAM78A	0.90312203	1.1965E-07
IL18	0.93448803	4.7977E-09
HMGA1	0.92912755	9.1903E-09
ATG9B	0.95509825	2.0763E-10
ITGA7	-0.90172362	1.345E-07
EAF2	0.92707032	1.1638E-08
CENPK	0.90388861	1.1214E-07
RESF1	0.90437562	1.0758E-07
POLQ	0.9110525	5.9521E-08
RHBDF2	0.94990632	5.1699E-10
LAT2	0.91006251	6.5167E-08
C12orf42	0.9492375	5.7736E-10
SP100	0.92104766	2.2373E-08
SPEG	-0.93223249	6.3476E-09
GUCY2D	0.96362593	3.5679E-11
CRLF3	0.93239274	6.2246E-09
ARHGDIB	0.94162517	1.8446E-09
ARMCX2	-0.93812585	2.9894E-09
CD53	0.90850699	7.4985E-08
PEF1	-0.92920423	9.1085E-09
FANCA	0.92578892	1.3435E-08
FUT7	0.90429018	1.0837E-07
VAV3	0.90266484	1.2434E-07

RCC2	0.91780955	3.1133E-08
EVL	0.94358184	1.3898E-09
KDM2A	0.9210318	2.241E-08
ADPGK	0.92278846	1.8623E-08
RGS10	0.90809529	7.7791E-08
CD80	0.92833001	1.008E-08
WDR54	0.90244574	1.2664E-07
STK32A	-0.92465781	1.5219E-08
UNC93B1	0.93447531	4.8054E-09
TAPBP	0.95273392	3.1857E-10
AP1G2	0.93844161	2.8653E-09
SCUBE3	-0.93437618	4.8659E-09
ORAI1	0.90431416	1.0815E-07
GEN1	0.93596922	3.9704E-09
CORO2A	0.90925875	7.009E-08
RINL	0.93168454	6.7845E-09
SH3BGR	-0.93282761	5.9012E-09
TMEM273	0.90781931	7.9722E-08
CAMSAP3	0.94471356	1.1745E-09
STRBP	0.95520193	2.0366E-10
NCR3	0.92422054	1.5962E-08
MYCL	0.92713156	1.1557E-08
KCTD2	-0.90259088	1.2511E-07
TLN2	-0.9033991	1.1689E-07
CCDC78	0.93374011	5.2701E-09
LILRA4	0.91798466	3.0592E-08
APOBEC3G	0.90410067	1.1014E-07
ARHGEF1	0.95380531	2.6312E-10
SGPP2	0.93235902	6.2503E-09
TTC7A	0.94506239	1.1143E-09
HSF5	0.95147016	3.9694E-10
EIF2B2	-0.91102601	5.9666E-08
SMPD3	0.91741352	3.2387E-08
MAGED2	-0.93918731	2.5899E-09
SNAI3	0.92695285	1.1793E-08
GTSF1	0.92578987	1.3434E-08
TSPAN33	0.9030904	1.1997E-07
KLHL30	-0.91778572	3.1207E-08
ASPHD2	0.90311228	1.1975E-07
FAM110A	0.90531142	9.9281E-08
BTC	-0.92371167	1.6866E-08
SPRYD3	-0.90048673	1.4894E-07
OPN3	0.93963257	2.4368E-09
SIPA1	0.93527581	4.3406E-09
USP3	0.91890964	2.787E-08
NBEAL2	0.94114624	1.974E-09
KCNAB2	0.92753333	1.1042E-08

CLECL1	0.93902323	2.6484E-09
SPAG5	0.90600668	9.3479E-08
PLN	-0.95241939	3.3668E-10
MTRF1L	-0.9025467	1.2558E-07
GGA2	0.94870719	6.2954E-10
BEX3	-0.94258499	1.6074E-09
LIG1	0.94247631	1.6329E-09
ARL2	-0.93130582	7.1016E-09
GRK2	0.96439787	2.981E-11
BICDL1	0.90736135	8.3019E-08
TCEAL7	-0.91547017	3.9201E-08
ADRA1D	-0.90380086	1.1298E-07
GSAP	0.95221554	3.489E-10
E2F5	0.90916389	7.0692E-08
HSPA4L	-0.95479824	2.1949E-10
EBI3	0.92478517	1.5008E-08
SCIMP	0.912286	5.3086E-08
SCRG1	-0.94337826	1.432E-09
NPR2	-0.93393209	5.1451E-09
GSTK1	0.90632097	9.0955E-08
RASSF7	0.94895734	6.0443E-10
NUDT9	-0.97300732	2.9183E-12
TMEM266	0.92940845	8.8938E-09
GPR19	0.92680269	1.1995E-08
TNFAIP8	0.90936395	6.9428E-08
ADRA2C	-0.94480257	1.1589E-09
ZNF101	0.90963354	6.7756E-08
FGD2	0.92391927	1.6492E-08
SLC12A3	0.91989872	2.5196E-08
MYOCD	-0.92051938	2.3634E-08
SYNPO	-0.93029219	8.0151E-09
FRMD8	0.90574425	9.5634E-08
RUBCNL	0.93962695	2.4387E-09
ACY3	0.90327735	1.181E-07
AP1S3	0.9401667	2.2637E-09
CSK	0.9268681	1.1907E-08
SMC6	0.90266145	1.2438E-07
LIMK1	0.91554158	3.893E-08
CACNA1F	0.9117305	5.5905E-08
GNGT2	0.90976272	6.6967E-08
UNC45A	-0.94375132	1.3555E-09
RUSC1	-0.90172296	1.345E-07
LURAP1	-0.94062568	2.1236E-09
RTL8C	-0.90138663	1.383E-07
MSRB2	-0.96145054	5.8025E-11
HDAC11	-0.94041619	2.1866E-09
CMAS	-0.90834281	7.6093E-08

NGRN	-0.95719601	1.3924E-10
ETNK2	-0.91479125	4.186E-08
ABCB11	0.90381201	1.1287E-07
TMEM106C	-0.90513254	1.0082E-07
SSH2	0.90329207	1.1795E-07
CENPB	-0.9134752	4.7467E-08
GRK6	0.92132346	2.1738E-08
ATXN7L1	0.94388358	1.3293E-09
CTD-2515O10	-0.90164719	1.3535E-07
TNFAIP8L1	0.92144093	2.1472E-08
SAMD10	0.90301824	1.207E-07
IRF5	0.9049073	1.028E-07
DYNLL2	-0.90052892	1.4842E-07
NPAS2	-0.93841381	2.876E-09
HAGHL	0.91348065	4.7442E-08
AMMECR1	0.91069505	6.1508E-08
STAG3	0.90646918	8.9786E-08
RASAL1	0.92786907	1.0627E-08
DENND6B	0.91801063	3.0513E-08
PTGIR	-0.92788863	1.0603E-08
EXTL2	-0.95017461	4.9437E-10
ZNF532	-0.95474014	2.2186E-10
ROM1	-0.92645877	1.2467E-08
CAMK1	-0.90556649	9.7117E-08
TRIP6	-0.90753146	8.1781E-08
DMTN	-0.90137582	1.3843E-07
CDK5RAP2	-0.90036264	1.5046E-07
ERBB2	-0.91942466	2.6448E-08
SLC22A17	-0.90339449	1.1694E-07
MORN3	0.93223521	6.3455E-09
SS18L2	0.94481157	1.1573E-09
CHD5	-0.9312954	7.1105E-09
CCDC8	-0.90809984	7.7759E-08
TMEM25	-0.9356147	4.1561E-09
FBXO46	0.92897257	9.3575E-09
CD99L2	-0.90639084	9.0402E-08
TMEM9	-0.92215788	1.9912E-08
RPL28	0.9082356	7.6825E-08
CDIPT	-0.94996563	5.1191E-10
PIGR	0.93915272	2.6022E-09
DENND4B	0.93226658	6.3213E-09
SOD1	-0.92767176	1.0869E-08
NLK	0.92406231	1.6239E-08
LRIF1	0.90138108	1.3837E-07
ZNF503	-0.91026365	6.3984E-08
TRABD	0.90646509	8.9818E-08
ACTRT3	-0.90278168	1.2313E-07

PRKD2	0.95520414	2.0357E-10
FAM53B	0.92497604	1.4697E-08
KDM2B	0.95115607	4.1886E-10
TRNP1	-0.908459	7.5308E-08
PIP5K1C	-0.93506474	4.4592E-09
ZNRF3	-0.96014646	7.6648E-11
LTB4R2	0.92805254	1.0406E-08
PTCRA	0.92745697	1.1138E-08
MCOLN2	0.91143913	5.7435E-08
ARID3B	0.90858616	7.4456E-08
ANO10	-0.94229189	1.6768E-09
KPNA6	-0.93476398	4.633E-09
PCDHGA7	-0.92081279	2.2926E-08
PCDHGA4	-0.93159377	6.8594E-09
NME7	-0.90533409	9.9087E-08
RAMP1	-0.90144161	1.3768E-07
C19orf54	0.93095097	7.4105E-09
ORAI2	0.91128029	5.8284E-08
SYDE1	-0.90218913	1.2939E-07
EFCAB12	0.90511192	1.01E-07
PCDHB15	-0.93742234	3.2829E-09
TMEM243	0.92710255	1.1595E-08
AHSA1	-0.9014093	1.3805E-07
ARHGEF17	-0.93053652	7.786E-09
NKD1	-0.90978536	6.683E-08
LRP5	-0.91253188	5.1879E-08
NRSN2	-0.91921216	2.7027E-08
ENTPD4	0.90538515	9.8651E-08
DENND3	0.93590103	4.0055E-09
CBY1	-0.93924975	2.568E-09
TOM1L2	-0.90264796	1.2452E-07
CCDC9B	-0.90078115	1.4538E-07
PCDHB14	-0.90912356	7.095E-08
SARS1	-0.90287816	1.2213E-07
ADCY9	-0.90388245	1.122E-07
AKTIP	-0.90682613	8.7023E-08
TRIM55	0.92011603	2.4639E-08
PFN2	-0.94562148	1.0235E-09
RILPL1	-0.92586066	1.3328E-08
PARP14	0.901191	1.4056E-07
LRFN3	-0.91400721	4.5126E-08
FITM2	-0.92789645	1.0594E-08
KIAA0319L	0.95415955	2.4675E-10
PREPL	-0.93364727	5.3315E-09
MAP9	-0.90853295	7.4812E-08
FEZ1	-0.94836903	6.6493E-10
ENDOD1	-0.91938498	2.6555E-08

PCDHGB4	-0.90316211	1.1925E-07
VSTM4	-0.90587628	9.4544E-08
MLLT1	-0.92969324	8.6019E-09
TCF3	0.93166615	6.7996E-09
NLRP1	0.91806125	3.0358E-08
WIZ	-0.93157313	6.8765E-09
DAB2IP	-0.9262218	1.2802E-08
FAM185A	-0.91207786	5.4128E-08
PAM	-0.90322429	1.1863E-07
RORC	0.90224234	1.2881E-07
RAB8A	0.91727061	3.285E-08
SLC25A4	-0.92156721	2.119E-08
PLPPR2	-0.94326036	1.457E-09
CEP170B	-0.91208477	5.4093E-08
TRPC4	-0.9213578	2.166E-08
PID1	-0.91681142	3.4378E-08
MANBAL	-0.9166182	3.5039E-08
BAG2	-0.92628723	1.2709E-08
PCDH20	-0.92959337	8.7033E-09
ZNF276	0.91643276	3.5684E-08
MFGE8	-0.90350232	1.1587E-07
RPRD1A	-0.91744514	3.2285E-08
IL9R	0.93776175	3.1383E-09
PGAP4	-0.92795696	1.0521E-08
SUCLA2	-0.94706101	8.1881E-10
DLG3	-0.93240457	6.2156E-09
FBLIM1	-0.92475683	1.5055E-08
SLC48A1	-0.9277627	1.0757E-08
B3GNT3	0.90054944	1.4817E-07
TMEM8B	-0.90931473	6.9737E-08
MRPL43	-0.9012788	1.3954E-07
KDF1	0.95593619	1.7741E-10
C1orf216	-0.92260534	1.8989E-08
SMARCD3	-0.93978189	2.3873E-09
RTL5	-0.91677758	3.4493E-08
CYB5R1	-0.96495932	2.6093E-11
RAB34	-0.92208582	2.0064E-08
ARMC10	-0.93909358	2.6232E-09
DBNDD2	-0.92677264	1.2035E-08
LRP3	-0.93544895	4.2455E-09
AOC3	-0.94315495	1.4796E-09
KIF7	-0.91399774	4.5167E-08
DCTN1	-0.98061394	1.7963E-13
AKAP1	-0.94721288	7.9947E-10
CYBC1	0.90238204	1.2732E-07
OSBPL5	-0.91290026	5.0115E-08
P3H3	-0.91797534	3.0621E-08

CLN8	0.93291341	5.8392E-09
CCND3	0.91375687	4.6215E-08
CYB5R3	-0.90543325	9.8242E-08
NACAD	-0.92170247	2.0891E-08
KLHL13	-0.92922252	9.0891E-09
CASP2	0.90545491	9.8059E-08
MAP6	-0.95609794	1.7205E-10
PFKP	-0.906659	8.8307E-08
PEG3	-0.92844803	9.9434E-09
SNPH	-0.92015799	2.4533E-08
FAM234A	-0.93664785	3.6351E-09
FAM161B	-0.92545655	1.3939E-08
KBTBD12	-0.93035748	7.9533E-09
PRAG1	0.90736938	8.296E-08
GRAMD1C	0.91442629	4.3354E-08
TYK2	0.90352652	1.1564E-07
PM20D2	-0.90455756	1.0592E-07
HABP4	-0.91448248	4.3121E-08
COPRS	-0.91237519	5.2646E-08
QSOX1	-0.91472402	4.2132E-08
ARMC9	-0.9225135	1.9176E-08
RELL2	0.91022154	6.423E-08
ATP6V1E1	-0.92524854	1.4263E-08
LPAR1	-0.90740587	8.2694E-08
OSR1	-0.92989741	8.3978E-09
BTBD10	-0.93740743	3.2894E-09
PFKM	-0.96785095	1.2672E-11
VILL	0.91999835	2.4939E-08
HSPB7	-0.90854987	7.4698E-08
KCNA5	-0.9224543	1.9297E-08
ST3GAL3	-0.91965782	2.5825E-08
LDOC1	-0.92653931	1.2355E-08
METTL24	-0.943167	1.477E-09
ZNF219	-0.9438643	1.3331E-09
TGFB1I1	-0.95264487	3.2361E-10
ACSS1	0.90647266	8.9758E-08
AAR2	-0.92716048	1.152E-08
ZBTB47	-0.94905428	5.9494E-10
HKDC1	0.91725441	3.2903E-08
KEAP1	-0.90285518	1.2237E-07
DDX19A	-0.93573444	4.0925E-09
HPD	-0.93057311	7.7522E-09
EHD2	-0.92952896	8.7693E-09
TYW3	-0.92442611	1.5609E-08
ADSS1	-0.93953563	2.4695E-09
PLEKHG3	-0.92632043	1.2662E-08
LZTS2	-0.91895845	2.7732E-08

CPNE6	-0.92036463	2.4016E-08
CHST3	-0.90300755	1.2081E-07
EXTL3	-0.91043834	6.2971E-08
DCUN1D4	-0.94092669	2.0359E-09
PTMS	-0.91469842	4.2236E-08
SIGMAR1	-0.95763737	1.2769E-10
RTL8B	-0.95169779	3.8169E-10
YDJC	0.90741373	8.2636E-08
DDX1	-0.91488976	4.1465E-08
NTPCR	-0.92627492	1.2727E-08
ACTN1	-0.92961284	8.6835E-09
EI24	-0.94424402	1.26E-09
THRA	-0.9279409	1.054E-08
TAGLN	-0.92690863	1.1852E-08
IFT81	-0.96747728	1.3962E-11
RTF2	-0.91675662	3.4564E-08
SMIM10	-0.91970188	2.5709E-08
CASC3	-0.92931774	8.9886E-09
LRCH4	0.90142839	1.3783E-07
PPP1R14A	-0.91566654	3.846E-08
TJP2	-0.91971644	2.5671E-08
DYNC1LI2	-0.95434819	2.3841E-10
GYG1	-0.94497217	1.1296E-09
PCDHGA5	-0.92740878	1.12E-08
SVIL	-0.93929604	2.5518E-09
ESD	-0.95605844	1.7334E-10
ZFYVE21	-0.95277328	3.1636E-10
HPCAL1	0.90981611	6.6644E-08
SOBP	-0.9333836	5.5093E-09
ACTN2	-0.91598743	3.7275E-08
COPS4	-0.91567277	3.8436E-08
POLR2C	-0.90680747	8.7165E-08
AOPEP	-0.9379934	3.0428E-09
TMEM47	-0.90573479	9.5712E-08
PCDHB11	-0.94295319	1.5238E-09
SNTA1	-0.92048157	2.3727E-08
DACT3	-0.9450652	1.1138E-09
MAP4	-0.90080176	1.4514E-07
HS1BP3	-0.90280315	1.2291E-07
ABCD3	-0.91897103	2.7697E-08
DAAM2	-0.91268246	5.1152E-08
CAND2	-0.91784659	3.1018E-08
FAXC	-0.93009968	8.1998E-09
CRYAB	-0.9063023	9.1104E-08
SLC5A5	0.91085305	6.0623E-08
PRKG1	-0.93005654	8.2417E-09
DUSP3	-0.92848542	9.9006E-09

KCNMB1	-0.92368387	1.6917E-08
MOCS2	-0.90837222	7.5894E-08
PDE8B	-0.92179494	2.0689E-08
WNK3	-0.92257397	1.9053E-08
LBHD1	0.91057339	6.2198E-08
FKBP7	-0.92241121	1.9385E-08
PACSIN3	-0.91991651	2.515E-08
MRFAP1	-0.92180902	2.0658E-08
THRB	-0.92086025	2.2814E-08
CLIC4	-0.93199686	6.5324E-09
AR	-0.93191739	6.5957E-09
ATP9A	-0.91033463	6.3571E-08
GPR135	-0.94269377	1.5823E-09
KIFBP	-0.90819481	7.7104E-08
UBE2D4	-0.91046485	6.2819E-08
NKIRAS1	-0.95432914	2.3924E-10
PDE3A	-0.93118896	7.2021E-09
ASIP	0.90241815	1.2694E-07
NGF	-0.90110474	1.4156E-07
SCOC	-0.94661038	8.7862E-10
FBXO8	-0.90859991	7.4365E-08
JAM3	-0.96041402	7.2448E-11
KCNQ4	-0.92304496	1.8119E-08
SNX21	-0.92381533	1.6679E-08
MTSS2	-0.93758773	3.2118E-09
MNAT1	-0.91351652	4.7281E-08
VXN	-0.90341441	1.1674E-07
PPP1R3C	-0.90746398	8.227E-08
SYNJ2BP	-0.90065795	1.4686E-07
ZCCHC14	-0.91680419	3.4402E-08
EPB41L1	-0.90433242	1.0798E-07
ARL1	-0.91249845	5.2042E-08
RRAGA	-0.93218638	6.3834E-09
TPM2	-0.91380989	4.5982E-08
PCDHGA2	-0.91042533	6.3046E-08
KCTD1	-0.90192955	1.3221E-07
RASL12	-0.91477485	4.1926E-08
DYNC1I1	-0.92689678	1.1868E-08
ASXL3	-0.91706118	3.3539E-08
AMOTL1	-0.92700014	1.173E-08
ADCY5	-0.91714044	3.3277E-08
SORBS1	-0.90943154	6.9006E-08
ARHGEF25	-0.95028538	4.8529E-10
C8orf88	-0.91906338	2.7439E-08
RBFOX2	-0.93128268	7.1214E-09
PRDM5	-0.92358228	1.7104E-08
IL17B	-0.90172402	1.3449E-07

GNA11	-0.93754245	3.2311E-09
BCL2L2	-0.94572403	1.0075E-09
SKP1	-0.9323703	6.2417E-09
PGRMC1	-0.95273934	3.1826E-10
TMEM109	-0.90275829	1.2337E-07
SLAIN2	-0.91015668	6.4611E-08
BMERB1	-0.9396328	2.4368E-09
DLG5	-0.92767003	1.0871E-08
NLGN1	-0.93865068	2.7856E-09
PCDHB4	-0.92126472	2.1872E-08
AMN	0.91658731	3.5145E-08
PHYH	-0.9498664	5.2043E-10
TMEM242	-0.92896059	9.3705E-09
NACC2	-0.94065631	2.1145E-09
GULP1	-0.91243167	5.2368E-08
FYCO1	-0.91761367	3.1748E-08
PCDH7	-0.90788122	7.9285E-08
WIPI2	0.90821907	7.6938E-08
POLR2K	-0.91511598	4.0569E-08
NFE2L1	-0.91018104	6.4468E-08
SMTN	-0.93025244	8.0529E-09
LGR4	-0.93119184	7.1996E-09
PCDHB10	-0.90355738	1.1534E-07
RGN	-0.91885174	2.8034E-08
FERMT2	-0.90497466	1.022E-07
TPPP	-0.92654669	1.2345E-08
SLC24A3	-0.90317327	1.1914E-07
AADAT	-0.90425422	1.0871E-07
NFIX	-0.94061568	2.1266E-09
HEPH	-0.91980571	2.5437E-08
SNX12	-0.90020944	1.5235E-07
BVES	-0.94231407	1.6715E-09
MYO1C	-0.90859949	7.4367E-08
PELI3	-0.90852387	7.4872E-08
ERC1	-0.93770907	3.1604E-09
TECPR2	-0.92084488	2.285E-08
TMEM181	-0.90495451	1.0238E-07
ANO1	-0.90022016	1.5222E-07
KCTD10	-0.91897555	2.7684E-08
CALD1	-0.91365393	4.6669E-08
ALDH1B1	-0.91075912	6.1148E-08
NFIC	-0.96001564	7.8777E-11
EMILIN1	-0.92839809	1.0001E-08
BAHCC1	-0.96340342	3.7549E-11
PPP2R3A	-0.92030403	2.4166E-08
BMPR1A	-0.91991384	2.5157E-08
WWTR1	-0.9207274	2.313E-08

DNAJB6	-0.90579843	9.5185E-08
DAG1	-0.91742907	3.2337E-08
RBPMS	-0.91799012	3.0576E-08
MYL9	-0.92800205	1.0466E-08
DYNC1I2	-0.93754938	3.2281E-09
VCL	-0.94774482	7.3486E-10
ARL3	-0.92008589	2.4716E-08
MSRB3	-0.9149548	4.1205E-08
BBS7	-0.90499917	1.0199E-07
SMARCA1	-0.90598736	9.3637E-08
NCKAP1	-0.92265793	1.8883E-08
WFS1	-0.9026055	1.2496E-07
PAIP1	-0.91580439	3.7947E-08
NADK2	-0.93081339	7.5333E-09
SPIRE1	-0.90975093	6.7039E-08
UBE2V2	-0.94055211	2.1455E-09
INSYN1	-0.9176305	3.1694E-08
MRGPRF	-0.92561436	1.3698E-08
SGCE	-0.90367072	1.1423E-07
NCS1	-0.92838869	1.0012E-08
DSTN	-0.92047612	2.374E-08
SHROOM3	-0.94666033	8.7181E-10
TACC2	-0.93273867	5.9661E-09
PCMT1	-0.92201622	2.0212E-08
OCRL	-0.95985579	8.145E-11
GPRASP2	-0.91528064	3.9928E-08
ATL3	-0.91749218	3.2134E-08
PDLIM3	-0.9224682	1.9268E-08
CFL2	-0.90304361	1.2045E-07
ABI2	-0.90001508	1.5479E-07
SPRYD7	-0.91888169	2.7949E-08
FAM210B	-0.90416688	1.0952E-07
CRTAP	-0.92548693	1.3892E-08
TEAD3	-0.90665134	8.8366E-08
FLNC	-0.93806042	3.0157E-09
EFS	-0.91575957	3.8113E-08
DIXDC1	-0.91237997	5.2622E-08
ATF7	-0.91150896	5.7065E-08
EID1	-0.90769554	8.0602E-08
STBD1	-0.91929729	2.6794E-08
LPP	-0.92648292	1.2434E-08
COL6A2	-0.90371472	1.1381E-07
CTNNA1	-0.93811367	2.9943E-09
ANKS1B	-0.92483559	1.4925E-08
NDN	-0.90686654	8.6715E-08
MXRA7	-0.90055204	1.4814E-07
TTLL7	-0.92104551	2.2378E-08

KCNIP3	-0.90926005	7.0082E-08
ARMCX1	-0.91668048	3.4824E-08
FLNA	-0.94140509	1.9031E-09
ATP2A2	-0.90010274	1.5369E-07
LMOD1	-0.92832329	1.0087E-08
PCDHB12	-0.90206028	1.3078E-07
SGCB	-0.92196347	2.0325E-08
MAP1A	-0.90427249	1.0854E-07
JPH2	-0.92850081	9.883E-09
PAWR	-0.93840019	2.8813E-09

List of DE mRNA whose expression correlates with PCED1B_AS1

DE mRNA	Pearson	p-value
GATA3	0.9486094	6.396E-10
IRF4	0.96906629	9.1705E-12
CXCL13	0.95239792	3.3795E-10
ICOS	0.960226	7.5377E-11
PIM2	0.97036388	6.3991E-12
CCR7	0.95847919	1.0797E-10
TMEM119	0.93437932	4.864E-09
CCR6	0.96346421	3.703E-11
SLAMF1	0.94853679	6.4716E-10
SDC1	0.95966891	8.4676E-11
KLHL42	-0.90729662	8.3495E-08
CCDC88C	0.9405707	2.14E-09
LRRC15	0.92579844	1.3421E-08
UBD	0.92666902	1.2176E-08
RUNX3	0.96498187	2.5952E-11
TAS1R3	0.94575171	1.0033E-09
GZMM	0.93246916	6.1667E-09
RHOH	0.96364582	3.5516E-11
CXCR5	0.93852743	2.8323E-09
RALGPS2	0.93516826	4.4007E-09
CD69	0.94423688	1.2613E-09
DERL3	0.96253968	4.5649E-11
RASSF6	0.94868991	6.313E-10
BHLHA15	0.94566671	1.0164E-09
C4orf50	0.92562639	1.368E-08
CXCR4	0.96355932	3.623E-11
IGLL5	0.97160287	4.4699E-12
ENSG0000027	0.9095578	6.8222E-08
CR2	0.95286335	3.1137E-10
LTB	0.97069834	5.8172E-12
CD79A	0.96014264	7.6709E-11
SLA2	0.94428002	1.2532E-09
IL21R	0.97367854	2.3613E-12
CPNE5	0.93959036	2.451E-09
GZMB	0.9469993	8.2678E-10
ALPK2	0.96486691	2.6675E-11
TIGIT	0.97271256	3.1973E-12
CYTIP	0.96150367	5.7359E-11
P2RY10	0.96787953	1.2578E-11
NPR1	-0.91668368	3.4813E-08
LTF	0.93567962	4.1215E-09
MEI1	0.96906234	9.1803E-12
CD5	0.96309726	4.0262E-11

RASSF5	0.936525	3.6939E-09
CD27	0.97054649	6.0753E-12
HSH2D	0.97032786	6.4647E-12
HLA-B	0.94289726	1.5363E-09
PYHIN1	0.96427764	3.0664E-11
FCRL3	0.97240203	3.5164E-12
NIBAN3	0.94223219	1.6913E-09
GPR183	0.90469836	1.0466E-07
PRDM1	0.94955249	5.482E-10
SHISA4	-0.93321485	5.6257E-09
BCL11B	0.95211561	3.5503E-10
SPIB	0.95430862	2.4014E-10
CD19	0.95919471	9.337E-11
BCL2L11	0.92683653	1.1949E-08
FCRL2	0.967259	1.4768E-11
C15orf48	0.90415856	1.096E-07
KLRB1	0.92332482	1.7584E-08
TCF7	0.96344381	3.7203E-11
MOXD1	0.91582077	3.7886E-08
TAGAP	0.9772832	6.8356E-13
BCL11A	0.95335946	2.8507E-10
MMP9	0.95474893	2.215E-10
MZB1	0.96190678	5.2522E-11
PNOC	0.96948691	8.1747E-12
PLD4	0.93781035	3.1181E-09
SPOCK2	0.97035875	6.4084E-12
SLAMF7	0.98016385	2.18E-13
NUGGC	0.95622931	1.678E-10
LAMP5	0.9020366	1.3104E-07
JSRP1	0.94432506	1.2449E-09
HLA-DOB	0.96577485	2.1419E-11
P2RY8	0.96535313	2.3734E-11
SH2D3A	0.97247201	3.4421E-12
LY9	0.97293896	2.981E-12
SH2D1A	0.97377471	2.2897E-12
RASGRP1	0.95409136	2.4983E-10
FCRLA	0.94602824	9.6154E-10
FDCSP	0.94674575	8.6026E-10
FCMR	0.96523192	2.444E-11
LAG3	0.921002	2.248E-08
AGAP2	0.9604529	7.1855E-11
ISG20	0.9639422	3.3162E-11
CSDC2	-0.94315315	1.48E-09
FAAH2	0.94541832	1.0557E-09
CD3E	0.98342524	4.788E-14
TRABD2A	0.95628118	1.6614E-10
ITK	0.95048034	4.6967E-10

PTPRCAP	0.97942836	2.9633E-13
CARMIL2	0.9806102	1.7993E-13
ZBP1	0.98311263	5.6062E-14
MS4A1	0.95255807	3.2859E-10
SIDT1	0.97261875	3.2909E-12
CCR4	0.92793894	1.0542E-08
LAX1	0.98030238	2.0549E-13
LCK	0.9843693	2.9178E-14
ZNF831	0.96843436	1.0867E-11
ACAP1	0.97295857	2.9629E-12
LAMP3	0.95274468	3.1796E-10
NT5C3A	0.90046064	1.4926E-07
TNFRSF13B	0.95481888	2.1866E-10
GPR18	0.92745624	1.1139E-08
NAV2	-0.92880973	9.536E-09
ARL4C	0.92295058	1.8303E-08
CLEC17A	0.952826	3.1343E-10
POU2AF1	0.97162399	4.442E-12
CD247	0.98220244	8.7321E-14
RTKN2	0.95932654	9.0878E-11
SYTL1	0.98232255	8.2471E-14
CIBAR2	0.92720163	1.1466E-08
CD3D	0.98254608	7.4072E-14
ARHGAP15	0.93113157	7.2518E-09
STAP1	0.96629453	1.884E-11
TENT5C	0.94690732	8.3879E-10
TJP3	0.95314824	2.9602E-10
NUP210	0.98137108	1.2836E-13
BLK	0.94939692	5.6243E-10
RAB11FIP4	0.97056185	6.0488E-12
NUAK2	0.94189604	1.7747E-09
IL7R	0.94728014	7.9104E-10
ENSG0000027	0.91298457	4.9719E-08
SP140	0.98026172	2.0909E-13
IL2RG	0.98556891	1.4861E-14
PLA2G2D	0.94875612	6.2455E-10
ERMN	0.95787745	1.2177E-10
CBFA2T3	0.94378014	1.3498E-09
HSPB1	-0.96850337	1.0669E-11
ZNF385D	-0.90777039	8.0069E-08
TESPA1	0.97593339	1.1114E-12
GZMK	0.95170892	3.8096E-10
LTK	0.91828927	2.9671E-08
PLCG2	0.98289571	6.2441E-14
CD226	0.97479476	1.64E-12
FAM177B	0.93207907	6.4674E-09
CD52	0.97337701	2.5987E-12

CXCR3	0.96836781	1.1061E-11
CCL5	0.93455489	4.7573E-09
TBC1D10C	0.98587854	1.2372E-14
ITGB7	0.98459399	2.5819E-14
SEMA7A	0.9692174	8.8011E-12
TNFRSF13C	0.95427773	2.415E-10
FCRL1	0.93983883	2.3687E-09
MTMR11	-0.92594544	1.3203E-08
LEF1	0.97874295	3.9062E-13
PDCD1	0.92916501	9.1502E-09
ADGRG5	0.97517232	1.4444E-12
CD1C	0.9573419	1.3533E-10
LTA	0.94599645	9.6626E-10
IKZF3	0.97640034	9.4239E-13
GPR132	0.93612522	3.891E-09
NPTXR	-0.93923425	2.5734E-09
ZAP70	0.98275373	6.6954E-14
C16orf54	0.95844448	1.0873E-10
CD48	0.97907187	3.4251E-13
NLRC3	0.979739	2.6065E-13
CD96	0.97069654	5.8202E-12
LAD1	0.93967769	2.4218E-09
TP73	0.95540054	1.9624E-10
FLT3	0.94825699	6.7703E-10
PRKCH	0.93149626	6.9406E-09
MC4R	0.9342561	4.9401E-09
SPAG4	0.93532542	4.3132E-09
SEPTIN1	0.94581276	9.9392E-10
FCRL5	0.97470474	1.6899E-12
TNFRSF9	0.9397424	2.4003E-09
TP53INP1	0.93732717	3.3245E-09
SMPDL3B	0.9577079	1.2593E-10
DLGAP5	0.92631828	1.2665E-08
CAMK4	0.92463352	1.5259E-08
PATL2	0.97079566	5.6569E-12
ANO9	0.95973554	8.3513E-11
RCSD1	0.95375861	2.6535E-10
BANK1	0.96200468	5.1403E-11
SEL1L3	0.97374405	2.3124E-12
RPS6KA6	-0.94575061	1.0034E-09
CD6	0.97286314	3.0519E-12
HLA-C	0.93124974	7.1497E-09
BTLA	0.9213781	2.1614E-08
SHISA8	0.90713811	8.4669E-08
ICOSLG	0.95816501	1.15E-10
CD40LG	0.96478723	2.7186E-11
TRAT1	0.94666734	8.7085E-10

SYNGR3	0.93835272	2.8997E-09
MYBL2	0.93192989	6.5857E-09
JAKMIP1	0.91064153	6.1811E-08
CD7	0.96992607	7.2378E-12
ARAP2	0.937015	3.4642E-09
JCHAIN	0.94789483	7.1749E-10
MAP4K1	0.9743771	1.8832E-12
CCL22	0.90723119	8.3978E-08
CD38	0.96680425	1.658E-11
MYB	0.93251252	6.134E-09
PVRIG	0.95477365	2.2049E-10
PARP15	0.9648501	2.6782E-11
P2RX5	0.93900114	2.6564E-09
RAC2	0.98297668	5.9989E-14
DPEP1	0.91792239	3.0784E-08
HLA-A	0.91651299	3.5403E-08
FOXP3	0.95524987	2.0185E-10
SIRPG	0.97493472	1.5649E-12
IL2RB	0.9583208	1.1146E-10
CTLA4	0.94713311	8.0958E-10
HS3ST3B1	0.95423121	2.4355E-10
PRKCQ	0.96780806	1.2815E-11
IPCEF1	0.94907045	5.9337E-10
CST7	0.94906158	5.9423E-10
KCNA3	0.92786466	1.0632E-08
STIM2	0.95686956	1.4837E-10
TIFAB	0.92794885	1.053E-08
LARGE2	0.92611205	1.296E-08
NUSAP1	0.94177234	1.8063E-09
GRAP2	0.96895649	9.4474E-12
DEXI	-0.93721023	3.3763E-09
PTK2B	0.97965509	2.6989E-13
VPREB3	0.93877173	2.7404E-09
XCR1	0.95810747	1.1632E-10
MYO1G	0.98363531	4.2991E-14
CARD11	0.97175247	4.2758E-12
SEMA4A	0.94022233	2.2463E-09
IL16	0.97735045	6.6671E-13
ATP10B	0.93751787	3.2417E-09
TLR10	0.94970767	5.3431E-10
TMEM156	0.96928655	8.6365E-12
SRMS	0.94351088	1.4044E-09
WNT10A	0.96499003	2.5902E-11
CD3G	0.96940917	8.3513E-12
SKAP1	0.98536615	1.672E-14
CIITA	0.97292585	2.9932E-12
SLC12A8	0.92747598	1.1114E-08

GMFG	0.97871656	3.9472E-13
GPR160	0.95576894	1.8311E-10
BEND4	0.92044905	2.3807E-08
ICAM3	0.97064687	5.9036E-12
POU2F2	0.97768887	5.873E-13
HLA-DRB5	0.92168593	2.0928E-08
B2M	0.91486494	4.1564E-08
ADH5	-0.95173658	3.7914E-10
KLHL6	0.98586305	1.2487E-14
ZBTB32	0.93694403	3.4967E-09
FCHO1	0.97171343	4.3257E-12
TRAF3IP3	0.98537863	1.66E-14
CD2	0.97958673	2.7763E-13
BIK	0.94717195	8.0464E-10
RBP5	0.9173357	3.2638E-08
ADAM8	0.95903692	9.6432E-11
PPP1R16B	0.95128792	4.0954E-10
MPZL3	0.93736398	3.3084E-09
RRM2	0.922671	1.8857E-08
ESR2	0.96204376	5.0962E-11
DEF6	0.98711718	5.6932E-15
KIF21B	0.97719124	7.0722E-13
S1PR4	0.9727736	3.1377E-12
CD83	0.94156242	1.8611E-09
MRAP2	-0.90171757	1.3456E-07
CYTH1	0.96910328	9.0788E-12
GPR174	0.96688732	1.6235E-11
EPSTI1	0.90799846	7.8464E-08
KIF1C	-0.97012126	6.8526E-12
TMEM178B	0.92333844	1.7558E-08
AIM2	0.95378536	2.6407E-10
SEMA4D	0.98277818	6.6157E-14
CCNE2	0.92633216	1.2645E-08
CLEC2B	0.90320693	1.188E-07
LIMD2	0.97418627	2.0045E-12
CLNK	0.95344232	2.8087E-10
NOL4L	0.92837785	1.0024E-08
CD74	0.97064726	5.9029E-12
RASAL3	0.98209632	9.1815E-14
PDE7A	0.97584458	1.1464E-12
CENPF	0.92519533	1.4347E-08
HEY2	-0.90062678	1.4724E-07
MKI67	0.94119204	1.9613E-09
CRTAM	0.94376331	1.3531E-09
ACADL	-0.94065016	2.1164E-09
APBA2	0.95468226	2.2424E-10
LGALS2	0.93783083	3.1096E-09

TSHR	0.93383371	5.2088E-09
PACSIN1	0.92090273	2.2713E-08
LIMS4	-0.94115343	1.972E-09
RIPOR2	0.92203612	2.017E-08
DAPP1	0.98871381	1.8589E-15
UBASH3A	0.97306251	2.8685E-12
CD37	0.97639692	9.4354E-13
LIME1	0.96373138	3.4822E-11
APOBEC3B	0.93191035	6.6014E-09
ADORA2A	0.91860888	2.8731E-08
HLA-DRB1	0.94558286	1.0295E-09
C16orf74	0.9313649	7.0513E-09
MYBPC2	0.92157958	2.1163E-08
GPR171	0.9347938	4.6155E-09
TOP2A	0.93676405	3.5802E-09
GZMA	0.93242573	6.1995E-09
TIAM1	0.96705961	1.554E-11
IDO1	0.92960103	8.6955E-09
SH2D2A	0.92657381	1.2307E-08
IL5RA	0.93227422	6.3154E-09
HLA-DQB1	0.90706597	8.5208E-08
CD22	0.93667463	3.6224E-09
ARID3A	0.9731873	2.7586E-12
TNFRSF4	0.93455313	4.7584E-09
RYR2	-0.92922049	9.0913E-09
CD1E	0.95720226	1.3907E-10
ASF1B	0.92463616	1.5255E-08
RCAN3	0.95614192	1.7061E-10
GPR141	0.91959155	2.6001E-08
SLAMF6	0.98127234	1.3422E-13
PSMB10	0.95016575	4.9511E-10
IL4I1	0.91166634	5.6239E-08
TPM3	0.91480824	4.1792E-08
CHST2	0.93493688	4.5324E-09
RHOF	0.954104	2.4926E-10
CELSR1	0.95951108	8.7487E-11
FMNL1	0.98050057	1.8869E-13
TMC8	0.9792698	3.1614E-13
AMPD1	0.92929775	9.0096E-09
SCML4	0.9560094	1.7497E-10
SHISAL2A	0.95572947	1.8448E-10
HLA-DQA1	0.94837051	6.6477E-10
ITPR3	0.96765257	1.3344E-11
OSBPL3	0.98455153	2.6426E-14
SMAP2	0.94249132	1.6293E-09
FYB1	0.97704653	7.4592E-13
KLRG1	0.93064253	7.6884E-09

BCL2A1	0.94623802	9.309E-10
GNLY	0.93895525	2.673E-09
PLCH2	0.97042826	6.2832E-12
INPP5D	0.97754672	6.1958E-13
CD70	0.92950603	8.7929E-09
IL6R	0.90396863	1.1138E-07
ITGAL	0.98581392	1.2858E-14
CORO7	0.95519623	2.0387E-10
HLA-F	0.90220678	1.292E-07
CNTNAP2	0.90783252	7.9629E-08
PAX5	0.93833281	2.9075E-09
THEMIS2	0.9398859	2.3533E-09
TBX21	0.97341372	2.5687E-12
ELMO1	0.93233279	6.2704E-09
GPSM3	0.98340629	4.8344E-14
DENND1C	0.98266181	7.0027E-14
GLCCI1	0.94509099	1.1095E-09
HMMR	0.92259422	1.9012E-08
PSD4	0.97062506	5.9405E-12
ARHGAP25	0.9770195	7.5335E-13
FBXL16	0.96162644	5.5846E-11
ABI3	0.92896806	9.3624E-09
RASGRF1	0.95597334	1.7617E-10
CHD7	0.9362335	3.8367E-09
TTN	0.90653901	8.9239E-08
KLHL14	0.9005367	1.4833E-07
CTSW	0.91851359	2.9008E-08
NKG7	0.94469369	1.178E-09
AKNA	0.95219014	3.5045E-10
SSTR3	0.94102676	2.0075E-09
KNTC1	0.94654108	8.8815E-10
EPHA1	0.96617279	1.9418E-11
GPA33	0.92181813	2.0639E-08
IKZF1	0.96383122	3.4027E-11
PLEKHG7	0.94477405	1.1638E-09
SELL	0.95549342	1.9286E-10
WAS	0.98314689	5.5109E-14
SEPTIN6	0.956042	1.7389E-10
IL27RA	0.98712139	5.6775E-15
ATAD5	0.93599873	3.9552E-09
DOCK8	0.96891694	9.5489E-12
CLEC2D	0.95555185	1.9075E-10
MCUB	0.94357769	1.3907E-09
BLM	0.97040711	6.3211E-12
ADAM28	0.97712706	7.2416E-13
UBE2C	0.92239054	1.9427E-08
CD79B	0.93903744	2.6433E-09

PTPN7	0.97966821	2.6843E-13
EVI2B	0.96902203	9.2812E-12
BUB1	0.94644649	9.013E-10
STK10	0.97409551	2.0645E-12
UBALD2	0.93676429	3.5801E-09
CFAP54	0.91521279	4.0191E-08
KLHDC7B	0.9595117	8.7476E-11
TCL1A	0.91289725	5.0129E-08
EZH2	0.92678112	1.2024E-08
LFNG	0.95444381	2.3427E-10
AURKB	0.92054963	2.3561E-08
MELK	0.90946827	6.8777E-08
MAPK13	0.9561767	1.6949E-10
CNR2	0.9259441	1.3205E-08
PBX4	0.91935554	2.6635E-08
PTPRC	0.95488841	2.1586E-10
HLA-DRA	0.96398153	3.286E-11
CCDC88B	0.97525327	1.4052E-12
MX2	0.92432571	1.578E-08
MAN2B1	0.94099306	2.017E-09
SLC37A1	0.91493426	4.1287E-08
PRKCB	0.91028188	6.3877E-08
ACOXL	0.92182122	2.0632E-08
ZNF93	0.90976023	6.6982E-08
ABCB1	0.92195104	2.0352E-08
KIFC1	0.93517723	4.3957E-09
GCSAM	0.958727	1.027E-10
BIN2	0.9857471	1.3379E-14
KCNN3	0.9222452	1.9729E-08
CSF2RA	0.97859331	4.1441E-13
NLRC5	0.9511362	4.2029E-10
ARHGAP9	0.98236679	8.0745E-14
ADRA2A	0.9065514	8.9143E-08
RAB37	0.95457892	2.2854E-10
GIPR	0.93386247	5.1901E-09
APOBEC3H	0.91496917	4.1148E-08
TNFRSF1B	0.90531759	9.9228E-08
CDK5R1	0.9157192	3.8263E-08
RGL4	0.94331016	1.4464E-09
ARHGAP45	0.97709028	7.3403E-13
CCDC18	0.92823077	1.0195E-08
FAM181B	-0.92448117	1.5515E-08
PCLAF	0.90642106	9.0164E-08
SFMBT2	0.96164329	5.5641E-11
CD244	0.90357414	1.1517E-07
SNX20	0.97660389	8.7609E-13
KLRK1	0.94267176	1.5874E-09

PASK	0.96210203	5.0311E-11
TNFSF13B	0.95643405	1.6135E-10
LPXN	0.97757243	6.1363E-13
CEACAM21	0.92425339	1.5905E-08
TSPAN13	0.93724717	3.3599E-09
STAT4	0.94613601	9.4569E-10
IRAG2	0.97214447	3.802E-12
RNASET2	0.96939236	8.3899E-12
NECTIN4	0.91623828	3.6371E-08
ARHGAP27	0.97300032	2.9246E-12
JAML	0.96725863	1.477E-11
GRAMD1B	0.93287594	5.8662E-09
MATK	0.93188458	6.6221E-09
TIGD3	0.92998554	8.311E-09
IRF8	0.96019372	7.5891E-11
BTK	0.97057333	6.029E-12
BID	0.95998907	7.9216E-11
CENPM	0.95417903	2.4588E-10
ACTC1	-0.90639635	9.0359E-08
HLA-DMA	0.97706744	7.4022E-13
NCAPH	0.90398223	1.1125E-07
CRYBG1	0.9470751	8.17E-10
STK17B	0.94482906	1.1543E-09
LYZ	0.93863962	2.7898E-09
NCKAP1L	0.94963382	5.4088E-10
LYN	0.93380617	5.2268E-09
COTL1	0.92174696	2.0794E-08
SYK	0.96601754	2.0178E-11
HLA-DPB1	0.9557427	1.8402E-10
CD8A	0.90280178	1.2292E-07
ARHGAP30	0.98115835	1.4126E-13
VAV1	0.97547492	1.3027E-12
PIK3CD	0.95167331	3.833E-10
TNFRSF10A	0.92790215	1.0587E-08
GPRIN3	0.93490107	4.5531E-09
SGO1	0.9512189	4.144E-10
MARCKS	0.91650717	3.5424E-08
TMEM163	0.94854159	6.4665E-10
ZC3H12D	0.9778091	5.6116E-13
AIF1	0.91996506	2.5025E-08
GAPT	0.94367936	1.37E-09
HOOK1	0.91426923	4.4011E-08
CYBA	0.98456362	2.6252E-14
SPN	0.97537145	1.3497E-12
ABCA7	0.96872604	1.0052E-11
TNFRSF18	0.95782228	1.2311E-10
PRF1	0.91081702	6.0824E-08

CD72	0.94276991	1.5649E-09
ESPL1	0.90450425	1.0641E-07
GPR55	0.93236218	6.2479E-09
SAPCD2	0.94049497	2.1627E-09
TTC39C	0.91518937	4.0282E-08
LCP2	0.956506	1.5914E-10
REEP2	-0.91305412	4.9394E-08
PLEK	0.95565854	1.8696E-10
TTC17	0.90499561	1.0202E-07
DOCK2	0.96347767	3.6915E-11
ZNF217	0.90985963	6.6381E-08
CLMN	0.92894062	9.3923E-09
FKBP11	0.94660017	8.8002E-10
TLR9	0.93629159	3.8079E-09
GRAP	0.93317245	5.6553E-09
NCAPG	0.914318	4.3806E-08
ALOX5	0.96846091	1.0791E-11
ADAMDEC1	0.92891046	9.4252E-09
TAP1	0.91752884	3.2017E-08
CCR5	0.94420886	1.2666E-09
CD8B	0.92594702	1.3201E-08
TACC3	0.96788385	1.2564E-11
RUNX2	0.9268181	1.1974E-08
SGSM1	-0.90436184	1.0771E-07
LSP1	0.95880475	1.011E-10
TTC22	0.95813452	1.157E-10
PRR11	0.92888816	9.4497E-09
SNAPC3	-0.91342298	4.7702E-08
SLFN12L	0.9615121	5.7254E-11
PTPN22	0.96653484	1.7743E-11
HLA-DOA	0.96140289	5.8628E-11
EXOC3L4	0.91461355	4.2582E-08
TMC6	0.97173194	4.302E-12
FGD3	0.97769354	5.8626E-13
SH3KBP1	0.95052488	4.6616E-10
DTHD1	0.92267741	1.8844E-08
IGFLR1	0.98135109	1.2953E-13
ZNF296	0.94135027	1.9179E-09
CORO1A	0.97441771	1.8582E-12
CBX4	0.95512819	2.0647E-10
PLAC8	0.94356956	1.3923E-09
LINGO3	0.92389706	1.6532E-08
JAK3	0.97448304	1.8187E-12
CFLAR	0.91750402	3.2096E-08
TMEM154	0.95776158	1.246E-10
GBP5	0.93712486	3.4145E-09
CRACR2A	0.94631729	9.1955E-10

LAPTM5	0.94404635	1.2976E-09
KNL1	0.92635843	1.2608E-08
HLA-DMB	0.96183072	5.3406E-11
CNTN4	-0.95719234	1.3934E-10
FLI1	0.93698486	3.478E-09
HJURP	0.91433386	4.3739E-08
CTSH	0.97184466	4.1599E-12
CDK1	0.9117149	5.5986E-08
ICA1	0.90801519	7.8347E-08
SELPLG	0.96523738	2.4407E-11
ANGPTL6	0.94003181	2.3064E-09
SH3BP1	0.95544364	1.9466E-10
NFKBIE	0.92164329	2.1022E-08
DBF4	0.94374654	1.3565E-09
LSR	0.90285555	1.2237E-07
HLA-DPA1	0.9438051	1.3448E-09
ARHGAP4	0.96643081	1.8211E-11
PTPN2	0.91462046	4.2553E-08
TXK	0.92674635	1.2071E-08
UCP2	0.95027004	4.8654E-10
CLEC9A	0.91457905	4.2723E-08
CDCA2	0.91239103	5.2568E-08
NDC80	0.93605665	3.9257E-09
PTPN6	0.97623339	1E-12
CCNB2	0.91116315	5.8917E-08
RASGRP3	0.9344868	4.7984E-09
THEMIS	0.93056166	7.7628E-09
ST8SIA4	0.9401614	2.2654E-09
GTSE1	0.91270349	5.1051E-08
NCF1	0.96484165	2.6836E-11
PLCB2	0.96347214	3.6962E-11
STK4	0.94983103	5.2349E-10
SAMD3	0.93339691	5.5002E-09
SASH3	0.96661179	1.7404E-11
NEK2	0.90652511	8.9348E-08
ADM2	0.96016443	7.6359E-11
BUB1B	0.92739083	1.1222E-08
ACSL5	0.95858924	1.056E-10
FERMT3	0.96128258	6.0175E-11
NFE2L3	0.95870546	1.0315E-10
CLEC4A	0.93269972	5.9948E-09
MARCKSL1	0.90043976	1.4951E-07
VNN2	0.92902889	9.2964E-09
HCN2	0.9543456	2.3852E-10
RNF166	0.95178382	3.7606E-10
GMIP	0.96899484	9.3498E-12
PSMB9	0.96989368	7.3035E-12

HCLS1	0.95683718	1.493E-10
ATP2A3	0.95783835	1.2272E-10
MAP3K9	0.90000395	1.5493E-07
DOK3	0.97564962	1.2267E-12
MDK	0.90192738	1.3224E-07
LRRK1	0.95568643	1.8598E-10
IL10RA	0.95613259	1.7092E-10
PRKCZ	0.94994496	5.1368E-10
RNF44	0.95745256	1.3242E-10
KMO	0.96278398	4.3216E-11
STAMBPL1	0.93930769	2.5477E-09
WDFY4	0.96433802	3.0233E-11
LPAR2	0.94113188	1.978E-09
SLC31A2	0.90138435	1.3833E-07
TNFRSF17	0.94366491	1.3729E-09
SP110	0.94833195	6.6891E-10
PAG1	0.96316032	3.969E-11
ZNF267	0.90849097	7.5093E-08
MICB	0.94491892	1.1387E-09
TASL	0.97159051	4.4863E-12
ANXA2R	0.96401837	3.258E-11
PIK3CG	0.93911327	2.6162E-09
ARRDC5	0.91787957	3.0916E-08
LGALS9	0.94790499	7.1633E-10
ODF3B	0.90449653	1.0648E-07
FANCD2	0.94717193	8.0464E-10
ITGA4	0.92988754	8.4076E-09
SH3TC1	0.92403614	1.6285E-08
TAF6	-0.90877116	7.3232E-08
LST1	0.96968199	7.7461E-12
PLAG1	0.93383594	5.2074E-09
TBXAS1	0.93250429	6.1402E-09
TTC24	0.94179978	1.7993E-09
BLNK	0.96506225	2.5457E-11
PRKX	0.95828488	1.1227E-10
CALHM6	0.90577899	9.5346E-08
RPS6KA1	0.96429718	3.0524E-11
CPNE7	0.90227375	1.2848E-07
TRERF1	0.92536209	1.4085E-08
ITGB1BP2	-0.90649406	8.9591E-08
CNTRL	0.92390794	1.6512E-08
DPEP2	0.96358335	3.603E-11
PARVG	0.96951812	8.1048E-12
CKAP2L	0.91458654	4.2692E-08
CDH1	0.90224765	1.2876E-07
LCP1	0.95425381	2.4255E-10
BMF	0.91866435	2.857E-08

DRAM2	0.93384996	5.1983E-09
ARMH1	0.92590681	1.326E-08
ZNF710	0.91846298	2.9157E-08
HCST	0.94591452	9.7851E-10
OXNAD1	0.93588609	4.0132E-09
HS3ST3A1	0.96646353	1.8062E-11
CCDC138	0.91096045	6.0027E-08
AOAH	0.91941816	2.6466E-08
ILDR1	0.91655922	3.5243E-08
SLC25A23	-0.93677905	3.5732E-09
UNC13D	0.97556361	1.2636E-12
CYTH4	0.95445808	2.3366E-10
E2F2	0.95882156	1.0075E-10
CD86	0.91652569	3.5359E-08
ZNF576	-0.90517809	1.0043E-07
FBXW7	0.92679722	1.2002E-08
PIK3R6	0.90923089	7.0267E-08
PSMB8	0.96067036	6.8616E-11
SIT1	0.97249289	3.4202E-12
TNFSF8	0.9365988	3.6584E-09
RASSF2	0.93114729	7.2382E-09
VAMP8	0.95593737	1.7737E-10
TDP1	0.93650584	3.7031E-09
CD300A	0.93193637	6.5805E-09
RP11-316O14	-0.91036634	6.3387E-08
EVI2A	0.93954741	2.4655E-09
SIGLEC10	0.94492191	1.1382E-09
HELZ2	0.91193103	5.4873E-08
ZBTB46	0.92406749	1.6229E-08
NCF4	0.95136005	4.0451E-10
GFI1	0.96050565	7.1057E-11
RGS14	0.94887864	6.1223E-10
ZNF814	0.92078428	2.2994E-08
ANKRD44	0.90003152	1.5458E-07
TPD52	0.91897903	2.7675E-08
CMTM7	0.96439723	2.9815E-11
DENND2D	0.95213867	3.536E-10
LILRB1	0.93905132	2.6383E-09
MCM5	0.93686742	3.532E-09
VSIG1	0.92633548	1.2641E-08
FCGR2B	0.92030195	2.4171E-08
CASP10	0.9654248	2.3326E-11
TMPRSS13	0.96973094	7.6417E-12
FHDC1	0.90350778	1.1582E-07
RND2	-0.91753442	3.1999E-08
SPNS3	0.92329601	1.7638E-08
KCNN4	0.91146757	5.7284E-08

ABHD17C	0.9148403	4.1663E-08
STXBP2	0.9731463	2.7943E-12
ISL2	0.94059417	2.133E-09
RNF125	0.92095878	2.2581E-08
TNFSF15	0.92622064	1.2804E-08
MTMR14	0.93765672	3.1825E-09
CLEC7A	0.91799154	3.0571E-08
JARID2	0.93376118	5.2562E-09
NME8	0.92183938	2.0593E-08
RNASE6	0.93423913	4.9506E-09
CD4	0.94244911	1.6393E-09
ASPM	0.92097915	2.2533E-08
AMPD3	0.95031029	4.8327E-10
TLR6	0.96158331	5.6374E-11
NELL2	0.91831294	2.9601E-08
C2	0.91543191	3.9347E-08
KBTBD8	0.92867281	9.6885E-09
IFNLR1	0.9606316	6.9184E-11
CIAO2A	0.96755042	1.3701E-11
MOB3A	0.96569005	2.1868E-11
KLK1	0.93084865	7.5017E-09
GNG2	0.90266741	1.2431E-07
CD1D	0.9570863	1.4225E-10
GIMAP4	0.92714731	1.1537E-08
KYNU	0.94342687	1.4219E-09
GRIK5	-0.95167463	3.8322E-10
TAP2	0.92680775	1.1988E-08
NABP1	0.90288764	1.2204E-07
NR2F6	-0.90873623	7.3462E-08
TREML2	0.93240926	6.212E-09
CLSPN	0.90469778	1.0466E-07
APOBEC3D	0.93987249	2.3577E-09
IL12RB1	0.96994609	7.1974E-12
CYB561D2	0.93924939	2.5681E-09
MFNG	0.96332411	3.8236E-11
KIF18B	0.90819773	7.7084E-08
N4BP3	0.91434359	4.3698E-08
GIMAP5	0.90240605	1.2706E-07
CCNA2	0.92575708	1.3483E-08
FAM78A	0.9521621	3.5216E-10
SLC30A7	0.92218023	1.9865E-08
IL18	0.95973306	8.3557E-11
ATG9B	0.91167182	5.621E-08
ITGA7	-0.95533778	1.9856E-10
NETO2	0.90708144	8.5092E-08
ZWINT	0.9210531	2.236E-08
EAF2	0.91208798	5.4077E-08

STAC3	0.91318357	4.8795E-08
CENPK	0.91136155	5.7848E-08
RESF1	0.90812651	7.7575E-08
POLQ	0.93008508	8.2139E-09
RHBDF2	0.97237204	3.5486E-12
LAT2	0.96174865	5.4375E-11
SP100	0.92487699	1.4858E-08
SPEG	-0.96446127	2.9369E-11
ITGAX	0.91766113	3.1598E-08
GUCY2D	0.93657528	3.6697E-09
CRLF3	0.94155955	1.8619E-09
REEP4	0.90451677	1.0629E-07
CCR2	0.92841676	9.9793E-09
ARHGDIB	0.96077144	6.7155E-11
CD53	0.9713705	4.7867E-12
ARHGAP19	0.92245742	1.929E-08
ABRACL	0.93606099	3.9235E-09
PEF1	-0.96340669	3.7521E-11
FANCA	0.92320672	1.7808E-08
FUT7	0.93456392	4.7519E-09
RHBDF1	-0.91292285	5.0009E-08
VAV3	0.9437062	1.3646E-09
ELAPOR1	0.90107759	1.4188E-07
RCC2	0.96986879	7.3544E-12
EVL	0.91615044	3.6685E-08
MIS18BP1	0.90825432	7.6696E-08
ADPGK	0.94598755	9.6758E-10
RGS10	0.92346874	1.7314E-08
CD80	0.91436476	4.361E-08
WDR54	0.93877945	2.7375E-09
UNC93B1	0.95749731	1.3126E-10
TAPBP	0.9316969	6.7744E-09
AP1G2	0.91244574	5.2299E-08
FNDC10	0.93290618	5.8444E-09
PIK3AP1	0.91136886	5.7809E-08
RHEBL1	0.94272342	1.5755E-09
PLCXD2	0.90806352	7.8011E-08
SCUBE3	-0.9309184	7.4394E-09
SCAMP5	0.95317422	2.9465E-10
ORAI1	0.92920335	9.1094E-09
GEN1	0.93140263	7.0193E-09
CORO2A	0.94219436	1.7005E-09
RINL	0.96987423	7.3432E-12
SH3BGR	-0.93685324	3.5386E-09
FAM111B	0.9064964	8.9572E-08
TMEM273	0.93373233	5.2752E-09
SNX10	0.94149815	1.8782E-09

MARCHF1	0.91889776	2.7904E-08
CAMSAP3	0.94385753	1.3344E-09
STRBP	0.91656793	3.5212E-08
NCR3	0.92359507	1.708E-08
MYCL	0.9263495	1.2621E-08
APOBR	0.93375136	5.2627E-09
KCTD2	-0.92442911	1.5604E-08
CCDC78	0.92684931	1.1932E-08
CD151	-0.90263063	1.247E-07
KCNAB3	0.91136742	5.7817E-08
APOBEC3G	0.96834645	1.1124E-11
ARHGEF1	0.90898317	7.1851E-08
FGR	0.93566802	4.1277E-09
MAP3K1	0.92472371	1.5109E-08
SGPP2	0.93941164	2.5118E-09
TYMP	0.90340072	1.1687E-07
ITGB2	0.94953387	5.4988E-10
TTC7A	0.93224091	6.3411E-09
MCTP2	0.96058439	6.9881E-11
HSF5	0.94119343	1.9609E-09
CYP2S1	0.9178404	3.1037E-08
BTN3A3	0.95072224	4.5089E-10
GIMAP2	0.94527737	1.0786E-09
PAFAH1B3	0.90578862	9.5266E-08
SMPD3	0.93262474	6.0502E-09
MAGED2	-0.92414305	1.6097E-08
SNAI3	0.96326312	3.8772E-11
MYO1F	0.94262226	1.5988E-09
GTSF1	0.91324932	4.8492E-08
TSPAN33	0.96789664	1.2522E-11
FASLG	0.92890415	9.4321E-09
TOGARAM2	0.90471045	1.0455E-07
TNNI2	0.92376005	1.6779E-08
KLHL30	-0.91794312	3.072E-08
SLC41A1	-0.92034918	2.4054E-08
NFAM1	0.91366081	4.6638E-08
SLC2A5	0.92830234	1.0112E-08
UTP18	0.91158943	5.6641E-08
ASPHD2	0.95494422	2.1364E-10
PTAFR	0.93896218	2.6705E-09
SRC	-0.91050528	6.2587E-08
FAM110A	0.94297131	1.5198E-09
TCEAL2	-0.92504647	1.4584E-08
BTC	-0.96305305	4.0668E-11
GIMAP1	0.92663157	1.2228E-08
SPRYD3	-0.92350568	1.7245E-08
CLSTN3	0.94015652	2.2669E-09

RCC1L	-0.91103926	5.9593E-08
PYCARD	0.93507235	4.4549E-09
CYRIB	0.90790203	7.9139E-08
OPN3	0.9430629	1.4996E-09
ATM	0.9195814	2.6028E-08
USP3	0.92975367	8.541E-09
NBEAL2	0.94789614	7.1734E-10
KCNAB2	0.96215057	4.9774E-11
CLECL1	0.94026334	2.2335E-09
PRAM1	0.90369568	1.1399E-07
ASCL2	0.91490314	4.1411E-08
SPAG5	0.91581908	3.7892E-08
NSMF	-0.91778528	3.1209E-08
CXCR6	0.91435879	4.3635E-08
TFEC	0.93117541	7.2138E-09
PLN	-0.95893562	9.8444E-11
MTRF1L	-0.92563527	1.3666E-08
GGA2	0.90877002	7.324E-08
BEX3	-0.9053858	9.8646E-08
ARL2	-0.94241365	1.6477E-09
GRK2	0.92886401	9.4762E-09
BICDL1	0.94326831	1.4553E-09
TCEAL7	-0.90903202	7.1536E-08
AGMAT	0.9305028	7.8173E-09
SPI1	0.93740813	3.2891E-09
ADRA1D	-0.95596136	1.7657E-10
APBB1IP	0.94479264	1.1606E-09
SH3BP2	0.96491723	2.6357E-11
CREB3	-0.92992195	8.3735E-09
GSAP	0.93881448	2.7245E-09
E2F5	0.90732663	8.3274E-08
HSPA4L	-0.92556864	1.3767E-08
EBI3	0.9160221	3.7149E-08
GPR65	0.9225267	1.9149E-08
SCIMP	0.9335087	5.4243E-09
SCRG1	-0.93699524	3.4732E-09
CTSS	0.92156967	2.1185E-08
NPR2	-0.93078907	7.5552E-09
SNX8	0.94116469	1.9689E-09
CD180	0.96385608	3.3831E-11
ARRB2	0.90220618	1.292E-07
GRIA2	-0.91267779	5.1174E-08
GSTK1	0.92422709	1.5951E-08
NUDT9	-0.93124659	7.1524E-09
TMEM266	0.91523084	4.0121E-08
GPR19	0.92309838	1.8016E-08
FUT8	0.91396185	4.5322E-08

ADRA2C	-0.96586253	2.0963E-11
ZNF101	0.9351396	4.4168E-09
FGD2	0.95788361	1.2162E-10
TRIM14	0.92510398	1.4492E-08
NTN1	-0.92723805	1.1419E-08
KISS1R	0.90001519	1.5479E-07
TMEM229B	0.92632315	1.2658E-08
MYOCD	-0.96772327	1.3101E-11
SYNPO	-0.91690248	3.407E-08
PARPBP	0.92653306	1.2364E-08
FRMD8	0.90877215	7.3226E-08
RUBCNL	0.94711771	8.1154E-10
PPME1	-0.90193212	1.3219E-07
ACRBP	0.93389165	5.1712E-09
LRRC8A	-0.91886993	2.7982E-08
ACY3	0.92084978	2.2838E-08
BTN3A1	0.95198319	3.6329E-10
AP1S3	0.90871215	7.3621E-08
GIMAP7	0.94325722	1.4577E-09
METAP1	-0.91632488	3.6064E-08
CSK	0.91487154	4.1537E-08
COL18A1	-0.90393922	1.1166E-07
GRAMD4	0.93056078	7.7636E-09
LIMK1	0.94181661	1.795E-09
LRRC8D	0.94014151	2.2716E-09
CSTA	0.94172029	1.8198E-09
GNGT2	0.9265529	1.2336E-08
MGAT3	0.94973617	5.3179E-10
UNC45A	-0.95606813	1.7303E-10
MYD88	0.90164873	1.3533E-07
TENT5B	-0.94140429	1.9033E-09
RUSC1	-0.93043488	7.8806E-09
SLC15A3	0.90397792	1.1129E-07
RRAS	-0.9346208	4.7178E-09
LURAP1	-0.92550388	1.3866E-08
ZNF215	0.91255431	5.177E-08
RTL8C	-0.92668604	1.2153E-08
MSRB2	-0.9559053	1.7845E-10
HDAC11	-0.96160637	5.6091E-11
CMAS	-0.92473884	1.5084E-08
NGRN	-0.97373726	2.3174E-12
RIPK3	0.93670246	3.6092E-09
AKT1	-0.91983317	2.5366E-08
ETNK2	-0.95849616	1.0761E-10
ITGAM	0.90813414	7.7522E-08
ADCY6	-0.9318527	6.6477E-09
TOR2A	0.92736825	1.1251E-08

CCDC107	-0.92395847	1.6422E-08
CNDP2	0.92508272	1.4526E-08
RMI2	0.90377886	1.1319E-07
TMEM106C	-0.94409469	1.2883E-09
H2AJ	-0.94209448	1.725E-09
MREG	0.91971733	2.5669E-08
SSH2	0.92840469	9.9932E-09
RAVER2	-0.93283225	5.8978E-09
CENPB	-0.94015876	2.2662E-09
CD84	0.90945589	6.8854E-08
ATXN7L1	0.93989819	2.3493E-09
KLC2	-0.91871467	2.8425E-08
SPEGNB	-0.92912671	9.1912E-09
SLF1	0.90812768	7.7567E-08
TNFAIP8L1	0.9268644	1.1911E-08
CASP1	0.92988816	8.4069E-09
PIK3R5	0.92336857	1.7501E-08
TMEM120A	-0.93824765	2.941E-09
VNN1	0.90525905	9.9731E-08
IRF5	0.95041624	4.7475E-10
RENBP	0.90583542	9.488E-08
DYNLL2	-0.93837835	2.8898E-09
OAS2	0.9087296	7.3506E-08
ADA2	0.93263793	6.0404E-09
HAGHL	0.93600832	3.9503E-09
AMMECR1	0.92837235	1.003E-08
JPT1	0.92864125	9.7239E-09
CKLF	0.92345411	1.7341E-08
PARP10	0.90059399	1.4763E-07
RASAL1	0.91507544	4.0728E-08
SPATA13	0.93053501	7.7874E-09
SARDH	0.91651645	3.5391E-08
DENND6B	0.91218126	5.3608E-08
TTLL11	-0.91796663	3.0648E-08
GALNT6	0.91934152	2.6673E-08
PTGIR	-0.91878351	2.8228E-08
FAM131A	-0.91331021	4.8214E-08
ZNF532	-0.94059946	2.1314E-09
EVI5L	-0.91137803	5.776E-08
ROM1	-0.91490315	4.1411E-08
TNFRSF8	0.90307947	1.2008E-07
CLIP2	-0.91768317	3.1528E-08
S1PR2	0.91844146	2.922E-08
SOWAHD	0.90326785	1.1819E-07
CAMK1	-0.92105865	2.2347E-08
SLC16A13	0.91125203	5.8436E-08
TRIP6	-0.94198623	1.752E-09

DMTN	-0.93034365	7.9664E-09
TTC16	0.93533127	4.3099E-09
C19orf38	0.92179785	2.0683E-08
PRDM11	-0.9224567	1.9292E-08
CDK5RAP2	-0.92742654	1.1177E-08
ERBB2	-0.92999705	8.2997E-09
SP140L	0.92149732	2.1346E-08
MORN3	0.93927469	2.5592E-09
SS18L2	0.95050316	4.6787E-10
LINS1	0.93914532	2.6048E-09
CHD5	-0.90778115	7.9992E-08
EXOC7	-0.9337287	5.2776E-09
CAPZA1	0.94267168	1.5874E-09
TMEM25	-0.95166644	3.8376E-10
NDST2	0.91127499	5.8312E-08
FBXO46	0.90583646	9.4872E-08
AQP3	0.92488412	1.4846E-08
CCNI2	0.93606503	3.9214E-09
COPS6	-0.91837128	2.9427E-08
EDEM2	0.91447491	4.3152E-08
CDIPT	-0.91680437	3.4401E-08
PIGR	0.90339409	1.1694E-07
DENND4B	0.91241227	5.2463E-08
SOD1	-0.95430301	2.4038E-10
SLAMF8	0.91796747	3.0645E-08
DOK2	0.92612071	1.2948E-08
SNX5	0.91852312	2.8981E-08
SLC12A4	-0.92516169	1.44E-08
RNF213	0.91740798	3.2405E-08
CCDC136	-0.92528543	1.4205E-08
SFXN1	0.9563346	1.6445E-10
GIMAP6	0.9083169	7.6269E-08
LRRC47	-0.92209069	2.0054E-08
SERGEF	-0.92037612	2.3987E-08
IL10RB	0.90422828	1.0895E-07
LY86	0.93807439	3.01E-09
HACD1	-0.95960799	8.5752E-11
C11orf21	0.93982141	2.3743E-09
LY96	0.93377312	5.2484E-09
PRKD2	0.94360482	1.3851E-09
FAM53B	0.93548359	4.2266E-09
TNS4	0.91131174	5.8115E-08
AQP1	-0.90580398	9.5139E-08
KDM2B	0.92926854	9.0404E-09
PIP5K1C	-0.97579054	1.1682E-12
TNFAIP8L2	0.91336264	4.7975E-08
ZNF853	-0.95285454	3.1185E-10

ZNRF3	-0.95786242	1.2213E-10
MCOLN2	0.92134484	2.1689E-08
LAIR1	0.90062495	1.4726E-07
IFNAR2	0.96998828	7.113E-12
RUSC2	-0.9131132	4.912E-08
SLC45A1	-0.92995812	8.3379E-09
ANO10	-0.91594625	3.7425E-08
KPNA6	-0.92930459	9.0025E-09
SV2A	-0.92716769	1.151E-08
MTFR2	0.92782632	1.0679E-08
RAMP1	-0.92810206	1.0347E-08
C19orf54	0.93532987	4.3107E-09
ORAI2	0.90087012	1.4432E-07
KMT5A	-0.9228793	1.8443E-08
DDX60L	0.92057471	2.3499E-08
CARD8	0.93994359	2.3347E-09
SYDE1	-0.94076307	2.0832E-09
NUP50	0.91194052	5.4824E-08
GRK5	-0.91699913	3.3746E-08
ARHGEF17	-0.96340848	3.7505E-11
NKD1	-0.94284926	1.547E-09
TMEM74	-0.91617586	3.6594E-08
LRP5	-0.91934134	2.6674E-08
CASP8	0.94532044	1.0715E-09
E2F7	0.90550344	9.7648E-08
PKD1	-0.92000187	2.493E-08
NRSN2	-0.91938221	2.6563E-08
NUDCD3	-0.95489372	2.1565E-10
TLCD4	-0.9354032	4.2704E-09
HENMT1	0.91370575	4.644E-08
ZDHHC13	0.92420619	1.5987E-08
MANSC1	-0.90254851	1.2556E-07
CALML4	0.91434688	4.3685E-08
LYNX1-SLURP	-0.90651841	8.94E-08
CBY1	-0.90130654	1.3922E-07
TOM1L2	-0.97481917	1.6267E-12
CAVIN3	-0.90342934	1.1659E-07
MRPS18A	-0.91151314	5.7043E-08
CCDC9B	-0.92065244	2.3311E-08
SARS1	-0.91251721	5.1951E-08
HADH	-0.95349235	2.7836E-10
ADCY9	-0.9185712	2.884E-08
NOL7	-0.90733383	8.3221E-08
AKTIP	-0.94297647	1.5187E-09
SLC2A4RG	-0.91654277	3.53E-08
DQX1	0.91903476	2.7519E-08
TRIM55	0.9226211	1.8958E-08

RHBDL2	0.90846523	7.5266E-08
MTCH1	-0.92130058	2.179E-08
RAB39B	0.90034799	1.5064E-07
PFN2	-0.93835028	2.9007E-09
LTB4R	0.91235052	5.2767E-08
RILPL1	-0.95379354	2.6368E-10
PARP14	0.92778266	1.0733E-08
FAM83H	0.91925262	2.6916E-08
RAB11FIP3	-0.94124554	1.9465E-09
MGST3	-0.92125744	2.1889E-08
KIAA0319L	0.90605536	9.3085E-08
ADCY7	0.90876428	7.3278E-08
FAM219B	-0.90623801	9.1616E-08
ARL11	0.90577109	9.5411E-08
DLGAP4	-0.95073552	4.4988E-10
CNNM1	-0.90075004	1.4575E-07
DGCR2	-0.93392668	5.1486E-09
FEZ1	-0.93569542	4.1131E-09
ENDOD1	-0.93679536	3.5656E-09
PRMT2	-0.93354624	5.399E-09
RAB1B	-0.92432485	1.5782E-08
VSTM4	-0.90344858	1.164E-07
WIZ	-0.94493069	1.1367E-09
DAB2IP	-0.95696103	1.4576E-10
GORASP1	-0.91212768	5.3877E-08
PAM	-0.91426784	4.4016E-08
RORC	0.90242671	1.2684E-07
LRTOMT	-0.93028665	8.0204E-09
RAB8A	0.91428181	4.3958E-08
SLC25A4	-0.9526508	3.2327E-10
SPATC1	0.90101146	1.4265E-07
SNAPIN	-0.92840415	9.9938E-09
PLPPR2	-0.90004691	1.5439E-07
CEP170B	-0.93819606	2.9614E-09
TRPC4	-0.9347177	4.6603E-09
ILK	-0.93882123	2.7221E-09
MANBAL	-0.97198796	3.9853E-12
BAG2	-0.9408831	2.0484E-09
PCDH20	-0.97008288	6.9269E-12
MFGE8	-0.93133805	7.0741E-09
RPRD1A	-0.92952377	8.7746E-09
TWSG1	-0.90424493	1.0879E-07
PLXNB1	-0.95286627	3.1121E-10
ANKRD27	-0.91578985	3.8001E-08
IGIP	-0.91170596	5.6032E-08
APOBEC3F	0.92555016	1.3796E-08
MRPL33	-0.95123247	4.1344E-10

PGAP4	-0.91701294	3.37E-08
PCBP4	-0.93459569	4.7329E-09
SUCLA2	-0.9012305	1.401E-07
NT5C3B	-0.9238924	1.654E-08
DLG3	-0.96060305	6.9605E-11
FBLIM1	-0.94424938	1.259E-09
SLC48A1	-0.94658246	8.8245E-10
B3GNT3	0.90902762	7.1565E-08
TMEM8B	-0.94234229	1.6647E-09
MRPL43	-0.91748871	3.2145E-08
TOLLIP	-0.95891151	9.8928E-11
ACSS2	-0.93614964	3.8787E-09
ADI1	-0.91566762	3.8456E-08
SMARCD3	-0.93938244	2.5218E-09
DMPK	-0.91344031	4.7624E-08
RTL5	-0.94749493	7.6462E-10
CYB5R1	-0.94086994	2.0522E-09
RAB34	-0.93458941	4.7366E-09
SRPK3	-0.93561013	4.1585E-09
SMS	-0.90579899	9.5181E-08
ARMC10	-0.90451832	1.0628E-07
DBNDD2	-0.96021177	7.5603E-11
RNF121	-0.90694789	8.6098E-08
NEO1	-0.90619907	9.1927E-08
AOC3	-0.95902911	9.6586E-11
BTBD6	-0.92323625	1.7752E-08
CEP85	0.93749067	3.2534E-09
KIF7	-0.91662233	3.5024E-08
DNAJB5	-0.94034829	2.2073E-09
DCTN1	-0.98043297	1.9428E-13
PRPF40B	-0.92633778	1.2637E-08
FANCG	0.90221815	1.2907E-07
AKAP1	-0.97802957	5.1589E-13
CYBC1	0.93884794	2.7122E-09
CYB5R3	-0.93163235	6.8274E-09
TSPAN32	0.91403597	4.5002E-08
IGFBP7	-0.91297245	4.9776E-08
NACAD	-0.94358537	1.3891E-09
ITPRIPL1	0.93641955	3.745E-09
LTBP3	-0.90576886	9.543E-08
FGF13	-0.92189174	2.0479E-08
CASP2	0.90123914	1.4E-07
SELENOM	-0.92235265	1.9506E-08
LIMS2	-0.94885845	6.1425E-10
RIC8B	-0.90891292	7.2306E-08
KCNE2	-0.92896044	9.3707E-09
MAP6	-0.9380831	3.0065E-09

PFKP	-0.95853122	1.0685E-10
ABCD1	-0.90890498	7.2358E-08
KANK1	-0.93981312	2.3771E-09
PEG3	-0.9198209	2.5398E-08
SNPH	-0.92929108	9.0167E-09
FAM234A	-0.91730053	3.2753E-08
C12orf75	-0.9147731	4.1933E-08
MTFR1L	-0.93042972	7.8854E-09
OBSL1	-0.93082165	7.5259E-09
FAM161B	-0.91098311	5.9902E-08
KBTBD12	-0.96763077	1.3419E-11
PRAG1	0.91146245	5.7311E-08
PIP4K2C	-0.95253172	3.3011E-10
GRAMD1C	0.92767562	1.0865E-08
PM20D2	-0.9231242	1.7966E-08
HABP4	-0.95543558	1.9496E-10
COPRS	-0.94064551	2.1177E-09
APOOL	-0.92913793	9.1792E-09
ZNF586	0.90700288	8.5682E-08
ARAP1	0.92104578	2.2377E-08
IKBKE	0.90379159	1.1307E-07
SCMH1	-0.9705659	6.0418E-12
CDK2AP1	-0.90645696	8.9882E-08
RELL2	0.91452488	4.2946E-08
PLEKHH3	-0.91003235	6.5347E-08
ATP6V1E1	-0.9504691	4.7055E-10
LPAR1	-0.90836665	7.5932E-08
OSR1	-0.92780221	1.0709E-08
SPECC1L	-0.92237642	1.9457E-08
CZIB	-0.94139678	1.9053E-09
BTBD10	-0.94853474	6.4737E-10
GLRX	0.91475395	4.2011E-08
PFKM	-0.93571961	4.1003E-09
VILL	0.90598897	9.3623E-08
ARHGEF10L	-0.90586011	9.4677E-08
HSPB7	-0.94381395	1.343E-09
KCNA5	-0.96279939	4.3066E-11
ST3GAL3	-0.96316022	3.969E-11
LDOC1	-0.95374261	2.6611E-10
ZNF219	-0.97317744	2.7672E-12
TGFB1I1	-0.97751237	6.2761E-13
AAR2	-0.93383152	5.2103E-09
ZBTB47	-0.97374063	2.3149E-12
MYL6	-0.91560313	3.8698E-08
KEAP1	-0.93067181	7.6616E-09
DDX19A	-0.90271498	1.2382E-07
TESMIN	0.90557615	9.7036E-08

EHD2	-0.93063648	7.6939E-09
SEPTIN3	0.90862058	7.4227E-08
ADSS1	-0.95086907	4.3982E-10
GPKOW	-0.9341575	5.0017E-09
PLEKHG3	-0.9585682	1.0605E-10
ADIRF	-0.91867673	2.8535E-08
LZTS2	-0.97123309	4.9832E-12
INPP5A	-0.95764509	1.275E-10
SPARCL1	-0.94252396	1.6217E-09
NR1H2	-0.91333306	4.811E-08
EXTL3	-0.92328323	1.7663E-08
DCUN1D4	-0.92606198	1.3033E-08
IGSF1	-0.90833251	7.6163E-08
PTMS	-0.94611866	9.4822E-10
ZBED3	-0.90349607	1.1594E-07
ARVCF	-0.93637953	3.7645E-09
RTL8B	-0.95885264	1.0012E-10
CD300LF	0.91979295	2.547E-08
ZSWIM9	-0.93817157	2.9711E-09
IFT43	-0.93237603	6.2373E-09
DDX1	-0.90306313	1.2025E-07
SCARA3	-0.90200076	1.3143E-07
NTPCR	-0.94833137	6.6897E-10
ACTN1	-0.94815946	6.8773E-10
EI24	-0.94610312	9.505E-10
THRA	-0.95789967	1.2123E-10
TAGLN	-0.96139049	5.8786E-11
IFT81	-0.90710451	8.492E-08
RTF2	-0.95030813	4.8345E-10
SMIM10	-0.97081131	5.6314E-12
CASC3	-0.97410161	2.0605E-12
CNIH4	-0.91544426	3.9299E-08
PPP1R14A	-0.95308553	2.9934E-10
TJP2	-0.91976805	2.5536E-08
NXPH3	-0.93248614	6.1539E-09
ADAMTSL5	-0.91307087	4.9316E-08
DYNC1LI2	-0.97862307	4.0958E-13
NAB2	-0.90394737	1.1158E-07
GALNT17	-0.91811186	3.0205E-08
GYG1	-0.97817342	4.8811E-13
APBB1	-0.96520603	2.4592E-11
SVIL	-0.95533715	1.9858E-10
ESD	-0.97905217	3.4523E-13
ZFYVE21	-0.93127877	7.1248E-09
SSBP2	-0.90708696	8.5051E-08
KALRN	-0.90615163	9.2308E-08
HPCAL1	0.93277607	5.9388E-09

RTL8A	-0.90317091	1.1916E-07
DNAJC19	-0.93000886	8.2882E-09
PCBD1	-0.91890583	2.7881E-08
PPP2R5D	-0.92295251	1.8299E-08
IGSF9B	-0.92634338	1.263E-08
SOBP	-0.95801075	1.1859E-10
UGGT2	-0.90919346	7.0504E-08
ACTN2	-0.95954875	8.6809E-11
JMJD8	-0.92299212	1.8222E-08
COPS4	-0.90417117	1.0948E-07
POLR2C	-0.96631321	1.8753E-11
AOPEP	-0.93830119	2.9199E-09
TMEM47	-0.94980012	5.2619E-10
CCDC134	0.94002677	2.308E-09
PCDHB11	-0.90910819	7.1048E-08
SNTA1	-0.97307601	2.8564E-12
DACT3	-0.98605688	1.1111E-14
PDLIM7	-0.91711247	3.3369E-08
MAP4	-0.94423689	1.2613E-09
HS1BP3	-0.95023868	4.891E-10
NFIA	-0.9293184	8.988E-09
SOD3	-0.90066513	1.4677E-07
C1QTNF2	-0.90151654	1.3683E-07
MINDY3	-0.91234375	5.2801E-08
DAAM2	-0.95455799	2.2942E-10
CAND2	-0.95469821	2.2358E-10
FAXC	-0.92256404	1.9073E-08
CRYAB	-0.94210141	1.7233E-09
S1PR3	-0.90481938	1.0357E-07
PRKG1	-0.9337689	5.2512E-09
DUSP3	-0.94818654	6.8474E-10
KCNMB1	-0.95620859	1.6846E-10
LRFN1	0.90929524	6.986E-08
MOCS2	-0.95519305	2.0399E-10
PERP	-0.92590706	1.326E-08
FOXO6	-0.90070433	1.463E-07
PDE8B	-0.97063409	5.9252E-12
WNK3	-0.95260913	3.2565E-10
PPP2R2B	-0.93272737	5.9744E-09
LBHD1	0.90459811	1.0556E-07
RAB23	-0.90210065	1.3034E-07
NDUFA4	-0.93729187	3.3401E-09
RASSF3	-0.92213977	1.995E-08
EBF4	-0.90491865	1.027E-07
SMIM10L1	-0.90002982	1.546E-07
PACSIN3	-0.9734352	2.5513E-12
ZNF771	-0.93583121	4.0418E-09

NDUFB8	-0.92732028	1.1313E-08
C5orf24	-0.91577049	3.8072E-08
MRFAP1	-0.92781052	1.0698E-08
THRB	-0.9671114	1.5336E-11
TSR2	-0.93454844	4.7612E-09
CLIC4	-0.92348608	1.7282E-08
FGF2	-0.90166575	1.3514E-07
MARK1	-0.95388694	2.5927E-10
AR	-0.93462848	4.7133E-09
ATP9A	-0.92347827	1.7296E-08
GPR135	-0.94909682	5.9082E-10
KIFBP	-0.93072554	7.6127E-09
UBE2D4	-0.9109816	5.9911E-08
NKIRAS1	-0.94137289	1.9118E-09
ARHGEF9	-0.92261327	1.8973E-08
VIPR2	-0.91111369	5.9186E-08
NDRG3	-0.90601893	9.338E-08
NGF	-0.94427732	1.2537E-09
CBX1	-0.93247411	6.1629E-09
SCOC	-0.92774816	1.0775E-08
JAM3	-0.95022071	4.9058E-10
KCNQ4	-0.96256958	4.5345E-11
ZNF768	-0.93060343	7.7243E-09
SNX21	-0.93104308	7.3292E-09
CASZ1	-0.94481871	1.1561E-09
COX7A1	-0.91686565	3.4194E-08
EPN2	-0.92068883	2.3223E-08
MTSS2	-0.94993068	5.149E-10
MNAT1	-0.92636457	1.26E-08
RNF185	-0.93771043	3.1598E-09
SGCA	-0.90428838	1.0839E-07
FAM126B	0.90215979	1.297E-07
PAK3	-0.92392932	1.6474E-08
ZCCHC14	-0.93923282	2.5739E-09
PURA	-0.90519532	1.0028E-07
TLN1	-0.93944156	2.5015E-09
TPM2	-0.95828477	1.1227E-10
PRKRA	-0.9252755	1.4221E-08
POLR2M	-0.90686419	8.6733E-08
MKKS	-0.90516876	1.0051E-07
ERI3	-0.90468828	1.0475E-07
KANSL1L	-0.92165213	2.1002E-08
HEXIM1	-0.90016911	1.5286E-07
EMP2	-0.9181352	3.0134E-08
RASL12	-0.96828237	1.1314E-11
DYNC1I1	-0.95101436	4.291E-10
TRMT61A	-0.91987035	2.5269E-08

FOXC1	-0.91212974	5.3866E-08
AMOTL1	-0.91411885	4.4648E-08
CALM1	-0.92020877	2.4405E-08
MCAM	-0.94560847	1.0255E-09
ADCY5	-0.95471201	2.2301E-10
SORBS1	-0.94146225	1.8877E-09
ARHGEF25	-0.95356419	2.748E-10
C8orf88	-0.94385396	1.3351E-09
HSPB8	-0.9417691	1.8072E-09
RBFOX2	-0.93763474	3.1918E-09
SORBS2	-0.90070531	1.4629E-07
RNF32	0.9041125	1.1003E-07
TBXA2R	-0.92856783	9.8068E-09
TBL1X	-0.91072888	6.1318E-08
PRDM5	-0.92768929	1.0848E-08
IL17B	-0.94431531	1.2467E-09
GNA11	-0.95963381	8.5294E-11
SYNC	-0.94092544	2.0363E-09
CEP104	-0.90540821	9.8455E-08
DUSP26	-0.92481547	1.4958E-08
BCL2L2	-0.97749936	6.3067E-13
NEXN	-0.90356804	1.1523E-07
SKP1	-0.93669311	3.6136E-09
PGRMC1	-0.94834557	6.6745E-10
SLAIN2	-0.93274368	5.9625E-09
BMERB1	-0.96050487	7.1069E-11
ALDH1L1	-0.90204981	1.309E-07
DLG5	-0.94729106	7.8967E-10
NLGN1	-0.92981958	8.4751E-09
NTF3	-0.92220544	1.9812E-08
CAMK2G	-0.94353338	1.3998E-09
CTPS1	-0.92208903	2.0057E-08
DYNC1LI1	-0.91607247	3.6966E-08
ARHGAP10	-0.93228676	6.3057E-09
FBXO10	0.90573144	9.574E-08
PHYH	-0.93226592	6.3218E-09
LTBP4	-0.93107913	7.2976E-09
TMEM242	-0.93700744	3.4677E-09
NACC2	-0.95909974	9.5203E-11
GULP1	-0.93225307	6.3317E-09
FYCO1	-0.9617691	5.4132E-11
RAB3GAP1	-0.91274791	5.0838E-08
PCDH7	-0.93451135	4.7836E-09
ZBTB4	-0.90427813	1.0848E-07
SBDS	-0.90678557	8.7333E-08
POLR2K	-0.93581259	4.0515E-09
RNF11	-0.90154624	1.3649E-07

ZEB1	-0.90844671	7.5391E-08
PDHA1	-0.90756494	8.1539E-08
NFE2L1	-0.91753818	3.1987E-08
EPM2A	-0.9521132	3.5517E-10
SMTN	-0.9812707	1.3431E-13
ST6GALNAC6	-0.94675176	8.5945E-10
LDB3	-0.92974452	8.5502E-09
RGN	-0.92567949	1.3599E-08
FERMT2	-0.92735888	1.1263E-08
TPPP	-0.92732236	1.131E-08
CAVIN1	-0.91643243	3.5685E-08
NFIX	-0.93593058	3.9902E-09
SNX12	-0.90668731	8.8088E-08
SUN2	-0.9266007	1.227E-08
KHDRBS3	-0.90888926	7.246E-08
BVES	-0.97209541	3.8586E-12
PPP2R1A	-0.91490844	4.139E-08
PEBP1	-0.91529427	3.9875E-08
UFSP2	-0.9093654	6.9419E-08
MYO1C	-0.94793806	7.1255E-10
PELI3	-0.94062027	2.1252E-09
ERC1	-0.90364641	1.1447E-07
TECPR2	-0.93310114	5.7054E-09
HDX	-0.90624195	9.1584E-08
LIX1L	-0.90828362	7.6496E-08
ANO1	-0.92627471	1.2727E-08
KCTD10	-0.92032346	2.4118E-08
AEBP2	-0.95291633	3.0846E-10
CALD1	-0.95304472	3.0152E-10
ALDH1B1	-0.94060511	2.1297E-09
NFIC	-0.94974123	5.3135E-10
PIP4P2	-0.9227289	1.8741E-08
MFAP4	-0.90652079	8.9382E-08
BAHCC1	-0.93955326	2.4635E-09
FBLN5	-0.92491513	1.4796E-08
PPP2R3A	-0.94650179	8.9359E-10
DCTN2	-0.9025881	1.2514E-07
BMPR1A	-0.94792578	7.1395E-10
DNAJB6	-0.93184998	6.6499E-09
DAG1	-0.91481506	4.1764E-08
PTPN11	-0.90174031	1.3431E-07
RBPMS	-0.96983954	7.4145E-12
MYL9	-0.95954617	8.6856E-11
STAC	-0.90218455	1.2944E-07
OTUD7B	-0.92112513	2.2193E-08
DYNC1I2	-0.93466776	4.6899E-09
VCL	-0.95153596	3.9248E-10

APLP2	-0.91587447	3.7688E-08
KIAA1191	-0.93405669	5.0654E-09
MSRB3	-0.92135226	2.1673E-08
BBS7	-0.91982152	2.5396E-08
NCKAP1	-0.92333341	1.7568E-08
WFS1	-0.94665311	8.7279E-10
PAIP1	-0.91077179	6.1077E-08
HOGA1	-0.95798189	1.1927E-10
NADK2	-0.90721739	8.408E-08
DNAJC18	-0.9351265	4.4242E-09
SPIRE1	-0.91451692	4.2978E-08
UBE2V2	-0.90246583	1.2643E-07
CLIP3	-0.91575305	3.8137E-08
PPP1R12B	-0.9494621	5.5643E-10
RAB5B	-0.92167304	2.0956E-08
MRGPRF	-0.95492856	2.1427E-10
TRAK2	-0.90714177	8.4642E-08
SGCE	-0.92764839	1.0898E-08
PTGES3L	-0.92383592	1.6642E-08
KCTD8	-0.92798933	1.0482E-08
ITPR1	-0.90931821	6.9715E-08
NCS1	-0.96311354	4.0114E-11
ANXA11	-0.93369377	5.3007E-09
RBSN	-0.93232259	6.2782E-09
DSTN	-0.95206492	3.5817E-10
SHROOM3	-0.97987126	2.4665E-13
FXYD1	-0.93412618	5.0214E-09
KCNMA1	-0.9427252	1.5751E-09
TACC2	-0.92585005	1.3344E-08
OCRL	-0.94280311	1.5574E-09
STRN3	-0.9257987	1.342E-08
CTTN	-0.91186599	5.5206E-08
ATL3	-0.90913606	7.087E-08
ACTA2	-0.9263236	1.2658E-08
PDLIM3	-0.92328348	1.7662E-08
PPP1R12A	-0.95423337	2.4346E-10
TCF7L1	-0.90610519	9.2682E-08
SBSPON	-0.93883241	2.7179E-09
CFL2	-0.95900795	9.7004E-11
ABI2	-0.92299033	1.8226E-08
SPRYD7	-0.93299628	5.7798E-09
NFU1	-0.91361185	4.6856E-08
CRTAP	-0.93864849	2.7864E-09
TEAD3	-0.94788585	7.1852E-10
ATN1	-0.94514836	1.0999E-09
MPP7	-0.95892183	9.8721E-11
FLNC	-0.96095682	6.4545E-11

FAM168B	-0.91560569	3.8688E-08
MTX2	-0.90069554	1.4641E-07
EFS	-0.90233044	1.2787E-07
CACFD1	-0.91279414	5.0618E-08
RDH14	-0.90605041	9.3125E-08
FAM20B	-0.90148854	1.3714E-07
DIXDC1	-0.92043496	2.3842E-08
PTPN21	-0.92508046	1.4529E-08
ATF7	-0.926646	1.2208E-08
SLC2A12	-0.90479239	1.0381E-07
PDK3	-0.9041581	1.096E-07
EID1	-0.93343696	5.4729E-09
STBD1	-0.90019112	1.5258E-07
LPP	-0.96715436	1.5169E-11
KIF13A	-0.92880087	9.5458E-09
ANKS1B	-0.96440107	2.9788E-11
MXI1	-0.90752219	8.1848E-08
RSU1	-0.9078475	7.9523E-08
TANC1	-0.91891981	2.7841E-08
MXRA7	-0.92861366	9.755E-09
TTLL7	-0.9576524	1.2731E-10
KCNIP3	-0.91761843	3.1733E-08
ARMCX1	-0.93101018	7.3581E-09
FLNA	-0.96783485	1.2726E-11
RIMKLB	-0.93006287	8.2355E-09
COL4A5	-0.90010448	1.5366E-07
FILIP1	-0.95923368	9.2628E-11
CORO1C	-0.90265478	1.2445E-07
ADD1	-0.9294541	8.8465E-09
FBXO17	-0.9206144	2.3403E-08
LMOD1	-0.96598264	2.0353E-11
SGCB	-0.90808919	7.7833E-08
ERP29	0.90146716	1.3739E-07
MPRIP	-0.90952675	6.8414E-08
JPH2	-0.95797175	1.1951E-10
PAWR	-0.95173637	3.7915E-10

List of DE mRNA whose expression correlates with TCL6

DE mRNA	Pearson	p-value
CXCL13	0.90696354	8.5979E-08
CXCR5	0.90196947	1.3178E-07
CR2	0.91141666	5.7554E-08
NIBAN3	0.94368983	1.3679E-09
SPIB	0.91438812	4.3513E-08
CD19	0.92727999	1.1365E-08
FCRL2	0.90508952	1.012E-07
BCL11A	0.92453032	1.5432E-08
SH2D3A	0.90901462	7.1648E-08
FDCSP	0.9122687	5.3172E-08
FCMR	0.90637867	9.0498E-08
CARMIL2	0.90397107	1.1136E-07
MS4A1	0.92615821	1.2894E-08
GPR18	0.93224045	6.3415E-09
CLEC17A	0.94495817	1.132E-09
STAP1	0.91135323	5.7892E-08
FAM177B	0.9388017	2.7293E-09
TBC1D10C	0.9020253	1.3117E-07
FCRL1	0.93711279	3.4199E-09
ADGRG5	0.91696982	3.3844E-08
SEPTIN1	0.90811397	7.7661E-08
BTLA	0.92178997	2.07E-08
CD40LG	0.90254009	1.2565E-07
MAP4K1	0.90060152	1.4754E-07
PARP15	0.90614354	9.2373E-08
P2RX5	0.92976934	8.5253E-09
FCER2	0.94259438	1.6052E-09
LARGE2	0.94004556	2.302E-09
VPREB3	0.92180843	2.066E-08
TLR10	0.92931894	8.9874E-09
CIITA	0.90241864	1.2693E-07
BEND4	0.92884513	9.497E-09
POU2F2	0.9004244	1.497E-07
FCHO1	0.91624475	3.6348E-08
LIMD2	0.91865474	2.8598E-08
PDE7A	0.90246361	1.2645E-07
PACSIN1	0.90920262	7.0446E-08
RIPOR2	0.91161265	5.6519E-08
CD37	0.91255232	5.178E-08
CD22	0.94564801	1.0193E-09
SNX22	0.95024195	4.8884E-10
RHOF	0.90993909	6.5903E-08
TMC8	0.90644604	8.9968E-08

SHISAL2A	0.91568505	3.839E-08
PAX5	0.90686784	8.6705E-08
TTN	0.90583429	9.489E-08
KLHL14	0.93459666	4.7323E-09
AKNA	0.90559806	9.6852E-08
SSTR3	0.90650467	8.9508E-08
NETO1	0.91506855	4.0755E-08
SEPTIN6	0.90736833	8.2968E-08
CLEC2D	0.90069656	1.464E-07
CD79B	0.94162989	1.8433E-09
KEL	0.93037378	7.938E-09
TCL1A	0.95485671	2.1713E-10
CNR2	0.92921359	9.0986E-09
CCDC88B	0.90023131	1.5208E-07
KIFC1	0.9051212	1.0092E-07
GCSAM	0.93229452	6.2997E-09
CDK5R1	0.92226236	1.9693E-08
CCDC18	0.90003364	1.5455E-07
IRAG2	0.9150232	4.0934E-08
P2RX5-TAX1B	0.92252475	1.9153E-08
FCRL4	0.94082115	2.0663E-09
ABCA7	0.90159603	1.3593E-07
CD72	0.90413627	1.098E-07
ESPL1	0.90468537	1.0477E-07
TLR9	0.92480077	1.4982E-08
GRAP	0.90796067	7.8728E-08
ZNF296	0.90221673	1.2909E-07
PLAC8	0.90370355	1.1392E-07
LINGO3	0.90626775	9.1379E-08
NPHS1	0.90194978	1.3199E-07
CDCA2	0.90186094	1.3297E-07
TROAP	0.90193925	1.3211E-07
ATP2A3	0.91480107	4.182E-08
RNF44	0.93655137	3.6812E-09
SP110	0.90381191	1.1287E-07
ANXA2R	0.9050578	1.0147E-07
COL19A1	0.92713686	1.155E-08
FANCD2	0.92427495	1.5868E-08
PLAG1	0.92926349	9.0458E-09
TTC24	0.90504374	1.016E-07
CNTRL	0.93088813	7.4664E-09
TREML2	0.92023632	2.4336E-08
C12orf42	0.95642662	1.6158E-10
FUT7	0.90526952	9.9641E-08
CDC42BPG	0.91184756	5.53E-08
AP1G2	0.90524316	9.9867E-08
GEN1	0.90758797	8.1373E-08

RINL	0.91355674	4.7101E-08
STRBP	0.95460794	2.2732E-10
CHST4	0.90408994	1.1024E-07
PVALB	0.94280833	1.5562E-09
GGA2	0.95050675	4.6758E-10
BEX3	-0.91653238	3.5336E-08
LIG1	0.92258927	1.9022E-08
ARL2	-0.91306288	4.9353E-08
PLK1	0.9138237	4.5922E-08
HTR3A	0.90810335	7.7735E-08
ZNF101	0.90275277	1.2343E-07
SMC6	0.94148446	1.8818E-09
RMI2	0.90204437	1.3096E-07
ZNF107	0.91024212	6.411E-08
LINGO4	0.91944901	2.6382E-08
SAMD10	0.90976412	6.6959E-08
STAG3	0.90669152	8.8056E-08
FBXO46	0.93198549	6.5414E-09
CD99L2	-0.92593549	1.3218E-08
PIGR	0.93455405	4.7578E-09
LRIF1	0.93388849	5.1733E-09
ZNF714	0.92089919	2.2721E-08
PRKD2	0.94409914	1.2874E-09
KDM2B	0.91422398	4.4201E-08
PTCRA	0.9007907	1.4527E-07
MYO7B	0.90561722	9.6692E-08
ANO10	-0.90815082	7.7407E-08
EFCAB12	0.91945965	2.6354E-08
TMEM243	0.9285673	9.8074E-09
CCNF	0.90757403	8.1473E-08
TCF3	0.9132061	4.8691E-08
PLPPR2	-0.94365577	1.3748E-09
BAG2	-0.90127936	1.3954E-07
HVCN1	0.91460742	4.2607E-08
FAM76B	0.92348537	1.7283E-08
KDF1	0.91190963	5.4982E-08
SMARCD3	-0.91440591	4.3438E-08
CYB5R1	-0.90668135	8.8134E-08
RAB34	-0.90164722	1.3535E-07
NUCB1	-0.90741614	8.2619E-08
P3H3	-0.93065217	7.6796E-09
CASP2	0.92841231	9.9844E-09
MAP6	-0.90486286	1.0319E-07
FAM234A	-0.91222058	5.3412E-08
C17orf99	0.90638023	9.0486E-08
MTF2	0.93337568	5.5147E-09
QSOX1	-0.92145847	2.1433E-08

VILL	0.90938718	6.9283E-08
TGFB1I1	-0.91108046	5.9368E-08
OBSCN	0.9022334	1.2891E-07
ACSS1	0.92890708	9.4289E-09
ZBTB47	-0.90569269	9.6062E-08
EHD2	-0.91296023	4.9833E-08
CHST3	-0.90512947	1.0085E-07
RTL8B	-0.91629692	3.6163E-08
ACTN1	-0.90210909	1.3025E-07
TAGLN	-0.90840349	7.5682E-08
IFT81	-0.93810144	2.9992E-09
SUSD3	0.94575197	1.0032E-09
SPECC1	-0.90696262	8.5986E-08
COX19	0.91237713	5.2636E-08
GTSF1L	0.92297282	1.826E-08
AOPEP	-0.91754225	3.1974E-08
MAP4K2	0.94004313	2.3028E-09
MRFAP1	-0.91618295	3.6569E-08
CLIC4	-0.90574607	9.5618E-08
JAM3	-0.90942538	6.9044E-08
RRAGA	-0.90312211	1.1965E-07
VHL	0.91577528	3.8054E-08
GNA11	-0.90444301	1.0697E-07
PGRMC1	-0.90121817	1.4024E-07
ARHGEF18	0.90584849	9.4773E-08
TNKS1BP1	-0.90081233	1.4501E-07
DCLRE1C	0.91388805	4.5641E-08
NEK8	0.90363631	1.1457E-07
FERMT2	-0.90164598	1.3537E-07
SLC24A3	-0.92322828	1.7767E-08
CAVIN1	-0.9009786	1.4304E-07
HEPH	-0.90882692	7.2867E-08
NFIC	-0.90545596	9.805E-08
EMILIN1	-0.90958379	6.8062E-08
SAR1A	-0.90311215	1.1975E-07
DNAJB6	-0.90383658	1.1264E-07
VCL	-0.92339806	1.7446E-08
ARL3	-0.91364954	4.6688E-08
SMARCA1	-0.90330598	1.1781E-07
ATL1	-0.90157074	1.3621E-07
OCRL	-0.91179528	5.5569E-08
PDLIM3	-0.93715047	3.403E-09
RTN4	-0.9104705	6.2786E-08
MTMR2	-0.91973876	2.5612E-08
STBD1	-0.90267302	1.2426E-07
CTNNA1	-0.93632564	3.791E-09
ATP2A2	-0.90582491	9.4967E-08

TCEAL9	-0.90742684	8.254E-08

List of DE mRNA whose expression correlates with LINC00892

DE mRNA	Pearson	p-value
NIBAN3	0.90947408	6.8741E-08
SHISA4	-0.90605251	9.3108E-08
FGFRL1	-0.90081935	1.4493E-07
CARMIL2	0.90939283	6.9247E-08
ACAP1	0.91400508	4.5135E-08
CLEC17A	0.92603278	1.3075E-08
CD52	0.90039721	1.5003E-07
TBC1D10C	0.90514145	1.0075E-07
NLRC3	0.90025728	1.5176E-07
SEPTIN1	0.91550297	3.9076E-08
PATL2	0.90093489	1.4356E-07
CD40LG	0.92402931	1.6297E-08
MYBL2	0.90296685	1.2123E-07
MAP4K1	0.90137406	1.3845E-07
PARP15	0.93115553	7.231E-09
NUSAP1	0.90679017	8.7298E-08
CIITA	0.90832621	7.6206E-08
POU2F2	0.90027005	1.516E-07
FCHO1	0.93004855	8.2494E-09
TRAF3IP3	0.90598265	9.3675E-08
KIF1C	-0.91299368	4.9676E-08
LIMD2	0.91163658	5.6394E-08
RASAL3	0.91584685	3.779E-08
PDE7A	0.90613388	9.2451E-08
PACSIN1	0.9068574	8.6784E-08
LIMS4	-0.92939886	8.9038E-09
CD37	0.91819092	2.9966E-08
CD22	0.92496113	1.4721E-08
TMC8	0.92371717	1.6856E-08
DENND1C	0.91243325	5.2361E-08
CLEC2D	0.92389397	1.6537E-08
CD79B	0.90968977	6.7412E-08
BUB1	0.90941289	6.9122E-08
EZH2	0.92480032	1.4983E-08
AURKB	0.91621852	3.6442E-08
CCDC88B	0.93383995	5.2048E-09
KIFC1	0.91387436	4.5701E-08
CDK5R1	0.91543262	3.9344E-08
ARHGAP45	0.9017539	1.3416E-07
PASK	0.90350436	1.1585E-07
ARHGAP27	0.91180239	5.5533E-08
BTK	0.90286588	1.2226E-07
FCRL4	0.93408023	5.0505E-09

SGO1	0.9020097	1.3134E-07
ABCA7	0.9127654	5.0755E-08
CD72	0.92664232	1.2213E-08
ESPL1	0.92013778	2.4584E-08
GRAP	0.92600711	1.3113E-08
TACC3	0.92849803	9.8862E-09
FANCI	0.92464379	1.5242E-08
EXOC3L4	0.9037676	1.133E-07
TMC6	0.93152831	6.9138E-09
JAK3	0.90539624	9.8557E-08
ARHGAP4	0.91611046	3.6829E-08
CDCA2	0.9261265	1.2939E-08
PTPN6	0.9068022	8.7206E-08
CCNB2	0.90404988	1.1061E-07
GTSE1	0.93150277	6.9351E-09
NCF1	0.9118802	5.5133E-08
TROAP	0.92642996	1.2508E-08
GMIP	0.90018429	1.5267E-07
RNF44	0.93172498	6.7514E-09
SP110	0.92504861	1.458E-08
FANCD2	0.96729466	1.4634E-11
TTC24	0.92426345	1.5888E-08
DPEP2	0.90502388	1.0177E-07
PARVG	0.9022168	1.2909E-07
ARMH1	0.90602334	9.3344E-08
UNC13D	0.90042217	1.4973E-07
PSMB8	0.9000814	1.5395E-07
CDCA5	0.91140237	5.763E-08
RGS14	0.92148132	2.1382E-08
ZNF814	0.90972978	6.7168E-08
DENND2D	0.9226952	1.8809E-08
WDR62	0.92479499	1.4992E-08
MCM5	0.91219804	5.3524E-08
TMPRSS13	0.91767366	3.1558E-08
CLSPN	0.90221801	1.2908E-07
KIF18B	0.91756017	3.1917E-08
ZWINT	0.90584827	9.4775E-08
POLQ	0.913164	4.8885E-08
FANCA	0.91880466	2.8168E-08
FUT7	0.90836095	7.597E-08
AURKA	0.90435526	1.0777E-07
EVL	0.92010897	2.4657E-08
ADPGK	0.91105557	5.9504E-08
UNC93B1	0.90504624	1.0158E-07
AP1G2	0.94648865	8.9542E-10
GEN1	0.9164559	3.5603E-08
P2RY11	0.90472626	1.0441E-07

CCDC78	0.91214705	5.378E-08
ARHGEF1	0.9365921	3.6616E-09
MAGED2	-0.90076784	1.4554E-07
GGT1	0.91423425	4.4158E-08
NBEAL2	0.90930695	6.9786E-08
GGA2	0.91869718	2.8476E-08
BEX3	-0.91442997	4.3338E-08
LIG1	0.94807942	6.9661E-10
CTC-479C5.12	0.9077896	7.9932E-08
GRK2	0.91738458	3.248E-08
PLK1	0.93931279	2.546E-09
GSAP	0.91055064	6.2327E-08
HSPA4L	-0.90326303	1.1824E-07
NPR2	-0.90839832	7.5717E-08
GSTK1	0.91694245	3.3935E-08
CACNA1F	0.91726545	3.2867E-08
FAM72B	0.90414116	1.0976E-07
NGRN	-0.91688048	3.4144E-08
RMI2	0.93365412	5.3269E-09
OGFR	0.90562332	9.6641E-08
EME1	0.90526384	9.9689E-08
ATXN7L1	0.90571354	9.5888E-08
SAMD10	0.91991758	2.5147E-08
HAGHL	0.90253546	1.257E-07
STAG3	0.90542927	9.8276E-08
PARP10	0.92751745	1.1062E-08
ZNF532	-0.9054162	9.8387E-08
FBXL2	-0.91193407	5.4857E-08
DMTN	-0.90115485	1.4098E-07
ERBB2	-0.90710938	8.4884E-08
SP140L	0.94046637	2.1714E-09
FBXO46	0.90365943	1.1434E-07
CD99L2	-0.92482919	1.4936E-08
PIGR	0.90562373	9.6637E-08
DENND4B	0.9524697	3.3372E-10
ZNF714	0.93827664	2.9295E-09
PRKD2	0.9284919	9.8932E-09
KDM2B	0.90720811	8.4149E-08
ZNRF3	-0.91776591	3.1269E-08
LTB4R2	0.91293151	4.9968E-08
MCOLN2	0.90690007	8.646E-08
SKA3	0.90910228	7.1086E-08
ANO10	-0.90074862	1.4577E-07
PCDHGA7	-0.90103597	1.4237E-07
PCDHGA4	-0.90711106	8.4871E-08
HELLS	0.92195407	2.0345E-08
FMOD	-0.91782061	3.1099E-08

EFCAB12	0.90494111	1.025E-07
ARHGEF17	-0.91028454	6.3862E-08
LRP5	-0.92566307	1.3624E-08
SIGIRR	0.91647054	3.5551E-08
CCNF	0.93637362	3.7674E-09
ADCY9	-0.91022642	6.4202E-08
PFN2	-0.90466035	1.05E-07
MGST3	-0.90181919	1.3343E-07
FEZ1	-0.92639228	1.2561E-08
ENDOD1	-0.90094576	1.4343E-07
DAB2IP	-0.91036179	6.3413E-08
PAM	-0.90715763	8.4524E-08
KIF22	0.90529199	9.9448E-08
ZNF276	0.90510602	1.0105E-07
TWSG1	-0.91213685	5.3831E-08
BLZF1	-0.91388058	4.5674E-08
MSH5	0.91304411	4.9441E-08
KDF1	0.90457658	1.0575E-07
CDIP1	-0.91732128	3.2685E-08
CDCA3	0.90992317	6.5999E-08
RAB34	-0.90616435	9.2206E-08
PDE1A	-0.91977697	2.5512E-08
NEO1	-0.90118092	1.4067E-07
RP11-736I24.!	0.90748245	8.2136E-08
CYBC1	0.91094755	6.0099E-08
P3H3	-0.92302186	1.8164E-08
CYB5R3	-0.91539632	3.9483E-08
IGFBP7	-0.90598919	9.3622E-08
CASP2	0.92954055	8.7574E-09
MAP6	-0.93530882	4.3223E-09
PEG3	-0.91681612	3.4362E-08
RHPN1	0.90615138	9.231E-08
DKK3	-0.90549748	9.7699E-08
TYK2	0.9027905	1.2304E-07
HABP4	-0.91085554	6.0609E-08
RELL2	0.91283473	5.0425E-08
TONSL	0.91886561	2.7995E-08
VILL	0.9201047	2.4668E-08
ST3GAL3	-0.90042638	1.4967E-07
TGFB1I1	-0.9013349	1.389E-07
ACSS1	0.90225467	1.2868E-07
ZBTB47	-0.92820345	1.0227E-08
EHD2	-0.90467745	1.0484E-07
SPARCL1	-0.90721611	8.4089E-08
CHST3	-0.9186699	2.8554E-08
RTL8B	-0.90484055	1.0339E-07
PCDHGA3	-0.90324761	1.1839E-07

ACTN1	-0.90968499	6.7441E-08
TAGLN	-0.91452408	4.2949E-08
ERGIC1	-0.92046694	2.3763E-08
LRCH4	0.93114141	7.2433E-09
COX19	0.90524157	9.9881E-08
UGGT2	-0.91524726	4.0057E-08
HDAC10	0.918146	3.0101E-08
AOPEP	-0.92286045	1.848E-08
TMEM47	-0.90704599	8.5358E-08
RAB11FIP5	-0.90076773	1.4554E-07
MAP4	-0.91619094	3.654E-08
PRKG1	-0.92189819	2.0465E-08
DUSP3	-0.91984889	2.5325E-08
DNMT1	0.90774084	8.0279E-08
MEN1	0.90563994	9.6502E-08
RAB23	-0.91572622	3.8237E-08
FKBP7	-0.90495021	1.0242E-07
SLC10A3	-0.92149515	2.1351E-08
NDUFA4	-0.90012504	1.5341E-07
MRFAP1	-0.93318849	5.6441E-09
CLIC4	-0.93721861	3.3725E-09
FGF2	-0.91660931	3.5069E-08
ATP9A	-0.90764877	8.0936E-08
KIFBP	-0.90191935	1.3233E-07
UBE2D4	-0.9213802	2.1609E-08
SCOC	-0.9277699	1.0748E-08
JAM3	-0.92057575	2.3497E-08
ZCCHC14	-0.92058181	2.3482E-08
ARL1	-0.92105061	2.2366E-08
RRAGA	-0.91695396	3.3897E-08
PCDHGA2	-0.90293243	1.2158E-07
EMP2	-0.9360777	3.915E-09
KIRREL1	-0.90618862	9.2011E-08
FOXC1	-0.92227099	1.9675E-08
AMOTL1	-0.95596172	1.7656E-10
CALM1	-0.90437035	1.0763E-07
MORF4L1	-0.92420074	1.5996E-08
RBFOX2	-0.95186029	3.7112E-10
LIMCH1	-0.90612416	9.2529E-08
PRDM5	-0.93004431	8.2536E-09
GNA11	-0.90119059	1.4056E-07
SKP1	-0.94105294	2.0001E-09
PGRMC1	-0.9348605	4.5766E-09
ITGB1	-0.91940094	2.6512E-08
TMEM109	-0.91896319	2.7719E-08
SLAIN2	-0.90345585	1.1633E-07
DLG5	-0.93356376	5.3872E-09

TNKS1BP1	-0.91032022	6.3654E-08
EFCAB8	0.9038448	1.1256E-07
GULP1	-0.91369398	4.6492E-08
DCLRE1C	0.92756209	1.1006E-08
SBDS	-0.94506634	1.1136E-09
RNF11	-0.93154225	6.9022E-09
PELO	-0.90678439	8.7342E-08
ZEB1	-0.91913909	2.7228E-08
PCDHB10	-0.90225519	1.2868E-07
FERMT2	-0.93127014	7.1322E-09
SLC24A3	-0.91002005	6.542E-08
CAVIN1	-0.92982672	8.468E-09
NFIX	-0.92068749	2.3226E-08
HEPH	-0.93002859	8.2689E-09
SNX12	-0.91144879	5.7383E-08
SH3RF1	-0.9297848	8.5098E-09
MYO1C	-0.9174512	3.2266E-08
ERC1	-0.91244213	5.2317E-08
TECPR2	-0.91073978	6.1256E-08
KCTD10	-0.91063469	6.185E-08
CALD1	-0.91275345	5.0812E-08
RAB18	-0.92694358	1.1806E-08
SEPTIN5	-0.91757119	3.1882E-08
PIP4P2	-0.91036883	6.3372E-08
PPP2R3A	-0.91812779	3.0156E-08
BMPR1A	-0.92286009	1.8481E-08
WWTR1	-0.91514795	4.0444E-08
DNAJB6	-0.91990289	2.5185E-08
DAG1	-0.94607411	9.5476E-10
OTUD7B	-0.91360516	4.6885E-08
VCL	-0.94441501	1.2282E-09
DYNLT3	-0.92050378	2.3673E-08
ARL3	-0.94722497	7.9795E-10
PLS3	-0.91206294	5.4203E-08
KIAA1191	-0.90092233	1.437E-07
MSRB3	-0.9383504	2.9006E-09
SMARCA1	-0.9250397	1.4595E-08
NCKAP1	-0.94346504	1.4139E-09
PAIP1	-0.90170459	1.3471E-07
SPIRE1	-0.92627622	1.2725E-08
SGCE	-0.90705289	8.5306E-08
ATL1	-0.92409152	1.6187E-08
DSTN	-0.91623526	3.6382E-08
ACTR10	-0.91863589	2.8653E-08
OCRL	-0.91896416	2.7716E-08
STRN3	-0.91419993	4.4303E-08
CTTN	-0.92601207	1.3106E-08

ATL3	-0.92934869	8.9562E-09
PDLIM3	-0.91962976	2.59E-08
RBMS3	-0.92166291	2.0978E-08
TMBIM1	-0.90125801	1.3978E-07
ABI2	-0.92268529	1.8828E-08
RTN4	-0.90272713	1.2369E-07
CRTAP	-0.92954605	8.7517E-09
MTMR2	-0.91124162	5.8492E-08
GJA1	-0.93088302	7.4709E-09
PGM1	-0.94155544	1.863E-09
DIXDC1	-0.92536179	1.4086E-08
PTPN21	-0.91031433	6.3689E-08
ATF7	-0.90161188	1.3575E-07
EID1	-0.90869736	7.3719E-08
FHOD3	-0.91602944	3.7122E-08
FAM177A1	-0.90704488	8.5367E-08
STBD1	-0.9232973	1.7636E-08
KIF13A	-0.90549285	9.7738E-08
CTNNA1	-0.93882696	2.7199E-09
SGCD	-0.92879658	9.5506E-09
NPTN	-0.91080775	6.0876E-08
LAMTOR3	-0.92711193	1.1583E-08
CAPN2	-0.91340661	4.7776E-08
MXRA7	-0.92204116	2.0159E-08
ESYT2	-0.92552677	1.3831E-08
TMEM43	-0.92518665	1.4361E-08
ATP2A2	-0.90057589	1.4785E-07
CAV2	-0.92886302	9.4773E-09
SEC23A	-0.91997556	2.4998E-08
SGCB	-0.931032	7.3389E-09
PAWR	-0.91574069	3.8183E-08

List of DE mRNA whose expression correlates with ITPK-IT1

DE mRNA	Pearson	p-value
PAN3	0.9320978	6.4527E-09
DYNLL1	-0.9024578	1.2652E-07
SMPD1	-0.91294478	4.9906E-08
BGN	-0.90703401	8.5448E-08
FAM98A	-0.91058607	6.2126E-08
COPS7A	-0.94309849	1.4919E-09
NCDN	-0.90453849	1.061E-07
SPRED3	-0.90447388	1.0669E-07
CD99L2	-0.90541483	9.8399E-08
ANO10	-0.90002327	1.5469E-07
PTPA	-0.92750021	1.1084E-08
ICMT	-0.92196339	2.0325E-08
CHST6	-0.916776	3.4498E-08
GPC1	-0.90755788	8.159E-08
B4GALT2	-0.91425049	4.409E-08
PRADC1	-0.9336071	5.3582E-09
TNFRSF11B	-0.91471052	4.2186E-08
TGM2	-0.94446004	1.22E-09
NIBAN2	-0.93211686	6.4377E-09
