# Supplementary material for: Identification of endoplasmic reticulum stress-associated lncRNAs influencing inflammation and VSMC function in abdominal aortic aneurysm
Source: Clin Sci (Lond). 2025 Mar 25;139(6):357–72. doi: 10.1042/CS20242476 (PMC12204013; doi:10.1042/CS20242476)
Supplement: Supplementary Table S3 [file CS-139-06-CS20242476-s004.pdf]

RNAseq analysis of aorta from AAA patients and donors. Protein_coding and lncRNA are shown. Expression value

nesembl	genesymbol	genetype	log2FoldChan	pvalue	padj
ENSG0000018	FDCSP	protein_codir	10.2757682	4.74E-13	8.13E-11
ENSG0000028	CTD-2643I7.4	protein_codir	9.51566165	1.98E-06	3.29E-05
ENSG0000013	MMP13	protein_codir	9.09033501	7.96E-10	4.19E-08
ENSG0000011	CR2	protein_codir	8.91998948	5.67E-15	2.18E-12
ENSG0000015	CXCL13	protein_codir	8.79179432	1.11E-17	2.66E-14
ENSG0000025	RP11-164H13	lncRNA	8.16320239	4.20E-07	8.64E-06
ENSG000002	IBSP	protein_codir	8.10577809	1.15E-14	3.93E-12
ENSG0000027	ENSG0000027	protein_codir	8.01024389	4.74E-15	1.93E-12
ENSG0000027	ENSG0000027	protein_codir	7.91694266	4.77E-12	5.73E-10
ENSG0000026	MMP12	protein_codir	7.8722689	2.37E-17	3.84E-14
ENSG0000015	CIBAR2	protein_codir	7.81792474	2.74E-12	3.61E-10
ENSG0000011	PLA2G2D	protein_codir	7.81757979	5.33E-12	6.18E-10
ENSG0000016	MC4R	protein_codir	7.78576121	2.14E-11	2.01E-09
ENSG0000016	FAM151A	protein_codir	7.78488919	2.28E-05	0.0002556
ENSG0000019	ALPK2	protein_codir	7.73001674	1.59E-14	5.20E-12
ENSG0000010	TCL1A	protein_codir	7.71017202	6.24E-09	2.37E-07
ENSG0000017	COL6A5	protein_codir	7.64426683	3.32E-09	1.38E-07
ENSG0000022	MIR4432HG	lncRNA	7.64419939	4.08E-09	1.64E-07
ENSG0000024	APELA	protein_codir	7.61700592	1.95E-09	8.73E-08
ENSG0000016	RASSF6	protein_codir	7.56486704	2.47E-15	1.16E-12
ENSG0000015	LAD1	protein_codir	7.44623511	1.94E-11	1.86E-09
ENSG0000027	RP11-1399P1!	lncRNA	7.39537567	2.45E-11	2.23E-09
ENSG0000016	FCAMR	protein_codir	7.38518831	2.63E-07	5.75E-06
ENSG0000026	SPIB	protein_codir	7.36062724	5.47E-14	1.35E-11
ENSG0000007	CD5L	protein_codir	7.33900262	1.90E-06	3.17E-05
ENSG0000012	SRMS	protein_codir	7.26828461	1.67E-10	1.11E-08
ENSG0000016	PNOC	protein_codir	7.19476106	9.54E-14	2.03E-11
ENSG0000016	FCRL4	protein_codir	7.16316083	1.89E-08	5.99E-07
ENSG0000013	IL21	protein_codir	7.15561207	9.29E-08	2.36E-06
ENSG0000009	DAZL	protein_codir	7.12994768	1.63E-09	7.53E-08
ENSG0000018	IL17REL	protein_codir	7.08116205	1.22E-08	4.16E-07
ENSG0000025	IGLL5	protein_codir	7.05392232	3.97E-15	1.66E-12
ENSG0000018	GP1BA	protein_codir	7.01666292	3.41E-12	4.31E-10
ENSG0000006	COL11A1	protein_codir	6.92710053	8.60E-20	1.19E-15
ENSG0000010	CD79A	protein_codir	6.91136283	5.96E-15	2.25E-12
ENSG0000027	ENSG0000027	protein_codir	6.90921914	2.93E-11	2.57E-09
ENSG0000013	FCRL2	protein_codir	6.83228411	5.81E-14	1.39E-11
ENSG0000001	LTF	protein_codir	6.82157208	1.88E-14	5.72E-12
ENSG0000018	LRRC26	protein_codir	6.81644604	1.11E-08	3.82E-07
ENSG0000010	CCL17	protein_codir	6.77585119	3.64E-08	1.05E-06
ENSG0000024	RP11-768B22.	lncRNA	6.74281145	7.03E-10	3.75E-08
ENSG0000023	LINC02576	lncRNA	6.74064386	1.63E-09	7.51E-08
ENSG0000017	BFSP2	protein_codir	6.68247086	1.21E-07	2.95E-06
ENSG0000025	RP11-16E12.2	lncRNA	6.67010796	5.77E-12	6.62E-10

ENSG0000016	FCRL1	protein_codir	6.63873091	1.13E-11	1.19E-09
ENSG0000027	AC006538.8	lncRNA	6.62473203	8.54E-07	1.60E-05
ENSG0000027	SSTR3	protein_codir	6.61913771	3.83E-09	1.56E-07
ENSG0000023	LINC01781	lncRNA	6.61745026	7.24E-10	3.84E-08
ENSG0000019	FAM177B	protein_codir	6.58388429	7.88E-12	8.63E-10
ENSG0000024	TNFRSF13B	protein_codir	6.57649541	1.75E-12	2.45E-10
ENSG0000026	RP11-960L18.	lncRNA	6.55389552	3.22E-09	1.35E-07
ENSG0000015	MS4A1	protein_codir	6.54528011	8.56E-13	1.30E-10
ENSG0000017	MZB1	protein_codir	6.54494151	9.36E-14	2.00E-11
ENSG0000013	FCRLA	protein_codir	6.54435647	4.03E-13	7.00E-11
ENSG0000019	HBG2	protein_codir	6.53738128	4.29E-06	6.22E-05
ENSG0000017	KRT72	protein_codir	6.49367396	1.93E-06	3.21E-05
ENSG0000023	LINC00494	lncRNA	6.48621785	3.76E-10	2.22E-08
ENSG0000011	HPCAL4	protein_codir	6.4733841	1.20E-08	4.09E-07
ENSG0000014	FCRL5	protein_codir	6.47065699	2.30E-11	2.14E-09
ENSG0000018	CLEC17A	protein_codir	6.46633052	1.81E-12	2.52E-10
ENSG0000016	NIBAN3	protein_codir	6.42470723	3.96E-14	1.05E-11
ENSG0000018	NCCRP1	protein_codir	6.40794786	2.91E-06	4.49E-05
ENSG0000017	CCR8	protein_codir	6.40241944	3.84E-07	8.03E-06
ENSG0000023	AC104024.1	lncRNA	6.36843002	2.07E-09	9.19E-08
ENSG0000011	BCL11A	protein_codir	6.36221231	6.81E-14	1.55E-11
ENSG0000020	CLLU1-AS1	lncRNA	6.35943801	0.00012304	0.00103771
ENSG0000013	EDAR	protein_codir	6.33169797	3.86E-07	8.07E-06
ENSG0000021	TCL1B	protein_codir	6.33114541	5.13E-05	0.0005025
ENSG0000022	FAM30A	lncRNA	6.32087514	3.56E-11	3.05E-09
ENSG0000014	CTD-2545M3.	protein_codir	6.27744709	2.35E-05	0.00026228
ENSG0000027	LINC01727	lncRNA	6.27156832	4.64E-07	9.41E-06
ENSG0000027	LINC01215	lncRNA	6.25101334	2.28E-14	6.59E-12
ENSG0000017	CD19	protein_codir	6.23507787	5.64E-14	1.37E-11
ENSG0000021	LY86-AS1	lncRNA	6.22933635	1.67E-07	3.88E-06
ENSG0000017	LRRC15	protein_codir	6.22487612	2.38E-16	1.81E-13
ENSG0000016	IL22RA2	protein_codir	6.200189	1.94E-05	0.00022346
ENSG0000016	TERT	protein_codir	6.17299938	9.23E-06	0.00012034
ENSG0000003	STAP1	protein_codir	6.16611018	3.39E-12	4.31E-10
ENSG0000025	TIFAB	protein_codir	6.15319184	9.87E-11	7.02E-09
ENSG0000012	CCR7	protein_codir	6.15217881	2.77E-17	4.02E-14
ENSG0000015	TNFRSF13C	protein_codir	6.15094447	1.08E-11	1.14E-09
ENSG0000011	ATP10B	protein_codir	6.14613902	1.52E-10	1.02E-08
ENSG0000022	IL21-AS1	lncRNA	6.14381183	3.33E-07	7.09E-06
ENSG0000016	CXCR5	protein_codir	6.13519187	7.84E-16	4.51E-13
ENSG0000020	LINC02397	lncRNA	6.12447694	6.21E-10	3.42E-08
ENSG0000012	VPREB3	protein_codir	6.11569766	1.17E-10	8.03E-09
ENSG0000022	LINC00582	lncRNA	6.09753039	1.61E-08	5.24E-07
ENSG0000012	IGLL1	protein_codir	6.06173544	7.23E-06	9.78E-05
ENSG0000013	MMP7	protein_codir	6.04651159	2.03E-09	9.07E-08
ENSG0000028	LINC01226	lncRNA	6.04392428	2.01E-05	0.00023054
ENSG0000022	DTNB-AS1	lncRNA	6.02263145	4.78E-08	1.33E-06

ENSG0000019	PAX5	protein_codir	6.02190582	2.61E-09	1.12E-07
ENSG0000015	DNAAF1	protein_codir	6.02010198	5.48E-14	1.35E-11
ENSG0000016	NPHS1	protein_codir	5.98945347	7.60E-08	1.99E-06
ENSG0000018	DCC	protein_codir	5.98800881	2.99E-13	5.47E-11
ENSG0000028	RP5-841K13.1	lncRNA	5.98277567	5.49E-07	1.09E-05
ENSG0000018	BHLHA15	protein_codir	5.96930922	2.58E-15	1.18E-12
ENSG0000023	LINC00402	lncRNA	5.95382694	3.96E-11	3.30E-09
ENSG0000018	KRT73	protein_codir	5.95304333	4.74E-07	9.58E-06
ENSG0000023	SHISA8	protein_codir	5.91181813	3.30E-11	2.84E-09
ENSG0000018	CNR2	protein_codir	5.89594559	6.73E-09	2.51E-07
ENSG0000011	POU2AF1	protein_codir	5.89498513	1.92E-12	2.65E-10
ENSG0000026	LINC02132	lncRNA	5.89146381	2.11E-06	3.46E-05
ENSG0000012	GPR18	protein_codir	5.88926136	1.74E-12	2.45E-10
ENSG0000011	AMPD1	protein_codir	5.87690264	1.61E-09	7.45E-08
ENSG0000026	DMRTC1	protein_codir	5.87671198	5.03E-06	7.16E-05
ENSG0000010	BIK	protein_codir	5.87416826	3.22E-10	1.96E-08
ENSG0000025	IGHV3OR16-1	protein_codir	5.86622739	3.14E-07	6.72E-06
ENSG0000016	CXCL5	protein_codir	5.86052728	6.28E-12	7.11E-10
ENSG0000016	TAS1R3	protein_codir	5.86013718	4.83E-16	2.97E-13
ENSG0000013	BLK	protein_codir	5.8573313	4.36E-12	5.37E-10
ENSG0000016	KIF26B	protein_codir	5.85452443	1.06E-17	2.66E-14
ENSG0000019	CLEC4C	protein_codir	5.85352388	3.40E-06	5.13E-05
ENSG0000024	RP11-553L6.2	lncRNA	5.83193483	2.13E-07	4.78E-06
ENSG0000016	FCRL3	protein_codir	5.8314831	3.40E-14	9.20E-12
ENSG0000017	CCR9	protein_codir	5.82516146	8.73E-06	0.00011494
ENSG0000007	TP73	protein_codir	5.81572852	1.95E-11	1.87E-09
ENSG0000018	PLEKHG7	protein_codir	5.80624896	4.20E-09	1.67E-07
ENSG0000023	LINC01281	lncRNA	5.79565981	6.93E-05	0.00064321
ENSG0000013	IRF4	protein_codir	5.77482009	4.05E-18	1.40E-14
ENSG0000014	CHST4	protein_codir	5.75373381	3.34E-06	5.09E-05
ENSG0000018	TCL6	lncRNA	5.731644	4.44E-07	9.05E-06
ENSG0000021	UBD	protein_codir	5.73116533	2.66E-16	1.89E-13
ENSG0000016	SPIC	protein_codir	5.72455517	5.53E-06	7.76E-05
ENSG0000000	ARX	protein_codir	5.7234757	3.26E-07	6.95E-06
ENSG0000020	CLEC6A	protein_codir	5.710199	1.38E-05	0.0001682
ENSG0000010	MMP9	protein_codir	5.7069164	7.87E-14	1.75E-11
ENSG0000016	LARGE2	protein_codir	5.680421	1.04E-10	7.34E-09
ENSG0000018	RGS7	protein_codir	5.67904218	7.26E-07	1.39E-05
ENSG0000000	WNT16	protein_codir	5.65850615	1.64E-07	3.83E-06
ENSG0000016	NLRP7	protein_codir	5.65433328	8.91E-07	1.66E-05
ENSG0000017	SLC23A1	protein_codir	5.65308068	1.59E-06	2.74E-05
ENSG0000020	LCN8	protein_codir	5.64700283	0.0007359	0.00447996
ENSG0000013	JCHAIN	protein_codir	5.64006894	4.71E-11	3.82E-09
ENSG0000012	BCL2L14	protein_codir	5.63332934	9.84E-07	1.81E-05
ENSG0000013	CCL25	protein_codir	5.62713241	2.58E-06	4.06E-05
ENSG0000019	UGT2B17	protein_codir	5.62395714	5.71E-07	1.13E-05
ENSG0000019	MMP1	protein_codir	5.62043794	5.28E-09	2.04E-07

ENSG0000025	RP11-297B17.	lncRNA	5.60750705	2.82E-06	4.36E-05
ENSG0000001	DPEP1	protein_codir	5.60190638	6.49E-11	4.92E-09
ENSG0000027	RP11-138I18.:	lncRNA	5.57508719	6.92E-05	0.00064321
ENSG0000011	CCR6	protein_codir	5.5713711	5.63E-17	6.77E-14
ENSG0000017	TLR10	protein_codir	5.56810936	1.58E-10	1.06E-08
ENSG0000013	EHF	protein_codir	5.54718053	4.55E-08	1.28E-06
ENSG0000012	OBP2A	protein_codir	5.52712358	5.21E-05	0.00050853
ENSG0000024	CTD-2540L5.6	lncRNA	5.52562713	0.00010846	0.00093088
ENSG0000027	RALGPS2-AS1	lncRNA	5.52202954	9.88E-09	3.47E-07
ENSG0000012	DLGAP5	protein_codir	5.51668921	2.60E-11	2.33E-09
ENSG0000028	RP11-385M4.	lncRNA	5.4979991	8.35E-06	0.0001108
ENSG0000025	LINC02422	lncRNA	5.49553109	4.16E-09	1.67E-07
ENSG0000024	ZBTB20-AS1	lncRNA	5.48385676	4.03E-05	0.00040748
ENSG0000013	KRT34	protein_codir	5.47551144	0.00024412	0.00182086
ENSG0000027	RP11-80I3.1	lncRNA	5.47444456	4.20E-06	6.12E-05
ENSG0000022	LTB	protein_codir	5.4731433	5.67E-15	2.18E-12
ENSG0000024	AC096579.15	lncRNA	5.46959647	1.33E-07	3.20E-06
ENSG0000010	LGALS2	protein_codir	5.4641469	6.96E-10	3.74E-08
ENSG0000023	OR2I1P	protein_codir	5.46234209	6.06E-06	8.42E-05
ENSG0000012	HOXC12	protein_codir	5.45645199	1.19E-07	2.92E-06
ENSG0000025	RP11-624C23.	lncRNA	5.45300769	2.28E-05	0.0002556
ENSG0000027	ENSG0000027	protein_codir	5.44937411	2.65E-06	4.16E-05
ENSG0000012	ZBP1	protein_codir	5.44913287	8.19E-13	1.25E-10
ENSG0000025	RP11-203E8.1	lncRNA	5.43233797	3.40E-06	5.13E-05
ENSG0000004	TNFRSF17	protein_codir	5.4254421	1.49E-07	3.52E-06
ENSG0000018	NUGGC	protein_codir	5.42450654	2.08E-13	4.00E-11
ENSG0000024	RP11-514O12	protein_codir	5.42262281	3.56E-06	5.34E-05
ENSG0000025	RP11-689B22.	lncRNA	5.42206792	6.70E-08	1.79E-06
ENSG0000016	CLDN3	protein_codir	5.41225952	0.00016474	0.00132509
ENSG0000010	FCER2	protein_codir	5.40793924	6.37E-11	4.85E-09
ENSG0000015	ALAS2	protein_codir	5.39864976	4.09E-08	1.17E-06
ENSG0000013	CD27	protein_codir	5.38601165	2.67E-14	7.61E-12
ENSG0000016	GJB2	protein_codir	5.38078121	5.21E-15	2.05E-12
ENSG0000026	CTD-2576D5.4	lncRNA	5.37163108	2.32E-06	3.73E-05
ENSG0000018	TIGIT	protein_codir	5.36829645	1.60E-14	5.20E-12
ENSG0000008	P2RX5	protein_codir	5.3658112	6.32E-11	4.84E-09
ENSG0000026	RP11-235E17.	protein_codir	5.3544146	0.00319873	0.01422422
ENSG0000024	LINC02362	lncRNA	5.35404306	4.84E-12	5.78E-10
ENSG0000024	LACTB2-AS1	lncRNA	5.32884605	1.85E-06	3.09E-05
ENSG0000012	TMEM156	protein_codir	5.32859485	1.62E-10	1.08E-08
ENSG0000024	MIR3150BHG	lncRNA	5.32146174	0.00021232	0.00161999
ENSG0000026	RP11-61F12.1	lncRNA	5.32107381	8.70E-07	1.63E-05
ENSG0000017	GRIN1	protein_codir	5.31796982	3.81E-05	0.00039007
ENSG0000024	RP11-412P11.	lncRNA	5.31550206	1.60E-06	2.75E-05
ENSG0000007	LAMP3	protein_codir	5.29932048	1.53E-12	2.18E-10
ENSG0000013	EGR4	protein_codir	5.29080247	8.49E-07	1.60E-05
ENSG0000009	DERL3	protein_codir	5.29023214	2.09E-15	9.99E-13

ENSG0000018	INSYN2A	protein_codir	5.26266338	9.25E-08	2.35E-06
ENSG0000013	SMPDL3B	protein_codir	5.25644285	2.43E-11	2.22E-09
ENSG0000026	U62631.5	lncRNA	5.25555722	3.06E-05	0.00032518
ENSG0000028	KB-1572B10.1	lncRNA	5.24787872	1.71E-06	2.90E-05
ENSG0000024	LINC00996	lncRNA	5.24144613	9.22E-08	2.35E-06
ENSG0000023	LINC01800	lncRNA	5.23965231	0.00020795	0.001595
ENSG0000024	HLA-DOB	protein_codir	5.22612553	2.35E-13	4.39E-11
ENSG0000010	OVOL3	protein_codir	5.22572061	0.00012933	0.00108243
ENSG0000016	GP9	protein_codir	5.19348277	0.00011685	0.00099124
ENSG0000017	KRT2	protein_codir	5.18370156	4.18E-05	0.00041978
ENSG0000013	NXPE4	protein_codir	5.17518859	1.34E-08	4.51E-07
ENSG0000012	SGO1	protein_codir	5.17243821	1.91E-08	6.02E-07
ENSG0000016	C15orf48	protein_codir	5.16921427	5.84E-14	1.39E-11
ENSG0000007	P2RY10	protein_codir	5.16799213	1.82E-14	5.64E-12
ENSG0000026	RP5-1028K7.2	lncRNA	5.16627614	7.74E-08	2.02E-06
ENSG0000017	ZNF80	protein_codir	5.15402439	2.93E-06	4.51E-05
ENSG0000016	GLB1L3	protein_codir	5.15214329	1.52E-06	2.64E-05
ENSG0000015	ACOXL	protein_codir	5.1451355	7.58E-09	2.78E-07
ENSG0000008	MYBPC2	protein_codir	5.12328282	9.42E-10	4.82E-08
ENSG0000004	FOXP3	protein_codir	5.1161236	6.56E-11	4.95E-09
ENSG0000022	LINC00626	lncRNA	5.10794968	5.75E-05	0.00055332
ENSG0000015	ADGRG5	protein_codir	5.10653649	1.25E-11	1.28E-09
ENSG0000016	PGLYRP2	protein_codir	5.10608096	1.57E-06	2.71E-05
ENSG0000016	ICOS	protein_codir	5.10569051	1.09E-17	2.66E-14
ENSG0000004	USH2A	protein_codir	5.1002112	6.19E-06	8.55E-05
ENSG0000012	FFAR1	protein_codir	5.09853086	0.0001649	0.00132588
ENSG0000026	LINC01735	lncRNA	5.09751955	6.86E-06	9.32E-05
ENSG0000002	SLAMF7	protein_codir	5.0915809	1.88E-13	3.68E-11
ENSG0000012	LY9	protein_codir	5.08996041	3.53E-13	6.37E-11
ENSG0000019	HSH2D	protein_codir	5.08736021	2.75E-14	7.64E-12
ENSG0000024	SLC5A4-AS1	lncRNA	5.07403205	4.69E-06	6.74E-05
ENSG0000010	PVALB	protein_codir	5.07300005	3.55E-06	5.33E-05
ENSG0000021	RP11-1036E2(	lncRNA	5.05766645	0.00047305	0.00312725
ENSG0000008	EPYC	protein_codir	5.05701862	6.37E-06	8.74E-05
ENSG0000017	XCR1	protein_codir	5.05603224	1.20E-10	8.22E-09
ENSG0000012	GJB6	protein_codir	5.05402757	2.06E-06	3.39E-05
ENSG0000011	AICDA	protein_codir	5.04239145	3.14E-06	4.80E-05
ENSG0000024	LINC02273	lncRNA	5.02949893	9.82E-12	1.05E-09
ENSG0000010	ATP4A	protein_codir	5.02842432	0.0065775	0.02506145
ENSG0000010	CLEC4M	protein_codir	5.02673757	0.00468923	0.01931928
ENSG0000011	SDC1	protein_codir	5.02512661	1.33E-16	1.26E-13
ENSG0000027	KLHDC7B-DT	lncRNA	5.01477832	9.20E-06	0.00012008
ENSG0000010	MYBL2	protein_codir	5.01377875	3.97E-11	3.31E-09
ENSG0000013	WNT10A	protein_codir	5.009162	1.80E-10	1.19E-08
ENSG0000016	AMER2	protein_codir	5.00827265	0.00014355	0.00118216
ENSG0000001	CD22	protein_codir	5.0007899	1.17E-09	5.78E-08
ENSG0000017	FOXB1	protein_codir	4.99197724	3.95E-06	5.84E-05

ENSG0000012	TNFSF11	protein_codir	4.98767079	2.20E-08	6.81E-07
ENSG0000022	AC023590.1	lncRNA	4.98762202	7.37E-05	0.0006773
ENSG0000018	IDO2	protein_codir	4.97228226	1.43E-05	0.00017375
ENSG0000012	LAX1	protein_codir	4.96460201	1.31E-12	1.91E-10
ENSG0000026	TBC1D3G	protein_codir	4.96156013	0.0986317	0.19964594
ENSG0000015	TDO2	protein_codir	4.95940157	2.04E-10	1.32E-08
ENSG0000011	KISS1R	protein_codir	4.95608358	1.31E-05	0.00016102
ENSG0000017	FAM216B	protein_codir	4.95607205	2.35E-05	0.00026246
ENSG0000014	GPA33	protein_codir	4.94307897	4.02E-09	1.63E-07
ENSG0000016	FCMR	protein_codir	4.94282598	4.85E-13	8.21E-11
ENSG0000016	HTR3A	protein_codir	4.93572154	1.24E-05	0.00015426
ENSG0000023	ELFN1-AS1	lncRNA	4.93394509	1.17E-06	2.10E-05
ENSG0000022	LTA	protein_codir	4.93013718	1.26E-11	1.28E-09
ENSG0000027	GRIN2B	protein_codir	4.92240513	8.53E-08	2.20E-06
ENSG0000028	LINC00706	lncRNA	4.92011989	7.17E-05	0.00066242
ENSG0000026	LINC01539	lncRNA	4.91662473	0.00081756	0.00487185
ENSG0000017	SERPINA9	protein_codir	4.9018761	0.00052683	0.00341331
ENSG0000022	LINGO3	protein_codir	4.89777372	4.38E-08	1.24E-06
ENSG0000027	TBC1D3F	protein_codir	4.89016331	0.12132311	0.23307674
ENSG0000016	LHX8	protein_codir	4.88733156	0.00048814	0.00320779
ENSG0000028	XXyac-YR21IF	lncRNA	4.88376028	0.00015656	0.00126771
ENSG0000018	CCR4	protein_codir	4.88160894	1.30E-12	1.90E-10
ENSG0000021	AC129492.6	lncRNA	4.88074826	4.79E-06	6.86E-05
ENSG0000010	PIM2	protein_codir	4.86854755	1.16E-17	2.66E-14
ENSG0000027	AP001059.7	lncRNA	4.85401098	2.33E-05	0.00026063
ENSG0000015	CD1E	protein_codir	4.85097782	1.33E-09	6.42E-08
ENSG0000018	ANO9	protein_codir	4.84906952	2.79E-11	2.48E-09
ENSG0000018	GABRG3	protein_codir	4.84432697	0.00030111	0.0021574
ENSG0000014	ADAMTS16	protein_codir	4.84375039	6.69E-09	2.51E-07
ENSG0000028	CTC-273B12.1	lncRNA	4.84253891	1.73E-05	0.00020243
ENSG0000008	SIRPG	protein_codir	4.84210093	6.79E-11	5.08E-09
ENSG0000018	TTC24	protein_codir	4.83848508	2.10E-07	4.73E-06
ENSG0000016	MEI1	protein_codir	4.83484998	1.96E-14	5.88E-12
ENSG0000018	C4orf50	protein_codir	4.8288778	2.93E-15	1.27E-12
ENSG0000011	IL12B	protein_codir	4.82503455	0.00010121	0.00087741
ENSG0000028	RP11-125O18	lncRNA	4.82492913	4.31E-05	0.00043111
ENSG0000015	IGF2BP1	protein_codir	4.82482543	1.40E-06	2.45E-05
ENSG0000010	STMN2	protein_codir	4.82470486	3.41E-07	7.24E-06
ENSG0000027	RP11-785D18	lncRNA	4.82353829	4.15E-06	6.07E-05
ENSG0000028	CH17-112M04	lncRNA	4.82162497	0.00022558	0.00170258
ENSG0000022	POTEM	protein_codir	4.82006581	3.40E-05	0.00035507
ENSG0000000	SLC4A1	protein_codir	4.81924693	9.81E-08	2.47E-06
ENSG0000025	LINC02413	lncRNA	4.81621274	0.0011234	0.00627251
ENSG0000027	LL21NC02-1C1	lncRNA	4.81269793	5.10E-07	1.02E-05
ENSG0000014	TIMD4	protein_codir	4.81056965	1.26E-07	3.07E-06
ENSG0000017	GCSAM	protein_codir	4.8014348	7.91E-09	2.88E-07
ENSG0000021	LINGO4	protein_codir	4.79830882	2.82E-05	0.00030488

ENSG0000007	SCT	protein_codir	4.79077824	0.00028844	0.00208948
ENSG0000018	PMCH	protein_codir	4.78949342	5.95E-05	0.00056889
ENSG0000017	TMEM125	protein_codir	4.7843005	5.16E-06	7.31E-05
ENSG0000012	AUNIP	protein_codir	4.78252337	1.90E-05	0.00022007
ENSG0000025	LINC02384	lncRNA	4.77876072	1.13E-10	7.84E-09
ENSG0000016	KLK4	protein_codir	4.77039175	0.00128475	0.00698933
ENSG0000017	ENTHD1	protein_codir	4.77016679	8.56E-05	0.00076671
ENSG0000027	MICOS10-NBL	protein_codir	4.76995869	0.02330517	0.06675884
ENSG0000018	BEND4	protein_codir	4.75596095	2.32E-10	1.49E-08
ENSG0000011	HHLA2	protein_codir	4.7556406	0.00018194	0.00143148
ENSG0000013	ERMN	protein_codir	4.75419725	5.34E-12	6.18E-10
ENSG0000010	CCL22	protein_codir	4.75031051	4.95E-11	3.98E-09
ENSG0000028	CERNA2	lncRNA	4.74955181	0.00091186	0.00531903
ENSG0000028	RP1-147M19.	lncRNA	4.74770941	9.54E-05	0.00083577
ENSG0000022	AC092484.1	lncRNA	4.74416034	0.00530651	0.02125373
ENSG0000028	RP11-686F15.	lncRNA	4.74353747	0.0001154	0.0009819
ENSG0000010	IL21R	protein_codir	4.73641667	7.21E-15	2.59E-12
ENSG0000018	SHISAL2A	protein_codir	4.73301461	1.76E-09	7.99E-08
ENSG0000023	RP11-96K19.2	lncRNA	4.731242	3.82E-06	5.67E-05
ENSG0000024	LINC00926	lncRNA	4.72738391	4.75E-12	5.72E-10
ENSG0000017	CNTNAP2	protein_codir	4.7265406	2.59E-09	1.12E-07
ENSG0000010	SHD	protein_codir	4.72156984	1.98E-05	0.00022667
ENSG0000026	RP11-693J15.	lncRNA	4.72065306	2.13E-09	9.37E-08
ENSG0000016	AIM2	protein_codir	4.71870736	4.94E-10	2.81E-08
ENSG0000016	CSF2	protein_codir	4.71549619	7.50E-05	0.0006862
ENSG0000019	CR1L	protein_codir	4.7144989	4.02E-05	0.00040735
ENSG0000027	CTD-2313J17.	lncRNA	4.70582647	8.58E-05	0.00076814
ENSG0000028	RP4-644F6.4	lncRNA	4.70498952	7.62E-05	0.00069443
ENSG0000017	KCNJ10	protein_codir	4.70264225	1.65E-09	7.57E-08
ENSG0000022	RP11-191L9.4	lncRNA	4.70156866	0.00018737	0.00146354
ENSG0000016	MMP10	protein_codir	4.6918526	0.00121952	0.00670044
ENSG0000023	ANKRD44-AS1	lncRNA	4.69072983	3.59E-06	5.38E-05
ENSG0000015	SPC25	protein_codir	4.6788226	1.26E-07	3.07E-06
ENSG0000024	HBB	protein_codir	4.67853594	2.52E-08	7.67E-07
ENSG0000012	FOXJ1	protein_codir	4.67590635	2.97E-05	0.00031893
ENSG0000016	RHOH	protein_codir	4.66890275	6.94E-16	4.08E-13
ENSG0000025	RP11-1084E5.	lncRNA	4.66796472	1.11E-05	0.00013905
ENSG0000027	CTA-384D8.34	lncRNA	4.65641886	5.82E-09	2.23E-07
ENSG0000022	RP11-13P5.1	lncRNA	4.6552392	0.00137561	0.00736538
ENSG0000015	GPR15	protein_codir	4.65348127	8.34E-07	1.57E-05
ENSG0000028	RP3-416J7.6	lncRNA	4.64215326	0.0030923	0.01384268
ENSG0000012	PAEP	protein_codir	4.63897738	6.56E-05	0.00061536
ENSG0000027	TBC1D3K	protein_codir	4.6353753	0.13298893	0.24943327
ENSG0000001	ZBTB32	protein_codir	4.63089302	2.69E-10	1.67E-08
ENSG0000015	BANK1	protein_codir	4.62630108	2.88E-11	2.54E-09
ENSG0000025	RP11-1023P1	lncRNA	4.62361547	0.00182723	0.00915105
ENSG0000013	CXCL9	protein_codir	4.62330658	8.67E-11	6.27E-09

ENSG0000014	HORMAD1	protein_codir	4.6215038	1.35E-05	0.00016554
ENSG0000017	PCP2	protein_codir	4.61973754	8.24E-05	0.00074196
ENSG0000016	FOXQ1	protein_codir	4.61818384	9.91E-05	0.00086194
ENSG0000025	CTD-2509G16	lncRNA	4.61179743	8.92E-05	0.00079232
ENSG0000015	CARMIL2	protein_codir	4.60878778	7.84E-13	1.20E-10
ENSG0000011	CD5	protein_codir	4.60878045	2.19E-14	6.44E-12
ENSG0000003	SLC18A1	protein_codir	4.60361647	0.00013853	0.00114622
ENSG0000017	ABCA13	protein_codir	4.60299999	8.73E-06	0.00011494
ENSG0000019	KEL	protein_codir	4.59518693	5.90E-09	2.26E-07
ENSG0000010	CCL24	protein_codir	4.59454578	9.11E-09	3.24E-07
ENSG0000028	RP1-240K6.4	lncRNA	4.58892866	3.86E-05	0.00039325
ENSG0000016	TRAT1	protein_codir	4.58403577	3.80E-11	3.21E-09
ENSG0000014	NECTIN4	protein_codir	4.57882733	1.27E-08	4.29E-07
ENSG0000026	CTC-281F24.1	lncRNA	4.57851583	0.00027833	0.00202945
ENSG0000012	GPR42	protein_codir	4.57568901	9.31E-07	1.73E-05
ENSG0000025	RP11-284N8.3	lncRNA	4.5710626	3.92E-13	6.85E-11
ENSG0000022	AC093642.4	lncRNA	4.56295388	0.00044466	0.00297448
ENSG0000017	C12orf42	protein_codir	4.56258342	1.59E-06	2.74E-05
ENSG0000028	RP11-681B3.4	lncRNA	4.56159031	7.08E-07	1.36E-05
ENSG0000016	PLD4	protein_codir	4.5614665	1.31E-13	2.69E-11
ENSG0000004	TNFRSF9	protein_codir	4.56052249	2.31E-11	2.14E-09
ENSG0000008	TCF7	protein_codir	4.55806381	6.32E-14	1.48E-11
ENSG0000005	CBLN4	protein_codir	4.5554231	1.15E-13	2.42E-11
ENSG0000010	MIOX	protein_codir	4.55000864	0.00023202	0.00174143
ENSG0000010	GZMB	protein_codir	4.54582909	1.00E-14	3.48E-12
ENSG0000007	MOXD1	protein_codir	4.54283944	6.39E-14	1.48E-11
ENSG0000012	EDN3	protein_codir	4.54033552	0.00032702	0.00231419
ENSG0000013	ADAMDEC1	protein_codir	4.53599808	2.98E-08	8.85E-07
ENSG0000016	LPO	protein_codir	4.53566978	0.00082754	0.00492174
ENSG0000016	TSHR	protein_codir	4.53082697	6.96E-10	3.74E-08
ENSG0000013	GUCY2D	protein_codir	4.52914809	1.64E-06	2.79E-05
ENSG0000003	CDH1	protein_codir	4.52299449	2.59E-07	5.68E-06
ENSG0000017	LINC02694	lncRNA	4.52009236	2.23E-06	3.62E-05
ENSG0000017	ZBED2	protein_codir	4.51847972	3.46E-06	5.21E-05
ENSG0000025	RP11-1143G9	lncRNA	4.51687322	2.24E-05	0.00025177
ENSG0000023	LINC00426	lncRNA	4.51506981	1.35E-10	9.17E-09
ENSG0000016	NETO1	protein_codir	4.50937854	3.85E-09	1.56E-07
ENSG0000018	CXCR3	protein_codir	4.50805852	8.38E-12	9.11E-10
ENSG0000028	RP11-512C24.	lncRNA	4.50666924	2.25E-07	5.01E-06
ENSG0000013	DSC1	protein_codir	4.49290619	0.00011466	0.00097748
ENSG0000010	NTSR1	protein_codir	4.48788094	9.75E-05	0.0008501
ENSG0000010	KCNK10	protein_codir	4.48558258	1.66E-06	2.82E-05
ENSG0000025	IFNG-AS1	lncRNA	4.48104971	7.28E-11	5.44E-09
ENSG0000017	KCNA3	protein_codir	4.46989901	9.15E-11	6.56E-09
ENSG0000017	SDR16C5	protein_codir	4.46886971	0.00027864	0.00203058
ENSG0000009	CRISP3	protein_codir	4.4669863	0.00019877	0.00153739
ENSG0000018	HBA2	protein_codir	4.46640299	4.25E-08	1.21E-06

ENSG0000008	CETP	protein_codir	4.46391658	1.53E-09	7.15E-08
ENSG0000027	CD24	protein_codir	4.46241851	4.42E-11	3.65E-09
ENSG0000016	ZG16B	protein_codir	4.457495	9.17E-05	0.00080876
ENSG0000025	RP11-519G16	lncRNA	4.45328888	0.00017357	0.0013804
ENSG0000020	HBA1	protein_codir	4.45328696	7.90E-07	1.50E-05
ENSG0000016	IKZF3	protein_codir	4.45288628	1.26E-11	1.28E-09
ENSG0000027	RP11-81H14.1	lncRNA	4.45162521	2.43E-06	3.87E-05
ENSG0000028	RP1-236J16.3	lncRNA	4.44957666	2.31E-06	3.72E-05
ENSG0000024	TRBV11-2	lncRNA	4.44481244	2.54E-07	5.60E-06
ENSG0000028	RP3-383B8.1	lncRNA	4.44353108	6.72E-05	0.00062714
ENSG0000014	ILDR1	protein_codir	4.44191974	3.19E-07	6.82E-06
ENSG0000025	RP11-380I10.4	lncRNA	4.44119842	0.00028012	0.00203923
ENSG0000025	RP4-622L5.2	lncRNA	4.43198086	0.01165234	0.03923049
ENSG0000019	CSAG1	protein_codir	4.42744306	0.00135216	0.00726931
ENSG0000013	CHIA	protein_codir	4.42659344	0.00312026	0.01394299
ENSG0000000	AOC1	protein_codir	4.42591841	8.35E-10	4.36E-08
ENSG0000025	RP11-1070N1	lncRNA	4.4201354	0.03071341	0.08289888
ENSG0000009	IL5RA	protein_codir	4.41749866	1.13E-09	5.61E-08
ENSG0000014	LIX1	protein_codir	4.41473905	0.01394514	0.04503271
ENSG0000012	CXCR4	protein_codir	4.41277389	3.84E-15	1.63E-12
ENSG0000021	PVRIG	protein_codir	4.41106235	5.55E-11	4.39E-09
ENSG0000025	CTB-114C7.4	lncRNA	4.40902282	7.70E-06	0.00010321
ENSG0000017	RP11-23P13.6	lncRNA	4.40647317	1.52E-11	1.50E-09
ENSG0000028	RP11-419K12.	lncRNA	4.40645465	0.00036552	0.00253918
ENSG0000026	RP11-615I2.2	lncRNA	4.40390327	4.41E-07	9.02E-06
ENSG0000021	PTPRCAP	protein_codir	4.40287229	7.48E-13	1.16E-10
ENSG0000023	UICLM	lncRNA	4.39730255	0.00010879	0.00093284
ENSG0000006	CDH3	protein_codir	4.38675243	8.91E-14	1.92E-11
ENSG0000011	MYB	protein_codir	4.38617146	5.48E-11	4.35E-09
ENSG0000018	RTKN2	protein_codir	4.38196779	2.14E-12	2.92E-10
ENSG0000016	PCLAF	protein_codir	4.38006174	1.00E-08	3.51E-07
ENSG0000024	TRIM39-RPP2	protein_codir	4.37993059	0.02633372	0.07347043
ENSG0000020	C6orf99	lncRNA	4.37914881	0.0001173	0.00099459
ENSG0000010	CLNK	protein_codir	4.37849487	5.54E-10	3.09E-08
ENSG0000021	GCGR	protein_codir	4.37522781	0.00035051	0.00245213
ENSG0000028	RP11-559F19.	protein_codir	4.37272061	0.0004816	0.00317449
ENSG0000014	SYTL1	protein_codir	4.36965772	2.40E-12	3.22E-10
ENSG0000013	PCDH8	protein_codir	4.36741221	9.44E-05	0.00082895
ENSG0000018	BTLA	protein_codir	4.36689214	3.27E-11	2.82E-09
ENSG0000024	DEFA1B	protein_codir	4.36635788	1.11E-05	0.00013917
ENSG0000012	COL10A1	protein_codir	4.36240445	3.93E-07	8.18E-06
ENSG0000007	SP140	protein_codir	4.36036638	5.15E-12	6.05E-10
ENSG0000014	REN	protein_codir	4.36000963	0.00076347	0.00461222
ENSG0000026	RP11-87G24.6	lncRNA	4.35794202	0.00790108	0.0289235
ENSG0000028	NABP1-OT1	lncRNA	4.35740133	0.00595517	0.02319377
ENSG0000028	RP11-517H2.8	lncRNA	4.35178123	0.00014365	0.00118261
ENSG0000015	CD1C	protein_codir	4.34364051	1.24E-11	1.28E-09

ENSG0000027	CTD-2342N23	lncRNA	4.34346889	0.00014385	0.00118389
ENSG000001C	SALL4	protein_codir	4.34237976	7.63E-08	2.00E-06
ENSG0000023	LINC01685	lncRNA	4.34108103	6.89E-05	0.00064163
ENSG0000009	DLL3	protein_codir	4.33538981	0.0001421	0.00117229
ENSG0000016	FAAH2	protein_codir	4.33113794	6.26E-13	9.99E-11
ENSG0000018	PRAME	protein_codir	4.32694532	0.00088751	0.00520998
ENSG0000011	TREML2	protein_codir	4.32515938	1.18E-06	2.11E-05
ENSG0000012	LAMP5	protein_codir	4.31826492	2.13E-13	4.01E-11
ENSG0000012	FLT3	protein_codir	4.31766128	2.00E-11	1.89E-09
ENSG0000023	AP003774.1	lncRNA	4.31041944	0.0006911	0.0042636
ENSG0000016	CD52	protein_codir	4.30141675	7.91E-12	8.63E-10
ENSG0000012	ZNF831	protein_codir	4.29840044	1.35E-12	1.96E-10
ENSG0000028	RP11-542M13	lncRNA	4.29812049	0.00544215	0.02168067
ENSG0000013	STRA6	protein_codir	4.29793847	2.32E-12	3.15E-10
ENSG0000022	RP11-404F10.	lncRNA	4.29604335	3.79E-06	5.63E-05
ENSG0000012	CPNE5	protein_codir	4.29414379	9.06E-15	3.21E-12
ENSG0000023	XXYLT1-AS2	lncRNA	4.29180195	0.00035553	0.00248098
ENSG0000022	LINC01991	lncRNA	4.28548147	0.00664528	0.0252709
ENSG0000004	GUCA1A	protein_codir	4.28498997	0.00011019	0.000944
ENSG0000000	PRSS21	protein_codir	4.28338757	3.64E-06	5.44E-05
ENSG0000022	ITPKB-IT1	lncRNA	4.28335029	2.21E-05	0.00024875
ENSG0000026	Z84812.4	lncRNA	4.28267143	0.00010278	0.00088956
ENSG0000028	RP11-360L9.9	lncRNA	4.28160001	0.00019348	0.00150322
ENSG0000012	SH2D3A	protein_codir	4.28011812	3.02E-13	5.48E-11
ENSG0000011	CYTIP	protein_codir	4.27598668	1.76E-14	5.51E-12
ENSG0000024	LINC02014	lncRNA	4.27475139	0.00031021	0.00221431
ENSG0000011	SLAMF1	protein_codir	4.2705049	8.74E-17	9.83E-14
ENSG0000011	MYF6	protein_codir	4.26157389	0.00408644	0.01733754
ENSG0000017	SLC2A14	protein_codir	4.25862757	0.00725467	0.02705198
ENSG0000011	TRIM67	protein_codir	4.25251343	0.00023531	0.0017623
ENSG0000022	LINC01501	lncRNA	4.24945685	0.06869652	0.15284338
ENSG0000018	SBSN	protein_codir	4.24687267	0.00059743	0.00378375
ENSG0000016	CTLA4	protein_codir	4.24132041	8.17E-11	5.97E-09
ENSG0000014	GPR174	protein_codir	4.2372715	4.30E-10	2.48E-08
ENSG0000020	FAM221B	protein_codir	4.23704518	0.03382227	0.089233
ENSG0000015	SNX22	protein_codir	4.23499509	1.40E-09	6.67E-08
ENSG0000026	RP11-713C5.1	lncRNA	4.23476726	0.00122564	0.00672632
ENSG0000015	MEPE	protein_codir	4.22767992	0.01219416	0.04059955
ENSG0000028	RP11-438D8.6	lncRNA	4.22694414	0.00156471	0.00814499
ENSG0000017	PRND	protein_codir	4.22448394	8.01E-08	2.08E-06
ENSG0000016	TAGAP	protein_codir	4.21896152	6.64E-14	1.53E-11
ENSG0000014	IL19	protein_codir	4.2153525	0.00010761	0.00092451
ENSG0000013	TPTE2	protein_codir	4.21257007	7.12E-07	1.37E-05
ENSG0000004	UTS2	protein_codir	4.20719997	0.00013671	0.00113421
ENSG0000027	LINC02091	lncRNA	4.20603203	0.0008152	0.00486515
ENSG0000015	WNT7A	protein_codir	4.20363127	4.47E-05	0.00044445
ENSG0000025	PARP11-AS1	lncRNA	4.1915928	0.00015322	0.00124653

ENSG0000010	CENPM	protein_codir	4.19158272	1.50E-08	4.94E-07
ENSG0000016	IL23R	protein_codir	4.19115054	5.33E-05	0.00051933
ENSG0000009	CYP26A1	protein_codir	4.18388887	3.45E-07	7.31E-06
ENSG0000017	PARP15	protein_codir	4.17787075	5.64E-11	4.45E-09
ENSG0000016	JSRP1	protein_codir	4.17471286	2.14E-13	4.01E-11
ENSG0000017	KSR2	protein_codir	4.17370051	0.00030365	0.00217221
ENSG0000028	LINC02009	lncRNA	4.17362124	0.00029809	0.00213963
ENSG0000018	PDCD1	protein_codir	4.17263338	1.20E-11	1.24E-09
ENSG0000016	IL7R	protein_codir	4.17042753	4.57E-12	5.56E-10
ENSG0000022	RP13-297E16.	lncRNA	4.16438681	5.30E-07	1.06E-05
ENSG0000022	AC112721.2	lncRNA	4.16369848	0.00077971	0.00468574
ENSG0000018	SH2D1A	protein_codir	4.16295205	3.59E-13	6.44E-11
ENSG0000024	RP13-452N2.1	lncRNA	4.16235921	0.00054016	0.00348659
ENSG0000027	RP11-104L21.	lncRNA	4.16044431	0.0022112	0.01063257
ENSG0000015	SPAG17	protein_codir	4.15788846	0.00050654	0.00330364
ENSG0000012	TEX11	protein_codir	4.15332685	2.58E-07	5.67E-06
ENSG0000014	ESR2	protein_codir	4.15187709	3.64E-10	2.17E-08
ENSG0000028	RP3-355M6.3	protein_codir	4.15163283	0.00138041	0.00738072
ENSG0000022	TAS2R46	protein_codir	4.13803714	0.00013038	0.00108856
ENSG0000017	RRM2	protein_codir	4.1363381	3.56E-10	2.13E-08
ENSG0000017	TIGD3	protein_codir	4.13561042	1.40E-08	4.67E-07
ENSG0000020	INSYN2B	protein_codir	4.12922433	4.39E-05	0.00043743
ENSG0000013	GPR55	protein_codir	4.12187696	2.32E-08	7.14E-07
ENSG0000023	LINC01634	lncRNA	4.12129163	0.02853798	0.07838297
ENSG0000017	TBC1D10C	protein_codir	4.11273343	8.79E-12	9.44E-10
ENSG0000027	ENSG0000027	protein_codir	4.11190193	0.13403869	0.2507891
ENSG0000014	MKI67	protein_codir	4.11125357	6.43E-10	3.52E-08
ENSG0000009	IL11	protein_codir	4.10937976	9.57E-08	2.42E-06
ENSG0000016	MAJIN	protein_codir	4.10919496	0.0016061	0.00831809
ENSG0000018	CALHM1	protein_codir	4.10447728	0.00140684	0.007497
ENSG0000027	PRSS2	protein_codir	4.100156	0.00103708	0.00589509
ENSG0000020	LINC02408	lncRNA	4.09992731	2.35E-05	0.00026276
ENSG0000023	LINC01891	lncRNA	4.09741869	0.00422629	0.01778881
ENSG0000024	LINC02086	lncRNA	4.09503522	7.80E-05	0.00070809
ENSG0000017	GPR25	protein_codir	4.08883621	0.00116151	0.00643935
ENSG0000010	VSIG1	protein_codir	4.08302275	6.23E-07	1.22E-05
ENSG0000024	LINC02325	lncRNA	4.07717007	2.42E-06	3.87E-05
ENSG0000018	TRABD2A	protein_codir	4.07693099	6.97E-13	1.09E-10
ENSG0000012	SYNGR3	protein_codir	4.07408695	3.95E-11	3.30E-09
ENSG0000028	RP11-382L24.	lncRNA	4.06369875	0.04557646	0.11227452
ENSG0000014	IL2RG	protein_codir	4.06253838	5.14E-12	6.05E-10
ENSG0000022	MIAT	lncRNA	4.0614212	2.21E-09	9.66E-08
ENSG0000027	GS1-72M22.1	lncRNA	4.06118168	4.43E-05	0.00044045
ENSG0000019	CRYBA4	protein_codir	4.06030378	0.00015518	0.00125948
ENSG0000025	LIX1-AS1	lncRNA	4.05699238	0.00181083	0.00909367
ENSG0000013	ADGRE3	protein_codir	4.05601679	3.23E-07	6.91E-06
ENSG0000026	GRAPLDR	lncRNA	4.05596828	3.05E-05	0.00032454

ENSG0000000	TRAF3IP3	protein_codir	4.05231481	3.10E-10	1.90E-08
ENSG0000028	RP4-620F22.3	lncRNA	4.05017855	8.00E-07	1.51E-05
ENSG0000022	RP11-510C10.	lncRNA	4.05015874	0.02660013	0.07404712
ENSG0000014	FHAD1	protein_codir	4.04774817	1.46E-08	4.83E-07
ENSG0000024	LINC01060	lncRNA	4.04331786	0.00084735	0.00501687
ENSG0000022	RP3-395M20.	lncRNA	4.03978379	1.48E-05	0.0001789
ENSG0000025	RP11-567J20.	lncRNA	4.03956778	0.01369989	0.0444225
ENSG0000023	DEFA3	protein_codir	4.03618309	0.00123646	0.00676926
ENSG0000016	TAC3	protein_codir	4.03115527	0.0061107	0.02368807
ENSG0000011	KIAA1549L	protein_codir	4.02604891	4.54E-10	2.59E-08
ENSG0000012	DNAH8	protein_codir	4.01589947	1.30E-05	0.00015989
ENSG0000024	RP11-60A8.1	lncRNA	4.01321105	0.00546525	0.02175074
ENSG0000011	ITK	protein_codir	4.01167701	7.04E-13	1.10E-10
ENSG0000022	ITGB2-AS1	lncRNA	4.00888284	1.71E-09	7.82E-08
ENSG0000026	RP11-20G6.3	lncRNA	4.00683471	0.21351507	0.35325784
ENSG0000007	ACAP1	protein_codir	4.00313326	1.48E-12	2.14E-10
ENSG0000028	LNCDAT	lncRNA	4.00306302	0.00638509	0.02452421
ENSG0000012	LINC00544	lncRNA	4.00251853	0.00045711	0.00304077
ENSG0000025	LINC01484	lncRNA	3.99719241	6.79E-06	9.24E-05
ENSG0000013	KERA	protein_codir	3.99641683	0.00226303	0.01083116
ENSG0000017	AURKB	protein_codir	3.99336302	6.37E-09	2.40E-07
ENSG0000018	ZPBP2	protein_codir	3.99291835	0.00367029	0.01584702
ENSG0000028	RP11-275E15.	lncRNA	3.99267917	0.02975257	0.08097812
ENSG0000025	RP11-234B24.	lncRNA	3.99046917	0.01775103	0.05412115
ENSG0000024	LINC01096	lncRNA	3.98990768	0.00056368	0.00360137
ENSG0000018	C16orf54	protein_codir	3.98847515	1.44E-11	1.43E-09
ENSG0000022	RP1-153P14.5	lncRNA	3.98837687	0.00867556	0.03111933
ENSG0000025	P2RX5-TAX1B	protein_codir	3.98608364	1.42E-08	4.74E-07
ENSG0000026	RP11-44F14.2	lncRNA	3.98439284	0.00227853	0.01089748
ENSG0000007	CDHR2	protein_codir	3.98319307	0.00127392	0.00693763
ENSG0000028	RP11-350J20.	protein_codir	3.98226723	0.00050176	0.00328014
ENSG0000012	KIAA0087	lncRNA	3.98027596	0.00042113	0.0028432
ENSG0000018	C17orf99	protein_codir	3.97715353	0.00048545	0.00319473
ENSG0000025	LINC02416	lncRNA	3.97650822	0.00615957	0.02382894
ENSG0000017	UBE2C	protein_codir	3.97641613	5.13E-09	2.00E-07
ENSG0000023	WASIR2	lncRNA	3.97534206	0.00137185	0.00735169
ENSG0000016	CD3G	protein_codir	3.96921773	1.86E-10	1.22E-08
ENSG0000019	EGFL6	protein_codir	3.96611586	5.40E-07	1.07E-05
ENSG0000018	PDE6G	protein_codir	3.96417162	9.61E-07	1.78E-05
ENSG0000017	OVOL1	protein_codir	3.9582687	0.00202613	0.009917
ENSG0000010	CD40LG	protein_codir	3.95732689	3.79E-11	3.21E-09
ENSG0000022	LINC01724	lncRNA	3.95599253	0.00538055	0.02148174
ENSG0000018	TNFRSF18	protein_codir	3.95469068	2.20E-08	6.81E-07
ENSG0000028	LINC01163	lncRNA	3.95442385	0.00149543	0.00786696
ENSG0000017	TRH	protein_codir	3.95392993	0.00029377	0.00211793
ENSG0000016	UBASH3A	protein_codir	3.95205704	8.40E-10	4.37E-08
ENSG0000023	AP001057.1	lncRNA	3.94632288	1.46E-05	0.00017655

ENSG0000027	RP11-705C15.	lncRNA	3.94531559	0.00291405	0.01317498
ENSG0000022	RP11-390F4.3	lncRNA	3.943149	4.27E-07	8.76E-06
ENSG0000026	RP11-876N24	lncRNA	3.94104904	0.01965387	0.05848307
ENSG0000018	LINC00158	lncRNA	3.93741934	0.00035342	0.00246936
ENSG0000020	LORICRIN	protein_codir	3.93523752	0.0325135	0.08650638
ENSG0000011	SPP1	protein_codir	3.93461459	5.37E-08	1.48E-06
ENSG0000025	RP11-328J6.1	lncRNA	3.93324651	0.01097509	0.0375024
ENSG0000000	CD79B	protein_codir	3.93244201	5.24E-09	2.03E-07
ENSG0000020	LIPN	protein_codir	3.930354	2.67E-05	0.00029187
ENSG0000012	BCL11B	protein_codir	3.91846757	5.28E-14	1.33E-11
ENSG0000000	TTC22	protein_codir	3.91765371	3.40E-08	9.95E-07
ENSG0000007	HMMR	protein_codir	3.91629618	3.09E-09	1.30E-07
ENSG0000001	CD6	protein_codir	3.91219	3.21E-11	2.79E-09
ENSG0000012	GTSF1L	protein_codir	3.91180343	0.00121543	0.00668233
ENSG0000010	SPOCK2	protein_codir	3.90817259	1.73E-13	3.47E-11
ENSG0000023	CHRM3-AS2	lncRNA	3.90570639	5.18E-09	2.01E-07
ENSG0000026	AC006129.2	lncRNA	3.90543803	1.83E-10	1.20E-08
ENSG0000015	ISL2	protein_codir	3.90289235	7.03E-07	1.35E-05
ENSG0000027	RP5-1184F4.7	lncRNA	3.90276414	0.01217843	0.04056541
ENSG0000014	IGFBP1	protein_codir	3.90208155	0.00011285	0.00096499
ENSG0000007	DAPP1	protein_codir	3.90154547	7.86E-10	4.15E-08
ENSG0000022	LINC00381	lncRNA	3.89905189	0.00430879	0.01805077
ENSG0000018	HEPACAM2	protein_codir	3.88870883	0.00051132	0.00332687
ENSG0000028	RP5-820A21.2	lncRNA	3.88712409	3.08E-05	0.00032724
ENSG0000028	RP11-7O11.7	protein_codir	3.88688052	0.2279322	0.37019214
ENSG0000016	SLAMF6	protein_codir	3.88591713	1.39E-09	6.62E-08
ENSG0000014	OR4D1	protein_codir	3.88561345	0.00059865	0.00378975
ENSG0000028	CTA-445C9.17	lncRNA	3.88512285	0.0002089	0.00160141
ENSG0000007	DCX	protein_codir	3.88503348	0.00284877	0.01294343
ENSG0000020	ARRDC5	protein_codir	3.88483523	1.72E-07	3.97E-06
ENSG0000013	TESPA1	protein_codir	3.88387965	6.88E-12	7.70E-10
ENSG0000018	SGCZ	protein_codir	3.88333231	0.05078908	0.12197152
ENSG0000012	HJURP	protein_codir	3.88193607	5.40E-08	1.48E-06
ENSG0000027	RP11-212I21.5	lncRNA	3.88132906	0.01613382	0.05047098
ENSG0000010	PF4V1	protein_codir	3.88109947	0.00499335	0.02024609
ENSG0000018	LCK	protein_codir	3.87812988	1.34E-12	1.95E-10
ENSG0000028	CTA-246H3.13	lncRNA	3.87521071	0.01103258	0.03764298
ENSG0000023	LINC02805	lncRNA	3.87239664	0.00129474	0.0070312
ENSG0000018	ASTL	protein_codir	3.87145165	9.44E-08	2.40E-06
ENSG0000028	RP3-413H6.3	lncRNA	3.87041361	0.00179283	0.00902462
ENSG0000022	LEMD1-DT	lncRNA	3.86782739	0.00080512	0.00481434
ENSG0000025	LINC02754	lncRNA	3.86727942	0.00199026	0.00978828
ENSG0000027	CTD-2035E11.	lncRNA	3.86224696	0.0002392	0.00178804
ENSG0000010	CACNA1I	protein_codir	3.86158423	2.30E-08	7.07E-07
ENSG0000022	LINC01857	lncRNA	3.85775984	1.45E-06	2.53E-05
ENSG0000028	H3C2	protein_codir	3.85661293	0.0028465	0.01293737
ENSG0000025	RP11-283G6.4	lncRNA	3.85628372	0.00121673	0.00668755

ENSG0000026	RP11-567M16	lncRNA	3.85347538	0.01693835	0.05227815
ENSG0000005	PRDM1	protein_codir	3.85136121	4.47E-14	1.16E-11
ENSG0000023	LNCARSR	lncRNA	3.84996719	0.00261343	0.01210107
ENSG0000008	HBQ1	protein_codir	3.84715285	0.01955243	0.05827732
ENSG0000017	AQP4	protein_codir	3.84601415	6.93E-05	0.00064321
ENSG0000025	DCANP1	protein_codir	3.84589733	0.00050314	0.00328527
ENSG0000012	PACSIN1	protein_codir	3.84572674	7.01E-10	3.75E-08
ENSG0000027	HEATR9	protein_codir	3.84205835	0.00029967	0.00214764
ENSG0000010	TJP3	protein_codir	3.84171156	3.66E-12	4.56E-10
ENSG0000015	FAM167A	protein_codir	3.83815433	3.42E-06	5.17E-05
ENSG0000027	H2AC14	protein_codir	3.83739882	0.00278886	0.01272986
ENSG0000026	CTD-2369P2.8	lncRNA	3.83436646	0.00011597	0.0009849
ENSG0000007	SLC12A3	protein_codir	3.83229264	1.37E-05	0.00016798
ENSG0000007	SNCB	protein_codir	3.8316185	0.00327144	0.01448495
ENSG0000024	RP11-223C24.	lncRNA	3.83003139	0.00060065	0.00379805
ENSG0000018	P2RY8	protein_codir	3.82678735	2.97E-13	5.47E-11
ENSG0000026	CTD-3222D19	lncRNA	3.82320517	0.00115055	0.0063914
ENSG0000020	NCR3	protein_codir	3.82300368	2.80E-06	4.34E-05
ENSG0000013	ITGB7	protein_codir	3.82130331	8.77E-12	9.44E-10
ENSG0000012	S1PR4	protein_codir	3.81759777	3.84E-10	2.26E-08
ENSG0000013	LEF1	protein_codir	3.81407563	1.19E-11	1.23E-09
ENSG0000019	CD3E	protein_codir	3.81272247	6.33E-13	1.00E-10
ENSG0000011	CD48	protein_codir	3.80907461	1.78E-11	1.72E-09
ENSG0000020	EXOC3L4	protein_codir	3.80887099	3.86E-08	1.11E-06
ENSG0000011	FGF23	protein_codir	3.80776384	0.06674708	0.14994341
ENSG0000024	AC008697.1	lncRNA	3.80708528	0.00027029	0.00197864
ENSG0000014	SCML4	protein_codir	3.80527503	1.73E-09	7.90E-08
ENSG0000010	KLF1	protein_codir	3.80233349	0.00011089	0.00094966
ENSG0000027	LINC01480	lncRNA	3.80220386	1.90E-07	4.33E-06
ENSG0000016	CILP2	protein_codir	3.80096387	7.92E-10	4.18E-08
ENSG0000010	SLC6A2	protein_codir	3.79972206	0.00016554	0.00132915
ENSG0000001	GIPR	protein_codir	3.79738268	8.93E-09	3.19E-07
ENSG0000018	TMPRSS6	protein_codir	3.79698486	0.00060643	0.00382672
ENSG0000012	HOXC13	protein_codir	3.7950277	0.007065	0.02649134
ENSG0000012	E2F8	protein_codir	3.79466402	1.95E-06	3.24E-05
ENSG0000018	ENPP7	protein_codir	3.79446921	0.00126027	0.00687512
ENSG0000017	CPNE7	protein_codir	3.79256898	2.20E-07	4.91E-06
ENSG0000006	SPAG4	protein_codir	3.79192875	2.16E-11	2.03E-09
ENSG0000011	GZMK	protein_codir	3.79057312	7.22E-12	8.04E-10
ENSG0000023	LILRA4	protein_codir	3.78910474	3.06E-06	4.69E-05
ENSG0000026	RP11-214O1.3	lncRNA	3.78805419	3.39E-05	0.00035403
ENSG0000028	AC000082.4	lncRNA	3.78790803	0.01054212	0.03629684
ENSG0000013	RAB11FIP4	protein_codir	3.7866996	4.40E-12	5.41E-10
ENSG0000009	NXPE1	protein_codir	3.78344162	0.00025105	0.00186249
ENSG0000018	ERCC6L	protein_codir	3.78318385	9.70E-07	1.79E-05
ENSG0000019	KLHL14	protein_codir	3.78237856	3.51E-09	1.45E-07
ENSG0000010	DNTT	protein_codir	3.78111698	0.03021988	0.08192709

ENSG0000007	TUBA3D	protein_codir	3.78064402	0.00953806	0.03351242
ENSG0000018	XKRX	protein_codir	3.77985356	2.80E-06	4.35E-05
ENSG0000016	KCNF1	protein_codir	3.77948051	0.00014754	0.00120996
ENSG0000023	CLEC2L	protein_codir	3.77870091	0.00129832	0.00704928
ENSG0000014	PLEKHS1	protein_codir	3.77693344	1.61E-05	0.00019068
ENSG0000026	CTD-3214H19	lncRNA	3.77613645	0.00119071	0.00656962
ENSG0000022	SLC12A8	protein_codir	3.77329741	2.07E-10	1.33E-08
ENSG0000026	MAP3K14-AS1	lncRNA	3.77030144	3.65E-10	2.17E-08
ENSG0000015	CPA5	protein_codir	3.76902687	0.00179544	0.00903443
ENSG0000018	BPIFB4	protein_codir	3.76888559	0.0008634	0.00509013
ENSG0000025	ETV3L	protein_codir	3.76812456	0.00558178	0.02208405
ENSG0000018	SP6	protein_codir	3.76793397	1.67E-09	7.65E-08
ENSG0000006	LTK	protein_codir	3.76535052	7.38E-12	8.19E-10
ENSG0000023	LINC02642	lncRNA	3.76359216	0.00055333	0.00354923
ENSG0000027	RP5-874C20.8	protein_codir	3.76194329	0.00676492	0.02562356
ENSG0000013	VRTN	protein_codir	3.76167305	0.03275156	0.08701406
ENSG0000016	CD3D	protein_codir	3.76166206	2.79E-12	3.64E-10
ENSG0000019	ADGRA1	protein_codir	3.7615775	0.00868374	0.03114466
ENSG0000028	RP11-157L14.	lncRNA	3.76005974	0.00103411	0.00588302
ENSG0000020	IGFL2	protein_codir	3.75998311	0.00193051	0.00954368
ENSG0000018	RTP5	protein_codir	3.75982853	1.30E-06	2.29E-05
ENSG0000010	MAP4K1	protein_codir	3.7597625	4.89E-11	3.96E-09
ENSG0000017	RASGRP1	protein_codir	3.75681062	3.90E-13	6.85E-11
ENSG0000026	RP4-647J21.1	lncRNA	3.75394372	4.85E-13	8.21E-11
ENSG0000026	RP4-555D20.2	lncRNA	3.75334451	4.12E-06	6.04E-05
ENSG0000025	RP11-664I21.(	lncRNA	3.75200128	0.00114079	0.00634233
ENSG0000007	SMC1B	protein_codir	3.75157454	0.00017186	0.00137112
ENSG0000010	CRTAM	protein_codir	3.74971978	6.54E-10	3.57E-08
ENSG0000016	KLK1	protein_codir	3.74948304	1.06E-06	1.93E-05
ENSG0000027	RP11-326K13.	lncRNA	3.74941969	0.00909363	0.03233895
ENSG0000020	HBM	protein_codir	3.7489731	0.00417519	0.01764136
ENSG0000026	AC009133.17	lncRNA	3.74896445	3.40E-07	7.22E-06
ENSG0000018	NCMAP	protein_codir	3.74842764	2.20E-06	3.57E-05
ENSG0000011	ZAP70	protein_codir	3.74766993	1.32E-11	1.33E-09
ENSG0000028	CH17-264L24.	lncRNA	3.74419253	7.35E-11	5.47E-09
ENSG0000013	CACNG6	protein_codir	3.74398566	0.00127226	0.00693195
ENSG0000016	DEUP1	protein_codir	3.74357271	0.00335208	0.01476863
ENSG0000000	E2F2	protein_codir	3.7434296	3.42E-07	7.26E-06
ENSG0000027	LINC01670	lncRNA	3.74054961	0.00299817	0.01349346
ENSG0000004	ROS1	protein_codir	3.73961271	0.00228919	0.01093743
ENSG0000013	NUSAP1	protein_codir	3.73869756	1.05E-10	7.36E-09
ENSG0000017	PIK3CD-AS1	lncRNA	3.73859611	7.68E-05	0.00069944
ENSG0000016	TMIGD2	protein_codir	3.73706163	5.07E-09	1.98E-07
ENSG0000014	AQP10	protein_codir	3.73611706	0.00109182	0.00613646
ENSG0000018	KLRC4	protein_codir	3.7321926	0.00059016	0.00374456
ENSG0000025	COPDA1	lncRNA	3.73193244	0.01049009	0.03616353
ENSG0000028	RP11-413N10	lncRNA	3.73080983	6.23E-05	0.00058995

ENSG0000018	SELL	protein_codir	3.73068413	4.18E-09	1.67E-07
ENSG0000023	RP3-470B24.5	lncRNA	3.73053033	0.0454213	0.11199216
ENSG0000028	RP11-305M3.	lncRNA	3.7299866	0.03982661	0.10117834
ENSG0000017	TSPEAR	protein_codir	3.72981839	0.00037828	0.00261204
ENSG0000009	SEL1L3	protein_codir	3.72929117	2.89E-11	2.54E-09
ENSG0000022	LINC00200	lncRNA	3.72803931	0.02766356	0.0764144
ENSG0000020	CRLF2	protein_codir	3.72785476	6.37E-06	8.74E-05
ENSG0000013	SIT1	protein_codir	3.72452321	3.96E-07	8.24E-06
ENSG0000022	GK-IT1	lncRNA	3.72387536	0.02403685	0.0684219
ENSG0000025	LINC02126	lncRNA	3.72263118	0.00270246	0.0124279
ENSG0000025	LINC01579	lncRNA	3.71371577	0.00997644	0.03473456
ENSG0000016	SPTA1	protein_codir	3.71211536	2.54E-06	4.01E-05
ENSG0000018	TMEM119	protein_codir	3.71192514	2.71E-17	4.02E-14
ENSG0000016	NLRC3	protein_codir	3.71074683	1.78E-11	1.72E-09
ENSG0000019	CD247	protein_codir	3.71058782	1.99E-12	2.74E-10
ENSG0000016	PYHIN1	protein_codir	3.71030365	3.01E-14	8.23E-12
ENSG0000013	NUP210	protein_codir	3.71004504	3.97E-12	4.92E-10
ENSG0000011	B3GAT2	protein_codir	3.7098799	0.0003034	0.0021715
ENSG0000025	RP11-383J24.	lncRNA	3.70976287	0.0066849	0.02537267
ENSG0000012	GPR31	protein_codir	3.70647818	0.00096439	0.00556781
ENSG0000012	SIRPD	protein_codir	3.70610931	0.00017795	0.00140706
ENSG0000019	DTHD1	protein_codir	3.70556853	4.14E-08	1.18E-06
ENSG0000014	DQX1	protein_codir	3.70463566	0.00017057	0.00136196
ENSG0000011	VSX2	protein_codir	3.70047744	0.00366606	0.01583371
ENSG0000023	PDXP-DT	lncRNA	3.69951985	0.01130802	0.03832481
ENSG0000023	LINC02257	lncRNA	3.69880828	0.00762041	0.02813093
ENSG0000026	TAT-AS1	lncRNA	3.69793405	0.00443254	0.01845445
ENSG0000025	RP11-486L19.	lncRNA	3.69754354	0.07328665	0.16032329
ENSG0000026	DNAH17-AS1	lncRNA	3.69658781	0.0037881	0.0162844
ENSG0000016	RAB3B	protein_codir	3.69461247	1.48E-07	3.49E-06
ENSG0000012	FBXL16	protein_codir	3.69056091	3.39E-09	1.41E-07
ENSG0000022	RUNX3-AS1	lncRNA	3.689234	0.00142614	0.00758227
ENSG0000011	CD2	protein_codir	3.68827213	3.14E-10	1.92E-08
ENSG0000022	PATL2	protein_codir	3.68656447	2.68E-11	2.39E-09
ENSG0000013	TOP2A	protein_codir	3.686165	1.04E-09	5.26E-08
ENSG0000018	DAZ1	protein_codir	3.68550561	0.01429793	0.04591962
ENSG0000011	IFNG	protein_codir	3.68410051	4.85E-08	1.35E-06
ENSG0000013	C7orf69	lncRNA	3.68000229	0.00546926	0.02175484
ENSG0000019	CARD11	protein_codir	3.67708636	1.38E-10	9.36E-09
ENSG0000018	SLC35F3	protein_codir	3.67619613	2.30E-06	3.71E-05
ENSG0000025	SMIM6	protein_codir	3.67546821	0.00260593	0.01207242
ENSG0000025	KIAA1210	protein_codir	3.67471015	0.03452533	0.09067041
ENSG0000014	ST8SIA2	protein_codir	3.67469689	0.0014841	0.00782117
ENSG0000027	RP11-357H14	lncRNA	3.67306778	0.0002521	0.00186773
ENSG0000022	AC012123.1	lncRNA	3.67267537	0.07019238	0.15538453
ENSG0000028	RP11-624D20	lncRNA	3.67176268	0.03784925	0.09716481
ENSG0000028	KB-836E9.1	lncRNA	3.67047058	0.00494292	0.02008584

ENSG0000023	VAV3-AS1	lncRNA	3.67034186	0.0192068	0.05751213
ENSG0000028	CTD-2360A17	lncRNA	3.66871772	0.00131912	0.00713696
ENSG0000011	RALGPS2	protein_codir	3.66563602	1.26E-15	6.81E-13
ENSG0000012	ADM2	protein_codir	3.66393171	1.06E-07	2.66E-06
ENSG0000023	LINC01934	lncRNA	3.66380179	9.89E-08	2.48E-06
ENSG0000028	RP1-118J21.2	lncRNA	3.66364681	0.00030348	0.0021715
ENSG0000025	RP11-219E7.1	lncRNA	3.66240539	0.00125796	0.0068675
ENSG0000026	MMP2-AS1	lncRNA	3.66129377	5.23E-05	0.0005104
ENSG0000023	LINC01055	lncRNA	3.65621578	1.44E-05	0.00017403
ENSG0000016	BUB1	protein_codir	3.65597413	5.64E-09	2.17E-07
ENSG0000002	RUNX3	protein_codir	3.65489243	3.64E-16	2.34E-13
ENSG0000024	RP11-125O18	lncRNA	3.65400947	0.00602702	0.02340309
ENSG0000028	RP11-1136L8.	lncRNA	3.65148122	0.00261037	0.01208892
ENSG0000014	PLAC8	protein_codir	3.65043874	4.33E-08	1.22E-06
ENSG0000020	MPIG6B	protein_codir	3.64708485	3.33E-09	1.39E-07
ENSG0000017	IGDCC3	protein_codir	3.64692304	0.00780208	0.02864082
ENSG0000026	GTSCR1	lncRNA	3.64371073	0.00420767	0.01773206
ENSG0000028	RP3-453I5.3	lncRNA	3.64202028	0.11613285	0.22547373
ENSG0000018	GPR1	protein_codir	3.64025918	5.91E-11	4.60E-09
ENSG0000028	RP11-206F17.	lncRNA	3.63962726	0.00330442	0.01460287
ENSG0000011	CLDN16	protein_codir	3.63858536	0.00178604	0.00900683
ENSG0000010	CEMIP	protein_codir	3.63631826	3.40E-08	9.95E-07
ENSG0000010	WNT2	protein_codir	3.63563231	4.65E-08	1.31E-06
ENSG0000011	SLC46A2	protein_codir	3.63306459	5.46E-07	1.09E-05
ENSG0000025	RP11-326C3.2	lncRNA	3.63044352	4.95E-08	1.38E-06
ENSG0000027	RP11-329B9.5	lncRNA	3.62586925	0.01794112	0.05453817
ENSG0000013	ACY3	protein_codir	3.62365211	1.48E-05	0.0001785
ENSG0000016	MELK	protein_codir	3.6228802	6.42E-09	2.41E-07
ENSG0000018	KIF18B	protein_codir	3.62122847	1.24E-06	2.20E-05
ENSG0000011	CD69	protein_codir	3.62091605	1.50E-15	7.82E-13
ENSG0000015	TMEM163	protein_codir	3.61982898	1.91E-08	6.03E-07
ENSG0000028	RP11-766N7.5	protein_codir	3.6187058	0.05780426	0.13401777
ENSG0000012	HS3ST3B1	protein_codir	3.61361686	8.38E-11	6.10E-09
ENSG0000010	SLA2	protein_codir	3.61275241	6.78E-15	2.46E-12
ENSG0000014	FNDC7	protein_codir	3.60995822	0.00617362	0.02387502
ENSG0000018	LINC02870	lncRNA	3.60931657	0.00018169	0.00143062
ENSG0000017	RBMXL2	protein_codir	3.60794506	0.00328487	0.01453046
ENSG0000026	PWRN2	lncRNA	3.6046919	0.07283379	0.15960562
ENSG0000015	JAKMIP1	protein_codir	3.60456097	4.26E-11	3.53E-09
ENSG0000022	LINC02577	lncRNA	3.60202858	0.00242095	0.01140874
ENSG0000026	CTD-2583P5.3	lncRNA	3.60159571	8.66E-05	0.00077373
ENSG0000016	PBK	protein_codir	3.60107961	4.67E-06	6.71E-05
ENSG0000025	RP11-263K4.5	lncRNA	3.60106445	0.03431099	0.09027143
ENSG0000027	RP11-327F22.	lncRNA	3.59987276	0.00062825	0.00393741
ENSG0000019	GZMM	protein_codir	3.59627093	5.59E-16	3.36E-13
ENSG0000017	GAPT	protein_codir	3.59079182	2.00E-08	6.24E-07
ENSG0000026	RP1-134E15.3	lncRNA	3.5853357	0.00011388	0.00097258

ENSG0000022	RP11-202G18	lncRNA	3.58506983	0.0015127	0.00793556
ENSG0000020	TRIM10	protein_codir	3.58411691	0.00127399	0.00693763
ENSG0000010	ICAM4	protein_codir	3.58354046	2.54E-10	1.60E-08
ENSG0000011	EVA1A	protein_codir	3.58126677	2.00E-08	6.26E-07
ENSG0000014	PLCH2	protein_codir	3.58061374	2.18E-09	9.55E-08
ENSG0000026	RP11-219A15	protein_codir	3.57397539	0.06605488	0.14879992
ENSG0000022	LINC01806	lncRNA	3.56994954	2.02E-06	3.33E-05
ENSG0000015	PLEKHG4B	protein_codir	3.56779307	1.06E-06	1.93E-05
ENSG0000016	TBXT	protein_codir	3.56625194	0.0709244	0.15656297
ENSG0000013	CA1	protein_codir	3.56552825	0.00499423	0.0202467
ENSG0000026	CSAG3	protein_codir	3.56524685	0.02428356	0.06897491
ENSG0000001	CCDC88C	protein_codir	3.56523172	2.04E-16	1.61E-13
ENSG0000028	RP11-339H12	lncRNA	3.56478932	0.00113008	0.00629798
ENSG0000011	PLPPR5	protein_codir	3.56415063	0.08141265	0.17298387
ENSG0000024	LINC00861	lncRNA	3.56310619	1.50E-06	2.60E-05
ENSG0000021	MEF2B	protein_codir	3.56135067	2.32E-07	5.14E-06
ENSG0000027	BORCS7-ASM	protein_codir	3.56057122	0.26976926	0.41756484
ENSG0000026	RP11-680C21.	lncRNA	3.55969902	0.00264712	0.01222226
ENSG0000001	CYP24A1	protein_codir	3.55913613	0.03532543	0.09220263
ENSG0000014	FAM163A	protein_codir	3.55908566	6.72E-05	0.00062758
ENSG0000020	AARD	protein_codir	3.55881092	3.11E-09	1.31E-07
ENSG0000007	SIDT1	protein_codir	3.55858746	1.02E-12	1.51E-10
ENSG0000016	ANKRD30BL	protein_codir	3.5555741	0.00163906	0.00845551
ENSG0000027	GS1-393G12.1	lncRNA	3.5547767	0.02151156	0.06271367
ENSG0000016	DEFA4	protein_codir	3.55463249	0.00306157	0.01372127
ENSG0000015	BTBD11	protein_codir	3.55252271	3.22E-16	2.12E-13
ENSG0000014	EPHA1	protein_codir	3.55192529	3.92E-09	1.59E-07
ENSG0000028	DISC2	lncRNA	3.55157937	0.01657992	0.05143631
ENSG0000026	RP11-945C19.	lncRNA	3.55071378	0.00200362	0.00984524
ENSG0000018	FUT7	protein_codir	3.54929625	1.87E-06	3.12E-05
ENSG0000017	APOBEC3B	protein_codir	3.5488463	8.59E-10	4.45E-08
ENSG0000015	CD96	protein_codir	3.54778628	1.78E-11	1.72E-09
ENSG0000025	RP11-493L12.	lncRNA	3.54735681	0.03759749	0.09668026
ENSG0000014	SKAP1	protein_codir	3.54633532	1.90E-10	1.24E-08
ENSG0000024	RP3-455J7.4	lncRNA	3.54580177	0.00034679	0.00242921
ENSG0000017	RP11-429E11.	lncRNA	3.54558378	0.00341363	0.01497056
ENSG0000026	RP13-580F15.	lncRNA	3.54496572	0.00015138	0.00123522
ENSG0000011	NEK2	protein_codir	3.54336752	1.06E-07	2.65E-06
ENSG0000011	RASAL1	protein_codir	3.54295509	3.46E-05	0.00036028
ENSG0000010	IL4I1	protein_codir	3.54260788	1.47E-09	6.93E-08
ENSG0000016	CXCL11	protein_codir	3.54131198	6.46E-10	3.53E-08
ENSG0000022	LINC02648	lncRNA	3.54076126	0.00102329	0.00583228
ENSG0000007	CAMSAP3	protein_codir	3.53965708	2.70E-06	4.22E-05
ENSG0000026	RP13-126P21.	lncRNA	3.53951679	0.01237248	0.04103362
ENSG0000010	CD37	protein_codir	3.53824082	8.40E-10	4.37E-08
ENSG0000025	RP11-111A22	lncRNA	3.53786017	0.00387978	0.01659847
ENSG0000018	ANKRD30B	protein_codir	3.53733055	0.00168974	0.00865234

ENSG0000028	RP5-967N21.1	protein_codir	3.53725433	0.18840633	0.32227552
ENSG0000016	UGT3A2	protein_codir	3.53513934	0.00165482	0.00851454
ENSG0000017	CDK5R2	protein_codir	3.533546	0.02391342	0.06811971
ENSG0000017	BEND2	protein_codir	3.53329541	0.08327398	0.17569531
ENSG0000023	PCDHA13	protein_codir	3.53201135	3.92E-05	0.0003983
ENSG0000013	IDO1	protein_codir	3.53102066	1.10E-09	5.47E-08
ENSG0000023	AC009495.2	lncRNA	3.52581406	0.00072011	0.00440323
ENSG0000020	CXorf65	protein_codir	3.52563303	9.51E-05	0.00083385
ENSG0000027	RP11-9N12.2	lncRNA	3.52490808	0.00019673	0.00152377
ENSG0000017	ISG20	protein_codir	3.52417923	6.17E-13	9.96E-11
ENSG0000016	PIGR	protein_codir	3.52173224	5.78E-05	0.00055553
ENSG0000028	RP11-496N12	lncRNA	3.52152083	0.02944027	0.08031809
ENSG0000028	RP11-358P11.	lncRNA	3.52043092	0.02410264	0.06854991
ENSG0000017	ZBTB44-DT	lncRNA	3.51937568	0.00079886	0.00478418
ENSG0000022	AC006460.2	lncRNA	3.51818349	2.81E-05	0.00030354
ENSG0000011	IL17F	protein_codir	3.51804102	0.08769141	0.18268493
ENSG0000026	RP11-161M6.	lncRNA	3.51408755	0.00169103	0.00865413
ENSG0000013	TROAP	protein_codir	3.51212304	9.87E-08	2.48E-06
ENSG0000022	KRTAP1-5	protein_codir	3.51000484	0.04722202	0.11520822
ENSG0000000	ITGAL	protein_codir	3.50856691	2.27E-09	9.88E-08
ENSG0000020	PABPN1L	protein_codir	3.50754617	0.02094709	0.06145428
ENSG0000023	LINC01010	lncRNA	3.5072952	3.59E-06	5.38E-05
ENSG0000010	ATP1A3	protein_codir	3.50620348	8.66E-07	1.62E-05
ENSG0000028	RP11-114N1.1	lncRNA	3.50605193	0.00617943	0.0238908
ENSG0000010	FA2H	protein_codir	3.50478282	4.98E-07	1.00E-05
ENSG0000027	XX-C00717C0	lncRNA	3.50344622	0.00438326	0.01829339
ENSG0000018	TENT5C	protein_codir	3.50280776	3.42E-12	4.31E-10
ENSG0000023	LINC02532	lncRNA	3.50159564	0.04362701	0.10853701
ENSG0000023	LINC02723	lncRNA	3.4996549	0.00222246	0.01067613
ENSG0000025	LINC00239	lncRNA	3.49482919	3.24E-05	0.00034078
ENSG0000014	LINC00525	lncRNA	3.49447798	0.00031026	0.00221431
ENSG0000028	RP11-29A19.3	lncRNA	3.49403471	0.00853867	0.03071831
ENSG0000008	BIRC5	protein_codir	3.49317949	2.88E-08	8.59E-07
ENSG0000027	RP11-568J23.	lncRNA	3.49236325	0.00359067	0.0155882
ENSG0000022	SATB1-AS1	lncRNA	3.49210746	6.28E-09	2.37E-07
ENSG0000010	SLC32A1	protein_codir	3.48742007	0.00211907	0.01027179
ENSG0000012	NPY	protein_codir	3.48512288	0.11724654	0.22702954
ENSG0000017	HASPIN	protein_codir	3.4845912	1.65E-06	2.80E-05
ENSG0000026	USP7-AS1	lncRNA	3.48434696	0.00169794	0.00868304
ENSG0000025	RP11-809N8.4	lncRNA	3.48270319	0.00563871	0.02225192
ENSG0000022	KIF1C-AS1	lncRNA	3.4824071	1.87E-05	0.00021708
ENSG0000017	HSF5	protein_codir	3.48214264	3.73E-06	5.55E-05
ENSG0000018	RUFY4	protein_codir	3.4818999	0.00013211	0.00110072
ENSG0000010	NCAPG	protein_codir	3.48140243	2.81E-08	8.42E-07
ENSG0000026	RP11-485G7.5	lncRNA	3.47830282	0.08262237	0.17469976
ENSG0000027	RP11-493L12.	lncRNA	3.47490119	0.01718409	0.05281235
ENSG0000012	KCNS1	protein_codir	3.47301538	3.66E-05	0.00037738

ENSG0000015	CD1A	protein_codir	3.4715899	0.00035888	0.00249991
ENSG0000012	MCHR1	protein_codir	3.47036095	8.06E-06	0.00010732
ENSG0000011	RIPOR2	protein_codir	3.47005099	7.50E-10	3.98E-08
ENSG0000016	AHSP	protein_codir	3.46878086	0.00433576	0.01813625
ENSG0000017	CCL19	protein_codir	3.46600233	1.22E-08	4.16E-07
ENSG0000028	AC104530.1	lncRNA	3.46532669	1.12E-08	3.86E-07
ENSG0000018	SEPTIN1	protein_codir	3.46524105	2.27E-11	2.12E-09
ENSG0000026	CTC-265F19.1	lncRNA	3.46471683	9.97E-05	0.00086622
ENSG0000008	RPH3A	protein_codir	3.46461985	3.72E-06	5.54E-05
ENSG0000016	SLAMF9	protein_codir	3.46367274	1.00E-05	0.00012861
ENSG0000005	TNIP3	protein_codir	3.46228045	1.30E-09	6.32E-08
ENSG0000028	RP11-328C17.	lncRNA	3.46168689	5.27E-08	1.45E-06
ENSG0000025	RP5-991G20.2	lncRNA	3.46125156	0.00209636	0.01018494
ENSG0000013	FCHO1	protein_codir	3.46095314	3.02E-10	1.86E-08
ENSG0000024	LINC01206	lncRNA	3.4594968	0.00722411	0.02697079
ENSG0000023	RP11-325F22.	lncRNA	3.45881105	3.03E-10	1.86E-08
ENSG0000014	NXPH2	protein_codir	3.45850676	0.00238264	0.01126472
ENSG0000019	BLM	protein_codir	3.45472082	4.63E-09	1.82E-07
ENSG0000019	CLEC9A	protein_codir	3.45431336	7.34E-08	1.94E-06
ENSG0000014	PTPN7	protein_codir	3.45388059	5.24E-09	2.03E-07
ENSG0000025	CTC-378H22.2	lncRNA	3.45205862	7.42E-07	1.42E-05
ENSG0000016	SGPP2	protein_codir	3.45050206	3.39E-06	5.13E-05
ENSG0000012	RFPL2	protein_codir	3.44919916	0.02156884	0.06284087
ENSG0000016	BMP10	protein_codir	3.44904033	0.09260636	0.19038241
ENSG0000010	NKX3-2	protein_codir	3.44839947	8.19E-07	1.55E-05
ENSG0000011	CD160	protein_codir	3.44820749	2.86E-05	0.00030875
ENSG0000028	RP11-433N2.1	lncRNA	3.44169635	0.01075823	0.0369121
ENSG0000026	RP11-649A18	lncRNA	3.44068789	0.01177799	0.03956077
ENSG0000016	TMC8	protein_codir	3.43866307	1.60E-09	7.45E-08
ENSG0000010	APOBEC3H	protein_codir	3.43847121	9.06E-09	3.23E-07
ENSG0000015	BUB1B	protein_codir	3.43820961	1.09E-07	2.71E-06
ENSG0000023	PRKCQ-AS1	lncRNA	3.4381169	1.33E-09	6.44E-08
ENSG0000023	RP5-1011O1.3	lncRNA	3.43754241	0.00061621	0.00387867
ENSG0000023	LINC02195	lncRNA	3.43706876	0.06473439	0.1465304
ENSG0000010	ACOD1	protein_codir	3.43577118	0.00720879	0.02692088
ENSG0000002	DEPDC1	protein_codir	3.43402485	1.16E-07	2.84E-06
ENSG0000015	GBP5	protein_codir	3.43338151	4.79E-08	1.33E-06
ENSG0000026	CTD-2561B21	lncRNA	3.43280507	0.00235353	0.01115956
ENSG0000028	RP11-406G20	lncRNA	3.43254697	0.04745113	0.11565481
ENSG0000017	PKHD1	protein_codir	3.430001	0.00416332	0.01759885
ENSG0000024	RP11-730K11.	lncRNA	3.42872725	0.00130982	0.00709913
ENSG0000007	ABCB11	protein_codir	3.42775594	2.34E-05	0.00026202
ENSG0000026	ARMH2	protein_codir	3.42677609	0.0258747	0.07250102
ENSG0000023	LINC02812	lncRNA	3.42548945	0.0002113	0.00161548
ENSG0000015	CDC25C	protein_codir	3.42469568	2.81E-05	0.00030375
ENSG0000027	CCL5	protein_codir	3.42330586	8.60E-12	9.31E-10
ENSG0000016	NPPC	protein_codir	3.42292646	7.86E-05	0.00071303

ENSG0000020	KB-1440D3.16	lncRNA	3.42167911	0.00170113	0.0086886
ENSG0000026	TTC39C-AS1	lncRNA	3.41698263	0.0016125	0.00834548
ENSG0000025	RP11-283G6.5	lncRNA	3.41693026	1.93E-05	0.00022258
ENSG0000024	PCDHA11	protein_codir	3.41674341	0.00149874	0.0078781
ENSG0000026	FMNL1-DT	lncRNA	3.41670192	1.55E-09	7.22E-08
ENSG0000015	C16orf74	protein_codir	3.41454614	9.36E-10	4.80E-08
ENSG0000010	ASF1B	protein_codir	3.4132189	1.34E-09	6.46E-08
ENSG0000028	LINC02452	lncRNA	3.41227727	0.00511875	0.02064542
ENSG0000018	ADRB3	protein_codir	3.41104615	0.00120713	0.00664294
ENSG0000015	CCNB2	protein_codir	3.41049648	8.72E-08	2.24E-06
ENSG0000026	RP11-53I6.2	lncRNA	3.40987175	0.00487331	0.01988012
ENSG0000028	RP11-80K21.4	lncRNA	3.4082131	0.00217637	0.01049064
ENSG0000019	OR2C3	protein_codir	3.40808874	0.0007213	0.00440854
ENSG0000018	SAPCD2	protein_codir	3.40802704	2.43E-08	7.43E-07
ENSG0000008	NME8	protein_codir	3.40749241	7.78E-07	1.48E-05
ENSG0000028	GS1-57L11.3	lncRNA	3.4050921	0.00482327	0.01973609
ENSG0000028	RP11-16D24.1	lncRNA	3.4018137	0.08472729	0.17810852
ENSG0000009	LYZ	protein_codir	3.40154856	1.67E-08	5.41E-07
ENSG0000022	RP11-109A6.3	lncRNA	3.4006727	0.00178541	0.00900683
ENSG0000010	IL2RB	protein_codir	3.39812741	8.07E-11	5.95E-09
ENSG0000020	PLPP4	protein_codir	3.39703584	5.38E-05	0.00052281
ENSG0000016	CXCL10	protein_codir	3.39591437	7.23E-08	1.91E-06
ENSG0000017	ZC3H12D	protein_codir	3.39234334	1.96E-08	6.16E-07
ENSG0000009	RGS1	protein_codir	3.39229218	1.03E-10	7.32E-09
ENSG0000016	LEXM	protein_codir	3.38973334	0.00430557	0.0180455
ENSG0000027	RP13-714J12.	lncRNA	3.38964406	0.01549778	0.04888634
ENSG0000013	KREMEN2	protein_codir	3.38923862	0.02208737	0.06402746
ENSG0000018	TNFRSF4	protein_codir	3.38799156	1.28E-09	6.26E-08
ENSG0000024	RP11-80H8.4	lncRNA	3.38731366	0.00201819	0.00989569
ENSG0000025	AC226119.5	lncRNA	3.38610616	0.05301793	0.12569574
ENSG0000027	PPIAL4H	protein_codir	3.38203978	0.02663262	0.0741213
ENSG0000012	SYT5	protein_codir	3.38131845	0.00136073	0.00730341
ENSG0000028	RP5-1091N2.1	protein_codir	3.38116511	0.00012848	0.00107662
ENSG0000016	BTNL3	protein_codir	3.3795822	0.00941689	0.03321148
ENSG0000022	RFPL4AL1	protein_codir	3.3792858	0.13522018	0.25222505
ENSG0000028	RP11-422P24.	lncRNA	3.37921104	0.00543764	0.02166586
ENSG0000012	OR7C1	protein_codir	3.37799103	0.00340115	0.01492527
ENSG0000027	RP11-861L17.	lncRNA	3.37671238	0.02860265	0.07853714
ENSG0000022	LCT-AS1	lncRNA	3.37623805	0.02209661	0.06404076
ENSG0000014	RAB19	protein_codir	3.37620216	4.38E-05	0.00043675
ENSG0000028	RP11-174M15	lncRNA	3.37450596	0.00809438	0.02950207
ENSG0000027	BX255923.3	lncRNA	3.37405578	0.00402536	0.01711258
ENSG0000016	ANKRD55	protein_codir	3.3710299	0.00015957	0.00129023
ENSG0000015	MAT1A	protein_codir	3.37055473	3.22E-05	0.00033879
ENSG0000025	LINC02446	lncRNA	3.3689585	2.40E-06	3.84E-05
ENSG0000017	HLA-DQB1	protein_codir	3.36895061	1.14E-09	5.68E-08
ENSG0000020	FAM201A	lncRNA	3.36643533	0.00075794	0.00458683

ENSG0000018	OR56B1	protein_codir	3.36558361	0.00431523	0.01806955
ENSG0000015	GPR78	protein_codir	3.36515544	0.00355485	0.01547439
ENSG0000015	NLRP14	protein_codir	3.36465474	4.72E-05	0.00046653
ENSG0000013	DUSP5	protein_codir	3.36311607	2.02E-11	1.91E-09
ENSG0000026	SCAT1	lncRNA	3.35902482	0.00030598	0.00218606
ENSG0000016	XIRP1	protein_codir	3.35778146	NA	NA
ENSG0000011	CUX2	protein_codir	3.3553523	5.93E-06	8.24E-05
ENSG0000006	PRKCQ	protein_codir	3.35517475	8.47E-11	6.16E-09
ENSG0000011	KLRB1	protein_codir	3.35382564	5.91E-14	1.39E-11
ENSG0000023	LINC01502	lncRNA	3.35363083	0.05219405	0.12419019
ENSG0000010	MXRA5	protein_codir	3.3507305	1.47E-09	6.93E-08
ENSG0000026	CSAG2	protein_codir	3.35029535	0.08954284	0.18552046
ENSG0000013	KLHDC7B	protein_codir	3.34873966	6.16E-09	2.34E-07
ENSG0000018	SLC36A2	protein_codir	3.34842045	0.02389901	0.06810136
ENSG0000010	PBX4	protein_codir	3.34805	6.86E-09	2.56E-07
ENSG0000028	RP11-158H18	lncRNA	3.34752109	0.02331164	0.06676353
ENSG0000011	CDC20	protein_codir	3.34636356	1.66E-07	3.87E-06
ENSG0000018	NTM	protein_codir	3.34620059	2.76E-09	1.18E-07
ENSG0000028	RP11-643E20.	lncRNA	3.34583822	0.00932543	0.03297932
ENSG0000000	CD38	protein_codir	3.34549326	4.95E-11	3.98E-09
ENSG0000011	IL23A	protein_codir	3.34087524	1.13E-06	2.05E-05
ENSG0000018	KCNK12	protein_codir	3.34040827	0.00011674	0.00099057
ENSG0000023	CTD-2020K17	lncRNA	3.34028341	3.57E-06	5.35E-05
ENSG0000005	RASGRF1	protein_codir	3.33561961	3.46E-09	1.43E-07
ENSG0000015	CAMK4	protein_codir	3.3337734	2.61E-11	2.33E-09
ENSG0000013	ANGPTL6	protein_codir	3.33325215	6.01E-08	1.63E-06
ENSG0000019	UGT2B15	protein_codir	3.33312826	0.02664061	0.07412991
ENSG0000023	AC007750.5	lncRNA	3.33187466	0.00960735	0.03371729
ENSG0000014	LGSN	protein_codir	3.33140954	0.02116883	0.06193021
ENSG0000015	GHRL	protein_codir	3.33116492	1.11E-07	2.75E-06
ENSG0000012	RAC2	protein_codir	3.33112071	6.34E-11	4.84E-09
ENSG0000011	MYCL	protein_codir	3.33046278	2.81E-06	4.36E-05
ENSG0000017	GP5	protein_codir	3.32864891	8.17E-11	5.97E-09
ENSG0000028	RP11-116G8.8	lncRNA	3.32810783	0.0076314	0.02814894
ENSG0000011	KIF20A	protein_codir	3.32776442	2.34E-06	3.75E-05
ENSG0000028	RP11-253D19	lncRNA	3.32388459	0.00713322	0.02670003
ENSG0000017	GTSF1	protein_codir	3.32361886	4.44E-06	6.40E-05
ENSG0000017	GPR171	protein_codir	3.32335641	9.45E-10	4.83E-08
ENSG0000013	KNL1	protein_codir	3.32282612	5.06E-08	1.40E-06
ENSG0000017	DPP10	protein_codir	3.32255638	0.05535973	0.12981032
ENSG0000015	LHFPL4	protein_codir	3.32036882	0.02294058	0.06594699
ENSG0000026	RP11-350O14	protein_codir	3.32003687	0.04743743	0.11563161
ENSG0000010	AREG	protein_codir	3.31960693	9.13E-10	4.70E-08
ENSG0000025	LINC02251	lncRNA	3.3195723	0.03470241	0.09098277
ENSG0000023	RP11-459O1.2	lncRNA	3.31948618	0.02031639	0.06001483
ENSG0000021	LINC00243	lncRNA	3.3179068	1.83E-05	0.00021272
ENSG0000028	RP11-196B3.5	lncRNA	3.3175341	0.0010183	0.00581227

ENSG0000023	RP11-363D14	lncRNA	3.31747815	0.00829386	0.030031
ENSG0000027	CEACAM20	protein_codir	3.31723268	0.02482404	0.07020685
ENSG0000027	RP11-627K11.	lncRNA	3.31687673	0.0277693	0.07666047
ENSG0000017	KLHL6	protein_codir	3.31659913	2.68E-10	1.67E-08
ENSG0000025	LINC02324	lncRNA	3.31518524	0.00060241	0.00380818
ENSG0000018	CFAP54	protein_codir	3.31429802	5.99E-09	2.28E-07
ENSG0000011	IRAG2	protein_codir	3.31412765	1.15E-08	3.96E-07
ENSG0000017	HOXB9	protein_codir	3.31241918	4.51E-07	9.16E-06
ENSG0000012	POF1B	protein_codir	3.31232157	2.07E-05	0.00023596
ENSG0000012	RGS13	protein_codir	3.31169317	8.04E-06	0.00010712
ENSG0000014	TM4SF19	protein_codir	3.31143216	0.01252677	0.04141546
ENSG0000015	DUSP2	protein_codir	3.30938243	5.04E-14	1.29E-11
ENSG0000010	GATA3	protein_codir	3.3054878	1.23E-18	6.78E-15
ENSG0000024	AL122127.25	lncRNA	3.30444249	0.0303148	0.08211188
ENSG0000017	CD7	protein_codir	3.30404666	4.58E-11	3.76E-09
ENSG0000018	IKZF1	protein_codir	3.30393083	4.06E-09	1.63E-07
ENSG0000023	RP3-462D8.2	lncRNA	3.30110341	0.05398797	0.12751392
ENSG0000002	POU2F2	protein_codir	3.29565742	2.34E-10	1.49E-08
ENSG0000022	LINC00539	lncRNA	3.29556614	3.60E-05	0.00037271
ENSG0000026	RP11-862L9.3	lncRNA	3.29536243	0.01272345	0.04191076
ENSG0000002	NLRP2	protein_codir	3.29369382	3.45E-06	5.21E-05
ENSG0000027	RP1-261G23.7	lncRNA	3.29291427	0.01519959	0.04818234
ENSG0000010	CLC	protein_codir	3.28914743	0.00104657	0.00593557
ENSG0000025	RP11-654D12	lncRNA	3.28892258	0.01714573	0.05273552
ENSG0000028	RP11-216B13.	lncRNA	3.28375659	4.04E-07	8.35E-06
ENSG0000014	MUC4	protein_codir	3.2836168	0.00778789	0.02861153
ENSG0000016	COL26A1	protein_codir	3.28122173	3.02E-05	0.00032255
ENSG0000023	RP11-354E11.	lncRNA	3.279772	6.02E-05	0.00057402
ENSG0000015	ADAM8	protein_codir	3.27705976	3.25E-10	1.97E-08
ENSG0000026	RP11-143K11.	lncRNA	3.2768813	0.02921082	0.079871
ENSG0000012	CD70	protein_codir	3.2758445	2.23E-09	9.73E-08
ENSG0000013	CHAD	protein_codir	3.27537076	7.03E-06	9.53E-05
ENSG0000027	LINC02391	lncRNA	3.27325031	0.0012241	0.0067227
ENSG0000016	NUAK2	protein_codir	3.27166993	4.50E-12	5.50E-10
ENSG0000016	ELFN2	protein_codir	3.26735683	4.93E-05	0.00048438
ENSG0000028	RP11-28G8.2	lncRNA	3.26707138	0.09924829	0.20066158
ENSG0000018	GPR132	protein_codir	3.26489844	1.26E-11	1.28E-09
ENSG0000015	PSMA8	protein_codir	3.26481205	0.00054558	0.00351501
ENSG0000013	RBP5	protein_codir	3.26001246	3.22E-10	1.96E-08
ENSG0000021	GPR33	protein_codir	3.25957316	0.07404688	0.16146967
ENSG0000025	CTC-248O19.1	lncRNA	3.25826412	0.03077917	0.08301148
ENSG0000028	RP11-820K3.7	lncRNA	3.25806728	0.02969553	0.08087866
ENSG0000025	KB-1615E4.2	lncRNA	3.2579657	0.00975961	0.03412932
ENSG0000020	RP11-89H19.1	lncRNA	3.25752454	0.01629859	0.0507961
ENSG0000026	CTD-2555A7.3	lncRNA	3.25724658	0.00127582	0.00694619
ENSG0000026	NFILZ	protein_codir	3.25613819	0.05540229	0.12989909
ENSG0000020	DENND1C	protein_codir	3.25252436	2.95E-09	1.25E-07

ENSG0000026	CTD-2370N5.3	protein_codir	3.25244079	0.00012145	0.00102643
ENSG0000023	HMGA1P4	lncRNA	3.25050028	0.00037601	0.00260153
ENSG0000013	NR5A1	protein_codir	3.24914972	0.01135881	0.03847564
ENSG0000025	RP11-400N9.1	lncRNA	3.24694236	0.01750286	0.05354182
ENSG0000026	RP11-863P13.	lncRNA	3.24692711	1.10E-08	3.79E-07
ENSG0000016	SNX20	protein_codir	3.24664672	1.04E-08	3.61E-07
ENSG0000023	AC091132.1	lncRNA	3.24495067	0.06119903	0.14007263
ENSG0000022	RP5-1091N2.9	lncRNA	3.24264884	4.79E-06	6.87E-05
ENSG0000018	KCNIP4	protein_codir	3.23861341	0.01469459	0.04695878
ENSG0000020	SLC12A5-AS1	lncRNA	3.23805537	0.00101971	0.00581546
ENSG0000026	CTA-363E6.6	lncRNA	3.23800202	0.0227273	0.06549262
ENSG0000020	IFIT1B	protein_codir	3.23772458	0.01421863	0.04574425
ENSG0000017	GPR160	protein_codir	3.23764923	2.28E-10	1.46E-08
ENSG0000010	NKAIN4	protein_codir	3.23693783	0.04933369	0.11930831
ENSG0000027	LINC02227	lncRNA	3.23424752	0.03515268	0.09184724
ENSG0000018	CDCA2	protein_codir	3.23183629	8.01E-08	2.08E-06
ENSG0000023	LINC00707	lncRNA	3.22958148	0.0017578	0.00890049
ENSG0000026	RP11-214O1.2	lncRNA	3.2295226	1.70E-08	5.49E-07
ENSG0000023	KIFC1	protein_codir	3.22920397	7.88E-09	2.88E-07
ENSG0000023	ECE1-AS1	lncRNA	3.22904077	2.52E-07	5.55E-06
ENSG0000015	TMEM171	protein_codir	3.22605606	0.00018763	0.0014652
ENSG0000022	HECW2-AS1	lncRNA	3.22579587	3.09E-07	6.62E-06
ENSG0000011	CENPF	protein_codir	3.22544335	6.16E-10	3.39E-08
ENSG0000013	BCL2L10	protein_codir	3.22477589	0.00991949	0.03459296
ENSG0000028	CTC-251H24.1	protein_codir	3.2243818	0.05638333	0.13164054
ENSG0000022	RP11-137H2.4	lncRNA	3.22429227	0.00041171	0.00279737
ENSG0000018	TMEM215	protein_codir	3.22399805	0.03649028	0.0945018
ENSG0000018	BNIP5	protein_codir	3.22288704	0.0208509	0.06124009
ENSG0000018	CD300E	protein_codir	3.22177731	1.89E-07	4.33E-06
ENSG0000008	COL19A1	protein_codir	3.22151413	1.77E-07	4.07E-06
ENSG0000028	RP11-231C14.	lncRNA	3.22131819	0.00224251	0.01075502
ENSG0000025	RP11-18H21.3	lncRNA	3.22021993	0.02275828	0.06555942
ENSG0000014	TICRR	protein_codir	3.21912066	1.37E-05	0.00016777
ENSG0000022	CCDC26	lncRNA	3.21755702	0.00105923	0.00599386
ENSG0000023	LINC01705	lncRNA	3.21670546	0.00620019	0.0239476
ENSG0000013	RTBDN	protein_codir	3.21478717	0.01294037	0.04247908
ENSG0000012	MYH2	protein_codir	3.21450921	0.05326481	0.12616191
ENSG0000010	CHRNA4	protein_codir	3.21442156	0.02869107	0.07872515
ENSG0000027	RP11-686O6.1	lncRNA	3.21434941	0.01812787	0.05495464
ENSG0000007	CNGB1	protein_codir	3.21303841	0.00414711	0.01754375
ENSG0000017	THEMIS	protein_codir	3.2128565	8.95E-08	2.30E-06
ENSG0000018	KRT79	protein_codir	3.21081922	0.03222475	0.08589534
ENSG0000027	RP11-92K15.3	lncRNA	3.20928136	0.00503673	0.02039502
ENSG0000014	PMAIP1	protein_codir	3.20681503	1.36E-09	6.51E-08
ENSG0000025	LINC02461	lncRNA	3.20621946	0.0319069	0.08522016
ENSG0000027	CTD-2240J17.	lncRNA	3.20583049	0.02844124	0.07818723
ENSG0000027	RP11-395B7.2	lncRNA	3.20532151	0.05091912	0.1221536

ENSG0000014	OCSTAMP	protein_codir	3.20485514	0.01806643	0.05485269
ENSG0000008	LAG3	protein_codir	3.20414525	5.02E-13	8.40E-11
ENSG0000023	LINC01871	lncRNA	3.20219071	1.08E-05	0.00013677
ENSG0000016	EN1	protein_codir	3.20218693	1.84E-08	5.85E-07
ENSG0000016	GPR183	protein_codir	3.20203915	4.07E-14	1.07E-11
ENSG0000018	KRT14	protein_codir	3.20020538	6.70E-05	0.00062614
ENSG0000017	PTCRA	protein_codir	3.19931804	8.06E-05	0.00072869
ENSG0000013	INHBE	protein_codir	3.19859595	0.00131981	0.00713926
ENSG0000014	CDCA7	protein_codir	3.19812394	6.16E-07	1.21E-05
ENSG0000010	IL2	protein_codir	3.19687849	0.00071548	0.00438175
ENSG0000023	LINC01305	lncRNA	3.1939782	0.00411069	0.01742169
ENSG0000014	GSDMC	protein_codir	3.19301566	0.00398226	0.01695546
ENSG0000025	LINC00824	lncRNA	3.18996206	0.00497129	0.02016954
ENSG0000010	RASAL3	protein_codir	3.18910644	5.99E-10	3.32E-08
ENSG0000009	CDHR5	protein_codir	3.18873407	0.00321762	0.01429196
ENSG0000026	RP11-105C19.	lncRNA	3.18713301	0.32379396	0.47610675
ENSG0000015	NCF1	protein_codir	3.1859751	9.53E-08	2.42E-06
ENSG0000018	EVI2B	protein_codir	3.18555382	5.61E-09	2.16E-07
ENSG0000022	RP3-370M22.	protein_codir	3.18202282	0.00071293	0.00436804
ENSG0000022	LINC01342	lncRNA	3.18132717	0.05639588	0.13164759
ENSG0000022	LINC01788	lncRNA	3.18108281	0.02397252	0.06827398
ENSG0000028	RP11-111G13	lncRNA	3.18103365	0.01028214	0.03558359
ENSG0000006	ASPM	protein_codir	3.17889534	8.50E-07	1.60E-05
ENSG0000018	PKP3	protein_codir	3.17639291	3.01E-05	0.00032181
ENSG0000025	AC022182.1	lncRNA	3.17380945	0.01121657	0.03810129
ENSG0000027	RP11-482H16	lncRNA	3.17267644	0.00026695	0.00195679
ENSG0000013	CD72	protein_codir	3.1726108	2.29E-08	7.06E-07
ENSG0000018	VSTM1	protein_codir	3.17105174	0.00239432	0.01130256
ENSG0000020	LINC02487	lncRNA	3.16913698	0.0682988	0.15228972
ENSG0000012	PKMYT1	protein_codir	3.16893131	6.42E-07	1.25E-05
ENSG0000028	RP11-655C2.4	lncRNA	3.16880941	0.00895632	0.03193153
ENSG0000017	SHCBP1	protein_codir	3.1685653	6.27E-09	2.37E-07
ENSG0000008	PTPRC	protein_codir	3.16632917	6.92E-09	2.57E-07
ENSG0000014	ST18	protein_codir	3.16596523	9.21E-07	1.71E-05
ENSG0000011	TNR	protein_codir	3.16573143	2.65E-06	4.15E-05
ENSG0000010	GRAP2	protein_codir	3.16462791	1.06E-10	7.44E-09
ENSG0000027	RP11-1143G9	lncRNA	3.16378089	0.00181314	0.00910359
ENSG0000027	LINC01749	lncRNA	3.16269234	0.07545223	0.16368456
ENSG0000016	SLC6A20	protein_codir	3.16174285	0.00473136	0.01942913
ENSG0000012	PSD4	protein_codir	3.16090214	3.20E-09	1.34E-07
ENSG0000016	KLK2	protein_codir	3.16020728	0.01955307	0.05827732
ENSG0000025	BBOX1-AS1	lncRNA	3.15794584	0.02344124	0.06705128
ENSG0000006	CLEC2D	protein_codir	3.15735272	4.53E-09	1.79E-07
ENSG0000006	CREB3L3	protein_codir	3.15653467	0.06170549	0.14106156
ENSG0000014	ADAM12	protein_codir	3.15498511	6.95E-06	9.43E-05
ENSG0000023	LINC01506	lncRNA	3.15462023	0.0036157	0.01567255
ENSG0000023	CNIH3-AS2	lncRNA	3.1542099	0.03925539	0.10004926

ENSG0000020	SLFN12L	protein_codir	3.15209485	3.61E-08	1.05E-06
ENSG0000016	TTLL10	protein_codir	3.15188915	0.00044511	0.00297549
ENSG0000024	RP11-423H2.3	lncRNA	3.14957714	1.19E-05	0.00014858
ENSG0000008	TMPRSS11E	protein_codir	3.14909241	0.01468268	0.04692612
ENSG0000014	IRF8	protein_codir	3.14798315	1.42E-08	4.74E-07
ENSG0000016	SVOP	protein_codir	3.14764364	0.08150829	0.17313382
ENSG0000018	FAM111B	protein_codir	3.14688131	2.61E-06	4.10E-05
ENSG0000024	BFSP2-AS1	lncRNA	3.14609248	0.02098545	0.06153731
ENSG0000004	ADAM28	protein_codir	3.1426171	5.06E-09	1.98E-07
ENSG0000016	TMEM266	protein_codir	3.14089135	1.15E-05	0.00014408
ENSG0000025	RP11-404P21.	protein_codir	3.13974798	0.01041245	0.03595347
ENSG0000026	CTD-2006K23	lncRNA	3.13879309	3.73E-07	7.84E-06
ENSG0000027	CH17-140K24	protein_codir	3.13463793	0.10340883	0.20689258
ENSG0000015	RBM46	protein_codir	3.13459934	0.12088297	0.23239282
ENSG0000016	SERPINB7	protein_codir	3.13208412	0.00841059	0.03034227
ENSG0000016	CKAP2L	protein_codir	3.13100734	2.52E-07	5.55E-06
ENSG0000027	RP11-484K9.4	lncRNA	3.1307938	0.00788116	0.02887002
ENSG0000002	DEF6	protein_codir	3.12604426	3.69E-10	2.19E-08
ENSG0000018	GRAPL	protein_codir	3.12247579	7.96E-06	0.00010635
ENSG0000018	LRRC55	protein_codir	3.12235353	0.00013192	0.00109981
ENSG0000023	RP5-943J3.1	lncRNA	3.12055139	0.03508586	0.09170733
ENSG0000009	CDC45	protein_codir	3.1203911	5.27E-06	7.45E-05
ENSG0000027	RP5-984P4.6	lncRNA	3.12016575	0.18645603	0.31981093
ENSG0000017	INSM1	protein_codir	3.11840225	0.00039744	0.00271984
ENSG0000025	GH1	protein_codir	3.11681923	0.01655359	0.05137194
ENSG0000018	FCAR	protein_codir	3.11624293	4.71E-08	1.32E-06
ENSG0000023	LINC02541	lncRNA	3.11389517	0.00010787	0.00092646
ENSG0000024	RP11-796E2.4	lncRNA	3.11350027	8.72E-09	3.12E-07
ENSG0000023	AF127936.3	lncRNA	3.11311327	2.37E-06	3.79E-05
ENSG0000018	GPR141	protein_codir	3.11231862	1.36E-09	6.51E-08
ENSG0000015	RGL4	protein_codir	3.11079912	9.79E-09	3.45E-07
ENSG0000024	RP11-495K9.6	lncRNA	3.11008072	0.0103921	0.03590118
ENSG0000013	LIMD2	protein_codir	3.10953336	5.41E-10	3.02E-08
ENSG0000023	RP1-151B14.6	lncRNA	3.10921532	0.04049479	0.10246116
ENSG0000009	GABRP	protein_codir	3.10895846	6.18E-05	0.00058673
ENSG0000025	RP11-1029J19	lncRNA	3.10823435	0.03848192	0.09842307
ENSG0000012	NPBWR2	protein_codir	3.10800649	0.02122165	0.06203217
ENSG0000028	RP11-348J12.	lncRNA	3.10708994	0.01326584	0.04330656
ENSG0000027	RP11-298P3.4	lncRNA	3.10453246	0.04584753	0.11266093
ENSG0000018	MIXL1	protein_codir	3.09877149	9.54E-05	0.00083548
ENSG0000022	AC093818.1	lncRNA	3.09860541	5.92E-10	3.28E-08
ENSG0000023	LINC01238	lncRNA	3.09720932	1.66E-07	3.87E-06
ENSG0000026	RP11-203B7.2	lncRNA	3.09637142	0.01349999	0.04391869
ENSG0000013	MYO1G	protein_codir	3.09629793	1.20E-10	8.22E-09
ENSG0000028	PTCSC1	lncRNA	3.09552115	0.01328106	0.04334431
ENSG0000016	GREM1	protein_codir	3.09485565	2.89E-06	4.46E-05
ENSG0000024	PCED1B-AS1	lncRNA	3.09448467	2.40E-10	1.52E-08

ENSG0000028	RP1-46C2.1	lncRNA	3.0941946	0.00900964	0.03209679
ENSG0000027	CTC-523E23.1	lncRNA	3.09191932	1.88E-08	5.96E-07
ENSG0000010	PPP1R16B	protein_codir	3.0910535	3.27E-10	1.98E-08
ENSG0000019	PLCG2	protein_codir	3.09103111	7.48E-12	8.27E-10
ENSG0000012	BTN1A1	protein_codir	3.08940726	0.00088094	0.00517696
ENSG0000028	RP11-209B12.	lncRNA	3.0884207	0.02710431	0.0751395
ENSG0000024	KB-1471A8.1	lncRNA	3.08719414	0.00056714	0.00362
ENSG0000013	BICDL1	protein_codir	3.08577	8.96E-06	0.0001173
ENSG0000019	MYT1	protein_codir	3.08513424	0.00159887	0.00828998
ENSG0000013	IL1RN	protein_codir	3.0849131	1.08E-07	2.69E-06
ENSG0000018	C12orf56	protein_codir	3.08278666	0.02219757	0.06425919
ENSG0000016	KASH5	protein_codir	3.08055503	0.00993503	0.0346384
ENSG0000017	KDF1	protein_codir	3.08005358	0.00029443	0.0021211
ENSG0000018	CLECL1	protein_codir	3.07566486	7.06E-06	9.56E-05
ENSG0000028	RP11-496H1.4	lncRNA	3.0754634	0.00747552	0.02772206
ENSG0000018	GPR19	protein_codir	3.07536283	1.15E-05	0.00014419
ENSG0000026	AC005307.1	lncRNA	3.07401851	0.08973311	0.18581709
ENSG0000012	CFP	protein_codir	3.07383353	4.21E-09	1.68E-07
ENSG0000012	CCR2	protein_codir	3.0727318	1.69E-06	2.86E-05
ENSG0000013	SEMA7A	protein_codir	3.07195463	9.57E-12	1.02E-09
ENSG0000010	TRPA1	protein_codir	3.06869009	1.27E-06	2.25E-05
ENSG0000022	HBD	protein_codir	3.06754304	0.0005519	0.00354336
ENSG0000022	TP73-AS3	lncRNA	3.06615799	0.08673236	0.18135724
ENSG0000025	RP11-624L4.1	lncRNA	3.06583923	4.42E-05	0.00043996
ENSG0000016	TP53INP1	protein_codir	3.06519867	2.34E-11	2.15E-09
ENSG0000014	MS4A3	protein_codir	3.06468452	0.02458269	0.06968359
ENSG0000028	SLFN12L	lncRNA	3.06460478	7.86E-08	2.05E-06
ENSG0000027	H2AC16	protein_codir	3.0644939	0.00127607	0.00694619
ENSG0000022	CFLAR-AS1	lncRNA	3.06330799	0.00509756	0.020587
ENSG0000025	RP11-318K15.	lncRNA	3.06324468	0.0030961	0.01385295
ENSG0000023	LINC01679	lncRNA	3.06316314	2.60E-11	2.33E-09
ENSG0000011	KMO	protein_codir	3.06291347	1.43E-07	3.41E-06
ENSG0000014	PLA1A	protein_codir	3.06287533	9.71E-11	6.93E-09
ENSG0000028	LINC01127	lncRNA	3.06143936	0.00015865	0.0012831
ENSG0000014	SIK1	protein_codir	3.06113722	0.00044449	0.00297403
ENSG0000025	RP11-834C11.	lncRNA	3.05923748	0.01029897	0.03563291
ENSG0000011	CD83	protein_codir	3.05446296	3.93E-10	2.31E-08
ENSG0000017	PHOSPHO1	protein_codir	3.05251527	3.81E-06	5.65E-05
ENSG0000017	CHST2	protein_codir	3.05059483	1.50E-09	7.05E-08
ENSG0000000	CEACAM21	protein_codir	3.0502074	1.10E-08	3.79E-07
ENSG0000028	RP1-97D16.9	lncRNA	3.04997607	1.14E-05	0.00014229
ENSG0000015	C11orf53	protein_codir	3.04897867	0.03210291	0.0856119
ENSG0000022	LINC01918	lncRNA	3.04759987	0.03801353	0.09748261
ENSG0000024	INSL3	protein_codir	3.04712811	1.83E-06	3.07E-05
ENSG0000016	EPB42	protein_codir	3.04678561	0.00352466	0.01536235
ENSG0000021	LINC01644	lncRNA	3.04676314	0.03504667	0.09163983
ENSG0000025	CTC-378H22.1	lncRNA	3.04544141	6.48E-06	8.87E-05

ENSG0000022	LINC02840	lncRNA	3.04490138	0.11419509	0.22261916
ENSG0000028	LL22NC03-N9	lncRNA	3.04484745	0.00969416	0.03396722
ENSG0000007	ICAM3	protein_codir	3.04427173	2.33E-10	1.49E-08
ENSG0000010	TUBB1	protein_codir	3.04381843	1.59E-05	0.00018942
ENSG0000013	ESPL1	protein_codir	3.04194213	2.31E-08	7.10E-07
ENSG0000022	EML4-AS1	lncRNA	3.04042383	0.00064957	0.00404742
ENSG0000011	OTX1	protein_codir	3.03990108	0.00224572	0.0107648
ENSG0000028	LINC01012	lncRNA	3.0387312	4.24E-05	0.00042506
ENSG0000024	LINC02265	lncRNA	3.03833505	0.01953342	0.05823685
ENSG0000017	CIITA	protein_codir	3.03536637	1.97E-10	1.29E-08
ENSG0000028	CTD-3094K11	lncRNA	3.03395247	0.07088959	0.15651244
ENSG0000026	RNF157-AS1	lncRNA	3.0338205	0.02002989	0.05937172
ENSG0000008	FCN1	protein_codir	3.03341948	3.68E-07	7.73E-06
ENSG0000028	RP3-369A17.6	protein_codir	3.03226798	0.19429661	0.32979796
ENSG0000027	RP11-1151B14	lncRNA	3.02909408	0.0826426	0.17469976
ENSG0000018	IFNLR1	protein_codir	3.0245888	9.33E-07	1.73E-05
ENSG0000021	MAGEA12	protein_codir	3.02345125	0.11942827	0.23010874
ENSG0000025	RP11-598F7.6	lncRNA	3.02302959	9.70E-05	0.00084645
ENSG0000024	RP11-1149O2	lncRNA	3.02285651	1.25E-09	6.12E-08
ENSG0000027	RP11-138A9.2	lncRNA	3.02232243	0.06395331	0.14519073
ENSG0000024	LRRD1	protein_codir	3.02215508	0.00782104	0.02869134
ENSG0000017	IL16	protein_codir	3.02108721	1.42E-10	9.60E-09
ENSG0000015	CD1B	protein_codir	3.01919814	0.00062874	0.00393958
ENSG0000025	HCAR3	protein_codir	3.01891805	1.69E-07	3.92E-06
ENSG0000012	TASL	protein_codir	3.01845813	1.67E-07	3.88E-06
ENSG0000017	CLEC12A	protein_codir	3.01814696	1.78E-09	8.09E-08
ENSG0000012	NCAPH	protein_codir	3.01773848	1.57E-08	5.15E-07
ENSG0000017	CDK5R1	protein_codir	3.01727118	9.73E-09	3.44E-07
ENSG0000026	AJ003147.9	lncRNA	3.01590718	0.18570647	0.3190033
ENSG0000016	MPZL3	protein_codir	3.01479512	3.29E-10	1.98E-08
ENSG0000024	RP11-430C7.5	lncRNA	3.01428928	3.68E-06	5.49E-05
ENSG0000015	DRC7	protein_codir	3.01405308	0.01084555	0.03714709
ENSG0000024	RP11-498P14.	lncRNA	3.01181101	0.27043236	0.41828648
ENSG0000022	LINC01320	lncRNA	3.01059557	0.0683049	0.15229102
ENSG0000009	MMP11	protein_codir	3.00813625	5.06E-10	2.86E-08
ENSG0000007	GTSE1	protein_codir	3.00751155	9.03E-08	2.31E-06
ENSG0000017	ESCO2	protein_codir	3.00640807	7.27E-07	1.39E-05
ENSG0000020	OLIG2	protein_codir	3.00590827	0.00445507	0.01853148
ENSG0000016	SAMD3	protein_codir	3.00583254	9.95E-08	2.50E-06
ENSG0000010	PROKR2	protein_codir	3.00512473	0.01403795	0.04526363
ENSG0000025	RP11-588H23	lncRNA	3.00487363	1.89E-06	3.15E-05
ENSG0000012	TREM1	protein_codir	3.00482195	1.68E-07	3.90E-06
ENSG0000017	LGALS9B	protein_codir	3.00415858	0.00477409	0.01956677
ENSG0000023	AP000251.3	lncRNA	3.00338083	0.19782201	0.33401663
ENSG0000015	CD8A	protein_codir	3.00227779	1.79E-08	5.75E-07
ENSG0000019	SPN	protein_codir	3.00189283	2.08E-08	6.48E-07
ENSG0000028	RP11-27J8.4	lncRNA	3.00091163	0.00139349	0.00743588

ENSG0000019	ARMH1	protein_codir	2.99998587	2.72E-07	5.93E-06
ENSG0000017	OLR1	protein_codir	2.99974199	1.26E-05	0.00015615
ENSG0000005	POLQ	protein_codir	2.99761347	1.55E-06	2.68E-05
ENSG0000027	RP11-158G18	lncRNA	2.99494238	0.02788773	0.07693359
ENSG0000023	RP1-212P9.3	lncRNA	2.99300032	0.00356461	0.01551197
ENSG0000013	PAX3	protein_codir	2.99044405	0.19298488	0.32809567
ENSG0000019	DNAH10	protein_codir	2.99005746	0.00013134	0.00109532
ENSG0000022	XX-C283C717.	lncRNA	2.98950743	0.12648135	0.24031196
ENSG0000012	EGR2	protein_codir	2.98925368	8.08E-11	5.95E-09
ENSG0000016	RAB26	protein_codir	2.98790777	7.38E-06	9.96E-05
ENSG0000023	BCL2L1-AS1	lncRNA	2.9876313	0.08915582	0.18495446
ENSG0000015	CD226	protein_codir	2.98743539	7.82E-12	8.60E-10
ENSG0000026	AKR1C8P	protein_codir	2.98719136	0.02271843	0.06548559
ENSG0000024	RP11-90P5.5	lncRNA	2.9856064	0.0225974	0.06523857
ENSG0000023	LINC00892	lncRNA	2.984712	2.72E-05	0.00029595
ENSG0000028	RP11-404H1.1	lncRNA	2.98377326	0.02403007	0.06840966
ENSG0000028	RP11-597K23.	lncRNA	2.98357903	0.02497342	0.07053541
ENSG0000011	DAO	protein_codir	2.9812063	0.0018619	0.00928261
ENSG0000026	RP11-160E2.1	lncRNA	2.9805857	0.00041345	0.0028045
ENSG0000024	CSTF3-DT	lncRNA	2.9787945	0.00357133	0.01553386
ENSG0000007	CST7	protein_codir	2.97834434	8.87E-11	6.38E-09
ENSG0000010	CHRNE	protein_codir	2.97776183	3.34E-10	2.01E-08
ENSG0000016	ADAM29	protein_codir	2.97756805	0.01416653	0.0456356
ENSG0000023	CEP250-AS1	lncRNA	2.97703899	4.83E-06	6.92E-05
ENSG0000013	EMX1	protein_codir	2.97686774	0.0426937	0.10679225
ENSG0000028	RP11-413G22	lncRNA	2.97624214	0.0034175	0.01498515
ENSG0000027	H3C11	protein_codir	2.97585309	0.01304356	0.04274091
ENSG0000027	ENSG0000027	protein_codir	2.97579557	0.08511729	0.17875444
ENSG0000026	RUNDC3A-AS	lncRNA	2.97574465	0.00108529	0.0061086
ENSG0000010	PAPLN	protein_codir	2.97477217	5.16E-11	4.11E-09
ENSG0000010	HOXA13	protein_codir	2.97412523	0.00041618	0.00281807
ENSG0000014	NPFFR1	protein_codir	2.97352136	4.82E-05	0.00047545
ENSG0000012	HOXD13	protein_codir	2.97320671	0.18827924	0.32214052
ENSG0000017	CD300LB	protein_codir	2.97178539	2.17E-09	9.51E-08
ENSG0000018	ANKRD18A	protein_codir	2.96752944	0.00068356	0.00422182
ENSG0000022	C5orf67	lncRNA	2.96210547	0.13717528	0.25489647
ENSG0000028	RP11-506H21	lncRNA	2.96142573	0.01189569	0.0398205
ENSG0000025	RP11-77K12.8	lncRNA	2.96043802	0.28760174	0.4372281
ENSG0000028	CTD-2325P2.5	lncRNA	2.95988127	0.00273547	0.01254418
ENSG0000019	HLA-DQA1	protein_codir	2.95984114	1.76E-09	8.00E-08
ENSG0000025	LYPD8	protein_codir	2.95938446	0.07548768	0.16373251
ENSG0000022	AP001434.2	lncRNA	2.95742074	0.00166684	0.00855727
ENSG0000023	DNAH8-AS1	lncRNA	2.95525227	0.08241794	0.17440879
ENSG0000026	TMEM178B	protein_codir	2.95434122	4.81E-10	2.74E-08
ENSG0000016	CCR5	protein_codir	2.95319583	3.01E-08	8.94E-07
ENSG0000024	RP11-260E18.	lncRNA	2.9523168	0.03404122	0.08970526
ENSG0000013	TMPRSS13	protein_codir	2.95229897	6.46E-07	1.26E-05

ENSG0000025	CTD-2116N17	protein_codir	2.95131501	1.45E-05	0.0001753
ENSG0000010	UPK3A	protein_codir	2.95005531	1.07E-05	0.00013539
ENSG0000017	FAM153A	protein_codir	2.94976803	2.09E-05	0.00023806
ENSG0000017	GRM8	protein_codir	2.9492437	0.00019654	0.00152271
ENSG0000024	RP11-377G16	lncRNA	2.9488774	0.02110911	0.06181443
ENSG0000028	RPS4Y2	protein_codir	2.94813704	0.0839643	0.17685457
ENSG0000000	MAP3K9	protein_codir	2.94781772	1.34E-07	3.23E-06
ENSG0000026	RP11-534L20.	lncRNA	2.94620261	0.02889124	0.07917857
ENSG0000022	LINC00237	lncRNA	2.94521255	0.09050449	0.18698994
ENSG0000012	CBFA2T3	protein_codir	2.94483523	5.35E-12	6.18E-10
ENSG0000019	TOX	protein_codir	2.9431506	1.11E-10	7.71E-09
ENSG0000013	DSG3	protein_codir	2.94297391	0.07199065	0.15826025
ENSG0000018	FFAR3	protein_codir	2.94240104	3.55E-08	1.03E-06
ENSG0000008	KCNK2	protein_codir	2.93880284	5.69E-09	2.19E-07
ENSG0000013	DYDC2	protein_codir	2.93869197	0.08462101	0.17793927
ENSG0000012	WDFY4	protein_codir	2.93860385	1.44E-07	3.43E-06
ENSG0000028	RP11-575F12.	lncRNA	2.93762404	0.27737021	0.42579842
ENSG0000025	AC034243.1	lncRNA	2.93427552	0.03767126	0.09681338
ENSG0000024	AC108004.3	lncRNA	2.93360265	0.02715526	0.07525115
ENSG0000025	RP11-736N17	lncRNA	2.9333591	0.04765013	0.11599654
ENSG0000026	CORO1A-AS1	lncRNA	2.93282127	6.62E-05	0.00062062
ENSG0000012	BPIFB1	protein_codir	2.93168034	0.07802266	0.16754672
ENSG0000019	PRTN3	protein_codir	2.9290818	0.07060287	0.15605404
ENSG0000022	LINC01678	lncRNA	2.9253299	6.67E-08	1.78E-06
ENSG0000013	RNF128	protein_codir	2.92532167	0.00049985	0.00326921
ENSG0000014	TLCD3B	protein_codir	2.92457977	0.00481223	0.01969385
ENSG0000010	SMPD3	protein_codir	2.92420924	4.23E-06	6.15E-05
ENSG0000021	KLRK1	protein_codir	2.92252703	1.05E-08	3.64E-07
ENSG0000028	RP11-209I11.	lncRNA	2.92229436	0.04498152	0.11117581
ENSG0000028	AC007326.13	protein_codir	2.92108688	0.03986656	0.10125189
ENSG0000022	RP11-265P11.	lncRNA	2.92022343	0.08263992	0.17469976
ENSG0000022	NRIR	lncRNA	2.92006139	0.00251667	0.01173962
ENSG0000011	LCT	protein_codir	2.91969266	0.02592122	0.0726036
ENSG0000017	TMEM154	protein_codir	2.91856911	4.72E-08	1.32E-06
ENSG0000017	CD8B	protein_codir	2.91791661	3.03E-08	8.98E-07
ENSG0000016	CXCL1	protein_codir	2.91658982	4.46E-10	2.56E-08
ENSG0000028	CTC-338M12.	lncRNA	2.9158996	0.02606588	0.07293483
ENSG0000018	CALHM6	protein_codir	2.91562014	2.14E-07	4.80E-06
ENSG0000018	CYP4F3	protein_codir	2.91542343	0.00037196	0.00257609
ENSG0000012	PIWIL1	protein_codir	2.91520341	0.13476213	0.25175043
ENSG0000016	GNG4	protein_codir	2.9149287	6.34E-06	8.72E-05
ENSG0000027	RP11-83N9.6	lncRNA	2.91481507	0.00367787	0.01587479
ENSG0000023	MAP3K20-AS1	lncRNA	2.91474585	0.13361664	0.25025372
ENSG0000010	ASIP	protein_codir	2.91386728	0.00194552	0.00960788
ENSG0000022	C12orf77	lncRNA	2.9137334	0.00546041	0.02173776
ENSG0000016	TAFA4	protein_codir	2.91347708	0.02893551	0.07926199
ENSG0000016	GFI1	protein_codir	2.91329766	5.01E-07	1.01E-05

ENSG0000013	TNS4	protein_codir	2.91211733	7.42E-05	0.00068042
ENSG0000024	DRAIC	lncRNA	2.91200957	0.00083735	0.00496834
ENSG0000028	RP11-507F16.	lncRNA	2.91154896	0.07189593	0.15812754
ENSG0000028	RP11-321P16.	lncRNA	2.90917686	0.00901875	0.03211802
ENSG0000022	FAM197Y7	lncRNA	2.90903448	0.07885966	0.16887937
ENSG0000016	GSDMA	protein_codir	2.90903195	4.25E-05	0.00042615
ENSG0000011	GPRC5D	protein_codir	2.90899797	0.00079976	0.00478749
ENSG0000023	RP11-104L21.	lncRNA	2.90858155	0.13044579	0.24577463
ENSG0000022	AJ006998.2	lncRNA	2.90853103	0.00147232	0.00777285
ENSG0000025	LINC02390	lncRNA	2.90824077	0.01254021	0.04144558
ENSG0000017	FLJ40194	lncRNA	2.90742282	0.00246857	0.01157195
ENSG0000013	SYT16	protein_codir	2.90561929	0.00494993	0.02010789
ENSG0000017	NLRP11	protein_codir	2.90535168	0.0035294	0.01537628
ENSG0000023	TLR8-AS1	lncRNA	2.90494621	0.02527655	0.0712168
ENSG0000019	HLA-DRB5	protein_codir	2.90494431	2.48E-10	1.56E-08
ENSG0000019	GRIN3A	protein_codir	2.90459072	5.36E-10	3.00E-08
ENSG0000024	RP11-637A17	lncRNA	2.90241714	3.73E-05	0.00038328
ENSG0000027	RP11-48G14.3	lncRNA	2.90232395	0.00645121	0.02474378
ENSG0000007	IPCEF1	protein_codir	2.90161336	8.60E-11	6.23E-09
ENSG0000026	RP5-1171I10.5	lncRNA	2.90058151	9.64E-07	1.78E-05
ENSG0000026	CTC-510F12.4	lncRNA	2.90021397	0.01295246	0.04250289
ENSG0000028	RP11-527D7.1	lncRNA	2.90006184	0.05083026	0.12203857
ENSG0000017	MYO1H	protein_codir	2.89806709	0.02895658	0.07931185
ENSG0000022	RP11-452K12.	lncRNA	2.89647518	0.00327358	0.0144921
ENSG0000006	CHI3L2	protein_codir	2.89445297	1.46E-09	6.91E-08
ENSG0000017	ZNF296	protein_codir	2.89433642	4.30E-08	1.22E-06
ENSG0000025	RP11-266O8.1	lncRNA	2.89390528	0.0078232	0.02869546
ENSG0000018	FANCA	protein_codir	2.89371039	1.84E-06	3.07E-05
ENSG0000025	RP11-567C2.1	lncRNA	2.89368989	0.05332944	0.12630416
ENSG0000025	RP11-44F14.5	lncRNA	2.89268423	0.07223583	0.15866033
ENSG0000007	DHRS9	protein_codir	2.89260894	1.14E-06	2.07E-05
ENSG0000012	SFTPA1	protein_codir	2.89110941	0.0727248	0.15944268
ENSG0000007	TXK	protein_codir	2.89051106	7.20E-08	1.91E-06
ENSG0000010	HMOX1	protein_codir	2.88999893	1.74E-07	4.02E-06
ENSG0000028	RP11-511P7.6	lncRNA	2.88980009	0.30076983	0.45185003
ENSG0000017	CPN2	protein_codir	2.88963847	0.00345704	0.01512411
ENSG0000023	LINC01036	lncRNA	2.8892588	0.01467414	0.04690968
ENSG0000015	MEGF11	protein_codir	2.88834965	0.00058251	0.00370306
ENSG0000027	RP11-318E3.9	lncRNA	2.88795274	0.02134541	0.06234115
ENSG0000017	LINC02880	lncRNA	2.88732825	0.0166948	0.05170558
ENSG0000026	RP1-45N11.1	lncRNA	2.88636717	0.01814536	0.05500161
ENSG0000016	ROR2	protein_codir	2.88391205	3.45E-08	1.01E-06
ENSG0000022	MIR663AHG	lncRNA	2.88372629	0.09081144	0.18745978
ENSG0000016	PF4	protein_codir	2.88345351	1.02E-05	0.00012986
ENSG0000022	LINC01876	lncRNA	2.88342933	0.01112281	0.03785262
ENSG0000027	RP4-583P15.1	protein_codir	2.88156261	5.09E-07	1.02E-05
ENSG0000017	CXCR6	protein_codir	2.88099003	7.63E-06	0.00010238

ENSG0000026	RP11-81K2.1	lncRNA	2.88063896	0.05886555	0.1359147
ENSG0000018	SEMA4D	protein_codir	2.88037109	5.05E-10	2.86E-08
ENSG0000017	RP11-16K12.1	lncRNA	2.88035845	0.00635768	0.02446011
ENSG0000016	CPLX4	protein_codir	2.87817086	0.05120031	0.1225316
ENSG0000027	SIK1B	protein_codir	2.8780652	2.56E-10	1.60E-08
ENSG0000017	ODAPH	protein_codir	2.87621577	0.01531723	0.04845514
ENSG0000012	DPPA4	protein_codir	2.87536669	0.00080788	0.00482875
ENSG0000019	SLC28A3	protein_codir	2.87402894	0.00018204	0.00143158
ENSG0000026	C19orf84	protein_codir	2.87397068	0.00187126	0.00931917
ENSG0000028	RP11-445P17.	lncRNA	2.87352601	0.13944767	0.25822671
ENSG0000011	NKX2-3	protein_codir	2.86884636	0.00696885	0.02625557
ENSG0000011	FOXM1	protein_codir	2.86719487	4.74E-08	1.33E-06
ENSG0000022	LINC02528	lncRNA	2.86706293	0.03286959	0.08727726
ENSG0000011	TNFAIP3	protein_codir	2.86648598	3.07E-12	3.96E-10
ENSG0000027	RP1-244F24.1	lncRNA	2.86587783	0.00072041	0.00440408
ENSG0000028	RP11-822I2.1	lncRNA	2.86536884	0.05409857	0.12769871
ENSG0000011	GRIN3B	protein_codir	2.86529434	0.0002791	0.00203237
ENSG0000028	RP11-799O21	lncRNA	2.86364936	0.15496466	0.27903539
ENSG0000027	RP11-201K10.	protein_codir	2.8634159	0.37597738	0.52949701
ENSG0000011	PASK	protein_codir	2.86252834	1.05E-08	3.64E-07
ENSG0000008	FYB1	protein_codir	2.86220623	1.85E-09	8.34E-08
ENSG0000015	GRAP	protein_codir	2.86203235	2.79E-08	8.36E-07
ENSG0000013	PIK3C2G	protein_codir	2.86199043	0.11990872	0.23087266
ENSG0000013	AGAP2	protein_codir	2.86182486	5.58E-13	9.09E-11
ENSG0000018	ATG9B	protein_codir	2.86137559	1.31E-06	2.31E-05
ENSG0000018	WNT7B	protein_codir	2.86123482	0.01362136	0.044225
ENSG0000012	FGD3	protein_codir	2.85952597	3.98E-08	1.14E-06
ENSG0000002	SH2D2A	protein_codir	2.858908	1.10E-09	5.48E-08
ENSG0000017	UTF1	protein_codir	2.85835451	0.00177013	0.00894294
ENSG0000026	CTD-2583A14	lncRNA	2.85724278	0.0097985	0.03423588
ENSG0000022	AC074289.1	lncRNA	2.85472627	4.06E-09	1.63E-07
ENSG0000018	DNAJB13	protein_codir	2.85086987	0.05568285	0.13041298
ENSG0000010	CORO1A	protein_codir	2.85001852	4.31E-08	1.22E-06
ENSG0000016	JAML	protein_codir	2.84951981	1.33E-08	4.49E-07
ENSG0000012	IL17C	protein_codir	2.84884989	0.01654471	0.05135593
ENSG0000012	ART1	protein_codir	2.84840812	0.03575473	0.09303394
ENSG0000010	KLC3	protein_codir	2.84726322	0.02188363	0.0635535
ENSG0000015	SAMSN1	protein_codir	2.84692315	6.05E-12	6.90E-10
ENSG0000023	LINC01122	lncRNA	2.84584848	0.05739909	0.1333246
ENSG0000017	CCNE2	protein_codir	2.84574513	5.15E-10	2.90E-08
ENSG0000018	KCNH8	protein_codir	2.8452976	1.73E-05	0.00020235
ENSG0000017	CTSW	protein_codir	2.84356654	3.54E-09	1.45E-07
ENSG0000023	CT69	lncRNA	2.84186565	0.02102225	0.0616254
ENSG0000009	VNN3	protein_codir	2.84178545	3.38E-06	5.12E-05
ENSG0000020	BTNL2	protein_codir	2.84154504	0.09990235	0.20162673
ENSG0000022	MIR137HG	lncRNA	2.84119422	0.00466537	0.01924731
ENSG0000015	KLRF1	protein_codir	2.84093294	1.80E-06	3.02E-05

ENSG0000017	B3GNT3	protein_codir	2.84019998	0.00028861	0.00208948
ENSG0000027	RP11-711K1.8	lncRNA	2.83820636	0.08229522	0.17422919
ENSG0000025	PCDHA12	protein_codir	2.83819493	0.00034159	0.00240313
ENSG0000023	RP11-66B24.2	lncRNA	2.83773283	0.0035801	0.01556223
ENSG0000021	RAD51AP2	protein_codir	2.83503122	0.06221174	0.1419721
ENSG0000011	KIF21B	protein_codir	2.83451307	3.73E-10	2.21E-08
ENSG0000024	RP11-807G9.2	lncRNA	2.83448956	0.06996922	0.15504587
ENSG0000010	TNNT1	protein_codir	2.83363591	0.01042232	0.03597405
ENSG0000013	GRP	protein_codir	2.8322727	0.0212016	0.06200413
ENSG0000012	CEACAM8	protein_codir	2.83185977	0.02932202	0.08005874
ENSG0000010	DOCK8	protein_codir	2.83144036	4.36E-09	1.72E-07
ENSG0000023	AC073046.25	lncRNA	2.83120372	0.1456619	0.26670081
ENSG0000010	NKG7	protein_codir	2.83013476	3.55E-09	1.45E-07
ENSG0000013	SDS	protein_codir	2.82990952	5.85E-06	8.17E-05
ENSG0000022	LINC01811	lncRNA	2.82847562	0.0058048	0.02274089
ENSG0000018	OR2T10	protein_codir	2.8275752	0.14189585	0.26148675
ENSG0000008	SLCO1A2	protein_codir	2.82694852	0.01783185	0.05430164
ENSG0000017	TSGA10IP	protein_codir	2.82575052	0.00135939	0.00729821
ENSG0000017	NR2F1	protein_codir	2.82567326	9.80E-07	1.81E-05
ENSG0000014	SPACA3	protein_codir	2.82351179	0.06305472	0.14352821
ENSG0000027	RP3-324O17.7	lncRNA	2.82327859	0.02134909	0.0623423
ENSG0000015	HMHB1	protein_codir	2.82192538	0.21243802	0.35206003
ENSG0000017	CHRNA9	protein_codir	2.82015082	0.08097229	0.17222044
ENSG0000013	REG4	protein_codir	2.81998697	0.22528983	0.36706613
ENSG0000025	LINC02457	lncRNA	2.81956418	0.00968412	0.03393927
ENSG0000027	CTD-2396E7.1	lncRNA	2.81947237	0.00253523	0.01180627
ENSG0000028	RP11-534K14.	lncRNA	2.81626269	0.00082438	0.00490825
ENSG0000023	TLR9	protein_codir	2.81456017	2.73E-08	8.20E-07
ENSG0000005	NFE2L3	protein_codir	2.81441331	1.13E-07	2.79E-06
ENSG0000011	FOLR1	protein_codir	2.81300233	0.01527764	0.04837979
ENSG0000015	BCL2L11	protein_codir	2.81274422	5.63E-14	1.37E-11
ENSG0000018	TMEM72	protein_codir	2.8126691	0.05106102	0.12239047
ENSG0000028	RP11-91E8.2	lncRNA	2.80915755	0.03861525	0.09870922
ENSG0000026	RP11-414J4.2	lncRNA	2.80801275	0.02283591	0.06571452
ENSG0000025	RP11-142C4.6	lncRNA	2.80619303	0.00042581	0.00286635
ENSG0000015	FBXO43	protein_codir	2.80617147	0.00198798	0.00978055
ENSG0000023	FILNC1	lncRNA	2.80609617	0.01184283	0.0397015
ENSG0000025	RP1-59M18.2	lncRNA	2.8036828	0.15723119	0.2821234
ENSG0000018	OR4C6	protein_codir	2.80330089	0.21210043	0.3516546
ENSG0000018	SNAI3	protein_codir	2.80157921	4.31E-06	6.24E-05
ENSG0000027	RP11-567P19.	lncRNA	2.80118832	0.00390092	0.01666421
ENSG0000016	ZBBX	protein_codir	2.80011417	0.22082102	0.36161962
ENSG0000027	RP4-555D20.4	lncRNA	2.7995037	0.02691481	0.07474231
ENSG0000017	FCER1A	protein_codir	2.79877596	3.98E-10	2.33E-08
ENSG0000023	AC006369.2	lncRNA	2.79853038	0.00038381	0.00264293
ENSG0000023	LINC01283	lncRNA	2.79760914	0.01826132	0.05528032
ENSG0000018	NELL2	protein_codir	2.79741329	8.88E-07	1.66E-05

ENSG0000023	AC016745.3	lncRNA	2.79732472	0.03331681	0.08817652
ENSG000000(	MATK	protein_codir	2.79625355	1.38E-08	4.63E-07
ENSG0000014	CCNA2	protein_codir	2.79618577	1.25E-06	2.22E-05
ENSG0000023	IATPR	lncRNA	2.79427098	0.0257226	0.07220103
ENSG0000028	RP11-495L19.	lncRNA	2.79219034	0.006126	0.02373074
ENSG0000018	TNFSF15	protein_codir	2.79188771	7.12E-07	1.37E-05
ENSG0000017	S100Z	protein_codir	2.79159509	6.34E-05	0.00059748
ENSG0000016	KLHDC8A	protein_codir	2.7910269	6.43E-06	8.81E-05
ENSG0000024	RP11-334E6.1	lncRNA	2.7904827	0.04615389	0.11322236
ENSG0000016	DRAXIN	protein_codir	2.78979648	3.25E-05	0.00034172
ENSG0000028	XX-2136C111.	lncRNA	2.78953481	0.0245522	0.0696298
ENSG0000016	CCDC78	protein_codir	2.78947668	2.95E-06	4.54E-05
ENSG0000016	CELF5	protein_codir	2.78873176	0.01255798	0.04148941
ENSG0000028	RP11-33I11.4	lncRNA	2.78825681	0.00630559	0.02428675
ENSG0000023	LINC01136	lncRNA	2.78703991	0.00192585	0.00952407
ENSG0000026	LINC00528	lncRNA	2.78701133	4.87E-06	6.97E-05
ENSG0000024	RP11-275H4.1	lncRNA	2.78669047	0.30417362	0.4555018
ENSG0000018	CTD-2600O9.1	lncRNA	2.78649844	0.30573769	0.45737286
ENSG0000023	MIR4422HG	lncRNA	2.78639956	0.12163272	0.23344421
ENSG0000023	AC079807.4	lncRNA	2.7861991	0.00665036	0.02527874
ENSG0000026	LINC02555	lncRNA	2.78612544	0.04040117	0.10229926
ENSG0000028	RP3-470L22.2	lncRNA	2.7857891	0.0035159	0.01533141
ENSG000002(	IQANK1	protein_codir	2.78501011	0.01100775	0.03758756
ENSG0000028	RP5-1189D6.2	protein_codir	2.7848617	0.16028168	0.28603605
ENSG0000025	RP11-753D20	lncRNA	2.78371385	0.10437596	0.20829942
ENSG0000014	GCNT3	protein_codir	2.78351011	0.01457851	0.04666341
ENSG0000014	CHRNA6	protein_codir	2.78338489	0.00022582	0.00170367
ENSG000001(	B3GAT1	protein_codir	2.78265923	7.99E-06	0.00010658
ENSG0000028	RP11-537P24.	lncRNA	2.78161913	0.01176612	0.03953049
ENSG0000026	RP11-810M2.	lncRNA	2.78094019	0.02460159	0.0697205
ENSG0000025	RP11-118B22.	lncRNA	2.7809298	0.0001519	0.00123832
ENSG000001(	GZMH	protein_codir	2.7805135	1.05E-06	1.92E-05
ENSG0000014	SIGLEC10	protein_codir	2.78011464	4.56E-07	9.27E-06
ENSG000002(	TMEM244	protein_codir	2.78010674	0.07545942	0.16368456
ENSG0000012	NKX2-2	protein_codir	2.77767574	0.02863435	0.07859817
ENSG000001(	PRSS33	protein_codir	2.77673796	0.07043826	0.15574004
ENSG0000027	CCL4L2	protein_codir	2.77633709	7.47E-06	0.00010064
ENSG0000028	RP11-24O17.2	lncRNA	2.77572618	0.06822954	0.15220902
ENSG0000025	RP11-305P14.	lncRNA	2.77550303	0.05255496	0.12494131
ENSG0000027	CTC-304I17.6	lncRNA	2.77461403	0.0400618	0.10165012
ENSG0000007	CELSR1	protein_codir	2.77429032	1.52E-09	7.15E-08
ENSG0000027	CTA-414D7.1	lncRNA	2.77348235	1.53E-06	2.66E-05
ENSG0000025	KRT73-AS1	lncRNA	2.77332095	0.06167518	0.14101559
ENSG0000011	BIN2	protein_codir	2.77262779	8.03E-09	2.92E-07
ENSG0000022	LINC02785	lncRNA	2.7719636	0.00042472	0.00286118
ENSG0000022	AC073115.7	lncRNA	2.77187704	0.0258473	0.07244063
ENSG0000016	PRKCB	protein_codir	2.76992332	7.38E-09	2.71E-07

ENSG0000025	MIR3142HG	lncRNA	2.76920095	0.0009783	0.0056305
ENSG0000026	AC137932.5	lncRNA	2.76911898	0.00948773	0.03339082
ENSG0000012	RUNX2	protein_codir	2.76722265	3.23E-08	9.50E-07
ENSG0000010	CACNA1F	protein_codir	2.76673026	1.61E-05	0.00019103
ENSG0000013	TTLL8	protein_codir	2.76642211	0.14876973	0.270847
ENSG0000028	LINC01622	lncRNA	2.76628876	0.15807982	0.28313109
ENSG0000026	RP11-594N15	lncRNA	2.76540839	8.82E-05	0.00078469
ENSG0000018	CYP26C1	protein_codir	2.76266357	0.00026075	0.00192152
ENSG0000014	NT5DC4	protein_codir	2.7624622	0.00694576	0.02618646
ENSG0000015	CERS3	protein_codir	2.76080119	0.02890355	0.07919803
ENSG0000018	TOGARAM2	protein_codir	2.75897253	4.97E-06	7.10E-05
ENSG0000008	CASS4	protein_codir	2.75866963	8.41E-09	3.03E-07
ENSG0000016	CADPS	protein_codir	2.7584438	6.80E-05	0.00063409
ENSG0000017	CA5A	protein_codir	2.75834332	0.10644563	0.21145197
ENSG0000007	TBX21	protein_codir	2.75794996	2.74E-09	1.17E-07
ENSG0000019	MDS2	lncRNA	2.75615277	0.00611471	0.02370028
ENSG0000020	XAGE1B	protein_codir	2.75569223	0.22709244	0.36928426
ENSG0000027	H2BC17	protein_codir	2.75493189	0.02571343	0.07218354
ENSG0000025	RP11-489O18	lncRNA	2.75430348	0.01474958	0.04709089
ENSG0000025	LINC01583	lncRNA	2.75310099	0.03910651	0.09972502
ENSG0000025	RP11-1060J15	lncRNA	2.75246337	0.38903223	0.54199598
ENSG0000013	CD180	protein_codir	2.75177045	1.04E-05	0.00013266
ENSG0000021	LINC02347	lncRNA	2.75146933	0.3111627	0.46295593
ENSG0000023	AC007040.8	lncRNA	2.75115578	0.09566339	0.19508439
ENSG0000014	SFRP2	protein_codir	2.75106057	7.88E-09	2.88E-07
ENSG0000018	HS3ST4	protein_codir	2.75080698	0.04039558	0.10229448
ENSG0000012	CD244	protein_codir	2.7495277	1.04E-08	3.61E-07
ENSG0000028	RP5-1022E24.	lncRNA	2.74918415	0.22699622	0.36916472
ENSG0000023	SLFN14	protein_codir	2.74865954	0.03940102	0.10035559
ENSG0000018	KIAA2012	protein_codir	2.74815538	0.01918924	0.05748525
ENSG0000016	CCDC88B	protein_codir	2.74733272	6.99E-09	2.59E-07
ENSG0000008	CPXM1	protein_codir	2.74732652	5.19E-05	0.00050759
ENSG0000018	BMP8A	protein_codir	2.74713343	3.92E-11	3.30E-09
ENSG0000010	PIK3CG	protein_codir	2.74624856	1.70E-07	3.93E-06
ENSG0000028	RP11-640G3.1	lncRNA	2.74612906	0.1118125	0.219316
ENSG0000016	ARHGAP25	protein_codir	2.74373148	3.23E-09	1.35E-07
ENSG0000016	TMEM63C	protein_codir	2.74339703	4.12E-05	0.00041633
ENSG0000028	CTD-2587M2.	lncRNA	2.74290576	0.14827714	0.2701639
ENSG0000018	RGPD2	protein_codir	2.74290234	1.08E-05	0.00013634
ENSG0000023	RP11-388P9.2	lncRNA	2.74287486	0.05596167	0.13087421
ENSG0000007	TRPM5	protein_codir	2.7418874	0.07334551	0.16037072
ENSG0000027	CCL4	protein_codir	2.74151938	1.95E-06	3.24E-05
ENSG0000018	ARHGAP45	protein_codir	2.7411553	9.80E-09	3.45E-07
ENSG0000027	TBC1D3C	protein_codir	2.73961237	0.16574434	0.29332085
ENSG0000025	RP11-758N13	lncRNA	2.73913316	0.00059685	0.00378097
ENSG0000018	H1-5	protein_codir	2.7390946	0.00049442	0.00324214
ENSG0000014	TRIM55	protein_codir	2.73891549	0.00017119	0.00136625

ENSG0000014	XCL2	protein_codir	2.73805248	1.90E-06	3.16E-05
ENSG0000016	DPEP2	protein_codir	2.73781764	2.45E-07	5.41E-06
ENSG0000013	FOXF2	protein_codir	2.73656542	1.91E-07	4.36E-06
ENSG0000026	LINC01543	lncRNA	2.73617173	0.10813034	0.21393697
ENSG0000017	TCERG1L	protein_codir	2.73473085	0.00977216	0.03416114
ENSG0000028	RP11-399B17.	lncRNA	2.73407339	3.27E-05	0.0003436
ENSG0000012	SOX21	protein_codir	2.73300845	0.033299	0.08814628
ENSG0000020	PDE7A	protein_codir	2.73232456	6.13E-10	3.39E-08
ENSG0000001	BTK	protein_codir	2.7315219	1.44E-08	4.78E-07
ENSG0000025	RP11-277P12.	protein_codir	2.73056823	0.01508004	0.04790232
ENSG0000009	KRT31	protein_codir	2.72888197	0.00638172	0.02451846
ENSG0000006	ABCA7	protein_codir	2.72661018	2.12E-08	6.57E-07
ENSG0000014	SLC27A2	protein_codir	2.72384727	5.59E-06	7.84E-05
ENSG0000017	RFLNA	protein_codir	2.72349426	2.85E-07	6.20E-06
ENSG0000020	ZYG11A	protein_codir	2.72316503	0.00206416	0.01006569
ENSG0000026	RP4-536B24.3	lncRNA	2.72298816	0.24244268	0.38733612
ENSG0000006	GAL	protein_codir	2.72291615	0.00254686	0.01184242
ENSG0000016	GABRG1	protein_codir	2.72204242	0.01282764	0.04218856
ENSG0000016	INPP5D	protein_codir	2.72170106	2.20E-09	9.62E-08
ENSG0000013	HOOK1	protein_codir	2.72167556	2.03E-08	6.32E-07
ENSG0000014	BCL2A1	protein_codir	2.72038014	1.96E-09	8.76E-08
ENSG0000019	SEMA4A	protein_codir	2.71933401	1.42E-10	9.60E-09
ENSG0000013	KIF11	protein_codir	2.71901975	9.32E-09	3.31E-07
ENSG0000013	CEP55	protein_codir	2.71876495	1.34E-07	3.22E-06
ENSG0000023	ELF3-AS1	lncRNA	2.71801616	0.09086126	0.1875346
ENSG0000012	TNFSF18	protein_codir	2.71786873	1.88E-06	3.13E-05
ENSG0000020	CPT1B	protein_codir	2.71675033	2.34E-06	3.75E-05
ENSG0000011	PTPN6	protein_codir	2.71624586	8.53E-08	2.20E-06
ENSG0000024	RP11-247C2.2	lncRNA	2.71261968	8.13E-05	0.00073419
ENSG0000026	AC104534.3	protein_codir	2.71201596	0.20989643	0.34906908
ENSG0000015	SKA1	protein_codir	2.71092096	2.99E-05	0.00032039
ENSG0000025	RP11-343H19	lncRNA	2.71086835	0.22819317	0.37055062
ENSG0000026	RP11-1094M1	lncRNA	2.70767041	3.64E-06	5.44E-05
ENSG0000011	SELPLG	protein_codir	2.70764234	5.82E-08	1.58E-06
ENSG0000018	CEND1	protein_codir	2.70651547	0.00033485	0.00236175
ENSG0000015	ATP2B2	protein_codir	2.7061366	0.00027412	0.00200189
ENSG0000017	LINC01561	lncRNA	2.70530551	0.04293395	0.10725584
ENSG0000023	KIF5C-AS1	lncRNA	2.70445632	0.1127291	0.22052395
ENSG0000014	CRIP3	protein_codir	2.70425184	0.00030424	0.00217527
ENSG0000015	HS3ST3A1	protein_codir	2.7039682	2.87E-07	6.21E-06
ENSG0000012	ARHGAP9	protein_codir	2.70357371	8.30E-09	3.00E-07
ENSG0000022	RP3-333H23.8	lncRNA	2.70245099	0.07645017	0.16518452
ENSG0000027	RP11-1191J2.	lncRNA	2.70116498	0.00029799	0.00213943
ENSG0000028	RP11-731B4.2	lncRNA	2.70088228	0.09357123	0.19196593
ENSG0000025	RP11-76C10.2	lncRNA	2.70043732	0.0653215	0.1475695
ENSG0000020	TTLL10-AS1	lncRNA	2.70039156	0.13032848	0.24559409
ENSG0000027	LRRC8D-DT	lncRNA	2.69827218	0.00019941	0.0015419

ENSG0000028	RP11-756K15.	lncRNA	2.69776746	0.10896083	0.21519471
ENSG0000025	RP11-62C7.2	lncRNA	2.69772692	0.0162776	0.05075494
ENSG0000025	RP11-407N17	lncRNA	2.6977168	0.04119421	0.10383136
ENSG0000024	SOCAR	lncRNA	2.69686255	0.00073557	0.00447893
ENSG0000026	NETO1-DT	lncRNA	2.69537039	0.15688579	0.28157681
ENSG0000016	SFTPB	protein_codir	2.69535468	0.06472455	0.14652014
ENSG0000012	SASH3	protein_codir	2.69498732	9.95E-08	2.50E-06
ENSG0000013	EPSTI1	protein_codir	2.69363409	4.48E-10	2.56E-08
ENSG0000015	DRD3	protein_codir	2.69229542	0.01727332	0.05300816
ENSG0000016	PPBP	protein_codir	2.69154814	0.00067471	0.00417837
ENSG0000027	CCL3	protein_codir	2.69084573	2.24E-06	3.64E-05
ENSG0000027	RP11-94C24.1	lncRNA	2.69058824	0.05833609	0.13502405
ENSG0000012	TNFSF9	protein_codir	2.69051734	3.92E-10	2.30E-08
ENSG0000025	OR13A1	protein_codir	2.6892949	0.01158025	0.03904477
ENSG0000028	RP1-77N19.1	lncRNA	2.68767776	0.04067194	0.10276817
ENSG0000024	SMIM34A	protein_codir	2.68754862	0.07464363	0.16237356
ENSG0000025	RP11-109E24.	lncRNA	2.68701704	0.10973687	0.21629432
ENSG0000023	RP4-742J24.2	lncRNA	2.68688272	0.06747498	0.15111157
ENSG0000019	SH2D1B	protein_codir	2.68524389	1.49E-05	0.00017957
ENSG0000027	CTC-137K3.1	lncRNA	2.68488977	0.02237278	0.06471213
ENSG0000022	LINC00578	lncRNA	2.68477685	7.07E-06	9.57E-05
ENSG0000023	RP11-472G21	lncRNA	2.68295458	0.17538539	0.30552788
ENSG0000008	CHRNA3	protein_codir	2.68278815	0.00863254	0.03098917
ENSG0000016	FBP1	protein_codir	2.68231417	1.42E-07	3.39E-06
ENSG0000025	RP11-403B2.5	lncRNA	2.68199597	0.15899155	0.28435838
ENSG0000013	PTPN22	protein_codir	2.68176727	3.71E-08	1.07E-06
ENSG0000019	DPP4	protein_codir	2.68118075	2.35E-09	1.02E-07
ENSG0000026	RP11-184E9.2	lncRNA	2.67809453	0.24770426	0.39305938
ENSG0000024	RP11-744N12	lncRNA	2.67603565	0.01554726	0.04898089
ENSG0000024	KRTAP5-8	protein_codir	2.67533324	0.04604624	0.1130386
ENSG0000028	RP11-546M8.	lncRNA	2.67520596	0.04713425	0.11502457
ENSG0000017	ADGRE1	protein_codir	2.67465652	7.69E-06	0.00010321
ENSG0000028	RP11-128M1.	lncRNA	2.67269361	0.02150119	0.06269005
ENSG0000014	RORC	protein_codir	2.67010714	0.00022753	0.00171377
ENSG0000013	C3orf20	protein_codir	2.6695919	0.04202924	0.10553161
ENSG0000025	RP11-356K23.	lncRNA	2.66955925	0.1850779	0.31818345
ENSG0000028	LBHD2	protein_codir	2.66889857	0.02583545	0.07241476
ENSG0000022	RP11-235C23.	lncRNA	2.66627164	0.25427375	0.40074414
ENSG0000014	CORIN	protein_codir	2.66488803	2.44E-05	0.00027128
ENSG0000013	RAX	protein_codir	2.66453545	0.09721453	0.19754595
ENSG0000022	OR3A2	protein_codir	2.66379112	0.00484575	0.01979927
ENSG0000019	CSF2RA	protein_codir	2.66313655	8.25E-09	2.99E-07
ENSG0000014	GZMA	protein_codir	2.66307212	1.06E-09	5.31E-08
ENSG0000016	OR1F1	protein_codir	2.66297575	0.15897347	0.28435838
ENSG0000017	MARCHF10	protein_codir	2.66235539	0.02468374	0.06990383
ENSG0000027	RP4-583P15.1	lncRNA	2.66078134	0.0246288	0.06976896
ENSG0000026	CTD-2105E13.	lncRNA	2.65947898	0.03621437	0.09395462

ENSG0000023	HLA-DQA2	protein_codir	2.65887005	0.00203967	0.00997266
ENSG0000023	LINC00427	lncRNA	2.65868149	0.07138427	0.15729027
ENSG0000020	HLA-DOA	protein_codir	2.65854643	3.76E-08	1.09E-06
ENSG0000014	ITGAX	protein_codir	2.65828775	1.63E-06	2.79E-05
ENSG0000016	SKA3	protein_codir	2.65778689	8.73E-05	0.00077788
ENSG0000014	ADAMTS18	protein_codir	2.65749101	8.97E-05	0.00079499
ENSG0000023	LINC00513	lncRNA	2.65700687	0.01765949	0.05388963
ENSG0000025	LINC02320	lncRNA	2.65689559	0.08145813	0.17305903
ENSG0000009	CLSPN	protein_codir	2.65535991	1.18E-06	2.11E-05
ENSG0000023	ITGA6-AS1	lncRNA	2.65529751	1.67E-05	0.00019667
ENSG0000028	RP11-277J24.1	lncRNA	2.65446845	0.066987	0.15033566
ENSG0000009	BLNK	protein_codir	2.6540593	2.11E-07	4.76E-06
ENSG0000018	UTS2R	protein_codir	2.65402519	4.10E-05	0.0004137
ENSG0000021	GPSM3	protein_codir	2.65384682	2.75E-09	1.18E-07
ENSG0000013	ADAMTS14	protein_codir	2.65300538	4.63E-06	6.66E-05
ENSG0000026	FENDRR	lncRNA	2.65214975	3.13E-05	0.00033139
ENSG0000028	RP11-1223D1	lncRNA	2.65184344	3.59E-08	1.04E-06
ENSG0000022	LINC01429	lncRNA	2.64908767	0.14679014	0.26823312
ENSG0000014	GOLGA6D	protein_codir	2.64856567	0.11653501	0.22607973
ENSG0000023	LINC01237	lncRNA	2.64852347	1.75E-05	0.00020513
ENSG0000027	RP11-435O5.6	lncRNA	2.64836912	0.1053417	0.20975689
ENSG0000015	ITGAD	protein_codir	2.64586196	0.00262983	0.01215866
ENSG0000022	RP3-467K16.4	lncRNA	2.6458408	0.0091635	0.03253578
ENSG0000018	GPRIN3	protein_codir	2.64430062	1.90E-08	6.02E-07
ENSG0000012	FAM78A	protein_codir	2.64366489	1.26E-06	2.23E-05
ENSG0000012	SLC12A5	protein_codir	2.64326419	4.00E-06	5.90E-05
ENSG0000016	NSG1	protein_codir	2.64293929	6.43E-07	1.25E-05
ENSG0000018	NOTUM	protein_codir	2.64228174	0.03588285	0.09328731
ENSG0000010	BRS3	protein_codir	2.64054133	0.41475664	0.56788635
ENSG0000026	PSMD7-DT	lncRNA	2.64008862	0.05560347	0.1302816
ENSG0000023	LINC00656	lncRNA	2.64007474	0.01249067	0.04132634
ENSG0000026	GP6-AS1	lncRNA	2.64001272	0.00749894	0.02778281
ENSG0000028	RP11-718D19	lncRNA	2.63873136	0.41508092	0.56819328
ENSG0000012	CD80	protein_codir	2.63780918	2.12E-06	3.47E-05
ENSG0000020	TMEM200C	protein_codir	2.63762997	2.43E-05	0.00026991
ENSG0000023	WAKMAR2	lncRNA	2.63737517	3.20E-08	9.44E-07
ENSG0000017	MBOAT4	protein_codir	2.6369266	0.06193845	0.1414536
ENSG0000028	GS1-53I10.2	lncRNA	2.63653351	0.10324354	0.20666666
ENSG0000028	TRG-AS1	lncRNA	2.63650513	1.10E-05	0.0001387
ENSG0000014	STAR	protein_codir	2.63621555	1.66E-05	0.00019649
ENSG0000028	RP11-57K16.1	lncRNA	2.63557373	3.69E-05	0.00037941
ENSG0000020	PRSS1	protein_codir	2.63535647	0.18980632	0.32416836
ENSG0000020	AADACL4	protein_codir	2.63503872	0.052179	0.12417576
ENSG0000017	HOPX	protein_codir	2.63473005	1.34E-08	4.51E-07
ENSG0000022	AC002064.5	lncRNA	2.63414811	0.03350309	0.08859943
ENSG0000028	RP3-528L19.1	lncRNA	2.63329421	0.00958226	0.03364204
ENSG0000024	KIR3DL2	protein_codir	2.63258065	0.00026375	0.00194003

ENSG0000024	PDE6B-AS1	lncRNA	2.63200727	0.01898549	0.05699774
ENSG0000011	RCAN3	protein_codir	2.63148347	1.34E-09	6.46E-08
ENSG0000024	LINC00880	lncRNA	2.6296508	0.00058391	0.00371006
ENSG0000023	CTAGE9	protein_codir	2.62876367	0.15262944	0.27585559
ENSG0000026	FAM215A	lncRNA	2.62813361	0.05152672	0.12311143
ENSG0000017	FBXO39	protein_codir	2.62809577	0.00029914	0.00214434
ENSG0000023	LINC01115	lncRNA	2.6278558	0.04910925	0.11883629
ENSG0000012	OCM	protein_codir	2.62756366	0.00983885	0.03436816
ENSG0000023	LINC01694	lncRNA	2.62654999	1.60E-05	0.00018961
ENSG0000011	AGMAT	protein_codir	2.62626897	9.12E-06	0.00011934
ENSG0000010	RLN2	protein_codir	2.62562673	0.011427	0.03865859
ENSG0000000	MASP2	protein_codir	2.62553747	0.41744927	0.57011218
ENSG0000026	LINC00563	lncRNA	2.62506586	0.06153479	0.14076452
ENSG0000018	SPNS3	protein_codir	2.62402542	6.63E-07	1.28E-05
ENSG0000018	BHLHE22	protein_codir	2.62304551	5.61E-08	1.53E-06
ENSG0000012	SOHLH2	protein_codir	2.62030866	0.00449243	0.01865036
ENSG0000025	LINC00605	lncRNA	2.6199443	0.17969454	0.31106108
ENSG0000012	EVI2A	protein_codir	2.6194851	4.27E-07	8.75E-06
ENSG0000023	RP1-69D17.3	lncRNA	2.61924674	0.11937828	0.23007663
ENSG0000020	FOXL2NB	protein_codir	2.6189028	0.15531405	0.27942771
ENSG0000015	ADRA2A	protein_codir	2.61852383	8.68E-09	3.12E-07
ENSG0000028	RP11-804C24.	lncRNA	2.61697964	0.03588779	0.09329136
ENSG0000012	AKR1D1	protein_codir	2.61585449	0.07969449	0.17014468
ENSG0000025	LINC00944	lncRNA	2.61536909	3.52E-06	5.29E-05
ENSG0000025	RP11-247L20.	lncRNA	2.61365063	0.12645007	0.2402692
ENSG0000023	RP11-365O16	lncRNA	2.61309731	0.03014554	0.08175764
ENSG0000020	LIME1	protein_codir	2.61308168	8.46E-10	4.39E-08
ENSG0000028	RP11-125H8.3	lncRNA	2.61301113	0.02247753	0.06493667
ENSG0000016	FSTL5	protein_codir	2.61212311	0.07632275	0.16503832
ENSG0000027	CH507-145C2	lncRNA	2.61211283	0.08643055	0.18087678
ENSG0000020	LINC00634	protein_codir	2.6113161	0.33271545	0.48547735
ENSG0000012	LIF	protein_codir	2.61125115	0.00106112	0.00600215
ENSG0000023	LINC02765	lncRNA	2.61029051	0.04004197	0.10162437
ENSG0000011	KIF14	protein_codir	2.61020114	1.53E-06	2.66E-05
ENSG0000017	SNX31	protein_codir	2.6101663	0.00166457	0.00854881
ENSG0000017	PAH	protein_codir	2.60943104	0.17156409	0.3008444
ENSG0000026	RP6-91H8.3	lncRNA	2.60823162	0.00221046	0.01063084
ENSG0000025	RP11-290C10.	lncRNA	2.60789735	0.2515779	0.39769813
ENSG0000028	RP11-673E11.	lncRNA	2.60784679	0.03167869	0.08483096
ENSG0000016	SCIMP	protein_codir	2.60699254	9.76E-06	0.00012588
ENSG0000013	STAT4	protein_codir	2.60570992	1.14E-08	3.92E-07
ENSG0000018	IL1RAPL2	protein_codir	2.60421802	0.11261278	0.22039007
ENSG0000023	AC005220.3	lncRNA	2.60309099	0.0496752	0.11994309
ENSG0000019	SULT1C2	protein_codir	2.60294617	0.01635475	0.05088064
ENSG0000017	CDK1	protein_codir	2.60248585	5.63E-08	1.53E-06
ENSG0000017	PRL	protein_codir	2.60185753	0.18513328	0.31823383
ENSG0000023	ERLNC1	lncRNA	2.60182885	0.26208202	0.40959121

ENSG0000023	FARP1-AS1	lncRNA	2.60137839	0.02171701	0.06316368
ENSG0000013	HEMGN	protein_codir	2.6013549	0.00083595	0.00496214
ENSG0000017	NLRP6	protein_codir	2.60094298	9.81E-06	0.00012649
ENSG0000025	CTD-2184D3.5	lncRNA	2.59882284	0.12242079	0.23463064
ENSG0000024	TMEM150C	protein_codir	2.59792858	4.40E-09	1.74E-07
ENSG0000023	RP3-439F8.1	lncRNA	2.59605802	0.00152407	0.0079816
ENSG0000026	CTD-2547L24.	lncRNA	2.59418736	0.00584355	0.02286101
ENSG0000010	SLC7A5	protein_codir	2.5934058	7.28E-08	1.92E-06
ENSG0000008	CEACAM6	protein_codir	2.59301401	0.07819042	0.16777652
ENSG0000024	HOXC13-AS	lncRNA	2.59186039	0.13245478	0.24873546
ENSG0000025	RP11-326C3.1	lncRNA	2.59177507	0.09710901	0.1973621
ENSG0000014	AC062028.1	lncRNA	2.59105565	0.0617629	0.14115774
ENSG0000010	SLC16A6	protein_codir	2.59061573	1.19E-06	2.13E-05
ENSG0000017	MAL	protein_codir	2.5870137	9.02E-09	3.22E-07
ENSG0000027	RP11-526I2.5	lncRNA	2.5869241	1.53E-08	5.05E-07
ENSG0000026	LINC01482	lncRNA	2.58663793	0.07747632	0.16672348
ENSG0000027	RP11-323P17.	lncRNA	2.58443538	0.00100616	0.00575959
ENSG0000013	AOAH	protein_codir	2.58419383	2.95E-07	6.36E-06
ENSG0000019	AJAP1	protein_codir	2.58259531	7.15E-09	2.65E-07
ENSG0000017	LEP	protein_codir	2.58209863	0.00018912	0.00147393
ENSG0000027	H2AC17	protein_codir	2.58108581	0.00477151	0.01956034
ENSG0000025	LMNTD2-AS1	lncRNA	2.57939861	4.04E-05	0.00040865
ENSG0000015	RGS18	protein_codir	2.57901067	7.48E-08	1.97E-06
ENSG0000007	CLUL1	protein_codir	2.5776416	0.00532138	0.02130096
ENSG0000023	RP11-542K23.	lncRNA	2.57762573	0.00636203	0.02447342
ENSG0000021	RBM38-AS1	lncRNA	2.57758345	1.90E-08	6.01E-07
ENSG0000011	LMNB1	protein_codir	2.57631331	1.41E-11	1.40E-09
ENSG0000022	AF196972.9	lncRNA	2.57541112	0.013254	0.0432867
ENSG0000022	RP11-479J7.2	lncRNA	2.57518402	0.05891549	0.13600315
ENSG0000015	OR3A3	protein_codir	2.57198217	0.17928799	0.31054399
ENSG0000007	CHAT	protein_codir	2.5710916	0.11216258	0.21982051
ENSG0000016	CHRNB2	protein_codir	2.57075133	0.03454892	0.09070734
ENSG0000010	EZH2	protein_codir	2.56988106	6.26E-09	2.37E-07
ENSG0000022	AC093159.1	lncRNA	2.56923014	0.18510644	0.31820749
ENSG0000018	NCR1	protein_codir	2.56893314	6.22E-06	8.58E-05
ENSG0000027	AC124789.1	lncRNA	2.56824645	3.04E-05	0.0003243
ENSG0000023	RP11-9M16.2	lncRNA	2.56816657	0.07622761	0.16488186
ENSG0000026	CTD-2105E13.	lncRNA	2.56629844	0.08826183	0.18361648
ENSG0000022	MIR194-2HG	lncRNA	2.56622077	0.05504814	0.12931023
ENSG0000027	H4C6	protein_codir	2.56508378	0.02417794	0.06871024
ENSG0000023	OSTN-AS1	lncRNA	2.56507429	0.04673206	0.11433093
ENSG0000028	RP11-63A11.2	lncRNA	2.56391785	0.26567034	0.41320412
ENSG0000010	CCNE1	protein_codir	2.56349217	5.17E-09	2.01E-07
ENSG0000014	ST14	protein_codir	2.56273977	3.57E-05	0.00037008
ENSG0000016	PGLYRP4	protein_codir	2.56068173	0.18602547	NA
ENSG0000022	LINC01393	lncRNA	2.56042839	0.00148922	0.0078422
ENSG0000001	CD74	protein_codir	2.56031132	5.90E-10	3.28E-08

ENSG0000010	CSF2RB	protein_codir	2.55964449	1.31E-09	6.36E-08
ENSG0000011	UNC5A	protein_codir	2.55960203	8.33E-05	0.00074894
ENSG0000027	RP11-157H4.1	lncRNA	2.55942561	0.03815789	0.09776633
ENSG0000026	AGK-DT	lncRNA	2.55899384	0.11961969	0.23041326
ENSG0000027	BMS1P14	lncRNA	2.55769077	0.19989295	0.33652586
ENSG0000023	LINC01675	lncRNA	2.55755258	0.06455705	0.14621284
ENSG0000011	VNN2	protein_codir	2.55720749	1.15E-07	2.83E-06
ENSG0000018	FMNL1	protein_codir	2.55712003	1.57E-09	7.31E-08
ENSG0000024	APOBEC3D	protein_codir	2.55695051	1.18E-06	2.11E-05
ENSG0000028	RP11-473M14	lncRNA	2.55488266	0.03374393	0.08910205
ENSG0000016	FNDC1	protein_codir	2.55403301	1.19E-05	0.00014843
ENSG0000027	RP11-405M12	lncRNA	2.55389177	0.18535882	0.31854223
ENSG0000013	OASL	protein_codir	2.55261646	4.42E-07	9.03E-06
ENSG0000028	RP11-1035L22	lncRNA	2.55180683	0.07815094	0.16771786
ENSG0000011	MMP8	protein_codir	2.55157212	0.01425613	0.0458227
ENSG0000022	FAM138B	lncRNA	2.54898446	0.02566032	0.07207018
ENSG0000016	MNDA	protein_codir	2.54839452	1.22E-07	2.97E-06
ENSG0000027	RP11-66B24.8	lncRNA	2.54828063	0.19075772	0.32537003
ENSG0000015	EME1	protein_codir	2.5480519	2.65E-05	0.00028997
ENSG0000020	HCP5	lncRNA	2.54789993	3.75E-10	2.22E-08
ENSG0000028	AP006222.3	lncRNA	2.54638659	0.11505992	0.22387864
ENSG0000028	RP11-394G3.3	lncRNA	2.54636496	0.00015982	0.00129184
ENSG0000026	RP11-498E2.9	lncRNA	2.54631659	0.02998616	0.08143734
ENSG0000027	RP4-555D20.3	lncRNA	2.54549187	0.12652169	0.24037207
ENSG0000016	GFI1B	protein_codir	2.54547177	0.00375568	0.01615508
ENSG0000012	ADORA2A	protein_codir	2.54482464	8.97E-10	4.64E-08
ENSG0000014	TMC6	protein_codir	2.54481793	3.96E-08	1.13E-06
ENSG0000022	RP11-407H12	lncRNA	2.54453283	0.25372415	NA
ENSG0000012	VGF	protein_codir	2.54255402	0.02665122	0.07414942
ENSG0000026	RASSF5	protein_codir	2.54170253	2.29E-14	6.59E-12
ENSG0000027	CCL3L1	protein_codir	2.54138734	4.14E-06	6.06E-05
ENSG0000011	FASLG	protein_codir	2.53899143	4.77E-06	6.85E-05
ENSG0000020	RP11-216L13.	protein_codir	2.53883808	0.07041739	0.15571883
ENSG0000022	LAMP5-AS1	lncRNA	2.53772904	0.17693193	0.30738834
ENSG0000022	CLDN10-AS1	lncRNA	2.53751444	0.36105547	NA
ENSG0000009	HCN2	protein_codir	2.53658573	1.25E-07	3.04E-06
ENSG0000022	RP11-561O23	lncRNA	2.53657687	0.18169806	NA
ENSG0000022	RP11-331F9.4	lncRNA	2.53648904	0.10130698	0.20376484
ENSG0000014	RGS20	protein_codir	2.53629489	0.00187462	0.00933422
ENSG0000010	PTGDS	protein_codir	2.53610102	9.95E-10	5.06E-08
ENSG0000010	CEACAM4	protein_codir	2.53484594	3.51E-08	1.02E-06
ENSG0000022	DBH-AS1	lncRNA	2.53460763	2.48E-06	3.94E-05
ENSG0000021	FAM72D	protein_codir	2.53379038	1.69E-05	0.00019832
ENSG0000010	HAMP	protein_codir	2.53276082	0.00013251	0.00110368
ENSG0000027	RP5-836N17.4	lncRNA	2.53268647	0.19163273	NA
ENSG0000009	CRTAC1	protein_codir	2.53252098	7.65E-05	0.00069707
ENSG0000023	RP3-448I9.2	lncRNA	2.53240319	0.25888995	NA

ENSG0000016	CSMD3	protein_codir	2.53233346	0.0428862	0.10714761
ENSG0000013	CCL21	protein_codir	2.53222663	0.00114054	0.00634218
ENSG0000018	ARHGAP30	protein_codir	2.53195462	1.81E-08	5.78E-07
ENSG0000025	RP11-584P21.	lncRNA	2.53172015	0.01724634	0.05295574
ENSG0000028	RP11-335J9.1	lncRNA	2.53157961	0.00577724	0.02266258
ENSG0000016	CDT1	protein_codir	2.5311905	9.94E-06	0.00012754
ENSG0000023	PAGE2B	protein_codir	2.52965598	0.14090456	NA
ENSG0000016	CLDN19	protein_codir	2.52961207	0.00429942	0.01803068
ENSG0000028	CCAT2	lncRNA	2.52793531	0.09256758	0.19031686
ENSG0000025	RP11-711D18	lncRNA	2.52793506	0.12588571	0.23955543
ENSG0000010	JAK3	protein_codir	2.52788645	4.55E-08	1.28E-06
ENSG0000016	ACSL6	protein_codir	2.52707365	6.24E-11	4.78E-09
ENSG0000007	PAX2	protein_codir	2.52702178	0.18884046	0.3228182
ENSG0000025	USP30-AS1	lncRNA	2.52498794	3.86E-06	5.72E-05
ENSG0000025	RP11-865I6.2	lncRNA	2.52477766	0.00286879	0.01301511
ENSG0000027	FUT8-AS1	lncRNA	2.52463395	8.09E-07	1.53E-05
ENSG0000028	RP1-92O14.7	lncRNA	2.52373339	0.27865596	NA
ENSG0000025	CACNA1C-AS2	lncRNA	2.52342058	3.21E-05	0.00033879
ENSG0000027	RP11-946P6.6	lncRNA	2.52283595	0.00289956	0.01312731
ENSG0000024	RP11-73G16.2	lncRNA	2.52264043	0.26347571	NA
ENSG0000026	RP11-513N24	lncRNA	2.52165935	0.02349054	0.06715057
ENSG0000018	LMNTD2	protein_codir	2.5212883	2.90E-07	6.26E-06
ENSG0000023	RP1-29C18.8	lncRNA	2.51892294	0.00151936	0.0079584
ENSG0000020	HLA-DRA	protein_codir	2.51890473	6.93E-09	2.57E-07
ENSG0000016	IL24	protein_codir	2.51872135	0.09589772	0.19541797
ENSG0000016	TTC16	protein_codir	2.51758209	4.14E-05	0.0004173
ENSG0000023	FNDC1-IT1	lncRNA	2.51735864	0.04129719	0.10404967
ENSG0000013	THEMIS2	protein_codir	2.51731527	2.68E-09	1.15E-07
ENSG0000010	KCNN4	protein_codir	2.51717637	6.94E-07	1.34E-05
ENSG0000012	DUSP4	protein_codir	2.51687036	6.53E-07	1.27E-05
ENSG0000024	HLA-DMB	protein_codir	2.51665547	5.11E-08	1.41E-06
ENSG0000019	LINC00336	lncRNA	2.5152749	0.15787949	0.28288235
ENSG0000018	SOWAHD	protein_codir	2.51469682	3.91E-05	0.00039802
ENSG0000028	RP11-520A21	lncRNA	2.51406114	0.00198419	0.00976712
ENSG0000026	RP11-325K4.2	lncRNA	2.51208668	6.26E-06	8.63E-05
ENSG0000001	WAS	protein_codir	2.50961514	4.19E-09	1.67E-07
ENSG0000028	RP11-27G14.5	lncRNA	2.5090652	0.20788788	0.34650071
ENSG0000028	CTB-161K23.4	protein_codir	2.50855862	0.09137287	0.18839049
ENSG0000017	EFCAB12	protein_codir	2.50834743	0.00011372	0.00097156
ENSG0000028	RP11-219G17	lncRNA	2.50788207	0.10484868	0.20906155
ENSG0000008	HAL	protein_codir	2.50756498	5.65E-06	7.91E-05
ENSG0000015	ANKRD22	protein_codir	2.50659641	0.00013081	0.00109151
ENSG0000028	RP11-316M20	lncRNA	2.50597411	0.00423224	0.01780364
ENSG0000026	RP11-403N16	lncRNA	2.5041592	0.18223463	NA
ENSG0000028	RP11-761I4.6	lncRNA	2.50347006	8.86E-05	0.00078817
ENSG0000016	KBTBD8	protein_codir	2.50331939	9.10E-07	1.70E-05
ENSG0000004	ARAP2	protein_codir	2.50266763	4.64E-11	3.80E-09

ENSG0000018	TREML4	protein_codir	2.50258899	0.37887649	0.53228193
ENSG0000025	RP11-119J18.	lncRNA	2.5018528	0.00370273	0.01596713
ENSG0000026	CTD-2583A14	protein_codir	2.50175461	0.36039322	NA
ENSG0000016	DENND2D	protein_codir	2.50142536	5.47E-07	1.09E-05
ENSG0000028	CH17-206L4.1	protein_codir	2.50106557	0.2710237	NA
ENSG0000022	FXYD7	protein_codir	2.50080311	9.62E-07	1.78E-05
ENSG0000027	RDM1	protein_codir	2.50068716	0.015585	0.04909419
ENSG0000017	FOXG1	protein_codir	2.50054785	0.02759178	0.07623138
ENSG0000027	EPOP	protein_codir	2.50029192	9.14E-08	2.33E-06
ENSG0000018	FOXD4L3	protein_codir	2.49973911	0.06929097	0.15380885
ENSG0000016	MYO7B	protein_codir	2.4993894	8.72E-05	0.00077725
ENSG0000014	NUF2	protein_codir	2.49858533	3.62E-06	5.40E-05
ENSG0000027	ZBTB8B	protein_codir	2.49839465	0.04117499	0.10382082
ENSG0000028	LINC02341	lncRNA	2.49781312	0.0285091	0.07831921
ENSG0000023	RP1-230L10.1	lncRNA	2.49775819	0.07573707	0.16406728
ENSG0000013	LUM	protein_codir	2.49765333	7.50E-07	1.43E-05
ENSG0000013	RHOF	protein_codir	2.49751802	1.51E-09	7.08E-08
ENSG0000006	PRKCZ	protein_codir	2.49714597	1.40E-07	3.34E-06
ENSG0000012	MATN4	protein_codir	2.49679409	0.0020146	0.00988688
ENSG0000010	IL27RA	protein_codir	2.49652937	4.27E-09	1.69E-07
ENSG0000016	NLRP3	protein_codir	2.4947855	1.23E-09	6.06E-08
ENSG0000010	SFRP4	protein_codir	2.49394851	1.19E-07	2.92E-06
ENSG0000008	SCTR	protein_codir	2.49268383	0.18989	NA
ENSG0000019	HLA-DRB1	protein_codir	2.49226005	8.99E-10	4.64E-08
ENSG0000010	CPZ	protein_codir	2.49167638	9.23E-07	1.72E-05
ENSG0000009	IL12RB1	protein_codir	2.49047361	1.19E-06	2.13E-05
ENSG0000014	SLC4A10	protein_codir	2.49014889	6.16E-06	8.52E-05
ENSG0000019	RP11-231C14.	lncRNA	2.49006386	9.10E-05	0.00080383
ENSG0000026	AC004490.1	lncRNA	2.48961696	0.03541027	0.09238917
ENSG0000017	RMI2	protein_codir	2.48948763	2.42E-05	0.0002691
ENSG0000015	MCOLN2	protein_codir	2.48760738	8.17E-05	0.00073714
ENSG0000010	TNFSF8	protein_codir	2.48721455	3.97E-07	8.24E-06
ENSG0000012	APOBEC3A	protein_codir	2.48636397	2.47E-05	0.00027351
ENSG0000022	HLA-DPB1	protein_codir	2.48488844	1.79E-08	5.75E-07
ENSG0000001	TACC3	protein_codir	2.48400045	3.07E-08	9.08E-07
ENSG0000018	C6orf223	lncRNA	2.48321423	0.00085546	0.00505566
ENSG0000011	CENPA	protein_codir	2.4819728	3.86E-05	0.00039374
ENSG0000024	CTB-138E5.1	lncRNA	2.48146528	0.14120615	NA
ENSG0000013	MYBPC3	protein_codir	2.48118226	3.42E-05	0.00035641
ENSG0000027	RP11-245D16	lncRNA	2.4806059	0.00171898	0.0087566
ENSG0000017	TMEM151B	protein_codir	2.47996373	0.01052087	0.03624177
ENSG0000017	FUT3	protein_codir	2.47923018	0.04425598	0.10972623
ENSG0000027	CCL3-AS1	lncRNA	2.47904839	0.0002514	0.0018641
ENSG0000007	TP63	protein_codir	2.47873199	9.63E-10	4.91E-08
ENSG0000025	SEPTIN14P5	lncRNA	2.47831775	0.03440063	0.09045557
ENSG0000025	KB-173C10.1	lncRNA	2.47769745	0.0059366	0.02313983
ENSG0000025	LINC02732	lncRNA	2.47749462	0.01146126	0.0387419

ENSG0000027	RP5-1092A3.4	lncRNA	2.47732458	0.00249117	0.01166006
ENSG0000028	GS1-24F4.4	lncRNA	2.47592432	0.26658289	NA
ENSG0000008	MT3	protein_codir	2.47548594	0.01807652	0.05486657
ENSG0000019	RCSD1	protein_codir	2.47425177	2.81E-11	2.48E-09
ENSG0000028	RP11-1415G1	lncRNA	2.47364827	0.2200328	NA
ENSG0000023	TNFRSF14-AS:	lncRNA	2.47335043	4.92E-05	0.00048422
ENSG0000014	DTL	protein_codir	2.47332052	1.78E-06	3.00E-05
ENSG0000025	RP11-611O2.:	lncRNA	2.4725601	0.36292017	NA
ENSG0000018	PLEKHN1	protein_codir	2.47255156	6.69E-07	1.29E-05
ENSG0000027	LINC01297	lncRNA	2.47124487	0.14696997	NA
ENSG0000012	CENPK	protein_codir	2.47070558	1.54E-06	2.67E-05
ENSG0000021	GAB4	protein_codir	2.4704996	0.11033806	0.21716933
ENSG0000017	RAB37	protein_codir	2.46985769	8.69E-09	3.12E-07
ENSG0000016	NFKBID	protein_codir	2.46949766	1.18E-08	4.04E-07
ENSG0000026	CTD-2535I10.	lncRNA	2.46932706	0.20589636	NA
ENSG0000023	ARHGEF38	protein_codir	2.46929027	0.169699	0.29835051
ENSG0000006	LPAR2	protein_codir	2.46915137	1.45E-07	3.43E-06
ENSG0000025	RP11-613F22.	lncRNA	2.46905067	0.07279836	0.15955884
ENSG0000018	ARL4C	protein_codir	2.46812031	1.77E-12	2.46E-10
ENSG0000027	RP5-1180E21.	lncRNA	2.46803716	1.40E-05	0.00017098
ENSG0000028	RP11-153P14.	lncRNA	2.46710424	0.00281424	0.01282487
ENSG0000016	SYK	protein_codir	2.46684971	1.79E-08	5.74E-07
ENSG0000020	PSMB8-AS1	lncRNA	2.46612312	2.40E-11	2.20E-09
ENSG0000016	KIF15	protein_codir	2.46511989	3.97E-07	8.24E-06
ENSG0000026	RP11-96D1.11	lncRNA	2.46247407	0.00720653	0.02691609
ENSG0000019	DLGAP2	protein_codir	2.4614029	0.00077048	0.00464038
ENSG0000026	RP11-572B2.1	lncRNA	2.46046242	0.00060051	0.00379805
ENSG0000007	ARHGAP15	protein_codir	2.45993493	2.98E-12	3.86E-10
ENSG0000023	LINC02154	lncRNA	2.45859407	0.04026076	0.1020279
ENSG0000028	FAM95C	protein_codir	2.45822492	0.01108921	0.03776155
ENSG0000011	IL10RA	protein_codir	2.45789102	1.39E-07	3.32E-06
ENSG0000013	LCP1	protein_codir	2.45729475	2.62E-07	5.74E-06
ENSG0000026	SPINT1-AS1	lncRNA	2.45660164	6.96E-05	0.00064507
ENSG0000018	IFNL1	protein_codir	2.4561913	0.08378916	0.17653748
ENSG0000026	AC010761.6	lncRNA	2.45603938	0.15235987	NA
ENSG0000025	RP11-493L12.	lncRNA	2.45527592	0.00118546	0.00654454
ENSG0000023	RP11-634B7.4	lncRNA	2.45389111	0.04192149	0.10532808
ENSG0000010	LILRB1	protein_codir	2.45353712	5.72E-07	1.13E-05
ENSG0000025	RP4-809F18.1	lncRNA	2.45277855	0.10591062	0.21064675
ENSG0000027	RP5-892K4.1	lncRNA	2.45254523	0.04210053	0.10565298
ENSG0000014	XCL1	protein_codir	2.45242232	5.74E-07	1.13E-05
ENSG0000026	RP1-168P16.1	lncRNA	2.45062554	0.11637537	0.22588107
ENSG0000025	RP11-1070N1	lncRNA	2.44967941	4.74E-07	9.58E-06
ENSG0000013	DOCK2	protein_codir	2.44964548	2.68E-08	8.08E-07
ENSG0000016	IGFN1	protein_codir	2.44955603	3.48E-05	0.00036141
ENSG0000025	B3GAT1-DT	lncRNA	2.44952692	0.06570066	0.14817115
ENSG0000018	CCR3	protein_codir	2.44918501	0.00012403	0.00104446

ENSG0000016	SYTL3	protein_codir	2.44892979	4.57E-08	1.29E-06
ENSG0000009	OSM	protein_codir	2.44821719	3.97E-06	5.86E-05
ENSG0000026	CTD-2192J16.	protein_codir	2.44695244	0.00246591	0.01156144
ENSG0000017	CST1	protein_codir	2.44672788	0.16794635	0.2963065
ENSG0000028	RP5-1153D9.6	lncRNA	2.44656768	0.15033624	0.27276403
ENSG0000007	OSBPL3	protein_codir	2.4451954	1.85E-09	8.34E-08
ENSG0000024	RP11-424M21	lncRNA	2.44496899	0.09525249	NA
ENSG0000008	SMAP2	protein_codir	2.44492651	1.85E-09	8.34E-08
ENSG0000028	RP11-667K14.	lncRNA	2.44452295	0.00059132	0.00374854
ENSG0000014	DOK3	protein_codir	2.44424879	1.35E-07	3.26E-06
ENSG0000018	ASCL2	protein_codir	2.44356044	7.36E-06	9.94E-05
ENSG0000005	SLC2A3	protein_codir	2.44262984	1.74E-06	2.94E-05
ENSG0000017	CLEC7A	protein_codir	2.44167658	7.71E-07	1.47E-05
ENSG0000016	GJB1	protein_codir	2.44130467	0.21359612	NA
ENSG0000015	ADCY8	protein_codir	2.44087932	0.0511998	0.1225316
ENSG0000016	MUC3A	protein_codir	2.44030957	9.35E-07	1.73E-05
ENSG0000026	RP11-145E5.5	protein_codir	2.43928035	0.11401086	0.22233848
ENSG0000027	H2BC3	protein_codir	2.43726087	0.12584812	0.23952074
ENSG0000012	CKS2	protein_codir	2.43622767	1.56E-11	1.53E-09
ENSG0000026	DLGAP1-AS3	lncRNA	2.43619263	0.12771244	0.24193673
ENSG0000016	INSM2	protein_codir	2.43528405	0.19697778	NA
ENSG0000010	TFEC	protein_codir	2.43443082	7.83E-06	0.00010478
ENSG0000011	CLEC4A	protein_codir	2.43328469	1.13E-07	2.79E-06
ENSG0000013	FKBP11	protein_codir	2.43291543	2.71E-08	8.15E-07
ENSG0000012	IL9R	protein_codir	2.43203606	0.00025469	0.00188193
ENSG0000020	KU-MEL-3	lncRNA	2.43182394	0.20309703	NA
ENSG0000017	OR2T8	protein_codir	2.43136456	0.20702461	NA
ENSG0000014	GRIK4	protein_codir	2.43103762	0.00074791	0.00453709
ENSG0000016	WNT10B	protein_codir	2.43084215	1.15E-06	2.08E-05
ENSG0000028	RP11-121P12.	lncRNA	2.43079953	0.0616891	0.14103575
ENSG0000012	PTK2B	protein_codir	2.43001345	1.14E-10	7.92E-09
ENSG0000018	CAPN12	protein_codir	2.42830906	1.53E-06	2.66E-05
ENSG0000011	TNNT2	protein_codir	2.42784788	0.01049879	0.03617472
ENSG0000026	RP11-650L12.	lncRNA	2.42767834	0.28391248	NA
ENSG0000028	RP11-575A19	lncRNA	2.42616896	0.21888463	NA
ENSG0000028	RP11-497D24	lncRNA	2.42593105	0.06709005	0.15046912
ENSG0000026	RP1-56K13.5	lncRNA	2.4256092	0.05267679	0.12512323
ENSG0000008	NDC80	protein_codir	2.42527408	8.29E-08	2.15E-06
ENSG0000028	RP11-326C3.1	protein_codir	2.42507848	0.4543951	NA
ENSG0000017	NPPA	protein_codir	2.42456818	0.05520314	0.12953111
ENSG0000018	LSAMP	protein_codir	2.42279304	6.11E-07	1.20E-05
ENSG0000018	SYN3	protein_codir	2.42230837	1.32E-07	3.19E-06
ENSG0000025	AF121898.3	lncRNA	2.41975257	0.29452688	NA
ENSG0000011	CRYBG1	protein_codir	2.41950862	1.57E-08	5.15E-07
ENSG0000027	RP11-77E14.2	lncRNA	2.41818331	0.04094145	0.10331685
ENSG0000016	FGF11	protein_codir	2.41737221	6.42E-12	7.24E-10
ENSG0000027	RP1-90G24.11	lncRNA	2.41733235	0.15532001	NA

ENSG0000022	RP11-492E3.2	lncRNA	2.4170577	0.00299649	0.01348898
ENSG0000019	C14orf178	lncRNA	2.41687686	0.21532859	NA
ENSG0000014	DNAJC5B	protein_codir	2.4167468	0.00068788	0.00424561
ENSG0000010	CA2	protein_codir	2.41589694	2.49E-08	7.60E-07
ENSG0000022	LINC00659	lncRNA	2.41571511	0.01857281	0.05603899
ENSG0000026	RP11-568N6.1	lncRNA	2.41566352	1.82E-06	3.05E-05
ENSG0000000	TNMD	protein_codir	2.41525081	0.0012385	0.0067764
ENSG0000008	C1QTNF3	protein_codir	2.41443954	1.10E-05	0.00013881
ENSG0000018	PNMA3	protein_codir	2.41409869	7.29E-05	0.00067148
ENSG0000010	ATP1B4	protein_codir	2.41340649	0.08244279	0.17443464
ENSG0000013	PARVG	protein_codir	2.41299048	2.49E-07	5.50E-06
ENSG0000016	CLEC4E	protein_codir	2.41294809	1.62E-06	2.77E-05
ENSG0000028	RP11-622O11	lncRNA	2.41280285	0.00137116	0.00734942
ENSG0000017	KCNAB3	protein_codir	2.41270077	3.09E-06	4.73E-05
ENSG0000023	LERFS	lncRNA	2.41213959	0.01300578	0.04265252
ENSG0000016	KRT1	protein_codir	2.41070785	0.00795232	0.02906485
ENSG0000015	PADI4	protein_codir	2.41064675	0.00018292	0.00143735
ENSG0000025	RP11-123K3.4	protein_codir	2.41055227	0.01947679	0.0581246
ENSG0000015	CXADR	protein_codir	2.41045405	0.00060253	0.00380818
ENSG0000018	GALR2	protein_codir	2.40989451	0.00225163	0.01078937
ENSG0000023	RP13-297E16.	lncRNA	2.40837347	0.12261186	0.23491534
ENSG0000026	RP11-25L3.3	lncRNA	2.40710265	0.20619223	NA
ENSG0000016	SPC24	protein_codir	2.40679866	6.88E-08	1.83E-06
ENSG0000006	STAG3	protein_codir	2.40677408	3.42E-05	0.00035599
ENSG0000026	CHMP1B-AS1	lncRNA	2.40630056	0.0024891	0.01165237
ENSG0000026	RP11-388C12.	lncRNA	2.40566602	0.01487723	0.04741902
ENSG0000015	UNC5D	protein_codir	2.40554427	0.11977039	0.23065529
ENSG0000027	H2BC9	protein_codir	2.40518517	0.00124433	0.00680561
ENSG0000021	POU5F1B	protein_codir	2.40492377	0.0070424	0.0264425
ENSG0000017	SERPINB9	protein_codir	2.40372809	3.45E-10	2.07E-08
ENSG0000028	RP11-793H4.2	lncRNA	2.40317394	0.05407832	0.12766535
ENSG0000020	DISP3	protein_codir	2.40306651	0.00311557	0.01392451
ENSG0000016	ISLR2	protein_codir	2.40218571	8.83E-06	0.00011592
ENSG0000027	RP4-568C11.4	lncRNA	2.40150779	0.00174697	0.0088616
ENSG0000008	SNX10	protein_codir	2.40086431	2.67E-06	4.18E-05
ENSG0000017	LINGO2	protein_codir	2.40070705	0.02822323	0.07768842
ENSG0000015	LRGUK	protein_codir	2.40054668	8.70E-05	0.00077573
ENSG0000021	EWSAT1	lncRNA	2.40023445	0.01419579	0.04570318
ENSG0000017	MC5R	protein_codir	2.39899908	0.29920206	NA
ENSG0000016	VSNL1	protein_codir	2.39865611	0.00129334	0.00702636
ENSG0000023	LINC02245	lncRNA	2.39747703	2.08E-06	3.42E-05
ENSG0000025	RP11-519G16	lncRNA	2.39745432	0.1483231	0.27022981
ENSG0000018	DUSP29	protein_codir	2.39631161	0.28314751	NA
ENSG0000018	WT1	protein_codir	2.39597541	0.00644884	0.02474192
ENSG0000028	RP4-655C5.10	lncRNA	2.3956117	0.10387983	NA
ENSG0000023	LINC01102	lncRNA	2.39543676	0.29443244	NA
ENSG0000013	GMFG	protein_codir	2.3951636	2.12E-10	1.36E-08

ENSG0000027	RP11-138A9.1	lncRNA	2.39510823	5.15E-05	0.00050389
ENSG0000011	ITGA4	protein_codir	2.39436998	1.89E-07	4.33E-06
ENSG0000016	LAPTM5	protein_codir	2.39431099	5.05E-08	1.40E-06
ENSG0000026	LINC02605	lncRNA	2.39381016	4.56E-07	9.26E-06
ENSG0000020	LINC00487	lncRNA	2.39336422	0.03447427	0.0905716
ENSG0000011	SLC1A2	protein_codir	2.39282644	1.11E-05	0.00013981
ENSG0000023	DNMT3L-AS1	lncRNA	2.39280114	0.22042596	NA
ENSG0000019	CFAP299	protein_codir	2.39275103	0.28805271	NA
ENSG0000015	TIAM1	protein_codir	2.3924633	1.08E-09	5.42E-08
ENSG0000020	PCDHA1	protein_codir	2.39245628	0.06824976	0.15221724
ENSG0000015	IL18	protein_codir	2.39210433	1.28E-06	2.27E-05
ENSG0000028	LINC02456	lncRNA	2.39175303	0.10393211	NA
ENSG0000028	RP11-429P3.9	lncRNA	2.39165498	0.19472091	0.33021377
ENSG0000018	WT1-AS	lncRNA	2.3910322	0.00306693	0.01374246
ENSG0000015	SORCS3	protein_codir	2.3907589	0.00430712	0.01804829
ENSG0000021	SCRT2	protein_codir	2.39072526	0.14687379	NA
ENSG0000013	PLCB2	protein_codir	2.39067842	9.60E-08	2.43E-06
ENSG0000025	RP11-115D19	lncRNA	2.39035857	0.07212465	0.15848762
ENSG0000022	CATIP-AS1	lncRNA	2.39035404	0.0003438	0.00241379
ENSG0000022	LINC00885	lncRNA	2.38894109	0.05605339	0.13103636
ENSG0000014	NLGN4X	protein_codir	2.38890066	4.08E-05	0.00041241
ENSG0000013	SLC6A11	protein_codir	2.38811165	0.00710649	0.02662163
ENSG0000019	SCAMP5	protein_codir	2.38671089	2.45E-06	3.90E-05
ENSG0000013	TNNI2	protein_codir	2.38636277	4.98E-06	7.10E-05
ENSG0000018	MX2	protein_codir	2.38633766	7.18E-09	2.65E-07
ENSG0000021	KB-1572G7.3	lncRNA	2.38609696	0.05256085	0.12494454
ENSG0000024	LUCAT1	lncRNA	2.38579769	4.16E-06	6.08E-05
ENSG0000002	BIRC3	protein_codir	2.38566793	6.90E-10	3.72E-08
ENSG0000010	LHX2	protein_codir	2.38402076	0.003937	0.0167938
ENSG0000025	RP11-704M14	lncRNA	2.38377538	0.2929234	NA
ENSG0000025	RP11-785H5.2	lncRNA	2.38240722	0.05315417	0.12599555
ENSG0000000	SELE	protein_codir	2.38199201	0.00188535	0.00937222
ENSG0000023	OR2W3	protein_codir	2.38035745	0.0063409	0.02440575
ENSG0000008	TMEM40	protein_codir	2.37979156	0.03024602	0.08198989
ENSG0000016	WDR64	protein_codir	2.37965701	0.00240843	0.01135943
ENSG0000015	HKDC1	protein_codir	2.37959616	0.00066494	0.00412991
ENSG0000019	MYH6	protein_codir	2.37957158	0.10701921	0.21225554
ENSG0000013	FOXN4	protein_codir	2.37947638	0.22404556	NA
ENSG0000013	RAB25	protein_codir	2.37806598	0.07569373	0.16402589
ENSG0000019	TAT	protein_codir	2.37751553	0.15384545	NA
ENSG0000017	VSTM2A	protein_codir	2.37649103	0.13670915	0.25423557
ENSG0000015	LYPD6B	protein_codir	2.37618732	0.00244505	0.01148688
ENSG0000027	RP4-539M6.2	lncRNA	2.37567114	0.02590888	0.0725764
ENSG0000027	DUSP5-DT	lncRNA	2.37563829	0.03414267	0.0898971
ENSG0000019	NPIPB9	protein_codir	2.37563387	0.00022518	0.00170027
ENSG0000024	LINC00881	lncRNA	2.37560454	0.21628855	NA
ENSG0000017	UCP2	protein_codir	2.37506185	7.26E-08	1.92E-06

ENSG0000022	RP1-40E16.2	lncRNA	2.37453513	0.14844813	NA
ENSG0000028	RP11-334L22.	lncRNA	2.37375824	0.11489967	0.22362969
ENSG0000018	FCRL6	protein_codir	2.37291359	5.04E-06	7.16E-05
ENSG0000014	FANCD2	protein_codir	2.37291355	1.78E-07	4.09E-06
ENSG0000027	CTD-2199O4.6	lncRNA	2.37244576	0.11269083	0.22046906
ENSG0000005	RELT	protein_codir	2.3721892	2.41E-10	1.52E-08
ENSG0000016	PLK1	protein_codir	2.37181938	9.13E-06	0.00011937
ENSG0000017	LRRTM4	protein_codir	2.37158518	0.00082677	0.00491821
ENSG0000014	SYT2	protein_codir	2.37146422	0.00110758	0.00620737
ENSG0000023	RC3H1-IT1	lncRNA	2.37068526	0.11795633	0.22806817
ENSG0000027	RP4-680D5.8	lncRNA	2.37000537	0.11367687	NA
ENSG0000011	ELAPOR1	protein_codir	2.36993546	2.03E-06	3.35E-05
ENSG0000026	AC027601.1	lncRNA	2.36937364	0.00261602	0.012109
ENSG0000027	AF131215.8	lncRNA	2.36925018	0.07219592	0.15858583
ENSG0000023	HLA-DPA1	protein_codir	2.36872514	6.54E-08	1.75E-06
ENSG0000020	RP11-401P9.6	lncRNA	2.36839364	0.09860474	0.1996189
ENSG0000013	KLRG1	protein_codir	2.36837928	1.88E-09	8.43E-08
ENSG0000016	CD300A	protein_codir	2.36818985	4.19E-07	8.62E-06
ENSG0000024	RP11-521D12	lncRNA	2.36763908	0.09256082	0.19031686
ENSG0000025	CTD-2284J15.	lncRNA	2.367631	1.65E-05	0.000195
ENSG0000017	ERICH3	protein_codir	2.36746342	0.00044789	0.0029881
ENSG0000023	ANKRD44-IT1	lncRNA	2.36611094	0.0093795	0.03312103
ENSG0000014	CTD-3222D19	protein_codir	2.36569086	0.18499056	0.3181271
ENSG0000022	LINC01645	lncRNA	2.36500975	0.03144061	0.08432624
ENSG0000015	WNT3A	protein_codir	2.3647044	0.2212502	NA
ENSG0000019	ZNF165	protein_codir	2.3638076	1.25E-06	2.21E-05
ENSG0000018	CXCR2	protein_codir	2.36352501	0.00130523	0.00707981
ENSG0000023	RP11-136K14.	lncRNA	2.36036792	0.02068447	0.06087016
ENSG0000008	STK17B	protein_codir	2.35973492	1.66E-08	5.39E-07
ENSG0000023	LINC01614	lncRNA	2.3592065	1.51E-05	0.00018109
ENSG0000014	CDCA5	protein_codir	2.35878179	4.47E-07	9.11E-06
ENSG0000025	RP11-826N14	lncRNA	2.35873516	0.23712503	0.38122196
ENSG0000027	ENSG0000027	protein_codir	2.35857131	0.10928287	0.21566112
ENSG0000025	RP11-333A23	lncRNA	2.3580473	0.0136654	0.04434713
ENSG0000028	RP11-326L24.	protein_codir	2.35798392	0.1960437	0.33192636
ENSG0000012	WNT1	protein_codir	2.35796989	5.27E-05	0.00051425
ENSG0000016	IL12A	protein_codir	2.35772744	6.47E-08	1.74E-06
ENSG0000028	AF131216.8	lncRNA	2.3560157	0.2309594	NA
ENSG0000020	NEU4	protein_codir	2.35579598	0.00019484	0.00151164
ENSG0000017	AMZ1	protein_codir	2.35427918	1.21E-09	5.97E-08
ENSG0000027	RP11-126O1.6	lncRNA	2.35233513	0.28788778	NA
ENSG0000011	GNLY	protein_codir	2.35101624	2.10E-09	9.29E-08
ENSG0000027	RP11-93B14.1	lncRNA	2.35026471	1.71E-06	2.90E-05
ENSG0000010	SLC5A5	protein_codir	2.34984732	0.00138627	0.00740167
ENSG0000022	LINC00937	lncRNA	2.34900144	0.0003334	0.00235272
ENSG0000024	LINC00920	lncRNA	2.34865561	1.61E-06	2.76E-05
ENSG0000022	LINP1	lncRNA	2.34849589	0.21811491	NA

ENSG0000018	OR56B2P	protein_codir	2.34841937	0.20901315	NA
ENSG0000028	RP11-399E6.6	lncRNA	2.34702928	0.40198667	0.5549771
ENSG0000022	PP12613	lncRNA	2.34695967	0.31001099	NA
ENSG0000007	WDR62	protein_codir	2.34623824	5.89E-07	1.16E-05
ENSG0000010	TSPAN13	protein_codir	2.34581994	1.13E-08	3.90E-07
ENSG0000014	KCNN3	protein_codir	2.34560582	8.08E-09	2.94E-07
ENSG0000012	ZWINT	protein_codir	2.34360938	1.40E-06	2.45E-05
ENSG0000027	RP11-416N2.4	lncRNA	2.34315964	0.00496509	0.02015812
ENSG0000028	RP11-798P24.	lncRNA	2.34306343	0.06404959	0.1453257
ENSG0000003	APBA2	protein_codir	2.34279845	6.88E-10	3.72E-08
ENSG0000010	AQP9	protein_codir	2.34207877	0.00039591	0.00271121
ENSG0000016	PTAFR	protein_codir	2.34158591	5.46E-06	7.67E-05
ENSG0000023	TRERNA1	lncRNA	2.34149001	0.39697414	NA
ENSG0000016	IL13	protein_codir	2.34143435	0.01223288	0.04069277
ENSG0000026	RP11-303E16.	lncRNA	2.34131611	0.00062035	0.00390121
ENSG0000018	PRF1	protein_codir	2.33917428	2.28E-08	7.04E-07
ENSG0000001	ACP3	protein_codir	2.33788222	3.78E-05	0.00038709
ENSG0000025	RP11-522N14	lncRNA	2.33752774	0.11998985	0.2309974
ENSG0000026	CTB-193M12.	lncRNA	2.33752278	0.04572047	0.11247022
ENSG0000023	MIR600HG	lncRNA	2.33732559	4.39E-06	6.34E-05
ENSG0000027	RP11-231E4.5	lncRNA	2.33660073	0.04022347	0.10197083
ENSG0000016	TMPRSS3	protein_codir	2.33616464	0.00178891	0.00901145
ENSG0000022	EDNRB-AS1	lncRNA	2.33582382	0.0252529	0.07117923
ENSG0000028	LLNLR-229A6.	lncRNA	2.33577248	0.24588404	0.39111478
ENSG0000027	RP11-415F23.	lncRNA	2.33549736	0.00026602	0.00195201
ENSG0000017	TLR6	protein_codir	2.33514706	8.82E-07	1.65E-05
ENSG0000026	RP11-147L13.	lncRNA	2.33397803	5.71E-05	0.00054951
ENSG0000011	PLEK	protein_codir	2.33319894	2.54E-08	7.72E-07
ENSG0000022	RP11-536K7.5	lncRNA	2.33279964	0.10846405	NA
ENSG0000011	CYP27B1	protein_codir	2.33265098	2.69E-05	0.00029345
ENSG0000024	RP11-431M7.	lncRNA	2.33105294	0.30725413	NA
ENSG0000018	FAM153B	protein_codir	2.33083567	0.0012862	0.00699444
ENSG0000010	GLCCI1	protein_codir	2.33043231	3.04E-09	1.29E-07
ENSG0000011	ACRBP	protein_codir	2.33040994	1.47E-05	0.00017761
ENSG0000028	RP1-29K1.6	lncRNA	2.32993053	0.12507431	0.23839297
ENSG0000027	FLJ16779	lncRNA	2.32968512	0.0118213	0.03965801
ENSG0000011	ADGB	protein_codir	2.3294106	0.13566233	0.25277716
ENSG0000023	RP11-310E22.	lncRNA	2.32865269	0.06827193	0.1522544
ENSG0000010	FGF9	protein_codir	2.3285524	1.96E-07	4.45E-06
ENSG0000014	CD53	protein_codir	2.32599716	1.81E-06	3.03E-05
ENSG0000013	METTL21C	protein_codir	2.32423978	0.10883132	NA
ENSG0000024	HOXA11-AS	lncRNA	2.32382782	0.00061765	0.00388597
ENSG0000019	SFMBT2	protein_codir	2.32343681	1.02E-08	3.57E-07
ENSG0000023	AC007283.5	lncRNA	2.32315877	0.02202965	0.06390705
ENSG0000009	CDC6	protein_codir	2.32086457	2.05E-07	4.63E-06
ENSG0000023	AC009784.3	lncRNA	2.32063964	0.00142394	0.00757495
ENSG0000015	SLAMF8	protein_codir	2.32037508	5.92E-05	0.00056662

ENSG0000018	SAMD7	protein_codir	2.32024661	0.30526781	NA
ENSG0000011	COL9A1	protein_codir	2.32002058	0.00204649	0.00999894
ENSG0000011	SGK1	protein_codir	2.31975616	6.98E-09	2.59E-07
ENSG0000025	CLEC12B	protein_codir	2.31967287	0.00309494	0.01385169
ENSG0000028	TMEM272	protein_codir	2.31941849	0.00698904	0.02631727
ENSG0000026	AD000684.2	lncRNA	2.31912987	0.01884007	0.05665971
ENSG0000012	F2RL3	protein_codir	2.31881167	2.67E-06	4.18E-05
ENSG0000011	MEP1A	protein_codir	2.31841004	0.017248	0.05295574
ENSG0000020	CR1	protein_codir	2.31837365	4.30E-05	0.00043025
ENSG0000028	C3orf36	lncRNA	2.31825545	2.36E-05	0.00026377
ENSG0000000	TFAP2B	protein_codir	2.31822555	0.05737088	0.13330391
ENSG0000025	RP5-1024G6.7	lncRNA	2.31800812	0.12847599	0.24303176
ENSG0000024	WNT5A-AS1	lncRNA	2.31773102	0.13113525	0.24681148
ENSG0000016	RHEBL1	protein_codir	2.31764025	2.30E-06	3.71E-05
ENSG0000023	RP11-154H17	lncRNA	2.31742561	0.21127391	NA
ENSG0000013	CHI3L1	protein_codir	2.31536357	0.00593569	0.02313955
ENSG0000026	GABRQ	protein_codir	2.31430292	0.00135294	0.00727006
ENSG0000010	TNFSF13B	protein_codir	2.31400902	1.05E-08	3.64E-07
ENSG0000022	APOC4-APOC2	protein_codir	2.312951	0.08778272	0.18279242
ENSG0000013	OIT3	protein_codir	2.31246296	0.00159643	0.00828045
ENSG0000025	RP11-527N22	lncRNA	2.31198243	0.00680446	0.0257451
ENSG0000011	CDCA3	protein_codir	2.31172878	0.00033819	0.00238331
ENSG0000023	LINC01410	lncRNA	2.31132187	0.00025254	0.00187001
ENSG0000027	LLNLR-470E3.	lncRNA	2.31114159	6.30E-05	0.000595
ENSG0000012	ASPHD2	protein_codir	2.31097371	5.37E-06	7.56E-05
ENSG0000012	MIR9-1HG	lncRNA	2.30904043	0.00112738	0.00628673
ENSG0000013	CRB3	protein_codir	2.30872008	0.00026551	0.00194934
ENSG0000013	BRIP1	protein_codir	2.30850249	1.22E-05	0.00015139
ENSG0000016	REL	protein_codir	2.3081024	5.78E-11	4.53E-09
ENSG0000026	RP11-438B23.	lncRNA	2.3074828	0.14201381	0.2616343
ENSG0000028	RP1-167A14.3	lncRNA	2.30745948	0.00036221	0.00251807
ENSG0000025	RP11-519C12.	lncRNA	2.30559424	0.07577548	NA
ENSG0000022	RP11-301G19	lncRNA	2.30543869	0.22997027	NA
ENSG0000017	ALOX15B	protein_codir	2.30494248	0.00176418	0.00892266
ENSG0000012	TNFRSF8	protein_codir	2.3048561	3.86E-05	0.00039325
ENSG0000007	RAP1GAP	protein_codir	2.30396433	0.00024714	0.00183889
ENSG0000014	SLC2A5	protein_codir	2.30287703	5.30E-06	7.48E-05
ENSG0000014	MARCHF1	protein_codir	2.30252397	2.68E-06	4.19E-05
ENSG0000025	CTD-2012K14	lncRNA	2.30237381	0.06674301	0.14994341
ENSG0000022	BACE2-IT1	lncRNA	2.30219686	0.30087933	NA
ENSG0000024	RP11-133F8.2	lncRNA	2.30047101	0.31462631	NA
ENSG0000017	ZNF648	protein_codir	2.30000144	0.18431543	0.31724261
ENSG0000026	CTC-523E23.1	lncRNA	2.29842834	0.00018865	0.00147101
ENSG0000026	CTD-2207O23	lncRNA	2.29765115	0.04453608	0.11028996
ENSG0000011	IL1R2	protein_codir	2.29758268	1.97E-05	0.00022631
ENSG0000026	RP11-1151B14	lncRNA	2.2970276	0.01426192	0.04583596
ENSG0000027	CTD-3154N5.2	lncRNA	2.29575989	0.14529623	0.26622541

ENSG0000018	SPATC1	protein_codir	2.29572003	0.00023105	0.00173553
ENSG0000018	ANKRD34B	protein_codir	2.2956964	0.03704784	0.09564084
ENSG0000028	RP11-726G1.2	lncRNA	2.29455033	3.50E-08	1.02E-06
ENSG0000012	PTGER2	protein_codir	2.29373328	2.46E-07	5.43E-06
ENSG0000013	PRKG2	protein_codir	2.29286562	6.37E-05	0.00059949
ENSG0000028	RP11-455M16	lncRNA	2.29268475	0.31421	NA
ENSG0000028	RP11-331G2.1	lncRNA	2.29177383	0.16756613	NA
ENSG0000022	RP11-445F6.2	lncRNA	2.29074362	0.00134237	0.00724003
ENSG0000024	LINC02481	lncRNA	2.2904136	0.00026465	0.00194352
ENSG0000026	RP11-631M21	lncRNA	2.28989322	0.22932696	NA
ENSG0000023	ARHGAP26-IT	lncRNA	2.28909785	0.20880018	NA
ENSG0000023	RP11-439E19.	lncRNA	2.28804425	0.41161751	NA
ENSG0000020	CCNI2	protein_codir	2.28695196	5.46E-05	0.0005292
ENSG0000023	AC008063.3	lncRNA	2.28554955	0.31458103	NA
ENSG0000024	LINC00877	lncRNA	2.28535357	6.16E-05	0.00058548
ENSG0000012	PIMREG	protein_codir	2.28506465	0.00040985	0.00278983
ENSG0000014	HTR2C	protein_codir	2.28298305	0.41087252	NA
ENSG0000010	GIMAP2	protein_codir	2.28248955	3.99E-06	5.88E-05
ENSG0000028	RP1-133I17.1	lncRNA	2.28220781	0.31628781	NA
ENSG0000016	KCTD19	protein_codir	2.28138727	0.0169818	0.05236668
ENSG0000011	C11orf21	protein_codir	2.28131629	7.17E-05	0.00066242
ENSG0000010	ICAM5	protein_codir	2.28095416	3.47E-07	7.34E-06
ENSG0000014	LMAN1L	protein_codir	2.28067129	0.01754906	0.05363562
ENSG0000026	CTD-2587H24	lncRNA	2.28059587	0.23929749	NA
ENSG0000011	GPR68	protein_codir	2.2804846	2.13E-06	3.47E-05
ENSG0000028	RP11-783N9.1	lncRNA	2.28001644	0.15234893	0.27543873
ENSG0000025	KB-173C10.2	lncRNA	2.27902895	0.07658645	0.16539258
ENSG0000017	GJC3	protein_codir	2.27892213	0.00380079	0.01633136
ENSG0000020	PKP4-AS1	lncRNA	2.278406	0.08264766	0.17469976
ENSG0000008	CXCL2	protein_codir	2.27782109	0.00029251	0.00211051
ENSG0000025	CD8B2	protein_codir	2.27752202	0.00371955	0.01602716
ENSG0000028	RP11-792D21	lncRNA	2.2773634	0.05838984	0.13511039
ENSG0000027	NEFL	protein_codir	2.27689146	4.30E-05	0.00043084
ENSG0000023	LINC00642	lncRNA	2.27675986	0.31353531	NA
ENSG0000028	RP11-517H2.7	lncRNA	2.27650611	0.24204121	NA
ENSG0000001	ALOX5	protein_codir	2.2764695	2.85E-08	8.52E-07
ENSG0000013	IGF2BP3	protein_codir	2.27548422	6.05E-05	0.00057683
ENSG0000027	RP11-405M12	lncRNA	2.2742466	0.00016742	0.00134074
ENSG0000010	CNFN	protein_codir	2.27276855	5.21E-05	0.00050853
ENSG0000016	LIPC	protein_codir	2.27265077	1.06E-06	1.94E-05
ENSG0000013	PRAM1	protein_codir	2.27211227	7.35E-06	9.93E-05
ENSG0000021	AC004540.5	lncRNA	2.27175058	7.25E-07	1.39E-05
ENSG0000020	FAM78B-AS1	lncRNA	2.27123657	0.06659482	0.14973533
ENSG0000001	CD4	protein_codir	2.27012537	8.40E-07	1.58E-05
ENSG0000008	ARHGAP4	protein_codir	2.26952585	6.77E-08	1.81E-06
ENSG0000027	RP11-61O11.1	lncRNA	2.26913447	0.23800115	NA
ENSG0000025	RP11-705C15.	lncRNA	2.26908008	2.68E-08	8.08E-07

ENSG0000016	SLC51A	protein_codir	2.26875466	0.01163163	0.03917962
ENSG0000020	SAP25	protein_codir	2.26874933	0.00040232	0.00274647
ENSG0000016	IFI27	protein_codir	2.26848296	2.93E-07	6.33E-06
ENSG0000028	AC005307.5	lncRNA	2.26818347	0.12054209	NA
ENSG0000025	LINC01337	lncRNA	2.26816311	0.31886844	NA
ENSG0000018	AQP12B	protein_codir	2.26734441	0.31522355	NA
ENSG0000026	RP11-173A16	lncRNA	2.26559834	0.02870318	0.07875056
ENSG0000014	VAV1	protein_codir	2.26500091	1.82E-08	5.80E-07
ENSG0000013	CDCA8	protein_codir	2.26459863	2.57E-05	0.00028335
ENSG0000014	FGD2	protein_codir	2.26450164	1.28E-05	0.00015784
ENSG0000028	RP11-826N14	protein_codir	2.26427005	0.03058903	0.08269026
ENSG0000016	PTGDR	protein_codir	2.26420775	5.88E-07	1.16E-05
ENSG0000016	FCRLB	protein_codir	2.2639536	7.19E-05	0.00066436
ENSG0000025	KLRF2	protein_codir	2.26350257	0.16729989	NA
ENSG0000019	RASGEF1A	protein_codir	2.26338419	1.28E-06	2.26E-05
ENSG0000010	ZP2	protein_codir	2.26222317	0.25642131	0.40311666
ENSG0000028	RP11-345P4.1	lncRNA	2.26167478	0.00016703	0.00133915
ENSG0000023	RP11-146I2.1	lncRNA	2.26141188	0.21906557	NA
ENSG0000010	NEFM	protein_codir	2.26098272	0.00096618	0.00557417
ENSG0000017	DMRT2	protein_codir	2.26047963	0.00034396	0.0024143
ENSG0000024	L1TD1	protein_codir	2.26047377	0.00069557	0.00428212
ENSG0000018	UBL4B	protein_codir	2.26046875	0.01962436	0.05843935
ENSG0000010	FSD1	protein_codir	2.25984528	0.00015021	0.00122783
ENSG0000027	HNF1B	protein_codir	2.25944535	0.23748375	NA
ENSG0000014	CMTM2	protein_codir	2.2588543	0.0006699	0.00415604
ENSG0000023	DARS1-AS1	lncRNA	2.25846338	3.02E-05	0.00032233
ENSG0000008	ORC1	protein_codir	2.25700799	1.23E-05	0.00015286
ENSG0000028	H3C3	protein_codir	2.25649387	0.04384107	0.10888322
ENSG0000011	LPXN	protein_codir	2.25615944	1.05E-08	3.65E-07
ENSG0000011	RPS6KA1	protein_codir	2.2550125	2.19E-07	4.89E-06
ENSG0000024	LINC02415	lncRNA	2.25489774	0.04303993	0.10740545
ENSG0000023	AC004538.3	lncRNA	2.25429235	0.0056919	0.02240139
ENSG0000028	NPBWR1	protein_codir	2.25332408	0.06198992	0.14153604
ENSG0000027	RP11-517H2.6	lncRNA	2.25240653	0.00054179	0.00349619
ENSG0000028	RP5-876B10.7	protein_codir	2.25230985	0.48765062	NA
ENSG0000011	CCL20	protein_codir	2.25120876	0.01225793	0.04076139
ENSG0000005	CYBA	protein_codir	2.25105205	2.07E-08	6.45E-07
ENSG0000027	RP11-446N19	lncRNA	2.25039538	0.01163331	0.03917962
ENSG0000025	RP13-494C23.	lncRNA	2.25016391	0.16621896	NA
ENSG0000025	RP11-760H22	lncRNA	2.24966149	8.63E-06	0.00011383
ENSG0000028	CCDC195	protein_codir	2.24884444	0.41772914	NA
ENSG0000010	CCL1	protein_codir	2.24712389	0.07770592	0.16708454
ENSG0000016	CDX2	protein_codir	2.24648611	0.48879375	NA
ENSG0000010	SIGLEC6	protein_codir	2.24584271	2.25E-05	0.00025295
ENSG0000025	RP11-463C8.4	protein_codir	2.24573537	0.41207821	NA
ENSG0000025	RP11-280G9.1	lncRNA	2.24541282	0.1821265	NA
ENSG0000023	HLA-B	protein_codir	2.24510986	2.77E-14	7.64E-12

ENSG0000026	RP11-667K14.	lncRNA	2.24413624	0.00193275	0.00955308
ENSG0000026	CCDC187	protein_codir	2.24090205	0.11240361	0.22013668
ENSG0000018	SHISA2	protein_codir	2.24023255	8.73E-07	1.64E-05
ENSG0000026	AC006486.9	protein_codir	2.24019689	0.22957991	0.37205058
ENSG0000008	ARHGAP28	protein_codir	2.24011902	9.60E-05	0.00083995
ENSG0000017	LRRC8E	protein_codir	2.23943551	0.00161452	0.0083523
ENSG0000023	RFX5-AS1	lncRNA	2.23898952	6.46E-05	0.00060767
ENSG0000025	RP11-351M8.	lncRNA	2.23861687	0.1488769	0.27100639
ENSG0000028	RP5-1171I10.9	lncRNA	2.23836927	0.07597687	0.16448355
ENSG0000017	OSCAR	protein_codir	2.23818252	2.10E-05	0.00023832
ENSG0000018	C9orf139	lncRNA	2.23768104	0.00014091	0.00116387
ENSG0000020	HLA-DMA	protein_codir	2.23744524	1.56E-08	5.11E-07
ENSG0000011	CD86	protein_codir	2.23677364	3.46E-07	7.34E-06
ENSG0000021	C10orf105	protein_codir	2.23671234	0.00011804	0.00099978
ENSG0000012	CHRNA2	protein_codir	2.23625645	0.11576352	NA
ENSG0000017	PKIA	protein_codir	2.23624644	3.79E-08	1.09E-06
ENSG0000027	USP6NL-AS1	lncRNA	2.23591533	0.12525388	NA
ENSG0000022	RP11-136K14.	lncRNA	2.23515069	0.09002654	0.18624655
ENSG0000019	DMBX1	protein_codir	2.23464454	0.19271186	NA
ENSG0000022	AP001046.6	lncRNA	2.2343768	0.0906441	0.18718429
ENSG0000028	RP11-115D10	lncRNA	2.23289913	2.69E-08	8.11E-07
ENSG0000028	RP3-333H23.1	lncRNA	2.23275547	0.00703901	0.02643696
ENSG0000014	FLG	protein_codir	2.23262123	0.06628031	0.14916175
ENSG0000013	CRACR2A	protein_codir	2.23261345	4.97E-08	1.38E-06
ENSG0000010	SH3BP1	protein_codir	2.23246049	6.16E-08	1.67E-06
ENSG0000026	RP11-148O21	lncRNA	2.23139533	0.05440181	0.1282282
ENSG0000027	H2BC14	protein_codir	2.23111397	0.1679093	0.29626005
ENSG0000025	RP11-10A14.3	lncRNA	2.22965842	0.03762037	0.09670519
ENSG0000009	ICAM1	protein_codir	2.22962224	7.12E-08	1.89E-06
ENSG0000025	RP11-328K2.1	lncRNA	2.22948476	0.31770042	NA
ENSG0000011	ST8SIA4	protein_codir	2.22906326	8.98E-08	2.30E-06
ENSG0000023	PAGE2	protein_codir	2.22706249	0.26196815	NA
ENSG0000012	TGM3	protein_codir	2.22650607	0.05179387	0.12353599
ENSG0000015	MAPK13	protein_codir	2.22643182	6.47E-09	2.43E-07
ENSG0000025	LINC02345	lncRNA	2.22615317	0.00413513	0.01750918
ENSG0000025	CTD-2325P2.4	lncRNA	2.22577129	0.21324213	0.3529754
ENSG0000026	LINC01887	lncRNA	2.22498475	0.33119443	NA
ENSG0000028	RP11-345F18.	lncRNA	2.2248441	0.00151397	0.0079407
ENSG0000028	RP4-671O14.6	lncRNA	2.22442876	3.73E-05	0.00038346
ENSG0000018	ITPRID1	protein_codir	2.22420603	0.17183155	NA
ENSG0000028	LINC01374	lncRNA	2.22377475	0.04696194	0.11468513
ENSG0000028	RP11-429J10.	lncRNA	2.22341035	0.236766	NA
ENSG0000027	RP11-415F23.	lncRNA	2.22280047	0.00039848	0.00272631
ENSG0000010	SYNGR4	protein_codir	2.22244062	0.25419776	NA
ENSG0000016	FCGR3B	protein_codir	2.22215792	0.00647899	0.02483
ENSG0000025	RP11-440I14.3	lncRNA	2.2211991	0.1763462	NA
ENSG0000000	HOXA11	protein_codir	2.22106182	0.00028056	0.0020419

ENSG0000019	ELOVL2	protein_codir	2.21989077	1.52E-07	3.57E-06
ENSG0000023	AC007278.3	lncRNA	2.21899608	0.00381556	0.01637951
ENSG0000018	OR52N2	protein_codir	2.21873292	0.10489338	0.20909029
ENSG0000027	CH507-254M2	lncRNA	2.21867655	0.01189128	0.03981896
ENSG0000023	DAPK1-IT1	lncRNA	2.21773891	0.24332031	NA
ENSG0000013	CYP2C9	protein_codir	2.21761028	0.14903616	0.27120694
ENSG0000023	AC005083.1	lncRNA	2.21746228	0.00051378	0.00334211
ENSG0000024	MNX1-AS1	lncRNA	2.21742453	0.33230011	NA
ENSG0000018	RHD	protein_codir	2.21726773	0.125067	NA
ENSG0000012	STRIP2	protein_codir	2.21711079	1.43E-05	0.00017368
ENSG0000010	HAS1	protein_codir	2.2166118	0.00184207	0.00920036
ENSG0000026	TPBGL	protein_codir	2.21649202	1.78E-06	2.99E-05
ENSG0000014	CXCL14	protein_codir	2.21649034	1.12E-07	2.77E-06
ENSG0000028	RP11-261K4.1	lncRNA	2.21646824	0.49470836	NA
ENSG0000024	RP11-410D17	lncRNA	2.21603173	0.13533825	0.25236582
ENSG0000023	RP11-399H11	lncRNA	2.21540349	0.32816928	NA
ENSG0000021	AATBC	lncRNA	2.21476787	0.00057521	0.00366574
ENSG0000016	AMN	protein_codir	2.21452582	0.00361289	0.01566286
ENSG0000009	UNC13D	protein_codir	2.21414199	3.27E-07	6.97E-06
ENSG0000023	RP11-447M12	lncRNA	2.21400796	0.4249892	NA
ENSG0000022	AC112721.1	lncRNA	2.21123237	0.10042903	NA
ENSG0000026	ADPGK-AS1	lncRNA	2.21108173	0.00065948	0.00410061
ENSG0000015	FMN2	protein_codir	2.21080457	9.13E-05	0.000806
ENSG0000026	RP11-845C23.	lncRNA	2.20988465	0.14137268	0.26076623
ENSG0000025	RP11-655C2.3	lncRNA	2.20987804	0.08303113	0.17529006
ENSG0000025	LINC01340	lncRNA	2.20969449	0.33410611	NA
ENSG0000028	RP11-148I22.1	lncRNA	2.20922165	0.33776346	NA
ENSG0000016	RNASE6	protein_codir	2.20736587	7.88E-07	1.49E-05
ENSG0000018	RXFP3	protein_codir	2.20730097	0.49652211	NA
ENSG0000027	RP11-278A23	lncRNA	2.20725135	0.08406663	0.17698912
ENSG0000018	RP5-1086D14	lncRNA	2.20656336	4.98E-06	7.10E-05
ENSG0000025	AE000661.37	lncRNA	2.20651047	6.06E-05	0.00057719
ENSG0000028	RP11-81A1.10	lncRNA	2.20619999	9.51E-05	0.000834
ENSG0000006	PRR11	protein_codir	2.206014	3.45E-08	1.01E-06
ENSG0000023	AC103563.8	lncRNA	2.20583986	0.23626411	NA
ENSG0000010	PTK6	protein_codir	2.20543424	0.00057291	0.00365195
ENSG0000009	WHRN	protein_codir	2.20532262	7.48E-09	2.75E-07
ENSG0000025	RP11-718G2.5	lncRNA	2.20509094	0.12031601	0.23139949
ENSG0000018	FAM72B	protein_codir	2.20508119	1.84E-05	0.00021372
ENSG0000024	RP11-155G14	lncRNA	2.20443034	0.01157757	0.03904444
ENSG0000026	GAPLINC	lncRNA	2.20389789	0.00046056	0.00305878
ENSG0000028	CTD-2319I12.	lncRNA	2.20311956	0.114106	0.22250832
ENSG0000028	RP1-149L1.2	lncRNA	2.20236859	0.24638581	NA
ENSG0000010	COMP	protein_codir	2.20183086	4.16E-05	0.00041855
ENSG0000023	ANKRD65	protein_codir	2.20094906	1.59E-09	7.41E-08
ENSG0000019	MAP1LC3C	protein_codir	2.20056404	3.79E-05	0.00038813
ENSG0000027	RP11-344P13.	lncRNA	2.19993965	0.03747631	0.09643149

ENSG0000022	RP1-80N2.2	lncRNA	2.19934602	0.05702099	0.13269097
ENSG0000022	RP11-438F14.	lncRNA	2.19827227	0.08445028	0.17762083
ENSG0000015	KCNJ6	protein_codir	2.19823887	0.00047657	0.00314676
ENSG0000027	OR2T11	protein_codir	2.19814146	0.33993191	NA
ENSG0000017	CRYBG2	protein_codir	2.19812366	0.00688496	0.02598916
ENSG0000016	SLC38A11	protein_codir	2.19776298	2.48E-07	5.48E-06
ENSG0000014	CDHR1	protein_codir	2.19752605	6.45E-06	8.83E-05
ENSG0000017	CDC42BPG	protein_codir	2.19691599	2.07E-06	3.40E-05
ENSG0000025	AC084117.3	lncRNA	2.19674761	0.19843899	0.33475141
ENSG0000015	SVOPL	protein_codir	2.19604548	0.26071712	NA
ENSG0000024	RP11-57A19.2	lncRNA	2.19494525	0.15662322	0.28117864
ENSG0000014	N4BP3	protein_codir	2.19478883	1.24E-06	2.21E-05
ENSG0000025	CTD-2540L5.5	lncRNA	2.19367771	0.49922392	NA
ENSG0000023	RP11-114M1.	lncRNA	2.19330526	0.05609014	0.13110008
ENSG0000028	RP11-9N12.3	lncRNA	2.19312335	0.49933403	NA
ENSG0000025	RP11-404P21.	lncRNA	2.1930757	0.1930744	NA
ENSG0000020	LST1	protein_codir	2.19191716	2.04E-07	4.62E-06
ENSG0000014	EAF2	protein_codir	2.19185222	1.40E-06	2.45E-05
ENSG0000019	FAM163B	protein_codir	2.19181286	0.00780436	0.02864537
ENSG0000011	KYNU	protein_codir	2.19164295	1.14E-06	2.07E-05
ENSG0000023	DLGAP4-AS1	lncRNA	2.19089646	0.00029208	0.00210813
ENSG0000023	TFAP2E-AS1	lncRNA	2.19067904	3.28E-06	5.00E-05
ENSG0000023	LINC01914	lncRNA	2.19058465	0.00172663	0.00878874
ENSG0000020	AC144450.2	lncRNA	2.19018001	0.18900794	NA
ENSG0000013	SPINK5	protein_codir	2.18993564	0.00288964	0.01309678
ENSG0000025	LINC02099	lncRNA	2.18941415	0.03890861	0.09932469
ENSG0000028	CTC-782O7.3	lncRNA	2.18882788	0.33025405	NA
ENSG0000021	EFCAB8	protein_codir	2.18860362	0.00353577	0.01540105
ENSG0000018	NEB	protein_codir	2.18766576	4.15E-06	6.07E-05
ENSG0000018	POTEG	protein_codir	2.18718028	0.25878276	NA
ENSG0000024	PRR5-ARHGAI	protein_codir	2.18627677	0.03488761	0.09131879
ENSG0000016	C2	protein_codir	2.18624134	9.03E-07	1.68E-05
ENSG0000017	B3GNT5	protein_codir	2.18538602	1.20E-07	2.94E-06
ENSG0000013	STAB2	protein_codir	2.18520002	0.00766549	0.02826336
ENSG0000020	KLRC2	protein_codir	2.18456927	0.00477182	0.01956034
ENSG0000022	HORMAD2-AS	lncRNA	2.1841438	0.08267864	0.17470615
ENSG0000016	TGFA	protein_codir	2.18326403	1.23E-08	4.18E-07
ENSG0000015	FUT6	protein_codir	2.18294858	0.17699752	NA
ENSG0000015	BATF	protein_codir	2.18253088	1.07E-05	0.00013504
ENSG0000012	PRKCG	protein_codir	2.1814959	0.00211748	0.01027132
ENSG0000017	FAM9B	protein_codir	2.1813782	0.24949458	NA
ENSG0000013	AMPD3	protein_codir	2.18103235	8.57E-07	1.61E-05
ENSG0000014	ZP1	protein_codir	2.18024777	0.04086801	0.10316923
ENSG0000022	RP11-545E17.	lncRNA	2.18006621	0.00010291	0.00089018
ENSG0000012	EPX	protein_codir	2.17972384	0.21560164	0.3557936
ENSG0000028	RP11-723P16.	lncRNA	2.17916506	0.11126037	0.21856412
ENSG0000023	RP4-704D21.2	lncRNA	2.17862991	0.33525131	NA

ENSG0000017	FPR2	protein_codir	2.17837752	0.00103277	0.00587904
ENSG0000028	PERCC1	protein_codir	2.17734999	0.08200812	0.17379455
ENSG0000012	IGFLR1	protein_codir	2.17561924	4.24E-08	1.20E-06
ENSG0000015	TTN	protein_codir	2.17544814	3.50E-09	1.44E-07
ENSG0000017	ANXA2R	protein_codir	2.1752961	1.69E-07	3.92E-06
ENSG0000027	RP11-353N4.6	lncRNA	2.17518027	0.18486148	NA
ENSG0000014	MCTP2	protein_codir	2.17426449	3.53E-06	5.30E-05
ENSG0000011	WWC1	protein_codir	2.17416577	0.00296011	0.01334613
ENSG0000012	PLPPR3	protein_codir	2.17300021	0.10895324	0.21519471
ENSG0000023	AC016683.5	lncRNA	2.17282448	0.3256907	NA
ENSG0000011	GALNT3	protein_codir	2.17089269	9.35E-06	0.00012142
ENSG0000013	KIF12	protein_codir	2.16986154	0.05018061	0.12086081
ENSG0000027	RP11-474P2.5	lncRNA	2.16949862	0.21072597	0.35006925
ENSG0000028	LINC01943	lncRNA	2.16823613	2.16E-06	3.52E-05
ENSG0000005	RAD51	protein_codir	2.16748262	1.71E-05	0.00020042
ENSG0000026	CTC-250I14.3	lncRNA	2.16705877	0.2546121	NA
ENSG0000022	AC012368.1	lncRNA	2.16601831	0.06821179	0.15219243
ENSG0000027	RP11-360I2.1	lncRNA	2.16487179	0.27604973	0.4241957
ENSG0000021	DIO1	protein_codir	2.16462585	0.04811736	0.11694839
ENSG0000017	HAS2	protein_codir	2.16434131	0.00014906	0.00122062
ENSG0000018	OR52N4	protein_codir	2.1637097	7.21E-05	0.00066544
ENSG0000001	FMO1	protein_codir	2.16326618	1.44E-05	0.00017403
ENSG0000023	RP11-213H15	lncRNA	2.16312913	0.05670055	0.13219119
ENSG0000024	CTC-441N14.2	lncRNA	2.16302695	0.16164074	0.28780074
ENSG0000015	NR4A2	protein_codir	2.16273072	3.69E-06	5.49E-05
ENSG0000020	SPRN	protein_codir	2.16204696	0.00223825	0.0107383
ENSG0000028	RP11-799O21	lncRNA	2.16186276	0.00483912	0.01977751
ENSG0000026	RP11-506E9.3	lncRNA	2.16152561	0.43147143	NA
ENSG0000018	S100A14	protein_codir	2.16116067	0.00998628	0.03476443
ENSG0000012	DBH	protein_codir	2.16034373	0.08239492	0.17438679
ENSG0000013	IL36B	protein_codir	2.16007205	0.3485125	NA
ENSG0000025	RP11-261P9.4	lncRNA	2.15966532	0.00162158	0.00838568
ENSG0000026	RP4-639J15.3	lncRNA	2.15947413	0.24676055	NA
ENSG0000018	CADM1	protein_codir	2.15934864	1.30E-08	4.41E-07
ENSG0000023	LINC01797	lncRNA	2.1589463	0.35543592	NA
ENSG0000016	DEGS2	protein_codir	2.1583467	5.79E-06	8.08E-05
ENSG0000020	C2CD4B	protein_codir	2.15770562	0.00040675	0.00277189
ENSG0000007	MBNL3	protein_codir	2.15761582	1.41E-09	6.72E-08
ENSG0000017	CD163L1	protein_codir	2.15753248	8.97E-05	0.00079499
ENSG0000019	SCN8A	protein_codir	2.15747474	4.22E-05	0.00042391
ENSG0000020	AC021218.2	lncRNA	2.1563851	0.25375484	NA
ENSG0000027	LINC02772	lncRNA	2.15637038	0.26702878	NA
ENSG0000023	RP3-467L1.4	lncRNA	2.15631219	0.10817465	0.21399398
ENSG0000014	SPATA22	protein_codir	2.15603582	0.01554388	0.04897581
ENSG0000024	RP11-26J3.1	lncRNA	2.15598925	0.13103424	0.24666237
ENSG0000022	LINC02158	lncRNA	2.15386771	0.00615895	0.02382894
ENSG0000012	TNFSF10	protein_codir	2.15383111	1.11E-09	5.52E-08

ENSG0000025	RP1-317E23.6	protein_codir	2.15338907	0.23156218	0.37430488
ENSG0000023	DIAPH2-AS1	lncRNA	2.15311574	0.25974733	NA
ENSG0000022	LINC01013	lncRNA	2.15233352	4.69E-06	6.74E-05
ENSG0000023	LINC01353	lncRNA	2.15143482	0.00168346	0.00862975
ENSG0000012	SV2C	protein_codir	2.15107652	0.00437813	0.01827473
ENSG0000028	RP11-26A3.1	lncRNA	2.15105307	0.05847434	0.13525343
ENSG0000018	ZNF93	protein_codir	2.15095048	7.70E-09	2.82E-07
ENSG0000022	RP11-93B14.4	lncRNA	2.15082472	0.35159264	NA
ENSG0000016	NLRP4	protein_codir	2.15077657	0.04373832	0.10871601
ENSG0000016	CAMP	protein_codir	2.15037551	0.00153036	0.00800142
ENSG0000027	RP11-44M6.7	lncRNA	2.14956524	0.00010456	0.0009016
ENSG0000028	RP1-111D6.5	lncRNA	2.14926961	0.34562693	NA
ENSG0000011	CHRNB4	protein_codir	2.14926826	0.01301232	0.04266893
ENSG0000008	LAT2	protein_codir	2.14853358	1.58E-06	2.73E-05
ENSG0000007	SEMA3A	protein_codir	2.14827105	1.61E-05	0.00019103
ENSG0000022	TNK2-AS1	lncRNA	2.14802437	0.00228926	0.01093743
ENSG0000027	RP11-481J2.3	lncRNA	2.1480163	0.02571375	0.07218354
ENSG0000028	RP11-345F18.	lncRNA	2.14759064	2.25E-06	3.64E-05
ENSG0000027	RP1-278C19.8	lncRNA	2.14656927	0.08024793	0.17101959
ENSG0000019	SIRPB2	protein_codir	2.14563894	4.70E-05	0.00046453
ENSG0000013	KIAA0319	protein_codir	2.14554643	0.00026838	0.00196568
ENSG0000009	IGF2-AS	lncRNA	2.1453633	0.00282466	0.01286138
ENSG0000023	AP001056.1	lncRNA	2.14531841	1.08E-05	0.00013639
ENSG0000025	RP11-930P14.	lncRNA	2.14512277	0.24977948	NA
ENSG0000007	FRMPD1	protein_codir	2.14505848	0.0215345	0.06276731
ENSG0000025	LINC02237	lncRNA	2.14478263	0.25254589	NA
ENSG0000018	APOBR	protein_codir	2.14458684	2.85E-06	4.41E-05
ENSG0000024	RP13-635I23.3	lncRNA	2.14394669	0.19306742	NA
ENSG0000026	AC008982.2	lncRNA	2.14372261	0.00175502	0.00889262
ENSG0000008	TPX2	protein_codir	2.14292946	2.37E-05	0.00026398
ENSG0000026	TSPOAP1-AS1	lncRNA	2.14158417	2.25E-08	6.95E-07
ENSG0000022	DLG1-AS1	lncRNA	2.14111829	0.27044753	NA
ENSG0000028	RP11-91A18.6	protein_codir	2.14014931	0.26266916	0.41015076
ENSG0000015	GOLGA7B	protein_codir	2.1395397	2.96E-05	0.00031796
ENSG0000026	FAM72C	protein_codir	2.13927663	0.02075007	0.06101026
ENSG0000011	VEGFA	protein_codir	2.13889879	1.35E-05	0.00016563
ENSG0000023	RP11-431N15	lncRNA	2.13786181	0.09572637	0.19518399
ENSG0000017	PTGER4	protein_codir	2.13772971	4.00E-10	2.33E-08
ENSG0000002	PRKCH	protein_codir	2.13741385	2.11E-11	1.99E-09
ENSG0000022	AC020743.2	lncRNA	2.13717156	0.44192239	NA
ENSG0000018	HCLS1	protein_codir	2.13614581	1.31E-07	3.18E-06
ENSG0000010	COL1A1	protein_codir	2.13548952	2.14E-06	3.50E-05
ENSG0000027	TP53TG3F	protein_codir	2.13531082	0.2630683	NA
ENSG0000027	ENSG0000027	protein_codir	2.13531082	0.2630683	NA
ENSG0000025	RP11-11N5.1	lncRNA	2.13512409	0.14561243	0.26666325
ENSG0000016	ASGR2	protein_codir	2.13506493	2.98E-07	6.41E-06
ENSG0000016	E2F7	protein_codir	2.13501483	0.00012209	0.00103092

ENSG0000017	TTLL6	protein_codir	2.13490476	0.01341291	0.04368687
ENSG000001C	TLR8	protein_codir	2.13467006	0.00019823	0.00153408
ENSG0000023	HLA-DQB2	protein_codir	2.13356597	0.00047331	0.00312821
ENSG0000007	CYP2W1	protein_codir	2.13338307	0.26824645	0.41613199
ENSG0000012	IRF1	protein_codir	2.13259979	8.11E-11	5.96E-09
ENSG0000015	DEPTOR	protein_codir	2.13224135	6.24E-08	1.68E-06
ENSG0000027	PIK3R6	protein_codir	2.13223453	3.92E-07	8.16E-06
ENSG0000026	AC007952.4	lncRNA	2.13094495	0.09409167	0.1927759
ENSG000001C	DKK4	protein_codir	2.13050178	0.35448534	NA
ENSG0000016	ANPEP	protein_codir	2.13013143	3.47E-06	5.22E-05
ENSG0000024	SMKR1	protein_codir	2.12971075	0.00605037	0.02347397
ENSG0000001	CLDN11	protein_codir	2.12932333	0.00012161	0.00102747
ENSG0000023	RP5-1195D24	lncRNA	2.12888699	0.35605141	NA
ENSG0000026	GNA15-DT	lncRNA	2.12855158	0.05779842	0.13401548
ENSG0000014	SLC26A7	protein_codir	2.12657705	0.0032846	0.01453046
ENSG0000026	RP11-161I6.2	lncRNA	2.12657045	0.17727507	0.30781024
ENSG0000028	XXbac-BPG18	lncRNA	2.12645274	0.00156372	0.00814137
ENSG000001C	RUBCNL	protein_codir	2.1253279	1.46E-05	0.0001762
ENSG000000C	PGLYRP1	protein_codir	2.12530218	0.00858084	0.03082363
ENSG0000016	ICOSLG	protein_codir	2.12473677	3.71E-11	3.16E-09
ENSG0000028	RCAN3AS	lncRNA	2.12413048	0.12584152	0.23952074
ENSG0000028	RP11-809M12	lncRNA	2.12395366	0.20007357	0.33668628
ENSG0000022	LINC02561	lncRNA	2.1238502	0.03325634	0.08807556
ENSG0000013	PLXNC1	protein_codir	2.12378731	3.74E-07	7.85E-06
ENSG0000015	FREM2	protein_codir	2.12210496	0.00541402	0.02159043
ENSG0000028	RP11-667E17.	lncRNA	2.12181466	0.23468768	0.37807372
ENSG0000026	RP11-20B24.4	lncRNA	2.12159106	0.27388847	NA
ENSG0000002	GRAMD1B	protein_codir	2.12131193	1.35E-08	4.53E-07
ENSG0000017	EXO1	protein_codir	2.12128174	0.00254446	0.0118393
ENSG0000026	RP13-638C3.4	lncRNA	2.12121405	0.00436018	0.01821635
ENSG0000027	RP11-455F5.6	lncRNA	2.12014961	0.27404653	NA
ENSG0000022	FNDC10	protein_codir	2.11994464	2.29E-06	3.70E-05
ENSG0000027	LL22NC03-N1	lncRNA	2.11929096	0.00041749	0.00282417
ENSG0000025	MIR3180-4	lncRNA	2.11911978	0.20244144	0.33953319
ENSG0000013	RASGEF1B	protein_codir	2.1190789	7.75E-07	1.47E-05
ENSG0000011	ARID3A	protein_codir	2.11869425	1.26E-09	6.18E-08
ENSG0000013	GCH1	protein_codir	2.11867183	1.85E-09	8.34E-08
ENSG0000014	PKLR	protein_codir	2.11815593	0.02874566	0.07885145
ENSG0000022	LINC01825	lncRNA	2.1179249	0.0264106	0.07365316
ENSG0000028	GS1-273L24.6	protein_codir	2.11786411	0.12480071	0.2379457
ENSG0000016	CXCL8	protein_codir	2.11752126	0.00464668	0.01918977
ENSG0000005	TBXAS1	protein_codir	2.11673328	2.07E-07	4.67E-06
ENSG0000004	LCP2	protein_codir	2.11651298	2.50E-08	7.60E-07
ENSG0000023	RP11-135A1.2	lncRNA	2.1164688	0.11398224	0.22231043
ENSG0000024	LY75-CD302	protein_codir	2.11615649	0.03242927	0.08634051
ENSG0000018	CCL13	protein_codir	2.11534598	0.00032721	0.00231437
ENSG0000026	RP11-282O18	lncRNA	2.11523516	0.35776186	NA

ENSG0000026	CTD-2126E3.4	lncRNA	2.11518663	0.20101841	0.33786457
ENSG0000016	CX3CR1	protein_codir	2.1150146	0.00036189	0.00251709
ENSG0000023	AC074366.3	lncRNA	2.11487145	0.44177541	NA
ENSG0000025	RP11-662I13.:	lncRNA	2.11484321	0.25485313	NA
ENSG0000022	DISC1-IT1	lncRNA	2.11424813	0.26124973	NA
ENSG0000023	AC012314.8	lncRNA	2.11317178	0.00196356	0.00968454
ENSG0000013	SLC6A6	protein_codir	2.11233654	7.08E-09	2.62E-07
ENSG0000028	RP11-499F19.	lncRNA	2.11190196	0.00593179	0.02313089
ENSG0000023	RP11-406A20	lncRNA	2.11151743	0.22633866	0.36835523
ENSG0000016	CYP2S1	protein_codir	2.11109787	3.91E-06	5.79E-05
ENSG0000018	KNTC1	protein_codir	2.11098171	3.84E-09	1.56E-07
ENSG0000015	CLDN14	protein_codir	2.11069591	0.00045938	0.00305371
ENSG0000013	MNX1	protein_codir	2.11061158	0.18327757	NA
ENSG0000027	RP11-617F23.	lncRNA	2.11006459	0.00519923	0.02092723
ENSG0000026	CTB-180A7.6	lncRNA	2.10969223	0.0779007	0.1673629
ENSG0000017	KCNG3	protein_codir	2.10956621	0.03677346	0.0950925
ENSG0000026	RP11-98D18.1	lncRNA	2.10847741	0.443067	NA
ENSG0000026	VPS9D1-AS1	lncRNA	2.10846385	0.00132424	0.00715762
ENSG0000028	CH507-42P11	lncRNA	2.10819455	0.03001618	0.08151086
ENSG0000012	CSTA	protein_codir	2.10758147	1.68E-05	0.00019822
ENSG0000010	COTL1	protein_codir	2.10725202	1.76E-08	5.67E-07
ENSG0000011	TTK	protein_codir	2.10664182	0.00039204	0.0026922
ENSG0000027	RP11-326C3.1	lncRNA	2.10595726	0.00146224	0.00773549
ENSG0000011	MREG	protein_codir	2.10570495	2.44E-05	0.00027127
ENSG0000022	LHFPL3-AS2	lncRNA	2.10527555	0.00411643	0.0174407
ENSG0000014	MTFR2	protein_codir	2.10527429	9.86E-05	0.00085875
ENSG0000017	ALOX12B	protein_codir	2.10516212	0.14287854	0.2627892
ENSG0000015	RSPH10B	protein_codir	2.10475584	0.09194072	0.18930988
ENSG0000028	RP11-433K2.8	lncRNA	2.10467724	0.02917781	0.07979909
ENSG0000017	MARCKSL1	protein_codir	2.10430639	1.13E-07	2.79E-06
ENSG0000023	XXbac-B33L19	lncRNA	2.10389209	0.36024616	NA
ENSG0000008	GMIP	protein_codir	2.10377521	1.28E-07	3.10E-06
ENSG0000027	RP11-157L3.1	lncRNA	2.10293375	0.04070343	0.10281952
ENSG0000016	TMEM71	protein_codir	2.10248511	6.24E-12	7.09E-10
ENSG0000027	RP11-408O19	lncRNA	2.1023314	0.01425302	0.04582061
ENSG0000027	RP11-761I4.5	lncRNA	2.10212112	0.129927	0.2450047
ENSG0000015	KCNK13	protein_codir	2.10200228	0.00044188	0.0029573
ENSG0000017	DIRC1	lncRNA	2.1016999	0.35753138	NA
ENSG0000010	NFATC2	protein_codir	2.10159517	6.95E-07	1.34E-05
ENSG0000015	CENPU	protein_codir	2.10153421	3.76E-05	0.0003859
ENSG0000016	CLDN1	protein_codir	2.10153192	0.00067757	0.00419091
ENSG0000013	THBS1	protein_codir	2.1011671	0.00012669	0.00106359
ENSG0000027	LLNLR-260G6.	lncRNA	2.10094174	0.27756237	NA
ENSG0000022	LINC01624	lncRNA	2.10055596	0.00366705	0.01583553
ENSG0000010	LFNG	protein_codir	2.10053838	6.36E-09	2.40E-07
ENSG0000017	CHD7	protein_codir	2.10022759	3.47E-09	1.43E-07
ENSG0000023	TMEM238	protein_codir	2.10020738	3.02E-05	0.00032255

ENSG0000011	ARG1	protein_codir	2.09970998	0.01935802	0.05787479
ENSG0000022	EPS15-AS1	lncRNA	2.09931115	0.18573743	0.31901422
ENSG0000007	CACNG5	protein_codir	2.09922071	0.02744964	0.0759146
ENSG0000018	SORCS2	protein_codir	2.0987494	8.10E-08	2.11E-06
ENSG0000025	PRODH2	protein_codir	2.09828532	0.36121487	NA
ENSG0000012	KIRREL2	protein_codir	2.09735635	0.01238158	0.04104409
ENSG0000016	TRPV3	protein_codir	2.09708036	0.00021581	0.00164067
ENSG0000018	FOXD4L4	protein_codir	2.09684626	0.07311849	0.16005172
ENSG0000025	RP11-611O2.5	lncRNA	2.09647417	0.00502225	0.02033938
ENSG0000019	GREB1	protein_codir	2.09643584	0.0022301	0.01070853
ENSG0000000	CASP10	protein_codir	2.09622516	6.39E-07	1.25E-05
ENSG0000024	RP4-753P9.3	lncRNA	2.09616673	0.06522811	0.14741841
ENSG0000028	SCYGR4	protein_codir	2.09615255	0.44462573	NA
ENSG0000016	CXCR1	protein_codir	2.09570986	0.00743084	0.02759342
ENSG0000015	TMED6	protein_codir	2.09483977	0.04614001	0.11322006
ENSG0000015	PIK3AP1	protein_codir	2.09459452	2.29E-06	3.70E-05
ENSG0000010	CTSH	protein_codir	2.0934677	5.41E-08	1.48E-06
ENSG0000023	NR2F1-AS1	lncRNA	2.09305451	3.05E-05	0.00032454
ENSG0000015	DPYSL5	protein_codir	2.09263348	0.28958001	0.43917196
ENSG0000013	DTX1	protein_codir	2.09167899	0.00015349	0.00124798
ENSG0000027	RP11-367H1.1	lncRNA	2.09147845	0.19078087	0.32537003
ENSG0000010	CALB1	protein_codir	2.09116647	0.51979519	NA
ENSG0000026	CTD-2659N19	lncRNA	2.09101391	0.10693775	0.21215845
ENSG0000024	RP11-231L11.	lncRNA	2.09047837	0.36203684	NA
ENSG0000023	AC010148.1	lncRNA	2.09042967	0.15952705	0.28509461
ENSG0000014	PLK4	protein_codir	2.09036663	1.33E-07	3.21E-06
ENSG0000026	AC005606.14	lncRNA	2.09032611	0.04547241	0.11209816
ENSG0000017	SOX11	protein_codir	2.09032325	0.01727467	0.05300816
ENSG0000027	RP11-26J3.4	protein_codir	2.08993441	0.2437042	0.38878935
ENSG0000015	TMEM45B	protein_codir	2.08928923	0.00648017	0.02483106
ENSG0000013	CA6	protein_codir	2.08905721	0.36955811	NA
ENSG0000024	LINC02234	lncRNA	2.08873377	0.10676445	0.21191765
ENSG0000026	CTD-2047H16	lncRNA	2.08819049	0.01253839	0.0414445
ENSG0000023	AC012360.6	lncRNA	2.08778766	0.04422224	0.10966225
ENSG0000028	RP11-663P9.3	lncRNA	2.0873846	0.2539589	0.4003393
ENSG0000018	EPHA10	protein_codir	2.08686259	0.00069875	0.00429643
ENSG0000028	RP11-495F22.	lncRNA	2.08626365	0.00259025	0.01200581
ENSG0000016	ITGB2	protein_codir	2.08588606	3.48E-06	5.24E-05
ENSG0000026	RP11-367F23.	lncRNA	2.08505191	0.10384864	0.20756277
ENSG0000014	ZNF215	protein_codir	2.08481225	1.85E-05	0.00021431
ENSG0000018	H3-5	protein_codir	2.08468749	0.00715527	0.0267572
ENSG0000026	KLF2-DT	lncRNA	2.08461982	0.07780771	0.16721514
ENSG0000010	EYA1	protein_codir	2.0843494	0.00180857	0.00908727
ENSG0000025	PCAT1	lncRNA	2.08398289	0.00035439	0.00247429
ENSG0000010	BDKRB1	protein_codir	2.08395909	0.00288268	0.0130717
ENSG0000028	RP11-144G7.4	lncRNA	2.08301104	0.07077517	0.15632229
ENSG0000016	SPATA48	protein_codir	2.08275554	0.45577023	NA

ENSG0000026	RP1-118J21.2	lncRNA	2.08257462	0.13332625	0.24986232
ENSG0000017	FRMPD2	protein_codir	2.08246564	0.10253813	0.20565705
ENSG0000011	C9	protein_codir	2.08211857	0.05869764	0.13562228
ENSG0000014	CYP11A1	protein_codir	2.08211348	0.00020644	0.00158559
ENSG0000015	RASGRP3	protein_codir	2.08187704	8.79E-08	2.26E-06
ENSG0000009	ORC6	protein_codir	2.08173404	7.89E-05	0.00071477
ENSG0000015	ARHGAP27	protein_codir	2.08172945	1.29E-08	4.35E-07
ENSG0000016	CFAP20DC	protein_codir	2.08136983	1.22E-05	0.00015131
ENSG0000026	CTB-61M7.2	lncRNA	2.08124524	0.05460635	0.12854577
ENSG0000028	RP3-468B3.6	lncRNA	2.07937926	0.1536122	NA
ENSG0000020	LINC01993	lncRNA	2.07930049	0.04658047	0.11401565
ENSG0000022	LINC02829	lncRNA	2.07919288	0.01103785	0.03765164
ENSG0000012	NCKAP1L	protein_codir	2.07914095	1.69E-08	5.47E-07
ENSG0000021	TTTY2B	lncRNA	2.0789562	0.36066978	NA
ENSG0000022	RFPL4A	protein_codir	2.07891104	0.08411478	0.17706351
ENSG0000028	RP11-419I17.1	lncRNA	2.07882106	0.00013642	0.0011325
ENSG0000011	TNFSF4	protein_codir	2.07865944	8.93E-05	0.00079257
ENSG0000027	RP11-354K4.2	lncRNA	2.07849869	0.52236291	NA
ENSG0000028	RP11-127I20.9	lncRNA	2.07849869	0.52236291	NA
ENSG0000028	RP11-195B3.3	lncRNA	2.07809338	0.52244517	NA
ENSG0000025	RP11-256L6.3	lncRNA	2.07775855	0.13366742	0.25031487
ENSG0000013	VAV3	protein_codir	2.07691128	2.02E-06	3.33E-05
ENSG0000006	CD84	protein_codir	2.07683287	2.58E-05	0.00028337
ENSG0000018	CAV3	protein_codir	2.07626518	0.16300824	0.28959204
ENSG0000026	CGB1	protein_codir	2.07605973	0.45471029	NA
ENSG0000021	BCRP3	lncRNA	2.07566686	0.04537607	0.11191061
ENSG0000027	AP001429.1	lncRNA	2.07527972	0.01705179	0.05251208
ENSG0000020	FAM153CP	lncRNA	2.07461744	0.0017814	0.00898999
ENSG0000026	RP11-874J12.4	lncRNA	2.0744547	0.45751329	NA
ENSG0000010	CACNG7	protein_codir	2.074393	0.1826811	0.31507755
ENSG0000023	RP1-8B1.4	lncRNA	2.07400645	0.01101297	0.0375993
ENSG0000027	H2BC7	protein_codir	2.07329161	0.01105161	0.03768928
ENSG0000022	HSD11B1-AS1	lncRNA	2.07263612	0.00167032	0.00857195
ENSG0000024	BGLAP	protein_codir	2.07262557	0.00222258	0.01067613
ENSG0000016	HTRA4	protein_codir	2.07239578	0.00321603	0.01429
ENSG0000028	CH17-420I24.	lncRNA	2.07155924	0.45828606	NA
ENSG0000022	RP11-371A19	lncRNA	2.07063193	0.05930272	0.13668875
ENSG0000025	KB-1980E6.3	lncRNA	2.06946175	0.02053862	0.06050305
ENSG0000010	ATP6V0A4	protein_codir	2.06934072	0.27497004	NA
ENSG0000026	PRR29-AS1	lncRNA	2.06889267	0.00285217	0.01295461
ENSG0000016	DNASE1L3	protein_codir	2.06838278	8.06E-05	0.00072882
ENSG0000027	RP5-1009E24.	lncRNA	2.06786486	0.09331274	0.19152098
ENSG0000021	C20orf202	protein_codir	2.06756881	0.00043454	0.00291525
ENSG0000026	LINC01917	lncRNA	2.06678081	0.35440639	NA
ENSG0000017	LKAAEAR1	protein_codir	2.06652079	0.00437672	0.01827473
ENSG0000014	MYO1F	protein_codir	2.06636162	4.41E-06	6.37E-05
ENSG0000023	LINC00299	lncRNA	2.06635125	9.72E-05	0.00084768

ENSG0000025	RP11-268P4.4	lncRNA	2.06625069	0.45145481	NA
ENSG0000015	KCNJ15	protein_codir	2.06620534	2.74E-05	0.00029757
ENSG0000010	TRIM14	protein_codir	2.06511748	1.28E-05	0.00015784
ENSG0000016	GBP4	protein_codir	2.06449953	4.77E-08	1.33E-06
ENSG0000018	EMID1	protein_codir	2.06298016	9.65E-06	0.00012473
ENSG0000025	LINC00603	lncRNA	2.0628674	0.09252481	0.19027141
ENSG0000011	BACH2	protein_codir	2.06145717	1.92E-05	0.00022189
ENSG0000016	VPREB1	protein_codir	2.06105653	0.36196932	NA
ENSG0000016	TRIM58	protein_codir	2.0608414	0.00013029	0.00108822
ENSG0000017	ZNF804A	protein_codir	2.05925009	1.75E-06	2.96E-05
ENSG0000016	EOMES	protein_codir	2.05912079	0.00077404	0.00465921
ENSG0000010	TUSC3	protein_codir	2.05877326	3.84E-07	8.02E-06
ENSG0000013	CCDC65	protein_codir	2.05843863	0.00065708	0.00408752
ENSG0000026	RP11-44F14.8	lncRNA	2.05814508	0.01540213	0.04865617
ENSG0000018	SLC18A3	protein_codir	2.05770759	0.46213192	0.61221563
ENSG0000012	SEPTIN6	protein_codir	2.05720771	4.23E-09	1.68E-07
ENSG0000016	CXCL3	protein_codir	2.05703	0.00398992	0.01697498
ENSG0000011	PLCH1	protein_codir	2.0569774	0.00116089	0.00643737
ENSG0000007	TPD52	protein_codir	2.05669332	5.40E-07	1.07E-05
ENSG0000025	CTD-2547L24.	lncRNA	2.05635043	4.23E-05	0.0004242
ENSG0000017	FLJ13224	lncRNA	2.05553284	0.0374925	0.09644974
ENSG0000025	RP11-175P13.	lncRNA	2.05531862	0.07326792	0.16030259
ENSG0000024	PCDHAC1	protein_codir	2.05492281	0.00176878	0.00894014
ENSG0000016	ERICH6	protein_codir	2.05453418	0.05540897	0.1299022
ENSG0000001	PLAUR	protein_codir	2.05443454	1.06E-06	1.93E-05
ENSG0000010	NEURL1	protein_codir	2.05247187	5.11E-05	0.00050052
ENSG0000013	FST	protein_codir	2.05239246	1.16E-06	2.09E-05
ENSG0000024	KLRK1-AS1	lncRNA	2.05236437	0.0005068	0.00330448
ENSG0000018	OLIG1	protein_codir	2.05146258	0.0185704	0.05603813
ENSG0000023	LINC01522	lncRNA	2.0505878	0.04752203	0.11576631
ENSG0000009	MADCAM1	protein_codir	2.04777429	0.00188472	0.00937099
ENSG0000016	GDNF	protein_codir	2.04774158	6.02E-05	0.00057421
ENSG0000026	RP11-802D6.1	lncRNA	2.04672322	0.09492913	0.19403113
ENSG0000018	FPR3	protein_codir	2.04634554	7.42E-05	0.00068048
ENSG0000028	RP11-740N7.7	lncRNA	2.04632268	0.36726477	NA
ENSG0000007	FCGR2B	protein_codir	2.04583088	6.33E-07	1.24E-05
ENSG0000016	CTSS	protein_codir	2.04532181	9.93E-06	0.00012753
ENSG0000028	RP11-109E12.	lncRNA	2.04524064	0.28267767	NA
ENSG0000022	PACRG-AS3	lncRNA	2.04523598	0.37350036	NA
ENSG0000009	RPGRIP1	protein_codir	2.04445066	7.28E-06	9.84E-05
ENSG0000010	ABI3	protein_codir	2.0444484	3.39E-09	1.41E-07
ENSG0000016	TMEM145	protein_codir	2.04431561	0.00305908	0.01371399
ENSG0000008	RDH8	protein_codir	2.0437577	0.36999142	NA
ENSG0000018	CD300LF	protein_codir	2.04319475	0.00081542	0.00486542
ENSG0000028	CTA-150C2.22	protein_codir	2.04318751	0.04705669	0.11484544
ENSG0000010	ELAVL2	protein_codir	2.04229204	0.00098928	0.00568215
ENSG0000015	CD1D	protein_codir	2.04224817	1.11E-06	2.01E-05

ENSG0000024	CTD-2313F11.	lncRNA	2.04137125	0.10340548	0.20689258
ENSG0000002	RNASET2	protein_codir	2.04085163	1.22E-08	4.16E-07
ENSG0000028	RP11-315A19	protein_codir	2.04045326	0.20224926	0.33931389
ENSG0000013	GOLM1	protein_codir	2.04027682	3.78E-07	7.91E-06
ENSG0000016	COL22A1	protein_codir	2.04004983	5.34E-05	0.00051972
ENSG0000012	HCST	protein_codir	2.03984936	2.75E-07	5.99E-06
ENSG0000017	PPP1R42	protein_codir	2.03926141	0.17519099	0.30530704
ENSG0000028	CTA-396D5.2	lncRNA	2.03859972	0.37167773	NA
ENSG0000015	MPZ	protein_codir	2.03814594	0.00373826	0.01609271
ENSG0000023	AC005537.2	lncRNA	2.03772222	0.1758285	0.30606815
ENSG0000027	RP13-753N3.3	lncRNA	2.03753204	0.02685047	0.07461615
ENSG0000014	GPR65	protein_codir	2.03742073	9.67E-06	0.00012494
ENSG0000016	IL6R	protein_codir	2.03726448	2.25E-09	9.80E-08
ENSG0000018	MFSD6L	protein_codir	2.036996	0.01816928	0.05504997
ENSG0000026	RP11-373L24.	lncRNA	2.03655917	0.00062348	0.00391286
ENSG0000013	CENPE	protein_codir	2.03620921	1.62E-07	3.80E-06
ENSG0000027	RP11-349F21.	lncRNA	2.03612117	0.08729222	0.18207303
ENSG0000014	NLRC5	protein_codir	2.03529828	8.26E-09	2.99E-07
ENSG0000027	RP11-93B14.9	lncRNA	2.03410194	1.96E-08	6.15E-07
ENSG0000018	PLAG1	protein_codir	2.03386941	2.06E-07	4.65E-06
ENSG0000010	MFNG	protein_codir	2.03320818	1.21E-06	2.15E-05
ENSG0000022	SFTA1P	lncRNA	2.03242068	0.00347326	0.01518142
ENSG0000024	MTFP1	protein_codir	2.03133842	1.17E-10	8.03E-09
ENSG0000019	NTNG2	protein_codir	2.03069324	4.14E-09	1.66E-07
ENSG0000007	STXBP2	protein_codir	2.02986305	6.98E-07	1.35E-05
ENSG0000028	RP11-736I24.5	protein_codir	2.02957872	0.00040127	0.00274133
ENSG0000026	RP11-667K14.	lncRNA	2.02862345	0.1602248	NA
ENSG0000022	RP11-538D16	lncRNA	2.02860329	0.46738944	NA
ENSG0000028	RP11-271K21.	protein_codir	2.02801243	0.21711021	NA
ENSG0000024	LINC00616	lncRNA	2.02788475	0.53268542	NA
ENSG0000023	AC021188.4	lncRNA	2.02642678	0.00212001	0.01027456
ENSG0000016	CD300LG	protein_codir	2.02620597	0.0001887	0.00147101
ENSG0000026	RP11-473M20	lncRNA	2.02606363	9.58E-05	0.00083885
ENSG0000027	RP11-946L16.	lncRNA	2.02573536	0.37756968	NA
ENSG0000028	RP11-736N17	lncRNA	2.0245687	0.22095617	0.36177659
ENSG0000010	GINS1	protein_codir	2.02433794	3.61E-06	5.39E-05
ENSG0000025	RP11-56G10.2	lncRNA	2.02399167	0.10031367	0.20217919
ENSG0000025	RP11-119H12	lncRNA	2.02362845	0.3209342	0.47308963
ENSG0000002	INSRR	protein_codir	2.02290509	0.00021132	0.00161548
ENSG0000027	RP11-457M11	lncRNA	2.02283337	0.09634082	0.19613277
ENSG0000018	CERKL	protein_codir	2.02164905	0.01241085	0.04112135
ENSG0000028	RP3-340B19.7	lncRNA	2.02149337	0.46582511	NA
ENSG0000011	TULP1	protein_codir	2.02117079	0.11592008	0.22512394
ENSG0000027	RP11-415F23.	lncRNA	2.02017439	0.00186598	0.00929958
ENSG0000009	ADA2	protein_codir	2.02010775	3.14E-05	0.00033302
ENSG0000016	LGALS9	protein_codir	2.02008551	1.73E-07	3.99E-06
ENSG0000020	PDCD4-AS1	lncRNA	2.02004738	7.63E-08	2.00E-06

ENSG0000027	CTD-2536I1.3	lncRNA	2.0190117	0.01107322	0.03773503
ENSG0000026	CTB-102L5.8	lncRNA	2.01884044	0.13449533	0.25143908
ENSG0000016	GNGT2	protein_codir	2.01883818	1.68E-05	0.00019831
ENSG0000023	RP1-232L24.3	lncRNA	2.01865903	0.28456694	NA
ENSG0000028	RP11-527L4.5	lncRNA	2.01843534	0.06867885	0.15281741
ENSG0000026	LINC01801	lncRNA	2.0170758	0.00073155	0.00445936
ENSG0000010	STEAP1B	protein_codir	2.01610358	3.21E-06	4.91E-05
ENSG0000025	RP11-44N11.1	lncRNA	2.01599602	0.01160645	0.03911299
ENSG0000016	RASEF	protein_codir	2.01583388	0.00107414	0.0060608
ENSG0000018	OR51E1	protein_codir	2.01560852	0.00013079	0.00109151
ENSG0000013	GINS2	protein_codir	2.01544414	0.00045043	0.0030014
ENSG0000016	CADM3	protein_codir	2.01378514	6.23E-07	1.22E-05
ENSG0000023	LINC02090	lncRNA	2.0137684	0.3677768	NA
ENSG0000023	CTA-992D9.7	lncRNA	2.0136902	0.37222209	NA
ENSG0000027	RP1-197B17.4	lncRNA	2.01241296	0.01944516	0.0580622
ENSG0000022	RABGAP1L-IT1	lncRNA	2.01231797	0.11216232	0.21982051
ENSG0000019	SERPINA1	protein_codir	2.01228131	0.00015531	0.00125978
ENSG0000010	XBP1	protein_codir	2.0106158	2.13E-07	4.78E-06
ENSG0000026	CTC-479C5.12	protein_codir	2.0104109	8.78E-06	0.00011541
ENSG0000027	ZNF280B	protein_codir	2.00948336	1.42E-05	0.00017266
ENSG0000026	RP5-837J1.4	lncRNA	2.00932056	0.11369461	0.22188736
ENSG0000027	RP5-827C21.6	lncRNA	2.00908244	0.17374286	0.30368625
ENSG0000018	UMODL1-AS1	lncRNA	2.00863838	0.13784051	0.25575406
ENSG0000019	KCNMB2	protein_codir	2.0072025	0.08025289	0.17101959
ENSG0000018	QRFP	protein_codir	2.00685027	0.0890403	0.18477033
ENSG0000014	FANCI	protein_codir	2.00627299	3.55E-08	1.03E-06
ENSG0000021	LINC01356	lncRNA	2.00618733	0.08690605	0.18154985
ENSG0000028	RP5-1136G13	lncRNA	2.00552668	0.53727716	NA
ENSG0000024	RP11-635L1.3	lncRNA	2.00544936	0.18472224	0.31778438
ENSG0000022	AP4B1-AS1	lncRNA	2.0051438	0.21346941	NA
ENSG0000026	RP11-192H23	lncRNA	2.004018	0.02831233	0.07789486
ENSG0000015	CYSLTR2	protein_codir	2.0033398	8.50E-06	0.00011257
ENSG0000025	RP11-293M10	lncRNA	2.00324191	0.38265667	NA
ENSG0000016	TSPY2	protein_codir	2.0028592	0.38100977	NA
ENSG0000021	KRTAP4-11	protein_codir	2.00216765	0.53796869	NA
ENSG0000000	SLC22A16	protein_codir	2.00214856	0.00699609	0.02633305
ENSG0000011	VNN1	protein_codir	2.00183228	2.94E-05	0.00031699
ENSG0000015	JPH3	protein_codir	2.00165057	0.00819453	0.02977674
ENSG0000027	DGKK	protein_codir	2.00139626	0.22928925	0.37178402
ENSG0000023	LINC01381	lncRNA	2.00124029	0.29986369	NA
ENSG0000027	RP11-338N10	lncRNA	2.00063462	0.17917459	0.31036702
ENSG0000011	IL1A	protein_codir	2.0003158	0.00640314	0.02458363
ENSG0000022	RP11-383B4.4	lncRNA	1.99998834	0.22955035	0.37203267
ENSG0000016	MUCL3	protein_codir	1.99962086	0.11212628	0.21979615
ENSG0000010	GRIN2D	protein_codir	1.9993079	2.04E-07	4.62E-06
ENSG0000024	CTC-340A15.2	lncRNA	1.99926128	0.07768665	0.16705897
ENSG0000025	RP11-1149M1	lncRNA	1.99908726	0.00289033	0.01309777

ENSG0000016	GRM2	protein_codir	1.99902803	9.69E-08	2.45E-06
ENSG0000025	CTD-2653D5.1	lncRNA	1.99860261	0.17259317	NA
ENSG0000013	ASCL1	protein_codir	1.99849071	0.01043483	0.03601276
ENSG0000013	CFHR4	protein_codir	1.99735232	0.38797944	0.54102045
ENSG0000027	RP13-1016M1	lncRNA	1.99712305	0.00941869	0.03321148
ENSG0000008	AURKA	protein_codir	1.9966516	2.01E-06	3.33E-05
ENSG0000028	RP11-505D17	lncRNA	1.9961596	0.06957677	0.15431707
ENSG0000017	SCUBE2	protein_codir	1.99614337	1.43E-05	0.00017388
ENSG0000018	LILRB4	protein_codir	1.99481371	0.00021666	0.00164582
ENSG0000027	RP11-182L21.	lncRNA	1.99446908	0.29999432	0.45096487
ENSG0000021	AP1G2	protein_codir	1.99401343	2.24E-06	3.63E-05
ENSG0000023	DPP4-DT	lncRNA	1.99311402	0.00999532	0.03479151
ENSG0000025	RP11-561P12.	lncRNA	1.99264619	0.30063469	NA
ENSG0000028	GS1-53I10.1	lncRNA	1.99206256	0.38359233	NA
ENSG0000017	FUT2	protein_codir	1.99192619	0.01666195	0.05161542
ENSG0000025	LINC01511	lncRNA	1.99187854	0.29864935	NA
ENSG0000023	LINC02518	lncRNA	1.99156769	0.04576938	0.11252289
ENSG0000023	AC018890.6	lncRNA	1.99051855	0.38336464	NA
ENSG0000016	FEV	protein_codir	1.98995927	0.10832522	0.21423046
ENSG0000010	KRT23	protein_codir	1.989686	0.01895094	0.05691257
ENSG0000013	NTS	protein_codir	1.9894313	NA	NA
ENSG0000018	EDDM3A	protein_codir	1.98906058	0.47121089	NA
ENSG0000022	LINC01483	lncRNA	1.98856673	0.29054552	NA
ENSG0000021	RP1-29C18.9	lncRNA	1.98826087	0.00126536	0.00689877
ENSG0000018	OR3A1	protein_codir	1.98804503	0.28031011	0.42943925
ENSG0000027	LA16c-359F1.	lncRNA	1.98780515	0.24110783	0.38571667
ENSG0000010	SLC8A3	protein_codir	1.98778595	7.46E-05	0.00068326
ENSG0000025	RP11-574O7.1	lncRNA	1.98767633	0.02396182	0.06825054
ENSG0000028	HELLPAR	lncRNA	1.98711569	0.00168637	0.00863987
ENSG0000026	RP5-1052I5.2	protein_codir	1.98649611	0.01698885	0.0523755
ENSG0000024	RP11-384F7.1	lncRNA	1.98640499	0.23909513	NA
ENSG0000016	RP11-25K19.1	lncRNA	1.98599633	0.00018829	0.00146905
ENSG0000024	CTC-338M12.	lncRNA	1.98542236	0.08647039	0.18094644
ENSG0000018	C6orf132	protein_codir	1.98541995	4.45E-07	9.07E-06
ENSG0000024	RP4-714D9.2	lncRNA	1.98437688	0.18596416	0.31926468
ENSG0000021	LINC01588	lncRNA	1.98423157	1.28E-05	0.00015797
ENSG0000025	RP11-794P6.1	lncRNA	1.98422901	0.26313733	0.41053177
ENSG0000008	SLC4A11	protein_codir	1.98409485	0.00054973	0.0035335
ENSG0000013	GIMAP4	protein_codir	1.98403576	1.13E-06	2.05E-05
ENSG0000015	RHBDL2	protein_codir	1.98350397	0.00017222	0.00137237
ENSG0000013	CALY	protein_codir	1.98282157	0.00338321	0.01487017
ENSG0000026	RP11-439E19.	lncRNA	1.98223571	3.54E-05	0.00036703
ENSG0000023	LEF1-AS1	lncRNA	1.98198156	0.01142822	0.03865859
ENSG0000025	CTD-2184C24	lncRNA	1.98092155	0.25915534	0.4062381
ENSG0000011	LY86	protein_codir	1.98073419	6.73E-05	0.00062837
ENSG0000028	CTD-2325P2.6	lncRNA	1.98014909	0.23780215	0.38189442
ENSG0000018	EDARADD	protein_codir	1.98009707	5.42E-06	7.63E-05

ENSG0000025	LINCR-0001	lncRNA	1.97973494	0.00087456	0.00514602
ENSG0000027	ANKRD20A3P	protein_codir	1.97957852	0.02618317	0.07318145
ENSG0000026	RP11-344E13.	lncRNA	1.9785168	0.48174303	0.62935118
ENSG0000010	CYTH1	protein_codir	1.9779461	4.22E-10	2.44E-08
ENSG0000017	PIK3CD	protein_codir	1.97714471	1.84E-08	5.87E-07
ENSG0000027	FOXD4L6	protein_codir	1.97650901	0.17869034	0.30970301
ENSG0000025	CTD-3051D23	lncRNA	1.97648664	0.35568375	0.50928232
ENSG0000025	RP11-238K6.1	lncRNA	1.97587977	0.25362178	0.39994481
ENSG0000028	RP1-37E16.13	protein_codir	1.97557905	0.08726486	0.18204621
ENSG0000022	RP4-758J18.7	lncRNA	1.97527203	0.00653834	0.02498117
ENSG0000023	SPATA3-AS1	lncRNA	1.97422287	0.47475013	NA
ENSG0000023	RP4-669P10.1	lncRNA	1.97372423	0.03425162	0.09014099
ENSG0000023	APOBEC3G	protein_codir	1.97342767	3.10E-06	4.75E-05
ENSG0000027	RP11-589C21.	lncRNA	1.97333199	0.38862567	NA
ENSG0000023	RP11-46F15.2	lncRNA	1.97313831	0.07736415	0.16654159
ENSG0000011	RPE65	protein_codir	1.97307787	0.07553066	0.16378714
ENSG0000026	CTD-2587H19	lncRNA	1.97302672	0.24286277	NA
ENSG0000022	RP4-583P15.1	lncRNA	1.97288497	0.03778309	0.09704008
ENSG0000025	PWRN1	lncRNA	1.97262231	0.16869301	0.29709937
ENSG0000006	NAV3	protein_codir	1.97228663	3.08E-09	1.30E-07
ENSG0000013	LSP1	protein_codir	1.97189212	3.35E-08	9.83E-07
ENSG0000010	EFNB3	protein_codir	1.97166505	0.00090677	0.00529604
ENSG0000020	CD300LD	protein_codir	1.97132663	0.13909714	0.25772207
ENSG0000010	ACSBG1	protein_codir	1.9704123	0.02808572	0.07738702
ENSG0000010	RNF125	protein_codir	1.97026592	7.08E-07	1.36E-05
ENSG0000023	AC073043.1	lncRNA	1.96780272	0.03162687	0.08473511
ENSG0000014	SLC39A12	protein_codir	1.96753901	0.04421617	0.10966225
ENSG0000027	RP11-1070N1	lncRNA	1.9675183	0.30368028	NA
ENSG0000023	EPN2-AS1	lncRNA	1.9674121	0.31248038	NA
ENSG0000021	TTLL13P	protein_codir	1.96711308	0.01123321	0.03814083
ENSG0000011	OAS2	protein_codir	1.96673848	3.10E-05	0.00032911
ENSG0000017	NETO2	protein_codir	1.96665685	1.35E-06	2.36E-05
ENSG0000018	VMO1	protein_codir	1.96638492	0.00135221	0.00726931
ENSG0000011	VAMP8	protein_codir	1.96634553	4.05E-07	8.37E-06
ENSG0000018	P2RY13	protein_codir	1.96624764	0.00079907	0.00478439
ENSG0000012	FOSB	protein_codir	1.9658971	0.00258364	0.01198122
ENSG0000021	IFI30	protein_codir	1.96546618	2.70E-05	0.00029415
ENSG0000018	GKN2	protein_codir	1.96468519	0.29996234	NA
ENSG0000014	ROPN1L	protein_codir	1.96410976	0.00192003	0.0095072
ENSG0000022	SPAG5-AS1	lncRNA	1.96335798	0.00012494	0.00105077
ENSG0000023	LINC02596	lncRNA	1.96281093	0.39114245	NA
ENSG0000017	ODF3B	protein_codir	1.96279871	1.73E-07	3.99E-06
ENSG0000001	SLC7A14	protein_codir	1.96255579	0.05642906	0.13169164
ENSG0000021	EML6	protein_codir	1.96240547	0.00018319	0.00143905
ENSG0000015	VWA5B1	protein_codir	1.96159242	0.13430758	0.2511901
ENSG0000016	LAIR2	protein_codir	1.96151813	0.00769918	0.02834595
ENSG0000013	CHL1	protein_codir	1.96086239	0.00022692	0.00171059

ENSG0000015	M1AP	protein_codir	1.96065982	0.00013681	0.00113476
ENSG0000015	PDK1	protein_codir	1.96028342	6.45E-09	2.42E-07
ENSG0000019	PTPRT	protein_codir	1.95934165	0.01209729	0.0403389
ENSG0000019	MPEG1	protein_codir	1.95919954	2.31E-05	0.00025876
ENSG0000025	RP11-187O7.3	lncRNA	1.95911245	0.09957619	0.20113313
ENSG0000016	TAMALIN	protein_codir	1.95872721	6.10E-08	1.65E-06
ENSG0000013	GNAT2	protein_codir	1.95849111	0.07379861	0.16109362
ENSG0000026	RP4-536B24.4	lncRNA	1.95716645	0.10638256	0.21137946
ENSG0000021	LINC00189	lncRNA	1.95688752	4.92E-05	0.00048422
ENSG0000023	AP001604.3	lncRNA	1.95684044	0.24308268	0.3880668
ENSG0000025	RP11-981P6.1	lncRNA	1.95675486	0.0151125	0.04796681
ENSG0000015	AGRP	protein_codir	1.95672299	0.38556195	NA
ENSG0000025	SPON1-AS1	lncRNA	1.95658925	0.39924306	NA
ENSG0000025	RP11-651L5.2	lncRNA	1.95547492	0.25447664	NA
ENSG0000003	DEPDC1B	protein_codir	1.95364015	0.00043871	0.00293747
ENSG0000028	RP11-513H8.2	lncRNA	1.95321416	0.02127327	0.06215017
ENSG0000023	RP3-527G5.1	lncRNA	1.95305891	0.17575967	0.30602547
ENSG0000019	OR2L13	protein_codir	1.95195363	0.18729205	0.32084639
ENSG0000008	P2RX7	protein_codir	1.95166505	6.22E-05	0.00058867
ENSG0000018	C11orf87	protein_codir	1.95137336	0.0497678	0.12009319
ENSG0000016	GRIK3	protein_codir	1.95027722	0.00165436	0.00851377
ENSG0000025	LINC02328	lncRNA	1.95002124	0.00016403	0.00132235
ENSG0000028	RP3-528L19.2	lncRNA	1.95001415	0.39893617	NA
ENSG0000023	UNQ6494	lncRNA	1.94963533	0.10829377	0.21419894
ENSG0000010	BMF	protein_codir	1.94922763	2.69E-07	5.88E-06
ENSG0000002	TNFRSF1B	protein_codir	1.94917374	9.59E-09	3.40E-07
ENSG0000014	VASH2	protein_codir	1.94867694	5.36E-06	7.54E-05
ENSG0000018	ZNF385C	protein_codir	1.94678326	1.50E-05	0.00018081
ENSG0000017	LGALS9C	protein_codir	1.94584034	0.06086069	0.1394882
ENSG0000024	LINC02065	lncRNA	1.94493651	0.48522032	NA
ENSG0000023	GLCCI1-DT	lncRNA	1.94489026	0.48608028	NA
ENSG0000026	RP11-322E11.	lncRNA	1.94431827	0.0039005	0.01666421
ENSG0000027	RP11-561I11.4	lncRNA	1.94420416	0.3052071	NA
ENSG0000014	CDH18	protein_codir	1.94397931	0.32538863	NA
ENSG0000028	KB-2007G4.1	lncRNA	1.94386493	0.04093543	0.1033111
ENSG0000019	SULT1A2	protein_codir	1.94375332	0.00029724	0.00213688
ENSG0000026	RP11-75C10.7	lncRNA	1.9436003	0.01932212	0.05780103
ENSG0000022	RP3-395M20.	lncRNA	1.94295324	0.55021884	NA
ENSG0000015	ELMO1	protein_codir	1.9423136	2.74E-09	1.17E-07
ENSG0000018	LCTL	protein_codir	1.94181477	0.11321992	0.22117081
ENSG0000016	DCLK3	protein_codir	1.94139164	0.00107911	0.0060814
ENSG0000016	BTNL9	protein_codir	1.94114796	2.72E-06	4.25E-05
ENSG0000009	MAGEB2	protein_codir	1.94097027	0.39366717	NA
ENSG0000019	OGDHL	protein_codir	1.93903119	0.08488237	0.17838861
ENSG0000007	APBB1IP	protein_codir	1.938671	9.31E-06	0.00012097
ENSG0000018	C3orf80	protein_codir	1.93718462	8.14E-08	2.11E-06
ENSG0000025	KHDRBS2-OT1	lncRNA	1.93673849	0.48753776	NA

ENSG0000017	ATAD5	protein_codir	1.9366179	4.34E-09	1.72E-07
ENSG0000027	RP11-12J10.4	lncRNA	1.9365549	0.02776002	0.07664252
ENSG0000014	PCSK6	protein_codir	1.93652626	0.0001384	0.00114551
ENSG0000025	CTD-3094K11	lncRNA	1.93596062	0.02016221	0.05966787
ENSG0000022	ERVMER34-1	protein_codir	1.93581667	0.00055577	0.00356238
ENSG0000017	PLA2G1B	protein_codir	1.9334384	0.12974818	0.24476776
ENSG0000024	PLCXD2	protein_codir	1.93311079	2.32E-06	3.72E-05
ENSG0000022	AC058791.1	lncRNA	1.9329655	0.00120779	0.00664525
ENSG0000028	RP3-329E20.3	lncRNA	1.93129572	0.15121735	NA
ENSG0000018	SPATA45	protein_codir	1.92970846	0.39346347	NA
ENSG0000022	RP3-340N1.2	lncRNA	1.92969801	0.04043753	0.10236316
ENSG0000020	CEMP1	protein_codir	1.92932134	0.01101764	0.03761059
ENSG0000027	CH507-9B2.1	protein_codir	1.92922708	8.10E-06	0.00010767
ENSG0000028	FAM237B	protein_codir	1.92893972	0.35577229	0.50935261
ENSG0000024	AP001062.8	lncRNA	1.92757064	0.0555414	0.13015896
ENSG0000010	GPR143	protein_codir	1.92737221	0.00872609	0.03126001
ENSG0000027	KB-1517D11.4	lncRNA	1.92728222	0.02446461	0.06943204
ENSG0000023	APOC2	protein_codir	1.92699297	0.01819523	0.05511047
ENSG0000019	SFTA2	protein_codir	1.92686398	0.1671607	0.29529764
ENSG0000028	RP11-309E23.	lncRNA	1.92555103	0.1156804	0.2247849
ENSG0000023	SAPCD1-AS1	lncRNA	1.92553294	0.042672	0.10674776
ENSG0000019	TLR7	protein_codir	1.92544808	0.0001615	0.00130427
ENSG0000023	RP11-432J24.	lncRNA	1.92542588	0.39317145	NA
ENSG0000022	LINC01655	lncRNA	1.92493663	0.40620239	NA
ENSG0000020	DUXAP8	lncRNA	1.92374138	5.43E-05	0.00052729
ENSG0000000	FGR	protein_codir	1.92372473	3.34E-06	5.08E-05
ENSG0000011	SLC16A10	protein_codir	1.92348647	0.00010357	0.00089481
ENSG0000022	STEAP2-AS1	lncRNA	1.92306738	0.22585168	NA
ENSG0000022	AC091493.2	lncRNA	1.92291323	0.49129672	NA
ENSG0000012	SLC10A2	protein_codir	1.92270464	0.40044935	NA
ENSG0000028	RP1-163O16.1	lncRNA	1.92204955	0.32268894	NA
ENSG0000017	GPR37L1	protein_codir	1.92142819	0.02850592	0.07831882
ENSG0000018	SPANXN3	protein_codir	1.92139692	0.49254235	NA
ENSG0000028	RP11-909M7.	lncRNA	1.92138049	0.00197725	0.00974167
ENSG0000026	MIA	protein_codir	1.92043028	0.00365151	0.01579061
ENSG0000018	PDE4B	protein_codir	1.92029432	2.82E-09	1.20E-07
ENSG0000000	HECW1	protein_codir	1.91960047	0.00418358	0.01766827
ENSG0000014	HUNK	protein_codir	1.91935936	0.00035585	0.00248259
ENSG0000026	RP11-321A17	lncRNA	1.9190879	0.31402532	NA
ENSG0000017	IL6-AS1	lncRNA	1.91892802	0.00305871	0.01371399
ENSG0000018	PABPC1L2A	protein_codir	1.91769586	0.49081498	NA
ENSG0000013	TESMIN	protein_codir	1.91757177	0.00069275	0.00427095
ENSG0000026	RP11-118B18.	lncRNA	1.91751368	0.49257127	NA
ENSG0000019	C5AR1	protein_codir	1.91736106	3.37E-06	5.11E-05
ENSG0000026	AC005954.3	lncRNA	1.91728959	0.24260928	0.38752687
ENSG0000026	RP11-235E17.	lncRNA	1.91692645	0.39913666	NA
ENSG0000027	RP11-147L13.	lncRNA	1.91623934	0.00446087	0.01855002

ENSG0000025	LYN	protein_codir	1.91531933	1.76E-08	5.66E-07
ENSG0000018	KRT5	protein_codir	1.91510594	0.03173509	0.08495088
ENSG0000007	PAG1	protein_codir	1.91487232	1.56E-07	3.67E-06
ENSG0000009	RASSF7	protein_codir	1.91437698	1.11E-05	0.00013917
ENSG0000025	RP11-649E7.7	lncRNA	1.91406403	0.31465174	0.4665386
ENSG0000028	RP3-359N14.3	lncRNA	1.91348348	0.01505196	0.04784613
ENSG0000026	RP11-1094M1	lncRNA	1.91308211	0.16549312	0.29293259
ENSG0000026	CTC-265F19.2	lncRNA	1.91284994	0.03168415	0.08483096
ENSG0000025	RP11-429P3.3	lncRNA	1.91252732	0.31040049	NA
ENSG0000011	BMP8B	protein_codir	1.91241187	0.00014995	0.00122644
ENSG0000027	AC131056.3	lncRNA	1.91223627	0.00132225	0.00714827
ENSG0000026	RP11-849F2.5	lncRNA	1.91178164	0.13938082	NA
ENSG0000025	CTD-2116N20	lncRNA	1.91152796	0.49079064	NA
ENSG0000022	AC114730.3	lncRNA	1.91049682	0.39519092	NA
ENSG0000028	RP11-352G9.2	lncRNA	1.91028027	0.48994091	NA
ENSG0000006	MCM10	protein_codir	1.90983493	0.00100675	0.00576181
ENSG0000028	LLNLF-96A1.1	lncRNA	1.90973831	0.11938872	0.23008069
ENSG0000000	MYH13	protein_codir	1.90933423	0.50096975	NA
ENSG0000027	RP11-169K17.	lncRNA	1.909187	0.08318336	0.17555776
ENSG0000026	AC008088.4	lncRNA	1.90840443	0.25556229	0.402247
ENSG0000028	CTB-85C5.1	lncRNA	1.90835376	0.0100397	0.03490732
ENSG0000023	LINC01048	lncRNA	1.9082225	0.40494022	NA
ENSG0000015	ARL11	protein_codir	1.9078061	0.00019535	0.00151493
ENSG0000026	CTB-41I6.2	lncRNA	1.90777232	0.00358184	0.0155649
ENSG0000006	TSPAN32	protein_codir	1.90760192	0.00041357	0.0028045
ENSG0000026	RP11-68I3.11	lncRNA	1.90721732	0.00054762	0.00352491
ENSG0000013	CD36	protein_codir	1.90703614	0.00014536	0.00119486
ENSG0000028	RP11-21K20.4	lncRNA	1.90689655	0.49075258	NA
ENSG0000015	CMTM7	protein_codir	1.90657055	5.45E-07	1.08E-05
ENSG0000018	GABRA5	protein_codir	1.9053449	0.15673311	NA
ENSG0000026	RP11-320H14	lncRNA	1.90478971	0.25352308	0.39985766
ENSG0000001	PRSS3	protein_codir	1.90447249	0.16434139	0.29143559
ENSG0000016	TEDC2	protein_codir	1.90421217	0.00174649	0.00886079
ENSG0000024	RP11-430C7.4	lncRNA	1.90279611	0.07330209	0.16033924
ENSG0000012	RHBDF2	protein_codir	1.90271663	1.58E-06	2.72E-05
ENSG0000012	SLC25A2	protein_codir	1.90230049	0.02975681	0.08098167
ENSG0000023	LINC01505	lncRNA	1.90182824	0.07382038	0.16112627
ENSG0000021	TENM3	protein_codir	1.9017776	0.00029406	0.00211953
ENSG0000021	HBG1	protein_codir	1.90145486	0.34227849	NA
ENSG0000023	MHENCR	lncRNA	1.90122658	6.65E-05	0.00062226
ENSG0000010	PPP1R17	protein_codir	1.90117	0.13148721	0.24735573
ENSG0000026	SERTM2	protein_codir	1.90116436	NA	NA
ENSG0000025	DDN-AS1	lncRNA	1.90086918	0.00071719	0.00439121
ENSG0000027	CH507-254M2	lncRNA	1.90055277	0.04066596	0.10276248
ENSG0000013	CLEC10A	protein_codir	1.90046488	1.63E-07	3.80E-06
ENSG0000017	ENC1	protein_codir	1.90024729	2.71E-06	4.23E-05
ENSG0000026	RP11-552F3.4	lncRNA	1.90019238	0.22982711	0.37226277

ENSG0000023	RP4-669H2.1	lncRNA	1.89994226	0.40320094	NA
ENSG0000009	ITPR3	protein_codir	1.89969894	1.83E-09	8.30E-08
ENSG0000026	CTC-244M17.	lncRNA	1.89969311	0.31415932	NA
ENSG0000025	TMPO-AS1	lncRNA	1.8992023	2.82E-05	0.00030495
ENSG0000028	RP11-363K21.	lncRNA	1.89915967	8.36E-05	0.00075125
ENSG0000018	PTGDR2	protein_codir	1.89913956	0.00157769	0.00820171
ENSG0000023	AC004947.2	lncRNA	1.89886164	0.01060532	0.03646508
ENSG0000011	NCF2	protein_codir	1.89883243	8.07E-06	0.00010733
ENSG0000018	MESP2	protein_codir	1.89851888	0.0087069	0.03121961
ENSG0000026	RP11-676J12.	lncRNA	1.89797808	0.2448307	NA
ENSG0000013	MORN3	protein_codir	1.89783238	4.75E-05	0.00046937
ENSG0000026	MIR924HG	lncRNA	1.89737395	0.19678546	0.3327954
ENSG0000026	RP11-626G11	lncRNA	1.89671837	0.01841725	0.05565477
ENSG0000009	TLL2	protein_codir	1.89602822	0.00048212	0.00317656
ENSG0000014	SLC13A5	protein_codir	1.89578268	0.09749727	0.19799114
ENSG0000013	IFI44L	protein_codir	1.8947342	4.53E-05	0.0004498
ENSG0000023	LINC00896	lncRNA	1.89460774	0.13153363	0.24740935
ENSG0000025	RP11-47I22.4	protein_codir	1.89388461	0.25495655	NA
ENSG0000023	NFAM1	protein_codir	1.89381399	5.18E-06	7.33E-05
ENSG0000026	PYCARD-AS1	lncRNA	1.89373545	0.00030925	0.00220822
ENSG0000016	PAQR6	protein_codir	1.89371855	2.69E-05	0.00029345
ENSG0000020	LINC02783	lncRNA	1.89356708	0.29971105	0.45072521
ENSG0000016	STXBP6	protein_codir	1.89354193	0.00014699	0.00120578
ENSG0000022	RP11-91I20.3	lncRNA	1.89328454	0.40847985	NA
ENSG0000010	RGCC	protein_codir	1.89299052	0.00046914	0.00310584
ENSG0000028	CTB-127M13.	protein_codir	1.89251777	0.20729472	0.34578332
ENSG0000028	RP11-1046B1	lncRNA	1.89134063	0.21216272	NA
ENSG0000026	CARD8-AS1	lncRNA	1.89113132	6.00E-08	1.63E-06
ENSG0000017	P2RY14	protein_codir	1.89104753	1.92E-07	4.37E-06
ENSG0000012	IL37	protein_codir	1.8908353	0.5611041	NA
ENSG0000026	ERFL	protein_codir	1.89026426	2.22E-05	0.0002504
ENSG0000026	ARHGAP28-A:	lncRNA	1.89017155	0.21336623	NA
ENSG0000010	CEP128	protein_codir	1.89003606	1.19E-07	2.92E-06
ENSG0000008	ZNF671	protein_codir	1.88998675	1.27E-06	2.25E-05
ENSG0000017	CYSLTR1	protein_codir	1.88987275	1.08E-07	2.69E-06
ENSG0000025	SLC5A8	protein_codir	1.88982414	0.5057233	NA
ENSG0000013	SULT4A1	protein_codir	1.88949599	0.15379792	0.27744096
ENSG0000027	H4C13	protein_codir	1.88915259	0.50592446	NA
ENSG0000023	RP11-439L18.	lncRNA	1.88886969	8.55E-06	0.00011302
ENSG0000022	AC013460.1	lncRNA	1.88868163	0.08576565	0.1798123
ENSG0000008	RAD54L	protein_codir	1.88863193	0.00116092	0.00643737
ENSG0000014	IGLON5	protein_codir	1.88700747	0.01028997	0.03560624
ENSG0000013	SLC31A2	protein_codir	1.88700707	1.45E-07	3.43E-06
ENSG0000013	DSG1	protein_codir	1.88644731	0.26447003	NA
ENSG0000022	LINC00337	lncRNA	1.88555098	0.29827869	0.44912144
ENSG0000015	SLA	protein_codir	1.88495152	8.97E-05	0.00079499
ENSG0000026	RP11-269G24	lncRNA	1.88427463	0.4949222	NA

ENSG0000016	CYBB	protein_codir	1.88399367	9.31E-05	0.00081879
ENSG0000028	RP5-881P19.8	lncRNA	1.88391853	0.40204037	NA
ENSG0000012	KIR2DL1	protein_codir	1.88370016	0.01614607	0.05047832
ENSG0000027	KB-68A7.2	lncRNA	1.88360487	0.49733744	NA
ENSG0000025	CTD-2034I4.2	lncRNA	1.8824092	0.17656353	0.30703282
ENSG0000018	SOCS1	protein_codir	1.88239368	1.61E-06	2.76E-05
ENSG0000023	RP11-522M21	lncRNA	1.88226636	0.32457845	NA
ENSG0000008	KCNH4	protein_codir	1.88176423	4.05E-06	5.96E-05
ENSG0000028	RP11-77G23.6	lncRNA	1.88160235	0.40357787	NA
ENSG0000014	PIK3R5	protein_codir	1.88156643	2.85E-05	0.00030737
ENSG0000011	CLEC2B	protein_codir	1.87789103	5.24E-10	2.95E-08
ENSG0000018	IRF7	protein_codir	1.87729696	9.17E-08	2.34E-06
ENSG0000015	TSPAN33	protein_codir	1.87726228	4.51E-06	6.50E-05
ENSG0000020	XXbac-BPG32	protein_codir	1.87635159	0.56414703	NA
ENSG0000017	TTC9B	protein_codir	1.87617137	0.41138268	NA
ENSG0000027	AC159540.2	lncRNA	1.87560981	0.50280998	NA
ENSG0000025	RP11-663P9.1	lncRNA	1.87541541	0.20471879	NA
ENSG0000016	TMC1	protein_codir	1.87534784	6.91E-05	0.00064242
ENSG0000024	RP11-252E2.1	lncRNA	1.87505276	0.11423737	0.22268587
ENSG0000018	EMILIN3	protein_codir	1.87404982	0.00821528	0.02983464
ENSG0000018	LINC02363	lncRNA	1.87401963	0.01788238	0.05438953
ENSG0000006	ADGRF5	protein_codir	1.87295115	9.78E-08	2.47E-06
ENSG0000009	EZR	protein_codir	1.87259683	2.10E-06	3.44E-05
ENSG0000017	UGT8	protein_codir	1.87231663	0.00067659	0.0041872
ENSG0000023	LINC00649	lncRNA	1.87178477	9.94E-05	0.00086434
ENSG0000010	SEPTIN3	protein_codir	1.87077696	0.00070159	0.00431198
ENSG0000023	KCNH1-IT1	lncRNA	1.86960071	0.4140435	NA
ENSG0000007	ATP2A3	protein_codir	1.86886329	1.32E-07	3.20E-06
ENSG0000027	RP11-340F14.	lncRNA	1.86883765	0.00203811	0.00996681
ENSG0000025	RP11-750H9.5	lncRNA	1.86871591	1.30E-07	3.15E-06
ENSG0000026	RP11-74E22.3	lncRNA	1.86796622	0.05087691	0.12212934
ENSG0000016	CYP4A22	protein_codir	1.86753137	0.09702672	0.19721908
ENSG0000017	LRRN2	protein_codir	1.86690318	4.71E-06	6.76E-05
ENSG0000026	RP11-720L2.2	lncRNA	1.86675102	0.32674664	NA
ENSG0000018	DHRS7C	protein_codir	1.86631607	0.51122873	NA
ENSG0000027	RP5-906C1.1	lncRNA	1.86594069	9.08E-05	0.00080182
ENSG0000019	PNMA5	protein_codir	1.86498484	0.05470612	0.12869555
ENSG0000020	CTA-373H7.7	lncRNA	1.86475154	0.00986988	0.03445477
ENSG0000026	RP11-68I3.4	lncRNA	1.8641909	0.00896238	0.03194532
ENSG0000011	NR4A3	protein_codir	1.86410681	0.00567172	0.02234307
ENSG0000028	RP11-19F9.2	lncRNA	1.86407846	0.18401692	0.31682754
ENSG0000019	ZNF695	protein_codir	1.86384332	0.02657949	0.07399713
ENSG0000021	MS4A4E	protein_codir	1.86369601	0.00324399	0.0143934
ENSG0000027	C17orf98	protein_codir	1.86346239	0.41618439	NA
ENSG0000025	SIGLEC14	protein_codir	1.86335207	0.00054681	0.0035205
ENSG0000014	FERMT3	protein_codir	1.86281501	1.11E-07	2.76E-06
ENSG0000016	P2RY12	protein_codir	1.86281267	0.0009355	0.00542941

ENSG0000017	LPAR3	protein_codir	1.86234043	0.00092363	0.00537517
ENSG0000009	TSPAN15	protein_codir	1.86199766	1.93E-07	4.40E-06
ENSG0000023	AC013463.2	lncRNA	1.86177837	0.01361046	0.0441948
ENSG0000017	CRACR2B	protein_codir	1.8615317	1.69E-05	0.00019902
ENSG0000025	AC022182.3	lncRNA	1.86107644	0.00691494	0.0260845
ENSG0000010	FOXF1	protein_codir	1.86103145	5.26E-05	0.00051271
ENSG0000018	RGPD1	protein_codir	1.86058591	0.00120947	0.00665314
ENSG0000028	RP11-36I17.3	lncRNA	1.86049631	0.4116993	NA
ENSG0000007	STK10	protein_codir	1.86004489	5.71E-09	2.19E-07
ENSG0000024	PSMB9	protein_codir	1.85973124	1.29E-07	3.14E-06
ENSG0000017	ADORA2B	protein_codir	1.859487	0.00419554	0.01770092
ENSG0000020	SMIM5	protein_codir	1.85867048	0.00015557	0.00126146
ENSG0000019	TMEM229B	protein_codir	1.85864013	1.35E-05	0.00016562
ENSG0000028	RP11-341N18	lncRNA	1.85807311	0.50840809	NA
ENSG0000027	LINC02804	lncRNA	1.85804476	0.00840148	0.03032131
ENSG0000024	AC007009.1	lncRNA	1.85703779	0.03093605	0.08334785
ENSG0000016	RSPH10B2	protein_codir	1.85703495	0.12138186	0.23315717
ENSG0000009	ITGA6	protein_codir	1.85662313	1.73E-10	1.15E-08
ENSG0000016	TREML1	protein_codir	1.85622074	0.00194558	0.00960788
ENSG0000026	CTD-2008L17.	lncRNA	1.85617823	0.25891968	0.40600441
ENSG0000015	CDA	protein_codir	1.85573644	1.51E-05	0.00018143
ENSG0000019	NRARP	protein_codir	1.85537225	8.13E-09	2.95E-07
ENSG0000019	H4C11	protein_codir	1.85449156	0.2971322	0.44767573
ENSG0000028	RP11-346B9.3	lncRNA	1.85414627	0.16343974	0.29017503
ENSG0000018	NPAP1	protein_codir	1.85406045	0.5093714	NA
ENSG0000027	LINC02427	lncRNA	1.85353057	0.00109503	0.00615228
ENSG0000016	S100P	protein_codir	1.8532438	0.02928197	0.07998893
ENSG0000018	H1-7	protein_codir	1.85260842	0.50750716	NA
ENSG0000026	RP11-154H12	lncRNA	1.8520852	0.14100685	0.26031744
ENSG0000000	MCUB	protein_codir	1.85203425	4.59E-09	1.81E-07
ENSG0000015	EXTL1	protein_codir	1.85087413	4.00E-05	0.00040523
ENSG0000017	C20orf197	lncRNA	1.85086811	0.00068584	0.00423403
ENSG0000028	RP5-968J1.1	lncRNA	1.85031489	0.00018336	0.00143954
ENSG0000012	CDH15	protein_codir	1.84971748	0.01220201	0.04060966
ENSG0000015	C9orf43	protein_codir	1.84965008	0.00035631	0.00248521
ENSG0000010	ACP5	protein_codir	1.84881224	0.00108533	0.0061086
ENSG0000027	MIR222HG	lncRNA	1.84841679	0.00155237	0.00808966
ENSG0000013	CH25H	protein_codir	1.84805737	0.0001349	0.0011206
ENSG0000007	SPAG5	protein_codir	1.84787659	7.54E-06	0.00010131
ENSG0000027	RP11-129K12.	lncRNA	1.84692266	0.07420769	0.16173096
ENSG0000018	GLTPD2	protein_codir	1.84691059	0.01152637	0.03891911
ENSG0000010	AKNA	protein_codir	1.84637585	3.69E-09	1.51E-07
ENSG0000001	BID	protein_codir	1.84573116	1.48E-08	4.91E-07
ENSG0000012	ADGRE2	protein_codir	1.84569784	9.39E-05	0.00082507
ENSG0000014	C17orf64	protein_codir	1.84564973	0.13522713	0.25222505
ENSG0000028	CTD-2536I1.4	lncRNA	1.84498541	0.2033988	0.34066308
ENSG0000025	RP11-66B24.1	lncRNA	1.84490554	0.43048829	NA

ENSG0000017	GPR82	protein_codir	1.84454646	0.00670382	0.02542354
ENSG0000020	C21orf62	protein_codir	1.84423717	0.00271519	0.01247107
ENSG0000026	CTB-50L17.9	lncRNA	1.84321935	0.17116719	NA
ENSG0000012	TNFRSF19	protein_codir	1.8429541	1.23E-05	0.00015212
ENSG0000013	KLRD1	protein_codir	1.84240306	8.62E-05	0.00077042
ENSG0000018	IQGAP3	protein_codir	1.8423319	5.06E-05	0.00049582
ENSG0000023	OR1J4	protein_codir	1.84206969	0.26435121	NA
ENSG0000008	ABCB1	protein_codir	1.84184148	7.74E-09	2.83E-07
ENSG0000028	FLICR	lncRNA	1.84039194	0.0110407	0.03765673
ENSG0000025	RP11-598F7.5	lncRNA	1.8402054	0.00340072	0.01492527
ENSG0000028	RP11-482G13	lncRNA	1.8401606	1.55E-06	2.68E-05
ENSG0000010	RUNDC3A	protein_codir	1.84000518	0.00113536	0.00631847
ENSG0000021	RP11-37N22.1	lncRNA	1.83993171	0.10705111	0.21228832
ENSG0000028	RP11-452F19.	lncRNA	1.83990153	0.04952324	0.1196508
ENSG0000024	XXbac-BPG18	protein_codir	1.83923232	0.01282052	0.04217014
ENSG0000014	SLC25A47	protein_codir	1.83870322	0.35563329	NA
ENSG0000010	EBI3	protein_codir	1.83836924	9.65E-06	0.00012474
ENSG0000018	CCSER1	protein_codir	1.83775317	3.56E-07	7.51E-06
ENSG0000026	RP11-87G24.3	lncRNA	1.83669355	0.42182479	NA
ENSG0000028	RP11-347D21	lncRNA	1.83660947	0.57253593	NA
ENSG0000016	C10orf82	protein_codir	1.83533614	0.02324404	0.0666252
ENSG0000018	DMRTC1B	protein_codir	1.8352039	0.0319307	0.08524317
ENSG0000026	RP11-109G23	lncRNA	1.83484701	0.0050731	0.02051144
ENSG0000025	IL21R-AS1	lncRNA	1.83481454	0.06660942	0.14975597
ENSG0000009	KIF4A	protein_codir	1.83479796	0.00016579	0.00133076
ENSG0000028	RP11-683I7.1	lncRNA	1.83460116	0.00951607	0.0334522
ENSG0000018	AC069277.2	lncRNA	1.83447823	0.42242296	NA
ENSG0000025	LINC02763	lncRNA	1.83430799	0.4300023	NA
ENSG0000023	LINC02863	lncRNA	1.83396461	0.1186875	0.22909819
ENSG0000022	LINC01087	lncRNA	1.8338095	0.4144629	NA
ENSG0000027	AC000068.10	lncRNA	1.83369017	0.15153042	0.27433446
ENSG0000022	CHL1-AS2	lncRNA	1.83368742	0.00543241	0.02164812
ENSG0000016	MFSD2A	protein_codir	1.83232383	4.92E-05	0.00048422
ENSG0000018	MACC1	protein_codir	1.83195433	0.0015026	0.0078939
ENSG0000028	RP11-255E6.7	lncRNA	1.83172809	0.42305223	NA
ENSG0000026	RP11-319G9.5	lncRNA	1.83168462	0.15774828	0.28281039
ENSG0000011	GRM6	protein_codir	1.8306067	0.41697428	NA
ENSG0000011	PTK7	protein_codir	1.82930132	5.46E-05	0.0005289
ENSG0000025	RP11-467H10	lncRNA	1.82928505	0.28347387	NA
ENSG0000018	FAM83H	protein_codir	1.82927014	0.0001854	0.00145256
ENSG0000009	RAPGEF4	protein_codir	1.82914057	3.06E-10	1.88E-08
ENSG0000010	UPK1A	protein_codir	1.82865862	0.04653888	0.11397445
ENSG0000026	CTC-246B18.1	lncRNA	1.82781079	0.05340836	0.12645854
ENSG0000018	ZNF267	protein_codir	1.8266736	1.59E-07	3.73E-06
ENSG0000024	PVT1	lncRNA	1.82660946	9.30E-06	0.00012097
ENSG0000017	SEZ6L2	protein_codir	1.82657279	5.88E-05	0.00056397
ENSG0000027	UHRF1	protein_codir	1.82644986	0.00072616	0.00443435

ENSG0000024	RP3-330M21.	lncRNA	1.82625061	0.06761871	0.15139662
ENSG0000017	SLC16A13	protein_codir	1.82593928	3.95E-05	0.00040115
ENSG0000003	CASR	protein_codir	1.82515661	0.44400853	0.5950852
ENSG0000012	GNMT	protein_codir	1.82473861	2.76E-05	0.00029917
ENSG0000016	NOD2	protein_codir	1.82418098	3.11E-07	6.67E-06
ENSG0000023	LINC01684	lncRNA	1.82368599	3.38E-05	0.00035312
ENSG0000027	AC068831.16	lncRNA	1.8235253	0.33094676	NA
ENSG0000017	ARL14	protein_codir	1.82326276	0.41508462	NA
ENSG0000015	PPARGC1B	protein_codir	1.82311785	3.45E-05	0.00035896
ENSG0000026	RP11-961A15	lncRNA	1.82271551	0.42388609	NA
ENSG0000026	CTB-31O20.9	lncRNA	1.82244073	0.50759934	NA
ENSG0000017	KCNG2	protein_codir	1.82220512	0.00498694	0.02022308
ENSG0000023	RP5-1120P11.	lncRNA	1.8220624	0.00825409	0.0299284
ENSG0000025	RP11-14I17.3	lncRNA	1.82123047	0.42584365	NA
ENSG0000028	RP11-27J8.3	lncRNA	1.8206692	1.70E-05	0.00020013
ENSG0000011	KHDRBS2	protein_codir	1.82060143	0.00342874	0.01502728
ENSG0000028	RP11-666A8.1	protein_codir	1.81996279	7.58E-05	0.00069153
ENSG0000025	RP11-293I14.:	protein_codir	1.81986155	0.07287567	0.15966544
ENSG0000011	PROX1	protein_codir	1.81962407	0.00170198	0.00868924
ENSG0000028	RP11-216B9.8	lncRNA	1.81939374	0.36369549	NA
ENSG0000010	CPVL	protein_codir	1.81937682	4.61E-06	6.65E-05
ENSG0000026	RP1-77H15.1	lncRNA	1.81934146	0.41978997	NA
ENSG0000013	C5AR2	protein_codir	1.81923523	2.37E-06	3.79E-05
ENSG0000017	HSPA6	protein_codir	1.81884583	0.00347089	0.01517345
ENSG0000007	DUSP13	protein_codir	1.81872972	0.12475453	0.23789838
ENSG0000027	PPIAL4E	protein_codir	1.818602	0.5763558	NA
ENSG0000027	PPIAL4F	protein_codir	1.818602	0.5763558	NA
ENSG0000024	RP11-902B17.	lncRNA	1.81788744	0.00020392	0.00157015
ENSG0000024	RP11-1E22.1	lncRNA	1.81786217	0.33263404	NA
ENSG0000028	CTD-2366F13.	lncRNA	1.8177527	0.43194106	NA
ENSG0000026	RP11-455O6.2	lncRNA	1.81766203	0.0665025	0.14956429
ENSG0000002	TYMP	protein_codir	1.81697655	3.45E-06	5.21E-05
ENSG0000016	DRGX	protein_codir	1.81624559	0.31817618	0.47017421
ENSG0000025	RP11-247L20.	lncRNA	1.8154568	0.52355898	NA
ENSG0000025	MSH5-SAPCD	protein_codir	1.81437031	0.00038733	0.00266521
ENSG0000010	CHRDL1	protein_codir	1.81431262	0.00053627	0.00346396
ENSG0000010	CYTH4	protein_codir	1.81333234	3.36E-07	7.14E-06
ENSG0000018	RINL	protein_codir	1.81201653	2.56E-06	4.04E-05
ENSG0000012	SRGN	protein_codir	1.81197933	5.40E-08	1.48E-06
ENSG0000023	LINC01426	lncRNA	1.81168147	3.90E-05	0.00039682
ENSG0000028	RP11-281P23.	lncRNA	1.8110259	0.00074107	0.00450451
ENSG0000014	C1orf162	protein_codir	1.81089166	2.11E-06	3.46E-05
ENSG0000016	TNFAIP8L2	protein_codir	1.81066105	7.71E-05	0.00070148
ENSG0000020	SPDYC	protein_codir	1.81056388	0.51886762	NA
ENSG0000024	GABPB1-AS1	lncRNA	1.81044491	0.00190386	0.00945086
ENSG0000023	RP11-432J24.	lncRNA	1.80992871	0.07281118	0.15956873
ENSG0000027	RP11-121A8.1	lncRNA	1.80968106	0.0011932	0.00657546

ENSG0000022	FLJ31104	lncRNA	1.80964425	0.00116989	0.00647672
ENSG0000018	SBK1	protein_codir	1.80956004	0.00031434	0.0022388
ENSG0000018	OLFML2A	protein_codir	1.80942808	7.02E-10	3.75E-08
ENSG0000016	CTRC	protein_codir	1.8093233	0.07110703	0.15681701
ENSG0000020	CDSN	protein_codir	1.80892339	0.42784887	NA
ENSG0000011	MDK	protein_codir	1.80883407	1.36E-07	3.27E-06
ENSG0000019	GIMAP5	protein_codir	1.80880463	1.24E-06	2.21E-05
ENSG0000017	GIMAP7	protein_codir	1.80838131	1.51E-05	0.00018139
ENSG0000013	IL6	protein_codir	1.80825301	0.01721061	0.05286444
ENSG0000022	IL6R-AS1	lncRNA	1.807744	3.74E-06	5.56E-05
ENSG0000028	RP11-683L23.	lncRNA	1.80727235	0.4292493	NA
ENSG0000028	RP1-46C2.3	lncRNA	1.80713263	0.2611321	NA
ENSG0000026	AD000671.6	protein_codir	1.8065541	2.16E-05	0.00024493
ENSG0000028	PEG13	lncRNA	1.8063679	5.81E-05	0.00055816
ENSG0000020	AIF1	protein_codir	1.80613898	1.99E-08	6.22E-07
ENSG0000026	CTD-3193K9.3	lncRNA	1.80607894	0.13701615	0.2546958
ENSG0000013	CD101	protein_codir	1.80603606	1.05E-06	1.92E-05
ENSG0000013	CNMD	protein_codir	1.80576044	0.51532464	NA
ENSG0000020	LRCOL1	protein_codir	1.80571808	0.0702204	0.15540766
ENSG0000023	RP11-547D24	lncRNA	1.8053175	0.13384491	0.25055757
ENSG0000012	MGAT3	protein_codir	1.80503999	1.68E-05	0.00019831
ENSG0000011	PADI2	protein_codir	1.80468765	2.56E-05	0.00028232
ENSG0000028	RP1-68O2.5	lncRNA	1.80462414	0.34254533	NA
ENSG0000028	RP5-1166H10	lncRNA	1.80388935	0.01157762	0.03904444
ENSG0000028	RP11-81G4.1	lncRNA	1.8037142	0.51757399	NA
ENSG0000028	RP11-852E15.	lncRNA	1.80368294	0.34265369	NA
ENSG0000013	FHDC1	protein_codir	1.80243853	6.51E-07	1.26E-05
ENSG0000010	IL7	protein_codir	1.80234339	1.03E-06	1.89E-05
ENSG0000025	LINC01181	lncRNA	1.80207216	0.42850945	NA
ENSG0000014	OTULINL	protein_codir	1.80184552	5.52E-05	0.00053406
ENSG0000028	RP11-256B12.	lncRNA	1.80164801	0.01415411	0.0456009
ENSG0000018	OR2AK2	protein_codir	1.80163795	0.52056059	NA
ENSG0000028	RP11-13A2.5	protein_codir	1.80109396	0.19786825	0.33407427
ENSG0000014	FAM135B	protein_codir	1.80106595	0.00941874	0.03321148
ENSG0000024	OR2A7	protein_codir	1.80101443	0.0058799	0.0229642
ENSG0000024	PSMD6-AS1	lncRNA	1.80079373	0.05590322	0.13077353
ENSG0000026	RP11-426C22.	lncRNA	1.80023967	0.00187621	0.00933878
ENSG0000013	GALNT6	protein_codir	1.79896254	3.70E-05	0.00038079
ENSG0000022	CITF22-49D8.	lncRNA	1.79796734	0.33733796	NA
ENSG0000026	RP11-67A1.2	lncRNA	1.79780257	0.25959731	0.40667448
ENSG0000025	RP11-598F7.3	lncRNA	1.79762359	0.01004533	0.0349216
ENSG0000011	PRSS16	protein_codir	1.79736149	0.00097851	0.00563053
ENSG0000017	CYP4F22	protein_codir	1.79711887	0.00471303	0.01937686
ENSG0000025	RP11-547D23	lncRNA	1.796958	0.16379471	0.29071497
ENSG0000028	RP11-507K12.	protein_codir	1.79680946	0.2487551	0.39443435
ENSG0000026	RP11-325K4.3	lncRNA	1.79647844	0.0003987	0.00272708
ENSG0000028	RP11-444C24.	lncRNA	1.79563194	0.52411724	NA

ENSG0000007	ADD2	protein_codir	1.7949424	3.06E-05	0.00032518
ENSG0000019	DCHS2	protein_codir	1.79481269	0.03859842	0.09868449
ENSG0000022	AC007255.8	lncRNA	1.79466989	0.01451153	0.04647593
ENSG0000013	FGF5	protein_codir	1.79410311	0.07623249	0.16488186
ENSG0000015	ACE	protein_codir	1.79315884	1.24E-06	2.21E-05
ENSG0000006	GNA15	protein_codir	1.79305555	3.65E-07	7.69E-06
ENSG0000024	RP11-221J22.	lncRNA	1.79301199	0.0732	0.16020476
ENSG0000023	LNCAROD	lncRNA	1.79242723	0.00800434	0.02922792
ENSG0000026	LINC02133	lncRNA	1.79228922	0.30450569	0.45594964
ENSG0000025	RP11-831A10	lncRNA	1.79222445	0.43280553	NA
ENSG0000022	RP5-1160K1.6	lncRNA	1.79197529	0.58202523	NA
ENSG0000026	CTC-360G5.6	lncRNA	1.79186548	0.58204867	NA
ENSG0000017	RAG2	protein_codir	1.79177354	0.51990619	NA
ENSG0000026	LINC01572	lncRNA	1.79130229	0.00178646	0.0090073
ENSG0000026	RP11-746M1.	lncRNA	1.79111741	0.21803619	NA
ENSG0000019	XRCC2	protein_codir	1.79104746	6.63E-05	0.00062062
ENSG0000014	PMP2	protein_codir	1.79102965	0.03987318	0.1012594
ENSG0000017	CDH4	protein_codir	1.79099231	0.04201445	0.10550407
ENSG0000014	MARVELD3	protein_codir	1.79021416	0.03406363	0.08974887
ENSG0000009	MAP3K1	protein_codir	1.79005705	3.38E-06	5.12E-05
ENSG0000021	GIMAP1	protein_codir	1.78983012	5.93E-06	8.24E-05
ENSG0000012	LINC02871	lncRNA	1.78958392	0.16475203	0.29205931
ENSG0000017	LBX2	protein_codir	1.78943568	1.11E-05	0.00013931
ENSG0000028	RP11-963H4.8	lncRNA	1.78940417	0.3652035	NA
ENSG0000004	TG	protein_codir	1.78933561	4.17E-06	6.09E-05
ENSG0000016	ITGAM	protein_codir	1.78927889	2.09E-05	0.00023818
ENSG0000023	ANKRD63	protein_codir	1.78927057	0.05178027	0.12351422
ENSG0000018	URAD	protein_codir	1.78817615	0.44140879	NA
ENSG0000016	GPRIN1	protein_codir	1.78815734	1.06E-05	0.00013466
ENSG0000018	GNG2	protein_codir	1.78814141	1.09E-06	1.98E-05
ENSG0000023	AC012456.4	lncRNA	1.78803526	0.43242872	NA
ENSG0000026	CTD-2349B8.1	lncRNA	1.78696298	0.43535219	NA
ENSG0000025	RP11-76E17.4	lncRNA	1.78592967	0.1603486	0.28610002
ENSG0000020	SLC44A4	protein_codir	1.78583784	0.00273278	0.01254019
ENSG0000017	CREG2	protein_codir	1.78579387	0.01353735	0.04402467
ENSG0000010	TMEM176B	protein_codir	1.78558558	3.36E-06	5.10E-05
ENSG0000026	RP11-389C8.2	lncRNA	1.78520785	0.11683757	0.22645992
ENSG0000013	STX11	protein_codir	1.78484272	1.45E-07	3.45E-06
ENSG0000014	NFKBIE	protein_codir	1.78477786	6.21E-08	1.67E-06
ENSG0000015	THY1	protein_codir	1.78467378	2.53E-05	0.00027953
ENSG0000021	LCNL1	protein_codir	1.78445478	0.012959	0.04251931
ENSG0000028	CTA-250D10.2	lncRNA	1.78437353	0.18918775	NA
ENSG0000010	NCF4	protein_codir	1.78346288	4.90E-07	9.89E-06
ENSG0000028	RP11-63G10.4	lncRNA	1.78299555	0.01507837	0.04790232
ENSG0000010	TIMP3	protein_codir	1.78296025	1.01E-06	1.85E-05
ENSG0000025	RPS6KB2-AS1	lncRNA	1.78275695	0.05213639	0.12410645
ENSG0000028	PRNCR1	lncRNA	1.78271931	0.00423545	0.01781109

ENSG0000006	BCAT1	protein_codir	1.78248952	3.80E-05	0.00038903
ENSG0000020	MICB	protein_codir	1.78243712	1.63E-07	3.80E-06
ENSG0000026	AP000265.1	lncRNA	1.78175305	0.14681367	0.26825836
ENSG0000016	ZDHHC19	protein_codir	1.78171731	0.00602072	0.0233852
ENSG0000012	FEZF1	protein_codir	1.78105408	0.1828846	0.31534977
ENSG0000026	SMIM22	protein_codir	1.78092569	0.35704391	NA
ENSG0000008	PILRA	protein_codir	1.7808213	9.69E-06	0.0001251
ENSG0000016	UBXN10	protein_codir	1.78046535	0.00024878	0.00184864
ENSG0000007	PTGS2	protein_codir	1.78022211	0.00510919	0.02061623
ENSG0000024	EGFLAM-AS2	lncRNA	1.77977364	0.30589493	NA
ENSG0000023	LINC01638	lncRNA	1.77973333	0.00418699	0.01767457
ENSG0000006	STYK1	protein_codir	1.779728	0.00014602	0.00119929
ENSG0000013	PAQR5	protein_codir	1.77959801	0.00175121	0.00887657
ENSG0000027	RP11-398A8.5	lncRNA	1.77945475	0.3504329	NA
ENSG0000024	LINC01962	lncRNA	1.77892912	0.01582538	0.04969836
ENSG0000015	FLI1	protein_codir	1.7788534	5.24E-08	1.45E-06
ENSG0000024	RP11-215P8.3	lncRNA	1.77877924	0.0819506	0.17377931
ENSG0000011	CD207	protein_codir	1.77860005	0.01856799	0.05603813
ENSG0000022	PHEX-AS1	lncRNA	1.77813609	0.58497786	NA
ENSG0000017	MYEOV	protein_codir	1.77805852	0.00054885	0.00353177
ENSG0000026	AC022154.7	lncRNA	1.77769197	0.43505656	NA
ENSG0000025	KLRC4-KLRK1	protein_codir	1.77722458	0.02981895	0.08108687
ENSG0000013	EMP1	protein_codir	1.77637865	0.00163811	0.00845377
ENSG0000012	CRHR1	protein_codir	1.77564752	0.04489645	0.11099535
ENSG0000002	GABRA1	protein_codir	1.77544911	0.58555188	NA
ENSG0000008	HSD17B2	protein_codir	1.77538477	0.29289347	0.44300519
ENSG0000025	RP11-5L12.1	lncRNA	1.77537338	0.23163591	NA
ENSG0000017	WDR49	protein_codir	1.77519131	0.00498434	0.02021547
ENSG0000015	AP1S3	protein_codir	1.77471252	1.51E-05	0.00018132
ENSG0000028	LINC01617	lncRNA	1.77466193	0.12684894	0.24082814
ENSG0000014	GLRA3	protein_codir	1.77463543	0.04041443	0.10231407
ENSG0000011	IL18RAP	protein_codir	1.77462611	7.49E-06	0.00010082
ENSG0000013	RAB33A	protein_codir	1.77351841	2.03E-05	0.00023166
ENSG0000010	QPRT	protein_codir	1.77326265	4.71E-07	9.54E-06
ENSG0000018	CSMD1	protein_codir	1.77319002	0.1758738	0.30610841
ENSG0000011	RAD51AP1	protein_codir	1.772587	4.21E-05	0.00042294
ENSG0000022	RP11-33A14.1	lncRNA	1.77236313	0.00018553	0.00145287
ENSG0000026	HID1-AS1	lncRNA	1.77235817	0.00449067	0.01864585
ENSG0000028	RP11-510D4.1	lncRNA	1.77234214	0.18814954	0.32199581
ENSG0000022	AC118754.4	lncRNA	1.77130012	0.52246721	NA
ENSG0000013	GABBR2	protein_codir	1.77127541	0.0954796	0.19481027
ENSG0000026	RP11-497H17	lncRNA	1.77126217	0.00677312	0.02564758
ENSG0000012	TAS2R5	protein_codir	1.77098271	0.00356923	0.01552719
ENSG0000025	RP11-91P24.6	lncRNA	1.7709295	0.01996032	0.05921633
ENSG0000027	RP11-50I19.2	lncRNA	1.77063959	0.00153891	0.00803532
ENSG0000025	CTD-2297D10	lncRNA	1.77061978	0.28905523	0.43859283
ENSG0000005	ITIH1	protein_codir	1.77039899	0.0033178	0.01465245

ENSG0000026	EML2-AS1	lncRNA	1.77032928	0.00181922	0.00911942
ENSG0000026	RP11-433M22	lncRNA	1.77031221	0.58665	NA
ENSG0000028	RP11-274J15.	lncRNA	1.7698341	0.44604618	NA
ENSG0000018	GABRD	protein_codir	1.76958228	0.00069565	0.00428212
ENSG0000024	MAST4-IT1	lncRNA	1.76944201	0.58683611	NA
ENSG0000017	GEN1	protein_codir	1.7688185	2.52E-06	3.99E-05
ENSG0000027	RP11-245P10.	lncRNA	1.76843823	0.58705083	NA
ENSG0000024	CDKN2B-AS1	lncRNA	1.76835852	0.00024118	0.00180134
ENSG0000028	LINC02795	lncRNA	1.76797417	0.10578833	0.21050964
ENSG0000022	LINC00570	lncRNA	1.7675629	0.03463413	0.09085331
ENSG0000021	TNFRSF25	protein_codir	1.76686665	8.48E-05	0.00076137
ENSG0000020	HLA-F	protein_codir	1.76675831	2.43E-09	1.05E-07
ENSG0000021	AC114730.5	lncRNA	1.76670858	0.52352503	NA
ENSG0000012	CXCL6	protein_codir	1.76636465	0.0055398	0.02195253
ENSG0000010	PIK3IP1	protein_codir	1.76632034	6.99E-10	3.75E-08
ENSG0000010	CORO2A	protein_codir	1.76631785	2.52E-06	3.99E-05
ENSG0000026	CTB-50L17.7	lncRNA	1.76578037	0.038148	0.09775913
ENSG0000027	RP11-426C22.	lncRNA	1.76564051	0.03505382	0.09163983
ENSG0000017	KRT27	protein_codir	1.76525668	0.07723538	0.16633815
ENSG0000026	RP11-666A8.7	lncRNA	1.76453239	0.06579027	0.14834902
ENSG0000017	TPSAB1	protein_codir	1.76355089	5.16E-05	0.00050482
ENSG0000025	RP11-113D6.6	lncRNA	1.76321706	0.44196322	NA
ENSG0000015	IPMK	protein_codir	1.76310483	4.04E-10	2.35E-08
ENSG0000015	BMP6	protein_codir	1.76287321	0.00041315	0.00280371
ENSG0000019	HMGA2-AS1	lncRNA	1.76278145	0.02979101	0.08103906
ENSG0000013	KDM6B	protein_codir	1.76249258	0.00011345	0.00096949
ENSG0000027	KB-1440D3.13	lncRNA	1.76242441	0.45256697	NA
ENSG0000013	BTG1	protein_codir	1.7618542	2.64E-12	3.49E-10
ENSG0000022	AC106875.1	lncRNA	1.76119501	0.58860125	NA
ENSG0000017	SLC9C1	protein_codir	1.76047793	0.03050278	0.08251584
ENSG0000001	MARCO	protein_codir	1.75970655	0.01056113	0.03634418
ENSG0000028	RP4-606D23.1	lncRNA	1.75947665	0.22627905	0.36830852
ENSG0000018	STAC3	protein_codir	1.75943648	1.45E-06	2.53E-05
ENSG0000023	RP11-13P5.2	lncRNA	1.75909752	0.0089476	0.0319128
ENSG0000023	RP4-530I15.9	lncRNA	1.75909169	0.26272817	0.41015076
ENSG0000025	CTD-2353F22.	lncRNA	1.75897512	0.02467579	0.06989492
ENSG0000009	JPH4	protein_codir	1.7585816	0.00119267	0.00657515
ENSG0000000	PRSS22	protein_codir	1.75819766	0.30356756	0.45484084
ENSG0000016	HCRT	protein_codir	1.75790662	0.20927829	0.34825078
ENSG0000025	RP11-677M14	lncRNA	1.7578279	0.20087217	0.33772153
ENSG0000023	IDI2-AS1	lncRNA	1.75757151	0.10704627	0.21228832
ENSG0000027	DACH1	protein_codir	1.7571487	1.07E-05	0.00013504
ENSG0000027	LINC02343	lncRNA	1.75710038	0.52966123	NA
ENSG0000028	AP000465.1	lncRNA	1.75707062	0.43533897	NA
ENSG0000017	HIC1	protein_codir	1.75697726	1.17E-07	2.88E-06
ENSG0000025	CTA-398F10.1	lncRNA	1.75686922	0.35827774	NA
ENSG0000000	IL32	protein_codir	1.7558716	2.31E-06	3.72E-05

ENSG0000025	CTD-2035E11.	lncRNA	1.755868	0.0015443	0.00805663
ENSG0000025	RP11-603J24.!	protein_codir	1.75582555	0.58975177	NA
ENSG0000017	CAVIN4	protein_codir	1.75582078	0.00497135	0.02016954
ENSG0000027	RP11-491F9.1	lncRNA	1.75565005	0.00152828	0.00799538
ENSG0000010	HAGHL	protein_codir	1.75563385	3.22E-05	0.00033918
ENSG0000013	ZNF331	protein_codir	1.75559552	3.80E-07	7.96E-06
ENSG0000027	MIR223HG	lncRNA	1.75536549	0.00656495	0.02503364
ENSG0000023	AC092162.1	lncRNA	1.7550933	0.07821996	0.16782688
ENSG0000026	AC004076.9	protein_codir	1.7548269	0.159679	0.28531078
ENSG0000027	AC002059.10	lncRNA	1.75476924	0.07990798	0.17045554
ENSG0000028	RP13-726E6.4	lncRNA	1.75393488	0.36431665	NA
ENSG0000013	LILRB2	protein_codir	1.75384466	7.38E-06	9.96E-05
ENSG0000025	LINC01500	lncRNA	1.75366571	0.15830065	0.28343472
ENSG0000010	GATA1	protein_codir	1.75363619	0.03063361	0.0827563
ENSG0000022	RNF32-AS1	lncRNA	1.75324943	0.00138389	0.00739752
ENSG0000016	SHH	protein_codir	1.7530877	0.21152649	0.350998
ENSG0000016	STRBP	protein_codir	1.75272622	2.73E-06	4.25E-05
ENSG0000012	IRF5	protein_codir	1.75270033	3.04E-05	0.00032398
ENSG0000027	RP11-532F6.3	lncRNA	1.75236447	0.00235286	0.01115956
ENSG0000018	BTN3A2	protein_codir	1.75227399	0.000124	0.00104446
ENSG0000014	CNKSR1	protein_codir	1.75222047	2.16E-05	0.00024486
ENSG0000027	RP11-305D15	lncRNA	1.75211424	0.09008385	0.18633386
ENSG0000022	RP11-307P5.1	lncRNA	1.75161309	0.44317985	NA
ENSG0000026	RP11-16C1.2	lncRNA	1.75157396	0.00319598	0.01421463
ENSG0000016	RBM47	protein_codir	1.75142895	1.66E-05	0.00019636
ENSG0000010	ESRP1	protein_codir	1.75138402	0.18468394	0.31775808
ENSG0000027	RP11-95F22.1	lncRNA	1.75131918	0.11918566	0.22986579
ENSG0000012	MYOD1	protein_codir	1.75077714	0.59083441	NA
ENSG0000025	AC078852.2	lncRNA	1.75016228	0.53036899	NA
ENSG0000025	RP11-167N4.2	lncRNA	1.74997983	0.15737968	0.28224897
ENSG0000024	RP11-742B18.	lncRNA	1.749781	0.01684314	0.05204893
ENSG0000013	CASP5	protein_codir	1.7490269	0.00170088	0.0086886
ENSG0000027	CTD-3116E22.	lncRNA	1.74865081	0.02076544	0.06104752
ENSG0000016	TK1	protein_codir	1.74845754	6.01E-05	0.00057341
ENSG0000011	MAK	protein_codir	1.74827248	0.00047416	0.00313238
ENSG0000026	PECAM1	protein_codir	1.74767272	1.95E-08	6.12E-07
ENSG0000017	TUBB8B	protein_codir	1.7473261	0.39008926	NA
ENSG0000016	CCDC138	protein_codir	1.7471904	2.89E-07	6.25E-06
ENSG0000010	ICAM2	protein_codir	1.74681889	3.83E-08	1.10E-06
ENSG0000015	HTR6	protein_codir	1.74654323	0.00125924	0.00687085
ENSG0000016	CD200R1	protein_codir	1.74624751	0.00097972	0.00563515
ENSG0000028	RP11-314E23.	lncRNA	1.74591677	0.45233236	NA
ENSG0000009	SH2D3C	protein_codir	1.74470748	5.31E-07	1.06E-05
ENSG0000025	LINC02244	lncRNA	1.74432423	0.24889538	0.39456411
ENSG0000014	KCNH5	protein_codir	1.74369531	0.5923546	NA
ENSG0000026	RP11-498C9.2	lncRNA	1.74368556	0.08264589	0.17469976
ENSG0000010	LILRA1	protein_codir	1.74363148	0.00041945	0.00283396

ENSG0000025	CRAT37	lncRNA	1.74337997	0.25215332	NA
ENSG0000017	SLC26A5	protein_codir	1.74329392	0.10292671	0.2062135
ENSG0000011	OPRD1	protein_codir	1.74312768	0.1627669	0.28923771
ENSG0000025	RP11-800A3.3	lncRNA	1.74254634	0.1564852	0.28098565
ENSG0000015	DNAH3	protein_codir	1.74230878	0.2036036	0.3409234
ENSG0000024	LINC02218	protein_codir	1.74152847	0.21773417	0.35811456
ENSG0000017	CBY2	protein_codir	1.7412821	0.43766604	NA
ENSG0000013	CCDC62	protein_codir	1.74111797	0.0011181	0.00625142
ENSG0000011	OTOF	protein_codir	1.74052015	0.00362776	0.01571745
ENSG0000013	HMGA1	protein_codir	1.74003322	1.29E-06	2.28E-05
ENSG0000018	SPATA12	protein_codir	1.73991891	0.02360552	0.06740948
ENSG0000014	PIF1	protein_codir	1.73986662	0.00067184	0.00416528
ENSG0000026	FIGNL2	protein_codir	1.73977797	8.58E-06	0.00011325
ENSG0000014	IQGAP2	protein_codir	1.73862867	4.32E-05	0.00043197
ENSG0000025	LINC01094	lncRNA	1.73819	0.00039522	0.00270803
ENSG0000020	CFI	protein_codir	1.73767958	9.93E-07	1.83E-05
ENSG0000018	FOXI2	protein_codir	1.73764322	0.00432668	0.01810102
ENSG0000027	RP11-480C16.	lncRNA	1.73725282	0.11301038	0.22088647
ENSG0000023	RP11-763B22.	lncRNA	1.73722087	0.02345999	0.067091
ENSG0000026	RP11-649A18	lncRNA	1.73697582	0.21273085	0.35234026
ENSG0000010	RASSF4	protein_codir	1.73685232	1.39E-05	0.00016971
ENSG0000022	RP11-809C18.	lncRNA	1.73602115	0.07697573	0.16593067
ENSG0000027	H3C7	protein_codir	1.73571657	0.44916416	NA
ENSG0000023	SLC12A9-AS1	lncRNA	1.73508877	0.20236233	NA
ENSG0000016	TMEM52B	protein_codir	1.73483385	0.00830694	0.03007047
ENSG0000028	RP11-112J3.2	protein_codir	1.73432999	0.53886241	NA
ENSG0000027	RP11-169K17.	lncRNA	1.73410006	0.14879361	0.27087263
ENSG0000022	RP11-262H14	lncRNA	1.73405505	0.04010978	0.10173706
ENSG0000017	RAB40A	protein_codir	1.73322014	0.00395125	0.01684938
ENSG0000018	TMEM121B	protein_codir	1.73296978	0.00150276	0.0078939
ENSG0000017	KBTBD11	protein_codir	1.73286126	0.00230938	0.01100691
ENSG0000010	GGT1	protein_codir	1.73277352	6.28E-06	8.64E-05
ENSG0000018	ZNF101	protein_codir	1.73270719	1.27E-05	0.00015755
ENSG0000006	KCNAB2	protein_codir	1.73247067	7.01E-06	9.51E-05
ENSG0000022	SAPCD1	protein_codir	1.73232722	0.00651075	0.02490679
ENSG0000016	RGS14	protein_codir	1.73184136	5.08E-07	1.02E-05
ENSG0000006	SPI1	protein_codir	1.73171135	9.20E-06	0.00012008
ENSG0000026	RP11-830F9.7	lncRNA	1.73135119	0.02800045	0.07719059
ENSG0000025	AF131215.4	lncRNA	1.73128036	0.03244161	0.08636503
ENSG0000021	CPLX3	protein_codir	1.7309171	0.1073187	0.21260525
ENSG0000028	RP11-184E24.	lncRNA	1.73065654	0.54413918	NA
ENSG0000016	VANGL2	protein_codir	1.73003903	0.00048893	0.0032115
ENSG0000022	RP5-997D24.3	lncRNA	1.72983864	0.36470703	NA
ENSG0000016	CXCL16	protein_codir	1.7293881	6.08E-06	8.43E-05
ENSG0000014	ARL5C	protein_codir	1.72921527	0.09801455	0.19880751
ENSG0000023	LINC01747	lncRNA	1.72851332	0.44709683	NA
ENSG0000026	RP11-96K19.4	lncRNA	1.72810357	0.0001463	0.00120087

ENSG0000028	RP11-46J23.2	lncRNA	1.72793108	0.29349432	NA
ENSG0000023	AC003104.1	lncRNA	1.72790284	0.00216913	0.01046672
ENSG0000023	PTPRN2-AS1	lncRNA	1.72765354	0.00104824	0.00594385
ENSG0000027	MARCKS	protein_codir	1.72720495	1.91E-08	6.02E-07
ENSG0000027	LLNLR-246C6.	lncRNA	1.72701317	0.3693427	NA
ENSG0000018	CSF1R	protein_codir	1.72621273	5.16E-05	0.00050458
ENSG0000026	RP11-156L14.	lncRNA	1.72613831	0.21328534	0.35302577
ENSG0000018	TAFA3	protein_codir	1.72545133	0.03772487	0.09690857
ENSG0000027	RP4-697K14.1	lncRNA	1.72516158	0.30709597	NA
ENSG0000022	C4B	protein_codir	1.72482461	0.00036731	0.00255028
ENSG0000016	MS4A14	protein_codir	1.72461704	0.00076302	0.00461149
ENSG0000021	TDRD15	protein_codir	1.72459891	0.16479886	0.29205931
ENSG0000028	CTC-789J18.1	lncRNA	1.72361417	0.15078256	0.27330451
ENSG0000012	SARDH	protein_codir	1.72358687	3.65E-05	0.00037684
ENSG0000018	CCDC73	protein_codir	1.72355876	0.38521186	0.53843884
ENSG0000028	AC025442.4	lncRNA	1.72292373	0.00518295	0.02087692
ENSG0000011	TSPAN11	protein_codir	1.72269746	1.05E-05	0.00013367
ENSG0000016	TMC4	protein_codir	1.72231194	0.00024557	0.00182868
ENSG0000025	RP11-490G2.2	lncRNA	1.72223441	0.53776434	NA
ENSG0000022	LINC02813	lncRNA	1.72175641	0.36315212	NA
ENSG0000016	CLEC4D	protein_codir	1.72149418	0.00588573	0.02298373
ENSG0000016	SHOC1	protein_codir	1.72128259	0.24589895	NA
ENSG0000027	CNNM3-DT	lncRNA	1.72038024	0.0108544	0.03717278
ENSG0000018	KIR2DL4	protein_codir	1.71988348	0.01343538	0.04373941
ENSG0000026	RP11-297C4.2	lncRNA	1.71938331	0.45862312	NA
ENSG0000028	RP11-1108I11	lncRNA	1.71840644	0.45319846	NA
ENSG0000017	LRG1	protein_codir	1.7182219	9.26E-05	0.00081476
ENSG0000013	IL2RA	protein_codir	1.7177273	3.04E-05	0.00032409
ENSG0000017	LINC00311	lncRNA	1.71767775	0.12019774	0.23127167
ENSG0000028	RP11-248M19	lncRNA	1.71670194	0.12951151	0.24445486
ENSG0000013	STAMBPL1	protein_codir	1.71660513	1.43E-07	3.41E-06
ENSG0000028	RP13-122B23.	protein_codir	1.71647827	0.02964959	0.08077175
ENSG0000015	LYPD1	protein_codir	1.71643459	8.97E-05	0.00079499
ENSG0000020	ZNF814	protein_codir	1.71639433	5.08E-07	1.02E-05
ENSG0000014	KIRREL3	protein_codir	1.71631884	0.00201204	0.0098766
ENSG0000025	RP11-252C15.	lncRNA	1.71625027	0.45637217	NA
ENSG0000015	CNTNAP4	protein_codir	1.71603637	0.03351846	0.08862084
ENSG0000022	LINC01344	lncRNA	1.71571519	0.36489226	NA
ENSG0000025	RP11-64B16.4	lncRNA	1.71500847	0.37469164	NA
ENSG0000008	OAS1	protein_codir	1.71449203	0.00042468	0.00286118
ENSG0000018	GJD3	protein_codir	1.7144612	2.61E-07	5.73E-06
ENSG0000011	BSPRY	protein_codir	1.71429244	0.00357197	0.0155342
ENSG0000014	LINC00266-1	lncRNA	1.71363811	0.02001213	0.0593318
ENSG0000016	CCDC178	protein_codir	1.71348094	0.00058108	0.00369548
ENSG0000025	RP11-468E2.6	protein_codir	1.71336856	0.01900305	0.05703187
ENSG0000012	IL22	protein_codir	1.71323874	0.54677904	NA
ENSG0000022	LINC02774	lncRNA	1.71317408	0.59892597	NA

ENSG0000028	RP11-359N11	lncRNA	1.71309275	0.05089642	0.1221536
ENSG0000016	DIRAS2	protein_codir	1.71284009	0.04560174	0.11230513
ENSG0000027	RP1-309K20.6	protein_codir	1.71280079	0.59900643	NA
ENSG0000019	ACSL5	protein_codir	1.71277737	1.10E-07	2.73E-06
ENSG0000019	PDCD1LG2	protein_codir	1.71261711	2.79E-07	6.06E-06
ENSG000002	KCNG1	protein_codir	1.71079174	0.00314892	0.01404377
ENSG0000016	NEURL3	protein_codir	1.71040208	0.04836702	0.11740021
ENSG0000016	CAVIN2	protein_codir	1.71001032	0.00033188	0.0023426
ENSG0000020	FCGR3A	protein_codir	1.70986907	0.00143144	0.00759874
ENSG0000017	LY6H	protein_codir	1.70952893	0.03385566	0.08930172
ENSG0000014	WNT9A	protein_codir	1.70938284	0.00604989	0.02347397
ENSG0000023	C11orf94	protein_codir	1.70798503	0.08703549	0.1817266
ENSG0000020	TRIM71	protein_codir	1.70798067	0.11514421	0.22401091
ENSG0000017	ZNF154	protein_codir	1.70759406	2.76E-06	4.29E-05
ENSG0000011	OLFM3	protein_codir	1.70755571	0.45504028	NA
ENSG0000018	AC002398.9	protein_codir	1.70731414	0.30312831	0.45440455
ENSG0000016	SMIM43	protein_codir	1.70649476	0.01706054	0.05253202
ENSG0000027	RP11-358B23.	lncRNA	1.70648848	0.00089535	0.00524489
ENSG0000022	RP4-712E4.1	lncRNA	1.70634888	0.315791	NA
ENSG0000025	RP1-68D18.2	lncRNA	1.70627852	0.00405658	0.01723206
ENSG0000022	AC083900.1	lncRNA	1.70607205	0.38397551	NA
ENSG0000014	SCN2B	protein_codir	1.70605819	0.00139345	0.00743588
ENSG0000010	HRH3	protein_codir	1.70574687	0.54848318	NA
ENSG0000016	CLMN	protein_codir	1.70558773	2.69E-08	8.11E-07
ENSG0000022	MROCKI	lncRNA	1.70543808	0.00162618	0.00840477
ENSG0000019	LRRC8B	protein_codir	1.70521191	1.19E-06	2.13E-05
ENSG0000010	NMU	protein_codir	1.70375059	0.17399637	0.30399372
ENSG0000020	RP11-807H22	lncRNA	1.70351086	0.26059576	NA
ENSG0000023	LINC00167	lncRNA	1.70304364	0.20020406	0.33686483
ENSG0000015	NOCT	protein_codir	1.70221536	9.62E-05	0.000841
ENSG0000014	NR1I3	protein_codir	1.70193415	0.45849093	NA
ENSG0000008	CDH7	protein_codir	1.70151538	0.12993638	0.24500566
ENSG0000027	RP11-70D24.4	lncRNA	1.7014977	0.16556464	0.29304039
ENSG0000017	LRRC8C	protein_codir	1.70128875	1.25E-10	8.52E-09
ENSG0000010	ODAM	protein_codir	1.70113466	0.25059711	NA
ENSG0000020	XKR4	protein_codir	1.70079967	0.00131527	0.00711889
ENSG0000016	TRIM17	protein_codir	1.70071702	0.00301134	0.01354173
ENSG0000027	AC015849.13	lncRNA	1.70016854	0.18573273	0.31901422
ENSG0000019	OR5B21	protein_codir	1.69994095	0.26575058	NA
ENSG0000013	E2F5	protein_codir	1.69971288	9.45E-06	0.00012253
ENSG0000016	IHH	protein_codir	1.69856563	0.12160981	0.23343268
ENSG0000027	RP11-20G13.5	lncRNA	1.69829141	0.07990692	0.17045554
ENSG0000028	CTD-2313D3.2	lncRNA	1.697831	0.3914659	NA
ENSG0000013	MAS1	protein_codir	1.69687707	0.06483138	0.14670186
ENSG0000013	ABHD17C	protein_codir	1.69632465	6.94E-07	1.34E-05
ENSG0000027	RP13-49I15.6	lncRNA	1.69621444	0.29819787	0.44906086
ENSG0000025	LINC00589	lncRNA	1.69617849	0.30342026	NA

ENSG0000018	C22orf34	lncRNA	1.69590578	5.18E-08	1.43E-06
ENSG0000017	RND1	protein_codir	1.69527637	0.02381046	0.06791747
ENSG0000025	LINC02389	lncRNA	1.69522951	0.31372882	NA
ENSG0000020	LINC02520	lncRNA	1.6949614	0.36851167	NA
ENSG0000027	RP3-395M20.	lncRNA	1.69495927	3.25E-05	0.00034146
ENSG0000024	LINC00964	lncRNA	1.6939161	0.21566907	0.35582669
ENSG0000010	RASSF2	protein_codir	1.6932068	4.03E-07	8.34E-06
ENSG0000012	FAM209A	protein_codir	1.69313265	0.0220474	0.06394509
ENSG0000027	ABC7-424044	protein_codir	1.69291552	1.30E-05	0.00016019
ENSG0000016	DDIAS	protein_codir	1.69251243	0.00010428	0.00090034
ENSG0000028	RP11-90D4.8	lncRNA	1.69201165	0.11367971	0.22188736
ENSG0000000	ASB4	protein_codir	1.69119363	0.2985683	0.44947166
ENSG0000016	ONECUT1	protein_codir	1.69103516	0.45974383	NA
ENSG0000011	MS4A6A	protein_codir	1.69045308	2.20E-05	0.00024807
ENSG0000022	RP11-423C15.	lncRNA	1.69035785	0.25139042	NA
ENSG0000013	TEX15	protein_codir	1.69010246	0.12698305	0.24098337
ENSG0000018	ZBED6CL	protein_codir	1.69009086	3.52E-05	0.00036541
ENSG0000025	RP1-78O14.1	lncRNA	1.68932802	0.01691197	0.05220839
ENSG0000025	RP11-12J10.3	protein_codir	1.68927981	0.00135398	0.00727428
ENSG0000027	H2BC10	protein_codir	1.68919913	0.05113667	0.12247613
ENSG0000019	THEM5	protein_codir	1.68909284	0.4509754	NA
ENSG0000024	FLJ12825	lncRNA	1.6890577	0.10717414	0.21244078
ENSG0000016	ENTPD3	protein_codir	1.68883371	0.00016853	0.00134883
ENSG0000021	TAS2R50	protein_codir	1.68865381	0.08053386	0.17156539
ENSG0000022	ARSD-AS1	lncRNA	1.68863837	0.36855552	NA
ENSG0000016	CLEC1B	protein_codir	1.68835554	0.42021975	0.57256522
ENSG0000027	RP3-508I15.2	lncRNA	1.68832174	0.00132944	0.0071801
ENSG0000028	PCBP3-OT1	lncRNA	1.68830213	0.03515178	0.09184724
ENSG0000028	RP11-240L7.5	protein_codir	1.68808301	0.02997252	0.08140853
ENSG0000019	OR2L3	protein_codir	1.68779366	0.55042459	NA
ENSG0000025	CTD-2655K5.1	lncRNA	1.6875377	0.30066217	NA
ENSG0000022	LINC01754	lncRNA	1.68688209	0.14223599	0.2618515
ENSG0000000	CACNG3	protein_codir	1.6867657	0.30126162	NA
ENSG0000014	ZMYND15	protein_codir	1.68525057	7.49E-05	0.0006858
ENSG0000014	CDKN2A	protein_codir	1.6850728	0.00070508	0.00433014
ENSG0000015	TSC22D3	protein_codir	1.68505566	3.15E-05	0.00033374
ENSG0000025	MIR3945HG	lncRNA	1.68503402	0.00715449	0.0267572
ENSG0000016	SLC15A2	protein_codir	1.68470757	0.00040465	0.00276005
ENSG0000027	H2AC12	protein_codir	1.68443895	0.05445836	0.12830785
ENSG0000025	RP11-134N1.2	lncRNA	1.68414806	0.10055109	0.20253474
ENSG0000028	TCAF2C	protein_codir	1.68350244	0.25227937	0.39842273
ENSG0000015	CACNA2D4	protein_codir	1.68290042	0.00010055	0.00087255
ENSG0000022	RP11-764K9.1	lncRNA	1.68269978	0.09304638	0.1910548
ENSG0000009	COL9A3	protein_codir	1.68259956	0.0005754	0.00366607
ENSG0000011	HELLS	protein_codir	1.68229057	9.60E-05	0.00083995
ENSG0000016	PTTG1	protein_codir	1.68203154	0.00042307	0.00285482
ENSG0000024	IL12A-AS1	lncRNA	1.68193435	0.16477627	0.29205931

ENSG0000008	IL12RB2	protein_codir	1.68181227	8.50E-06	0.00011257
ENSG0000017	MIR31HG	lncRNA	1.68180675	0.03911336	0.0997333
ENSG0000024	RP11-555M1.	lncRNA	1.68102713	0.54969247	NA
ENSG0000016	ACER1	protein_codir	1.68091569	0.03842604	0.09830744
ENSG0000012	SH3TC1	protein_codir	1.68084974	1.94E-07	4.41E-06
ENSG0000014	GINS4	protein_codir	1.68049484	2.86E-06	4.42E-05
ENSG0000025	LINC02365	lncRNA	1.67997644	0.30694255	NA
ENSG0000023	DLX6-AS1	lncRNA	1.67962998	0.03555295	0.09266513
ENSG0000026	CORO7	protein_codir	1.67946971	2.33E-09	1.01E-07
ENSG0000028	RP11-49I4.5	lncRNA	1.67942546	0.24850369	NA
ENSG0000025	RP11-17A4.2	lncRNA	1.67931788	0.37640171	NA
ENSG0000023	CCDC192	protein_codir	1.67849937	0.40441408	0.55738108
ENSG0000026	RP11-16E23.3	lncRNA	1.67844718	0.28455173	0.43414456
ENSG0000025	AP000442.1	lncRNA	1.67815464	0.03563503	0.092809
ENSG0000013	COX4I2	protein_codir	1.67755919	4.32E-06	6.25E-05
ENSG0000013	CELA1	protein_codir	1.67735578	0.25438551	NA
ENSG0000001	SLC11A1	protein_codir	1.67703301	0.00154666	0.00806413
ENSG0000022	PTPRK-AS1	lncRNA	1.6755892	0.60704703	NA
ENSG0000012	GRM4	protein_codir	1.675352	0.14816629	0.26998488
ENSG0000019	PELI1	protein_codir	1.67514508	1.89E-05	0.00021917
ENSG0000025	SMIM35	protein_codir	1.67504569	0.00825415	0.0299284
ENSG0000016	FOXI1	protein_codir	1.67455892	0.46684158	NA
ENSG0000027	LINC00547	lncRNA	1.67440481	0.47241609	NA
ENSG0000017	HSD11B2	protein_codir	1.67383715	0.01310842	0.04292289
ENSG0000018	GSAP	protein_codir	1.67277258	9.42E-06	0.0001221
ENSG0000026	RP11-739B23.	lncRNA	1.6719649	0.38142269	NA
ENSG0000010	ACTR3C	protein_codir	1.67138766	0.0002991	0.00214434
ENSG0000028	RP11-417E7.3	lncRNA	1.67138349	0.05359054	0.12678121
ENSG0000025	RP6-65G23.3	lncRNA	1.67115065	0.11390453	0.22220958
ENSG0000012	CCRL2	protein_codir	1.67089808	3.29E-06	5.01E-05
ENSG0000018	ESPN	protein_codir	1.67086563	0.02381614	0.06791965
ENSG0000025	LINC02207	lncRNA	1.67081702	0.00560044	0.02214201
ENSG0000024	CTC-505O3.3	lncRNA	1.67038079	0.26612021	0.4137139
ENSG0000024	WDR86-AS1	lncRNA	1.67036592	0.0012683	0.00691206
ENSG0000000	DNAH9	protein_codir	1.6697121	0.00442822	0.01844676
ENSG0000028	RP11-15H20.8	protein_codir	1.66923625	0.12744812	0.24161694
ENSG0000025	LINC02295	lncRNA	1.66914287	0.46738121	NA
ENSG0000015	CLEC1A	protein_codir	1.66866986	2.80E-05	0.00030323
ENSG0000014	DOK2	protein_codir	1.66842739	5.96E-05	0.0005697
ENSG0000026	AC115522.3	lncRNA	1.66797689	0.08391374	0.17676156
ENSG0000013	VSTM2L	protein_codir	1.66791375	NA	NA
ENSG0000012	KLK14	protein_codir	1.66783299	0.00250551	0.01171729
ENSG0000018	LRRC70	protein_codir	1.66780845	2.43E-06	3.88E-05
ENSG0000026	NARF-AS2	lncRNA	1.667415	5.27E-07	1.05E-05
ENSG0000014	CRB2	protein_codir	1.6666823	0.02048799	0.06037966
ENSG0000008	ACHE	protein_codir	1.66651415	0.00092267	0.00537186
ENSG0000024	SERPINB10	protein_codir	1.66621886	0.13235102	0.24861473

ENSG0000023	LINC01031	lncRNA	1.66530958	0.15041031	0.27282673
ENSG0000022	RFPL1S	lncRNA	1.6652322	0.00012834	0.00107577
ENSG0000012	CCDC18	protein_codir	1.66502727	9.78E-09	3.45E-07
ENSG0000022	RP4-644L1.2	lncRNA	1.66480854	0.09994918	0.20169477
ENSG0000028	RP11-496H1.3	lncRNA	1.66401219	0.01384222	0.04475791
ENSG0000025	RP11-815J21.	lncRNA	1.66248934	0.01188368	0.03979959
ENSG0000023	AC024084.1	lncRNA	1.66234661	0.206859	NA
ENSG0000028	CTD-2008P7.1	lncRNA	1.66232434	0.02026922	0.05991394
ENSG0000027	RP11-387D10	lncRNA	1.66212141	0.27225625	NA
ENSG000000(	ICA1	protein_codir	1.6617612	5.67E-08	1.54E-06
ENSG000002(	SUGCT-AS1	lncRNA	1.66174098	0.01369482	0.04442172
ENSG0000025	RP11-830F9.5	lncRNA	1.66164109	0.0816767	0.1733719
ENSG0000024	RP11-758P17.	lncRNA	1.66145299	0.32948329	NA
ENSG0000019	GK	protein_codir	1.66111346	1.33E-06	2.33E-05
ENSG0000016	VCAM1	protein_codir	1.66050715	5.54E-07	1.10E-05
ENSG0000012	HVCN1	protein_codir	1.6596235	0.00024773	0.00184233
ENSG0000022	RBM15-AS1	lncRNA	1.65957615	0.03480215	0.09114998
ENSG0000012	MMP19	protein_codir	1.65943099	0.00176919	0.00894014
ENSG0000021	C19orf38	protein_codir	1.65878557	4.16E-05	0.00041864
ENSG0000018	RELN	protein_codir	1.65876358	0.01125702	0.03819174
ENSG0000014	KCNH1	protein_codir	1.65864564	0.01885279	0.05667354
ENSG0000026	RP11-667K14.	lncRNA	1.6585601	0.01233758	0.04093069
ENSG0000026	CTD-3128G10	lncRNA	1.65849385	0.05731992	0.1332015
ENSG0000025	AF131215.3	lncRNA	1.65780666	0.03070586	0.08289888
ENSG0000023	AC073254.1	lncRNA	1.65777614	0.56488395	NA
ENSG0000024	RP11-115N4.1	lncRNA	1.65765776	0.06593914	0.14858766
ENSG0000028	RP11-431H16	lncRNA	1.6564187	0.0264487	0.0737281
ENSG0000024	C4A	protein_codir	1.65568561	0.00041088	0.00279454
ENSG0000024	AC132217.4	lncRNA	1.65534223	0.00971922	0.03403391
ENSG0000022	LINC01357	lncRNA	1.65516493	0.05983653	0.13767785
ENSG000002(	HLA-C	protein_codir	1.65441289	3.25E-11	2.81E-09
ENSG0000028	RP11-1430O6	lncRNA	1.65377484	0.13224512	0.24849379
ENSG0000019	F5	protein_codir	1.6536409	0.00084669	0.00501407
ENSG0000028	CTD-3074O7.1	lncRNA	1.65356591	0.18320605	0.31578577
ENSG0000028	RP11-305O20	lncRNA	1.65349651	0.5588235	NA
ENSG0000013	F12	protein_codir	1.65323066	0.00453386	0.0187885
ENSG0000025	LINC01608	lncRNA	1.65294294	0.10331753	0.20676981
ENSG000001(	ASPA	protein_codir	1.65263449	8.72E-05	0.00077725
ENSG0000011	NR5A2	protein_codir	1.65227999	0.00042345	0.00285611
ENSG0000025	ALG1L2	protein_codir	1.65226426	0.19799133	0.33421754
ENSG0000014	SSH2	protein_codir	1.65203279	2.47E-05	0.00027361
ENSG0000011	PROX2	protein_codir	1.65192282	0.12628865	0.24016737
ENSG0000022	RP11-426L16.	lncRNA	1.6515089	0.46735263	NA
ENSG0000018	PLA2G2C	protein_codir	1.65101015	0.01041114	0.03595346
ENSG0000013	KIF1A	protein_codir	1.65039381	0.03992495	0.1013629
ENSG000001(	CCN4	protein_codir	1.65029147	6.13E-06	8.49E-05
ENSG0000015	VENTX	protein_codir	1.65028361	6.88E-05	0.00064059

ENSG0000010	EBF3	protein_codir	1.64981172	8.27E-06	0.00010985
ENSG0000018	CTAG1B	protein_codir	1.64945685	0.42560176	0.57747694
ENSG0000026	RP11-2C24.4	lncRNA	1.64864827	0.12492766	0.23816275
ENSG0000019	B3GNT6	protein_codir	1.64788759	0.33531596	NA
ENSG0000021	TTTY2	lncRNA	1.64784725	0.47179013	NA
ENSG0000023	ACBD3-AS1	lncRNA	1.64762852	0.33134009	NA
ENSG0000019	ZNF107	protein_codir	1.64754656	2.70E-05	0.00029426
ENSG0000016	CPA6	protein_codir	1.64694126	0.02772632	0.07655712
ENSG0000027	RP11-284F21.	lncRNA	1.64631376	0.00142982	0.00759359
ENSG0000025	HIF1A-AS1	lncRNA	1.64618471	0.13321935	0.24974673
ENSG0000023	AC026202.3	lncRNA	1.64591632	0.00162036	0.00838097
ENSG0000025	RP11-864J10.4	lncRNA	1.64554821	0.2998721	0.45091122
ENSG0000024	PCP4L1	protein_codir	1.64549443	0.03394509	0.08948778
ENSG0000026	CTD-2588C8.8	lncRNA	1.64506771	0.56205856	NA
ENSG0000011	WNT5A	protein_codir	1.64468358	0.00285048	0.01294907
ENSG0000013	HAVCR2	protein_codir	1.64421574	0.00019264	0.00149752
ENSG0000017	GDPD4	protein_codir	1.64408873	0.480165	NA
ENSG0000024	UGT1A10	protein_codir	1.64400289	0.56308881	NA
ENSG0000028	RP11-708D7.1	lncRNA	1.64314169	0.08735185	0.18218199
ENSG0000028	RP11-521H5.1	lncRNA	1.64294767	0.20357662	0.34089889
ENSG0000000	MMP25	protein_codir	1.6417317	0.00813169	0.02961461
ENSG0000025	RP11-1149O2	lncRNA	1.64170528	0.12900207	0.24377846
ENSG0000028	RP11-7F17.10	lncRNA	1.64123096	0.17679847	0.30721783
ENSG0000028	CTC-448F2.8	lncRNA	1.64087471	0.19874409	0.33503489
ENSG0000026	CTD-3096M3.	lncRNA	1.64043514	0.39701047	NA
ENSG0000025	RP11-23P13.4	lncRNA	1.64031865	0.61470447	NA
ENSG0000013	APLNR	protein_codir	1.63990924	1.66E-05	0.00019644
ENSG0000005	HERPUD1	protein_codir	1.63978176	4.43E-08	1.25E-06
ENSG0000018	CHST13	protein_codir	1.63959918	0.00062383	0.00391416
ENSG0000022	AC105393.2	lncRNA	1.63941797	0.48143181	NA
ENSG0000011	BTN3A3	protein_codir	1.63882075	3.98E-06	5.87E-05
ENSG0000016	LAIR1	protein_codir	1.6387952	8.17E-05	0.00073714
ENSG0000027	RP11-29B2.6	lncRNA	1.63875229	0.14850856	0.27051417
ENSG0000028	RP11-415F23.	lncRNA	1.63869507	0.02299498	0.06607587
ENSG0000027	MILR1	protein_codir	1.63868923	6.87E-05	0.00063988
ENSG0000025	RP11-1070N1	lncRNA	1.63843658	0.28521453	0.43474754
ENSG0000028	RP5-964C11.1	lncRNA	1.63758395	0.29664857	0.44714219
ENSG0000026	RP11-661C3.2	lncRNA	1.63650058	0.31778999	NA
ENSG0000014	ECE2	protein_codir	1.63600742	0.27108151	0.41905587
ENSG0000017	ERN1	protein_codir	1.63546603	5.49E-08	1.50E-06
ENSG0000025	RP11-108K14.	protein_codir	1.63481143	0.00055727	0.00357037
ENSG0000028	RP11-177B4.3	lncRNA	1.63303884	0.0528199	0.12535536
ENSG0000027	GS1-114I9.1	lncRNA	1.63289287	0.02945634	0.08035398
ENSG0000017	STARD5	protein_codir	1.63192799	2.24E-05	0.0002523
ENSG0000016	AQP3	protein_codir	1.63101745	5.39E-05	0.000524
ENSG0000028	RP11-394J1.3	lncRNA	1.63101739	0.07643165	0.16516124
ENSG0000018	SH2D7	protein_codir	1.63086924	0.38866281	NA

ENSG0000016	LRRIQ3	protein_codir	1.63072228	0.00385748	0.01652096
ENSG0000018	WFDC10B	protein_codir	1.63064498	0.24877665	0.3944438
ENSG0000010	TOX3	protein_codir	1.63007263	0.18471321	0.31778438
ENSG0000025	CTD-2003C8.2	lncRNA	1.63003768	0.00747941	0.02773275
ENSG0000013	PKIB	protein_codir	1.63000124	0.00546943	0.02175484
ENSG0000024	PARAL1	lncRNA	1.6298078	0.30891182	0.46051495
ENSG0000013	CCNB1	protein_codir	1.62978376	0.00017552	0.00139068
ENSG0000027	RP11-861A13	lncRNA	1.62973297	0.00230254	0.01098191
ENSG0000016	C11orf65	protein_codir	1.62888239	0.00762742	0.028138
ENSG0000027	RP1-170O19.2	lncRNA	1.62858992	0.01701675	0.0524264
ENSG0000026	CTB-102L5.7	lncRNA	1.62842933	0.00462392	0.01911581
ENSG0000023	DNM3-IT1	lncRNA	1.62829331	0.40604522	NA
ENSG0000024	AC110769.3	lncRNA	1.62822019	0.30094456	0.4519896
ENSG0000025	TMEM179	protein_codir	1.62813726	0.12635862	0.24022749
ENSG0000024	RP11-292E2.1	lncRNA	1.62714722	0.48248795	NA
ENSG0000017	LINC00469	lncRNA	1.627113	0.31453686	NA
ENSG0000015	VEGFC	protein_codir	1.62684094	3.97E-08	1.14E-06
ENSG0000018	DOCK8-AS1	lncRNA	1.62650917	0.00129896	0.00705136
ENSG0000017	COL24A1	protein_codir	1.62583709	0.00015631	0.00126643
ENSG0000015	CCDC173	protein_codir	1.62534536	0.02593954	0.07263281
ENSG0000013	PRR5L	protein_codir	1.62512085	3.09E-05	0.00032803
ENSG0000010	GPR50	protein_codir	1.62475516	0.29219898	0.44222114
ENSG0000027	OR10A6	protein_codir	1.62428441	0.47829884	NA
ENSG0000015	HHEX	protein_codir	1.6242553	0.00017203	0.00137166
ENSG0000028	RP4-669P10.2	lncRNA	1.62375902	0.05051852	0.12149443
ENSG0000027	RP11-16E23.5	lncRNA	1.62291057	0.00385088	0.01650037
ENSG0000012	EREG	protein_codir	1.62236261	0.01759912	0.05375888
ENSG0000012	RIPK3	protein_codir	1.62176663	1.94E-05	0.00022363
ENSG0000018	IGFL3	protein_codir	1.62039361	0.47313363	NA
ENSG0000017	NABP1	protein_codir	1.6183232	1.16E-06	2.09E-05
ENSG0000027	HEXD-IT1	lncRNA	1.61816154	0.01220075	0.04060966
ENSG0000010	E2F1	protein_codir	1.6175399	0.00028755	0.00208458
ENSG0000019	SRGAP3	protein_codir	1.6171504	2.29E-05	0.00025678
ENSG0000025	RP11-360L9.7	lncRNA	1.61704202	0.28285893	NA
ENSG0000025	LINC00637	lncRNA	1.61621233	0.49475388	NA
ENSG0000024	AC012442.5	lncRNA	1.61552273	0.07602573	0.16456353
ENSG0000025	CLEC5A	protein_codir	1.61450779	0.0019508	0.00963021
ENSG0000022	ACAP2-IT1	lncRNA	1.61440345	0.03225982	0.08596393
ENSG0000011	SERPINI2	protein_codir	1.61431096	0.11976801	0.23065529
ENSG0000016	TM4SF18	protein_codir	1.61422905	1.72E-05	0.00020155
ENSG0000022	CSE1L-AS1	lncRNA	1.61346984	0.3448945	NA
ENSG0000010	DLX5	protein_codir	1.6134414	0.00178587	0.00900683
ENSG0000022	RP11-253D19	lncRNA	1.61331942	0.40236777	NA
ENSG0000012	HROB	protein_codir	1.61260195	1.34E-05	0.00016506
ENSG0000023	SERPINB9P1	lncRNA	1.61221852	0.02368404	0.06759877
ENSG0000010	PYCARD	protein_codir	1.61161141	6.35E-06	8.72E-05
ENSG0000027	RP11-104H15	lncRNA	1.61141544	0.13565787	0.25277716

ENSG0000025	LINC01267	lncRNA	1.61080654	0.35498768	0.50862477
ENSG0000018	LPAR5	protein_codir	1.61052235	0.0007979	0.00477943
ENSG0000019	MB	protein_codir	1.61046812	0.14315394	0.26315552
ENSG0000008	TFAP2C	protein_codir	1.61007335	0.00017214	0.00137212
ENSG0000023	DCTN1-AS1	lncRNA	1.6097829	0.38609596	NA
ENSG0000013	FAM222A	protein_codir	1.60976431	0.00022169	0.00168078
ENSG0000009	SMIM24	protein_codir	1.60966762	0.08704059	0.1817266
ENSG0000027	CH507-513H4	lncRNA	1.60853819	9.48E-05	0.00083234
ENSG0000028	RP13-52K8.2	lncRNA	1.60851639	0.30883868	0.4604407
ENSG0000011	IGSF21	protein_codir	1.60841511	0.0003228	0.00228607
ENSG0000027	CTD-3203P2.3	lncRNA	1.60807306	0.01167039	0.03927576
ENSG0000018	SSR4	protein_codir	1.60797655	5.87E-06	8.18E-05
ENSG0000025	RP11-177H2.2	lncRNA	1.60764003	0.3517816	0.50530639
ENSG0000028	RP11-305P14.	lncRNA	1.60693394	0.20185007	0.3389118
ENSG0000022	AP001468.58	lncRNA	1.60617343	0.62214564	NA
ENSG0000025	RP11-488C13.	lncRNA	1.60578536	0.03362797	0.0888383
ENSG0000023	USP12-AS2	lncRNA	1.60575058	0.48449946	NA
ENSG0000013	PPP1R1B	protein_codir	1.60546422	0.01773895	0.05410791
ENSG0000025	CTD-2066L21.	lncRNA	1.60538231	0.43872938	0.58984158
ENSG0000018	SOCS3	protein_codir	1.60405998	0.00041764	0.00282452
ENSG0000010	SIGLEC5	protein_codir	1.60306154	0.00911611	0.03240086
ENSG0000014	PLA2G7	protein_codir	1.60267735	0.01461363	0.04674756
ENSG0000010	HCK	protein_codir	1.60232877	3.38E-06	5.13E-05
ENSG0000028	AC012488.3	protein_codir	1.60166299	0.62313034	NA
ENSG0000014	SECTM1	protein_codir	1.60165028	2.16E-07	4.85E-06
ENSG0000027	RP4-753F5.1	lncRNA	1.60107835	0.38188712	0.53538836
ENSG0000020	VWA7	protein_codir	1.60073664	0.00013974	0.00115525
ENSG0000013	C1QTNF6	protein_codir	1.59974213	0.00015223	0.00124069
ENSG0000022	AC010084.1	lncRNA	1.59961272	0.03983519	0.10119083
ENSG0000022	RP4-633I8.4	lncRNA	1.59928956	0.21219685	0.35175111
ENSG0000009	CDC7	protein_codir	1.59870995	1.79E-05	0.00020855
ENSG0000026	RP11-665J16.	lncRNA	1.59851692	0.573907	NA
ENSG0000024	RP11-432B6.3	protein_codir	1.59817307	0.1905965	0.32523634
ENSG0000020	RP11-473M20	lncRNA	1.59749666	0.57127354	NA
ENSG0000020	VCX3B	protein_codir	1.59693928	0.57714167	NA
ENSG0000015	LRRC36	protein_codir	1.59682233	0.0764797	0.1652354
ENSG0000017	TRIB1	protein_codir	1.59658216	0.00138957	0.00741783
ENSG0000018	CLDN4	protein_codir	1.59610616	0.00427945	0.01796328
ENSG0000019	DAPK1	protein_codir	1.59509974	1.24E-05	0.00015426
ENSG0000026	RP11-923I11.4	lncRNA	1.59507796	0.27999786	NA
ENSG0000024	GDNF-AS1	lncRNA	1.59477802	0.11368586	0.22188736
ENSG0000013	HIGD1B	protein_codir	1.59357332	0.00434374	0.01816415
ENSG0000027	RP11-759A24	lncRNA	1.59333811	0.4261913	0.57793615
ENSG0000013	CASP1	protein_codir	1.59255004	2.77E-05	0.0003008
ENSG0000027	CITF22-49E9.3	lncRNA	1.59248807	0.00221482	0.01064625
ENSG0000028	RP11-217L21.	lncRNA	1.59236123	0.41138023	NA
ENSG0000016	SPINT1	protein_codir	1.59189369	0.00207146	0.01009771

ENSG0000013	PLAAT4	protein_codir	1.59161236	1.72E-07	3.97E-06
ENSG0000015	KCNS2	protein_codir	1.59105985	0.01056107	0.03634418
ENSG0000017	TENM3-AS1	lncRNA	1.59068501	0.01887078	0.05670884
ENSG0000018	C2CD4C	protein_codir	1.59063694	0.00065382	0.00406999
ENSG0000024	SEMA6A-AS2	lncRNA	1.59058076	0.01004892	0.03492592
ENSG0000014	TNFRSF21	protein_codir	1.5903966	9.64E-05	0.0008419
ENSG0000017	DNAI2	protein_codir	1.59033638	0.08061881	0.17171987
ENSG0000011	PEX5L	protein_codir	1.59016847	0.056258	0.13141457
ENSG0000028	LINC02834	lncRNA	1.58951232	0.23885622	NA
ENSG0000026	DISC1FP1	lncRNA	1.58937213	0.1119776	0.21953587
ENSG0000010	LAMA1	protein_codir	1.58935027	0.01013753	0.03519339
ENSG0000010	PDGFB	protein_codir	1.58917153	1.36E-05	0.00016676
ENSG0000010	ALOX12	protein_codir	1.58884878	0.13024773	0.24549216
ENSG0000018	HCAR2	protein_codir	1.58878012	0.00472055	0.01940489
ENSG0000019	CCDC189	protein_codir	1.58848924	0.00024934	0.00185188
ENSG0000016	GBX2	protein_codir	1.58811893	0.20569505	0.34371694
ENSG0000018	OR2G6	protein_codir	1.58806301	0.50086139	0.64598606
ENSG0000026	LINC01977	lncRNA	1.58805121	0.21870826	0.35920331
ENSG0000025	RP11-338E21.	lncRNA	1.58761105	0.62619958	NA
ENSG0000028	CTB-30L5.3	lncRNA	1.58607556	0.05266285	0.12511815
ENSG0000019	RP11-573D15	lncRNA	1.58568732	0.06465789	0.14640245
ENSG0000016	CHRM1	protein_codir	1.58545217	0.57834109	NA
ENSG0000022	LINC02668	lncRNA	1.58506358	0.04877867	0.11823317
ENSG0000027	RP4-569D19.8	lncRNA	1.58487425	0.43152171	NA
ENSG0000027	H2AB1	protein_codir	1.58448967	0.12766911	0.24190405
ENSG0000010	BMP7	protein_codir	1.58445253	0.03840496	0.09826777
ENSG0000023	RP11-379K17.	lncRNA	1.58405549	0.0020405	0.00997465
ENSG0000014	CRABP2	protein_codir	1.58382845	0.00103516	0.00588731
ENSG0000016	CCNF	protein_codir	1.5809324	0.00016257	0.00131217
ENSG0000026	CTB-55O6.10	lncRNA	1.58080079	0.23955211	0.38389493
ENSG0000028	RP4-592A1.5	lncRNA	1.58044265	0.28295913	NA
ENSG0000027	CTAGE15	protein_codir	1.58027785	0.19569925	0.33146527
ENSG0000022	LINC01940	lncRNA	1.57968398	0.20596879	0.34398707
ENSG0000022	RP11-536O18	lncRNA	1.57942206	0.00151896	0.0079584
ENSG0000016	B2M	protein_codir	1.57940016	2.53E-10	1.59E-08
ENSG0000028	RP11-764D10	lncRNA	1.57936876	0.11746909	0.22731704
ENSG0000023	RP11-343J3.2	lncRNA	1.57909262	0.11015762	0.2169224
ENSG0000018	IZUMO1	protein_codir	1.57908335	0.09761955	0.19815183
ENSG0000018	RHCE	protein_codir	1.578756	0.00307278	0.0137642
ENSG0000026	LINC00562	lncRNA	1.57846226	0.00949983	0.03342416
ENSG0000018	PRKX	protein_codir	1.57845265	2.12E-07	4.78E-06
ENSG0000015	CDC42SE2	protein_codir	1.57839503	5.46E-08	1.49E-06
ENSG0000010	P2RX1	protein_codir	1.5783629	NA	NA
ENSG0000018	CLDN7	protein_codir	1.57833018	0.00264258	0.01220852
ENSG0000001	TYROBP	protein_codir	1.57799758	3.57E-05	0.0003701
ENSG0000017	SELP	protein_codir	1.57773897	2.34E-05	0.00026115
ENSG0000020	LINC01446	lncRNA	1.57759684	0.35707315	0.51065916

ENSG0000014	ACKR2	protein_codir	1.57659489	0.0021572	0.01041641
ENSG0000023	LUARIS	lncRNA	1.57638546	0.26169358	0.40909988
ENSG0000028	RP11-867O8.9	protein_codir	1.57625268	0.00021277	0.00162202
ENSG0000027	RP11-127I20.8	lncRNA	1.57611297	0.00303919	0.01364918
ENSG0000012	CCDC170	protein_codir	1.57610356	3.01E-05	0.00032179
ENSG0000027	CTD-2227E11.	lncRNA	1.57585597	0.1167164	0.22632867
ENSG0000020	MSH5	protein_codir	1.57582497	0.00029008	0.00209737
ENSG0000015	F11R	protein_codir	1.5752339	1.57E-07	3.70E-06
ENSG0000020	LILRB3	protein_codir	1.57505956	0.00030306	0.00216968
ENSG0000015	RAB39B	protein_codir	1.57488439	0.00017405	0.00138255
ENSG0000016	PLXDC1	protein_codir	1.57483305	0.00011257	0.00096345
ENSG0000026	RP11-32B5.8	lncRNA	1.57312699	0.0292246	0.07989544
ENSG0000004	CAPG	protein_codir	1.57305511	0.00034333	0.0024118
ENSG0000012	KIAA1217	protein_codir	1.57269707	1.05E-05	0.00013385
ENSG0000022	LINC01714	lncRNA	1.57247105	0.48859134	NA
ENSG0000028	LINC00602	lncRNA	1.57173172	0.57966953	NA
ENSG0000025	RP11-20G13.2	lncRNA	1.57168998	0.35222824	NA
ENSG0000011	THBS4	protein_codir	1.57076664	0.02436988	0.06919161
ENSG0000017	TMEM51	protein_codir	1.57044724	9.27E-06	0.00012066
ENSG0000023	JARID2-AS1	lncRNA	1.56985628	0.28653788	0.43612757
ENSG0000028	CTD-2301A4.6	lncRNA	1.56983419	0.00226058	0.01082477
ENSG0000028	RP5-838N20.7	lncRNA	1.56956798	0.63014838	NA
ENSG0000027	RP11-474C8.8	lncRNA	1.56956798	0.63014838	NA
ENSG0000028	RP11-818F20.	lncRNA	1.56956798	0.63014838	NA
ENSG0000021	KRTAP29-1	protein_codir	1.56956798	0.63014838	NA
ENSG0000027	CTA-384D8.36	lncRNA	1.56956798	0.63014838	NA
ENSG0000022	RP11-569A11	lncRNA	1.56956776	0.63014843	NA
ENSG0000024	CTD-2227I18.	lncRNA	1.56956776	0.63014843	NA
ENSG0000022	AC004691.5	lncRNA	1.56956776	0.63014843	NA
ENSG0000019	SLC22A25	protein_codir	1.56956776	0.63014843	NA
ENSG0000025	RP11-73M14.	lncRNA	1.56956776	0.63014843	NA
ENSG0000022	LMO7DN-IT1	lncRNA	1.56956776	0.63014843	NA
ENSG0000026	RP11-1035H1	lncRNA	1.56956776	0.63014843	NA
ENSG0000026	DHX35-DT	lncRNA	1.56956776	0.63014843	NA
ENSG0000026	SOCS3-DT	lncRNA	1.56942103	0.03073295	0.08293543
ENSG0000021	CELA2B	protein_codir	1.56937307	0.42671408	NA
ENSG0000013	STK33	protein_codir	1.56920622	0.00427805	0.01796016
ENSG0000013	CKAP2	protein_codir	1.56916776	1.13E-06	2.06E-05
ENSG0000023	RP11-244N9.4	lncRNA	1.56907846	0.5804125	NA
ENSG0000019	MMP17	protein_codir	1.569006	4.48E-05	0.0004446
ENSG0000020	MAS1L	protein_codir	1.56824935	0.15298042	0.2763272
ENSG0000028	CTB-30L5.2	lncRNA	1.56820365	0.48791168	NA
ENSG0000012	SPINK2	protein_codir	1.56809345	0.03208866	0.08558215
ENSG0000014	VWA5B2	protein_codir	1.5677557	0.03541547	0.092394
ENSG0000027	ZNF30-AS1	lncRNA	1.56767817	0.32161026	0.47377863
ENSG0000016	HK3	protein_codir	1.56752274	0.00067755	0.00419091
ENSG0000018	P2RX2	protein_codir	1.56751055	0.04940613	0.11944106

ENSG0000024	RP11-360F5.3	lncRNA	1.56693119	0.00325168	0.01441826
ENSG0000020	TMEM273	protein_codir	1.56646389	2.62E-06	4.11E-05
ENSG0000012	C22orf24	lncRNA	1.56589055	0.00117492	0.00649933
ENSG0000026	THY1-AS1	lncRNA	1.56561438	0.01774447	0.05411308
ENSG0000017	SUMO4	protein_codir	1.5652874	0.01791479	0.05446412
ENSG0000022	LINC02471	lncRNA	1.56481918	0.58039645	NA
ENSG0000028	RP11-84K19.1	lncRNA	1.56480223	0.5802329	NA
ENSG0000010	LSR	protein_codir	1.56476181	6.50E-08	1.74E-06
ENSG0000011	LRRC31	protein_codir	1.56472369	0.57947609	NA
ENSG0000014	SLC10A6	protein_codir	1.56422847	0.00738798	0.02747857
ENSG0000013	CEMIP2	protein_codir	1.56418214	4.93E-06	7.04E-05
ENSG0000027	CTC-463N11.4	lncRNA	1.56412886	0.09177233	0.18901952
ENSG0000022	HEPN1	protein_codir	1.56375014	0.63142386	NA
ENSG0000016	SIGLEC7	protein_codir	1.56298871	0.00535661	0.02140166
ENSG0000011	MDFI	protein_codir	1.56266797	0.00058856	0.00373531
ENSG0000023	LINC01282	lncRNA	1.56237775	0.5814349	NA
ENSG0000028	RP11-411P18.	lncRNA	1.56221294	0.5794637	NA
ENSG0000016	PRR35	protein_codir	1.56213071	0.57848547	NA
ENSG0000028	RP11-219B17.	lncRNA	1.56206379	0.42892396	NA
ENSG0000025	RP11-461F11.	lncRNA	1.56197793	0.6318126	NA
ENSG0000024	RP11-11N9.4	lncRNA	1.5618856	0.0014285	0.00759043
ENSG0000016	FGF19	protein_codir	1.5618338	0.5749819	NA
ENSG0000009	NLRC4	protein_codir	1.56152054	0.0066995	0.02541212
ENSG0000025	EPHA5-AS1	lncRNA	1.56145907	0.34706089	NA
ENSG0000017	LGALS4	protein_codir	1.56121799	0.01946441	0.05809456
ENSG0000027	RP11-478J18.	lncRNA	1.5606994	0.58314665	NA
ENSG0000026	RP3-431P23.5	lncRNA	1.56065733	0.00233721	0.01111216
ENSG0000025	RP11-38H17.1	lncRNA	1.56061449	0.63211174	NA
ENSG0000018	HECW1-IT1	lncRNA	1.56061143	0.58188059	NA
ENSG0000028	RP11-622I12.1	lncRNA	1.56052605	0.00060493	0.00381814
ENSG0000021	RPTN	protein_codir	1.56046213	0.57974582	NA
ENSG0000024	LINC01093	lncRNA	1.56005955	0.20222154	0.33929757
ENSG0000015	SLC7A7	protein_codir	1.55989768	6.31E-05	0.00059601
ENSG0000020	LY6G6F	protein_codir	1.55960238	0.50122808	NA
ENSG0000016	NBEAL2	protein_codir	1.55940105	6.82E-06	9.28E-05
ENSG0000023	RP11-109P14.	lncRNA	1.55937375	0.58358871	NA
ENSG0000017	DIRAS1	protein_codir	1.55933511	0.00212312	0.0102842
ENSG0000013	RDH16	protein_codir	1.55925522	0.00149104	0.00785027
ENSG0000025	RP11-17A4.3	lncRNA	1.55920737	0.58080637	NA
ENSG0000025	SELENOP	protein_codir	1.55903004	5.49E-05	0.00053178
ENSG0000028	RP5-912I13.2	lncRNA	1.55893813	0.06507058	0.14712386
ENSG0000026	RP11-866E20.	lncRNA	1.55892846	0.57621139	NA
ENSG0000013	IFI44	protein_codir	1.55890655	7.96E-05	0.00072044
ENSG0000005	PSD	protein_codir	1.55839589	0.00091171	0.00531903
ENSG0000017	IRX3	protein_codir	1.55839228	0.00137574	0.00736538
ENSG0000027	CTC-428G20.6	lncRNA	1.55808949	0.00665174	0.02527874
ENSG0000023	RERE-AS1	lncRNA	1.55783745	0.01227352	0.04078086

ENSG0000007	ARHGEF1	protein_codir	1.55734668	3.26E-06	4.97E-05
ENSG000001C	PLEK2	protein_codir	1.55645049	0.00653091	0.02496661
ENSG000001C	TNFRSF10A	protein_codir	1.55617808	1.86E-08	5.91E-07
ENSG0000017	RLN3	protein_codir	1.5560007	0.13156136	0.24742781
ENSG0000019	LINC00173	lncRNA	1.55578368	0.00650271	0.02489081
ENSG0000016	RAPSN	protein_codir	1.55545948	0.03014514	0.08175764
ENSG0000018	DBX2	protein_codir	1.55540542	0.02954319	0.08052727
ENSG0000017	CNBD1	protein_codir	1.55501576	0.49779215	NA
ENSG000001C	SLC1A6	protein_codir	1.55501514	0.49778761	NA
ENSG0000023	AC004160.4	lncRNA	1.55494667	0.42043376	NA
ENSG0000026	CTB-39G8.3	lncRNA	1.5546484	0.50137505	NA
ENSG0000028	RP5-981L23.7	lncRNA	1.55419613	0.0674718	0.15111157
ENSG0000019	DLL1	protein_codir	1.5538071	2.52E-06	3.99E-05
ENSG0000028	CH17-67K23.2	lncRNA	1.55368579	0.00846473	0.03050172
ENSG0000024	LINC00698	lncRNA	1.55365488	0.49741854	NA
ENSG0000026	AC010524.4	lncRNA	1.55357254	0.4965678	NA
ENSG0000028	RP11-543N17	lncRNA	1.55342412	0.49964159	NA
ENSG0000025	RP13-487K5.1	lncRNA	1.55326143	0.04378723	0.10880821
ENSG0000017	CCL11	protein_codir	1.55272964	0.07180763	0.15795848
ENSG0000027	RP11-452C13.	lncRNA	1.55262001	0.04517216	0.11157255
ENSG0000027	NUP153-AS1	lncRNA	1.55210049	7.78E-06	0.00010422
ENSG0000025	RP11-486M23	lncRNA	1.5501597	0.58763575	NA
ENSG0000028	CTB-157D17.2	lncRNA	1.55013489	0.2825025	0.43206816
ENSG0000027	THBS1-AS1	lncRNA	1.54962161	0.09000374	0.18622393
ENSG0000025	PKIA-AS1	lncRNA	1.54959408	0.24788327	NA
ENSG000000C	CAMK1G	protein_codir	1.54865844	0.00636953	0.02449121
ENSG000001C	ANGPT4	protein_codir	1.54771688	0.00254728	0.01184242
ENSG0000006	SYT1	protein_codir	1.54755449	2.17E-05	0.00024563
ENSG0000028	RP11-168F16.	lncRNA	1.54735669	0.08569172	0.17972552
ENSG0000018	TUBA8	protein_codir	1.54701613	0.00214607	0.01037174
ENSG0000011	SLC26A8	protein_codir	1.54608222	0.02172021	0.06316368
ENSG0000016	SLC7A3	protein_codir	1.54586424	0.17525503	0.30535856
ENSG0000022	PIK3IP1-DT	lncRNA	1.54519416	0.03452829	0.09067041
ENSG0000027	RP11-131L12.	lncRNA	1.54483288	0.50792642	NA
ENSG0000018	POU3F1	protein_codir	1.54478218	0.00095288	0.00551292
ENSG0000011	FLVCR2	protein_codir	1.54443616	0.00012256	0.00103419
ENSG0000019	MUC2	protein_codir	1.54358668	0.41950896	NA
ENSG0000016	CD300C	protein_codir	1.54358548	0.00150756	0.00791464
ENSG0000021	SP9	protein_codir	1.54350244	0.4189847	NA
ENSG0000017	KISS1	protein_codir	1.54329352	0.41768871	NA
ENSG000000C	MAP3K14	protein_codir	1.54323489	2.97E-07	6.41E-06
ENSG0000013	NCAN	protein_codir	1.54202173	0.32007166	0.47221725
ENSG0000017	C5orf64	lncRNA	1.54198445	0.0318977	0.08522016
ENSG0000016	IGF2	protein_codir	1.54175483	0.00418682	0.01767457
ENSG0000014	CTRL	protein_codir	1.5413579	0.00148841	0.00783942
ENSG0000022	CCDC18-AS1	lncRNA	1.5412117	0.0011576	0.00642155
ENSG000001C	RASL10A	protein_codir	1.54081143	0.00493351	0.02005646

ENSG0000027	RP11-5G9.5	lncRNA	1.54035465	0.2297441	0.3721938
ENSG0000023	LINC02331	lncRNA	1.54016637	0.23507004	0.37853513
ENSG0000018	TSPAN10	protein_codir	1.54002829	0.01802041	0.05474304
ENSG0000022	RP1-111D6.3	lncRNA	1.53976751	0.13961248	0.2584342
ENSG0000027	LARGE1-AS1	lncRNA	1.53924984	0.5072657	NA
ENSG0000018	S1PR5	protein_codir	1.53870868	0.00038235	0.00263484
ENSG0000002	HSD17B6	protein_codir	1.53862389	0.02106582	0.06173346
ENSG0000025	RP11-91H12.4	lncRNA	1.53853114	0.63696196	NA
ENSG0000017	EVX2	protein_codir	1.53790062	0.42328829	NA
ENSG0000023	CCDC188	protein_codir	1.53759744	0.00381333	0.01637249
ENSG0000016	ERAP2	protein_codir	1.53748911	0.03196554	0.08530307
ENSG0000023	AC003958.2	lncRNA	1.53729929	0.50499954	NA
ENSG0000027	RP11-732M18	lncRNA	1.53626971	0.59265381	NA
ENSG0000025	CTD-2340D6.1	lncRNA	1.53615184	0.59053387	NA
ENSG0000028	AC009412.8	protein_codir	1.53561907	0.63760232	NA
ENSG0000002	BTN3A1	protein_codir	1.53542182	1.48E-05	0.00017881
ENSG0000013	TTC29	protein_codir	1.53535917	0.37419993	NA
ENSG0000016	DIPK1B	protein_codir	1.53530863	7.33E-05	0.0006741
ENSG0000025	RP11-667M19	lncRNA	1.53514851	0.27031712	0.41815508
ENSG0000026	CTB-31O20.3	lncRNA	1.53459965	0.21233614	0.35193977
ENSG0000024	RRS1-AS1	lncRNA	1.53431165	0.07955049	0.16996029
ENSG0000025	LINC02414	lncRNA	1.53384512	0.42728157	NA
ENSG0000016	NDST3	protein_codir	1.53374003	0.09184858	0.18914836
ENSG0000020	C17orf107	protein_codir	1.53341234	0.00030728	0.00219472
ENSG0000013	SAMD10	protein_codir	1.53336943	2.85E-05	0.0003078
ENSG0000011	BMP5	protein_codir	1.53306998	0.00219189	0.01055546
ENSG0000024	CTD-3080P12	lncRNA	1.53274389	0.17742544	0.30801324
ENSG0000013	H19	lncRNA	1.53272446	0.00031432	0.0022388
ENSG0000017	LRRC8D	protein_codir	1.53253293	1.61E-05	0.00019105
ENSG0000027	RP5-1098D14	lncRNA	1.53142913	0.19061533	0.32524838
ENSG0000027	POU2F1-DT	lncRNA	1.53142113	0.09985176	0.20157162
ENSG0000028	RP11-262H14	lncRNA	1.53116329	0.00013941	0.00115315
ENSG0000013	EDNRB	protein_codir	1.53088196	0.00028499	0.00206761
ENSG0000025	RP11-665C16.	lncRNA	1.53074907	0.58817903	NA
ENSG0000027	RP11-372B4.3	lncRNA	1.53065712	0.02973596	0.08096285
ENSG0000017	CABP4	protein_codir	1.53040355	0.00168626	0.00863987
ENSG0000023	LINC00421	lncRNA	1.53036036	0.50260982	NA
ENSG0000022	RP11-550P17.	lncRNA	1.53026835	0.50141819	NA
ENSG0000012	TNN	protein_codir	1.5298519	0.02178063	0.06331099
ENSG0000014	SHROOM2	protein_codir	1.52983042	2.12E-09	9.35E-08
ENSG0000027	RP11-342K6.4	lncRNA	1.52942295	0.39899061	0.55219325
ENSG0000028	RP11-807H22	protein_codir	1.52936079	0.37682632	NA
ENSG0000027	CTB-55O6.13	lncRNA	1.52908245	0.27083072	0.41878538
ENSG0000025	LINC01152	lncRNA	1.52868339	0.19090725	0.32550517
ENSG0000017	TLR1	protein_codir	1.52804364	0.00042547	0.00286481
ENSG0000000	TMEM176A	protein_codir	1.5272038	6.93E-06	9.40E-05
ENSG0000013	MRO	protein_codir	1.52606394	0.01049774	0.03617472

ENSG0000026	CTD-3193O13	lncRNA	1.52580451	0.51136111	NA
ENSG0000026	RP11-521L9.2	lncRNA	1.52576696	0.21173098	0.35123182
ENSG0000018	GJB3	protein_codir	1.52560239	0.03127427	0.08403832
ENSG0000023	AC007278.2	lncRNA	1.5253307	0.11555669	0.22460772
ENSG0000016	FAM171A2	protein_codir	1.52523105	0.00094259	0.00546596
ENSG0000020	FAM155A	protein_codir	1.52520501	2.07E-05	0.00023567
ENSG0000020	PSMB10	protein_codir	1.52417777	1.39E-09	6.64E-08
ENSG0000026	LINC02137	lncRNA	1.52403748	0.2628312	0.41015076
ENSG0000001	FYN	protein_codir	1.5236997	1.72E-14	5.47E-12
ENSG0000017	MTSS1	protein_codir	1.52306789	4.07E-06	5.97E-05
ENSG0000014	CARD14	protein_codir	1.52291981	0.00854754	0.03073198
ENSG0000019	FAM180B	protein_codir	1.52229427	0.02570235	0.07218087
ENSG0000017	CEACAM3	protein_codir	1.52221495	0.01526275	0.04833818
ENSG0000028	RP3-365E2.1	lncRNA	1.52175798	0.32886809	0.4811854
ENSG0000015	RNF166	protein_codir	1.52163441	1.25E-07	3.06E-06
ENSG0000022	AC016999.2	lncRNA	1.52113398	0.14195069	0.2615664
ENSG0000016	DISC1	protein_codir	1.52100517	1.72E-06	2.92E-05
ENSG0000007	PAFAH1B3	protein_codir	1.52070401	4.18E-06	6.10E-05
ENSG0000027	CTA-228A9.4	lncRNA	1.52020481	0.14419565	0.26454227
ENSG0000019	NOXO1	protein_codir	1.52013243	0.06047875	0.1387971
ENSG0000025	LINC02705	lncRNA	1.51989141	0.11292184	0.22076028
ENSG0000025	RP11-696F12.	lncRNA	1.51982905	0.09446725	0.19334461
ENSG0000017	C1orf127	protein_codir	1.51954454	0.00176115	0.00891386
ENSG0000012	MYCT1	protein_codir	1.51878177	8.44E-05	0.00075757
ENSG0000026	TXNIP	protein_codir	1.51852727	1.53E-05	0.00018255
ENSG0000027	SRCIN1	protein_codir	1.51840426	0.00835519	0.0302114
ENSG0000002	HMGB3	protein_codir	1.51829103	1.65E-06	2.80E-05
ENSG0000027	RP11-133K1.1	lncRNA	1.51757745	0.1641454	0.2911441
ENSG0000020	C16orf96	protein_codir	1.51708079	0.00380515	0.01634754
ENSG0000027	CTD-2287O16	lncRNA	1.51706609	0.64168829	NA
ENSG0000025	RP4-541C22.5	lncRNA	1.51706609	0.64168829	NA
ENSG0000027	JARID2-DT	lncRNA	1.51700156	0.09976481	0.20144046
ENSG0000018	PRR5	protein_codir	1.51674113	5.49E-06	7.70E-05
ENSG0000022	AP001476.2	lncRNA	1.51669693	0.25201194	0.39813611
ENSG0000028	LLNLR-263F3.	lncRNA	1.51650036	0.00791853	0.02897588
ENSG0000018	PARPBP	protein_codir	1.51648209	1.39E-05	0.00016961
ENSG0000014	NFKBIZ	protein_codir	1.51630856	7.22E-07	1.38E-05
ENSG0000026	FOXF2-DT	lncRNA	1.51559034	0.59404629	NA
ENSG0000025	RASGRF2-AS1	lncRNA	1.51558228	0.03261665	0.08672236
ENSG0000028	AC005613.1	lncRNA	1.51524816	0.58417258	NA
ENSG0000016	CPA3	protein_codir	1.51511705	0.00711287	0.0266383
ENSG0000028	CTD-2349B8.2	lncRNA	1.51420976	0.6423183	NA
ENSG0000026	RP11-138P22.	lncRNA	1.51420976	0.6423183	NA
ENSG0000022	AP000233.4	lncRNA	1.51420976	0.6423183	NA
ENSG0000014	LHX9	protein_codir	1.51390914	0.06094903	0.13964376
ENSG0000022	RP11-439A17	lncRNA	1.5136375	0.59297574	NA
ENSG0000018	MAGEB17	protein_codir	1.51361954	0.16217341	0.28849863

ENSG0000023	PTCHD1-AS	lncRNA	1.51349977	0.58964076	NA
ENSG0000028	RP11-426A21	lncRNA	1.51347999	0.58916484	NA
ENSG0000011	WNT5B	protein_codir	1.51335004	2.04E-05	0.00023269
ENSG0000010	CCL8	protein_codir	1.51291823	6.34E-05	0.00059748
ENSG0000014	CYP1A1	protein_codir	1.51272751	0.19163847	0.32628015
ENSG0000021	CEACAM16	protein_codir	1.51242756	0.59393096	NA
ENSG0000013	HMGCS2	protein_codir	1.51242633	0.59390029	NA
ENSG0000023	ABCA9-AS1	lncRNA	1.51241491	0.59361596	NA
ENSG0000027	RP11-618L22.	lncRNA	1.5121735	0.50929068	NA
ENSG0000018	OR2T29	protein_codir	1.51215417	0.58720382	NA
ENSG0000028	RP11-69K20.1	lncRNA	1.51207639	0.16148057	0.28761806
ENSG0000023	LINC02539	lncRNA	1.51205496	0.58480197	NA
ENSG0000025	CTD-2017C7.2	lncRNA	1.5118139	0.04067969	0.10277835
ENSG0000013	SINHCAF	protein_codir	1.51177096	1.99E-10	1.30E-08
ENSG0000024	STRC	protein_codir	1.51172953	0.01963077	0.05844872
ENSG0000027	DOC2B	protein_codir	1.51170771	2.98E-07	6.42E-06
ENSG0000025	RP11-426C22.	lncRNA	1.5113863	0.00226029	0.01082477
ENSG0000025	RP11-96D1.3	lncRNA	1.51126566	0.31476912	0.4665834
ENSG0000028	ARHGAP11B	protein_codir	1.51122038	0.00654797	0.02499901
ENSG0000013	ETS1	protein_codir	1.51067541	1.11E-06	2.01E-05
ENSG0000028	RP11-323N12	lncRNA	1.51034018	0.00776937	0.0285739
ENSG0000024	RP11-457P14.	lncRNA	1.51030276	0.24732698	0.39261861
ENSG0000012	KCNJ2	protein_codir	1.51005594	9.28E-05	0.00081652
ENSG0000022	RP11-203J24.	lncRNA	1.50943636	0.50562149	NA
ENSG0000014	ANO7	protein_codir	1.50942491	4.45E-05	0.00044236
ENSG0000012	RFPL1	protein_codir	1.5090062	0.64346667	NA
ENSG0000018	PABPC1L2B	protein_codir	1.5090062	0.64346667	NA
ENSG0000010	SORCS1	protein_codir	1.50899337	0.00660609	0.02513917
ENSG0000026	RP11-146F11.	lncRNA	1.50878719	0.51526848	NA
ENSG0000013	SERPINF1	protein_codir	1.50865142	1.94E-05	0.00022345
ENSG0000019	OR2T3	protein_codir	1.50848439	0.50960584	NA
ENSG0000016	DCSTAMP	protein_codir	1.50846382	0.065463	0.14779781
ENSG0000026	CARHSP1-DT	lncRNA	1.50813242	0.50316134	NA
ENSG0000021	LAT	protein_codir	1.50776087	9.82E-05	0.00085565
ENSG0000010	MEIS3	protein_codir	1.50767464	0.00015188	0.00123832
ENSG0000015	ANKRD29	protein_codir	1.50720672	2.97E-05	0.00031894
ENSG0000017	GIPC3	protein_codir	1.50719551	0.00079004	0.00473955
ENSG0000027	RP11-439M11	lncRNA	1.50699705	0.33705919	0.48994253
ENSG0000016	RECQL4	protein_codir	1.50597042	0.00052214	0.00338691
ENSG0000025	RP11-164P12.	lncRNA	1.50592502	0.25682914	NA
ENSG0000014	MMP3	protein_codir	1.50544844	0.11042288	0.21728981
ENSG0000026	CTD-2349P21	lncRNA	1.5052527	0.52415283	NA
ENSG0000011	EPCAM	protein_codir	1.50523345	0.04138154	0.10421794
ENSG0000028	RP11-122F24.	lncRNA	1.50508076	0.16427945	0.29134444
ENSG0000025	LINC02691	lncRNA	1.50506871	0.20294426	0.34009734
ENSG0000018	TMEM150B	protein_codir	1.50471827	0.00406856	0.01727496
ENSG0000026	RP11-521O16	lncRNA	1.50431668	0.42626055	NA

ENSG0000004	RAB27B	protein_codir	1.50411354	9.53E-06	0.00012353
ENSG0000022	MEIS1-AS3	lncRNA	1.50400144	0.5953299	NA
ENSG0000027	RP11-45A17.4	lncRNA	1.50368973	0.03434575	0.09034569
ENSG0000028	RP11-666A8.1	lncRNA	1.50323244	0.42601038	NA
ENSG0000027	LINC01954	lncRNA	1.50319412	0.4254839	NA
ENSG0000026	RP11-616M22	lncRNA	1.50313676	0.42469777	NA
ENSG0000016	TFF3	protein_codir	1.50305459	0.04334539	0.10797254
ENSG0000025	LINC01146	lncRNA	1.50271844	0.0227467	0.06553685
ENSG0000005	ENTPD2	protein_codir	1.50220385	0.01546549	0.0488012
ENSG0000014	RHCG	protein_codir	1.50218619	0.20876985	0.34761414
ENSG0000024	CTD-2260A17	lncRNA	1.50195563	0.03385835	0.08930172
ENSG0000023	FRGCA	lncRNA	1.50179958	0.60423972	NA
ENSG0000012	H1-3	protein_codir	1.5016797	0.00302158	0.01358556
ENSG0000001	SLC38A5	protein_codir	1.50167388	0.0003499	0.0024491
ENSG0000013	NPL	protein_codir	1.50084605	0.00097821	0.0056305
ENSG0000017	RESF1	protein_codir	1.50079687	1.54E-06	2.67E-05
ENSG0000018	OTOP2	protein_codir	1.50066189	0.64530992	NA
ENSG0000016	WNT4	protein_codir	1.5004989	0.04504141	0.11129395
ENSG0000028	KB-1440D3.20	lncRNA	1.5003729	0.64537379	NA
ENSG0000017	EXOC3L1	protein_codir	1.50036976	0.0001833	0.0014395
ENSG0000005	LY75	protein_codir	1.49999433	0.00072148	0.0044087
ENSG0000010	FAM20A	protein_codir	1.49985768	0.000181	0.00142673
ENSG0000008	NOS1	protein_codir	1.49894494	0.05119649	0.1225316
ENSG0000006	CASP8	protein_codir	1.49863204	0.00011812	0.00100017
ENSG0000028	RP11-153I24.5	lncRNA	1.4983872	0.37836657	0.53196665
ENSG0000014	NLRP12	protein_codir	1.49803353	0.01204212	0.04018891
ENSG0000016	UGT1A6	protein_codir	1.49801419	0.5204633	NA
ENSG0000009	LAMB4	protein_codir	1.49794445	0.02834582	0.0779637
ENSG0000024	RP11-768F21.	lncRNA	1.49785021	0.03328076	0.08811488
ENSG0000028	RP4-738P15.7	lncRNA	1.4970006	0.22509327	0.36685409
ENSG0000017	XKR3	protein_codir	1.49684533	0.26865397	0.41635185
ENSG0000017	OR2B11	protein_codir	1.49502578	0.60637798	NA
ENSG0000026	CTD-3148I10.9	protein_codir	1.49347264	0.5128668	NA
ENSG0000028	AOX3P-AOX2I	lncRNA	1.49336033	0.64692457	NA
ENSG0000014	SLC22A9	protein_codir	1.49273377	0.51426816	NA
ENSG0000026	RP11-701H16	lncRNA	1.49256977	0.60003644	NA
ENSG0000023	LINC01309	lncRNA	1.4922843	0.60446934	NA
ENSG0000012	PLEKHG1	protein_codir	1.49224485	2.51E-11	2.28E-09
ENSG0000026	CTD-2616J11.	lncRNA	1.49218754	0.60093872	NA
ENSG0000024	UBR5-AS1	lncRNA	1.49185673	2.50E-05	0.00027678
ENSG0000023	RP11-335O4.3	lncRNA	1.49183975	0.36472014	NA
ENSG0000018	FREM3	protein_codir	1.49170888	0.1020655	0.20490255
ENSG0000016	ANKLE1	protein_codir	1.49167681	0.00201971	0.00989789
ENSG0000019	ZSCAN5B	protein_codir	1.49129321	0.26223597	0.40974393
ENSG0000027	LINC00540	lncRNA	1.49094513	0.00749202	0.0277609
ENSG0000017	CDH5	protein_codir	1.49091737	4.74E-06	6.80E-05
ENSG0000025	RP11-320M16	lncRNA	1.49085119	0.43297735	NA

ENSG0000018	CARMIL3	protein_codir	1.49082221	0.00807798	0.02944618
ENSG000000	PKP2	protein_codir	1.4905295	0.0049411	0.02008138
ENSG0000023	CARD11-AS1	lncRNA	1.48963568	0.5201264	NA
ENSG0000019	MFAP5	protein_codir	1.48958928	0.00154713	0.00806413
ENSG0000021	FAM138C	lncRNA	1.48952254	0.51698795	NA
ENSG0000019	ESRRG	protein_codir	1.48930677	0.01337357	0.0435947
ENSG0000025	LINC02105	lncRNA	1.48924938	0.50953748	NA
ENSG0000010	MEFV	protein_codir	1.48911896	0.00062478	0.00391921
ENSG0000011	CSF3R	protein_codir	1.48794693	0.00083187	0.00494539
ENSG0000017	GOLGA8J	protein_codir	1.48778777	0.27570603	0.42380902
ENSG0000026	RP11-449L23.	lncRNA	1.48774535	0.64816737	NA
ENSG0000018	AC000068.5	lncRNA	1.48746902	0.00695722	0.02622596
ENSG0000010	SLC52A3	protein_codir	1.48742789	0.00024476	0.00182464
ENSG0000015	TMEM132D	protein_codir	1.48736389	0.60565271	NA
ENSG0000023	PGM5P4-AS1	lncRNA	1.4870433	0.01041005	0.03595346
ENSG0000025	CXXC5-AS1	lncRNA	1.48673438	0.19046041	0.32510454
ENSG0000026	CLEC19A	protein_codir	1.48673033	0.1308235	0.24630847
ENSG0000023	TMEM18-DT	lncRNA	1.48668217	0.1218262	0.23375058
ENSG0000019	ADA	protein_codir	1.48654756	2.60E-05	0.00028615
ENSG0000010	P2RX3	protein_codir	1.48604909	0.29222664	0.44223877
ENSG0000025	RP11-443C10.	lncRNA	1.48574822	0.29997388	0.45096487
ENSG0000020	KRTAP5-10	protein_codir	1.48545724	0.33156602	0.48428627
ENSG0000013	IER3	protein_codir	1.48481541	0.00449705	0.01866676
ENSG0000017	HTR1D	protein_codir	1.484714	0.1036797	0.20732938
ENSG0000028	CH507-154B1	lncRNA	1.48441522	0.01663848	0.05157163
ENSG0000004	RIPOR3	protein_codir	1.48387721	0.00581584	0.02277455
ENSG0000028	RP11-88I21.3	lncRNA	1.4838244	0.52407113	NA
ENSG0000020	LINC00862	lncRNA	1.48354718	0.10853534	0.21458455
ENSG0000028	RP11-2G16.1	lncRNA	1.48350127	0.05120079	0.1225316
ENSG0000025	LINC02728	lncRNA	1.48334973	0.00176401	0.00892266
ENSG0000020	GPR52	protein_codir	1.48290653	0.11415887	0.22257998
ENSG0000016	TAP1	protein_codir	1.48269783	2.99E-08	8.88E-07
ENSG0000027	TMEM14B-DT	lncRNA	1.48206344	0.14613729	0.26741169
ENSG0000026	ARLNC1	lncRNA	1.48190574	0.05426586	0.12797428
ENSG0000010	MCM5	protein_codir	1.48174452	6.02E-07	1.18E-05
ENSG0000010	CSK	protein_codir	1.48128912	1.54E-05	0.00018397
ENSG0000016	PITX2	protein_codir	1.48097614	0.07393666	0.16129298
ENSG0000024	P2RY11	protein_codir	1.48073712	2.52E-06	3.99E-05
ENSG0000012	ATP8A1	protein_codir	1.48060399	2.90E-07	6.28E-06
ENSG0000025	AF131215.6	lncRNA	1.48045184	0.52070601	NA
ENSG0000018	PPP3R2	protein_codir	1.48045184	0.52070601	NA
ENSG0000001	DCN	protein_codir	1.4798049	0.00296434	0.01336299
ENSG0000028	RP1-93H18.7	protein_codir	1.4796917	0.20742071	0.34595169
ENSG0000014	FGF7	protein_codir	1.47940823	0.00050385	0.00328912
ENSG0000026	RP11-73M18.	lncRNA	1.47928467	0.28512331	0.43470445
ENSG0000024	CACNA1C-AS1	lncRNA	1.47925743	0.00142478	0.0075765
ENSG000000	KCNQ1	protein_codir	1.47918779	5.63E-06	7.89E-05

ENSG0000028	RP11-187K14.	lncRNA	1.47910877	0.05285649	0.12541073
ENSG0000013	CNOT6L	protein_codir	1.4789146	1.13E-07	2.79E-06
ENSG0000023	AC073115.6	lncRNA	1.47866374	0.03442246	0.09050436
ENSG0000022	RP11-405O10	lncRNA	1.47863499	0.12105353	0.23262357
ENSG0000022	RP11-367B6.2	lncRNA	1.47836472	0.39534656	0.54833142
ENSG0000010	RPS6KA5	protein_codir	1.4782107	1.10E-06	2.01E-05
ENSG0000016	DYNLRB2	protein_codir	1.4781655	0.02815169	0.07751467
ENSG0000012	TMEM74B	protein_codir	1.47807055	0.00712749	0.0266822
ENSG0000023	AC091177.1	lncRNA	1.47787652	0.44854711	NA
ENSG0000019	TPSB2	protein_codir	1.47781952	0.00710818	0.02662434
ENSG0000015	CYRIB	protein_codir	1.4769329	6.41E-06	8.78E-05
ENSG0000016	CLDND2	protein_codir	1.4766299	0.01199165	0.04005295
ENSG0000025	RP11-586D19	lncRNA	1.47610752	0.23471809	0.37810065
ENSG0000023	NEBL-AS1	lncRNA	1.47587835	0.59435688	NA
ENSG0000028	AL449214.1	lncRNA	1.47561328	0.0748314	0.16260976
ENSG0000021	CCDC183	protein_codir	1.47504709	0.01012312	0.03515263
ENSG0000020	C2orf72	protein_codir	1.47476104	0.01986972	0.0590298
ENSG0000022	C2orf16	protein_codir	1.4746282	0.12563694	0.23921356
ENSG0000026	TTC39A-AS1	lncRNA	1.47377114	0.60700233	NA
ENSG0000024	CECR3	lncRNA	1.47369281	0.59638394	NA
ENSG0000014	CRHBP	protein_codir	1.47368995	0.01983975	0.05897257
ENSG0000018	ALDH1A3	protein_codir	1.47356698	0.00395702	0.0168662
ENSG0000017	C11orf80	protein_codir	1.47349838	9.83E-06	0.00012662
ENSG0000000	ITGA2B	protein_codir	1.47344435	0.00870874	0.03122213
ENSG0000018	HMX2	protein_codir	1.47300761	0.5184499	NA
ENSG0000026	AC004381.7	lncRNA	1.47296309	0.51411522	NA
ENSG0000016	CYP7A1	protein_codir	1.47272577	0.1104986	0.21739237
ENSG0000016	BMPER	protein_codir	1.47241858	0.02323472	0.06661232
ENSG0000026	IKBKE	protein_codir	1.47185123	0.00051433	0.00334408
ENSG0000027	RP11-85B7.5	lncRNA	1.47166279	0.39895288	0.55219325
ENSG0000027	RP11-442O1.3	lncRNA	1.47164813	0.06477031	0.14658767
ENSG0000026	FAM226B	lncRNA	1.4715018	0.01223005	0.04069277
ENSG0000018	DNAH14	protein_codir	1.47132465	0.00045146	0.00300539
ENSG0000023	LLPH-DT	lncRNA	1.47085612	0.12804951	0.24240764
ENSG0000023	LINC01388	lncRNA	1.47053835	0.52472319	NA
ENSG0000022	C2CD4D	protein_codir	1.47022724	0.00490487	0.01997532
ENSG0000021	RP11-43F13.3	lncRNA	1.46995285	0.00976059	0.03412932
ENSG0000017	CD28	protein_codir	1.46969404	0.00230676	0.01099634
ENSG0000013	EDEM1	protein_codir	1.46915375	3.88E-08	1.11E-06
ENSG0000028	RP11-115H11	lncRNA	1.46876844	0.60238806	NA
ENSG0000024	RP11-296I10.3	lncRNA	1.46773762	0.38020872	NA
ENSG0000028	RP11-267P7.1	lncRNA	1.46734505	0.31007515	0.4618105
ENSG0000026	RP11-421E14.	lncRNA	1.4664758	0.04907608	0.11876984
ENSG0000023	PRKAG2-AS1	lncRNA	1.46610907	9.62E-05	0.00084084
ENSG0000018	CLDN5	protein_codir	1.46555089	0.00201765	0.00989478
ENSG0000016	SLC43A2	protein_codir	1.46445473	3.51E-05	0.00036426
ENSG0000021	KRT222	protein_codir	1.4635444	0.00014003	0.00115724

ENSG0000018	OR4F6	protein_codir	1.46332111	0.65358447	NA
ENSG0000026	LINC01882	lncRNA	1.46326944	0.0138232	0.0447216
ENSG0000023	DCST1-AS1	lncRNA	1.46308743	0.03675593	0.09505608
ENSG0000009	STX1B	protein_codir	1.46263949	0.00101925	0.00581407
ENSG0000017	SOX7	protein_codir	1.46249037	0.00280452	0.0127913
ENSG0000020	HLA-G	protein_codir	1.4620084	0.00397799	0.01693987
ENSG0000013	RAMP2	protein_codir	1.46200135	9.12E-07	1.70E-05
ENSG0000016	IL1RAPL1	protein_codir	1.46190134	0.05952919	0.13710775
ENSG0000012	SNAI1	protein_codir	1.46177963	0.00339594	0.01491189
ENSG0000014	IGSF11	protein_codir	1.46172903	0.00739422	0.02749448
ENSG0000012	LRRC61	protein_codir	1.46112621	0.00046286	0.00307017
ENSG0000012	MYO5C	protein_codir	1.46100708	7.29E-05	0.00067154
ENSG0000018	TNFAIP8L1	protein_codir	1.46097673	2.73E-05	0.00029635
ENSG0000014	UGCG	protein_codir	1.46094505	9.22E-08	2.35E-06
ENSG0000016	SOX17	protein_codir	1.46087133	0.01637942	0.05095084
ENSG0000023	RP11-557H15	lncRNA	1.46081618	0.00488554	0.0199142
ENSG0000028	RP11-438N16	lncRNA	1.46071197	0.42613718	0.57793615
ENSG0000017	APOLD1	protein_codir	1.46060988	0.00384659	0.01648965
ENSG0000028	RP11-90P13.2	lncRNA	1.4603635	0.26291883	0.41022585
ENSG0000016	MAD2L1	protein_codir	1.45994745	1.47E-05	0.00017795
ENSG0000012	PTPRB	protein_codir	1.4595367	1.69E-06	2.86E-05
ENSG0000026	LINC01478	lncRNA	1.45922656	0.35202304	0.50559615
ENSG0000012	FAM110A	protein_codir	1.45894391	5.71E-06	7.98E-05
ENSG0000028	RP11-472N13	lncRNA	1.45821546	0.08848225	0.18393014
ENSG0000018	GPR3	protein_codir	1.45771379	0.01192432	0.03988734
ENSG0000021	SOGA3	protein_codir	1.45764488	2.78E-05	0.00030085
ENSG0000028	RP11-22E12.2	lncRNA	1.45711514	0.05637145	0.13162393
ENSG0000014	TAAR1	protein_codir	1.45708419	0.45866984	NA
ENSG0000023	AC064853.2	lncRNA	1.45690898	0.53284605	NA
ENSG0000028	RP11-422P15.	lncRNA	1.45684667	0.6104106	NA
ENSG0000027	RP5-1142A6.1	lncRNA	1.45672931	0.00105817	0.00598904
ENSG0000028	CH17-262O2.!	lncRNA	1.45643466	0.43672864	NA
ENSG0000027	RP11-76P2.4	lncRNA	1.45604718	0.21034987	0.34959164
ENSG0000025	RP11-474P2.4	lncRNA	1.45593299	0.52959533	NA
ENSG0000023	RP11-95M15.	lncRNA	1.45592825	0.53067627	NA
ENSG0000009	TBX15	protein_codir	1.45587001	3.66E-07	7.70E-06
ENSG0000009	CEBPE	protein_codir	1.4557366	0.0084278	0.03038848
ENSG0000014	KIF2C	protein_codir	1.45533365	0.00037702	0.00260635
ENSG0000022	AC002456.2	lncRNA	1.45528277	0.00779449	0.02862818
ENSG0000011	IL18R1	protein_codir	1.45508302	5.79E-05	0.00055679
ENSG0000012	CIT	protein_codir	1.45491644	1.93E-05	0.00022224
ENSG0000013	CYP1B1	protein_codir	1.45478024	0.00079279	0.0047509
ENSG0000020	HLA-A	protein_codir	1.45416109	6.51E-11	4.93E-09
ENSG0000020	OR6K3	protein_codir	1.45394622	0.37479551	0.52831744
ENSG0000025	RP11-77A13.1	lncRNA	1.45389786	0.65567927	NA
ENSG0000024	LINC00968	lncRNA	1.45388593	0.0005492	0.00353177
ENSG0000024	FAM13A-AS1	lncRNA	1.45382414	0.0007289	0.00444828

ENSG0000001	IL20RA	protein_codir	1.45339482	0.02965043	0.08077175
ENSG0000016	RSPO1	protein_codir	1.45284285	0.00767127	0.02827508
ENSG0000023	LINC00841	lncRNA	1.45255834	0.00878129	0.03140885
ENSG0000027	BCAN-AS1	lncRNA	1.45209917	0.02586639	0.07248677
ENSG0000018	VSTM2B	protein_codir	1.45177385	0.28678567	0.43637177
ENSG0000010	REC8	protein_codir	1.45168968	0.00049979	0.00326921
ENSG0000028	RP11-876B24.	lncRNA	1.45168511	0.07610424	0.16472056
ENSG0000023	BACH1-IT3	lncRNA	1.4516162	0.65618688	NA
ENSG0000013	FXYD2	protein_codir	1.45047052	0.00163456	0.00844333
ENSG0000012	OMG	protein_codir	1.45014571	0.02679559	0.07450112
ENSG0000011	HAVCR1	protein_codir	1.44991841	0.04733051	0.11543031
ENSG0000027	WI2-87327B8	lncRNA	1.44978869	0.0115064	0.03887544
ENSG0000025	RSF1-IT1	lncRNA	1.44951685	0.45715919	NA
ENSG0000016	TTYH1	protein_codir	1.44942338	0.03005471	0.08159945
ENSG0000026	SIGLEC5	protein_codir	1.44928782	0.01286698	0.04228267
ENSG0000026	MUC22	protein_codir	1.44893613	0.52539331	NA
ENSG0000008	PKP1	protein_codir	1.44836567	0.02054466	0.06051441
ENSG0000028	RP11-556O5.7	lncRNA	1.44793448	0.0180887	0.0548841
ENSG0000027	RP11-294N21	lncRNA	1.44783315	0.25743423	0.40422371
ENSG0000027	RP3-406P24.4	lncRNA	1.44775418	0.52745995	NA
ENSG0000023	FOXD3-AS1	lncRNA	1.44708171	0.10648342	0.21149662
ENSG0000024	LINC00900	lncRNA	1.44695228	0.01074461	0.03686994
ENSG0000019	EVL	protein_codir	1.44679725	2.07E-06	3.41E-05
ENSG0000025	CTD-3065B20	lncRNA	1.44669299	0.60286109	NA
ENSG0000026	RP11-109E24.	lncRNA	1.44668612	0.38434514	NA
ENSG0000017	CBX2	protein_codir	1.44653004	0.00238229	0.01126472
ENSG0000014	RGS16	protein_codir	1.4463424	0.00155918	0.0081208
ENSG0000025	AP000350.10	protein_codir	1.4460809	0.657419	NA
ENSG0000018	FOXD3	protein_codir	1.44597875	0.02012532	0.05958423
ENSG0000023	RP5-1139I1.1	lncRNA	1.44561787	0.60983621	NA
ENSG0000025	RP11-324E6.6	lncRNA	1.44551275	0.33407804	NA
ENSG0000027	RP11-693J15.	lncRNA	1.44521948	0.41044377	0.56338619
ENSG0000025	RP11-21C4.1	lncRNA	1.44506896	0.0049234	0.02002419
ENSG0000025	RP11-350F16.	lncRNA	1.4448314	0.53432555	NA
ENSG0000022	CTD-2527I21.	lncRNA	1.4448123	0.31660263	0.46832502
ENSG0000024	LILRA6	protein_codir	1.44444571	0.0012525	0.00684216
ENSG0000009	CECR2	protein_codir	1.44440824	0.00398827	0.01697498
ENSG0000013	FIBCD1	protein_codir	1.44429466	0.06554181	0.14790954
ENSG0000023	RP11-286H14	protein_codir	1.4442606	0.08491225	0.17841585
ENSG0000014	CCDC33	protein_codir	1.44424256	0.38431546	0.53756674
ENSG0000016	HPGD	protein_codir	1.44423965	0.00427337	0.01794321
ENSG0000016	TIPARP	protein_codir	1.44366793	0.00380826	0.01635327
ENSG0000013	SLC39A8	protein_codir	1.44281352	4.03E-05	0.0004076
ENSG0000018	AC005037.3	lncRNA	1.44248311	0.04846732	0.11760233
ENSG0000013	KIF20B	protein_codir	1.44220127	8.53E-09	3.07E-07
ENSG0000018	TOR3A	protein_codir	1.44123228	2.32E-06	3.73E-05
ENSG0000006	DMRT3	protein_codir	1.44096722	0.00389785	0.01666025

ENSG0000004	BARX2	protein_codir	1.44085089	0.13155756	0.24742781
ENSG0000015	BMP3	protein_codir	1.43984378	0.00879505	0.03144586
ENSG0000018	DDX60L	protein_codir	1.43970547	0.00010464	0.00090204
ENSG0000018	IL3RA	protein_codir	1.43968747	2.97E-05	0.00031869
ENSG0000018	NUTM1	protein_codir	1.43891821	0.26240568	0.40986512
ENSG0000026	LINC01532	lncRNA	1.43866199	0.52903498	NA
ENSG0000016	C7orf57	protein_codir	1.43850041	0.20092905	0.33775548
ENSG0000022	RP1-69D17.4	lncRNA	1.43832059	0.02808855	0.0773871
ENSG0000010	TFR2	protein_codir	1.43827366	0.00170123	0.0086886
ENSG0000028	RP11-231C18.	protein_codir	1.43802685	0.36325336	0.51719458
ENSG0000025	RP11-290F24.	lncRNA	1.43802289	0.26678299	0.41435901
ENSG0000027	RP11-596C23.	lncRNA	1.43794601	0.01518127	0.04813532
ENSG0000018	LRRC14B	protein_codir	1.43781119	0.1579048	0.28290935
ENSG0000012	ITPR2	protein_codir	1.43772529	1.31E-06	2.30E-05
ENSG0000011	OAS3	protein_codir	1.43765427	0.00103873	0.00590078
ENSG0000028	RP5-1007F24.	lncRNA	1.43753233	0.30174021	0.45296291
ENSG0000026	RP11-886P16.	lncRNA	1.43724827	0.61030748	NA
ENSG0000028	ZNF511-PRAP	protein_codir	1.43722819	0.61166472	NA
ENSG0000026	RP11-143J12.	lncRNA	1.43715304	0.61680707	NA
ENSG0000019	FAM72A	protein_codir	1.43708928	3.54E-05	0.00036703
ENSG0000013	IL10	protein_codir	1.43671435	0.00066363	0.00412325
ENSG0000027	LA16c-360A4.	lncRNA	1.43666726	0.05202744	0.12393398
ENSG0000024	REPIN1-AS1	lncRNA	1.43649741	0.21777561	0.3581614
ENSG0000005	OPN3	protein_codir	1.43640208	6.48E-06	8.86E-05
ENSG0000026	RP11-227G15	lncRNA	1.43637751	0.28511151	0.43470445
ENSG0000022	RP11-348F1.3	lncRNA	1.43617605	0.05343859	0.12651927
ENSG0000027	RP11-523H20	lncRNA	1.43573276	0.27969002	0.42868127
ENSG0000013	EPHB2	protein_codir	1.43553867	1.54E-05	0.00018363
ENSG0000023	RP5-963E22.4	lncRNA	1.43541423	0.36181308	0.51572882
ENSG0000014	NCEH1	protein_codir	1.43377327	1.42E-05	0.00017279
ENSG0000027	CTD-2024P10	lncRNA	1.43338665	0.46349469	NA
ENSG0000012	TRERF1	protein_codir	1.43320953	2.21E-07	4.92E-06
ENSG0000016	HID1	protein_codir	1.43259049	7.43E-05	0.00068144
ENSG0000013	POPDC3	protein_codir	1.4320857	0.06234702	0.14222206
ENSG0000013	CLSTN3	protein_codir	1.43188269	6.21E-06	8.57E-05
ENSG0000013	CILP	protein_codir	1.4310623	0.06824767	0.15221724
ENSG0000013	GIMAP6	protein_codir	1.43079527	6.36E-05	0.00059949
ENSG0000014	SH3KBP1	protein_codir	1.43078478	4.11E-08	1.17E-06
ENSG0000017	CYP7B1	protein_codir	1.43075966	2.85E-05	0.00030737
ENSG0000015	FCGR1A	protein_codir	1.43067008	0.00188085	0.00935684
ENSG0000016	ZNF66	protein_codir	1.43053365	6.20E-05	0.00058769
ENSG0000013	EPS8L1	protein_codir	1.43038683	0.00620898	0.02396813
ENSG0000027	RP11-439C15.	lncRNA	1.42956491	0.5308201	NA
ENSG0000027	RP11-474P2.6	lncRNA	1.42946118	0.01529944	0.04842562
ENSG0000013	SLC34A1	protein_codir	1.42943939	0.53492231	NA
ENSG0000022	MYCNUT	lncRNA	1.42938368	0.53676274	NA
ENSG0000010	SEC14L5	protein_codir	1.42928456	0.01984186	0.05897257

ENSG0000021	BASP1-AS1	lncRNA	1.42904647	0.04019005	0.1019128
ENSG0000027	GPIHBP1	protein_codir	1.42902335	0.006962	0.02623336
ENSG0000016	FAM107A	protein_codir	1.429	0.00408123	0.01732075
ENSG0000017	HAP1	protein_codir	1.42856042	0.04620537	0.11332543
ENSG0000007	CDC14A	protein_codir	1.4284323	9.98E-10	5.07E-08
ENSG0000027	CTD-3224K15	lncRNA	1.42829315	0.23790484	0.3820087
ENSG0000027	CCL15-CCL14	protein_codir	1.42826848	0.00491592	0.02000849
ENSG0000010	MYH1	protein_codir	1.42809415	0.15890566	0.28425999
ENSG0000026	EDDM13	protein_codir	1.42789798	0.2657651	0.41324281
ENSG0000016	LINGO1	protein_codir	1.42739892	0.00012657	0.00106286
ENSG0000021	FBXO16	protein_codir	1.42718436	0.00120047	0.00661155
ENSG0000016	RELL2	protein_codir	1.42713056	0.0005213	0.00338229
ENSG0000010	STK4	protein_codir	1.42702696	9.61E-08	2.43E-06
ENSG0000012	TEX14	protein_codir	1.42693856	0.01473942	0.04706935
ENSG0000025	LINC02395	lncRNA	1.42688893	0.46498684	NA
ENSG0000026	LINC01227	lncRNA	1.4266258	0.42772935	0.57953812
ENSG0000012	APOBEC3F	protein_codir	1.42645134	0.00026314	0.00193606
ENSG0000023	AC007879.2	lncRNA	1.42619695	0.04232594	0.10609334
ENSG0000028	RP11-181B11.	lncRNA	1.42589733	0.3037056	0.45499756
ENSG0000010	BIRC7	protein_codir	1.42584109	0.04699766	0.11474193
ENSG0000011	BTNL8	protein_codir	1.425162	0.08897721	0.1847012
ENSG0000011	ARHGDIB	protein_codir	1.42511312	1.69E-06	2.86E-05
ENSG0000025	RP11-429D19	lncRNA	1.4237123	0.2651524	0.41275486
ENSG0000010	NECAB2	protein_codir	1.4235535	0.00151229	0.00793491
ENSG0000025	GS1-393G12.1	lncRNA	1.42341231	0.24394312	0.38908062
ENSG0000028	CTD-2004A9.2	lncRNA	1.4233564	0.41172264	0.56475133
ENSG0000025	LINC02823	lncRNA	1.42289902	0.0877204	0.18269019
ENSG0000006	ANKRD44	protein_codir	1.42256639	5.32E-07	1.06E-05
ENSG0000015	KRTCAP3	protein_codir	1.42251604	0.00871838	0.03124859
ENSG0000000	BAIAP2L1	protein_codir	1.42234697	9.75E-05	0.0008501
ENSG0000025	RP11-1105G2	lncRNA	1.42149575	0.00097725	0.00562796
ENSG0000016	ARHGEF3	protein_codir	1.42123806	3.11E-09	1.31E-07
ENSG0000025	RP11-688G15	lncRNA	1.42017788	0.21610253	0.35636468
ENSG0000019	CFAP43	protein_codir	1.41988252	0.01266918	0.04177185
ENSG0000015	CMIP	protein_codir	1.41973292	5.97E-09	2.28E-07
ENSG0000022	RP1-122O8.7	lncRNA	1.41972554	0.31001903	0.46180161
ENSG0000022	RP11-415J8.3	lncRNA	1.41962269	0.39826718	NA
ENSG0000016	HS3ST6	protein_codir	1.41950167	0.39017541	NA
ENSG0000016	LRRC71	protein_codir	1.41944243	0.08871702	0.1843064
ENSG0000016	GJB7	protein_codir	1.41925852	0.6289801	NA
ENSG0000027	SPDYE10P	lncRNA	1.41923333	0.3833774	0.53677111
ENSG0000026	RP1-80N2.3	lncRNA	1.41883856	0.07869557	0.16861155
ENSG0000014	DUSP10	protein_codir	1.41877455	5.34E-06	7.52E-05
ENSG0000022	SUCLA2-AS1	lncRNA	1.41867047	0.45973611	NA
ENSG0000017	OBP2B	protein_codir	1.41845919	0.44877454	NA
ENSG0000007	ATP2B1	protein_codir	1.41840754	1.21E-08	4.13E-07
ENSG0000028	RP5-1139B12.	lncRNA	1.41840468	0.66359312	NA

ENSG0000016	FAM83B	protein_codir	1.41840468	0.66359312	NA
ENSG0000028	RP11-445K13.	lncRNA	1.41840468	0.66359312	NA
ENSG0000019	NOL4L	protein_codir	1.4183709	5.54E-10	3.09E-08
ENSG0000018	KCNIP1	protein_codir	1.41817243	0.00946249	0.03332321
ENSG0000016	ZMAT4	protein_codir	1.41761402	0.06417498	0.14555042
ENSG0000014	TNFAIP8	protein_codir	1.4173609	1.22E-05	0.00015125
ENSG0000026	AC006129.1	lncRNA	1.41729812	0.07288421	0.15966544
ENSG0000015	EPB41	protein_codir	1.4170212	1.18E-06	2.11E-05
ENSG0000026	RP13-20L14.4	lncRNA	1.41688417	0.13359457	0.25022935
ENSG0000010	MAN2B1	protein_codir	1.416786	7.28E-09	2.68E-07
ENSG0000016	ELOVL7	protein_codir	1.41669342	0.00082302	0.0049012
ENSG0000027	ATP2B1-AS1	lncRNA	1.41665652	1.34E-08	4.51E-07
ENSG0000022	RP11-123K19.	lncRNA	1.41623137	0.46242836	NA
ENSG0000028	RP11-449F7.4	lncRNA	1.41598686	0.41701748	0.5696915
ENSG0000022	LINC01693	lncRNA	1.41451317	0.45928002	NA
ENSG0000028	CTD-2021K4.2	lncRNA	1.41435741	0.45579716	NA
ENSG0000000	CALCR	protein_codir	1.41429287	0.00659437	0.02511178
ENSG0000014	ATP6V0D2	protein_codir	1.41426887	0.13272691	0.24907713
ENSG0000018	CYP27C1	protein_codir	1.41418894	0.00029517	0.00212586
ENSG0000022	APOL6	protein_codir	1.41418662	6.38E-06	8.75E-05
ENSG0000026	RP11-25D3.1	lncRNA	1.41414571	0.4000777	NA
ENSG0000016	BCL6B	protein_codir	1.41393959	1.57E-06	2.71E-05
ENSG0000025	AP002954.4	lncRNA	1.41367856	0.05034258	0.12119429
ENSG0000017	MCTP1	protein_codir	1.41362366	4.52E-06	6.51E-05
ENSG0000025	PDE2A-AS2	lncRNA	1.4134755	0.00685971	0.0259151
ENSG0000015	PTPRN2	protein_codir	1.41343973	3.94E-06	5.83E-05
ENSG0000016	SCGB3A1	protein_codir	1.4131789	0.0970315	0.19721908
ENSG0000010	LYL1	protein_codir	1.41303284	0.00048418	0.00318707
ENSG0000026	RP11-666A8.9	lncRNA	1.41259262	0.30707388	0.4588002
ENSG0000025	XXbac-BPG24	protein_codir	1.41218299	0.00079166	0.00474514
ENSG0000026	CCDC194	protein_codir	1.41193247	0.09454425	0.19344487
ENSG0000018	GBP6	protein_codir	1.41169067	0.01607193	0.05030135
ENSG0000017	C15orf54	lncRNA	1.41147817	0.15501084	0.27908216
ENSG0000011	RSPH4A	protein_codir	1.41102398	8.69E-05	0.00077559
ENSG0000014	PTPRJ	protein_codir	1.41075988	8.68E-05	0.00077456
ENSG0000014	ADAD2	protein_codir	1.41072438	0.04612034	0.11319032
ENSG0000018	LINC01551	lncRNA	1.41072242	0.62143508	NA
ENSG0000019	GATA3-AS1	lncRNA	1.41042643	0.25149606	0.39762586
ENSG0000024	RP11-543C4.1	lncRNA	1.4103447	0.01021672	0.03541934
ENSG0000013	KCNC3	protein_codir	1.41032555	0.00226165	0.01082802
ENSG0000028	RP13-685P2.9	lncRNA	1.41004572	0.30752825	0.4592513
ENSG0000010	ZFR2	protein_codir	1.4095683	0.06504272	0.14708367
ENSG0000017	C1QA	protein_codir	1.40956531	8.22E-05	0.00074082
ENSG0000011	VWF	protein_codir	1.40857674	1.08E-05	0.00013625
ENSG0000017	EMB	protein_codir	1.40767076	0.00026248	0.00193172
ENSG0000016	RNASE3	protein_codir	1.40765755	0.04278212	0.10696207
ENSG0000010	RNF32	protein_codir	1.40764605	0.00300681	0.01352557

ENSG0000020	HSD17B8	protein_codir	1.40725441	1.63E-06	2.79E-05
ENSG0000028	RP11-617F9.2	lncRNA	1.40649248	0.07093863	0.15657064
ENSG0000023	RP11-323C15.	lncRNA	1.40616135	0.37339782	0.52699274
ENSG0000013	SLCO2B1	protein_codir	1.40612566	0.00097654	0.00562621
ENSG0000027	RP11-66B24.9	lncRNA	1.40593793	0.29020558	0.43987916
ENSG0000020	DENND6B	protein_codir	1.40542438	3.66E-05	0.00037776
ENSG0000016	TAL1	protein_codir	1.40538483	0.00250162	0.01170304
ENSG0000015	TKTL2	protein_codir	1.4053474	0.06971039	0.15455138
ENSG0000018	TET3	protein_codir	1.40520477	1.18E-05	0.00014719
ENSG0000026	TUBB8	protein_codir	1.405073	0.30797794	0.45972819
ENSG0000027	KB-1836B5.4	lncRNA	1.40478952	0.40191426	NA
ENSG0000024	LINC02458	lncRNA	1.4044836	0.07016395	0.15535741
ENSG0000018	OR2AG2	protein_codir	1.40448066	0.06400955	0.14529452
ENSG0000027	C16orf95-DT	lncRNA	1.40442038	0.04357447	0.10842583
ENSG0000017	SPINK6	protein_codir	1.40394137	0.62379137	NA
ENSG0000023	RP11-723O4.2	lncRNA	1.40388448	0.04221703	0.10586839
ENSG0000015	PLCL2	protein_codir	1.40366761	5.92E-09	2.26E-07
ENSG0000028	RP11-556N21	lncRNA	1.40352265	0.38737104	NA
ENSG0000004	ZIC2	protein_codir	1.40320735	0.36174074	0.51572882
ENSG0000011	UNC93B1	protein_codir	1.40296271	2.21E-06	3.58E-05
ENSG0000000	CCL26	protein_codir	1.40287779	0.01153707	0.03894573
ENSG0000018	SATL1	protein_codir	1.4027895	0.63616437	NA
ENSG0000025	RP11-716H6.2	lncRNA	1.4026679	0.66711374	NA
ENSG0000014	RGS10	protein_codir	1.40219879	2.11E-06	3.46E-05
ENSG0000007	ST6GALNAC2	protein_codir	1.40150376	0.00254711	0.01184242
ENSG0000013	MYBPH	protein_codir	1.4013697	0.07545329	0.16368456
ENSG0000028	RP11-15A1.10	lncRNA	1.40120453	0.12590104	0.23955543
ENSG0000002	ABCC2	protein_codir	1.40102017	4.65E-05	0.00045982
ENSG0000011	VDR	protein_codir	1.40094286	0.00044673	0.00298361
ENSG0000017	ZNF217	protein_codir	1.40046102	2.67E-08	8.08E-07
ENSG0000020	NPY4R	protein_codir	1.4000978	0.19076559	0.32537003
ENSG0000012	IL1B	protein_codir	1.40000566	0.00669989	0.02541212
ENSG0000024	C5orf17	lncRNA	1.39992151	0.09531191	0.19456873
ENSG0000021	ARHGAP19	protein_codir	1.39974991	1.84E-06	3.07E-05
ENSG0000017	SP7	protein_codir	1.39960654	0.11398842	0.22231043
ENSG0000016	LEAP2	protein_codir	1.39930955	0.03046743	0.08243638
ENSG0000028	RP4-564M11.	lncRNA	1.39897032	0.3732241	0.52682832
ENSG0000014	FCGR2A	protein_codir	1.39876856	0.00057671	0.0036702
ENSG0000016	SUN3	protein_codir	1.39845396	0.6680577	NA
ENSG0000028	RP11-542M13	lncRNA	1.39845396	0.6680577	NA
ENSG0000013	PDE6B	protein_codir	1.39826062	0.00042599	0.00286635
ENSG0000025	LINC02376	lncRNA	1.39815901	0.54042714	NA
ENSG0000014	TPM3	protein_codir	1.39755432	1.47E-09	6.93E-08
ENSG0000018	CALHM3	protein_codir	1.39751628	0.48602982	NA
ENSG0000028	RP11-496O3.3	lncRNA	1.39677594	0.31638503	0.46812848
ENSG0000027	PRAG1	protein_codir	1.39545727	0.00048258	0.00317882
ENSG0000014	DUOXA1	protein_codir	1.39542024	0.00463049	0.01913722

ENSG0000025	RP11-897M7.	lncRNA	1.39537553	0.25006534	0.39596426
ENSG0000026	RP11-120M18	lncRNA	1.39524372	0.01902435	0.05707718
ENSG0000013	TAPBPL	protein_codir	1.39446173	2.55E-11	2.31E-09
ENSG0000014	TCTE1	protein_codir	1.39438445	0.01357768	0.04410544
ENSG0000013	FAM83F	protein_codir	1.39404448	0.01873457	0.0563996
ENSG0000027	RP11-103B5.4	lncRNA	1.39394722	0.10646573	0.21147669
ENSG0000026	SULT1A3	protein_codir	1.39389474	0.000116	0.0009849
ENSG0000016	FOLR2	protein_codir	1.39374264	9.07E-05	0.00080179
ENSG0000028	RP11-5O24.1	lncRNA	1.39371703	0.24297457	0.38796148
ENSG0000022	EMX2OS	lncRNA	1.39347805	0.01987381	0.0590298
ENSG0000015	ZNF233	protein_codir	1.39320482	0.02047066	0.06034658
ENSG0000027	RP11-66B24.7	lncRNA	1.39310005	0.04767453	0.11603546
ENSG0000028	AC008079.10	lncRNA	1.39308186	0.09569715	0.19513881
ENSG0000027	RP11-21K12.3	lncRNA	1.3927182	0.49491779	0.64096885
ENSG0000023	LILRA2	protein_codir	1.39271316	0.00332066	0.01465826
ENSG0000024	AC005062.2	lncRNA	1.39267369	0.10134424	0.20379787
ENSG0000015	TCHH	protein_codir	1.39234361	0.00148368	0.00782116
ENSG0000000	DLEC1	protein_codir	1.39214893	0.00041803	0.00282504
ENSG0000025	RSF1-IT2	lncRNA	1.39158799	0.30830854	0.45997309
ENSG0000013	DHH	protein_codir	1.3906839	0.00678688	0.02568561
ENSG0000011	KIF17	protein_codir	1.39036066	1.82E-05	0.00021126
ENSG0000025	RP11-173D3.1	lncRNA	1.38982788	0.34356599	NA
ENSG0000015	TNFRSF14	protein_codir	1.38942581	3.84E-05	0.00039243
ENSG0000028	RP11-172F7.1	lncRNA	1.38896237	0.34476496	NA
ENSG0000016	AGBL2	protein_codir	1.38878713	0.00372747	0.01605628
ENSG0000022	AC046143.3	lncRNA	1.38864036	0.01605302	0.05027035
ENSG0000019	ADAMTSL2	protein_codir	1.38856298	6.57E-05	0.00061589
ENSG0000013	SCN7A	protein_codir	1.38816305	0.03832863	0.09812186
ENSG0000024	CTD-2135J3.3	lncRNA	1.3873879	0.02054964	0.06052261
ENSG0000019	RFX8	protein_codir	1.38679034	0.00769845	0.02834595
ENSG0000028	RP11-118J22.	lncRNA	1.38651245	0.26412779	0.41162245
ENSG0000011	TFAP2E	protein_codir	1.38642923	0.00044527	0.00297549
ENSG0000026	RP11-399O19	lncRNA	1.3860889	0.50948998	0.65375348
ENSG0000020	RP11-830F9.6	lncRNA	1.38600924	0.00029662	0.00213406
ENSG0000015	CCDC102B	protein_codir	1.38590115	0.00010691	0.00091965
ENSG0000013	BRCA2	protein_codir	1.38546751	1.16E-05	0.00014473
ENSG0000011	ELMOD1	protein_codir	1.3847937	0.06619465	0.14901754
ENSG0000016	TMEM200A	protein_codir	1.38439756	0.00074231	0.00451001
ENSG0000023	RP3-393E18.2	lncRNA	1.38432783	0.06662059	0.14976889
ENSG0000014	SYT14	protein_codir	1.38416536	0.46685826	0.61649576
ENSG0000028	CTD-2336O2.1	lncRNA	1.38414549	0.08240616	0.17439723
ENSG0000028	RP11-563M4.	lncRNA	1.38396402	0.35424165	0.50784565
ENSG0000022	AC007879.5	lncRNA	1.38382178	0.05292588	0.12552952
ENSG0000000	HS3ST1	protein_codir	1.38370328	0.00014124	0.00116624
ENSG0000016	ZC3H12A	protein_codir	1.38367183	0.00011862	0.00100373
ENSG0000016	BDH1	protein_codir	1.38319495	0.00054493	0.00351169
ENSG0000023	FAM225A	lncRNA	1.38315699	0.00542745	0.02163147

ENSG0000021	FER1L6	protein_codir	1.38313963	0.13196502	0.24803497
ENSG0000025	RP4-647C14.3	lncRNA	1.38255168	0.06787122	0.15172746
ENSG0000016	TTC39C	protein_codir	1.38254967	2.45E-08	7.49E-07
ENSG0000009	CMA1	protein_codir	1.38227246	0.00646762	0.02479428
ENSG0000018	LCN10	protein_codir	1.38226021	0.00556409	0.02202354
ENSG0000028	LINC02775	lncRNA	1.38198665	0.67175141	NA
ENSG0000015	RP11-523H20	protein_codir	1.38197847	0.02795774	0.07709592
ENSG0000025	MAFA-AS1	lncRNA	1.38181268	0.32323676	0.47548995
ENSG0000026	RP11-7K24.3	lncRNA	1.38176541	0.12990354	0.24497718
ENSG0000027	RP11-407N8.5	lncRNA	1.38152499	0.5526861	NA
ENSG0000017	CRLF3	protein_codir	1.38130133	1.64E-06	2.79E-05
ENSG0000019	ZNF536	protein_codir	1.38076641	0.06440662	0.14594396
ENSG0000010	NFKBIA	protein_codir	1.38050889	4.21E-05	0.00042263
ENSG0000012	LRFN1	protein_codir	1.38007651	0.00146481	0.00774464
ENSG0000022	LINC01058	lncRNA	1.37983184	0.19940034	0.33592177
ENSG0000028	RP11-51I5.3	lncRNA	1.37972311	0.20607635	0.34413163
ENSG0000008	SH3BP2	protein_codir	1.37933899	9.31E-06	0.00012097
ENSG0000006	CALCRL	protein_codir	1.3791164	1.97E-06	3.27E-05
ENSG0000018	CFAP77	protein_codir	1.37902451	0.55749944	NA
ENSG0000023	RP11-305L7.3	lncRNA	1.37891023	0.02534679	0.07137103
ENSG0000019	GPRACR	lncRNA	1.3787188	0.11350589	0.22166673
ENSG0000013	HLX	protein_codir	1.37855468	2.78E-06	4.32E-05
ENSG0000013	XAF1	protein_codir	1.37823654	0.00197871	0.00974711
ENSG0000011	ZC2HC1B	protein_codir	1.37780211	0.48491701	NA
ENSG0000013	SAT1	protein_codir	1.37777154	8.35E-05	0.0007511
ENSG0000022	RP11-84A14.4	lncRNA	1.37738034	0.27416695	NA
ENSG0000026	RP11-227G15	lncRNA	1.37692993	0.25601717	0.4026876
ENSG0000028	RP11-549D23	lncRNA	1.37689753	0.00792249	0.02897622
ENSG0000001	SEMA3G	protein_codir	1.37598977	0.0001452	0.00119392
ENSG0000015	RHPN1	protein_codir	1.37597337	0.00046544	0.0030836
ENSG0000027	PTGER4P2-CD	lncRNA	1.37528314	0.04802417	0.11675749
ENSG0000017	TCIM	protein_codir	1.37469972	0.00675415	0.0255863
ENSG0000010	PLLP	protein_codir	1.37444982	0.00516504	0.02082298
ENSG0000025	IKBKB-DT	lncRNA	1.3739675	0.1721995	0.30165219
ENSG0000014	ARRB2	protein_codir	1.37372787	1.06E-05	0.00013417
ENSG0000016	OBSCN-AS1	lncRNA	1.37358752	0.0018619	0.00928261
ENSG0000026	RP11-873E20.	lncRNA	1.37302245	0.00227543	0.01088644
ENSG0000014	DPEP3	protein_codir	1.37300956	0.08936823	0.18531161
ENSG0000023	LINC01888	lncRNA	1.3727459	0.40182507	0.55490487
ENSG0000009	NCR2	protein_codir	1.37264314	0.64226275	NA
ENSG0000012	PAIP2B	protein_codir	1.37235562	1.52E-05	0.00018221
ENSG0000025	LINC00924	lncRNA	1.37214377	0.0016883	0.00864813
ENSG0000025	RP11-603J24.	lncRNA	1.37196179	0.48126081	NA
ENSG0000014	TAGLN3	protein_codir	1.37189501	0.35713931	0.51072258
ENSG0000011	ADTRP	protein_codir	1.37188356	0.00471119	0.01937509
ENSG0000018	PRR34	lncRNA	1.37142514	7.49E-05	0.0006858
ENSG0000020	CCDC160	protein_codir	1.37127913	0.15319849	0.27664873

ENSG0000027	RP11-5C23.1	lncRNA	1.37079197	0.02612572	0.07307264
ENSG0000018	CLCN1	protein_codir	1.37071114	0.06878969	0.15296443
ENSG0000001	ANLN	protein_codir	1.37063971	0.00037074	0.00256959
ENSG0000011	CNTRL	protein_codir	1.36987582	2.33E-07	5.18E-06
ENSG0000023	MIR155HG	lncRNA	1.36977404	0.00108548	0.0061086
ENSG0000026	RP11-98D18.1	lncRNA	1.369677	0.06730881	0.15086176
ENSG0000019	CACNA1E	protein_codir	1.36916957	0.04006925	0.10165452
ENSG0000006	FAR2	protein_codir	1.36892242	3.29E-05	0.00034529
ENSG0000028	LINC01176	lncRNA	1.36877785	0.00653428	0.02496913
ENSG0000000	ACSM3	protein_codir	1.36851169	2.02E-05	0.00023062
ENSG0000027	CH507-24F1.2	lncRNA	1.3683731	0.12922428	0.24409076
ENSG0000010	COCH	protein_codir	1.36812278	0.00580543	0.02274089
ENSG0000026	AC100830.4	lncRNA	1.36785624	0.03613948	0.09379631
ENSG0000028	RP1-230L10.4	lncRNA	1.36766094	0.55568954	NA
ENSG0000013	DSC2	protein_codir	1.3674322	0.00071999	0.00440323
ENSG0000017	ASPHD1	protein_codir	1.36739327	0.01069983	0.03674367
ENSG0000028	CTD-2015F14.	lncRNA	1.36737193	0.03272324	0.0869639
ENSG0000011	STC2	protein_codir	1.36670803	0.01058804	0.03642344
ENSG0000005	COL23A1	protein_codir	1.36652633	0.00031358	0.00223514
ENSG0000023	C2CD4D-AS1	lncRNA	1.36615527	0.00224535	0.0107648
ENSG0000017	SAMD9L	protein_codir	1.36561	0.00010069	0.00087347
ENSG0000014	SPATA9	protein_codir	1.36506423	0.02188477	0.0635535
ENSG0000023	AC008440.5	lncRNA	1.36504985	0.13496072	0.25201914
ENSG0000010	MLNR	protein_codir	1.36478047	0.38852828	0.54148507
ENSG0000028	RP11-17G11.2	lncRNA	1.36410352	0.01674644	0.05184807
ENSG0000015	MMP21	protein_codir	1.36394404	0.00488304	0.01990694
ENSG0000021	GBP7	protein_codir	1.36350893	0.2216467	0.36268234
ENSG0000017	RAB39A	protein_codir	1.36298575	0.01325912	0.04329709
ENSG0000027	CH507-152C1	lncRNA	1.36288317	0.62559491	NA
ENSG0000022	AP001631.10	lncRNA	1.36288317	0.62559491	NA
ENSG0000008	SEMA5B	protein_codir	1.36245959	1.09E-05	0.00013729
ENSG0000004	TDP1	protein_codir	1.36244378	4.06E-07	8.40E-06
ENSG0000025	CTD-2154I11.	lncRNA	1.36237905	0.00837391	0.03025256
ENSG0000023	PROX1-AS1	lncRNA	1.36197065	0.35882569	0.51247559
ENSG0000014	PTH2R	protein_codir	1.36191079	0.03470324	0.09098277
ENSG0000027	RP11-461L13.	lncRNA	1.36136116	0.41368472	NA
ENSG0000023	AC091133.1	lncRNA	1.36119887	0.4079561	NA
ENSG0000020	ERICH4	protein_codir	1.36104599	0.64530478	NA
ENSG0000014	ATM	protein_codir	1.36096206	6.59E-06	9.00E-05
ENSG0000022	RP11-121A14	lncRNA	1.36064572	0.28414118	0.43373381
ENSG0000007	SCARF1	protein_codir	1.36020503	3.53E-07	7.45E-06
ENSG0000017	C14orf39	protein_codir	1.35985706	0.12550666	0.23902355
ENSG0000021	AC087491.2	lncRNA	1.3593942	0.41306082	NA
ENSG0000028	RP11-128M1.	lncRNA	1.35933672	0.47537389	NA
ENSG0000011	SLC5A9	protein_codir	1.35871529	0.00654241	0.0249928
ENSG0000025	TMC3-AS1	lncRNA	1.35864583	0.00479341	0.01964012
ENSG0000026	RP11-84A19.4	lncRNA	1.35863286	0.05411978	0.12773785

ENSG0000014	GFRA3	protein_codir	1.35836468	0.04735635	0.11547474
ENSG0000007	ST6GALNAC1	protein_codir	1.35834776	0.01117475	0.03799664
ENSG0000026	RP11-645C24.	lncRNA	1.358094	0.0044027	0.01836899
ENSG0000012	UNC5CL	protein_codir	1.35782126	6.66E-05	0.00062273
ENSG0000026	LINC01842	lncRNA	1.35772672	0.36180552	NA
ENSG0000022	AC064875.2	lncRNA	1.35735473	0.01942571	0.05802295
ENSG0000018	JPT1	protein_codir	1.35724036	3.32E-05	0.00034786
ENSG0000026	RP5-1028K7.3	protein_codir	1.35675162	0.03038155	0.08226039
ENSG0000019	MT1F	protein_codir	1.35529213	0.02408912	0.06852128
ENSG0000013	PTPRE	protein_codir	1.35517705	1.10E-05	0.00013875
ENSG0000011	HGD	protein_codir	1.35454575	0.05182548	0.12357935
ENSG0000022	LYPLAL1-AS1	lncRNA	1.35427846	0.12344061	0.2360447
ENSG0000026	CTD-2006K23	lncRNA	1.35338163	0.13702118	0.2546958
ENSG0000018	KCTD16	protein_codir	1.35329334	0.00467799	0.01928161
ENSG0000026	RP11-179B15.	lncRNA	1.35264467	0.23894621	0.38319075
ENSG0000027	RP1-313I6.12	lncRNA	1.35246102	0.00086343	0.00509013
ENSG0000015	SPON2	protein_codir	1.35232526	0.00061418	0.00386765
ENSG0000024	TDGF1	protein_codir	1.35219399	0.25163771	0.39776991
ENSG0000025	EGILA	lncRNA	1.35206099	0.42770827	0.57953812
ENSG0000028	CTD-2201E18.	lncRNA	1.35200971	3.39E-05	0.00035447
ENSG0000018	FAM162B	protein_codir	1.35147069	0.00352263	0.0153559
ENSG0000022	RP11-390P2.4	lncRNA	1.35133549	0.00431796	0.01807824
ENSG0000026	RP5-1050D4.3	lncRNA	1.35128434	0.33838817	NA
ENSG0000011	SLC15A3	protein_codir	1.35117748	1.76E-05	0.00020555
ENSG0000022	RP11-417O11	lncRNA	1.35107323	0.25946635	0.40653849
ENSG0000023	AOAH-IT1	lncRNA	1.35072129	0.30024643	0.45123552
ENSG0000024	ARAP1-AS2	lncRNA	1.35070425	0.26333852	0.41069508
ENSG0000026	RP11-927P21.	lncRNA	1.35066579	0.33409378	0.4870511
ENSG0000011	DNAH6	protein_codir	1.35034559	0.00039143	0.00269007
ENSG0000027	RP11-505K9.5	lncRNA	1.34991482	0.05718912	0.13295885
ENSG0000015	USP43	protein_codir	1.34914522	0.03517488	0.09188784
ENSG0000014	TRPM2	protein_codir	1.34861415	0.0017981	0.00904454
ENSG0000024	RP11-834C11.	lncRNA	1.3481642	0.12266497	0.23496819
ENSG0000027	PGM5P3-AS1	lncRNA	1.34794871	0.02004152	0.05939344
ENSG0000007	RBFOX1	protein_codir	1.34771499	0.28628638	0.43580412
ENSG0000018	MATN1-AS1	lncRNA	1.34704529	5.29E-06	7.47E-05
ENSG0000011	HAO2	protein_codir	1.34693366	0.41095413	0.56391858
ENSG0000024	STIM2-AS1	lncRNA	1.3468759	0.15898775	0.28435838
ENSG0000025	SBNO1-AS1	lncRNA	1.34672287	0.0033287	0.01468436
ENSG0000025	KCNK4-TEX40	lncRNA	1.34659912	0.65339451	NA
ENSG0000027	RP11-73K9.3	lncRNA	1.34551789	0.19293275	0.32802723
ENSG0000019	MYL4	protein_codir	1.34466559	0.01779182	0.05422157
ENSG0000000	DBF4	protein_codir	1.34402984	6.39E-08	1.72E-06
ENSG0000014	CDH22	protein_codir	1.3437458	0.06923382	0.1537416
ENSG0000022	ZMIZ1-AS1	lncRNA	1.34339216	0.01311522	0.04294006
ENSG0000017	TCAF2	protein_codir	1.34326303	2.47E-06	3.92E-05
ENSG0000015	ADAMTS12	protein_codir	1.34316756	0.00076916	0.00463806

ENSG0000017	MAB21L3	protein_codir	1.34302679	0.06745597	0.15110575
ENSG0000027	RP11-245J9.6	lncRNA	1.34133363	0.22491622	0.36666338
ENSG0000027	RP5-1116H23	lncRNA	1.34026042	0.31986501	0.47198802
ENSG0000025	TAS2R30	protein_codir	1.33992953	0.0518979	0.12367732
ENSG0000013	SSTR4	protein_codir	1.3397123	0.33711474	0.48995522
ENSG0000027	TMEM269	protein_codir	1.33887561	0.03715901	0.09584721
ENSG0000018	TNFAIP2	protein_codir	1.33884837	4.06E-05	0.00041052
ENSG0000028	RP11-33P21.1	lncRNA	1.33795167	0.4203895	NA
ENSG0000025	CTC-321K16.1	lncRNA	1.3373375	0.18342282	0.31602135
ENSG0000013	PRDM12	protein_codir	1.33725404	0.29432077	0.44462833
ENSG0000014	DYRK3	protein_codir	1.33714107	0.00119208	0.00657453
ENSG0000023	AC006373.1	lncRNA	1.336231	0.38038156	0.5336858
ENSG0000025	RP11-732A19	lncRNA	1.33547494	0.48318248	NA
ENSG0000028	RP11-102L12.	lncRNA	1.33501743	0.00564342	0.02226096
ENSG0000008	EPB41L3	protein_codir	1.33499861	5.53E-05	0.00053522
ENSG0000013	HELZ2	protein_codir	1.33475338	4.65E-07	9.42E-06
ENSG0000026	RP11-35G9.5	lncRNA	1.33427965	0.00071366	0.00437151
ENSG0000027	CTD-2024I7.1	lncRNA	1.33379476	0.55613166	NA
ENSG0000028	EXOC3L2	protein_codir	1.33329729	1.63E-06	2.79E-05
ENSG0000023	RP11-452K12.	lncRNA	1.33247724	0.33600465	NA
ENSG0000028	CTB-189B5.4	lncRNA	1.33241656	0.2917127	NA
ENSG0000023	MIR4280HG	lncRNA	1.33202622	0.15996915	0.28564098
ENSG0000016	KCNK5	protein_codir	1.33168237	8.10E-05	0.00073187
ENSG0000026	AC139099.5	lncRNA	1.33165744	0.26779499	0.41563883
ENSG0000016	SCN11A	protein_codir	1.33164534	0.00165679	0.00852154
ENSG0000021	FIRRE	lncRNA	1.33115134	0.01838369	0.05558983
ENSG0000028	RP11-342I1.3	lncRNA	1.33110759	0.00105564	0.00597844
ENSG0000011	LIFR	protein_codir	1.33056435	0.00186101	0.00928151
ENSG0000017	FPR1	protein_codir	1.33051176	0.00272818	0.01252325
ENSG0000014	MS4A2	protein_codir	1.33034587	0.01461496	0.04674756
ENSG0000022	LINC01441	lncRNA	1.33015839	0.63756494	NA
ENSG0000014	ADCYAP1	protein_codir	1.33015333	0.00610652	0.02367852
ENSG0000012	PLAU	protein_codir	1.32996103	0.00420015	0.01770846
ENSG0000024	MIR210HG	lncRNA	1.32968396	0.00395393	0.0168563
ENSG0000018	MEX3B	protein_codir	1.3289824	3.57E-07	7.51E-06
ENSG0000026	RP11-147L13.	lncRNA	1.32887295	0.0314216	0.08429143
ENSG0000017	CA8	protein_codir	1.32883524	0.00598579	0.02329214
ENSG0000025	LNCOG	lncRNA	1.32850087	0.14709288	0.26857311
ENSG0000007	TRIP13	protein_codir	1.32819826	0.00085042	0.00502857
ENSG0000017	ZNF683	protein_codir	1.32775308	0.05322042	0.1260936
ENSG0000014	DMRTA2	protein_codir	1.3277477	0.19465792	0.33014748
ENSG0000028	RP11-1D19.1	lncRNA	1.32710215	0.15177623	0.27461903
ENSG0000027	RP11-837J7.4	lncRNA	1.3268696	0.24214261	0.38701278
ENSG0000023	AC010883.5	lncRNA	1.32684658	0.01643373	0.05107457
ENSG0000015	CLIC2	protein_codir	1.32629785	4.20E-09	1.67E-07
ENSG0000022	RP11-1086F1	lncRNA	1.32619276	0.04139008	0.10422994
ENSG0000025	RP11-197N18	lncRNA	1.32612863	0.00708316	0.02654862

ENSG0000003	MSR1	protein_codir	1.32558747	0.00807466	0.02944187
ENSG0000024	LINC02147	lncRNA	1.32534719	0.11480924	0.22348518
ENSG0000019	NUP62CL	protein_codir	1.32531674	0.00995662	0.03468742
ENSG0000026	NPY4R2	protein_codir	1.32465989	0.28295501	0.43243163
ENSG0000017	SULT1B1	protein_codir	1.32462872	0.01886983	0.05670884
ENSG0000027	RP11-285G1.1	lncRNA	1.32451586	0.0746514	0.16237767
ENSG0000006	KIF26A	protein_codir	1.32372388	1.94E-05	0.00022345
ENSG0000016	SVEP1	protein_codir	1.32326053	0.00017509	0.00138842
ENSG0000012	FNDC11	protein_codir	1.32295299	0.00656082	0.02502207
ENSG0000023	MEIKIN	protein_codir	1.32249833	0.01839563	0.05561376
ENSG0000023	SCIRT	lncRNA	1.32245305	0.05441184	0.12824089
ENSG0000026	ATP2C2-AS1	lncRNA	1.32242387	0.06199513	0.14153625
ENSG0000023	RP13-254B10.	lncRNA	1.32184946	0.03158729	0.08465368
ENSG0000028	RP11-2L8.3	lncRNA	1.32170649	0.43107458	NA
ENSG0000028	CH507-528H1	lncRNA	1.32123062	4.34E-05	0.00043365
ENSG0000027	RP1-170O19.2	lncRNA	1.32111423	0.68545729	NA
ENSG0000019	BAZ1A	protein_codir	1.32038898	1.80E-07	4.12E-06
ENSG0000023	LARGE-IT1	lncRNA	1.3198211	0.49750594	NA
ENSG0000027	RP11-234G16	lncRNA	1.31915904	0.50532005	NA
ENSG0000027	LINC02604	lncRNA	1.31912011	0.00335361	0.01477299
ENSG0000010	SIRPB1	protein_codir	1.31910146	0.01981689	0.05891327
ENSG0000010	CCL7	protein_codir	1.31812151	0.07786518	0.16731261
ENSG0000020	PRR9	protein_codir	1.31800629	0.58769238	0.71797875
ENSG0000022	LINC01635	lncRNA	1.31794108	0.25595502	0.40268158
ENSG0000014	RNF44	protein_codir	1.31792541	1.42E-07	3.39E-06
ENSG0000015	OXNAD1	protein_codir	1.31791563	2.80E-07	6.08E-06
ENSG0000008	KCNN2	protein_codir	1.31686676	0.00430754	0.01804829
ENSG0000020	DEFA1	protein_codir	1.31575206	0.29035657	0.44008388
ENSG0000015	ADPGK	protein_codir	1.31429078	2.10E-06	3.44E-05
ENSG0000017	GNG7	protein_codir	1.31305334	0.00029593	0.00212968
ENSG0000026	RP11-676J12.	lncRNA	1.31267387	0.4253043	0.57721511
ENSG0000025	CHKB-CPT1B	protein_codir	1.3123353	0.00249882	0.0116919
ENSG0000026	RP11-104N10	lncRNA	1.31206252	0.19381601	0.32923443
ENSG0000016	TONSL	protein_codir	1.31148121	0.00056651	0.00361695
ENSG0000022	AP000692.9	lncRNA	1.31092284	0.0315521	0.08457581
ENSG0000026	RP11-166B2.3	lncRNA	1.31017855	0.01934236	0.05784279
ENSG0000018	ASCL4	protein_codir	1.30986819	0.29624174	0.44669961
ENSG0000018	TSPEAR-AS2	lncRNA	1.30967269	0.03223521	0.08590666
ENSG0000014	C1QL2	protein_codir	1.30926731	0.28517841	0.43474046
ENSG0000021	LTB4R	protein_codir	1.30891376	0.00017546	0.00139063
ENSG0000014	CBX4	protein_codir	1.30754101	4.31E-08	1.22E-06
ENSG0000027	RP11-666O2.2	lncRNA	1.3071595	0.11031541	0.21714022
ENSG0000018	TMEM132C	protein_codir	1.30701499	0.05448183	0.12832919
ENSG0000025	RP11-875O11	lncRNA	1.30678242	0.30567382	NA
ENSG0000022	MIR181A1HG	lncRNA	1.30635201	0.04453926	0.11028996
ENSG0000016	CCR1	protein_codir	1.3060908	0.007776	0.02857925
ENSG0000018	KLHL33	protein_codir	1.30577566	0.00858064	0.03082363

ENSG0000022	KIAA2012-AS1	lncRNA	1.30561272	0.15843726	0.28360577
ENSG0000018	ACP7	protein_codir	1.30531492	0.27711923	0.4255314
ENSG0000023	AC002480.3	lncRNA	1.30498082	0.02372562	0.06770345
ENSG0000018	FAM53B	protein_codir	1.30477146	7.31E-05	0.00067268
ENSG0000012	PLG	protein_codir	1.30419114	0.25274779	0.39895383
ENSG0000021	PRCD	protein_codir	1.30380842	0.00680791	0.02575108
ENSG0000025	FXYD6-FXYD2	protein_codir	1.30352821	0.40061541	0.5538314
ENSG0000013	RSAD2	protein_codir	1.30330536	0.00244713	0.01149095
ENSG0000014	EFHD2	protein_codir	1.30310572	7.69E-07	1.46E-05
ENSG0000015	DRC1	protein_codir	1.30243489	0.12342406	0.2360294
ENSG0000014	VIP	protein_codir	1.3022796	0.03529576	0.09215133
ENSG0000016	DDIT4	protein_codir	1.30210964	0.00391839	0.01672991
ENSG0000019	STK31	protein_codir	1.30177786	0.01097984	0.037514
ENSG0000016	S100B	protein_codir	1.30175953	0.00854256	0.03071831
ENSG0000026	CTB-83J4.2	lncRNA	1.30057143	0.30031122	0.45130833
ENSG0000016	ZNF714	protein_codir	1.30015367	6.32E-05	0.00059611
ENSG0000010	GRK3	protein_codir	1.29946924	0.00020091	0.0015505
ENSG0000023	MFRP	protein_codir	1.29912997	0.14988241	0.27217305
ENSG0000025	RP11-96H19.1	lncRNA	1.29893548	8.11E-05	0.00073242
ENSG0000016	CIAO2A	protein_codir	1.29882704	9.39E-07	1.74E-05
ENSG0000015	PITPNC1	protein_codir	1.29845557	4.08E-07	8.42E-06
ENSG0000018	CCBE1	protein_codir	1.29839555	0.03112107	0.08376184
ENSG0000026	LINC01970	lncRNA	1.29716038	0.05358246	0.1267804
ENSG0000008	TESC	protein_codir	1.29697986	4.05E-06	5.96E-05
ENSG0000011	PTBP3	protein_codir	1.29659416	3.24E-10	1.97E-08
ENSG0000013	ADAM19	protein_codir	1.29650558	1.04E-05	0.00013263
ENSG0000010	SLC17A9	protein_codir	1.29610937	0.00468762	0.0193184
ENSG0000013	RNF144B	protein_codir	1.29582181	2.72E-05	0.00029551
ENSG0000016	SLFN5	protein_codir	1.29571066	2.62E-06	4.11E-05
ENSG0000018	LUZP2	protein_codir	1.29564924	0.05907133	0.1362805
ENSG0000016	KIR3DL1	protein_codir	1.2954754	0.1018725	0.20461918
ENSG0000023	RASA3-IT1	lncRNA	1.29528215	0.47057833	NA
ENSG0000025	RP11-579D7.2	lncRNA	1.29496228	0.48972848	NA
ENSG0000028	RP11-624A4.2	lncRNA	1.29392357	0.32295345	0.47514911
ENSG0000007	FGF4	protein_codir	1.2939173	0.57053143	NA
ENSG0000016	MS4A7	protein_codir	1.29381552	0.00124788	0.00681831
ENSG0000012	PODXL	protein_codir	1.29347889	2.06E-06	3.40E-05
ENSG0000022	ITPRIP-AS1	lncRNA	1.29335217	0.52235463	NA
ENSG0000023	TRPM2-AS	lncRNA	1.29280379	0.35310609	0.50669867
ENSG0000028	RP11-339H12	lncRNA	1.29275176	0.01585124	0.04976823
ENSG0000025	MIR9-3HG	lncRNA	1.29273457	0.02228795	0.06448026
ENSG0000023	MACORIS	lncRNA	1.29259801	0.06874174	0.15290705
ENSG0000027	RP11-548P2.2	lncRNA	1.29248516	0.08205677	0.17388431
ENSG0000025	RP11-863K10.	lncRNA	1.29189453	0.57651376	NA
ENSG0000026	XKR7	protein_codir	1.29182998	0.17657166	0.30703282
ENSG0000015	RETREG1	protein_codir	1.29121741	0.00263569	0.01218373
ENSG0000013	ABHD12B	protein_codir	1.29115009	0.17892393	0.31003005

ENSG0000016	SHANK2	protein_codir	1.29080713	0.10043493	0.20234975
ENSG0000017	S1PR1	protein_codir	1.29079189	5.81E-05	0.00055777
ENSG0000016	AK8	protein_codir	1.29062122	0.00146306	0.00773687
ENSG0000016	REEP4	protein_codir	1.29044886	1.68E-06	2.85E-05
ENSG0000013	SERPINE2	protein_codir	1.28952471	0.00455711	0.01886952
ENSG0000024	AC024704.2	lncRNA	1.2891429	0.3477775	NA
ENSG0000022	RP4-635E18.7	lncRNA	1.28870385	0.03625058	0.09401324
ENSG0000026	RP3-512B11.3	lncRNA	1.28819092	0.07975594	0.17024955
ENSG0000014	ZNF710	protein_codir	1.28814371	2.73E-07	5.95E-06
ENSG0000015	UBASH3B	protein_codir	1.2878374	0.0003805	0.0026267
ENSG0000023	SCAT8	lncRNA	1.28710124	0.13749159	0.25524383
ENSG0000017	LRRC25	protein_codir	1.28673551	0.00159019	0.00825737
ENSG0000015	PIEZO2	protein_codir	1.28668718	0.00101226	0.00578495
ENSG0000028	RP11-314O13	protein_codir	1.2863933	0.32652025	0.47881546
ENSG0000023	CTD-2192J16.	lncRNA	1.2862199	0.50724194	NA
ENSG0000007	IGF2BP2	protein_codir	1.28613116	0.00089958	0.0052608
ENSG0000016	EVPL	protein_codir	1.28596571	0.00251393	0.01173575
ENSG0000016	SLC2A6	protein_codir	1.28592595	0.00055288	0.00354798
ENSG0000012	MIS18BP1	protein_codir	1.28543593	2.10E-06	3.44E-05
ENSG0000025	KB-1507C5.3	lncRNA	1.2851948	0.27797773	0.42658878
ENSG0000013	CPM	protein_codir	1.28488095	0.00173194	0.00880797
ENSG0000027	ENSG0000027	protein_codir	1.28362925	0.04437776	0.10996894
ENSG0000012	DLL4	protein_codir	1.28360987	0.00076266	0.00461033
ENSG0000026	CTD-2529O21	lncRNA	1.28353821	0.31669181	0.46842839
ENSG0000010	LIG1	protein_codir	1.28310989	8.73E-06	0.00011494
ENSG0000028	RP11-305E17.	lncRNA	1.2822271	0.36931038	0.52286749
ENSG0000020	AGER	protein_codir	1.282102	0.01225242	0.04074797
ENSG0000017	NRROS	protein_codir	1.28209946	0.00662901	0.02521598
ENSG0000015	ROBO4	protein_codir	1.28208229	4.39E-05	0.00043743
ENSG0000018	CLRN3	protein_codir	1.28096944	0.34996152	NA
ENSG0000013	MSI1	protein_codir	1.27991566	0.01533781	0.04850358
ENSG0000027	PDE7A-DT	lncRNA	1.27989852	0.29469313	0.44502046
ENSG0000017	GIMAP8	protein_codir	1.27924539	0.00053119	0.00343434
ENSG0000017	AMIGO3	protein_codir	1.27910273	0.041617	0.10471545
ENSG0000000	SCN4A	protein_codir	1.27892598	0.00880559	0.03147946
ENSG0000023	SLC25A25-AS1	lncRNA	1.27880718	0.00252804	0.01178077
ENSG0000010	CARD8	protein_codir	1.27873818	0.00010827	0.00092954
ENSG0000026	AC005262.3	lncRNA	1.27870187	0.57974009	NA
ENSG0000012	C3	protein_codir	1.27849291	0.00021009	0.00160874
ENSG0000026	CTD-2008P7.1	lncRNA	1.27812748	0.5173212	NA
ENSG0000025	RP11-30K9.5	lncRNA	1.27761595	0.24597763	0.39119607
ENSG0000023	TXNDC5	protein_codir	1.27751942	0.00015353	0.00124798
ENSG0000013	SLC22A23	protein_codir	1.27749677	1.56E-06	2.70E-05
ENSG0000024	KIR2DL3	protein_codir	1.27747127	0.08369893	0.17636286
ENSG0000025	CTD-2329K10	lncRNA	1.2773257	0.51088384	NA
ENSG0000028	RP1-221C16.8	lncRNA	1.27731021	0.05464565	0.12861266
ENSG0000025	REELD1	protein_codir	1.27707113	0.04584343	0.11266087

ENSG0000025	RP11-575F12.	lncRNA	1.27568128	0.21118164	0.3505732
ENSG0000015	ADGRF4	protein_codir	1.27558792	0.4861625	0.63335731
ENSG0000028	LINC00279	lncRNA	1.27496239	0.40079575	NA
ENSG0000015	AFAP1L1	protein_codir	1.27429116	0.00068308	0.00421979
ENSG0000016	SH2B2	protein_codir	1.27418767	0.00078416	0.00470736
ENSG0000019	ENTPD7	protein_codir	1.27377905	0.00017202	0.00137166
ENSG0000028	CTA-351O6.1	lncRNA	1.27325124	0.65320998	NA
ENSG0000022	RP11-106M7.	lncRNA	1.27261395	0.34012549	0.49311144
ENSG0000010	CTSC	protein_codir	1.27210066	1.86E-05	0.00021597
ENSG0000025	RP11-596D21	lncRNA	1.27157486	0.45032734	NA
ENSG0000023	L3MBTL2-AS1	lncRNA	1.27148994	2.99E-05	0.00032039
ENSG0000017	GRAMD1C	protein_codir	1.27103501	0.00048643	0.00320018
ENSG0000016	RP11-93B14.6	lncRNA	1.27094127	0.07662037	0.16539701
ENSG0000027	RP11-544M22	protein_codir	1.27063246	0.01355858	0.04406416
ENSG0000018	ABHD16B	protein_codir	1.27058726	0.09592506	0.19544483
ENSG0000024	RGMB-AS1	lncRNA	1.27042842	0.07811747	0.16768514
ENSG0000011	DLX2	protein_codir	1.27041199	0.17976555	0.31111752
ENSG0000017	HS6ST2	protein_codir	1.27039619	0.00930361	0.032919
ENSG0000018	OSBP2	protein_codir	1.27028087	0.00017309	0.00137737
ENSG0000007	CEACAM1	protein_codir	1.27021395	5.61E-06	7.87E-05
ENSG0000011	ULBP1	protein_codir	1.27015814	0.02030751	0.0600109
ENSG0000018	AC010969.1	lncRNA	1.26988863	0.38016454	0.53357105
ENSG0000026	PPIAL4A	protein_codir	1.26961977	0.69713145	NA
ENSG0000024	TEX35	protein_codir	1.26961977	0.69713145	NA
ENSG0000022	AC009229.6	lncRNA	1.26961977	0.69713145	NA
ENSG0000028	RP11-198M19	lncRNA	1.26961977	0.69713145	NA
ENSG0000028	RP11-385A18	lncRNA	1.26961977	0.69713145	NA
ENSG0000028	RP11-413B21.	lncRNA	1.26961977	0.69713145	NA
ENSG0000028	RP11-122D19	lncRNA	1.26961977	0.69713145	NA
ENSG0000028	RP11-15B4.2	lncRNA	1.26961977	0.69713145	NA
ENSG0000024	ZBTB20-AS5	lncRNA	1.26961977	0.69713145	NA
ENSG0000022	RP11-135A1.3	lncRNA	1.26961977	0.69713145	NA
ENSG0000028	RP11-12P19.4	lncRNA	1.26961977	0.69713145	NA
ENSG0000024	RP11-72L22.1	lncRNA	1.26961977	0.69713145	NA
ENSG0000013	NMUR2	protein_codir	1.26961977	0.69713145	NA
ENSG0000025	LINC01932	lncRNA	1.26961977	0.69713145	NA
ENSG0000020	TSBP1	protein_codir	1.26961977	0.69713145	NA
ENSG0000024	RP11-257K9.8	protein_codir	1.26961977	0.69713145	NA
ENSG0000024	HOTTIP	lncRNA	1.26961977	0.69713145	NA
ENSG0000025	LINC01606	lncRNA	1.26961977	0.69713145	NA
ENSG0000025	RP11-320N21	lncRNA	1.26961977	0.69713145	NA
ENSG0000017	TRHR	protein_codir	1.26961977	0.69713145	NA
ENSG0000014	PLPPR1	protein_codir	1.26961977	0.69713145	NA
ENSG0000025	LINC01475	lncRNA	1.26961977	0.69713145	NA
ENSG0000023	NRXN2-AS1	lncRNA	1.26961977	0.69713145	NA
ENSG0000027	RP11-255P5.3	lncRNA	1.26961977	0.69713145	NA
ENSG0000027	RP11-282A11	lncRNA	1.26961977	0.69713145	NA

ENSG0000025	RP11-406H23	lncRNA	1.26961977	0.69713145	NA
ENSG0000025	RP11-1079K1(	lncRNA	1.26961977	0.69713145	NA
ENSG0000028	RP11-701C7.1	lncRNA	1.26961977	0.69713145	NA
ENSG0000020	SERPINB11	protein_codir	1.26961977	0.69713145	NA
ENSG0000028	RP4-610C12.5	lncRNA	1.26961977	0.69713145	NA
ENSG0000016	WFDC13	protein_codir	1.26961977	0.69713145	NA
ENSG0000024	AL109761.5	lncRNA	1.26961977	0.69713145	NA
ENSG0000023	AF015262.2	lncRNA	1.26961977	0.69713145	NA
ENSG0000023	RP1-5O6.5	lncRNA	1.26961977	0.69713145	NA
ENSG0000015	XAGE2	protein_codir	1.26961977	0.69713145	NA
ENSG0000027	TEX28	protein_codir	1.26961977	0.69713145	NA
ENSG0000023	AC079305.11	lncRNA	1.26945895	0.15907912	0.28447817
ENSG0000016	SEC11C	protein_codir	1.26905689	0.00013326	0.00110866
ENSG0000027	AP001059.6	lncRNA	1.26897408	0.18060783	0.31218401
ENSG0000028	CTB-31E20.1	protein_codir	1.26895404	0.66462496	NA
ENSG0000025	RP6-91H8.2	lncRNA	1.26888922	0.66165761	NA
ENSG0000025	LINC02842	lncRNA	1.26887961	0.66122015	NA
ENSG0000023	AC141930.2	lncRNA	1.26886836	0.66070888	NA
ENSG0000025	CTD-2555A7.2	lncRNA	1.26886749	0.66066975	NA
ENSG0000024	UGT1A7	protein_codir	1.26885546	0.66012435	NA
ENSG0000028	RP11-562N2.2	lncRNA	1.26884149	0.65949218	NA
ENSG0000022	CNIH3-AS1	lncRNA	1.26883625	0.65925581	NA
ENSG0000022	AC079779.7	lncRNA	1.26883402	0.65915504	NA
ENSG0000027	C17orf50	protein_codir	1.2688326	0.65909106	NA
ENSG0000027	AC002128.5	lncRNA	1.26883006	0.65897621	NA
ENSG0000019	GRM3	protein_codir	1.26882728	0.65885112	NA
ENSG0000025	MIR124-1HG	lncRNA	1.26881271	0.65819476	NA
ENSG0000025	LINC00524	lncRNA	1.26876991	0.65627633	NA
ENSG0000028	RP11-320E16.	lncRNA	1.2687676	0.65617312	NA
ENSG0000022	RP11-252P19.	lncRNA	1.26874437	0.65513739	NA
ENSG0000023	AC006007.1	lncRNA	1.2687397	0.65492973	NA
ENSG0000021	AC099552.4	lncRNA	1.26873639	0.65478252	NA
ENSG0000025	FER1L6-AS2	lncRNA	1.26873021	0.65450821	NA
ENSG0000023	RP11-520A21	lncRNA	1.26872704	0.65436737	NA
ENSG0000026	RP11-575I8.1	lncRNA	1.26872248	0.65416506	NA
ENSG0000020	TRIM15	protein_codir	1.26871416	0.65379654	NA
ENSG0000022	LINC01776	lncRNA	1.2687026	0.65328508	NA
ENSG0000027	PADI6	protein_codir	1.26867745	0.65217545	NA
ENSG0000023	RP11-561O23	lncRNA	1.26866303	0.65154124	NA
ENSG0000021	KRTAP2-2	protein_codir	1.26866144	0.65147105	NA
ENSG0000027	BANCR	lncRNA	1.26864633	0.65080852	NA
ENSG0000024	CASC8	lncRNA	1.26863928	0.65049945	NA
ENSG0000027	RP11-54H7.4	lncRNA	1.26863695	0.65039734	NA
ENSG0000025	RP11-18H21.2	lncRNA	1.26863135	0.65015272	NA
ENSG0000028	RP11-242C24.	lncRNA	1.26863126	0.6501488	NA
ENSG0000027	RP11-438D14	lncRNA	1.26863037	0.65010976	NA
ENSG0000022	AC009227.2	lncRNA	1.26861163	0.64929151	NA

ENSG0000026	RP11-378I6.1	lncRNA	1.26861041	0.64923825	NA
ENSG0000026	LINC01894	lncRNA	1.26826506	0.17086624	0.29994442
ENSG0000013	PCNX2	protein_codir	1.26802492	5.95E-07	1.17E-05
ENSG0000025	LINC01605	lncRNA	1.26770067	0.02803448	0.07726127
ENSG0000018	WBP2NL	protein_codir	1.26765646	0.0160917	0.05035149
ENSG0000027	RP11-282A11	lncRNA	1.26729248	0.50996398	NA
ENSG0000016	CDYL2	protein_codir	1.26721017	1.98E-07	4.49E-06
ENSG0000024	AC002116.8	lncRNA	1.26700643	0.09362017	0.19200929
ENSG0000027	RP11-60L3.6	lncRNA	1.26686379	0.5814693	NA
ENSG0000009	CD209	protein_codir	1.26684552	0.0379205	0.09729347
ENSG0000023	RP11-436D10	lncRNA	1.2668252	0.58012892	NA
ENSG0000022	CTD-2384B11	lncRNA	1.26678094	0.57859853	NA
ENSG0000028	RP11-421J10.	lncRNA	1.26677627	0.5784374	NA
ENSG0000017	LINC02873	lncRNA	1.26676746	0.57813387	NA
ENSG0000025	CTA-363E6.1	lncRNA	1.26675058	0.57755288	NA
ENSG0000026	AC009120.5	lncRNA	1.26674964	0.57752047	NA
ENSG0000022	AC007879.4	lncRNA	1.26670469	0.5759792	NA
ENSG0000025	PITPNM2-AS1	lncRNA	1.26668005	0.57513736	NA
ENSG0000028	RP11-473C18.	lncRNA	1.26668005	0.57513736	NA
ENSG0000013	FITM1	protein_codir	1.26665	0.00548169	0.02179104
ENSG0000016	LINC01116	lncRNA	1.26623904	0.00026783	0.00196216
ENSG0000012	PLBD1	protein_codir	1.26598035	6.90E-05	0.00064177
ENSG0000021	AC114730.8	lncRNA	1.26573726	0.1751475	0.30526749
ENSG0000018	GCNT1	protein_codir	1.26560712	0.0020118	0.0098766
ENSG0000013	CEP85	protein_codir	1.2654813	0.0003831	0.00263936
ENSG0000018	IFITM1	protein_codir	1.26475754	4.42E-07	9.03E-06
ENSG0000016	ZNF385A	protein_codir	1.26475207	2.04E-06	3.36E-05
ENSG0000013	HSPA12B	protein_codir	1.26461814	7.31E-05	0.00067268
ENSG0000017	LINC00324	lncRNA	1.264563	0.00066279	0.00411931
ENSG0000017	RGS19	protein_codir	1.26446968	0.00010758	0.00092451
ENSG0000026	NCMAP-DT	lncRNA	1.26436102	0.50438153	NA
ENSG0000028	CTD-2266L18.	lncRNA	1.26432045	0.50327036	NA
ENSG0000014	CELA2A	protein_codir	1.26429353	0.50253513	NA
ENSG0000027	SND1-IT1	lncRNA	1.26423006	0.05573752	0.13049677
ENSG0000018	PEAK3	protein_codir	1.26350769	0.00845548	0.03047634
ENSG0000017	NINJ2	protein_codir	1.26346975	8.50E-06	0.00011257
ENSG0000016	COL6A3	protein_codir	1.26336096	0.00058292	0.00370464
ENSG0000023	AC004019.13	lncRNA	1.26333024	0.4570056	NA
ENSG0000023	RP11-57H12.2	lncRNA	1.26281657	0.69867911	NA
ENSG0000015	CDCP2	protein_codir	1.2619947	0.28366133	0.43328868
ENSG0000027	ORAI1	protein_codir	1.26182789	2.45E-06	3.90E-05
ENSG0000009	SEMA6A	protein_codir	1.26172912	0.00022151	0.00167984
ENSG0000023	AC016768.1	lncRNA	1.26149025	0.69898097	NA
ENSG0000028	RP11-373A6.2	lncRNA	1.26149025	0.69898097	NA
ENSG0000025	RP11-133K1.6	lncRNA	1.26149025	0.69898097	NA
ENSG0000020	LINC01531	lncRNA	1.26149025	0.69898097	NA
ENSG0000021	SIPA1	protein_codir	1.26124551	6.60E-06	9.01E-05

ENSG0000012	BHLHE23	protein_codir	1.2611498	0.66775896	NA
ENSG0000023	MYO16-AS1	lncRNA	1.26114191	0.58096598	NA
ENSG0000022	EGFR-AS1	lncRNA	1.26110807	0.66418711	NA
ENSG0000013	MYH8	protein_codir	1.26109298	0.66290799	NA
ENSG0000016	PPP1R14D	protein_codir	1.2610745	0.66135038	NA
ENSG0000025	AF131216.5	lncRNA	1.26107134	0.66108459	NA
ENSG0000028	CTD-3212A4.2	lncRNA	1.2609859	0.65400902	NA
ENSG0000015	SUSD3	protein_codir	1.2609173	0.0009844	0.00565732
ENSG0000018	SSTR2	protein_codir	1.26055265	7.61E-05	0.00069361
ENSG0000013	TBC1D4	protein_codir	1.26037676	2.89E-09	1.23E-07
ENSG0000027	RP11-269M20	lncRNA	1.26011334	0.59085839	NA
ENSG0000013	ZNF670-ZNF6	protein_codir	1.26006292	0.58762103	NA
ENSG0000018	KRT12	protein_codir	1.26004933	0.58675538	NA
ENSG0000017	FOXE1	protein_codir	1.26002894	0.58546107	NA
ENSG0000026	PPIAL4C	protein_codir	1.26002411	0.39571863	0.54862708
ENSG0000026	CTD-2083E4.7	lncRNA	1.25997836	0.58227562	NA
ENSG0000026	CTC-527H23.3	lncRNA	1.25980948	0.27828868	0.42692364
ENSG0000020	AC015969.3	lncRNA	1.25973279	0.44906946	NA
ENSG0000028	RP1-111C20.3	lncRNA	1.25950577	0.16121759	0.28726082
ENSG0000010	RNF43	protein_codir	1.25935056	0.02317168	0.06648679
ENSG0000026	AC068499.10	lncRNA	1.25904012	0.43614539	0.5873969
ENSG0000016	ABCG1	protein_codir	1.25903547	0.00055313	0.00354875
ENSG0000005	TMCC3	protein_codir	1.25897422	0.00023116	0.00173592
ENSG0000025	TAS2R31	protein_codir	1.25857729	0.03890996	0.09932469
ENSG0000025	RP13-977J11.4	lncRNA	1.25851714	0.50311397	NA
ENSG0000028	RP11-167J8.5	lncRNA	1.25851538	0.50303118	NA
ENSG0000010	RARRES2	protein_codir	1.2583083	5.04E-06	7.16E-05
ENSG0000014	ABRACL	protein_codir	1.25818707	1.84E-06	3.07E-05
ENSG0000028	CTD-2062A1.3	lncRNA	1.25766075	0.16593749	0.29356864
ENSG0000017	CALCB	protein_codir	1.25736978	0.03304734	0.08766496
ENSG0000000	THSD7A	protein_codir	1.25729748	2.56E-05	0.000282
ENSG0000015	GUCY1A2	protein_codir	1.25721907	0.01809982	0.05489973
ENSG0000027	RP11-345P4.1	lncRNA	1.2571873	0.22991487	0.37232589
ENSG0000026	PCAT19	lncRNA	1.2571618	0.00190614	0.00946048
ENSG0000021	PPM1N	protein_codir	1.25643474	0.00626082	0.02414128
ENSG0000018	A3GALT2	protein_codir	1.2560991	0.35317465	0.50673682
ENSG0000018	PRSS57	protein_codir	1.25560256	0.23076021	0.37331441
ENSG0000017	HES7	protein_codir	1.25552487	0.0299726	0.08140853
ENSG0000020	RP11-261N11	lncRNA	1.25544101	0.70035835	NA
ENSG0000026	RP11-368L12.	lncRNA	1.25526991	0.66744171	NA
ENSG0000028	RP11-509I21.4	lncRNA	1.25526511	0.66658448	NA
ENSG0000018	FYB2	protein_codir	1.25526483	0.66653518	NA
ENSG0000018	OR1R1P	protein_codir	1.25526394	0.66637597	NA
ENSG0000026	RP11-583F2.2	lncRNA	1.2552614	0.6659242	NA
ENSG0000026	RP11-149I2.4	lncRNA	1.25526007	0.66568736	NA
ENSG0000027	OR4E1	protein_codir	1.25525661	0.66507332	NA
ENSG0000028	RP11-757O6.8	lncRNA	1.25524423	0.66288874	NA

ENSG0000026	RP11-10O17.3	lncRNA	1.25522352	0.65927908	NA
ENSG0000028	RP11-844P9.5	lncRNA	1.2552048	0.65606341	NA
ENSG0000028	RP11-119P22.	lncRNA	1.25520365	0.6558675	NA
ENSG0000026	AF003625.3	lncRNA	1.25477021	0.59145654	NA
ENSG0000019	PPP1R14C	protein_codir	1.25466008	0.0696953	0.15453032
ENSG0000028	RP11-550P23.	lncRNA	1.25448377	0.3195708	0.47162946
ENSG0000013	GSTM1	protein_codir	1.25432898	0.36491229	0.51883442
ENSG0000025	RP11-829H16	lncRNA	1.25424683	0.14287772	0.2627892
ENSG0000025	HOXC-AS2	lncRNA	1.25420994	0.08728102	0.18206342
ENSG0000022	AC017101.10	lncRNA	1.25385625	0.70071935	NA
ENSG0000028	SSU72P5	protein_codir	1.25385625	0.70071935	NA
ENSG0000015	GPR158	protein_codir	1.25282813	0.06894051	0.15323812
ENSG0000019	ITPRIPL1	protein_codir	1.25262561	0.00041718	0.00282353
ENSG0000010	RCVRN	protein_codir	1.25251562	0.03660289	0.09474013
ENSG0000014	AZIN2	protein_codir	1.25230019	0.00036882	0.00255883
ENSG0000018	ZSCAN4	protein_codir	1.25223176	0.20061435	0.33739075
ENSG0000017	HTR1F	protein_codir	1.25190911	0.00476129	0.01952868
ENSG0000026	MALT1-AS1	lncRNA	1.25161914	0.02602433	0.07284807
ENSG0000011	MUC5B	protein_codir	1.25149878	0.51074788	NA
ENSG0000019	SULT1A1	protein_codir	1.25125098	0.0017182	0.00875639
ENSG0000012	SFRP5	protein_codir	1.25082019	0.10110863	0.20346182
ENSG0000028	RP5-855F14.4	lncRNA	1.25076887	0.70142283	NA
ENSG0000002	C8B	protein_codir	1.2507538	0.70142627	NA
ENSG0000016	CYP2C19	protein_codir	1.2507538	0.70142627	NA
ENSG0000028	RP11-1081L13	lncRNA	1.25072138	0.67123887	NA
ENSG0000022	MYCBP2-AS2	lncRNA	1.25071896	0.66982484	NA
ENSG0000025	CTD-2616J11.	lncRNA	1.25071681	0.66857849	NA
ENSG0000022	RP11-12A16.3	lncRNA	1.25066377	0.43441077	0.58586062
ENSG0000014	ASGR1	protein_codir	1.25051265	0.00147239	0.00777285
ENSG0000028	RP4-777O23.3	protein_codir	1.25039066	0.37436381	0.52797639
ENSG0000026	RP11-334C17.	lncRNA	1.25015186	0.01215816	0.04050765
ENSG0000015	SLC5A11	protein_codir	1.2495016	0.08689372	0.18154346
ENSG0000027	RP4-673M15.	lncRNA	1.24853769	0.00175073	0.00887657
ENSG0000017	TMEM196	protein_codir	1.24847343	0.14066357	0.2599403
ENSG0000015	C1QC	protein_codir	1.24842575	0.00269924	0.01242219
ENSG0000014	GABRB2	protein_codir	1.24840126	0.00665478	0.02528267
ENSG0000022	RP11-501J20.	lncRNA	1.24838803	0.24464531	0.38963807
ENSG0000028	RP11-14A10.1	lncRNA	1.24825093	0.01600185	0.05013283
ENSG0000023	RP1-12G14.7	lncRNA	1.24822931	0.70200167	NA
ENSG0000011	APOC3	protein_codir	1.24822931	0.70200167	NA
ENSG0000010	F9	protein_codir	1.24822931	0.70200167	NA
ENSG0000013	ITGB4	protein_codir	1.24816829	0.00016733	0.00134054
ENSG0000013	SP110	protein_codir	1.24800041	1.50E-07	3.53E-06
ENSG0000028	RP11-131M11	lncRNA	1.2479856	0.67770977	NA
ENSG0000023	NCK1-DT	lncRNA	1.24790108	0.00089959	0.0052608
ENSG0000012	BHLHE41	protein_codir	1.24789091	2.00E-05	0.00022937
ENSG0000016	SEMA6B	protein_codir	1.24780573	0.00022733	0.0017127

ENSG0000019	ZNF729	protein_codir	1.24776583	0.32782842	0.48022387
ENSG0000011	FOLR3	protein_codir	1.24767631	0.16024082	0.28599157
ENSG0000023	THBS3-AS1	lncRNA	1.2471973	0.01127461	0.03823731
ENSG0000017	DSG4	protein_codir	1.24705425	0.70226956	NA
ENSG0000020	XAGE1A	protein_codir	1.24705425	0.70226956	NA
ENSG0000014	VWDE	protein_codir	1.24705325	0.12923024	0.24409076
ENSG0000024	RP11-17A1.3	lncRNA	1.24697944	0.02339036	0.0669404
ENSG0000026	CTD-2373H9.5	lncRNA	1.24665565	0.59002415	NA
ENSG0000014	TMEM236	protein_codir	1.24663869	0.00472978	0.01942913
ENSG0000027	RP4-678D15.1	lncRNA	1.24620914	0.51333864	NA
ENSG0000017	SCN4B	protein_codir	1.24602895	0.0088863	0.03173103
ENSG0000028	MYOCOS	protein_codir	1.24588683	0.26094544	0.40818447
ENSG0000014	TAF4B	protein_codir	1.2454474	8.75E-06	0.00011502
ENSG0000015	RUNX1	protein_codir	1.2453597	7.14E-05	0.00066001
ENSG0000015	CES5A	protein_codir	1.24509663	0.60161434	0.72868747
ENSG0000022	LINC02611	lncRNA	1.24507034	0.0016038	0.00830953
ENSG0000011	IFIT3	protein_codir	1.24505412	0.00073395	0.00447103
ENSG0000022	PELATON	lncRNA	1.24465249	0.04169995	0.10490506
ENSG0000011	SLC6A12	protein_codir	1.24423737	0.0384748	0.09842307
ENSG0000020	FAM99A	lncRNA	1.24403062	0.70295905	NA
ENSG0000026	RP11-118E18.	lncRNA	1.24403062	0.70295905	NA
ENSG0000026	RP11-21J18.1	lncRNA	1.24403062	0.70295905	NA
ENSG0000026	CTC-513N18.6	lncRNA	1.24403062	0.70295905	NA
ENSG0000020	HLA-E	protein_codir	1.24368707	7.71E-13	1.19E-10
ENSG0000014	ILDR2	protein_codir	1.24293637	0.05645081	0.13173128
ENSG0000019	RGPD4	protein_codir	1.24231429	0.01078707	0.03698979
ENSG0000018	ZNF467	protein_codir	1.24225142	8.05E-05	0.00072844
ENSG0000028	CTD-2017F17.	lncRNA	1.24224651	0.12918957	0.24404733
ENSG0000020	PSMB8	protein_codir	1.2412462	3.94E-07	8.20E-06
ENSG0000028	RP11-18O11.3	lncRNA	1.2412254	0.50720147	NA
ENSG0000016	RALGDS	protein_codir	1.2411303	1.32E-07	3.19E-06
ENSG0000014	RNF175	protein_codir	1.24104124	0.08016884	0.17091959
ENSG0000028	RP11-167N4.8	lncRNA	1.24100794	0.15010412	0.27243237
ENSG0000023	RP1-45C12.1	lncRNA	1.24084453	0.01969725	0.05859954
ENSG0000026	PPM1J-DT	lncRNA	1.24052615	0.58252266	NA
ENSG0000016	MEIOB	protein_codir	1.24051234	0.5842275	NA
ENSG0000026	AC100830.5	lncRNA	1.24051073	0.5844272	NA
ENSG0000024	LINC01513	lncRNA	1.24049293	0.5866413	NA
ENSG0000025	CTD-2501E16.	lncRNA	1.24046181	0.59055376	NA
ENSG0000028	RP11-134O15	lncRNA	1.24045658	0.59121672	NA
ENSG0000016	ACTBL2	protein_codir	1.24044482	0.59271251	NA
ENSG0000026	AC005306.3	lncRNA	1.24017803	0.02560608	0.0719596
ENSG0000014	PLPP2	protein_codir	1.24003845	0.01219456	0.04059955
ENSG0000025	CTD-2331C18	lncRNA	1.23998081	0.65915711	NA
ENSG0000028	RP11-215D10	lncRNA	1.23993689	0.66636284	NA
ENSG0000011	KCNJ13	protein_codir	1.23991445	0.67011933	NA
ENSG0000025	LINC00639	lncRNA	1.23991264	0.00352953	0.01537628

ENSG0000028	RP11-753C18.	lncRNA	1.23986081	0.33440156	NA
ENSG0000015	TRIM63	protein_codir	1.23978396	0.14376358	0.26394844
ENSG0000023	AC068196.1	lncRNA	1.23972623	0.70394101	NA
ENSG0000026	LINC01443	lncRNA	1.23972623	0.70394101	NA
ENSG0000026	RP11-791M20	lncRNA	1.23972623	0.70394101	NA
ENSG0000022	RP11-218C14.	lncRNA	1.23926139	0.51954018	NA
ENSG0000009	SERPIND1	protein_codir	1.23903817	0.25056653	0.39653054
ENSG0000015	LRRC43	protein_codir	1.23899524	0.00443843	0.01847062
ENSG0000022	LINC02554	lncRNA	1.23877236	0.59225311	NA
ENSG0000007	WNT8B	protein_codir	1.23836642	0.51533015	NA
ENSG0000013	FXYD6	protein_codir	1.23833549	0.00753339	0.02787676
ENSG0000017	C3AR1	protein_codir	1.23803532	0.00935097	0.03304923
ENSG0000007	MGAT4A	protein_codir	1.23801692	7.60E-05	0.00069289
ENSG0000027	RP11-302M6.	lncRNA	1.23783054	0.59364054	NA
ENSG0000016	SCNN1B	protein_codir	1.23756386	0.00793116	0.02899901
ENSG0000024	PLA2G4E-AS1	lncRNA	1.23740081	0.59416607	NA
ENSG0000025	AC007950.1	lncRNA	1.23738777	0.59540445	NA
ENSG0000021	TAS2R13	protein_codir	1.2373001	0.21311823	0.35281261
ENSG0000027	CTD-2024F21.	lncRNA	1.23727187	0.47261903	NA
ENSG0000027	AFAP1-AS1	lncRNA	1.23713883	0.00039993	0.00273281
ENSG0000025	RP11-156K13.	lncRNA	1.23695281	0.16381862	0.29073252
ENSG0000018	CHRM4	protein_codir	1.23685959	0.00020963	0.00160607
ENSG0000010	PPY	protein_codir	1.23667191	0.67417806	NA
ENSG0000023	IRGM	protein_codir	1.23662743	0.05523914	0.12956055
ENSG0000026	RP11-15A1.3	lncRNA	1.23661368	0.00013653	0.00113313
ENSG0000027	LINC01666	lncRNA	1.2362205	0.67517934	NA
ENSG0000013	BRDT	protein_codir	1.2359414	0.34493033	0.49790903
ENSG0000027	RP11-734K23.	lncRNA	1.2356757	0.00531799	0.02129045
ENSG0000018	BEGAIN	protein_codir	1.23531228	0.00256789	0.01192017
ENSG0000016	CIP2A	protein_codir	1.23530724	1.30E-06	2.29E-05
ENSG0000018	TBX1	protein_codir	1.23505017	0.04338608	0.10805805
ENSG0000026	RP11-304L19.	lncRNA	1.23501262	0.42469273	0.57664011
ENSG0000011	C7	protein_codir	1.23489138	0.0069938	0.02632802
ENSG0000028	RP11-779M2.	lncRNA	1.23370909	0.7053145	NA
ENSG0000007	CAMK2A	protein_codir	1.23351704	0.0250512	0.07069726
ENSG0000022	KIF25-AS1	lncRNA	1.23331657	0.402694	0.55566344
ENSG0000026	RP11-235E17.	lncRNA	1.2332386	0.28266837	0.43217827
ENSG0000023	GS1-115G20.1	lncRNA	1.2329606	0.40556687	NA
ENSG0000010	ATP8B4	protein_codir	1.23279719	0.00041803	0.00282504
ENSG0000023	MCF2L-AS1	lncRNA	1.23237092	0.11850047	0.22889593
ENSG0000016	PAQR4	protein_codir	1.23216178	0.0004639	0.00307635
ENSG0000007	SLC1A3	protein_codir	1.23189965	0.00940148	0.03317604
ENSG0000027	RP11-274B21.	lncRNA	1.23186632	0.02521629	0.07110504
ENSG0000015	RAB11FIP1	protein_codir	1.23164259	1.07E-05	0.00013562
ENSG0000028	RP11-534G20	lncRNA	1.23161222	0.12430074	0.23723731
ENSG0000023	RP11-421L21.	lncRNA	1.23135969	0.08873488	0.18431657
ENSG0000025	LINC02289	lncRNA	1.23108314	0.01748023	0.05349036

ENSG0000027	AC000068.9	lncRNA	1.2308867	0.3073093	0.45903318
ENSG0000028	RP11-96C13.1	lncRNA	1.23061952	0.51580632	NA
ENSG0000003	IKZF2	protein_codir	1.23053799	1.37E-05	0.00016742
ENSG0000013	APOE	protein_codir	1.23051802	0.0187352	0.0563996
ENSG0000017	ACOT12	protein_codir	1.23025438	0.46539526	NA
ENSG0000017	SH2D4B	protein_codir	1.22993546	0.1269237	0.24090384
ENSG0000016	OR1Q1	protein_codir	1.22975656	0.52355985	NA
ENSG0000023	TNF	protein_codir	1.22970683	0.05176324	0.12350558
ENSG0000018	TMEM86B	protein_codir	1.22943968	0.00219211	0.01055546
ENSG0000012	STIL	protein_codir	1.22901484	0.00278387	0.01271336
ENSG0000027	LINC02084	lncRNA	1.2289918	0.04469529	0.11059701
ENSG0000027	RP4-713A8.1	lncRNA	1.2289627	0.44545057	0.59652581
ENSG0000010	CBLN1	protein_codir	1.22879895	0.01547765	0.04883397
ENSG0000015	GLYATL2	protein_codir	1.22858869	0.09889763	0.20007057
ENSG0000025	RP11-281P23.	lncRNA	1.22855048	0.39655078	0.54939469
ENSG0000024	THBS4-AS1	lncRNA	1.2281463	0.03824062	0.09794195
ENSG0000000	NOS2	protein_codir	1.22706072	0.0338226	0.089233
ENSG0000011	CYB561D2	protein_codir	1.22692985	1.19E-06	2.13E-05
ENSG0000013	ZNF20	protein_codir	1.2267522	0.00089085	0.00522404
ENSG0000012	PLP1	protein_codir	1.22653091	0.05280314	0.12532635
ENSG0000018	DNAH17	protein_codir	1.22587039	0.0568921	0.13248122
ENSG0000022	AC009229.5	lncRNA	1.22548043	0.15995463	0.28564098
ENSG0000016	ORAI2	protein_codir	1.22520178	0.0001024	0.00088664
ENSG0000023	FAM53B-AS1	lncRNA	1.22515903	0.40702286	NA
ENSG0000010	TBC1D9	protein_codir	1.22490134	2.99E-05	0.00032039
ENSG0000010	COL20A1	protein_codir	1.22473182	0.21244807	0.35206003
ENSG0000015	IRX6	protein_codir	1.22472198	0.11018886	0.21696843
ENSG0000023	PRKG1-AS1	lncRNA	1.22451207	0.52117971	NA
ENSG0000028	CTD-3214H19	protein_codir	1.22424344	0.30822877	0.45994527
ENSG0000010	FBXW7	protein_codir	1.22410491	3.56E-07	7.50E-06
ENSG0000023	ZNF687-AS1	lncRNA	1.22389347	0.03679927	0.09513251
ENSG0000027	AJ011931.1	lncRNA	1.22372105	0.36074003	0.51462423
ENSG0000018	CARD9	protein_codir	1.22368174	0.00560682	0.02216204
ENSG0000017	PCDHB1	protein_codir	1.22328649	0.52572609	NA
ENSG0000025	RP11-325L7.2	lncRNA	1.22261627	0.59610205	NA
ENSG0000026	CTD-2616J11.	lncRNA	1.22258326	0.26916968	0.41694053
ENSG0000019	H3C4	protein_codir	1.22176203	0.0717232	0.15784819
ENSG0000012	TRPV5	protein_codir	1.22145682	0.30335024	0.45466321
ENSG0000013	GPR84	protein_codir	1.22140028	0.00700751	0.02636884
ENSG0000023	RP11-761N21	lncRNA	1.2209397	0.11325619	0.22122602
ENSG0000026	RP11-24M17.	lncRNA	1.22071983	0.49198238	NA
ENSG0000007	TRAF4	protein_codir	1.22051259	2.16E-05	0.00024493
ENSG0000016	VWA2	protein_codir	1.22021865	0.06092029	0.13960164
ENSG0000011	QPCT	protein_codir	1.22001107	0.00293753	0.01326378
ENSG0000025	LINC02769	lncRNA	1.21994058	0.60284821	NA
ENSG0000026	RP5-951N9.2	lncRNA	1.21990478	0.60413425	NA
ENSG0000022	TMEM252-DT	lncRNA	1.21988451	0.6048648	NA

ENSG0000028	RP11-404C6.6	lncRNA	1.21986474	0.10415503	0.20802099
ENSG0000017	CTD-2527I21.	lncRNA	1.2197615	0.40061346	0.5538314
ENSG0000022	ASH1L-IT1	lncRNA	1.21905137	0.38465792	NA
ENSG0000013	CMPK2	protein_codir	1.2185828	0.001729	0.00879645
ENSG0000017	CNGB3	protein_codir	1.21820809	0.67372684	NA
ENSG0000028	RP11-241K3.5	lncRNA	1.2181868	0.13125342	0.24700003
ENSG0000001	CLCA4	protein_codir	1.21813258	0.67731208	NA
ENSG0000022	RP1-266L20.4	lncRNA	1.21810318	0.67872173	NA
ENSG0000028	CMB9-22P13.	lncRNA	1.21805269	0.68116569	NA
ENSG0000018	TMEM95	protein_codir	1.21803177	0.54409624	NA
ENSG0000013	EMILIN2	protein_codir	1.21801985	0.00097574	0.00562392
ENSG0000022	BCAR3-AS1	lncRNA	1.21801489	0.20269892	0.33984122
ENSG0000016	ANKRD33B	protein_codir	1.21797337	7.44E-06	0.00010024
ENSG0000012	HELB	protein_codir	1.21780171	3.40E-05	0.00035507
ENSG0000028	RP11-34C15.3	lncRNA	1.21751082	0.70901521	NA
ENSG0000028	AP001066.12	lncRNA	1.21751082	0.70901521	NA
ENSG0000018	AKAP14	protein_codir	1.21751082	0.70901521	NA
ENSG0000018	UBALD2	protein_codir	1.21722319	5.71E-09	2.19E-07
ENSG0000011	DLGAP3	protein_codir	1.21698752	0.00053467	0.00345437
ENSG0000012	ELF1	protein_codir	1.21685948	3.98E-07	8.26E-06
ENSG0000026	RP11-85G18.6	lncRNA	1.21650949	0.46019464	NA
ENSG0000019	FGF16	protein_codir	1.21614086	0.10116747	0.20355836
ENSG0000025	AC004069.2	lncRNA	1.21612209	0.08177425	0.17352526
ENSG0000027	LINC01126	lncRNA	1.21577061	0.00751389	0.02781954
ENSG0000027	RP11-496H1.2	lncRNA	1.21553901	0.074606	0.16233006
ENSG0000023	RP11-229P13.	lncRNA	1.21524036	0.60463368	NA
ENSG0000016	TCP11L2	protein_codir	1.21487425	5.92E-08	1.61E-06
ENSG0000014	DPT	protein_codir	1.21479808	0.00745122	0.02765051
ENSG0000023	AC159540.1	lncRNA	1.21441234	0.00507755	0.02051821
ENSG0000013	RNF122	protein_codir	1.21369883	0.01318366	0.04310692
ENSG0000011	TGIF2	protein_codir	1.21346879	1.07E-06	1.95E-05
ENSG0000025	MGAM2	protein_codir	1.2134267	0.04510082	0.11141085
ENSG0000026	RHEX	protein_codir	1.21267361	0.00138048	0.00738072
ENSG0000023	AF196970.3	lncRNA	1.2123511	0.59749304	NA
ENSG0000014	SLC5A12	protein_codir	1.21225678	0.33511291	0.48797019
ENSG0000019	H2AC13	protein_codir	1.21168624	0.21402214	0.3538848
ENSG0000016	BAALC	protein_codir	1.21147071	0.00022673	0.00170957
ENSG0000022	RP11-284F21.	lncRNA	1.21131913	0.01573594	0.04946248
ENSG0000010	CD33	protein_codir	1.21047866	0.00660568	0.02513917
ENSG0000028	AP001055.8	lncRNA	1.21036327	0.030063	0.08160589
ENSG0000026	RP11-524O1.4	lncRNA	1.21031139	0.34870143	0.50198757
ENSG0000024	TNFRSF6B	protein_codir	1.21027938	0.00188399	0.00936907
ENSG0000026	MYO15B	protein_codir	1.21015348	0.00146168	0.00773403
ENSG0000026	RPH3AL-AS1	lncRNA	1.20999254	0.01022945	0.03545892
ENSG0000012	HOXC11	protein_codir	1.20966969	0.2443279	0.38935707
ENSG0000027	RP11-348N5.9	lncRNA	1.20944886	0.03038464	0.08226039
ENSG0000022	RP11-344B5.2	lncRNA	1.20918169	0.00592651	0.0231186

ENSG0000026	RP11-95O2.1	lncRNA	1.20823587	0.6088668	NA
ENSG0000018	ODF3L1	protein_codir	1.2081923	0.02915966	0.07976526
ENSG0000022	RBM26-AS1	lncRNA	1.20810671	0.00067813	0.00419296
ENSG0000026	RP1-140C12.2	lncRNA	1.20798761	0.07899875	0.16911683
ENSG0000026	BSG-AS1	lncRNA	1.20721034	0.08811348	0.18339821
ENSG0000010	BST1	protein_codir	1.20695935	5.90E-05	0.00056528
ENSG0000026	DSCAS	lncRNA	1.20632457	0.30318927	0.45447127
ENSG0000022	MIR646HG	lncRNA	1.20586261	0.0004352	0.00291894
ENSG0000012	HIVEP3	protein_codir	1.20579862	2.17E-05	0.00024499
ENSG0000017	DNAH12	protein_codir	1.20549739	0.08223682	0.17414513
ENSG0000015	TEPP	protein_codir	1.20544852	0.09442373	0.19331283
ENSG0000016	SLC25A45	protein_codir	1.20518477	0.00089865	0.00525976
ENSG0000013	DUOX1	protein_codir	1.20464508	0.00680912	0.02575214
ENSG0000019	LRRTM3	protein_codir	1.20461348	0.26084296	0.40807038
ENSG0000020	TAP2	protein_codir	1.20412153	1.16E-06	2.09E-05
ENSG0000011	SH2B3	protein_codir	1.20403045	5.04E-06	7.16E-05
ENSG0000019	GRK6	protein_codir	1.20370643	2.60E-05	0.00028597
ENSG0000026	CTD-2231E14.	lncRNA	1.20266471	0.10322109	0.2066367
ENSG0000027	TBC1D3D	protein_codir	1.20231094	0.68175586	0.79068171
ENSG0000022	AC007362.3	lncRNA	1.20228559	0.46617381	NA
ENSG0000017	PCED1B	protein_codir	1.2022272	0.00099534	0.00571428
ENSG0000025	CTD-2240H23	lncRNA	1.20214351	0.68864842	NA
ENSG0000017	OR4F21	protein_codir	1.20206657	0.69150046	NA
ENSG0000028	CTD-2201E18.	lncRNA	1.20198589	0.46341846	NA
ENSG0000028	RP4-669P10.2	lncRNA	1.20173318	0.07298567	0.15985796
ENSG0000019	OR2L5	protein_codir	1.20152449	0.71266901	NA
ENSG0000022	NCBP2-AS1	lncRNA	1.20152449	0.71266901	NA
ENSG0000022	CCDC183-AS1	lncRNA	1.20146593	0.00049602	0.003248
ENSG0000024	CFHR1	protein_codir	1.2014654	0.06803142	0.15194294
ENSG0000013	SLC37A2	protein_codir	1.20133008	0.01196871	0.03999708
ENSG0000014	SYTL5	protein_codir	1.20102621	0.23002231	0.37238557
ENSG0000027	RP11-637O19	protein_codir	1.20061822	0.43803093	0.58924676
ENSG0000028	RP4-695O20.1	lncRNA	1.20045285	0.16624982	0.29406468
ENSG0000028	AC079584.3	lncRNA	1.20023217	0.04687823	0.11453133
ENSG0000022	LINC01771	lncRNA	1.20023048	0.27201894	0.42003484
ENSG0000013	CTSV	protein_codir	1.19998544	0.09410768	0.1927944
ENSG0000011	GRK7	protein_codir	1.19977998	0.06727327	0.15079432
ENSG0000018	PARVB	protein_codir	1.19932024	1.95E-05	0.00022365
ENSG0000023	CAVIN2-AS1	lncRNA	1.19908389	0.11222744	0.21991365
ENSG0000027	CTD-3194G12	lncRNA	1.19904113	0.43250145	NA
ENSG0000014	TENM4	protein_codir	1.19899252	0.00117273	0.00648853
ENSG0000022	RP11-151A6.4	lncRNA	1.19875001	0.15784197	0.28287019
ENSG0000010	RENBP	protein_codir	1.19866383	3.04E-05	0.00032409
ENSG0000025	RP11-529H2.2	lncRNA	1.19860517	0.46821749	NA
ENSG0000017	SPTBN2	protein_codir	1.19790813	0.00018208	0.00143158
ENSG0000028	LINC00921	lncRNA	1.19768593	0.00023365	0.00175225
ENSG0000026	AC005606.15	lncRNA	1.19744141	0.31005397	0.46180385

ENSG0000013	ARRB1	protein_codir	1.19731179	0.00010482	0.00090333
ENSG0000017	RCC2	protein_codir	1.19727819	2.03E-06	3.35E-05
ENSG0000016	B4GALNT2	protein_codir	1.19721146	0.5490605	NA
ENSG0000018	PDE2A	protein_codir	1.1971259	0.00287517	0.01304191
ENSG0000010	SLC5A1	protein_codir	1.19675282	0.41712282	NA
ENSG0000011	PRPH2	protein_codir	1.19643321	0.00031298	0.00223143
ENSG0000026	RP11-529K1.4	lncRNA	1.19629366	0.16238718	0.28873014
ENSG0000014	LRRTM2	protein_codir	1.19628091	0.01525548	0.04832761
ENSG0000020	NXPE2	protein_codir	1.19624219	0.11435777	0.22282621
ENSG0000017	ZCCHC12	protein_codir	1.19609599	0.03760281	0.09668495
ENSG0000017	MALT1	protein_codir	1.19547112	4.71E-07	9.54E-06
ENSG0000022	COL4A2-AS2	lncRNA	1.19540536	0.48753336	NA
ENSG0000023	RP1-90L6.3	lncRNA	1.19536498	0.18799023	0.32181133
ENSG0000017	RP11-567M16	lncRNA	1.19531763	0.49061572	NA
ENSG0000026	RP11-15H20.7	lncRNA	1.19503021	0.6033145	NA
ENSG0000017	ABCD2	protein_codir	1.19479194	0.00247896	0.01161474
ENSG0000022	RP11-168O16	lncRNA	1.19432177	0.34890979	0.50226134
ENSG0000026	RP11-408H20	lncRNA	1.1942781	0.59727614	NA
ENSG0000018	ANKRD45	protein_codir	1.19415243	0.02484989	0.07026049
ENSG0000016	FBXO41	protein_codir	1.1938802	0.00322858	0.01433195
ENSG0000018	MAPK15	protein_codir	1.19313684	0.06334075	0.14403685
ENSG0000025	RP11-546K22.	lncRNA	1.19295378	0.43170651	NA
ENSG0000011	ADGRG6	protein_codir	1.19267823	0.00116519	0.00645461
ENSG0000022	CCR5AS	lncRNA	1.1923282	0.04266434	0.10674776
ENSG0000028	VSIG10L2	protein_codir	1.1919429	0.08382335	0.17658462
ENSG0000025	LINC02148	lncRNA	1.19177479	0.46677387	NA
ENSG0000006	NGEF	protein_codir	1.1914391	0.04282707	0.10704153
ENSG0000028	CTC-490E21.1	lncRNA	1.19139592	0.53262304	NA
ENSG0000011	NRP2	protein_codir	1.19092939	0.00010075	0.00087369
ENSG0000025	KRTAP5-9	protein_codir	1.1908844	0.13786478	0.25578191
ENSG0000012	ADCY7	protein_codir	1.190684	0.00019165	0.0014907
ENSG0000010	NEIL3	protein_codir	1.19047875	0.05506326	0.12931276
ENSG0000010	DBP	protein_codir	1.19047826	0.00086004	0.00507572
ENSG0000016	GALR1	protein_codir	1.19035016	0.1743889	0.30442558
ENSG0000011	CFAP92	protein_codir	1.18988977	0.01144261	0.03869779
ENSG0000027	RP11-150D20	lncRNA	1.18945764	0.43232405	NA
ENSG0000022	AC002117.1	lncRNA	1.18920009	0.03370947	0.08903658
ENSG0000028	RP11-71L14.5	lncRNA	1.18878612	0.54846211	NA
ENSG0000026	RP11-309M7.	lncRNA	1.18854919	0.71563894	NA
ENSG0000028	RP11-99L6.1	lncRNA	1.18854919	0.71563894	NA
ENSG0000025	RP11-470M17	lncRNA	1.188549	0.71563898	NA
ENSG0000024	RP11-499P20.	lncRNA	1.18844792	0.03924161	0.10002338
ENSG0000025	ZFHX4-AS1	lncRNA	1.18840477	0.10683851	0.21202675
ENSG0000023	LINC01564	lncRNA	1.18743488	0.1649568	0.29222672
ENSG0000016	CRABP1	protein_codir	1.18740881	0.29515849	0.44555268
ENSG0000011	NCOA7	protein_codir	1.18710074	7.88E-06	0.00010534
ENSG0000011	LEPR	protein_codir	1.18705291	0.00325807	0.01444195

ENSG0000014	CTSK	protein_codir	1.18689865	0.00076574	0.0046239
ENSG0000025	AP000487.6	lncRNA	1.18685376	0.30034155	0.45132936
ENSG0000024	SEMA6A-AS1	lncRNA	1.18636056	0.03727828	0.09609202
ENSG0000018	CNTN2	protein_codir	1.18615948	0.05722134	0.13301241
ENSG0000015	FGF18	protein_codir	1.18576501	0.03336321	0.08827397
ENSG0000025	KDM2B-DT	lncRNA	1.18555233	0.06832728	0.15232864
ENSG0000023	LINC01765	lncRNA	1.18534867	0.37173903	0.52516147
ENSG0000017	NCKAP5	protein_codir	1.18529145	0.00010006	0.00086877
ENSG0000014	ZNF92	protein_codir	1.18429674	0.00069194	0.00426687
ENSG0000015	TSPAN7	protein_codir	1.18423206	0.00029843	0.00214152
ENSG0000011	PCDH17	protein_codir	1.18391256	0.00015285	0.00124427
ENSG0000011	SOD2	protein_codir	1.18377174	9.20E-05	0.00081005
ENSG0000028	RP11-526D8.1	lncRNA	1.18375597	0.16870845	0.29709937
ENSG0000012	ATP13A4	protein_codir	1.18360484	0.02929372	0.08000725
ENSG0000027	RP11-295M18	lncRNA	1.18236994	0.03302427	0.08762058
ENSG0000000	LAP3	protein_codir	1.18216802	7.57E-08	1.99E-06
ENSG0000014	PGGHG	protein_codir	1.18205056	0.00307662	0.0137792
ENSG0000017	RP11-404O13	lncRNA	1.1815336	0.07131463	0.15719952
ENSG0000011	TRPM6	protein_codir	1.18144623	0.00414088	0.01752545
ENSG0000012	ACVR1C	protein_codir	1.18144431	0.04508721	0.11138718
ENSG0000021	SIAH3	protein_codir	1.18101552	0.12766963	0.24190405
ENSG0000016	FRMPD4	protein_codir	1.18096024	0.11418628	0.22261771
ENSG0000027	PP2672	lncRNA	1.18014637	0.43832854	NA
ENSG0000018	SPDYE17	protein_codir	1.18010191	0.42019935	NA
ENSG0000013	TTYH3	protein_codir	1.17933659	0.00025862	0.00190791
ENSG0000014	MYH15	protein_codir	1.17924982	0.03061075	0.08272182
ENSG0000015	CENPH	protein_codir	1.17894767	2.78E-05	0.00030094
ENSG0000016	FEN1	protein_codir	1.1783911	1.77E-05	0.00020703
ENSG0000015	LY96	protein_codir	1.17782288	7.25E-05	0.00066851
ENSG0000025	CTD-2501M5.	lncRNA	1.17773007	0.7181186	NA
ENSG0000017	DLK2	protein_codir	1.17730678	0.08549307	0.17941357
ENSG0000000	JARID2	protein_codir	1.17705189	7.76E-07	1.47E-05
ENSG0000027	RP11-322E11.	lncRNA	1.17703283	0.21200164	0.35157095
ENSG0000018	RNF182	protein_codir	1.17682643	0.02332235	0.06678727
ENSG0000023	LINC01412	lncRNA	1.17645822	0.36487226	0.51880422
ENSG0000014	CBX8	protein_codir	1.17609996	0.00145735	0.00771409
ENSG0000014	SLC25A48	protein_codir	1.1760761	0.04495253	0.1111141
ENSG0000026	WDR7-OT1	lncRNA	1.17587642	0.67810653	0.7880724
ENSG0000012	CALML4	protein_codir	1.17587395	0.00014174	0.00116968
ENSG0000022	LINC01772	lncRNA	1.1758256	0.00917251	0.0325594
ENSG0000010	RASD1	protein_codir	1.17558511	0.04733395	0.11543031
ENSG0000011	KRT18	protein_codir	1.17548094	0.0272556	0.07548378
ENSG0000015	FCER1G	protein_codir	1.17545096	0.00092917	0.00540061
ENSG0000013	ENPEP	protein_codir	1.17521504	0.00165626	0.00852036
ENSG0000015	WNT9B	protein_codir	1.17507923	0.01958718	0.05835379
ENSG0000026	RP11-1260E13	lncRNA	1.17372844	0.53844924	NA
ENSG0000025	ANK3-DT	lncRNA	1.1736757	0.49399996	NA

ENSG0000018	SP140L	protein_codir	1.17355492	4.73E-05	0.00046727
ENSG0000023	C5orf58	protein_codir	1.17350181	0.54654182	NA
ENSG0000018	LINC00313	lncRNA	1.17277949	0.15420617	0.27801629
ENSG0000016	NEK8	protein_codir	1.17214446	0.00423285	0.01780364
ENSG0000004	FAM214A	protein_codir	1.1719848	1.62E-08	5.29E-07
ENSG0000022	LINC02575	lncRNA	1.17134214	0.05120419	0.1225316
ENSG0000010	GGA2	protein_codir	1.17133654	8.44E-06	0.00011195
ENSG0000023	AL132709.1	lncRNA	1.17129882	0.47605357	0.62425104
ENSG0000016	TOR2A	protein_codir	1.1708661	2.16E-05	0.00024482
ENSG0000024	TMEM158	protein_codir	1.17084909	0.01403512	0.04525978
ENSG0000025	RAB44	protein_codir	1.17079094	0.01024195	0.03548897
ENSG0000017	TRABD	protein_codir	1.17058359	6.65E-05	0.00062226
ENSG0000010	MMD	protein_codir	1.17044195	0.00063567	0.00397945
ENSG0000018	KCNE1	protein_codir	1.17041044	0.02323726	0.06661269
ENSG0000018	SLC22A10	protein_codir	1.17017899	0.54369445	NA
ENSG0000027	TBC1D3B	protein_codir	1.17007921	0.01622388	0.05065467
ENSG0000020	ANKRD28	protein_codir	1.16998379	0.00192163	0.00951172
ENSG0000017	FGA	protein_codir	1.16994303	0.68630738	NA
ENSG0000016	CDC25A	protein_codir	1.16991788	0.01439943	0.04616508
ENSG0000027	RP5-1186P10.	lncRNA	1.16973032	0.48091305	0.62853444
ENSG0000025	HOXC-AS1	lncRNA	1.16919456	0.1616363	0.28780074
ENSG0000017	PRR15	protein_codir	1.16917404	0.00909379	0.03233895
ENSG0000023	AC084809.3	lncRNA	1.16870011	0.2450159	0.39004837
ENSG0000001	IGF1	protein_codir	1.16810773	0.01312806	0.0429719
ENSG0000015	TMEM86A	protein_codir	1.16774636	0.01219234	0.04059955
ENSG0000018	ADAP2	protein_codir	1.16731958	0.00225585	0.01080773
ENSG0000010	APOL4	protein_codir	1.1672181	0.0031708	0.01412085
ENSG0000014	CMTM3	protein_codir	1.16719289	2.18E-06	3.54E-05
ENSG0000018	C11orf72	lncRNA	1.16716285	0.41970518	0.57220283
ENSG0000019	PRIM1	protein_codir	1.16676443	0.00130238	0.00706713
ENSG0000019	CYRIA	protein_codir	1.16622622	0.00032034	0.00227505
ENSG0000018	H2AW	protein_codir	1.16603609	0.00088556	0.00520078
ENSG0000017	LMO7DN	lncRNA	1.16547616	0.13854516	0.2568372
ENSG0000012	TMIGD3	protein_codir	1.16531988	0.00349737	0.01527232
ENSG0000005	TARBP1	protein_codir	1.16527627	0.00021397	0.00162895
ENSG0000028	RP1-120G22.1	protein_codir	1.16506943	0.29569852	0.44612406
ENSG0000025	RCCD1-AS1	lncRNA	1.16461725	0.5421292	NA
ENSG0000011	TBC1D30	protein_codir	1.16456359	4.79E-05	0.00047223
ENSG0000016	EML5	protein_codir	1.16426242	0.00018192	0.00143148
ENSG0000023	CDRT4	protein_codir	1.16330205	0.14141702	0.26083058
ENSG0000025	RP11-712L6.5	lncRNA	1.16315109	0.19386076	0.32928075
ENSG0000019	ZNF521	protein_codir	1.16240422	0.0001377	0.00114106
ENSG0000019	FCGR1B	protein_codir	1.16225949	0.01066209	0.03662771
ENSG0000013	IL18BP	protein_codir	1.16211827	3.99E-05	0.00040471
ENSG0000020	LINC01270	lncRNA	1.16165397	0.00385321	0.01650778
ENSG0000028	RP11-256E16.	lncRNA	1.16113388	0.20180163	0.33887165
ENSG0000016	CCDC191	protein_codir	1.16055835	5.09E-06	7.22E-05

ENSG0000028	RP11-247M1.	lncRNA	1.16031406	0.5010941	NA
ENSG0000016	TXLNB	protein_codir	1.1599056	0.00018157	0.00143043
ENSG0000022	WDR11-AS1	lncRNA	1.15965536	0.08799586	0.18318101
ENSG0000027	RP11-406H21	lncRNA	1.15915995	0.42891065	NA
ENSG0000022	SOX21-AS1	lncRNA	1.15906005	0.32831448	0.48069651
ENSG0000013	NFATC1	protein_codir	1.15856142	0.00015098	0.00123269
ENSG0000015	RNF144A	protein_codir	1.15828491	0.00024867	0.00184831
ENSG0000022	RP11-536C5.2	lncRNA	1.15804356	0.22061654	0.36137046
ENSG0000027	LINC02033	lncRNA	1.15796364	0.16645202	0.29430926
ENSG0000022	LINC01204	lncRNA	1.15628906	0.51031294	NA
ENSG0000025	LINC02241	lncRNA	1.15614453	0.39291597	NA
ENSG0000011	TRPV4	protein_codir	1.15606857	0.00435895	0.01821635
ENSG0000021	HBE1	protein_codir	1.15593378	0.42428195	0.57629025
ENSG0000012	HOXD1	protein_codir	1.1555706	1.03E-05	0.00013153
ENSG0000015	ENKUR	protein_codir	1.15547263	0.06037676	0.1386552
ENSG0000016	SFMBT1	protein_codir	1.15524812	8.17E-10	4.28E-08
ENSG0000000	ABCC8	protein_codir	1.15497591	0.15013703	0.27247419
ENSG0000014	LCN2	protein_codir	1.1544924	0.13958695	0.25842157
ENSG0000014	SLC18B1	protein_codir	1.15437425	0.00061281	0.00386078
ENSG0000024	LINC02891	lncRNA	1.15427486	0.28341859	0.43298974
ENSG0000028	RP11-290O12	lncRNA	1.15387014	0.01909607	0.057246
ENSG0000010	CDH20	protein_codir	1.15372594	0.09788494	0.19857377
ENSG0000027	TM4SF19-DYN	protein_codir	1.15323037	0.38565083	0.53880702
ENSG0000026	RP11-769O8.3	lncRNA	1.15315186	0.03489649	0.09133337
ENSG0000014	TLE3	protein_codir	1.15306301	1.03E-10	7.32E-09
ENSG0000018	SOX2	protein_codir	1.15303899	0.06278924	0.14308901
ENSG0000027	RP3-468O1.6	lncRNA	1.15266323	0.15275231	0.27602347
ENSG0000010	SIGLEC8	protein_codir	1.15252167	0.06092551	0.13960201
ENSG0000028	RP11-401F2.5	lncRNA	1.15233367	0.1150376	0.22387853
ENSG0000027	RP1-102E24.1	lncRNA	1.15218619	0.03472736	0.09102008
ENSG0000024	C4orf48	protein_codir	1.152002	0.00239774	0.01131674
ENSG0000019	CCDC154	protein_codir	1.15169191	0.02684594	0.07461108
ENSG0000026	RP11-936I5.1	lncRNA	1.15151402	0.03168398	0.08483096
ENSG0000018	FAM166A	protein_codir	1.15096627	0.34447792	0.49756721
ENSG0000016	RNASE2	protein_codir	1.15077604	0.0335495	0.08866492
ENSG0000025	LINC00923	lncRNA	1.15064584	0.06121443	0.1400897
ENSG0000013	ULBP2	protein_codir	1.15051193	0.03744225	0.09637978
ENSG0000011	F3	protein_codir	1.15020341	0.00910374	0.03236519
ENSG0000028	XXbac-BPG34	lncRNA	1.15014898	0.43325784	NA
ENSG0000025	C1orf210	protein_codir	1.15009445	0.41679188	0.56949616
ENSG0000011	SLC27A6	protein_codir	1.14967202	0.21102051	0.35038995
ENSG0000028	LL0XNC01-30I	protein_codir	1.1491317	0.15647479	0.28098565
ENSG0000011	PLPPR4	protein_codir	1.14889778	0.00236954	0.01121814
ENSG0000022	RP11-195B3.1	lncRNA	1.14861886	0.46773964	0.61723302
ENSG0000014	FRMPD3	protein_codir	1.14841476	0.01363741	0.04426669
ENSG0000013	RNF138	protein_codir	1.14822626	1.98E-10	1.29E-08
ENSG0000011	HBEGF	protein_codir	1.14807361	0.00908828	0.03233522

ENSG0000021	GOLGA6B	protein_codir	1.14805266	0.61623781	NA
ENSG0000027	RP11-699C17.	lncRNA	1.14753193	0.44628047	NA
ENSG0000023	RHOA-IT1	lncRNA	1.14743842	0.0274588	0.07591917
ENSG0000023	LIX1L-AS1	lncRNA	1.14721124	0.26279113	0.41015076
ENSG0000023	AC009133.12	lncRNA	1.14694044	0.01562956	0.04919526
ENSG0000018	OPCML	protein_codir	1.14668356	0.05706127	0.13276324
ENSG0000018	SLC25A18	protein_codir	1.14576219	0.05923338	0.13656676
ENSG0000012	FFAR2	protein_codir	1.14563824	0.04776202	0.1161972
ENSG0000013	TM6SF1	protein_codir	1.14505407	0.00325222	0.01441834
ENSG0000025	RP11-433J8.1	lncRNA	1.14429322	0.14208624	0.26168046
ENSG0000016	CTSB	protein_codir	1.14429086	0.00156171	0.00813244
ENSG0000014	PLIN2	protein_codir	1.1441506	0.00469718	0.01934053
ENSG0000015	GAL3ST2	protein_codir	1.14361573	0.21017215	0.34933831
ENSG0000022	AC007182.6	lncRNA	1.14321899	0.32508699	0.47739806
ENSG0000011	GABRG2	protein_codir	1.14319513	0.68411085	NA
ENSG0000010	CYP2D6	protein_codir	1.14311695	0.03124197	0.08397266
ENSG0000017	NKPD1	protein_codir	1.14301439	0.10404445	0.20785397
ENSG0000012	ADCY4	protein_codir	1.14296745	0.0003048	0.00217872
ENSG0000010	RLN1	protein_codir	1.1429489	0.27074644	0.41868512
ENSG0000027	RP11-96O20.5	lncRNA	1.14289459	0.22833234	0.37064586
ENSG0000028	RP11-4B12.1	lncRNA	1.14274689	0.15096408	0.27352584
ENSG0000022	ZGLP1	protein_codir	1.1427131	0.01657028	0.05141795
ENSG0000015	TEX9	protein_codir	1.14253233	0.00483409	0.01976281
ENSG0000021	RP11-174G6.1	lncRNA	1.14247751	0.48758902	NA
ENSG0000018	F8	protein_codir	1.14244181	0.01785293	0.0543328
ENSG0000006	RASGRP2	protein_codir	1.1422708	0.00424862	0.0178556
ENSG0000028	XXbac-BPGBP	lncRNA	1.14184755	0.39796907	0.55097272
ENSG0000026	CTC-523E23.5	lncRNA	1.1411349	0.20436734	0.34197419
ENSG0000023	SEMA3F-AS1	lncRNA	1.14092978	0.16887578	0.29735746
ENSG0000012	ABCC4	protein_codir	1.14033517	0.00018725	0.00146314
ENSG0000014	CPNE9	protein_codir	1.14025857	0.16233873	0.28869092
ENSG0000000	ADAM22	protein_codir	1.13998216	0.00408621	0.01733754
ENSG0000027	RP11-116D17	lncRNA	1.13994468	0.23674655	0.3807243
ENSG0000018	SIGIRR	protein_codir	1.13983374	0.00015643	0.00126701
ENSG0000017	SLCO2A1	protein_codir	1.13973988	0.00029563	0.00212806
ENSG0000023	AC017116.11	lncRNA	1.1393971	0.11680097	0.22643663
ENSG0000013	BMP2K	protein_codir	1.13911828	3.17E-06	4.84E-05
ENSG0000016	MTMR14	protein_codir	1.13879594	7.61E-07	1.45E-05
ENSG0000018	LCN12	protein_codir	1.13851623	0.06080129	0.13937518
ENSG0000016	NPIPB12	protein_codir	1.13798771	0.00781833	0.02868904
ENSG0000010	SAMHD1	protein_codir	1.13794907	2.99E-05	0.00032039
ENSG0000010	RASIP1	protein_codir	1.13762506	0.00010756	0.00092451
ENSG0000024	RP11-422N16	lncRNA	1.13743864	0.45614278	NA
ENSG0000027	RP11-102N12	lncRNA	1.13736854	0.01068449	0.03669553
ENSG0000018	FIGLA	protein_codir	1.13729246	0.54793128	NA
ENSG0000017	MYRIP	protein_codir	1.13724231	0.00241795	0.01139656
ENSG0000010	LIMK1	protein_codir	1.13677247	1.60E-05	0.00018961

ENSG0000027	RP1-30M3.5	lncRNA	1.13602089	0.00090884	0.00530702
ENSG0000025	TRIL	protein_codir	1.1359408	0.00450743	0.01869578
ENSG0000026	SNHG25	lncRNA	1.13569914	0.48966311	NA
ENSG0000018	SAGE1	protein_codir	1.13550932	0.40986219	0.56285277
ENSG0000024	RP4-598P13.1	lncRNA	1.13505146	0.17876447	0.30977317
ENSG0000022	LINC01422	lncRNA	1.13488907	0.0050451	0.02042594
ENSG0000017	TDRD12	protein_codir	1.13443437	0.23056438	0.37308324
ENSG0000012	NR4A1	protein_codir	1.13425858	0.04536465	0.11190245
ENSG0000016	STOX1	protein_codir	1.13388199	0.01828004	0.05532486
ENSG0000020	HCG27	lncRNA	1.13387705	0.04130427	0.1040518
ENSG0000028	BISPR	lncRNA	1.13353256	0.00056218	0.00359667
ENSG0000013	ZGRF1	protein_codir	1.13341119	0.00124299	0.00679962
ENSG0000009	NLRP1	protein_codir	1.13328306	0.00021331	0.00162531
ENSG0000010	SYT17	protein_codir	1.13321803	0.00448951	0.01864579
ENSG0000027	RP11-350G8.9	lncRNA	1.13276955	0.09675667	0.1968198
ENSG0000025	LINC02055	lncRNA	1.131735	0.15504693	0.27909256
ENSG0000012	RAMP3	protein_codir	1.13165892	0.00020148	0.0015531
ENSG0000026	RP11-24N18.1	lncRNA	1.13131458	0.4008346	0.55396806
ENSG0000025	CTXND1	protein_codir	1.13124042	0.10172946	0.20442108
ENSG0000016	PCDH19	protein_codir	1.13116194	0.02118994	0.06198541
ENSG0000019	SLC29A3	protein_codir	1.13105597	0.0007374	0.00448737
ENSG0000015	SH2D6	protein_codir	1.13078847	0.22755943	0.36980415
ENSG0000024	RP11-268G12	lncRNA	1.13069458	0.6890643	NA
ENSG0000015	C16orf89	protein_codir	1.13060808	0.03882262	0.09915726
ENSG0000025	RP11-14I17.2	lncRNA	1.13017809	0.55438472	NA
ENSG0000017	GRK2	protein_codir	1.12991401	8.84E-06	0.00011599
ENSG0000026	RP11-1035H1	protein_codir	1.12983098	0.25781695	0.40475782
ENSG0000015	CCSAP	protein_codir	1.12947965	2.07E-08	6.44E-07
ENSG0000025	LINC01605	lncRNA	1.12937902	0.08119477	0.17262717
ENSG0000026	NARF-IT1	lncRNA	1.12937792	0.03789004	0.09725146
ENSG0000016	PDPN	protein_codir	1.12865501	0.01953528	0.05823685
ENSG0000022	PGAM4	protein_codir	1.12836434	0.24633878	0.39160603
ENSG0000025	LINC01619	lncRNA	1.12815355	0.02563071	0.07200853
ENSG0000011	PROC	protein_codir	1.12782592	0.08772032	0.18269019
ENSG0000017	NRXN1	protein_codir	1.1275112	0.05090328	0.1221536
ENSG0000023	AP001626.2	lncRNA	1.12738424	0.50662949	NA
ENSG0000008	NID2	protein_codir	1.12738262	0.0007792	0.0046837
ENSG0000018	SHISA6	protein_codir	1.12706151	0.00480285	0.01966129
ENSG0000018	FAM78B	protein_codir	1.12702805	0.00104917	0.00594786
ENSG0000025	RP11-1078H9	lncRNA	1.1268127	0.51707679	NA
ENSG0000007	N4BP2	protein_codir	1.12647957	1.96E-06	3.26E-05
ENSG0000027	RP11-996F15.	lncRNA	1.12612428	0.15881523	0.28413503
ENSG0000018	MYLPF	protein_codir	1.12607655	0.15645995	0.28098565
ENSG0000028	CYP3A7-CYP3.	protein_codir	1.12604149	0.45935084	NA
ENSG0000018	RAB42	protein_codir	1.12587784	0.01079339	0.03700055
ENSG0000027	RP11-165D6.1	lncRNA	1.12580461	0.42184208	0.57382638
ENSG0000027	LINC02139	lncRNA	1.12541859	0.2316854	0.3743945

ENSG0000025	RASA2-IT1	lncRNA	1.12514642	0.27589011	0.42404479
ENSG0000024	RP11-77I22.2	lncRNA	1.12491537	0.3423917	0.49563968
ENSG0000016	SLC37A1	protein_codir	1.12471245	7.27E-09	2.68E-07
ENSG0000025	LINC01956	lncRNA	1.1247034	0.48199359	0.62949985
ENSG0000027	ATP6V0D1-DT	lncRNA	1.1246722	0.01729053	0.05304505
ENSG0000010	PRKD2	protein_codir	1.12360384	7.29E-05	0.00067154
ENSG0000017	PARP14	protein_codir	1.12327695	0.00018245	0.00143408
ENSG0000027	CCL16	protein_codir	1.12323225	0.1701543	0.29898897
ENSG0000027	AC004067.5	lncRNA	1.12302245	0.02524746	0.07117114
ENSG0000023	LINC01637	lncRNA	1.1228081	0.01317222	0.0430895
ENSG0000026	NAPA-AS1	lncRNA	1.12280339	0.00387979	0.01659847
ENSG0000015	CLGN	protein_codir	1.12274592	0.07550453	0.16374333
ENSG0000005	CDK17	protein_codir	1.12217377	7.92E-06	0.00010582
ENSG0000012	ENOX1	protein_codir	1.12217181	0.00184203	0.00920036
ENSG0000010	ZNF85	protein_codir	1.12203029	0.00195569	0.00965264
ENSG0000025	RP11-779O18	lncRNA	1.12196718	0.14337516	0.26345698
ENSG0000018	CHEK2	protein_codir	1.12191374	0.00029758	0.00213792
ENSG0000012	SEC14L1	protein_codir	1.1215095	2.27E-06	3.68E-05
ENSG0000013	NAPSA	protein_codir	1.12126777	0.07358263	0.16080008
ENSG0000028	RP5-1042K10.	protein_codir	1.12103233	0.06726424	0.15079432
ENSG0000024	RP11-146D12	lncRNA	1.12074381	0.03890325	0.09932469
ENSG0000012	FOXA2	protein_codir	1.1207209	0.58148085	0.71284959
ENSG0000028	RP11-314E10.	lncRNA	1.12025984	0.52073852	0.66339669
ENSG0000026	PIK3R5-DT	lncRNA	1.11951108	0.0771591	0.16623513
ENSG0000022	AC008781.7	lncRNA	1.119242	0.45494921	NA
ENSG0000026	LA16c-OS12.2	lncRNA	1.11914081	0.19454395	0.33002234
ENSG0000003	TLL1	protein_codir	1.11898885	0.00091213	0.00531948
ENSG0000010	DMXL2	protein_codir	1.11894514	0.00106698	0.00602904
ENSG0000028	RP11-707O23	lncRNA	1.11874925	0.60432539	0.7309278
ENSG0000028	RP11-236P2.2	lncRNA	1.11868013	0.00076783	0.00463449
ENSG0000028	PCBP2-OT1	lncRNA	1.1184022	0.08122448	0.17267705
ENSG0000016	EMCN	protein_codir	1.11822059	0.00404499	0.01718547
ENSG0000015	L3MBTL4	protein_codir	1.11784151	8.32E-05	0.00074818
ENSG0000021	FAM24B	protein_codir	1.11760058	0.01191025	0.03985957
ENSG0000027	CTD-2587H24	lncRNA	1.11755955	0.00271455	0.01247105
ENSG0000022	TSBP1-AS1	lncRNA	1.11721772	0.00806875	0.02942422
ENSG0000014	DIPK2B	protein_codir	1.11700859	0.00024652	0.00183524
ENSG0000025	RP11-661A12	lncRNA	1.11688368	0.11265105	0.22043373
ENSG0000012	KIF25	protein_codir	1.11666634	0.15428736	0.2781259
ENSG0000016	SGPL1	protein_codir	1.11665121	3.69E-05	0.00038006
ENSG0000026	RP11-196G11	lncRNA	1.11598523	0.04139924	0.10424351
ENSG0000025	CTC-573N18.1	lncRNA	1.1157832	0.4644462	NA
ENSG0000012	ARL4A	protein_codir	1.11556753	1.26E-05	0.00015615
ENSG0000018	ADARB2	protein_codir	1.11542174	0.24190415	0.38674419
ENSG0000026	ZNF45-AS1	lncRNA	1.11537605	0.23807828	0.38219832
ENSG0000015	TRIM74	protein_codir	1.11516938	0.04994363	0.12041229
ENSG0000017	RNF213	protein_codir	1.11506714	6.00E-05	0.0005727

ENSG0000013	DGKB	protein_codir	1.11491358	0.03026493	0.08202506
ENSG0000008	SLC15A1	protein_codir	1.11460477	0.36197702	0.51588262
ENSG0000017	GPR34	protein_codir	1.11450789	0.0195689	0.05831638
ENSG0000016	CDS1	protein_codir	1.11431866	0.0014092	0.00750523
ENSG0000027	CTA-217C2.2	lncRNA	1.11429072	0.09393597	0.19251211
ENSG0000027	RP11-141M3.	lncRNA	1.11427664	0.03464783	0.090872
ENSG0000013	GAS2L3	protein_codir	1.11354383	0.00202495	0.009913
ENSG0000014	LRRC46	protein_codir	1.11295642	0.02873985	0.07884333
ENSG0000019	L1CAM	protein_codir	1.11289761	0.05032884	0.12117728
ENSG0000013	ZBTB46	protein_codir	1.11280957	4.77E-07	9.62E-06
ENSG0000023	STXBP5-AS1	lncRNA	1.11268887	0.0009274	0.00539258
ENSG0000007	CACNG4	protein_codir	1.1126108	0.24698251	0.39221838
ENSG0000026	LA16c-366D1.	lncRNA	1.11247221	0.14935934	0.27158542
ENSG0000006	SLC9A7	protein_codir	1.11223972	0.00363564	0.01574172
ENSG0000018	C5orf52	protein_codir	1.11217685	0.55110642	NA
ENSG0000022	AF230666.2	lncRNA	1.11204298	0.56033806	NA
ENSG0000022	GRASLND	lncRNA	1.11192537	0.04566587	0.11239192
ENSG0000015	PPP1R15B	protein_codir	1.11174071	3.18E-05	0.00033589
ENSG0000027	RP11-35O15.2	lncRNA	1.11145256	0.3788399	0.53228193
ENSG0000025	DIO3OS	lncRNA	1.11143094	0.01441373	0.04620507
ENSG0000025	RP11-87C12.2	protein_codir	1.11126548	0.13316086	0.24967097
ENSG0000017	SMPDL3A	protein_codir	1.11118881	0.00104285	0.00591934
ENSG0000013	KLF4	protein_codir	1.11102978	0.0052416	0.02104566
ENSG0000010	CLDN15	protein_codir	1.11083972	0.00205074	0.01001567
ENSG0000015	STC1	protein_codir	1.11072065	0.07470595	0.16245795
ENSG0000021	DENND1B	protein_codir	1.11060969	1.38E-05	0.00016901
ENSG0000028	RP11-407H3.1	lncRNA	1.10914345	0.46448267	NA
ENSG0000027	CTC-251D13.1	lncRNA	1.10897991	0.11467584	0.22327272
ENSG0000011	CHKA	protein_codir	1.10857919	8.21E-05	0.00073994
ENSG0000012	ARHGAP40	protein_codir	1.10848196	0.43378297	0.5852257
ENSG0000017	NCBP2L	protein_codir	1.10839897	0.40777134	NA
ENSG0000013	RHPN2	protein_codir	1.1081906	0.00381829	0.01638867
ENSG0000017	TMTC2	protein_codir	1.10782463	1.15E-06	2.07E-05
ENSG0000023	COA6-AS1	lncRNA	1.10729706	0.01485767	0.0473703
ENSG0000012	AVPR2	protein_codir	1.10720336	0.01964562	0.05846662
ENSG0000027	RP11-713D19	lncRNA	1.10707825	0.63158477	NA
ENSG0000018	DIPK1C	protein_codir	1.10624793	0.03141022	0.08426909
ENSG0000028	RP11-728E14.	lncRNA	1.10623449	0.26934049	0.41718172
ENSG0000025	RP11-346D14	lncRNA	1.1059409	0.52522096	NA
ENSG0000020	NOTCH4	protein_codir	1.10583479	0.0010779	0.00607584
ENSG0000021	COL28A1	protein_codir	1.10568154	0.09773131	0.19832039
ENSG0000023	AC002480.4	lncRNA	1.10547146	0.04006384	0.10165012
ENSG0000021	ZNF726	protein_codir	1.10534242	0.00521894	0.02098516
ENSG0000020	KLRC3	protein_codir	1.10493075	0.07411354	0.16155126
ENSG0000016	ATG16L2	protein_codir	1.10481136	0.00187701	0.00934109
ENSG0000007	NEBL	protein_codir	1.10455979	0.00046041	0.00305878
ENSG0000027	SMIM34B	protein_codir	1.10439569	0.27714116	0.42554141

ENSG0000028	XXbac-BPG30	lncRNA	1.10413538	0.57997178	0.71178941
ENSG0000016	PI16	protein_codir	1.1039461	0.06944094	0.15407767
ENSG0000027	RP11-737O24	lncRNA	1.10382979	0.06054644	0.13890804
ENSG0000026	RAB5C-AS1	lncRNA	1.1036074	0.51798435	NA
ENSG0000010	SOX10	protein_codir	1.10342309	0.07767373	0.16704419
ENSG0000023	LINC02631	lncRNA	1.10325536	0.12444433	0.23743799
ENSG0000025	CLLU1	lncRNA	1.10263847	0.39787097	0.55089213
ENSG0000014	STON2	protein_codir	1.10173964	0.00294901	0.0133004
ENSG0000023	STARD13-IT1	lncRNA	1.10149799	0.53930714	0.67849001
ENSG0000018	DLEU7	protein_codir	1.10145862	0.01963598	0.05844872
ENSG0000024	FER1L5	protein_codir	1.10141206	0.21014965	0.34933831
ENSG0000025	CTD-2026D20	lncRNA	1.10108501	0.57626679	NA
ENSG0000028	CTC-325H20.9	lncRNA	1.10088489	0.4279081	0.57972819
ENSG0000027	RP1-257I20.14	lncRNA	1.10024799	0.13439383	0.25131737
ENSG0000010	NDRG1	protein_codir	1.10003041	0.00015332	0.00124699
ENSG0000016	FRAT1	protein_codir	1.09972873	0.00181349	0.0091037
ENSG0000016	IFI16	protein_codir	1.09971954	1.09E-06	1.98E-05
ENSG0000013	CAPN11	protein_codir	1.09967177	0.00410069	0.017383
ENSG0000028	RP11-187D20	lncRNA	1.09936371	0.55864269	NA
ENSG0000010	CDKN3	protein_codir	1.09932419	0.02020046	0.05974664
ENSG0000013	PRICKLE1	protein_codir	1.0992886	2.37E-05	0.00026398
ENSG0000012	HOXD9	protein_codir	1.09910279	0.02921278	0.079871
ENSG0000020	H3C14	protein_codir	1.09899054	0.26234361	0.40979135
ENSG0000020	H3C15	protein_codir	1.09899054	0.26234361	0.40979135
ENSG0000016	MS4A6E	protein_codir	1.09890839	0.38229572	0.53578343
ENSG0000013	GFPT2	protein_codir	1.09860314	0.02172081	0.06316368
ENSG0000014	LBR	protein_codir	1.09826948	9.74E-08	2.46E-06
ENSG0000003	FLT4	protein_codir	1.09820993	0.00820523	0.02981168
ENSG0000019	MYO5A	protein_codir	1.09795665	4.55E-05	0.00045156
ENSG0000016	LBHD1	protein_codir	1.09785903	0.00153899	0.00803532
ENSG0000022	LINC01150	lncRNA	1.09783064	0.00797955	0.02914693
ENSG0000013	USHBP1	protein_codir	1.09754186	0.00546395	0.02174871
ENSG0000010	PON3	protein_codir	1.09750967	0.08801722	0.18321168
ENSG0000010	OIP5	protein_codir	1.09749936	0.03252578	0.08653074
ENSG0000026	RP11-552C15.	lncRNA	1.09726318	0.4903621	0.63714547
ENSG0000014	CCDC150	protein_codir	1.09718123	0.03335868	0.08827043
ENSG0000022	AC068580.5	lncRNA	1.09714164	0.40490066	0.55784032
ENSG0000026	LINC01238	lncRNA	1.09710456	0.085817	0.17987431
ENSG0000028	RP11-23B15.5	lncRNA	1.09708706	0.10140405	0.20389135
ENSG0000010	CRISPLD2	protein_codir	1.09702308	0.02120557	0.06200484
ENSG0000013	SHISAL1	protein_codir	1.09643642	0.00857759	0.03081995
ENSG0000027	RP11-417L19.	lncRNA	1.09617816	0.14329717	0.26337347
ENSG0000023	TAPBP	protein_codir	1.09600147	2.24E-06	3.63E-05
ENSG0000022	LINC00954	lncRNA	1.09591704	0.07863408	0.16849289
ENSG0000016	ASPG	protein_codir	1.09582804	0.16855426	0.29697836
ENSG0000010	TGM5	protein_codir	1.09519662	0.12753254	0.24171061
ENSG0000028	SCO2	protein_codir	1.09475147	0.00017338	0.00137922

ENSG0000016	ADORA1	protein_codir	1.09469524	0.03562818	0.09279991
ENSG0000025	RP11-102L12.	lncRNA	1.09445518	0.05510229	0.12936041
ENSG0000017	C1QB	protein_codir	1.09423986	0.00703814	0.02643696
ENSG0000016	HEXD	protein_codir	1.0940805	0.00087209	0.00513367
ENSG0000011	IL1RL1	protein_codir	1.09388603	0.07660228	0.16539258
ENSG0000028	RP11-134F2.8	protein_codir	1.09356865	0.00180523	0.0090738
ENSG0000025	RP11-702L15.	lncRNA	1.09321405	0.46001991	NA
ENSG0000016	DHRS13	protein_codir	1.0931841	7.86E-06	0.00010514
ENSG0000016	RNF149	protein_codir	1.09260414	2.99E-07	6.43E-06
ENSG0000027	RP11-728K20.	lncRNA	1.09246674	0.5164737	NA
ENSG0000015	SLC5A10	protein_codir	1.09239727	0.16262933	0.28907795
ENSG0000016	MATN1	protein_codir	1.09193722	0.10739356	0.21271886
ENSG0000022	LINC02798	lncRNA	1.09191573	0.00044862	0.00299226
ENSG0000013	NHSL1	protein_codir	1.09184795	6.49E-06	8.87E-05
ENSG0000012	CD68	protein_codir	1.09159423	0.00153047	0.00800142
ENSG0000017	CCDC144A	protein_codir	1.09118767	0.02735231	0.07569088
ENSG0000028	RP11-134L10.	lncRNA	1.08955769	0.00025838	0.00190709
ENSG0000007	PABPC1	protein_codir	1.0894356	2.40E-10	1.52E-08
ENSG0000016	CHAF1A	protein_codir	1.08943213	6.01E-08	1.63E-06
ENSG0000023	RP11-239E10.	lncRNA	1.08904085	0.57807432	NA
ENSG0000027	RP11-302M6.	protein_codir	1.08867541	0.73838864	NA
ENSG0000000	WDR54	protein_codir	1.08860697	2.13E-06	3.47E-05
ENSG0000016	LOXHD1	protein_codir	1.08847313	0.03258772	0.08665378
ENSG0000016	FMNL3	protein_codir	1.08822559	4.14E-05	0.0004173
ENSG0000016	NOS3	protein_codir	1.08796089	0.00239957	0.0113215
ENSG0000017	RASGRP4	protein_codir	1.08742013	0.00410093	0.017383
ENSG0000028	RP11-263K14.	lncRNA	1.08699701	0.39198764	NA
ENSG0000028	PIGY-DT	lncRNA	1.08692965	0.43930289	0.59032528
ENSG0000017	GATM	protein_codir	1.08687761	7.20E-05	0.00066444
ENSG0000010	STIM2	protein_codir	1.08668941	9.52E-11	6.81E-09
ENSG0000013	GPAT3	protein_codir	1.08659727	0.00343333	0.01504499
ENSG0000013	ALOX5AP	protein_codir	1.08651875	0.00281432	0.01282487
ENSG0000016	CHP2	protein_codir	1.08629004	0.38065904	0.53399374
ENSG0000018	TEDC1	protein_codir	1.08621005	0.01182754	0.03967412
ENSG0000012	NFE2	protein_codir	1.08601919	0.10902752	0.21529565
ENSG0000023	RNF148	protein_codir	1.08533991	0.06155475	0.14077522
ENSG0000006	TIE1	protein_codir	1.08497159	0.00229557	0.01095811
ENSG0000019	PIM3	protein_codir	1.0843275	2.62E-05	0.00028788
ENSG0000023	LINC01134	lncRNA	1.08413809	0.09084775	0.18752073
ENSG0000017	FUT1	protein_codir	1.08404642	0.00041181	0.00279737
ENSG0000016	FABP5	protein_codir	1.083955	0.01421388	0.0457401
ENSG0000013	CUZD1	protein_codir	1.08385777	0.04933887	0.11930831
ENSG0000028	ASAP1-IT2	lncRNA	1.08384994	0.05898601	0.13612915
ENSG0000025	RP11-138B4.1	lncRNA	1.08366447	0.17582175	0.30606815
ENSG0000018	RTN4RL2	protein_codir	1.08345358	0.00160928	0.00833301
ENSG0000021	CKLF	protein_codir	1.08343598	3.37E-05	0.00035281
ENSG0000025	RP11-770J1.4	lncRNA	1.08341183	0.69813049	NA

ENSG0000015	ABCA6	protein_codir	1.08321414	0.00374482	0.01611593
ENSG0000020	SYT15	protein_codir	1.08314857	0.01186922	0.0397608
ENSG0000013	RTP4	protein_codir	1.08313176	0.00892792	0.03185908
ENSG0000028	CTD-2206N4.6	lncRNA	1.08281682	0.05172345	0.12344263
ENSG0000027	SYNRG	protein_codir	1.08271313	1.63E-06	2.79E-05
ENSG0000012	HOXD11	protein_codir	1.08245806	0.27730645	0.42574787
ENSG0000000	USH1C	protein_codir	1.08191913	0.2872765	0.43680578
ENSG0000026	CTD-2561B21	lncRNA	1.08169578	0.70465277	NA
ENSG0000022	RP11-574F21.	lncRNA	1.08167782	0.13502605	0.25210705
ENSG0000025	RP11-280K24.	lncRNA	1.08166294	0.5012826	0.6461091
ENSG0000015	UBXN11	protein_codir	1.08149149	0.00220642	0.01061513
ENSG0000027	RP11-227G15	lncRNA	1.08136908	0.08199791	0.17379455
ENSG0000025	RP11-61A14.1	lncRNA	1.0812752	0.29421903	0.4445404
ENSG0000010	HAS3	protein_codir	1.08125348	1.66E-06	2.82E-05
ENSG0000025	RP11-996F15.	lncRNA	1.08115805	0.01499752	0.04773902
ENSG0000027	RP11-248M19	lncRNA	1.08047955	0.32528565	0.477591
ENSG0000010	ITGB8	protein_codir	1.08023332	0.02111432	0.06182311
ENSG0000013	TCN1	protein_codir	1.07946774	0.25922465	0.40629808
ENSG0000025	LINC02361	lncRNA	1.07941898	0.00147848	0.00780049
ENSG0000010	DENND3	protein_codir	1.07900169	0.00013431	0.00111599
ENSG0000025	PTX4	protein_codir	1.0789569	0.1880688	0.32189762
ENSG0000010	ELF4	protein_codir	1.07888951	1.80E-08	5.77E-07
ENSG0000012	PTGFR	protein_codir	1.07883561	0.01142652	0.03865859
ENSG0000000	TRAPPC6A	protein_codir	1.07856341	2.16E-06	3.52E-05
ENSG0000026	LA16c-425C2.	lncRNA	1.07849179	0.70476576	NA
ENSG0000016	ADRB2	protein_codir	1.07835492	8.01E-06	0.00010683
ENSG0000016	PLB1	protein_codir	1.07835201	0.00043112	0.00289507
ENSG0000025	SHANK3	protein_codir	1.07815282	0.00033574	0.00236737
ENSG0000012	TMOD2	protein_codir	1.07780521	3.43E-05	0.00035719
ENSG0000025	SIGLEC12	protein_codir	1.07770085	0.1199167	0.23087266
ENSG0000013	CNDP2	protein_codir	1.07750478	2.38E-05	0.00026485
ENSG0000001	STAB1	protein_codir	1.07674293	0.00048063	0.00316979
ENSG0000017	PLEKHD1	protein_codir	1.07664013	0.04555902	0.11225158
ENSG0000005	GALC	protein_codir	1.07656531	2.09E-05	0.00023805
ENSG0000018	TSHZ2	protein_codir	1.07640902	0.00234085	0.01112235
ENSG0000016	PROCA1	protein_codir	1.07632883	0.00522888	0.02100985
ENSG0000023	LINC01424	lncRNA	1.07601813	0.49891265	0.64452608
ENSG0000026	STAG1-DT	lncRNA	1.07591157	0.44932697	0.60017117
ENSG0000023	LINC01284	lncRNA	1.07561479	0.0876909	0.18268493
ENSG0000011	RASGRF2	protein_codir	1.07515851	0.0006005	0.00379805
ENSG0000002	ADGRA2	protein_codir	1.07514163	6.56E-05	0.00061551
ENSG0000011	MROH9	protein_codir	1.07460277	0.30196071	0.45322001
ENSG0000019	ZNF257	protein_codir	1.07434099	0.01099291	0.037554
ENSG0000022	AP001628.6	lncRNA	1.07416579	0.64525796	NA
ENSG0000026	PAQR5-DT	lncRNA	1.07397548	0.63874671	NA
ENSG0000027	RP11-436D23	lncRNA	1.07388906	0.63825798	NA
ENSG0000027	RP11-345N11	lncRNA	1.07386479	0.64316499	NA

ENSG0000017	OR5AN1	protein_codir	1.07385268	0.58540862	NA
ENSG0000017	ANKK1	protein_codir	1.07372889	0.08582138	0.17987431
ENSG0000023	RP4-537K23.4	lncRNA	1.07360811	0.63667486	NA
ENSG0000026	S1PR2	protein_codir	1.07332751	3.91E-05	0.00039738
ENSG0000026	CH17-351M24	lncRNA	1.07328893	0.63921015	NA
ENSG0000027	RP11-61L19.2	lncRNA	1.07311097	0.34296986	0.49612341
ENSG0000020	C1orf53	protein_codir	1.07310397	0.02271297	0.06548559
ENSG0000016	TTC21A	protein_codir	1.07304953	0.00604705	0.02347098
ENSG0000023	RP11-433J20.	lncRNA	1.0729015	0.21408336	0.35390394
ENSG0000022	LINC01389	lncRNA	1.07273864	0.52876775	NA
ENSG0000026	LA16c-390E6.	lncRNA	1.0727219	0.64688729	NA
ENSG0000010	CDKL1	protein_codir	1.07270483	5.62E-05	0.00054244
ENSG0000023	RP11-488L18.	lncRNA	1.07269722	0.64171954	NA
ENSG0000015	ROBO3	protein_codir	1.07238908	0.00541802	0.02160324
ENSG0000017	NMUR1	protein_codir	1.07226033	0.08849583	0.18394453
ENSG0000025	RP11-517I3.1	lncRNA	1.07224738	0.11784662	0.22790391
ENSG0000017	CD164L2	protein_codir	1.07216845	0.63475394	NA
ENSG0000005	PARP12	protein_codir	1.07211627	0.00073319	0.0044674
ENSG0000025	CKLF-CMTM1	protein_codir	1.07208503	0.00767336	0.02827508
ENSG0000013	HAPLN2	protein_codir	1.07124085	0.20330826	0.3405734
ENSG0000027	RP11-443B20.	lncRNA	1.07112594	0.03134822	0.08413723
ENSG0000027	RP11-244H3.4	protein_codir	1.06980947	0.00213477	0.01033158
ENSG0000017	EFCAB5	protein_codir	1.06954836	0.123166	0.23568275
ENSG0000000	FMO3	protein_codir	1.06906951	0.01714819	0.05273722
ENSG0000006	DGKA	protein_codir	1.0689518	0.0033631	0.01480299
ENSG0000024	LY6E-DT	lncRNA	1.06875533	0.01544823	0.04876675
ENSG0000023	CTD-2201G3.1	lncRNA	1.068323	0.15470482	0.27867313
ENSG0000025	RP11-293M10	lncRNA	1.0683104	0.00048801	0.0032077
ENSG0000014	SNCA	protein_codir	1.06795538	0.00066807	0.00414609
ENSG0000025	TBX5-AS1	lncRNA	1.06795532	0.12714958	0.24123309
ENSG0000021	ACKR1	protein_codir	1.06731026	0.01485005	0.04735742
ENSG0000027	RP11-128N14	lncRNA	1.06730793	0.52449163	NA
ENSG0000018	PLGLB1	protein_codir	1.0667684	0.05665116	0.13210951
ENSG0000025	RP11-22P4.2	lncRNA	1.06670475	0.40322014	0.55630421
ENSG0000011	DNAJC4	protein_codir	1.06662185	3.01E-05	0.00032179
ENSG0000028	RP11-951I11.!	lncRNA	1.06628206	0.18048391	0.3120284
ENSG0000012	TNFRSF10B	protein_codir	1.06606344	1.66E-06	2.82E-05
ENSG0000010	CLEC11A	protein_codir	1.06580796	0.00469418	0.01933392
ENSG0000027	RP1-20C7.6	lncRNA	1.06569214	0.161016	0.28693864
ENSG0000023	ADAMTSL4-AS	lncRNA	1.06539845	0.03075057	0.08295867
ENSG0000022	TNKS2-AS1	lncRNA	1.06475535	0.20995593	0.34912599
ENSG0000015	DDX4	protein_codir	1.06453565	0.65725842	NA
ENSG0000023	SGO1-AS1	lncRNA	1.06420185	0.17268144	0.30231425
ENSG0000014	RASGEF1C	protein_codir	1.06393332	0.14352198	0.26360402
ENSG0000017	PTPN2	protein_codir	1.06379951	7.14E-08	1.90E-06
ENSG0000026	DIS3L-AS1	lncRNA	1.06378283	0.58386067	NA
ENSG0000015	TXNDC11	protein_codir	1.06376227	0.00013119	0.00109437

ENSG0000027	RP11-408A13	lncRNA	1.0636466	0.06836218	0.15239296
ENSG0000009	TPSD1	protein_codir	1.06352881	0.19850304	0.33479812
ENSG0000026	AC007192.4	protein_codir	1.0634116	0.62255716	0.74535857
ENSG0000027	RP11-466F5.1	lncRNA	1.06325911	0.58121355	NA
ENSG0000025	CTC-342M10.	lncRNA	1.06313261	0.58057798	NA
ENSG0000017	PGBD5	protein_codir	1.06283244	0.00967281	0.03390825
ENSG0000027	RP11-797D24	lncRNA	1.06279654	0.30826697	0.45994527
ENSG0000014	IGSF6	protein_codir	1.06277049	0.03445355	0.0905716
ENSG0000027	RP11-290H9.5	lncRNA	1.06276565	0.57874252	NA
ENSG0000004	EPHA3	protein_codir	1.06263135	0.00664816	0.02527839
ENSG0000026	RP11-524F11.	lncRNA	1.06234926	0.41140444	0.56442433
ENSG0000011	POLR3G	protein_codir	1.06230965	0.00027236	0.00199167
ENSG0000016	OSR2	protein_codir	1.06215848	0.00477761	0.01957826
ENSG0000026	MRC1	protein_codir	1.062113	0.01333234	0.04347058
ENSG0000018	AIFM3	protein_codir	1.06191393	0.02485674	0.07027055
ENSG0000023	DYRK3-AS1	lncRNA	1.06172786	0.18488509	0.31800006
ENSG0000005	SLC4A8	protein_codir	1.06133206	0.02784068	0.07681146
ENSG0000005	LAMC2	protein_codir	1.06109097	0.00807577	0.02944202
ENSG0000014	PLEKHA6	protein_codir	1.06087178	0.02606057	0.07292735
ENSG0000024	MIATNB	lncRNA	1.06068627	0.00220865	0.01062399
ENSG0000015	FGD5	protein_codir	1.06057597	4.06E-06	5.97E-05
ENSG0000016	SFXN1	protein_codir	1.06024449	6.35E-05	0.00059839
ENSG0000019	OXCT2	protein_codir	1.0602144	0.00073432	0.00447233
ENSG0000018	USP18	protein_codir	1.06020276	0.00116446	0.00645185
ENSG0000020	ADAMTSL4-AS	lncRNA	1.05999948	0.00831328	0.03008948
ENSG0000028	RP11-454P7.5	lncRNA	1.05992906	0.49948549	NA
ENSG0000018	FLRT2	protein_codir	1.05984915	0.00039197	0.0026922
ENSG0000023	LINC01664	lncRNA	1.05939445	0.20507202	0.34292482
ENSG0000005	PTPRN	protein_codir	1.05901433	0.13769994	0.25557909
ENSG0000010	MAP2K6	protein_codir	1.05897009	0.00846894	0.03051294
ENSG0000026	LIF-AS2	lncRNA	1.05887705	0.46770201	0.61723302
ENSG0000016	ADCY1	protein_codir	1.05886947	0.02770197	0.0765052
ENSG0000014	ESPNL	protein_codir	1.05854211	0.06559728	0.14798244
ENSG0000028	RP11-120L14.	lncRNA	1.05826775	0.43288734	NA
ENSG0000026	CTD-2525I3.2	lncRNA	1.05812307	0.35754322	0.51104036
ENSG0000011	SEMA5A	protein_codir	1.05761604	0.00058254	0.00370306
ENSG0000019	SCOC-AS1	lncRNA	1.0574513	0.04185588	0.1051973
ENSG0000017	EMX2	protein_codir	1.05705316	0.1373995	0.25514146
ENSG0000013	ERP27	protein_codir	1.05702488	0.00057898	0.00368332
ENSG0000013	TSGA10	protein_codir	1.05696433	0.00261522	0.01210732
ENSG0000022	RP11-211N8.2	lncRNA	1.0566905	0.11384682	0.22214406
ENSG0000026	RP11-315D16	protein_codir	1.05641642	1.52E-05	0.00018221
ENSG0000016	TMEM88	protein_codir	1.05635461	0.00419999	0.01770846
ENSG0000026	RP11-343C2.9	protein_codir	1.05593367	0.51193494	0.65561474
ENSG0000018	SLC47A2	protein_codir	1.05583488	0.53838734	0.67798267
ENSG0000019	H3C12	protein_codir	1.05576552	0.55309317	0.68952532
ENSG0000012	NRN1	protein_codir	1.05554257	0.00307143	0.01376039

ENSG0000028	RP5-892C22.1	lncRNA	1.05538026	0.13265092	0.24901913
ENSG0000018	SFTPA2	protein_codir	1.05532045	0.11191961	0.21946293
ENSG0000022	AC144450.1	lncRNA	1.05501085	0.65476519	NA
ENSG0000004	NEDD4L	protein_codir	1.05414208	2.30E-05	0.00025795
ENSG0000014	GALNT1	protein_codir	1.05401099	0.00027816	0.00202876
ENSG0000028	RP11-468B6.4	lncRNA	1.05362249	0.06580817	0.14836516
ENSG0000005	FSTL4	protein_codir	1.05308569	0.35401944	0.50761134
ENSG0000028	RP11-641J8.4	protein_codir	1.05293249	0.51434978	0.65757111
ENSG0000026	LINC02591	lncRNA	1.05279834	0.56391625	0.69829111
ENSG0000023	AF011889.2	lncRNA	1.05272853	0.1696927	0.29835051
ENSG0000025	SENCR	lncRNA	1.05244279	0.00019787	0.00153168
ENSG0000019	ARHGAP11A	protein_codir	1.05216018	0.00177962	0.00898268
ENSG0000025	RP1-16A9.1	lncRNA	1.05206587	0.21695176	0.35723189
ENSG0000027	TBC1D3L	protein_codir	1.05175133	0.01560086	0.04912816
ENSG0000022	AL022341.3	lncRNA	1.05162461	0.23645383	0.3803867
ENSG0000017	ZNF404	protein_codir	1.05121466	0.00354122	0.01542235
ENSG0000025	RP11-525K10.	lncRNA	1.0510198	0.26484487	0.41238158
ENSG0000018	NKAIN2	protein_codir	1.05099575	0.00150349	0.00789623
ENSG0000015	CLEC4F	protein_codir	1.05091706	0.10486581	0.20906551
ENSG0000007	GSDMB	protein_codir	1.05084386	0.02769794	0.07650171
ENSG0000013	LPAR6	protein_codir	1.05054173	0.00054681	0.0035205
ENSG0000025	LINC00520	lncRNA	1.0505139	0.3620524	0.51593682
ENSG0000026	SPON1	protein_codir	1.05028619	0.02247555	0.06493667
ENSG0000028	RP11-548N1.2	lncRNA	1.05024655	0.40881759	0.56181542
ENSG0000025	LINC02515	lncRNA	1.05009415	0.36806834	0.5215765
ENSG0000018	B4GALNT4	protein_codir	1.05001577	0.12967459	0.24466236
ENSG0000012	GNA13	protein_codir	1.04996379	5.03E-06	7.16E-05
ENSG0000000	CEACAM7	protein_codir	1.04990546	0.65012707	NA
ENSG0000024	RP11-421F16.	lncRNA	1.04966486	0.59196443	NA
ENSG0000027	CCL14	protein_codir	1.04927629	0.00818417	0.02974691
ENSG0000011	CLIC5	protein_codir	1.04904191	0.00156695	0.00815512
ENSG0000025	RP11-273B20.	lncRNA	1.04875336	0.08954226	0.18552046
ENSG0000025	LINC02352	lncRNA	1.04859865	0.02269487	0.06545185
ENSG0000017	JMJD1C	protein_codir	1.048549	1.50E-05	0.00018028
ENSG0000026	RP11-304L19.	lncRNA	1.04797643	0.35705592	0.51065916
ENSG0000012	KIF18A	protein_codir	1.04783675	0.03150547	0.0844754
ENSG0000027	TMEM191B	protein_codir	1.04778783	0.10193182	0.20469369
ENSG0000025	RP11-150O12	lncRNA	1.04762651	0.2062838	0.3443882
ENSG0000003	TMSB10	protein_codir	1.04740388	2.33E-05	0.00026063
ENSG0000025	RP11-307C19.	lncRNA	1.046849	0.28541506	0.43493319
ENSG0000026	CTC-326K19.6	protein_codir	1.04681381	0.49936334	0.64484189
ENSG0000009	TREM2	protein_codir	1.04673618	0.0947684	0.19378516
ENSG0000010	MAP3K8	protein_codir	1.04668583	0.00031123	0.0022201
ENSG0000016	GAB3	protein_codir	1.04640119	0.00020413	0.00157134
ENSG0000015	DCK	protein_codir	1.04622633	9.99E-05	0.00086782
ENSG0000013	DNAJB1	protein_codir	1.04621707	0.02587672	0.07250102
ENSG0000022	RP11-114M1.	lncRNA	1.04593378	0.59743291	NA

ENSG0000014	NR6A1	protein_codir	1.0458555	0.0028605	0.01298816
ENSG0000018	SNORC	protein_codir	1.04557872	0.01632818	0.05081512
ENSG0000014	FATE1	protein_codir	1.04556759	0.27417405	0.42222956
ENSG0000028	GIMAP1-GIM/	protein_codir	1.04550145	0.05513458	0.12942522
ENSG0000017	MOB3A	protein_codir	1.0448602	9.64E-07	1.78E-05
ENSG0000010	DKK1	protein_codir	1.04485724	0.04517436	0.11157255
ENSG0000013	GLUL	protein_codir	1.04373299	4.50E-05	0.00044719
ENSG0000010	IGDCC4	protein_codir	1.04372993	0.00432352	0.0180933
ENSG0000027	RP3-508I15.2	lncRNA	1.04363259	0.11666822	0.22626437
ENSG0000027	RP11-346C4.3	lncRNA	1.04349229	0.21868413	0.35918505
ENSG0000018	SPATA32	protein_codir	1.04337549	0.47788404	0.62581941
ENSG0000028	CTD-2201E18.	lncRNA	1.04324827	0.01806998	0.05485743
ENSG0000026	CTB-96E2.7	lncRNA	1.04320773	0.59042223	NA
ENSG0000019	PAPSS2	protein_codir	1.0428984	7.70E-06	0.00010321
ENSG0000027	U2AF1L5	protein_codir	1.04286064	0.00535561	0.02140166
ENSG0000026	RP11-154H12	lncRNA	1.04284532	0.58757306	NA
ENSG0000023	HOXB-AS2	lncRNA	1.04248939	0.34083706	0.49393565
ENSG0000028	F8A2	protein_codir	1.042279	0.16479705	0.29205931
ENSG0000027	F8A3	protein_codir	1.042279	0.16479705	0.29205931
ENSG0000025	SIGLEC10-AS1	lncRNA	1.04222009	0.46342862	0.61337303
ENSG0000027	RP3-402G11.2	lncRNA	1.0418808	0.06289072	0.14326118
ENSG0000011	RGS2	protein_codir	1.04182396	0.00573597	0.02254233
ENSG0000022	LINC02068	lncRNA	1.04172657	0.27878775	0.42757053
ENSG0000025	CTD-2012K14	lncRNA	1.04144328	0.1725786	0.30220129
ENSG0000012	WNK4	protein_codir	1.04118049	0.31529211	0.467137
ENSG0000003	FUT8	protein_codir	1.04080641	1.16E-05	0.00014546
ENSG0000017	ZNF556	protein_codir	1.04077827	0.41341434	0.56647835
ENSG0000010	RP1	protein_codir	1.04041991	0.19627835	0.33216284
ENSG0000020	SAMD9	protein_codir	1.04011695	0.00234634	0.01114268
ENSG0000010	RHOXF1	protein_codir	1.04009042	0.17591423	0.30614019
ENSG0000016	PLEKHA2	protein_codir	1.03970552	1.19E-06	2.13E-05
ENSG0000021	CXorf49B	protein_codir	1.03928122	0.48721383	NA
ENSG0000025	GPR162	protein_codir	1.03909843	0.00081681	0.00486843
ENSG0000006	RIMBP2	protein_codir	1.03869795	0.05674329	0.13227599
ENSG0000002	CCDC28A	protein_codir	1.0385073	4.79E-05	0.00047245
ENSG0000012	NT5C3A	protein_codir	1.03835365	1.69E-12	2.40E-10
ENSG0000019	ZNF724	protein_codir	1.03820786	0.07499028	0.16288388
ENSG0000026	CTC-429P9.4	protein_codir	1.03799557	0.27331011	0.42125141
ENSG0000016	ASRGL1	protein_codir	1.0379847	0.00056349	0.00360102
ENSG0000016	CYGB	protein_codir	1.03796338	0.00603746	0.02344036
ENSG0000028	RP11-562I5.4	lncRNA	1.03770482	0.28688057	0.43641576
ENSG0000026	MMP25-AS1	lncRNA	1.03740415	0.00966937	0.03390478
ENSG0000011	GLP1R	protein_codir	1.03680014	0.18603721	0.31931409
ENSG0000014	TNFRSF11A	protein_codir	1.03670286	0.04770918	0.11610958
ENSG0000026	CTD-2006C1.1	protein_codir	1.03669097	0.04327725	0.10786119
ENSG0000016	SLC9C2	protein_codir	1.03657256	0.65442688	NA
ENSG0000016	HESX1	protein_codir	1.03591594	0.04457087	0.11032869

ENSG0000022	RP3-340B19.3	lncRNA	1.03564042	0.67343713	0.78467625
ENSG0000007	MCM2	protein_codir	1.03489046	0.00075637	0.00457831
ENSG0000025	RP11-762I7.5	protein_codir	1.03468523	0.27456429	0.42264776
ENSG0000018	SAXO2	protein_codir	1.0345792	0.11069173	0.21769478
ENSG0000013	AOC2	protein_codir	1.03436111	0.0264586	0.07374233
ENSG0000026	RP11-483P21.	lncRNA	1.03388889	0.00739925	0.02750571
ENSG0000014	PSTPIP1	protein_codir	1.03378331	0.00479812	0.01965122
ENSG0000013	MMRN1	protein_codir	1.0332505	0.07733002	0.16649947
ENSG0000015	GRIP1	protein_codir	1.03310362	0.03467824	0.09093449
ENSG0000015	CHD1	protein_codir	1.03273861	6.26E-05	0.00059211
ENSG0000028	RP11-900F13.	lncRNA	1.03256755	0.3843028	0.53756674
ENSG0000018	SPATA13	protein_codir	1.03249554	3.54E-05	0.0003669
ENSG0000028	MICB-DT	lncRNA	1.03221888	0.04764494	0.11599412
ENSG0000011	STMN1	protein_codir	1.03199317	0.00013278	0.00110528
ENSG0000016	NTNG1	protein_codir	1.03149588	0.00829508	0.03003149
ENSG0000028	RP11-139D23	lncRNA	1.0314325	0.65515982	NA
ENSG0000010	HDAC10	protein_codir	1.03140242	0.00117751	0.0065111
ENSG0000009	TBC1D2	protein_codir	1.03140152	0.00059089	0.00374663
ENSG0000024	RP11-373E16.	lncRNA	1.03133162	0.65352747	NA
ENSG0000019	RAMP2-AS1	lncRNA	1.03074522	0.00336293	0.01480299
ENSG0000011	STAT1	protein_codir	1.03056133	0.00054482	0.00351169
ENSG0000027	RP11-434H6.6	lncRNA	1.03056084	0.02063835	0.06076445
ENSG0000026	RP11-96D1.10	lncRNA	1.03054435	0.11650285	0.22604722
ENSG0000017	LINC00174	lncRNA	1.03036845	0.01428162	0.0458779
ENSG0000025	RP11-585P4.5	lncRNA	1.03034343	0.01243666	0.04118713
ENSG0000018	HOXC9	protein_codir	1.03017739	0.10674757	0.21191093
ENSG0000018	KPNA2	protein_codir	1.03017098	3.37E-06	5.11E-05
ENSG0000004	DSG2	protein_codir	1.03016085	0.01785389	0.0543328
ENSG0000010	TSPAN14	protein_codir	1.03011891	5.13E-06	7.27E-05
ENSG0000015	ABI3BP	protein_codir	1.02947054	0.00171831	0.00875639
ENSG0000027	CTD-2302E22.	lncRNA	1.02939585	0.04060102	0.10266412
ENSG0000018	EPHB3	protein_codir	1.02916562	3.99E-05	0.00040496
ENSG0000015	SFXN2	protein_codir	1.02913584	0.00264666	0.01222216
ENSG0000021	ZNF90	protein_codir	1.02910355	0.00536472	0.02142786
ENSG0000011	SLC22A18	protein_codir	1.02906441	0.00950869	0.03344328
ENSG0000027	RP11-888D10	lncRNA	1.02891915	0.00078163	0.00469317
ENSG0000013	IQCA1	protein_codir	1.02886681	0.03878001	0.09907684
ENSG0000015	ZNF276	protein_codir	1.02849133	0.00024781	0.00184242
ENSG0000024	POU5F2	protein_codir	1.02849056	0.08579234	0.1798546
ENSG0000010	NOVA2	protein_codir	1.02843652	0.00084667	0.00501407
ENSG0000027	RP11-114O18	lncRNA	1.02836436	0.39968445	0.55298726
ENSG0000011	PCDH12	protein_codir	1.02827593	0.0006439	0.00402455
ENSG0000022	RP11-217B7.2	lncRNA	1.02805889	0.25337619	0.39969447
ENSG0000028	RP11-259O2.4	lncRNA	1.02756861	0.65298862	NA
ENSG0000011	ADGRD1	protein_codir	1.02702459	0.00935119	0.03304923
ENSG0000010	LHX6	protein_codir	1.02686978	0.02287417	0.06581088
ENSG0000010	DLX4	protein_codir	1.02671491	0.15035924	0.27276994

ENSG0000028	RP11-706O15	lncRNA	1.02662662	0.00299351	0.01348129
ENSG0000022	RP11-134G8.7	lncRNA	1.02652314	0.00659907	0.02511937
ENSG0000026	RP11-324I22.3	lncRNA	1.02640029	0.05114554	0.12248676
ENSG0000015	CNTNAP3B	protein_codir	1.02609618	0.01434625	0.04603199
ENSG0000023	RP3-468B3.2	lncRNA	1.0260688	0.65237928	NA
ENSG0000023	LINC02006	lncRNA	1.02606875	0.65537058	NA
ENSG0000023	LINC01445	lncRNA	1.02606871	0.65786773	NA
ENSG0000016	KRT82	protein_codir	1.02606867	0.65988786	NA
ENSG0000028	RP11-529K1.5	lncRNA	1.02606867	0.66004737	NA
ENSG0000028	RP11-811I7.3	lncRNA	1.02606867	0.66011757	NA
ENSG0000022	RP11-563D10	lncRNA	1.02606866	0.66058975	NA
ENSG0000027	RP11-392E22.	lncRNA	1.02606865	0.66068276	NA
ENSG0000028	THRIL	lncRNA	1.02606864	0.66121772	NA
ENSG0000022	LINC00392	lncRNA	1.02606863	0.66181895	NA
ENSG0000026	SLC14A2-AS1	lncRNA	1.0260686	0.66335821	NA
ENSG0000027	CTD-2014B16	lncRNA	1.0260686	0.66365471	NA
ENSG0000028	TEX13D	protein_codir	1.02606858	0.66454843	NA
ENSG0000028	RP11-447M4.	lncRNA	1.02606855	0.66588305	NA
ENSG0000028	RP11-325K19.	lncRNA	1.02606854	0.66653281	NA
ENSG0000026	LINC01413	lncRNA	1.02606849	0.66923445	NA
ENSG0000028	RP11-546M8.	lncRNA	1.02606724	0.71785194	NA
ENSG0000028	RP3-488M23.	lncRNA	1.02606721	0.71858317	NA
ENSG0000020	C9orf92	lncRNA	1.02606719	0.71930583	NA
ENSG0000028	LINC00628	lncRNA	1.02606718	0.71971748	NA
ENSG0000026	RP11-299P2.1	lncRNA	1.02606717	0.71998546	NA
ENSG0000023	CAMTA1-IT1	lncRNA	1.02606715	0.72052694	NA
ENSG0000027	CTD-2049O4.2	lncRNA	1.02606715	0.7206252	NA
ENSG0000024	RP11-625I7.1	lncRNA	1.02606715	0.72066997	NA
ENSG0000024	CTB-35F21.1	lncRNA	1.02606714	0.7209439	NA
ENSG0000025	GYPB	protein_codir	1.02606713	0.72106327	NA
ENSG0000024	LINC02517	lncRNA	1.0260671	0.72194843	NA
ENSG0000027	AGBL1	protein_codir	1.02606708	0.72252423	NA
ENSG0000024	LINC00973	lncRNA	1.02606706	0.72313463	NA
ENSG0000027	RP11-407G23	lncRNA	1.02606705	0.72343173	NA
ENSG0000016	PRAP1	protein_codir	1.02606703	0.72400006	NA
ENSG0000026	GS1-204I12.4	lncRNA	1.02606702	0.72419709	NA
ENSG0000020	CTD-2008L17.	lncRNA	1.02606702	0.72440756	NA
ENSG0000026	RP11-264M12	lncRNA	1.026067	0.72482843	NA
ENSG0000024	RP11-231E6.1	lncRNA	1.026067	0.72483467	NA
ENSG0000017	SLC17A8	protein_codir	1.026067	0.72485037	NA
ENSG0000018	FSD2	protein_codir	1.02606699	0.72506785	NA
ENSG0000017	TRIML2	protein_codir	1.02606699	0.72530933	NA
ENSG0000022	RP11-553K8.2	lncRNA	1.02606694	0.72670139	NA
ENSG0000020	FAM83A-AS1	lncRNA	1.02606693	0.72682286	NA
ENSG0000026	RP11-793A3.2	lncRNA	1.02606691	0.72731275	NA
ENSG0000028	RP11-389G6.5	lncRNA	1.02606691	0.72748746	NA
ENSG0000016	TCP10L2	protein_codir	1.0260669	0.72761235	NA

ENSG0000027	RP11-529E10.	lncRNA	1.0260669	0.72762079	NA
ENSG0000021	C3orf56	protein_codir	1.02606688	0.72817704	NA
ENSG0000024	CTD-2503O16	lncRNA	1.02606688	0.72831938	NA
ENSG0000025	LINC01965	lncRNA	1.02606687	0.72857124	NA
ENSG0000016	TMEM82	protein_codir	1.02606687	0.72858767	NA
ENSG0000026	RP11-106M3.	lncRNA	1.02606686	0.72884338	NA
ENSG0000025	CTD-2531D15	lncRNA	1.02606686	0.72893031	NA
ENSG0000018	OR52K2	protein_codir	1.02606685	0.72919212	NA
ENSG0000017	WDR87	protein_codir	1.02606683	0.72951397	NA
ENSG0000024	LINC01259	lncRNA	1.02606681	0.73019339	NA
ENSG0000022	CTA-796E4.4	lncRNA	1.0260668	0.73041975	NA
ENSG0000022	LINC01980	lncRNA	1.0260668	0.73045559	NA
ENSG0000028	RP11-517P14.	lncRNA	1.0260668	0.7304896	NA
ENSG0000027	RP11-356M6.	lncRNA	1.0260668	0.73054052	NA
ENSG0000017	AP000679.2	lncRNA	1.0260668	0.73054052	NA
ENSG0000026	BBS7-DT	lncRNA	1.02606679	0.73062469	NA
ENSG0000022	LINC01185	lncRNA	1.02606679	0.73062826	NA
ENSG0000018	CYP4F2	protein_codir	1.02606679	0.73063211	NA
ENSG0000023	RP11-183I6.2	lncRNA	1.02606679	0.73071534	NA
ENSG0000017	DYDC1	protein_codir	1.02606678	0.73084401	NA
ENSG0000013	STOML3	protein_codir	1.02606678	0.73086	NA
ENSG0000024	LINC02513	lncRNA	1.02606678	0.73095599	NA
ENSG0000022	AC011997.1	lncRNA	1.02606678	0.73099722	NA
ENSG0000023	AC074389.5	lncRNA	1.02606677	0.73113005	NA
ENSG0000019	OR14L1P	protein_codir	1.02606676	0.73149302	NA
ENSG0000026	LINC00868	lncRNA	1.02606675	0.73185161	NA
ENSG0000026	CTB-129P6.11	lncRNA	1.02606675	0.73185161	NA
ENSG0000025	CTD-2530H12	lncRNA	1.02606673	0.73218583	NA
ENSG0000025	SLC1A2-AS1	lncRNA	1.02606673	0.73229153	NA
ENSG0000016	VCX3A	protein_codir	1.02606673	0.73236951	NA
ENSG0000022	LINC02865	lncRNA	1.02606672	0.73267234	NA
ENSG0000020	IFITM5	protein_codir	1.02606672	0.73267234	NA
ENSG0000027	RP4-616B8.6	lncRNA	1.02606669	0.73322485	NA
ENSG0000026	RP11-440L14.	lncRNA	1.02606668	0.73356471	NA
ENSG0000026	AC005307.4	lncRNA	1.02606668	0.73369059	NA
ENSG0000027	RP11-285E23.	lncRNA	1.02606667	0.73378075	NA
ENSG0000024	LINC01019	lncRNA	1.02606666	0.73411098	NA
ENSG0000022	AC093690.1	lncRNA	1.02606666	0.73412895	NA
ENSG0000028	RP11-527D7.4	lncRNA	1.02606664	0.73457054	NA
ENSG0000028	RP11-90P5.10	lncRNA	1.02606664	0.73468007	NA
ENSG0000019	SLC2A7	protein_codir	1.02606664	0.73469849	NA
ENSG0000023	LINC01507	lncRNA	1.02606664	0.73469849	NA
ENSG0000028	XXyac-YR14ID	lncRNA	1.02606661	0.73526916	NA
ENSG0000022	RP3-395M20.	lncRNA	1.02606661	0.73544538	NA
ENSG0000024	GLYCTK-AS1	lncRNA	1.0260666	0.7355671	NA
ENSG0000022	GS1-519E5.1	lncRNA	1.02606659	0.73580863	NA
ENSG0000025	RP11-74K11.2	lncRNA	1.02606651	0.73792585	NA

ENSG0000028	RP11-511B7.2	lncRNA	1.02606644	0.73969599	NA
ENSG0000023	ERVH48-1	protein_codir	1.02606642	0.73997831	NA
ENSG0000016	CYP3A4	protein_codir	1.02606637	0.7412182	NA
ENSG0000026	RP11-1260E13	lncRNA	1.02606633	0.74220613	NA
ENSG0000027	RP11-168F9.2	lncRNA	1.02606583	0.75317102	NA
ENSG0000021	LACTBL1	protein_codir	1.02606583	0.75317102	NA
ENSG0000018	CATSPER4	protein_codir	1.02606583	0.75317102	NA
ENSG0000023	PEF1-AS1	lncRNA	1.02606583	0.75317102	NA
ENSG0000020	C1orf141	protein_codir	1.02606583	0.75317102	NA
ENSG0000028	RP11-366L18.	lncRNA	1.02606583	0.75317102	NA
ENSG0000022	RP11-86H7.6	lncRNA	1.02606583	0.75317102	NA
ENSG0000022	LINC01708	lncRNA	1.02606583	0.75317102	NA
ENSG0000022	RP11-495P10.	lncRNA	1.02606583	0.75317102	NA
ENSG0000023	LINC01732	lncRNA	1.02606583	0.75317102	NA
ENSG0000022	LINC01939	lncRNA	1.02606583	0.75317102	NA
ENSG0000022	AC016722.3	lncRNA	1.02606583	0.75317102	NA
ENSG0000022	AC018462.2	lncRNA	1.02606583	0.75317102	NA
ENSG0000017	REG3A	protein_codir	1.02606583	0.75317102	NA
ENSG0000028	RP11-173H9.2	lncRNA	1.02606583	0.75317102	NA
ENSG0000023	AC007556.3	lncRNA	1.02606583	0.75317102	NA
ENSG0000022	RAPGEF4-AS1	lncRNA	1.02606583	0.75317102	NA
ENSG0000016	TGM4	protein_codir	1.02606583	0.75317102	NA
ENSG0000023	ITIH4-AS1	lncRNA	1.02606583	0.75317102	NA
ENSG0000028	ERC2-IT1	lncRNA	1.02606583	0.75317102	NA
ENSG0000024	LINC02050	lncRNA	1.02606583	0.75317102	NA
ENSG0000020	CD200R1L	protein_codir	1.02606583	0.75317102	NA
ENSG0000023	LINC01471	lncRNA	1.02606583	0.75317102	NA
ENSG0000022	LINC01209	lncRNA	1.02606583	0.75317102	NA
ENSG0000028	RP11-1263C18	lncRNA	1.02606583	0.75317102	NA
ENSG0000024	STX18-IT1	lncRNA	1.02606583	0.75317102	NA
ENSG0000028	RP11-416B16.	lncRNA	1.02606583	0.75317102	NA
ENSG0000017	UGT2A1	protein_codir	1.02606583	0.75317102	NA
ENSG0000028	RP11-73G16.5	lncRNA	1.02606583	0.75317102	NA
ENSG0000025	RP11-597D13	lncRNA	1.02606583	0.75317102	NA
ENSG0000028	RP11-499F19.	lncRNA	1.02606583	0.75317102	NA
ENSG0000018	MARCHF11	protein_codir	1.02606583	0.75317102	NA
ENSG0000024	CTC-529L17.2	lncRNA	1.02606583	0.75317102	NA
ENSG0000015	FAM81B	protein_codir	1.02606583	0.75317102	NA
ENSG0000024	LINC02115	lncRNA	1.02606583	0.75317102	NA
ENSG0000024	RP11-114J13.	lncRNA	1.02606583	0.75317102	NA
ENSG0000027	AC005609.17	lncRNA	1.02606583	0.75317102	NA
ENSG0000017	FAM71B	protein_codir	1.02606583	0.75317102	NA
ENSG0000025	RP11-542A14	lncRNA	1.02606583	0.75317102	NA
ENSG0000018	OR2V1	protein_codir	1.02606583	0.75317102	NA
ENSG0000012	SLC17A1	protein_codir	1.02606583	0.75317102	NA
ENSG0000028	RP11-344J7.3	lncRNA	1.02606583	0.75317102	NA
ENSG0000024	SPATS1	protein_codir	1.02606583	0.75317102	NA

ENSG0000026	RP3-523K23.2	lncRNA	1.02606583	0.75317102	NA
ENSG0000024	CALHM6-AS1	lncRNA	1.02606583	0.75317102	NA
ENSG0000023	EZR-AS1	lncRNA	1.02606583	0.75317102	NA
ENSG0000023	LINC02529	lncRNA	1.02606583	0.75317102	NA
ENSG0000028	RP3-495K2.5	lncRNA	1.02606583	0.75317102	NA
ENSG0000026	RP4-777O23.1	lncRNA	1.02606583	0.75317102	NA
ENSG0000023	AC007349.7	lncRNA	1.02606583	0.75317102	NA
ENSG0000021	GNAT3	protein_codir	1.02606583	0.75317102	NA
ENSG0000025	RP11-419C23.	lncRNA	1.02606583	0.75317102	NA
ENSG0000025	CTD-2544N14	lncRNA	1.02606583	0.75317102	NA
ENSG0000025	RP11-598P20.	protein_codir	1.02606583	0.75317102	NA
ENSG0000025	RP11-246K15.	lncRNA	1.02606583	0.75317102	NA
ENSG0000025	RP11-1114I9.1	lncRNA	1.02606583	0.75317102	NA
ENSG0000028	RP11-100L22.	lncRNA	1.02606583	0.75317102	NA
ENSG0000025	RP11-96B2.1	lncRNA	1.02606583	0.75317102	NA
ENSG0000017	IFNB1	protein_codir	1.02606583	0.75317102	NA
ENSG0000022	RP11-370B11.	lncRNA	1.02606583	0.75317102	NA
ENSG0000025	RP11-101E3.5	protein_codir	1.02606583	0.75317102	NA
ENSG0000017	LCN15	protein_codir	1.02606583	0.75317102	NA
ENSG0000027	RP11-216L13.	lncRNA	1.02606583	0.75317102	NA
ENSG0000018	TMEM210	protein_codir	1.02606583	0.75317102	NA
ENSG0000020	RP11-291L22.	lncRNA	1.02606583	0.75317102	NA
ENSG0000022	LINC02667	lncRNA	1.02606583	0.75317102	NA
ENSG0000024	TUB-AS1	lncRNA	1.02606583	0.75317102	NA
ENSG0000010	DBX1	protein_codir	1.02606583	0.75317102	NA
ENSG0000028	PAUPAR	lncRNA	1.02606583	0.75317102	NA
ENSG0000025	AP000593.7	lncRNA	1.02606583	0.75317102	NA
ENSG0000013	MMP20	protein_codir	1.02606583	0.75317102	NA
ENSG0000017	FOXR1	protein_codir	1.02606583	0.75317102	NA
ENSG0000025	RP11-536G4.2	lncRNA	1.02606583	0.75317102	NA
ENSG0000025	RP11-394J1.2	lncRNA	1.02606583	0.75317102	NA
ENSG0000025	RP11-497G19	lncRNA	1.02606583	0.75317102	NA
ENSG0000028	RP11-347I19.9	lncRNA	1.02606583	0.75317102	NA
ENSG0000025	LINC02393	lncRNA	1.02606583	0.75317102	NA
ENSG0000028	RP11-395N17	lncRNA	1.02606583	0.75317102	NA
ENSG0000022	LINC00354	lncRNA	1.02606583	0.75317102	NA
ENSG0000012	PROZ	protein_codir	1.02606583	0.75317102	NA
ENSG0000025	LINC02332	lncRNA	1.02606583	0.75317102	NA
ENSG0000025	RP11-104E19.	lncRNA	1.02606583	0.75317102	NA
ENSG0000025	RP11-259K15.	lncRNA	1.02606583	0.75317102	NA
ENSG0000025	RP11-96D24.1	lncRNA	1.02606583	0.75317102	NA
ENSG0000016	OTX2	protein_codir	1.02606583	0.75317102	NA
ENSG0000025	RP11-1127D7	lncRNA	1.02606583	0.75317102	NA
ENSG0000025	CTD-2062F14.	lncRNA	1.02606583	0.75317102	NA
ENSG0000025	RP11-403B2.6	lncRNA	1.02606583	0.75317102	NA
ENSG0000026	GOLGA6L7	protein_codir	1.02606583	0.75317102	NA
ENSG0000025	RP11-96O20.2	lncRNA	1.02606583	0.75317102	NA

ENSG0000025	RP11-69G7.1	lncRNA	1.02606583	0.75317102	NA
ENSG0000026	RP11-315D16	lncRNA	1.02606583	0.75317102	NA
ENSG0000026	RP11-304L19.	lncRNA	1.02606583	0.75317102	NA
ENSG0000026	CTC-508F8.1	lncRNA	1.02606583	0.75317102	NA
ENSG0000016	MT1B	protein_codir	1.02606583	0.75317102	NA
ENSG0000026	RP11-432I5.4	lncRNA	1.02606583	0.75317102	NA
ENSG0000028	RP11-107C10.	lncRNA	1.02606583	0.75317102	NA
ENSG0000026	RP11-899L11.	lncRNA	1.02606583	0.75317102	NA
ENSG0000012	PRDM7	protein_codir	1.02606583	0.75317102	NA
ENSG0000026	RP11-68I3.5	lncRNA	1.02606583	0.75317102	NA
ENSG0000027	RP11-697E22.	lncRNA	1.02606583	0.75317102	NA
ENSG0000019	KRT35	protein_codir	1.02606583	0.75317102	NA
ENSG0000026	RP11-527L4.6	lncRNA	1.02606583	0.75317102	NA
ENSG0000022	AC006947.1	lncRNA	1.02606583	0.75317102	NA
ENSG0000018	C17orf77	lncRNA	1.02606583	0.75317102	NA
ENSG0000028	RP11-243E13.	lncRNA	1.02606583	0.75317102	NA
ENSG0000028	LINC01902	lncRNA	1.02606583	0.75317102	NA
ENSG0000027	RP11-321M21	lncRNA	1.02606583	0.75317102	NA
ENSG0000028	RP11-862L9.4	lncRNA	1.02606583	0.75317102	NA
ENSG0000026	CTC-265F19.3	lncRNA	1.02606583	0.75317102	NA
ENSG0000026	CTD-2623N2.5	lncRNA	1.02606583	0.75317102	NA
ENSG0000026	RP11-678G14	lncRNA	1.02606583	0.75317102	NA
ENSG0000026	CTC-457E21.9	lncRNA	1.02606583	0.75317102	NA
ENSG0000026	CTC-512J12.6	protein_codir	1.02606583	0.75317102	NA
ENSG0000016	ELSPBP1	protein_codir	1.02606583	0.75317102	NA
ENSG0000028	CTB-60B18.23	protein_codir	1.02606583	0.75317102	NA
ENSG0000026	CTD-2568A17	lncRNA	1.02606583	0.75317102	NA
ENSG0000026	CTD-3022G6.1	lncRNA	1.02606583	0.75317102	NA
ENSG0000027	RP5-1068H6.6	lncRNA	1.02606583	0.75317102	NA
ENSG0000012	OTOR	protein_codir	1.02606583	0.75317102	NA
ENSG0000027	RP5-1025A1.3	lncRNA	1.02606583	0.75317102	NA
ENSG0000023	RP1-269M15.	lncRNA	1.02606583	0.75317102	NA
ENSG0000022	AP000705.7	lncRNA	1.02606583	0.75317102	NA
ENSG0000016	CRYAA	protein_codir	1.02606583	0.75317102	NA
ENSG0000012	RFPL3	protein_codir	1.02606583	0.75317102	NA
ENSG0000024	APOBEC3B-AS	lncRNA	1.02606583	0.75317102	NA
ENSG0000027	CTA-280A3.2	lncRNA	1.02606583	0.75317102	NA
ENSG0000023	RP1-146A15.1	lncRNA	1.02606583	0.75317102	NA
ENSG0000010	PPP1R2C	protein_codir	1.02606583	0.75317102	NA
ENSG0000028	RP1-230G1.7	lncRNA	1.02606583	0.75317102	NA
ENSG0000017	TGIF2LY	protein_codir	1.02606583	0.75317102	NA
ENSG0000028	RP11-450B24.	lncRNA	1.02606583	0.75317102	NA
ENSG0000023	HAR1B	lncRNA	1.02570892	0.04875172	0.11818316
ENSG0000018	WDR86	protein_codir	1.02567109	0.01833657	0.05546556
ENSG0000012	KLF2	protein_codir	1.02555308	0.00161396	0.00835101
ENSG0000010	FLT1	protein_codir	1.02500392	0.00016437	0.00132313
ENSG0000027	RP11-295P9.1	lncRNA	1.02500314	0.35845604	0.51210654

ENSG0000013	SYT6	protein_codir	1.02461321	0.09305697	0.1910548
ENSG0000028	LINC00895	lncRNA	1.02417964	0.59661714	NA
ENSG0000017	PARP10	protein_codir	1.0241314	3.46E-05	0.00035979
ENSG0000010	SH3GL2	protein_codir	1.02388724	0.37786352	0.53155702
ENSG0000012	DOCK4	protein_codir	1.02374909	0.0003969	0.00271683
ENSG0000027	CTD-2017F17.	lncRNA	1.02364145	0.06450965	0.14614146
ENSG0000015	CLSTN2	protein_codir	1.02344935	0.03137871	0.0842009
ENSG0000015	DRAM2	protein_codir	1.02340058	2.69E-07	5.88E-06
ENSG0000018	TCN2	protein_codir	1.02305885	0.00018463	0.00144831
ENSG0000022	LINC02851	lncRNA	1.02272347	0.59253277	NA
ENSG0000025	LINC02617	lncRNA	1.02269227	0.59218522	NA
ENSG0000025	CYP2A6	protein_codir	1.02256419	0.59452002	NA
ENSG0000024	KRTAP5-7	protein_codir	1.02256042	0.42845544	0.58015194
ENSG0000010	PPIF	protein_codir	1.02228187	0.0007252	0.00442947
ENSG0000025	LINC02547	lncRNA	1.02222817	0.58706761	NA
ENSG0000013	TLR2	protein_codir	1.02213374	0.01800922	0.0547271
ENSG0000021	LINC01139	lncRNA	1.0221071	0.10604019	0.21082854
ENSG0000026	RP11-963H4.3	lncRNA	1.02195782	0.35685159	0.51050091
ENSG0000025	RP11-872D17	lncRNA	1.02192537	0.45818442	0.60867043
ENSG0000017	CMTM8	protein_codir	1.02161197	0.00243096	0.01144031
ENSG0000010	MCM4	protein_codir	1.02158256	0.00020428	0.00157205
ENSG0000023	LINC01819	lncRNA	1.02124859	0.1160118	0.22525454
ENSG0000010	LIPG	protein_codir	1.0210179	0.01678117	0.0519217
ENSG0000024	CTC-329D1.2	lncRNA	1.02100315	0.13465253	0.25164946
ENSG0000023	LINC01524	lncRNA	1.02052943	0.6171054	0.74086983
ENSG0000017	SLC16A11	protein_codir	1.02042905	0.03175776	0.0849951
ENSG0000014	SLC16A3	protein_codir	1.02027147	0.00131451	0.00711619
ENSG0000028	SH3PXD2A-AS	lncRNA	1.01984983	0.22068896	0.36143407
ENSG0000024	RP11-254I22.3	lncRNA	1.01956908	0.59436665	NA
ENSG0000007	TCF3	protein_codir	1.0192315	0.0002126	0.00162117
ENSG0000024	TMEFF1	protein_codir	1.01906754	0.35441538	0.50801557
ENSG0000010	GLA	protein_codir	1.01836272	0.00010037	0.00087124
ENSG0000018	ARL15	protein_codir	1.01784098	5.91E-07	1.16E-05
ENSG0000025	RP11-454K7.1	lncRNA	1.01760876	0.36629836	0.52020717
ENSG0000019	ZNF681	protein_codir	1.01743977	0.01852854	0.05593598
ENSG0000028	AC003099.2	lncRNA	1.01733036	0.227915	0.37019026
ENSG0000006	FAM107B	protein_codir	1.01721924	0.00255119	0.01185461
ENSG0000019	MME	protein_codir	1.01721897	0.06947791	0.15414732
ENSG0000023	RP11-245P10.	lncRNA	1.01688016	0.53422857	NA
ENSG0000011	FZD10	protein_codir	1.01682429	0.03342144	0.08841955
ENSG0000016	GPR182	protein_codir	1.01670174	0.1209955	0.2325444
ENSG0000018	TRPV2	protein_codir	1.01633839	1.66E-05	0.00019567
ENSG0000013	ACVRL1	protein_codir	1.01632778	1.29E-05	0.00015937
ENSG0000015	OBSCN	protein_codir	1.01614805	0.00064896	0.00404609
ENSG0000025	CTD-2314G24	lncRNA	1.01587486	0.24576536	0.39100087
ENSG0000000	GAS7	protein_codir	1.01586398	0.00327044	0.01448425
ENSG0000019	PNP	protein_codir	1.01584683	0.01652882	0.05131811

ENSG0000010	VSX1	protein_codir	1.01580614	0.35451147	0.50810061
ENSG0000014	MLIP	protein_codir	1.01570741	0.01473173	0.04706112
ENSG0000012	APOL3	protein_codir	1.01566624	7.26E-05	0.00066971
ENSG0000016	ENHO	protein_codir	1.01555296	0.05093883	0.1221718
ENSG0000024	FMN1	protein_codir	1.01524743	0.00472307	0.0194124
ENSG0000026	CTD-2192J16.	lncRNA	1.01502041	0.45633	0.60697494
ENSG0000027	RP11-25K21.6	lncRNA	1.01453928	0.52208688	0.66460984
ENSG0000014	S100A8	protein_codir	1.01424485	0.05424293	0.12795197
ENSG0000026	LINC01836	lncRNA	1.01412216	0.09229594	0.18991387
ENSG0000023	LRRC37A2	protein_codir	1.01370074	0.00290587	0.0131456
ENSG0000016	CYYR1	protein_codir	1.0130663	0.00051474	0.00334599
ENSG0000028	RP11-180P8.1	lncRNA	1.01305639	0.39226454	0.54522967
ENSG0000018	SPNS2	protein_codir	1.012913	9.37E-06	0.00012164
ENSG0000015	LINC00467	lncRNA	1.01283832	0.0010009	0.00573305
ENSG0000011	MS4A4A	protein_codir	1.01283436	0.01315683	0.04305078
ENSG0000013	CHIT1	protein_codir	1.01270726	0.31953545	0.4716277
ENSG0000016	GFRA2	protein_codir	1.01269224	0.01626357	0.05072264
ENSG0000001	SYT7	protein_codir	1.01248868	0.02220803	0.06428271
ENSG0000018	NKAIN3	protein_codir	1.01242702	0.09334581	0.19156039
ENSG0000028	LLNLR-269E7.	lncRNA	1.0121388	0.60589567	NA
ENSG0000005	MXD1	protein_codir	1.01209899	0.00174898	0.00887014
ENSG0000013	GCM1	protein_codir	1.01163776	0.35661818	0.51021984
ENSG0000023	RP11-344F13.	lncRNA	1.01142321	0.54412965	0.68235463
ENSG0000008	OVGP1	protein_codir	1.01115247	0.0097225	0.03403391
ENSG0000016	COL3A1	protein_codir	1.01099054	0.01878813	0.05653428
ENSG0000000	MEOX1	protein_codir	1.01050102	0.00536328	0.02142518
ENSG0000019	UBE2J1	protein_codir	1.01013208	2.11E-05	0.00023937
ENSG0000027	RP11-91J19.3	lncRNA	1.00995781	0.03518728	0.09189417
ENSG0000018	RBM33	protein_codir	1.00979169	7.00E-07	1.35E-05
ENSG0000021	RP11-717D12	lncRNA	1.00975136	0.23923541	0.38352092
ENSG0000028	CTD-2007H18	lncRNA	1.00915753	0.59835166	NA
ENSG0000012	GRPR	protein_codir	1.00838798	0.08980156	0.18590143
ENSG0000023	RP11-334J6.6	lncRNA	1.0083848	0.38935737	0.54233953
ENSG0000023	RP11-305L7.1	lncRNA	1.0082413	0.06696022	0.15030658
ENSG0000007	SCARB1	protein_codir	1.00789079	0.00124736	0.00681812
ENSG0000025	RP11-843A23	lncRNA	1.00771438	0.32213835	0.4743036
ENSG0000022	DNMBP-AS1	lncRNA	1.00755229	0.16441513	0.29154763
ENSG0000023	TM4SF19-AS1	lncRNA	1.00749167	0.04693959	0.11464068
ENSG0000017	DAB1	protein_codir	1.00746111	NA	NA
ENSG0000013	LOXL2	protein_codir	1.00743864	0.00140663	0.007497
ENSG0000014	PTGES	protein_codir	1.00732929	0.0018402	0.00919763
ENSG0000028	RP11-72O13.2	lncRNA	1.00698101	0.55739599	NA
ENSG0000027	RP11-329B9.4	lncRNA	1.00678591	0.00196414	0.00968566
ENSG0000010	NEFH	protein_codir	1.0064195	0.05350682	0.12662363
ENSG0000017	STAP2	protein_codir	1.00620058	0.01215813	0.04050765
ENSG0000024	SOX2-OT	lncRNA	1.00598143	0.1318781	0.24788847
ENSG0000010	RETN	protein_codir	1.00589349	0.11528052	0.22419715

ENSG0000028	LINC01488	lncRNA	1.00578553	0.60464893	NA
ENSG0000025	LINC02568	lncRNA	1.00577258	0.42798498	0.57975815
ENSG0000027	RP11-757A13	lncRNA	1.00550357	0.60204488	NA
ENSG0000006	CTNNA2	protein_codir	1.00538351	0.31020637	0.46198102
ENSG0000016	BDKRB2	protein_codir	1.00528289	0.01032151	0.03568406
ENSG0000028	SCAANT1	lncRNA	1.00515359	0.59611369	NA
ENSG0000018	AGMO	protein_codir	1.00509741	0.01726474	0.05299089
ENSG0000012	TPST2	protein_codir	1.00451543	1.71E-05	0.00020126
ENSG0000023	LINC02889	lncRNA	1.00443235	0.12205839	0.23413104
ENSG0000022	AC005481.5	lncRNA	1.00430605	0.15368746	0.27731415
ENSG0000015	FRMD1	protein_codir	1.00420477	0.1591992	0.28460077
ENSG0000025	MYG1-AS1	lncRNA	1.00381324	0.09840854	0.19938703
ENSG0000026	CTD-2293H3.1	lncRNA	1.00340224	0.13213389	0.24831857
ENSG0000016	MAP4K2	protein_codir	1.00320729	0.00157874	0.00820562
ENSG0000021	ATAD3C	protein_codir	1.00301696	0.024684	0.06990383
ENSG0000017	EIF2AK3	protein_codir	1.00294821	3.71E-06	5.53E-05
ENSG0000015	TBCEL	protein_codir	1.00228162	5.90E-05	0.00056528
ENSG0000010	ADAP1	protein_codir	1.00212543	6.33E-06	8.71E-05
ENSG0000026	CTD-2012K14	lncRNA	1.00194873	0.25806432	0.4050771
ENSG0000010	FCGRT	protein_codir	1.00144276	6.05E-06	8.40E-05
ENSG0000019	DENND4B	protein_codir	1.00130496	5.80E-05	0.00055756
ENSG0000017	B3GNTL1	protein_codir	1.00116303	0.00260824	0.01208111
ENSG0000010	SLCO4A1	protein_codir	1.0011345	0.02504141	0.07069131
ENSG0000026	CTB-31N19.3	lncRNA	1.00104661	0.07404003	0.16146747
ENSG0000025	RP11-934B9.3	lncRNA	1.00098799	0.36470003	0.51866036
ENSG0000025	LBX2-AS1	lncRNA	1.0006434	0.00414526	0.01753862
ENSG0000028	RP11-145F16.	lncRNA	1.00040295	0.55415379	NA
ENSG0000015	NCAM2	protein_codir	1.00008665	0.01885288	0.05667354
ENSG0000016	S100A9	protein_codir	1.00000657	0.03301726	0.0876104
ENSG0000015	RASA2	protein_codir	0.99989788	1.58E-07	3.70E-06
ENSG0000021	LTB4R2	protein_codir	0.99964616	7.90E-05	0.00071573
ENSG0000007	MYO3B	protein_codir	0.99961485	0.18956655	0.32385899
ENSG0000026	CTD-2267D19	lncRNA	0.99950612	2.58E-06	4.06E-05
ENSG0000025	LINC00989	lncRNA	0.9994839	0.09625865	0.19597993
ENSG0000019	TMEM26	protein_codir	0.99903832	0.1015274	0.20405957
ENSG0000010	PPARGC1A	protein_codir	0.99896275	0.10008046	0.20185645
ENSG0000021	C1orf220	lncRNA	0.99874194	0.24487558	0.38991489
ENSG0000010	C1GALT1	protein_codir	0.99873617	1.08E-09	5.41E-08
ENSG0000018	TMEM221	protein_codir	0.99869334	0.06253205	0.14257348
ENSG0000016	FAM102A	protein_codir	0.9985716	1.23E-06	2.19E-05
ENSG0000016	MGAT5B	protein_codir	0.99836832	0.00222716	0.01069627
ENSG0000018	MB21D2	protein_codir	0.99828431	0.00093411	0.00542246
ENSG0000016	LRRTM1	protein_codir	0.99811464	0.32491524	0.47724733
ENSG0000028	RP11-15N24.6	lncRNA	0.99789243	0.61390542	NA
ENSG0000022	LINC02542	lncRNA	0.9974511	0.00775138	0.02851453
ENSG0000016	SERINC5	protein_codir	0.99733067	9.09E-08	2.33E-06
ENSG0000017	DCDC1	protein_codir	0.99722939	0.09446118	0.19334461

ENSG0000027	RP11-713M15	lncRNA	0.9971996	0.01322464	0.04321123
ENSG0000028	RP11-519C12.	lncRNA	0.99705307	0.66648683	NA
ENSG0000017	ADAMTS20	protein_codir	0.9963143	0.73206332	NA
ENSG0000028	RP3-341E18.2	protein_codir	0.99630531	0.73016541	NA
ENSG0000015	CLIC6	protein_codir	0.99615462	0.02455635	0.0696298
ENSG0000012	SSUH2	protein_codir	0.99602699	0.04284279	0.10705852
ENSG0000017	DSCAM	protein_codir	0.99597712	0.26358026	0.41102568
ENSG0000016	TMEM184A	protein_codir	0.99596976	0.01629999	0.0507961
ENSG0000009	PITPNM3	protein_codir	0.99588718	0.03599743	0.09349717
ENSG0000028	RP11-457M11	lncRNA	0.99572842	0.08702958	0.1817266
ENSG0000019	C2CD4A	protein_codir	0.99552712	0.14859034	0.27059175
ENSG0000023	LINC01798	lncRNA	0.99541041	0.37852625	0.53204827
ENSG0000014	C9orf72	protein_codir	0.99539511	3.17E-06	4.84E-05
ENSG0000017	ADGRF3	protein_codir	0.99536931	0.08504372	0.17863773
ENSG0000018	CHRM5	protein_codir	0.99521075	0.10873488	0.21484065
ENSG0000010	CELF4	protein_codir	0.99510083	0.17015958	0.29898897
ENSG0000018	MRGPRX2	protein_codir	0.99507978	0.72748154	NA
ENSG0000028	RP3-431A14.7	lncRNA	0.99479004	0.47075154	0.61979967
ENSG0000015	RAB3C	protein_codir	0.99465564	0.0218794	0.0635535
ENSG0000013	SLX1A	protein_codir	0.99435065	0.00015428	0.00125292
ENSG0000018	FAM43A	protein_codir	0.9942936	0.00159481	0.00827515
ENSG0000017	CADM2	protein_codir	0.99410205	0.15282574	0.27610196
ENSG0000022	LINC02828	lncRNA	0.99400323	0.32829578	0.48069651
ENSG0000017	SMAGP	protein_codir	0.9939056	1.40E-05	0.00017098
ENSG0000010	CTSG	protein_codir	0.99364444	0.11439312	0.22286365
ENSG0000027	RP11-375N15	lncRNA	0.99292997	0.05551333	0.13010421
ENSG0000018	GDF3	protein_codir	0.99277519	0.17245294	0.30201954
ENSG0000028	RP11-340F14.	lncRNA	0.99239748	0.66510267	NA
ENSG0000017	TGIF1	protein_codir	0.99232618	9.90E-05	0.00086137
ENSG0000028	RP11-139E24.	lncRNA	0.99231305	0.1960732	0.33192636
ENSG0000013	STARD8	protein_codir	0.99216724	0.00054588	0.00351616
ENSG0000011	IL17A	protein_codir	0.99207048	0.66058548	NA
ENSG0000026	AC000032.2	lncRNA	0.99114897	0.60167111	NA
ENSG0000022	RP11-413M3.	lncRNA	0.99105174	0.59625286	NA
ENSG0000018	PPP1R27	protein_codir	0.99096327	0.53675124	0.67685108
ENSG0000009	PALM	protein_codir	0.99068566	0.01015448	0.03523471
ENSG0000025	RP11-231I16.	lncRNA	0.989944	0.56326206	NA
ENSG0000026	RP11-104H15	lncRNA	0.98973355	0.33596763	0.48887972
ENSG0000008	TTC39A	protein_codir	0.98953061	0.05407982	0.12766535
ENSG0000016	CFAP47	protein_codir	0.9893316	0.6109392	NA
ENSG0000019	LEKR1	protein_codir	0.98931639	0.07696816	0.16592729
ENSG0000026	CYLD-AS1	lncRNA	0.98913016	0.056752	0.13227599
ENSG0000015	GPR153	protein_codir	0.98774138	0.00080008	0.00478837
ENSG0000004	ANO2	protein_codir	0.98774072	0.0167487	0.05184925
ENSG0000026	RP11-35G9.3	lncRNA	0.98712289	0.01070899	0.03677055
ENSG0000012	LRRC4	protein_codir	0.98704078	0.00338405	0.01487133
ENSG0000025	RP11-4O1.2	lncRNA	0.98700262	0.00017924	0.00141513

ENSG0000008	CMTM1	protein_codir	0.98663697	0.00181762	0.00911452
ENSG0000028	SLC7A14-AS1	lncRNA	0.98654657	0.27820776	0.42682322
ENSG0000012	BTN2A2	protein_codir	0.98648791	0.00544699	0.02169381
ENSG0000012	KDR	protein_codir	0.98526755	0.00158457	0.00823442
ENSG0000021	NRAS	protein_codir	0.98434286	1.61E-05	0.00019103
ENSG0000022	BACH1-IT2	lncRNA	0.98432354	0.07332658	0.16036472
ENSG0000016	SLC18A2	protein_codir	0.98425176	0.03190046	0.08522016
ENSG0000023	RP3-475N16.1	lncRNA	0.98418217	0.00577844	0.02266408
ENSG0000028	RP11-206M19	lncRNA	0.98409727	0.01244063	0.04119531
ENSG0000026	CTB-31O20.2	lncRNA	0.9840617	0.00016735	0.00134054
ENSG0000027	RP11-302L19.	lncRNA	0.9840454	0.07782087	0.1672304
ENSG0000018	ERC2	protein_codir	0.98367843	0.07427372	0.16182379
ENSG0000024	CTD-2587M2.	lncRNA	0.98367652	0.01397589	0.04511618
ENSG0000027	RP1-228P16.8	lncRNA	0.98358439	0.06466727	0.14640245
ENSG0000015	DLG2	protein_codir	0.98354678	0.00036957	0.00256281
ENSG0000011	LIN7A	protein_codir	0.98327786	0.00950016	0.03342416
ENSG0000017	ZDHHC13	protein_codir	0.98298414	0.00013733	0.00113869
ENSG0000018	PLCXD1	protein_codir	0.98291736	0.0065081	0.02490009
ENSG0000018	CTD-3064H18	lncRNA	0.98251762	0.41775903	0.5704506
ENSG0000023	AC114730.2	lncRNA	0.98234002	0.06986345	0.15486585
ENSG0000013	APOC1	protein_codir	0.98233772	0.16838595	0.29677876
ENSG0000013	MDFIC	protein_codir	0.98211028	2.86E-07	6.21E-06
ENSG0000026	CTD-2240E14.	lncRNA	0.98196194	0.50754616	0.65198944
ENSG0000014	ABL2	protein_codir	0.98131669	0.00737389	0.02743727
ENSG0000011	EYA4	protein_codir	0.98113577	0.01105388	0.0376901
ENSG0000027	SCARNA10	lncRNA	0.98063178	0.44773029	0.59857049
ENSG0000028	RP11-157L14.	lncRNA	0.9800825	0.27776125	0.42628025
ENSG0000012	SLC25A19	protein_codir	0.97991608	0.00016553	0.00132915
ENSG0000017	FAM90A1	protein_codir	0.9795509	0.04112104	0.10371318
ENSG0000027	RP11-283G6.6	lncRNA	0.97933498	0.57168696	NA
ENSG0000017	CD163	protein_codir	0.97931085	0.04657607	0.11401499
ENSG0000025	RP11-326C3.1	lncRNA	0.9791355	0.1195231	0.23025933
ENSG0000024	LINC02029	lncRNA	0.97906267	0.45256626	0.60307233
ENSG0000026	SLC9A3R1-AS1	lncRNA	0.97896716	0.57763568	0.70990022
ENSG0000013	PRRG3	protein_codir	0.9787111	0.03671726	0.09496854
ENSG0000026	CTD-2369P2.1	protein_codir	0.97839453	0.32039414	0.47244067
ENSG0000018	PLCB1	protein_codir	0.97833459	0.00283007	0.0128762
ENSG0000013	CIDEB	protein_codir	0.97808746	0.00020729	0.00159084
ENSG0000026	AC010547.9	protein_codir	0.97795395	0.10117636	0.20356142
ENSG0000016	WDR81	protein_codir	0.97775905	6.49E-05	0.00060943
ENSG0000025	RP11-90E5.1	lncRNA	0.97736735	0.6712104	NA
ENSG0000023	RP11-334A14	lncRNA	0.97682019	0.36600283	0.51990321
ENSG0000017	CHST11	protein_codir	0.97655062	0.000292	0.00210813
ENSG0000007	ST6GAL1	protein_codir	0.97647158	0.00344709	0.01508935
ENSG0000013	SLF1	protein_codir	0.97634423	2.70E-05	0.00029415
ENSG0000016	NTN3	protein_codir	0.97569055	0.15630197	0.28082094
ENSG0000014	ST8SIA6	protein_codir	0.97552522	0.01566534	0.04927419

ENSG0000024	UGT1A1	protein_codir	0.97544636	0.6137431	NA
ENSG0000028	RP11-36B6.2	lncRNA	0.97513794	0.01184322	0.0397015
ENSG0000026	RP11-407G23	lncRNA	0.97486454	0.44708548	0.5979035
ENSG0000012	OPN4	protein_codir	0.97475769	0.32991534	0.4824621
ENSG0000013	FGFBP2	protein_codir	0.9743663	0.07977488	0.17026367
ENSG0000016	SNRK	protein_codir	0.97397958	2.39E-07	5.30E-06
ENSG0000023	RP11-195C7.1	lncRNA	0.97349668	0.21355308	0.3532784
ENSG0000019	SULF2	protein_codir	0.97339225	0.00018523	0.00145218
ENSG0000007	LLGL2	protein_codir	0.97324114	0.00164124	0.00845901
ENSG0000020	MUC12	protein_codir	0.97284736	0.14743344	0.26901708
ENSG0000025	NHLRC4	protein_codir	0.97282478	0.00977129	0.03416114
ENSG0000018	KIF24	protein_codir	0.97224797	0.00415363	0.01756596
ENSG0000018	FAM43B	protein_codir	0.97199997	0.02914533	0.07974184
ENSG0000016	TRPV6	protein_codir	0.97185278	0.28254165	0.43207057
ENSG0000016	NOSTRIN	protein_codir	0.97180164	0.00888806	0.03173323
ENSG0000018	FRAT2	protein_codir	0.97148507	0.00254031	0.01182397
ENSG0000018	GPR137C	protein_codir	0.97103846	0.00057086	0.00364055
ENSG0000017	NIM1K	protein_codir	0.97099154	0.02552513	0.07178407
ENSG0000015	TBC1D31	protein_codir	0.97095439	0.00244292	0.01148092
ENSG0000023	AP001189.4	lncRNA	0.97086242	0.20120736	0.33814035
ENSG0000027	RP11-171I2.4	lncRNA	0.97072621	0.05162076	0.12324016
ENSG0000028	CTA-215D11.5	lncRNA	0.97072347	0.28703137	0.43652918
ENSG0000010	CORO7-PAM1	protein_codir	0.97071311	0.32697452	0.47922489
ENSG0000014	MED12L	protein_codir	0.97061079	0.00472571	0.01942034
ENSG0000017	USP50	protein_codir	0.97055641	0.12262327	0.2349209
ENSG0000017	LRRC37A	protein_codir	0.97047012	0.03190974	0.08522016
ENSG0000016	ABCA1	protein_codir	0.97037617	0.00469956	0.01934053
ENSG0000025	PINX1	protein_codir	0.96969699	0.47408697	0.62255894
ENSG0000001	GABRA3	protein_codir	0.96960599	0.4757952	0.62406037
ENSG0000011	CEP85L	protein_codir	0.96929781	1.84E-07	4.22E-06
ENSG0000013	RAB30	protein_codir	0.96917403	6.81E-05	0.00063468
ENSG0000023	LINC02773	lncRNA	0.96899935	0.0546062	0.12854577
ENSG0000026	RP11-385D13	lncRNA	0.96880231	0.21397701	0.35385254
ENSG0000027	RP11-18C24.8	lncRNA	0.96799603	0.57415589	NA
ENSG0000015	LRRK1	protein_codir	0.96793911	1.37E-07	3.29E-06
ENSG0000027	RP11-248J18.	lncRNA	0.96783332	0.00743539	0.02760479
ENSG0000016	SHE	protein_codir	0.96778066	0.00060963	0.00384167
ENSG0000014	TRPM8	protein_codir	0.96772347	0.29954196	0.45061814
ENSG0000023	LINC02595	lncRNA	0.9674264	0.21009718	0.34927677
ENSG0000026	CTD-3154N5.	lncRNA	0.96701685	0.67283981	NA
ENSG0000018	CYP4F12	protein_codir	0.96690294	0.03124171	0.08397266
ENSG0000023	AC009542.2	lncRNA	0.9667304	0.58857509	0.7187033
ENSG0000025	SNHG1	lncRNA	0.96667373	0.00185632	0.00926313
ENSG0000024	ZBED5-AS1	lncRNA	0.96654871	8.60E-05	0.00076927
ENSG0000015	ABCA5	protein_codir	0.96649966	0.00013318	0.00110827
ENSG0000007	CDH17	protein_codir	0.96643111	0.16924255	0.29777546
ENSG0000009	CCDC80	protein_codir	0.96624614	0.01041491	0.03595748

ENSG0000018	LINC01006	lncRNA	0.96606844	0.00026114	0.00192385
ENSG0000016	RAC3	protein_codir	0.96599158	0.03433906	0.09033668
ENSG0000000	TKTL1	protein_codir	0.96586362	0.11266506	0.22044553
ENSG0000016	ELF3	protein_codir	0.96585449	0.07444574	0.16207073
ENSG0000010	CRYBB1	protein_codir	0.96575797	0.00659265	0.02511178
ENSG0000026	CTC-239J10.1	lncRNA	0.96574878	0.73125042	NA
ENSG0000018	IDH2	protein_codir	0.96563121	2.81E-06	4.36E-05
ENSG0000027	RP11-214K3.1	lncRNA	0.96561546	0.28442261	0.43401947
ENSG0000016	INHBB	protein_codir	0.96551451	0.01625113	0.05071247
ENSG0000026	PAPOLA-DT	lncRNA	0.96542691	0.1407127	0.25994816
ENSG0000027	RP11-272L14.	lncRNA	0.9653042	0.36818219	0.52166413
ENSG0000012	APAF1	protein_codir	0.96527892	2.66E-06	4.17E-05
ENSG0000025	RP11-130F10.	lncRNA	0.96510496	0.06297916	0.14339167
ENSG0000014	ZNF385B	protein_codir	0.96497435	0.05424165	0.12795197
ENSG0000024	RP11-242C19.	lncRNA	0.96464909	0.18792568	0.32176997
ENSG0000013	SORL1	protein_codir	0.96447047	0.00247599	0.01160477
ENSG0000019	UAP1L1	protein_codir	0.9642299	0.00111843	0.00625201
ENSG0000028	RP11-627C1.1	lncRNA	0.96416863	0.66897122	NA
ENSG0000021	RP11-287D1.4	lncRNA	0.96412254	0.44464421	0.59579261
ENSG0000017	GPR35	protein_codir	0.96376487	0.0052383	0.02103545
ENSG0000018	RAD51B	protein_codir	0.96363799	6.66E-05	0.00062273
ENSG0000023	PSMD6-AS2	lncRNA	0.96359011	0.01107822	0.03774276
ENSG0000011	CCND3	protein_codir	0.96315377	0.00040989	0.00278983
ENSG0000024	IQCJ-SCHIP1-/	lncRNA	0.96279545	0.73081924	NA
ENSG0000025	RP11-797A18	lncRNA	0.96235019	0.42218725	0.57416967
ENSG0000017	CD14	protein_codir	0.96185199	0.00414265	0.01753024
ENSG0000016	LRRC38	protein_codir	0.96056041	0.343526	0.49668576
ENSG0000027	RP4-620E11.8	lncRNA	0.96049608	0.09997867	0.20172481
ENSG0000027	XXbac-BPG30	lncRNA	0.95949659	0.13428391	0.25116283
ENSG0000014	ARRDC4	protein_codir	0.95948143	0.00017684	0.00139909
ENSG0000022	HDAC2-AS2	lncRNA	0.95946451	0.01192881	0.03989751
ENSG0000009	DSP	protein_codir	0.95935688	0.04347357	0.10824305
ENSG0000027	STPG3-AS1	lncRNA	0.95904692	0.16914902	0.29770572
ENSG0000028	RP11-22B23.4	lncRNA	0.95893574	0.46542313	0.61515944
ENSG0000011	MAN1A1	protein_codir	0.95864101	7.39E-05	0.0006791
ENSG0000015	HK2	protein_codir	0.95835288	0.00570446	0.02244404
ENSG0000018	LINC00943	lncRNA	0.95794619	0.66862651	NA
ENSG0000025	LINC02125	lncRNA	0.95789782	0.67036614	NA
ENSG0000026	AC135048.13	lncRNA	0.95750856	0.03828403	0.09803194
ENSG0000022	XIST	lncRNA	0.95683171	NA	NA
ENSG0000028	RP11-373E16.	lncRNA	0.95678204	0.20575968	0.34380414
ENSG0000002	NR1H3	protein_codir	0.956713	0.00909401	0.03233895
ENSG0000022	LINC01118	lncRNA	0.95629409	0.19342327	0.3287487
ENSG0000015	OLAH	protein_codir	0.95620748	0.12386008	0.23663385
ENSG0000006	ATP11A	protein_codir	0.95593285	4.39E-05	0.00043776
ENSG0000018	KANK3	protein_codir	0.95577262	0.00280639	0.01279717
ENSG0000022	BAIAP2-DT	lncRNA	0.95571709	0.00015604	0.00126462

ENSG0000015	RAPGEF6	protein_codir	0.95563701	0.00014277	0.00117641
ENSG0000016	ATF7IP2	protein_codir	0.95560954	0.01228411	0.04079811
ENSG0000013	KMT5C	protein_codir	0.95560578	0.00283071	0.0128762
ENSG0000027	LLNLR-284B4.	lncRNA	0.95555585	0.13375094	0.25043732
ENSG0000010	NAMPT	protein_codir	0.95530381	0.02742094	0.07584281
ENSG0000025	LINC02228	lncRNA	0.95424198	0.68182275	NA
ENSG0000025	RP11-248N22	lncRNA	0.95389507	0.61091166	NA
ENSG0000009	IZUMO4	protein_codir	0.95382565	0.00879089	0.03143656
ENSG0000018	KCNQ3	protein_codir	0.95378636	0.00719408	0.02688776
ENSG0000018	MAPK11	protein_codir	0.95374942	0.00049492	0.00324388
ENSG0000016	CCNL1	protein_codir	0.95374388	0.00923411	0.03272338
ENSG0000028	DGCR12	lncRNA	0.9533738	0.05816975	0.13470685
ENSG0000022	RP11-58A12.3	lncRNA	0.95312577	0.52556684	NA
ENSG0000028	CTC-359D24.6	protein_codir	0.9530981	0.18639237	0.31976133
ENSG0000023	LINC01436	lncRNA	0.9527287	0.24948366	0.39533784
ENSG0000017	BAIAP2	protein_codir	0.95270563	0.00496717	0.02016361
ENSG0000024	CTD-3064M3.	lncRNA	0.95242053	0.3745663	0.52814125
ENSG0000028	SMIM33	protein_codir	0.95237711	0.54101493	0.67983769
ENSG0000010	RRN3P2	lncRNA	0.95215541	0.00252954	0.01178575
ENSG0000025	RP11-755F10.	lncRNA	0.95173402	0.42293677	0.57499082
ENSG0000016	PDE9A	protein_codir	0.9516507	0.00219011	0.01054952
ENSG0000009	EPB41L4B	protein_codir	0.95162788	0.08215367	0.17400955
ENSG0000027	KB-431C1.5	lncRNA	0.95158142	0.23474081	0.37811519
ENSG0000028	CTD-2022J23.	lncRNA	0.95146072	0.6721753	NA
ENSG0000013	PIM1	protein_codir	0.95142928	0.03160095	0.08467387
ENSG0000007	UBE2T	protein_codir	0.95091409	0.02635993	0.07352668
ENSG0000013	ADAM10	protein_codir	0.95050977	2.16E-07	4.83E-06
ENSG0000012	CLN6	protein_codir	0.95046828	0.0002169	0.0016472
ENSG0000023	NPIPB2	protein_codir	0.95035133	0.07085495	0.15646096
ENSG0000007	FSCN1	protein_codir	0.95034147	0.00010687	0.00091951
ENSG0000018	RIPPLY3	protein_codir	0.94954579	0.56296895	NA
ENSG0000018	KCND2	protein_codir	0.94852864	0.04456744	0.11032869
ENSG0000012	F13A1	protein_codir	0.94848243	0.03108805	0.08368927
ENSG0000028	RP11-74C13.6	lncRNA	0.94845288	0.68274543	NA
ENSG0000022	HSBP1L1	protein_codir	0.94843039	0.00479917	0.01965122
ENSG0000024	RP11-54O7.14	lncRNA	0.94833114	0.42045559	0.57266056
ENSG0000024	LINC02261	lncRNA	0.94827947	0.68638609	NA
ENSG0000026	RP11-799D4.4	lncRNA	0.94805679	0.00177723	0.00897642
ENSG0000016	NKX6-3	protein_codir	0.94764787	0.42147305	0.5734525
ENSG0000027	CTD-3075F15.	lncRNA	0.947535	0.21947093	0.3602631
ENSG0000020	TMEM170B	protein_codir	0.94744368	0.00283068	0.0128762
ENSG0000028	RP11-386G11	protein_codir	0.94736474	0.07244181	0.15900705
ENSG0000023	TSPEAR-AS1	lncRNA	0.94706147	0.04886388	0.11838476
ENSG0000024	RP11-292E2.2	lncRNA	0.94634849	0.63205441	NA
ENSG0000010	SNX8	protein_codir	0.94631201	1.02E-05	0.00013068
ENSG0000025	RP11-488L18.	lncRNA	0.94615864	0.01123394	0.03814083
ENSG0000027	RP11-134L10.	lncRNA	0.94613832	0.00108402	0.00610435

ENSG0000028	RP11-379H18	lncRNA	0.94598458	0.11291622	0.22076028
ENSG0000017	MYD88	protein_codir	0.94571457	1.71E-05	0.00020046
ENSG0000019	GRM7	protein_codir	0.94538867	0.02724995	0.07547569
ENSG0000012	CHTF18	protein_codir	0.9452467	0.00474848	0.01948493
ENSG0000021	RP1-257A7.4	lncRNA	0.94489241	0.05499568	0.12920898
ENSG0000017	ZBTB18	protein_codir	0.94469768	9.26E-07	1.72E-05
ENSG0000028	FAM106C	lncRNA	0.94325169	0.12305643	0.23552205
ENSG0000018	ZNF804B	protein_codir	0.94324351	0.60094929	0.72815176
ENSG0000015	ZFYVE28	protein_codir	0.94315733	0.00069689	0.00428687
ENSG0000027	CTD-2260A17	lncRNA	0.94303757	0.36160103	0.51558621
ENSG0000014	RNF157	protein_codir	0.94297961	0.0338937	0.08936936
ENSG0000011	CEP72	protein_codir	0.94293588	2.19E-05	0.00024715
ENSG0000022	AC092155.4	lncRNA	0.94289054	0.33435001	0.48725927
ENSG0000028	RP11-118B23.	lncRNA	0.94285285	0.02068528	0.06087016
ENSG0000028	CTD-2040G12	lncRNA	0.94280637	0.29277702	0.44291111
ENSG0000018	OXTR	protein_codir	0.94232363	0.09272375	0.19059536
ENSG0000027	RP11-731C17.	lncRNA	0.94229673	0.03234352	0.08615374
ENSG0000028	RP11-468E2.1	lncRNA	0.94223378	0.21532663	0.35548843
ENSG0000016	RBM15	protein_codir	0.94209186	4.89E-06	6.98E-05
ENSG0000009	CREM	protein_codir	0.941903	0.01132202	0.03836512
ENSG0000010	SLC7A6	protein_codir	0.94172153	0.00020608	0.00158332
ENSG0000022	LINC01546	lncRNA	0.94168867	0.17192554	0.30128694
ENSG0000025	STX16-NPEPL1	protein_codir	0.94113042	0.01206947	0.04025586
ENSG0000018	HMX3	protein_codir	0.94108402	0.19957739	0.33609704
ENSG0000018	CCDC157	protein_codir	0.9409203	0.00574257	0.02255864
ENSG0000022	AC008746.3	lncRNA	0.9407768	0.42805906	0.57978577
ENSG0000026	SLX1B-SULT1A	lncRNA	0.94077179	0.04557023	0.11226919
ENSG0000028	CTD-2574D22	protein_codir	0.94059241	0.16702121	0.29512673
ENSG0000024	LINC02405	lncRNA	0.94027388	0.63283644	NA
ENSG0000026	ARHGAP19-SL	protein_codir	0.93990189	0.47134557	0.62025003
ENSG0000023	RPL34-DT	lncRNA	0.93990102	0.18042989	0.31199178
ENSG0000017	CYBC1	protein_codir	0.93980538	0.00040471	0.00276005
ENSG0000018	SV2B	protein_codir	0.93960383	0.00806861	0.02942422
ENSG0000024	LINC01213	lncRNA	0.93958694	0.46707267	0.61664583
ENSG0000017	MAP3K11	protein_codir	0.93947129	4.43E-10	2.55E-08
ENSG0000010	KCNN1	protein_codir	0.93913023	0.10067364	0.20271736
ENSG0000022	SH3BP5-AS1	lncRNA	0.93883108	0.01560112	0.04912816
ENSG0000016	SYVN1	protein_codir	0.93865945	1.87E-05	0.0002162
ENSG0000023	LINC00852	lncRNA	0.93825684	0.00125838	0.0068675
ENSG0000019	C3orf35	lncRNA	0.9382262	0.05133754	0.12280821
ENSG0000012	ODF2L	protein_codir	0.93806756	0.0002545	0.00188149
ENSG0000016	TDRD10	protein_codir	0.93769849	0.02010446	0.05953933
ENSG0000012	TEKT3	protein_codir	0.93768375	0.02250224	0.0649845
ENSG0000016	HENMT1	protein_codir	0.93766532	0.0001321	0.00110072
ENSG0000022	RP11-251M1.	lncRNA	0.93693558	0.07382523	0.16112627
ENSG0000005	CDK14	protein_codir	0.93665906	0.00017294	0.0013769
ENSG0000012	TMEM131L	protein_codir	0.93660253	0.00096606	0.00557417

ENSG0000013	BHLHE40	protein_codir	0.93647445	0.02155575	0.06280935
ENSG0000013	VILL	protein_codir	0.93566954	0.0006031	0.00381094
ENSG0000019	ARHGEF15	protein_codir	0.93562977	0.00042989	0.00288898
ENSG0000011	EFCC1	protein_codir	0.93505346	0.01949177	0.05815109
ENSG0000025	RP11-110I1.6	lncRNA	0.93502484	0.46488055	0.61479535
ENSG0000018	MAP7D2	protein_codir	0.9349468	0.30731854	0.45903318
ENSG0000014	UNC80	protein_codir	0.93470816	0.18380978	0.31653897
ENSG0000018	GAS1	protein_codir	0.93393834	0.00147712	0.00779632
ENSG0000010	VRK1	protein_codir	0.93382413	1.28E-05	0.00015797
ENSG0000012	TEK	protein_codir	0.93344874	0.00020031	0.00154726
ENSG0000026	RP11-729L2.2	protein_codir	0.93312726	0.13878869	0.25723686
ENSG0000024	AC007620.3	lncRNA	0.93247332	0.10789455	0.21356224
ENSG0000024	UBL7-AS1	lncRNA	0.93238863	0.00362983	0.01572397
ENSG0000025	NPIPB11	protein_codir	0.931921	0.03182426	0.08511123
ENSG0000013	THSD1	protein_codir	0.93165821	0.00618566	0.02390817
ENSG0000024	LINC02453	lncRNA	0.93091469	0.16078726	0.28667028
ENSG0000027	LINC00551	lncRNA	0.93089015	0.10774234	0.2133309
ENSG0000017	RNF212	protein_codir	0.93085122	0.00510916	0.02061623
ENSG0000016	RBP7	protein_codir	0.93074131	0.00823088	0.02987343
ENSG0000027	LINC02550	lncRNA	0.93013615	0.16534795	0.29275521
ENSG0000011	CAPZA1	protein_codir	0.9300669	5.04E-05	0.00049461
ENSG0000000	CFLAR	protein_codir	0.9297444	4.67E-08	1.31E-06
ENSG0000014	ARHGAP18	protein_codir	0.9290951	0.00082101	0.00489031
ENSG0000024	ECSCR	protein_codir	0.92906083	0.00520567	0.02094096
ENSG0000028	RP11-178C10.	lncRNA	0.92876191	0.41897659	0.57157628
ENSG0000028	RP11-204E9.3	lncRNA	0.92866918	0.53161399	NA
ENSG0000011	GSG1	protein_codir	0.92822754	0.52037381	0.66310149
ENSG0000027	RP11-348N5.7	lncRNA	0.92777347	0.03117057	0.08385421
ENSG0000026	LINC02594	lncRNA	0.92771392	0.26396227	0.41141233
ENSG0000017	CES3	protein_codir	0.92769752	0.01038532	0.03588222
ENSG0000000	SS18L2	protein_codir	0.92746828	4.78E-05	0.00047156
ENSG0000014	GBGT1	protein_codir	0.92745452	0.00322663	0.01432559
ENSG0000024	BMS1P4-AGA	lncRNA	0.92719197	0.01172011	0.03941426
ENSG0000024	ARHGAP22-IT	lncRNA	0.92694814	0.69071977	NA
ENSG0000024	PCDHAC2	protein_codir	0.92693547	0.08419588	0.1771802
ENSG0000019	H2BU1	protein_codir	0.92653551	0.21807836	0.35848476
ENSG0000028	CTD-2033C11	lncRNA	0.92627504	0.30892183	0.46051495
ENSG0000027	RP3-324O17.8	lncRNA	0.92613116	0.4754329	0.62367402
ENSG0000028	RP3-475N16.9	lncRNA	0.92596091	0.02189975	0.06358364
ENSG0000027	RP13-516M14	lncRNA	0.92562977	0.23437076	0.37776123
ENSG0000017	TMEM52	protein_codir	0.9252851	0.10583095	0.21053
ENSG0000027	RP11-532F6.4	lncRNA	0.92515344	0.53315965	NA
ENSG0000009	PALMD	protein_codir	0.92498379	0.01124459	0.03815892
ENSG0000016	FAM111A	protein_codir	0.92493621	0.00069603	0.00428282
ENSG0000025	RP11-660L16.	lncRNA	0.92486032	5.33E-05	0.00051972
ENSG0000025	RP11-864I4.4	lncRNA	0.9245657	0.53419082	NA
ENSG0000016	NFIL3	protein_codir	0.92455231	0.03318452	0.08792751

ENSG0000014	SLC20A1	protein_codir	0.92439029	0.00023457	0.00175771
ENSG0000013	CAPN9	protein_codir	0.92402473	0.51906712	0.66192736
ENSG0000010	RPL28	protein_codir	0.92390408	5.73E-05	0.00055108
ENSG0000015	NRG1	protein_codir	0.92372662	0.07923314	0.16948724
ENSG0000018	GRID1	protein_codir	0.92358592	0.05005789	0.12061903
ENSG0000028	RP11-239A17	lncRNA	0.92336213	0.6838542	NA
ENSG0000026	RP11-521I2.3	lncRNA	0.92334207	0.09875083	0.19984598
ENSG0000013	REM2	protein_codir	0.92325219	0.00829033	0.03002216
ENSG0000028	RP11-237N2.1	lncRNA	0.9230533	0.74474418	NA
ENSG0000028	RP11-657K20.	lncRNA	0.92262677	0.18022465	0.31173628
ENSG0000019	IGF2R	protein_codir	0.92174987	0.0092748	0.03285916
ENSG0000020	CD177	protein_codir	0.92146111	0.25895804	0.40604151
ENSG0000022	RP3-508I15.14	lncRNA	0.9214351	0.17288701	0.30254952
ENSG0000011	WNT6	protein_codir	0.92135473	0.00772046	0.02840915
ENSG0000016	DACT2	protein_codir	0.9211874	0.05759477	0.13368862
ENSG0000011	ECT2	protein_codir	0.92117316	3.85E-05	0.00039243
ENSG0000017	PRSS30P	lncRNA	0.92069319	0.10263318	0.20578792
ENSG0000016	SGO2	protein_codir	0.92066506	0.0011772	0.00651068
ENSG0000028	RELA-DT	lncRNA	0.92024513	0.00379327	0.01630411
ENSG0000003	USP2	protein_codir	0.92020508	0.10869675	0.21479604
ENSG0000024	APOBEC3C	protein_codir	0.92017556	0.0014921	0.00785136
ENSG0000027	RP11-425D17	lncRNA	0.92015002	0.3214758	0.47365633
ENSG0000015	TMEM123	protein_codir	0.92013262	9.13E-06	0.00011936
ENSG0000014	ATXN7L1	protein_codir	0.91918552	2.67E-05	0.00029187
ENSG0000012	SIGLEC9	protein_codir	0.91866253	0.0438822	0.10896838
ENSG0000011	ZNF430	protein_codir	0.91815372	0.00087062	0.00512716
ENSG0000022	AKT3-IT1	lncRNA	0.9175794	0.35757656	0.51106156
ENSG0000023	CYP1B1-AS1	lncRNA	0.91742704	0.01731205	0.05308157
ENSG0000015	RPGR	protein_codir	0.91729945	0.00170478	0.00870031
ENSG0000027	TYW1B	protein_codir	0.91713553	0.00505856	0.02047443
ENSG0000023	LINC01191	lncRNA	0.9171346	0.1327102	0.24906426
ENSG0000013	TRPM1	protein_codir	0.91694821	0.56757882	0.70134785
ENSG0000028	RP11-660K21.	protein_codir	0.91672935	0.40885192	0.56181542
ENSG0000014	CDC42EP2	protein_codir	0.91669606	0.03329315	0.08813926
ENSG0000014	NEIL1	protein_codir	0.91657708	0.05568978	0.13041817
ENSG0000008	PTPRH	protein_codir	0.91640867	0.13684234	0.25443188
ENSG0000028	RP11-392A14	lncRNA	0.91628874	0.07096895	0.15661253
ENSG0000022	LINC01133	lncRNA	0.916261	0.05240825	0.12463542
ENSG0000015	PSD3	protein_codir	0.91584633	0.0199471	0.05918346
ENSG0000024	LINC01252	lncRNA	0.91565569	0.09533426	0.19458559
ENSG0000025	ZNF878	protein_codir	0.91560059	0.24363328	0.38872111
ENSG0000011	HRH2	protein_codir	0.91534075	0.00300726	0.01352557
ENSG0000006	LZTS1	protein_codir	0.91494223	0.00733535	0.02729753
ENSG0000028	LINC01669	lncRNA	0.91479037	0.39562255	0.54852141
ENSG0000011	IVNS1ABP	protein_codir	0.91472889	0.00031894	0.0022675
ENSG0000017	LCORL	protein_codir	0.91457721	6.10E-06	8.46E-05
ENSG0000028	RP11-350J20.	lncRNA	0.91431754	0.22311775	0.36451629

ENSG0000016	CAMKV	protein_codir	0.91420852	0.58707317	NA
ENSG0000020	ZNF551	protein_codir	0.9139265	0.0023375	0.01111216
ENSG0000023	RP3-508I15.1!	lncRNA	0.91382063	0.10249693	0.2056192
ENSG0000012	RDH10	protein_codir	0.91366077	0.01273065	0.04192946
ENSG0000016	ATF5	protein_codir	0.9135069	0.00216773	0.01046177
ENSG0000018	RALYL	protein_codir	0.9134525	0.53437599	0.67493437
ENSG0000027	CTD-2589H19	lncRNA	0.91325862	0.17268154	0.30231425
ENSG0000007	KIF22	protein_codir	0.91320533	0.00023778	0.00177786
ENSG0000016	ANKZF1	protein_codir	0.91295705	0.01119677	0.03805277
ENSG0000013	PRCP	protein_codir	0.91278889	2.15E-05	0.00024388
ENSG0000022	RP11-218L14.	lncRNA	0.91257789	0.50999524	NA
ENSG0000011	DTX4	protein_codir	0.91254261	0.01675908	0.05186977
ENSG0000008	RBL1	protein_codir	0.91252395	0.00021659	0.00164573
ENSG0000014	NPAT	protein_codir	0.91233028	3.32E-05	0.00034786
ENSG0000018	RASSF10	protein_codir	0.91217223	0.2441687	0.38923928
ENSG0000003	C6	protein_codir	0.91201816	0.12773068	0.24193673
ENSG0000010	RIPK2	protein_codir	0.91197893	2.96E-06	4.56E-05
ENSG0000028	RP11-674E16.	lncRNA	0.91144297	0.39223944	0.54522967
ENSG0000015	SYCP2L	protein_codir	0.91091183	0.08608918	0.18028532
ENSG0000009	NUP50	protein_codir	0.91074983	0.00011282	0.00096499
ENSG0000028	AC011380.9	lncRNA	0.91066594	0.62301794	0.74573155
ENSG0000013	DRAM1	protein_codir	0.91033257	6.87E-06	9.33E-05
ENSG0000020	PKHD1L1	protein_codir	0.91029164	0.14211183	0.2616927
ENSG0000014	CNKSR2	protein_codir	0.91002216	0.00593166	0.02313089
ENSG0000025	RFPL4B	protein_codir	0.90972419	0.54717409	0.68492855
ENSG0000014	SESN3	protein_codir	0.90935765	0.00113281	0.00630812
ENSG0000007	ITM2A	protein_codir	0.90931706	0.0171942	0.0528199
ENSG0000013	HCN4	protein_codir	0.9091343	0.18326626	0.31585314
ENSG0000028	RP11-752G15	lncRNA	0.90877203	0.17094535	0.3000261
ENSG0000010	TYK2	protein_codir	0.90853014	0.0004869	0.00320195
ENSG0000009	YPEL3	protein_codir	0.9084963	2.53E-05	0.0002792
ENSG0000012	EQTN	protein_codir	0.90838916	0.49414787	0.64037743
ENSG0000020	CFAP44	protein_codir	0.90817553	0.00255528	0.01186565
ENSG0000028	RP4-613B23.7	lncRNA	0.90805454	0.75180302	0.84190423
ENSG0000004	INPP4A	protein_codir	0.90803557	2.69E-10	1.67E-08
ENSG0000025	IQCH-AS1	lncRNA	0.90795636	0.0012469	0.00681699
ENSG0000015	ADAMTS4	protein_codir	0.90783563	0.17826952	0.30922591
ENSG0000017	CLEC14A	protein_codir	0.90765075	0.00049336	0.00323782
ENSG0000024	RP11-875O11	lncRNA	0.90736515	0.403933	0.55702379
ENSG0000018	DRICH1	protein_codir	0.90700972	0.2171088	0.35736663
ENSG0000019	IL1RAP	protein_codir	0.90681073	0.0056502	0.02228066
ENSG0000015	SLFN13	protein_codir	0.90637219	0.0004148	0.00281127
ENSG0000019	SFI1	protein_codir	0.90634252	0.00481139	0.01969332
ENSG0000020	ACCSL	protein_codir	0.9057389	0.78122583	NA
ENSG0000013	SKIL	protein_codir	0.90565014	5.97E-05	0.00057015
ENSG0000022	FANCG	protein_codir	0.90560383	0.00039383	0.00270153
ENSG0000013	TNNT3	protein_codir	0.90544713	0.03087946	0.08322508

ENSG0000027	AP000569.9	lncRNA	0.9053932	0.34148018	0.49471191
ENSG0000010	RELB	protein_codir	0.90521304	0.0002921	0.00210813
ENSG0000001	UTP18	protein_codir	0.9048905	5.35E-06	7.54E-05
ENSG0000009	CMTM6	protein_codir	0.90444547	3.36E-06	5.11E-05
ENSG0000022	AC005082.12	lncRNA	0.90405436	0.03683007	0.09519432
ENSG0000014	ANXA9	protein_codir	0.90395136	0.1111068	0.21837116
ENSG0000018	INSIG1	protein_codir	0.90394306	0.01679943	0.05195971
ENSG0000026	CTB-50L17.16	lncRNA	0.90387222	0.43053894	0.5820315
ENSG0000028	KBTBD11-OT1	protein_codir	0.90375491	0.14840772	0.27036615
ENSG0000010	XYLT1	protein_codir	0.90354862	0.0003271	0.0023142
ENSG0000027	THBS1-IT1	lncRNA	0.90327866	0.0677172	0.15154342
ENSG0000007	IL4R	protein_codir	0.90307442	9.53E-05	0.00083532
ENSG0000016	SYCE2	protein_codir	0.90289199	0.1002638	0.20212025
ENSG0000020	TMEM88B	protein_codir	0.90282679	0.30348367	0.45478916
ENSG0000019	RPL39	protein_codir	0.90223852	1.95E-05	0.00022456
ENSG0000026	MIR23AHG	lncRNA	0.90203578	0.05615438	0.13121691
ENSG0000021	CXorf49	protein_codir	0.90193451	0.5925654	NA
ENSG0000013	ATP8B3	protein_codir	0.90188454	0.01466442	0.04688406
ENSG0000022	LINC02803	lncRNA	0.90119247	0.1514696	0.27424388
ENSG0000027	RNU11	lncRNA	0.8993472	0.64558461	NA
ENSG0000027	RP11-139H15	lncRNA	0.89914139	0.20784086	0.34646234
ENSG0000006	NGFR	protein_codir	0.89882819	0.03182721	0.08511123
ENSG0000016	SERPINB12	protein_codir	0.89862447	0.70110004	NA
ENSG0000018	MAPK12	protein_codir	0.89851991	0.00163757	0.00845259
ENSG0000014	NKX6-2	protein_codir	0.89813028	0.41743894	0.57011218
ENSG0000027	CTD-2013N17	lncRNA	0.89778341	0.46149503	0.61166528
ENSG0000022	ELFN1	protein_codir	0.89769636	0.00041347	0.0028045
ENSG0000016	ABHD15	protein_codir	0.89761296	0.00077313	0.00465528
ENSG0000008	NKAIN1	protein_codir	0.89758407	0.26076489	0.40801758
ENSG0000026	RP11-356J5.1	lncRNA	0.89739374	0.23497349	0.37840171
ENSG0000017	GLRX	protein_codir	0.89738216	0.00057904	0.00368332
ENSG0000027	RP11-284F21.	lncRNA	0.89724919	0.53925876	0.67849001
ENSG0000023	LINC01829	lncRNA	0.89724368	0.37042246	0.52383741
ENSG0000020	CENPW	protein_codir	0.89717603	0.0460277	0.11301054
ENSG0000025	RP11-872D17	protein_codir	0.8968146	0.12180135	0.23371915
ENSG0000018	ALKAL2	protein_codir	0.89597667	0.19064951	0.32526651
ENSG0000015	GPM6A	protein_codir	0.89571338	0.14528706	0.26622541
ENSG0000026	L3MBTL4-AS1	lncRNA	0.89557765	0.10193169	0.20469369
ENSG0000023	CT70	lncRNA	0.89522178	0.4539504	0.60434289
ENSG0000018	SLIT1	protein_codir	0.89505922	0.04958133	0.1197583
ENSG0000011	GPR75	protein_codir	0.89494653	0.00176463	0.00892332
ENSG0000026	ZNF571-AS1	lncRNA	0.89473139	0.02413712	0.06862953
ENSG0000011	PFKFB4	protein_codir	0.89456406	0.00484587	0.01979927
ENSG0000017	TYMS	protein_codir	0.89437936	0.01728041	0.05301988
ENSG0000017	RMI1	protein_codir	0.89434418	0.0054079	0.02157224
ENSG0000005	MCF2L2	protein_codir	0.89382132	0.07759925	0.16690999
ENSG0000015	ADAMTS5	protein_codir	0.89380933	0.00696033	0.02623062

ENSG0000028	RP11-383D22	lncRNA	0.89345076	0.6533835	NA
ENSG0000017	ARID3B	protein_codir	0.89341283	8.57E-05	0.00076757
ENSG0000023	CTA-126B4.7	lncRNA	0.89340025	0.56974645	0.70336553
ENSG0000024	NAIP	protein_codir	0.89334427	0.00704192	0.0264425
ENSG0000022	CTAGE4	protein_codir	0.89321646	0.26430945	0.41180408
ENSG0000012	EGLN3	protein_codir	0.89305778	0.00048652	0.00320018
ENSG0000025	AC074212.5	lncRNA	0.89239972	0.03456222	0.09072501
ENSG0000011	ATP6V1B1	protein_codir	0.89238592	0.16501625	0.29231329
ENSG0000015	ACSS1	protein_codir	0.89232218	0.00064862	0.00404609
ENSG0000010	CA9	protein_codir	0.89230773	0.28055695	0.42973508
ENSG0000001	MBTD1	protein_codir	0.89223263	9.90E-06	0.00012725
ENSG0000022	EBF2	protein_codir	0.892195	0.01123511	0.03814083
ENSG0000013	OSTF1	protein_codir	0.89217195	1.73E-06	2.93E-05
ENSG0000011	PMFBP1	protein_codir	0.89216203	0.08974044	0.18581835
ENSG0000026	LINC00922	lncRNA	0.89205435	0.22025682	0.36110248
ENSG0000020	LY6G6C	protein_codir	0.89180976	0.33521317	0.48806473
ENSG0000011	CTH	protein_codir	0.8916669	0.00142737	0.00758735
ENSG0000017	KCNE3	protein_codir	0.89159157	0.03439541	0.09045048
ENSG0000008	SIGLEC1	protein_codir	0.8915337	0.04931697	0.1192867
ENSG0000023	RP11-478K15.	lncRNA	0.89110738	0.50970011	0.65386748
ENSG0000016	TMOD4	protein_codir	0.89089657	0.14715062	0.26863161
ENSG0000025	LINC02721	lncRNA	0.89074404	0.54771126	NA
ENSG0000025	KBTBD11-OT1	lncRNA	0.89031403	0.5178281	0.66098587
ENSG0000012	HOXD10	protein_codir	0.89026809	0.34931032	0.50278551
ENSG0000026	RP11-1006G1	lncRNA	0.89012209	0.14934901	0.27158542
ENSG0000008	KDM2B	protein_codir	0.8900952	7.52E-05	0.00068749
ENSG0000027	CDC37L1-DT	lncRNA	0.88954673	0.08021277	0.17097367
ENSG0000018	LINC02610	lncRNA	0.88933527	0.11970344	0.23055851
ENSG0000012	ZFP36	protein_codir	0.88923616	0.08726618	0.18204621
ENSG0000007	LRCH4	protein_codir	0.88911443	0.00091977	0.00535723
ENSG0000000	SKAP2	protein_codir	0.88882312	2.68E-05	0.0002928
ENSG0000018	NPIPB4	protein_codir	0.88868654	0.0302871	0.08206903
ENSG0000022	CTC-529I10.2	lncRNA	0.88864194	0.14628488	0.26757538
ENSG0000025	RP11-304M2.	lncRNA	0.88779665	0.41910633	0.57172504
ENSG0000020	COL15A1	protein_codir	0.88774187	0.01457701	0.04666341
ENSG0000028	RP11-388F6.5	protein_codir	0.88759002	0.00336008	0.01479677
ENSG0000007	RARB	protein_codir	0.88693568	4.15E-06	6.07E-05
ENSG0000007	GRAMD4	protein_codir	0.88692758	1.58E-05	0.00018822
ENSG0000025	CTC-756D1.2	lncRNA	0.88680619	0.41424569	0.56733604
ENSG0000011	SLC19A2	protein_codir	0.88648441	0.12358451	0.23622174
ENSG0000028	RP11-1378G1	lncRNA	0.88619166	0.07750922	0.16675531
ENSG0000027	LL22NC03-2H	lncRNA	0.88585272	0.14937198	0.27158542
ENSG0000028	RP1-163M9.8	lncRNA	0.8857531	0.66398857	0.77758594
ENSG0000015	ASAP1	protein_codir	0.88561336	2.55E-08	7.73E-07
ENSG0000018	KCNJ14	protein_codir	0.88531155	0.01963548	0.05844872
ENSG0000010	CD40	protein_codir	0.88508472	0.00024732	0.00183974
ENSG0000016	ZNF281	protein_codir	0.88492462	5.51E-05	0.00053344

ENSG0000028	RP11-396D4.2	lncRNA	0.88452612	0.136122	0.25345083
ENSG0000011	COL12A1	protein_codir	0.88441771	0.03814077	0.09774966
ENSG0000027	RP11-347I19.8	lncRNA	0.8843766	0.70548463	NA
ENSG0000026	LA16c-306A4.	lncRNA	0.88430213	0.16827528	0.29669731
ENSG0000014	RPS2	protein_codir	0.88393237	2.96E-05	0.00031842
ENSG0000008	DOCK9	protein_codir	0.88353235	2.06E-05	0.00023503
ENSG0000025	RP11-847H18	lncRNA	0.88348581	0.31242727	0.46421223
ENSG0000024	RORA-AS1	lncRNA	0.88318796	0.22695601	0.36912761
ENSG0000024	RP5-940J5.6	lncRNA	0.88281642	0.03177415	0.08503073
ENSG0000024	NPIPB5	protein_codir	0.88268977	0.03326996	0.08810318
ENSG0000026	CTB-25B13.9	lncRNA	0.88226866	0.50010915	0.64546797
ENSG0000025	RP11-323I15.5	lncRNA	0.88219417	0.58839004	0.71854503
ENSG0000016	DNAAF3	protein_codir	0.8819353	0.15873332	0.28404369
ENSG0000015	TSPAN18	protein_codir	0.88151938	0.00119988	0.00660964
ENSG0000013	KCP	protein_codir	0.88148924	0.06696817	0.15030658
ENSG0000011	SCNN1A	protein_codir	0.88121141	0.02206388	0.06398619
ENSG0000027	RP11-2C24.7	lncRNA	0.88080875	0.28581064	0.43536785
ENSG0000019	SIRPA	protein_codir	0.88036923	0.00128243	0.00697807
ENSG0000018	JAG2	protein_codir	0.87972299	0.00078048	0.00468908
ENSG0000008	RFX3	protein_codir	0.87944357	8.18E-05	0.00073789
ENSG0000028	HCP5B	lncRNA	0.87920913	0.26633854	0.41390146
ENSG0000019	IL27	protein_codir	0.87857688	0.15494148	0.27901185
ENSG0000018	SPDYE4	protein_codir	0.87854662	0.55202469	NA
ENSG0000013	RAB20	protein_codir	0.87836675	0.00022222	0.0016836
ENSG0000023	LINC00398	lncRNA	0.87798855	0.23252094	0.37552499
ENSG0000024	BACH1-IT1	lncRNA	0.87776262	0.0791803	0.16938733
ENSG0000018	RAP2B	protein_codir	0.87718635	0.00084838	0.00502082
ENSG0000017	DCP2	protein_codir	0.87696081	0.00019864	0.00153679
ENSG0000027	RP11-894J14.	protein_codir	0.87695366	0.30458812	0.45604259
ENSG0000013	IQCN	protein_codir	0.87694864	0.02474339	0.07002894
ENSG0000027	LINC02371	lncRNA	0.87683497	0.4978038	0.64351497
ENSG0000023	LINC01780	lncRNA	0.87670464	0.60050538	NA
ENSG0000016	INTS6L	protein_codir	0.87632634	0.00458099	0.01895495
ENSG0000016	CDC20B	protein_codir	0.87616508	0.51728974	NA
ENSG0000022	PCAT6	lncRNA	0.87595323	0.04992217	0.12040258
ENSG0000024	RP11-34P13.1	lncRNA	0.87585157	0.00526679	0.02112835
ENSG0000006	PACC1	protein_codir	0.87565973	0.00045762	0.00304348
ENSG0000028	LINC02887	lncRNA	0.87546049	0.04575738	0.11251949
ENSG0000017	SLCO4C1	protein_codir	0.87516935	0.05423922	0.12795197
ENSG0000012	AC105760.3	lncRNA	0.87511972	0.61353104	0.73795791
ENSG0000017	GPR22	protein_codir	0.87504046	0.76009482	0.84830366
ENSG0000013	HS6ST1	protein_codir	0.87476119	1.77E-05	0.00020702
ENSG0000014	PADI1	protein_codir	0.87439118	0.3766398	0.53021363
ENSG0000027	RP11-142E9.1	lncRNA	0.87414663	0.01834202	0.05547597
ENSG0000025	CRTC3-AS1	lncRNA	0.8740123	0.38547449	0.53866381
ENSG0000017	GPR4	protein_codir	0.8740043	0.02536669	0.07140523
ENSG0000024	RP5-1157M23	lncRNA	0.87396737	0.14601781	0.26724616

ENSG0000028	RP1-278C19.9	lncRNA	0.87378713	0.30417103	0.4555018
ENSG0000027	RP1-309F20.4	lncRNA	0.8735573	0.57391285	0.70667705
ENSG0000014	PAMR1	protein_codir	0.87328927	0.2684617	0.41625412
ENSG0000016	RPL22L1	protein_codir	0.87321841	0.00385085	0.01650037
ENSG0000026	HSPB9	protein_codir	0.87290301	0.49942147	0.64485162
ENSG0000006	HES2	protein_codir	0.87280622	0.28180915	0.43124653
ENSG0000012	PZP	protein_codir	0.87270175	0.11768925	0.22767928
ENSG0000013	KANK4	protein_codir	0.87259856	0.14987492	0.27217305
ENSG0000018	LINC00471	lncRNA	0.87256351	0.12348325	0.23610989
ENSG0000011	ZNF541	protein_codir	0.87253416	0.0796655	0.17010909
ENSG0000014	FCN3	protein_codir	0.87244336	0.11166446	0.21912794
ENSG0000018	DPYD	protein_codir	0.87235669	0.00093123	0.00540894
ENSG0000017	RBKS	protein_codir	0.87185471	0.0418979	0.10528796
ENSG0000016	PROK2	protein_codir	0.87173448	0.19633874	0.33220765
ENSG0000010	SLC5A4	protein_codir	0.87133144	0.01723898	0.05293982
ENSG0000013	MAPK8IP3	protein_codir	0.870308	0.00594893	0.02318134
ENSG0000022	RP11-112J3.1	lncRNA	0.87025782	0.23130632	0.37404316
ENSG0000017	PWWP2B	protein_codir	0.87023986	9.20E-05	0.00081005
ENSG0000023	RP4-738P15.1	lncRNA	0.8700132	0.56747526	0.70130274
ENSG0000016	CDCP1	protein_codir	0.8698256	0.04530027	0.11184171
ENSG0000015	FOXO1	protein_codir	0.86961051	0.00039918	0.00272906
ENSG0000020	ADGRG1	protein_codir	0.86896839	0.00780107	0.02864082
ENSG0000009	FLT3LG	protein_codir	0.86827964	0.00018092	0.00142646
ENSG0000026	RP11-600F24.	lncRNA	0.86805249	0.02684331	0.07461108
ENSG0000013	ARHGEF16	protein_codir	0.86796989	0.02931239	0.080044
ENSG0000026	PTOV1-AS2	lncRNA	0.86779957	0.03007105	0.08161411
ENSG0000028	AC000050.1	lncRNA	0.86760926	0.02887755	0.07915819
ENSG0000015	GLT1D1	protein_codir	0.86722287	0.13045098	0.24577463
ENSG0000017	C1QTNF4	protein_codir	0.86716784	0.05709751	0.1328028
ENSG0000022	LINC01117	lncRNA	0.86657227	0.12351263	0.23614253
ENSG0000018	DEFB132	protein_codir	0.8665195	0.6796519	0.78946865
ENSG0000028	RP11-758P17.	lncRNA	0.86604843	0.58980971	0.71957879
ENSG0000025	AC140725.4	lncRNA	0.86581149	0.60351525	NA
ENSG0000024	PWP2	protein_codir	0.86573941	0.29155307	0.44136453
ENSG0000017	DNHD1	protein_codir	0.86570605	0.00665917	0.02529588
ENSG0000011	FHL2	protein_codir	0.86502725	0.00824446	0.029907
ENSG0000024	RP11-506M13	lncRNA	0.86462728	0.48901796	0.63599736
ENSG0000013	GSC	protein_codir	0.86457883	0.03345611	0.08849434
ENSG0000025	RP11-1100L3.	lncRNA	0.8644254	0.19003877	0.32450514
ENSG0000021	FAM209B	protein_codir	0.86434712	0.07390547	0.16125042
ENSG0000025	RP11-326C3.1	lncRNA	0.86415382	0.03321167	0.08797412
ENSG0000019	ARRDC1	protein_codir	0.86388213	0.00517975	0.02087012
ENSG0000015	GRM1	protein_codir	0.86370673	0.15875533	0.28406466
ENSG0000018	DNAH2	protein_codir	0.86358803	0.05696464	0.13263271
ENSG0000018	SHISA7	protein_codir	0.86355776	0.325336	0.477591
ENSG0000010	PDGFRL	protein_codir	0.86348072	0.0763471	0.16506513
ENSG0000026	CTD-2105E13.	protein_codir	0.86338533	0.60537029	NA

ENSG0000022	FTCDNL1	protein_codir	0.86303369	0.08573542	0.17976293
ENSG0000016	STK32C	protein_codir	0.86283025	9.31E-06	0.000121
ENSG0000025	RP11-521C20.	lncRNA	0.86276439	0.47741136	0.62540797
ENSG0000022	RP11-276H7.3	lncRNA	0.86258746	0.62656319	0.74822411
ENSG0000001	ISL1	protein_codir	0.86243313	0.25849191	0.40554107
ENSG0000023	CATIP-AS2	lncRNA	0.86207447	0.62735741	0.7487173
ENSG0000025	CAPNS2	protein_codir	0.86142856	0.40623881	0.55919841
ENSG0000017	RP11-262H14	lncRNA	0.86101286	0.00305381	0.01369877
ENSG0000010	CAPS	protein_codir	0.86067075	0.01317334	0.0430895
ENSG0000016	OR2C1	protein_codir	0.86060093	0.49540603	0.64134589
ENSG0000027	LA16c-329F2.	lncRNA	0.86023775	0.24675061	0.39199171
ENSG0000027	CTA-992D9.1	lncRNA	0.86021132	0.56533188	NA
ENSG0000012	RNASE1	protein_codir	0.86013081	0.01525394	0.04832761
ENSG0000026	RP11-381P6.1	lncRNA	0.86005147	0.43928993	0.59032528
ENSG0000016	LDHC	protein_codir	0.85995244	0.49496848	0.64096885
ENSG0000008	ZNF586	protein_codir	0.85992784	0.00050886	0.00331481
ENSG0000019	NPIPB13	protein_codir	0.85985742	0.07142736	0.15734149
ENSG0000003	HOXC8	protein_codir	0.85974731	0.10081179	0.20297577
ENSG0000027	RP11-494O16	lncRNA	0.85970473	0.57628858	NA
ENSG0000019	ZNF675	protein_codir	0.85953264	0.03503076	0.09161534
ENSG0000011	XRN1	protein_codir	0.85944284	1.78E-07	4.09E-06
ENSG0000015	TPPP3	protein_codir	0.8592289	0.02789749	0.07695282
ENSG0000027	ADRA2B	protein_codir	0.85876732	0.11180845	0.219316
ENSG0000020	TP53TG3C	protein_codir	0.85850961	0.63485883	0.75476654
ENSG0000027	RP11-153I24.	lncRNA	0.85849166	0.56317845	0.69765827
ENSG0000010	CEP152	protein_codir	0.85839776	0.00075832	0.00458811
ENSG0000023	SNHG7	lncRNA	0.85795095	0.00060354	0.00381235
ENSG0000007	GUCY2C	protein_codir	0.85773961	0.2696636	0.41751145
ENSG0000015	IFNAR2	protein_codir	0.85770847	8.30E-05	0.00074733
ENSG0000028	SMIM41	protein_codir	0.85747444	0.22168477	0.36269005
ENSG0000023	BHLHE40-AS1	lncRNA	0.8573527	0.02198304	0.06379559
ENSG0000006	ACSM2B	protein_codir	0.85695199	0.34826478	0.50159427
ENSG0000028	RP11-112B10.	protein_codir	0.85687681	0.45032308	0.60105175
ENSG0000027	RP11-717F1.2	lncRNA	0.85675277	0.18505675	0.31818345
ENSG0000025	EBLN2	protein_codir	0.85674651	0.04662834	0.11411261
ENSG0000010	DOT1L	protein_codir	0.85649319	2.28E-05	0.0002556
ENSG0000019	PRC1	protein_codir	0.85637252	2.75E-05	0.00029892
ENSG0000023	LINC-PINT	lncRNA	0.85579395	0.02185329	0.06349547
ENSG0000026	RP11-196G11	lncRNA	0.85452041	0.35134867	0.50490377
ENSG0000011	GBP1	protein_codir	0.85428481	0.00151789	0.00795675
ENSG0000026	AC005262.2	lncRNA	0.85406925	0.42879382	0.5803825
ENSG0000014	MCL1	protein_codir	0.85398052	0.00486101	0.01984057
ENSG0000026	HEXA-AS1	lncRNA	0.8536824	0.22525562	0.36703205
ENSG0000018	OR10H1	protein_codir	0.85359195	0.77471562	NA
ENSG0000007	SPAG6	protein_codir	0.85350775	0.30291212	0.45420374
ENSG0000011	KLHL24	protein_codir	0.85334673	7.84E-05	0.00071155
ENSG0000026	RP11-165M1.	lncRNA	0.85325581	0.61115943	NA

ENSG0000028	RP11-875O11	lncRNA	0.85302435	0.13187596	0.24788847
ENSG0000022	RP11-498J9.2	lncRNA	0.85294652	0.31670643	0.46842839
ENSG0000007	NAALAD2	protein_codir	0.85293998	0.05702541	0.13269097
ENSG0000014	LINS1	protein_codir	0.85285446	4.80E-05	0.00047295
ENSG0000027	CTD-2562G15	lncRNA	0.85240939	0.33382007	0.48672915
ENSG0000026	ANKRD20A1	protein_codir	0.85209481	0.11244973	0.22014895
ENSG0000021	ARHGAP27P1	lncRNA	0.85193253	0.0112417	0.03815381
ENSG0000022	TAX1BP1-AS1	lncRNA	0.85168254	0.7725604	NA
ENSG0000027	RP11-427L15.	lncRNA	0.85145721	0.02410415	0.06854991
ENSG0000028	LINC01002	lncRNA	0.8511625	0.00248646	0.01164465
ENSG0000016	ARL5B	protein_codir	0.85098053	0.00677694	0.02565856
ENSG0000026	CTD-2542L18.	lncRNA	0.85097747	0.15465054	0.27861508
ENSG0000027	RP11-180C16.	lncRNA	0.8509675	0.12498378	0.23825329
ENSG0000018	FBLL1	protein_codir	0.85094835	0.27969823	0.42868127
ENSG0000023	AL163953.3	lncRNA	0.85025856	0.02984508	0.08112598
ENSG0000015	PABPC3	protein_codir	0.84997632	0.01279577	0.04210378
ENSG0000016	GCSAML	protein_codir	0.84982184	0.07886336	0.16887937
ENSG0000011	DNAH1	protein_codir	0.8495107	0.0032231	0.01431223
ENSG0000020	MAFB	protein_codir	0.84945759	0.00358464	0.01557219
ENSG0000023	ELOVL2-AS1	lncRNA	0.84926229	0.56086345	0.6957891
ENSG0000017	OPLAH	protein_codir	0.8491042	0.00163736	0.00845259
ENSG0000017	MFSD4A	protein_codir	0.84907655	0.02691952	0.07474787
ENSG0000006	DIP2B	protein_codir	0.84872505	0.00017952	0.0014167
ENSG0000010	DDX25	protein_codir	0.84847214	0.13234999	0.24861473
ENSG0000002	IFNGR1	protein_codir	0.84818285	7.47E-05	0.00068464
ENSG0000021	SCART1	protein_codir	0.84817909	0.00704431	0.02644607
ENSG0000017	CNIH2	protein_codir	0.84789272	0.02347139	0.06711666
ENSG0000019	AFDN-DT	lncRNA	0.8477353	0.1868544	0.32035496
ENSG0000016	ANGPTL4	protein_codir	0.84766906	0.19183993	0.32655114
ENSG0000016	DTX3L	protein_codir	0.84708025	0.00094558	0.00547871
ENSG0000027	RP5-1112D6.8	lncRNA	0.84705794	0.07353318	0.16071744
ENSG0000016	ADGRL4	protein_codir	0.84668499	0.00369906	0.01595627
ENSG0000018	SLITRK2	protein_codir	0.84661688	0.07527039	0.16337781
ENSG0000020	C5orf60	lncRNA	0.84656492	0.67563564	NA
ENSG0000019	S100A10	protein_codir	0.84633057	0.00025879	0.00190861
ENSG0000014	CNBD2	protein_codir	0.8459155	0.00118996	0.00656795
ENSG0000018	LINC00334	lncRNA	0.84580856	0.41680448	0.56949616
ENSG0000010	SUSD6	protein_codir	0.8456579	5.28E-10	2.96E-08
ENSG0000018	TDRP	protein_codir	0.84562692	0.01825286	0.05526077
ENSG0000024	CTB-161M19.	lncRNA	0.84536096	0.36451217	0.51845235
ENSG0000027	RP11-467P9.1	lncRNA	0.84512983	0.4630429	0.61298257
ENSG0000027	RP11-500G10	lncRNA	0.84498298	0.50725978	0.65171249
ENSG0000021	CAPN14	protein_codir	0.84461611	0.06246949	0.14245437
ENSG0000016	SCNN1D	protein_codir	0.84432946	0.04409812	0.10943308
ENSG0000027	RP11-305O6.4	lncRNA	0.84412404	0.14778065	0.26941925
ENSG0000021	GPC2	protein_codir	0.84394483	0.05488263	0.12903118
ENSG0000014	PGD	protein_codir	0.84367361	0.00105206	0.00596303

ENSG0000017	SYCE1	protein_codir	0.84345403	0.2329322	0.37597371
ENSG0000016	F2RL2	protein_codir	0.84342862	0.09797657	0.19874507
ENSG0000019	CCDC180	protein_codir	0.84334102	0.01258327	0.04155309
ENSG0000021	RSC1A1	protein_codir	0.84319717	0.20416528	0.34169818
ENSG0000010	ISM1	protein_codir	0.84318127	0.01940256	0.05797886
ENSG0000027	RP11-1057B6.	lncRNA	0.84307772	0.44043056	0.59146653
ENSG0000016	ITGA2	protein_codir	0.8430065	0.00702266	0.0264007
ENSG0000017	GPR152	protein_codir	0.8426842	0.60583338	NA
ENSG0000023	RPS18	protein_codir	0.8426231	3.54E-07	7.47E-06
ENSG0000025	RP11-603J24.	lncRNA	0.84157458	0.54268256	NA
ENSG0000028	LINC02666	lncRNA	0.84153524	0.25252065	0.39873202
ENSG0000026	HEATR6-DT	lncRNA	0.84150022	0.25304569	0.39930995
ENSG0000013	RBM38	protein_codir	0.84125893	0.00049534	0.0032451
ENSG0000016	ERAP1	protein_codir	0.84123519	3.27E-05	0.00034349
ENSG0000015	NBL1	protein_codir	0.84111318	0.00520108	0.02093127
ENSG0000021	KANSL1-AS1	lncRNA	0.84053277	0.0868312	0.18148149
ENSG0000027	H2AC8	protein_codir	0.84045561	0.07298945	0.15985796
ENSG0000026	RP11-212I21.4	lncRNA	0.84042985	0.14654594	0.26799967
ENSG0000012	USP6	protein_codir	0.84034183	0.27182713	0.4198025
ENSG0000025	RP11-468E2.5	lncRNA	0.84014641	0.17655935	0.30703282
ENSG0000013	BORA	protein_codir	0.8399295	0.0408522	0.10314749
ENSG0000016	PLEKHA7	protein_codir	0.83979482	0.0018603	0.00927968
ENSG0000022	WARS2-IT1	lncRNA	0.83974418	0.16783486	0.29618547
ENSG0000016	CDCA7L	protein_codir	0.83941485	0.04739792	0.11556591
ENSG0000018	ZNF721	protein_codir	0.8394084	2.59E-06	4.07E-05
ENSG0000015	POU4F1	protein_codir	0.83873007	0.21711122	0.35736663
ENSG0000018	SLC51B	protein_codir	0.83795902	0.6798792	0.78953785
ENSG0000025	NR4A1AS	lncRNA	0.83778881	0.24698966	0.39221838
ENSG0000007	TESK2	protein_codir	0.837236	5.69E-05	0.00054864
ENSG0000017	CERS6	protein_codir	0.83712322	0.00032928	0.00232663
ENSG0000015	PRKCA	protein_codir	0.83654061	0.00191187	0.00947996
ENSG0000027	LINC02266	lncRNA	0.83607328	0.5765522	NA
ENSG0000022	XXbac-BPG18	lncRNA	0.83589613	0.34473456	0.49783377
ENSG0000026	CTD-2349P21	lncRNA	0.83585583	0.40198588	0.5549771
ENSG0000026	CCER2	protein_codir	0.83562546	0.21342792	0.35319827
ENSG0000009	TFAP4	protein_codir	0.83559942	0.00122973	0.00674315
ENSG0000025	RP11-727A23	lncRNA	0.83544497	0.13389583	0.25060668
ENSG0000015	NMNAT2	protein_codir	0.83538532	0.03152341	0.0845153
ENSG0000025	LINC00648	lncRNA	0.83531931	0.37781307	0.53154019
ENSG0000017	ANKRD36C	protein_codir	0.83403117	0.00135236	0.00726931
ENSG0000016	DAGLB	protein_codir	0.83397852	0.00182617	0.00914743
ENSG0000026	RP11-296K13.	lncRNA	0.8336376	0.38986128	0.54287716
ENSG0000014	CACNA1B	protein_codir	0.83331873	0.22041808	0.36126371
ENSG0000026	RP11-797A18	lncRNA	0.83307138	0.13268564	0.24905045
ENSG0000028	RP11-325J6.2	lncRNA	0.83300041	0.3758407	0.5293585
ENSG0000019	AVPR1B	protein_codir	0.83280332	0.59259398	0.72138241
ENSG0000028	RP11-712C19.	lncRNA	0.83269296	0.21419337	0.35406194

ENSG0000006	ZMYND12	protein_codir	0.83240728	0.02207162	0.0639952
ENSG0000025	RP11-411B6.6	protein_codir	0.83208401	0.00548018	0.02178821
ENSG0000022	LINC01068	lncRNA	0.83201307	0.55376411	0.68998803
ENSG0000012	SPATS2	protein_codir	0.83194633	0.00038184	0.00263255
ENSG0000017	MLLT3	protein_codir	0.8317946	1.03E-05	0.00013177
ENSG0000025	SH3TC2-DT	lncRNA	0.83165002	0.60158516	0.72868747
ENSG0000025	RP11-802E16.	lncRNA	0.83123253	0.1167207	0.22632867
ENSG0000018	SREBF2-AS1	lncRNA	0.83112576	0.02624756	0.07329467
ENSG0000000	KDM7A	protein_codir	0.83111739	5.31E-06	7.49E-05
ENSG0000016	LRRC2	protein_codir	0.8309841	0.05488006	0.12903118
ENSG0000018	OAF	protein_codir	0.83072473	0.011241	0.03815381
ENSG0000010	SMCHD1	protein_codir	0.83059503	1.38E-05	0.00016897
ENSG0000016	SYNPO2L	protein_codir	0.83056965	0.19774274	0.33394402
ENSG0000028	AC007326.11	lncRNA	0.83053503	0.62864328	0.74980038
ENSG0000022	SZT2-AS1	lncRNA	0.83051633	0.5816165	0.71295259
ENSG0000016	LDB2	protein_codir	0.83046497	0.0008326	0.00494761
ENSG0000013	DGLUCY	protein_codir	0.8302389	9.89E-05	0.0008606
ENSG0000026	RP11-723O4.9	lncRNA	0.83019077	0.23565049	0.37927083
ENSG0000016	DEFB1	protein_codir	0.82991764	0.19861439	0.33488471
ENSG0000024	CTC-537E7.3	lncRNA	0.8299021	0.60619138	0.73254314
ENSG0000025	RP11-122C5.3	lncRNA	0.82989631	0.34077737	0.49387506
ENSG0000020	ACOXL-AS1	lncRNA	0.82959833	0.12148217	0.23328496
ENSG0000027	RP11-57G10.8	lncRNA	0.82954912	0.50458616	0.64922167
ENSG0000026	LINC01569	lncRNA	0.82929804	0.05277269	0.12529716
ENSG0000016	MOBP	protein_codir	0.829135	0.18267106	0.31507755
ENSG0000018	LILRA5	protein_codir	0.82837762	0.04975387	0.12009106
ENSG0000022	LINC01535	lncRNA	0.82826044	0.20764355	0.34615614
ENSG0000012	DNAJB9	protein_codir	0.82813848	9.65E-05	0.00084253
ENSG0000011	TIMELESS	protein_codir	0.82794102	0.00548965	0.02181013
ENSG0000025	RP11-343C2.7	protein_codir	0.82794029	0.60082032	0.72805937
ENSG0000018	C19orf71	protein_codir	0.82718291	0.05012498	0.12075351
ENSG0000020	LINC01257	lncRNA	0.82713667	0.67637768	NA
ENSG0000007	SEC31B	protein_codir	0.82688329	0.02360127	0.06740662
ENSG0000014	UNC45B	protein_codir	0.82681945	0.21549161	0.35565451
ENSG0000027	GOLGA6L10	protein_codir	0.82681045	0.07659599	0.16539258
ENSG0000022	LINC02794	lncRNA	0.82670615	0.79992497	NA
ENSG0000010	UPB1	protein_codir	0.82666689	0.05654623	0.13190936
ENSG0000026	CTD-2540B15	lncRNA	0.82621849	0.11926202	0.22994883
ENSG0000026	RP11-676J12.4	lncRNA	0.82615652	0.22920267	0.37173045
ENSG0000011	HSD11B1	protein_codir	0.82584991	0.06828483	0.15227087
ENSG0000027	RP11-474N24	lncRNA	0.82547587	0.23720616	0.38130801
ENSG0000018	CXorf38	protein_codir	0.82537888	4.21E-06	6.13E-05
ENSG0000013	MATN3	protein_codir	0.82485723	0.09932353	0.20079899
ENSG0000027	RP4-563E14.1	lncRNA	0.82460146	0.12835314	0.24284928
ENSG0000025	RP11-887P2.5	lncRNA	0.82425916	0.22993039	0.37232589
ENSG0000007	PTPN18	protein_codir	0.82149116	0.00056244	0.00359681
ENSG0000017	EGFL7	protein_codir	0.82145762	0.01175291	0.03950233

ENSG0000028	RP11-426A21	lncRNA	0.82127453	0.61538322	NA
ENSG0000026	AC005786.7	lncRNA	0.82046571	0.06575579	0.14828338
ENSG0000014	MMEL1	protein_codir	0.82037524	0.06813447	0.15207063
ENSG0000023	AC093484.4	lncRNA	0.81998042	0.17301688	0.30271927
ENSG0000013	DDX60	protein_codir	0.81995522	0.00414041	0.01752545
ENSG0000017	AZU1	protein_codir	0.81987053	0.12285809	0.23524024
ENSG0000025	RP11-762I7.4	lncRNA	0.819576	0.25044933	0.39636778
ENSG0000023	ENO1-AS1	lncRNA	0.81957194	0.36070467	0.51460036
ENSG0000010	PSME2	protein_codir	0.81944416	0.00023469	0.00175811
ENSG0000015	PDCD4	protein_codir	0.81929029	1.81E-08	5.78E-07
ENSG0000018	NUDT14	protein_codir	0.81926375	0.00088142	0.00517864
ENSG0000013	MYO7A	protein_codir	0.81921302	0.01732873	0.05312092
ENSG0000023	LINC01275	lncRNA	0.81879281	0.336999	0.48994253
ENSG0000018	ZNF732	protein_codir	0.81854567	0.33585279	0.48876411
ENSG0000025	RP11-347C18.	lncRNA	0.81835408	0.0561511	0.13121691
ENSG0000027	RP3-337H4.9	lncRNA	0.81822781	0.18025213	0.31176429
ENSG0000021	TSPAN4	protein_codir	0.8179681	8.27E-07	1.56E-05
ENSG0000028	RP13-20L14.1	lncRNA	0.81779666	0.51703815	0.66022163
ENSG0000011	MFSD1	protein_codir	0.81751289	0.00069607	0.00428282
ENSG0000027	AL133245.2	lncRNA	0.81718686	0.16833862	0.29675215
ENSG0000012	KRT36	protein_codir	0.81713857	0.26285649	0.41015175
ENSG0000026	AC002550.5	lncRNA	0.81695605	0.52847694	0.66993451
ENSG0000018	SNN	protein_codir	0.81691848	0.00068178	0.00421272
ENSG0000018	FSIP2	protein_codir	0.81666088	0.07751961	0.16676467
ENSG0000017	FJX1	protein_codir	0.81621414	0.04028443	0.10205979
ENSG0000017	FAM170B	protein_codir	0.81589654	0.59956209	NA
ENSG0000010	ARHGEF18	protein_codir	0.81588821	0.00340764	0.01494903
ENSG0000011	ST3GAL5	protein_codir	0.81540294	0.00202086	0.00989874
ENSG0000018	CHST15	protein_codir	0.81479022	0.00450607	0.01869296
ENSG0000018	CLN8	protein_codir	0.81473817	0.00040872	0.00278396
ENSG0000013	PLVAP	protein_codir	0.81449996	0.00121693	0.00668755
ENSG0000023	AC009505.2	lncRNA	0.8142324	0.35462031	0.50815113
ENSG0000028	RP11-253D19	lncRNA	0.81398823	0.46078355	0.61119156
ENSG0000026	TEN1-CDK3	protein_codir	0.81395162	0.02311191	0.06635665
ENSG0000010	LIPA	protein_codir	0.81384837	0.01988957	0.05905084
ENSG0000018	PRR36	protein_codir	0.81357958	0.06653187	0.14961817
ENSG0000010	TUBB4A	protein_codir	0.81332542	0.1970603	0.33310449
ENSG0000026	RP11-258F1.1	lncRNA	0.81293537	0.01294924	0.04249738
ENSG0000015	ZNF776	protein_codir	0.81285508	5.50E-05	0.00053287
ENSG0000016	GUSB	protein_codir	0.81275357	6.43E-07	1.25E-05
ENSG0000005	IL17RB	protein_codir	0.81275195	0.04537097	0.11190804
ENSG0000014	MTF2	protein_codir	0.81260295	0.00048702	0.00320195
ENSG0000021	PRR22	protein_codir	0.81255391	0.09689215	0.19699383
ENSG0000024	LINC02082	lncRNA	0.81248567	0.53496268	0.67536654
ENSG0000015	CACNA1D	protein_codir	0.81239016	0.03729428	0.09612429
ENSG0000021	RPS29	protein_codir	0.81215705	0.00012601	0.0010588
ENSG0000027	KCNE1B	protein_codir	0.81200892	0.44728152	0.59810775

ENSG0000013	RB1	protein_codir	0.81165671	3.43E-05	0.0003568
ENSG0000014	HDC	protein_codir	0.81148746	0.17323552	0.30292909
ENSG0000017	IL20RB	protein_codir	0.81143149	0.03475978	0.09107049
ENSG0000028	RP4-604G5.4	lncRNA	0.81141754	0.4837826	0.63114994
ENSG0000016	CITED2	protein_codir	0.81116928	0.01116207	0.03796286
ENSG0000008	CACNA1S	protein_codir	0.81056535	0.60401582	0.73061596
ENSG0000005	LAMA3	protein_codir	0.81042546	0.00143998	0.00763531
ENSG0000020	H1-10-AS1	lncRNA	0.81013653	0.03120536	0.08390693
ENSG0000009	SLC7A4	protein_codir	0.80977108	0.28279113	0.43224632
ENSG0000027	RP11-489E7.4	lncRNA	0.80970174	0.18543648	0.31863603
ENSG0000026	CTD-3222D19	lncRNA	0.80966865	0.22658114	0.36864815
ENSG0000019	HOXC6	protein_codir	0.80934691	0.07573287	0.16406728
ENSG0000015	KIT	protein_codir	0.80921902	0.0214817	0.06265137
ENSG0000017	PRKCE	protein_codir	0.80892328	1.85E-05	0.00021489
ENSG0000017	TMC7	protein_codir	0.80888275	0.00328143	0.01452223
ENSG0000025	RP11-505E24.	lncRNA	0.808832	0.62567557	NA
ENSG0000019	ATP2A1	protein_codir	0.8085853	0.07794677	0.16743581
ENSG0000018	FOXL2	protein_codir	0.80848695	0.39392591	0.54688281
ENSG0000017	FBXO46	protein_codir	0.80818369	5.35E-05	0.0005206
ENSG0000019	ZNF460	protein_codir	0.80811559	0.01420086	0.0457142
ENSG0000016	SPDYA	protein_codir	0.80760886	0.12720891	0.2413125
ENSG0000018	RELL1	protein_codir	0.80755758	8.59E-05	0.0007689
ENSG0000028	RP11-348N12	lncRNA	0.80742	0.10923913	0.21560559
ENSG0000026	NBAT1	lncRNA	0.80718962	0.45959287	0.61008099
ENSG0000012	ATP1B2	protein_codir	0.80711087	0.08096656	0.17222044
ENSG0000027	CTB-113D17.1	lncRNA	0.80691057	0.31880565	0.47087766
ENSG0000025	RP11-468E2.2	protein_codir	0.80664738	0.05238985	0.1246024
ENSG0000028	RP11-571I18.(	lncRNA	0.80655207	0.37956426	0.53294519
ENSG0000014	EML4	protein_codir	0.80633894	9.19E-05	0.00081005
ENSG0000012	BAIAP2L2	protein_codir	0.80606513	0.10172693	0.20442108
ENSG0000013	JUND	protein_codir	0.80600884	0.03600146	0.09349886
ENSG0000013	RNF17	protein_codir	0.80583867	0.60761071	0.73352016
ENSG0000023	AC009495.3	lncRNA	0.80575857	0.69338121	0.79876498
ENSG0000013	TMEM243	protein_codir	0.80567324	0.0001146	0.00097726
ENSG0000012	BCL2L12	protein_codir	0.80526906	0.0002525	0.00187001
ENSG0000019	SNHG12	lncRNA	0.80519883	0.02791466	0.07699249
ENSG0000027	H4C12	protein_codir	0.80448116	0.0851187	0.17875444
ENSG0000015	OR14K1	protein_codir	0.8041163	0.78753727	NA
ENSG0000004	MAGEC2	protein_codir	0.80411628	0.78855494	NA
ENSG0000023	AC093627.7	lncRNA	0.80411627	0.78889847	NA
ENSG0000022	RP11-418J17.	lncRNA	0.80411627	0.78897965	NA
ENSG0000026	LINC02675	lncRNA	0.80411625	0.78983493	NA
ENSG0000025	LINC02318	lncRNA	0.80411625	0.78985155	NA
ENSG0000027	ENSG0000027	protein_codir	0.80411624	0.79023518	NA
ENSG0000027	ENSG0000027	protein_codir	0.80411624	0.79023518	NA
ENSG0000025	RP1-276E15.1	lncRNA	0.80411623	0.79048106	NA
ENSG0000022	AFF2-IT1	lncRNA	0.80411623	0.7907214	NA

ENSG0000023	LINC01056	lncRNA	0.80411622	0.79105071	NA
ENSG0000027	GGTLC3	protein_codir	0.80411622	0.79116424	NA
ENSG0000027	RP11-314C9.2	lncRNA	0.80411621	0.79130398	NA
ENSG0000023	AP002856.4	lncRNA	0.80411621	0.79170081	NA
ENSG0000027	RP11-115D19	lncRNA	0.8041162	0.79190019	NA
ENSG0000028	RP11-22E12.1	lncRNA	0.80411618	0.79286709	NA
ENSG0000011	IL5	protein_codir	0.80411616	0.79346378	NA
ENSG0000017	HIGD2B	protein_codir	0.80411616	0.79368805	NA
ENSG0000023	LINC02558	lncRNA	0.80411615	0.79414149	NA
ENSG0000023	LINC01293	lncRNA	0.80411615	0.79422526	NA
ENSG0000025	LINC00911	lncRNA	0.80411613	0.79470668	NA
ENSG0000025	LINC02404	lncRNA	0.80411613	0.79477275	NA
ENSG0000010	CST4	protein_codir	0.80411613	0.79477275	NA
ENSG0000025	RP11-364L4.3	lncRNA	0.80411611	0.79554687	NA
ENSG0000020	RP11-812E19.	lncRNA	0.80411609	0.79653309	NA
ENSG0000025	CTC-308K20.2	lncRNA	0.80411609	0.79658916	NA
ENSG0000023	LINC01251	lncRNA	0.80411607	0.79745579	NA
ENSG0000019	AC107218.3	lncRNA	0.80411607	0.79754399	NA
ENSG0000014	SHISAL2B	protein_codir	0.80411606	0.79756759	NA
ENSG0000019	EPS8L3	protein_codir	0.80411606	0.79788505	NA
ENSG0000018	AQP12A	protein_codir	0.80411606	0.79788505	NA
ENSG0000028	RP11-314L11.	lncRNA	0.80411605	0.79826015	NA
ENSG0000028	RP11-264M12	lncRNA	0.80411605	0.79831937	NA
ENSG0000024	RP11-155L15.	lncRNA	0.80411604	0.79848537	NA
ENSG0000025	LINC02489	lncRNA	0.80411604	0.79870827	NA
ENSG0000017	KRT74	protein_codir	0.80411603	0.79876508	NA
ENSG0000028	RP11-1077A2	lncRNA	0.80411603	0.79876508	NA
ENSG0000026	CTD-3222D19	lncRNA	0.80411602	0.79928353	NA
ENSG0000024	LINC00635	lncRNA	0.80411602	0.79938423	NA
ENSG0000022	ABCC5-AS1	lncRNA	0.80411601	0.79972962	NA
ENSG0000024	CTB-57H20.1	lncRNA	0.80411601	0.79975839	NA
ENSG0000028	RP11-346I3.7	lncRNA	0.80411601	0.79986415	NA
ENSG0000025	RP11-4O3.1	lncRNA	0.804116	0.80003697	NA
ENSG0000023	SMYD3-IT1	lncRNA	0.804116	0.80005463	NA
ENSG0000023	RP4-760C5.3	lncRNA	0.804116	0.80023239	NA
ENSG0000025	CTD-2017C7.1	lncRNA	0.804116	0.8002581	NA
ENSG0000023	SYP-AS1	lncRNA	0.80411599	0.80066226	NA
ENSG0000025	RP11-158M2.	lncRNA	0.80411598	0.80103478	NA
ENSG0000012	INSL6	protein_codir	0.80411597	0.8010584	NA
ENSG0000018	SPEM1	protein_codir	0.80411597	0.80113903	NA
ENSG0000026	LINC02003	lncRNA	0.80411594	0.80224753	NA
ENSG0000022	RP11-184A2.3	lncRNA	0.80411593	0.80265642	NA
ENSG0000026	RP11-1058N1	lncRNA	0.80411593	0.80286215	NA
ENSG0000023	LINC00443	lncRNA	0.80411592	0.80294574	NA
ENSG0000025	RP11-158L12.	lncRNA	0.80411592	0.8030094	NA
ENSG0000026	RP3-430N8.1	lncRNA	0.80411591	0.80358959	NA
ENSG0000023	XXbac-BPG25	lncRNA	0.80411589	0.80409516	NA

ENSG0000027	RP11-465B22.	lncRNA	0.80411586	0.80534216	NA
ENSG0000023	RP5-1056L3.1	lncRNA	0.80411586	0.80534216	NA
ENSG0000023	LINC01307	lncRNA	0.80411586	0.80534216	NA
ENSG0000023	CNTN4-AS1	lncRNA	0.80411586	0.80534216	NA
ENSG0000027	RP1-256G22.2	lncRNA	0.80411586	0.80534216	NA
ENSG0000028	RP11-367G6.4	lncRNA	0.80411586	0.80534216	NA
ENSG0000028	SMIM40	protein_codir	0.80411586	0.80534216	NA
ENSG0000025	RP11-703H8.7	lncRNA	0.80411586	0.80534216	NA
ENSG0000028	RP11-196H14	lncRNA	0.80411586	0.80534216	NA
ENSG0000026	RP11-227G15	lncRNA	0.80411586	0.80534216	NA
ENSG0000018	MC2R	protein_codir	0.80411586	0.80534216	NA
ENSG0000027	LA16c-60D12.	lncRNA	0.80411586	0.80534216	NA
ENSG0000028	OR4F29	protein_codir	0.80411586	0.80534216	NA
ENSG0000024	RP11-54O7.2	lncRNA	0.80411586	0.80534216	NA
ENSG0000023	RP11-181G12	lncRNA	0.80411586	0.80534216	NA
ENSG0000023	LINC01346	lncRNA	0.80411586	0.80534216	NA
ENSG0000028	RP11-84A14.8	lncRNA	0.80411586	0.80534216	NA
ENSG0000028	LINC02786	lncRNA	0.80411586	0.80534216	NA
ENSG0000028	RP11-334L9.2	lncRNA	0.80411586	0.80534216	NA
ENSG0000019	GUCA2A	protein_codir	0.80411586	0.80534216	NA
ENSG0000022	MED8-AS1	lncRNA	0.80411586	0.80534216	NA
ENSG0000022	RP4-533D7.5	lncRNA	0.80411586	0.80534216	NA
ENSG0000023	RP11-89F3.2	lncRNA	0.80411586	0.80534216	NA
ENSG0000023	RP11-316M1.	lncRNA	0.80411586	0.80534216	NA
ENSG0000027	RP11-126K1.9	lncRNA	0.80411586	0.80534216	NA
ENSG0000023	RP11-98D18.2	lncRNA	0.80411586	0.80534216	NA
ENSG0000028	RP11-139D23	lncRNA	0.80411586	0.80534216	NA
ENSG0000023	RP11-144L1.4	lncRNA	0.80411586	0.80534216	NA
ENSG0000019	OR6N1	protein_codir	0.80411586	0.80534216	NA
ENSG0000019	OR10J1	protein_codir	0.80411586	0.80534216	NA
ENSG0000022	RP1-35C21.2	lncRNA	0.80411586	0.80534216	NA
ENSG0000023	LINC02818	lncRNA	0.80411586	0.80534216	NA
ENSG0000025	RGS21	protein_codir	0.80411586	0.80534216	NA
ENSG0000028	RP11-543B16.	protein_codir	0.80411586	0.80534216	NA
ENSG0000022	LPGAT1-AS1	lncRNA	0.80411586	0.80534216	NA
ENSG0000028	LINC02815	lncRNA	0.80411586	0.80534216	NA
ENSG0000017	C1orf100	protein_codir	0.80411586	0.80534216	NA
ENSG0000028	RP11-433K2.9	lncRNA	0.80411586	0.80534216	NA
ENSG0000018	OR2T34	protein_codir	0.80411586	0.80534216	NA
ENSG0000018	OR14I1	protein_codir	0.80411586	0.80534216	NA
ENSG0000022	LINC01247	lncRNA	0.80411586	0.80534216	NA
ENSG0000022	AC019048.1	lncRNA	0.80411586	0.80534216	NA
ENSG0000023	LINC01804	lncRNA	0.80411586	0.80534216	NA
ENSG0000023	GACAT3	lncRNA	0.80411586	0.80534216	NA
ENSG0000015	MSGN1	protein_codir	0.80411586	0.80534216	NA
ENSG0000023	SIX3-AS1	lncRNA	0.80411586	0.80534216	NA
ENSG0000027	RP11-493E12.	lncRNA	0.80411586	0.80534216	NA

ENSG0000023	AC016700.2	lncRNA	0.80411586	0.80534216	NA
ENSG0000021	FOXI3	protein_codir	0.80411586	0.80534216	NA
ENSG0000013	IL36RN	protein_codir	0.80411586	0.80534216	NA
ENSG0000023	LINC01826	lncRNA	0.80411586	0.80534216	NA
ENSG0000015	ACMSD	protein_codir	0.80411586	0.80534216	NA
ENSG0000023	AC104088.1	lncRNA	0.80411586	0.80534216	NA
ENSG0000016	NEUROD1	protein_codir	0.80411586	0.80534216	NA
ENSG0000024	AC007879.3	lncRNA	0.80411586	0.80534216	NA
ENSG0000023	LANCL1-AS1	lncRNA	0.80411586	0.80534216	NA
ENSG0000022	AC072062.3	lncRNA	0.80411586	0.80534216	NA
ENSG0000023	AC114803.3	lncRNA	0.80411586	0.80534216	NA
ENSG0000016	ALPG	protein_codir	0.80411586	0.80534216	NA
ENSG0000028	RP11-669E3.1	lncRNA	0.80411586	0.80534216	NA
ENSG0000028	RP11-852E15.	lncRNA	0.80411586	0.80534216	NA
ENSG0000022	LINC02585	lncRNA	0.80411586	0.80534216	NA
ENSG0000014	LRTM1	protein_codir	0.80411586	0.80534216	NA
ENSG0000027	RP11-80H18.4	lncRNA	0.80411586	0.80534216	NA
ENSG0000024	CFAP20DC-AS	lncRNA	0.80411586	0.80534216	NA
ENSG0000022	PRICKLE2-AS3	lncRNA	0.80411586	0.80534216	NA
ENSG0000024	LINC02027	lncRNA	0.80411586	0.80534216	NA
ENSG0000023	CTD-2021J15.	lncRNA	0.80411586	0.80534216	NA
ENSG0000026	RP11-2A4.4	lncRNA	0.80411586	0.80534216	NA
ENSG0000015	TRIM42	protein_codir	0.80411586	0.80534216	NA
ENSG0000023	LINC02877	lncRNA	0.80411586	0.80534216	NA
ENSG0000024	LINC01487	lncRNA	0.80411586	0.80534216	NA
ENSG0000024	RP11-298O21	lncRNA	0.80411586	0.80534216	NA
ENSG0000022	NAALADL2-AS	lncRNA	0.80411586	0.80534216	NA
ENSG0000023	FGF12-AS2	lncRNA	0.80411586	0.80534216	NA
ENSG0000028	RP11-528A4.5	lncRNA	0.80411586	0.80534216	NA
ENSG0000023	XXYLT1-AS1	lncRNA	0.80411586	0.80534216	NA
ENSG0000025	RP11-20I20.2	lncRNA	0.80411586	0.80534216	NA
ENSG0000024	RP11-669M16	lncRNA	0.80411586	0.80534216	NA
ENSG0000026	LINC02473	lncRNA	0.80411586	0.80534216	NA
ENSG0000026	LINC02616	lncRNA	0.80411586	0.80534216	NA
ENSG0000028	RP11-395I6.4	lncRNA	0.80411586	0.80534216	NA
ENSG0000024	PARM1-AS1	lncRNA	0.80411586	0.80534216	NA
ENSG0000028	RP11-398J16.	lncRNA	0.80411586	0.80534216	NA
ENSG0000024	RP11-556I14.	lncRNA	0.80411586	0.80534216	NA
ENSG0000025	RP11-173E2.2	lncRNA	0.80411586	0.80534216	NA
ENSG0000017	SLC6A19	protein_codir	0.80411586	0.80534216	NA
ENSG0000024	IRX4-AS1	lncRNA	0.80411586	0.80534216	NA
ENSG0000024	RP11-417J1.1	lncRNA	0.80411586	0.80534216	NA
ENSG0000024	LINC02150	lncRNA	0.80411586	0.80534216	NA
ENSG0000025	RP11-804N13	lncRNA	0.80411586	0.80534216	NA
ENSG0000025	LINC02109	lncRNA	0.80411586	0.80534216	NA
ENSG0000028	RP11-49C20.1	lncRNA	0.80411586	0.80534216	NA
ENSG0000028	CTD-2062O1.4	lncRNA	0.80411586	0.80534216	NA

ENSG0000025	LINC02106	lncRNA	0.80411586	0.80534216	NA
ENSG0000025	CTD-2313F11.	lncRNA	0.80411586	0.80534216	NA
ENSG0000028	AC020930.1	lncRNA	0.80411586	0.80534216	NA
ENSG0000025	LINC02161	lncRNA	0.80411586	0.80534216	NA
ENSG0000028	RP11-65F13.4	lncRNA	0.80411586	0.80534216	NA
ENSG0000024	CTC-448D22.1	lncRNA	0.80411586	0.80534216	NA
ENSG0000025	LINC02201	lncRNA	0.80411586	0.80534216	NA
ENSG0000024	RP11-43D2.2	lncRNA	0.80411586	0.80534216	NA
ENSG0000024	CTC-573M9.1	lncRNA	0.80411586	0.80534216	NA
ENSG0000027	AC005609.20	lncRNA	0.80411586	0.80534216	NA
ENSG0000028	AC005740.7	lncRNA	0.80411586	0.80534216	NA
ENSG0000022	ARHGAP26-AS	lncRNA	0.80411586	0.80534216	NA
ENSG0000025	CTC-340I23.2	lncRNA	0.80411586	0.80534216	NA
ENSG0000025	CTC-436K13.1	lncRNA	0.80411586	0.80534216	NA
ENSG0000025	RP11-541P9.3	lncRNA	0.80411586	0.80534216	NA
ENSG0000018	SMIM23	protein_codir	0.80411586	0.80534216	NA
ENSG0000025	CTB-33O18.3	lncRNA	0.80411586	0.80534216	NA
ENSG0000028	RP11-69L16.8	lncRNA	0.80411586	0.80534216	NA
ENSG0000024	RP3-510L9.1	lncRNA	0.80411586	0.80534216	NA
ENSG0000022	RP11-330A16	lncRNA	0.80411586	0.80534216	NA
ENSG0000028	XXbac-BPG24	lncRNA	0.80411586	0.80534216	NA
ENSG0000023	EHMT2-AS1	lncRNA	0.80411586	0.80534216	NA
ENSG0000028	C2-AS1	lncRNA	0.80411586	0.80534216	NA
ENSG0000022	HLA-DQB1-AS	lncRNA	0.80411586	0.80534216	NA
ENSG0000022	LINC01276	lncRNA	0.80411586	0.80534216	NA
ENSG0000028	RP1-20C7.7	protein_codir	0.80411586	0.80534216	NA
ENSG0000013	SLC22A7	protein_codir	0.80411586	0.80534216	NA
ENSG0000023	RP3-449H6.1	lncRNA	0.80411586	0.80534216	NA
ENSG0000028	RP11-406O16	lncRNA	0.80411586	0.80534216	NA
ENSG0000023	RP11-462G2.1	lncRNA	0.80411586	0.80534216	NA
ENSG0000013	CGA	protein_codir	0.80411586	0.80534216	NA
ENSG0000022	LINC02531	lncRNA	0.80411586	0.80534216	NA
ENSG0000023	RP1-142L7.5	lncRNA	0.80411586	0.80534216	NA
ENSG0000022	TAB2-AS1	lncRNA	0.80411586	0.80534216	NA
ENSG0000023	RP11-125D12	lncRNA	0.80411586	0.80534216	NA
ENSG0000026	AIRN	lncRNA	0.80411586	0.80534216	NA
ENSG0000026	RP1-39J2.1	lncRNA	0.80411586	0.80534216	NA
ENSG0000022	RP3-495K2.3	lncRNA	0.80411586	0.80534216	NA
ENSG0000014	FERD3L	protein_codir	0.80411586	0.80534216	NA
ENSG0000023	SEC61G-DT	lncRNA	0.80411586	0.80534216	NA
ENSG0000027	SPDYE8	protein_codir	0.80411586	0.80534216	NA
ENSG0000028	SPDYE11	protein_codir	0.80411586	0.80534216	NA
ENSG0000010	PAX4	protein_codir	0.80411586	0.80534216	NA
ENSG0000016	SSMEM1	protein_codir	0.80411586	0.80534216	NA
ENSG0000027	FAM131B-AS1	lncRNA	0.80411586	0.80534216	NA
ENSG0000022	AC078942.1	lncRNA	0.80411586	0.80534216	NA
ENSG0000028	RP4-708P22.1	lncRNA	0.80411586	0.80534216	NA

ENSG0000028	CTD-2336O2.!	lncRNA	0.80411586	0.80534216	NA
ENSG0000028	RP11-481A20	lncRNA	0.80411586	0.80534216	NA
ENSG0000028	RP11-205M5.:	protein_codir	0.80411586	0.80534216	NA
ENSG0000025	RP11-386G21	lncRNA	0.80411586	0.80534216	NA
ENSG0000025	RP11-1D12.1	lncRNA	0.80411586	0.80534216	NA
ENSG0000025	RP11-257P3.3	lncRNA	0.80411586	0.80534216	NA
ENSG0000024	LINC00536	lncRNA	0.80411586	0.80534216	NA
ENSG0000025	RP11-557C18.	lncRNA	0.80411586	0.80534216	NA
ENSG0000028	RP11-370K2.1	lncRNA	0.80411586	0.80534216	NA
ENSG0000028	CTD-3065J16.	lncRNA	0.80411586	0.80534216	NA
ENSG0000022	RP11-143M1.	lncRNA	0.80411586	0.80534216	NA
ENSG0000018	FAM205C	protein_codir	0.80411586	0.80534216	NA
ENSG0000016	OR13J1	protein_codir	0.80411586	0.80534216	NA
ENSG0000020	C9orf135	protein_codir	0.80411586	0.80534216	NA
ENSG0000023	RP11-508N12	lncRNA	0.80411586	0.80534216	NA
ENSG0000023	RP11-175D17	lncRNA	0.80411586	0.80534216	NA
ENSG0000028	RP11-228B15.	lncRNA	0.80411586	0.80534216	NA
ENSG0000023	RP11-298E9.5	lncRNA	0.80411586	0.80534216	NA
ENSG0000028	RP11-799O21	lncRNA	0.80411586	0.80534216	NA
ENSG0000023	RP11-534L6.2	lncRNA	0.80411586	0.80534216	NA
ENSG0000023	RP11-442O18	lncRNA	0.80411586	0.80534216	NA
ENSG0000023	LINC01166	lncRNA	0.80411586	0.80534216	NA
ENSG0000022	MRPL23-AS1	lncRNA	0.80411586	0.80534216	NA
ENSG0000018	CTC-343N3.1	lncRNA	0.80411586	0.80534216	NA
ENSG0000027	OR51B2	protein_codir	0.80411586	0.80534216	NA
ENSG0000016	OR51I1	protein_codir	0.80411586	0.80534216	NA
ENSG0000024	RIC3-DT	lncRNA	0.80411586	0.80534216	NA
ENSG0000025	LINC02751	lncRNA	0.80411586	0.80534216	NA
ENSG0000022	Z83001.1	lncRNA	0.80411586	0.80534216	NA
ENSG0000028	RP11-472I20.:	lncRNA	0.80411586	0.80534216	NA
ENSG0000025	RP11-864I4.3	lncRNA	0.80411586	0.80534216	NA
ENSG0000028	RP11-867O8.:	lncRNA	0.80411586	0.80534216	NA
ENSG0000025	OR7E11P	lncRNA	0.80411586	0.80534216	NA
ENSG0000025	CTD-2562J17.	lncRNA	0.80411586	0.80534216	NA
ENSG0000025	LINC02720	lncRNA	0.80411586	0.80534216	NA
ENSG0000023	RP11-867G2.!	lncRNA	0.80411586	0.80534216	NA
ENSG0000025	RP11-817J15.	lncRNA	0.80411586	0.80534216	NA
ENSG0000025	ALG9-IT1	lncRNA	0.80411586	0.80534216	NA
ENSG0000022	AP006216.10	lncRNA	0.80411586	0.80534216	NA
ENSG0000028	LINC01395	lncRNA	0.80411586	0.80534216	NA
ENSG0000028	LINC02717	lncRNA	0.80411586	0.80534216	NA
ENSG0000025	RP5-1096D14	lncRNA	0.80411586	0.80534216	NA
ENSG0000025	RP11-1038A1	lncRNA	0.80411586	0.80534216	NA
ENSG0000025	RP11-273B20.	lncRNA	0.80411586	0.80534216	NA
ENSG0000028	RP11-22B23.!	lncRNA	0.80411586	0.80534216	NA
ENSG0000019	PRB3	protein_codir	0.80411586	0.80534216	NA
ENSG0000012	PRB2	protein_codir	0.80411586	0.80534216	NA

ENSG0000021	C12orf71	protein_codir	0.80411586	0.80534216	NA
ENSG0000025	LINC02450	lncRNA	0.80411586	0.80534216	NA
ENSG0000028	RP11-499A10	lncRNA	0.80411586	0.80534216	NA
ENSG0000018	KRT77	protein_codir	0.80411586	0.80534216	NA
ENSG0000013	TPH2	protein_codir	0.80411586	0.80534216	NA
ENSG0000025	LINC02882	lncRNA	0.80411586	0.80534216	NA
ENSG0000025	RP11-390N6.1	lncRNA	0.80411586	0.80534216	NA
ENSG0000028	RP11-1105G2	lncRNA	0.80411586	0.80534216	NA
ENSG0000025	RP11-778J16.	lncRNA	0.80411586	0.80534216	NA
ENSG0000025	RP11-144F15.	lncRNA	0.80411586	0.80534216	NA
ENSG0000017	CCDC63	protein_codir	0.80411586	0.80534216	NA
ENSG0000025	RP1-71H24.1	lncRNA	0.80411586	0.80534216	NA
ENSG0000021	LINC02418	lncRNA	0.80411586	0.80534216	NA
ENSG0000028	RP11-897M7.	lncRNA	0.80411586	0.80534216	NA
ENSG0000027	RP13-977J11.	lncRNA	0.80411586	0.80534216	NA
ENSG0000023	LINC01046	lncRNA	0.80411586	0.80534216	NA
ENSG0000028	RP11-45B20.6	lncRNA	0.80411586	0.80534216	NA
ENSG0000028	RP11-223E19.	lncRNA	0.80411586	0.80534216	NA
ENSG0000028	RP11-168P13.	lncRNA	0.80411586	0.80534216	NA
ENSG0000016	ERICH6B	protein_codir	0.80411586	0.80534216	NA
ENSG0000023	LINC00462	lncRNA	0.80411586	0.80534216	NA
ENSG0000022	C13orf42	protein_codir	0.80411586	0.80534216	NA
ENSG0000028	RP11-275O3.1	lncRNA	0.80411586	0.80534216	NA
ENSG0000027	RP11-255P5.2	lncRNA	0.80411586	0.80534216	NA
ENSG0000025	RP11-219E7.2	lncRNA	0.80411586	0.80534216	NA
ENSG0000025	LINC00596	lncRNA	0.80411586	0.80534216	NA
ENSG0000025	LINC02327	lncRNA	0.80411586	0.80534216	NA
ENSG0000025	RP11-463J10.	lncRNA	0.80411586	0.80534216	NA
ENSG0000025	RP11-486O13	lncRNA	0.80411586	0.80534216	NA
ENSG0000020	ACOT6	protein_codir	0.80411586	0.80534216	NA
ENSG0000025	RP11-661G16	lncRNA	0.80411586	0.80534216	NA
ENSG0000028	RP11-44N21.5	lncRNA	0.80411586	0.80534216	NA
ENSG0000025	LINC01193	lncRNA	0.80411586	0.80534216	NA
ENSG0000015	GOLGA8F	protein_codir	0.80411586	0.80534216	NA
ENSG0000028	WI2-2413G8.1	lncRNA	0.80411586	0.80534216	NA
ENSG0000026	RP11-133K1.7	lncRNA	0.80411586	0.80534216	NA
ENSG0000025	RP11-394B5.2	lncRNA	0.80411586	0.80534216	NA
ENSG0000017	LDHAL6B	protein_codir	0.80411586	0.80534216	NA
ENSG0000025	RP11-16B9.1	lncRNA	0.80411586	0.80534216	NA
ENSG0000025	RP11-317G6.1	lncRNA	0.80411586	0.80534216	NA
ENSG0000028	RP11-1152M1	lncRNA	0.80411586	0.80534216	NA
ENSG0000025	RP11-253M7.	lncRNA	0.80411586	0.80534216	NA
ENSG0000027	RP11-762H8.4	lncRNA	0.80411586	0.80534216	NA
ENSG0000025	RP11-382A20	lncRNA	0.80411586	0.80534216	NA
ENSG0000025	RP11-815J21.4	lncRNA	0.80411586	0.80534216	NA
ENSG0000028	RP11-825M14	lncRNA	0.80411586	0.80534216	NA
ENSG0000025	RP11-24J19.1	lncRNA	0.80411586	0.80534216	NA

ENSG0000025	CTD-2313J17.	lncRNA	0.80411586	0.80534216	NA
ENSG0000025	RP11-522B15.	lncRNA	0.80411586	0.80534216	NA
ENSG0000026	LA16c-316G12	lncRNA	0.80411586	0.80534216	NA
ENSG0000026	RP11-165E7.1	lncRNA	0.80411586	0.80534216	NA
ENSG0000017	RP11-276H1.3	lncRNA	0.80411586	0.80534216	NA
ENSG0000026	CTA-363E6.7	lncRNA	0.80411586	0.80534216	NA
ENSG0000017	ZG16	protein_codir	0.80411586	0.80534216	NA
ENSG0000026	LINC02184	lncRNA	0.80411586	0.80534216	NA
ENSG0000026	RP11-177N22	lncRNA	0.80411586	0.80534216	NA
ENSG0000026	LINC02128	lncRNA	0.80411586	0.80534216	NA
ENSG0000028	RP11-26L20.5	lncRNA	0.80411586	0.80534216	NA
ENSG0000026	LINC02141	lncRNA	0.80411586	0.80534216	NA
ENSG0000026	RP11-96H17.1	lncRNA	0.80411586	0.80534216	NA
ENSG0000026	RP11-403P17.	lncRNA	0.80411586	0.80534216	NA
ENSG0000026	RP11-96D1.7	lncRNA	0.80411586	0.80534216	NA
ENSG0000026	RP11-449J10.	lncRNA	0.80411586	0.80534216	NA
ENSG0000022	GAS8-AS1	lncRNA	0.80411586	0.80534216	NA
ENSG0000018	OR1E1	protein_codir	0.80411586	0.80534216	NA
ENSG0000026	RP11-599B13.	lncRNA	0.80411586	0.80534216	NA
ENSG0000026	RP11-283C24.	lncRNA	0.80411586	0.80534216	NA
ENSG0000026	AC010761.9	lncRNA	0.80411586	0.80534216	NA
ENSG0000026	RP11-20B24.2	lncRNA	0.80411586	0.80534216	NA
ENSG0000026	CTC-304I17.5	lncRNA	0.80411586	0.80534216	NA
ENSG0000026	RP11-799D4.2	lncRNA	0.80411586	0.80534216	NA
ENSG0000021	KRTAP2-1	protein_codir	0.80411586	0.80534216	NA
ENSG0000022	LINC00974	lncRNA	0.80411586	0.80534216	NA
ENSG0000027	RP11-229E13.	lncRNA	0.80411586	0.80534216	NA
ENSG0000026	MIR2117HG	lncRNA	0.80411586	0.80534216	NA
ENSG0000026	LINC01976	lncRNA	0.80411586	0.80534216	NA
ENSG0000028	CTC-264K15.5	protein_codir	0.80411586	0.80534216	NA
ENSG0000026	LINC02074	lncRNA	0.80411586	0.80534216	NA
ENSG0000026	RP11-703M24	lncRNA	0.80411586	0.80534216	NA
ENSG0000026	LINC01254	lncRNA	0.80411586	0.80534216	NA
ENSG0000026	RP11-856M7.	lncRNA	0.80411586	0.80534216	NA
ENSG0000026	RP11-527H14	lncRNA	0.80411586	0.80534216	NA
ENSG0000026	RP11-126K15.	lncRNA	0.80411586	0.80534216	NA
ENSG0000026	AC004623.2	lncRNA	0.80411586	0.80534216	NA
ENSG0000027	CTD-3020H12	lncRNA	0.80411586	0.80534216	NA
ENSG0000026	CTC-215O4.4	lncRNA	0.80411586	0.80534216	NA
ENSG0000018	C19orf67	protein_codir	0.80411586	0.80534216	NA
ENSG0000014	CIB3	protein_codir	0.80411586	0.80534216	NA
ENSG0000028	CTD-2562J15.	lncRNA	0.80411586	0.80534216	NA
ENSG0000026	CTD-2626G11	lncRNA	0.80411586	0.80534216	NA
ENSG0000026	AC005780.1	lncRNA	0.80411586	0.80534216	NA
ENSG0000026	AC008991.1	lncRNA	0.80411586	0.80534216	NA
ENSG0000026	CTD-2162K18	lncRNA	0.80411586	0.80534216	NA
ENSG0000000	LGALS14	protein_codir	0.80411586	0.80534216	NA

ENSG0000026	CTC-435M10.	lncRNA	0.80411586	0.80534216	NA
ENSG0000010	CGB2	protein_codir	0.80411586	0.80534216	NA
ENSG0000022	NTF4	protein_codir	0.80411586	0.80534216	NA
ENSG0000026	CTD-3187F8.1	lncRNA	0.80411586	0.80534216	NA
ENSG0000026	AC006272.2	lncRNA	0.80411586	0.80534216	NA
ENSG0000022	LILRB1-AS1	lncRNA	0.80411586	0.80534216	NA
ENSG0000018	FAM71E2	protein_codir	0.80411586	0.80534216	NA
ENSG0000027	RP4-550H1.7	lncRNA	0.80411586	0.80534216	NA
ENSG0000010	HNF4A	protein_codir	0.80411586	0.80534216	NA
ENSG0000026	WI2-87327B8	lncRNA	0.80411586	0.80534216	NA
ENSG0000027	KRTAP7-1	protein_codir	0.80411586	0.80534216	NA
ENSG0000022	FAM243A	protein_codir	0.80411586	0.80534216	NA
ENSG0000022	LINC01671	lncRNA	0.80411586	0.80534216	NA
ENSG0000028	AJ011931.2	lncRNA	0.80411586	0.80534216	NA
ENSG0000028	KB-1440D3.21	lncRNA	0.80411586	0.80534216	NA
ENSG0000022	AP000345.2	lncRNA	0.80411586	0.80534216	NA
ENSG0000027	CTA-243E7.2	lncRNA	0.80411586	0.80534216	NA
ENSG0000023	CTA-984G1.5	lncRNA	0.80411586	0.80534216	NA
ENSG0000016	KCNJ4	protein_codir	0.80411586	0.80534216	NA
ENSG0000023	RP11-431J24.	lncRNA	0.80411586	0.80534216	NA
ENSG0000018	NYX	protein_codir	0.80411586	0.80534216	NA
ENSG0000013	BMP15	protein_codir	0.80411586	0.80534216	NA
ENSG0000014	AWAT2	protein_codir	0.80411586	0.80534216	NA
ENSG0000021	TCP11X2	protein_codir	0.80411586	0.80534216	NA
ENSG0000022	AMMECR1-IT1	lncRNA	0.80411586	0.80534216	NA
ENSG0000028	RP5-1142C11.	lncRNA	0.80411586	0.80534216	NA
ENSG0000013	RHOXF2	protein_codir	0.80411586	0.80534216	NA
ENSG0000028	HSFX4	protein_codir	0.80411586	0.80534216	NA
ENSG0000023	FAM197Y6	lncRNA	0.80411586	0.80534216	NA
ENSG0000025	RP11-65G9.1	lncRNA	0.80411586	0.80534216	NA
ENSG0000022	LINC01208	lncRNA	0.80408933	0.64274707	NA
ENSG0000017	RAD9A	protein_codir	0.80384084	0.01107225	0.03773503
ENSG0000013	CTSL	protein_codir	0.80382381	0.00656985	0.02504318
ENSG0000010	CASP2	protein_codir	0.8035269	0.00042397	0.00285819
ENSG0000016	PPP1R36	protein_codir	0.80339734	0.0383509	0.09816978
ENSG0000010	CCM2L	protein_codir	0.80336285	0.01274821	0.0419823
ENSG0000024	RP1-179N16.6	lncRNA	0.80328364	0.12689473	0.24088195
ENSG0000014	CFAP74	protein_codir	0.80324597	0.30924328	0.46089463
ENSG0000026	RP13-487P22.	lncRNA	0.80258745	0.42844217	0.58015194
ENSG0000027	CYB561D2	lncRNA	0.80241994	0.03446168	0.0905716
ENSG0000015	FANCC	protein_codir	0.80171188	0.00143919	0.00763442
ENSG0000015	FAM81A	protein_codir	0.80158308	0.02602992	0.07285633
ENSG0000022	HAGLR	lncRNA	0.80092715	0.01114846	0.03792126
ENSG0000022	RP1-167A14.2	lncRNA	0.8008949	0.60202283	0.7290048
ENSG0000028	RP11-399F4.3	lncRNA	0.80069111	0.65013353	NA
ENSG0000028	RP11-314L11.	lncRNA	0.80058551	0.27705107	0.42547404
ENSG0000013	HAUS8	protein_codir	0.80030666	0.00533105	0.02132684

ENSG0000026	AQP4-AS1	lncRNA	0.80006853	0.53447557	0.67499841
ENSG0000016	NAGS	protein_codir	0.79936724	0.06507115	0.14712386
ENSG0000024	RP11-36B15.1	lncRNA	0.79919496	0.28082527	0.43009837
ENSG0000018	ZNRF2	protein_codir	0.79840121	0.00012801	0.00107367
ENSG0000021	LTC4S	protein_codir	0.7980656	0.00176925	0.00894014
ENSG0000025	RP11-48G14.2	lncRNA	0.79789456	0.45579909	0.60650243
ENSG0000023	CTA-221G9.7	lncRNA	0.79771454	0.43598267	0.58720638
ENSG0000013	TBR1	protein_codir	0.79740238	0.54490053	0.68304224
ENSG0000026	LINC01081	lncRNA	0.79690627	0.38451669	0.53773929
ENSG0000017	PAQR8	protein_codir	0.79664986	0.00110865	0.00620737
ENSG0000022	PPP3CB-AS1	lncRNA	0.7963118	0.00649224	0.02487042
ENSG0000026	RP11-476K15.	lncRNA	0.79600938	0.73479072	NA
ENSG0000015	PARP8	protein_codir	0.79588755	0.00244257	0.01148092
ENSG0000003	GRN	protein_codir	0.79587559	0.00015282	0.00124427
ENSG0000010	PKD2L1	protein_codir	0.79575981	0.26544911	0.4130305
ENSG0000015	DCLRE1C	protein_codir	0.79575492	0.00402295	0.01710497
ENSG0000016	S100A12	protein_codir	0.79557844	0.18644693	0.31981093
ENSG0000025	RP11-66B24.4	lncRNA	0.79529634	0.0063702	0.02449121
ENSG0000016	EN2	protein_codir	0.7951797	0.33014779	0.4826998
ENSG0000024	AMY2B	protein_codir	0.79506711	0.03573881	0.09302421
ENSG0000015	LYPD5	protein_codir	0.79449801	0.03293408	0.08742329
ENSG0000022	ZNF469	protein_codir	0.79446384	0.04359233	0.10846052
ENSG0000019	SUCNR1	protein_codir	0.79407531	0.05442229	0.12825458
ENSG0000026	RP11-973H7.3	lncRNA	0.79401556	0.00707175	0.02651305
ENSG0000027	SYNPO2L-AS1	lncRNA	0.79394137	0.19855888	0.33487185
ENSG0000015	IFNGR2	protein_codir	0.79367087	5.89E-05	0.00056464
ENSG0000027	RP11-342K6.1	lncRNA	0.79363633	0.5734362	0.7063252
ENSG0000025	RP11-563P16.	lncRNA	0.79315521	0.49878722	0.64448461
ENSG0000017	SPHK1	protein_codir	0.79296052	0.0221517	0.06418022
ENSG0000022	LINC02586	lncRNA	0.79290297	0.34945693	0.5029441
ENSG0000025	RP11-567J20.	lncRNA	0.7924139	0.05129186	0.12273076
ENSG0000011	APOA1	protein_codir	0.79195036	0.17611037	0.30640431
ENSG0000013	DNAJC1	protein_codir	0.79152082	2.26E-05	0.00025411
ENSG0000026	RP11-1348G1	lncRNA	0.79141665	0.59138222	0.72060604
ENSG0000025	DND1	protein_codir	0.79138608	0.20898681	0.34791246
ENSG0000014	TLNRD1	protein_codir	0.79126132	0.00232182	0.01105478
ENSG0000026	TSHZ3-AS1	lncRNA	0.79100028	0.73508462	NA
ENSG0000011	TACR1	protein_codir	0.79090159	0.05932998	0.13671734
ENSG0000026	AL136419.6	lncRNA	0.79010799	0.02556781	0.07187115
ENSG0000017	FOSL1	protein_codir	0.7897257	0.25658048	0.40323747
ENSG0000028	RP11-16O9.3	protein_codir	0.78906627	0.45776741	0.60822971
ENSG0000027	RP11-802O23	lncRNA	0.78896746	0.02380989	0.06791747
ENSG0000020	RP11-863P13.	lncRNA	0.78892998	0.35512217	0.50879107
ENSG0000023	RP11-310E22.	lncRNA	0.78889422	0.38611767	0.53918817
ENSG0000022	AC073283.7	lncRNA	0.78877263	0.24404429	0.38915209
ENSG0000028	CTC-756D1.4	lncRNA	0.78865284	0.32917306	0.48152958
ENSG0000027	RP11-347C18.	lncRNA	0.78806803	0.14376685	0.26394844

ENSG0000028	GS1-215N23.2	lncRNA	0.78801092	0.78626664	NA
ENSG0000021	KRTAP2-3	protein_codir	0.78785924	0.78177748	NA
ENSG0000011	ELK3	protein_codir	0.7878089	3.66E-05	0.00037738
ENSG0000026	RP11-2N1.2	lncRNA	0.78775423	0.77872568	NA
ENSG0000025	RP11-810P8.1	lncRNA	0.78774889	0.77857145	NA
ENSG0000018	WASHC1	protein_codir	0.78771889	0.03349191	0.08858053
ENSG0000016	SYNPR	protein_codir	0.78770735	0.77737733	NA
ENSG0000028	LINC01097	lncRNA	0.78769538	0.77703442	NA
ENSG0000014	DSN1	protein_codir	0.78766195	7.50E-05	0.00068623
ENSG0000028	GS1-146J4.4	lncRNA	0.78754147	0.57221881	0.70531434
ENSG0000011	NPC2	protein_codir	0.78740669	3.41E-05	0.00035534
ENSG0000026	AC002116.7	lncRNA	0.78719332	0.26713295	0.41480924
ENSG0000024	RP11-875O11	lncRNA	0.78712321	0.00367341	0.01585801
ENSG0000017	HEG1	protein_codir	0.78705381	0.00089936	0.0052608
ENSG0000014	DENND2A	protein_codir	0.78697466	0.09077924	0.18742133
ENSG0000011	GCA	protein_codir	0.78690076	2.60E-05	0.00028597
ENSG0000023	LINC01358	lncRNA	0.78680123	0.0603038	0.13855678
ENSG0000012	MTRF1	protein_codir	0.78620161	0.00313006	0.01397919
ENSG0000023	RP11-61L14.6	lncRNA	0.78614425	0.60763045	NA
ENSG0000012	PRDX4	protein_codir	0.78613735	0.0013403	0.00723031
ENSG0000014	SNTG1	protein_codir	0.78604283	0.73412331	NA
ENSG0000025	RP11-582J16.	lncRNA	0.78601156	0.49122865	0.63794129
ENSG0000025	RP11-368P15.	lncRNA	0.78587566	0.73018887	NA
ENSG0000023	RP11-335O13	lncRNA	0.78585683	0.729749	NA
ENSG0000028	RP11-27G14.4	lncRNA	0.78585293	0.72965816	NA
ENSG0000023	UBE2E2-AS1	lncRNA	0.78578435	0.72806372	NA
ENSG0000025	RP11-334E6.3	lncRNA	0.78576601	0.72763907	NA
ENSG0000025	PPIAL4D	protein_codir	0.78574866	0.72723797	NA
ENSG0000025	RP11-664I21.5	lncRNA	0.78569134	0.72591726	NA
ENSG0000016	HMGB2	protein_codir	0.78567956	0.00237199	0.01122784
ENSG0000006	EYA2	protein_codir	0.7853658	0.01373717	0.04451725
ENSG0000027	CCL18	protein_codir	0.78533234	0.32980238	0.48232244
ENSG0000022	AC112715.2	lncRNA	0.78531771	0.55141751	0.68836844
ENSG0000025	RTL1	protein_codir	0.78482263	0.66216393	NA
ENSG0000010	AMMECR1	protein_codir	0.78472146	3.31E-05	0.00034707
ENSG0000025	RP11-794G24	lncRNA	0.7844142	0.53642495	0.67659407
ENSG0000016	ELAVL4	protein_codir	0.78439977	0.15825516	0.28337164
ENSG0000001	CLK1	protein_codir	0.78439544	0.01178638	0.03957931
ENSG0000026	INSYN1-AS1	lncRNA	0.78390559	0.64199784	NA
ENSG0000022	AC091633.3	lncRNA	0.7838251	0.21155119	0.35101789
ENSG0000013	SDCBP	protein_codir	0.78344215	5.11E-05	0.00050063
ENSG0000028	RP11-567P17.	lncRNA	0.78340871	0.67802547	NA
ENSG0000005	NNAT	protein_codir	0.78322568	0.17030135	0.29918097
ENSG0000021	LRRC69	protein_codir	0.78281651	0.13806256	0.25613163
ENSG0000023	ZEB2-AS1	lncRNA	0.78272349	0.05223581	0.12427883
ENSG0000016	RSKR	protein_codir	0.78271669	0.06407705	0.14536412
ENSG0000010	COBL	protein_codir	0.78226971	0.1314593	0.24732006

ENSG0000016	ESM1	protein_codir	0.78222116	0.10257786	0.20569193
ENSG0000018	H2AX	protein_codir	0.78201446	0.0009671	0.00557763
ENSG0000010	MAG	protein_codir	0.78177357	0.36818684	0.52166413
ENSG0000017	ZNF439	protein_codir	0.78177091	0.01398073	0.04512431
ENSG0000012	LYPD3	protein_codir	0.78165959	0.00832771	0.03012483
ENSG0000025	ZNF710-AS1	lncRNA	0.78153345	0.00928555	0.03287615
ENSG0000017	PDE7B	protein_codir	0.78131346	0.03126264	0.08402006
ENSG0000025	RP11-80A15.1	lncRNA	0.78121432	0.0719722	0.15824489
ENSG0000018	NUTM2B	protein_codir	0.78101364	0.01569435	0.04935419
ENSG0000013	ACP2	protein_codir	0.78100942	0.00069507	0.00428212
ENSG0000014	FBXO15	protein_codir	0.78071293	0.17639451	0.30680204
ENSG0000016	CTNND2	protein_codir	0.78058399	0.24019829	0.38468496
ENSG0000000	MSL3	protein_codir	0.78052276	0.002629	0.01215752
ENSG0000027	RP11-574K11.	lncRNA	0.78041118	0.03003916	0.08156523
ENSG0000027	RP13-580B18.	lncRNA	0.78027061	0.36505538	0.51893101
ENSG0000025	TRIM7-AS1	lncRNA	0.78010607	0.44559035	0.59665421
ENSG0000024	LINC02217	lncRNA	0.77991023	0.68881353	NA
ENSG0000021	MIR17HG	lncRNA	0.77957882	0.14234925	0.26195525
ENSG0000015	PIP	protein_codir	0.77903687	0.73183818	NA
ENSG0000017	SIGLECL1	protein_codir	0.77903066	0.73157777	NA
ENSG0000028	RP1-73H22.5	protein_codir	0.7789979	0.73020859	NA
ENSG0000024	LINCR-0003	lncRNA	0.77893943	0.72778651	NA
ENSG0000015	MAGEC1	protein_codir	0.77889252	0.68581297	NA
ENSG0000025	RP11-2E17.2	lncRNA	0.77885469	0.68462922	NA
ENSG0000015	SIM2	protein_codir	0.77872034	0.52875354	0.67022362
ENSG0000028	RP11-427I6.5	lncRNA	0.77871616	0.68034337	NA
ENSG0000028	CH507-9B2.9	protein_codir	0.77868675	0.00304512	0.01366469
ENSG0000012	SLC17A3	protein_codir	0.77797703	0.70071196	0.80496046
ENSG0000023	AP001596.6	lncRNA	0.77781095	0.68550332	NA
ENSG0000024	CTD-2193P3.2	lncRNA	0.7776803	0.68106939	NA
ENSG0000027	XXbac-BPGBP	lncRNA	0.77767325	0.68083231	NA
ENSG0000026	AC003005.2	lncRNA	0.7776659	0.68058565	NA
ENSG0000026	RP1-66C13.4	protein_codir	0.77764437	0.63463582	0.75463412
ENSG0000011	CCN6	protein_codir	0.77763629	0.67959413	NA
ENSG0000013	AOX1	protein_codir	0.77695035	0.09760831	0.19814358
ENSG0000028	RP11-169L18.	lncRNA	0.77670169	0.31419889	0.46606731
ENSG0000025	FZD10-AS1	lncRNA	0.77664407	0.04791583	0.11650983
ENSG0000026	CTC-548K16.6	lncRNA	0.77614062	0.36280533	0.51666323
ENSG0000019	KRT39	protein_codir	0.77591662	0.7354875	NA
ENSG0000016	CYP3A7	protein_codir	0.77591218	0.73519542	NA
ENSG0000017	CATSPERE	protein_codir	0.77553376	0.05142441	0.12294141
ENSG0000008	ADAMTS2	protein_codir	0.77546575	0.02079513	0.06110878
ENSG0000023	EMSLR	lncRNA	0.77543103	0.4181961	0.57087809
ENSG0000019	HSD17B11	protein_codir	0.77500927	1.06E-06	1.93E-05
ENSG0000006	ERBB3	protein_codir	0.77478245	0.23443316	0.3777959
ENSG0000025	RP11-983P16.	lncRNA	0.77447448	0.43747623	0.5887642
ENSG0000026	RP5-906A24.2	lncRNA	0.7743586	0.78899949	NA

ENSG0000018	GOLGA8M	protein_codir	0.77419956	0.0373095	0.09614559
ENSG0000027	LINC02826	lncRNA	0.774148	0.68834216	NA
ENSG0000025	LINC02598	lncRNA	0.77383294	0.73701806	NA
ENSG0000028	CFAP97D2	protein_codir	0.77375561	0.73047181	NA
ENSG0000018	PYCR1	protein_codir	0.77361701	0.04071638	0.10283343
ENSG0000010	SPTLC2	protein_codir	0.77358274	0.0005767	0.0036702
ENSG0000011	ARFGEF3	protein_codir	0.77357723	0.07660635	0.16539258
ENSG0000012	TSKS	protein_codir	0.77342859	0.0505511	0.12154796
ENSG0000018	RP11-797H7.5	lncRNA	0.77340672	0.40078585	0.5539284
ENSG0000026	RP11-739L10.	lncRNA	0.7733553	0.64807829	NA
ENSG0000016	TVP23A	protein_codir	0.77290543	0.01078757	0.03698979
ENSG0000027	CTB-32O4.3	lncRNA	0.77251512	0.74329546	NA
ENSG0000006	HYAL2	protein_codir	0.77232858	0.00218201	0.01051601
ENSG0000024	CFB	protein_codir	0.77218798	0.00476197	0.01952868
ENSG0000014	NCAPG2	protein_codir	0.77199682	3.32E-05	0.00034804
ENSG0000027	RP11-65L3.2	lncRNA	0.7717508	0.02242499	0.064836
ENSG0000009	PTGS1	protein_codir	0.77173878	0.03305478	0.08767628
ENSG0000010	CCDC134	protein_codir	0.77150001	0.00123572	0.00676793
ENSG0000028	RP11-844P9.6	lncRNA	0.77140733	0.69272304	NA
ENSG0000019	CASP4	protein_codir	0.77139547	0.00084255	0.00499385
ENSG0000016	RSPH1	protein_codir	0.77113232	0.13295548	0.2494044
ENSG0000025	CTD-2066L21.	lncRNA	0.77108663	0.49226905	0.63884186
ENSG0000016	LRRC56	protein_codir	0.77093678	0.05360306	0.12679999
ENSG0000027	CTB-58E17.9	lncRNA	0.77069742	0.54743163	0.68518882
ENSG0000014	FBXO10	protein_codir	0.77061645	0.00370699	0.0159805
ENSG0000013	SLC38A4	protein_codir	0.77040861	0.10580101	0.2105197
ENSG0000026	RP11-159D12	lncRNA	0.77037886	0.08089057	0.17209965
ENSG0000028	RP11-545M17	lncRNA	0.77029239	0.4385246	0.58965238
ENSG0000008	EDEM2	protein_codir	0.77004598	5.73E-05	0.00055108
ENSG0000027	RP11-4B16.4	lncRNA	0.77003398	0.13411042	0.2509063
ENSG0000018	NRIP1	protein_codir	0.76983622	0.0005419	0.00349619
ENSG0000025	RP11-659G9.3	lncRNA	0.7698225	0.79222014	NA
ENSG0000027	RP4-736L20.3	lncRNA	0.76967002	0.74440658	NA
ENSG0000017	TMEM51-AS1	lncRNA	0.76954161	0.07138144	0.15729027
ENSG0000021	C5orf49	protein_codir	0.76951002	0.23283064	0.37589328
ENSG0000028	RP11-76P17.4	lncRNA	0.76941732	0.07103429	0.15670093
ENSG0000000	RALA	protein_codir	0.76935392	2.65E-06	4.15E-05
ENSG0000016	ATF3	protein_codir	0.76860363	0.22788059	0.37019026
ENSG0000018	ANKRD20A2P	protein_codir	0.76845772	0.46347007	0.61337303
ENSG0000019	ZNF138	protein_codir	0.76836845	0.0083285	0.03012483
ENSG0000026	RP11-715J22.	lncRNA	0.76791438	0.09701524	0.19721506
ENSG0000027	AP000255.6	lncRNA	0.7678814	0.30012619	0.45107953
ENSG0000025	RP11-507K2.2	lncRNA	0.76733538	0.81404468	NA
ENSG0000012	ACVR2A	protein_codir	0.76713194	0.0003484	0.00243921
ENSG0000027	RP5-875H18.1	lncRNA	0.76710398	0.74372677	NA
ENSG0000011	DSE	protein_codir	0.76699137	0.0023065	0.01099634
ENSG0000026	LMO7-AS1	lncRNA	0.76671921	0.17995966	0.31137542

ENSG0000027	LINC02012	lncRNA	0.76664761	0.38401225	0.53736034
ENSG0000018	CAMK1D	protein_codir	0.76663992	4.32E-05	0.0004324
ENSG0000015	UBE2L6	protein_codir	0.76649423	9.64E-05	0.0008419
ENSG0000020	C1RL-AS1	lncRNA	0.76647776	0.05580331	0.13059544
ENSG0000022	LINC02766	lncRNA	0.76633452	0.3376366	0.4904556
ENSG0000016	HEY1	protein_codir	0.7663131	0.03218719	0.08580888
ENSG0000016	TBATA	protein_codir	0.76618298	0.79450919	NA
ENSG0000028	RP11-55C5.1	lncRNA	0.76611327	0.53784018	0.67748191
ENSG0000027	H4C4	protein_codir	0.76592297	0.51140778	0.65512491
ENSG0000026	LA16c-358B7.	lncRNA	0.76584313	0.47600094	0.6242413
ENSG0000006	CBFB	protein_codir	0.765655	4.24E-08	1.20E-06
ENSG0000023	ZNF736	protein_codir	0.76561825	0.01534994	0.04853084
ENSG0000013	SLC14A2	protein_codir	0.76533779	0.02927606	0.07998068
ENSG0000025	CTC-435M10.	protein_codir	0.76491833	0.35417546	0.50780348
ENSG0000017	PRSS36	protein_codir	0.76469067	0.05108527	0.12242734
ENSG0000010	CARD10	protein_codir	0.76459235	0.00940742	0.03318853
ENSG0000018	LINC00304	lncRNA	0.76409655	0.17067355	0.29970141
ENSG0000000	TFPI	protein_codir	0.76396762	0.02997143	0.08140853
ENSG0000007	NFKB2	protein_codir	0.76393996	0.00251456	0.01173575
ENSG0000027	RP1-267L14.6	lncRNA	0.76387926	0.28859459	0.43818276
ENSG0000027	RP5-1112D6.7	lncRNA	0.76343207	0.1443595	0.26479011
ENSG0000027	RP11-26J3.3	lncRNA	0.76325168	0.01395777	0.04506296
ENSG0000016	FGFR4	protein_codir	0.76320015	0.09293244	0.19092488
ENSG0000015	PAN3	protein_codir	0.76286499	1.49E-07	3.51E-06
ENSG0000014	TTBK1	protein_codir	0.76280811	0.13555618	0.25266984
ENSG0000027	LINC02019	lncRNA	0.76259854	0.02828458	0.07782628
ENSG0000014	TM7SF2	protein_codir	0.76258454	0.00413081	0.01749625
ENSG0000026	AC005523.2	lncRNA	0.76256484	0.09243306	0.19012519
ENSG0000021	OR2A42	protein_codir	0.7625025	0.52715163	0.66883771
ENSG0000017	SLC16A5	protein_codir	0.76199929	0.00010222	0.00088535
ENSG0000018	SLC8A1	protein_codir	0.76193836	0.05367952	0.12694822
ENSG0000016	OLFML2B	protein_codir	0.76185227	0.0065471	0.02499901
ENSG0000026	RP11-395I6.3	lncRNA	0.76169912	0.16783276	0.29618547
ENSG0000013	DUSP6	protein_codir	0.76100915	0.00067103	0.00416213
ENSG0000010	SIPA1L3	protein_codir	0.76093418	0.00465822	0.01922882
ENSG0000014	CNIH3	protein_codir	0.76046407	0.03305863	0.08767806
ENSG0000027	RP11-497G19	lncRNA	0.76024364	0.6903136	NA
ENSG0000028	RP11-20O22.1	lncRNA	0.76024354	0.69032044	NA
ENSG0000023	CTA-407F11.8	lncRNA	0.76008504	0.62511004	0.74723186
ENSG0000015	BTG2	protein_codir	0.76005202	0.12642811	0.2402692
ENSG0000018	EFHC2	protein_codir	0.75991116	0.27313959	0.42101208
ENSG0000015	RPIA	protein_codir	0.75987389	5.29E-06	7.47E-05
ENSG0000012	TMPO	protein_codir	0.75983687	0.0006078	0.00383447
ENSG0000015	ZNF837	protein_codir	0.75974739	0.00337787	0.01485619
ENSG0000026	RP11-298D21	lncRNA	0.75961473	0.73771079	NA
ENSG0000023	LINC01873	lncRNA	0.75959981	0.73896191	NA
ENSG0000026	AC061992.2	lncRNA	0.75928182	0.09965178	0.20124165

ENSG0000024	RP11-98D18.3	lncRNA	0.75902924	0.79329129	NA
ENSG0000018	CELF2-AS1	lncRNA	0.75844712	0.64664227	NA
ENSG0000024	PLEKHO2	protein_codir	0.75841353	0.00025286	0.00187087
ENSG0000026	DLGAP1-AS2	lncRNA	0.75747172	0.03418775	0.09000721
ENSG0000025	LINC00491	lncRNA	0.75710667	0.64795782	NA
ENSG0000006	OGFR	protein_codir	0.75709663	2.64E-05	0.00028981
ENSG0000023	RP3-339A18.6	lncRNA	0.75686403	0.69466898	NA
ENSG0000023	ELOA-AS1	lncRNA	0.75681394	0.01543896	0.04875061
ENSG0000018	OR52N1	protein_codir	0.7564517	0.748036	NA
ENSG0000017	DSCAML1	protein_codir	0.75624282	0.31049432	0.46231014
ENSG0000017	CEP19	protein_codir	0.75588726	0.00229037	0.01094014
ENSG0000018	UTS2B	protein_codir	0.75577586	0.07661552	0.16539701
ENSG0000025	CHMP1B	protein_codir	0.75562955	0.00054945	0.00353254
ENSG0000027	RP11-462D18	lncRNA	0.75557752	0.43072198	0.58216497
ENSG0000025	RP11-482D24	lncRNA	0.7555578	0.66218175	NA
ENSG0000010	TLE4	protein_codir	0.75548388	7.09E-05	0.00065623
ENSG0000007	GPR137B	protein_codir	0.75536665	0.00958655	0.03365284
ENSG0000013	PRH2	protein_codir	0.75497252	0.63303051	0.75334149
ENSG0000028	RP5-931K24.3	protein_codir	0.7548856	0.4799088	0.62766745
ENSG0000010	OXT	protein_codir	0.7546694	0.38934041	0.54233953
ENSG0000014	JDP2	protein_codir	0.75462131	0.00093139	0.00540894
ENSG0000027	CTD-3193K9.1	lncRNA	0.75441762	0.33234496	0.48506488
ENSG0000014	ECM1	protein_codir	0.75434016	0.04766472	0.11602181
ENSG0000026	CTD-2538G9.5	lncRNA	0.7542355	0.52239225	0.66481346
ENSG0000013	BST2	protein_codir	0.75419918	0.01283868	0.04221202
ENSG0000027	MMP28	protein_codir	0.75409115	0.02593102	0.07261631
ENSG0000018	C19orf54	protein_codir	0.75340691	0.00010169	0.00088099
ENSG0000016	ALDH16A1	protein_codir	0.75326846	0.00077621	0.0046688
ENSG0000014	HMGCLL1	protein_codir	0.75317315	0.12430479	0.23723731
ENSG0000012	CHST8	protein_codir	0.75316497	0.13306317	0.24950473
ENSG0000012	RREB1	protein_codir	0.7531378	1.44E-07	3.42E-06
ENSG0000025	RP11-989F5.3	lncRNA	0.7530695	0.47558696	0.6238465
ENSG0000012	CD93	protein_codir	0.7530156	0.01326793	0.04330656
ENSG0000026	RP11-680F20.	lncRNA	0.75256886	0.61551306	NA
ENSG0000018	SLC4A5	protein_codir	0.75227626	0.00021145	0.00161601
ENSG0000026	RP11-567M16	lncRNA	0.75223971	0.20669355	0.34496802
ENSG0000017	KDM2A	protein_codir	0.75193582	2.08E-06	3.42E-05
ENSG0000020	RP11-332M2.	lncRNA	0.75168153	0.00027874	0.00203077
ENSG0000012	PAX8	protein_codir	0.75165101	0.08980749	0.18590143
ENSG0000015	ART3	protein_codir	0.75095008	0.32997045	0.48249982
ENSG0000024	LINC01197	lncRNA	0.75067444	0.04056652	0.10258627
ENSG0000016	SLC30A7	protein_codir	0.75057986	1.28E-06	2.27E-05
ENSG0000017	CFAP46	protein_codir	0.75054373	0.371171	0.5246005
ENSG0000011	STK38	protein_codir	0.75035315	2.37E-05	0.00026404
ENSG0000027	RP11-1046B1(	lncRNA	0.74974683	0.33470193	0.48762882
ENSG0000023	RP11-160E2.1	lncRNA	0.74953456	0.56407625	0.69833196
ENSG0000023	RP11-160E2.1	lncRNA	0.74953456	0.56407625	0.69833196

ENSG0000025	RP11-361H10	lncRNA	0.7493267	0.52839197	0.66988829
ENSG0000015	PSTPIP2	protein_codir	0.74932514	0.0004615	0.00306338
ENSG0000017	AKAP13	protein_codir	0.74920918	5.64E-05	0.00054398
ENSG0000016	AXDND1	protein_codir	0.74912647	0.46036981	0.6109068
ENSG0000013	DNMT1	protein_codir	0.74891956	0.00146021	0.00772771
ENSG0000026	CTA-331P3.1	lncRNA	0.74847047	0.58684097	0.71716064
ENSG0000018	NXPH4	protein_codir	0.74840194	0.0963829	0.196204
ENSG0000018	PTRHD1	protein_codir	0.74839879	4.47E-05	0.00044423
ENSG0000014	DPYS	protein_codir	0.74806829	0.17746731	0.30806656
ENSG0000016	DNAAF2	protein_codir	0.74800056	0.00038103	0.00262838
ENSG0000023	AC073257.2	lncRNA	0.74779333	0.37756663	0.53122053
ENSG0000016	TRAPPC10	protein_codir	0.74777271	0.00014974	0.00122509
ENSG0000000	BAIAP3	protein_codir	0.74774206	0.06929194	0.15380885
ENSG0000012	TNFSF14	protein_codir	0.7476824	0.13705348	0.2547387
ENSG0000019	KIF19	protein_codir	0.74766144	0.08208448	0.17391792
ENSG0000010	SLC1A5	protein_codir	0.7467514	0.00251136	0.01173269
ENSG0000010	CXCL12	protein_codir	0.74660878	0.00938109	0.03312103
ENSG0000010	RHOV	protein_codir	0.74643448	0.51258166	0.65619956
ENSG0000018	TMEM102	protein_codir	0.74619523	0.01326769	0.04330656
ENSG0000013	CENPO	protein_codir	0.74610757	0.0060113	0.02335521
ENSG0000013	RPS15A	protein_codir	0.74608819	9.77E-06	0.00012601
ENSG0000023	LINC02884	lncRNA	0.74597153	0.0761112	0.16472271
ENSG0000013	PHLDA1	protein_codir	0.74588528	0.05074162	0.12189993
ENSG0000000	PDK4	protein_codir	0.74561224	0.14118282	0.26059005
ENSG0000025	NAV2-AS2	lncRNA	0.74547258	0.52749879	0.66906621
ENSG0000013	ANKRD36	protein_codir	0.74474017	0.01790543	0.05444164
ENSG0000021	LIPE-AS1	lncRNA	0.74452395	0.0238188	0.06792021
ENSG0000010	HPN	protein_codir	0.74450692	0.12000025	0.23100131
ENSG0000016	ATXN7	protein_codir	0.74444958	0.00031987	0.00227233
ENSG0000022	RP11-73O6.3	lncRNA	0.74421773	0.14683267	0.26827535
ENSG0000027	CH507-145C2	lncRNA	0.74383079	0.18361858	0.31627971
ENSG0000018	ODF3L2	protein_codir	0.7435672	0.37721863	0.53083907
ENSG0000018	OGFRP1	lncRNA	0.74356478	0.00254531	0.01184122
ENSG0000013	ANO3	protein_codir	0.74323921	0.15997757	0.28564098
ENSG0000028	RP11-848P1.1	lncRNA	0.74321763	0.10728131	0.21256167
ENSG0000026	CTD-2561B21	lncRNA	0.74303808	0.73032855	0.82650811
ENSG0000017	RPS27	protein_codir	0.74284803	6.16E-05	0.00058548
ENSG0000011	MOGS	protein_codir	0.7425866	0.00059961	0.00379497
ENSG0000006	SP100	protein_codir	0.74239624	1.60E-06	2.75E-05
ENSG0000025	RP11-290L1.3	lncRNA	0.74234876	0.28895673	0.43851565
ENSG0000024	LINC00847	lncRNA	0.74202759	0.00625706	0.02413351
ENSG0000019	AKAP17A	protein_codir	0.74195977	0.00103574	0.00588869
ENSG0000014	NKD2	protein_codir	0.74174696	0.10664163	0.21176518
ENSG0000024	RP11-65D17.1	lncRNA	0.74171004	0.69713921	NA
ENSG0000013	SLC46A3	protein_codir	0.74147748	0.01550541	0.04889922
ENSG0000027	RP11-89C3.3	lncRNA	0.74139133	0.53112814	0.67234189
ENSG0000018	IFIT1	protein_codir	0.74136818	0.07723261	0.16633815

ENSG0000027	RP11-96K19.5	lncRNA	0.7413544	0.23165819	0.37437243
ENSG0000010	MAST1	protein_codir	0.74129575	0.32965758	0.48213622
ENSG0000011	CNR1	protein_codir	0.74123523	0.08783043	0.18286421
ENSG0000017	MMRN2	protein_codir	0.7411304	0.01342218	0.04371046
ENSG0000015	C10orf90	protein_codir	0.740934	0.55634168	0.69220042
ENSG0000026	RP13-122B23.	lncRNA	0.74088703	0.12062989	0.23192242
ENSG0000023	KDM5C-IT1	lncRNA	0.74082387	0.59384384	0.72258544
ENSG0000018	BRI3BP	protein_codir	0.7406346	0.01688755	0.05215632
ENSG0000009	FKBP5	protein_codir	0.74040428	0.05272419	0.12520352
ENSG0000016	ALPL	protein_codir	0.74025205	0.27203639	0.4200383
ENSG0000020	RP11-304F15.	lncRNA	0.73989204	0.22700556	0.36916472
ENSG0000027	RP11-49G2.3	lncRNA	0.73988299	0.32997607	0.48249982
ENSG0000017	TNFRSF10C	protein_codir	0.73984413	0.08906129	0.18479552
ENSG0000027	CELF2-DT	lncRNA	0.73974007	0.70422706	NA
ENSG0000022	AC003991.3	lncRNA	0.73933619	0.7457891	NA
ENSG0000014	TRAPPC14	protein_codir	0.73922643	0.00433901	0.01814711
ENSG0000013	PRPH	protein_codir	0.73915297	0.16402254	0.29102999
ENSG0000018	CSF1	protein_codir	0.73888014	0.00010857	0.00093153
ENSG0000014	INA	protein_codir	0.73880931	0.31476352	0.4665834
ENSG0000010	CHKB	protein_codir	0.73876898	0.02653196	0.07389223
ENSG0000026	COMMD3-BN	protein_codir	0.73839978	0.49383734	0.64010262
ENSG0000028	RP11-556N21	lncRNA	0.73799468	0.75546716	NA
ENSG0000026	RP5-882C2.2	lncRNA	0.73799151	0.07583364	0.16423785
ENSG0000015	MIDEAS	protein_codir	0.73787055	2.12E-05	0.00024129
ENSG0000023	RP11-421L21.	lncRNA	0.73767543	0.05010994	0.12072894
ENSG0000016	TMEM170A	protein_codir	0.73714045	1.37E-05	0.00016798
ENSG0000011	BIRC2	protein_codir	0.73713106	1.43E-08	4.76E-07
ENSG0000027	RP11-426L16.	protein_codir	0.73710799	0.43034266	0.58182311
ENSG0000022	LINC01907	lncRNA	0.73701528	0.22088177	0.36167622
ENSG0000012	C10orf95	protein_codir	0.73676237	0.26835219	0.41618774
ENSG0000025	RP11-1012A1	protein_codir	0.7367014	0.52401708	0.66608457
ENSG0000017	MBOAT1	protein_codir	0.73621885	0.00504787	0.02043413
ENSG0000028	RP13-459H7.1	lncRNA	0.7360042	0.54354068	0.68198763
ENSG0000010	CDH23	protein_codir	0.73599551	0.00685434	0.02590189
ENSG0000017	COQ2	protein_codir	0.73596243	0.00158902	0.00825288
ENSG0000017	VWA1	protein_codir	0.73587824	0.05603383	0.13101281
ENSG0000015	CELF3	protein_codir	0.73565399	0.5358618	0.67603814
ENSG0000025	LINC00517	lncRNA	0.73558684	0.40064027	0.55383805
ENSG0000026	RP11-50D9.3	lncRNA	0.73541974	0.70485212	NA
ENSG0000011	SLC12A7	protein_codir	0.73525738	2.33E-05	0.00026115
ENSG0000023	ZNF32-AS2	lncRNA	0.73484117	0.52185002	0.6644308
ENSG0000015	GFRA1	protein_codir	0.73481483	0.07235162	0.15886412
ENSG0000027	RP11-109E12.	lncRNA	0.7346391	0.50830369	0.65272971
ENSG0000025	MIR3179-1	lncRNA	0.73460023	0.5695583	0.70324574
ENSG0000020	TECPR1	protein_codir	0.73457809	0.0013595	0.00729821
ENSG0000027	RP5-894A10.6	lncRNA	0.73443864	0.02459876	0.06971964
ENSG0000016	IRF2	protein_codir	0.73404137	3.96E-06	5.85E-05

ENSG0000025	MAPK10-AS1	lncRNA	0.73388502	0.54134134	0.68015502
ENSG000000(	HEATR5B	protein_codir	0.73336088	8.49E-07	1.60E-05
ENSG000001€	PCSK9	protein_codir	0.73324366	0.0719382	0.15820607
ENSG0000012	TAS2R3	protein_codir	0.73323486	0.44110472	0.59208399
ENSG0000025	SLC22A18AS	protein_codir	0.73303443	0.19436024	0.3298384
ENSG0000007	SEMA3C	protein_codir	0.73301539	0.09536987	0.19462701
ENSG000000(	MYCBP2	protein_codir	0.73261099	0.00012491	0.00105077
ENSG0000014	CSRNP1	protein_codir	0.7324995	0.12683978	0.2408273
ENSG0000013	SETDB2	protein_codir	0.73248904	0.00017839	0.00140937
ENSG000001(	ACR	protein_codir	0.73178576	0.11040651	0.2172731
ENSG0000028	RP4-545L17.1	lncRNA	0.73162351	0.39404149	0.54701578
ENSG0000013	MEN1	protein_codir	0.73158015	0.00151925	0.0079584
ENSG000002(	RFPL3S	lncRNA	0.73128113	0.16131993	0.28738753
ENSG000002(	LINC01556	lncRNA	0.73108744	0.71724985	NA
ENSG000002(	NT5M	protein_codir	0.73091873	0.02281928	0.06568034
ENSG0000028	UPK3BL2	protein_codir	0.73057494	0.14514291	0.26606804
ENSG000001(	SLC9A3R1	protein_codir	0.73037394	0.0119385	0.03991542
ENSG0000023	KLF7-IT1	lncRNA	0.73032968	0.05091967	0.1221536
ENSG0000024	AC010226.4	lncRNA	0.73024574	0.01173569	0.03946186
ENSG000001(	PCOLCE	protein_codir	0.73012327	0.00901289	0.03210419
ENSG0000025	EHD4-AS1	lncRNA	0.72992777	0.70628613	NA
ENSG0000017	UCP3	protein_codir	0.72983112	0.03531853	0.09220205
ENSG0000022	RP4-622L5.7	lncRNA	0.72979865	0.14674918	0.26817601
ENSG0000018	SEMA4B	protein_codir	0.72978411	0.00380792	0.01635327
ENSG0000018	GINS3	protein_codir	0.72952791	0.03640541	0.09434391
ENSG0000022	PRR29	protein_codir	0.72952326	0.05200047	0.12388968
ENSG0000028	CTD-2547L24.	lncRNA	0.72947772	0.53262692	0.67352921
ENSG0000022	FAM229A	protein_codir	0.72945245	0.05748886	0.13348821
ENSG0000023	RPS28	protein_codir	0.72938017	0.00018197	0.00143148
ENSG000001(	HOXA5	protein_codir	0.72930338	0.0623376	0.14221233
ENSG0000014	SNX30	protein_codir	0.72911495	0.00595101	0.02318493
ENSG0000027	GS1-393G12.1	protein_codir	0.72881783	0.43023633	0.58176479
ENSG0000015	ABHD3	protein_codir	0.72872059	0.00682226	0.02579125
ENSG0000022	RP11-383C5.3	lncRNA	0.72869362	0.75472966	NA
ENSG0000015	BACH1	protein_codir	0.7286849	0.00893005	0.03186255
ENSG0000025	CTB-131B5.5	lncRNA	0.72846076	0.13049494	0.24584068
ENSG0000007	RAD18	protein_codir	0.72844895	2.71E-06	4.23E-05
ENSG0000019	GM2A	protein_codir	0.72826143	0.00208944	0.01015848
ENSG0000028	CH507-513H4	lncRNA	0.72747561	0.02621574	0.07324285
ENSG0000028	CH507-513H4	lncRNA	0.72747561	0.02621574	0.07324285
ENSG0000028	CH507-513H4	lncRNA	0.72747561	0.02621574	0.07324285
ENSG0000026	Z69720.2	lncRNA	0.72724013	0.42068752	0.57286345
ENSG0000013	PCED1A	protein_codir	0.72701125	0.00739545	0.02749526
ENSG0000028	RP11-169N13	protein_codir	0.72699589	0.79774549	0.87423259
ENSG0000028	CTC-459F4.9	lncRNA	0.72581359	0.09140498	0.18843153
ENSG0000018	CA13	protein_codir	0.72570152	0.04126568	0.10398304
ENSG0000025	LINC02588	lncRNA	0.7256029	0.67183638	0.7835811

ENSG0000017	SLC35G5	protein_codir	0.7255983	0.32692901	0.47918591
ENSG0000025	RP1-197B17.3	lncRNA	0.72553481	0.07632138	0.16503832
ENSG0000025	TUBB3	protein_codir	0.72547062	0.16270648	0.28914896
ENSG0000023	RP11-34P13.7	lncRNA	0.72510268	0.14952011	0.27174742
ENSG0000016	NEMP1	protein_codir	0.72500327	6.28E-05	0.00059323
ENSG0000014	HIGD2A	protein_codir	0.724858	4.03E-07	8.34E-06
ENSG0000026	RP11-127I20.5	lncRNA	0.72485698	0.79432025	NA
ENSG0000026	MIA-RAB4B	protein_codir	0.72479614	0.7238538	0.82180376
ENSG0000026	RP11-388M20	lncRNA	0.7245048	0.70552032	NA
ENSG0000008	GP6	protein_codir	0.72444146	0.2438065	0.38888517
ENSG0000016	BSCL2	protein_codir	0.72419771	0.02331103	0.06676353
ENSG0000015	CFAP161	protein_codir	0.72418747	0.23984573	0.38429419
ENSG0000018	TMEM105	lncRNA	0.72417825	0.59516858	0.72365541
ENSG0000011	SIPA1L2	protein_codir	0.72412474	0.00166015	0.00853144
ENSG0000018	ZNF530	protein_codir	0.72402267	0.00567232	0.02234307
ENSG0000023	HCG24	lncRNA	0.72382554	0.75834861	NA
ENSG0000023	RP1-159A19.4	lncRNA	0.72381835	0.75847408	NA
ENSG0000022	ADIPOQ-AS1	lncRNA	0.72364591	0.7615069	NA
ENSG0000028	POLR2J3	protein_codir	0.72348357	0.06006805	0.13809552
ENSG0000027	LINC01730	lncRNA	0.72339224	0.36366677	0.51765071
ENSG0000017	ABO	protein_codir	0.72329916	0.10225572	0.20518268
ENSG0000028	RP5-1171I10.8	protein_codir	0.72284919	0.54509667	0.6830872
ENSG0000021	RP3-382I10.7	protein_codir	0.72273344	0.44996834	0.60082792
ENSG0000014	EIF4A3	protein_codir	0.72244759	0.00208653	0.0101496
ENSG0000023	PITRM1-AS1	lncRNA	0.72193824	0.06353657	0.14438704
ENSG0000024	AF011889.5	protein_codir	0.72166008	0.04250601	0.10648671
ENSG0000022	AC015971.2	lncRNA	0.72159588	0.60551044	NA
ENSG0000026	RP11-457M11	lncRNA	0.72159513	0.23057679	0.37308324
ENSG0000010	PALD1	protein_codir	0.72151519	0.00448952	0.01864579
ENSG0000012	G0S2	protein_codir	0.72150802	0.11700125	0.22664992
ENSG0000020	COL5A2	protein_codir	0.72148488	0.07433719	0.16191027
ENSG0000020	COL6A6	protein_codir	0.72129881	0.28794998	0.43763707
ENSG0000014	MMS22L	protein_codir	0.72128125	0.0005576	0.00357164
ENSG0000026	RP11-74C13.4	lncRNA	0.72116598	0.50919621	0.65347749
ENSG0000010	RP2	protein_codir	0.72104301	0.00350968	0.01531395
ENSG0000025	FAR1-IT1	lncRNA	0.72096469	0.33740516	0.49027412
ENSG0000000	C1orf112	protein_codir	0.72068318	3.39E-06	5.13E-05
ENSG0000012	FLRT1	protein_codir	0.72032231	0.26599028	0.41354638
ENSG0000016	RET	protein_codir	0.71996784	0.10220618	0.20512628
ENSG0000022	AP001469.7	lncRNA	0.71958005	0.59541887	NA
ENSG0000026	RP11-461A8.4	lncRNA	0.71951899	0.20976324	0.3488896
ENSG0000022	LINC00365	lncRNA	0.71933867	0.68193557	0.79078108
ENSG0000025	CTD-2589M5.	lncRNA	0.71920707	0.57893424	NA
ENSG0000028	RASSF1-AS1	lncRNA	0.71914265	0.20666731	0.34494508
ENSG0000011	LOX	protein_codir	0.71897692	0.08828754	0.18361908
ENSG0000018	BCL2L15	protein_codir	0.71839467	0.07194309	0.15820607
ENSG0000014	PRDM15	protein_codir	0.71829786	0.0153166	0.04845514

ENSG0000019	EPHB4	protein_codir	0.71826544	0.00595153	0.02318493
ENSG0000010	DHX58	protein_codir	0.71824025	0.00922569	0.03270196
ENSG0000026	AC003005.4	protein_codir	0.71815254	0.09101105	0.18775957
ENSG0000025	MIR3180-1	lncRNA	0.71814439	0.29315357	0.44327722
ENSG0000011	IFIH1	protein_codir	0.71798259	0.01590766	0.0499
ENSG0000018	TMC3	protein_codir	0.7178661	0.46538167	0.61513408
ENSG0000014	ARHGEF19	protein_codir	0.71773273	0.01560481	0.04912856
ENSG0000017	ZWILCH	protein_codir	0.71770815	6.85E-05	0.00063782
ENSG0000026	C10orf95-AS1	lncRNA	0.71767775	0.0605709	0.13895084
ENSG0000008	NLK	protein_codir	0.7173748	6.17E-05	0.00058596
ENSG0000007	CDH19	protein_codir	0.71733265	0.28505943	0.43465505
ENSG0000027	RP11-506H21	lncRNA	0.71732356	0.40264436	0.55564904
ENSG0000014	RGL1	protein_codir	0.71726961	0.01377261	0.04459025
ENSG0000018	SRL	protein_codir	0.71707369	0.12889768	0.24370958
ENSG0000024	CEBPA	protein_codir	0.71696064	0.08947774	0.18545513
ENSG0000010	CABP7	protein_codir	0.71666909	0.27238978	0.42032548
ENSG0000013	GGH	protein_codir	0.71648458	0.00384632	0.01648965
ENSG0000022	HMSD	protein_codir	0.7162991	0.26865315	0.41635185
ENSG0000024	RP4-539M6.1	protein_codir	0.71626714	0.3264487	0.4787542
ENSG0000003	DAPK2	protein_codir	0.71591578	0.01625439	0.05071694
ENSG0000017	ZIK1	protein_codir	0.71583132	0.00112354	0.00627251
ENSG0000025	CTD-2651B20	lncRNA	0.7157439	0.15185891	0.27472798
ENSG0000010	DDX58	protein_codir	0.71559912	0.01181188	0.03964086
ENSG0000018	AC097382.5	lncRNA	0.71551674	0.53647842	0.67663062
ENSG0000028	AC004067.6	protein_codir	0.71541098	0.53717368	0.67713646
ENSG0000018	CFAP73	protein_codir	0.71537386	0.3643275	0.51832314
ENSG0000013	BCO1	protein_codir	0.71514549	0.5235563	0.66567468
ENSG0000011	PRR4	protein_codir	0.71506637	0.11152418	0.21889552
ENSG0000012	FAM117A	protein_codir	0.7150225	0.00022403	0.00169481
ENSG0000008	PHACTR3	protein_codir	0.71476837	0.43764432	0.58889877
ENSG0000016	WDR88	protein_codir	0.71472035	0.71319435	NA
ENSG0000025	RP11-1082L8.	lncRNA	0.71465262	0.12333641	0.2359212
ENSG0000016	LY6E	protein_codir	0.71455219	0.00275377	0.01260929
ENSG0000023	C19orf81	protein_codir	0.71437487	0.32533404	0.477591
ENSG0000019	HES5	protein_codir	0.71432019	0.33386052	0.48676244
ENSG0000010	AQP8	protein_codir	0.71422941	0.47002712	0.61914568
ENSG0000013	SEC14L4	protein_codir	0.71402643	0.45200229	0.60254773
ENSG0000011	WIPF1	protein_codir	0.71366569	0.00073744	0.00448737
ENSG0000016	HFM1	protein_codir	0.71363108	0.16331304	0.29002161
ENSG0000006	TTC7A	protein_codir	0.7136244	3.50E-06	5.25E-05
ENSG0000013	TRIM22	protein_codir	0.71360421	0.00724803	0.02703564
ENSG0000027	CTD-3216D2.5	lncRNA	0.71349549	0.69692879	0.80171483
ENSG0000028	RP1-76B20.16	lncRNA	0.71332803	0.66744003	NA
ENSG0000027	RP4-657D16.6	lncRNA	0.71319536	0.47379903	0.6222104
ENSG0000017	RIMKLA	protein_codir	0.71319319	0.04555244	0.11225158
ENSG0000026	RP11-96D1.9	lncRNA	0.71315691	0.24326597	0.3883234
ENSG0000013	DCAF15	protein_codir	0.71301151	0.00113253	0.00630783

ENSG0000025	RP11-73E17.2	lncRNA	0.712648	0.00290018	0.01312731
ENSG0000016	CNNM3	protein_codir	0.71253969	1.02E-05	0.00013072
ENSG0000022	TEX22	protein_codir	0.7121616	0.17422423	0.30419239
ENSG0000008	TUT7	protein_codir	0.71192008	1.30E-05	0.00016019
ENSG0000018	ACSM5	protein_codir	0.71138939	0.0822429	0.17414513
ENSG0000016	ISCA2	protein_codir	0.71123184	0.00020467	0.00157422
ENSG0000012	FRMD8	protein_codir	0.71113897	1.42E-05	0.00017279
ENSG0000018	ARSI	protein_codir	0.71106314	0.09195873	0.18932282
ENSG0000018	FHIT	protein_codir	0.71105378	0.0126349	0.04167871
ENSG0000013	BARX1	protein_codir	0.71100439	0.25992621	0.40702812
ENSG0000014	ITPKB	protein_codir	0.71081288	2.86E-07	6.21E-06
ENSG0000013	ENPP2	protein_codir	0.71043929	0.03274457	0.08701223
ENSG0000023	RP4-569M23.	lncRNA	0.71009952	0.33436006	0.48725927
ENSG0000013	COL5A1	protein_codir	0.70999476	0.05527637	0.12962585
ENSG0000023	GPX1	protein_codir	0.70987649	0.00599084	0.02329642
ENSG0000010	KLF5	protein_codir	0.70982069	0.04198509	0.10543993
ENSG0000023	CTC-490G23.2	lncRNA	0.70970806	0.71387453	NA
ENSG0000025	RP11-182J1.5	lncRNA	0.70970623	0.52342208	0.6656346
ENSG0000015	ADAM17	protein_codir	0.70960578	2.23E-05	0.00025156
ENSG0000011	APH1A	protein_codir	0.70947148	3.88E-08	1.11E-06
ENSG0000010	DNM1	protein_codir	0.70943483	0.01545963	0.04878826
ENSG0000015	KCNE4	protein_codir	0.70923875	0.04207342	0.10561908
ENSG0000022	RP11-432J24.	lncRNA	0.70884486	0.50912081	0.65343486
ENSG0000016	GLYCTK	protein_codir	0.70881319	0.02603912	0.07287472
ENSG0000017	STAT2	protein_codir	0.70871924	0.00499742	0.02025366
ENSG0000024	ARHGAP8	protein_codir	0.70850622	0.10028577	0.20213771
ENSG0000023	AC006372.4	lncRNA	0.70847051	0.66068961	0.77499546
ENSG0000026	RP11-314A20	lncRNA	0.70846636	0.81173677	NA
ENSG0000019	ZNF100	protein_codir	0.70842213	0.02163004	0.06298237
ENSG0000020	VIT	protein_codir	0.70837124	0.18164093	0.31363607
ENSG0000014	USP6NL	protein_codir	0.70821575	6.99E-05	0.00064714
ENSG0000016	MYO1A	protein_codir	0.70819418	0.23770635	0.38177877
ENSG0000019	ZNF860	protein_codir	0.70776495	0.05635925	0.13160658
ENSG0000028	RP11-244H3.5	protein_codir	0.70771884	0.02723621	0.0754452
ENSG0000026	LINC01841	lncRNA	0.7076756	0.39039414	0.54346297
ENSG0000018	CDNF	protein_codir	0.70710537	0.01789243	0.05441412
ENSG0000020	FBXO48	protein_codir	0.70699147	0.00052836	0.00342007
ENSG0000024	PLA2G4B	protein_codir	0.70689208	0.12903848	0.24381353
ENSG0000019	ENTPD4	protein_codir	0.7068889	0.00013389	0.00111321
ENSG0000025	RP11-417L19.	lncRNA	0.70685804	0.57318322	0.70615641
ENSG0000010	ZBED4	protein_codir	0.70672403	0.00100769	0.0057648
ENSG0000012	OPRL1	protein_codir	0.70660258	0.04329918	0.1079061
ENSG0000016	UCN	protein_codir	0.70648042	0.12228419	0.23443658
ENSG0000012	XRCC3	protein_codir	0.70623521	0.08720626	0.18201745
ENSG0000018	SIRT7	protein_codir	0.70416759	0.0137019	0.04442381
ENSG0000027	RP11-145M9.	lncRNA	0.70414803	0.51108025	0.65488456
ENSG0000005	RRP12	protein_codir	0.70397658	0.04266221	0.10674776

ENSG0000008	ALG6	protein_codir	0.70386832	0.00365148	0.01579061
ENSG0000015	RAD9B	protein_codir	0.70353751	0.02198064	0.06379559
ENSG0000012	PNKD	protein_codir	0.70352595	0.00061721	0.00388408
ENSG0000026	MTRNR2L12	protein_codir	0.70349722	0.51117408	0.6549744
ENSG0000013	LMX1B	protein_codir	0.70342177	0.19370933	0.32910449
ENSG0000027	ARHGEF2-AS2	lncRNA	0.70304106	0.17942254	0.31073807
ENSG0000022	LINC01165	lncRNA	0.7028371	0.42732236	0.5790719
ENSG0000022	RP4-575N6.4	lncRNA	0.70266324	0.31348497	0.4651832
ENSG0000017	SNTG2	protein_codir	0.7026491	0.23156036	0.37430488
ENSG0000027	RP11-379F4.7	lncRNA	0.70247821	0.1672477	0.29541354
ENSG0000010	APOL1	protein_codir	0.70243453	0.01212712	0.04041887
ENSG0000013	TRMO	protein_codir	0.70200907	0.00042356	0.00285611
ENSG0000019	PTPN1	protein_codir	0.70199266	0.01502236	0.04778589
ENSG0000027	RP11-394O2.3	lncRNA	0.70197797	0.10532949	0.20975689
ENSG0000023	RNASEH2B-AS	lncRNA	0.70188634	0.18134085	0.313255
ENSG0000027	RP11-74J13.9	lncRNA	0.70162643	0.43698783	0.58821599
ENSG0000026	LINC02367	lncRNA	0.70082702	0.2901614	0.43987916
ENSG0000028	RP11-312A15	lncRNA	0.70077369	0.53443108	0.67497308
ENSG0000023	LINC01704	lncRNA	0.7004485	0.37142304	0.52484863
ENSG0000018	FANCB	protein_codir	0.70038475	0.0230257	0.06613664
ENSG0000016	CASP3	protein_codir	0.7003747	0.00033713	0.00237662
ENSG0000011	ARRDC3	protein_codir	0.70031487	0.04097326	0.10338768
ENSG0000025	RP11-284M14	lncRNA	0.70027172	0.04882072	0.11831431
ENSG0000022	ZNF492	protein_codir	0.70013713	0.22964517	0.372099
ENSG0000011	MAPKAPK3	protein_codir	0.70002867	0.00232068	0.01105123
ENSG0000010	RAPGEF1	protein_codir	0.69995394	0.00133345	0.00719756
ENSG0000015	ZFP36L2	protein_codir	0.69994766	3.05E-05	0.00032448
ENSG0000011	SYT10	protein_codir	0.69978346	0.5995099	0.72726652
ENSG0000017	CLVS1	protein_codir	0.69962665	0.37960329	0.53294581
ENSG0000020	SPIRE2	protein_codir	0.69961701	0.05899721	0.13614361
ENSG0000001	DDX11	protein_codir	0.69946796	0.12706529	0.2411063
ENSG0000016	NPIPB3	protein_codir	0.69932023	0.13043079	0.24577012
ENSG0000028	RP11-295P9.1	protein_codir	0.69910806	0.36939855	0.52289811
ENSG0000022	AC004951.6	lncRNA	0.69879192	0.11217627	0.21983174
ENSG0000027	H2BC8	protein_codir	0.69877311	0.14400072	0.26421976
ENSG0000020	APOM	protein_codir	0.69876705	0.05521879	0.12954582
ENSG0000020	RP11-497E19.	lncRNA	0.69876401	0.10727964	0.21256167
ENSG0000027	AC006273.4	lncRNA	0.698715	0.71779152	NA
ENSG0000027	RP11-386J22.	lncRNA	0.69854273	0.38382822	0.53713003
ENSG0000000	ABCB5	protein_codir	0.69820353	0.34364386	0.49677815
ENSG0000009	PABPC4	protein_codir	0.69817709	2.29E-05	0.00025678
ENSG0000025	TRIM34	protein_codir	0.69762452	0.0081479	0.02965706
ENSG0000028	AP000331.1	lncRNA	0.69724175	0.56108527	0.69593925
ENSG0000011	CDKN1B	protein_codir	0.69628153	0.00056775	0.00362236
ENSG0000018	UPP1	protein_codir	0.69626504	0.06299453	0.14341484
ENSG0000010	RUNDC3B	protein_codir	0.69625119	0.0645256	0.14616559
ENSG0000022	AP001347.6	lncRNA	0.69623101	0.24085803	0.38540013

ENSG0000011	TLX2	protein_codir	0.69618606	0.43973371	0.59073176
ENSG0000017	FOXN2	protein_codir	0.69614071	8.97E-05	0.000795
ENSG0000028	ANAPC1P2	lncRNA	0.69610193	0.00013005	0.0010871
ENSG0000025	RP5-1021I20.1	lncRNA	0.6960908	0.08890534	0.18462898
ENSG0000014	LYST	protein_codir	0.69581116	2.57E-05	0.0002827
ENSG0000024	RP11-184M15	lncRNA	0.69580269	0.30818542	0.45993847
ENSG0000011	ASIC1	protein_codir	0.69547591	0.11336603	0.22140926
ENSG0000005	NPFFR2	protein_codir	0.69482586	0.42117152	0.57317471
ENSG0000028	RP13-465B17.	lncRNA	0.69478288	0.65675546	0.7718665
ENSG0000028	RP11-762B21.	lncRNA	0.69473107	0.32305926	0.47527948
ENSG0000019	ZNF431	protein_codir	0.69462345	0.00911484	0.0324005
ENSG0000023	LINC01001	lncRNA	0.69458056	0.03896422	0.09942643
ENSG0000016	SLC9B1	protein_codir	0.69443734	0.09514853	0.19432139
ENSG0000023	RP11-338C15.	lncRNA	0.6943485	0.55883542	0.69442238
ENSG0000025	RP11-43A14.1	lncRNA	0.69399884	0.64863255	NA
ENSG0000024	MARS2	protein_codir	0.69394408	0.01622698	0.05065467
ENSG0000013	BAAT	protein_codir	0.69390987	0.35010555	0.50364142
ENSG0000014	EPHA2	protein_codir	0.6938729	0.00195949	0.00966794
ENSG0000025	LINC00485	lncRNA	0.69353452	0.7378391	0.83217429
ENSG0000024	CFAP61-AS1	lncRNA	0.69344768	0.5751925	0.70777942
ENSG0000024	MIR4300HG	lncRNA	0.69313709	0.6048937	NA
ENSG0000012	BATF3	protein_codir	0.69287303	0.01815107	0.05500836
ENSG0000027	XXbac-B135H	lncRNA	0.69244863	0.44974898	0.60059728
ENSG0000011	CDX1	protein_codir	0.69201754	0.2086353	0.34745294
ENSG0000011	PTCH2	protein_codir	0.69181024	0.04862817	0.11793046
ENSG0000018	POMK	protein_codir	0.69179286	0.00019337	0.00150276
ENSG0000025	RP11-881M11	lncRNA	0.69174881	0.27624636	0.42445062
ENSG0000027	RP11-131L23.	lncRNA	0.69160566	0.54660601	0.68440356
ENSG0000028	RP11-697E2.1	protein_codir	0.69151224	0.32548941	0.4777604
ENSG0000006	RORA	protein_codir	0.69140576	0.00181625	0.00911349
ENSG0000026	RP11-848P1.2	lncRNA	0.6913659	0.08312891	0.17546966
ENSG0000023	LINC01141	lncRNA	0.69135874	0.57149694	0.70479733
ENSG0000017	ACSM6	protein_codir	0.69134355	0.66919506	0.78143015
ENSG0000019	TOP1	protein_codir	0.69127029	8.79E-05	0.0007828
ENSG0000018	ZFP36L1	protein_codir	0.69072906	0.03159885	0.08467387
ENSG0000014	BCL10	protein_codir	0.69072438	0.00032129	0.00227923
ENSG0000019	C9orf163	lncRNA	0.69047437	0.51718856	0.6603221
ENSG0000012	B9D2	protein_codir	0.69037145	0.00034774	0.00243521
ENSG0000021	XXcos-LUCA1	lncRNA	0.6903046	0.41468646	0.56785523
ENSG0000016	CYB561A3	protein_codir	0.69029526	0.00872463	0.03126001
ENSG0000028	RP11-104C10.	lncRNA	0.69006234	0.6826146	NA
ENSG0000025	CCDC71L	protein_codir	0.68994981	0.0758631	0.16427587
ENSG0000028	SMIM38	protein_codir	0.68973871	0.15634321	0.28085849
ENSG0000026	RP11-116O18	lncRNA	0.68961709	0.09196071	0.18932282
ENSG0000014	PARP1	protein_codir	0.68952833	0.00261666	0.01210994
ENSG0000011	MCM9	protein_codir	0.68949932	0.00019356	0.00150337
ENSG0000027	CTA-223H9.9	lncRNA	0.68947216	0.54940166	0.68668937

ENSG0000027	RP11-362K14.	lncRNA	0.689068	0.02704165	0.07502666
ENSG0000027	RP11-46J23.1	lncRNA	0.68901501	0.19138171	0.32597007
ENSG0000011	ST8SIA1	protein_codir	0.68887385	0.04692771	0.1146218
ENSG0000023	LINC01659	lncRNA	0.68851103	0.76774108	NA
ENSG0000028	LINC02204	lncRNA	0.68838888	0.15708157	0.28189155
ENSG0000028	CTD-2021K4.1	protein_codir	0.68830082	0.07660123	0.16539258
ENSG0000026	FAM157C	lncRNA	0.68824489	0.00486101	0.01984057
ENSG0000010	SPATA6L	protein_codir	0.68821051	0.02492578	0.0704406
ENSG0000026	RP11-886H22	protein_codir	0.68715634	0.76147256	NA
ENSG0000010	CDK5RAP3	protein_codir	0.68710701	0.03078717	0.08301685
ENSG0000014	NRBF2	protein_codir	0.68700161	6.92E-06	9.39E-05
ENSG0000028	RP11-392A14	lncRNA	0.68683811	0.31453457	0.46643999
ENSG0000010	TIMP1	protein_codir	0.68595088	0.08101269	0.17227984
ENSG0000016	TM4SF4	protein_codir	0.68589902	0.56749997	0.70130274
ENSG0000024	AP000295.9	protein_codir	0.68588125	0.26073481	0.40801758
ENSG0000016	ZDHHC12	protein_codir	0.68533635	0.00555232	0.0219927
ENSG0000026	RP11-400F19.	lncRNA	0.68526144	0.60274799	0.72969097
ENSG0000016	GRIK2	protein_codir	0.68521027	0.21339489	0.35318204
ENSG0000014	WARS1	protein_codir	0.68519228	0.00209371	0.01017389
ENSG0000018	ARAP1	protein_codir	0.68490441	0.00051069	0.00332361
ENSG0000016	POC1A	protein_codir	0.68466574	0.0178147	0.05426736
ENSG0000025	RP11-443B7.3	lncRNA	0.68466077	0.22010575	0.3609405
ENSG0000014	GAS2	protein_codir	0.68424705	0.03664796	0.09484789
ENSG0000012	BICC1	protein_codir	0.68404369	0.02710853	0.0751395
ENSG0000028	CTC-338M12.	lncRNA	0.68389514	0.51992108	0.66273861
ENSG0000028	RP11-704O17	lncRNA	0.68369623	0.61507294	0.73907224
ENSG0000013	CBLN3	protein_codir	0.68309237	0.05289731	0.12547437
ENSG0000024	LINC01550	lncRNA	0.68286689	0.0586736	0.13558945
ENSG0000028	RP11-449K6.5	protein_codir	0.68265055	0.53142688	0.67254853
ENSG0000023	RB1-DT	lncRNA	0.68260471	0.19523152	0.33093744
ENSG0000015	COLEC12	protein_codir	0.68255666	0.02710526	0.0751395
ENSG0000010	ELL	protein_codir	0.68224046	0.00146291	0.00773687
ENSG0000027	DUSP14	protein_codir	0.68194947	0.03077766	0.08301148
ENSG0000000	GCLC	protein_codir	0.68180024	0.00020482	0.00157492
ENSG0000028	RP11-29H23.8	lncRNA	0.68112072	0.0306239	0.08273815
ENSG0000013	N4BP2L1	protein_codir	0.680566	8.93E-05	0.00079257
ENSG0000013	FAM117B	protein_codir	0.67977846	0.00614792	0.02379564
ENSG0000025	CTD-2517M22	lncRNA	0.67971474	0.10040758	0.2023265
ENSG0000012	LRIF1	protein_codir	0.67965303	6.20E-05	0.00058769
ENSG0000014	TRIM50	protein_codir	0.67950969	0.1116947	0.21916792
ENSG0000011	NANOG	protein_codir	0.6792964	0.53514638	0.6755367
ENSG0000016	HSPB3	protein_codir	0.67915466	0.44984454	0.60069165
ENSG0000017	ZNF266	protein_codir	0.67882099	0.02276225	0.06556403
ENSG0000018	C3orf62	protein_codir	0.67842501	0.0321892	0.08580888
ENSG0000020	AC012358.7	lncRNA	0.6784237	0.50258596	0.64733372
ENSG0000024	IL10RB	protein_codir	0.67839354	6.70E-05	0.00062617
ENSG0000016	IKBIP	protein_codir	0.67830383	0.00295691	0.01333384

ENSG0000011	C6orf62	protein_codir	0.67828692	0.00107489	0.0060626
ENSG0000028	RP11-347L15.	lncRNA	0.67824269	0.66944554	NA
ENSG0000025	NOX5	protein_codir	0.67787653	0.05279743	0.12532635
ENSG0000019	ZNF418	protein_codir	0.6776791	0.04274271	0.10688852
ENSG0000009	NRCAM	protein_codir	0.67744845	0.14536316	0.26631272
ENSG0000011	NID1	protein_codir	0.67715364	0.03640926	0.09434436
ENSG0000014	PNRC1	protein_codir	0.67708343	0.02939503	0.08021842
ENSG0000016	RAB8B	protein_codir	0.67611467	0.00010896	0.00093404
ENSG0000019	ZNF700	protein_codir	0.67601639	0.05253133	0.12489586
ENSG0000012	KLF16	protein_codir	0.67599262	0.02041298	0.06021001
ENSG0000014	CABLES2	protein_codir	0.67598135	8.79E-05	0.00078247
ENSG0000005	USP36	protein_codir	0.67588062	0.00078061	0.00468908
ENSG0000013	SELENOI	protein_codir	0.67586218	7.74E-05	0.00070326
ENSG0000010	VSIR	protein_codir	0.67547891	0.00455752	0.01886952
ENSG0000011	NKTR	protein_codir	0.67524577	0.0312967	0.08406252
ENSG0000001	ETV7	protein_codir	0.67518386	0.00973834	0.03406445
ENSG0000013	WASHC4	protein_codir	0.67515504	0.00099706	0.00572057
ENSG0000016	PCSK7	protein_codir	0.67480072	0.01638101	0.05095084
ENSG0000010	PCSK1N	protein_codir	0.67464741	0.27531097	0.42346099
ENSG0000016	HHIP	protein_codir	0.67424046	0.17610631	0.30640431
ENSG0000018	TPRG1	protein_codir	0.67375988	0.01206288	0.04023874
ENSG0000010	SEZ6L	protein_codir	0.67363279	0.2067124	0.34497864
ENSG0000017	PODN	protein_codir	0.67360811	0.142151	0.26174737
ENSG0000025	RP11-351I24.1	lncRNA	0.67333149	0.60338235	0.73008479
ENSG0000022	RABGAP1L-DT	lncRNA	0.67329511	0.2950924	0.44550162
ENSG0000015	B4GALT3	protein_codir	0.67329025	0.00103808	0.00589832
ENSG0000006	BCL3	protein_codir	0.67314162	0.01951536	0.05820628
ENSG0000010	MED26	protein_codir	0.67313078	1.14E-05	0.00014335
ENSG0000016	PPP2R3B	protein_codir	0.67293272	0.01573878	0.04946575
ENSG0000022	CROCC2	protein_codir	0.67285963	0.35321445	0.5067676
ENSG0000010	BPI	protein_codir	0.6726208	0.14989394	0.27217608
ENSG0000013	MPP1	protein_codir	0.67256798	0.00286041	0.01298816
ENSG0000014	CACNG8	protein_codir	0.67253959	0.17105107	0.30017348
ENSG0000011	DCLRE1B	protein_codir	0.67249529	0.00066761	0.00414461
ENSG0000027	RP11-329B9.3	lncRNA	0.67189675	0.2195139	0.36028678
ENSG0000028	LINCADL	lncRNA	0.67181318	0.42924898	0.5808847
ENSG0000023	AC007036.4	lncRNA	0.67181135	0.52945062	0.67083013
ENSG0000013	IRAK2	protein_codir	0.6717671	0.00718546	0.02685919
ENSG0000017	PPFIA3	protein_codir	0.67172373	0.00655937	0.02502207
ENSG0000014	USP3	protein_codir	0.67172354	6.60E-06	9.01E-05
ENSG0000028	CTD-2143A15	lncRNA	0.67168165	0.45624125	0.60691534
ENSG0000016	CCDC28B	protein_codir	0.6716257	0.01387557	0.04484476
ENSG0000015	PCDH15	protein_codir	0.67154367	0.5441121	0.68235463
ENSG0000012	ARHGEF6	protein_codir	0.67145583	0.00023379	0.0017528
ENSG0000011	HPCAL1	protein_codir	0.67118624	0.00109853	0.00616697
ENSG0000025	RP11-30K9.4	lncRNA	0.67105312	0.68115235	0.79034734
ENSG0000020	PCMTD2	protein_codir	0.67077815	2.71E-05	0.00029544

ENSG0000013	MDGA2	protein_codir	0.67059635	0.4737015	0.6221119
ENSG0000025	RP11-605F22.	lncRNA	0.67048262	0.60705954	0.73317765
ENSG0000007	ACER3	protein_codir	0.67010292	1.54E-06	2.67E-05
ENSG0000014	DUOX2	protein_codir	0.67003655	0.22536785	0.36712828
ENSG0000027	RP4-616B8.5	lncRNA	0.66989628	0.47311593	0.62154976
ENSG0000028	CTC-89C10.1	lncRNA	0.66916943	0.70258392	0.80628402
ENSG0000011	PTPN5	protein_codir	0.66911217	0.31946631	0.47157822
ENSG0000016	RACGAP1	protein_codir	0.66885644	0.00342259	0.01500267
ENSG0000022	RP11-207C16.	lncRNA	0.66883089	0.59634499	0.72463919
ENSG0000022	CERS6-AS1	lncRNA	0.66877954	0.52778356	0.66933201
ENSG0000019	MBP	protein_codir	0.66874575	0.00149185	0.00785136
ENSG0000018	SERTM1	protein_codir	0.66867799	0.3843946	0.53765022
ENSG0000018	ADGRG3	protein_codir	0.66864639	0.22055439	0.36130295
ENSG0000026	RP11-395N3.1	lncRNA	0.6684714	0.66040255	NA
ENSG0000017	TMEM9B	protein_codir	0.66836591	6.14E-06	8.50E-05
ENSG0000027	RP3-449M8.9	lncRNA	0.66816176	0.47161657	0.62040626
ENSG0000011	RPS12	protein_codir	0.66781791	0.00021004	0.00160874
ENSG0000028	RP11-594N15	lncRNA	0.66745237	0.44056526	0.59153237
ENSG0000013	RNASEH2B	protein_codir	0.66742308	0.00056187	0.00359563
ENSG0000013	QRSL1	protein_codir	0.66741703	0.00476016	0.01952868
ENSG0000016	SMC6	protein_codir	0.66721282	1.59E-05	0.00018883
ENSG0000025	RP11-361D15	lncRNA	0.66699762	0.3859286	0.53913259
ENSG0000019	SLC39A10	protein_codir	0.66625893	0.00031076	0.00221733
ENSG0000024	RP11-1079K10	lncRNA	0.66595824	0.16849804	0.29691948
ENSG0000022	AC004540.4	lncRNA	0.66592016	0.36219922	0.51606616
ENSG0000024	ARHGDIG	protein_codir	0.6657701	0.21636931	0.35669812
ENSG0000011	GNPTAB	protein_codir	0.66544221	0.0019577	0.00966084
ENSG0000013	TTLL9	protein_codir	0.66509768	0.13724151	0.25498524
ENSG0000012	LHX4	protein_codir	0.66509016	0.61962577	0.74302365
ENSG0000022	RP5-983L19.2	lncRNA	0.66504313	0.60622384	0.73255031
ENSG0000025	RP11-115H15	lncRNA	0.66477083	0.66539355	NA
ENSG0000017	MEX3C	protein_codir	0.66451939	9.50E-05	0.00083365
ENSG0000025	RP11-10J21.4	lncRNA	0.66449598	0.53959845	0.67876801
ENSG0000011	TCIRG1	protein_codir	0.66442543	0.02276942	0.06557783
ENSG0000017	ARL6IP1	protein_codir	0.6644134	2.46E-06	3.91E-05
ENSG0000011	CREBL2	protein_codir	0.66431994	0.00064766	0.0040444
ENSG0000017	ZNF581	protein_codir	0.66430015	0.00076948	0.00463837
ENSG0000027	KLC4-AS1	lncRNA	0.66418439	0.65841281	NA
ENSG0000026	RP11-505K9.1	lncRNA	0.66418008	0.05057671	0.12157779
ENSG0000020	FOXD4L5	protein_codir	0.66369931	0.5701245	0.70367498
ENSG0000027	RP4-740C4.7	lncRNA	0.66366434	0.23271202	0.37576766
ENSG0000028	AC068831.18	protein_codir	0.66328127	0.11990415	0.23087266
ENSG0000022	RP1-101K10.6	lncRNA	0.66311044	0.01971165	0.05863605
ENSG0000016	PIGA	protein_codir	0.66305511	0.00759284	0.02805166
ENSG0000012	MPDU1	protein_codir	0.66294484	0.0025107	0.01173269
ENSG0000012	VIL1	protein_codir	0.66284306	0.58565427	0.71624916
ENSG0000025	RP11-152H18	lncRNA	0.66275228	0.53659092	0.67674161

ENSG0000007	LXN	protein_codir	0.66250881	0.02736787	0.07572636
ENSG0000012	SIX1	protein_codir	0.66234109	0.07092467	0.15656297
ENSG0000027	RP4-694B14.8	lncRNA	0.66210749	0.21744355	0.35767917
ENSG0000010	GDPD3	protein_codir	0.66193991	0.15063949	0.27311688
ENSG0000013	PARP9	protein_codir	0.66185169	0.0052225	0.02099637
ENSG0000000	ALDH3B1	protein_codir	0.661806	0.00315394	0.01405935
ENSG0000019	ZNF124	protein_codir	0.6605032	0.00794644	0.02904721
ENSG0000028	RP11-175O15	lncRNA	0.66041112	0.59951821	0.72726652
ENSG0000016	FBLN2	protein_codir	0.66007779	0.13295415	0.2494044
ENSG0000013	SLC38A6	protein_codir	0.65999414	0.00565083	0.02228066
ENSG0000005	MPHOSPH9	protein_codir	0.65986175	0.01112768	0.03785987
ENSG0000028	RP11-770J1.8	protein_codir	0.65981044	0.02522837	0.07113186
ENSG0000010	SCG3	protein_codir	0.65979765	0.34341537	0.49660379
ENSG0000016	CSNK1G1	protein_codir	0.659694	5.89E-05	0.00056464
ENSG0000014	TTYH2	protein_codir	0.65957913	4.58E-05	0.00045364
ENSG0000027	CFAP206	protein_codir	0.65957176	0.16267396	0.28911258
ENSG0000025	NACA2	protein_codir	0.65956596	0.22260027	0.36379998
ENSG0000021	MXD3	protein_codir	0.6595454	0.07689596	0.16586227
ENSG0000026	CTB-30L5.1	lncRNA	0.65948492	0.40571636	0.55867413
ENSG0000016	CGAS	protein_codir	0.65884141	0.00064561	0.00403344
ENSG0000013	BPIFA2	protein_codir	0.65871689	0.78407653	NA
ENSG0000013	SLC52A1	protein_codir	0.65856219	0.3424077	0.49563968
ENSG0000014	LRRC4C	protein_codir	0.65839068	0.06777471	0.1515771
ENSG0000027	STAG3L5P-PV	lncRNA	0.65823858	0.05267491	0.12512323
ENSG0000014	LHFPL2	protein_codir	0.65791249	0.00767708	0.02828342
ENSG0000022	AC092535.3	lncRNA	0.65750404	0.4105123	0.56345225
ENSG0000015	GNA14	protein_codir	0.65730836	0.06181202	0.14122326
ENSG0000026	RP5-963E22.5	lncRNA	0.65724095	0.73595292	NA
ENSG0000025	SCARNA9	lncRNA	0.65713636	0.06903443	0.15336049
ENSG0000018	DEFB124	protein_codir	0.6570444	0.43573028	0.58698095
ENSG0000027	CTD-3222D19	lncRNA	0.6569879	0.12814974	0.24254751
ENSG0000026	GLIS2-AS1	lncRNA	0.65690132	0.62503302	0.74721812
ENSG0000026	RP11-674P19.	lncRNA	0.65663043	0.41201271	0.56500626
ENSG0000010	KLHDC4	protein_codir	0.65662933	0.01250972	0.04137449
ENSG0000023	LINC00856	lncRNA	0.65645402	0.20535246	0.34328985
ENSG0000016	AVPR1A	protein_codir	0.65618117	0.26811262	0.4159797
ENSG0000017	SMCR8	protein_codir	0.65608159	0.00271402	0.01247066
ENSG0000026	RP11-120K18.	lncRNA	0.65588422	0.67371729	0.78479356
ENSG0000011	SLC38A1	protein_codir	0.65587323	0.01102428	0.03762394
ENSG0000018	FNBP1	protein_codir	0.6558641	0.00238442	0.01126737
ENSG0000012	PI3	protein_codir	0.65573012	0.29332618	0.44346539
ENSG0000015	USF1	protein_codir	0.65570082	0.00458801	0.0189787
ENSG0000011	ATP6V1A	protein_codir	0.65568008	0.00616672	0.02385169
ENSG0000028	CTD-2093G19	lncRNA	0.65532973	0.32579222	0.47804975
ENSG0000010	PLGRKT	protein_codir	0.65522753	0.00029797	0.00213943
ENSG0000018	FAM87A	lncRNA	0.65496122	0.42020258	0.57256522
ENSG0000013	GRHL1	protein_codir	0.65480502	0.23636447	0.38028698

ENSG0000018	SHTN1	protein_codir	0.65471397	0.01576119	0.0495193
ENSG0000013	ENTPD1	protein_codir	0.6546089	0.01811184	0.05491812
ENSG0000012	KCNJ5	protein_codir	0.65450229	0.18922412	0.32333397
ENSG0000016	GPR85	protein_codir	0.65432949	0.02921223	0.079871
ENSG0000027	RP1-142L7.9	lncRNA	0.65426072	0.34334721	0.49653123
ENSG0000010	PARP4	protein_codir	0.65409346	0.00487358	0.01988012
ENSG0000026	RP1-266L20.9	lncRNA	0.65341431	0.23160183	0.37432515
ENSG0000011	GALNT12	protein_codir	0.65306312	0.00243983	0.01147226
ENSG0000012	DYRK2	protein_codir	0.65305467	0.00117211	0.00648639
ENSG0000026	RP11-194N12	lncRNA	0.65286121	0.42813057	0.57985419
ENSG0000016	LMX1A	protein_codir	0.65256983	0.32851619	0.48082333
ENSG0000026	RP11-669C19.	lncRNA	0.65248523	0.61608354	0.74002902
ENSG0000023	LINC00574	lncRNA	0.65238081	0.09021014	0.18649731
ENSG0000025	CTC-329D1.3	lncRNA	0.65205863	0.7018991	NA
ENSG0000017	CEP135	protein_codir	0.6520416	0.00290539	0.0131456
ENSG0000017	DELEC1	lncRNA	0.65137888	0.36805796	0.5215765
ENSG0000028	RP11-577H5.6	lncRNA	0.6513285	0.3321538	0.48496527
ENSG0000006	CNN2	protein_codir	0.65127082	0.00185598	0.00926313
ENSG0000013	CCT6B	protein_codir	0.65103708	0.0204494	0.06029811
ENSG0000016	TOPBP1	protein_codir	0.65086045	3.35E-05	0.00035119
ENSG0000026	A1BG-AS1	lncRNA	0.65085485	0.03954311	0.10061545
ENSG0000011	ABCG2	protein_codir	0.65078922	0.0310823	0.08368194
ENSG0000020	CDKL4	protein_codir	0.65074957	0.53172355	0.6727533
ENSG0000017	WDR97	protein_codir	0.65064808	0.08652151	0.1810397
ENSG0000023	AC138035.2	lncRNA	0.65036001	0.07805639	0.16759309
ENSG0000017	NHLH1	protein_codir	0.64953573	0.15230503	0.27541344
ENSG0000025	CTD-2213F21.	lncRNA	0.64934817	0.60322744	0.73001562
ENSG0000012	DEPDC7	protein_codir	0.64925246	0.06617328	0.14899372
ENSG0000016	YDJC	protein_codir	0.6492524	0.00084969	0.00502536
ENSG0000015	EPHB1	protein_codir	0.64922678	0.12735892	0.24149755
ENSG0000025	MAP1LC3B2	protein_codir	0.64883572	0.02469062	0.06991541
ENSG0000011	LAMA4	protein_codir	0.64840647	0.03387906	0.08934781
ENSG0000026	LINC02544	lncRNA	0.6482028	0.25457825	0.40106385
ENSG0000026	CTD-2012K14	lncRNA	0.64788107	0.69999335	NA
ENSG0000020	CAPN8	protein_codir	0.64787275	0.40783044	0.56085823
ENSG0000009	BTAF1	protein_codir	0.64749344	0.00014973	0.00122509
ENSG0000011	IFIT2	protein_codir	0.64746221	0.09512529	0.19429571
ENSG0000010	NOP53	protein_codir	0.64733516	0.0006081	0.0038355
ENSG0000027	RP11-180M15	lncRNA	0.64727408	0.29088553	0.44063057
ENSG0000014	DGKZ	protein_codir	0.64713116	0.00063775	0.00399156
ENSG0000013	PRR7	protein_codir	0.6469802	0.00580166	0.02273258
ENSG0000015	C4orf19	protein_codir	0.64697683	0.50418195	0.64890519
ENSG0000013	DNA2	protein_codir	0.64689644	0.04870891	0.1181159
ENSG0000021	SULT1A4	protein_codir	0.64688444	0.29890046	0.44992265
ENSG0000015	ANKFN1	protein_codir	0.64687596	0.39087518	0.54382286
ENSG0000023	SDCBP2-AS1	lncRNA	0.64678318	0.01725433	0.05296929
ENSG0000006	BCAS1	protein_codir	0.64669733	0.28293885	0.43243163

ENSG0000021	GOLGA8B	protein_codir	0.64622375	0.05489298	0.12903354
ENSG0000025	NPIPB8	protein_codir	0.64616424	0.41656185	0.56932683
ENSG0000007	CLTCL1	protein_codir	0.64607413	0.01201821	0.04012366
ENSG0000028	RP11-574F11.	lncRNA	0.64606208	0.03411466	0.08984905
ENSG0000011	FBXO5	protein_codir	0.64602052	0.00026127	0.00192385
ENSG0000027	RP11-449P15.	lncRNA	0.64557789	0.01459233	0.04669682
ENSG0000015	DONSON	protein_codir	0.64542705	0.00623735	0.0240642
ENSG0000028	CTD-3138B18	protein_codir	0.64529996	0.05056365	0.12155697
ENSG0000026	CTB-83J4.1	lncRNA	0.64494251	0.46713358	0.61668242
ENSG0000027	CH17-373J23.	lncRNA	0.64461787	0.44832174	0.59917965
ENSG0000019	GSTK1	protein_codir	0.64457593	1.10E-05	0.00013897
ENSG0000026	RP5-867C24.4	lncRNA	0.64445835	0.32019322	0.47232776
ENSG0000011	WASF1	protein_codir	0.64431769	0.01354497	0.04403908
ENSG0000011	TP53I3	protein_codir	0.64398732	0.01531315	0.04845333
ENSG0000013	ETNK1	protein_codir	0.64360171	6.90E-05	0.00064177
ENSG0000027	GOLGA6L22	protein_codir	0.64349165	0.73705425	NA
ENSG0000019	CTC-301O7.4	lncRNA	0.64343209	0.11891228	0.22946671
ENSG0000025	RP11-864I4.1	protein_codir	0.6431507	0.02848193	0.0782757
ENSG0000015	PPEF2	protein_codir	0.64312268	0.30275682	0.45416336
ENSG0000013	CCNH	protein_codir	0.64307909	0.01232576	0.0408998
ENSG0000020	C11orf91	protein_codir	0.64298893	0.36377608	0.51777868
ENSG0000024	AC093627.9	lncRNA	0.64287422	0.00827386	0.02998611
ENSG0000016	PBX3	protein_codir	0.6428545	0.01014034	0.03519871
ENSG0000013	KLRC1	protein_codir	0.64258016	0.13237836	0.24864266
ENSG0000017	RHOG	protein_codir	0.64257371	0.00077662	0.00467023
ENSG0000006	POLD1	protein_codir	0.64216443	0.0083818	0.03027086
ENSG0000016	TNFSF13	protein_codir	0.64211076	0.01179025	0.03958268
ENSG0000010	LGMN	protein_codir	0.6419848	0.01928879	0.0577326
ENSG0000019	RPL12	protein_codir	0.64194956	0.00089817	0.00525802
ENSG0000013	SLC7A10	protein_codir	0.64189597	0.46472843	0.61468246
ENSG0000011	GNAT1	protein_codir	0.64187055	0.49286707	0.63928752
ENSG0000013	LRRIQ1	protein_codir	0.64186432	0.27155866	0.41958693
ENSG0000026	DNAAF3-AS1	lncRNA	0.64164806	0.56836108	0.70212406
ENSG0000025	RP11-15E18.1	lncRNA	0.64141725	0.41629896	0.56907583
ENSG0000017	ZFAS1	lncRNA	0.64128931	0.00768008	0.0282907
ENSG0000019	SSBP3-AS1	lncRNA	0.64076505	0.3286233	0.48092913
ENSG0000027	AC007950.2	lncRNA	0.64061737	0.53505273	0.67544935
ENSG0000014	ACKR3	protein_codir	0.64040669	0.10336757	0.20683999
ENSG0000017	PPM1E	protein_codir	0.64030057	0.33550455	0.48836025
ENSG0000027	RP1-100J12.1	lncRNA	0.64018742	0.39359243	0.54666715
ENSG0000016	YPEL4	protein_codir	0.6400549	0.21915023	0.35975806
ENSG0000022	CADM3-AS1	lncRNA	0.63980603	0.19106937	0.32564933
ENSG0000017	MAF	protein_codir	0.63972115	0.00239396	0.01130256
ENSG0000024	RP11-710F7.3	lncRNA	0.63948792	0.37849363	0.53204827
ENSG0000008	B4GALT1	protein_codir	0.63944444	0.00600745	0.0233468
ENSG0000016	HSPBAP1	protein_codir	0.63897256	0.02112413	0.06183325
ENSG0000028	N4BP2L2-IT2	lncRNA	0.63888783	0.21737749	0.3576249

ENSG0000016	MLKL	protein_codir	0.63886312	0.03575508	0.09303394
ENSG0000015	ALLC	protein_codir	0.638846	0.55951659	0.69496102
ENSG0000027	RP11-278C7.3	lncRNA	0.6387356	0.18329311	0.31587668
ENSG0000023	WARS2-AS1	lncRNA	0.63864624	0.00064869	0.00404609
ENSG0000014	NTRK2	protein_codir	0.63860612	0.06267578	0.14285401
ENSG0000016	TPP1	protein_codir	0.63809549	0.00615998	0.02382894
ENSG0000026	CTD-2325M2.	lncRNA	0.63794962	0.43891251	0.58997292
ENSG0000016	CTHRC1	protein_codir	0.63774712	0.12026973	0.23137404
ENSG0000007	ZCWPW1	protein_codir	0.63753349	0.01210076	0.04034559
ENSG0000008	SNX5	protein_codir	0.63749615	5.97E-05	0.00057006
ENSG0000017	FUCA1	protein_codir	0.63738959	0.01308292	0.04284955
ENSG0000018	PTCH1	protein_codir	0.6372611	0.00104393	0.00592306
ENSG0000014	TMEM108	protein_codir	0.63716454	0.14892173	0.27107013
ENSG0000014	ARHGAP26	protein_codir	0.63711625	0.0008094	0.00483572
ENSG0000011	GABRR2	protein_codir	0.63701482	0.18837857	0.32226796
ENSG0000026	RP11-6N17.2	lncRNA	0.63697287	0.56017132	0.69539898
ENSG0000015	CHST9	protein_codir	0.63667327	0.43246181	0.58374524
ENSG0000008	STRADB	protein_codir	0.63661961	0.00041557	0.00281465
ENSG0000010	SUV39H1	protein_codir	0.63644807	0.00089192	0.00522697
ENSG0000010	CNTNAP3	protein_codir	0.6363704	0.24280475	0.38777644
ENSG0000018	H2BC4	protein_codir	0.63579802	0.12589797	0.23955543
ENSG0000017	DENND5B	protein_codir	0.63553685	0.00167119	0.00857482
ENSG0000022	SPATA13	lncRNA	0.63532181	0.3996792	0.55298726
ENSG0000023	LINC01431	lncRNA	0.63527568	0.11678228	0.22641629
ENSG0000008	FOLH1	protein_codir	0.6350672	0.11805509	0.22820908
ENSG0000013	LDLR	protein_codir	0.63432052	0.17118797	0.3003374
ENSG0000026	RP11-146F11.	lncRNA	0.63431814	0.07411114	0.16155126
ENSG0000012	TNFAIP6	protein_codir	0.63424811	0.11692265	0.22656276
ENSG0000007	BAZ2A	protein_codir	0.63386756	3.76E-07	7.88E-06
ENSG0000016	BEAN1	protein_codir	0.63357018	0.05821267	0.13479493
ENSG0000013	DCLK1	protein_codir	0.63352267	0.08136122	0.17290267
ENSG0000012	CDKN1C	protein_codir	0.63321672	0.06392532	0.14514707
ENSG0000022	RP11-295G24	lncRNA	0.63307679	0.46572885	0.61535739
ENSG0000027	AC006946.16	lncRNA	0.632912	0.33136188	0.48409032
ENSG0000025	LINC01220	lncRNA	0.63290024	0.18253783	0.31488945
ENSG0000022	SEPTIN7-DT	lncRNA	0.63283671	0.00313232	0.01398331
ENSG0000018	ZNF792	protein_codir	0.63256958	0.05881457	0.13583564
ENSG0000015	PKD1L1	protein_codir	0.63233258	0.12927865	0.24413211
ENSG0000025	RP11-613M10	protein_codir	0.63135184	0.28933916	0.43889327
ENSG0000014	MAP3K21	protein_codir	0.63135001	0.04220413	0.10584565
ENSG0000015	C16orf86	protein_codir	0.63123588	0.00275258	0.0126075
ENSG0000026	APOC4	protein_codir	0.63107727	0.67151772	NA
ENSG0000024	RP11-517B11.	lncRNA	0.63085855	0.37189635	0.52530312
ENSG0000017	ZFPM1	protein_codir	0.63078653	0.00167893	0.00861133
ENSG0000025	LINC02615	lncRNA	0.63068931	0.26874013	0.41641529
ENSG0000026	RP4-591C20.9	lncRNA	0.63063958	0.00134768	0.00725875
ENSG0000010	SUSD1	protein_codir	0.63028501	0.00795453	0.0290691

ENSG0000013	DOCK10	protein_codir	0.63005536	0.06097177	0.13965081
ENSG0000011	SLC4A9	protein_codir	0.62989776	0.52303484	0.66526467
ENSG0000025	CA3-AS1	lncRNA	0.62986231	0.1377482	0.25565148
ENSG0000019	FKBP1C	protein_codir	0.6298294	0.01805498	0.05482995
ENSG0000022	NUP50-DT	lncRNA	0.62978813	0.01445755	0.04631918
ENSG0000028	CH507-254M2	lncRNA	0.62966131	0.31093078	0.46268564
ENSG0000011	PIK3R3	protein_codir	0.62949466	0.02123836	0.06206788
ENSG0000027	RP11-379F4.8	lncRNA	0.62884918	0.3444736	0.49756721
ENSG0000012	RIBC2	protein_codir	0.62865046	0.32552903	0.47779317
ENSG0000027	RP5-899E9.1	lncRNA	0.62831461	0.23860295	0.38286258
ENSG0000028	RP3-487J7.4	lncRNA	0.62829253	0.47429566	0.62271455
ENSG0000011	DNPH1	protein_codir	0.62816019	0.01030125	0.03563634
ENSG0000017	RP11-89N17.1	lncRNA	0.62761972	0.46240885	0.61252374
ENSG0000027	LINC02035	lncRNA	0.62732017	0.00059044	0.00374463
ENSG0000012	AKAP9	protein_codir	0.62731216	6.31E-06	8.68E-05
ENSG0000022	RP11-278A23	lncRNA	0.62707307	0.18119455	0.31306102
ENSG0000013	HERC6	protein_codir	0.62679057	0.04728063	0.1153225
ENSG0000015	KLF10	protein_codir	0.62678935	0.08402728	0.1769564
ENSG0000015	PRDM8	protein_codir	0.62672337	0.11695399	0.22660605
ENSG0000019	TFDP1	protein_codir	0.62670444	0.00192002	0.0095072
ENSG0000027	RP11-368I7.6	lncRNA	0.62611604	0.17374548	0.30368625
ENSG0000014	OGT	protein_codir	0.62605673	0.04555563	0.11225158
ENSG0000028	SNHG4	lncRNA	0.62567948	0.05508716	0.12934508
ENSG0000020	ZDHHC18	protein_codir	0.62554118	0.00017411	0.00138259
ENSG0000024	COX19	protein_codir	0.6249844	0.00115457	0.00641247
ENSG0000018	OLFML1	protein_codir	0.62492446	0.05824269	0.13484811
ENSG0000028	CTD-2134A5.3	lncRNA	0.62460905	0.29450592	0.4447992
ENSG0000008	FAT2	protein_codir	0.62448265	0.35205247	0.50559948
ENSG0000018	GPR157	protein_codir	0.62422028	0.00012392	0.00104446
ENSG0000014	RPL11	protein_codir	0.62393716	0.00017604	0.00139358
ENSG0000028	CTC-563A5.5	lncRNA	0.62390791	0.78521809	NA
ENSG0000018	HDDC3	protein_codir	0.62364386	0.00751666	0.0278223
ENSG0000013	CLDN10	protein_codir	0.6234629	0.53073404	0.67199498
ENSG0000016	TRANK1	protein_codir	0.62326413	0.03828641	0.09803194
ENSG0000011	ACAP2	protein_codir	0.62318256	0.00020145	0.0015531
ENSG0000027	PPP4R3B-DT	lncRNA	0.62304154	0.13330294	0.24983559
ENSG0000024	CTC-498J12.3	lncRNA	0.62303541	0.61309837	0.7376508
ENSG0000011	CDH6	protein_codir	0.62287997	0.03128738	0.08405382
ENSG0000010	RGS9	protein_codir	0.62284091	0.13830302	0.2564916
ENSG0000022	RP11-131L23.	lncRNA	0.62272292	0.37299028	0.52657899
ENSG0000025	RP11-21A7A.2	lncRNA	0.62267174	0.67730443	NA
ENSG0000018	CYP4A11	protein_codir	0.62266653	0.49382404	0.64010262
ENSG0000017	GPBAR1	protein_codir	0.62251116	0.10755514	0.21299726
ENSG0000023	LINC00342	lncRNA	0.62250553	0.13389335	0.25060668
ENSG0000012	MGME1	protein_codir	0.62233533	0.01027353	0.03556719
ENSG0000014	SLC25A25	protein_codir	0.62224753	0.13370581	0.2503698
ENSG0000026	CTD-3220F14.	lncRNA	0.62223264	0.17563445	0.30582672

ENSG0000002	CYP3A43	protein_codir	0.62195425	0.59060557	0.72013622
ENSG0000013	CHRM3	protein_codir	0.62170954	0.12192125	0.23388421
ENSG0000024	SEC24B-AS1	lncRNA	0.62161677	0.05110604	0.1224665
ENSG0000012	SPCS3	protein_codir	0.6210958	0.00201871	0.00989648
ENSG0000017	SLC22A1	protein_codir	0.62087529	0.17666606	0.30709631
ENSG0000028	CTD-2530N21	lncRNA	0.62039073	0.50568018	0.65025776
ENSG0000026	RP11-529K1.3	protein_codir	0.62024485	0.44576981	0.59674618
ENSG0000024	RPL36A	protein_codir	0.62012327	0.00373159	0.01607149
ENSG0000022	AC097381.1	lncRNA	0.61999804	0.39127008	0.54415305
ENSG0000011	CISH	protein_codir	0.61991738	0.04129966	0.10404967
ENSG0000028	RP11-95J9.3	lncRNA	0.61982891	0.0275504	0.07612466
ENSG0000012	VPS13C	protein_codir	0.61952638	0.00074283	0.00451217
ENSG0000014	SLC26A1	protein_codir	0.61947756	0.27600945	0.42416439
ENSG0000018	CLEC20A	protein_codir	0.61944671	0.37810574	0.53173527
ENSG0000028	RP11-24J23.3	lncRNA	0.61944539	0.75718325	NA
ENSG0000026	RP11-77K12.3	lncRNA	0.61934407	0.55581969	0.69170676
ENSG0000018	CACNB4	protein_codir	0.6192755	0.03191859	0.08522405
ENSG0000010	YPEL1	protein_codir	0.61889588	0.02281784	0.06568034
ENSG0000017	ALOXE3	protein_codir	0.6188488	0.55404248	0.69030374
ENSG0000005	PRSS8	protein_codir	0.61870638	0.65783302	0.77263769
ENSG0000012	IFI6	protein_codir	0.61862919	0.06546555	0.14779781
ENSG0000016	DUSP18	protein_codir	0.61862038	0.01130749	0.03832481
ENSG0000025	ARAP1-AS1	lncRNA	0.61823888	0.48974834	0.63656986
ENSG0000018	FAM89A	protein_codir	0.61816747	0.02329797	0.06674514
ENSG0000010	ASB9	protein_codir	0.61816165	0.05168208	0.12336083
ENSG0000028	RP11-361A21	lncRNA	0.61772778	0.6635638	0.77730153
ENSG0000018	LRRC37B	protein_codir	0.61740044	0.00074014	0.0045008
ENSG0000018	UBA7	protein_codir	0.61737833	0.01290518	0.04238805
ENSG0000018	ISG15	protein_codir	0.61710807	0.07306929	0.15996938
ENSG0000000	NIPAL3	protein_codir	0.61701925	0.04284694	0.1070592
ENSG0000025	GPR142	protein_codir	0.61682114	0.49263773	0.63919947
ENSG0000007	FAM76B	protein_codir	0.6167619	0.00025308	0.00187197
ENSG0000014	PLPP5	protein_codir	0.61649084	0.01227499	0.04078086
ENSG0000014	GEMIN7	protein_codir	0.61617544	0.01420727	0.04572417
ENSG0000026	RP13-753N3.1	lncRNA	0.61606534	0.52609897	0.66792032
ENSG0000016	RPL29	protein_codir	0.61572085	0.0004311	0.00289507
ENSG0000017	CATSPER1	protein_codir	0.61563303	0.11182018	0.219316
ENSG0000018	NRN1L	protein_codir	0.61544262	0.30698737	0.45872057
ENSG0000019	WDHD1	protein_codir	0.61544013	0.01122745	0.03812889
ENSG0000018	PMEL	protein_codir	0.6154324	0.15374164	0.27735927
ENSG0000011	ATAD2B	protein_codir	0.61542584	1.77E-05	0.00020703
ENSG0000018	FES	protein_codir	0.61541899	0.00770066	0.02834761
ENSG0000018	SAMD11	protein_codir	0.61538689	0.07306549	0.15996938
ENSG0000017	ANGPTL7	protein_codir	0.61526589	0.27104414	0.41902406
ENSG0000022	RP11-110G21	lncRNA	0.61501028	0.01871719	0.05636721
ENSG0000017	PPM1D	protein_codir	0.61483049	0.00031499	0.00224168
ENSG0000022	GUSBP11	lncRNA	0.61474389	0.0575498	0.13360726

ENSG0000018	IFNE	protein_codir	0.61456954	0.69747225	NA
ENSG0000026	SRGAP2	protein_codir	0.61420155	0.00015362	0.00124833
ENSG0000010	NAGPA	protein_codir	0.61404009	0.00448998	0.01864579
ENSG0000026	RP11-464D20	lncRNA	0.61389941	0.45239809	0.60296459
ENSG0000022	CEBPD	protein_codir	0.61387094	0.11269306	0.22046906
ENSG0000027	LINC01451	lncRNA	0.61378249	0.53113062	0.67234189
ENSG0000011	CBLB	protein_codir	0.61365145	0.00797601	0.02913986
ENSG0000016	CCDC68	protein_codir	0.61346157	0.08225054	0.17414795
ENSG0000016	TMPRSS5	protein_codir	0.61344999	0.17445714	0.30452545
ENSG0000017	TNK1	protein_codir	0.61331162	0.04992791	0.12040591
ENSG0000020	ECT2L	protein_codir	0.61324848	0.52620475	0.66800457
ENSG0000018	TLR5	protein_codir	0.61291088	0.08762604	0.18259006
ENSG0000017	KLF11	protein_codir	0.61254553	0.05181613	0.12357428
ENSG0000028	RP11-13J12.3	lncRNA	0.61236918	0.42337054	0.57535398
ENSG0000016	ZNF367	protein_codir	0.61202666	0.02400205	0.06833694
ENSG0000012	RAP2C	protein_codir	0.61179989	1.22E-06	2.18E-05
ENSG0000025	RHPN1-AS1	lncRNA	0.61174311	0.29962462	0.45071794
ENSG0000016	SLC39A2	protein_codir	0.61173299	0.4792327	0.62702071
ENSG0000021	TTLL3	protein_codir	0.61144953	0.09575015	0.19521807
ENSG0000025	OSGEPL1-AS1	lncRNA	0.61129721	0.47241812	0.6210976
ENSG0000027	RP11-52A20.2	lncRNA	0.61126668	0.2655485	0.41310932
ENSG0000017	MLF1	protein_codir	0.611035	0.12663089	0.24052989
ENSG0000016	U2AF1L4	protein_codir	0.6109335	0.04380992	0.108845
ENSG0000028	CTD-2587H24	lncRNA	0.60969402	0.2894458	0.43901663
ENSG0000016	PRKCD	protein_codir	0.60963282	0.00798005	0.02914693
ENSG0000025	RP11-473O4.3	lncRNA	0.60929396	0.66282638	NA
ENSG0000018	CCK	protein_codir	0.60907252	0.61778142	0.74151276
ENSG0000014	ADD3	protein_codir	0.60848086	0.00210851	0.01023676
ENSG0000024	RP11-68L18.1	lncRNA	0.60842761	0.44330068	0.59428068
ENSG0000028	RP11-173A6.4	protein_codir	0.60792062	0.69561929	NA
ENSG0000014	KDM6A	protein_codir	0.60774269	2.80E-06	4.35E-05
ENSG0000018	C1S	protein_codir	0.60719923	0.01178969	0.03958268
ENSG0000022	OGFR-AS1	lncRNA	0.60681359	0.66077804	0.7750352
ENSG0000023	AC009404.2	lncRNA	0.60652673	0.11102369	0.2182544
ENSG0000019	MDM4	protein_codir	0.60638759	0.01683376	0.05203094
ENSG0000025	RP13-884E18.	lncRNA	0.6063499	0.57268475	0.70573117
ENSG0000011	FBXO6	protein_codir	0.60632566	0.00302934	0.01361158
ENSG0000025	CTD-3203P2.1	lncRNA	0.60614017	0.26780263	0.41563883
ENSG0000018	VHLL	protein_codir	0.60605982	0.69267677	NA
ENSG0000008	FXYD3	protein_codir	0.60596321	0.53689102	0.67699644
ENSG0000024	CTD-2083E4.6	lncRNA	0.60589093	0.51367151	0.65702682
ENSG0000010	ATP13A1	protein_codir	0.60565659	0.00400297	0.01702262
ENSG0000027	RP11-326I11.5	lncRNA	0.60538135	0.27733695	0.42577103
ENSG0000019	SRGAP1	protein_codir	0.60537831	0.00165261	0.00850794
ENSG0000022	ANKRD10-IT1	lncRNA	0.60496853	0.15738292	0.28224897
ENSG0000026	RP4-806M20.	lncRNA	0.60491217	0.63415488	NA
ENSG0000017	TSSK6	protein_codir	0.60486585	0.08127237	0.17275226

ENSG0000026	RP11-159D12	protein_codir	0.60458549	0.27508466	0.42320716
ENSG0000022	MIR503HG	lncRNA	0.60458093	0.2309476	0.37359568
ENSG0000018	RPH3AL	protein_codir	0.60451473	0.01785712	0.05433663
ENSG0000018	TSEN54	protein_codir	0.60440521	0.01150804	0.03887622
ENSG0000017	MST1	protein_codir	0.60438486	0.1036384	0.2072768
ENSG0000022	RP1-309F20.3	lncRNA	0.60423361	0.23734345	0.38143991
ENSG0000020	RP11-798L4.1	lncRNA	0.60371608	0.48124256	0.62881633
ENSG0000027	ZSWIM8-AS1	lncRNA	0.60370783	0.51262121	0.65621977
ENSG0000028	RP11-265N6.5	protein_codir	0.60356505	0.17778374	0.30850067
ENSG0000013	SWAP70	protein_codir	0.60349734	2.04E-05	0.00023279
ENSG0000017	SLC30A1	protein_codir	0.60321329	0.07225796	0.15869631
ENSG0000018	NAT8L	protein_codir	0.60310798	0.42259929	0.57462492
ENSG0000019	CCDC69	protein_codir	0.60306359	0.00033834	0.00238331
ENSG0000022	RP4-564F22.5	lncRNA	0.60297109	0.39118087	0.54408378
ENSG0000016	MIDN	protein_codir	0.60279724	0.05292965	0.12552952
ENSG0000025	RP11-73G16.1	lncRNA	0.60238968	0.75658538	NA
ENSG0000027	RP5-1056H1.2	lncRNA	0.60221279	0.55771797	0.69344424
ENSG0000018	RP11-863K10.	lncRNA	0.60211436	0.07882919	0.16884545
ENSG0000007	ATP12A	protein_codir	0.60209299	0.42481421	0.57674835
ENSG0000025	RP11-66N24.3	lncRNA	0.602053	0.13698389	0.25466077
ENSG0000010	PLTP	protein_codir	0.60181465	0.11527466	0.22419715
ENSG0000009	GGT5	protein_codir	0.60163791	0.00343971	0.01506632
ENSG0000016	DUS2	protein_codir	0.60145331	0.06185747	0.14130372
ENSG0000017	SLFN11	protein_codir	0.60140989	1.08E-05	0.00013674
ENSG0000019	SYCP2	protein_codir	0.60116952	0.03539789	0.09236559
ENSG0000000	SLC7A2	protein_codir	0.60114876	0.10727926	0.21256167
ENSG0000012	MAX	protein_codir	0.60099749	7.49E-08	1.97E-06
ENSG0000015	RPL30	protein_codir	0.6009771	0.00012027	0.0010171
ENSG0000014	RUBCN	protein_codir	0.60095371	0.00092952	0.00540148
ENSG0000028	RP11-21N3.2	lncRNA	0.600754	0.38023604	0.53360786
ENSG0000026	LA16c-325D7.	lncRNA	0.60049016	0.5065804	0.65105163
ENSG0000024	RP5-894A10.2	lncRNA	0.59999333	0.11709651	0.22680263
ENSG0000002	DERA	protein_codir	0.59992518	1.72E-07	3.98E-06
ENSG0000023	RP11-515I12.1	lncRNA	0.59992298	0.3673585	0.52108007
ENSG0000013	CEP350	protein_codir	0.59990593	0.00257885	0.01196503
ENSG0000014	AK7	protein_codir	0.59988055	0.18503451	0.31818286
ENSG0000027	CCDC28A-AS1	lncRNA	0.59958104	0.22998826	0.37238557
ENSG0000017	BCL2	protein_codir	0.59945241	0.01460175	0.04671931
ENSG0000008	ZBTB25	protein_codir	0.59923143	0.01197805	0.0400186
ENSG0000017	SLITRK4	protein_codir	0.59912191	0.09141705	0.18844235
ENSG0000012	ZBTB1	protein_codir	0.59899673	0.00041198	0.00279785
ENSG0000022	RP11-2L8.1	lncRNA	0.59877638	0.46500296	0.61489835
ENSG0000015	THEM4	protein_codir	0.59875862	0.00163555	0.00844528
ENSG0000010	HIF1A	protein_codir	0.59862594	0.07174257	0.15787129
ENSG0000016	MCM7	protein_codir	0.59853308	0.00140132	0.00747337
ENSG0000010	ZC3HAV1	protein_codir	0.59845968	0.00171887	0.0087566
ENSG0000016	MFSD12	protein_codir	0.59797927	0.0093967	0.03316766

ENSG0000028	RP11-15L13.5	protein_codir	0.59780535	0.72841686	0.82540175
ENSG0000012	TAS2R10	protein_codir	0.59776543	0.43886414	0.58996533
ENSG0000025	LINC00534	lncRNA	0.5977515	0.59897248	0.72682122
ENSG0000016	SCNN1G	protein_codir	0.59765219	0.6634564	0.77729229
ENSG0000007	EDN1	protein_codir	0.59759396	0.05423229	0.12795197
ENSG0000009	NRP1	protein_codir	0.5973437	0.00701717	0.02638884
ENSG0000004	MYO16	protein_codir	0.59732335	0.10384641	0.20756277
ENSG0000023	LINC01563	lncRNA	0.59731947	0.55323889	0.6896136
ENSG0000014	RPL37	protein_codir	0.5972887	0.00094107	0.00545998
ENSG0000024	AC006547.8	lncRNA	0.59727054	0.58587262	0.71642102
ENSG0000024	LINC01586	lncRNA	0.59695503	0.79911368	NA
ENSG0000017	PHYKPL	protein_codir	0.59688313	0.0245595	0.0696298
ENSG0000028	RP11-345J4.1	lncRNA	0.5968727	0.00937625	0.03311664
ENSG0000026	RP11-1024P1	lncRNA	0.59660854	0.0030542	0.01369877
ENSG0000010	RIC1	protein_codir	0.59644918	5.91E-05	0.00056544
ENSG0000017	CLDN22	protein_codir	0.59634206	0.64402713	0.76176734
ENSG0000015	KCNJ16	protein_codir	0.59626876	0.45757905	0.60813693
ENSG0000016	RPL26	protein_codir	0.59609419	0.00022224	0.0016836
ENSG0000016	COQ8A	protein_codir	0.59608809	0.00928848	0.03288231
ENSG0000017	GRB2	protein_codir	0.59596974	0.00023976	0.0017917
ENSG0000023	AC009473.1	lncRNA	0.59536106	0.4486728	0.59950378
ENSG0000012	LRRC6	protein_codir	0.59535525	0.08501469	0.17859033
ENSG0000014	CELF6	protein_codir	0.59521	0.15648258	0.28098565
ENSG0000017	ZIC4	protein_codir	0.59497928	0.39247417	0.54547057
ENSG0000025	TGIF2-RAB5IF	protein_codir	0.59493967	0.56189051	0.69675028
ENSG0000025	VASH1-AS1	lncRNA	0.59454677	0.04066527	0.10276248
ENSG0000026	CISTR	lncRNA	0.59450354	0.8001003	NA
ENSG0000017	B3GNT2	protein_codir	0.59419923	0.00182339	0.00913845
ENSG0000013	FIGNL1	protein_codir	0.59416089	0.01795708	0.05458067
ENSG0000020	C6orf163	protein_codir	0.59414224	0.29827862	0.44912144
ENSG0000010	BMX	protein_codir	0.59394441	0.19019829	0.32471732
ENSG0000021	DNAJC9	protein_codir	0.59394398	7.47E-06	0.00010066
ENSG0000027	RP11-278C7.4	lncRNA	0.59382706	0.46387276	0.61375643
ENSG0000024	AC005329.7	lncRNA	0.59376545	0.18373165	0.31643501
ENSG0000010	MMP15	protein_codir	0.59376428	0.02027392	0.05992143
ENSG0000025	LINC02506	lncRNA	0.59369152	0.7974293	NA
ENSG0000014	ADM	protein_codir	0.59369016	0.25016704	0.3961026
ENSG0000026	NBPF11	protein_codir	0.59344436	0.00647455	0.02481643
ENSG0000013	RPL36	protein_codir	0.59334066	0.00112526	0.00627783
ENSG0000027	RP11-20I20.4	lncRNA	0.59311231	0.63765412	0.75709693
ENSG0000027	MAP3K4-AS1	lncRNA	0.59305289	0.20369679	0.34101741
ENSG0000013	SLCO1C1	protein_codir	0.59275654	0.17578772	0.30603572
ENSG0000011	EEF1B2	protein_codir	0.59265966	0.00048169	0.00317449
ENSG0000012	MND1	protein_codir	0.5926326	0.36413517	0.51815628
ENSG0000028	RP11-115L11.	lncRNA	0.59259287	0.79388365	NA
ENSG0000017	PIPOX	protein_codir	0.59235285	0.09052616	0.18699667
ENSG0000010	RNASEH2A	protein_codir	0.59231106	0.02780219	0.07672788

ENSG0000015	SCLT1	protein_codir	0.59230968	0.00016475	0.00132509
ENSG0000023	LINC01611	lncRNA	0.59230062	0.76360178	NA
ENSG0000025	THAP9-AS1	lncRNA	0.59220593	0.0013142	0.00711589
ENSG0000022	SPATA13-AS1	lncRNA	0.59212734	0.792404	NA
ENSG0000010	KLHL4	protein_codir	0.5920203	0.24248615	0.38738316
ENSG0000018	SLC9A9	protein_codir	0.59163968	0.05103201	0.12235279
ENSG0000016	BEST1	protein_codir	0.59157741	0.01159707	0.03909094
ENSG0000027	CTD-2515O10	lncRNA	0.59157121	0.42974381	0.58124962
ENSG0000026	LINC01711	lncRNA	0.59135207	0.38723655	0.54028459
ENSG0000026	CTC-260E6.6	lncRNA	0.59107668	0.22535148	0.36712828
ENSG0000016	HSF2BP	protein_codir	0.59102375	0.11304282	0.22091862
ENSG0000028	RP11-400F19.	lncRNA	0.59086555	0.4108013	0.56374253
ENSG0000012	DDX39A	protein_codir	0.59061596	0.02977852	0.08102479
ENSG0000016	NBPF20	protein_codir	0.59047722	0.00940068	0.03317604
ENSG0000025	PSMA3-AS1	lncRNA	0.59026825	0.01774081	0.05410791
ENSG0000003	MYOM2	protein_codir	0.59010029	0.23662022	0.38060977
ENSG0000027	RP11-490B18.	lncRNA	0.58975115	0.67022755	0.78243733
ENSG0000022	TMEM233	protein_codir	0.58970034	0.06744204	0.15108679
ENSG0000017	RPS6KA3	protein_codir	0.58925917	0.00105526	0.00597751
ENSG0000013	SLC38A2	protein_codir	0.58908411	0.00163851	0.00845423
ENSG0000027	RP11-480D4.6	lncRNA	0.58892134	0.35610413	0.5096692
ENSG0000014	SLC44A3	protein_codir	0.58854434	0.06464627	0.14639092
ENSG0000026	CTD-2653B5.1	lncRNA	0.58852011	0.68238553	0.79114672
ENSG0000013	FBN2	protein_codir	0.58849202	0.37578519	0.52930731
ENSG0000023	LINC02669	lncRNA	0.58820914	0.39178881	0.54473732
ENSG0000016	SLC30A8	protein_codir	0.58815231	0.49436214	0.64047535
ENSG0000010	PIEZO1	protein_codir	0.58814846	0.00107264	0.00605482
ENSG0000017	SLC38A9	protein_codir	0.58812653	0.00450146	0.01867869
ENSG0000025	BMF-AS1	lncRNA	0.58796877	0.76679185	NA
ENSG0000018	RNF222	protein_codir	0.58795033	0.57948196	0.71131468
ENSG0000016	RAB3IL1	protein_codir	0.58782328	0.02239024	0.06475587
ENSG0000018	FANCM	protein_codir	0.587624	5.55E-05	0.00053707
ENSG0000017	PPIH	protein_codir	0.58761983	0.00636684	0.02448513
ENSG0000015	HNMT	protein_codir	0.58761556	0.0012155	0.00668233
ENSG0000019	GMFB	protein_codir	0.58759756	0.00020909	0.00160238
ENSG0000023	MSH2-OT1	lncRNA	0.58729074	0.52532012	0.66724956
ENSG0000012	CD274	protein_codir	0.5869843	0.03164836	0.08478445
ENSG0000025	RP11-158M2.	lncRNA	0.58687707	0.63822779	0.75740533
ENSG0000024	LINC00461	lncRNA	0.58665874	0.49519314	0.6411905
ENSG0000017	ANKRD20A4P	protein_codir	0.58650256	0.41487639	0.56797452
ENSG0000017	SSC5D	protein_codir	0.58642364	0.16493574	0.29222672
ENSG0000017	MCC	protein_codir	0.58635123	0.00442639	0.01844272
ENSG0000006	IFI35	protein_codir	0.5861829	0.0028484	0.01294343
ENSG0000011	PLEKHB2	protein_codir	0.58616232	0.00555699	0.02200487
ENSG0000026	CTD-2342J14.	lncRNA	0.58609107	0.41599134	0.56881259
ENSG0000015	VSIG4	protein_codir	0.58593144	0.18394426	0.31674192
ENSG0000022	FAM155A-IT1	lncRNA	0.58584537	0.6715041	NA

ENSG000000	CELF2	protein_codir	0.58553839	2.47E-05	0.00027351
ENSG000001	SMYD2	protein_codir	0.58541504	0.00209874	0.01019291
ENSG000001	ZBTB33	protein_codir	0.58531878	0.00443596	0.0184631
ENSG000000	MMP2	protein_codir	0.58491181	0.10101936	0.2033366
ENSG000001	LINC01558	lncRNA	0.58466955	0.40947877	0.56250888
ENSG000002	SERPINB1	protein_codir	0.58466166	6.41E-05	0.00060308
ENSG000002	LINC01187	lncRNA	0.58465678	0.70895566	0.81099008
ENSG000001	SHLD1	protein_codir	0.5844309	0.01570504	0.0493822
ENSG000002	RP11-647F2.2	lncRNA	0.58424778	0.76808995	NA
ENSG000001	SERINC2	protein_codir	0.58421309	0.10051795	0.20248749
ENSG000002	CTD-3105H18	protein_codir	0.58419657	0.49017586	0.63696341
ENSG000002	AC147651.1	lncRNA	0.58413949	0.702345	0.80613235
ENSG000001	BIN1	protein_codir	0.58409502	0.00192058	0.00950822
ENSG000001	CNPY3	protein_codir	0.58398037	0.00567738	0.0223566
ENSG000000	CCNK	protein_codir	0.58381044	2.24E-05	0.0002523
ENSG000001	A1BG	protein_codir	0.58373866	0.05018218	0.12086081
ENSG000002	AF131215.2	lncRNA	0.58360688	0.08902888	0.18476052
ENSG000001	SMC2	protein_codir	0.58354009	0.00211892	0.01027179
ENSG000002	RP5-1021I20.(	lncRNA	0.58350214	0.67174486	NA
ENSG000001	UHMK1	protein_codir	0.58349636	0.01007965	0.03501249
ENSG000001	R3HDM4	protein_codir	0.58344924	0.00294612	0.01329465
ENSG000001	LMBR1L	protein_codir	0.58338477	0.01165268	0.03923049
ENSG000001	RPL13	protein_codir	0.58336005	0.002845	0.01293269
ENSG000001	RNASEK-C17o	protein_codir	0.58313826	0.20592047	0.34396876
ENSG000002	LINC00511	lncRNA	0.58288799	0.16536381	0.29275998
ENSG000002	GAS5	lncRNA	0.58265097	0.01355906	0.04406416
ENSG000000	HHAT	protein_codir	0.58261826	0.01941196	0.0579944
ENSG000002	CTD-2008L17.	lncRNA	0.58254235	0.76307474	NA
ENSG000001	NECAP2	protein_codir	0.58238239	0.00165254	0.00850794
ENSG000002	ZMYM4-AS1	lncRNA	0.58216588	0.85839488	NA
ENSG000002	RP11-435D7.3	lncRNA	0.58216588	0.85839488	NA
ENSG000002	RP11-145M4.	lncRNA	0.58216588	0.85839488	NA
ENSG000002	RP4-629B2.1	lncRNA	0.58216588	0.85839488	NA
ENSG000002	LINC01787	lncRNA	0.58216588	0.85839488	NA
ENSG000002	RP4-787H6.1	lncRNA	0.58216588	0.85839488	NA
ENSG000002	RP11-439A17	lncRNA	0.58216588	0.85839488	NA
ENSG000002	PPIAL4G	protein_codir	0.58216588	0.85839488	NA
ENSG000002	RP11-98D18.1	lncRNA	0.58216588	0.85839488	NA
ENSG000002	RP11-438F14.	protein_codir	0.58216588	0.85839488	NA
ENSG000002	LINC01865	lncRNA	0.58216588	0.85839488	NA
ENSG000002	AC141930.3	lncRNA	0.58216588	0.85839488	NA
ENSG000002	AC092580.3	lncRNA	0.58216588	0.85839488	NA
ENSG000002	MYCNOS	lncRNA	0.58216588	0.85839488	NA
ENSG000002	RP11-414K7.1	lncRNA	0.58216588	0.85839488	NA
ENSG000002	RP11-179A20	lncRNA	0.58216588	0.85839488	NA
ENSG000002	RP11-261A24	lncRNA	0.58216588	0.85839488	NA
ENSG000002	AC114752.3	lncRNA	0.58216588	0.85839488	NA

ENSG0000023	LINC02579	lncRNA	0.58216588	0.85839488	NA
ENSG0000018	LYG2	protein_codir	0.58216588	0.85839488	NA
ENSG0000023	LINC01849	lncRNA	0.58216588	0.85839488	NA
ENSG0000028	RP11-67L14.2	lncRNA	0.58216588	0.85839488	NA
ENSG0000028	RP11-313N8.1	lncRNA	0.58216588	0.85839488	NA
ENSG0000028	RP11-279E17.	lncRNA	0.58216588	0.85839488	NA
ENSG0000023	AC104777.3	lncRNA	0.58216588	0.85839488	NA
ENSG0000028	RP11-744C22.	lncRNA	0.58216588	0.85839488	NA
ENSG0000016	ZSWIM2	protein_codir	0.58216588	0.85839488	NA
ENSG0000022	LINC01802	lncRNA	0.58216588	0.85839488	NA
ENSG0000028	RP11-803J6.2	lncRNA	0.58216588	0.85839488	NA
ENSG0000023	AC067956.1	lncRNA	0.58216588	0.85839488	NA
ENSG0000022	AC093843.1	lncRNA	0.58216588	0.85839488	NA
ENSG0000016	CCDC140	lncRNA	0.58216588	0.85839488	NA
ENSG0000023	GCSIR	lncRNA	0.58216588	0.85839488	NA
ENSG0000017	TEX44	protein_codir	0.58216588	0.85839488	NA
ENSG0000022	AC097713.3	lncRNA	0.58216588	0.85839488	NA
ENSG0000028	RP11-18I13.1	lncRNA	0.58216588	0.85839488	NA
ENSG0000025	RP11-536I6.2	lncRNA	0.58216588	0.85839488	NA
ENSG0000016	RTP3	protein_codir	0.58216588	0.85839488	NA
ENSG0000027	RP11-157F20.	lncRNA	0.58216588	0.85839488	NA
ENSG0000028	RP11-321A23	lncRNA	0.58216588	0.85839488	NA
ENSG0000028	CSNKA2IP	protein_codir	0.58216588	0.85839488	NA
ENSG0000014	ADGRG7	protein_codir	0.58216588	0.85839488	NA
ENSG0000024	RP11-49I4.3	lncRNA	0.58216588	0.85839488	NA
ENSG0000024	LINC02024	lncRNA	0.58216588	0.85839488	NA
ENSG0000024	LINC02004	lncRNA	0.58216588	0.85839488	NA
ENSG0000024	SLC9A9-AS2	lncRNA	0.58216588	0.85839488	NA
ENSG0000024	RP11-167H9.4	lncRNA	0.58216588	0.85839488	NA
ENSG0000017	GPR149	protein_codir	0.58216588	0.85839488	NA
ENSG0000026	RP11-646E18.	lncRNA	0.58216588	0.85839488	NA
ENSG0000018	HTR3E	protein_codir	0.58216588	0.85839488	NA
ENSG0000024	RP13-497K6.1	lncRNA	0.58216588	0.85839488	NA
ENSG0000028	LINC02357	lncRNA	0.58216588	0.85839488	NA
ENSG0000024	SNCA-AS1	lncRNA	0.58216588	0.85839488	NA
ENSG0000028	RP11-68O1.1	lncRNA	0.58216588	0.85839488	NA
ENSG0000015	POU4F2	protein_codir	0.58216588	0.85839488	NA
ENSG0000025	LINC02507	lncRNA	0.58216588	0.85839488	NA
ENSG0000027	RP11-11N5.3	lncRNA	0.58216588	0.85839488	NA
ENSG0000024	LINC02119	lncRNA	0.58216588	0.85839488	NA
ENSG0000028	CTD-2249K22	lncRNA	0.58216588	0.85839488	NA
ENSG0000028	RP11-2N5.4	lncRNA	0.58216588	0.85839488	NA
ENSG0000022	CTC-546K23.1	lncRNA	0.58216588	0.85839488	NA
ENSG0000020	GRXCR2	protein_codir	0.58216588	0.85839488	NA
ENSG0000016	TLX3	protein_codir	0.58216588	0.85839488	NA
ENSG0000025	RP11-536N17	lncRNA	0.58216588	0.85839488	NA
ENSG0000025	RP11-1252I4.1	lncRNA	0.58216588	0.85839488	NA

ENSG0000027	RP11-679B17.	lncRNA	0.58216588	0.85839488	NA
ENSG0000014	SLC17A4	protein_codir	0.58216588	0.85839488	NA
ENSG0000023	HCG21	lncRNA	0.58216588	0.85839488	NA
ENSG0000028	RP3-475N16.8	lncRNA	0.58216588	0.85839488	NA
ENSG0000018	GSTA5	protein_codir	0.58216588	0.85839488	NA
ENSG0000025	RP3-468K18.5	protein_codir	0.58216588	0.85839488	NA
ENSG0000028	RP1-130E4.4	lncRNA	0.58216588	0.85839488	NA
ENSG0000028	RP11-351J23.	lncRNA	0.58216588	0.85839488	NA
ENSG0000022	AC005281.1	protein_codir	0.58216588	0.85839488	NA
ENSG0000023	AC007349.5	lncRNA	0.58216588	0.85839488	NA
ENSG0000028	RP4-537P9.1	lncRNA	0.58216588	0.85839488	NA
ENSG0000012	GNGT1	protein_codir	0.58216588	0.85839488	NA
ENSG0000027	GS1-293C5.1	lncRNA	0.58216588	0.85839488	NA
ENSG0000024	AC073934.6	lncRNA	0.58216588	0.85839488	NA
ENSG0000014	STRA8	protein_codir	0.58216588	0.85839488	NA
ENSG0000024	RP11-545G3.1	lncRNA	0.58216588	0.85839488	NA
ENSG0000028	RP5-876K2.1	lncRNA	0.58216588	0.85839488	NA
ENSG0000023	LINC01287	lncRNA	0.58216588	0.85839488	NA
ENSG0000025	CTD-2530N21	lncRNA	0.58216588	0.85839488	NA
ENSG0000025	RP11-566H8.3	lncRNA	0.58216588	0.85839488	NA
ENSG0000025	RP11-770E5.3	lncRNA	0.58216588	0.85839488	NA
ENSG0000025	RP11-434I12.4	lncRNA	0.58216588	0.85839488	NA
ENSG0000025	RP11-172E10.	lncRNA	0.58216588	0.85839488	NA
ENSG0000025	RP11-700E23.	lncRNA	0.58216588	0.85839488	NA
ENSG0000025	KB-1615E4.3	lncRNA	0.58216588	0.85839488	NA
ENSG0000025	KB-1639H6.2	lncRNA	0.58216588	0.85839488	NA
ENSG0000022	AF178030.2	lncRNA	0.58216588	0.85839488	NA
ENSG0000025	RP11-10J21.3	lncRNA	0.58216588	0.85839488	NA
ENSG0000023	RP11-264I13.2	lncRNA	0.58216588	0.85839488	NA
ENSG0000027	RP11-318K12.	lncRNA	0.58216588	0.85839488	NA
ENSG0000022	RP11-575L7.2	lncRNA	0.58216588	0.85839488	NA
ENSG0000028	RP11-526D8.1	lncRNA	0.58216588	0.85839488	NA
ENSG0000022	RP11-196I18.4	lncRNA	0.58216588	0.85839488	NA
ENSG0000023	TEX48	protein_codir	0.58216588	0.85839488	NA
ENSG0000019	OR1J2	protein_codir	0.58216588	0.85839488	NA
ENSG0000023	RP11-85O21.2	lncRNA	0.58216588	0.85839488	NA
ENSG0000017	CALML5	protein_codir	0.58216588	0.85839488	NA
ENSG0000028	RP11-393E10.	lncRNA	0.58216588	0.85839488	NA
ENSG0000023	LINC02634	lncRNA	0.58216588	0.85839488	NA
ENSG0000028	RP11-314J18.	lncRNA	0.58216588	0.85839488	NA
ENSG0000023	TLX1NB	lncRNA	0.58216588	0.85839488	NA
ENSG0000028	RP11-190J1.1	lncRNA	0.58216588	0.85839488	NA
ENSG0000023	EDRF1-AS1	lncRNA	0.58216588	0.85839488	NA
ENSG0000023	RP11-109A6.2	lncRNA	0.58216588	0.85839488	NA
ENSG0000025	RP11-304M2.	lncRNA	0.58216588	0.85839488	NA
ENSG0000020	KRTAP5-1	protein_codir	0.58216588	0.85839488	NA
ENSG0000022	OR52B4	protein_codir	0.58216588	0.85839488	NA

ENSG0000018	OR52N5	protein_codir	0.58216588	0.85839488	NA
ENSG0000024	ALKBH3-AS1	lncRNA	0.58216588	0.85839488	NA
ENSG0000025	LINC02685	lncRNA	0.58216588	0.85839488	NA
ENSG0000024	P4HA3-AS1	lncRNA	0.58216588	0.85839488	NA
ENSG0000017	OR2AT4	protein_codir	0.58216588	0.85839488	NA
ENSG0000025	RP11-113K21.	lncRNA	0.58216588	0.85839488	NA
ENSG0000025	RP11-317J19.	lncRNA	0.58216588	0.85839488	NA
ENSG0000025	RP11-688I9.4	lncRNA	0.58216588	0.85839488	NA
ENSG0000028	TEX52	protein_codir	0.58216588	0.85839488	NA
ENSG0000025	GAU1	lncRNA	0.58216588	0.85839488	NA
ENSG0000025	RP11-90D4.3	lncRNA	0.58216588	0.85839488	NA
ENSG0000025	RP11-625L16.	lncRNA	0.58216588	0.85839488	NA
ENSG0000015	LMNTD1	protein_codir	0.58216588	0.85839488	NA
ENSG0000025	RP11-438D14	lncRNA	0.58216588	0.85839488	NA
ENSG0000025	RP1-228P16.5	lncRNA	0.58216588	0.85839488	NA
ENSG0000024	RP11-588G21	lncRNA	0.58216588	0.85839488	NA
ENSG0000025	RP11-497G19	lncRNA	0.58216588	0.85839488	NA
ENSG0000025	RP11-407A16	lncRNA	0.58216588	0.85839488	NA
ENSG0000028	RP11-67L17.1	lncRNA	0.58216588	0.85839488	NA
ENSG0000028	RP11-531P20.	lncRNA	0.58216588	0.85839488	NA
ENSG0000022	SOX1-OT	lncRNA	0.58216588	0.85839488	NA
ENSG0000025	RP11-203M5.	lncRNA	0.58216588	0.85839488	NA
ENSG0000025	FOXG1-AS1	lncRNA	0.58216588	0.85839488	NA
ENSG0000025	RP11-269C4.2	lncRNA	0.58216588	0.85839488	NA
ENSG0000025	RP11-561B11.	lncRNA	0.58216588	0.85839488	NA
ENSG0000025	RP11-486O13	lncRNA	0.58216588	0.85839488	NA
ENSG0000010	LRRC74A	protein_codir	0.58216588	0.85839488	NA
ENSG0000025	RP11-1029J19	lncRNA	0.58216588	0.85839488	NA
ENSG0000027	GOLGA6L6	protein_codir	0.58216588	0.85839488	NA
ENSG0000026	RP11-473C18.	lncRNA	0.58216588	0.85839488	NA
ENSG0000025	RP11-718O11	lncRNA	0.58216588	0.85839488	NA
ENSG0000025	RP11-56B16.4	lncRNA	0.58216588	0.85839488	NA
ENSG0000025	CTD-2184D3.7	lncRNA	0.58216588	0.85839488	NA
ENSG0000018	REC114	protein_codir	0.58216588	0.85839488	NA
ENSG0000026	CTD-2026K11	lncRNA	0.58216588	0.85839488	NA
ENSG0000027	RP11-685G9.4	lncRNA	0.58216588	0.85839488	NA
ENSG0000025	ANKRD34C-AS	lncRNA	0.58216588	0.85839488	NA
ENSG0000028	RP11-354M13	lncRNA	0.58216588	0.85839488	NA
ENSG0000028	LINC02852	lncRNA	0.58216588	0.85839488	NA
ENSG0000014	RPL3L	protein_codir	0.58216588	0.85839488	NA
ENSG0000026	RP11-127I20.7	lncRNA	0.58216588	0.85839488	NA
ENSG0000014	SEPTIN12	protein_codir	0.58216588	0.85839488	NA
ENSG0000026	RP11-141O15	lncRNA	0.58216588	0.85839488	NA
ENSG0000026	AC135050.5	lncRNA	0.58216588	0.85839488	NA
ENSG0000010	PRSS54	protein_codir	0.58216588	0.85839488	NA
ENSG0000018	OR1G1	protein_codir	0.58216588	0.85839488	NA
ENSG0000026	RP11-141J13.	lncRNA	0.58216588	0.85839488	NA

ENSG0000023	AC022816.2	lncRNA	0.58216588	0.85839488	NA
ENSG0000026	RP11-849N15	lncRNA	0.58216588	0.85839488	NA
ENSG0000027	RP11-333J10.	lncRNA	0.58216588	0.85839488	NA
ENSG0000021	KRTAP2-4	protein_codir	0.58216588	0.85839488	NA
ENSG0000028	CTD-2534H9.1	lncRNA	0.58216588	0.85839488	NA
ENSG0000013	PYY	protein_codir	0.58216588	0.85839488	NA
ENSG0000025	STH	protein_codir	0.58216588	0.85839488	NA
ENSG0000026	RP11-304F15.	lncRNA	0.58216588	0.85839488	NA
ENSG0000028	RP11-583F2.7	lncRNA	0.58216588	0.85839488	NA
ENSG0000009	APOH	protein_codir	0.58216588	0.85839488	NA
ENSG0000026	CTD-2561B21	lncRNA	0.58216588	0.85839488	NA
ENSG0000026	RP11-498C9.1	lncRNA	0.58216588	0.85839488	NA
ENSG0000026	RP11-737O24	lncRNA	0.58216588	0.85839488	NA
ENSG0000026	RP11-838N2.5	lncRNA	0.58216588	0.85839488	NA
ENSG0000026	RP11-789C17.	lncRNA	0.58216588	0.85839488	NA
ENSG0000026	RP11-1157N2	lncRNA	0.58216588	0.85839488	NA
ENSG0000026	RP11-449D8.5	lncRNA	0.58216588	0.85839488	NA
ENSG0000026	LINC01908	lncRNA	0.58216588	0.85839488	NA
ENSG0000026	LINC01897	lncRNA	0.58216588	0.85839488	NA
ENSG0000026	MIR122HG	lncRNA	0.58216588	0.85839488	NA
ENSG0000026	LINC01910	lncRNA	0.58216588	0.85839488	NA
ENSG0000028	RP11-644A7.3	lncRNA	0.58216588	0.85839488	NA
ENSG0000020	C18orf63	protein_codir	0.58216588	0.85839488	NA
ENSG0000026	CTB-31O20.6	lncRNA	0.58216588	0.85839488	NA
ENSG0000026	AC092316.1	lncRNA	0.58216588	0.85839488	NA
ENSG0000017	OR7G2	protein_codir	0.58216588	0.85839488	NA
ENSG0000026	CTC-499B15.7	lncRNA	0.58216588	0.85839488	NA
ENSG0000026	LINC01855	lncRNA	0.58216588	0.85839488	NA
ENSG0000026	CTC-429P9.2	lncRNA	0.58216588	0.85839488	NA
ENSG0000020	LGALS7	protein_codir	0.58216588	0.85839488	NA
ENSG0000026	AC093063.3	lncRNA	0.58216588	0.85839488	NA
ENSG0000010	CEACAM5	protein_codir	0.58216588	0.85839488	NA
ENSG0000026	AC007193.6	lncRNA	0.58216588	0.85839488	NA
ENSG0000026	CTC-273B12.7	protein_codir	0.58216588	0.85839488	NA
ENSG0000026	CTB-33G10.11	lncRNA	0.58216588	0.85839488	NA
ENSG0000010	LIM2	protein_codir	0.58216588	0.85839488	NA
ENSG0000024	TARM1	protein_codir	0.58216588	0.85839488	NA
ENSG0000016	TMEM190	protein_codir	0.58216588	0.85839488	NA
ENSG0000014	ZIM3	protein_codir	0.58216588	0.85839488	NA
ENSG0000025	LINC00261	lncRNA	0.58216588	0.85839488	NA
ENSG0000028	RP11-359G22	lncRNA	0.58216588	0.85839488	NA
ENSG0000028	AC005914.1	lncRNA	0.58216588	0.85839488	NA
ENSG0000022	SAMSN1-AS1	lncRNA	0.58216588	0.85839488	NA
ENSG0000028	AF222684.1	lncRNA	0.58216588	0.85839488	NA
ENSG0000015	TMPRSS15	protein_codir	0.58216588	0.85839488	NA
ENSG0000022	AP000477.3	lncRNA	0.58216588	0.85839488	NA
ENSG0000023	AP001615.9	lncRNA	0.58216588	0.85839488	NA

ENSG0000027	CTA-390C10.9	lncRNA	0.58216588	0.85839488	NA
ENSG0000022	RP3-438O4.4	lncRNA	0.58216588	0.85839488	NA
ENSG0000010	SEC14L3	protein_codir	0.58216588	0.85839488	NA
ENSG0000023	MRTFA-AS1	lncRNA	0.58216588	0.85839488	NA
ENSG0000028	XX-FW80414D	lncRNA	0.58216588	0.85839488	NA
ENSG0000028	CTA-343C1.2	lncRNA	0.58216588	0.85839488	NA
ENSG0000023	CLDN34	protein_codir	0.58216588	0.85839488	NA
ENSG0000028	RP11-351K23.	lncRNA	0.58216588	0.85839488	NA
ENSG0000028	RP1-296G17.4	lncRNA	0.58216588	0.85839488	NA
ENSG0000028	RP5-1015P16.	lncRNA	0.58216588	0.85839488	NA
ENSG0000026	MAGEA9B	protein_codir	0.58216588	0.85839488	NA
ENSG0000016	PASD1	protein_codir	0.58216588	0.85839488	NA
ENSG0000012	CTAG2	protein_codir	0.58216588	0.85839488	NA
ENSG0000027	ENSG0000027	protein_codir	0.58216588	0.85839488	NA
ENSG0000022	CAMTA1-AS1	lncRNA	0.58216588	0.85839488	NA
ENSG0000027	PRAMEF18	protein_codir	0.58216588	0.85839488	NA
ENSG0000022	RP11-422P22.	lncRNA	0.58216588	0.85839488	NA
ENSG0000017	RNF186	protein_codir	0.58216588	0.85839488	NA
ENSG0000022	TEX46	protein_codir	0.58216588	0.85839488	NA
ENSG0000023	LINC02800	lncRNA	0.58216588	0.85839488	NA
ENSG0000027	RP1-317E23.7	lncRNA	0.58216588	0.85839488	NA
ENSG0000025	RP1-34M23.5	lncRNA	0.58216588	0.85839488	NA
ENSG0000023	RP11-296A18	lncRNA	0.58216588	0.85839488	NA
ENSG0000017	INSL5	protein_codir	0.58216588	0.85839488	NA
ENSG0000023	DEPDC1-AS1	lncRNA	0.58216588	0.85839488	NA
ENSG0000023	DPYD-IT1	lncRNA	0.58216588	0.85839488	NA
ENSG0000028	RP11-84O12.5	lncRNA	0.58216588	0.85839488	NA
ENSG0000016	HJV	protein_codir	0.58216588	0.85839488	NA
ENSG0000016	IVL	protein_codir	0.58216588	0.85839488	NA
ENSG0000028	RP11-274N19	lncRNA	0.58216588	0.85839488	NA
ENSG0000028	RP11-122G18	lncRNA	0.58216588	0.85839488	NA
ENSG0000028	RP4-797M17.	lncRNA	0.58216588	0.85839488	NA
ENSG0000012	RGSL1	protein_codir	0.58216588	0.85839488	NA
ENSG0000023	LINC01222	lncRNA	0.58216588	0.85839488	NA
ENSG0000028	RP11-80N9.1	lncRNA	0.58216588	0.85839488	NA
ENSG0000028	LINC00538	lncRNA	0.58216588	0.85839488	NA
ENSG0000023	RP5-940F7.2	lncRNA	0.58216588	0.85839488	NA
ENSG0000028	RP4-764D2.2	lncRNA	0.58216588	0.85839488	NA
ENSG0000023	RP11-177F15.	lncRNA	0.58216588	0.85839488	NA
ENSG0000020	OR2T5	protein_codir	0.58216588	0.85839488	NA
ENSG0000017	OR2T35	protein_codir	0.58216588	0.85839488	NA
ENSG0000023	LINC01250	lncRNA	0.58216588	0.85839488	NA
ENSG0000023	AC019118.3	lncRNA	0.58216588	0.85839488	NA
ENSG0000023	MIR7515HG	lncRNA	0.58216588	0.85839488	NA
ENSG0000022	AC016730.1	lncRNA	0.58216588	0.85839488	NA
ENSG0000028	RP11-355B11.	lncRNA	0.58216588	0.85839488	NA
ENSG0000023	AC012370.2	lncRNA	0.58216588	0.85839488	NA

ENSG0000023	AC007389.3	lncRNA	0.58216588	0.85839488	NA
ENSG0000028	RP11-92A1.1	lncRNA	0.58216588	0.85839488	NA
ENSG0000020	NMS	protein_codir	0.58216588	0.85839488	NA
ENSG0000022	AC073987.1	lncRNA	0.58216588	0.85839488	NA
ENSG0000013	IL36A	protein_codir	0.58216588	0.85839488	NA
ENSG0000022	AC079154.1	lncRNA	0.58216588	0.85839488	NA
ENSG0000028	LINC01958	lncRNA	0.58216588	0.85839488	NA
ENSG0000022	TANK-AS1	lncRNA	0.58216588	0.85839488	NA
ENSG0000018	KCNH7	protein_codir	0.58216588	0.85839488	NA
ENSG0000022	AC009495.4	lncRNA	0.58216588	0.85839488	NA
ENSG0000022	TTC21B-AS1	lncRNA	0.58216588	0.85839488	NA
ENSG0000027	RP11-347P5.1	lncRNA	0.58216588	0.85839488	NA
ENSG0000019	SPAG16-DT	lncRNA	0.58216588	0.85839488	NA
ENSG0000022	AC106876.2	lncRNA	0.58216588	0.85839488	NA
ENSG0000024	UGT1A4	protein_codir	0.58216588	0.85839488	NA
ENSG0000019	AC079612.1	lncRNA	0.58216588	0.85839488	NA
ENSG0000017	OR6B3	protein_codir	0.58216588	0.85839488	NA
ENSG0000021	AC011298.2	lncRNA	0.58216588	0.85839488	NA
ENSG0000023	AC114730.7	lncRNA	0.58216588	0.85839488	NA
ENSG0000023	AC090952.5	lncRNA	0.58216588	0.85839488	NA
ENSG0000022	ZNF385D-AS1	lncRNA	0.58216588	0.85839488	NA
ENSG0000028	RP13-546I2.2	lncRNA	0.58216588	0.85839488	NA
ENSG0000023	C3orf84	protein_codir	0.58216588	0.85839488	NA
ENSG0000028	RP11-24O17.1	lncRNA	0.58216588	0.85839488	NA
ENSG0000023	RP11-451B8.1	lncRNA	0.58216588	0.85839488	NA
ENSG0000024	LINC02042	lncRNA	0.58216588	0.85839488	NA
ENSG0000018	ARGFX	protein_codir	0.58216588	0.85839488	NA
ENSG0000025	TMEM108-AS	lncRNA	0.58216588	0.85839488	NA
ENSG0000023	PLSCR5	protein_codir	0.58216588	0.85839488	NA
ENSG0000013	GPR87	protein_codir	0.58216588	0.85839488	NA
ENSG0000014	SPATA16	protein_codir	0.58216588	0.85839488	NA
ENSG0000022	RP11-211G3.3	lncRNA	0.58216588	0.85839488	NA
ENSG0000028	RP11-586D19	lncRNA	0.58216588	0.85839488	NA
ENSG0000028	RP11-1156I21	lncRNA	0.58216588	0.85839488	NA
ENSG0000028	LINC02498	lncRNA	0.58216588	0.85839488	NA
ENSG0000028	AC007106.2	lncRNA	0.58216588	0.85839488	NA
ENSG0000015	TMPRSS11D	protein_codir	0.58216588	0.85839488	NA
ENSG0000013	CSN2	protein_codir	0.58216588	0.85839488	NA
ENSG0000025	LINC02483	lncRNA	0.58216588	0.85839488	NA
ENSG0000024	RP11-767N15	lncRNA	0.58216588	0.85839488	NA
ENSG0000015	SLC25A31	protein_codir	0.58216588	0.85839488	NA
ENSG0000025	RP11-98O2.1	lncRNA	0.58216588	0.85839488	NA
ENSG0000028	RP11-308D13	lncRNA	0.58216588	0.85839488	NA
ENSG0000024	RP11-6E9.4	lncRNA	0.58216588	0.85839488	NA
ENSG0000025	CTD-2215L10.	lncRNA	0.58216588	0.85839488	NA
ENSG0000024	LINC02212	lncRNA	0.58216588	0.85839488	NA
ENSG0000025	RP11-466P24.	lncRNA	0.58216588	0.85839488	NA

ENSG0000024	RP11-78C3.1	lncRNA	0.58216588	0.85839488	NA
ENSG0000028	LINC01949	lncRNA	0.58216588	0.85839488	NA
ENSG0000025	RP11-414H23	lncRNA	0.58216588	0.85839488	NA
ENSG0000025	CTC-459M5.1	lncRNA	0.58216588	0.85839488	NA
ENSG0000017	FNDC9	protein_codir	0.58216588	0.85839488	NA
ENSG0000017	EIF4E1B	protein_codir	0.58216588	0.85839488	NA
ENSG0000028	RP1-80B9.4	lncRNA	0.58216588	0.85839488	NA
ENSG0000022	RP3-470L22.1	lncRNA	0.58216588	0.85839488	NA
ENSG0000028	RP1-45P21.8	lncRNA	0.58216588	0.85839488	NA
ENSG0000020	TRIM31	protein_codir	0.58216588	0.85839488	NA
ENSG0000022	LINCMD1	lncRNA	0.58216588	0.85839488	NA
ENSG0000024	GSTA2	protein_codir	0.58216588	0.85839488	NA
ENSG0000022	LINC01626	lncRNA	0.58216588	0.85839488	NA
ENSG0000022	RP11-554D15	lncRNA	0.58216588	0.85839488	NA
ENSG0000023	RP11-16C18.3	lncRNA	0.58216588	0.85839488	NA
ENSG0000028	RP11-64I5.3	lncRNA	0.58216588	0.85839488	NA
ENSG0000011	PRDM13	protein_codir	0.58216588	0.85839488	NA
ENSG0000022	LINC02526	lncRNA	0.58216588	0.85839488	NA
ENSG0000021	RP11-306O13	lncRNA	0.58216588	0.85839488	NA
ENSG0000023	SYNJ2-IT1	lncRNA	0.58216588	0.85839488	NA
ENSG0000011	C6orf118	protein_codir	0.58216588	0.85839488	NA
ENSG0000022	PRKAR1B-AS2	lncRNA	0.58216588	0.85839488	NA
ENSG0000022	AC005019.3	lncRNA	0.58216588	0.85839488	NA
ENSG0000022	AC099342.1	lncRNA	0.58216588	0.85839488	NA
ENSG0000026	RP5-978I12.1	lncRNA	0.58216588	0.85839488	NA
ENSG0000025	HOXA10-HOX	protein_codir	0.58216588	0.85839488	NA
ENSG0000025	RP1-170O19.1	lncRNA	0.58216588	0.85839488	NA
ENSG0000028	CTA-348C20.1	lncRNA	0.58216588	0.85839488	NA
ENSG0000028	CTC-736O2.1	lncRNA	0.58216588	0.85839488	NA
ENSG0000023	AC003092.1	lncRNA	0.58216588	0.85839488	NA
ENSG0000016	MUC17	protein_codir	0.58216588	0.85839488	NA
ENSG0000026	SPDYE6	protein_codir	0.58216588	0.85839488	NA
ENSG0000023	RP11-514P8.2	lncRNA	0.58216588	0.85839488	NA
ENSG0000027	RP11-62J1.4	lncRNA	0.58216588	0.85839488	NA
ENSG0000023	AC008154.4	lncRNA	0.58216588	0.85839488	NA
ENSG0000028	RNY5	lncRNA	0.58216588	0.85839488	NA
ENSG0000022	AC004941.3	lncRNA	0.58216588	0.85839488	NA
ENSG0000023	ZNF775-AS1	lncRNA	0.58216588	0.85839488	NA
ENSG0000016	GBX1	protein_codir	0.58216588	0.85839488	NA
ENSG0000020	AC006019.3	lncRNA	0.58216588	0.85839488	NA
ENSG0000015	HTR5A	protein_codir	0.58216588	0.85839488	NA
ENSG0000028	RP11-439C15.	lncRNA	0.58216588	0.85839488	NA
ENSG0000024	AF131216.6	lncRNA	0.58216588	0.85839488	NA
ENSG0000028	RP11-148O21	lncRNA	0.58216588	0.85839488	NA
ENSG0000028	RP11-481A20	lncRNA	0.58216588	0.85839488	NA
ENSG0000018	RP11-161I2.1	lncRNA	0.58216588	0.85839488	NA
ENSG0000028	RP11-875O11	protein_codir	0.58216588	0.85839488	NA

ENSG0000025	RP11-299D14	lncRNA	0.58216588	0.85839488	NA
ENSG0000025	HMBOX1-IT1	lncRNA	0.58216588	0.85839488	NA
ENSG0000025	RP11-51J9.4	lncRNA	0.58216588	0.85839488	NA
ENSG0000025	RP11-27P7.1	lncRNA	0.58216588	0.85839488	NA
ENSG0000025	RP11-3N13.2	lncRNA	0.58216588	0.85839488	NA
ENSG0000025	LINC02886	lncRNA	0.58216588	0.85839488	NA
ENSG0000014	PSKH2	protein_codir	0.58216588	0.85839488	NA
ENSG0000026	KB-1000E4.2	lncRNA	0.58216588	0.85839488	NA
ENSG0000025	KB-1460A1.3	lncRNA	0.58216588	0.85839488	NA
ENSG0000024	RP11-713M15	lncRNA	0.58216588	0.85839488	NA
ENSG0000028	TMEM75	lncRNA	0.58216588	0.85839488	NA
ENSG0000028	RP11-1057N3	lncRNA	0.58216588	0.85839488	NA
ENSG0000025	RP11-398H6.1	lncRNA	0.58216588	0.85839488	NA
ENSG0000025	RP11-909N17	lncRNA	0.58216588	0.85839488	NA
ENSG0000025	RP11-56F10.3	lncRNA	0.58216588	0.85839488	NA
ENSG0000027	RP11-569G13	lncRNA	0.58216588	0.85839488	NA
ENSG0000020	OR13C3	protein_codir	0.58216588	0.85839488	NA
ENSG0000023	RP11-406O23	lncRNA	0.58216588	0.85839488	NA
ENSG0000017	OR1N2	protein_codir	0.58216588	0.85839488	NA
ENSG0000016	SOHLH1	protein_codir	0.58216588	0.85839488	NA
ENSG0000023	RP11-526P5.2	lncRNA	0.58216588	0.85839488	NA
ENSG0000017	UCN3	protein_codir	0.58216588	0.85839488	NA
ENSG0000028	ICI-14BB7.1	lncRNA	0.58216588	0.85839488	NA
ENSG0000028	FAM245B	lncRNA	0.58216588	0.85839488	NA
ENSG0000023	LINC02621	lncRNA	0.58216588	0.85839488	NA
ENSG0000022	RP11-227H15	lncRNA	0.58216588	0.85839488	NA
ENSG0000024	RP11-429G19	lncRNA	0.58216588	0.85839488	NA
ENSG0000021	MUC5AC	protein_codir	0.58216588	0.85839488	NA
ENSG0000022	AC109309.4	lncRNA	0.58216588	0.85839488	NA
ENSG0000018	OR56A5	protein_codir	0.58216588	0.85839488	NA
ENSG0000016	OR2D2	protein_codir	0.58216588	0.85839488	NA
ENSG0000017	OR10A3	protein_codir	0.58216588	0.85839488	NA
ENSG0000017	C11orf16	protein_codir	0.58216588	0.85839488	NA
ENSG0000025	RP11-390K5.3	lncRNA	0.58216588	0.85839488	NA
ENSG0000025	LINC02750	lncRNA	0.58216588	0.85839488	NA
ENSG0000018	OR5AK2	protein_codir	0.58216588	0.85839488	NA
ENSG0000017	OR5B12	protein_codir	0.58216588	0.85839488	NA
ENSG0000016	MS4A5	protein_codir	0.58216588	0.85839488	NA
ENSG0000021	MS4A18	protein_codir	0.58216588	0.85839488	NA
ENSG0000025	RP11-881M11	lncRNA	0.58216588	0.85839488	NA
ENSG0000025	GRM5-AS1	lncRNA	0.58216588	0.85839488	NA
ENSG0000028	RP11-794P6.8	lncRNA	0.58216588	0.85839488	NA
ENSG0000025	RP11-794P6.6	lncRNA	0.58216588	0.85839488	NA
ENSG0000019	OR10D3	protein_codir	0.58216588	0.85839488	NA
ENSG0000019	OR8D1	protein_codir	0.58216588	0.85839488	NA
ENSG0000025	RP11-687M24	lncRNA	0.58216588	0.85839488	NA
ENSG0000026	RP11-673E11.	lncRNA	0.58216588	0.85839488	NA

ENSG0000025	RP11-319E16.	lncRNA	0.58216588	0.85839488	NA
ENSG0000025	MIR200CHG	lncRNA	0.58216588	0.85839488	NA
ENSG0000012	TAS2R8	protein_codir	0.58216588	0.85839488	NA
ENSG0000028	RP11-161A14	lncRNA	0.58216588	0.85839488	NA
ENSG0000025	RP11-625L16.	lncRNA	0.58216588	0.85839488	NA
ENSG0000028	RP11-612B6.1	lncRNA	0.58216588	0.85839488	NA
ENSG0000025	RP11-513G19	lncRNA	0.58216588	0.85839488	NA
ENSG0000025	RP11-843B15.	lncRNA	0.58216588	0.85839488	NA
ENSG0000024	RP11-847H18	lncRNA	0.58216588	0.85839488	NA
ENSG0000025	RP11-845M18	lncRNA	0.58216588	0.85839488	NA
ENSG0000016	KRT84	protein_codir	0.58216588	0.85839488	NA
ENSG0000018	KRT3	protein_codir	0.58216588	0.85839488	NA
ENSG0000025	RP11-81K13.1	lncRNA	0.58216588	0.85839488	NA
ENSG0000025	RP1-97G4.1	lncRNA	0.58216588	0.85839488	NA
ENSG0000025	RP11-248E9.6	lncRNA	0.58216588	0.85839488	NA
ENSG0000025	RP11-256L6.2	lncRNA	0.58216588	0.85839488	NA
ENSG0000025	RP11-554E23.	lncRNA	0.58216588	0.85839488	NA
ENSG0000028	RP11-781C15.	lncRNA	0.58216588	0.85839488	NA
ENSG0000025	RP1-46F2.3	lncRNA	0.58216588	0.85839488	NA
ENSG0000025	RP11-780K2.1	lncRNA	0.58216588	0.85839488	NA
ENSG0000027	RP11-240G22	lncRNA	0.58216588	0.85839488	NA
ENSG0000025	RP11-7M8.2	lncRNA	0.58216588	0.85839488	NA
ENSG0000027	RP13-820C6.4	lncRNA	0.58216588	0.85839488	NA
ENSG0000022	RP11-61K9.2	lncRNA	0.58216588	0.85839488	NA
ENSG0000023	RP11-37L2.1	lncRNA	0.58216588	0.85839488	NA
ENSG0000027	RP11-266E6.3	lncRNA	0.58216588	0.85839488	NA
ENSG0000028	RP11-98D3.2	lncRNA	0.58216588	0.85839488	NA
ENSG0000008	CPB2	protein_codir	0.58216588	0.85839488	NA
ENSG0000023	LINC00364	lncRNA	0.58216588	0.85839488	NA
ENSG0000026	LINC00555	lncRNA	0.58216588	0.85839488	NA
ENSG0000023	COL4A2-AS1	lncRNA	0.58216588	0.85839488	NA
ENSG0000017	OR4N2	protein_codir	0.58216588	0.85839488	NA
ENSG0000018	OR6S1	protein_codir	0.58216588	0.85839488	NA
ENSG0000025	RP11-388E23.	lncRNA	0.58216588	0.85839488	NA
ENSG0000028	CTD-2216L14.	lncRNA	0.58216588	0.85839488	NA
ENSG0000025	RP11-148E17.	lncRNA	0.58216588	0.85839488	NA
ENSG0000025	CTD-2058B24	lncRNA	0.58216588	0.85839488	NA
ENSG0000028	RP11-1150C1	lncRNA	0.58216588	0.85839488	NA
ENSG0000017	GPHB5	protein_codir	0.58216588	0.85839488	NA
ENSG0000017	GPX2	protein_codir	0.58216588	0.85839488	NA
ENSG0000025	RP3-414A15.1	lncRNA	0.58216588	0.85839488	NA
ENSG0000025	RP11-789A21	lncRNA	0.58216588	0.85839488	NA
ENSG0000025	RP11-944C7.1	lncRNA	0.58216588	0.85839488	NA
ENSG0000025	RP11-895M11	lncRNA	0.58216588	0.85839488	NA
ENSG0000017	SERPINA6	protein_codir	0.58216588	0.85839488	NA
ENSG0000025	RP11-61O1.1	lncRNA	0.58216588	0.85839488	NA
ENSG0000025	LINC00677	lncRNA	0.58216588	0.85839488	NA

ENSG0000026	RP11-2F9.4	lncRNA	0.58216588	0.85839488	NA
ENSG0000025	RP11-701H24	lncRNA	0.58216588	0.85839488	NA
ENSG0000025	RP11-720L8.1	lncRNA	0.58216588	0.85839488	NA
ENSG0000027	RP11-56B16.6	lncRNA	0.58216588	0.85839488	NA
ENSG0000026	RP11-1129I3.1	lncRNA	0.58216588	0.85839488	NA
ENSG0000025	RP11-255M2.	lncRNA	0.58216588	0.85839488	NA
ENSG0000028	RP11-753A21	lncRNA	0.58216588	0.85839488	NA
ENSG0000025	RP11-66B24.5	lncRNA	0.58216588	0.85839488	NA
ENSG0000026	LA16c-395F10	lncRNA	0.58216588	0.85839488	NA
ENSG0000026	LA16c-361A3.	lncRNA	0.58216588	0.85839488	NA
ENSG0000027	LA16c-380H5.	lncRNA	0.58216588	0.85839488	NA
ENSG0000026	RP11-473I1.5	lncRNA	0.58216588	0.85839488	NA
ENSG0000026	CTD-2194A8.2	lncRNA	0.58216588	0.85839488	NA
ENSG0000027	RP11-452L6.8	lncRNA	0.58216588	0.85839488	NA
ENSG0000027	TP53TG3E	protein_codir	0.58216588	0.85839488	NA
ENSG0000016	C16orf78	protein_codir	0.58216588	0.85839488	NA
ENSG0000026	CTD-2012K14	lncRNA	0.58216588	0.85839488	NA
ENSG0000023	AC009095.4	lncRNA	0.58216588	0.85839488	NA
ENSG0000026	RP11-432I5.2	lncRNA	0.58216588	0.85839488	NA
ENSG0000026	RP11-384M15	lncRNA	0.58216588	0.85839488	NA
ENSG0000026	RP11-328J14.	lncRNA	0.58216588	0.85839488	NA
ENSG0000027	RP11-77K12.1	lncRNA	0.58216588	0.85839488	NA
ENSG0000026	RP11-863P13.	lncRNA	0.58216588	0.85839488	NA
ENSG0000022	AL450226.2	lncRNA	0.58216588	0.85839488	NA
ENSG0000026	CTD-3060P21	lncRNA	0.58216588	0.85839488	NA
ENSG0000017	OR1A1	protein_codir	0.58216588	0.85839488	NA
ENSG0000028	RP11-477N12	lncRNA	0.58216588	0.85839488	NA
ENSG0000026	RP11-214O1.1	lncRNA	0.58216588	0.85839488	NA
ENSG0000027	RP11-219A15	lncRNA	0.58216588	0.85839488	NA
ENSG0000027	CTB-96E2.2	protein_codir	0.58216588	0.85839488	NA
ENSG0000024	KRTAP4-2	protein_codir	0.58216588	0.85839488	NA
ENSG0000028	RP5-1067M6.	lncRNA	0.58216588	0.85839488	NA
ENSG0000026	RP11-433M22	lncRNA	0.58216588	0.85839488	NA
ENSG0000024	CTD-2377D24	lncRNA	0.58216588	0.85839488	NA
ENSG0000026	LINC01982	lncRNA	0.58216588	0.85839488	NA
ENSG0000026	CTD-2319I12.	lncRNA	0.58216588	0.85839488	NA
ENSG0000026	PRKCA-AS1	lncRNA	0.58216588	0.85839488	NA
ENSG0000022	ROCR	lncRNA	0.58216588	0.85839488	NA
ENSG0000026	RP11-75C10.9	lncRNA	0.58216588	0.85839488	NA
ENSG0000026	RP13-638C3.3	lncRNA	0.58216588	0.85839488	NA
ENSG0000026	RP11-806L2.2	lncRNA	0.58216588	0.85839488	NA
ENSG0000026	LINC01892	lncRNA	0.58216588	0.85839488	NA
ENSG0000028	RP11-802O4.1	lncRNA	0.58216588	0.85839488	NA
ENSG0000026	RP11-905K4.1	lncRNA	0.58216588	0.85839488	NA
ENSG0000028	RP11-60G10.1	lncRNA	0.58216588	0.85839488	NA
ENSG0000015	RIT2	protein_codir	0.58216588	0.85839488	NA
ENSG0000028	AC008993.3	lncRNA	0.58216588	0.85839488	NA

ENSG0000023	OR7E24	protein_codir	0.58216588	0.85839488	NA
ENSG0000025	CTD-2006C1.1	protein_codir	0.58216588	0.85839488	NA
ENSG0000026	CALR3	protein_codir	0.58216588	0.85839488	NA
ENSG0000026	CTD-3032J10.	lncRNA	0.58216588	0.85839488	NA
ENSG0000026	AC005197.2	lncRNA	0.58216588	0.85839488	NA
ENSG0000012	IRGC	protein_codir	0.58216588	0.85839488	NA
ENSG0000020	RP11-352D3.2	lncRNA	0.58216588	0.85839488	NA
ENSG0000027	MCM8-AS1	lncRNA	0.58216588	0.85839488	NA
ENSG0000016	LINC01620	lncRNA	0.58216588	0.85839488	NA
ENSG0000012	SEMG1	protein_codir	0.58216588	0.85839488	NA
ENSG0000023	RP5-955M13.	lncRNA	0.58216588	0.85839488	NA
ENSG0000012	GCNT7	protein_codir	0.58216588	0.85839488	NA
ENSG0000012	CTCFL	protein_codir	0.58216588	0.85839488	NA
ENSG0000028	RP11-40E19.1	lncRNA	0.58216588	0.85839488	NA
ENSG0000016	TFF2	protein_codir	0.58216588	0.85839488	NA
ENSG0000018	KRTAP10-6	protein_codir	0.58216588	0.85839488	NA
ENSG0000022	AC004471.9	lncRNA	0.58216588	0.85839488	NA
ENSG0000022	AC002472.11	lncRNA	0.58216588	0.85839488	NA
ENSG0000027	RP1-63G5.8	lncRNA	0.58216588	0.85839488	NA
ENSG0000022	RP5-1039K5.1	lncRNA	0.58216588	0.85839488	NA
ENSG0000023	RP3-508I15.1	lncRNA	0.58216588	0.85839488	NA
ENSG0000028	TBC1D22A-AS	lncRNA	0.58216588	0.85839488	NA
ENSG0000010	GLRA2	protein_codir	0.58216588	0.85839488	NA
ENSG0000022	PNMA6F	protein_codir	0.58216588	0.85839488	NA
ENSG0000028	RP11-400O10	lncRNA	0.58216588	0.85839488	NA
ENSG0000023	TTTY18	lncRNA	0.58216588	0.85839488	NA
ENSG0000023	AC007359.6	lncRNA	0.58216588	0.85839488	NA
ENSG0000023	AC008175.1	lncRNA	0.58216588	0.85839488	NA
ENSG0000022	LINC00266-4F	lncRNA	0.58216588	0.85839488	NA
ENSG0000023	LRRC8C-DT	lncRNA	0.58214359	0.02632948	0.07347043
ENSG0000018	PLA2G6	protein_codir	0.58213672	0.06154544	0.14077522
ENSG0000028	XX-FW83563E	lncRNA	0.58211268	0.17221765	0.30166486
ENSG0000012	PFKFB2	protein_codir	0.58189001	0.00283868	0.01290819
ENSG0000013	CYP2E1	protein_codir	0.58175769	0.10179995	0.20452651
ENSG0000024	CFAP57	protein_codir	0.58150784	0.27224754	0.42018778
ENSG0000025	RP11-468E2.4	protein_codir	0.58107414	0.21094248	0.35032358
ENSG0000025	RP11-192H23	protein_codir	0.58106541	0.46553472	0.61525604
ENSG0000018	SMYD3	protein_codir	0.58083325	0.00363126	0.01572565
ENSG0000018	H1-6	protein_codir	0.58071198	0.84028711	NA
ENSG0000010	CAPN15	protein_codir	0.5806499	0.00637977	0.02451846
ENSG0000014	SCAMP2	protein_codir	0.58064013	0.00065746	0.00408894
ENSG0000006	CYB5R4	protein_codir	0.58059557	0.00173305	0.00880945
ENSG0000026	CTD-2545M3.	lncRNA	0.58050408	0.44679194	0.59773091
ENSG0000020	TATDN3	protein_codir	0.58004037	0.00235225	0.01115956
ENSG0000016	MIOS	protein_codir	0.57991808	4.45E-05	0.00044236
ENSG0000025	LINC02321	lncRNA	0.5797725	0.56381708	0.69826148
ENSG0000019	ZBTB44	protein_codir	0.57976829	0.00029719	0.00213688

ENSG0000016	USP24	protein_codir	0.57950945	0.00018611	0.0014552
ENSG0000010	CHIC2	protein_codir	0.57941641	0.00407401	0.01729279
ENSG0000025	EIF5AL1	protein_codir	0.57939961	0.14346477	0.26356098
ENSG0000016	MARCHF8	protein_codir	0.57939336	0.00052411	0.00339812
ENSG0000027	RP11-305K5.1	lncRNA	0.5791311	0.12339135	0.23598318
ENSG0000017	LIPT2	protein_codir	0.57859914	0.05161756	0.12324016
ENSG0000019	ELANE	protein_codir	0.57835865	0.28815389	0.43782651
ENSG0000018	PRR19	protein_codir	0.57826471	0.33707297	0.48994253
ENSG0000026	PLCE1-AS1	lncRNA	0.57795041	0.4867081	0.63394849
ENSG0000016	ZNF394	protein_codir	0.57772998	1.85E-05	0.00021489
ENSG0000012	PILRB	protein_codir	0.57754875	0.09298684	0.19100821
ENSG0000016	RPSA	protein_codir	0.57749881	0.00252681	0.01177699
ENSG0000012	SOX9	protein_codir	0.57730227	0.11352565	0.22168051
ENSG0000012	SHBG	protein_codir	0.57697517	0.37349179	0.52704457
ENSG0000013	DTNB	protein_codir	0.57672279	0.00021554	0.0016391
ENSG0000026	RP11-49K24.8	lncRNA	0.57672244	0.65885326	0.773288
ENSG0000011	MACROH2A1	protein_codir	0.57669453	0.00135201	0.00726931
ENSG0000011	PIKFYVE	protein_codir	0.5766924	0.00144897	0.00767709
ENSG0000020	SRRM2-AS1	lncRNA	0.57652545	0.15035688	0.27276994
ENSG0000026	RP11-180I4.4	lncRNA	0.57645544	0.57344809	0.7063252
ENSG0000025	OVCH1-AS1	lncRNA	0.57645422	0.20369608	0.34101741
ENSG0000016	YIF1B	protein_codir	0.57641956	0.02628865	0.07337974
ENSG0000027	RP11-425A6.5	lncRNA	0.57627219	0.47652513	0.62469145
ENSG0000018	DGCR6	protein_codir	0.57604651	0.17023171	0.29907766
ENSG0000016	TEPSIN	protein_codir	0.57566928	0.06803325	0.15194294
ENSG0000007	HOXA9	protein_codir	0.5755735	0.30559828	0.45724825
ENSG0000021	TSTD1	protein_codir	0.57555269	0.19690404	0.33295516
ENSG0000010	ERICH1	protein_codir	0.57553632	0.01436871	0.04608799
ENSG0000025	RP11-151N17	lncRNA	0.57546528	0.51328438	0.65682518
ENSG0000017	MUC20	protein_codir	0.57536335	0.11241954	0.22013797
ENSG0000026	RP11-712P20.	lncRNA	0.5751829	0.40706111	0.55999878
ENSG0000018	RALGAPA2	protein_codir	0.57516358	0.01988572	0.05904574
ENSG0000025	SYS1-DBNDD2	protein_codir	0.57515232	0.22949865	0.37199252
ENSG0000010	ARSA	protein_codir	0.57512295	0.002572	0.01193728
ENSG0000025	RP11-728F11.	lncRNA	0.57509464	0.31973096	0.47181542
ENSG0000027	RP11-171I2.2	lncRNA	0.57493109	0.05037298	0.12123577
ENSG0000023	RP11-475I24.3	lncRNA	0.57468912	0.21810203	0.35848476
ENSG0000011	TMED5	protein_codir	0.57437502	0.00525495	0.02108699
ENSG0000005	THOC3	protein_codir	0.57411549	0.00634867	0.02442884
ENSG0000018	SLITRK6	protein_codir	0.57401798	0.33506235	0.48795859
ENSG0000028	RP11-511P7.5	protein_codir	0.57399976	0.05310388	0.1258887
ENSG0000006	KIF2A	protein_codir	0.57399532	0.00254625	0.01184242
ENSG0000027	RP11-147L13.	lncRNA	0.57386243	0.00760799	0.02810012
ENSG0000022	DHRSX-IT1	lncRNA	0.57373659	0.33683159	0.48992921
ENSG0000016	LRR1	protein_codir	0.5735477	0.00011496	0.00097939
ENSG0000010	NUDT1	protein_codir	0.57343163	0.00066647	0.00413848
ENSG0000027	ZNF426-DT	lncRNA	0.57330183	0.15575495	0.28003851

ENSG0000026	RP11-264B17.	lncRNA	0.57324211	0.61715869	0.7409016
ENSG0000028	RP11-114N19	lncRNA	0.57320971	0.29853173	0.44945044
ENSG0000016	NELL1	protein_codir	0.57318167	0.51906896	0.66192736
ENSG0000028	RP11-382A20	lncRNA	0.57315874	0.14757366	0.26919088
ENSG0000017	HPSE	protein_codir	0.57304319	0.18308563	0.31561759
ENSG0000028	RP11-353K11.	lncRNA	0.57274537	0.73694915	NA
ENSG0000026	ZNF587B	protein_codir	0.57263105	0.00339305	0.01490159
ENSG0000012	RTN2	protein_codir	0.57226101	0.00699142	0.02632267
ENSG0000027	RP1-111B22.3	lncRNA	0.57217996	0.65472312	0.77013128
ENSG0000010	ANKRD12	protein_codir	0.57202904	0.00114398	0.00635875
ENSG0000025	ZNF486	protein_codir	0.57193866	0.01464706	0.04683938
ENSG0000018	TRAIP	protein_codir	0.57144016	0.08661795	0.18121405
ENSG0000026	ZNF528-AS1	lncRNA	0.57132066	0.17322168	0.30292909
ENSG0000027	RP11-378J18.	lncRNA	0.57124182	0.0689703	0.15327091
ENSG0000017	CSRNP3	protein_codir	0.57085577	0.15374259	0.27735927
ENSG0000021	CRYGS	protein_codir	0.57080291	0.25276572	0.39895932
ENSG0000016	ARPC5	protein_codir	0.57053176	0.00028876	0.00209006
ENSG0000015	AFF2	protein_codir	0.57020962	0.21738466	0.3576249
ENSG0000011	ARPC3	protein_codir	0.57015689	4.17E-05	0.00041928
ENSG0000019	POTEH	protein_codir	0.57014703	0.81004145	NA
ENSG0000022	AC079145.4	lncRNA	0.57012895	0.41539388	0.56831677
ENSG0000022	AKR1B15	protein_codir	0.56995751	0.63212886	0.75256031
ENSG0000019	RYR1	protein_codir	0.569777	0.01885252	0.05667354
ENSG0000022	TAF1A-AS1	lncRNA	0.56975966	0.20975903	0.3488896
ENSG0000009	PSME1	protein_codir	0.56932117	1.50E-05	0.00018028
ENSG0000027	ITGB8-AS1	lncRNA	0.56924061	0.22721594	0.3693981
ENSG0000024	STX18-AS1	lncRNA	0.56918596	0.04232106	0.10609072
ENSG0000010	GLI3	protein_codir	0.56910407	0.02336716	0.06688093
ENSG0000028	RP11-229P13.	protein_codir	0.56907767	0.69964662	0.8039707
ENSG0000024	CTD-2036P10	lncRNA	0.56904859	0.2058438	0.34388228
ENSG0000028	RP11-172F4.7	lncRNA	0.56900863	0.41553647	0.5684837
ENSG0000015	CFAP70	protein_codir	0.56898502	0.07288382	0.15966544
ENSG0000028	CTD-2506P8.7	lncRNA	0.56889219	0.6470098	0.76415754
ENSG0000015	ALX3	protein_codir	0.56867216	0.62119376	0.74430815
ENSG0000017	CHD2	protein_codir	0.56855534	5.44E-05	0.00052819
ENSG0000014	SLC12A9	protein_codir	0.56846741	0.01374867	0.04453852
ENSG0000027	CTD-2035E11.	lncRNA	0.56827217	0.40782942	0.56085823
ENSG0000018	FFAR4	protein_codir	0.5682231	0.37040013	0.52383264
ENSG0000010	AP1B1	protein_codir	0.56800366	0.00544702	0.02169381
ENSG0000024	PRR7-AS1	lncRNA	0.56796957	0.59104951	0.72035952
ENSG0000027	RP4-583P15.1	protein_codir	0.56769305	0.06963408	0.15443177
ENSG0000015	FAM124A	protein_codir	0.56751441	0.06791504	0.15177711
ENSG0000015	GXYLT1	protein_codir	0.56750447	0.00088057	0.00517583
ENSG0000025	RP11-781A6.1	lncRNA	0.56740489	0.74982587	0.84051097
ENSG0000027	LINC01607	lncRNA	0.56738574	0.69201484	0.79782317
ENSG0000025	POLG2	protein_codir	0.56711601	0.11862425	0.22906516
ENSG0000026	RP13-467H17	lncRNA	0.56709257	0.51128699	0.65505829

ENSG0000015	HS2ST1	protein_codir	0.56697544	0.00088049	0.00517583
ENSG0000022	PSG8-AS1	lncRNA	0.56695897	0.77611946	NA
ENSG0000000	TAC1	protein_codir	0.56694751	0.551517	0.6884304
ENSG0000023	UFL1-AS1	lncRNA	0.56612227	0.62381886	NA
ENSG0000028	C8orf44-SGK3	protein_codir	0.5661116	0.52577028	0.66769839
ENSG0000027	RP11-5C23.2	lncRNA	0.56598819	0.24513642	0.39021774
ENSG0000019	CDC42SE1	protein_codir	0.56595438	9.01E-05	0.00079689
ENSG0000019	ZNF26	protein_codir	0.56595235	0.02026685	0.05991336
ENSG0000015	TEKT5	protein_codir	0.56544265	0.41430878	0.56739432
ENSG0000026	LINC02193	lncRNA	0.56523457	0.03602171	0.09354264
ENSG0000017	GOLGA8A	protein_codir	0.56522367	0.1447182	0.26537755
ENSG0000016	JMJD7-PLA2G	protein_codir	0.56520794	0.09828673	0.19917706
ENSG0000027	RP4-549L20.3	lncRNA	0.56500954	0.33076736	0.48337537
ENSG0000020	DPP9-AS1	lncRNA	0.56476874	0.51621879	0.65938875
ENSG0000022	TMEM72-AS1	lncRNA	0.56470348	0.27560963	0.42370801
ENSG0000014	RPS3	protein_codir	0.56456636	0.0007558	0.00457587
ENSG0000010	ZFAND5	protein_codir	0.56447162	0.01712093	0.05267098
ENSG0000028	CTD-2215E18.	lncRNA	0.56413213	0.38836147	0.54136185
ENSG0000016	CLEC3B	protein_codir	0.56386927	0.23027104	0.3726977
ENSG0000011	TFCP2L1	protein_codir	0.56358965	0.2960399	0.44649277
ENSG0000006	PDIA5	protein_codir	0.5633607	0.00071824	0.00439569
ENSG0000012	TMEM255A	protein_codir	0.56307953	0.25686701	0.40361058
ENSG0000017	GCNT4	protein_codir	0.56286595	0.07949657	0.16986713
ENSG0000017	EGR3	protein_codir	0.56276908	0.21288931	0.35253928
ENSG0000018	EFCAB10	protein_codir	0.56276635	0.77380983	NA
ENSG0000015	SST	protein_codir	0.56265216	0.80112044	0.8765748
ENSG0000022	SLC8A1-AS1	lncRNA	0.56249995	0.48558662	0.63281793
ENSG0000027	SMIM32	protein_codir	0.5623384	0.71148009	NA
ENSG0000028	RP11-61J19.7	lncRNA	0.56226523	0.4928217	0.63926436
ENSG0000027	RP11-380N8.7	lncRNA	0.56212797	0.62660424	0.74824077
ENSG0000016	VAV2	protein_codir	0.5619808	0.00259754	0.01203761
ENSG0000013	CCM2	protein_codir	0.56191771	0.01302571	0.04270776
ENSG0000024	PPP1R35-AS1	lncRNA	0.56189016	0.44949918	0.60034237
ENSG0000016	RPL27A	protein_codir	0.56173116	0.00204116	0.00997465
ENSG0000017	RPS9	protein_codir	0.56171735	0.00181643	0.00911349
ENSG0000017	PPP1CA	protein_codir	0.56165558	0.01023071	0.03545892
ENSG0000024	CORT	protein_codir	0.56151723	0.42203369	0.57401736
ENSG0000015	KCNB1	protein_codir	0.56145996	0.35099906	0.50458512
ENSG0000027	RP11-514P8.1	lncRNA	0.56145909	0.17721531	0.30773358
ENSG0000026	GRAMD1A-AS	lncRNA	0.56131695	0.76728049	NA
ENSG0000017	NRIP3	protein_codir	0.56097548	0.04998193	0.12048237
ENSG0000010	ELMO3	protein_codir	0.56094035	0.16854901	0.29697836
ENSG0000024	AC000120.7	lncRNA	0.56092661	0.26267889	0.41015076
ENSG0000027	TIMMDC1-DT	lncRNA	0.56077892	0.74705172	NA
ENSG0000028	RP1-274L7.5	lncRNA	0.56070929	0.32700766	0.47922489
ENSG0000014	CDH11	protein_codir	0.56058401	0.13207458	0.24822399
ENSG0000019	NBPF4	protein_codir	0.56053841	0.46083679	0.6112018

ENSG0000018	RNASE10	protein_codir	0.56039505	0.37220521	0.52560498
ENSG0000001	POLA2	protein_codir	0.56038741	0.02635696	0.07352582
ENSG0000009	EFNA2	protein_codir	0.5602924	0.54930205	0.68668937
ENSG0000012	STYXL1	protein_codir	0.5602855	0.03810775	0.09768315
ENSG0000022	RP11-472N13	lncRNA	0.55975472	0.32261482	0.4747726
ENSG0000026	CTD-2162K18	lncRNA	0.55969458	0.27304993	0.42092085
ENSG0000010	MEOX2	protein_codir	0.55936499	0.06926232	0.15379254
ENSG0000022	STK4-AS1	lncRNA	0.55915436	0.34396457	0.4971377
ENSG0000018	PLCXD3	protein_codir	0.55909322	0.26524678	0.41283197
ENSG0000023	TMEM254-AS	lncRNA	0.55888588	0.10353437	0.20712874
ENSG0000023	EXOSC10-AS1	lncRNA	0.55841964	0.4669995	0.61659382
ENSG0000018	FAM110C	protein_codir	0.55824379	0.22828339	0.37060995
ENSG0000028	RP11-506P9.1	lncRNA	0.55769157	0.66133211	NA
ENSG0000017	BOK	protein_codir	0.55766582	0.01027925	0.03557806
ENSG0000028	CH17-353B19	lncRNA	0.55745308	0.46148133	0.61166528
ENSG0000025	RP11-603J24.	lncRNA	0.5571497	0.36059727	0.51451062
ENSG0000023	KIAA0040	protein_codir	0.55695592	6.62E-05	0.00062062
ENSG0000019	FAM169A	protein_codir	0.5569325	0.1869984	0.32050238
ENSG0000020	LRRC3C	protein_codir	0.55685033	0.60231638	0.72929632
ENSG0000013	PAN2	protein_codir	0.55660739	0.03950274	0.10054052
ENSG0000019	ANKRD36B	protein_codir	0.55641047	0.0692817	0.15380885
ENSG0000014	TTC13	protein_codir	0.55558427	0.00348261	0.01521749
ENSG0000009	IRAK3	protein_codir	0.5554615	0.03031035	0.08210788
ENSG0000014	FAM171A1	protein_codir	0.55537456	0.00516164	0.02081233
ENSG0000027	RP11-210K20.	lncRNA	0.55534103	0.55184697	0.68867213
ENSG0000017	ARL6IP6	protein_codir	0.5550616	7.64E-05	0.00069579
ENSG0000025	CTD-2516F10.	lncRNA	0.55502967	0.09253963	0.19028771
ENSG0000026	CTC-510F12.6	lncRNA	0.55486126	0.72551331	NA
ENSG0000027	AP000240.9	lncRNA	0.55483326	0.36076349	0.51463111
ENSG0000018	C7orf61	protein_codir	0.55479248	0.22539379	0.36714889
ENSG0000018	OPTC	protein_codir	0.5546759	0.72127028	0.82010192
ENSG0000018	PERM1	protein_codir	0.55466311	0.43618499	0.58742159
ENSG0000008	ME2	protein_codir	0.55447255	0.00919411	0.03260674
ENSG0000023	SNHG15	lncRNA	0.55445276	0.14874036	0.27081139
ENSG0000017	CCDC57	protein_codir	0.55439319	0.03184297	0.08513876
ENSG0000027	RP11-434E6.4	lncRNA	0.5543021	0.4097077	0.5627394
ENSG0000027	CITF22-1A6.3	lncRNA	0.55429537	0.221241	0.36217855
ENSG0000014	SLC43A1	protein_codir	0.55367498	0.00359391	0.01559525
ENSG0000023	SETSIP	protein_codir	0.55348258	0.66772297	0.78052382
ENSG0000023	CDC42-IT1	lncRNA	0.55317902	0.52588314	0.66774974
ENSG0000016	ZNF761	protein_codir	0.55315275	0.00497155	0.02016954
ENSG0000012	PPAT	protein_codir	0.55313222	0.00779747	0.02863248
ENSG0000016	LRRC45	protein_codir	0.55285996	0.01941588	0.05799985
ENSG0000017	TMEM37	protein_codir	0.55239675	0.13447347	0.25141523
ENSG0000013	AKIRIN2	protein_codir	0.55230412	0.00064246	0.00401645
ENSG0000018	YOD1	protein_codir	0.55228128	0.02096895	0.06150833
ENSG0000018	AATK	protein_codir	0.55211777	0.17108352	0.30019229

ENSG0000016	TSC22D4	protein_codir	0.55211454	0.00753639	0.02788039
ENSG0000011	C1orf21	protein_codir	0.5520412	0.10510777	0.20942697
ENSG0000018	NOG	protein_codir	0.55199026	0.17669449	0.30711106
ENSG0000026	CTD-2192J16.	protein_codir	0.55184114	0.41585482	0.56872203
ENSG0000011	ABHD14B	protein_codir	0.55167542	0.00059565	0.00377421
ENSG0000023	FMR1-IT1	lncRNA	0.55153517	0.60731143	0.73328867
ENSG0000014	MTG1	protein_codir	0.55132354	0.04430831	0.10982639
ENSG0000013	PNCK	protein_codir	0.55125224	0.49712245	0.64289904
ENSG0000000	ZMYND10	protein_codir	0.55094638	0.29302128	0.44312569
ENSG0000028	RP11-232L2.3	lncRNA	0.55045277	0.81082022	NA
ENSG0000016	SLC6A5	protein_codir	0.55026336	0.80910815	NA
ENSG0000019	CBWD3	protein_codir	0.54988801	0.02570809	0.07218354
ENSG0000017	RHNO1	protein_codir	0.54983112	0.00031486	0.00224136
ENSG0000010	INPP4B	protein_codir	0.54968421	0.0524864	0.12481054
ENSG0000015	MIS18A	protein_codir	0.54908009	0.00365531	0.01579963
ENSG0000025	ZNF253	protein_codir	0.54894132	0.13886909	0.25736861
ENSG0000012	ZNF211	protein_codir	0.54875196	0.0310089	0.08350062
ENSG0000014	RPL13A	protein_codir	0.54840702	0.00118116	0.00652735
ENSG0000019	MEIG1	protein_codir	0.54818242	0.66774067	0.78052382
ENSG0000013	RP11-798G7.5	lncRNA	0.5479114	0.46999232	0.61914568
ENSG0000015	SHROOM4	protein_codir	0.54789442	0.02192619	0.06365371
ENSG0000013	CTDSPL2	protein_codir	0.5476289	7.04E-05	0.00065213
ENSG0000024	SAP30L-AS1	lncRNA	0.54750624	0.28000501	0.42908002
ENSG0000028	RP11-103P4.1	lncRNA	0.54738163	0.81664096	NA
ENSG0000016	SLC50A1	protein_codir	0.54734908	0.00010281	0.00088956
ENSG0000010	PLAT	protein_codir	0.54723192	0.12794801	0.24226531
ENSG0000018	KCNJ12	protein_codir	0.5470592	0.17330142	0.30302514
ENSG0000012	CHN1	protein_codir	0.54684907	0.01783907	0.05430563
ENSG0000010	TCF20	protein_codir	0.54679445	3.96E-05	0.00040215
ENSG0000000	CYB561	protein_codir	0.54671344	0.00704933	0.02645056
ENSG0000025	EEF1G	protein_codir	0.54663941	0.00033926	0.00238826
ENSG0000026	CTC-479C5.10	lncRNA	0.54648545	0.29017051	0.43987916
ENSG0000028	RP11-350A18	lncRNA	0.54635701	0.77321038	0.85740963
ENSG0000009	MCOLN1	protein_codir	0.54631203	0.00574468	0.02255955
ENSG0000011	POMC	protein_codir	0.54624449	0.13219639	0.24841913
ENSG0000015	NGLY1	protein_codir	0.54622322	0.00558502	0.0220937
ENSG0000025	SUGT1P4-STR	lncRNA	0.54582711	0.03121816	0.08393319
ENSG0000023	PRMT5-AS1	lncRNA	0.54517306	0.77169455	NA
ENSG0000028	RP11-318M2.	lncRNA	0.54517306	0.77225093	NA
ENSG0000027	AC005609.18	lncRNA	0.54517305	0.77373668	NA
ENSG0000023	RP11-384C4.6	lncRNA	0.54517305	0.77437746	NA
ENSG0000028	LLNLR-470E3.	lncRNA	0.54517305	0.77532639	NA
ENSG0000022	Z69666.2	lncRNA	0.54517305	0.77547493	NA
ENSG0000025	RP11-150C16.	lncRNA	0.54517305	0.77644987	NA
ENSG0000022	XX-C2158C6.3	lncRNA	0.54517305	0.77697454	NA
ENSG0000024	RP11-1026M7	lncRNA	0.54517305	0.77720641	NA
ENSG0000027	RP3-412A9.17	lncRNA	0.54517305	0.77735985	NA

ENSG0000023	LINC01497	lncRNA	0.54517304	0.77903723	NA
ENSG0000027	CH507-42P11	protein_codir	0.54517304	0.78091255	NA
ENSG0000026	CTD-2521M24	lncRNA	0.54517297	0.80698626	NA
ENSG0000028	RP11-354P17.	lncRNA	0.54517297	0.80703351	NA
ENSG0000023	RP11-109P14.	lncRNA	0.54517296	0.80749826	NA
ENSG0000025	RP11-1038A1	lncRNA	0.54517296	0.80765428	NA
ENSG0000017	FABP6	protein_codir	0.54517296	0.80776667	NA
ENSG0000028	PACRG-AS1	lncRNA	0.54517296	0.80791926	NA
ENSG0000024	RP11-252I13.1	lncRNA	0.54517296	0.80943226	NA
ENSG0000023	AC073842.19	lncRNA	0.54517295	0.81055349	NA
ENSG0000022	AC000067.1	lncRNA	0.54517295	0.81099647	NA
ENSG0000020	SERPINB5	protein_codir	0.54517295	0.8115962	NA
ENSG0000025	RP11-181B11.	lncRNA	0.54517295	0.81175232	NA
ENSG0000027	PNRC1-DT	lncRNA	0.54517295	0.8118276	NA
ENSG0000028	CTB-66A9.1	lncRNA	0.54517295	0.81209521	NA
ENSG0000012	DNAI1	protein_codir	0.54517295	0.81215819	NA
ENSG0000023	RP11-467I20.(	lncRNA	0.54517295	0.81223467	NA
ENSG0000025	RP11-227D13	lncRNA	0.54517295	0.81247345	NA
ENSG0000018	SOX1	protein_codir	0.54517295	0.81270081	NA
ENSG0000023	SNAP47-AS1	lncRNA	0.54517294	0.81326842	NA
ENSG0000022	C7orf65	lncRNA	0.54517294	0.813513	NA
ENSG0000016	CAPN13	protein_codir	0.54517294	0.81352806	NA
ENSG0000024	RP11-372E1.4	lncRNA	0.54517294	0.81366437	NA
ENSG0000026	CTB-60E11.9	lncRNA	0.54517294	0.81453465	NA
ENSG0000026	RP11-178F10.	lncRNA	0.54517294	0.81476673	NA
ENSG0000010	LHX3	protein_codir	0.54517294	0.81481158	NA
ENSG0000025	CTD-2562J17.	lncRNA	0.54517294	0.81536409	NA
ENSG0000018	OR51B4	protein_codir	0.54517293	0.81692201	NA
ENSG0000017	PLAC1	protein_codir	0.54517293	0.8169712	NA
ENSG0000025	SERPINE3	protein_codir	0.54517293	0.81712521	NA
ENSG0000027	RP5-1116H23	lncRNA	0.54517293	0.8173511	NA
ENSG0000028	RP11-57H12.7	lncRNA	0.54517293	0.81759446	NA
ENSG0000024	LINC01498	lncRNA	0.54517292	0.81815686	NA
ENSG0000016	ZNF280A	protein_codir	0.54517292	0.81821294	NA
ENSG0000025	CTA-797E19.1	lncRNA	0.54517292	0.81823916	NA
ENSG0000019	SLC30A10	protein_codir	0.54517292	0.81829647	NA
ENSG0000019	CYP2F1	protein_codir	0.54517292	0.818502	NA
ENSG0000025	RP11-756P10.	lncRNA	0.54517292	0.81934865	NA
ENSG0000023	AL157902.3	lncRNA	0.54517279	0.84405871	NA
ENSG0000014	BARHL2	protein_codir	0.54517279	0.84469184	NA
ENSG0000028	RP11-411D10	lncRNA	0.54517278	0.8456707	NA
ENSG0000023	LINC01681	lncRNA	0.54517278	0.84606216	NA
ENSG0000025	RDH10-AS1	lncRNA	0.54517278	0.84642539	NA
ENSG0000022	NTM-AS1	lncRNA	0.54517278	0.84652705	NA
ENSG0000024	RP11-538P18.	lncRNA	0.54517278	0.84668662	NA
ENSG0000027	RP1-138B7.6	protein_codir	0.54517277	0.84772141	NA
ENSG0000028	LLNLR-229A1?	lncRNA	0.54517277	0.84777692	NA

ENSG0000026	CTD-2651B20	lncRNA	0.54517277	0.84812228	NA
ENSG0000028	RP3-467D16.3	lncRNA	0.54517276	0.84852977	NA
ENSG0000022	LINC01767	lncRNA	0.54517276	0.84865584	NA
ENSG0000026	RP11-849N15	lncRNA	0.54517276	0.84938491	NA
ENSG0000026	ZFP3-DT	lncRNA	0.54517276	0.84940388	NA
ENSG0000024	RP11-259O2.3	lncRNA	0.54517276	0.84956563	NA
ENSG0000026	RP1-56K13.3	lncRNA	0.54517276	0.84964848	NA
ENSG0000019	RP11-57C13.3	lncRNA	0.54517275	0.84999411	NA
ENSG0000022	RP3-395P12.2	lncRNA	0.54517275	0.8499971	NA
ENSG0000028	RP11-21K20.5	lncRNA	0.54517275	0.85017287	NA
ENSG0000020	LINC02346	lncRNA	0.54517275	0.85050074	NA
ENSG0000022	RP11-165F24.	lncRNA	0.54517275	0.85078438	NA
ENSG0000027	RP11-488L1.1	lncRNA	0.54517275	0.85108695	NA
ENSG0000028	RP11-10L7.4	lncRNA	0.54517274	0.85165394	NA
ENSG0000017	TAC4	protein_codir	0.54517274	0.85204227	NA
ENSG0000027	RP11-355N15	lncRNA	0.54517274	0.8520714	NA
ENSG0000026	RP11-20I23.1	lncRNA	0.54517274	0.85209982	NA
ENSG0000026	CTD-3126B10	lncRNA	0.54517274	0.85209982	NA
ENSG0000026	RP11-538I12.3	lncRNA	0.54517273	0.85286636	NA
ENSG0000023	HCFC1-AS1	lncRNA	0.54517273	0.85305203	NA
ENSG0000022	AC068491.2	lncRNA	0.54517273	0.8533703	NA
ENSG0000015	PLA2G4D	protein_codir	0.54517273	0.85362092	NA
ENSG0000026	RP11-256I23.3	lncRNA	0.54517272	0.85416279	NA
ENSG0000025	RP11-973D8.4	lncRNA	0.54517272	0.85418738	NA
ENSG0000022	ETV7-AS1	lncRNA	0.54517272	0.85485342	NA
ENSG0000025	RP11-614O9.1	lncRNA	0.54517271	0.85514685	NA
ENSG0000026	CTB-33G10.6	lncRNA	0.54517271	0.85545433	NA
ENSG0000022	RP5-1119A7.1	lncRNA	0.54517271	0.8560706	NA
ENSG0000023	RP11-313D6.3	lncRNA	0.54517269	0.85870125	NA
ENSG0000025	SLC2A9-AS1	lncRNA	0.5451726	0.86730681	NA
ENSG0000024	RP11-18D7.2	lncRNA	0.5451726	0.86730681	NA
ENSG0000028	RP11-680F23.	lncRNA	0.5451726	0.86730681	NA
ENSG0000018	ATP13A5	protein_codir	0.5451726	0.86730681	NA
ENSG0000023	RP11-69L16.4	lncRNA	0.5451726	0.86730681	NA
ENSG0000028	RP1-236J16.2	lncRNA	0.5451726	0.86730681	NA
ENSG0000025	RP11-730G20	lncRNA	0.5451726	0.86730681	NA
ENSG0000028	RP11-90J7.3	lncRNA	0.5451726	0.86730681	NA
ENSG0000028	RP11-15D14.4	lncRNA	0.5451726	0.86730681	NA
ENSG0000024	RP11-598F7.4	lncRNA	0.5451726	0.86730681	NA
ENSG0000025	RP5-875H18.4	lncRNA	0.5451726	0.86730681	NA
ENSG0000023	TMEM26-AS1	lncRNA	0.54496899	0.4672695	0.61680289
ENSG0000010	UNC5B	protein_codir	0.54496587	0.12723438	0.24131771
ENSG0000023	EMC1-AS1	lncRNA	0.54463822	0.16804286	0.2964389
ENSG0000022	LINC01341	lncRNA	0.54460409	0.30217352	0.45349014
ENSG0000012	ALG5	protein_codir	0.54452272	0.00015028	0.00122805
ENSG0000026	RPL17	protein_codir	0.54443165	0.00165796	0.00852437
ENSG0000018	DUSP28	protein_codir	0.54398924	0.00327026	0.01448425

ENSG0000026	AC005546.2	lncRNA	0.54398153	0.71709282	0.81715091
ENSG0000026	CTD-3193O13	lncRNA	0.54389488	0.78281111	NA
ENSG0000024	FOXP1-IT1	lncRNA	0.54373564	0.33685154	0.48992921
ENSG0000016	ARHGAP42	protein_codir	0.54370528	0.01650349	0.05125965
ENSG0000016	AMDHD2	protein_codir	0.54369415	0.00262914	0.01215752
ENSG0000027	C8orf89	protein_codir	0.54334839	0.57673147	0.70910449
ENSG0000022	C2orf92	protein_codir	0.54317523	0.13346709	0.25005841
ENSG0000013	RFC3	protein_codir	0.54313332	0.0089794	0.03199728
ENSG0000013	RAPGEF5	protein_codir	0.54313208	0.02853274	0.07837636
ENSG0000022	RP11-197P3.5	lncRNA	0.54296119	0.52750129	0.66906621
ENSG0000017	TRIM73	protein_codir	0.54280976	0.18871672	0.32262662
ENSG0000022	RP11-57H14.3	lncRNA	0.54267742	0.78227059	NA
ENSG0000023	BZW1-AS1	lncRNA	0.54266305	0.66593624	0.77927283
ENSG0000018	RP1L1	protein_codir	0.54244535	0.1860279	0.31931409
ENSG0000013	GDF1	protein_codir	0.54236644	0.296559	0.44706613
ENSG0000025	RP11-680F20.	lncRNA	0.54235301	0.81782392	0.88807929
ENSG0000013	SH3BP5	protein_codir	0.54221579	0.00521478	0.02097456
ENSG0000013	RC3H1	protein_codir	0.54206489	6.83E-07	1.32E-05
ENSG0000016	PXK	protein_codir	0.54204267	0.00425028	0.01785983
ENSG0000026	CTD-2619J13.	lncRNA	0.54202718	0.16078162	0.28667028
ENSG0000013	SLC39A11	protein_codir	0.54191633	0.01483938	0.04733384
ENSG0000014	ADAMTS17	protein_codir	0.54191338	0.06530195	0.14754908
ENSG0000027	DCTN6-DT	lncRNA	0.54191187	0.17417033	0.30417394
ENSG0000028	CTB-51E13.2	lncRNA	0.54176606	0.65847073	0.77312369
ENSG0000025	DENND11	protein_codir	0.54154682	0.00375483	0.01615396
ENSG0000011	GMNN	protein_codir	0.54151637	0.03372404	0.08905804
ENSG0000008	FXYD5	protein_codir	0.5414712	0.01125144	0.03817748
ENSG0000017	CTC1	protein_codir	0.54139086	0.03296442	0.08748701
ENSG0000019	DNM3	protein_codir	0.54136105	0.02563316	0.07200853
ENSG0000022	RNF207-AS1	lncRNA	0.54128901	0.74594268	NA
ENSG0000016	JAK1	protein_codir	0.54127987	0.00020529	0.00157763
ENSG0000023	AL512380.1	lncRNA	0.54127712	0.74587586	NA
ENSG0000016	TM4SF20	protein_codir	0.54107912	0.7447673	NA
ENSG0000014	PCYOX1L	protein_codir	0.54091361	0.0362585	0.0940237
ENSG0000028	DNAJB5-DT	lncRNA	0.54061882	0.65072002	0.76682682
ENSG0000022	TEX41	lncRNA	0.54056953	0.08558481	0.17957433
ENSG0000027	RP11-359K18.	lncRNA	0.54011331	0.44689653	0.59779553
ENSG0000014	RPS3A	protein_codir	0.53970461	0.00086273	0.00508833
ENSG0000010	NFKB1	protein_codir	0.53966674	0.00289675	0.01311826
ENSG0000010	LACTB	protein_codir	0.53961012	0.00305019	0.01368523
ENSG0000017	ZDHHC24	protein_codir	0.53950509	0.00072951	0.00444891
ENSG0000028	RP3-466P17.4	lncRNA	0.53925516	0.53519925	0.67557257
ENSG0000025	C17orf49	protein_codir	0.53912871	0.01312158	0.04295579
ENSG0000027	RP11-342I1.2	lncRNA	0.53862842	0.37870173	0.53217236
ENSG0000010	CDC25B	protein_codir	0.53838523	0.02473769	0.07002717
ENSG0000013	ZSWIM6	protein_codir	0.53818257	0.01869107	0.05630357
ENSG0000016	ACSF2	protein_codir	0.53816489	0.03724544	0.09603426

ENSG0000011	AKAP7	protein_codir	0.53800799	0.00050133	0.00327811
ENSG0000011	CFHR3	protein_codir	0.53798446	0.34412513	0.49723966
ENSG0000015	C2orf50	protein_codir	0.53797193	0.33635533	0.48934076
ENSG0000010	SLC38A7	protein_codir	0.53796569	0.02301801	0.06612142
ENSG0000013	DYSF	protein_codir	0.53794268	0.04324023	0.10780784
ENSG0000016	SNHG16	lncRNA	0.53769837	0.02108724	0.06177002
ENSG0000012	PLAGL2	protein_codir	0.53769182	0.00972258	0.03403391
ENSG0000016	DEDD2	protein_codir	0.53765468	0.00110865	0.00620737
ENSG0000017	NPTX1	protein_codir	0.53762554	0.40052575	0.5537906
ENSG0000010	LILRB5	protein_codir	0.53761536	0.14180874	0.26134586
ENSG0000010	VTN	protein_codir	0.53749033	0.08266216	0.17470615
ENSG0000014	GCNA	protein_codir	0.53734379	0.17769192	0.30841769
ENSG0000018	NPW	protein_codir	0.53727234	0.48534523	0.63267034
ENSG0000023	NPAS2-AS1	lncRNA	0.53721605	0.74975419	NA
ENSG0000028	RP11-592P9.4	lncRNA	0.5371907	0.66745288	0.78031951
ENSG0000015	DKK2	protein_codir	0.5368628	0.28673878	0.43637177
ENSG0000025	LINC02688	lncRNA	0.53682884	0.32612465	0.47838775
ENSG0000022	LINC00858	lncRNA	0.53676708	0.74733877	NA
ENSG0000019	VEPH1	protein_codir	0.53649557	0.49987371	0.64519426
ENSG0000028	RP11-124O10	lncRNA	0.53645492	0.72860264	NA
ENSG0000022	LINC00484	lncRNA	0.53611454	0.47541477	0.62367402
ENSG0000019	GARS1-DT	lncRNA	0.53574363	0.15700814	0.28177809
ENSG0000028	AC005753.3	lncRNA	0.53554162	0.26197185	0.40944219
ENSG0000026	C17orf113	protein_codir	0.53525509	0.02972928	0.08095461
ENSG0000010	SETX	protein_codir	0.53524165	0.00521597	0.02097629
ENSG0000028	CTA-392E5.2	lncRNA	0.53510712	0.76271584	0.85019823
ENSG0000026	NUDT19-DT	lncRNA	0.5345818	0.16383788	0.29074802
ENSG0000004	WDR37	protein_codir	0.53449414	0.00730586	0.0272098
ENSG0000026	RP11-775C24.	lncRNA	0.53443927	0.58924238	0.71910899
ENSG0000012	RNF170	protein_codir	0.53394492	0.00014554	0.00119568
ENSG0000019	ZNF334	protein_codir	0.53358723	0.12328982	0.23586137
ENSG0000016	AGPAT3	protein_codir	0.5335509	0.00245788	0.01152999
ENSG0000025	LINC02253	lncRNA	0.53353636	0.78521708	NA
ENSG0000016	PKN3	protein_codir	0.5334962	0.03180721	0.08510271
ENSG0000010	DYRK1B	protein_codir	0.5334146	0.00193522	0.00956358
ENSG0000012	MSX2	protein_codir	0.53332647	0.23990483	0.38433489
ENSG0000027	CCL23	protein_codir	0.53326209	0.2420042	0.38683697
ENSG0000014	CCDC15	protein_codir	0.53317199	0.04383967	0.10888322
ENSG0000010	RPA3	protein_codir	0.53314921	0.00560711	0.02216204
ENSG0000022	RP5-981O7.2	lncRNA	0.53307112	0.60323101	0.73001562
ENSG0000011	CLK4	protein_codir	0.53235882	0.03836492	0.09818747
ENSG0000017	MTM1	protein_codir	0.53231175	0.00164341	0.00846847
ENSG0000011	PCSK4	protein_codir	0.53195407	0.15117803	0.27381387
ENSG0000016	KIFC2	protein_codir	0.53131251	0.12525629	0.23864101
ENSG0000021	AC009403.2	lncRNA	0.53129956	0.12740594	0.24155354
ENSG0000024	LINC02202	lncRNA	0.53125881	0.11767717	0.22767185
ENSG0000017	TRAPPC3L	protein_codir	0.53121267	0.3307345	0.48335293

ENSG0000012	NMI	protein_codir	0.53101462	0.00614129	0.02377996
ENSG0000014	ARHGAP39	protein_codir	0.53099126	0.0596831	0.13741641
ENSG0000026	CAMTA2-AS1	lncRNA	0.53091891	0.70451901	NA
ENSG0000022	TINCR	protein_codir	0.53079307	0.10785116	0.21350695
ENSG0000025	RP11-467L24.	lncRNA	0.53071792	0.75357555	NA
ENSG0000022	RPL41	protein_codir	0.53058038	0.00147848	0.00780049
ENSG0000010	DDHD1	protein_codir	0.53052532	0.00211054	0.01024484
ENSG0000013	SMPD2	protein_codir	0.53043209	0.00574525	0.02255955
ENSG0000016	C9orf116	protein_codir	0.53041086	0.0143446	0.04603199
ENSG0000016	PCF11	protein_codir	0.53030405	0.00032143	0.00227923
ENSG0000011	MLPH	protein_codir	0.5300294	0.27185328	0.4198025
ENSG0000012	EPB41L4A	protein_codir	0.52993956	0.02350539	0.06717215
ENSG0000018	RPS17	protein_codir	0.52993894	0.00249807	0.01169038
ENSG0000011	ABCD4	protein_codir	0.52984505	0.0053561	0.02140166
ENSG0000025	RP11-158M2.	lncRNA	0.52983607	0.18091683	0.31263242
ENSG0000012	TSHZ3	protein_codir	0.52976668	0.06843077	0.15246092
ENSG0000010	AP3B2	protein_codir	0.52948905	0.42529055	0.57721511
ENSG0000028	CTB-54I1.2	lncRNA	0.52947328	0.30398336	0.45526626
ENSG0000020	CHKB-DT	lncRNA	0.52881897	0.19139235	0.32597007
ENSG0000023	RP11-350J20.	lncRNA	0.52879355	0.40092764	0.55406868
ENSG0000026	CTD-2659N19	lncRNA	0.52860798	0.47469031	0.622907
ENSG0000025	BRWD1-AS2	lncRNA	0.52844607	0.35877201	0.51242542
ENSG0000003	ARHGAP31	protein_codir	0.52842174	0.00223279	0.01071585
ENSG0000027	RP11-108K14.	lncRNA	0.52841958	0.60978345	0.73525318
ENSG0000016	NDST2	protein_codir	0.52823886	5.23E-05	0.00051079
ENSG0000027	CTD-2081C10	lncRNA	0.52815783	0.24029007	0.38474271
ENSG0000017	RNF227	protein_codir	0.52814448	0.02249243	0.06496298
ENSG0000015	HEATR3	protein_codir	0.5280779	0.00937197	0.03310997
ENSG0000028	TPTEP2-CSNK	protein_codir	0.52787234	0.34041026	0.49343783
ENSG0000015	CCDC122	protein_codir	0.52784963	0.03984106	0.10119645
ENSG0000015	ELK4	protein_codir	0.52770323	0.000437	0.00292749
ENSG0000014	CHEK1	protein_codir	0.52761762	0.08243932	0.17443464
ENSG0000025	ZNF550	protein_codir	0.52737075	0.01129272	0.03828933
ENSG0000023	RP1-140A9.1	lncRNA	0.52716745	0.59858935	0.72655207
ENSG0000013	MATN2	protein_codir	0.52682864	0.24496809	0.39001721
ENSG0000012	GGA3	protein_codir	0.52663206	0.00718216	0.02685048
ENSG0000008	ANKRD24	protein_codir	0.52653312	0.14036334	0.25955897
ENSG0000023	HIPK1-AS1	lncRNA	0.52652253	0.35977149	0.51356084
ENSG0000026	RMRP	lncRNA	0.52650777	0.7500441	0.84065325
ENSG0000016	C21orf58	protein_codir	0.52639235	0.18454262	0.31757428
ENSG0000014	ANK2	protein_codir	0.52618469	0.15985854	0.28555767
ENSG0000016	MSS51	protein_codir	0.5261138	0.23911207	0.38338995
ENSG0000013	ZSWIM4	protein_codir	0.52599591	0.0173276	0.05312092
ENSG0000026	RP11-697N18	lncRNA	0.52573463	0.50438952	0.64908161
ENSG0000020	MFSD2B	protein_codir	0.52563523	0.49435406	0.64047535
ENSG0000013	ARHGAP20	protein_codir	0.52555801	0.15775741	0.28281039
ENSG0000013	NASP	protein_codir	0.52554013	0.00242981	0.0114388

ENSG0000023	DGUOK-AS1	lncRNA	0.52552221	0.47151273	0.62032875
ENSG0000012	RNF113A	protein_codir	0.52527283	0.00252409	0.01176629
ENSG0000022	LINC02048	lncRNA	0.52479331	0.79737626	NA
ENSG0000016	NYAP1	protein_codir	0.52467377	0.16244006	0.28880556
ENSG0000017	ALG10B	protein_codir	0.52454358	0.00114989	0.00638904
ENSG0000026	RP11-249C24.	lncRNA	0.52440792	0.78365637	NA
ENSG0000015	LGI4	protein_codir	0.52379036	0.17627826	0.30663847
ENSG0000017	LPL	protein_codir	0.52363296	0.33209668	0.48493314
ENSG0000025	RP11-111M22	lncRNA	0.52293122	0.04939146	0.11942504
ENSG0000010	FGF14	protein_codir	0.52291335	0.18064589	0.31223025
ENSG0000008	ZNF506	protein_codir	0.52247972	0.00792194	0.02897622
ENSG0000011	GPX7	protein_codir	0.52212336	0.02292534	0.06591006
ENSG0000011	SIAE	protein_codir	0.52197066	0.00842999	0.03039241
ENSG0000014	KSR1	protein_codir	0.5219049	0.00551291	0.02187425
ENSG0000000	ZNF195	protein_codir	0.52176097	0.05094515	0.12217634
ENSG0000010	HOXA6	protein_codir	0.52140083	0.31325513	0.46499203
ENSG0000025	RP3-403A15.5	protein_codir	0.52134333	0.06122117	0.1400935
ENSG0000015	GDPD1	protein_codir	0.52129844	0.01497465	0.04768272
ENSG0000022	SNHG31	lncRNA	0.52096802	0.26533801	0.41292742
ENSG0000012	LRRC39	protein_codir	0.52090683	0.26995637	0.41778421
ENSG0000014	CDKN2B	protein_codir	0.52069111	0.13064848	0.24606281
ENSG0000020	RAET1G	protein_codir	0.52035254	0.2953831	0.44571421
ENSG0000021	CTD-3193O13	lncRNA	0.52021492	0.45012222	0.6008591
ENSG0000017	TMEM70	protein_codir	0.5200816	0.0406131	0.10267587
ENSG0000014	KIAA0319L	protein_codir	0.5199499	0.00019043	0.00148331
ENSG0000019	DCAF12	protein_codir	0.51991761	0.0016734	0.00858455
ENSG0000010	NIN	protein_codir	0.5198335	0.01108474	0.03775525
ENSG0000013	ATP1A4	protein_codir	0.51975857	0.36124053	0.5151463
ENSG0000013	PNPLA7	protein_codir	0.51961836	0.12295026	0.23538408
ENSG0000013	TRAF3	protein_codir	0.51952191	0.01135696	0.03847409
ENSG0000009	CAPN3	protein_codir	0.51925116	0.2154867	0.35565451
ENSG0000027	RP11-390K5.6	lncRNA	0.51924205	0.35369702	0.50732819
ENSG0000027	RP11-434P11.	lncRNA	0.51888029	0.47157306	0.62037856
ENSG0000010	N4BP1	protein_codir	0.51866639	0.00105776	0.00598904
ENSG0000024	AC007566.10	lncRNA	0.51846507	0.04940674	0.11944106
ENSG0000013	PPP3CA	protein_codir	0.51843221	0.00127734	0.00695176
ENSG0000027	AJ271736.10	lncRNA	0.51801772	0.66201493	0.77598357
ENSG0000028	RP1-240K6.5	lncRNA	0.51783295	0.51396621	0.65727136
ENSG0000016	SCN3B	protein_codir	0.51777396	0.27372783	0.42177755
ENSG0000025	LINC02714	lncRNA	0.51756774	0.55203345	0.6887949
ENSG0000022	RP11-523O18	lncRNA	0.51748995	0.72572836	NA
ENSG0000023	DPP10-AS1	lncRNA	0.51730802	0.78648118	NA
ENSG0000014	NIPA2	protein_codir	0.51724745	0.00028763	0.0020846
ENSG0000018	RCC1	protein_codir	0.51717012	0.07898013	0.16909254
ENSG0000019	TRPV1	protein_codir	0.5171604	0.03818041	0.09780588
ENSG0000012	ISLR	protein_codir	0.5169273	0.10675252	0.21191093
ENSG0000017	LRRC63	protein_codir	0.51688077	0.40024496	0.55352103

ENSG0000016	C5orf63	protein_codir	0.51684993	0.16819039	0.29658549
ENSG0000011	TFEB	protein_codir	0.51657698	0.03714702	0.09582522
ENSG0000013	GGACT	protein_codir	0.51631275	0.06112508	0.13996377
ENSG0000024	AC092620.2	lncRNA	0.51577286	0.49058784	0.63734837
ENSG0000019	ERO1A	protein_codir	0.51575097	1.39E-05	0.00016933
ENSG0000024	TMEM225B	protein_codir	0.51571733	0.15126826	0.27395117
ENSG0000021	TTC34	protein_codir	0.51571366	0.29365744	0.4439176
ENSG0000011	RPS15	protein_codir	0.51566264	0.00566344	0.02232033
ENSG0000007	DNM2	protein_codir	0.51560917	0.00010867	0.0009321
ENSG0000027	AC005609.16	lncRNA	0.51526346	0.52303045	0.66526467
ENSG0000027	RP11-158H5.8	lncRNA	0.51511041	0.50113475	0.6461091
ENSG0000017	CDCA4	protein_codir	0.51510655	0.00551008	0.02186613
ENSG0000017	MACROD2	protein_codir	0.51478624	0.40697837	0.55996526
ENSG0000015	ZNF256	protein_codir	0.51465858	0.01268865	0.04182606
ENSG0000028	RP11-564C4.1	lncRNA	0.51456123	0.79638831	0.87351358
ENSG0000016	UVSSA	protein_codir	0.51455741	0.07705021	0.16605234
ENSG0000014	EAF1	protein_codir	0.51431439	0.07134456	0.15725296
ENSG0000027	HNRNPD-DT	lncRNA	0.51426495	0.21350305	0.35325784
ENSG0000027	RP11-290D2.6	lncRNA	0.51388173	0.02700625	0.07494349
ENSG0000013	AFG1L	protein_codir	0.51365801	0.0027122	0.01246648
ENSG0000026	THOC1-DT	lncRNA	0.51353501	0.51066053	0.65458968
ENSG0000026	RP11-186B7.4	lncRNA	0.51317145	0.22219926	0.3633596
ENSG0000013	VHL	protein_codir	0.51310623	0.0030997	0.01386231
ENSG0000017	OR5B2	protein_codir	0.51285818	0.74077825	0.83446696
ENSG0000014	TOB1	protein_codir	0.51260935	0.02164706	0.06301559
ENSG0000028	RP11-120J1.2	lncRNA	0.51259433	0.78685531	NA
ENSG0000016	DDX21	protein_codir	0.51255173	0.07643342	0.16516124
ENSG0000025	CTC-455F18.1	lncRNA	0.51248173	0.65297221	0.7687265
ENSG0000016	HNRNPF	protein_codir	0.51242266	0.00025105	0.00186249
ENSG0000010	FNBP4	protein_codir	0.51231847	0.01779734	0.05422543
ENSG0000011	TMCO6	protein_codir	0.51194657	0.00726933	0.02709931
ENSG0000025	CTD-2325P2.3	lncRNA	0.5119318	0.85550376	NA
ENSG0000027	RP11-65L3.4	lncRNA	0.51179503	0.14700972	0.26849226
ENSG0000018	NEMP2	protein_codir	0.51174289	0.00188579	0.00937222
ENSG0000010	FAM83E	protein_codir	0.51157289	0.42370703	0.57569797
ENSG0000026	HOXB7	protein_codir	0.51154111	0.09285571	0.190824
ENSG0000022	KCNQ1-AS1	lncRNA	0.51124249	0.78908686	NA
ENSG0000027	RP11-531F16.	lncRNA	0.51119437	0.62891655	0.74993201
ENSG0000011	NME5	protein_codir	0.51116193	0.14704944	0.26854705
ENSG0000018	SECISBP2	protein_codir	0.51110916	0.00513043	0.02068952
ENSG0000010	RPL18A	protein_codir	0.51107351	0.00634261	0.02440891
ENSG0000026	SPACA6P-AS	lncRNA	0.51101045	0.54794288	0.68561124
ENSG0000027	RP11-333J10.	lncRNA	0.51093474	0.40813902	0.56115765
ENSG0000013	AKAP12	protein_codir	0.51073348	0.2522815	0.39842273
ENSG0000008	RPLP0	protein_codir	0.51063382	0.00379404	0.01630487
ENSG0000017	SLC29A2	protein_codir	0.51049602	0.1327864	0.24915492
ENSG0000021	SLX1A-SULT1A	lncRNA	0.51043246	0.17219411	0.30165219

ENSG0000016	TENT2	protein_codir	0.51043134	0.00012883	0.00107923
ENSG0000018	ZNF548	protein_codir	0.51029914	0.00047512	0.0031379
ENSG0000018	MPPED1	protein_codir	0.51021595	0.7869813	NA
ENSG0000026	SNHG8	lncRNA	0.50987617	0.03878058	0.09907684
ENSG0000018	DHTKD1	protein_codir	0.50973676	0.00200823	0.0098626
ENSG0000016	H1-4	protein_codir	0.50965503	0.08981423	0.18590145
ENSG0000010	PABPC1L	protein_codir	0.50960652	0.2510655	0.39713815
ENSG0000023	TMEM147-AS	lncRNA	0.50948678	0.1209418	0.23248974
ENSG0000016	ATXN2L	protein_codir	0.5093809	0.00097746	0.005628
ENSG0000025	GS1-57L11.1	lncRNA	0.5093447	0.45840053	0.60879081
ENSG0000028	RP5-899B16.3	lncRNA	0.50931766	0.37959723	0.53294581
ENSG0000024	TMEM161B-A	lncRNA	0.50931361	0.11459857	0.22316703
ENSG0000017	KCTD12	protein_codir	0.50906457	0.05040062	0.12128115
ENSG0000018	TMEM106A	protein_codir	0.50902335	0.01751747	0.05356864
ENSG0000014	P4HA3	protein_codir	0.50896506	0.21736066	0.3576249
ENSG0000018	TIGD2	protein_codir	0.5087348	0.08355191	0.17612025
ENSG0000023	INTS6-AS1	lncRNA	0.5086037	0.19114089	0.32570248
ENSG0000018	LIMK2	protein_codir	0.50853001	0.0018308	0.00916231
ENSG0000019	HELZ	protein_codir	0.50848706	0.00363779	0.01574855
ENSG0000014	NTN5	protein_codir	0.50829797	0.2213811	0.36234664
ENSG0000026	RP11-403A21	lncRNA	0.5081529	0.48191784	0.62949022
ENSG0000017	SUZ12	protein_codir	0.50809719	9.64E-05	0.00084189
ENSG0000027	RP1-202O8.3	lncRNA	0.5078592	0.33797223	0.49076247
ENSG0000011	OGFRL1	protein_codir	0.50742416	0.02066961	0.06084351
ENSG0000020	RACK1	protein_codir	0.50738512	0.0003041	0.00217487
ENSG0000024	RP11-190P13.	lncRNA	0.50715112	0.2854385	0.43494491
ENSG0000026	RP3-522J7.6	lncRNA	0.50710251	0.60979162	0.73525318
ENSG0000026	ZNF850	protein_codir	0.50708653	0.01739798	0.05327409
ENSG0000017	GRINA	protein_codir	0.50665925	0.00017815	0.00140828
ENSG0000016	BNC1	protein_codir	0.50648764	0.52694732	0.66867063
ENSG0000018	TMEM255B	protein_codir	0.5064741	0.06054456	0.13890804
ENSG0000000	PLXND1	protein_codir	0.5064392	0.02981115	0.08107575
ENSG0000023	HM13-IT1	lncRNA	0.50639666	0.44574771	0.59674618
ENSG0000018	RNFT1	protein_codir	0.50639454	0.02045894	0.06031981
ENSG0000010	TOP3B	protein_codir	0.50637163	0.10132483	0.20378591
ENSG0000015	MFSD6	protein_codir	0.50614833	0.00473914	0.01945239
ENSG0000017	TANC2	protein_codir	0.50584012	0.06837407	0.15239296
ENSG0000016	TUBA3FP	lncRNA	0.50543698	0.19941491	0.33592582
ENSG0000019	HCAR1	protein_codir	0.50537836	0.4581997	0.60867043
ENSG0000026	RP11-678G14	lncRNA	0.50517212	0.4390389	0.59011409
ENSG0000023	ASMTL-AS1	lncRNA	0.50496114	0.29240282	0.44245691
ENSG0000007	CHFR	protein_codir	0.50487517	0.00729619	0.02718111
ENSG0000022	TFAP2A-AS1	lncRNA	0.50467824	0.61053243	0.73587737
ENSG0000028	RP11-810C16.	lncRNA	0.50462459	0.57604273	0.70857309
ENSG0000004	OFD1	protein_codir	0.50461435	0.02990659	0.0812612
ENSG0000028	RP1-168L15.7	lncRNA	0.5044903	0.07267872	0.15937374
ENSG0000011	PLA2G4A	protein_codir	0.50447826	0.12008031	0.2311021

ENSG0000027	RP11-419I17.:	lncRNA	0.50425683	0.46584977	0.61548771
ENSG0000027	TBC1D3I	protein_codir	0.50423238	0.58624543	0.71665481
ENSG0000026	RP11-295M3.	lncRNA	0.50408491	0.79937304	NA
ENSG0000016	KRT24	protein_codir	0.50390102	0.71465154	0.81494489
ENSG0000027	RP1-198K11.5	lncRNA	0.50353348	0.11123197	0.21852386
ENSG0000027	RP11-351C21.	lncRNA	0.50301433	0.54042101	0.67946774
ENSG0000018	UTY	protein_codir	0.50300378	0.35376462	0.50739881
ENSG0000028	RP11-310J24.	lncRNA	0.50294998	0.61853726	0.74216799
ENSG0000012	TMEM175	protein_codir	0.50285257	0.01625662	0.05071814
ENSG0000021	NEURL1B	protein_codir	0.50280284	0.09854445	0.19958918
ENSG0000013	SYTL2	protein_codir	0.50280042	0.19058511	0.32523634
ENSG0000006	ZFAT	protein_codir	0.50227783	0.02094679	0.06145428
ENSG0000015	CD109	protein_codir	0.50216527	0.18796356	0.32179716
ENSG0000016	SCN9A	protein_codir	0.50213168	0.08563574	0.17962175
ENSG0000025	POC1B-GALN	protein_codir	0.50211219	0.43062912	0.58209642
ENSG0000026	LINC01915	lncRNA	0.502078	0.20595802	0.34398707
ENSG0000028	RP11-310I9.2	lncRNA	0.50204818	0.54791655	0.68560934
ENSG0000028	ADORA3	protein_codir	0.5019204	0.396753	0.54953702
ENSG0000023	EIF2AK3-DT	lncRNA	0.50188866	0.2552758	0.40195642
ENSG0000007	FAP	protein_codir	0.50161272	0.13551366	0.25261731
ENSG0000018	TAL2	protein_codir	0.50136967	0.51083103	0.65471708
ENSG0000028	RP11-540O11	lncRNA	0.50103472	0.58001236	0.71180758
ENSG0000011	RMDN2	protein_codir	0.50100063	0.00837297	0.03025256
ENSG0000026	CTD-2537I9.1	lncRNA	0.50095859	0.4799652	0.62771148
ENSG0000015	PABIR3	protein_codir	0.50089394	0.00573797	0.02254379
ENSG0000026	AC002310.11	protein_codir	0.50085849	0.57834585	0.71052008
ENSG0000014	HMGA2	protein_codir	0.50071025	0.36945781	0.5229552
ENSG0000010	SLC17A7	protein_codir	0.50068204	0.19108811	0.32565273
ENSG0000013	FMO5	protein_codir	0.50047993	0.01218236	0.0405736
ENSG0000016	RAB8A	protein_codir	0.50044252	0.00022952	0.00172608
ENSG0000025	HOXC-AS3	lncRNA	0.5003846	0.6028006	0.72970487
ENSG0000017	ZNF519	protein_codir	0.50033953	0.10936659	0.21578006
ENSG0000010	CRYM	protein_codir	0.50006492	0.36500951	0.51889252
ENSG0000024	LINC02709	lncRNA	0.49955437	0.28240545	0.43196758
ENSG0000010	FOXRED2	protein_codir	0.49940401	0.11446655	0.22294381
ENSG0000027	RP13-554M15	lncRNA	0.49903018	0.72337169	0.82154484
ENSG0000012	TUBGCP6	protein_codir	0.49869503	0.03767403	0.09681338
ENSG0000015	DAB2	protein_codir	0.49866782	0.0642321	0.14564409
ENSG0000017	P2RY6	protein_codir	0.49866715	0.11094841	0.21812194
ENSG0000013	TMEM62	protein_codir	0.49848899	0.0001177	0.00099749
ENSG0000023	C10orf143	protein_codir	0.49844314	0.01175344	0.03950233
ENSG0000022	CERS1	protein_codir	0.49811604	0.19706471	0.33310449
ENSG0000013	GTF2B	protein_codir	0.49811243	0.00050952	0.00331832
ENSG0000020	H2BC18	protein_codir	0.49781077	0.26497536	0.41252576
ENSG0000010	RSPH6A	protein_codir	0.49766602	0.71779398	0.81768002
ENSG0000026	CTB-176F20.3	lncRNA	0.49760575	0.73073035	0.82674407
ENSG0000014	EFNA3	protein_codir	0.4974467	0.08771947	0.18269019

ENSG0000017	MCRIP2	protein_codir	0.49739635	0.02036246	0.06011878
ENSG0000028	ASDURF	protein_codir	0.4973876	0.37701801	0.53063787
ENSG0000014	RPL32	protein_codir	0.49706519	0.00332488	0.01467217
ENSG0000024	HAS2-AS1	lncRNA	0.49656643	0.17148425	0.30074258
ENSG0000014	SLC5A2	protein_codir	0.49642857	0.33628389	0.48927938
ENSG0000014	ULK3	protein_codir	0.49602791	0.04324649	0.1078137
ENSG0000028	RP11-66D17.1	lncRNA	0.49591514	0.76571962	NA
ENSG0000026	GOLGA8T	protein_codir	0.49579757	0.43028813	0.58177786
ENSG0000011	C5orf15	protein_codir	0.49577551	0.00037292	0.00258084
ENSG0000016	AIRE	protein_codir	0.49564529	0.46790628	0.61728943
ENSG0000028	RP11-392A14	lncRNA	0.49562589	0.31235531	0.46415525
ENSG0000018	KREMEN1	protein_codir	0.49562052	0.0346388	0.09085695
ENSG0000013	PRPF38B	protein_codir	0.49554181	0.01695682	0.05231764
ENSG0000014	HMCN2	protein_codir	0.49528527	NA	NA
ENSG0000026	RP11-47L3.1	lncRNA	0.49490898	0.12960386	0.24458728
ENSG0000016	C12orf50	protein_codir	0.49490502	0.5528879	0.68937374
ENSG0000013	KCNMB4	protein_codir	0.49485076	0.06352964	0.14438318
ENSG0000009	IGFALS	protein_codir	0.49467101	0.31997704	0.47210288
ENSG0000022	LINC00242	lncRNA	0.49460104	0.19977288	0.33638524
ENSG0000014	TMEM140	protein_codir	0.4945365	0.07437316	0.16195102
ENSG0000020	STUM	protein_codir	0.49447347	0.23998649	0.38441263
ENSG0000021	AC074286.1	lncRNA	0.49400771	0.09095739	0.18766288
ENSG0000012	NLN	protein_codir	0.49400637	0.03115486	0.08383613
ENSG0000010	GIT1	protein_codir	0.49390616	3.31E-06	5.04E-05
ENSG0000016	CLIC3	protein_codir	0.49380252	0.21277292	0.35236767
ENSG0000028	CTD-2192J16.	protein_codir	0.49367247	0.10684563	0.21202675
ENSG0000020	RPA4	protein_codir	0.49349861	0.48819175	0.63531172
ENSG0000003	BAK1	protein_codir	0.49344603	0.00134426	0.00724882
ENSG0000018	TAFA1	protein_codir	0.49330577	0.24437378	0.38939328
ENSG0000008	STARD7	protein_codir	0.49330122	0.00186915	0.00931205
ENSG0000009	SMARCB1	protein_codir	0.49271283	0.00040371	0.00275525
ENSG0000010	ZNF862	protein_codir	0.49216744	0.02647042	0.07375975
ENSG0000002	FAS	protein_codir	0.49177036	0.00207515	0.01011037
ENSG0000001	QPCTL	protein_codir	0.49176908	0.02141013	0.06249713
ENSG0000000	MAD1L1	protein_codir	0.49146763	0.00092895	0.00540044
ENSG0000003	PI4K2B	protein_codir	0.49132707	0.00599532	0.0233095
ENSG0000025	RP11-299G20	lncRNA	0.49104972	0.41057484	0.56351009
ENSG0000018	CRELD2	protein_codir	0.49086826	0.03132958	0.08410995
ENSG0000023	AC002451.3	lncRNA	0.49061802	0.27121613	0.41921705
ENSG0000017	CHCHD7	protein_codir	0.4903604	2.62E-06	4.11E-05
ENSG0000025	CDK3	protein_codir	0.49028439	0.23728767	0.38140816
ENSG0000025	RP11-463O12	lncRNA	0.48997988	0.57339881	0.7063252
ENSG0000028	CCDC15-DT	lncRNA	0.48990683	0.41074516	0.56371584
ENSG0000005	TTC17	protein_codir	0.48989255	2.61E-08	7.91E-07
ENSG0000016	OXGR1	protein_codir	0.4898489	0.50172817	0.64640974
ENSG0000019	SERPINB2	protein_codir	0.48974509	0.55509577	0.69109356
ENSG0000019	BLOC1S2	protein_codir	0.48966825	0.00336932	0.0148233

ENSG0000022	RP11-197M22	lncRNA	0.48962601	0.67010831	0.78236427
ENSG0000011	TTR	protein_codir	0.48939948	0.74538246	0.83739938
ENSG0000016	CCDC103	protein_codir	0.48938981	0.33088686	0.48349884
ENSG0000006	FGFR3	protein_codir	0.48898516	0.15461627	0.27857153
ENSG0000013	FLNB	protein_codir	0.48884826	0.01026716	0.03554956
ENSG0000012	WWP1	protein_codir	0.48880532	0.00414076	0.01752545
ENSG0000017	LRRN4CL	protein_codir	0.488462	0.30968284	0.46139764
ENSG0000017	PEAK1	protein_codir	0.48838224	0.00782045	0.02869134
ENSG0000013	CHST12	protein_codir	0.48816638	0.00029753	0.00213792
ENSG0000018	HPDL	protein_codir	0.48801616	0.4348426	0.58627123
ENSG0000023	AC087294.2	lncRNA	0.48759036	0.41184248	0.56480528
ENSG0000016	RAD21	protein_codir	0.48757048	0.0003925	0.00269471
ENSG0000009	EFHC1	protein_codir	0.48748165	0.06086776	0.13949282
ENSG0000025	CEP95	protein_codir	0.48710456	0.01278894	0.04209134
ENSG0000012	DACH2	protein_codir	0.48708421	0.61948925	0.74292445
ENSG0000017	FBXO34	protein_codir	0.48705176	0.00034206	0.00240585
ENSG0000025	RPL36A-HNRN	protein_codir	0.48703977	0.00119017	0.00656795
ENSG0000006	SLC6A16	protein_codir	0.48690123	0.17560544	0.30581371
ENSG0000010	KDM4C	protein_codir	0.48687328	5.90E-05	0.00056528
ENSG0000014	TIFA	protein_codir	0.48654453	0.05376952	0.12710902
ENSG0000017	CST2	protein_codir	0.48650262	0.614931	0.73897704
ENSG0000025	HOXA10	protein_codir	0.48619192	0.36831796	0.52176961
ENSG0000016	CEP57	protein_codir	0.48618741	0.00010448	0.00090127
ENSG0000027	RP11-647K16.	lncRNA	0.48598808	0.32561087	0.47783711
ENSG0000022	TTTY10	lncRNA	0.48592877	0.322298	0.47448808
ENSG0000026	RP11-524F11.	lncRNA	0.4858923	0.83381579	NA
ENSG0000014	BOC	protein_codir	0.48584566	0.26845997	0.41625412
ENSG0000010	PEX11G	protein_codir	0.48565319	0.03847923	0.09842307
ENSG0000013	GCHFR	protein_codir	0.48552432	0.07147048	0.15741744
ENSG0000013	SLC49A4	protein_codir	0.48544325	0.02245074	0.06489684
ENSG0000006	SBNO2	protein_codir	0.48542787	0.05065567	0.12174641
ENSG0000028	LINC02348	lncRNA	0.48538872	0.42601019	0.57786079
ENSG0000022	SLC16A1-AS1	lncRNA	0.48537535	0.11646133	0.22598441
ENSG0000012	PROSER1	protein_codir	0.48517426	0.00393189	0.01677981
ENSG0000010	RPL34	protein_codir	0.48513345	0.0114451	0.03870148
ENSG0000025	SMIM3	protein_codir	0.48508737	0.07972837	0.17020385
ENSG0000023	RP11-733O18	lncRNA	0.48494928	0.09591921	0.19544483
ENSG0000027	AL133243.2	lncRNA	0.48457979	0.10830814	0.21421201
ENSG0000012	XPNPEP2	protein_codir	0.4845205	0.1890739	0.32311725
ENSG0000025	CERNA1	lncRNA	0.48439502	0.27995283	0.42902386
ENSG0000022	IL10RB-DT	lncRNA	0.48420072	0.24817064	0.39366373
ENSG0000027	RP6-74O6.6	lncRNA	0.48419247	0.4614039	0.61163255
ENSG0000013	GCC2	protein_codir	0.48404634	6.09E-05	0.00057947
ENSG0000014	EBP	protein_codir	0.48402701	0.00064696	0.00404093
ENSG0000015	PIGF	protein_codir	0.48401678	0.02010584	0.05953933
ENSG0000006	EIF4B	protein_codir	0.48395012	0.00654987	0.02499901
ENSG0000011	PPWD1	protein_codir	0.48373583	0.00591381	0.02308033

ENSG0000023	CFAP58-DT	lncRNA	0.48255546	0.35697089	0.51059222
ENSG0000017	SHMT1	protein_codir	0.48245107	0.05864432	0.1355558
ENSG0000027	LINC00221	lncRNA	0.48239417	0.80543787	0.87940387
ENSG0000024	RP11-1277A3	lncRNA	0.48227652	0.08435151	0.17745362
ENSG0000013	SARAF	protein_codir	0.48214183	0.00436221	0.01822207
ENSG0000006	UHRF1BP1	protein_codir	0.48205875	0.019751	0.05873412
ENSG0000028	RP11-125B2.1	lncRNA	0.48200778	0.29792696	0.44875076
ENSG0000025	ITFG2-AS1	lncRNA	0.48199648	0.34507349	0.49797726
ENSG0000013	TDG	protein_codir	0.48193764	6.64E-05	0.00062128
ENSG0000010	ANKRD26	protein_codir	0.48163323	0.01750572	0.05354464
ENSG0000026	XXbac-BPG32	protein_codir	0.48162211	0.33178469	0.48458004
ENSG0000000	REV3L	protein_codir	0.48157639	0.04218438	0.10582493
ENSG0000000	CFH	protein_codir	0.48140623	0.1666263	0.29455548
ENSG0000010	EVX1	protein_codir	0.48135147	0.67225452	0.78383502
ENSG0000019	PAX9	protein_codir	0.48108222	0.49714112	0.64289904
ENSG0000011	SPCS2	protein_codir	0.48106605	0.00145413	0.00769851
ENSG0000018	LINC00910	lncRNA	0.4808409	0.15803242	0.2830829
ENSG0000028	RP13-516M14	lncRNA	0.48071654	0.2598759	0.40701856
ENSG0000017	KBTBD2	protein_codir	0.48063349	0.00029672	0.00213422
ENSG0000011	MADD	protein_codir	0.48036588	0.00037708	0.00260635
ENSG0000017	AGTRAP	protein_codir	0.48030345	0.03098546	0.08345376
ENSG0000000	M6PR	protein_codir	0.48029613	0.00261901	0.01211676
ENSG0000000	SPPL2B	protein_codir	0.48029186	0.06501475	0.14705204
ENSG0000012	RPL21	protein_codir	0.48022412	0.00694454	0.02618541
ENSG0000016	SLC25A44	protein_codir	0.48017922	0.04651612	0.11394903
ENSG0000010	CPD	protein_codir	0.48012843	0.0914353	0.1884659
ENSG0000020	CFAP99	protein_codir	0.4797965	0.62404796	0.74651153
ENSG0000012	LYPLA1	protein_codir	0.47977841	0.00583189	0.02282189
ENSG0000021	DYNLT2B	protein_codir	0.47976107	0.06787529	0.15172746
ENSG0000017	GLDC	protein_codir	0.47962962	0.28580445	0.43536785
ENSG0000026	CTD-3162L10.	lncRNA	0.47940577	0.45321863	0.60370864
ENSG0000027	CTD-2027I19.	lncRNA	0.47929842	0.73253421	0.82828816
ENSG0000027	CTB-75G16.3	lncRNA	0.47889335	0.52318371	0.66539273
ENSG0000010	LARP4B	protein_codir	0.47888037	1.73E-05	0.00020218
ENSG0000023	RP11-65N13.8	lncRNA	0.47882328	0.80049351	NA
ENSG0000011	GPD2	protein_codir	0.47876358	0.00641521	0.02462314
ENSG0000026	KLF14	protein_codir	0.47855086	0.57686174	0.70921325
ENSG0000012	CAT	protein_codir	0.47841754	0.06616953	0.14899372
ENSG0000020	PPP1R10	protein_codir	0.47824293	0.00272908	0.01252531
ENSG0000014	ASXL2	protein_codir	0.47812252	0.02487321	0.0703027
ENSG0000017	RPP38-DT	lncRNA	0.47751562	0.6201186	0.74348551
ENSG0000026	CTC-559E9.5	lncRNA	0.47750245	0.12055755	0.23179949
ENSG0000009	OSBPL8	protein_codir	0.47749338	0.01704557	0.05250349
ENSG0000026	RP11-178C3.1	protein_codir	0.47744351	0.63035007	0.75102532
ENSG0000017	DENND4A	protein_codir	0.47743509	0.00852313	0.03068415
ENSG0000017	RABEP2	protein_codir	0.47742423	0.05212035	0.12407895
ENSG0000013	ACVR1B	protein_codir	0.47725381	0.00299668	0.01348898

ENSG0000015	PAXBP1	protein_codir	0.47723334	0.02388655	0.06809233
ENSG0000011	ATP6V0B	protein_codir	0.47713903	0.01616904	0.05052114
ENSG0000027	ENSG0000027	protein_codir	0.47709001	0.63886153	0.75792598
ENSG0000012	IDUA	protein_codir	0.47704028	0.05773945	0.13388999
ENSG0000017	TNFRSF10D	protein_codir	0.47695817	0.08187364	0.17365611
ENSG0000028	RP11-164H16	lncRNA	0.47695456	0.75393902	NA
ENSG0000015	CREB3L1	protein_codir	0.47692312	0.14435418	0.26479011
ENSG0000001	BRCA1	protein_codir	0.47689832	0.11048344	0.21737801
ENSG0000025	RP11-84C10.4	lncRNA	0.47685426	0.83503949	NA
ENSG0000023	GAS6-AS1	lncRNA	0.47684448	0.38855372	0.54149321
ENSG0000013	CDH24	protein_codir	0.47683391	0.13948796	0.25827295
ENSG0000010	SYNGR2	protein_codir	0.47655845	0.02279507	0.06563118
ENSG0000011	HES1	protein_codir	0.47623269	0.23628658	0.38020596
ENSG0000010	CTSZ	protein_codir	0.47620285	0.07242531	0.15898801
ENSG0000018	FAM221A	protein_codir	0.47619429	0.07695625	0.16591901
ENSG0000028	RP4-543J19.8	lncRNA	0.47605478	0.63898397	0.75794224
ENSG0000012	PLD2	protein_codir	0.47598435	0.00419728	0.01770449
ENSG0000014	NARF	protein_codir	0.47551376	0.00671489	0.025455
ENSG0000028	SPEM3	protein_codir	0.47546239	0.86604029	NA
ENSG0000019	PSAP	protein_codir	0.47534101	0.01171425	0.03939935
ENSG0000011	APC2	protein_codir	0.4752756	0.41513769	0.56820633
ENSG0000018	SFXN4	protein_codir	0.47522945	0.01480171	0.0472355
ENSG0000010	POLE2	protein_codir	0.47439072	0.15301258	0.27636721
ENSG0000020	RP5-1096D14	lncRNA	0.4743872	0.77529106	NA
ENSG0000015	CLEC18C	protein_codir	0.47435249	0.55186025	0.68867213
ENSG0000007	FBLN1	protein_codir	0.47435222	0.30476158	0.45622367
ENSG0000013	RDH12	protein_codir	0.47428091	0.58603696	0.71651862
ENSG0000016	PPFIBP2	protein_codir	0.47426662	0.00511785	0.0206448
ENSG0000010	CENPI	protein_codir	0.47414506	0.09457773	0.19347037
ENSG0000013	KCNH3	protein_codir	0.47374627	0.37889722	0.53228193
ENSG0000013	TANK	protein_codir	0.47340144	4.34E-07	8.88E-06
ENSG0000022	ZNF880	protein_codir	0.47327993	0.07212793	0.15848762
ENSG0000017	TMEM107	protein_codir	0.47319834	0.01879211	0.0565401
ENSG0000021	FAM74A1	lncRNA	0.4728795	0.80229217	NA
ENSG0000026	RP11-120K24.	lncRNA	0.47283525	0.72870855	NA
ENSG0000012	KDM4B	protein_codir	0.47280432	0.00102953	0.00586663
ENSG0000013	MYCN	protein_codir	0.47265983	0.34488426	0.49789376
ENSG0000011	LTA4H	protein_codir	0.47261639	0.00469938	0.01934053
ENSG0000009	DTX2	protein_codir	0.472571	0.01943376	0.05803444
ENSG0000024	LINC02223	lncRNA	0.47249733	0.62146456	0.74448522
ENSG0000016	GNL3	protein_codir	0.4723327	0.01713825	0.05271838
ENSG0000020	AGAP6	protein_codir	0.47231325	0.21551589	0.35567333
ENSG0000010	LGALS3BP	protein_codir	0.47215876	0.04052553	0.10251078
ENSG0000012	CNOT1	protein_codir	0.47212517	0.00335179	0.01476863
ENSG0000022	AC084809.2	lncRNA	0.47208532	0.45598872	0.60666708
ENSG0000010	PPP6R1	protein_codir	0.47191203	0.01233848	0.04093069
ENSG0000025	CTD-3065B20	lncRNA	0.47160476	0.76262659	NA

ENSG0000013	NECTIN2	protein_codir	0.47149288	0.03714246	0.09582241
ENSG0000023	CDK6-AS1	lncRNA	0.47140793	0.63797874	0.75733756
ENSG0000002	SNAPC1	protein_codir	0.47133533	0.09431156	0.19312613
ENSG0000025	LINC01489	lncRNA	0.47130711	0.64160675	0.75962014
ENSG0000011	HECA	protein_codir	0.47121542	0.00844289	0.03043496
ENSG0000021	HAUS3	protein_codir	0.47109987	0.00069441	0.00428023
ENSG0000020	HSPA1A	protein_codir	0.47095195	0.42866969	0.5802998
ENSG0000013	RPS24	protein_codir	0.47077534	0.01645405	0.05112622
ENSG0000011	MOB1A	protein_codir	0.47069713	0.0005851	0.00371503
ENSG0000016	GPR146	protein_codir	0.47050489	0.19140617	0.32597351
ENSG0000016	NCSTN	protein_codir	0.47044815	0.00014161	0.00116894
ENSG0000016	RAB24	protein_codir	0.47028793	0.0186527	0.05621864
ENSG0000018	RPL35A	protein_codir	0.47019024	0.0044305	0.01845077
ENSG0000028	RP11-179A9.4	lncRNA	0.4701551	0.64724155	0.76416225
ENSG0000015	PCDH1	protein_codir	0.4701303	0.06765739	0.15145867
ENSG0000021	RP5-832C2.5	lncRNA	0.47011681	0.19079369	0.3253718
ENSG0000013	LMO2	protein_codir	0.46992356	0.06548105	0.14782074
ENSG0000024	ZNF487	protein_codir	0.46989371	0.01698409	0.05236668
ENSG0000026	CEACAM16-A	lncRNA	0.46968843	0.35216102	0.50572267
ENSG0000013	ANKEF1	protein_codir	0.4695882	0.06812039	0.15207063
ENSG0000018	NDOR1	protein_codir	0.46955449	0.07856571	0.16838557
ENSG0000016	RPS14	protein_codir	0.46946384	0.01711796	0.05266773
ENSG0000015	KDM8	protein_codir	0.46940418	0.02708972	0.07512231
ENSG0000011	CCDC88A	protein_codir	0.46933624	0.02404314	0.0684257
ENSG0000023	RP11-613M10	lncRNA	0.46929004	0.27692017	0.42529666
ENSG0000021	GRID2IP	protein_codir	0.46920612	0.19081761	0.32538194
ENSG0000016	ENPP6	protein_codir	0.46918916	0.21799967	0.35842318
ENSG0000028	RP11-325I22.4	lncRNA	0.46917911	0.13817042	0.25629731
ENSG0000012	PRRG2	protein_codir	0.46917306	0.23622446	0.38012815
ENSG0000018	SELENOF	protein_codir	0.46900764	0.00032133	0.00227923
ENSG0000013	AVIL	protein_codir	0.46895702	0.15468746	0.27866342
ENSG0000026	CTD-2561J22.	lncRNA	0.46890193	0.50733611	0.65174992
ENSG0000016	NOXRED1	protein_codir	0.46889646	0.46014267	0.61069341
ENSG0000018	MAGED4B	protein_codir	0.46845246	0.39580577	0.54872034
ENSG0000019	FAM3D	protein_codir	0.46841905	0.60299193	0.72989032
ENSG0000021	CEP43	protein_codir	0.46832547	0.03166197	0.08481268
ENSG0000008	ASAP3	protein_codir	0.46825504	0.03197851	0.0853131
ENSG0000020	TBC1D8	protein_codir	0.46825093	0.02120809	0.06200566
ENSG0000028	RP11-153M7.	lncRNA	0.46811282	0.71295243	0.8139776
ENSG0000026	RP11-798G7.7	lncRNA	0.4680471	0.51952475	0.66229454
ENSG0000019	RORB	protein_codir	0.46797519	0.3146144	0.46650828
ENSG0000006	HIPK2	protein_codir	0.4678364	0.09452669	0.19342326
ENSG0000010	RPS19	protein_codir	0.4677724	0.00741661	0.02755453
ENSG0000028	RP11-731J8.4	lncRNA	0.46757218	0.84012044	NA
ENSG0000025	ANKHD1-EIF4	protein_codir	0.46753495	0.026512	0.07384645
ENSG0000017	FAM71D	protein_codir	0.4675088	0.54654827	0.68440356
ENSG0000022	AC017074.2	lncRNA	0.46736653	0.49150566	0.63824102

ENSG0000027	LINC02538	lncRNA	0.46733975	0.79499173	0.8726174
ENSG0000028	RP11-2B2.1	lncRNA	0.46723256	0.53148866	0.67254853
ENSG0000013	CCNA1	protein_codir	0.46723047	0.34867856	0.50198081
ENSG0000015	NCK1	protein_codir	0.46718829	0.00101028	0.00577483
ENSG0000019	H4C3	protein_codir	0.46711017	0.58899018	0.71896003
ENSG0000017	JUP	protein_codir	0.46699146	0.06551621	0.14787592
ENSG0000012	MBD4	protein_codir	0.46690006	0.02154925	0.06279825
ENSG0000013	TCF19	protein_codir	0.46671448	0.02738746	0.07576577
ENSG0000027	RP11-425M5.	lncRNA	0.46643122	0.49274183	0.63923913
ENSG0000027	RP11-324E6.1	lncRNA	0.46629352	0.71909461	0.81863864
ENSG0000026	CTD-2332E11.	lncRNA	0.46601974	0.70876632	0.81086684
ENSG0000005	SOAT1	protein_codir	0.46590105	0.00569127	0.02240139
ENSG0000022	EPB41L4A-AS:	lncRNA	0.4658546	0.09505231	0.19418231
ENSG0000006	CDON	protein_codir	0.46574317	0.13253259	0.24886464
ENSG0000028	CTD-3220F14.	lncRNA	0.46573019	0.338778	0.49163866
ENSG0000024	LINC01585	lncRNA	0.46570082	0.75922059	NA
ENSG0000011	EHD1	protein_codir	0.46561353	0.08273368	0.17476896
ENSG0000015	PI4K2A	protein_codir	0.4655271	0.00579781	0.02272394
ENSG0000024	LINC01322	lncRNA	0.46552556	0.82773369	0.89577637
ENSG0000013	USPL1	protein_codir	0.46546215	0.00028083	0.00204282
ENSG0000026	CTD-2510F5.6	protein_codir	0.46537707	0.84743811	0.90915075
ENSG0000025	ZHX1-C8orf76	protein_codir	0.46527207	0.25021669	0.3961585
ENSG0000018	ZC3H6	protein_codir	0.46495056	0.00661703	0.02517388
ENSG0000009	HIVEP1	protein_codir	0.4649197	0.02837126	0.07801036
ENSG0000017	SMAD1	protein_codir	0.46490158	0.01376867	0.04458274
ENSG0000010	TAZ	protein_codir	0.46487458	0.07736767	0.16654159
ENSG0000000	RPS20	protein_codir	0.46480214	0.00562665	0.02222022
ENSG0000018	RFX7	protein_codir	0.46476446	0.00727364	0.02711174
ENSG0000012	PMS2	protein_codir	0.46476008	0.01776648	0.05416227
ENSG0000010	PRPS2	protein_codir	0.46474856	0.00345741	0.01512411
ENSG0000027	RP11-373D23	lncRNA	0.46464676	0.49271977	0.63923913
ENSG0000012	P4HA1	protein_codir	0.4646279	0.07616416	0.16475988
ENSG0000022	AC073343.13	lncRNA	0.46450198	0.28857598	0.43817859
ENSG0000010	FBXO24	protein_codir	0.46440332	0.39639019	0.54925485
ENSG0000004	PER3	protein_codir	0.46433558	0.15816541	0.28324766
ENSG0000013	PER2	protein_codir	0.46423338	0.25075525	0.396761
ENSG0000026	CTB-113P19.4	lncRNA	0.46422986	0.51610097	0.65929924
ENSG0000028	DINOL	lncRNA	0.46407299	0.57899399	0.71100009
ENSG0000015	TMEM69	protein_codir	0.46381574	0.00160479	0.00831288
ENSG0000017	RPLP2	protein_codir	0.46379674	0.03903813	0.09957823
ENSG0000025	RP11-363E6.3	lncRNA	0.46366812	0.55236511	0.68914646
ENSG0000008	EPS15	protein_codir	0.46345352	7.16E-05	0.00066185
ENSG0000007	SEL1L	protein_codir	0.46337656	0.05144126	0.12296042
ENSG0000023	TONSL-AS1	lncRNA	0.46296425	0.54069496	0.67959021
ENSG0000019	ITSN2	protein_codir	0.46285511	0.00023294	0.00174738
ENSG0000020	TTC23L	protein_codir	0.46261824	0.37474457	0.52829969
ENSG0000013	POSTN	protein_codir	0.46254133	0.40402834	0.55712745

ENSG0000012	RAP1B	protein_codir	0.46238545	0.0001725	0.00137382
ENSG0000013	ALG10	protein_codir	0.46221269	0.0775016	0.1667519
ENSG0000016	DCST2	protein_codir	0.46209692	0.36522373	0.51905605
ENSG0000011	PHTF1	protein_codir	0.46200963	0.00715267	0.02675471
ENSG0000028	RP11-640F22.	lncRNA	0.46193439	0.49842815	0.64417133
ENSG0000010	DECR1	protein_codir	0.46191192	0.0011401	0.00634158
ENSG0000015	MX1	protein_codir	0.46167744	0.16524269	0.29263086
ENSG0000025	LINC02733	lncRNA	0.46165232	0.86730593	NA
ENSG0000017	ZNF497	protein_codir	0.4614747	0.28830713	0.43792878
ENSG0000017	GOLGA8Q	protein_codir	0.46146847	0.55512237	0.69109356
ENSG0000013	NIP7	protein_codir	0.46137209	0.04283211	0.10704153
ENSG0000027	CTD-2600O9.2	lncRNA	0.46126592	0.68854174	0.79560556
ENSG0000027	RP11-571I18.4	lncRNA	0.46103014	0.54942304	0.68668937
ENSG0000018	PCBP3	protein_codir	0.46102015	0.22557065	0.36737198
ENSG0000014	HMBOX1	protein_codir	0.46076313	0.00253245	0.01179731
ENSG0000023	LINC00106	lncRNA	0.46057331	0.3497641	0.50328129
ENSG0000026	AP005530.2	lncRNA	0.4605495	0.80262149	0.87773629
ENSG0000010	SYNDIG1	protein_codir	0.46053539	0.16963174	0.29829845
ENSG0000012	KIAA1549	protein_codir	0.46032926	0.05475986	0.12879735
ENSG0000027	SENP3-EIF4A1	protein_codir	0.46029618	0.29077194	0.44049589
ENSG0000014	HHIPL2	protein_codir	0.46027417	0.36366743	0.51765071
ENSG0000025	FAM160A1-DT	lncRNA	0.46014859	0.8437727	NA
ENSG0000023	AC022431.3	lncRNA	0.46004443	0.84490094	NA
ENSG0000011	FKBP15	protein_codir	0.45994189	0.02261383	0.06525902
ENSG0000025	RP11-156K13.	lncRNA	0.4598815	0.68284003	0.7914079
ENSG0000014	RPS8	protein_codir	0.45983748	0.00431	0.0180531
ENSG0000016	OMA1	protein_codir	0.45982742	0.02983156	0.08110174
ENSG0000011	TXNDC12	protein_codir	0.45956595	0.00037641	0.00260367
ENSG0000021	LINC00339	lncRNA	0.45943562	0.07936169	0.16963086
ENSG0000027	RP11-157E16.	lncRNA	0.45924576	0.69792985	0.80239854
ENSG0000018	ST6GALNAC3	protein_codir	0.45898614	0.07472721	0.1624796
ENSG0000027	RP11-507K2.6	lncRNA	0.45890827	0.2551877	0.40184424
ENSG0000027	RP11-130L8.1	lncRNA	0.45878661	0.5345557	0.67503788
ENSG0000013	RPS6	protein_codir	0.45877186	0.00346297	0.01514362
ENSG0000018	ZNF75D	protein_codir	0.45873686	0.00103758	0.0058967
ENSG0000017	ACSF3	protein_codir	0.45848895	0.04976669	0.12009319
ENSG0000009	ABLIM1	protein_codir	0.45830385	0.09432944	0.19314843
ENSG0000016	SMCO4	protein_codir	0.45824626	0.05184609	0.12360717
ENSG0000020	FAM71F2	protein_codir	0.45804195	0.29593465	0.44643153
ENSG0000020	ATP6V0E2-AS	lncRNA	0.45794008	0.04633694	0.11357054
ENSG0000010	TMED8	protein_codir	0.45789026	0.02534219	0.07136535
ENSG0000013	PPP2R1B	protein_codir	0.45785383	0.0306992	0.08289888
ENSG0000025	AC005519.4	lncRNA	0.45755883	0.35910522	0.51279528
ENSG0000014	PROSER2	protein_codir	0.45699252	0.14355206	0.26364172
ENSG0000012	TGFBI	protein_codir	0.45684437	0.18398206	0.31678726
ENSG0000022	CYTOR	lncRNA	0.45642663	0.21812924	0.35850817
ENSG0000014	ANKMY1	protein_codir	0.4564064	0.02494888	0.07048772

ENSG0000002	SPAST	protein_codir	0.45632189	0.00412787	0.01748646
ENSG0000007	WIPI1	protein_codir	0.45609008	0.01682469	0.05201455
ENSG0000028	CCDC39	protein_codir	0.45598316	0.40617379	0.55916463
ENSG0000016	F2RL1	protein_codir	0.45588823	0.22463359	0.36634269
ENSG0000018	SIAH2	protein_codir	0.45586616	0.00056288	0.00359794
ENSG0000010	MCTS2P	protein_codir	0.45579227	0.55055678	0.68754254
ENSG0000014	ARHGAP17	protein_codir	0.45564563	0.00078938	0.00473661
ENSG0000027	RP11-428J1.4	lncRNA	0.45532193	0.22954569	0.37203267
ENSG0000010	GSTZ1	protein_codir	0.45529479	0.05977285	0.13757719
ENSG0000013	CARD6	protein_codir	0.45526842	0.16213425	0.28844754
ENSG0000021	CBWD6	protein_codir	0.45471798	0.17172709	0.30109199
ENSG0000006	DRD4	protein_codir	0.45469483	0.31059527	0.46238567
ENSG0000028	LLNLR-225F10	protein_codir	0.45452692	0.6078769	0.73377737
ENSG0000017	TCEANC	protein_codir	0.45451258	0.05489222	0.12903354
ENSG0000016	DUS1L	protein_codir	0.45445046	0.01418074	0.0456654
ENSG0000011	HPX	protein_codir	0.45421275	0.31122067	0.46300947
ENSG0000025	LINC02700	lncRNA	0.45404941	0.47650126	0.62468981
ENSG0000015	GALNT14	protein_codir	0.45393781	0.40018217	0.5534818
ENSG0000016	SERTAD3	protein_codir	0.45383676	0.01490727	0.04749555
ENSG0000015	ABCA9	protein_codir	0.45375893	0.42136255	0.57335864
ENSG0000010	MYH3	protein_codir	0.45359388	0.14075454	0.25999067
ENSG0000015	FAM126B	protein_codir	0.45356159	0.00231714	0.01103817
ENSG0000023	AC093326.3	lncRNA	0.45345172	0.63444746	0.7545057
ENSG0000028	RP11-1289C1	protein_codir	0.45325666	0.34271543	0.49586676
ENSG0000027	RP11-546K22.	lncRNA	0.45312997	0.63903697	0.75794224
ENSG0000026	RP11-384L8.1	lncRNA	0.45306165	0.22237027	0.36355314
ENSG0000024	ERICH6-AS1	lncRNA	0.45293722	0.19105543	0.32564933
ENSG0000010	AHR	protein_codir	0.45264487	0.0664092	0.14939493
ENSG0000000	LUC7L	protein_codir	0.45260129	0.17543021	0.30558669
ENSG0000018	F2R	protein_codir	0.4525562	0.20744815	0.34597657
ENSG0000028	RP11-384O8.2	protein_codir	0.45228338	0.41656502	0.56932683
ENSG0000017	ATR	protein_codir	0.45221483	0.0148781	0.04741902
ENSG0000026	RP11-849F2.9	lncRNA	0.45196023	0.41867407	0.5713329
ENSG0000023	MORC2-AS1	lncRNA	0.45184843	0.42225859	0.57423842
ENSG0000016	COX18	protein_codir	0.45179811	0.00906162	0.03225283
ENSG0000019	RPS4X	protein_codir	0.45124152	0.00304323	0.01365918
ENSG0000014	SLC6A3	protein_codir	0.45101893	0.34989453	0.50344274
ENSG0000028	CTD-2192J16.	lncRNA	0.45095105	0.60107273	0.72823743
ENSG0000016	SHANK1	protein_codir	0.4506507	0.30382319	0.45505106
ENSG0000016	TMEM65	protein_codir	0.45039954	0.00310446	0.01387914
ENSG0000011	FGF12	protein_codir	0.45016637	0.22302057	0.36440063
ENSG0000008	ITGAE	protein_codir	0.45008401	0.01685883	0.05207927
ENSG0000023	RP13-314C10.	lncRNA	0.44971408	0.5888515	0.71882253
ENSG0000018	HMCES	protein_codir	0.44964049	0.06690139	0.15019234
ENSG0000016	PMF1	protein_codir	0.44962671	0.01265307	0.04172869
ENSG0000028	RP11-69M1.9	lncRNA	0.44945588	0.64539631	0.76296208
ENSG0000018	SYNE4	protein_codir	0.44935385	0.42888935	0.58045491

ENSG0000023	RP4-564M11.	lncRNA	0.44932813	0.6105655	0.73587737
ENSG0000028	RP11-335G13	lncRNA	0.44928919	0.63478194	0.75476425
ENSG0000017	KCNK7	protein_codir	0.44923293	0.34378049	0.49689764
ENSG0000027	RP5-908M14.	lncRNA	0.44907628	0.24041839	0.38483662
ENSG0000017	TMEM134	protein_codir	0.44902235	0.05882717	0.13585337
ENSG0000018	RNF133	protein_codir	0.44900405	0.62058626	0.74385247
ENSG0000014	SDE2	protein_codir	0.44899427	0.06625148	0.14912768
ENSG0000021	TRIM59	protein_codir	0.4489917	0.20672856	0.34498477
ENSG0000026	AC005339.2	lncRNA	0.44896753	0.74208584	0.83541704
ENSG0000022	RP11-290D2.3	lncRNA	0.44893399	0.62380197	0.74624958
ENSG0000008	GRAMD1A	protein_codir	0.44887944	0.00687517	0.02596355
ENSG0000027	RP1-178F15.4	lncRNA	0.44862622	0.2721852	0.42015062
ENSG0000010	NXT2	protein_codir	0.44854946	0.01736804	0.05321674
ENSG0000024	STARD4-AS1	lncRNA	0.4481333	0.37479422	0.52831744
ENSG0000028	LINC01260	lncRNA	0.44803694	0.55160854	0.68845132
ENSG0000012	SERP1	protein_codir	0.44789828	0.00148129	0.00781233
ENSG0000013	LRIG3	protein_codir	0.44787795	0.13103827	0.24666237
ENSG0000011	YPEL5	protein_codir	0.44782605	0.03742517	0.09635376
ENSG0000024	LINC01234	lncRNA	0.44758907	0.67882441	0.78873965
ENSG0000028	CCDC163	protein_codir	0.44746521	0.16761377	0.29591884
ENSG0000011	QKI	protein_codir	0.44729753	0.01981764	0.05891327
ENSG0000018	KIAA0825	protein_codir	0.44721839	0.0503549	0.12121338
ENSG0000017	FBXL14	protein_codir	0.44716928	0.00754839	0.02790984
ENSG0000014	HCN3	protein_codir	0.44693257	0.06246747	0.14245437
ENSG0000018	CLEC4G	protein_codir	0.44674248	0.51505086	0.65829268
ENSG0000014	CEP170	protein_codir	0.44637188	0.00138451	0.00739801
ENSG0000009	MKNK2	protein_codir	0.4462429	0.03896116	0.09942643
ENSG0000016	GLB1L	protein_codir	0.4462175	0.05346311	0.1265448
ENSG0000016	SPRY1	protein_codir	0.44619157	0.20054789	0.33736115
ENSG0000014	DLX1	protein_codir	0.44610697	0.5664401	0.70049032
ENSG0000014	CHMP7	protein_codir	0.44557539	0.037007	0.09555328
ENSG0000008	RCOR1	protein_codir	0.44553813	0.00432531	0.018098
ENSG0000027	RP11-343L5.2	lncRNA	0.445238	0.22791764	0.37019026
ENSG0000013	CSNK1G2	protein_codir	0.44509038	0.00825307	0.0299284
ENSG0000024	PPAN-P2RY11	protein_codir	0.44500953	0.13495617	0.25201914
ENSG0000008	SMOX	protein_codir	0.44497193	0.14793114	0.26965801
ENSG0000013	ARHGEF39	protein_codir	0.44497166	0.30290747	0.45420374
ENSG0000016	SSR2	protein_codir	0.44491265	0.00051017	0.00332101
ENSG0000025	RP11-488C13.	lncRNA	0.44468057	0.59379174	0.72255387
ENSG0000014	CENPC	protein_codir	0.4446693	0.00095934	0.0055443
ENSG0000025	RP11-593F23.	lncRNA	0.4446678	0.67143025	0.78325594
ENSG0000017	B3GNT4	protein_codir	0.44461573	0.31899737	0.47103488
ENSG0000012	SDF2L1	protein_codir	0.44454819	0.101765	0.20447761
ENSG0000026	RP11-254F7.2	lncRNA	0.44446179	0.06837598	0.15239296
ENSG0000017	CD248	protein_codir	0.44429943	0.24624678	0.39154649
ENSG0000027	RP5-908M14.	lncRNA	0.44419489	0.23277575	0.37582663
ENSG0000016	HIC2	protein_codir	0.44397127	0.02551131	0.07176841

ENSG0000013	PODNL1	protein_codir	0.44395421	0.30139835	0.45254811
ENSG0000011	GPR89A	protein_codir	0.44321052	0.00011278	0.00096499
ENSG0000013	PDE6A	protein_codir	0.44317208	0.54783279	0.6855356
ENSG0000026	RP11-400F19.	lncRNA	0.44311353	0.28887055	0.43843305
ENSG0000026	RP4-657D16.3	lncRNA	0.44294477	0.19792041	0.3341215
ENSG0000015	MOV10	protein_codir	0.44246113	0.06665801	0.14984082
ENSG0000010	SEC23B	protein_codir	0.4423596	0.05769804	0.13381644
ENSG0000009	CATSPERG	protein_codir	0.44233221	0.12679869	0.24076584
ENSG0000012	HOXD3	protein_codir	0.44223684	0.24345743	0.38853031
ENSG0000027	C2orf15	protein_codir	0.44220622	0.27304214	0.42092085
ENSG0000018	ZBTB2	protein_codir	0.44210489	0.01252003	0.04139867
ENSG0000017	RPL38	protein_codir	0.44192925	0.01169458	0.03935239
ENSG0000009	WDR76	protein_codir	0.44181673	0.08606768	0.18028532
ENSG0000008	ITPKC	protein_codir	0.44154772	0.33399848	0.48693787
ENSG0000024	ABHD14A	protein_codir	0.44134542	0.07260862	0.15925118
ENSG0000024	CCDC13	protein_codir	0.4411741	0.19431181	0.32980349
ENSG0000016	LRATD2	protein_codir	0.44115103	0.18273458	0.31513044
ENSG0000028	RP11-376P6.4	lncRNA	0.44107559	0.85212517	NA
ENSG0000017	ZPLD1	protein_codir	0.44107527	0.51285377	0.65648705
ENSG0000012	STAT5A	protein_codir	0.44102923	0.00049811	0.00325942
ENSG0000028	PRR33	protein_codir	0.44031355	0.67731412	0.78753785
ENSG0000018	HOXC10	protein_codir	0.44028994	0.60613964	0.73251266
ENSG0000013	SLC37A4	protein_codir	0.44024847	0.00918505	0.03259244
ENSG0000025	RP11-298I3.1	lncRNA	0.44024153	0.24834346	0.39389264
ENSG0000025	RP11-395I14.:	lncRNA	0.44022849	0.62932477	0.7502985
ENSG0000015	OTOA	protein_codir	0.44017232	0.45201988	0.60254773
ENSG0000013	AMDHD1	protein_codir	0.44014332	0.4299506	0.58146792
ENSG0000019	OSTC	protein_codir	0.44004571	0.00760951	0.02810199
ENSG0000020	NEU1	protein_codir	0.43988854	0.0093534	0.03305284
ENSG0000015	TNIK	protein_codir	0.4397212	0.01918637	0.05748525
ENSG0000021	AC090616.2	lncRNA	0.43969106	0.27755244	0.42605449
ENSG0000008	ERP29	protein_codir	0.43951933	0.01505717	0.04784619
ENSG0000019	ZNF429	protein_codir	0.43944184	0.11173114	0.21920828
ENSG0000020	MROH6	protein_codir	0.43943463	0.10930346	0.21568634
ENSG0000017	AGAP5	protein_codir	0.43936322	0.33569166	0.48860685
ENSG0000017	UBE2O	protein_codir	0.43905707	0.00019125	0.00148837
ENSG0000016	CASP7	protein_codir	0.43905682	0.01428545	0.04588489
ENSG0000014	WRAP53	protein_codir	0.43832172	0.01002591	0.03487162
ENSG0000017	PRR18	protein_codir	0.43830281	0.5939652	0.7226376
ENSG0000016	KCTD5	protein_codir	0.43816389	0.00390034	0.01666421
ENSG0000023	AC007743.1	lncRNA	0.43798239	0.14164948	0.26117214
ENSG0000028	RP5-1047A19	lncRNA	0.43794799	0.19875259	0.33503489
ENSG0000018	PTP4A3	protein_codir	0.43791164	0.23009037	0.37244893
ENSG0000027	CH507-338C2	lncRNA	0.43790983	0.54254947	0.68118139
ENSG0000017	HTRA3	protein_codir	0.43787035	0.25466264	0.40114868
ENSG0000018	ZNF490	protein_codir	0.43758522	0.16220525	0.28852497
ENSG0000005	TBC1D22A	protein_codir	0.43757431	0.00213639	0.01033578

ENSG0000028	RP11-14H3.7	lncRNA	0.43747663	0.36662456	0.52051867
ENSG0000019	ZNF98	protein_codir	0.43730346	0.39601475	0.54889985
ENSG0000020	H2AC18	protein_codir	0.43730229	0.25279223	0.39897837
ENSG0000018	AP001062.7	lncRNA	0.43716495	0.05501006	0.12923176
ENSG0000011	PARP11	protein_codir	0.4371381	0.02322132	0.06659636
ENSG0000011	BIRC6	protein_codir	0.43704057	0.01322944	0.04321668
ENSG0000018	H2AC20	protein_codir	0.43701426	0.22939989	0.3718761
ENSG0000016	HPGDS	protein_codir	0.43698175	0.14294033	0.26281533
ENSG0000023	HLCS-IT1	lncRNA	0.436934	0.82221238	NA
ENSG0000014	CREB3L4	protein_codir	0.43685594	0.06871335	0.15286578
ENSG0000016	MINAR1	protein_codir	0.43670625	0.16782997	0.29618547
ENSG0000024	LINC02242	lncRNA	0.43636434	0.84849926	NA
ENSG0000016	HNRNPH1	protein_codir	0.43615844	0.05483409	0.12894999
ENSG0000027	XXbac-BPG15	lncRNA	0.43615442	0.66780559	0.78056667
ENSG0000013	LRP4	protein_codir	0.43612812	0.36720044	0.52106844
ENSG0000026	SNHG19	lncRNA	0.43612345	0.17415905	0.30417394
ENSG0000028	RP11-489G11	lncRNA	0.43582767	0.53878466	0.67814747
ENSG0000011	ETV3	protein_codir	0.43568401	0.09242631	0.19012519
ENSG0000017	ADPRM	protein_codir	0.43562575	0.01616969	0.05052114
ENSG0000024	RP11-700H6.1	lncRNA	0.43558239	0.62091415	0.74411632
ENSG0000010	PMP22	protein_codir	0.43544963	0.09490398	0.19400843
ENSG0000026	AC140912.1	lncRNA	0.43539706	0.80303269	NA
ENSG0000007	NDE1	protein_codir	0.43519178	0.00229068	0.01094014
ENSG0000025	RP11-114F10.	lncRNA	0.43510419	0.83063785	NA
ENSG0000017	FAM91A1	protein_codir	0.43487299	0.0053533	0.0213977
ENSG0000016	SPICE1	protein_codir	0.43484478	0.01237819	0.04103778
ENSG0000010	AURKC	protein_codir	0.43465496	0.198615	0.33488471
ENSG0000022	RP11-513I15.(	lncRNA	0.43432989	0.0578879	0.13417247
ENSG0000026	LINC02562	lncRNA	0.43429335	0.85380244	NA
ENSG0000015	SLC6A1	protein_codir	0.43416406	0.23427491	0.37765104
ENSG0000010	JOSD1	protein_codir	0.43394705	0.0292643	0.07996197
ENSG0000011	SEC24A	protein_codir	0.43391415	0.05272277	0.12520352
ENSG0000008	RNF13	protein_codir	0.43390377	0.00096129	0.00555224
ENSG0000016	LRFN5	protein_codir	0.43389274	0.25876163	0.40587178
ENSG0000027	RP11-297J22.	lncRNA	0.43383943	0.54407144	0.68234361
ENSG0000024	ERVFRD-1	protein_codir	0.43352949	0.54768656	0.68538367
ENSG0000015	JAM2	protein_codir	0.4333527	0.13632989	0.25369416
ENSG0000023	RP11-196G11	lncRNA	0.43310005	0.58672114	0.71705034
ENSG0000018	SKOR1	protein_codir	0.43294548	0.11667115	0.22626437
ENSG0000011	SPRING1	protein_codir	0.43288993	0.02301091	0.06610789
ENSG0000013	SLC2A11	protein_codir	0.43272755	0.13682071	0.25440878
ENSG0000012	SLC10A7	protein_codir	0.4327056	0.02040045	0.06017947
ENSG0000013	RPL35	protein_codir	0.43229527	0.01317314	0.0430895
ENSG0000027	CTB-181H17.1	lncRNA	0.43196803	0.40711473	0.56004128
ENSG0000025	RP11-360L9.4	lncRNA	0.43196139	0.50118962	0.6461091
ENSG0000026	CTD-3096M3.	lncRNA	0.43195881	0.85174496	NA
ENSG0000018	ZNRF1	protein_codir	0.43172412	0.01244382	0.04119601

ENSG0000010 CCL2	protein_codir	0.43161452	0.44928701	0.60015028
ENSG0000013 AP4B1	protein_codir	0.43158539	0.09305794	0.1910548
ENSG0000017 RSL1D1	protein_codir	0.4315849	0.00081296	0.00485387
ENSG0000027 RP11-298E9.7	lncRNA	0.43151542	0.68155816	0.79059129
ENSG0000027 ARL2-SNX15	protein_codir	0.43150469	0.85748789	0.9148569
ENSG0000011 IL1RL2	protein_codir	0.43150401	0.30182702	0.45304398
ENSG0000011 WRAP73	protein_codir	0.431149	0.01738979	0.05326485
ENSG0000010 RPL19	protein_codir	0.43111992	0.00253778	0.01181416
ENSG0000011 RLF	protein_codir	0.43081715	0.01810825	0.05491594
ENSG0000027 RP11-295H24	lncRNA	0.43068818	0.36006426	0.51387252
ENSG0000008 KAT6A	protein_codir	0.43060524	0.00212519	0.01029061
ENSG0000024 KCTD21-AS1	lncRNA	0.43047941	0.15685196	0.28154683
ENSG0000022 ROR1-AS1	lncRNA	0.43017743	0.62255321	0.74535857
ENSG0000012 ZNF436	protein_codir	0.43017004	0.1511439	0.27377981
ENSG0000013 TULP4	protein_codir	0.43016006	0.0023634	0.01119486
ENSG0000017 SPTY2D1	protein_codir	0.43001368	0.03077892	0.08301148
ENSG0000000 MPO	protein_codir	0.42976695	0.42262602	0.57462492
ENSG0000025 LINC02482	lncRNA	0.42974582	0.36692807	0.5208425
ENSG0000014 MPZL2	protein_codir	0.42968433	0.22979494	0.37223249
ENSG0000012 FAM124B	protein_codir	0.42965823	0.24719373	0.39243596
ENSG0000010 RECQL5	protein_codir	0.42947496	0.13165105	0.24754592
ENSG0000028 RP11-147H23	protein_codir	0.42913363	0.5849385	0.71569068
ENSG0000010 KHNYN	protein_codir	0.42901719	0.00086177	0.00508486
ENSG0000020 HMGN1	protein_codir	0.42890253	0.07571699	0.16406239
ENSG0000015 UGT2B4	protein_codir	0.42886478	0.82983852	NA
ENSG0000014 TMEM44	protein_codir	0.42882898	0.08519108	0.17887924
ENSG0000026 RP5-902P8.12	lncRNA	0.42873642	0.4407657	0.59174397
ENSG0000014 ABCA12	protein_codir	0.42864533	0.88208543	NA
ENSG0000017 IQCK	protein_codir	0.42856512	0.0065273	0.0249597
ENSG0000028 RP4-773A18.8	lncRNA	0.42841879	0.68729985	0.79487572
ENSG0000028 RP11-181P4.2	lncRNA	0.4283126	0.82551516	NA
ENSG0000025 MEX3A	protein_codir	0.42828655	0.36279183	0.51666323
ENSG0000012 ZMIZ2	protein_codir	0.42813999	0.06278827	0.14308901
ENSG0000026 RP11-448G15	lncRNA	0.42804048	0.23212197	0.37496834
ENSG0000014 TUT1	protein_codir	0.42798038	0.03763283	0.09672611
ENSG0000013 MNS1	protein_codir	0.42780261	0.21355228	0.3532784
ENSG0000028 RP11-249O6.2	lncRNA	0.42776113	0.37044343	0.52384024
ENSG0000000 LAMP2	protein_codir	0.42773474	0.01118702	0.03803365
ENSG0000011 RPS13	protein_codir	0.42760248	0.00750685	0.02780464
ENSG0000019 SERTAD1	protein_codir	0.42758858	0.32782761	0.48022387
ENSG0000013 TPT1	protein_codir	0.42723366	0.00074514	0.00452225
ENSG0000014 ZCCHC7	protein_codir	0.4272137	0.01492061	0.04753257
ENSG0000006 SLC12A2	protein_codir	0.42704888	0.03800765	0.09748085
ENSG0000005 NOP58	protein_codir	0.42704458	0.02513411	0.07089503
ENSG0000027 RP3-402G11.2	lncRNA	0.42663155	0.08836168	0.183721
ENSG0000008 PPP1R15A	protein_codir	0.42656935	0.325825	0.47804975
ENSG0000010 MRGBP	protein_codir	0.42650616	6.19E-05	0.00058762

ENSG0000016	ZCCHC18	protein_codir	0.42647436	0.26831403	0.41617529
ENSG0000018	PRR14L	protein_codir	0.42635274	0.02147479	0.06265137
ENSG0000027	RP11-490B18.	lncRNA	0.42611198	0.71932183	0.8187227
ENSG0000014	AFF3	protein_codir	0.42606645	0.24884862	0.39453526
ENSG0000018	MCEMP1	protein_codir	0.42594844	0.48881558	0.63579404
ENSG0000017	CEBPB	protein_codir	0.4259231	0.2945305	0.4447992
ENSG0000016	GATD3A	protein_codir	0.42591647	NA	NA
ENSG0000017	SYNE3	protein_codir	0.42534665	0.00330891	0.01461806
ENSG0000025	RP11-439C15.	lncRNA	0.4253047	0.59244562	0.72132899
ENSG0000016	AKR7A3	protein_codir	0.42513874	0.39282596	0.54584963
ENSG0000012	TCF15	protein_codir	0.42505738	0.31351397	0.46520124
ENSG0000026	KB-1836B5.1	lncRNA	0.42492245	0.46299221	0.61298257
ENSG0000018	TMEM132E	protein_codir	0.42486378	0.35179351	0.50530639
ENSG0000025	RP11-475A13	lncRNA	0.42485176	0.89506352	0.93738499
ENSG0000022	LINC01205	lncRNA	0.42484514	0.89635438	NA
ENSG0000017	PCARE	protein_codir	0.42468114	0.85660614	0.91435072
ENSG0000023	LINC01700	lncRNA	0.42463816	0.78346726	0.86452657
ENSG0000013	GIT2	protein_codir	0.42459519	0.00959323	0.033672
ENSG0000015	BUB3	protein_codir	0.42458492	0.00106231	0.00600758
ENSG0000027	RP11-344N10	lncRNA	0.42451857	0.3763383	0.52992424
ENSG0000008	PSEN1	protein_codir	0.42427148	9.15E-05	0.00080732
ENSG0000019	LCOR	protein_codir	0.4241509	0.00397469	0.01693149
ENSG0000018	ZNF678	protein_codir	0.42403699	0.05036774	0.12123373
ENSG0000024	RP11-572M11	lncRNA	0.4238186	0.15585959	0.28015371
ENSG0000008	CRYBG3	protein_codir	0.42376821	0.00788124	0.02887002
ENSG0000015	PRXL2B	protein_codir	0.42376818	0.02687361	0.07465792
ENSG0000027	LINC01235	lncRNA	0.4235842	0.47208583	0.62083491
ENSG0000021	TREX1	protein_codir	0.42357718	0.03752365	0.09650834
ENSG0000016	PATL1	protein_codir	0.42356533	0.00109676	0.0061608
ENSG0000013	OLFM1	protein_codir	0.42344117	0.0651775	0.14732813
ENSG0000013	CEPT1	protein_codir	0.42342114	0.04998936	0.12048237
ENSG0000025	AL928654.7	protein_codir	0.42312673	0.47084381	0.61986207
ENSG0000026	NBPF12	protein_codir	0.42309997	0.01436058	0.04606726
ENSG0000018	CMTR2	protein_codir	0.42306634	0.02224565	0.06437814
ENSG0000020	MUC19	protein_codir	0.42284285	0.70165181	0.80557106
ENSG0000013	CIR1	protein_codir	0.42276765	0.01195285	0.03994888
ENSG0000028	PAGR1	protein_codir	0.42269392	0.00725307	0.02704967
ENSG0000006	MTMR1	protein_codir	0.42266231	0.02809167	0.07738763
ENSG0000023	SNHG20	lncRNA	0.42254124	0.15766876	0.28268817
ENSG0000017	MARCHF3	protein_codir	0.42252568	0.22195575	0.36305561
ENSG0000003	MAT2B	protein_codir	0.42252443	0.0088396	0.03157655
ENSG0000019	CRACDL	protein_codir	0.42242543	0.13873149	0.2571481
ENSG0000014	RPS11	protein_codir	0.4223725	0.01595939	0.0500282
ENSG0000023	AC016910.1	lncRNA	0.422335	0.66557502	0.77908044
ENSG0000028	RP11-439D8.4	lncRNA	0.42232068	0.34402095	0.4971558
ENSG0000016	H3-3A	protein_codir	0.42220703	9.07E-05	0.00080179
ENSG0000010	CRACD	protein_codir	0.42218897	0.31494048	0.46674122

ENSG0000012	PPP3CC	protein_codir	0.42192783	0.00254372	0.01183785
ENSG0000019	MVB12B	protein_codir	0.42182429	0.03946318	0.10046535
ENSG0000016	RFWD3	protein_codir	0.42178705	0.03196858	0.08530307
ENSG0000016	TLCD1	protein_codir	0.42166009	0.26695259	0.41455248
ENSG0000006	TRAM1	protein_codir	0.42157192	0.02321228	0.06658252
ENSG0000028	RP11-679B19.	lncRNA	0.42149568	0.37182034	0.52522261
ENSG0000011	NEK6	protein_codir	0.42132546	0.07550409	0.16374333
ENSG0000002	ZNF839	protein_codir	0.42098901	0.04624236	0.11339649
ENSG0000013	DERL1	protein_codir	0.42096121	0.00233901	0.01111744
ENSG0000013	CYP2J2	protein_codir	0.42087727	0.5525023	0.6891931
ENSG0000027	YTHDF3-DT	lncRNA	0.42076134	0.31345333	0.4651696
ENSG0000011	COLEC11	protein_codir	0.42052579	0.39268122	0.54570341
ENSG0000024	ARHGEF35-AS	lncRNA	0.42005312	0.03059121	0.08269026
ENSG0000018	EYS	protein_codir	0.41970402	0.81121332	0.88347115
ENSG0000016	ERMAP	protein_codir	0.41952646	0.00597089	0.02324071
ENSG0000026	RP11-61J19.5	lncRNA	0.41934681	0.16761949	0.29591884
ENSG0000010	KLF3	protein_codir	0.41931255	0.07824424	0.16785289
ENSG0000026	RP6-99M1.3	lncRNA	0.41921276	0.69311164	0.79852105
ENSG0000018	PAK2	protein_codir	0.41913384	0.01082563	0.03708805
ENSG0000014	WTAP	protein_codir	0.41899712	0.01842801	0.05567933
ENSG0000026	RP11-295D4.1	lncRNA	0.41891217	0.16280828	0.28929264
ENSG0000016	CENPN	protein_codir	0.41883912	0.07328899	0.16032329
ENSG0000017	RIN1	protein_codir	0.41883166	0.20551059	0.34348009
ENSG0000027	RBAKDN	lncRNA	0.41879762	0.6966111	0.8014829
ENSG0000023	PIK3CD-AS2	lncRNA	0.41872266	0.24526339	0.39037486
ENSG0000014	DOP1B	protein_codir	0.41870435	0.05754046	0.13359679
ENSG0000005	LIMA1	protein_codir	0.4184742	0.03741783	0.09635283
ENSG0000024	RAD51-AS1	lncRNA	0.41830307	0.32590283	0.47813854
ENSG0000012	ORMDL1	protein_codir	0.41827392	0.04046991	0.10242636
ENSG0000022	RP1-317E23.3	lncRNA	0.41813727	0.28048302	0.42964567
ENSG0000026	CTD-2619J13.	lncRNA	0.41796032	0.58336941	0.71437876
ENSG0000010	DEPDC5	protein_codir	0.41795634	0.02094339	0.06145428
ENSG0000017	PLK3	protein_codir	0.41791389	0.19991769	0.33654699
ENSG0000028	TMDD1	protein_codir	0.41769745	0.33841612	0.49127647
ENSG0000027	CTD-3148I10.	lncRNA	0.41764143	0.57671214	0.70910449
ENSG0000028	GABPB1-IT1	lncRNA	0.41740631	0.00822902	0.02987061
ENSG0000007	KDM5A	protein_codir	0.41719415	0.00018569	0.00145377
ENSG0000013	ARPC5L	protein_codir	0.41711726	0.0312767	0.08403832
ENSG0000026	RP11-571M6.	lncRNA	0.41696126	0.65651089	0.77174118
ENSG0000021	DDX3X	protein_codir	0.41693716	0.03917225	0.09987424
ENSG0000016	QSOX2	protein_codir	0.41675213	0.03972922	0.10100525
ENSG0000011	S100PBP	protein_codir	0.41666975	0.00826896	0.02997225
ENSG0000022	LINC00623	lncRNA	0.41659686	0.00918536	0.03259244
ENSG0000028	RP11-326I11.	lncRNA	0.41654197	0.39450109	0.54754373
ENSG0000013	MICAL1	protein_codir	0.41631342	0.11428553	0.22274831
ENSG0000014	PRPSAP2	protein_codir	0.41626965	0.03687313	0.0952694
ENSG0000013	ITPKA	protein_codir	0.41611636	0.46064325	0.61104373

ENSG0000016	TET2	protein_codir	0.41598275	0.01512388	0.04799191
ENSG0000010	MRPS31	protein_codir	0.41596711	0.00689942	0.02604017
ENSG0000010	DNMBP	protein_codir	0.41593441	0.09228097	0.18989722
ENSG0000017	METRNL	protein_codir	0.41588166	0.24463207	0.38963807
ENSG0000027	RP11-305O4.3	lncRNA	0.41583021	0.71757844	0.81753527
ENSG0000028	RP11-128N14	lncRNA	0.41569247	0.32430194	0.47666138
ENSG0000027	RP11-448A19	lncRNA	0.41556978	0.10898092	0.21521901
ENSG0000017	CES4A	protein_codir	0.41546747	0.24411683	0.38920407
ENSG0000019	TMEM116	protein_codir	0.41529439	0.07968133	0.17012975
ENSG0000018	MGAT4C	protein_codir	0.41517114	0.51324638	0.65682518
ENSG0000010	SEPSECS	protein_codir	0.4150838	0.09981045	0.20150316
ENSG0000026	AC007292.3	lncRNA	0.41505712	0.254528	0.40101381
ENSG0000022	LINC02076	lncRNA	0.41501035	0.5544004	0.69059392
ENSG0000017	ZNF518B	protein_codir	0.41497597	0.011931	0.03990002
ENSG0000027	RP11-74J13.8	lncRNA	0.4149516	0.41691446	0.56960711
ENSG0000016	FBN1	protein_codir	0.41460493	0.17508397	0.30519528
ENSG0000017	ZNF318	protein_codir	0.41456566	0.037437	0.09637525
ENSG0000026	ATP2A1-AS1	lncRNA	0.41453551	0.55284398	0.68937374
ENSG0000015	CCDC17	protein_codir	0.41452864	0.30277562	0.45416336
ENSG0000017	SLC19A1	protein_codir	0.4145192	0.10032646	0.20219022
ENSG0000021	ZNF337-AS1	lncRNA	0.41440409	0.12989576	0.24497718
ENSG0000007	UBE2D1	protein_codir	0.41421918	0.00153151	0.00800382
ENSG0000023	MYLK-AS1	lncRNA	0.4140455	0.23669409	0.3806621
ENSG0000017	PER1	protein_codir	0.41396881	0.21340532	0.35318204
ENSG0000022	LINC01315	lncRNA	0.41381207	0.21952448	0.36028678
ENSG0000017	EPHX4	protein_codir	0.41334201	0.40250921	0.55550688
ENSG0000026	LCN6	protein_codir	0.41323061	0.38034699	0.53366441
ENSG0000010	DDX17	protein_codir	0.41298147	0.03119187	0.08389103
ENSG0000018	ZNF600	protein_codir	0.41286756	0.05080144	0.12199058
ENSG0000018	RFLNB	protein_codir	0.41278175	0.20413792	0.34167309
ENSG0000013	MRPS25	protein_codir	0.41273378	0.04848543	0.11763594
ENSG0000016	OTUD1	protein_codir	0.4125686	0.16866886	0.29709937
ENSG0000019	NAGA	protein_codir	0.41244115	0.02695923	0.07482053
ENSG0000003	PNKP	protein_codir	0.41243685	0.09120032	0.18805172
ENSG0000024	PDXP	protein_codir	0.41241586	0.03262062	0.08672456
ENSG0000012	SLC25A35	protein_codir	0.41239082	0.11865906	0.22909012
ENSG0000017	C12orf66	protein_codir	0.41216475	0.01108416	0.03775525
ENSG0000016	TMEM161B	protein_codir	0.4121236	0.00918991	0.03260023
ENSG0000016	TEX264	protein_codir	0.41203492	0.00062106	0.00390478
ENSG0000016	CA4	protein_codir	0.41186002	0.42962256	0.5812194
ENSG0000010	TRMT1	protein_codir	0.41160977	0.08921291	0.18503121
ENSG0000022	RP5-1086K13.	lncRNA	0.41152077	0.45904495	0.60947082
ENSG0000018	SUMO3	protein_codir	0.41105624	0.00091956	0.00535715
ENSG0000015	HSPA13	protein_codir	0.41099961	0.05596507	0.13087421
ENSG0000012	IRF3	protein_codir	0.410808	0.12842726	0.24295622
ENSG0000011	ERBIN	protein_codir	0.41079936	0.00584755	0.0228702
ENSG0000018	TMPRSS2	protein_codir	0.41071791	0.74118819	0.8346319

ENSG0000006	PSME4	protein_codir	0.41060013	0.00300694	0.01352557
ENSG0000007	UNG	protein_codir	0.41055729	0.00823793	0.02989116
ENSG0000006	GLP2R	protein_codir	0.41026875	0.37963711	0.5329662
ENSG0000015	INO80C	protein_codir	0.41016923	0.0099419	0.03465801
ENSG0000023	LINC00987	lncRNA	0.41009945	0.15778155	0.28283531
ENSG0000016	CAMK2N2	protein_codir	0.41004743	0.5779151	0.7101386
ENSG0000028	RP11-19D22.1	lncRNA	0.40991018	0.58428901	0.71521283
ENSG0000011	CCNC	protein_codir	0.40990782	0.00074098	0.00450451
ENSG0000015	ING1	protein_codir	0.40988637	0.02925539	0.07995586
ENSG0000016	CRTC2	protein_codir	0.40971685	0.01049194	0.03616466
ENSG0000014	IDNK	protein_codir	0.40956703	0.0307005	0.08289888
ENSG0000014	DOCK11	protein_codir	0.40954629	0.05541302	0.1299022
ENSG0000014	SUSD4	protein_codir	0.40950315	0.35335621	0.50694465
ENSG0000000	SLC25A13	protein_codir	0.40942448	0.02307927	0.06627672
ENSG0000016	GPR155	protein_codir	0.4092705	0.0190972	0.057246
ENSG0000023	LINC01762	lncRNA	0.40925568	0.74911159	0.83998303
ENSG0000023	DICER1-AS1	lncRNA	0.40924914	0.23201362	0.37481524
ENSG0000014	ATP6V1B2	protein_codir	0.40916282	0.04140647	0.1042522
ENSG0000016	SRSF2	protein_codir	0.4089205	0.01744593	0.05339723
ENSG0000027	AC006946.17	lncRNA	0.40885111	0.65555902	0.77088471
ENSG0000010	SRSF5	protein_codir	0.4087359	0.00248676	0.01164465
ENSG0000007	NFATC3	protein_codir	0.40870928	0.00425908	0.01789139
ENSG0000017	GRIK1-AS1	lncRNA	0.40860174	0.60651568	0.73277472
ENSG0000018	BRICD5	protein_codir	0.408448	0.38084813	0.53420473
ENSG0000013	UBAC2	protein_codir	0.40836482	3.64E-05	0.00037644
ENSG0000018	APOD	protein_codir	0.40796731	0.42973042	0.58124962
ENSG0000011	USP34	protein_codir	0.40764332	0.01339366	0.04363961
ENSG0000018	RNF135	protein_codir	0.40758397	0.03357369	0.08872038
ENSG0000021	NUP62	protein_codir	0.40757717	0.00963796	0.03380322
ENSG0000028	RP11-181E22.	lncRNA	0.40757263	0.69443944	0.79973465
ENSG0000014	METTL21A	protein_codir	0.40736636	0.01831648	0.05541086
ENSG0000028	RP3-510L9.2	lncRNA	0.40734742	0.65649529	0.77174118
ENSG0000027	RP11-407N8.6	lncRNA	0.40699194	0.76671561	0.85293561
ENSG0000028	LINC02801	lncRNA	0.40692631	0.74241605	0.83552202
ENSG0000016	FZD5	protein_codir	0.40681436	0.03587325	0.09328731
ENSG0000020	LY6G5C	protein_codir	0.40678783	0.06307463	0.14356172
ENSG0000010	RPS16	protein_codir	0.40673714	0.01729359	0.05304539
ENSG0000025	RP13-884E18.	lncRNA	0.40668696	0.6570835	0.77210237
ENSG0000013	ZSCAN5A	protein_codir	0.4066722	0.01529548	0.04842458
ENSG0000006	TNK2	protein_codir	0.40653602	0.08750262	0.18237416
ENSG0000018	SDR42E1	protein_codir	0.40650155	0.45621008	0.60690311
ENSG0000015	HAUS1	protein_codir	0.40650043	0.00202096	0.00989874
ENSG0000014	CACNA1A	protein_codir	0.40648502	0.31552265	0.4673031
ENSG0000016	XRRA1	protein_codir	0.40639213	0.07803341	0.16755678
ENSG0000017	CIDEA	protein_codir	0.40638866	0.62875766	0.74985794
ENSG0000025	FDXACB1	protein_codir	0.40628562	0.26403504	0.41150254
ENSG0000019	UVRAG	protein_codir	0.40626274	0.0049117	0.01999723

ENSG0000015	NCAPD3	protein_codir	0.40613603	0.01598794	0.05009493
ENSG0000016	UBXN7	protein_codir	0.4059911	0.00828683	0.03001733
ENSG0000017	CLCN5	protein_codir	0.40592767	0.0468128	0.1144322
ENSG0000028	RP3-453A3.2	lncRNA	0.40591044	0.46208966	0.61220713
ENSG0000017	SLC25A33	protein_codir	0.40577558	0.16675959	0.294721
ENSG0000018	GSX2	protein_codir	0.40559142	0.864851	NA
ENSG0000016	SNAPC4	protein_codir	0.40548035	0.03780848	0.09708722
ENSG0000012	KCNC1	protein_codir	0.40546999	0.74271592	0.83567307
ENSG0000006	RPL18	protein_codir	0.40546318	0.02730955	0.07561044
ENSG0000014	RXRG	protein_codir	0.40539652	0.54068971	0.67959021
ENSG0000019	MT-CO1	protein_codir	0.40533286	0.17842861	0.30944357
ENSG0000023	LINC02814	lncRNA	0.40519303	0.61147039	0.73658271
ENSG0000007	POLB	protein_codir	0.40518681	0.00159541	0.00827674
ENSG0000018	IRAK1	protein_codir	0.40498663	0.0094414	0.03327441
ENSG0000017	DPP7	protein_codir	0.40496173	0.01540368	0.04865617
ENSG0000005	ARID4B	protein_codir	0.40481168	0.00075365	0.00456786
ENSG0000012	SLC35B1	protein_codir	0.40467927	0.00657089	0.02504318
ENSG0000027	RP11-8P13.5	lncRNA	0.40465015	0.79576498	0.87311916
ENSG0000025	TEN1	protein_codir	0.40464527	0.05322227	0.1260936
ENSG0000016	ARF6	protein_codir	0.40463435	0.01154205	0.03894988
ENSG0000013	TEC	protein_codir	0.40461717	0.064965	0.14695604
ENSG0000022	LAMTOR5-AS1	lncRNA	0.40448949	0.23867019	0.38290374
ENSG0000017	RASA4B	protein_codir	0.40441759	0.1637982	0.29071497
ENSG0000012	RBBP6	protein_codir	0.4043358	0.01310234	0.04290807
ENSG0000023	RP11-46H11.1	lncRNA	0.40404598	0.5999112	0.7274609
ENSG0000003	ARID4A	protein_codir	0.40387937	0.00235243	0.01115956
ENSG0000025	RP11-549B18.	lncRNA	0.40387408	0.42616577	0.57793615
ENSG0000014	TAMM41	protein_codir	0.40382557	0.05527358	0.12962585
ENSG0000010	SPG11	protein_codir	0.40374926	0.01614013	0.05047445
ENSG0000011	NOP2	protein_codir	0.40368871	0.09177226	0.18901952
ENSG0000011	AKAP3	protein_codir	0.40366049	0.23892845	0.38319075
ENSG0000018	ZNF292	protein_codir	0.40355649	0.01772606	0.05407486
ENSG0000017	PUS1	protein_codir	0.40348712	0.04266461	0.10674776
ENSG0000006	ATG2B	protein_codir	0.4031683	6.12E-05	0.0005825
ENSG0000005	LETMD1	protein_codir	0.40313249	0.01412819	0.04552801
ENSG0000023	RP11-34P13.8	lncRNA	0.40304221	0.56351318	0.69794771
ENSG0000013	EGF	protein_codir	0.40300884	0.48894762	0.63593583
ENSG0000018	MSANTD1	protein_codir	0.40295357	0.44915078	0.60002634
ENSG0000011	MDM1	protein_codir	0.40282826	0.03900901	0.09951316
ENSG0000022	RP11-31F19.1	lncRNA	0.40281409	0.33453327	0.48746024
ENSG0000025	RP11-618G20	lncRNA	0.40279518	0.70803225	0.81050442
ENSG0000012	CCDC32	protein_codir	0.40270541	0.03327452	0.08810683
ENSG0000018	FGD6	protein_codir	0.40262095	0.03422312	0.09007457
ENSG0000023	AC006372.6	lncRNA	0.40206402	0.65112521	0.76714065
ENSG0000022	ARHGAP29-AS	lncRNA	0.40159288	0.74840367	0.83965258
ENSG0000018	SEPTIN9	protein_codir	0.40119236	0.01820467	0.05513301
ENSG0000022	DHDDS-AS1	lncRNA	0.40100432	0.49159701	0.63826961

ENSG0000025	CTD-2336O2.1	lncRNA	0.40097389	0.00928506	0.03287615
ENSG0000027	RP11-140H17	lncRNA	0.40088995	0.58124264	0.71271606
ENSG0000016	BTD	protein_codir	0.40083524	0.03446907	0.0905716
ENSG0000012	STK35	protein_codir	0.40079846	0.03555041	0.09266513
ENSG0000018	MITF	protein_codir	0.40074498	0.18636442	0.31973324
ENSG0000021	HEXA	protein_codir	0.40042814	0.00186949	0.00931206
ENSG0000017	PLEKHG5	protein_codir	0.40042452	0.12375876	0.23647298
ENSG0000011	COMMD9	protein_codir	0.40041575	0.02644348	0.0737225
ENSG0000017	C19orf18	protein_codir	0.4000341	0.46260995	0.61267259
ENSG0000020	CCDC85C	protein_codir	0.39998219	0.11358108	0.22173518
ENSG0000023	RP4-781K5.6	lncRNA	0.39987367	0.7373641	0.83177441
ENSG0000011	MORC1	protein_codir	0.3997332	0.72782179	0.82488461
ENSG0000014	NCOA2	protein_codir	0.3993028	0.02360008	0.06740662
ENSG0000013	RINT1	protein_codir	0.39929478	0.00273899	0.01255617
ENSG0000013	SPX	protein_codir	0.39899389	0.42310292	0.57510008
ENSG0000021	LINC00612	lncRNA	0.39896024	0.55000165	0.68722215
ENSG0000014	IGFBP4	protein_codir	0.39891802	0.28807855	0.4377602
ENSG0000016	ALOX15	protein_codir	0.39870667	0.60325404	0.73001562
ENSG0000018	FAM3B	protein_codir	0.39868529	0.45679164	0.60729652
ENSG0000013	COQ10A	protein_codir	0.3986649	0.08949686	0.18547838
ENSG0000015	PHF6	protein_codir	0.39863785	0.08896222	0.1847012
ENSG0000019	CTSE	protein_codir	0.39851727	0.72864572	0.82549461
ENSG0000013	ZMYM5	protein_codir	0.39847968	0.02947617	0.08038427
ENSG0000027	RP11-295H24	lncRNA	0.39841941	0.7303342	0.82650811
ENSG0000015	AHCYL2	protein_codir	0.3983146	0.00497066	0.02016954
ENSG0000010	RPL3	protein_codir	0.39819674	0.0171113	0.05265757
ENSG0000028	RP11-685G11	lncRNA	0.39805064	0.76771791	NA
ENSG0000014	CNOT9	protein_codir	0.39803203	0.0010332	0.00588026
ENSG0000009	ZNF184	protein_codir	0.3980119	0.01504116	0.04781732
ENSG0000017	ZNF366	protein_codir	0.39791443	0.36444604	0.51843838
ENSG0000026	CTC-444N24.1	lncRNA	0.39759586	0.21001824	0.34916654
ENSG0000015	TMEM268	protein_codir	0.39704328	0.019024	0.05707718
ENSG0000012	EXOSC9	protein_codir	0.39694482	0.00237969	0.01125463
ENSG0000021	NUDT19	protein_codir	0.39682866	0.08980333	0.18590143
ENSG0000022	LINC02620	lncRNA	0.39663201	0.86415722	NA
ENSG0000024	USP2-AS1	lncRNA	0.3965527	0.47135281	0.62025003
ENSG0000007	RPL31	protein_codir	0.39638827	0.01571037	0.04939332
ENSG0000014	OAZ3	protein_codir	0.3962263	0.34849039	0.50176221
ENSG0000016	DCBLD1	protein_codir	0.3959761	0.14756213	0.26919088
ENSG0000024	RP11-274B21.	lncRNA	0.39582989	0.01861861	0.05613427
ENSG0000018	C8orf76	protein_codir	0.39578202	0.04632056	0.11354045
ENSG0000016	PTPN13	protein_codir	0.3956205	0.12868461	0.2433764
ENSG0000013	USP37	protein_codir	0.39534532	0.00838726	0.03028581
ENSG0000013	NPHP4	protein_codir	0.39520931	0.03279891	0.0871231
ENSG0000016	CPLX1	protein_codir	0.39510405	0.23682292	0.38080277
ENSG0000012	GTF3A	protein_codir	0.39507136	0.00532538	0.02130925
ENSG0000015	ATP13A2	protein_codir	0.39481575	0.11019781	0.2169706

ENSG0000011	DPH5	protein_codir	0.39477323	0.0142665	0.04584002
ENSG0000013	TRAFD1	protein_codir	0.39448321	0.01204049	0.04018833
ENSG0000020	C21orf62-AS1	lncRNA	0.3942595	0.21667456	0.35694568
ENSG0000016	SHOX2	protein_codir	0.39424682	0.1538774	0.27754809
ENSG0000025	CTB-131B5.2	lncRNA	0.39412699	0.59049475	0.72005368
ENSG0000016	PRMT9	protein_codir	0.394114	0.04749032	0.11571968
ENSG0000016	LRP1B	protein_codir	0.39410585	0.52254411	0.66494451
ENSG0000018	EFCAB6	protein_codir	0.39409067	0.09547882	0.19481027
ENSG0000027	RP11-666A8.1	lncRNA	0.39405568	0.7194721	0.81878785
ENSG0000008	TBX5	protein_codir	0.39391818	0.54197373	0.68079254
ENSG0000013	ARPC1B	protein_codir	0.39387533	0.08159073	0.17325595
ENSG0000024	RP11-159F24.	lncRNA	0.39372695	0.62174351	0.74470339
ENSG0000017	CCDC96	protein_codir	0.39361427	0.01587205	0.04982226
ENSG0000015	RABGAP1L	protein_codir	0.39342939	0.04674798	0.11433448
ENSG0000016	GPRC5B	protein_codir	0.39337899	0.25039505	0.39632729
ENSG0000007	VPS9D1	protein_codir	0.39333861	0.07212674	0.15848762
ENSG0000010	CLCN3	protein_codir	0.39333211	0.0331032	0.08776256
ENSG0000026	CTC-232P5.3	lncRNA	0.39315741	0.69879943	0.80316511
ENSG0000016	DHRSX	protein_codir	0.3931004	0.0106055	0.03646508
ENSG0000027	RP11-42O4.2	lncRNA	0.39257192	0.73619144	0.83094207
ENSG0000007	LMAN1	protein_codir	0.39242077	0.04253889	0.10654976
ENSG0000023	LIMD1-AS1	lncRNA	0.3923443	0.43976128	0.59074007
ENSG0000020	C6orf136	protein_codir	0.39234186	0.04049108	0.10246116
ENSG0000010	VCPKMT	protein_codir	0.39211304	0.06566359	0.14809966
ENSG0000022	ADM5	protein_codir	0.39170742	0.37513911	0.52866689
ENSG0000015	CACNA2D3	protein_codir	0.39169711	0.33909022	0.49197204
ENSG0000015	JMY	protein_codir	0.39167523	0.06787634	0.15172746
ENSG0000020	TMEM191C	protein_codir	0.39155758	0.38525763	0.53847557
ENSG0000028	RP11-216B9.9	lncRNA	0.39150114	0.69731001	0.80188618
ENSG0000025	RP11-1348G1	lncRNA	0.3913844	0.26393382	0.41141233
ENSG0000013	TAOK3	protein_codir	0.3912711	0.00094163	0.00546156
ENSG0000028	CTD-3247F14.	lncRNA	0.39105785	0.64816787	0.76469975
ENSG0000024	ACY1	protein_codir	0.39088713	0.05922046	0.13655612
ENSG0000024	CASC11	lncRNA	0.39082874	0.50310507	0.64785128
ENSG0000016	ZBTB43	protein_codir	0.39078922	0.01662161	0.05153669
ENSG0000011	IQCG	protein_codir	0.39049723	0.04577933	0.11253337
ENSG0000010	MAPK8	protein_codir	0.39040082	0.001706	0.00870491
ENSG0000016	RICTOR	protein_codir	0.39005639	0.02103991	0.06167063
ENSG0000028	TBCE	protein_codir	0.38982328	0.19127695	0.32585391
ENSG0000024	TICAM2	protein_codir	0.38976812	0.08665678	0.18126781
ENSG0000013	SLC19A3	protein_codir	0.38974711	0.53051688	0.67184211
ENSG0000013	MBD2	protein_codir	0.38950151	0.00134518	0.00725053
ENSG0000010	VRK3	protein_codir	0.38947888	0.0115167	0.03890071
ENSG0000019	SH3BGRL2	protein_codir	0.38936412	0.20230693	0.33934881
ENSG0000014	CCDC97	protein_codir	0.38919999	0.00021497	0.00163521
ENSG0000012	KLK8	protein_codir	0.38919305	0.8407603	NA
ENSG0000018	AGAP4	protein_codir	0.38918739	0.22228213	0.36343056

ENSG0000023	LINC01119	lncRNA	0.38915071	0.34221358	0.4955145
ENSG0000000	FUCA2	protein_codir	0.38908004	0.00665283	0.02527874
ENSG0000026	RP11-533E19.	lncRNA	0.38890285	0.46099608	0.61129731
ENSG0000027	RP5-967N21.1	lncRNA	0.38889773	0.48314663	0.63061811
ENSG0000013	HSD17B7	protein_codir	0.38855767	0.14470444	0.26536994
ENSG0000022	UBA52	protein_codir	0.38851767	0.00819307	0.02977536
ENSG0000016	LRTM2	protein_codir	0.38851147	0.79794924	NA
ENSG0000013	RFX1	protein_codir	0.3884046	0.01012324	0.03515263
ENSG0000024	BCKDHA	protein_codir	0.38820583	0.00075357	0.00456786
ENSG0000022	STIM1-AS1	lncRNA	0.3880961	0.61260929	0.73739512
ENSG0000004	SPDL1	protein_codir	0.38800089	0.01696567	0.05233909
ENSG0000017	RPS7	protein_codir	0.38800048	0.01474701	0.04708816
ENSG0000027	CTC-435M10.	lncRNA	0.38782001	0.60657318	0.73281214
ENSG0000028	RP11-723O4.1	lncRNA	0.38770243	0.66895454	0.78132186
ENSG0000025	PRANCR	lncRNA	0.38762198	0.13960723	0.2584342
ENSG0000010	ERF	protein_codir	0.38758742	0.22880404	0.37125882
ENSG0000015	GPR61	protein_codir	0.38741935	0.79744236	NA
ENSG0000022	CTD-2320J21.	lncRNA	0.38738758	0.82233286	NA
ENSG0000028	CTA-941F9.10	lncRNA	0.38736357	0.34186215	0.49506279
ENSG0000003	CENPQ	protein_codir	0.38736265	0.02035333	0.06010466
ENSG0000013	ERCC5	protein_codir	0.38728965	0.00242835	0.01143387
ENSG0000028	FAS-AS1	lncRNA	0.38699289	0.32268035	0.4747726
ENSG0000012	TRIM24	protein_codir	0.38694517	0.01226396	0.04077161
ENSG0000012	CCDC77	protein_codir	0.38682225	0.01465813	0.04686938
ENSG0000019	C6orf141	protein_codir	0.38615251	0.55184307	0.68867213
ENSG0000017	QARS1	protein_codir	0.38561698	0.0022913	0.01094014
ENSG0000011	GUCA1B	protein_codir	0.38506996	0.23696297	0.38100577
ENSG0000018	ACSM2A	protein_codir	0.38452384	0.69188436	0.79770604
ENSG0000023	RFX3-AS1	lncRNA	0.38435291	0.27185308	0.4198025
ENSG0000027	MYO19	protein_codir	0.38428456	0.09137826	0.18839049
ENSG0000013	STARD3	protein_codir	0.38421051	0.02115993	0.06191727
ENSG0000013	TLR4	protein_codir	0.3840074	0.17303951	0.30273968
ENSG0000018	ZBTB37	protein_codir	0.38394078	0.08341257	0.17589366
ENSG0000017	GATA2	protein_codir	0.38380138	0.23664317	0.38062452
ENSG0000025	RP11-3D4.3	lncRNA	0.38364651	0.49076125	0.63748416
ENSG0000019	ZNF813	protein_codir	0.38357667	0.16871845	0.29709937
ENSG0000024	FLJ42969	lncRNA	0.38347866	0.77489359	0.85874738
ENSG0000017	AFF1	protein_codir	0.38330781	0.02888139	0.07916086
ENSG0000023	AP001469.9	lncRNA	0.38329378	0.53309634	0.67390366
ENSG0000015	ADK	protein_codir	0.38318496	0.00165992	0.00853144
ENSG0000013	XPO4	protein_codir	0.38305534	0.03351998	0.08862084
ENSG0000010	IKBKB	protein_codir	0.38303647	0.03247145	0.08641115
ENSG0000026	RP11-1000B6.	lncRNA	0.3829708	0.26372732	0.41118536
ENSG0000027	RP11-129M16	lncRNA	0.38280127	0.0749693	0.16286394
ENSG0000019	SPG7	protein_codir	0.38272863	0.15431859	0.2781259
ENSG0000017	ASXL1	protein_codir	0.38270081	0.00816084	0.02968152
ENSG0000020	RP11-689P11.	lncRNA	0.38261438	0.25715304	0.40388576

ENSG0000027	RP11-437B10.	protein_codir	0.38247257	0.32079022	0.47292372
ENSG0000011	ACAD10	protein_codir	0.38233744	0.04997979	0.12048237
ENSG0000008	RPL6	protein_codir	0.38224834	0.00738277	0.02746621
ENSG0000014	FAM193B	protein_codir	0.38224384	0.23395295	0.37724243
ENSG0000013	SLC43A3	protein_codir	0.38206593	0.09855737	0.19960072
ENSG0000027	CTD-2047H16	lncRNA	0.38204737	0.64736347	0.76420819
ENSG0000017	RTTN	protein_codir	0.38187068	0.00360242	0.01562829
ENSG0000011	RPS25	protein_codir	0.38185489	0.00872741	0.03126068
ENSG0000028	RP11-31B16.2	lncRNA	0.3817401	0.84571666	NA
ENSG0000014	PMM2	protein_codir	0.38165377	0.04532476	0.11185399
ENSG0000023	PRKAR1B-AS1	lncRNA	0.38151979	0.2440065	0.38911429
ENSG0000012	KANSL1	protein_codir	0.38151213	0.00206054	0.01005069
ENSG0000016	NIPBL	protein_codir	0.38147023	0.00578309	0.022678
ENSG0000027	CTB-58E17.1	lncRNA	0.3812592	0.20204859	0.33916297
ENSG0000006	CDK13	protein_codir	0.38107705	0.00060406	0.00381438
ENSG0000023	SOX9-AS1	lncRNA	0.38090843	0.55312225	0.68953045
ENSG0000017	ANKRD13D	protein_codir	0.3807394	0.10651942	0.21155291
ENSG0000016	TAF10	protein_codir	0.38043147	0.01711276	0.05265757
ENSG0000013	SLC31A1	protein_codir	0.38035555	0.10550891	0.21002927
ENSG0000010	AVL9	protein_codir	0.38022253	0.00289306	0.01310584
ENSG0000027	RP5-1014D13	lncRNA	0.38021058	0.29897269	0.44995781
ENSG0000018	VMAC	protein_codir	0.380193	0.0867747	0.18137711
ENSG0000010	PHF12	protein_codir	0.38002529	0.00142146	0.0075647
ENSG0000008	TXLNG	protein_codir	0.38001606	0.01289037	0.04234946
ENSG0000010	ALG13	protein_codir	0.37986512	0.01420435	0.0457201
ENSG0000010	BNIP3L	protein_codir	0.3798478	0.04760403	0.11590474
ENSG0000016	OTUD4	protein_codir	0.37916284	0.02350252	0.06717092
ENSG0000020	ZNF525	protein_codir	0.37899847	0.1525239	0.27570092
ENSG0000026	CTD-2540F13.	lncRNA	0.37865064	0.48277227	0.63018905
ENSG0000010	STX4	protein_codir	0.37846254	0.04953756	0.11966861
ENSG0000024	EIF4EBP3	protein_codir	0.37839668	0.1111541	0.21840194
ENSG0000021	AC016747.3	lncRNA	0.3783103	0.15566682	0.27991651
ENSG0000016	DIP2A	protein_codir	0.37825589	0.00692125	0.02610474
ENSG0000026	RP11-720N19	lncRNA	0.37821351	0.82283408	NA
ENSG0000012	METTL8	protein_codir	0.37800821	0.00276923	0.01266958
ENSG0000017	SYT9	protein_codir	0.37773217	0.35843236	0.5120992
ENSG0000013	TEX10	protein_codir	0.37763188	0.00163939	0.00845565
ENSG0000027	RP11-343C2.1	protein_codir	0.37763051	0.04576598	0.11252289
ENSG0000027	RP11-350J20.	lncRNA	0.37759686	0.82260827	NA
ENSG0000005	CCDC85A	protein_codir	0.3774395	0.41877258	0.57141086
ENSG0000012	TRAF2	protein_codir	0.37713857	0.10916096	0.21551291
ENSG0000007	FOSL2	protein_codir	0.37713317	0.3037983	0.45505106
ENSG0000011	ZBTB24	protein_codir	0.37694763	0.0737942	0.16109362
ENSG0000014	CISD2	protein_codir	0.37689443	0.00059976	0.00379507
ENSG0000027	RP11-154H23	lncRNA	0.37688877	0.60038193	0.72767014
ENSG0000019	SFT2D1	protein_codir	0.37667839	0.00451016	0.01870432
ENSG0000016	ARHGAP12	protein_codir	0.37663606	0.02092354	0.06141427

ENSG0000008	IGBP1	protein_codir	0.37660171	0.00642482	0.02465661
ENSG0000014	TAF5	protein_codir	0.37647879	0.04566178	0.11239192
ENSG0000003	SH3YL1	protein_codir	0.3763953	0.00686779	0.02594207
ENSG0000014	NXF3	protein_codir	0.37639438	0.48260013	0.6300239
ENSG0000019	ZNF140	protein_codir	0.37631625	0.00148782	0.00783782
ENSG0000013	TMBIM6	protein_codir	0.3763145	0.00081628	0.00486789
ENSG0000019	ARL9	protein_codir	0.37629324	0.41408337	0.56716998
ENSG0000011	HYAL1	protein_codir	0.37610192	0.37805011	0.53170186
ENSG0000027	RP11-532F6.5	lncRNA	0.37608141	0.87651266	NA
ENSG0000027	RP11-177G23	lncRNA	0.37608036	0.64689868	0.76413257
ENSG0000019	ZNF765	protein_codir	0.37602258	0.02827353	0.07780363
ENSG0000016	ATAD3B	protein_codir	0.37585925	0.20283711	0.33998033
ENSG0000012	RNF2	protein_codir	0.37581211	0.00277337	0.01268221
ENSG0000014	PCTP	protein_codir	0.37579848	0.06521347	0.14739738
ENSG0000017	GAK	protein_codir	0.37568075	0.03071179	0.08289888
ENSG0000016	MGAT2	protein_codir	0.37562691	0.07043376	0.15574004
ENSG0000010	EEF1D	protein_codir	0.37549757	0.02174527	0.06322153
ENSG0000013	TARBP2	protein_codir	0.37547676	0.11005644	0.21677011
ENSG0000013	SLC27A1	protein_codir	0.37528257	0.02803321	0.07726127
ENSG0000014	EFCAB11	protein_codir	0.37526865	0.10018852	0.20203015
ENSG0000018	P2RY4	protein_codir	0.375004	0.49061056	0.63734837
ENSG0000026	RP11-142O6.1	lncRNA	0.37491929	0.69054225	0.79699039
ENSG0000004	ZNF800	protein_codir	0.37485636	0.01071616	0.03679058
ENSG0000012	TBC1D15	protein_codir	0.37452285	0.00833344	0.03013876
ENSG0000013	FHOD1	protein_codir	0.3744397	0.07236181	0.15887386
ENSG0000024	MUC20-OT1	lncRNA	0.37439297	0.24680796	0.39199171
ENSG0000028	RP11-76P17.3	lncRNA	0.37421272	0.78100329	0.86305957
ENSG0000018	CCDC159	protein_codir	0.37400064	0.24576096	0.39100087
ENSG0000011	RNF19B	protein_codir	0.37398326	0.27469906	0.42273292
ENSG0000010	SP4	protein_codir	0.3737165	0.06771409	0.15154342
ENSG0000013	THAP12	protein_codir	0.37339299	0.01628267	0.0507593
ENSG0000022	CACNA2D1-AS	lncRNA	0.37338966	0.80329762	NA
ENSG0000024	SRP14-AS1	lncRNA	0.37335267	0.19071285	0.32534122
ENSG0000015	ARID5B	protein_codir	0.37335062	0.24960164	0.39545673
ENSG0000012	DDX27	protein_codir	0.37321933	0.00393671	0.0167938
ENSG0000028	LINC02680	lncRNA	0.37320095	0.69157081	0.79754429
ENSG0000014	RPL7A	protein_codir	0.3727695	0.00941401	0.03320752
ENSG0000012	ABCC10	protein_codir	0.37276567	0.08289892	0.17506446
ENSG0000027	GAR1-DT	lncRNA	0.37275817	0.87723389	0.92717583
ENSG0000006	RASSF1	protein_codir	0.37267088	0.03456734	0.09072982
ENSG0000026	RP11-254F19.	lncRNA	0.37257132	0.66220782	0.77615871
ENSG0000022	CCNL2	protein_codir	0.37250283	0.2423499	0.38725006
ENSG0000020	PBX2	protein_codir	0.3721391	0.0053868	0.02150048
ENSG0000027	NBPF19	protein_codir	0.37211808	0.03459645	0.09078035
ENSG0000017	DNAJC22	protein_codir	0.37176162	0.48100562	0.62862386
ENSG0000016	PUS10	protein_codir	0.37172966	0.08199184	0.17379455
ENSG0000001	ANGEL1	protein_codir	0.37148817	0.01088136	0.03724665

ENSG0000024	SIAH2-AS1	lncRNA	0.37148157	0.67688283	0.78722219
ENSG0000023	AC079807.2	lncRNA	0.37142846	0.52155743	0.66413481
ENSG0000014	GLYAT	protein_codir	0.37108671	0.76092481	0.84900457
ENSG0000018	TACSTD2	protein_codir	0.37105752	0.29320367	0.4433287
ENSG0000009	TECR	protein_codir	0.37097756	0.01857049	0.05603813
ENSG0000016	COX6B2	protein_codir	0.37086265	0.61228049	0.73720347
ENSG0000024	EGFL8	protein_codir	0.37081258	0.30281568	0.45418241
ENSG0000027	BMS1P4	lncRNA	0.37053106	0.42143253	0.57342561
ENSG0000017	FZD8	protein_codir	0.37026825	0.17776564	0.30850067
ENSG0000013	PARP6	protein_codir	0.37025502	0.02623551	0.07327585
ENSG0000027	RP11-18I14.1	lncRNA	0.37010201	0.78951389	0.86874521
ENSG0000025	RP11-6I2.3	lncRNA	0.36945322	0.53665022	0.67676445
ENSG0000018	TDRKH	protein_codir	0.36936194	0.11681223	0.22644258
ENSG0000026	ZNF224	protein_codir	0.36936097	0.21787311	0.35827907
ENSG0000012	CDH26	protein_codir	0.36928448	0.33017305	0.48271118
ENSG0000017	TMPRSS9	protein_codir	0.36917972	0.52981796	0.67111085
ENSG0000013	VPS37B	protein_codir	0.36909512	0.12819372	0.24258087
ENSG0000013	ZNF141	protein_codir	0.36884009	0.10293739	0.2062135
ENSG0000017	C8G	protein_codir	0.3687539	0.57066884	0.70413091
ENSG0000023	RP1-315G1.3	lncRNA	0.36874553	0.70492345	0.8079838
ENSG0000028	RP5-1090P18.	lncRNA	0.36849611	0.19619483	0.33210156
ENSG0000020	ZBTB48	protein_codir	0.36844708	0.09970771	0.20133988
ENSG0000016	CEP120	protein_codir	0.36804837	0.01305801	0.04278318
ENSG0000026	OVCA2	protein_codir	0.36774946	0.01250612	0.04136755
ENSG0000005	NDC1	protein_codir	0.36769898	0.02934309	0.08010836
ENSG0000024	LINC02005	lncRNA	0.36750146	0.85513922	NA
ENSG0000023	BRWD1-IT1	lncRNA	0.36725428	0.83723157	0.90230748
ENSG0000019	FCHSD1	protein_codir	0.36722502	0.15367016	0.27730104
ENSG0000027	RP11-214K3.2	lncRNA	0.36704668	0.55775269	0.6934562
ENSG0000014	CDKAL1	protein_codir	0.3670459	0.00604981	0.02347397
ENSG0000010	FAM83D	protein_codir	0.36696188	0.55785118	0.693485
ENSG0000006	COL17A1	protein_codir	0.36676124	0.51802218	0.66111139
ENSG0000006	CTDP1	protein_codir	0.36656772	0.01356916	0.04408659
ENSG0000011	ELOVL3	protein_codir	0.36654186	0.58863325	0.7187033
ENSG0000010	NPM3	protein_codir	0.36642669	0.09768155	0.19824854
ENSG0000028	RP11-147I3.4	lncRNA	0.36635567	0.7000866	0.80434242
ENSG0000018	OR7D2	protein_codir	0.3661305	0.74746468	0.83878334
ENSG0000025	RP11-1167A1	lncRNA	0.366003	0.61294603	0.73760758
ENSG0000010	SUFU	protein_codir	0.36597883	0.05259854	0.12502338
ENSG0000012	GJA3	protein_codir	0.36565376	0.58867577	0.7187033
ENSG0000027	RP11-78O7.2	lncRNA	0.36544761	0.28690939	0.43641576
ENSG0000027	RP11-621L6.3	lncRNA	0.36543198	0.83115085	NA
ENSG0000011	NR3C1	protein_codir	0.36532099	0.05073282	0.12188939
ENSG0000026	RP11-678G15	lncRNA	0.36529372	0.83155558	0.89848045
ENSG0000010	CHADL	protein_codir	0.36526839	0.27408755	0.42211989
ENSG0000000	REXO5	protein_codir	0.36517434	0.03626823	0.0940237
ENSG0000027	C2orf49-DT	lncRNA	0.36508064	0.17312672	0.30281552

ENSG0000028	RP1-90J20.15	lncRNA	0.36505021	0.36921104	0.52281215
ENSG0000019	ZNF624	protein_codir	0.3649612	0.19826913	0.3345466
ENSG0000025	RP11-164J13.	protein_codir	0.36493752	0.21792116	0.35833674
ENSG0000013	TUBB2B	protein_codir	0.36483438	0.42685983	0.57867225
ENSG0000019	DDI2	protein_codir	0.3647066	0.00877397	0.03138675
ENSG0000017	GPR150	protein_codir	0.36454136	0.39896528	0.55219325
ENSG0000026	RP11-33B1.4	lncRNA	0.3641816	0.6826366	0.7913327
ENSG0000010	PCK2	protein_codir	0.36410516	0.07566967	0.16399844
ENSG0000027	CTD-2306A12	lncRNA	0.36387112	0.66882604	0.78129653
ENSG0000010	MED25	protein_codir	0.36382221	0.00428202	0.0179703
ENSG0000007	SLC25A43	protein_codir	0.36381018	0.02971052	0.0809115
ENSG0000020	MACO1	protein_codir	0.36380579	0.00044767	0.0029881
ENSG0000010	EP300	protein_codir	0.36378592	0.01385931	0.04479744
ENSG0000024	EFNA4	protein_codir	0.36376373	0.11923958	0.22993766
ENSG0000025	RP11-831H9.1	protein_codir	0.36369705	0.8975427	NA
ENSG0000013	VARS2	protein_codir	0.36359809	0.15835563	0.28349641
ENSG0000027	CTB-58E17.3	lncRNA	0.3632529	0.47982541	0.62761781
ENSG0000027	TAF9	protein_codir	0.36313363	0.00270491	0.01243707
ENSG0000018	C17orf58	protein_codir	0.36310754	0.04571022	0.11246359
ENSG0000018	PPP1CC	protein_codir	0.3630977	0.01030859	0.0356528
ENSG0000014	MALL	protein_codir	0.36285527	0.22425703	0.36592303
ENSG0000018	NLRP10	protein_codir	0.36275608	0.64509828	0.76277149
ENSG0000026	RP11-737O24	lncRNA	0.36272934	0.61475828	0.73885486
ENSG0000013	SLC35D2	protein_codir	0.36256867	0.01291484	0.04241472
ENSG0000027	RP11-324L17.	lncRNA	0.36216998	0.66133227	0.77556035
ENSG0000007	VASH1	protein_codir	0.36208504	0.18169285	0.31369393
ENSG0000022	PPP3R1	protein_codir	0.3618527	0.0031227	0.01395164
ENSG0000014	GTF2H2	protein_codir	0.36149246	0.17521275	0.30532342
ENSG0000010	RNMT	protein_codir	0.36144105	0.01088621	0.03725201
ENSG0000007	SLC25A40	protein_codir	0.36142224	0.0114212	0.03864906
ENSG0000027	CTA-243E7.1	lncRNA	0.3613843	0.81099308	NA
ENSG0000026	RP11-296A16	protein_codir	0.36135993	0.79275714	0.87106465
ENSG0000000	DLX6	protein_codir	0.36124877	0.66228392	0.77621497
ENSG0000026	ZNF407-AS1	lncRNA	0.36123473	0.06525969	0.14747771
ENSG0000014	FUOM	protein_codir	0.36111868	0.12574576	0.23937541
ENSG0000018	H1-10	protein_codir	0.3610817	0.21512196	0.35523547
ENSG0000011	AVPI1	protein_codir	0.36078875	0.23733298	0.38143991
ENSG0000027	GS1-166A23.2	lncRNA	0.3607004	0.66475254	0.77824962
ENSG0000013	MGAT1	protein_codir	0.36056623	0.00587478	0.02295393
ENSG0000022	KANSL1L-AS1	lncRNA	0.36045215	0.41563636	0.56857464
ENSG0000016	SLC49A3	protein_codir	0.36044219	0.17914756	0.31033966
ENSG0000028	LINC00891	lncRNA	0.36042473	0.36101841	0.51491497
ENSG0000026	CTD-2639E6.9	lncRNA	0.36039073	0.73467429	0.82975686
ENSG0000023	FAM138A	lncRNA	0.36021591	0.91209513	NA
ENSG0000023	RP5-857K21.2	lncRNA	0.36021591	0.91209513	NA
ENSG0000022	RP11-206L10.	lncRNA	0.36021591	0.91209513	NA
ENSG0000028	RP5-907A6.1	lncRNA	0.36021591	0.91209513	NA

ENSG0000016	CCDC27	protein_codir	0.36021591	0.91209513	NA
ENSG0000022	RP11-84A14.5	lncRNA	0.36021591	0.91209513	NA
ENSG0000028	RP5-1077B9.6	lncRNA	0.36021591	0.91209513	NA
ENSG0000027	HNRNPCL2	protein_codir	0.36021591	0.91209513	NA
ENSG0000028	RP1-37C10.7	lncRNA	0.36021591	0.91209513	NA
ENSG0000014	PADI3	protein_codir	0.36021591	0.91209513	NA
ENSG0000024	RP1-184J9.2	lncRNA	0.36021591	0.91209513	NA
ENSG0000028	C1orf232	protein_codir	0.36021591	0.91209513	NA
ENSG0000022	RP1-50O24.6	lncRNA	0.36021591	0.91209513	NA
ENSG0000022	RP11-460I13.2	lncRNA	0.36021591	0.91209513	NA
ENSG0000025	RP11-84A19.3	lncRNA	0.36021591	0.91209513	NA
ENSG0000028	RP11-566C13.	lncRNA	0.36021591	0.91209513	NA
ENSG0000013	GJA9	protein_codir	0.36021591	0.91209513	NA
ENSG0000022	RP1-144F13.3	lncRNA	0.36021591	0.91209513	NA
ENSG0000023	LINC02784	lncRNA	0.36021591	0.91209513	NA
ENSG0000023	RP11-67L3.2	lncRNA	0.36021591	0.91209513	NA
ENSG0000023	NFIA-AS1	lncRNA	0.36021591	0.91209513	NA
ENSG0000028	RP11-430G17	lncRNA	0.36021591	0.91209513	NA
ENSG0000013	ANGPTL3	protein_codir	0.36021591	0.91209513	NA
ENSG0000023	RP4-694A7.2	lncRNA	0.36021591	0.91209513	NA
ENSG0000022	HHLA3-AS1	lncRNA	0.36021591	0.91209513	NA
ENSG0000026	RP11-510C10.	lncRNA	0.36021591	0.91209513	NA
ENSG0000028	RP11-386I14.5	lncRNA	0.36021591	0.91209513	NA
ENSG0000028	RP11-118B23.	lncRNA	0.36021591	0.91209513	NA
ENSG0000027	RP4-665J23.4	lncRNA	0.36021591	0.91209513	NA
ENSG0000028	RP4-672J20.3	lncRNA	0.36021591	0.91209513	NA
ENSG0000023	RP5-896L10.1	lncRNA	0.36021591	0.91209513	NA
ENSG0000022	RP5-837M10.	lncRNA	0.36021591	0.91209513	NA
ENSG0000022	LINC01709	lncRNA	0.36021591	0.91209513	NA
ENSG0000017	AMY1B	protein_codir	0.36021591	0.91209513	NA
ENSG0000018	AMY1C	protein_codir	0.36021591	0.91209513	NA
ENSG0000025	RP5-1160K1.8	lncRNA	0.36021591	0.91209513	NA
ENSG0000014	KCNA10	protein_codir	0.36021591	0.91209513	NA
ENSG0000027	LINC01632	lncRNA	0.36021591	0.91209513	NA
ENSG0000023	LINC02806	lncRNA	0.36021591	0.91209513	NA
ENSG0000027	RP4-790G17.7	lncRNA	0.36021591	0.91209513	NA
ENSG0000022	LINC01527	lncRNA	0.36021591	0.91209513	NA
ENSG0000016	SPRR3	protein_codir	0.36021591	0.91209513	NA
ENSG0000016	SPRR1B	protein_codir	0.36021591	0.91209513	NA
ENSG0000022	UBE2Q1-AS1	lncRNA	0.36021591	0.91209513	NA
ENSG0000022	RP11-565P22.	lncRNA	0.36021591	0.91209513	NA
ENSG0000026	RP3-518E13.2	lncRNA	0.36021591	0.91209513	NA
ENSG0000022	RP11-12M5.3	lncRNA	0.36021591	0.91209513	NA
ENSG0000023	LINC01351	lncRNA	0.36021591	0.91209513	NA
ENSG0000022	LINC02770	lncRNA	0.36021591	0.91209513	NA
ENSG0000023	RP11-203F10.	lncRNA	0.36021591	0.91209513	NA
ENSG0000028	RP11-212H11	lncRNA	0.36021591	0.91209513	NA

ENSG0000023	RP11-78B10.2	lncRNA	0.36021591	0.91209513	NA
ENSG0000023	RP11-385M4.	lncRNA	0.36021591	0.91209513	NA
ENSG0000028	RP11-318L16.	lncRNA	0.36021591	0.91209513	NA
ENSG0000023	RP11-122M14	lncRNA	0.36021591	0.91209513	NA
ENSG0000022	RP11-176D17	lncRNA	0.36021591	0.91209513	NA
ENSG0000022	LINC02869	lncRNA	0.36021591	0.91209513	NA
ENSG0000022	LINC02474	lncRNA	0.36021591	0.91209513	NA
ENSG0000028	RP11-3L21.3	lncRNA	0.36021591	0.91209513	NA
ENSG0000022	ITPKB-AS1	lncRNA	0.36021591	0.91209513	NA
ENSG0000022	RP4-613A2.1	lncRNA	0.36021591	0.91209513	NA
ENSG0000022	RP5-858B6.1	lncRNA	0.36021591	0.91209513	NA
ENSG0000028	RP5-855F14.3	lncRNA	0.36021591	0.91209513	NA
ENSG0000022	RP4-781K5.4	lncRNA	0.36021591	0.91209513	NA
ENSG0000028	RP4-781K5.7	lncRNA	0.36021591	0.91209513	NA
ENSG0000022	RP11-261C10.	lncRNA	0.36021591	0.91209513	NA
ENSG0000022	RP11-278H7.4	lncRNA	0.36021591	0.91209513	NA
ENSG0000028	RP11-278H7.6	lncRNA	0.36021591	0.91209513	NA
ENSG0000027	OR2L8	protein_codir	0.36021591	0.91209513	NA
ENSG0000019	OR2T6	protein_codir	0.36021591	0.91209513	NA
ENSG0000019	OR2T2	protein_codir	0.36021591	0.91209513	NA
ENSG0000023	AC113607.3	lncRNA	0.36021591	0.91209513	NA
ENSG0000023	AC092687.5	lncRNA	0.36021591	0.91209513	NA
ENSG0000023	AC008278.2	lncRNA	0.36021591	0.91209513	NA
ENSG0000021	LINC01913	lncRNA	0.36021591	0.91209513	NA
ENSG0000025	RHOQ-AS1	lncRNA	0.36021591	0.91209513	NA
ENSG0000027	RP11-15I20.1	lncRNA	0.36021591	0.91209513	NA
ENSG0000027	RP11-310N16	protein_codir	0.36021591	0.91209513	NA
ENSG0000023	AC093110.3	lncRNA	0.36021591	0.91209513	NA
ENSG0000016	GKN1	protein_codir	0.36021591	0.91209513	NA
ENSG0000028	RP11-467P9.2	lncRNA	0.36021591	0.91209513	NA
ENSG0000027	RP11-466M21	lncRNA	0.36021591	0.91209513	NA
ENSG0000014	NAT8	protein_codir	0.36021591	0.91209513	NA
ENSG0000028	RP11-372J12.	lncRNA	0.36021591	0.91209513	NA
ENSG0000028	RP11-372J12.	lncRNA	0.36021591	0.91209513	NA
ENSG0000022	CTNNA2-AS1	lncRNA	0.36021591	0.91209513	NA
ENSG0000011	SMYD1	protein_codir	0.36021591	0.91209513	NA
ENSG0000022	AC092675.3	lncRNA	0.36021591	0.91209513	NA
ENSG0000023	LINC01104	lncRNA	0.36021591	0.91209513	NA
ENSG0000018	SLC9A4	protein_codir	0.36021591	0.91209513	NA
ENSG0000028	RP11-437E9.1	lncRNA	0.36021591	0.91209513	NA
ENSG0000023	RP11-448J14.	lncRNA	0.36021591	0.91209513	NA
ENSG0000022	LINC01594	lncRNA	0.36021591	0.91209513	NA
ENSG0000013	IL36G	protein_codir	0.36021591	0.91209513	NA
ENSG0000028	RP11-505O20	lncRNA	0.36021591	0.91209513	NA
ENSG0000026	RP11-521O16	lncRNA	0.36021591	0.91209513	NA
ENSG0000020	LINC01854	lncRNA	0.36021591	0.91209513	NA
ENSG0000023	AC133785.1	lncRNA	0.36021591	0.91209513	NA

ENSG0000022	LINC01120	lncRNA	0.36021591	0.91209513	NA
ENSG0000023	NCKAP5-IT1	lncRNA	0.36021591	0.91209513	NA
ENSG0000023	AC023128.1	lncRNA	0.36021591	0.91209513	NA
ENSG0000023	LINC01817	lncRNA	0.36021591	0.91209513	NA
ENSG0000015	G6PC2	protein_codir	0.36021591	0.91209513	NA
ENSG0000023	AC073834.3	lncRNA	0.36021591	0.91209513	NA
ENSG0000022	AC020595.1	lncRNA	0.36021591	0.91209513	NA
ENSG0000028	RP11-315C24.	lncRNA	0.36021591	0.91209513	NA
ENSG0000026	RP11-764E7.1	lncRNA	0.36021591	0.91209513	NA
ENSG0000022	LINC01821	lncRNA	0.36021591	0.91209513	NA
ENSG0000022	AC010746.3	lncRNA	0.36021591	0.91209513	NA
ENSG0000028	RP11-294E24.	lncRNA	0.36021591	0.91209513	NA
ENSG0000023	LINC01792	lncRNA	0.36021591	0.91209513	NA
ENSG0000027	GPR1-AS	lncRNA	0.36021591	0.91209513	NA
ENSG0000023	DYTN	protein_codir	0.36021591	0.91209513	NA
ENSG0000028	RP11-309L6.1	lncRNA	0.36021591	0.91209513	NA
ENSG0000011	CRYGD	protein_codir	0.36021591	0.91209513	NA
ENSG0000028	RP11-475H17	lncRNA	0.36021591	0.91209513	NA
ENSG0000028	RP11-421A16	lncRNA	0.36021591	0.91209513	NA
ENSG0000022	AC012462.3	lncRNA	0.36021591	0.91209513	NA
ENSG0000023	SMARCAL1-AS	lncRNA	0.36021591	0.91209513	NA
ENSG0000022	AC073641.2	lncRNA	0.36021591	0.91209513	NA
ENSG0000023	AC068138.1	lncRNA	0.36021591	0.91209513	NA
ENSG0000022	AC068134.8	lncRNA	0.36021591	0.91209513	NA
ENSG0000019	CHRNG	protein_codir	0.36021591	0.91209513	NA
ENSG0000024	UGT1A8	protein_codir	0.36021591	0.91209513	NA
ENSG0000023	AC079135.1	lncRNA	0.36021591	0.91209513	NA
ENSG0000022	AC107079.1	lncRNA	0.36021591	0.91209513	NA
ENSG0000028	RP11-275G7.3	lncRNA	0.36021591	0.91209513	NA
ENSG0000018	OR6B2	protein_codir	0.36021591	0.91209513	NA
ENSG0000022	AC104809.2	lncRNA	0.36021591	0.91209513	NA
ENSG0000028	RP11-475G3.1	lncRNA	0.36021591	0.91209513	NA
ENSG0000022	AC027119.1	lncRNA	0.36021591	0.91209513	NA
ENSG0000028	RP11-109J15.	lncRNA	0.36021591	0.91209513	NA
ENSG0000026	LINC02011	lncRNA	0.36021591	0.91209513	NA
ENSG0000023	LINC01967	lncRNA	0.36021591	0.91209513	NA
ENSG0000028	RP11-331G2.1	lncRNA	0.36021591	0.91209513	NA
ENSG0000028	RP11-333B11.	lncRNA	0.36021591	0.91209513	NA
ENSG0000017	CCDC13-AS1	lncRNA	0.36021591	0.91209513	NA
ENSG0000017	TOPAZ1	protein_codir	0.36021591	0.91209513	NA
ENSG0000028	LINC01988	lncRNA	0.36021591	0.91209513	NA
ENSG0000026	LRRC2-AS1	lncRNA	0.36021591	0.91209513	NA
ENSG0000027	RP11-884K10.	lncRNA	0.36021591	0.91209513	NA
ENSG0000028	RP11-122D19	lncRNA	0.36021591	0.91209513	NA
ENSG0000024	RP11-875H7.5	lncRNA	0.36021591	0.91209513	NA
ENSG0000024	RP11-85I21.1	lncRNA	0.36021591	0.91209513	NA
ENSG0000028	RP11-81D17.1	lncRNA	0.36021591	0.91209513	NA

ENSG0000023	OR5K2	protein_codir	0.36021591	0.91209513	NA
ENSG0000024	RP11-227H4.5	lncRNA	0.36021591	0.91209513	NA
ENSG0000028	RP11-93B21.2	lncRNA	0.36021591	0.91209513	NA
ENSG0000021	LINC00488	lncRNA	0.36021591	0.91209513	NA
ENSG0000024	RP11-271C24.	lncRNA	0.36021591	0.91209513	NA
ENSG0000028	RP11-416B23.	lncRNA	0.36021591	0.91209513	NA
ENSG0000016	TEX55	protein_codir	0.36021591	0.91209513	NA
ENSG0000028	RP11-10G15.5	lncRNA	0.36021591	0.91209513	NA
ENSG0000025	MYLK-AS2	lncRNA	0.36021591	0.91209513	NA
ENSG0000024	CCDC37-DT	lncRNA	0.36021591	0.91209513	NA
ENSG0000028	RP11-305F5.3	lncRNA	0.36021591	0.91209513	NA
ENSG0000028	RP11-491D10	lncRNA	0.36021591	0.91209513	NA
ENSG0000019	LINC01565	lncRNA	0.36021591	0.91209513	NA
ENSG0000011	RBP2	protein_codir	0.36021591	0.91209513	NA
ENSG0000024	RP11-319G6.3	lncRNA	0.36021591	0.91209513	NA
ENSG0000026	RP11-442N1.2	lncRNA	0.36021591	0.91209513	NA
ENSG0000024	LINC02032	lncRNA	0.36021591	0.91209513	NA
ENSG0000024	LINC02046	lncRNA	0.36021591	0.91209513	NA
ENSG0000028	RP11-455G2.1	lncRNA	0.36021591	0.91209513	NA
ENSG0000028	RP11-206M11	lncRNA	0.36021591	0.91209513	NA
ENSG0000026	RP11-166N6.3	protein_codir	0.36021591	0.91209513	NA
ENSG0000024	RP11-166N6.2	lncRNA	0.36021591	0.91209513	NA
ENSG0000016	CLRN1	protein_codir	0.36021591	0.91209513	NA
ENSG0000028	RP11-25K24.3	lncRNA	0.36021591	0.91209513	NA
ENSG0000024	STRIT1	protein_codir	0.36021591	0.91209513	NA
ENSG0000024	RP11-12K11.2	lncRNA	0.36021591	0.91209513	NA
ENSG0000023	RP11-543D10	lncRNA	0.36021591	0.91209513	NA
ENSG0000028	RP11-292L5.1	lncRNA	0.36021591	0.91209513	NA
ENSG0000023	RP11-496B10.	lncRNA	0.36021591	0.91209513	NA
ENSG0000028	RP11-329B9.6	lncRNA	0.36021591	0.91209513	NA
ENSG0000011	KNG1	protein_codir	0.36021591	0.91209513	NA
ENSG0000023	LINC02037	lncRNA	0.36021591	0.91209513	NA
ENSG0000022	AC128709.4	lncRNA	0.36021591	0.91209513	NA
ENSG0000023	AC128709.2	lncRNA	0.36021591	0.91209513	NA
ENSG0000023	AC055764.1	lncRNA	0.36021591	0.91209513	NA
ENSG0000025	RP11-460I19.1	lncRNA	0.36021591	0.91209513	NA
ENSG0000016	OTOP1	protein_codir	0.36021591	0.91209513	NA
ENSG0000008	LINC01587	lncRNA	0.36021591	0.91209513	NA
ENSG0000024	LINC02360	lncRNA	0.36021591	0.91209513	NA
ENSG0000025	LINC02270	lncRNA	0.36021591	0.91209513	NA
ENSG0000028	KCNIP4-IT1	lncRNA	0.36021591	0.91209513	NA
ENSG0000025	RP11-380P13.	lncRNA	0.36021591	0.91209513	NA
ENSG0000028	RP11-20M7.1	lncRNA	0.36021591	0.91209513	NA
ENSG0000025	PHOX2B-AS1	lncRNA	0.36021591	0.91209513	NA
ENSG0000025	LINC02475	lncRNA	0.36021591	0.91209513	NA
ENSG0000028	RP11-731J8.3	lncRNA	0.36021591	0.91209513	NA
ENSG0000024	RP11-752D24	lncRNA	0.36021591	0.91209513	NA

ENSG0000016	PDCL2	protein_codir	0.36021591	0.91209513	NA
ENSG0000024	RP11-725D20	lncRNA	0.36021591	0.91209513	NA
ENSG0000025	RP11-12K22.1	lncRNA	0.36021591	0.91209513	NA
ENSG0000024	RP11-807H7.2	lncRNA	0.36021591	0.91209513	NA
ENSG0000013	UGT2A3	protein_codir	0.36021591	0.91209513	NA
ENSG0000025	RP11-542G1.1	lncRNA	0.36021591	0.91209513	NA
ENSG0000025	RP11-297P16.	lncRNA	0.36021591	0.91209513	NA
ENSG0000016	TACR3	protein_codir	0.36021591	0.91209513	NA
ENSG0000024	COL25A1-DT	lncRNA	0.36021591	0.91209513	NA
ENSG0000025	RP11-380D23	lncRNA	0.36021591	0.91209513	NA
ENSG0000024	RP11-255I10.1	lncRNA	0.36021591	0.91209513	NA
ENSG0000024	RP11-269F21.	lncRNA	0.36021591	0.91209513	NA
ENSG0000028	AC004704.2	lncRNA	0.36021591	0.91209513	NA
ENSG0000028	RP11-97F8.3	lncRNA	0.36021591	0.91209513	NA
ENSG0000027	RP11-130C6.1	lncRNA	0.36021591	0.91209513	NA
ENSG0000028	JADRR	lncRNA	0.36021591	0.91209513	NA
ENSG0000025	RP11-371F15.	lncRNA	0.36021591	0.91209513	NA
ENSG0000028	RP11-301H24	lncRNA	0.36021591	0.91209513	NA
ENSG0000028	CTD-2172F7.1	lncRNA	0.36021591	0.91209513	NA
ENSG0000017	FGB	protein_codir	0.36021591	0.91209513	NA
ENSG0000025	ASIC5	protein_codir	0.36021591	0.91209513	NA
ENSG0000028	RP11-364P22.	lncRNA	0.36021591	0.91209513	NA
ENSG0000025	RP11-138A23	lncRNA	0.36021591	0.91209513	NA
ENSG0000025	LINC02233	lncRNA	0.36021591	0.91209513	NA
ENSG0000024	LINC02477	lncRNA	0.36021591	0.91209513	NA
ENSG0000028	RP11-366M4.	lncRNA	0.36021591	0.91209513	NA
ENSG0000010	ANXA10	protein_codir	0.36021591	0.91209513	NA
ENSG0000028	RP11-613O14	lncRNA	0.36021591	0.91209513	NA
ENSG0000023	LINC01098	lncRNA	0.36021591	0.91209513	NA
ENSG0000024	LINC00290	lncRNA	0.36021591	0.91209513	NA
ENSG0000027	RP11-335L23.	lncRNA	0.36021591	0.91209513	NA
ENSG0000024	LINC02437	lncRNA	0.36021591	0.91209513	NA
ENSG0000017	ZFP42	protein_codir	0.36021591	0.91209513	NA
ENSG0000024	LINC02434	lncRNA	0.36021591	0.91209513	NA
ENSG0000024	CTD-2383I20.	lncRNA	0.36021591	0.91209513	NA
ENSG0000025	CTD-2072I24.	lncRNA	0.36021591	0.91209513	NA
ENSG0000028	CTB-40H15.5	lncRNA	0.36021591	0.91209513	NA
ENSG0000025	RP11-19O2.2	lncRNA	0.36021591	0.91209513	NA
ENSG0000024	RP11-5N11.2	lncRNA	0.36021591	0.91209513	NA
ENSG0000014	UGT3A1	protein_codir	0.36021591	0.91209513	NA
ENSG0000024	EGFLAM-AS4	lncRNA	0.36021591	0.91209513	NA
ENSG0000024	EGFLAM-AS1	lncRNA	0.36021591	0.91209513	NA
ENSG0000025	LINC00604	lncRNA	0.36021591	0.91209513	NA
ENSG0000028	RP11-386E5.1	lncRNA	0.36021591	0.91209513	NA
ENSG0000024	RP11-159F24.	lncRNA	0.36021591	0.91209513	NA
ENSG0000024	RP11-2O17.2	lncRNA	0.36021591	0.91209513	NA
ENSG0000028	RP11-95I19.4	protein_codir	0.36021591	0.91209513	NA

ENSG0000025	CTC-498J12.1	lncRNA	0.36021591	0.91209513	NA
ENSG0000025	LINC01385	lncRNA	0.36021591	0.91209513	NA
ENSG0000024	LINC01333	lncRNA	0.36021591	0.91209513	NA
ENSG0000024	CTC-366B18.2	lncRNA	0.36021591	0.91209513	NA
ENSG0000025	RP11-107N7.1	lncRNA	0.36021591	0.91209513	NA
ENSG0000025	LINC02059	lncRNA	0.36021591	0.91209513	NA
ENSG0000025	RP11-61G23.2	lncRNA	0.36021591	0.91209513	NA
ENSG0000025	CTD-2091N23	lncRNA	0.36021591	0.91209513	NA
ENSG0000024	CTC-506B8.1	lncRNA	0.36021591	0.91209513	NA
ENSG0000025	LINC02163	lncRNA	0.36021591	0.91209513	NA
ENSG0000024	ZNF474-AS1	lncRNA	0.36021591	0.91209513	NA
ENSG0000019	TEX43	protein_codir	0.36021591	0.91209513	NA
ENSG0000025	CTC-228N24.2	lncRNA	0.36021591	0.91209513	NA
ENSG0000016	IL3	protein_codir	0.36021591	0.91209513	NA
ENSG0000027	AC004775.5	lncRNA	0.36021591	0.91209513	NA
ENSG0000027	RP11-381K20.	lncRNA	0.36021591	0.91209513	NA
ENSG0000027	AC005609.19	lncRNA	0.36021591	0.91209513	NA
ENSG0000028	AC005592.4	lncRNA	0.36021591	0.91209513	NA
ENSG0000025	CTD-2050E21.	lncRNA	0.36021591	0.91209513	NA
ENSG0000028	CTB-107A22.1	lncRNA	0.36021591	0.91209513	NA
ENSG0000024	MARCOL	protein_codir	0.36021591	0.91209513	NA
ENSG0000025	AC012613.2	lncRNA	0.36021591	0.91209513	NA
ENSG0000025	GRPEL2-AS1	lncRNA	0.36021591	0.91209513	NA
ENSG0000025	CTC-345K18.2	lncRNA	0.36021591	0.91209513	NA
ENSG0000025	CTC-348L5.1	lncRNA	0.36021591	0.91209513	NA
ENSG0000025	CTC-529G1.1	lncRNA	0.36021591	0.91209513	NA
ENSG0000025	CTB-78F1.2	lncRNA	0.36021591	0.91209513	NA
ENSG0000028	CTB-114C7.5	lncRNA	0.36021591	0.91209513	NA
ENSG0000021	EFCAB9	protein_codir	0.36021591	0.91209513	NA
ENSG0000025	RP11-779O18	lncRNA	0.36021591	0.91209513	NA
ENSG0000024	CTC-430J12.2	lncRNA	0.36021591	0.91209513	NA
ENSG0000024	RP11-826N14	lncRNA	0.36021591	0.91209513	NA
ENSG0000025	RP11-843P14.	lncRNA	0.36021591	0.91209513	NA
ENSG0000025	RP11-843P14.	lncRNA	0.36021591	0.91209513	NA
ENSG0000024	LINC01574	lncRNA	0.36021591	0.91209513	NA
ENSG0000024	RP11-1026M7	lncRNA	0.36021591	0.91209513	NA
ENSG0000025	RP11-798K23.	lncRNA	0.36021591	0.91209513	NA
ENSG0000024	CTC-338M12.	lncRNA	0.36021591	0.91209513	NA
ENSG0000026	RP3-416J7.4	lncRNA	0.36021591	0.91209513	NA
ENSG0000027	RP5-856G1.1	lncRNA	0.36021591	0.91209513	NA
ENSG0000023	RP3-406P24.3	lncRNA	0.36021591	0.91209513	NA
ENSG0000028	RP11-274H24	lncRNA	0.36021591	0.91209513	NA
ENSG0000028	RP1-258E1.2	lncRNA	0.36021591	0.91209513	NA
ENSG0000023	STMND1	protein_codir	0.36021591	0.91209513	NA
ENSG0000028	CTA-14H9.7	lncRNA	0.36021591	0.91209513	NA
ENSG0000028	RP1-29K1.7	lncRNA	0.36021591	0.91209513	NA
ENSG0000020	OR2H2	protein_codir	0.36021591	0.91209513	NA

ENSG0000023	DDR1-DT	lncRNA	0.36021591	0.91209513	NA
ENSG0000027	XXbac-BPG29	lncRNA	0.36021591	0.91209513	NA
ENSG0000026	RP11-298J23.	lncRNA	0.36021591	0.91209513	NA
ENSG0000020	POLH-AS1	lncRNA	0.36021591	0.91209513	NA
ENSG0000027	RP11-444E17.	protein_codir	0.36021591	0.91209513	NA
ENSG0000015	ADGRF1	protein_codir	0.36021591	0.91209513	NA
ENSG0000012	OPN5	protein_codir	0.36021591	0.91209513	NA
ENSG0000017	GSTA3	protein_codir	0.36021591	0.91209513	NA
ENSG0000013	HCRTR2	protein_codir	0.36021591	0.91209513	NA
ENSG0000026	RP1-71H19.2	lncRNA	0.36021591	0.91209513	NA
ENSG0000025	RP1-149L1.1	lncRNA	0.36021591	0.91209513	NA
ENSG0000023	RP11-415D17	lncRNA	0.36021591	0.91209513	NA
ENSG0000023	LINC02540	lncRNA	0.36021591	0.91209513	NA
ENSG0000028	RP3-357D13.5	lncRNA	0.36021591	0.91209513	NA
ENSG0000028	RP3-492P14.3	lncRNA	0.36021591	0.91209513	NA
ENSG0000022	TBX18-AS1	lncRNA	0.36021591	0.91209513	NA
ENSG0000016	HTR1E	protein_codir	0.36021591	0.91209513	NA
ENSG0000022	CASC6	lncRNA	0.36021591	0.91209513	NA
ENSG0000028	RP11-596A13	lncRNA	0.36021591	0.91209513	NA
ENSG0000028	RP1-84D21.2	lncRNA	0.36021591	0.91209513	NA
ENSG0000028	RP1-273N12.4	lncRNA	0.36021591	0.91209513	NA
ENSG0000018	LIN28B	protein_codir	0.36021591	0.91209513	NA
ENSG0000018	RFX6	protein_codir	0.36021591	0.91209513	NA
ENSG0000017	VGLL2	protein_codir	0.36021591	0.91209513	NA
ENSG0000023	TRDN-AS1	lncRNA	0.36021591	0.91209513	NA
ENSG0000022	RP11-102N11	lncRNA	0.36021591	0.91209513	NA
ENSG0000023	LINC00326	lncRNA	0.36021591	0.91209513	NA
ENSG0000023	RP1-283K11.2	lncRNA	0.36021591	0.91209513	NA
ENSG0000023	RP1-287H17.1	lncRNA	0.36021591	0.91209513	NA
ENSG0000023	MYB-AS1	lncRNA	0.36021591	0.91209513	NA
ENSG0000028	RP3-388E23.3	lncRNA	0.36021591	0.91209513	NA
ENSG0000023	RP1-38C16.2	lncRNA	0.36021591	0.91209513	NA
ENSG0000028	RP11-371H1.1	lncRNA	0.36021591	0.91209513	NA
ENSG0000028	RP11-10J5.2	lncRNA	0.36021591	0.91209513	NA
ENSG0000028	RP3-468K18.7	protein_codir	0.36021591	0.91209513	NA
ENSG0000028	RP11-545I5.5	lncRNA	0.36021591	0.91209513	NA
ENSG0000023	UST-AS2	lncRNA	0.36021591	0.91209513	NA
ENSG0000015	RAET1L	protein_codir	0.36021591	0.91209513	NA
ENSG0000028	RP11-732M18	lncRNA	0.36021591	0.91209513	NA
ENSG0000023	RP1-249F5.3	lncRNA	0.36021591	0.91209513	NA
ENSG0000023	RP11-235G24	lncRNA	0.36021591	0.91209513	NA
ENSG0000028	RP3-495O10.4	lncRNA	0.36021591	0.91209513	NA
ENSG0000023	RP11-252P19.	lncRNA	0.36021591	0.91209513	NA
ENSG0000023	RP4-655C5.4	lncRNA	0.36021591	0.91209513	NA
ENSG0000028	RP3-431P23.6	lncRNA	0.36021591	0.91209513	NA
ENSG0000023	RP11-503C24.	lncRNA	0.36021591	0.91209513	NA
ENSG0000023	RP5-1086L22.	lncRNA	0.36021591	0.91209513	NA

ENSG0000022	AC073957.15	lncRNA	0.36021591	0.91209513	NA
ENSG0000022	AC091729.7	lncRNA	0.36021591	0.91209513	NA
ENSG0000023	AC004895.4	lncRNA	0.36021591	0.91209513	NA
ENSG0000022	PRPS1L1	protein_codir	0.36021591	0.91209513	NA
ENSG0000022	AC007091.1	lncRNA	0.36021591	0.91209513	NA
ENSG0000026	RP11-486P11.	lncRNA	0.36021591	0.91209513	NA
ENSG0000019	NPSR1-AS1	lncRNA	0.36021591	0.91209513	NA
ENSG0000028	RP11-23B6.1	lncRNA	0.36021591	0.91209513	NA
ENSG0000023	AC083864.3	lncRNA	0.36021591	0.91209513	NA
ENSG0000028	RP11-111G20	lncRNA	0.36021591	0.91209513	NA
ENSG0000004	ZPBP	protein_codir	0.36021591	0.91209513	NA
ENSG0000028	RP5-847G17.1	lncRNA	0.36021591	0.91209513	NA
ENSG0000028	GS1-281N8.1	lncRNA	0.36021591	0.91209513	NA
ENSG0000022	VSTM2A-OT1	lncRNA	0.36021591	0.91209513	NA
ENSG0000028	RP11-328P23.	lncRNA	0.36021591	0.91209513	NA
ENSG0000022	RP11-3P22.2	lncRNA	0.36021591	0.91209513	NA
ENSG0000023	AC083884.8	lncRNA	0.36021591	0.91209513	NA
ENSG0000028	SPDYE13	protein_codir	0.36021591	0.91209513	NA
ENSG0000028	SPDYE14	protein_codir	0.36021591	0.91209513	NA
ENSG0000028	SPDYE15	protein_codir	0.36021591	0.91209513	NA
ENSG0000023	AC074183.4	lncRNA	0.36021591	0.91209513	NA
ENSG0000024	ERVW-1	protein_codir	0.36021591	0.91209513	NA
ENSG0000023	TFPI2-DT	lncRNA	0.36021591	0.91209513	NA
ENSG0000023	BET1-AS1	lncRNA	0.36021591	0.91209513	NA
ENSG0000028	CTB-161A2.8	lncRNA	0.36021591	0.91209513	NA
ENSG0000014	ZAN	protein_codir	0.36021591	0.91209513	NA
ENSG0000017	SPDYE2B	protein_codir	0.36021591	0.91209513	NA
ENSG0000009	SLC26A3	protein_codir	0.36021591	0.91209513	NA
ENSG0000020	C7orf66	lncRNA	0.36021591	0.91209513	NA
ENSG0000015	PPP1R3A	protein_codir	0.36021591	0.91209513	NA
ENSG0000024	RP11-264K23.	lncRNA	0.36021591	0.91209513	NA
ENSG0000024	RP5-921G16.2	lncRNA	0.36021591	0.91209513	NA
ENSG0000028	AC002057.2	lncRNA	0.36021591	0.91209513	NA
ENSG0000025	TAS2R38	protein_codir	0.36021591	0.91209513	NA
ENSG0000027	MTRNR2L6	protein_codir	0.36021591	0.91209513	NA
ENSG0000022	OR2A5	protein_codir	0.36021591	0.91209513	NA
ENSG0000010	NOBOX	protein_codir	0.36021591	0.91209513	NA
ENSG0000028	RP4-622E21.1	lncRNA	0.36021591	0.91209513	NA
ENSG0000025	AC144568.2	lncRNA	0.36021591	0.91209513	NA
ENSG0000028	AF067845.3	lncRNA	0.36021591	0.91209513	NA
ENSG0000025	AC133633.1	lncRNA	0.36021591	0.91209513	NA
ENSG0000028	RP11-16G12.1	lncRNA	0.36021591	0.91209513	NA
ENSG0000024	GS1-24F4.2	lncRNA	0.36021591	0.91209513	NA
ENSG0000016	DEFA6	protein_codir	0.36021591	0.91209513	NA
ENSG0000017	LINC00208	lncRNA	0.36021591	0.91209513	NA
ENSG0000021	ZNF705D	protein_codir	0.36021591	0.91209513	NA
ENSG0000025	RP11-13N12.2	lncRNA	0.36021591	0.91209513	NA

ENSG0000025	RP11-1080G1	lncRNA	0.36021591	0.91209513	NA
ENSG0000025	RP11-1105O1	lncRNA	0.36021591	0.91209513	NA
ENSG0000025	AC100802.3	lncRNA	0.36021591	0.91209513	NA
ENSG0000006	ADAM7	protein_codir	0.36021591	0.91209513	NA
ENSG0000028	RP11-219J21.	lncRNA	0.36021591	0.91209513	NA
ENSG0000025	INTS9-AS1	lncRNA	0.36021591	0.91209513	NA
ENSG0000026	RP4-676L2.1	lncRNA	0.36021591	0.91209513	NA
ENSG0000025	RP11-486M23	lncRNA	0.36021591	0.91209513	NA
ENSG0000028	RP11-317J8.1	protein_codir	0.36021591	0.91209513	NA
ENSG0000025	RP11-431M3.	lncRNA	0.36021591	0.91209513	NA
ENSG0000025	RP11-346L1.2	lncRNA	0.36021591	0.91209513	NA
ENSG0000028	RP11-113H14	lncRNA	0.36021591	0.91209513	NA
ENSG0000025	RP11-162D9.3	lncRNA	0.36021591	0.91209513	NA
ENSG0000025	CERNA3	lncRNA	0.36021591	0.91209513	NA
ENSG0000025	RP11-91I20.1	lncRNA	0.36021591	0.91209513	NA
ENSG0000028	RP11-26L21.1	lncRNA	0.36021591	0.91209513	NA
ENSG0000027	RP11-706J10.	lncRNA	0.36021591	0.91209513	NA
ENSG0000019	FABP12	protein_codir	0.36021591	0.91209513	NA
ENSG0000025	RP11-386D6.2	lncRNA	0.36021591	0.91209513	NA
ENSG0000025	RP11-90D11.1	lncRNA	0.36021591	0.91209513	NA
ENSG0000027	KB-1958F4.2	lncRNA	0.36021591	0.91209513	NA
ENSG0000022	RP11-44N12.2	lncRNA	0.36021591	0.91209513	NA
ENSG0000025	RP11-402L5.1	lncRNA	0.36021591	0.91209513	NA
ENSG0000015	ODF1	protein_codir	0.36021591	0.91209513	NA
ENSG0000025	RP11-174I12.1	lncRNA	0.36021591	0.91209513	NA
ENSG0000025	RP11-103H7.3	lncRNA	0.36021591	0.91209513	NA
ENSG0000028	RP11-382A18	lncRNA	0.36021591	0.91209513	NA
ENSG0000025	RP11-737F9.2	lncRNA	0.36021591	0.91209513	NA
ENSG0000013	HHLA1	protein_codir	0.36021591	0.91209513	NA
ENSG0000028	CTC-215C12.2	lncRNA	0.36021591	0.91209513	NA
ENSG0000024	ZFAT-AS1	lncRNA	0.36021591	0.91209513	NA
ENSG0000025	RP11-343P9.1	lncRNA	0.36021591	0.91209513	NA
ENSG0000025	LINC01300	lncRNA	0.36021591	0.91209513	NA
ENSG0000025	RP11-706C16.	lncRNA	0.36021591	0.91209513	NA
ENSG0000016	CYP11B1	protein_codir	0.36021591	0.91209513	NA
ENSG0000026	LY6L	protein_codir	0.36021591	0.91209513	NA
ENSG0000025	RP11-299M14	lncRNA	0.36021591	0.91209513	NA
ENSG0000012	MLANA	protein_codir	0.36021591	0.91209513	NA
ENSG0000028	RP11-513M16	lncRNA	0.36021591	0.91209513	NA
ENSG0000012	IFNA6	protein_codir	0.36021591	0.91209513	NA
ENSG0000018	IFNA2	protein_codir	0.36021591	0.91209513	NA
ENSG0000022	CYP4F26P	lncRNA	0.36021591	0.91209513	NA
ENSG0000017	C9orf131	protein_codir	0.36021591	0.91209513	NA
ENSG0000028	RP11-344B23.	lncRNA	0.36021591	0.91209513	NA
ENSG0000022	RP11-113O24	lncRNA	0.36021591	0.91209513	NA
ENSG0000028	FAM240B	protein_codir	0.36021591	0.91209513	NA
ENSG0000016	PRKACG	protein_codir	0.36021591	0.91209513	NA

ENSG0000020	C9orf57	protein_codir	0.36021591	0.91209513	NA
ENSG0000028	RP11-204A14	lncRNA	0.36021591	0.91209513	NA
ENSG0000023	RP11-165J3.5	lncRNA	0.36021591	0.91209513	NA
ENSG0000023	BARX1-DT	lncRNA	0.36021591	0.91209513	NA
ENSG0000023	RP11-547C13.	lncRNA	0.36021591	0.91209513	NA
ENSG0000028	RP11-373O10	lncRNA	0.36021591	0.91209513	NA
ENSG0000028	RP11-122F10.	lncRNA	0.36021591	0.91209513	NA
ENSG0000023	RP11-67K19.3	lncRNA	0.36021591	0.91209513	NA
ENSG0000023	RP11-281A20	lncRNA	0.36021591	0.91209513	NA
ENSG0000013	OR1J1	protein_codir	0.36021591	0.91209513	NA
ENSG0000017	OR1N1	protein_codir	0.36021591	0.91209513	NA
ENSG0000017	OR1L3	protein_codir	0.36021591	0.91209513	NA
ENSG0000016	OR1K1	protein_codir	0.36021591	0.91209513	NA
ENSG0000023	RP11-85O21.4	lncRNA	0.36021591	0.91209513	NA
ENSG0000022	RP13-225O21	lncRNA	0.36021591	0.91209513	NA
ENSG0000023	RP11-379C10.	lncRNA	0.36021591	0.91209513	NA
ENSG0000020	RP11-339B21.	lncRNA	0.36021591	0.91209513	NA
ENSG0000026	RP11-65J3.15	lncRNA	0.36021591	0.91209513	NA
ENSG0000023	RP11-544A12	lncRNA	0.36021591	0.91209513	NA
ENSG0000024	RP11-544A12	lncRNA	0.36021591	0.91209513	NA
ENSG0000028	RP11-643E14.	lncRNA	0.36021591	0.91209513	NA
ENSG0000028	PRRT1B	protein_codir	0.36021591	0.91209513	NA
ENSG0000012	BARHL1	protein_codir	0.36021591	0.91209513	NA
ENSG0000023	RP11-98L5.5	protein_codir	0.36021591	0.91209513	NA
ENSG0000028	WI2-1959D15	lncRNA	0.36021591	0.91209513	NA
ENSG0000028	RP13-122B23.	lncRNA	0.36021591	0.91209513	NA
ENSG0000020	ADARB2-AS1	lncRNA	0.36021591	0.91209513	NA
ENSG0000023	LINC02645	lncRNA	0.36021591	0.91209513	NA
ENSG0000028	RP11-118K6.5	lncRNA	0.36021591	0.91209513	NA
ENSG0000028	RP11-453F1.1	lncRNA	0.36021591	0.91209513	NA
ENSG0000028	RP11-34C15.4	lncRNA	0.36021591	0.91209513	NA
ENSG0000019	AKR1C4	protein_codir	0.36021591	0.91209513	NA
ENSG0000023	RP11-445P17.	lncRNA	0.36021591	0.91209513	NA
ENSG0000022	LINC00710	lncRNA	0.36021591	0.91209513	NA
ENSG0000023	RP11-7C6.1	lncRNA	0.36021591	0.91209513	NA
ENSG0000022	EBLN1	protein_codir	0.36021591	0.91209513	NA
ENSG0000028	RP11-108B14.	lncRNA	0.36021591	0.91209513	NA
ENSG0000027	RP11-573G6.1	lncRNA	0.36021591	0.91209513	NA
ENSG0000023	RP11-301N24	lncRNA	0.36021591	0.91209513	NA
ENSG0000023	RP13-16H11.5	lncRNA	0.36021591	0.91209513	NA
ENSG0000024	RP11-445N18	lncRNA	0.36021591	0.91209513	NA
ENSG0000023	RP11-67C2.2	lncRNA	0.36021591	0.91209513	NA
ENSG0000027	CH17-335B8.4	lncRNA	0.36021591	0.91209513	NA
ENSG0000025	RP11-534L6.5	lncRNA	0.36021591	0.91209513	NA
ENSG0000027	RP11-63A2.2	lncRNA	0.36021591	0.91209513	NA
ENSG0000022	LINC02671	lncRNA	0.36021591	0.91209513	NA
ENSG0000018	C10orf99	protein_codir	0.36021591	0.91209513	NA

ENSG0000022	FAM245A	lncRNA	0.36021591	0.91209513	NA
ENSG0000022	RP11-57C13.6	lncRNA	0.36021591	0.91209513	NA
ENSG0000022	LINC00502	lncRNA	0.36021591	0.91209513	NA
ENSG0000027	RP11-400G3.5	protein_codir	0.36021591	0.91209513	NA
ENSG0000023	RP11-34D15.2	lncRNA	0.36021591	0.91209513	NA
ENSG0000022	LINC02624	lncRNA	0.36021591	0.91209513	NA
ENSG0000023	LINC02674	lncRNA	0.36021591	0.91209513	NA
ENSG0000027	RP11-95I16.6	lncRNA	0.36021591	0.91209513	NA
ENSG0000017	TEX36	protein_codir	0.36021591	0.91209513	NA
ENSG0000023	LINC02646	lncRNA	0.36021591	0.91209513	NA
ENSG0000022	RP11-122K13.	lncRNA	0.36021591	0.91209513	NA
ENSG0000028	RP11-108K14.	lncRNA	0.36021591	0.91209513	NA
ENSG0000018	OR52B6	protein_codir	0.36021591	0.91209513	NA
ENSG0000018	OR56A1	protein_codir	0.36021591	0.91209513	NA
ENSG0000016	OR10A5	protein_codir	0.36021591	0.91209513	NA
ENSG0000017	OR10A4	protein_codir	0.36021591	0.91209513	NA
ENSG0000025	RP11-51B23.3	lncRNA	0.36021591	0.91209513	NA
ENSG0000025	LINC02683	lncRNA	0.36021591	0.91209513	NA
ENSG0000025	RP11-396O20	lncRNA	0.36021591	0.91209513	NA
ENSG0000025	RP11-396O20	lncRNA	0.36021591	0.91209513	NA
ENSG0000025	NAV2-AS5	lncRNA	0.36021591	0.91209513	NA
ENSG0000025	CTD-3012A18	lncRNA	0.36021591	0.91209513	NA
ENSG0000025	RP1-65P5.5	lncRNA	0.36021591	0.91209513	NA
ENSG0000025	LINC02759	lncRNA	0.36021591	0.91209513	NA
ENSG0000025	LINC02710	lncRNA	0.36021591	0.91209513	NA
ENSG0000017	OR4X1	protein_codir	0.36021591	0.91209513	NA
ENSG0000023	OR4A47	protein_codir	0.36021591	0.91209513	NA
ENSG0000028	RP11-347H15	lncRNA	0.36021591	0.91209513	NA
ENSG0000015	TRIM48	protein_codir	0.36021591	0.91209513	NA
ENSG0000025	RP11-624G17	lncRNA	0.36021591	0.91209513	NA
ENSG0000007	MS4A12	protein_codir	0.36021591	0.91209513	NA
ENSG0000012	MYRF-AS1	lncRNA	0.36021591	0.91209513	NA
ENSG0000014	SLC22A8	protein_codir	0.36021591	0.91209513	NA
ENSG0000025	RP11-770G2.2	lncRNA	0.36021591	0.91209513	NA
ENSG0000024	RP11-867G23	lncRNA	0.36021591	0.91209513	NA
ENSG0000013	ALDH3B2	protein_codir	0.36021591	0.91209513	NA
ENSG0000025	RP11-805J14.	lncRNA	0.36021591	0.91209513	NA
ENSG0000025	AP000487.4	lncRNA	0.36021591	0.91209513	NA
ENSG0000023	SHANK2-AS2	lncRNA	0.36021591	0.91209513	NA
ENSG0000025	RP11-632K5.2	lncRNA	0.36021591	0.91209513	NA
ENSG0000025	RP11-702H23	lncRNA	0.36021591	0.91209513	NA
ENSG0000028	RP11-158C6.4	lncRNA	0.36021591	0.91209513	NA
ENSG0000025	CTD-2562J17.	lncRNA	0.36021591	0.91209513	NA
ENSG0000025	RP11-258O13	lncRNA	0.36021591	0.91209513	NA
ENSG0000025	CTD-2537O9.:	lncRNA	0.36021591	0.91209513	NA
ENSG0000015	CCDC83	protein_codir	0.36021591	0.91209513	NA
ENSG0000025	RP11-164N3.:	lncRNA	0.36021591	0.91209513	NA

ENSG0000025	UBTFL1	protein_codir	0.36021591	0.91209513	NA
ENSG0000025	RP11-644L4.1	lncRNA	0.36021591	0.91209513	NA
ENSG0000028	RP11-822P4.2	lncRNA	0.36021591	0.91209513	NA
ENSG0000025	LINC02715	lncRNA	0.36021591	0.91209513	NA
ENSG0000025	LINC02703	lncRNA	0.36021591	0.91209513	NA
ENSG0000011	APOA5	protein_codir	0.36021591	0.91209513	NA
ENSG0000011	APOA4	protein_codir	0.36021591	0.91209513	NA
ENSG0000028	RP11-831A10	lncRNA	0.36021591	0.91209513	NA
ENSG0000018	OR8D4	protein_codir	0.36021591	0.91209513	NA
ENSG0000019	OR8G1	protein_codir	0.36021591	0.91209513	NA
ENSG0000025	RP11-712L6.7	lncRNA	0.36021591	0.91209513	NA
ENSG0000021	KIRREL3-AS3	lncRNA	0.36021591	0.91209513	NA
ENSG0000025	RP11-168K9.1	lncRNA	0.36021591	0.91209513	NA
ENSG0000023	AP003025.2	lncRNA	0.36021591	0.91209513	NA
ENSG0000023	AP004372.1	lncRNA	0.36021591	0.91209513	NA
ENSG0000025	OPCML-IT1	lncRNA	0.36021591	0.91209513	NA
ENSG0000025	AP004550.1	lncRNA	0.36021591	0.91209513	NA
ENSG0000025	RP11-283I3.4	lncRNA	0.36021591	0.91209513	NA
ENSG0000025	RP11-1038A1	lncRNA	0.36021591	0.91209513	NA
ENSG0000021	ACSM4	protein_codir	0.36021591	0.91209513	NA
ENSG0000028	RP11-266K4.1	protein_codir	0.36021591	0.91209513	NA
ENSG0000025	RP11-705C15.	lncRNA	0.36021591	0.91209513	NA
ENSG0000018	CLEC2A	protein_codir	0.36021591	0.91209513	NA
ENSG0000025	RP11-133L14.	lncRNA	0.36021591	0.91209513	NA
ENSG0000025	PRB1	protein_codir	0.36021591	0.91209513	NA
ENSG0000025	RP11-392P7.7	lncRNA	0.36021591	0.91209513	NA
ENSG0000025	RP11-424M22	lncRNA	0.36021591	0.91209513	NA
ENSG0000025	LINC02378	lncRNA	0.36021591	0.91209513	NA
ENSG0000013	SLCO1B1	protein_codir	0.36021591	0.91209513	NA
ENSG0000027	RP11-729I10.3	lncRNA	0.36021591	0.91209513	NA
ENSG0000028	RP11-283G6.7	lncRNA	0.36021591	0.91209513	NA
ENSG0000025	LINC02400	lncRNA	0.36021591	0.91209513	NA
ENSG0000027	RP11-139E19.	lncRNA	0.36021591	0.91209513	NA
ENSG0000025	RP11-386G11	lncRNA	0.36021591	0.91209513	NA
ENSG0000018	TMPRSS12	protein_codir	0.36021591	0.91209513	NA
ENSG0000017	KRT83	protein_codir	0.36021591	0.91209513	NA
ENSG0000017	KRT75	protein_codir	0.36021591	0.91209513	NA
ENSG0000017	KRT78	protein_codir	0.36021591	0.91209513	NA
ENSG0000026	RP11-686F15.	lncRNA	0.36021591	0.91209513	NA
ENSG0000028	RP11-181L23.	protein_codir	0.36021591	0.91209513	NA
ENSG0000028	RP11-150C16.	lncRNA	0.36021591	0.91209513	NA
ENSG0000025	RP11-274J7.2	lncRNA	0.36021591	0.91209513	NA
ENSG0000025	RP11-196H14	lncRNA	0.36021591	0.91209513	NA
ENSG0000025	RP11-366L20.	lncRNA	0.36021591	0.91209513	NA
ENSG0000025	RP11-123O10	lncRNA	0.36021591	0.91209513	NA
ENSG0000027	RP11-578B16.	lncRNA	0.36021591	0.91209513	NA
ENSG0000025	RP11-2H8.2	lncRNA	0.36021591	0.91209513	NA

ENSG0000025	RP11-20E24.1	lncRNA	0.36021591	0.91209513	NA
ENSG0000025	LINC02820	lncRNA	0.36021591	0.91209513	NA
ENSG0000028	RP11-730K16.	lncRNA	0.36021591	0.91209513	NA
ENSG0000025	RP11-1105G2	lncRNA	0.36021591	0.91209513	NA
ENSG0000025	RP11-755O11	lncRNA	0.36021591	0.91209513	NA
ENSG0000025	RP11-341G23	lncRNA	0.36021591	0.91209513	NA
ENSG0000025	KCCAT198	lncRNA	0.36021591	0.91209513	NA
ENSG0000028	RP11-411G2.2	lncRNA	0.36021591	0.91209513	NA
ENSG0000027	RP11-116D17	lncRNA	0.36021591	0.91209513	NA
ENSG0000018	CCDC60	protein_codir	0.36021591	0.91209513	NA
ENSG0000021	RP11-83B20.1	lncRNA	0.36021591	0.91209513	NA
ENSG0000027	RP11-143E21.	lncRNA	0.36021591	0.91209513	NA
ENSG0000025	RP13-820C6.2	lncRNA	0.36021591	0.91209513	NA
ENSG0000025	RP11-503G7.1	lncRNA	0.36021591	0.91209513	NA
ENSG0000027	FAM230C	lncRNA	0.36021591	0.91209513	NA
ENSG0000023	LINC00463	lncRNA	0.36021591	0.91209513	NA
ENSG0000022	LINC01076	lncRNA	0.36021591	0.91209513	NA
ENSG0000028	RP11-545M8.	lncRNA	0.36021591	0.91209513	NA
ENSG0000022	LINC01079	lncRNA	0.36021591	0.91209513	NA
ENSG0000022	LINC00457	lncRNA	0.36021591	0.91209513	NA
ENSG0000023	SMIM2-IT1	lncRNA	0.36021591	0.91209513	NA
ENSG0000022	MED4-AS1	lncRNA	0.36021591	0.91209513	NA
ENSG0000023	LINC01065	lncRNA	0.36021591	0.91209513	NA
ENSG0000027	LINC02338	lncRNA	0.36021591	0.91209513	NA
ENSG0000028	RP11-105A24	lncRNA	0.36021591	0.91209513	NA
ENSG0000022	LINC01442	lncRNA	0.36021591	0.91209513	NA
ENSG0000023	LINC00347	lncRNA	0.36021591	0.91209513	NA
ENSG0000022	RP11-182M2(	lncRNA	0.36021591	0.91209513	NA
ENSG0000027	RP11-29P20.1	lncRNA	0.36021591	0.91209513	NA
ENSG0000027	RP11-471M2.	lncRNA	0.36021591	0.91209513	NA
ENSG0000022	LINC00379	lncRNA	0.36021591	0.91209513	NA
ENSG0000023	LINC00456	lncRNA	0.36021591	0.91209513	NA
ENSG0000028	RP11-419D4.1	lncRNA	0.36021591	0.91209513	NA
ENSG0000026	RP11-98F14.1	lncRNA	0.36021591	0.91209513	NA
ENSG0000025	RP11-203M5.	lncRNA	0.36021591	0.91209513	NA
ENSG0000025	RP11-219E7.4	lncRNA	0.36021591	0.91209513	NA
ENSG0000016	OR5AU1	protein_codir	0.36021591	0.91209513	NA
ENSG0000016	OR10G3	protein_codir	0.36021591	0.91209513	NA
ENSG0000025	OR10G2	protein_codir	0.36021591	0.91209513	NA
ENSG0000022	OR4E2	protein_codir	0.36021591	0.91209513	NA
ENSG0000025	OR6J1	protein_codir	0.36021591	0.91209513	NA
ENSG0000025	LINC02306	lncRNA	0.36021591	0.91209513	NA
ENSG0000025	CTD-2503I6.1	lncRNA	0.36021591	0.91209513	NA
ENSG0000025	LINC02313	lncRNA	0.36021591	0.91209513	NA
ENSG0000025	RP11-116N8.1	lncRNA	0.36021591	0.91209513	NA
ENSG0000025	RP11-964E11.	lncRNA	0.36021591	0.91209513	NA
ENSG0000025	CTD-2058B24	lncRNA	0.36021591	0.91209513	NA

ENSG0000028	RP11-1074O1	lncRNA	0.36021591	0.91209513	NA
ENSG0000025	LINC02310	lncRNA	0.36021591	0.91209513	NA
ENSG0000025	RP11-463J10.	lncRNA	0.36021591	0.91209513	NA
ENSG0000025	RP11-108M12	lncRNA	0.36021591	0.91209513	NA
ENSG0000010	CCDC198	protein_codir	0.36021591	0.91209513	NA
ENSG0000025	RP11-193F5.1	lncRNA	0.36021591	0.91209513	NA
ENSG0000025	RP11-840I19.	lncRNA	0.36021591	0.91209513	NA
ENSG0000025	RP4-693M11.	lncRNA	0.36021591	0.91209513	NA
ENSG0000028	RP11-7F17.11	lncRNA	0.36021591	0.91209513	NA
ENSG0000016	NGB	protein_codir	0.36021591	0.91209513	NA
ENSG0000014	FAM181A	protein_codir	0.36021591	0.91209513	NA
ENSG0000028	RP11-986E7.8	lncRNA	0.36021591	0.91209513	NA
ENSG0000027	RP11-179A9.3	lncRNA	0.36021591	0.91209513	NA
ENSG0000025	LINC02292	lncRNA	0.36021591	0.91209513	NA
ENSG0000028	RP11-359N5.2	lncRNA	0.36021591	0.91209513	NA
ENSG0000017	C14orf177	lncRNA	0.36021591	0.91209513	NA
ENSG0000025	RP11-661D19	lncRNA	0.36021591	0.91209513	NA
ENSG0000025	RP11-982M15	lncRNA	0.36021591	0.91209513	NA
ENSG0000025	RP11-521B24.	lncRNA	0.36021591	0.91209513	NA
ENSG0000027	AB019440.50	lncRNA	0.36021591	0.91209513	NA
ENSG0000027	LINC00226	lncRNA	0.36021591	0.91209513	NA
ENSG0000026	AC144833.1	lncRNA	0.36021591	0.91209513	NA
ENSG0000025	RP11-932O9.4	lncRNA	0.36021591	0.91209513	NA
ENSG0000025	RP11-348B17.	lncRNA	0.36021591	0.91209513	NA
ENSG0000028	RP11-1000B6.	lncRNA	0.36021591	0.91209513	NA
ENSG0000025	RP11-16O9.2	lncRNA	0.36021591	0.91209513	NA
ENSG0000026	RP11-107F6.3	lncRNA	0.36021591	0.91209513	NA
ENSG0000018	PLA2G4E	protein_codir	0.36021591	0.91209513	NA
ENSG0000027	CTD-2008A1.3	lncRNA	0.36021591	0.91209513	NA
ENSG0000013	SLC28A2	protein_codir	0.36021591	0.91209513	NA
ENSG0000028	RP11-640I2.2	lncRNA	0.36021591	0.91209513	NA
ENSG0000028	RP11-6L16.1	lncRNA	0.36021591	0.91209513	NA
ENSG0000025	RP11-142J21.	lncRNA	0.36021591	0.91209513	NA
ENSG0000025	RP11-209K10.	lncRNA	0.36021591	0.91209513	NA
ENSG0000025	RP11-111E14.	lncRNA	0.36021591	0.91209513	NA
ENSG0000025	RP11-809H16	lncRNA	0.36021591	0.91209513	NA
ENSG0000025	RP11-138H8.2	lncRNA	0.36021591	0.91209513	NA
ENSG0000026	RP11-1006G1	lncRNA	0.36021591	0.91209513	NA
ENSG0000016	TBC1D21	protein_codir	0.36021591	0.91209513	NA
ENSG0000028	CTD-2311M21	lncRNA	0.36021591	0.91209513	NA
ENSG0000028	RP11-358L4.2	lncRNA	0.36021591	0.91209513	NA
ENSG0000028	RP11-210M15	lncRNA	0.36021591	0.91209513	NA
ENSG0000025	RP11-379K22.	lncRNA	0.36021591	0.91209513	NA
ENSG0000026	RP11-382A20	lncRNA	0.36021591	0.91209513	NA
ENSG0000014	RLBP1	protein_codir	0.36021591	0.91209513	NA
ENSG0000028	RP11-94P2.1	lncRNA	0.36021591	0.91209513	NA
ENSG0000025	RP11-739G5.1	lncRNA	0.36021591	0.91209513	NA

ENSG0000025	RP11-327J17.	lncRNA	0.36021591	0.91209513	NA
ENSG0000025	LINC02157	lncRNA	0.36021591	0.91209513	NA
ENSG0000024	FAM138E	lncRNA	0.36021591	0.91209513	NA
ENSG0000028	LA16c-360B4.	lncRNA	0.36021591	0.91209513	NA
ENSG0000026	LA16c-390E6.	lncRNA	0.36021591	0.91209513	NA
ENSG0000022	LA16c-395F10	lncRNA	0.36021591	0.91209513	NA
ENSG0000026	LINC01570	lncRNA	0.36021591	0.91209513	NA
ENSG0000026	LINC02152	lncRNA	0.36021591	0.91209513	NA
ENSG0000028	LITAFD	protein_codir	0.36021591	0.91209513	NA
ENSG0000026	RP11-457I16.:	lncRNA	0.36021591	0.91209513	NA
ENSG0000028	LINC02858	lncRNA	0.36021591	0.91209513	NA
ENSG0000016	PDILT	protein_codir	0.36021591	0.91209513	NA
ENSG0000027	RP11-426C22.	lncRNA	0.36021591	0.91209513	NA
ENSG0000026	CTD-2358C21	lncRNA	0.36021591	0.91209513	NA
ENSG0000026	RP11-67H24.2	lncRNA	0.36021591	0.91209513	NA
ENSG0000021	RP11-989E6.3	lncRNA	0.36021591	0.91209513	NA
ENSG0000026	RP11-1437A8	lncRNA	0.36021591	0.91209513	NA
ENSG0000028	CH17-342O10	lncRNA	0.36021591	0.91209513	NA
ENSG0000026	ITFG1-AS1	lncRNA	0.36021591	0.91209513	NA
ENSG0000028	RP11-3M1.5	lncRNA	0.36021591	0.91209513	NA
ENSG0000014	ABCC12	protein_codir	0.36021591	0.91209513	NA
ENSG0000026	RP11-401P9.1	lncRNA	0.36021591	0.91209513	NA
ENSG0000028	RP11-437L7.2	lncRNA	0.36021591	0.91209513	NA
ENSG0000025	CTD-3032H12	lncRNA	0.36021591	0.91209513	NA
ENSG0000026	RP11-256I9.2	lncRNA	0.36021591	0.91209513	NA
ENSG0000026	RP11-70O5.2	lncRNA	0.36021591	0.91209513	NA
ENSG0000026	CTD-2033A16	lncRNA	0.36021591	0.91209513	NA
ENSG0000026	AC004158.3	lncRNA	0.36021591	0.91209513	NA
ENSG0000026	LINC01228	lncRNA	0.36021591	0.91209513	NA
ENSG0000027	RP11-510J16.	lncRNA	0.36021591	0.91209513	NA
ENSG0000026	RP11-483P21.	lncRNA	0.36021591	0.91209513	NA
ENSG0000027	RP11-118F19.	lncRNA	0.36021591	0.91209513	NA
ENSG0000016	LINC00917	lncRNA	0.36021591	0.91209513	NA
ENSG0000026	RP11-158I3.3	lncRNA	0.36021591	0.91209513	NA
ENSG0000026	LA16c-444G7.	lncRNA	0.36021591	0.91209513	NA
ENSG0000025	CTD-2555A7.:	lncRNA	0.36021591	0.91209513	NA
ENSG0000020	LINC02138	lncRNA	0.36021591	0.91209513	NA
ENSG0000026	AC144836.1	lncRNA	0.36021591	0.91209513	NA
ENSG0000022	AC099684.1	lncRNA	0.36021591	0.91209513	NA
ENSG0000026	CTD-3195I5.4	lncRNA	0.36021591	0.91209513	NA
ENSG0000026	LINC01975	lncRNA	0.36021591	0.91209513	NA
ENSG0000026	DHX33-DT	lncRNA	0.36021591	0.91209513	NA
ENSG0000012	AIPL1	protein_codir	0.36021591	0.91209513	NA
ENSG0000016	TEKT1	protein_codir	0.36021591	0.91209513	NA
ENSG0000026	RP11-530N7.3	lncRNA	0.36021591	0.91209513	NA
ENSG0000026	RP11-589P10.	lncRNA	0.36021591	0.91209513	NA
ENSG0000025	SLC35G6	protein_codir	0.36021591	0.91209513	NA

ENSG0000026	RP11-849F2.4	lncRNA	0.36021591	0.91209513	NA
ENSG0000028	RP11-477N12	lncRNA	0.36021591	0.91209513	NA
ENSG0000027	RP11-799N11	lncRNA	0.36021591	0.91209513	NA
ENSG0000026	MYH4	protein_codir	0.36021591	0.91209513	NA
ENSG0000026	RP11-628O18	lncRNA	0.36021591	0.91209513	NA
ENSG0000028	RP11-642C21.	lncRNA	0.36021591	0.91209513	NA
ENSG0000022	MYOCD-AS1	lncRNA	0.36021591	0.91209513	NA
ENSG0000026	RP11-131K5.1	lncRNA	0.36021591	0.91209513	NA
ENSG0000028	RP11-849N15	lncRNA	0.36021591	0.91209513	NA
ENSG0000019	AC090286.4	lncRNA	0.36021591	0.91209513	NA
ENSG0000026	CTB-187M2.2	lncRNA	0.36021591	0.91209513	NA
ENSG0000026	RP11-121A13	lncRNA	0.36021591	0.91209513	NA
ENSG0000026	LINC01992	lncRNA	0.36021591	0.91209513	NA
ENSG0000026	RP11-192H23	lncRNA	0.36021591	0.91209513	NA
ENSG0000027	CTD-3194G12	lncRNA	0.36021591	0.91209513	NA
ENSG0000027	CTC-268N12.3	lncRNA	0.36021591	0.91209513	NA
ENSG0000017	KRT20	protein_codir	0.36021591	0.91209513	NA
ENSG0000020	KRT40	protein_codir	0.36021591	0.91209513	NA
ENSG0000018	KRTAP1-1	protein_codir	0.36021591	0.91209513	NA
ENSG0000020	KRTAP4-8	protein_codir	0.36021591	0.91209513	NA
ENSG0000013	KRT33B	protein_codir	0.36021591	0.91209513	NA
ENSG0000010	KRT32	protein_codir	0.36021591	0.91209513	NA
ENSG0000026	RP5-905N1.2	lncRNA	0.36021591	0.91209513	NA
ENSG0000026	ATXN7L3-AS1	lncRNA	0.36021591	0.91209513	NA
ENSG0000026	RP11-6N17.3	lncRNA	0.36021591	0.91209513	NA
ENSG0000022	PRAC2	protein_codir	0.36021591	0.91209513	NA
ENSG0000024	RP11-94C24.6	lncRNA	0.36021591	0.91209513	NA
ENSG0000026	RP11-506D12	lncRNA	0.36021591	0.91209513	NA
ENSG0000026	DYNLL2-DT	lncRNA	0.36021591	0.91209513	NA
ENSG0000026	IGBP1P2	protein_codir	0.36021591	0.91209513	NA
ENSG0000026	RP11-579O24	lncRNA	0.36021591	0.91209513	NA
ENSG0000026	RP11-619I22.1	lncRNA	0.36021591	0.91209513	NA
ENSG0000028	RP11-156L14.	lncRNA	0.36021591	0.91209513	NA
ENSG0000026	CTD-2501B8.1	protein_codir	0.36021591	0.91209513	NA
ENSG0000028	RP11-214C8.6	lncRNA	0.36021591	0.91209513	NA
ENSG0000026	RP11-855A2.3	lncRNA	0.36021591	0.91209513	NA
ENSG0000026	RP11-57A1.1	lncRNA	0.36021591	0.91209513	NA
ENSG0000026	CTD-3010D24	lncRNA	0.36021591	0.91209513	NA
ENSG0000028	CTD-2582D11	lncRNA	0.36021591	0.91209513	NA
ENSG0000026	CTD-2514K5.4	lncRNA	0.36021591	0.91209513	NA
ENSG0000017	FADS6	protein_codir	0.36021591	0.91209513	NA
ENSG0000026	RP11-474I11.8	lncRNA	0.36021591	0.91209513	NA
ENSG0000026	RP11-285E9.5	lncRNA	0.36021591	0.91209513	NA
ENSG0000027	RP11-353N14	lncRNA	0.36021591	0.91209513	NA
ENSG0000026	RP11-498C9.1	lncRNA	0.36021591	0.91209513	NA
ENSG0000026	RP11-1376P1(	lncRNA	0.36021591	0.91209513	NA
ENSG0000026	RP11-720L2.4	lncRNA	0.36021591	0.91209513	NA

ENSG0000026	RP11-806L2.5	lncRNA	0.36021591	0.91209513	NA
ENSG0000026	RP11-769O8.1	lncRNA	0.36021591	0.91209513	NA
ENSG0000025	LINC01895	lncRNA	0.36021591	0.91209513	NA
ENSG0000026	RP11-172F10.	lncRNA	0.36021591	0.91209513	NA
ENSG0000026	RP11-190I17.1	lncRNA	0.36021591	0.91209513	NA
ENSG0000026	RP11-835E18.	lncRNA	0.36021591	0.91209513	NA
ENSG0000026	RP11-286N3.2	lncRNA	0.36021591	0.91209513	NA
ENSG0000028	RP11-146G7.5	lncRNA	0.36021591	0.91209513	NA
ENSG0000026	LINC00668	lncRNA	0.36021591	0.91209513	NA
ENSG0000026	RP11-805F19.	lncRNA	0.36021591	0.91209513	NA
ENSG0000026	RP11-627G18	lncRNA	0.36021591	0.91209513	NA
ENSG0000026	PCAT18	lncRNA	0.36021591	0.91209513	NA
ENSG0000022	AC015933.2	lncRNA	0.36021591	0.91209513	NA
ENSG0000026	DSG1-AS1	lncRNA	0.36021591	0.91209513	NA
ENSG0000026	RP11-379L18.	lncRNA	0.36021591	0.91209513	NA
ENSG0000026	RP11-797E24.	lncRNA	0.36021591	0.91209513	NA
ENSG0000028	RP11-1119E1	lncRNA	0.36021591	0.91209513	NA
ENSG0000028	ELOA3D	protein_codir	0.36021591	0.91209513	NA
ENSG0000028	ELOA3B	protein_codir	0.36021591	0.91209513	NA
ENSG0000028	ELOA3	protein_codir	0.36021591	0.91209513	NA
ENSG0000026	MIR4527HG	lncRNA	0.36021591	0.91209513	NA
ENSG0000026	RP11-99A1.2	lncRNA	0.36021591	0.91209513	NA
ENSG0000026	RP11-879F14.	lncRNA	0.36021591	0.91209513	NA
ENSG0000028	RP11-299P2.2	lncRNA	0.36021591	0.91209513	NA
ENSG0000020	SERPINB4	protein_codir	0.36021591	0.91209513	NA
ENSG0000026	LINC01924	lncRNA	0.36021591	0.91209513	NA
ENSG0000017	LINC00305	lncRNA	0.36021591	0.91209513	NA
ENSG0000026	LINC01541	lncRNA	0.36021591	0.91209513	NA
ENSG0000028	RP11-625N15	lncRNA	0.36021591	0.91209513	NA
ENSG0000026	RP11-321M21	lncRNA	0.36021591	0.91209513	NA
ENSG0000026	LINC01927	lncRNA	0.36021591	0.91209513	NA
ENSG0000027	RP11-196B3.3	lncRNA	0.36021591	0.91209513	NA
ENSG0000026	CTB-50L17.5	lncRNA	0.36021591	0.91209513	NA
ENSG0000026	CTD-2265O21	lncRNA	0.36021591	0.91209513	NA
ENSG0000026	CTB-55O6.4	lncRNA	0.36021591	0.91209513	NA
ENSG0000018	OR7A5	protein_codir	0.36021591	0.91209513	NA
ENSG0000017	OR10H5	protein_codir	0.36021591	0.91209513	NA
ENSG0000026	CTC-429P9.1	lncRNA	0.36021591	0.91209513	NA
ENSG0000026	CTD-2528A14	lncRNA	0.36021591	0.91209513	NA
ENSG0000026	CTD-2521M24	lncRNA	0.36021591	0.91209513	NA
ENSG0000026	CTC-559E9.8	lncRNA	0.36021591	0.91209513	NA
ENSG0000026	RP11-420K14.	lncRNA	0.36021591	0.91209513	NA
ENSG0000026	AC003973.4	lncRNA	0.36021591	0.91209513	NA
ENSG0000027	CTB-32O4.4	lncRNA	0.36021591	0.91209513	NA
ENSG0000026	VSTM2B-DT	lncRNA	0.36021591	0.91209513	NA
ENSG0000026	CTC-565M22.	lncRNA	0.36021591	0.91209513	NA
ENSG0000027	LINC00904	lncRNA	0.36021591	0.91209513	NA

ENSG0000022	HPN-AS1	lncRNA	0.36021591	0.91209513	NA
ENSG0000022	LINC01766	lncRNA	0.36021591	0.91209513	NA
ENSG0000026	CTD-3162L10.	lncRNA	0.36021591	0.91209513	NA
ENSG0000028	CTD-2528L19.	lncRNA	0.36021591	0.91209513	NA
ENSG0000026	AC005789.9	lncRNA	0.36021591	0.91209513	NA
ENSG0000026	SERTAD3-AS1	lncRNA	0.36021591	0.91209513	NA
ENSG0000019	CYP2B6	protein_codir	0.36021591	0.91209513	NA
ENSG0000027	AC020956.3	lncRNA	0.36021591	0.91209513	NA
ENSG0000028	CTC-204F22.7	lncRNA	0.36021591	0.91209513	NA
ENSG0000026	IGFL2-AS1	lncRNA	0.36021591	0.91209513	NA
ENSG0000026	NOP53-AS1	lncRNA	0.36021591	0.91209513	NA
ENSG0000010	KCNA7	protein_codir	0.36021591	0.91209513	NA
ENSG0000026	GFY	protein_codir	0.36021591	0.91209513	NA
ENSG0000016	IZUMO2	protein_codir	0.36021591	0.91209513	NA
ENSG0000026	CTB-191K22.5	lncRNA	0.36021591	0.91209513	NA
ENSG0000026	CTD-3099C6.5	lncRNA	0.36021591	0.91209513	NA
ENSG0000026	ERVV-2	protein_codir	0.36021591	0.91209513	NA
ENSG0000019	VN1R2	protein_codir	0.36021591	0.91209513	NA
ENSG0000020	DPRX	protein_codir	0.36021591	0.91209513	NA
ENSG0000021	CTB-61M7.1	lncRNA	0.36021591	0.91209513	NA
ENSG0000018	NLRP9	protein_codir	0.36021591	0.91209513	NA
ENSG0000023	UBOX5-AS1	lncRNA	0.36021591	0.91209513	NA
ENSG0000027	RP4-681N20.5	lncRNA	0.36021591	0.91209513	NA
ENSG0000022	PLCB1-IT1	lncRNA	0.36021591	0.91209513	NA
ENSG0000023	RP4-796I8.1	lncRNA	0.36021591	0.91209513	NA
ENSG0000023	RP5-1185K9.1	lncRNA	0.36021591	0.91209513	NA
ENSG0000023	RP4-753D10.5	lncRNA	0.36021591	0.91209513	NA
ENSG0000012	CST11	protein_codir	0.36021591	0.91209513	NA
ENSG0000017	CST9	protein_codir	0.36021591	0.91209513	NA
ENSG0000022	LINC01733	lncRNA	0.36021591	0.91209513	NA
ENSG0000013	DEFB118	protein_codir	0.36021591	0.91209513	NA
ENSG0000027	RP1-310O13.1	lncRNA	0.36021591	0.91209513	NA
ENSG0000015	WFDC8	protein_codir	0.36021591	0.91209513	NA
ENSG0000028	RP11-321P16.	lncRNA	0.36021591	0.91209513	NA
ENSG0000028	RP5-1050C22.	lncRNA	0.36021591	0.91209513	NA
ENSG0000028	RP5-831D17.4	lncRNA	0.36021591	0.91209513	NA
ENSG0000023	RP11-80K6.2	lncRNA	0.36021591	0.91209513	NA
ENSG0000025	RP5-823G15.5	lncRNA	0.36021591	0.91209513	NA
ENSG0000019	RP4-724E16.2	lncRNA	0.36021591	0.91209513	NA
ENSG0000020	RP11-560A15	lncRNA	0.36021591	0.91209513	NA
ENSG0000022	RP4-813D12.3	lncRNA	0.36021591	0.91209513	NA
ENSG0000022	RP4-718J7.4	lncRNA	0.36021591	0.91209513	NA
ENSG0000027	CH507-9B2.8	lncRNA	0.36021591	0.91209513	NA
ENSG0000027	CH507-236L2	lncRNA	0.36021591	0.91209513	NA
ENSG0000027	AL078471.5	lncRNA	0.36021591	0.91209513	NA
ENSG0000022	AF127577.10	lncRNA	0.36021591	0.91209513	NA
ENSG0000024	C21orf91-OT1	lncRNA	0.36021591	0.91209513	NA

ENSG0000023	CHODL-AS1	lncRNA	0.36021591	0.91209513	NA
ENSG0000023	LINC02573	lncRNA	0.36021591	0.91209513	NA
ENSG0000019	CYYR1-AS1	lncRNA	0.36021591	0.91209513	NA
ENSG0000022	AP000282.2	lncRNA	0.36021591	0.91209513	NA
ENSG0000023	BRWD1-AS1	lncRNA	0.36021591	0.91209513	NA
ENSG0000022	AP001610.5	lncRNA	0.36021591	0.91209513	NA
ENSG0000027	RP1-101D8.1	lncRNA	0.36021591	0.91209513	NA
ENSG0000022	AP001476.3	lncRNA	0.36021591	0.91209513	NA
ENSG0000022	PSLNR	lncRNA	0.36021591	0.91209513	NA
ENSG0000028	AC007666.5	lncRNA	0.36021591	0.91209513	NA
ENSG0000028	XXbac-B461K1	lncRNA	0.36021591	0.91209513	NA
ENSG0000027	AC007308.7	lncRNA	0.36021591	0.91209513	NA
ENSG0000028	FAM246A	protein_codir	0.36021591	0.91209513	NA
ENSG0000022	AP000355.2	lncRNA	0.36021591	0.91209513	NA
ENSG0000027	CTA-243E7.4	lncRNA	0.36021591	0.91209513	NA
ENSG0000027	CTA-992D9.9	lncRNA	0.36021591	0.91209513	NA
ENSG0000023	CTA-503F6.2	lncRNA	0.36021591	0.91209513	NA
ENSG0000023	CTA-929C8.5	lncRNA	0.36021591	0.91209513	NA
ENSG0000023	CTA-929C8.8	lncRNA	0.36021591	0.91209513	NA
ENSG0000028	AC000035.3	lncRNA	0.36021591	0.91209513	NA
ENSG0000028	LL22NC03-44	protein_codir	0.36021591	0.91209513	NA
ENSG0000018	BPIFC	protein_codir	0.36021591	0.91209513	NA
ENSG0000017	ISX	protein_codir	0.36021591	0.91209513	NA
ENSG0000028	CTA-286B10.8	lncRNA	0.36021591	0.91209513	NA
ENSG0000027	CTA-212A2.2	lncRNA	0.36021591	0.91209513	NA
ENSG0000026	RP5-1119A7.1	lncRNA	0.36021591	0.91209513	NA
ENSG0000028	LL22NC01-132	lncRNA	0.36021591	0.91209513	NA
ENSG0000023	RP5-1039K5.1	lncRNA	0.36021591	0.91209513	NA
ENSG0000022	RP1-32I10.10	lncRNA	0.36021591	0.91209513	NA
ENSG0000023	RP11-536P6.3	lncRNA	0.36021591	0.91209513	NA
ENSG0000028	CTA-299D3.9	lncRNA	0.36021591	0.91209513	NA
ENSG0000028	CTA-722E9.1	lncRNA	0.36021591	0.91209513	NA
ENSG0000022	AC000036.4	lncRNA	0.36021591	0.91209513	NA
ENSG0000018	VCX	protein_codir	0.36021591	0.91209513	NA
ENSG0000018	FAM9C	protein_codir	0.36021591	0.91209513	NA
ENSG0000021	RP1-122K4.3	lncRNA	0.36021591	0.91209513	NA
ENSG0000028	RP11-1037J10	lncRNA	0.36021591	0.91209513	NA
ENSG0000017	CBLL2	protein_codir	0.36021591	0.91209513	NA
ENSG0000027	ZNF630-AS1	lncRNA	0.36021591	0.91209513	NA
ENSG0000028	RP11-402P6.1	protein_codir	0.36021591	0.91209513	NA
ENSG0000023	PHKA1-AS1	lncRNA	0.36021591	0.91209513	NA
ENSG0000027	RP13-216E22.	lncRNA	0.36021591	0.91209513	NA
ENSG0000028	RP11-485F13.	lncRNA	0.36021591	0.91209513	NA
ENSG0000027	RP4-769N13.7	lncRNA	0.36021591	0.91209513	NA
ENSG0000022	TDGF1P3	protein_codir	0.36021591	0.91209513	NA
ENSG0000018	PRR32	protein_codir	0.36021591	0.91209513	NA
ENSG0000013	USP26	protein_codir	0.36021591	0.91209513	NA

ENSG0000026	CT45A10	protein_codir	0.36021591	0.91209513	NA
ENSG0000016	GPR101	protein_codir	0.36021591	0.91209513	NA
ENSG0000021	XX-FW80269A	lncRNA	0.36021591	0.91209513	NA
ENSG0000019	MAGEA1	protein_codir	0.36021591	0.91209513	NA
ENSG0000010	OPN1LW	protein_codir	0.36021591	0.91209513	NA
ENSG0000022	LINC00280	lncRNA	0.36021591	0.91209513	NA
ENSG0000023	TSPY4	protein_codir	0.36021591	0.91209513	NA
ENSG0000023	TSPY10	protein_codir	0.36021591	0.91209513	NA
ENSG0000022	TTTY22	lncRNA	0.36021591	0.91209513	NA
ENSG0000013	TTTY9B	lncRNA	0.36021591	0.91209513	NA
ENSG0000016	HSFY2	protein_codir	0.36021591	0.91209513	NA
ENSG0000018	TTTY13	lncRNA	0.36021591	0.91209513	NA
ENSG0000016	PRY2	protein_codir	0.36021591	0.91209513	NA
ENSG0000026	ENSG0000026	protein_codir	0.36021591	0.91209513	NA
ENSG0000025	AC099668.5	lncRNA	0.36008894	0.75790306	0.84673049
ENSG0000019	EOLA1	protein_codir	0.36005104	0.00133124	0.00718729
ENSG0000008	EIF2AK1	protein_codir	0.35987009	0.00095783	0.00553923
ENSG0000026	RP11-679B19.	lncRNA	0.35973817	0.85920248	NA
ENSG0000016	RPN1	protein_codir	0.35971822	0.01304324	0.04274091
ENSG0000017	C8orf31	lncRNA	0.35959427	0.36186219	0.5157722
ENSG0000010	TRIM9	protein_codir	0.3595206	0.4773161	0.62534251
ENSG0000023	LINC01473	lncRNA	0.3592841	0.42085812	0.57295797
ENSG0000026	RP11-45M22.	lncRNA	0.3589844	0.69113312	0.79727255
ENSG0000015	CDK19	protein_codir	0.35889675	0.02645082	0.0737281
ENSG0000015	SPATA2L	protein_codir	0.35886296	0.24954642	0.39539193
ENSG0000018	LMLN	protein_codir	0.35872089	0.05045276	0.12136433
ENSG0000014	CHAC2	protein_codir	0.35843444	0.2475529	0.39288693
ENSG0000019	METTL9	protein_codir	0.35841065	0.005787	0.02268798
ENSG0000023	RP11-47I22.3	lncRNA	0.35840466	0.55688883	0.69272516
ENSG0000025	AC006277.2	lncRNA	0.3581241	0.46236341	0.61249293
ENSG0000018	GPAT2	protein_codir	0.35811859	0.31504973	0.46685302
ENSG0000025	AP5B1	protein_codir	0.35808812	0.00124776	0.00681831
ENSG0000011	AREL1	protein_codir	0.35799816	0.05012888	0.12075351
ENSG0000015	FMNL2	protein_codir	0.35770197	0.09298199	0.19100821
ENSG0000012	ELL3	protein_codir	0.3575551	0.49763011	0.64336237
ENSG0000015	ASTN1	protein_codir	0.3575472	0.47422758	0.62267493
ENSG0000027	KMT2B	protein_codir	0.35745406	0.00563032	0.02222275
ENSG0000016	RBBP4	protein_codir	0.3573458	0.00419129	0.01769002
ENSG0000027	RP11-147L13.	lncRNA	0.35727076	0.27035625	0.41819218
ENSG0000025	RP11-638I2.2	lncRNA	0.35719221	0.64880908	0.76496656
ENSG0000017	GAA	protein_codir	0.35692888	0.0254245	0.07153878
ENSG0000025	LINC02285	lncRNA	0.35689661	0.53752494	0.67730602
ENSG0000007	MKNK1	protein_codir	0.35686825	0.03233702	0.08614472
ENSG0000019	ZNF136	protein_codir	0.35684598	0.01081994	0.03707775
ENSG0000011	SLCO1B3	protein_codir	0.35672333	0.85485743	NA
ENSG0000024	TWF2	protein_codir	0.35665393	0.02061566	0.06071059
ENSG0000022	ARL17B	protein_codir	0.35652945	0.35861599	0.51230024

ENSG0000026	UQCRFS1-DT	lncRNA	0.35650903	0.59274412	0.72153339
ENSG0000025	RP11-218E20.	lncRNA	0.3564778	0.57455385	0.70721488
ENSG0000027	WDR5-DT	lncRNA	0.35643499	0.6113688	0.73658271
ENSG0000011	SRSF9	protein_codir	0.35634573	0.00121081	0.0066592
ENSG0000015	VOPP1	protein_codir	0.35633565	0.13329489	0.24983559
ENSG0000013	P2RX4	protein_codir	0.35593841	0.06849119	0.15252964
ENSG0000016	EIF4A1	protein_codir	0.35563649	0.08423025	0.17722554
ENSG0000013	CCDC59	protein_codir	0.35558032	0.00284208	0.01292153
ENSG0000022	ORM1	protein_codir	0.35531911	0.7445258	0.83681137
ENSG0000012	MTERF4	protein_codir	0.35520259	0.05677709	0.13228142
ENSG0000014	MINK1	protein_codir	0.35509642	0.00098428	0.00565732
ENSG0000014	RSPO3	protein_codir	0.35496883	NA	NA
ENSG0000020	AF064858.6	lncRNA	0.35481307	0.50793869	0.65237232
ENSG0000016	FLVCR1	protein_codir	0.35474516	0.09172899	0.18896369
ENSG0000004	JADE2	protein_codir	0.35462489	0.07633643	0.16505497
ENSG0000010	VAC14	protein_codir	0.35456278	0.0478177	0.11631219
ENSG0000025	RP11-661A12	lncRNA	0.35417197	0.78079485	0.86289827
ENSG0000027	RIMBP3B	protein_codir	0.35415873	0.60891106	0.73460773
ENSG0000016	ART5	protein_codir	0.35410312	0.76233222	0.84994211
ENSG0000020	C4orf46	protein_codir	0.35395361	0.02734094	0.07566701
ENSG0000026	RP1-178F10.1	lncRNA	0.35395099	0.66592073	0.77927283
ENSG0000018	NHLRC3	protein_codir	0.35378281	0.01514994	0.04806356
ENSG0000013	DAGLA	protein_codir	0.35370738	0.20089903	0.33774613
ENSG0000027	AC001226.7	lncRNA	0.3536836	0.74376276	0.83629536
ENSG0000026	RP11-319G9.3	lncRNA	0.35361128	0.58696988	0.71728644
ENSG0000027	PIGW	protein_codir	0.35349256	0.13158618	0.24744763
ENSG0000017	NFATC2IP	protein_codir	0.3534331	0.0909022	0.18760509
ENSG0000023	RP11-509J21.	lncRNA	0.35341766	0.7682838	0.85406096
ENSG0000026	RP11-715J22.4	lncRNA	0.35304756	0.62019937	0.74355007
ENSG0000016	MAPKAPK2	protein_codir	0.3529912	0.10631869	0.21127813
ENSG0000016	CCDC141	protein_codir	0.35294526	0.10398555	0.20778843
ENSG0000026	RANBP3-DT	lncRNA	0.35280729	0.75792256	0.84673049
ENSG0000025	PCDHA10	protein_codir	0.35240622	0.47561943	0.62385946
ENSG0000026	AF131215.9	lncRNA	0.35209255	0.36955353	0.523055
ENSG0000015	EPG5	protein_codir	0.35194915	0.06404538	0.1453257
ENSG0000011	CUL9	protein_codir	0.3519272	0.03582367	0.09318608
ENSG0000012	ZNF706	protein_codir	0.35152026	0.0009033	0.00527799
ENSG0000001	ZNF200	protein_codir	0.35136785	0.04494296	0.11110039
ENSG0000001	PHF7	protein_codir	0.3512733	0.02005223	0.05941242
ENSG0000011	RPA2	protein_codir	0.35120564	0.01502161	0.04778589
ENSG0000019	MRTFA	protein_codir	0.351167	0.00049008	0.00321749
ENSG0000010	CEP192	protein_codir	0.35115206	0.05033113	0.12117728
ENSG0000016	DFFB	protein_codir	0.35113244	0.1997534	0.33637294
ENSG0000003	SOX30	protein_codir	0.35104306	0.57887787	0.7108891
ENSG0000025	GASAL1	lncRNA	0.35103879	0.32837164	0.48073099
ENSG0000027	RP11-339B21.	lncRNA	0.35102853	0.42383305	0.57584086
ENSG0000011	PIGZ	protein_codir	0.35101339	0.17678756	0.30721783

ENSG0000028	RP1-34B20.21	protein_codir	0.35096571	0.61390737	0.73824977
ENSG0000015	GALK2	protein_codir	0.35081893	0.00166306	0.00854265
ENSG0000017	PFN4	protein_codir	0.35072123	0.40693548	0.55993414
ENSG0000016	STXBP5	protein_codir	0.3506833	0.02142772	0.06254187
ENSG0000012	WIPF3	protein_codir	0.35063897	0.34610712	0.49913735
ENSG0000027	CASTOR2	protein_codir	0.35050347	0.09814245	0.19897926
ENSG0000021	ZNF705E	protein_codir	0.3504489	0.67355857	0.784707
ENSG0000017	MIR4435-2HG	lncRNA	0.35031222	0.17282962	0.30246825
ENSG0000025	CTD-2002H8.2	lncRNA	0.35027587	0.71854016	0.81826005
ENSG0000017	NADSYN1	protein_codir	0.35019395	0.16211965	0.28844754
ENSG0000015	XKR8	protein_codir	0.35012479	0.05988075	0.13772304
ENSG0000009	CDV3	protein_codir	0.34988833	0.06514232	0.14726066
ENSG0000013	AGTPBP1	protein_codir	0.34949998	0.04338754	0.10805805
ENSG0000013	COX4I1	protein_codir	0.34943966	0.00553275	0.02193721
ENSG0000013	IREB2	protein_codir	0.34935607	0.05227481	0.12435021
ENSG0000018	SERPINA5	protein_codir	0.34935213	0.57214192	0.7052825
ENSG0000019	L3MBTL3	protein_codir	0.34920893	0.01762424	0.05380586
ENSG0000010	UGDH	protein_codir	0.34914768	0.40964597	0.5626826
ENSG0000017	NAA16	protein_codir	0.34909754	0.01464635	0.04683938
ENSG0000025	RP11-1391J7.	lncRNA	0.34904158	0.40112013	0.55416857
ENSG0000010	LEPROTL1	protein_codir	0.34890926	0.04643146	0.11377191
ENSG0000027	RP11-190C22.	lncRNA	0.34888002	0.83925066	NA
ENSG0000014	CEP131	protein_codir	0.34853999	0.11744206	0.22729659
ENSG0000023	RP11-337C18.	lncRNA	0.34849788	0.53334988	0.67410074
ENSG0000012	SIRT5	protein_codir	0.34824547	0.05112925	0.12247058
ENSG0000018	BRWD1	protein_codir	0.34805202	0.00434646	0.01817275
ENSG0000013	ARHGAP32	protein_codir	0.34780109	0.02987238	0.08119219
ENSG0000018	SF3B3	protein_codir	0.34770395	0.00542719	0.02163147
ENSG0000012	AANAT	protein_codir	0.34760333	0.76689486	0.85306629
ENSG0000022	MYOSLID	lncRNA	0.34754913	0.39056715	0.54358603
ENSG0000012	NDP	protein_codir	0.34728985	0.46094015	0.61125249
ENSG0000016	ZNF646	protein_codir	0.34717739	0.04011284	0.10173706
ENSG0000025	TMEM9B-AS1	lncRNA	0.34716364	0.4160871	0.56884254
ENSG0000022	AC012363.4	lncRNA	0.34697506	0.84238899	NA
ENSG0000010	DICER1	protein_codir	0.34692388	0.04207938	0.10561908
ENSG0000011	PRLR	protein_codir	0.34682574	0.44573572	0.59674618
ENSG0000026	RP11-728E14.	lncRNA	0.34679552	0.81066045	0.88314762
ENSG0000017	MSL2	protein_codir	0.34662151	0.03580039	0.09313428
ENSG0000021	IRF9	protein_codir	0.34622976	0.07517885	0.16324213
ENSG0000024	ADH1C	protein_codir	0.34617091	0.51045766	0.65440178
ENSG0000017	MLXIP	protein_codir	0.34614366	0.07280089	0.15955884
ENSG0000026	RP11-95D17.1	lncRNA	0.34613288	0.07162655	0.15767317
ENSG0000011	NAA40	protein_codir	0.34607468	0.13957573	0.25841812
ENSG0000024	AP5Z1	protein_codir	0.34604909	0.05507983	0.12934067
ENSG0000011	AMOTL2	protein_codir	0.34592946	0.32191379	0.47412461
ENSG0000016	TMEM143	protein_codir	0.34588434	0.07881201	0.16882176
ENSG0000019	INKA2	protein_codir	0.34586512	0.21205827	0.3516058

ENSG0000027	RP11-229P13.	protein_codir	0.34584736	0.79213843	0.87083519
ENSG0000016	CNST	protein_codir	0.34570098	0.02066781	0.06084351
ENSG0000014	LRRC27	protein_codir	0.34569749	0.07753052	0.16677516
ENSG0000011	FAAH	protein_codir	0.34562242	0.3531166	0.50669867
ENSG0000026	LA16c-390E6.	lncRNA	0.34549168	0.54372318	0.68204823
ENSG0000006	ARFGEF1	protein_codir	0.34529963	0.01894028	0.05688676
ENSG0000012	WDR11	protein_codir	0.34497848	0.02259966	0.06523857
ENSG0000027	RP11-981G7.6	lncRNA	0.34493706	0.44321101	0.59421815
ENSG0000024	OIP5-AS1	lncRNA	0.34493302	0.00775219	0.02851453
ENSG0000027	RP11-850A17	lncRNA	0.34473694	0.59977625	0.72733667
ENSG0000027	RP11-116D17	lncRNA	0.34465464	0.88357273	NA
ENSG0000028	RP11-418I22.:	lncRNA	0.34459148	0.86161025	NA
ENSG0000016	SCARA5	protein_codir	0.34449633	0.55508445	0.69109356
ENSG0000012	RPL23	protein_codir	0.34447968	0.02630705	0.07341627
ENSG0000019	ZNF544	protein_codir	0.34438042	0.05565011	0.13034736
ENSG0000012	EXOSC8	protein_codir	0.34418238	0.07100294	0.15664999
ENSG0000026	RP11-552M11	lncRNA	0.34406081	0.26330505	0.41068927
ENSG0000015	ZFHX2-AS1	lncRNA	0.34404516	0.36450279	0.51845235
ENSG0000023	RP3-404F18.5	lncRNA	0.34403919	0.71362378	0.81434014
ENSG0000021	HIGD1C	protein_codir	0.34386466	0.76768434	0.8535479
ENSG0000019	ZNF273	protein_codir	0.34380838	0.15590972	0.28022558
ENSG0000013	TUT4	protein_codir	0.34378792	0.04340077	0.10808127
ENSG0000021	CCL27	protein_codir	0.34368132	0.77221718	0.85667505
ENSG0000028	RP11-398C13.	protein_codir	0.34357579	0.01607385	0.05030135
ENSG0000026	RP11-387H17	lncRNA	0.34337664	0.70979309	0.81148359
ENSG0000024	AC093627.10	lncRNA	0.34335036	0.32050712	0.4725568
ENSG0000028	PANO1	protein_codir	0.3432786	0.38413205	0.53744629
ENSG0000004	SNX29	protein_codir	0.34326233	0.08549752	0.17941357
ENSG0000025	RP11-867G23	lncRNA	0.34318975	0.62415103	0.74653783
ENSG0000015	ABCA10	protein_codir	0.34272888	0.48561101	0.63281793
ENSG0000024	OR2AE1	protein_codir	0.34269693	0.8471085	NA
ENSG0000011	DLEU2L	lncRNA	0.34262636	0.36922425	0.52281215
ENSG0000014	TBC1D32	protein_codir	0.34258336	0.24537516	0.39052626
ENSG0000019	YTHDF2	protein_codir	0.34251448	0.00111226	0.00622381
ENSG0000028	RP3-426I6.8	lncRNA	0.34230422	0.84334238	NA
ENSG0000027	RP11-188D8.1	lncRNA	0.34229704	0.71057982	0.81197363
ENSG0000013	TMOD3	protein_codir	0.3418132	0.01535834	0.04854071
ENSG0000016	SIN3A	protein_codir	0.3417952	0.01485633	0.0473703
ENSG0000026	DTX2P1-UPK3	lncRNA	0.34175657	0.07425391	0.16181024
ENSG0000009	ERMP1	protein_codir	0.34131212	0.08601749	0.18021718
ENSG0000027	RP11-181E10.	lncRNA	0.34119312	0.50308711	0.64785128
ENSG0000014	MRRF	protein_codir	0.34105658	0.03446093	0.0905716
ENSG0000019	SNHG17	lncRNA	0.34103992	0.30692274	0.45867364
ENSG0000015	LRBA	protein_codir	0.34097142	0.12616447	0.23997135
ENSG0000028	RP4-671O14.7	lncRNA	0.34078711	0.69462286	0.7997617
ENSG0000006	RNF4	protein_codir	0.34040725	0.00103529	0.00588731
ENSG0000010	NOD1	protein_codir	0.34030371	0.02189184	0.06356736

ENSG0000018	DDN	protein_codir	0.34029283	0.44482001	0.59594144
ENSG0000020	ARID3C	protein_codir	0.34002195	0.74299175	0.83590355
ENSG0000026	CTD-2083E4.4	lncRNA	0.33999626	0.47658122	0.62473532
ENSG0000027	RP5-864K19.6	protein_codir	0.33998913	0.82473021	0.89356743
ENSG0000016	PRSS12	protein_codir	0.33994013	0.59220801	0.72117496
ENSG0000006	PMS1	protein_codir	0.33954937	0.02693352	0.07477171
ENSG0000010	MINPP1	protein_codir	0.33951479	0.01847545	0.05580011
ENSG0000004	SCML1	protein_codir	0.33951054	0.16973849	0.29840093
ENSG0000014	PGAP2	protein_codir	0.33930936	0.12308219	0.23555503
ENSG0000010	IFT27	protein_codir	0.33924864	0.10542123	0.20988498
ENSG0000013	ATP5MC2	protein_codir	0.33906339	0.00711892	0.02665733
ENSG0000012	PSPC1	protein_codir	0.33900668	0.00670706	0.02542883
ENSG0000008	TDRD3	protein_codir	0.33895511	0.01223171	0.04069277
ENSG0000010	RAB3A	protein_codir	0.33893691	0.20754106	0.34606279
ENSG0000028	RP4-696F19.1	lncRNA	0.33887984	0.84530417	0.90774888
ENSG0000011	KCNA1	protein_codir	0.33886332	0.56326482	0.69767139
ENSG0000016	CDC40	protein_codir	0.33852471	0.02389653	0.06810136
ENSG0000027	FCGBP	protein_codir	0.33843746	0.59085315	0.72026017
ENSG0000011	ZNF451	protein_codir	0.33836652	0.00093331	0.00541899
ENSG0000017	C2CD2L	protein_codir	0.33835863	0.0419095	0.10530753
ENSG0000016	TFB2M	protein_codir	0.33791741	0.05950936	0.13707611
ENSG0000022	RP5-864K19.4	lncRNA	0.33791026	0.39604397	0.54891281
ENSG0000025	ZNF718	protein_codir	0.33779885	0.10876003	0.21487497
ENSG0000011	HDAC1	protein_codir	0.33749037	0.06460955	0.14631976
ENSG0000028	RP11-430H15	lncRNA	0.33723209	0.65886265	0.773288
ENSG0000011	DUSP16	protein_codir	0.33711665	0.15330973	0.27675914
ENSG0000026	RP11-863P13.	lncRNA	0.33705672	0.64706178	0.76415754
ENSG0000011	PRDM2	protein_codir	0.336985	0.01153622	0.03894573
ENSG0000019	IRAK4	protein_codir	0.3366347	0.05141114	0.12293327
ENSG0000009	SIRT1	protein_codir	0.33654953	0.05655153	0.13191059
ENSG0000010	C20orf27	protein_codir	0.33636195	0.05932353	0.13671387
ENSG0000008	FKBP1A	protein_codir	0.33606779	0.01849125	0.05583643
ENSG0000022	LINC02593	lncRNA	0.33605649	0.36980058	0.52317226
ENSG0000016	SIGLEC11	protein_codir	0.33600702	0.46505238	0.61491735
ENSG0000010	SGK3	protein_codir	0.33592578	0.04802613	0.11675749
ENSG0000016	EGFLAM	protein_codir	0.33580153	0.23407863	0.37740073
ENSG0000021	CCDC7	protein_codir	0.33576655	0.15243596	0.27557804
ENSG0000024	SNHG3	lncRNA	0.33563802	0.1908232	0.32538194
ENSG0000013	SLC40A1	protein_codir	0.33561618	0.34953534	0.50300453
ENSG0000013	PTPRQ	protein_codir	0.33538615	0.47115296	0.62015082
ENSG0000014	TNIP1	protein_codir	0.33533427	0.00566418	0.02232033
ENSG0000019	KB-1592A4.15	lncRNA	0.33492202	0.72466389	0.82235165
ENSG0000017	SPSB4	protein_codir	0.33485905	0.51019737	0.65422838
ENSG0000011	IL26	protein_codir	0.33467136	0.88545777	NA
ENSG0000010	EIF3E	protein_codir	0.33463849	0.00335439	0.01477409
ENSG0000023	SMIM27	protein_codir	0.33459644	0.08548927	0.17941357
ENSG0000027	RP11-332H14	lncRNA	0.33451182	0.53780986	0.67747461

ENSG0000025	AP000438.2	lncRNA	0.33403438	0.90741798	NA
ENSG0000019	PRPF40A	protein_codir	0.33391343	7.54E-05	0.0006886
ENSG0000023	TTN-AS1	lncRNA	0.33378447	0.06293671	0.14331866
ENSG0000014	G3BP1	protein_codir	0.33366028	0.01502447	0.04778625
ENSG0000017	LDLRAD3	protein_codir	0.33335886	0.23780602	0.38189442
ENSG0000012	TBCC	protein_codir	0.33331288	0.10020076	0.20204009
ENSG0000015	SFR1	protein_codir	0.33329773	0.00810027	0.02951698
ENSG0000022	FAM174C	protein_codir	0.33324912	0.06795989	0.15186056
ENSG0000008	PXN	protein_codir	0.33308525	0.00896317	0.03194532
ENSG0000016	DBF4B	protein_codir	0.33303945	0.26165437	0.4090849
ENSG0000013	CYP2C8	protein_codir	0.33299274	0.55286364	0.68937374
ENSG0000011	DENND10	protein_codir	0.33290318	0.00270095	0.01242507
ENSG0000004	PHLPP2	protein_codir	0.33285788	0.04369546	0.10862903
ENSG0000013	FAM13A	protein_codir	0.33276045	0.06601107	0.14871337
ENSG0000019	ZNF121	protein_codir	0.33273683	0.08724024	0.18203964
ENSG0000012	APOL2	protein_codir	0.33248369	0.06939259	0.15399514
ENSG0000010	CEP76	protein_codir	0.33234432	0.02108196	0.06176112
ENSG0000011	PAPOLG	protein_codir	0.33231606	0.03056456	0.0826425
ENSG0000027	CTAGE6	protein_codir	0.33231286	0.74562018	0.83756424
ENSG0000025	RP11-867G23	lncRNA	0.33225174	0.88624013	NA
ENSG0000012	ATP6V1F	protein_codir	0.33178724	0.01951332	0.05820628
ENSG0000012	OARD1	protein_codir	0.33155666	0.0108216	0.03707883
ENSG0000018	SLC2A4	protein_codir	0.33153589	0.66599975	0.77927283
ENSG0000010	HERC1	protein_codir	0.33121362	0.01665684	0.05161117
ENSG0000026	TMEM220-AS	lncRNA	0.33117788	0.28381442	0.43340182
ENSG0000018	LIN54	protein_codir	0.33117159	0.0327509	0.08701406
ENSG0000016	NCKAP5L	protein_codir	0.33113038	0.05137666	0.1228698
ENSG0000010	GSDMD	protein_codir	0.33104627	0.07828801	0.16793373
ENSG0000018	TMEM259	protein_codir	0.33089262	0.12678744	0.24076102
ENSG0000010	STAG2	protein_codir	0.33084946	0.04987781	0.12032712
ENSG0000010	PLA2G15	protein_codir	0.33038687	0.24125145	0.38590172
ENSG0000018	ZNF395	protein_codir	0.3303261	0.11868635	0.22909819
ENSG0000012	MT1G	protein_codir	0.3302355	0.72803566	0.82502543
ENSG0000018	TTC32	protein_codir	0.32991759	0.27463277	0.42270297
ENSG0000012	PUS7L	protein_codir	0.32980876	0.11517649	0.22405794
ENSG0000015	TDRD9	protein_codir	0.32968844	0.49575381	0.6416621
ENSG0000020	OXLD1	protein_codir	0.32956014	0.16227455	0.2886042
ENSG0000028	AC005618.10	lncRNA	0.32944943	0.6529325	0.76871984
ENSG0000014	CBWD5	protein_codir	0.32940535	0.1807425	0.31235815
ENSG0000023	RP1-92O14.3	lncRNA	0.3293882	0.60559422	0.73201317
ENSG0000022	AC104653.1	lncRNA	0.32909751	0.54644751	0.68432919
ENSG0000017	ORAI3	protein_codir	0.32890202	0.06338133	0.14409392
ENSG0000016	BRINP3	protein_codir	0.32845108	0.5156452	0.6588389
ENSG0000020	SOX18	protein_codir	0.32840308	0.32646114	0.4787542
ENSG0000026	RP11-73M7.9	lncRNA	0.32830285	0.8287293	NA
ENSG0000017	MTHFR	protein_codir	0.32812979	0.02318196	0.06650937
ENSG0000012	MASTL	protein_codir	0.32800337	0.04552737	0.11221362

ENSG0000015	FCHO2	protein_codir	0.32798694	0.10134554	0.20379787
ENSG0000011	SRSF7	protein_codir	0.3278614	0.10404739	0.20785397
ENSG0000014	GFOD1	protein_codir	0.32766492	0.19559294	0.33136705
ENSG0000028	RP5-971C3.1	lncRNA	0.3276529	0.56643794	0.70049032
ENSG0000014	RNF145	protein_codir	0.3273493	0.01347256	0.04384495
ENSG0000006	IDI1	protein_codir	0.32731878	0.06838485	0.15239296
ENSG0000014	RETREG3	protein_codir	0.32722728	0.01675691	0.05186887
ENSG0000027	CTD-2553L13.	lncRNA	0.32699154	0.31599201	0.46774743
ENSG0000013	DCAF7	protein_codir	0.32682408	0.01560343	0.04912856
ENSG0000021	POLR2J4	lncRNA	0.32647971	0.1515649	0.27433446
ENSG0000010	CDK6	protein_codir	0.32647761	0.22788317	0.37019026
ENSG0000027	RP11-574K11.	protein_codir	0.32647294	0.2408681	0.38540013
ENSG0000019	NTRK1	protein_codir	0.32646207	0.54429868	0.68253561
ENSG0000019	OCLN	protein_codir	0.32645253	0.4394578	0.59046492
ENSG0000013	C11orf1	protein_codir	0.32643586	0.04352288	0.10831699
ENSG0000017	ATF7IP	protein_codir	0.32599025	0.16761736	0.29591884
ENSG0000016	SNRNP25	protein_codir	0.32588759	0.08512986	0.1787643
ENSG0000016	COL1A2	protein_codir	0.32554571	0.39225244	0.54522967
ENSG0000015	CNNM4	protein_codir	0.3255201	0.03280564	0.08713258
ENSG0000017	ANKLE2	protein_codir	0.32548028	0.01500903	0.04776616
ENSG0000022	SMCR2	lncRNA	0.32542752	0.86846652	NA
ENSG0000018	CALN1	protein_codir	0.32526429	0.66810611	0.7807197
ENSG0000012	UBL3	protein_codir	0.32512034	0.10208407	0.20492492
ENSG0000022	OR2A1	protein_codir	0.32511562	0.6811329	0.79034734
ENSG0000015	ENPP3	protein_codir	0.32508765	0.33055849	0.48312126
ENSG0000025	MAILR	lncRNA	0.32496113	0.3383066	0.49114472
ENSG0000027	RP11-254F19.	lncRNA	0.32481696	0.68304898	0.79161685
ENSG0000013	DUSP9	protein_codir	0.32472978	0.81600469	0.88676619
ENSG0000025	MAGEL2	protein_codir	0.32466298	0.59487057	0.72338859
ENSG0000017	ADCK5	protein_codir	0.32464127	0.21699115	0.35725415
ENSG0000010	NMRK1	protein_codir	0.32455849	0.09865819	0.19967314
ENSG0000003	BOD1L1	protein_codir	0.32455821	8.31E-05	0.00074793
ENSG0000017	RPS21	protein_codir	0.32442193	0.09605559	0.19568191
ENSG0000019	APRT	protein_codir	0.3244208	0.02912159	0.07969269
ENSG0000013	REPS1	protein_codir	0.32426593	2.44E-07	5.41E-06
ENSG0000019	AADACL2	protein_codir	0.32426404	0.79400499	0.87201947
ENSG0000009	MSH2	protein_codir	0.3241707	0.17906616	0.31021811
ENSG0000027	KB-208E9.1	lncRNA	0.3240443	0.61101341	0.73630848
ENSG0000019	ZNF33B	protein_codir	0.32401471	0.06941331	0.15402873
ENSG0000012	EGR1	protein_codir	0.32355969	0.41722183	0.56994247
ENSG0000011	CLINT1	protein_codir	0.32354951	0.00022089	0.00167609
ENSG0000024	UBE2V1	protein_codir	0.32354535	0.00074821	0.00453788
ENSG0000020	PRR13	protein_codir	0.32351875	0.00904154	0.03218551
ENSG0000027	CTD-3035K23	lncRNA	0.32348774	0.76675971	0.85295031
ENSG0000011	PHF3	protein_codir	0.32340363	0.0479064	0.11649716
ENSG0000013	NDUFB5	protein_codir	0.32335198	0.00658512	0.02508702
ENSG0000018	PLSCR1	protein_codir	0.32331928	0.16860174	0.2970264

ENSG0000014	RPL10	protein_codir	0.32329379	0.0111892	0.03803639
ENSG0000019	SPOCK3	protein_codir	0.32326698	0.73985695	0.83363313
ENSG0000017	OR2M4	protein_codir	0.32322263	0.92109157	NA
ENSG0000027	RP11-481J13.	lncRNA	0.32322263	0.92109157	NA
ENSG0000018	ASB18	protein_codir	0.32322263	0.92109157	NA
ENSG0000028	RP11-816B4.3	lncRNA	0.32322263	0.92109157	NA
ENSG0000025	LINC02435	lncRNA	0.32322263	0.92109157	NA
ENSG0000025	LY6G6F-LY6G(	protein_codir	0.32322263	0.92109157	NA
ENSG0000025	RP11-300A12	lncRNA	0.32322263	0.92109157	NA
ENSG0000026	RP11-356C4.5	lncRNA	0.32322263	0.92109157	NA
ENSG0000028	RP1-148E22.2	lncRNA	0.32322263	0.92109157	NA
ENSG0000015	PGLYRP3	protein_codir	0.32322263	0.92109157	NA
ENSG0000025	CTD-2089N3.:	lncRNA	0.32322263	0.92109157	NA
ENSG0000022	LINC02247	lncRNA	0.32322263	0.92109157	NA
ENSG0000027	RP11-87C12.6	lncRNA	0.32322263	0.92109157	NA
ENSG0000025	RP11-326A13	lncRNA	0.32322263	0.92109157	NA
ENSG0000026	CTD-2529O21	lncRNA	0.32322263	0.92109157	NA
ENSG0000018	TEX19	protein_codir	0.32322263	0.92109157	NA
ENSG0000018	ANKRD62	protein_codir	0.32322263	0.92109157	NA
ENSG0000026	RP11-794M8.	lncRNA	0.32322263	0.92109157	NA
ENSG0000027	RP11-2N1.3	lncRNA	0.32322263	0.92109157	NA
ENSG0000028	LL22NC01-132	lncRNA	0.32322263	0.92109157	NA
ENSG0000028	RP1-167O5.3	lncRNA	0.32322248	0.91847468	NA
ENSG0000027	CTC-492K19.7	lncRNA	0.32322244	0.91773665	NA
ENSG0000022	TH2LCRR	lncRNA	0.32322237	0.91647804	NA
ENSG0000026	RP11-352G18	lncRNA	0.32322236	0.91629831	NA
ENSG0000025	RP11-708L7.6	lncRNA	0.32322236	0.91625311	NA
ENSG0000026	RP11-26L20.4	lncRNA	0.32322236	0.91623769	NA
ENSG0000016	TEKT4	protein_codir	0.32322234	0.91584904	NA
ENSG0000018	LRRC74B	protein_codir	0.32322233	0.91577986	NA
ENSG0000025	RP11-495O10	lncRNA	0.32322233	0.91565724	NA
ENSG0000013	G6PC1	protein_codir	0.32322232	0.91555997	NA
ENSG0000026	RP11-1055B8.	lncRNA	0.32322231	0.91531126	NA
ENSG0000028	RP11-19B4.2	lncRNA	0.3232223	0.91518868	NA
ENSG0000024	RP11-556N21	lncRNA	0.32322229	0.91498818	NA
ENSG0000027	CMP21-97G8.	lncRNA	0.32322229	0.91494275	NA
ENSG0000022	ADGRF5-AS1	lncRNA	0.32322227	0.91459495	NA
ENSG0000023	RP5-827C21.2	lncRNA	0.32322227	0.91451876	NA
ENSG0000022	AC023137.2	lncRNA	0.32322226	0.91433068	NA
ENSG0000025	PCAT4	lncRNA	0.32322226	0.91421837	NA
ENSG0000027	RP11-236B18.	lncRNA	0.32322225	0.9140361	NA
ENSG0000023	RP11-316M21	lncRNA	0.32322224	0.91398983	NA
ENSG0000026	CTD-2199O4.3	lncRNA	0.32322224	0.9138942	NA
ENSG0000027	RP11-1123I8.:	lncRNA	0.32322224	0.91385634	NA
ENSG0000022	AC026188.1	lncRNA	0.32322223	0.91377216	NA
ENSG0000025	IRAIN	lncRNA	0.32322223	0.91377216	NA
ENSG0000023	AC002511.3	lncRNA	0.32322223	0.91371772	NA

ENSG0000003	BEST2	protein_codir	0.32322223	0.91368933	NA
ENSG0000026	SPDYE9	protein_codir	0.32322223	0.9136244	NA
ENSG0000014	ASB10	protein_codir	0.32322222	0.91355323	NA
ENSG0000018	OTOG	protein_codir	0.32322222	0.91352755	NA
ENSG0000028	RP11-133M9.	lncRNA	0.32322222	0.91351664	NA
ENSG0000014	HTR3B	protein_codir	0.32322222	0.91351664	NA
ENSG0000025	RP11-856F16.	lncRNA	0.32322221	0.91335642	NA
ENSG0000022	LHFPL3-AS1	lncRNA	0.32322221	0.91328245	NA
ENSG0000023	MEIOSIN	protein_codir	0.32322221	0.9132034	NA
ENSG0000025	RP11-421P23.	lncRNA	0.3232222	0.91310505	NA
ENSG0000027	TMEM271	protein_codir	0.3232222	0.91307678	NA
ENSG0000027	RP11-379K22.	lncRNA	0.3232222	0.91300081	NA
ENSG0000025	RP11-425A23	lncRNA	0.3232222	0.91296085	NA
ENSG0000025	FRMD6-AS2	lncRNA	0.32322218	0.91269743	NA
ENSG0000010	CRX	protein_codir	0.32322218	0.91256976	NA
ENSG0000025	RP11-348J24.	lncRNA	0.32322218	0.91253421	NA
ENSG0000026	LA16c-329F2.	lncRNA	0.32322217	0.9123736	NA
ENSG0000020	SMIM9	protein_codir	0.32322216	0.91226528	NA
ENSG0000028	RP4-705O1.2	lncRNA	0.32322216	0.91224583	NA
ENSG0000023	AC003986.7	lncRNA	0.32322216	0.91216636	NA
ENSG0000023	MLIP-IT1	lncRNA	0.32322214	0.91180811	NA
ENSG0000026	RP11-18F14.4	lncRNA	0.32322214	0.91176382	NA
ENSG0000026	LINC01978	lncRNA	0.32322214	0.91176337	NA
ENSG0000026	CTD-3193O13	lncRNA	0.32322214	0.91162407	NA
ENSG0000018	LHFPL3	protein_codir	0.32322211	0.91113249	NA
ENSG0000023	RPS6KA2-IT1	lncRNA	0.3232221	0.91071963	NA
ENSG0000018	KRTAP13-4	protein_codir	0.32322209	0.91058128	NA
ENSG0000024	RP11-61G19.1	lncRNA	0.32322207	0.91012554	NA
ENSG0000017	SPACA5	protein_codir	0.32322204	0.90928376	NA
ENSG0000022	AP000221.1	lncRNA	0.32322155	0.89429469	NA
ENSG0000024	CTD-2249K22	lncRNA	0.32322155	0.89418214	NA
ENSG0000024	RP11-651P23.	lncRNA	0.32322155	0.89407824	NA
ENSG0000025	LINC02392	lncRNA	0.32322154	0.89399099	NA
ENSG0000027	CTA-268H5.12	lncRNA	0.32322153	0.89355668	NA
ENSG0000023	AC069155.1	lncRNA	0.32322152	0.89318594	NA
ENSG0000023	LINC02590	lncRNA	0.32322152	0.89315775	NA
ENSG0000026	CTD-2636A23	lncRNA	0.32322151	0.89284817	NA
ENSG0000028	RP3-508I15.2	lncRNA	0.32322151	0.89280357	NA
ENSG0000014	ABCG8	protein_codir	0.32322151	0.89261786	NA
ENSG0000025	CTC-564N23.2	lncRNA	0.3232215	0.89235094	NA
ENSG0000026	RP11-264B17.	lncRNA	0.3232215	0.89223906	NA
ENSG0000017	KRT13	protein_codir	0.32322149	0.89183581	NA
ENSG0000027	RP11-85E16.1	lncRNA	0.32322148	0.8915235	NA
ENSG0000025	CTC-806A22.1	lncRNA	0.32322148	0.89125302	NA
ENSG0000023	RP11-187A9.3	lncRNA	0.32322147	0.89102046	NA
ENSG0000023	RMDN2-AS1	lncRNA	0.32322147	0.8909371	NA
ENSG0000000	NOX1	protein_codir	0.32322147	0.89089875	NA

ENSG0000028	AP001436.1	lncRNA	0.32322146	0.89052852	NA
ENSG0000026	LINC01070	lncRNA	0.32322146	0.89045807	NA
ENSG0000025	CTD-2647L4.5	lncRNA	0.32322141	0.88820382	NA
ENSG0000018	NKX2-6	protein_codir	0.32322141	0.88815089	NA
ENSG0000017	IGSF22	protein_codir	0.32295676	0.38418257	0.53748594
ENSG0000010	PPP1R9B	protein_codir	0.322781	0.02718007	0.0753048
ENSG0000015	MMS19	protein_codir	0.32275883	0.03460959	0.09079758
ENSG0000012	NAGK	protein_codir	0.32260411	0.16141957	0.28752796
ENSG0000018	RNPC3	protein_codir	0.32232959	0.20356069	0.34089288
ENSG0000011	MIIP	protein_codir	0.32210273	0.15442164	0.27829347
ENSG0000022	C10orf55	lncRNA	0.32203421	0.43236933	0.58364894
ENSG0000017	NR1D2	protein_codir	0.32193873	0.31269806	0.46441196
ENSG0000024	STON1	protein_codir	0.32175542	0.44951776	0.60034237
ENSG0000010	ECH1	protein_codir	0.32151757	0.0024165	0.01139165
ENSG0000017	VN1R1	protein_codir	0.32151158	0.36875618	0.52228325
ENSG0000027	RP11-771K4.3	lncRNA	0.3214827	0.55607959	0.69194934
ENSG0000006	SUGP2	protein_codir	0.32128086	0.16709081	0.29521194
ENSG0000027	RP11-262A16	lncRNA	0.32111672	0.29076798	0.44049589
ENSG0000024	AC051649.12	lncRNA	0.32104691	0.85372516	0.91285717
ENSG0000015	C1R	protein_codir	0.32098196	0.25871772	0.405849
ENSG0000027	CTD-2349P21	lncRNA	0.32089015	0.72508327	0.82279375
ENSG0000010	CLCN7	protein_codir	0.32083839	0.05864927	0.13555588
ENSG0000028	EBLN3P	lncRNA	0.32083417	0.01880526	0.0565735
ENSG0000027	ENSG0000027	protein_codir	0.32081724	0.33137058	0.48409032
ENSG0000000	RHBDD2	protein_codir	0.32075072	0.03074337	0.08295166
ENSG0000015	MITD1	protein_codir	0.32060558	0.10437417	0.20829942
ENSG0000019	CD2AP	protein_codir	0.32051761	0.03937224	0.10031004
ENSG0000014	DIS3L2	protein_codir	0.32047043	0.02163676	0.06299222
ENSG0000017	ZNF791	protein_codir	0.32040418	0.05732198	0.1332015
ENSG0000009	SLC7A8	protein_codir	0.32031746	0.25597228	0.40268541
ENSG0000019	KCNRG	protein_codir	0.32022257	0.52920772	0.67069606
ENSG0000028	RP11-213H15	lncRNA	0.32004533	0.60466457	0.73114594
ENSG0000003	ZFYVE16	protein_codir	0.32003076	0.04207693	0.10561908
ENSG0000018	ZNF708	protein_codir	0.3200011	0.1934285	0.3287487
ENSG0000027	RP11-434H6.7	lncRNA	0.31992456	0.54348845	0.68195308
ENSG0000022	RP11-276E15.	lncRNA	0.31976987	0.73034081	0.82650811
ENSG0000008	SULT2B1	protein_codir	0.31976031	0.75985571	0.84811272
ENSG0000027	RP11-131L12.	lncRNA	0.31972828	0.40529239	0.55820164
ENSG0000012	YEATS4	protein_codir	0.319713	0.05985276	0.13769227
ENSG0000017	ZNF738	protein_codir	0.31965174	0.21597888	0.35624585
ENSG0000010	METTL4	protein_codir	0.31963688	0.0752788	0.16338209
ENSG0000016	CDK12	protein_codir	0.31963255	0.02355346	0.0672956
ENSG0000018	TRAK1	protein_codir	0.3195468	0.00781141	0.02866744
ENSG0000017	PLEKHF2	protein_codir	0.31944379	0.24625467	0.39154649
ENSG0000001	HHATL	protein_codir	0.31944315	0.73414033	0.82952692
ENSG0000019	ZNF772	protein_codir	0.31940939	0.33843292	0.49127647
ENSG0000022	AC092614.2	lncRNA	0.31939652	0.51625534	0.65940494

ENSG0000008	NOP14	protein_codir	0.3193141	0.02345571	0.0670857
ENSG0000010	TOM1	protein_codir	0.31917193	0.05446812	0.12830785
ENSG0000025	CTSO	protein_codir	0.31900721	0.09620529	0.19591462
ENSG0000023	DAAM2-AS1	lncRNA	0.31896374	0.34487992	0.49789376
ENSG0000027	RP11-7F17.8	lncRNA	0.31891473	0.11751818	0.22739612
ENSG0000014	SETDB1	protein_codir	0.31889048	0.04603072	0.11301054
ENSG0000011	KDM3A	protein_codir	0.31884226	0.02781617	0.07675151
ENSG0000021	CFAP45	protein_codir	0.318688	0.44125626	0.59222984
ENSG0000028	RP11-485M7.	lncRNA	0.31866297	0.56962758	0.70328075
ENSG0000016	ZNF589	protein_codir	0.31864388	0.27258839	0.42048023
ENSG0000010	SLC2A9	protein_codir	0.31858001	0.26127542	0.4086312
ENSG0000014	RFX5	protein_codir	0.31852872	0.06590171	0.14852755
ENSG0000025	RP11-108O10	protein_codir	0.31805243	0.72943409	0.82603402
ENSG0000013	TBC1D14	protein_codir	0.31801529	0.08079989	0.17196074
ENSG0000005	ATP11B	protein_codir	0.31773043	0.08919472	0.18500737
ENSG0000027	ENSG0000027	protein_codir	0.31760074	0.59563345	0.72399753
ENSG0000010	PRODH	protein_codir	0.31742318	0.61537681	0.73934088
ENSG0000026	RP11-174G6.5	lncRNA	0.31742312	0.42036787	0.57259756
ENSG0000008	COL5A3	protein_codir	0.31741213	0.33283405	0.48557344
ENSG0000014	OSBPL11	protein_codir	0.31740826	0.07949683	0.16986713
ENSG0000022	LINC01537	lncRNA	0.31705415	0.81587394	0.88669388
ENSG0000028	RP11-490F3.3	lncRNA	0.31701683	0.64237407	0.76036563
ENSG0000026	SF1-DT	lncRNA	0.31701269	0.53569193	0.67592176
ENSG0000008	CYLD	protein_codir	0.31686069	0.13557935	0.25269597
ENSG0000018	FBXL6	protein_codir	0.3168121	0.17498813	0.30510525
ENSG0000023	ZKSCAN7-AS1	lncRNA	0.31674448	0.80446374	0.87875375
ENSG0000025	RP4-785G19.5	lncRNA	0.31668651	0.75551806	0.84493698
ENSG0000008	FAM135A	protein_codir	0.31658077	0.01705217	0.05251208
ENSG0000017	ZNF274	protein_codir	0.31645818	0.08570129	0.17972901
ENSG0000018	H3C13	protein_codir	0.3163585	0.75904484	0.84754279
ENSG0000008	AKR1B1	protein_codir	0.31606378	0.07675009	0.16562523
ENSG0000028	RP11-744C22.	lncRNA	0.31598206	0.75761788	0.84659975
ENSG0000015	KCNJ1	protein_codir	0.315935	0.67799047	0.78800965
ENSG0000027	RP11-775C24.	lncRNA	0.31588538	0.3322179	0.48498276
ENSG0000022	UBAC2-AS1	lncRNA	0.31554973	0.38204121	0.53552467
ENSG0000017	POLD4	protein_codir	0.31552591	0.2097204	0.34888137
ENSG0000020	ZNF783	protein_codir	0.31547118	0.04574816	0.11250682
ENSG0000015	HYDIN	protein_codir	0.31531586	0.54278447	0.68140669
ENSG0000013	NUP58	protein_codir	0.31529595	0.05435634	0.12814288
ENSG0000016	MIR202HG	lncRNA	0.31521367	0.80088461	0.87645264
ENSG0000008	RPS5	protein_codir	0.31509169	0.06747288	0.15111157
ENSG0000013	KYAT3	protein_codir	0.31508474	0.00105801	0.00598904
ENSG0000014	LYSMD2	protein_codir	0.31502734	0.06743031	0.15107276
ENSG0000027	CHASERR	lncRNA	0.31476313	0.02701256	0.07495348
ENSG0000021	ZSWIM7	protein_codir	0.31448916	0.08560301	0.17958136
ENSG0000012	SDCBP2	protein_codir	0.31439587	0.26469618	0.41223053
ENSG0000010	PLD3	protein_codir	0.31435598	0.09469785	0.1936874

ENSG0000022	TIGD1	protein_codir	0.31428411	0.21832972	0.35879495
ENSG0000018	ADAT2	protein_codir	0.31426491	0.34643532	0.49949225
ENSG0000016	KCNT2	protein_codir	0.3142231	0.36730723	0.52108007
ENSG0000017	SHISA3	protein_codir	0.3142112	0.47853526	0.62638548
ENSG0000020	CTD-2026K11	lncRNA	0.31418638	0.4904436	0.63722139
ENSG0000025	AC100830.3	lncRNA	0.31418366	0.56481228	0.69911783
ENSG0000013	IL15RA	protein_codir	0.31413323	0.05063943	0.12171796
ENSG0000016	ADAR	protein_codir	0.3139946	0.03190956	0.08522016
ENSG0000012	RPS10	protein_codir	0.31393459	0.07957407	0.16996643
ENSG0000000	RBM5	protein_codir	0.31373963	0.16808366	0.29647301
ENSG0000022	TNS1-AS1	lncRNA	0.31373933	0.7007505	0.80497125
ENSG0000022	LINC01504	lncRNA	0.31368404	0.56058875	0.69560453
ENSG0000010	BBC3	protein_codir	0.31361407	0.23379101	0.37706906
ENSG0000026	RP11-425D10	lncRNA	0.31351527	0.57399697	0.70671762
ENSG0000013	RIN2	protein_codir	0.31342346	0.21103969	0.35040073
ENSG0000027	RP11-91K8.5	lncRNA	0.31334263	0.85693098	NA
ENSG0000028	RP11-206L10.	lncRNA	0.31330688	0.76214774	0.84983934
ENSG0000021	ZNF254	protein_codir	0.31326923	0.06235693	0.14223291
ENSG0000016	TMEM41B	protein_codir	0.3132103	0.00461174	0.01907277
ENSG0000016	TMEM169	protein_codir	0.31285448	0.44276371	0.59384906
ENSG0000023	THAP7-AS1	lncRNA	0.31283033	0.16836386	0.29676808
ENSG0000018	NAP1L1	protein_codir	0.31267006	0.00537183	0.02145314
ENSG0000017	PHLDB3	protein_codir	0.312372	0.05472691	0.12873081
ENSG0000014	RNPEPL1	protein_codir	0.31231189	0.10859447	0.21465079
ENSG0000014	NOTCH1	protein_codir	0.31206546	0.04049331	0.10246116
ENSG0000016	ZNF474	protein_codir	0.31191812	0.53749994	0.67730602
ENSG0000023	TMEM253	protein_codir	0.31177293	0.60792741	0.73380627
ENSG0000014	FEM1C	protein_codir	0.31165522	0.24974743	0.39564233
ENSG0000018	MRTFB	protein_codir	0.31148266	0.08987075	0.18599056
ENSG0000011	KDM5B	protein_codir	0.31121945	0.02964854	0.08077175
ENSG0000026	RP13-638C3.2	lncRNA	0.31115535	0.57750409	0.70977007
ENSG0000028	DERPC	protein_codir	0.31107538	0.19843482	0.33475141
ENSG0000023	XX-C2158C12.	lncRNA	0.31098713	0.59969207	0.72726652
ENSG0000026	RP11-366L5.1	lncRNA	0.31082844	0.40050868	0.5537906
ENSG0000016	KLK13	protein_codir	0.31073702	0.80967137	0.88241815
ENSG0000011	EXOC8	protein_codir	0.31056541	0.10384902	0.20756277
ENSG0000026	LA16c-349E10	lncRNA	0.31046335	0.54292246	0.68149056
ENSG0000010	BRD1	protein_codir	0.31035644	0.00139476	0.00744121
ENSG0000019	ZNF652	protein_codir	0.31034518	0.03020768	0.08190205
ENSG0000017	PIGG	protein_codir	0.31008954	0.05951048	0.13707611
ENSG0000016	COQ4	protein_codir	0.3100347	0.06669977	0.14987371
ENSG0000013	RBM39	protein_codir	0.30968003	0.07360322	0.16081964
ENSG0000018	PCGF3	protein_codir	0.30957783	0.07359183	0.16080746
ENSG0000022	ZNF451-AS1	lncRNA	0.30943442	0.67484813	0.78577232
ENSG0000017	POLE	protein_codir	0.30932069	0.16267555	0.28911258
ENSG0000006	SRBD1	protein_codir	0.30923443	0.06149259	0.14067965
ENSG0000004	CLPTM1L	protein_codir	0.30920703	0.09735815	0.19776657

ENSG0000006	TGFBR3	protein_codir	0.30919688	0.42723263	0.57897871
ENSG0000023	LINC01968	lncRNA	0.30898635	0.84253026	0.90610878
ENSG0000012	HPCA	protein_codir	0.30871407	0.6308438	0.75151383
ENSG0000025	RP11-691N7.6	protein_codir	0.30864397	0.88168238	0.9298659
ENSG0000013	TPP2	protein_codir	0.30862601	0.0040322	0.0171364
ENSG0000012	SGPP1	protein_codir	0.30861851	0.06914288	0.15356436
ENSG0000008	WDR70	protein_codir	0.3085538	0.00014969	0.00122509
ENSG0000017	DLEU1	lncRNA	0.3084384	0.18033918	0.31187579
ENSG0000015	MIA2	protein_codir	0.30840871	0.00274303	0.01256843
ENSG0000018	SMYD4	protein_codir	0.30836239	0.07850653	0.16830586
ENSG0000026	RP11-20I23.8	lncRNA	0.30829942	0.26395611	0.41141233
ENSG0000014	PTRH2	protein_codir	0.30827381	0.08058162	0.1716539
ENSG0000026	RP11-626G11	lncRNA	0.30819732	0.80806597	0.88122486
ENSG0000013	UACA	protein_codir	0.30808724	0.26946265	0.41730072
ENSG0000007	SLC24A1	protein_codir	0.30786833	0.0603292	0.13860361
ENSG0000021	ATP6V1G2	protein_codir	0.30786551	0.35031615	0.50389189
ENSG0000025	MGC15885	lncRNA	0.30783417	0.92481919	NA
ENSG0000025	RP11-455F5.3	lncRNA	0.30779948	0.34807237	0.50138723
ENSG0000017	NSG2	protein_codir	0.3076955	0.7812782	0.86317659
ENSG0000014	C1orf131	protein_codir	0.30764955	0.11113488	0.21839939
ENSG0000017	FKBP2	protein_codir	0.30764444	0.18570797	0.3190033
ENSG0000018	RBM11	protein_codir	0.30722305	0.48081778	0.62846942
ENSG0000016	MTNR1A	protein_codir	0.30699566	0.91440978	NA
ENSG0000013	APPL2	protein_codir	0.30695171	0.00822262	0.02985133
ENSG0000019	OR6Y1	protein_codir	0.30694713	0.91384632	NA
ENSG0000027	RP11-321P16.	lncRNA	0.30689656	0.91326312	NA
ENSG0000027	RP5-1016A21	lncRNA	0.30684725	0.9126984	NA
ENSG0000010	CHN2	protein_codir	0.30684497	0.23345314	0.37670011
ENSG0000028	RP4-736L20.5	lncRNA	0.30683034	0.47144451	0.62029808
ENSG0000023	MYCL-AS1	lncRNA	0.30681311	0.91230954	NA
ENSG0000024	MIR1302-2HG	lncRNA	0.30679754	0.91213274	NA
ENSG0000016	ZMAT1	protein_codir	0.30672704	0.32832511	0.48069651
ENSG0000021	SPINK13	protein_codir	0.3067208	0.91126664	NA
ENSG0000018	PRKAR1B	protein_codir	0.30665907	0.1980015	0.33421754
ENSG0000016	STX5	protein_codir	0.30664979	0.03687636	0.0952694
ENSG0000025	RP11-554D14	lncRNA	0.30663445	0.64822553	0.76469975
ENSG0000019	ZNF43	protein_codir	0.30650619	0.23645399	0.3803867
ENSG0000026	NCOA4	protein_codir	0.30649726	0.09344975	0.19175944
ENSG0000015	C12orf45	protein_codir	0.30637526	0.10699655	0.21223701
ENSG0000013	ARGLU1	protein_codir	0.30631327	0.20623716	0.34435197
ENSG0000026	SNAI3-AS1	lncRNA	0.306237	0.26031474	0.4075441
ENSG0000015	DGKE	protein_codir	0.30611981	0.05519194	0.12951584
ENSG0000013	KL	protein_codir	0.30610674	0.33693927	0.48992921
ENSG0000018	OR6A2	protein_codir	0.30599476	0.82064626	NA
ENSG0000015	ANAPC1	protein_codir	0.30587322	0.0483125	0.11730911
ENSG0000016	EFNA1	protein_codir	0.30577001	0.29396507	0.44426106
ENSG0000028	GS1-114I9.3	protein_codir	0.30573304	0.82045854	0.89003043

ENSG0000001	PGM3	protein_codir	0.30570961	0.08871305	0.1843064
ENSG0000008	NECAP1	protein_codir	0.30563358	0.0241751	0.06870922
ENSG0000013	PREB	protein_codir	0.30551697	0.19426069	0.32975725
ENSG0000025	RP11-73M18.	protein_codir	0.30544394	0.86196142	0.91771546
ENSG0000012	RNF114	protein_codir	0.30534407	0.00114503	0.00636334
ENSG0000017	GLB1	protein_codir	0.30534406	0.05384626	0.12725552
ENSG0000023	PSG1	protein_codir	0.30517325	0.89534848	NA
ENSG0000023	LINC01361	lncRNA	0.30515489	0.89517446	NA
ENSG0000028	RP11-964E11.	lncRNA	0.3051192	0.89483689	NA
ENSG0000025	LINC02749	lncRNA	0.30509744	0.89463165	NA
ENSG0000025	CTD-2339F6.1	lncRNA	0.30506299	0.69088595	0.79712059
ENSG0000025	UVRAG-DT	lncRNA	0.30501353	0.72411718	0.8219608
ENSG0000028	RP11-182E21.	lncRNA	0.30500538	0.89376789	NA
ENSG0000001	SNAI2	protein_codir	0.30498362	0.2625333	0.41001808
ENSG0000016	XPO6	protein_codir	0.30486184	0.03671865	0.09496854
ENSG0000018	ZDHHC23	protein_codir	0.30481757	0.26863572	0.41635185
ENSG0000026	AC137932.4	lncRNA	0.30477511	0.46935287	0.61863643
ENSG0000015	ZNF773	protein_codir	0.30470592	0.1797804	0.31112372
ENSG0000025	RP11-328C8.4	lncRNA	0.30458971	0.61437762	0.73851363
ENSG0000015	GABRA2	protein_codir	0.30455647	0.54546917	0.68344486
ENSG0000025	RP11-701P16.	lncRNA	0.3044937	0.7952758	0.8728793
ENSG0000015	GPAT4	protein_codir	0.30445254	0.00043741	0.00292951
ENSG0000027	RP11-216L13.	lncRNA	0.30437457	0.28955149	0.43915283
ENSG0000010	CLASRP	protein_codir	0.30423442	0.22222317	0.36337718
ENSG0000013	TRPC6	protein_codir	0.30405181	0.29019138	0.43987916
ENSG0000016	ZNF668	protein_codir	0.30383457	0.05770993	0.13383277
ENSG0000025	RP11-111A21	lncRNA	0.30378041	0.49254347	0.63910789
ENSG0000019	ZNF808	protein_codir	0.30376693	0.13297147	0.24941746
ENSG0000010	BLOC1S6	protein_codir	0.30359559	0.01812327	0.05494674
ENSG0000026	RP11-363E7.4	lncRNA	0.30330143	0.32627001	0.47855014
ENSG0000018	LINC00994	lncRNA	0.30323944	0.82462007	NA
ENSG0000028	CTD-2117H17	lncRNA	0.30322166	0.65723732	0.77211947
ENSG0000018	D2HGDH	protein_codir	0.30320675	0.30946412	0.46114912
ENSG0000011	SSR3	protein_codir	0.30307891	0.05262004	0.12505296
ENSG0000013	DAP3	protein_codir	0.30300396	0.00098103	0.00564146
ENSG0000015	SETBP1	protein_codir	0.30264493	0.20462151	0.3422958
ENSG0000010	SNRNP70	protein_codir	0.30259611	0.29535313	0.44571421
ENSG0000025	RP11-168L7.1	lncRNA	0.30258848	0.87322738	NA
ENSG0000015	FARP1	protein_codir	0.30246827	0.22536296	0.36712828
ENSG0000018	METTL7A	protein_codir	0.30233933	0.18224159	0.31447662
ENSG0000010	PRELID3B	protein_codir	0.30226151	0.03117657	0.08386218
ENSG0000028	CTD-2012J19.	lncRNA	0.30224073	0.86113895	0.91722872
ENSG0000028	CH507-154B1	lncRNA	0.30223283	0.66639211	0.77947529
ENSG0000013	INTS14	protein_codir	0.30210935	0.00067639	0.00418688
ENSG0000012	GLIPR2	protein_codir	0.30206671	0.05676668	0.13227839
ENSG0000002	GCLM	protein_codir	0.30173313	0.27293015	0.42082126
ENSG0000016	MFSD3	protein_codir	0.30171603	0.13911932	0.25773486

ENSG0000021	ZNF99	protein_codir	0.30168496	0.63003322	0.75083168
ENSG0000011	OPRM1	protein_codir	0.3015927	0.9159485	NA
ENSG0000026	CTB-60B18.1(	protein_codir	0.3015296	0.62494443	0.74719558
ENSG0000013	PUDP	protein_codir	0.30147229	0.07114237	0.15687491
ENSG0000027	INO80B-WBP:	protein_codir	0.30103827	0.52436221	0.66631346
ENSG0000017	KLHL15	protein_codir	0.30087983	0.35864275	0.51230024
ENSG0000013	TTLL4	protein_codir	0.30066364	0.12644902	0.2402692
ENSG0000027	RP11-376P6.3	lncRNA	0.30058372	0.7613311	0.84936436
ENSG0000018	PENK	protein_codir	0.30057211	0.6827502	0.79135179
ENSG0000010	MANBA	protein_codir	0.30048766	0.09549965	0.19482239
ENSG0000028	RP11-2P2.2	lncRNA	0.30044419	0.91436895	NA
ENSG0000011	COQ5	protein_codir	0.30034196	0.04615332	0.11322236
ENSG0000010	PTN	protein_codir	0.30026602	0.51046656	0.65440178
ENSG0000025	RP11-351M8.	lncRNA	0.2999848	0.64350913	0.76135027
ENSG0000017	POLH	protein_codir	0.29991672	0.11803903	0.22819612
ENSG0000025	RP11-134O21	lncRNA	0.2999042	0.68056766	0.79006269
ENSG0000011	FRMD4B	protein_codir	0.29988834	0.30586693	0.45744231
ENSG0000027	RP1-286D6.5	lncRNA	0.29977479	0.63188773	0.75237053
ENSG0000018	EHMT1	protein_codir	0.29974113	0.00532479	0.02130925
ENSG0000013	SPDYE1	protein_codir	0.29965068	0.77626463	0.85957699
ENSG0000013	ZNF227	protein_codir	0.29957429	0.03005772	0.08159958
ENSG0000025	LINC02622	lncRNA	0.29955104	0.89888725	NA
ENSG0000010	CKM	protein_codir	0.29953281	0.75238907	0.84235858
ENSG0000013	APH1B	protein_codir	0.29940566	0.05379603	0.12715843
ENSG0000009	TGM1	protein_codir	0.29932291	0.58964973	0.71952658
ENSG0000013	MARCHF9	protein_codir	0.29914311	0.19124411	0.32584374
ENSG0000013	Aug-04	protein_codir	0.29907454	0.04953986	0.11966861
ENSG0000026	RP11-989E6.1	lncRNA	0.29896895	0.82473757	0.89356743
ENSG0000021	ZNF611	protein_codir	0.29874389	0.05847081	0.13525343
ENSG0000016	ANKRD49	protein_codir	0.29873708	0.16197484	0.2882382
ENSG0000005	UNKL	protein_codir	0.29872527	0.08180485	0.17356353
ENSG0000017	GLMN	protein_codir	0.29862926	0.07247043	0.15902765
ENSG0000017	ACER2	protein_codir	0.29860302	0.27133877	0.41938315
ENSG0000017	CFAP53	protein_codir	0.29859175	0.49420417	0.6403903
ENSG0000017	PIDD1	protein_codir	0.29853071	0.38042272	0.53371644
ENSG0000013	AGAP3	protein_codir	0.2984608	0.06218554	0.14193575
ENSG0000019	OR52K1	protein_codir	0.29815507	0.89589359	NA
ENSG0000018	RSBN1L	protein_codir	0.29810685	0.06821659	0.15219243
ENSG0000011	OLFML3	protein_codir	0.29804806	0.43855659	0.58966669
ENSG0000007	PPP2R5C	protein_codir	0.29802539	0.00849393	0.03059885
ENSG0000013	RPL27	protein_codir	0.29798853	0.06865978	0.1528041
ENSG0000010	SEC14L2	protein_codir	0.29783154	0.34042208	0.49343783
ENSG0000014	RHOT2	protein_codir	0.29764366	0.21720625	0.35746874
ENSG0000026	RP11-283I3.6	lncRNA	0.29760962	0.12116738	0.23280994
ENSG0000018	NUTM2G	protein_codir	0.2975291	0.83869389	NA
ENSG0000016	DLC1	protein_codir	0.29747201	0.39803535	0.55103686
ENSG0000026	RP11-789C17.	lncRNA	0.29724933	0.54442477	0.68263175

ENSG0000023	AC127904.2	lncRNA	0.29720942	0.50988089	0.65404562
ENSG0000019	GTF2IRD2	protein_codir	0.29713003	0.22354449	0.36503274
ENSG0000021	DNASE1	protein_codir	0.29693192	0.43180163	0.58301494
ENSG0000028	RP4-681L3.3	lncRNA	0.29689302	0.7071515	0.8097647
ENSG0000020	TRPC5OS	protein_codir	0.29687043	0.87654279	NA
ENSG0000011	SELENOK	protein_codir	0.29685769	0.141478	0.26090822
ENSG0000018	FAM183A	protein_codir	0.29681746	0.87582045	NA
ENSG0000008	F11	protein_codir	0.29668123	0.87398558	NA
ENSG0000007	ANKRD13A	protein_codir	0.29654159	0.05707022	0.13277286
ENSG0000012	NUDCD1	protein_codir	0.29637618	0.03060783	0.08272182
ENSG0000027	SRD5A2	protein_codir	0.29627523	0.85726567	NA
ENSG0000016	PTX3	protein_codir	0.29615348	0.63488382	0.75476654
ENSG0000017	ZNF543	protein_codir	0.29609024	0.17187749	0.30128694
ENSG0000011	RPN2	protein_codir	0.2959898	0.10319698	0.20660341
ENSG0000026	CTD-2616J11.	protein_codir	0.29595056	0.75548278	0.84493698
ENSG0000026	RP5-1022P6.7	lncRNA	0.29569086	0.91749887	NA
ENSG0000019	RP1-253P7.4	lncRNA	0.29565269	0.8858178	0.93233319
ENSG0000018	RPL14	protein_codir	0.29559734	0.05867361	0.13558945
ENSG0000012	BAZ2B	protein_codir	0.29539896	0.00023012	0.00172948
ENSG0000022	LINC00466	lncRNA	0.29532053	0.85720933	NA
ENSG0000009	HNRNPC	protein_codir	0.29524098	0.01653674	0.05133694
ENSG0000026	RP11-715F3.2	lncRNA	0.29520091	0.6190381	0.74248012
ENSG0000001	TTC19	protein_codir	0.29514921	0.00391481	0.01671723
ENSG0000019	RPL10A	protein_codir	0.29514669	0.05918533	0.13652069
ENSG0000009	HDAC6	protein_codir	0.29509018	0.07573416	0.16406728
ENSG0000013	ABI1	protein_codir	0.29499885	0.01448002	0.04638041
ENSG0000017	RBM44	protein_codir	0.2948585	0.33508728	0.48795859
ENSG0000024	OR2A1-AS1	lncRNA	0.29474885	0.29336592	0.44350119
ENSG0000018	HACD4	protein_codir	0.29474727	0.25189334	0.39806007
ENSG0000024	ATP5MGL	protein_codir	0.29469538	0.70253391	0.80628402
ENSG0000026	RP11-597M12	lncRNA	0.29418678	0.87850819	NA
ENSG0000028	RP11-369L4.2	lncRNA	0.29415301	0.87781126	NA
ENSG0000012	IPPK	protein_codir	0.29396291	0.00492621	0.02002971
ENSG0000017	MUC13	protein_codir	0.29395379	0.70976738	0.81148359
ENSG0000027	RP11-44N12.5	lncRNA	0.2939176	0.62321801	0.74574829
ENSG0000014	DUOXA2	protein_codir	0.29385022	0.86952605	0.92263963
ENSG0000025	RP11-642D21	lncRNA	0.29381568	0.71857677	0.818268
ENSG0000013	SLTM	protein_codir	0.29376016	0.03595422	0.09340252
ENSG0000019	TLK1	protein_codir	0.29357269	0.04455205	0.11031175
ENSG0000012	WFDC3	protein_codir	0.29346527	0.60715748	0.73319734
ENSG0000018	CIB1	protein_codir	0.29342095	0.04644968	0.11380645
ENSG0000012	PDS5A	protein_codir	0.29336484	0.01067395	0.03666391
ENSG0000025	CTB-32H22.1	lncRNA	0.29330811	0.88057464	NA
ENSG0000010	SLC30A4	protein_codir	0.29321446	0.1113156	0.21861042
ENSG0000018	RPS23	protein_codir	0.29312377	0.09960304	0.20115793
ENSG0000016	ZNF75A	protein_codir	0.29300721	0.16534049	0.29275521
ENSG0000027	RP4-680D5.9	lncRNA	0.29294558	0.89913348	NA

ENSG0000026	RP11-416I2.1	lncRNA	0.29288209	0.89699918	NA
ENSG0000001	COX15	protein_codir	0.29286678	0.03746273	0.09641227
ENSG0000012	PDE11A	protein_codir	0.29279181	0.67637196	0.7868169
ENSG0000003	CLEC16A	protein_codir	0.2927376	0.03114378	0.0838148
ENSG0000012	FOXA1	protein_codir	0.29266877	0.71450903	0.81487899
ENSG0000017	DALRD3	protein_codir	0.2926427	0.0777167	0.16708454
ENSG0000023	PSMG3-AS1	lncRNA	0.29263637	0.19588233	0.33167423
ENSG0000016	MAT2A	protein_codir	0.29251579	0.38310774	0.53647514
ENSG0000020	SCGB2B2	protein_codir	0.29241491	0.30921981	0.46088453
ENSG0000026	RP11-214K3.2	lncRNA	0.29241055	0.55232259	0.68912453
ENSG0000025	LINC02206	lncRNA	0.2922874	0.87930026	NA
ENSG0000018	HHIPL1	protein_codir	0.29220263	0.42027552	0.57258471
ENSG0000013	NUMB	protein_codir	0.29219517	0.00273514	0.01254418
ENSG0000018	CFAP65	protein_codir	0.29216063	0.8566993	NA
ENSG0000025	RP11-371E8.4	protein_codir	0.2919193	0.91836347	NA
ENSG0000017	RNF34	protein_codir	0.29190422	0.02265912	0.06536922
ENSG0000028	RP11-103G8.5	lncRNA	0.29184947	0.76812376	0.85395178
ENSG0000025	RP11-612B6.6	lncRNA	0.29182157	0.74407998	0.83651454
ENSG0000019	RPL23A	protein_codir	0.29168893	0.06583314	0.14839693
ENSG0000001	CEP68	protein_codir	0.29157198	0.09427897	0.19310177
ENSG0000010	RASA4	protein_codir	0.29152793	0.15089253	0.27343207
ENSG0000025	RP11-841O20	lncRNA	0.29146616	0.8595897	NA
ENSG0000025	RP11-59N23.1	lncRNA	0.29142944	0.85880147	NA
ENSG0000014	FBN3	protein_codir	0.29136389	0.83178123	0.89863371
ENSG0000012	CCNT1	protein_codir	0.29131241	0.07933726	0.16963086
ENSG0000026	NBPF15	protein_codir	0.29124449	0.18984139	0.3242082
ENSG0000023	GRM7-AS1	lncRNA	0.29121228	0.83904758	0.90348905
ENSG0000007	SELENOO	protein_codir	0.29120521	0.14147643	0.26090822
ENSG0000013	NAAA	protein_codir	0.2910475	0.10805883	0.2138108
ENSG0000017	MZT2A	protein_codir	0.29094604	0.10015182	0.20197089
ENSG0000012	NCOA3	protein_codir	0.29070702	0.09355684	0.19195067
ENSG0000017	GLIS1	protein_codir	0.29069572	0.5297055	0.67104565
ENSG0000027	RP11-219B17.	lncRNA	0.29062555	0.79483045	0.87247504
ENSG0000014	PDSS1	protein_codir	0.29056654	0.22719996	0.3693981
ENSG0000011	SFPQ	protein_codir	0.29044108	0.05185421	0.12361586
ENSG0000027	RP11-474O21	lncRNA	0.29028525	0.65115538	0.76714348
ENSG0000006	ZBTB11	protein_codir	0.29006898	0.05505814	0.12931171
ENSG0000011	SMC4	protein_codir	0.29004868	0.11574764	0.22489974
ENSG0000016	KLF13	protein_codir	0.29002821	0.10862357	0.21468214
ENSG0000017	FAM210A	protein_codir	0.28998724	0.04976214	0.12009319
ENSG0000028	RP11-890B15.	lncRNA	0.28970196	0.90068798	NA
ENSG0000024	RP11-30K9.6	lncRNA	0.28963949	0.57560758	0.70813244
ENSG0000014	PML	protein_codir	0.28936079	0.02720226	0.07535871
ENSG0000014	SFXN5	protein_codir	0.28934693	0.15154635	0.27433446
ENSG0000017	DMXL1	protein_codir	0.28929755	0.13158982	0.24744763
ENSG0000010	ZMYND8	protein_codir	0.28925053	0.10159119	0.20417293
ENSG0000024	CASC9	lncRNA	0.28923666	0.868444	0.92191649

ENSG0000026	RP11-220I1.5	lncRNA	0.28912834	0.86766875	NA
ENSG0000010	NUFIP2	protein_codir	0.28909244	0.0933048	0.19151891
ENSG0000016	GMEB1	protein_codir	0.2889574	0.00793937	0.0290252
ENSG0000025	RP11-1069G1	lncRNA	0.28888941	0.78280862	0.86427811
ENSG0000026	UPK3BL1	protein_codir	0.28888283	0.57308487	0.70609823
ENSG0000014	UHRF2	protein_codir	0.28886383	0.1741467	0.30417394
ENSG0000019	DDX39B	protein_codir	0.28879235	0.21561794	0.35579922
ENSG0000010	NDFIP2	protein_codir	0.28876924	0.05590476	0.13077353
ENSG0000028	RP11-506N21	lncRNA	0.28875601	0.90076939	NA
ENSG0000027	CH17-262A2.1	lncRNA	0.28874887	0.73381938	0.82926604
ENSG0000027	RAB7B	protein_codir	0.28857792	0.26784108	0.41566077
ENSG0000024	LINC00886	lncRNA	0.2879793	0.44002593	0.5909857
ENSG0000013	PIK3C2B	protein_codir	0.28795952	0.23355154	0.37677085
ENSG0000028	CTD-2591A1.2	lncRNA	0.28782898	0.49419439	0.6403903
ENSG0000023	LINC01814	lncRNA	0.28773407	0.61619829	0.74004088
ENSG0000017	SAA1	protein_codir	0.28762571	0.5717763	0.70495765
ENSG0000019	MT-CO3	protein_codir	0.28762072	0.41474172	0.56788635
ENSG0000017	AGFG1	protein_codir	0.28758585	0.11429998	0.22276075
ENSG0000011	FOXRED1	protein_codir	0.28752987	0.19628724	0.33216284
ENSG0000011	SWT1	protein_codir	0.28745048	0.06010821	0.13817635
ENSG0000028	RP11-654K19.	protein_codir	0.28744187	0.82462619	0.89356743
ENSG0000010	NPRL3	protein_codir	0.28742222	0.03746535	0.09641227
ENSG0000026	LA16c-431H6.	protein_codir	0.28740881	0.30151369	0.45267207
ENSG0000013	MARCHF7	protein_codir	0.28736708	0.03115778	0.08383613
ENSG0000016	SLC25A6	protein_codir	0.28729573	0.06861199	0.15271829
ENSG0000005	SDCCAG8	protein_codir	0.28728957	0.00214731	0.01037592
ENSG0000028	RP1-29C18.11	lncRNA	0.28715997	0.74878248	0.83968218
ENSG0000015	OXA1L	protein_codir	0.28714305	0.00016421	0.0013227
ENSG0000010	GABPB1	protein_codir	0.28710914	0.16032588	0.28607797
ENSG0000018	NPM1	protein_codir	0.28704316	0.07545965	0.16368456
ENSG0000022	EDRF1-DT	lncRNA	0.28682172	0.51999604	0.66275918
ENSG0000008	PALB2	protein_codir	0.28664202	0.16869454	0.29709937
ENSG0000028	CTC-786C10.3	lncRNA	0.28656051	0.63162595	0.75218854
ENSG0000019	TRRAP	protein_codir	0.28649199	0.0629723	0.14338787
ENSG0000027	FAM193B-DT	lncRNA	0.28642994	0.67799642	0.78800965
ENSG0000026	AC005785.2	lncRNA	0.28636929	0.50798858	0.65240605
ENSG0000012	PFDN5	protein_codir	0.28629739	0.05853913	0.1353353
ENSG0000014	CELF1	protein_codir	0.28622281	0.01038107	0.03587204
ENSG0000026	CTD-2126E3.3	lncRNA	0.28607357	0.70488197	0.8079838
ENSG0000010	ABHD12	protein_codir	0.28602272	0.09426085	0.19307955
ENSG0000025	ZNF346-IT1	lncRNA	0.28574846	0.55144297	0.68836911
ENSG0000028	RP11-715F3.4	lncRNA	0.28572162	0.65625633	0.77160614
ENSG0000011	AKR1A1	protein_codir	0.28571412	0.10447329	0.20846355
ENSG0000026	RP11-863P13.	lncRNA	0.2856564	0.90499932	NA
ENSG0000016	ATRIP	protein_codir	0.28559768	0.08159715	0.17325595
ENSG0000025	AL161668.5	lncRNA	0.28534887	0.90347716	NA
ENSG0000013	DIAPH1	protein_codir	0.28530734	0.19206624	0.32681552

ENSG000001З	CHRND	protein_codir	0.2852901	0.92218644	NA
ENSG0000019	TOGARAM1	protein_codir	0.28525821	0.01824679	0.05525103
ENSG000002(	ZNF805	protein_codir	0.28516372	0.05319569	0.12606305
ENSG0000019	SPTSSB	protein_codir	0.28507162	0.66488628	0.7783732
ENSG000002З	ECI2-DT	lncRNA	0.28499103	0.63345734	0.75368708
ENSG000001(	TMC5	protein_codir	0.28457497	0.78831347	0.8678738
ENSG000002(	ZNF468	protein_codir	0.28456243	0.14548139	0.26645863
ENSG0000019	CNGA1	protein_codir	0.28446827	0.57687129	0.70921325
ENSG0000027	RP11-162A12	lncRNA	0.28441944	0.50955947	0.65375476
ENSG0000027	RP11-385F5.5	lncRNA	0.28441748	0.36554291	0.51935675
ENSG000001	DDX55	protein_codir	0.28439048	0.17296848	0.30267293
ENSG000001(	PPM1M	protein_codir	0.2842469	0.03879192	0.09909664
ENSG0000027	PKD1L3	protein_codir	0.28421727	0.60711463	0.73317765
ENSG000001{	GPATCH8	protein_codir	0.28418796	0.00882009	0.03152313
ENSG000002(	AC010524.2	lncRNA	0.28404306	0.76552627	0.85229908
ENSG000002(	CTD-2319I12..	lncRNA	0.28403049	0.40441344	0.55738108
ENSG000001(	ABHD17B	protein_codir	0.28389063	0.24576995	0.39100087
ENSG0000015	TYSND1	protein_codir	0.28387045	0.13460704	0.25159684
ENSG000002{	RP11-434N15	protein_codir	0.28381018	0.84515427	0.9076232
ENSG000001(	RP9	protein_codir	0.28359402	0.27612619	0.42428959
ENSG0000014	TMEM209	protein_codir	0.2835373	0.17698333	0.30743896
ENSG0000017	R3HDM2	protein_codir	0.28350731	0.00028106	0.00204397
ENSG000001(	FBL	protein_codir	0.28343364	0.0737046	0.16097746
ENSG000002{	RP11-566D24	protein_codir	0.28319253	0.58187386	0.7131731
ENSG0000012	MOB3B	protein_codir	0.28309975	0.28502829	0.43463155
ENSG0000025	RP11-499F3.2	lncRNA	0.28302161	0.72149726	0.8202155
ENSG0000012	SRSF6	protein_codir	0.2829071	0.00803807	0.02934332
ENSG000001{	SLC25A10	protein_codir	0.28290008	0.24997383	0.39588745
ENSG000001(	NOLC1	protein_codir	0.2828868	0.24676099	0.39199171
ENSG0000017	MTBP	protein_codir	0.28276354	0.14174769	0.26128345
ENSG000000(	CELSR3	protein_codir	0.2826699	0.39007423	0.54311892
ENSG0000027	RP1-197B17.5	lncRNA	0.28261895	0.65952621	0.77390237
ENSG0000019	PPTC7	protein_codir	0.28247457	0.09811279	0.19896293
ENSG0000027	XKR5	protein_codir	0.28239652	0.69061878	0.79699536
ENSG000001(	HBP1	protein_codir	0.28233171	0.08826488	0.18361648
ENSG000001(	INTS6	protein_codir	0.28220681	0.08723579	0.18203964
ENSG000001{	TMEM17	protein_codir	0.28209337	0.32846518	0.48077416
ENSG000000{	SESN1	protein_codir	0.28200038	0.20763454	0.34615614
ENSG000001З	VAMP1	protein_codir	0.28185148	0.4411284	0.592087
ENSG000001{	SLIT3	protein_codir	0.28169447	0.3765386	0.53012519
ENSG000001	ASB3	protein_codir	0.28150602	0.09106182	0.18783626
ENSG0000019	SLC35F1	protein_codir	0.28129398	0.59824786	0.72635242
ENSG000001(	RPGRIP1L	protein_codir	0.28128112	0.21168529	0.35120142
ENSG0000014	RPS27A	protein_codir	0.28103543	0.08443638	0.17760511
ENSG000000З	MFAP3	protein_codir	0.28100933	0.119428	0.23010874
ENSG0000015	SMG1	protein_codir	0.28099191	0.07362159	0.16083433
ENSG000001(	RYBP	protein_codir	0.28083088	0.23981487	0.38427143

ENSG0000020	MT1A	protein_codir	0.28075065	0.75795055	0.84673049
ENSG0000017	SLC35E3	protein_codir	0.28049432	0.14669371	0.26816336
ENSG0000010	KLHL5	protein_codir	0.28011642	0.00895164	0.03192248
ENSG0000021	AC091801.1	lncRNA	0.28007919	0.86518263	NA
ENSG0000024	SNHG6	lncRNA	0.2800529	0.12725195	0.24132785
ENSG0000011	PPARD	protein_codir	0.2799328	0.11039085	0.21725776
ENSG0000011	SLC2A1	protein_codir	0.27980946	0.30340548	0.45472133
ENSG0000011	DCPS	protein_codir	0.27979159	0.07616199	0.16475988
ENSG0000025	MAPK6-DT	lncRNA	0.27965523	0.85731715	0.91475872
ENSG0000016	IL20	protein_codir	0.2793511	0.88349601	NA
ENSG0000016	CSTB	protein_codir	0.27933295	0.28600499	0.43554283
ENSG0000025	RP11-855O10	lncRNA	0.27931948	0.88435864	NA
ENSG0000022	INE1	lncRNA	0.27917326	0.56447834	0.69876711
ENSG0000024	AC006160.5	lncRNA	0.27867515	0.82727008	0.8953799
ENSG0000023	RP5-1050D4.2	lncRNA	0.27864963	0.90448107	NA
ENSG0000025	RP11-977B10.	lncRNA	0.27854149	0.61151654	0.73658271
ENSG0000015	MGAT5	protein_codir	0.27841695	0.12723786	0.24131771
ENSG0000007	SLC44A1	protein_codir	0.27833785	0.22227454	0.36343056
ENSG0000010	IMPDH1	protein_codir	0.27823573	0.13266756	0.24903343
ENSG0000023	PPP1R3E	protein_codir	0.27818008	0.28277235	0.43224153
ENSG0000022	RP11-456H18	lncRNA	0.27806471	0.87396962	0.92523044
ENSG0000027	RP11-214N9.1	lncRNA	0.27783256	0.63883991	0.75792598
ENSG0000026	ZNF566-AS1	lncRNA	0.27773639	0.40108029	0.55414125
ENSG0000016	FAM227B	protein_codir	0.2776698	0.19468585	0.33017458
ENSG0000007	EPB41L2	protein_codir	0.27762681	0.14331064	0.26337347
ENSG0000028	RP11-701P16.	lncRNA	0.27758242	0.86623	NA
ENSG0000001	GLT8D1	protein_codir	0.27750007	0.04415046	0.10952833
ENSG0000016	STK17A	protein_codir	0.27732762	0.24339944	0.38848267
ENSG0000012	DDX31	protein_codir	0.27714477	0.07913851	0.16933728
ENSG0000021	SELENOH	protein_codir	0.27707897	0.03192043	0.08522405
ENSG0000026	CTD-2571L23.	lncRNA	0.27706606	0.87283865	NA
ENSG0000013	NEDD1	protein_codir	0.27696397	0.02274807	0.06553685
ENSG0000012	BEST3	protein_codir	0.27692889	0.86815818	NA
ENSG0000023	RP11-220I1.2	lncRNA	0.27685027	0.83471366	0.90045724
ENSG0000014	COMMD3	protein_codir	0.27664821	0.09115379	0.18800222
ENSG0000013	KRAS	protein_codir	0.27637983	0.08042897	0.17136838
ENSG0000017	FAM241A	protein_codir	0.27625569	0.18904463	0.32310722
ENSG0000014	KLHL3	protein_codir	0.27605864	0.21823307	0.35865746
ENSG0000025	RP11-701B16.	lncRNA	0.27598025	0.88592792	NA
ENSG0000021	PAPOLB	protein_codir	0.27571472	0.79713661	0.8738951
ENSG0000013	FASTKD1	protein_codir	0.27569896	0.30262188	0.45401502
ENSG0000016	C2CD3	protein_codir	0.27554407	0.0070546	0.02646673
ENSG0000018	MAFF	protein_codir	0.27531924	0.62746831	0.7487362
ENSG0000018	ATP6V0C	protein_codir	0.27530842	0.03133906	0.0841272
ENSG0000017	MANEA	protein_codir	0.27528803	0.18433144	0.3172504
ENSG0000013	SENP7	protein_codir	0.27512982	0.02171749	0.06316368
ENSG0000016	RBPJ	protein_codir	0.27488786	0.01183365	0.03968247

ENSG0000007	NCK2	protein_codir	0.27479874	0.07850163	0.16830586
ENSG0000019	LDB1	protein_codir	0.27462896	0.06607523	0.14880935
ENSG0000016	PIP4P1	protein_codir	0.27460016	0.04309729	0.10751967
ENSG0000028	RP11-856M9.	lncRNA	0.27457434	0.59577098	0.72413284
ENSG0000013	ERMARD	protein_codir	0.27455466	0.17322525	0.30292909
ENSG0000016	TP53I13	protein_codir	0.27451402	0.27396486	0.42201983
ENSG0000018	COL25A1	protein_codir	0.27435921	0.54506539	0.6830872
ENSG0000028	RP11-112E16.	lncRNA	0.27421479	0.80332339	0.87813322
ENSG0000017	COX5A	protein_codir	0.27403794	0.03518156	0.09189417
ENSG0000026	RP11-388M20	lncRNA	0.27401136	0.62154169	0.74451303
ENSG0000003	ALG1	protein_codir	0.2739982	0.08328133	0.1756974
ENSG0000019	ZNF628	protein_codir	0.27380302	0.09079854	0.18744716
ENSG0000014	GIGYF1	protein_codir	0.27379181	0.12015618	0.23122091
ENSG0000012	LSM8	protein_codir	0.27371043	0.02333707	0.0668225
ENSG0000022	RP11-20J15.3	lncRNA	0.27369276	0.43115595	0.58249634
ENSG0000010	RBBP8	protein_codir	0.2736484	0.15913596	0.28452454
ENSG0000013	ACTR2	protein_codir	0.27359372	0.14921234	0.27140236
ENSG0000019	SZT2	protein_codir	0.27358218	0.08175067	0.17350187
ENSG0000015	WIPI2	protein_codir	0.27350102	0.00403161	0.0171364
ENSG0000023	LINC01409	lncRNA	0.27347176	0.61691625	0.74070713
ENSG0000017	TMEM39A	protein_codir	0.27330653	0.01816513	0.05504344
ENSG0000016	LENG8	protein_codir	0.27327818	0.39224774	0.54522967
ENSG0000027	RP13-1039J1.4	lncRNA	0.27316482	0.54685551	0.68459181
ENSG0000003	RNF19A	protein_codir	0.27309435	0.07408421	0.16152556
ENSG0000028	CTD-2308N23	lncRNA	0.27303415	0.70459811	0.80774498
ENSG0000025	SAA2-SAA4	protein_codir	0.27285149	0.77184229	0.85643115
ENSG0000025	SLC25A30-AS1	lncRNA	0.27267038	0.83971536	0.90406714
ENSG0000011	CPEB4	protein_codir	0.27253801	0.18662217	0.32006474
ENSG0000015	PDIA4	protein_codir	0.27251128	0.21170548	0.35121061
ENSG0000002	VRK2	protein_codir	0.27243709	0.04298054	0.10732506
ENSG0000011	KLF7	protein_codir	0.27236442	0.0661814	0.14899987
ENSG0000028	RP4-798C17.7	lncRNA	0.27213729	0.52234544	0.66481346
ENSG0000010	EFTUD2	protein_codir	0.27207575	0.06539534	0.14771174
ENSG0000002	ARNTL2	protein_codir	0.2719554	0.33693359	0.48992921
ENSG0000028	RP11-486D20	lncRNA	0.27192805	0.85816421	NA
ENSG0000013	WDFY2	protein_codir	0.27179289	0.17107162	0.30019047
ENSG0000017	FOS	protein_codir	0.27176546	0.61067192	0.73596119
ENSG0000023	AC005532.5	lncRNA	0.27155227	0.63234419	0.75278421
ENSG0000013	PRMT7	protein_codir	0.27154799	0.21648722	0.35680731
ENSG0000013	EIF2S3	protein_codir	0.27142627	0.00748677	0.02774886
ENSG0000016	TASOR	protein_codir	0.27063416	0.06867931	0.15281741
ENSG0000025	LINC02747	lncRNA	0.27036414	0.87367692	NA
ENSG0000011	MAD2L2	protein_codir	0.27027014	0.07434273	0.16191027
ENSG0000000	SLC25A5	protein_codir	0.27014069	0.10717347	0.21244078
ENSG0000006	BICRA	protein_codir	0.2701339	0.0422003	0.10584565
ENSG0000014	DGKQ	protein_codir	0.2700344	0.21300694	0.35269178
ENSG0000011	TPI1	protein_codir	0.26999775	0.05494419	0.12910997

ENSG0000017	C3orf38	protein_codir	0.26993795	0.15328019	0.27672389
ENSG0000014	CYP4B1	protein_codir	0.26988734	0.68367126	0.79218924
ENSG0000013	PCNA	protein_codir	0.26979328	0.21689998	0.35716792
ENSG0000013	SRRM1	protein_codir	0.26966055	0.0159815	0.05008046
ENSG0000020	STARD7-AS1	lncRNA	0.26942822	0.04888867	0.11841616
ENSG0000016	STARD4	protein_codir	0.26930156	0.05948561	0.13704166
ENSG0000018	ENO4	protein_codir	0.26928467	0.55489797	0.69099558
ENSG0000025	CTA-392E5.1	lncRNA	0.26922229	0.90736174	NA
ENSG0000016	TAF1D	protein_codir	0.26916649	0.28199256	0.43140769
ENSG0000017	NRTN	protein_codir	0.26892873	0.62498144	0.74720749
ENSG0000018	ZACN	protein_codir	0.26878809	0.46622564	0.61586647
ENSG0000027	RP11-38M8.1	lncRNA	0.26878033	0.57141238	0.70474433
ENSG0000025	RP11-809O17	lncRNA	0.26868023	0.34743508	0.50064388
ENSG0000019	PIWIL2	protein_codir	0.2686284	0.53245893	0.67344823
ENSG0000011	MORN1	protein_codir	0.26844425	0.24965332	0.39551592
ENSG0000006	ERLEC1	protein_codir	0.26841051	0.17790564	0.30867226
ENSG0000016	ZNF22	protein_codir	0.26814425	0.00705556	0.02646673
ENSG0000011	EHBP1	protein_codir	0.26806965	0.32038867	0.47244067
ENSG0000027	RP11-797D24	lncRNA	0.26791109	0.78469645	0.86530766
ENSG0000021	ZNF433-AS1	lncRNA	0.26788434	0.25936922	0.40645012
ENSG0000019	SIAH1	protein_codir	0.26767265	0.04674311	0.11433448
ENSG0000027	RP11-750H9.7	lncRNA	0.26759766	0.79882416	0.87494639
ENSG0000017	ANGEL2	protein_codir	0.26757333	0.04598261	0.11294262
ENSG0000013	TMEM258	protein_codir	0.26741924	0.06502916	0.14706504
ENSG0000016	KMT2D	protein_codir	0.2673865	0.04273198	0.10687832
ENSG0000010	HPS4	protein_codir	0.26721175	0.07092949	0.15656297
ENSG0000025	RP11-109N23	lncRNA	0.26712885	0.56527814	0.69956906
ENSG0000011	VAX2	protein_codir	0.26706674	0.6134757	0.7379434
ENSG0000019	GLMP	protein_codir	0.26706412	0.10999491	0.2167256
ENSG0000016	SH3TC2	protein_codir	0.26669195	0.51916205	0.66201549
ENSG0000018	CDK10	protein_codir	0.26667138	0.3513971	0.50494708
ENSG0000016	BRWD3	protein_codir	0.26663517	0.05458376	0.12851448
ENSG0000014	IFNAR1	protein_codir	0.26659768	0.06403555	0.14531772
ENSG0000013	UNC79	protein_codir	0.26648267	0.48031114	0.6280747
ENSG0000028	RP4-570D2.2	lncRNA	0.26632621	0.71917864	0.81863864
ENSG0000012	NUP153	protein_codir	0.26630355	0.19713065	0.33319555
ENSG0000027	RP11-326N17	lncRNA	0.26601927	0.84472949	NA
ENSG0000012	HERPUD2	protein_codir	0.2659874	0.01613694	0.05047098
ENSG0000014	MEGF10	protein_codir	0.26598484	0.68563541	0.79358212
ENSG0000015	CHAF1B	protein_codir	0.26591404	0.16092844	0.28683813
ENSG0000028	RP11-1145F2	lncRNA	0.26561669	0.77755981	0.86071402
ENSG0000015	CARNMT1	protein_codir	0.26559403	0.21635879	0.35669812
ENSG0000008	SLC35C2	protein_codir	0.26551253	0.01907551	0.05719341
ENSG0000014	NUP210L	protein_codir	0.26536983	0.75094184	0.8412498
ENSG0000017	IL17RA	protein_codir	0.26523557	0.14910618	0.2712986
ENSG0000024	RP11-285F7.2	lncRNA	0.26510546	0.49665728	0.64251401
ENSG0000001	CHDH	protein_codir	0.26509805	0.34769779	0.50090825

ENSG0000014	IMPA2	protein_codir	0.26507464	0.37035848	0.52380056
ENSG0000008	ZNF264	protein_codir	0.26506919	0.05115142	0.12249021
ENSG0000023	ZRANB2-AS1	lncRNA	0.26481274	0.89276032	NA
ENSG0000024	HS3ST5	protein_codir	0.26475205	0.8005302	0.876244
ENSG0000001	AKAP8L	protein_codir	0.26465063	0.28903407	0.43858483
ENSG0000011	RARRES1	protein_codir	0.26457358	0.54312548	0.68165247
ENSG0000025	CENPS-CORT	protein_codir	0.2644918	0.69980612	0.80411553
ENSG0000016	NSA2	protein_codir	0.26436962	0.0196341	0.05844872
ENSG0000011	PDE4D	protein_codir	0.26424589	0.48988551	0.63666014
ENSG0000026	CTD-3247F14.	lncRNA	0.26417048	0.54152639	0.68032563
ENSG0000018	BOLA2	protein_codir	0.26404966	0.32110706	0.47323926
ENSG0000028	RP11-168F24.	lncRNA	0.26396235	0.86153947	NA
ENSG0000016	ZEB2	protein_codir	0.26394211	0.13113535	0.24681148
ENSG0000016	RPL8	protein_codir	0.26391906	0.12969827	0.24469032
ENSG0000013	PCBD2	protein_codir	0.26382937	0.04456481	0.11032869
ENSG0000012	MCF2L	protein_codir	0.26382705	0.42537843	0.57728736
ENSG0000004	TNC	protein_codir	0.26381653	0.59221468	0.72117496
ENSG0000027	AC139100.4	lncRNA	0.26371207	0.69274725	0.79836869
ENSG0000017	HSD17B13	protein_codir	0.26364171	0.54025192	0.67931158
ENSG0000019	CEP290	protein_codir	0.26362845	0.00313132	0.01398107
ENSG0000014	UBXN4	protein_codir	0.26361219	4.28E-06	6.21E-05
ENSG0000027	RP11-424N24	lncRNA	0.26356246	0.83060084	0.89799593
ENSG0000012	RPL5	protein_codir	0.26341599	0.09068099	0.18723249
ENSG0000023	PITPNA-AS1	lncRNA	0.26323294	0.17680041	0.30721783
ENSG0000015	SUPV3L1	protein_codir	0.26315788	0.09767348	0.19824673
ENSG0000018	KDM4D	protein_codir	0.26300432	0.41411184	0.56718085
ENSG0000028	RP11-934B9.9	lncRNA	0.26299695	0.71405236	0.81463073
ENSG0000025	RP11-727F15.	lncRNA	0.26296282	0.64035467	0.75885311
ENSG0000019	POM121	protein_codir	0.26292481	0.02917284	0.0797934
ENSG0000011	TTC31	protein_codir	0.26291932	0.14689411	0.2683521
ENSG0000024	RP11-247I13.:	lncRNA	0.262875	0.55414713	0.69037184
ENSG0000024	RP11-552M11	lncRNA	0.26269662	0.75701542	0.84616616
ENSG0000014	LTO1	protein_codir	0.26267217	0.13551885	0.25261731
ENSG0000027	RP4-545L17.1	lncRNA	0.26266502	0.87372431	NA
ENSG0000019	ZNF28	protein_codir	0.26265385	0.22733211	0.36949997
ENSG0000015	DIPK1A	protein_codir	0.26256073	0.08093678	0.17218469
ENSG0000012	EIF2AK4	protein_codir	0.26252566	0.11686059	0.22648862
ENSG0000020	SYS1	protein_codir	0.26251312	0.02978426	0.08103242
ENSG0000028	RP11-65B7.2	protein_codir	0.26250007	0.87474671	NA
ENSG0000019	CCDC167	protein_codir	0.26248118	0.3247562	0.47716598
ENSG0000009	SF3A1	protein_codir	0.26235971	0.0155307	0.04895662
ENSG0000021	TAS2R14	protein_codir	0.26198174	0.50397208	0.64871017
ENSG0000018	TBK1	protein_codir	0.26163519	0.00293207	0.01324347
ENSG0000010	CST3	protein_codir	0.26160263	0.30784279	0.45957612
ENSG0000013	EXOC6	protein_codir	0.26156144	0.01413156	0.04553355
ENSG0000022	RP11-108M9.	lncRNA	0.26155489	0.80996058	0.88266369
ENSG0000011	GON4L	protein_codir	0.26136505	0.01198952	0.04005208

ENSG0000014	ZNF697	protein_codir	0.26124777	0.25951522	0.406592
ENSG0000018	HSPA14	protein_codir	0.26117589	0.02705968	0.07506161
ENSG0000025	AP003419.11	protein_codir	0.26110361	0.33416012	0.4870964
ENSG0000018	FAM167B	protein_codir	0.26085686	0.3819052	0.53538836
ENSG0000010	CUBN	protein_codir	0.2608129	0.2296686	0.37211514
ENSG0000024	UBAP1L	protein_codir	0.26077982	0.51593285	0.65917593
ENSG0000018	TMEM179B	protein_codir	0.26076664	0.00251558	0.01173853
ENSG0000011	ITFG2	protein_codir	0.26062068	0.1100472	0.21677011
ENSG0000025	RP11-982M15	lncRNA	0.260547	0.89307747	NA
ENSG0000018	LINC00482	lncRNA	0.2605335	0.6082267	0.73410337
ENSG0000023	RP11-285G1.2	lncRNA	0.26003931	0.8979804	0.93937123
ENSG0000023	STPG4	protein_codir	0.25979325	0.77481362	0.85869321
ENSG0000014	RPL7	protein_codir	0.25978965	0.09722093	0.19754595
ENSG0000010	AKAP8	protein_codir	0.25969595	0.01323135	0.04321781
ENSG0000016	MTMR10	protein_codir	0.25954684	0.0242995	0.06901309
ENSG0000014	FURIN	protein_codir	0.25950575	0.23991013	0.38433489
ENSG0000027	RP5-1116H23	lncRNA	0.25936869	0.87932568	NA
ENSG0000014	SOX13	protein_codir	0.25935027	0.39729265	0.55017413
ENSG0000013	TTC9	protein_codir	0.2592761	0.4815705	0.62915554
ENSG0000012	PLGLB2	protein_codir	0.25924695	0.51141014	0.65512491
ENSG0000016	TLR3	protein_codir	0.25919567	0.40251119	0.55550688
ENSG0000016	ZMYND19	protein_codir	0.25902468	0.16197425	0.2882382
ENSG0000021	B3GALT9	protein_codir	0.25901809	0.38713461	0.54019692
ENSG0000003	NUP160	protein_codir	0.25891476	0.02798182	0.07715463
ENSG0000018	CCDC66	protein_codir	0.25887719	0.19415607	0.32962017
ENSG0000010	UNC119	protein_codir	0.25882418	0.20261437	0.33972008
ENSG0000014	HAUS6	protein_codir	0.25856139	0.01350715	0.04393682
ENSG0000028	JAKMIP2-AS1	lncRNA	0.25854925	0.90883452	NA
ENSG0000016	CERCAM	protein_codir	0.25844889	0.30601898	0.45757061
ENSG0000016	ALB	protein_codir	0.25831664	0.72030615	0.81935899
ENSG0000018	PDZD7	protein_codir	0.2583066	0.44676803	0.59773091
ENSG0000015	NDUFAF6	protein_codir	0.25819832	0.09042107	0.18687744
ENSG0000026	LINC01140	lncRNA	0.25799963	0.51236351	0.65598109
ENSG0000001	PTBP1	protein_codir	0.25799555	0.03669746	0.09493152
ENSG0000000	SYN1	protein_codir	0.25796604	0.53022853	0.67150773
ENSG0000013	RBM26	protein_codir	0.25792966	0.13731306	0.25508384
ENSG0000017	RAB4B-EGLN2	protein_codir	0.25788002	0.56760564	0.70134961
ENSG0000020	EEF1AKMT2	protein_codir	0.25751355	0.03130068	0.08406502
ENSG0000023	RP11-71N10.1	lncRNA	0.25743386	0.81278873	0.88487287
ENSG0000015	MORC3	protein_codir	0.25740797	0.02708546	0.07511803
ENSG0000016	CSGALNACT2	protein_codir	0.25737462	0.16158633	0.28776932
ENSG0000023	LINC01816	lncRNA	0.25733908	0.58668307	0.71705034
ENSG0000023	TMEM114	protein_codir	0.25733719	0.87455421	0.92555894
ENSG0000007	CFAP20	protein_codir	0.25714323	0.02039767	0.06017769
ENSG0000013	ITM2B	protein_codir	0.25709396	0.10640313	0.21138275
ENSG0000023	AC024560.2	lncRNA	0.25701459	0.76585258	0.85242185
ENSG0000001	CLCN6	protein_codir	0.25670446	0.07847167	0.16826237

ENSG0000008	HSPB11	protein_codir	0.25651663	0.06824641	0.15221724
ENSG0000018	C11orf71	protein_codir	0.25650252	0.20921108	0.34818088
ENSG0000016	RILP	protein_codir	0.25648683	0.12818173	0.24258087
ENSG0000019	TCF4	protein_codir	0.25638141	0.03566803	0.09285992
ENSG0000010	TBC1D12	protein_codir	0.25633994	0.11800667	0.22814954
ENSG0000025	LINC02145	lncRNA	0.25618893	0.66805903	0.7807197
ENSG0000014	RBMX	protein_codir	0.25616942	0.04569233	0.11242959
ENSG0000004	R3HDM1	protein_codir	0.25616879	0.0376808	0.09681338
ENSG0000017	RNF139	protein_codir	0.25612786	0.15370772	0.27733259
ENSG0000007	ALPK1	protein_codir	0.25594616	0.19211208	0.32687339
ENSG0000017	ZNF169	protein_codir	0.25590372	0.47214408	0.62083491
ENSG0000013	CARS2	protein_codir	0.25586483	0.10739915	0.21271886
ENSG0000011	DNAH7	protein_codir	0.25567936	0.27387161	0.42195202
ENSG0000025	CTD-2647L4.4	lncRNA	0.25561282	0.244607	0.38962597
ENSG0000012	TEP1	protein_codir	0.25557684	0.18855527	0.3224504
ENSG0000026	RP11-3P17.5	lncRNA	0.25541399	0.93078297	NA
ENSG0000014	FAM171B	protein_codir	0.25540635	0.38236654	0.53581755
ENSG0000003	ZCCHC8	protein_codir	0.25539966	0.08546724	0.17939093
ENSG0000012	LIAS	protein_codir	0.25525232	0.27058853	0.41848117
ENSG0000013	RALGPS1	protein_codir	0.25518505	0.26668103	0.41427053
ENSG0000020	CTC-425O23.2	lncRNA	0.25503001	0.59598001	0.72435502
ENSG0000016	MYSM1	protein_codir	0.25499915	0.04266591	0.10674776
ENSG0000017	TSHZ1	protein_codir	0.25488861	0.11956587	0.23032566
ENSG0000018	POLR1D	protein_codir	0.25482595	0.04363277	0.10854157
ENSG0000010	FERMT1	protein_codir	0.25481703	0.65881754	0.773288
ENSG0000011	PRKD3	protein_codir	0.2547506	0.10304096	0.20636583
ENSG0000020	C2orf91	lncRNA	0.25464083	0.7376894	0.83207339
ENSG0000015	ALG8	protein_codir	0.25453581	0.10854489	0.21458806
ENSG0000015	QTRT2	protein_codir	0.25452611	0.04484399	0.11088549
ENSG0000027	H2AC19	protein_codir	0.25446125	0.53136741	0.67251835
ENSG0000018	DDX28	protein_codir	0.25443346	0.21405523	0.35390394
ENSG0000013	UBAP2	protein_codir	0.25431434	0.00112521	0.00627783
ENSG0000008	DNMT3B	protein_codir	0.25425806	0.39625324	0.54912019
ENSG0000017	USF3	protein_codir	0.25423125	0.18318003	0.31576062
ENSG0000013	PAPSS1	protein_codir	0.25413983	0.10815885	0.21397805
ENSG0000010	DDX5	protein_codir	0.25412582	0.02610499	0.07302947
ENSG0000011	APBB3	protein_codir	0.25408575	0.36781006	0.5213596
ENSG0000012	SLC35B3	protein_codir	0.25400782	0.01401427	0.04520839
ENSG0000027	RP1-151F17.2	lncRNA	0.25397738	0.43313802	0.58445806
ENSG0000027	RP11-139H15	lncRNA	0.25381775	0.78253352	0.86402462
ENSG0000011	GLI1	protein_codir	0.25380865	0.4875223	0.63464985
ENSG0000021	VPS33B-DT	lncRNA	0.25378794	0.72554841	0.82315248
ENSG0000016	CPSF4	protein_codir	0.25374566	0.03593103	0.09336861
ENSG0000028	RP11-464C19.	lncRNA	0.25373222	0.86217517	0.91783689
ENSG0000027	LINC02809	lncRNA	0.25360813	0.6962055	0.80104961
ENSG0000019	DIO3	protein_codir	0.25353634	0.64431199	0.76203902
ENSG0000000	REX1BD	protein_codir	0.25344824	0.19203691	0.32678575

ENSG0000027	RP11-225H22	lncRNA	0.25342536	0.73905746	0.83324012
ENSG0000015	PRXL2C	protein_codir	0.25338358	0.28134917	0.4307813
ENSG0000015	ADIPOR1	protein_codir	0.25322936	0.04858978	0.11785807
ENSG0000018	H2BC13	protein_codir	0.25305568	0.78075366	0.86289827
ENSG0000027	RP11-173P15.	lncRNA	0.25287967	0.74250899	0.83556464
ENSG0000028	RP11-192G13	lncRNA	0.25276557	0.78888521	0.86822639
ENSG0000012	TMTC4	protein_codir	0.2526862	0.2083731	0.34709999
ENSG0000019	PIGN	protein_codir	0.2526013	0.14291889	0.26281533
ENSG0000026	CEBPA-DT	lncRNA	0.25248178	0.51446948	0.65767134
ENSG0000016	CCDC74A	protein_codir	0.25240719	0.42092259	0.5729858
ENSG0000024	CTAGE8	protein_codir	0.25239214	0.5003376	0.64564215
ENSG0000006	KDM4A	protein_codir	0.25238216	0.06097209	0.13965081
ENSG0000010	CSF3	protein_codir	0.25236083	0.79568396	0.87311344
ENSG0000010	USP42	protein_codir	0.25234956	0.0427644	0.10693036
ENSG0000013	HRK	protein_codir	0.25227464	0.65140917	0.767377
ENSG0000025	RP11-72M17.	lncRNA	0.25221285	0.55917552	0.6946311
ENSG0000023	ARNILA	lncRNA	0.25212654	0.50046414	0.64574216
ENSG0000019	PRMT6	protein_codir	0.25210926	0.2385118	0.38276078
ENSG0000014	PAOX	protein_codir	0.25173842	0.30288412	0.45420374
ENSG0000027	XXbac-BPGBP	lncRNA	0.25167985	0.73476925	0.82979624
ENSG0000022	PLEKHM1	protein_codir	0.2516397	0.02991439	0.08127438
ENSG0000018	LRRIQ4	protein_codir	0.2516109	0.79227685	0.87091683
ENSG0000020	CNEP1R1	protein_codir	0.25156774	0.14486846	0.26561783
ENSG0000011	TBCCD1	protein_codir	0.25140187	0.02314927	0.06643628
ENSG0000027	RP11-141B14.	lncRNA	0.2513197	0.64429044	0.76203902
ENSG0000013	FDX1	protein_codir	0.25108794	0.0720313	0.15833702
ENSG0000024	RP11-620J15.	lncRNA	0.25099505	0.72270927	0.82105954
ENSG0000011	TCERG1	protein_codir	0.25091281	0.05468432	0.12867447
ENSG0000026	CTD-3214H19	protein_codir	0.250895	0.89885764	0.93983381
ENSG0000013	SCAF11	protein_codir	0.25086604	0.0707035	0.15621395
ENSG0000006	VMP1	protein_codir	0.25086345	0.34771927	0.50091307
ENSG0000020	FANK1	protein_codir	0.2508602	0.44540935	0.59651435
ENSG0000017	ZNF552	protein_codir	0.25077009	0.39072185	0.54369174
ENSG0000013	MPHOSPH6	protein_codir	0.25069326	0.09940193	0.20086927
ENSG0000011	DNAJC16	protein_codir	0.25047487	0.07036299	0.15562347
ENSG0000008	USP40	protein_codir	0.25045051	0.06778113	0.1515771
ENSG0000016	ABCE1	protein_codir	0.25041267	0.09516449	0.1943396
ENSG0000027	FAM106A	lncRNA	0.25031687	0.45891203	0.60932364
ENSG0000017	LACC1	protein_codir	0.25024453	0.16919943	0.29773845
ENSG0000008	PPEF1	protein_codir	0.25007171	0.64900592	0.76510067
ENSG0000017	DPAGT1	protein_codir	0.24971623	0.14923277	0.27140378
ENSG0000028	RP11-527B17.	lncRNA	0.24955649	0.84347891	0.90639867
ENSG0000018	CLN3	protein_codir	0.24949125	0.04179353	0.1050735
ENSG0000011	PRRC2C	protein_codir	0.24949086	0.01694461	0.05228578
ENSG0000007	REXO1	protein_codir	0.24948429	0.092509	0.19025305
ENSG0000012	RPS4Y1	protein_codir	0.24924063	0.62560374	0.74756951
ENSG0000017	CCDC106	protein_codir	0.24912051	0.17868911	0.30970301

ENSG0000025	RP11-770E5.1	lncRNA	0.24900048	0.84848014	0.90974462
ENSG0000022	RP11-481G8.2	lncRNA	0.2489637	0.87132074	NA
ENSG0000018	SATB1	protein_codir	0.24874229	0.31550874	0.4673031
ENSG0000023	CASTOR1	protein_codir	0.2486648	0.22002751	0.36086128
ENSG0000027	RP11-438L19.	lncRNA	0.2486526	0.59075475	0.72022274
ENSG0000023	RP11-120D5.1	lncRNA	0.24865231	0.7327004	0.82837433
ENSG0000013	ACAP3	protein_codir	0.24864117	0.28865791	0.43825481
ENSG0000008	RSBN1	protein_codir	0.24862779	0.05806725	0.13451458
ENSG0000016	PDXK	protein_codir	0.24856877	0.16967214	0.29834125
ENSG0000017	SLC25A22	protein_codir	0.24852653	0.29063521	0.4404337
ENSG0000026	RP11-498C9.3	lncRNA	0.24841926	0.46083796	0.6112018
ENSG0000022	MEG9	lncRNA	0.24836324	0.70226171	0.80607025
ENSG0000026	RP11-318A15	lncRNA	0.24815469	0.76743238	0.85332054
ENSG0000027	RP11-350N15	lncRNA	0.24813491	0.4960867	0.64201644
ENSG0000010	ATP11C	protein_codir	0.24812173	0.20213546	0.33919705
ENSG0000027	RBAK-RBAKDI	protein_codir	0.24811321	0.69049122	0.7969648
ENSG0000025	RP11-638I2.6	lncRNA	0.24809842	0.76203113	0.84979756
ENSG0000015	CACHD1	protein_codir	0.24801302	0.53706503	0.67708518
ENSG0000013	MUTYH	protein_codir	0.24799599	0.28916971	0.43871835
ENSG0000023	LINC01828	lncRNA	0.24784953	0.79640908	0.87351358
ENSG0000027	RP11-3D4.4	lncRNA	0.2474717	0.78247344	0.86399282
ENSG0000027	AC114271.2	lncRNA	0.24719332	0.37565751	0.52916157
ENSG0000019	ATG7	protein_codir	0.24707335	0.16648127	0.29434212
ENSG0000026	RP11-209D14	lncRNA	0.24704966	0.78332344	0.86444755
ENSG0000014	GALNS	protein_codir	0.24697183	0.2049943	0.3428156
ENSG0000000	TSPOAP1	protein_codir	0.2468916	0.30656494	0.4582133
ENSG0000027	RP11-44N11.2	lncRNA	0.24685211	0.68843049	0.79555041
ENSG0000006	WAPL	protein_codir	0.24680503	0.13167825	0.24758021
ENSG0000023	RP1-225E12.2	lncRNA	0.24667792	0.74848281	0.83965258
ENSG0000028	CTBP1-AS	lncRNA	0.24645907	0.52088649	0.66346262
ENSG0000024	CNTF	protein_codir	0.24638376	0.43164144	0.58286578
ENSG0000015	OXSM	protein_codir	0.2461621	0.28299702	0.43244137
ENSG0000017	SRP72	protein_codir	0.24610928	0.04673411	0.11433093
ENSG0000012	SNX6	protein_codir	0.24598762	0.11510203	0.22394463
ENSG0000028	CTD-2124B8.3	lncRNA	0.24597756	0.64596384	0.76343693
ENSG0000008	BAX	protein_codir	0.24595963	0.14617895	0.26745369
ENSG0000019	EME2	protein_codir	0.24595632	0.45127402	0.60200409
ENSG0000028	AC002407.3	lncRNA	0.24585162	0.90101583	NA
ENSG0000027	RP11-442J21.	lncRNA	0.24574471	0.90157718	NA
ENSG0000013	PRKCSH	protein_codir	0.24573969	0.00183851	0.00919423
ENSG0000017	RPL4	protein_codir	0.24572103	0.10467987	0.20878523
ENSG0000015	PTDSS1	protein_codir	0.24555919	0.13063081	0.2460463
ENSG0000026	RP11-439E19.	lncRNA	0.24549629	0.33708826	0.48994253
ENSG0000028	LINC01338	lncRNA	0.24536652	0.886064	NA
ENSG0000011	RNGTT	protein_codir	0.24535095	0.1746641	0.3047134
ENSG0000009	HSD3B7	protein_codir	0.24531143	0.14062122	0.25988345
ENSG0000023	RP6-109B7.4	lncRNA	0.24526439	0.75302101	0.84289525

ENSG000001:	PPHLN1	protein_codir	0.24515947	0.01843143	0.05567933
ENSG000001:	TAF1B	protein_codir	0.2451219	0.16831702	0.29673302
ENSG000001:	CCDC148	protein_codir	0.24503972	0.63766639	0.75709693
ENSG000000:	DCT	protein_codir	0.24503591	0.49729456	0.64297705
ENSG000002:	DGCR5	lncRNA	0.2449274	0.69026073	0.79685451
ENSG000001:	UXS1	protein_codir	0.24479863	0.02525572	0.07117989
ENSG000002:	RP13-516M1	lncRNA	0.24474265	0.73447739	0.82970035
ENSG000001:	WDR43	protein_codir	0.24436417	0.20977635	0.34889039
ENSG000001:	TBC1D24	protein_codir	0.24429984	0.07388335	0.16122762
ENSG000001:	PGLS	protein_codir	0.24415458	0.09092433	0.18762272
ENSG000001:	FAM102B	protein_codir	0.24411152	0.33854159	0.49135673
ENSG000001:	MISP3	protein_codir	0.24409585	0.43059046	0.58207266
ENSG000000:	MARK2	protein_codir	0.24408977	0.0764203	0.16515889
ENSG000002:	HELZ-AS1	lncRNA	0.2440871	0.65779867	0.7726302
ENSG000001:	KATNAL2	protein_codir	0.24377522	0.35078607	0.50438395
ENSG000001:	CSTF1	protein_codir	0.24374688	0.15972675	0.28535917
ENSG000001:	APEX1	protein_codir	0.2436451	0.04882594	0.11831659
ENSG000001:	DNAI3	protein_codir	0.24348942	0.60395003	0.73056978
ENSG000001:	PIP4K2A	protein_codir	0.24332065	0.12746676	0.24163568
ENSG000001:	PAFAH1B2	protein_codir	0.24317006	0.03522913	0.09199477
ENSG000002:	RP11-8P13.6	lncRNA	0.24314881	0.91638763	NA
ENSG000002:	LGALS8-AS1	lncRNA	0.24312176	0.77049905	0.85556576
ENSG000001:	B3GALT2	protein_codir	0.24299826	0.58144305	0.71283489
ENSG000001:	ZNF251	protein_codir	0.24294096	0.22475947	0.36646142
ENSG000001:	ZNF236	protein_codir	0.24291167	0.01239701	0.0410903
ENSG000002:	TUSC8	lncRNA	0.24290569	0.91791481	NA
ENSG000001:	NME9	protein_codir	0.24288128	0.6770536	0.78734455
ENSG000000:	ZNF275	protein_codir	0.24284907	0.14579078	0.2668837
ENSG000001:	CDK5RAP1	protein_codir	0.24282907	0.18603918	0.31931409
ENSG000001:	SRD5A3	protein_codir	0.24280974	0.25945109	0.40653765
ENSG000002:	AF131216.7	lncRNA	0.24269602	0.6333084	0.75357479
ENSG000002:	MT1H	protein_codir	0.24233299	0.82955419	0.89714894
ENSG000002:	ZNF232-AS1	lncRNA	0.24228281	0.52789147	0.6694074
ENSG000002:	CTD-2005H7.:	lncRNA	0.24219038	0.83265533	NA
ENSG000001:	ATXN7L2	protein_codir	0.24216962	0.39855472	0.55172822
ENSG000001:	KLHL28	protein_codir	0.2421661	0.19056874	0.32522915
ENSG000001:	FAM193A	protein_codir	0.24207542	0.00942854	0.03323756
ENSG000002:	RP11-319G6.1	lncRNA	0.24199076	0.34708075	0.50027213
ENSG000001:	NACA	protein_codir	0.24199053	0.05760025	0.13368862
ENSG000001:	SPCS1	protein_codir	0.24187153	0.05594783	0.13085605
ENSG000002:	HAUS5	protein_codir	0.24186095	0.26755336	0.41532779
ENSG000001:	ZNF852	protein_codir	0.24181002	0.34675375	0.49988719
ENSG000002:	RP11-138C9.1	lncRNA	0.24177404	0.83291532	0.89934015
ENSG000002:	MCTS1	protein_codir	0.24176481	0.16510573	0.29241553
ENSG000001:	MDN1	protein_codir	0.24174361	0.09323739	0.19139478
ENSG000001:	TECTA	protein_codir	0.24170305	0.32900221	0.48133063
ENSG000002:	POLR2J2	protein_codir	0.24166149	0.67759034	0.78779063

ENSG0000007	PHRF1	protein_codir	0.24120863	0.00094421	0.00547189
ENSG0000027	POC1B-AS1	lncRNA	0.2411799	0.38615604	0.53918817
ENSG0000017	NAT1	protein_codir	0.24110132	0.37212519	0.52551885
ENSG0000022	HOTAIR	lncRNA	0.24104218	NA	NA
ENSG0000022	PROB1	protein_codir	0.24099755	0.49332226	0.63980404
ENSG0000010	RPS6KB1	protein_codir	0.24095868	0.07554062	0.16378759
ENSG0000013	HNRNPD	protein_codir	0.24091368	0.03098461	0.08345376
ENSG0000026	KCNQ1OT1	lncRNA	0.24090796	0.50195281	0.64666899
ENSG0000020	HSPA1B	protein_codir	0.24085713	0.71298464	0.81398073
ENSG0000010	PPP4R3A	protein_codir	0.24085705	0.03651177	0.09454858
ENSG0000010	SMARCD2	protein_codir	0.24071744	0.09271119	0.19058373
ENSG0000010	FBXL20	protein_codir	0.24057965	0.05202804	0.12393398
ENSG0000026	ASB16-AS1	lncRNA	0.24056232	0.29538462	0.44571421
ENSG0000023	RP11-385F5.4	lncRNA	0.24048599	0.61153433	0.73658271
ENSG0000019	IRF1-AS1	lncRNA	0.24044246	0.25003263	0.39595709
ENSG0000009	G2E3	protein_codir	0.24043591	0.07854516	0.16836766
ENSG0000024	C1QTNF9	protein_codir	0.24035812	0.69983089	0.80411553
ENSG0000017	RCE1	protein_codir	0.24015099	0.09687128	0.19696591
ENSG0000005	YIPF1	protein_codir	0.24010914	0.03206652	0.08553136
ENSG0000027	RP11-767C1.2	lncRNA	0.24009511	0.80897299	0.88193542
ENSG0000013	FAM186B	protein_codir	0.24007766	0.55081423	0.68783295
ENSG0000020	PRDX6-AS1	lncRNA	0.24000437	0.43265961	0.58395352
ENSG0000026	AC002551.1	lncRNA	0.23973614	0.87231468	0.92423105
ENSG0000020	SNX2	protein_codir	0.23969246	0.05520821	0.129532
ENSG0000027	RP11-554D20	lncRNA	0.23966919	0.78014053	0.8625548
ENSG0000017	BEND3	protein_codir	0.23916673	0.26792035	0.41575144
ENSG0000006	GNAI3	protein_codir	0.23911107	0.07331403	0.16035268
ENSG0000024	LY6G5B	protein_codir	0.23910631	0.49443269	0.64053616
ENSG0000013	TMX1	protein_codir	0.23908582	0.1329383	0.2494044
ENSG0000010	GGA1	protein_codir	0.2390536	0.26279808	0.41015076
ENSG0000009	MACROH2A2	protein_codir	0.23904066	0.22451562	0.36623789
ENSG0000026	ZNF8-ERVK3-1	lncRNA	0.23902913	0.43115696	0.58249634
ENSG0000015	CPT2	protein_codir	0.23863884	0.28382677	0.43340182
ENSG0000020	LINC01121	lncRNA	0.23861951	0.7018267	0.80573836
ENSG0000017	ZNF692	protein_codir	0.23855278	0.50071647	0.64585947
ENSG0000013	VPS36	protein_codir	0.23852178	0.01756289	0.05366602
ENSG0000018	KLHL34	protein_codir	0.23851155	0.58580448	0.71636942
ENSG0000025	RHOXF1-AS1	lncRNA	0.23821699	0.71901299	0.81863864
ENSG0000025	RP11-549J18.	lncRNA	0.23817299	0.48581759	0.6330274
ENSG0000026	RP11-326K13.	lncRNA	0.23803657	0.46825833	0.61763586
ENSG0000016	ZNF701	protein_codir	0.23803141	0.27342567	0.42140601
ENSG0000018	ANKFY1	protein_codir	0.23770254	0.04471091	0.11060594
ENSG0000019	RPS26	protein_codir	0.23750028	0.57370602	0.70649481
ENSG0000028	AC006130.5	lncRNA	0.23707767	0.54505272	0.6830872
ENSG0000016	SHKBP1	protein_codir	0.23693947	0.19195461	0.32670608
ENSG0000027	RP11-45A17.2	lncRNA	0.2369127	0.62072367	0.7439749
ENSG0000022	RP11-82L18.2	lncRNA	0.23680289	0.7038058	0.80713819

ENSG0000015	CARHSP1	protein_codir	0.23680264	0.19539641	0.33113566
ENSG0000027	RP11-90B9.3	lncRNA	0.23633024	0.79337323	0.87167225
ENSG0000014	CCDC81	protein_codir	0.23629919	0.53245852	0.67344823
ENSG0000006	GPBP1	protein_codir	0.23614815	0.00540101	0.02154787
ENSG0000011	NUP98	protein_codir	0.23601528	0.14873433	0.27081139
ENSG0000016	IL15	protein_codir	0.2359822	0.29138682	0.44121033
ENSG0000014	SIDT2	protein_codir	0.23591905	0.12299294	0.23543315
ENSG0000012	ARAP3	protein_codir	0.23588482	0.24875641	0.39443435
ENSG0000014	PPIP5K2	protein_codir	0.23576603	0.07483464	0.16260976
ENSG0000014	LYG1	protein_codir	0.23563581	0.64779499	0.76447378
ENSG0000015	TRIM11	protein_codir	0.23554667	0.13635372	0.25369416
ENSG0000015	C21orf91	protein_codir	0.23551721	0.14852494	0.27052617
ENSG0000016	KIAA1841	protein_codir	0.235515	0.10563478	0.21023437
ENSG0000012	HOXB8	protein_codir	0.23538192	0.59649846	0.7247619
ENSG0000025	TRAPPC2B	protein_codir	0.23537888	0.07656744	0.16537321
ENSG0000004	YTHDC2	protein_codir	0.2349803	0.03535413	0.09226011
ENSG0000023	ZNF674-AS1	lncRNA	0.2348606	0.44617522	0.59711985
ENSG0000013	CASP9	protein_codir	0.23471796	0.14098366	0.26030942
ENSG0000017	MFSD4B	protein_codir	0.23461444	0.33755927	0.49039486
ENSG0000011	ID2	protein_codir	0.23449021	0.46125541	0.6114944
ENSG0000016	ANKRD23	protein_codir	0.23441072	0.45980265	0.61033012
ENSG0000013	SYCP3	protein_codir	0.2341955	0.70105876	0.80519141
ENSG0000026	RP11-403P17.	protein_codir	0.23411764	0.79751734	0.87413289
ENSG0000009	GEMIN2	protein_codir	0.23402394	0.2246827	0.3663795
ENSG0000026	RP11-20I23.1	lncRNA	0.23400427	0.36726137	0.52108007
ENSG0000014	ABT1	protein_codir	0.23391888	0.03028396	0.08206859
ENSG0000015	TATDN2	protein_codir	0.2338637	0.0669686	0.15030658
ENSG0000011	RFC5	protein_codir	0.23385592	0.12929941	0.24415463
ENSG0000013	HNRNPA1L2	protein_codir	0.23371852	0.59963685	0.72726652
ENSG0000009	SUCO	protein_codir	0.23346121	0.08558713	0.17957433
ENSG0000014	GALM	protein_codir	0.23343514	0.37262355	0.52614194
ENSG0000016	ORC5	protein_codir	0.23316813	0.14508141	0.26597296
ENSG0000012	ARMC7	protein_codir	0.2328194	0.28667701	0.43630256
ENSG0000016	RNF168	protein_codir	0.23277823	0.05564282	0.13034133
ENSG0000025	RP11-298I3.5	protein_codir	0.23269675	0.7636918	0.85083972
ENSG0000011	ALDH5A1	protein_codir	0.23269324	0.4255321	0.57743092
ENSG0000012	IQSEC3	protein_codir	0.23259692	0.42262374	0.57462492
ENSG0000027	CTB-147N14.6	lncRNA	0.23248558	0.40819038	0.56115765
ENSG0000017	C2orf69	protein_codir	0.23244351	0.11239788	0.22013668
ENSG0000014	FBLN7	protein_codir	0.2323818	0.46175294	0.61188966
ENSG0000000	NADK	protein_codir	0.23234758	0.09202623	0.18942947
ENSG0000024	XACT	lncRNA	0.23186998	NA	NA
ENSG0000027	RP11-123K3.9	lncRNA	0.2316104	0.85362044	0.91285717
ENSG0000020	DOK6	protein_codir	0.23154152	0.52918704	0.67069606
ENSG0000010	NDRG4	protein_codir	0.23148932	0.56404298	0.69833196
ENSG0000002	MRE11	protein_codir	0.23140986	0.07655964	0.1653693
ENSG0000013	WDR36	protein_codir	0.23136238	0.21662117	0.35692159

ENSG0000015	FBXO4	protein_codir	0.23126998	0.04572106	0.11247022
ENSG0000016	CREBRF	protein_codir	0.23119432	0.26986207	0.41768509
ENSG0000007	NSF	protein_codir	0.2311771	0.20228746	0.33933675
ENSG0000013	PPARG	protein_codir	0.23108498	0.57960349	0.71143225
ENSG0000009	USP48	protein_codir	0.23100851	0.02072625	0.06096475
ENSG0000025	RP11-99E15.2	lncRNA	0.23095543	0.87907274	NA
ENSG0000018	LIN9	protein_codir	0.23083006	0.27164858	0.41962717
ENSG0000028	RP11-326F6.1	lncRNA	0.23056969	0.2814386	0.43082104
ENSG0000011	SRSF11	protein_codir	0.23051267	0.36225132	0.51611377
ENSG0000017	HOXD8	protein_codir	0.23043843	0.56507907	0.69935403
ENSG0000004	TRIT1	protein_codir	0.23043133	0.07932974	0.16963086
ENSG0000010	ASAH1	protein_codir	0.23039283	0.18020485	0.31172155
ENSG0000010	EIF3D	protein_codir	0.2303846	0.01148166	0.03880136
ENSG0000014	STK11IP	protein_codir	0.23027286	0.22615682	0.36817466
ENSG0000010	GTPBP1	protein_codir	0.23026501	0.07478343	0.16254965
ENSG0000008	FAM234B	protein_codir	0.23023513	0.28412162	0.43372792
ENSG0000027	RP11-260M2.	lncRNA	0.23002416	0.69728824	0.80188618
ENSG0000014	PXDNL	protein_codir	0.22996807	0.64645996	0.76366384
ENSG0000016	RPL9	protein_codir	0.22987339	0.20596538	0.34398707
ENSG0000027	RP11-834C11.	lncRNA	0.22976864	0.71999982	0.81920049
ENSG0000019	SMC5	protein_codir	0.22976275	0.05718101	0.13295222
ENSG0000020	RP11-498P14.	lncRNA	0.22968677	0.7082706	0.81057795
ENSG0000009	DNAJB11	protein_codir	0.22963626	0.14912796	0.27130249
ENSG0000008	FTL	protein_codir	0.22947341	0.34234768	0.49560909
ENSG0000026	CTD-2006C1.1	lncRNA	0.22941797	0.81916014	0.88900602
ENSG0000025	PCDHGA10	protein_codir	0.22899112	0.40131431	0.55435365
ENSG0000013	PPCDC	protein_codir	0.22878918	0.27512798	0.42323773
ENSG0000019	LAGE3	protein_codir	0.2282992	0.17354539	0.30343252
ENSG0000013	ZNF557	protein_codir	0.22823478	0.36751849	0.52114716
ENSG0000012	DUSP1	protein_codir	0.22823471	0.58913243	0.71907012
ENSG0000001	APBA3	protein_codir	0.22819039	0.20197769	0.33908484
ENSG0000023	RP11-374M1.	lncRNA	0.22799841	0.84624987	0.90842326
ENSG0000018	C1orf116	protein_codir	0.22798164	0.775457	0.85906154
ENSG0000009	TRPM7	protein_codir	0.2278836	0.13152965	0.24740935
ENSG0000018	AFMID	protein_codir	0.22788097	0.1968401	0.33286742
ENSG0000015	ATP6V0D1	protein_codir	0.22768205	0.1228071	0.23515892
ENSG0000016	ADGRV1	protein_codir	0.22758046	0.79470727	0.87244385
ENSG0000024	LINC02211	lncRNA	0.2275624	0.85382743	0.91285717
ENSG0000027	RP4-794I6.4	lncRNA	0.22748396	0.68964275	0.79628497
ENSG0000005	GDI2	protein_codir	0.22746118	0.03786838	0.0972049
ENSG0000013	RARA	protein_codir	0.22734719	0.36126086	0.5151463
ENSG0000002	ZDHHC6	protein_codir	0.22726441	0.1038018	0.20751344
ENSG0000007	TMEM131	protein_codir	0.22722698	0.06318494	0.14375355
ENSG0000017	RPS6KB2	protein_codir	0.22718528	0.23118948	0.37390622
ENSG0000017	CLK3	protein_codir	0.2271798	0.16005677	0.28574546
ENSG0000012	THOC2	protein_codir	0.22681987	0.03678709	0.09511885
ENSG0000012	PHF24	protein_codir	0.22669962	0.58543589	0.71613578

ENSG0000024	NSUN6	protein_codir	0.22665366	0.32433362	0.47667188
ENSG0000026	AC005944.2	lncRNA	0.2264748	0.53185626	0.67285955
ENSG0000012	STX16	protein_codir	0.22625152	0.20839245	0.3471113
ENSG0000008	XRN2	protein_codir	0.22618554	0.14649004	0.2679329
ENSG0000014	ZNF182	protein_codir	0.22606319	0.26191195	0.40937175
ENSG0000016	RFT1	protein_codir	0.22598193	0.17077528	0.29984192
ENSG0000011	UAP1	protein_codir	0.22593295	0.55139061	0.68836597
ENSG0000019	MT-CO2	protein_codir	0.22590619	0.49814932	0.64390135
ENSG0000000	AK2	protein_codir	0.22580117	0.01111338	0.03783452
ENSG0000027	INSIG1-DT	lncRNA	0.2257548	0.49178721	0.63842652
ENSG0000010	HM13	protein_codir	0.22570826	0.15183837	0.27471346
ENSG0000022	AC009950.2	lncRNA	0.22564961	0.70934465	0.81126705
ENSG0000020	TMSB4X	protein_codir	0.22550664	0.15075175	0.27329088
ENSG0000011	RPL24	protein_codir	0.22546957	0.11520688	0.2240855
ENSG0000019	FAM3C	protein_codir	0.22536866	0.19107433	0.32564933
ENSG0000022	DANCR	lncRNA	0.2253348	0.31267173	0.46441196
ENSG0000012	SLC36A1	protein_codir	0.22523483	0.3711633	0.5246005
ENSG0000011	NAA25	protein_codir	0.22522938	0.09678574	0.19684993
ENSG0000022	AC104134.2	lncRNA	0.22515189	0.89638354	NA
ENSG0000024	EAF1-AS1	lncRNA	0.22507723	0.67601929	0.78653915
ENSG0000021	FAM187A	protein_codir	0.22485282	0.57885357	0.7108891
ENSG0000016	VWCE	protein_codir	0.22468441	0.48698361	0.63421762
ENSG0000014	PAXX	protein_codir	0.22463264	0.36937535	0.52289624
ENSG0000015	PHKG2	protein_codir	0.22457058	0.25829188	0.40531912
ENSG0000012	MCM8	protein_codir	0.22456253	0.2415831	0.38629798
ENSG0000023	LINC00345	lncRNA	0.22454235	0.71818192	0.81798699
ENSG0000027	RP11-219D15	lncRNA	0.22452837	0.80780161	0.88100614
ENSG0000013	POC1B	protein_codir	0.22445734	0.06326005	0.14388887
ENSG0000006	QSER1	protein_codir	0.22415682	0.24501044	0.39004837
ENSG0000016	SPSB3	protein_codir	0.22393114	0.33280706	0.48555972
ENSG0000012	C16orf70	protein_codir	0.22371532	0.20710953	0.34555788
ENSG0000028	CTD-2267D19	lncRNA	0.22344413	0.77759552	0.86071402
ENSG0000010	TRIB3	protein_codir	0.22337102	0.57311679	0.70610607
ENSG0000023	SMG7-AS1	lncRNA	0.22335407	0.82375179	0.89283147
ENSG0000015	TNNI1	protein_codir	0.22329558	0.85513678	0.91336696
ENSG0000026	RP11-378A13	lncRNA	0.22265931	0.30252984	0.45392623
ENSG0000015	C4orf33	protein_codir	0.222657	0.08828655	0.18361908
ENSG0000013	C12orf65	protein_codir	0.22259688	0.28598521	0.43553769
ENSG0000013	LPIN1	protein_codir	0.22250103	0.25573811	0.40245494
ENSG0000011	TPO	protein_codir	0.22240053	0.68499403	0.79303915
ENSG0000016	SRRM2	protein_codir	0.2222581	0.15451933	0.27843318
ENSG0000017	YES1	protein_codir	0.2221521	0.39046119	0.54346594
ENSG0000013	PPT1	protein_codir	0.22197881	0.25562449	0.40232197
ENSG0000027	CWC25	protein_codir	0.22195122	0.22945487	0.37194339
ENSG0000010	ETFB	protein_codir	0.22186984	0.11868812	0.22909819
ENSG0000016	METTL17	protein_codir	0.22186689	0.35812546	0.51171367
ENSG0000020	OR2L2	protein_codir	0.2217746	0.78321425	0.86437958

ENSG0000004	TSPAN17	protein_codir	0.2216114	0.11240292	0.22013668
ENSG0000019	AMZ2	protein_codir	0.22130804	0.02173087	0.06318628
ENSG0000027	XXbac-BPG29	lncRNA	0.22124349	0.40281173	0.55578279
ENSG0000027	TMCC1-DT	lncRNA	0.22116037	0.61858976	0.74216799
ENSG0000027	RP11-152F13.	lncRNA	0.22107849	0.67952977	0.78937833
ENSG0000016	ZFYVE19	protein_codir	0.22093415	0.21455968	0.35451892
ENSG0000024	RP11-281P23.	lncRNA	0.22084922	0.89902547	0.93983381
ENSG0000027	RP11-803D5.4	lncRNA	0.22076602	0.78735755	0.86727081
ENSG0000007	MARK3	protein_codir	0.2205927	0.12155858	0.23335306
ENSG0000011	RPL22	protein_codir	0.22058185	0.09915049	0.20047851
ENSG0000016	TBC1D2B	protein_codir	0.22049497	0.09858521	0.19961837
ENSG0000011	RND3	protein_codir	0.22045312	0.59308871	0.72185777
ENSG0000019	ZNF493	protein_codir	0.22027184	0.44182135	0.59290184
ENSG0000010	CCNJ	protein_codir	0.22023496	0.13560152	0.25272024
ENSG0000019	RPL37A	protein_codir	0.22010497	0.20785232	0.34646234
ENSG0000010	SARS2	protein_codir	0.22004011	0.30649752	0.45813732
ENSG0000002	PHF20	protein_codir	0.21973606	0.11699696	0.22664992
ENSG0000026	RP11-819C21.	lncRNA	0.21961268	0.28882026	0.43843305
ENSG0000026	CTD-2017D11	lncRNA	0.21959658	0.53356726	0.67428293
ENSG0000017	IQCB1	protein_codir	0.21957008	0.21681307	0.35706738
ENSG0000004	ADAMTS6	protein_codir	0.21956704	0.51898943	0.66191762
ENSG0000027	RP11-372K14.	lncRNA	0.2194087	0.64280848	0.7607208
ENSG0000014	TMF1	protein_codir	0.21940761	0.14067852	0.2599403
ENSG0000021	ASIC3	protein_codir	0.21936257	0.59028197	0.71993235
ENSG0000014	POU2F1	protein_codir	0.21928711	0.16813651	0.29652835
ENSG0000019	ZNF335	protein_codir	0.21926636	0.20976303	0.3488896
ENSG0000028	RP11-358L8.1	lncRNA	0.21918317	0.8827164	NA
ENSG0000018	TMEM50A	protein_codir	0.21907703	0.07075476	0.1563022
ENSG0000016	ADAMTS9	protein_codir	0.21896045	0.71289476	0.81394541
ENSG0000016	PROKR1	protein_codir	0.21879009	0.76641488	0.85277279
ENSG0000012	SLC66A2	protein_codir	0.21863576	0.11135436	0.21865544
ENSG0000018	NR2F2	protein_codir	0.21856688	0.42818133	0.57986606
ENSG0000016	RFC4	protein_codir	0.21856436	0.24197745	0.3868166
ENSG0000021	ATF6B	protein_codir	0.21856073	0.05157051	0.12319474
ENSG0000008	PPP1R13B	protein_codir	0.21843648	0.15280026	0.27607399
ENSG0000017	HDAC3	protein_codir	0.21843447	0.01437223	0.04609395
ENSG0000012	RPP40	protein_codir	0.21840345	0.38950363	0.54246119
ENSG0000014	PIK3R1	protein_codir	0.21816245	0.3810384	0.5343902
ENSG0000010	TAF1C	protein_codir	0.21811454	0.46918717	0.61857898
ENSG0000017	SWSAP1	protein_codir	0.21811095	0.47254364	0.62112289
ENSG0000025	ARPC4-TTLL3	protein_codir	0.21802753	0.45099572	0.60170523
ENSG0000028	RP11-553D4.3	protein_codir	0.21794658	0.30085015	0.45192153
ENSG0000023	C2orf74	protein_codir	0.21785868	0.53626372	0.67642161
ENSG0000023	CTB-107G13.1	lncRNA	0.21784396	0.8503982	0.91091713
ENSG0000022	RP11-162D16	lncRNA	0.21773538	0.7111802	0.81259244
ENSG0000017	USP32	protein_codir	0.21762464	0.18707253	0.32054987
ENSG0000019	KPNA5	protein_codir	0.21756424	0.0937751	0.19222713

ENSG0000027	CTD-2017F17.	lncRNA	0.21752869	0.84346371	0.90639867
ENSG0000028	AC000085.4	lncRNA	0.21751023	0.8196058	0.8893499
ENSG0000013	PHF11	protein_codir	0.21745607	0.15211546	0.27510666
ENSG0000014	DPH7	protein_codir	0.21701147	0.35592492	0.50951832
ENSG0000017	RIOX2	protein_codir	0.2168822	0.05998724	0.13795566
ENSG0000017	SLC25A20	protein_codir	0.21682279	0.20908327	0.34801039
ENSG0000013	UBE2G1	protein_codir	0.21681753	0.15605608	0.28043389
ENSG0000018	APOO	protein_codir	0.21670995	0.28605074	0.43556544
ENSG0000022	MIR3681HG	lncRNA	0.21657434	0.7807121	0.86289827
ENSG0000000	TENM1	protein_codir	0.2165713	0.45395734	0.60434289
ENSG0000016	CEP295	protein_codir	0.21647838	0.18216949	0.31438084
ENSG0000014	MANF	protein_codir	0.21637342	0.29300333	0.44312281
ENSG0000028	RP11-401F12.	lncRNA	0.21618226	0.90161307	0.9414598
ENSG0000027	RP11-177J6.1	lncRNA	0.21609948	0.65801421	0.77275192
ENSG0000016	R3HCC1L	protein_codir	0.21609005	0.09050936	0.18698994
ENSG0000016	TRIM66	protein_codir	0.21607205	0.38727613	0.54029101
ENSG0000010	PCNX1	protein_codir	0.21605379	0.18644969	0.31981093
ENSG0000028	RP11-775C24.	protein_codir	0.21603986	0.864245	0.91911928
ENSG0000014	ZNF787	protein_codir	0.21601577	0.16068558	0.28659019
ENSG0000013	MTO1	protein_codir	0.21601361	0.09647027	0.19633843
ENSG0000028	CTA-38K21.6	lncRNA	0.21594743	0.63662818	0.75630281
ENSG0000006	SNX24	protein_codir	0.21588449	0.22286462	0.36416735
ENSG0000025	RP11-380O24	lncRNA	0.2157964	0.75958583	0.84799674
ENSG0000015	CNOT8	protein_codir	0.21571704	0.03548908	0.09255107
ENSG0000014	PAK1	protein_codir	0.21549028	0.20397205	0.34143685
ENSG0000013	SNRPA1	protein_codir	0.21522855	0.29919361	0.4502167
ENSG0000027	RP11-390P24.	lncRNA	0.21520597	0.4746819	0.622907
ENSG0000018	SETD2	protein_codir	0.21501843	0.08530412	0.17907739
ENSG0000012	DEK	protein_codir	0.21501209	0.10145909	0.20396682
ENSG0000022	AC092198.1	lncRNA	0.21497372	0.85415824	0.91289133
ENSG0000017	CLCF1	protein_codir	0.21484138	0.62322093	0.74574829
ENSG0000016	PTER	protein_codir	0.21474131	0.36311713	0.51702727
ENSG0000018	C6orf120	protein_codir	0.21457391	0.28488151	0.43452768
ENSG0000011	UBE3D	protein_codir	0.21450981	0.32238904	0.47452145
ENSG0000022	RP5-884G6.2	lncRNA	0.21448952	0.57611318	0.70862819
ENSG0000015	ZDHHC7	protein_codir	0.21448264	0.07773941	0.16712037
ENSG0000012	NUP85	protein_codir	0.2142708	0.23293491	0.37597371
ENSG0000028	RP11-11N7.6	lncRNA	0.21425318	0.79116147	0.87007286
ENSG0000017	PSTK	protein_codir	0.21417214	0.32019786	0.47232776
ENSG0000023	ZNF737	protein_codir	0.21414785	0.58260552	0.7137848
ENSG0000008	SLC8B1	protein_codir	0.21412931	0.15087944	0.2734263
ENSG0000011	FGFR1OP2	protein_codir	0.21391734	0.07114465	0.15687491
ENSG0000027	WDR5B-DT	lncRNA	0.21325826	0.60568815	0.73206313
ENSG0000018	UROS	protein_codir	0.21316473	0.06179214	0.14118953
ENSG0000016	SPRED1	protein_codir	0.21299987	0.33979803	0.49276602
ENSG0000010	WDR91	protein_codir	0.21299684	0.37320822	0.52682832
ENSG0000019	COLCA1	lncRNA	0.21295105	0.64348327	0.76135027

ENSG0000018	ZDHHC20	protein_codir	0.21290302	0.24679182	0.39199171
ENSG0000028	KB-1995A5.6	protein_codir	0.21270348	0.17436504	0.30440317
ENSG0000011	BBOF1	protein_codir	0.21249861	0.39160201	0.54455984
ENSG0000022	NUTM2B-AS1	lncRNA	0.21247796	0.20156939	0.33860519
ENSG0000016	C15orf39	protein_codir	0.21246089	0.17489582	0.30501916
ENSG0000025	AC156455.1	lncRNA	0.21245663	0.60540858	0.73188537
ENSG0000018	MORN5	protein_codir	0.21231491	0.88795537	0.93348541
ENSG0000014	EIF3H	protein_codir	0.21223313	0.04471662	0.11061016
ENSG0000025	MINCR	lncRNA	0.21215753	0.49865715	0.64434669
ENSG0000010	RHBDD3	protein_codir	0.21205108	0.18000792	0.31143941
ENSG0000028	C13orf46	protein_codir	0.21172456	0.42084944	0.57295797
ENSG0000016	RAB4B	protein_codir	0.21171421	0.41479021	0.56788635
ENSG0000001	SLC6A7	protein_codir	0.21154547	0.72226199	0.82077252
ENSG0000011	PRPF4B	protein_codir	0.21143806	0.04924311	0.11911848
ENSG0000015	CATIP	protein_codir	0.21130093	0.78417118	0.86505563
ENSG0000017	CLK2	protein_codir	0.21124412	0.3837083	0.53707106
ENSG0000017	ZNF131	protein_codir	0.21113139	0.02877181	0.07890749
ENSG0000009	IKZF5	protein_codir	0.21112824	0.04784827	0.1163763
ENSG0000014	CREG1	protein_codir	0.21110089	0.24217023	0.38701278
ENSG0000012	EPC1	protein_codir	0.21108973	0.07024682	0.15543279
ENSG0000023	LINC01625	lncRNA	0.21108885	0.77827262	0.86112999
ENSG0000009	RFFL	protein_codir	0.21087419	0.1001468	0.20197089
ENSG0000026	MADCAM1-AS	lncRNA	0.21080816	0.64612723	0.76350479
ENSG0000018	NSMCE3	protein_codir	0.21068738	0.05653508	0.13190563
ENSG0000016	WEE1	protein_codir	0.21066599	0.51926703	0.66202711
ENSG0000015	SPEF2	protein_codir	0.21058734	0.35565994	0.50928232
ENSG0000023	ASH1L-AS1	lncRNA	0.2105555	0.41872501	0.57137418
ENSG0000007	THOC1	protein_codir	0.21046943	0.35520423	0.50888224
ENSG0000016	HEPACAM	protein_codir	0.21019895	0.74230954	0.83552202
ENSG0000016	C2orf68	protein_codir	0.21013765	0.21275443	0.35235817
ENSG0000009	CIRBP	protein_codir	0.21010609	0.22258725	0.36379998
ENSG0000011	BICRAL	protein_codir	0.21006594	0.161312	0.28738753
ENSG0000016	NSD1	protein_codir	0.20985792	0.1705237	0.29951444
ENSG0000011	ALG2	protein_codir	0.20981725	0.10371686	0.20738868
ENSG0000019	TMEM63A	protein_codir	0.20967997	0.24411916	0.38920407
ENSG0000014	NUDT22	protein_codir	0.20960288	0.25887337	0.40597788
ENSG0000013	TAF8	protein_codir	0.20947505	0.12455859	0.23759036
ENSG0000013	SHFL	protein_codir	0.20935152	0.22898107	0.37145878
ENSG0000017	TRIM72	protein_codir	0.20916875	0.66468827	0.77822032
ENSG0000006	ZNF76	protein_codir	0.2091214	0.25620095	0.40288489
ENSG0000006	KLF6	protein_codir	0.20908566	0.59993137	0.7274609
ENSG0000015	ARMC12	protein_codir	0.20898053	0.62632155	0.74803257
ENSG0000009	SRPK1	protein_codir	0.20884688	0.06312609	0.14365515
ENSG0000015	FSIP1	protein_codir	0.20883638	0.58268669	0.71385258
ENSG0000013	ABHD13	protein_codir	0.20875339	0.09833489	0.19925242
ENSG0000013	CREBZF	protein_codir	0.20870673	0.31841438	0.47044319
ENSG0000014	GALNT13	protein_codir	0.20862949	0.65526679	0.7706067

ENSG0000025	RP11-626H12	lncRNA	0.20860646	0.73151738	0.82737552
ENSG0000021	PLIN5	protein_codir	0.20838224	0.63562753	0.75539058
ENSG0000010	C14orf93	protein_codir	0.20836691	0.20945482	0.34850253
ENSG0000018	WDR27	protein_codir	0.20828437	0.45472973	0.60519612
ENSG0000018	DAZAP2	protein_codir	0.20798291	0.07583334	0.16423785
ENSG0000017	RSPH9	protein_codir	0.20787179	0.61127573	0.73652819
ENSG0000011	RBM25	protein_codir	0.20778916	0.35553747	0.50914839
ENSG0000006	ISOC2	protein_codir	0.20774422	0.15580556	0.28007481
ENSG0000028	RP11-807C20.	protein_codir	0.20770276	0.44536154	0.59649334
ENSG0000017	INHBC	protein_codir	0.20766855	0.81661463	0.88721958
ENSG0000015	USP25	protein_codir	0.20762034	0.16488338	0.29217162
ENSG0000026	RP11-266J6.2	lncRNA	0.20762018	0.75798014	0.84673049
ENSG0000023	CTB-89H12.4	lncRNA	0.20741995	0.55907026	0.69456282
ENSG0000011	SF3B1	protein_codir	0.20740482	0.02705581	0.07505841
ENSG0000028	RP11-122G18	lncRNA	0.2073235	0.62445414	0.74677102
ENSG0000024	ACTN3	protein_codir	0.20727571	0.73401066	0.82944826
ENSG0000024	RP11-894P9.1	lncRNA	0.20719954	0.88665002	0.9328181
ENSG0000014	CCDC39	lncRNA	0.20714887	0.81303373	0.88506983
ENSG0000011	IL1R1	protein_codir	0.20713462	0.48412619	0.63149079
ENSG0000008	WNT11	protein_codir	0.20709125	0.67455535	0.78553076
ENSG0000028	RP11-266K4.1	lncRNA	0.206988	0.74084256	0.83450537
ENSG0000012	MBOAT7	protein_codir	0.20684939	0.20757831	0.34608915
ENSG0000017	ZHX2	protein_codir	0.2066947	0.33096001	0.48358014
ENSG0000015	TRAPPC8	protein_codir	0.20668165	0.08338811	0.17585718
ENSG0000010	ACBD5	protein_codir	0.20650015	0.15359202	0.27717816
ENSG0000025	PXN-AS1	lncRNA	0.20648462	0.36204741	0.51593682
ENSG0000014	COP1	protein_codir	0.20624984	0.03495041	0.09143116
ENSG0000019	FUT4	protein_codir	0.2062478	0.29901989	0.45000433
ENSG0000017	CYCS	protein_codir	0.20613164	0.38487226	0.53811079
ENSG0000001	TTC27	protein_codir	0.20562688	0.25598662	0.40268541
ENSG0000027	RP11-1348G1	lncRNA	0.20562562	0.52784577	0.66938017
ENSG0000019	NCOR2	protein_codir	0.20529766	0.14024426	0.2593777
ENSG0000024	PISD	protein_codir	0.20526323	0.06095421	0.13964376
ENSG0000010	TRMU	protein_codir	0.20513624	0.43098202	0.58237395
ENSG0000016	PUSL1	protein_codir	0.20497507	0.12938619	0.24426839
ENSG0000027	RP11-106D4.2	lncRNA	0.20487099	0.6584173	0.77309381
ENSG0000019	STMN3	protein_codir	0.20485723	0.27140712	0.41944184
ENSG0000007	ZFYVE26	protein_codir	0.20476947	0.15500141	0.27908216
ENSG0000016	DNAJC7	protein_codir	0.20472883	0.08909591	0.18484407
ENSG0000027	RP11-462G22	lncRNA	0.20463835	0.90571345	NA
ENSG0000012	POLM	protein_codir	0.20451505	0.4012594	0.55433053
ENSG0000017	TIGD5	protein_codir	0.20443061	0.09436219	0.19320116
ENSG0000007	RAPGEF3	protein_codir	0.20435729	0.56083884	0.6957891
ENSG0000013	RAP1GAP2	protein_codir	0.20430117	0.56708663	0.70095917
ENSG0000016	VAMP5	protein_codir	0.20422513	0.1210234	0.23258186
ENSG0000027	KB-1958F4.1	lncRNA	0.20401899	0.68110559	0.79034734
ENSG0000012	INHBA	protein_codir	0.20387106	0.62408218	0.74652013

ENSG0000010	RAB40AL	protein_codir	0.20385187	0.81885946	0.88881939
ENSG0000010	IQCH	protein_codir	0.20365516	0.55970741	0.6951228
ENSG0000013	PPAN	protein_codir	0.20349054	0.27543718	0.42360793
ENSG0000027	RP11-313P22.	lncRNA	0.20341317	0.66944872	0.78169332
ENSG0000013	MDM2	protein_codir	0.20337387	0.27156177	0.41958693
ENSG0000022	HAGLROS	lncRNA	0.20335155	0.74026108	0.8339524
ENSG0000014	OSMR	protein_codir	0.20311172	0.46883467	0.61824845
ENSG0000017	SIK2	protein_codir	0.20304199	0.35957092	0.51338065
ENSG0000026	SNHG22	lncRNA	0.20303475	0.46011139	0.61068123
ENSG0000012	CNTFR	protein_codir	0.20295328	0.77896332	0.86165329
ENSG0000014	RNF165	protein_codir	0.20292288	0.66016286	0.77445202
ENSG0000017	KRT15	protein_codir	0.20272883	0.87436397	0.92549755
ENSG0000010	PSMA6	protein_codir	0.20268346	0.06532168	0.1475695
ENSG0000020	PCDHA7	protein_codir	0.20233802	0.73448841	0.82970035
ENSG0000010	SOS2	protein_codir	0.2020353	0.14912036	0.27130249
ENSG0000015	TEX30	protein_codir	0.20189352	0.37869709	0.53217236
ENSG0000022	RP11-384C4.2	lncRNA	0.20185653	0.89160259	0.93542747
ENSG0000028	RALY-AS1	lncRNA	0.20145565	0.51015239	0.65421159
ENSG0000022	RP13-188A5.1	lncRNA	0.20134521	0.44561126	0.59665421
ENSG0000028	CTD-2587H24	lncRNA	0.20130518	0.77522024	0.85892045
ENSG0000010	RBM23	protein_codir	0.20113222	0.02079012	0.06110679
ENSG0000010	ZNF419	protein_codir	0.20103076	0.3823034	0.53578343
ENSG0000022	SLC9A3-AS1	lncRNA	0.20101229	0.62264093	0.7454095
ENSG0000010	PYGL	protein_codir	0.20089258	0.45829614	0.60871071
ENSG0000016	BOLA2B	protein_codir	0.20088076	0.32946431	0.48190459
ENSG0000025	AP003068.23	lncRNA	0.20076232	0.52263097	0.66494775
ENSG0000010	ERLIN1	protein_codir	0.20065852	0.2599055	0.40701877
ENSG0000018	MAML2	protein_codir	0.20049038	0.30714042	0.45884999
ENSG0000016	CCDC12	protein_codir	0.20048277	0.19996067	0.33655781
ENSG0000023	AF131217.1	lncRNA	0.2004567	0.63906611	0.75794426
ENSG0000014	INCENP	protein_codir	0.20029798	0.25978198	0.40691762
ENSG0000028	RP5-1111F22.	lncRNA	0.20026004	0.62464729	0.74693732
ENSG0000011	FAF2	protein_codir	0.20025246	0.07513778	0.16316577
ENSG0000016	ZBTB39	protein_codir	0.20008091	0.3426755	0.49586676
ENSG0000007	NRDC	protein_codir	0.20007086	0.02349824	0.06716563
ENSG0000012	ZNF549	protein_codir	0.19999271	0.35530947	0.50897079
ENSG0000007	TMEM260	protein_codir	0.19999018	0.25068802	0.39667735
ENSG0000020	SDHD	protein_codir	0.19994271	0.03767764	0.09681338
ENSG0000025	ADNP-AS1	lncRNA	0.19991384	0.43821955	0.58935148
ENSG0000006	DNTTIP2	protein_codir	0.1998671	0.34514115	0.49803034
ENSG0000008	AP4E1	protein_codir	0.19977221	0.16169255	0.28782857
ENSG0000008	SRRT	protein_codir	0.1997415	0.25476316	0.40124071
ENSG0000012	NAA50	protein_codir	0.1995719	0.22374424	0.36521577
ENSG0000011	KLF12	protein_codir	0.1992522	0.363002	0.51691665
ENSG0000003	UBA6	protein_codir	0.19921507	0.06421648	0.14562063
ENSG0000025	RP11-661A12	lncRNA	0.19911683	0.79434622	0.8722555
ENSG0000019	YRDC	protein_codir	0.1988246	0.28117956	0.43054548

ENSG0000015	ATP6V1C1	protein_codir	0.19871243	0.07251704	0.15910085
ENSG0000023	MORF4L2-AS1	lncRNA	0.19861841	0.77284695	0.85709829
ENSG0000012	MED13L	protein_codir	0.19849619	0.28297499	0.43243163
ENSG0000011	CNOT6	protein_codir	0.19848926	0.16014715	0.28585138
ENSG0000015	UTRN	protein_codir	0.19838217	0.4242075	0.57623623
ENSG0000013	TUBGCP2	protein_codir	0.19830407	0.19607783	0.33192636
ENSG0000022	FAM225B	lncRNA	0.19825505	0.67123557	0.78318378
ENSG0000017	CLP1	protein_codir	0.19821914	0.31445827	0.46637693
ENSG0000013	KATNBL1	protein_codir	0.19821081	0.12165339	0.23346766
ENSG0000010	PNPO	protein_codir	0.19805317	0.22434117	0.3660387
ENSG0000000	STPG1	protein_codir	0.19804255	0.40004956	0.5533261
ENSG0000008	LPCAT2	protein_codir	0.19800148	0.50371002	0.64844888
ENSG0000010	APMAP	protein_codir	0.19796689	0.26571474	0.41321103
ENSG0000007	POLD3	protein_codir	0.19796463	0.14780607	0.2694478
ENSG0000014	ISG20L2	protein_codir	0.19770197	0.14119575	0.26059651
ENSG0000020	MIR1915HG	lncRNA	0.19767051	0.4409784	0.59200075
ENSG0000017	DENND6A	protein_codir	0.1976082	0.05039469	0.12127745
ENSG0000016	C15orf40	protein_codir	0.19755472	0.2395259	0.3838752
ENSG0000009	TEKT2	protein_codir	0.19752388	0.81651726	0.88718026
ENSG0000015	SLC38A10	protein_codir	0.19745912	0.0496014	0.11979632
ENSG0000013	USP15	protein_codir	0.19745674	0.19660711	0.33257523
ENSG0000022	RP11-79N23.1	lncRNA	0.19742649	0.77174832	0.85639568
ENSG0000028	RP4-808A1.2	lncRNA	0.19737516	0.91873823	NA
ENSG0000020	ABHD16A	protein_codir	0.19724093	0.05446669	0.12830785
ENSG0000028	RP1-317G22.4	lncRNA	0.19723302	0.88246158	NA
ENSG0000023	LINC00211	lncRNA	0.19722069	0.80544098	0.87940387
ENSG0000010	RP1-37E16.12	protein_codir	0.19709688	0.84061828	0.90472185
ENSG0000012	ATG4C	protein_codir	0.19701841	0.34297539	0.49612341
ENSG0000026	C17orf114	protein_codir	0.19689769	0.7533323	0.84305172
ENSG0000028	RP1-234M6.2	lncRNA	0.19686746	0.9452636	NA
ENSG0000008	PTPN4	protein_codir	0.19684693	0.25914018	0.4062381
ENSG0000027	RP11-687E1.2	lncRNA	0.19673373	0.86833356	0.92190248
ENSG0000013	SYT4	protein_codir	0.19665274	0.86501198	0.91958088
ENSG0000027	RP11-88E10.5	lncRNA	0.19649502	0.59553237	0.72397025
ENSG0000013	C6orf52	protein_codir	0.19646297	0.73510445	0.83008121
ENSG0000016	NUDT13	protein_codir	0.19638271	0.4850332	0.63239317
ENSG0000017	NIPAL4	protein_codir	0.19633131	0.65078314	0.76683576
ENSG0000013	CHST5	protein_codir	0.196241	0.76152448	0.84948732
ENSG0000019	TPK1	protein_codir	0.19617228	0.13643168	0.25382211
ENSG0000017	ZNF518A	protein_codir	0.19607327	0.22257602	0.36379998
ENSG0000019	CH17-340M24	lncRNA	0.19599877	0.45654604	0.60711612
ENSG0000010	SMG9	protein_codir	0.19597294	0.26972995	0.4175274
ENSG0000011	CPSF3	protein_codir	0.19590149	0.14990378	0.27217608
ENSG0000027	RP11-615I2.6	lncRNA	0.19586627	0.76337906	0.85066225
ENSG0000022	AC018647.3	lncRNA	0.19561069	0.66411499	0.7776546
ENSG0000013	GABARAPL1	protein_codir	0.1956098	0.50817737	0.65261817
ENSG0000024	RBM14-RBM4	protein_codir	0.19557638	0.282551	0.43207057

ENSG0000027	CTC-529I10.1	lncRNA	0.19556821	0.81612417	0.88686114
ENSG0000008	CCNT2	protein_codir	0.19550518	0.32134246	0.47351039
ENSG0000014	CLTRN	protein_codir	0.19527943	0.75014984	0.84069688
ENSG0000016	MELTF	protein_codir	0.19509515	0.5779325	0.7101386
ENSG0000001	NCAPD2	protein_codir	0.19498657	0.34230826	0.49559963
ENSG0000011	EXOC2	protein_codir	0.19473688	0.1298908	0.24497718
ENSG0000014	ZNF41	protein_codir	0.1946583	0.14943494	0.271682
ENSG0000014	SHPRH	protein_codir	0.1945965	0.17164783	0.30097213
ENSG0000013	TMEM19	protein_codir	0.19453418	0.27565711	0.4237574
ENSG0000011	AUP1	protein_codir	0.19450702	0.27553221	0.42365973
ENSG0000020	LINC01973	lncRNA	0.19446519	0.88457011	NA
ENSG0000016	ABHD18	protein_codir	0.19427486	0.23318087	0.37632673
ENSG0000016	ZNF83	protein_codir	0.19418714	0.50284623	0.64758886
ENSG0000006	MAPK6	protein_codir	0.19409728	0.33300598	0.48572969
ENSG0000010	LMF2	protein_codir	0.19406609	0.16194457	0.28822146
ENSG0000002	ERP44	protein_codir	0.1938995	0.01654799	0.05136033
ENSG0000004	PHF23	protein_codir	0.19388717	0.1095975	0.21611216
ENSG0000025	RP11-243M5.	lncRNA	0.19379847	0.89049619	0.93474124
ENSG0000028	XXbac-BPGBP	lncRNA	0.19373039	0.58492859	0.71569068
ENSG0000013	FBXO11	protein_codir	0.19372631	0.02018752	0.05972359
ENSG0000015	SCAF4	protein_codir	0.19364467	0.04180488	0.10509246
ENSG0000028	LINC01348	lncRNA	0.19364006	0.76547545	0.85227685
ENSG0000012	SSR1	protein_codir	0.19350749	0.21858332	0.35908352
ENSG0000012	PRKRIP1	protein_codir	0.19348953	0.36471732	0.51866036
ENSG0000010	OAZ1	protein_codir	0.19342155	0.09662369	0.19656378
ENSG0000005	RC3H2	protein_codir	0.19341958	0.20256484	0.33965766
ENSG0000010	TNRC6B	protein_codir	0.19334378	0.10004034	0.20179028
ENSG0000015	ZNF547	protein_codir	0.19328466	0.55548579	0.69138468
ENSG0000010	AMH	protein_codir	0.19328168	0.72336061	0.82154484
ENSG0000015	MAGED4	protein_codir	0.19300178	0.63211005	0.75256031
ENSG0000013	GALNT15	protein_codir	0.19297337	0.78221897	0.86391907
ENSG0000019	TOMM20L	protein_codir	0.19295299	0.86257154	0.91812345
ENSG0000024	SCAMP1-AS1	lncRNA	0.19281661	0.31540771	0.4672262
ENSG0000010	FSD1L	protein_codir	0.19255239	0.22634776	0.36835523
ENSG0000016	ING2	protein_codir	0.19237515	0.28719095	0.43672378
ENSG0000017	LMNB2	protein_codir	0.19219036	0.32705462	0.47926828
ENSG0000025	PRKDC	protein_codir	0.19213681	0.38811706	0.54115772
ENSG0000016	RRP1B	protein_codir	0.19199809	0.09052271	0.18699667
ENSG0000018	RGS6	protein_codir	0.1919136	0.67771357	0.78783334
ENSG0000027	DCP1A	protein_codir	0.19190714	0.12512267	0.23845223
ENSG0000011	ARID1A	protein_codir	0.19163741	0.13394391	0.25066269
ENSG0000016	LSM6	protein_codir	0.19155326	0.09956898	0.20113313
ENSG0000000	ABCB4	protein_codir	0.19152054	0.54918343	0.68663467
ENSG0000016	DCXR	protein_codir	0.19138952	0.22749964	0.36975051
ENSG0000012	ERGIC3	protein_codir	0.19137735	0.13894931	0.25746544
ENSG0000024	LINC02062	lncRNA	0.19129641	0.6811146	0.79034734
ENSG0000010	HNRNPL	protein_codir	0.19127746	0.06954304	0.15425465

ENSG0000014	RALB	protein_codir	0.19110166	0.16916703	0.29771844
ENSG0000014	PRIM2	protein_codir	0.19104062	0.13520563	0.2522207
ENSG0000026	RP11-738E22.	lncRNA	0.19099218	0.81455947	0.88596253
ENSG0000018	METTL23	protein_codir	0.19099072	0.0858343	0.17987431
ENSG0000027	RP11-362F19.	lncRNA	0.19098878	0.82709114	0.8953799
ENSG0000017	SERTAD2	protein_codir	0.19095488	0.22790227	0.37019026
ENSG0000010	COA1	protein_codir	0.19082886	0.2447016	0.38968279
ENSG0000017	BASP1	protein_codir	0.19065566	0.36679814	0.52068483
ENSG0000027	RP11-476H16	lncRNA	0.19064875	0.90902452	NA
ENSG0000010	NIPAL2	protein_codir	0.19059516	0.43451195	0.58594255
ENSG0000013	MRPL44	protein_codir	0.19054003	0.27553098	0.42365973
ENSG0000017	ZFAND4	protein_codir	0.19040715	0.30344161	0.4547508
ENSG0000011	SMG7	protein_codir	0.1902209	0.07426502	0.1618176
ENSG0000024	RP11-550I24.:	lncRNA	0.19015899	0.3369376	0.48992921
ENSG0000018	TMEM252	protein_codir	0.18993601	0.8870815	0.93309434
ENSG0000027	CTD-2555O16	lncRNA	0.18985137	0.48744715	0.63458193
ENSG0000010	DHRS12	protein_codir	0.18979272	0.18906375	0.32311725
ENSG0000026	CTD-2639E6.4	lncRNA	0.18974431	0.8459332	0.90829482
ENSG0000013	SOX3	protein_codir	0.18971118	0.85977955	0.91627547
ENSG0000026	KDM7A-DT	lncRNA	0.1896646	0.41998387	0.57244149
ENSG0000010	DNAJC2	protein_codir	0.18960506	0.30178398	0.453004
ENSG0000017	CNTROB	protein_codir	0.18932662	0.19110718	0.32566512
ENSG0000007	ZZEF1	protein_codir	0.18923596	0.12445297	0.23743808
ENSG0000024	AC012358.8	lncRNA	0.18923449	0.55248872	0.6891931
ENSG0000010	ARRDC2	protein_codir	0.18915062	0.33828148	0.49113407
ENSG0000013	CDK9	protein_codir	0.18912888	0.14089392	0.26016112
ENSG0000012	MCEE	protein_codir	0.18908483	0.21250358	0.35207124
ENSG0000026	RP11-981G7.1	lncRNA	0.18902875	0.66406265	0.77763972
ENSG0000023	RP13-152O15	lncRNA	0.18872741	0.89476978	0.9372906
ENSG0000017	CNP	protein_codir	0.18871618	0.03284386	0.08721732
ENSG0000022	LINC02470	lncRNA	0.18826311	0.89130574	0.93525813
ENSG0000007	RNF126	protein_codir	0.18819031	0.28199212	0.43140769
ENSG0000013	RFTN1	protein_codir	0.18803811	0.40457422	0.55754115
ENSG0000028	RP11-23I22.1	lncRNA	0.18796681	0.77724506	0.86045567
ENSG0000027	RP11-820I16.4	lncRNA	0.18780008	0.69254771	0.79827091
ENSG0000013	RABGGTB	protein_codir	0.18778377	0.31180567	0.46351305
ENSG0000027	RP11-47A8.5	lncRNA	0.18750908	0.31376505	0.46549877
ENSG0000015	TAGLN2	protein_codir	0.18749738	0.25660963	0.40323747
ENSG0000010	PPIL2	protein_codir	0.18748989	0.26440611	0.41187168
ENSG0000019	PCBP2	protein_codir	0.18745649	0.12353747	0.23616451
ENSG0000001	SLC25A39	protein_codir	0.1874011	0.17778518	0.30850067
ENSG0000014	DYNLT1	protein_codir	0.18723457	0.25533825	0.40203183
ENSG0000005	GATB	protein_codir	0.18722184	0.29298568	0.44312038
ENSG0000003	ASTE1	protein_codir	0.18714476	0.26845644	0.41625412
ENSG0000025	RP11-256L11.	lncRNA	0.18695126	0.90350198	0.94272956
ENSG0000012	MTIF3	protein_codir	0.18675284	0.09880436	0.19993966
ENSG0000014	TKFC	protein_codir	0.18665375	0.18152084	0.3135071

ENSG0000018	FBF1	protein_codir	0.18654301	0.39325322	0.5463059
ENSG0000017	C5orf34	protein_codir	0.18654061	0.31911543	0.47115883
ENSG0000014	DUSP11	protein_codir	0.18648154	0.07147844	0.15742242
ENSG0000016	CRYL1	protein_codir	0.18635199	0.15326793	0.27672389
ENSG0000007	SDHA	protein_codir	0.18623976	0.12424999	0.2371655
ENSG0000016	KRTCAP2	protein_codir	0.18590515	0.1821747	0.31438084
ENSG0000026	RP11-573G6.6	lncRNA	0.18588697	0.76249818	0.85002421
ENSG0000006	TRAM2	protein_codir	0.185667	0.26483185	0.41238158
ENSG0000001	ELOVL5	protein_codir	0.18550261	0.23908338	0.38336622
ENSG0000014	POLG	protein_codir	0.18539542	0.12931419	0.24416583
ENSG0000010	USF2	protein_codir	0.1853319	0.07062076	0.15608109
ENSG0000004	PARP3	protein_codir	0.18510994	0.03498512	0.09151331
ENSG0000024	CTD-2260A17	lncRNA	0.18510721	0.56712833	0.70097934
ENSG0000015	APPL1	protein_codir	0.1850125	0.19556102	0.33133329
ENSG0000018	DHRS4L2	protein_codir	0.18492387	0.31950427	0.47160688
ENSG0000027	RP11-81A1.6	lncRNA	0.18482049	0.49532295	0.6412684
ENSG0000011	LGALS8	protein_codir	0.18478386	0.13594769	0.25321163
ENSG0000027	RP11-227G15	lncRNA	0.18476693	0.88759925	0.93341903
ENSG0000018	SLC25A42	protein_codir	0.18461776	0.41979436	0.57229616
ENSG0000012	TRIM25	protein_codir	0.18455442	0.44889954	0.59974872
ENSG0000018	IBA57	protein_codir	0.1844804	0.24810095	0.3935984
ENSG0000017	JUNB	protein_codir	0.18443202	0.69582875	0.80072482
ENSG0000006	HDAC7	protein_codir	0.18442638	0.06005301	0.13808393
ENSG0000015	SON	protein_codir	0.18441129	0.0368359	0.09520049
ENSG0000016	PPP4R2	protein_codir	0.184387	0.07488172	0.16269928
ENSG0000014	RHBDD1	protein_codir	0.18432772	0.18611601	0.31936657
ENSG0000012	CCDC93	protein_codir	0.18431647	0.12824088	0.2426535
ENSG0000023	DHCR24-DT	lncRNA	0.18425673	0.9140223	NA
ENSG0000010	HSCB	protein_codir	0.18407199	0.21425153	0.35413688
ENSG0000014	MTA2	protein_codir	0.18393941	0.07942799	0.16974629
ENSG0000019	ZNF277	protein_codir	0.18355575	0.15536827	0.27950705
ENSG0000025	LNCOC1	lncRNA	0.18343307	0.66905136	0.78136146
ENSG0000017	MAN1B1	protein_codir	0.18341769	0.12671473	0.24066383
ENSG0000001	LYPLA2	protein_codir	0.18340377	0.29491268	0.44530332
ENSG0000018	SLC35E2B	protein_codir	0.18331308	0.23840114	0.3826499
ENSG0000018	ATP6V0A2	protein_codir	0.18321995	0.11171867	0.21919938
ENSG0000011	VIPR1	protein_codir	0.18316844	0.53704466	0.67708518
ENSG0000012	ARFGEF2	protein_codir	0.18300488	0.25474005	0.4012272
ENSG0000025	LINC02156	lncRNA	0.18287652	0.85887008	0.91587957
ENSG0000026	RP11-454K7.3	lncRNA	0.18282929	0.87345123	0.92497464
ENSG0000012	CASD1	protein_codir	0.18273637	0.05546496	0.13000188
ENSG0000018	DCUN1D3	protein_codir	0.18269697	0.59088303	0.72026017
ENSG0000016	TRA2A	protein_codir	0.182628	0.19988889	0.33652586
ENSG0000015	ZUP1	protein_codir	0.18249577	0.21498663	0.35503323
ENSG0000028	RP11-103P16.	lncRNA	0.18239451	0.79552652	0.87310032
ENSG0000018	ZFP92	protein_codir	0.18239331	0.58863353	0.7187033
ENSG0000013	ATG4D	protein_codir	0.18236821	0.37019249	0.52361943

ENSG0000010	LSM5	protein_codir	0.18222345	0.16470286	0.29202034
ENSG0000017	SP3	protein_codir	0.18221128	0.24136522	0.386039
ENSG0000010	PLEKHJ1	protein_codir	0.18221074	0.3709184	0.52430308
ENSG0000001	STARD3NL	protein_codir	0.18219944	0.21098434	0.35037202
ENSG0000007	MCM6	protein_codir	0.18201808	0.22311159	0.36451629
ENSG0000016	STX18	protein_codir	0.18185522	0.09061836	0.18715913
ENSG0000012	RPAP2	protein_codir	0.18167838	0.24036283	0.38479229
ENSG0000016	C18orf54	protein_codir	0.18163572	0.42799669	0.57975815
ENSG0000027	RP3-500L14.2	lncRNA	0.181572	0.87598081	0.92642786
ENSG0000013	SELENOS	protein_codir	0.18155988	0.31345896	0.4651696
ENSG0000010	ZMIZ1	protein_codir	0.18096088	0.31144303	0.46322328
ENSG0000017	CTDNEP1	protein_codir	0.18092738	0.12423663	0.23715638
ENSG0000006	U2AF2	protein_codir	0.18091704	0.07842956	0.16821125
ENSG0000020	MT1M	protein_codir	0.18087869	0.84060151	0.90472185
ENSG0000011	WARS2	protein_codir	0.18086266	0.28401215	0.4336327
ENSG0000019	CTBP1-DT	lncRNA	0.18085688	0.20516243	0.34303705
ENSG0000019	ZNF587	protein_codir	0.18084083	0.35889446	0.51254731
ENSG0000021	MAP10	protein_codir	0.18076694	0.60404111	0.73061596
ENSG0000022	AC007036.6	lncRNA	0.18050506	0.94885548	NA
ENSG0000007	EED	protein_codir	0.18046845	0.36523731	0.51905605
ENSG0000018	RGS9BP	protein_codir	0.18029459	0.77692767	0.86017326
ENSG0000011	AAK1	protein_codir	0.18024106	0.03955126	0.1006269
ENSG0000011	INO80D	protein_codir	0.17997999	0.12036565	0.23147885
ENSG0000013	WDR74	protein_codir	0.17967615	0.30590115	0.45746872
ENSG0000027	RP11-363E6.4	lncRNA	0.1796501	0.76414105	0.85113424
ENSG0000025	ZNF345	protein_codir	0.17955665	0.46044787	0.61095168
ENSG0000002	BCLAF1	protein_codir	0.17949615	0.03810039	0.09767335
ENSG0000001	ZC3H3	protein_codir	0.17945026	0.03641241	0.09434436
ENSG0000023	KANTR	protein_codir	0.1794062	0.52690932	0.66867063
ENSG0000000	RBM6	protein_codir	0.17929729	0.52421747	0.66624731
ENSG0000025	RP11-624L4.2	lncRNA	0.17927521	0.85198577	0.91191478
ENSG0000010	GSR	protein_codir	0.17926528	0.16071951	0.2866322
ENSG0000024	TMEM35B	protein_codir	0.17919546	0.02836174	0.07799973
ENSG0000015	DPYSL4	protein_codir	0.17912338	0.74576519	0.83765594
ENSG0000010	INTS9	protein_codir	0.17894512	0.27024002	0.41810033
ENSG0000015	FBRS	protein_codir	0.17893458	0.16692266	0.29497146
ENSG0000018	ST20	protein_codir	0.17892857	0.66200333	0.77598357
ENSG0000013	SAFB2	protein_codir	0.17887809	0.26612798	0.4137139
ENSG0000027	RP11-423G4.1	lncRNA	0.17874143	0.81265164	0.8847585
ENSG0000026	RP11-497H16	lncRNA	0.17872005	0.47012047	0.6191754
ENSG0000013	SLC5A6	protein_codir	0.17869271	0.39315756	0.54620048
ENSG0000014	FAU	protein_codir	0.17828014	0.31997578	0.47210288
ENSG0000005	KMT2C	protein_codir	0.17797627	0.19737767	0.33353143
ENSG0000008	TGDS	protein_codir	0.17781559	0.1514035	0.27414218
ENSG0000025	RP11-512M8.	protein_codir	0.17776066	0.93705803	0.96260434
ENSG0000028	RP11-280F2.3	lncRNA	0.17770373	0.84375083	0.90660953
ENSG0000017	TOP3A	protein_codir	0.1777037	0.03154075	0.08455357

ENSG0000013	ATP6V1G1	protein_codir	0.17752251	0.04145676	0.10436932
ENSG0000010	PATZ1	protein_codir	0.17742888	0.22563337	0.36745246
ENSG0000012	UXT	protein_codir	0.17730068	0.26484699	0.41238158
ENSG0000000	CIAPIN1	protein_codir	0.1772944	0.15075566	0.27329088
ENSG0000013	C1orf159	protein_codir	0.17713907	0.51846411	0.66143089
ENSG0000026	RP11-800A3.4	lncRNA	0.17712573	0.86560983	0.9201102
ENSG0000012	CBX3	protein_codir	0.17690633	0.01423808	0.04579133
ENSG0000017	LEMD3	protein_codir	0.17652192	0.2119116	0.3514681
ENSG0000017	EIF3K	protein_codir	0.17638327	0.14123028	0.26062539
ENSG0000015	PSMG3	protein_codir	0.17637742	0.34485313	0.49789376
ENSG0000025	RP11-385D13	protein_codir	0.17628994	0.846515	0.90855461
ENSG0000016	BATF2	protein_codir	0.1762315	0.60260048	0.72957635
ENSG0000028	RP11-496B10.	lncRNA	0.1761999	0.72850542	0.82542232
ENSG0000008	CPOX	protein_codir	0.17618613	0.13513573	0.25213841
ENSG0000019	TYW1	protein_codir	0.17616827	0.10891793	0.2151526
ENSG0000013	RFXAP	protein_codir	0.17616553	0.22712301	0.36931223
ENSG0000026	MYMX	protein_codir	0.17604245	0.77048363	0.85556576
ENSG0000016	LRWD1	protein_codir	0.17591593	0.28992352	0.43959638
ENSG0000028	NOTCH2NLB	protein_codir	0.1757437	0.65372076	0.76924707
ENSG0000010	JADE3	protein_codir	0.17571088	0.34507076	0.49797726
ENSG0000022	FALEC	lncRNA	0.17567468	0.77656244	0.85980336
ENSG0000017	VCPIP1	protein_codir	0.17551854	0.38322963	0.53661066
ENSG0000012	DMAC2L	protein_codir	0.17531454	0.20381818	0.34119997
ENSG0000014	WDR93	protein_codir	0.17493915	0.80870763	0.88171573
ENSG0000016	ZNF608	protein_codir	0.17484733	0.4642273	0.61413727
ENSG0000000	CREBBP	protein_codir	0.17466939	0.30837617	0.45997569
ENSG0000024	PPIL3	protein_codir	0.17466883	0.46937617	0.61863761
ENSG0000016	LMAN2	protein_codir	0.17463489	0.18014768	0.31166169
ENSG0000008	CD82	protein_codir	0.17459488	0.54844001	0.68597239
ENSG0000028	RP11-423H2.4	lncRNA	0.17458957	0.94025936	NA
ENSG0000009	PCSK5	protein_codir	0.17423648	0.69184271	0.79769132
ENSG0000016	SF1	protein_codir	0.17420223	0.14197398	0.26157836
ENSG0000018	ZADH2	protein_codir	0.1739335	0.37503623	0.52858388
ENSG0000011	ACYP1	protein_codir	0.17387239	0.49408331	0.6403238
ENSG0000027	AP000254.8	lncRNA	0.17384726	0.5902793	0.71993235
ENSG0000012	SLPI	protein_codir	0.17352131	0.75367456	0.84335338
ENSG0000027	RP11-84C13.2	lncRNA	0.17352052	0.87616963	0.9265248
ENSG0000011	DDX59	protein_codir	0.17347453	0.22934008	0.37184462
ENSG0000014	FAM83A	protein_codir	0.17337727	0.91854428	0.95180806
ENSG0000018	FAM227A	protein_codir	0.17333404	0.70419769	0.80748703
ENSG0000012	MAD2L1BP	protein_codir	0.17324975	0.32034941	0.47244067
ENSG0000012	CHD6	protein_codir	0.17319495	0.2426182	0.38752687
ENSG0000028	RP11-337N6.3	lncRNA	0.17310273	0.67790824	0.78797548
ENSG0000011	PRPF3	protein_codir	0.17304432	0.34346006	0.49664241
ENSG0000007	NUP37	protein_codir	0.17299257	0.29805587	0.4488715
ENSG0000028	RP11-342K6.5	lncRNA	0.1729913	0.84814963	0.90953148
ENSG0000014	AFG3L2	protein_codir	0.17293148	0.13634991	0.25369416

ENSG0000017	OR1L8	protein_codir	0.17281366	0.75236028	0.84235858
ENSG0000011	RAB35	protein_codir	0.17278086	0.06308822	0.1435808
ENSG0000016	WDR90	protein_codir	0.17249416	0.43922224	0.59027434
ENSG0000014	TBCD	protein_codir	0.17248356	0.09530021	0.19455923
ENSG0000024	RP11-306G20	lncRNA	0.17242202	0.91300829	0.94847729
ENSG0000021	ZNF134	protein_codir	0.17230285	0.30201808	0.45328149
ENSG0000009	MRPS33	protein_codir	0.17227907	0.19882622	0.33511809
ENSG0000007	CLNS1A	protein_codir	0.1720825	0.06037437	0.1386552
ENSG0000010	CCDC34	protein_codir	0.17207358	0.5565183	0.69238899
ENSG0000019	MT-ND3	protein_codir	0.17197952	0.57912097	0.71112441
ENSG0000005	CYFIP2	protein_codir	0.17190334	0.61799716	0.74168252
ENSG0000013	CCNG2	protein_codir	0.17176937	0.45931006	0.60976419
ENSG0000016	SYAP1	protein_codir	0.17174819	0.46594831	0.6155385
ENSG0000020	EFCAB2	protein_codir	0.17174761	0.47180307	0.62056292
ENSG0000013	CRB1	protein_codir	0.17166971	0.77152465	0.8563195
ENSG0000011	TRIM62	protein_codir	0.17166158	0.29514923	0.44555268
ENSG0000001	MKS1	protein_codir	0.171555	0.38549466	0.53866381
ENSG0000024	NDUFB2-AS1	lncRNA	0.17148412	0.64127986	0.7594295
ENSG0000012	KRI1	protein_codir	0.17146811	0.3951053	0.5481069
ENSG0000010	TMEM205	protein_codir	0.17145796	0.04024613	0.10200019
ENSG0000014	NOSIP	protein_codir	0.17143755	0.26489071	0.41241721
ENSG0000016	RBPMS2	protein_codir	0.17130551	0.67030031	0.78248921
ENSG0000013	COL2A1	protein_codir	0.17118268	0.84268193	0.90612798
ENSG0000027	RP11-218F10.	lncRNA	0.17106483	0.80270604	0.87773629
ENSG0000015	NUP205	protein_codir	0.17098526	0.22055017	0.36130295
ENSG0000000	LASP1	protein_codir	0.17087549	0.35052276	0.50408407
ENSG0000015	RAD17	protein_codir	0.17086282	0.17561463	0.30581371
ENSG0000015	SLC13A3	protein_codir	0.17083354	0.50624115	0.65073675
ENSG0000011	DUSP22	protein_codir	0.17075945	0.13179432	0.24778158
ENSG0000010	SF3A2	protein_codir	0.17058864	0.21969783	0.36046418
ENSG0000020	RP11-111F5.4	lncRNA	0.17056938	0.58966271	0.71952658
ENSG0000022	PGA3	protein_codir	0.17056232	0.8771048	0.92714723
ENSG0000001	GPRC5A	protein_codir	0.17043002	0.81848566	0.88851842
ENSG0000014	PPP4C	protein_codir	0.170351	0.22624434	0.36827374
ENSG0000013	DBNL	protein_codir	0.17033169	0.24432024	0.38935707
ENSG0000018	SP1	protein_codir	0.17032942	0.19560723	0.33137094
ENSG0000009	CWF19L1	protein_codir	0.17016061	0.22727855	0.36945639
ENSG0000026	SCGB1B2P	lncRNA	0.17000012	0.74678135	0.83825335
ENSG0000025	KB-1460A1.1	lncRNA	0.16997897	0.81295633	0.88502045
ENSG0000011	THG1L	protein_codir	0.16993887	0.23014324	0.37251267
ENSG0000014	ERVK3-1	protein_codir	0.16990742	0.29526012	0.44563304
ENSG0000019	C20orf204	protein_codir	0.16975846	0.64718511	0.76416225
ENSG0000026	AC005523.3	lncRNA	0.16961588	0.89624694	0.93828515
ENSG0000013	ABCB7	protein_codir	0.16955319	0.07956505	0.16996029
ENSG0000010	UBE2S	protein_codir	0.16945254	0.46292502	0.61298257
ENSG0000007	MAP4K4	protein_codir	0.16918228	0.16906313	0.29761146
ENSG0000028	RP13-511M20	lncRNA	0.16897263	0.6084956	0.73426748

ENSG0000010	ISM2	protein_codir	0.16890518	0.87280924	0.92452507
ENSG0000018	ZNF746	protein_codir	0.16888754	0.09043486	0.18688501
ENSG0000012	HSPH1	protein_codir	0.16882872	0.66148146	0.77566944
ENSG0000018	STING1	protein_codir	0.16882315	0.27818796	0.42682322
ENSG0000019	C1orf174	protein_codir	0.16881228	0.1130817	0.22096333
ENSG0000018	ZNF33A	protein_codir	0.16863575	0.14297701	0.26286527
ENSG0000024	CDRT1	protein_codir	0.16859608	0.74981695	0.84051097
ENSG0000025	RP11-307C19.	lncRNA	0.1685335	0.87046727	0.92310639
ENSG0000011	TCF21	protein_codir	0.16829982	0.77077668	0.85579884
ENSG0000026	RP4-761J14.1	lncRNA	0.16810701	0.67729128	0.78753785
ENSG0000010	PIGL	protein_codir	0.16805763	0.54942608	0.68668937
ENSG0000025	RP11-649E7.5	lncRNA	0.16802349	0.69401039	0.79935185
ENSG0000026	RP11-20G6.2	lncRNA	0.1679138	0.90366523	0.94281848
ENSG0000011	DDX6	protein_codir	0.16786322	0.28209086	0.43151027
ENSG0000019	CFD	protein_codir	0.16786282	0.76614275	0.85264175
ENSG0000014	MFHAS1	protein_codir	0.16773049	0.34224999	0.49554124
ENSG0000010	FAM118A	protein_codir	0.16751528	0.64712754	0.76416225
ENSG0000021	FASTKD5	protein_codir	0.16746712	0.2724377	0.42037593
ENSG0000023	RP13-143G15	lncRNA	0.16738879	0.8475971	0.90915075
ENSG0000017	ZBTB21	protein_codir	0.16730057	0.64635585	0.7635735
ENSG0000011	ATG2A	protein_codir	0.16729061	0.02915358	0.07975653
ENSG0000023	AC018816.3	lncRNA	0.16718837	0.73376531	0.82926604
ENSG0000012	RRBP1	protein_codir	0.16715528	0.36541478	0.51923906
ENSG0000013	BZW2	protein_codir	0.16699309	0.4505302	0.60125842
ENSG0000012	EAPP	protein_codir	0.16692376	0.18694256	0.32044644
ENSG0000028	RP11-185O17	lncRNA	0.16686226	0.92330409	NA
ENSG0000028	RP4-809E13.5	lncRNA	0.16680591	0.57199713	0.70519844
ENSG0000010	UBE2D3	protein_codir	0.16669018	0.12520174	0.23855353
ENSG0000015	POC5	protein_codir	0.16656236	0.45081914	0.60157477
ENSG0000012	AARS2	protein_codir	0.16652672	0.38324337	0.53661066
ENSG0000015	DYRK1A	protein_codir	0.1664015	0.09580771	0.19531831
ENSG0000011	DNAJC27	protein_codir	0.16639304	0.55612353	0.69196017
ENSG0000015	ZNF222	protein_codir	0.16618232	0.45166773	0.60225271
ENSG0000016	LRATD1	protein_codir	0.166025	0.48050573	0.62823992
ENSG0000028	CH17-408M7.	lncRNA	0.16602374	0.46115194	0.61139096
ENSG0000013	ORC3	protein_codir	0.16595985	0.24945609	0.39531684
ENSG0000010	HMOX2	protein_codir	0.16589241	0.15956904	0.28515119
ENSG0000009	MED15	protein_codir	0.1657578	0.25085298	0.39687016
ENSG0000023	TRIM26	protein_codir	0.1656823	0.08002951	0.17067524
ENSG0000014	HMG20A	protein_codir	0.16558378	0.25467567	0.40114868
ENSG0000017	BNC2	protein_codir	0.16547977	0.56092085	0.6958154
ENSG0000016	AP1G1	protein_codir	0.16525934	0.18695815	0.32045328
ENSG0000011	PODXL2	protein_codir	0.16508511	0.56432613	0.69861
ENSG0000026	CTC-297N7.1	lncRNA	0.16503751	0.88305505	0.93082114
ENSG0000025	RP11-313F23.	lncRNA	0.16501934	0.90767097	0.94496314
ENSG0000011	DOK1	protein_codir	0.16490085	0.45759071	0.60813693
ENSG0000007	ITCH	protein_codir	0.16480303	0.28590592	0.43546496

ENSG0000007	ADCYAP1R1	protein_codir	0.16477069	0.73610756	0.83089994
ENSG0000018	ZNF567	protein_codir	0.16475142	0.23101633	0.37367978
ENSG0000016	NUDT21	protein_codir	0.16467135	0.2327399	0.37579072
ENSG0000016	MAP2K1	protein_codir	0.16463819	0.28735674	0.43687967
ENSG0000024	SPCS3-AS1	lncRNA	0.16449163	0.70558939	0.80857063
ENSG0000028	RP3-436N22.5	protein_codir	0.16447693	0.88882224	0.9340189
ENSG0000025	LINC01301	lncRNA	0.16442638	0.66343775	0.77729229
ENSG0000019	FLVCR1-DT	lncRNA	0.16435491	0.70277022	0.80635233
ENSG0000010	PGK1	protein_codir	0.16424241	0.31157866	0.46332521
ENSG0000026	RP1-283E3.8	lncRNA	0.16423112	0.43949232	0.59046492
ENSG0000014	OSBPL10	protein_codir	0.16422452	0.37386715	0.52738564
ENSG0000026	ZSCAN5A-AS1	lncRNA	0.16417691	0.83135062	0.89837105
ENSG0000028	RP11-281A20	lncRNA	0.16405745	0.67292571	0.78430689
ENSG0000012	ABHD17A	protein_codir	0.1637447	0.27916796	0.4280348
ENSG0000027	RP11-90M2.5	lncRNA	0.16361759	0.88294766	0.93080207
ENSG0000009	GNPTG	protein_codir	0.1635841	0.12232458	0.23447879
ENSG0000019	MCMBP	protein_codir	0.16352489	0.25316212	0.39947084
ENSG0000010	ACIN1	protein_codir	0.16342009	0.26281791	0.41015076
ENSG0000023	RGS5	lncRNA	0.16301385	0.55012834	0.68731564
ENSG0000019	ZNF799	protein_codir	0.16277885	0.46628376	0.61591379
ENSG0000023	FAM133B	protein_codir	0.16265007	0.40558778	0.55855278
ENSG0000007	CAPZB	protein_codir	0.16257568	0.13343465	0.25002953
ENSG0000028	C2orf81	protein_codir	0.16240158	0.52264123	0.66494775
ENSG0000017	SPDYE5	protein_codir	0.16235674	0.93380029	NA
ENSG0000022	DHFR	protein_codir	0.16232587	0.48237596	0.62982056
ENSG0000019	PLEKHG4	protein_codir	0.16221364	0.47238	0.62108514
ENSG0000022	PTPRD-AS1	lncRNA	0.16218622	0.72239296	0.82088758
ENSG0000009	RNF31	protein_codir	0.16215435	0.19260221	0.32760641
ENSG0000016	MICU2	protein_codir	0.16212799	0.01828892	0.05534569
ENSG0000018	ZNF609	protein_codir	0.16206751	0.2370276	0.38108751
ENSG0000024	H2AZ1-DT	lncRNA	0.16196636	0.78959462	0.86876482
ENSG0000003	PIAS1	protein_codir	0.16196514	0.24988102	0.39578586
ENSG0000027	RP11-438J1.1	protein_codir	0.16190957	0.68262631	0.7913327
ENSG0000013	STX6	protein_codir	0.16186874	0.26282717	0.41015076
ENSG0000026	C1QTNF1-AS1	lncRNA	0.16158071	0.85716345	0.91475872
ENSG0000010	UBR5	protein_codir	0.16146025	0.24236274	0.38725006
ENSG0000016	SAFB	protein_codir	0.16137943	0.15207609	0.27507149
ENSG0000013	COLGALT1	protein_codir	0.16124124	0.38637706	0.53943964
ENSG0000027	RP11-392O17	lncRNA	0.1611221	0.78971822	0.8688316
ENSG0000027	MLLT6	protein_codir	0.16110673	0.1745373	0.30462686
ENSG0000013	NOL11	protein_codir	0.16104114	0.29425629	0.44455523
ENSG0000013	RTCA	protein_codir	0.16098365	0.11806226	0.22820908
ENSG0000018	LRRK2	protein_codir	0.16081576	0.46589751	0.61552135
ENSG0000023	TMA7	protein_codir	0.16079045	0.19914969	0.33558522
ENSG0000012	PAIP2	protein_codir	0.1607856	0.11282866	0.22068836
ENSG0000013	WDR33	protein_codir	0.1604171	0.07722705	0.16633815
ENSG0000016	DCAF16	protein_codir	0.16029325	0.15137296	0.27410485

ENSG0000024	GHRLOS	lncRNA	0.1602457	0.69696585	0.80171483
ENSG0000008	SLC4A4	protein_codir	0.16018417	0.65180462	0.76771188
ENSG0000019	SVIP	protein_codir	0.16017493	0.33849184	0.49131035
ENSG0000011	CEBPZ	protein_codir	0.16010683	0.32263336	0.4747726
ENSG0000026	PARD6G-AS1	lncRNA	0.15996006	0.64839178	0.76470306
ENSG0000014	MARCHF6	protein_codir	0.15990861	0.08724985	0.18203964
ENSG0000008	NOA1	protein_codir	0.15984351	0.16298373	0.28956714
ENSG0000026	GOLGA8S	protein_codir	0.15983429	0.72664595	0.82389002
ENSG0000008	WDFY1	protein_codir	0.15978305	0.36530454	0.51912488
ENSG0000014	PDIA6	protein_codir	0.15972485	0.41812455	0.57087113
ENSG0000011	GTF3C2	protein_codir	0.1597244	0.15108924	0.27371671
ENSG0000014	ZNF750	protein_codir	0.15970363	0.83580567	0.90133346
ENSG0000014	RANBP10	protein_codir	0.15960476	0.27540231	0.4235779
ENSG0000017	FAM156B	protein_codir	0.15959624	0.74605519	0.83779214
ENSG0000008	MTIF2	protein_codir	0.1595434	0.15175156	0.27459238
ENSG0000016	SELENON	protein_codir	0.15946083	0.40270511	0.55566344
ENSG0000014	RMC1	protein_codir	0.15942764	0.23653804	0.38049974
ENSG0000011	CPSF6	protein_codir	0.15936348	0.14221482	0.26182998
ENSG0000011	COPZ1	protein_codir	0.15929998	0.13567782	0.25277716
ENSG0000014	DISP2	protein_codir	0.1591964	0.79114123	0.87007286
ENSG0000010	RBM3	protein_codir	0.15916992	0.62194566	0.74480312
ENSG0000027	PAXIP1-DT	lncRNA	0.15916578	0.47757171	0.6255587
ENSG0000024	DNAJC25-GN(	protein_codir	0.15915721	0.46919528	0.61857898
ENSG0000017	MOB1B	protein_codir	0.15887751	0.31501708	0.4668297
ENSG0000027	CTD-2026K11	lncRNA	0.15884727	0.65860221	0.77321231
ENSG0000010	SC5D	protein_codir	0.15882935	0.4524677	0.60302827
ENSG0000014	CSNK1D	protein_codir	0.15879233	0.25885708	0.40597539
ENSG0000013	HNRNPA1	protein_codir	0.15869601	0.27979878	0.42881158
ENSG0000002	MIPEP	protein_codir	0.15861593	0.28253923	0.43207057
ENSG0000017	CCDC71	protein_codir	0.15856989	0.30823996	0.45994527
ENSG0000022	LINC02587	lncRNA	0.15846788	0.68693974	0.79469217
ENSG0000011	TRIM38	protein_codir	0.15846506	0.3896377	0.54259319
ENSG0000006	DDX20	protein_codir	0.15839475	0.24737523	0.39267264
ENSG0000020	MRPS18B	protein_codir	0.1583835	0.12877473	0.2435135
ENSG0000011	UBN1	protein_codir	0.15827176	0.11455927	0.22310866
ENSG0000026	RP11-158H5.7	lncRNA	0.15826469	0.6462468	0.76354265
ENSG0000015	SLC30A6	protein_codir	0.15822933	0.32841163	0.48073099
ENSG0000017	ATPAF2	protein_codir	0.15818167	0.39556569	0.5484701
ENSG0000018	LSM10	protein_codir	0.15812305	0.2685362	0.41629496
ENSG0000017	POLR1C	protein_codir	0.15810495	0.46517318	0.61491735
ENSG0000010	NSD2	protein_codir	0.1580736	0.28602013	0.43554283
ENSG0000023	LINC01779	lncRNA	0.15799637	0.89940044	NA
ENSG0000016	METTL3	protein_codir	0.15794796	0.5092014	0.65347749
ENSG0000016	ZNF610	protein_codir	0.1578579	0.62434344	0.74667096
ENSG0000025	RP11-91P24.7	lncRNA	0.15782578	0.90811774	NA
ENSG0000018	ZNF529	protein_codir	0.15782355	0.25545783	0.40214685
ENSG0000022	LINC00115	lncRNA	0.15779845	0.68892205	0.79581878

ENSG0000017	PLEKHM3	protein_codir	0.15760136	0.37910812	0.53252129
ENSG0000013	TMEM60	protein_codir	0.15759889	0.33507647	0.48795859
ENSG0000011	CCKBR	protein_codir	0.15757229	0.90258166	0.94215725
ENSG0000013	RTN4IP1	protein_codir	0.15753175	0.42700736	0.57878703
ENSG0000008	CAD	protein_codir	0.15750705	0.34551832	0.49844438
ENSG0000010	ASCC2	protein_codir	0.1574958	0.08831696	0.18365565
ENSG0000018	SDR42E2	protein_codir	0.1574155	0.89037726	0.93470999
ENSG0000025	LINC02308	lncRNA	0.1572332	0.87674479	0.92691641
ENSG0000017	GTPBP2	protein_codir	0.15711419	0.37878027	0.53225026
ENSG0000021	LINC00598	lncRNA	0.15706164	0.70602027	0.8088622
ENSG0000028	RP11-949J7.8	protein_codir	0.1569686	0.74236958	0.83552202
ENSG0000006	PIGV	protein_codir	0.15686781	0.26228607	0.40975741
ENSG0000014	SPIN2A	protein_codir	0.15673213	0.69264112	0.79834527
ENSG0000025	RP11-90B9.2	lncRNA	0.15670501	0.88424991	0.93128621
ENSG0000010	TNKS2	protein_codir	0.15661069	0.25811293	0.40510735
ENSG0000006	ADCK1	protein_codir	0.15648423	0.48413532	0.63149079
ENSG0000013	ETV6	protein_codir	0.1564327	0.37509504	0.52863175
ENSG0000025	MGAM	protein_codir	0.15630988	0.78720684	0.86717825
ENSG0000026	BOLA2-SMG1	protein_codir	0.15627238	0.45591728	0.60660125
ENSG0000014	WDTC1	protein_codir	0.15622321	0.17972775	0.31109109
ENSG0000027	CTB-186H2.3	lncRNA	0.1561681	0.89025759	0.93462849
ENSG0000011	AFTPH	protein_codir	0.156154	0.23768153	0.3817611
ENSG0000014	REXO4	protein_codir	0.15604107	0.1291853	0.24404733
ENSG0000016	RPP14	protein_codir	0.15603124	0.15503394	0.27908736
ENSG0000019	KIAA0753	protein_codir	0.15598181	0.38927369	0.54227767
ENSG0000003	SLC4A7	protein_codir	0.15598026	0.31441474	0.46633741
ENSG0000013	ZSCAN10	protein_codir	0.1559239	0.94679989	NA
ENSG0000018	ZNF559	protein_codir	0.15591113	0.23735905	0.38144277
ENSG0000023	LINC02574	lncRNA	0.15587553	0.79274361	0.87106465
ENSG0000024	RP11-480D4.2	lncRNA	0.15587086	0.83875407	0.90326662
ENSG0000023	AC003075.4	lncRNA	0.15577616	0.59182708	0.72092546
ENSG0000010	MIER2	protein_codir	0.15576983	0.31270236	0.46441196
ENSG0000012	CHRNA10	protein_codir	0.15568623	0.69856749	0.80303135
ENSG0000008	PIBF1	protein_codir	0.15559412	0.19164529	0.32628015
ENSG0000017	SNTB1	protein_codir	0.15548923	0.47685698	0.62497817
ENSG0000027	RP5-1007H16	lncRNA	0.155439	0.89778502	0.93923799
ENSG0000023	XXbac-B476C2	lncRNA	0.15538869	0.8714164	0.92365991
ENSG0000019	SLC22A4	protein_codir	0.15538327	0.67336324	0.78467407
ENSG0000027	RP11-161M6.	lncRNA	0.15530128	0.80125437	0.87666954
ENSG0000010	GSS	protein_codir	0.15523973	0.30842924	0.46002893
ENSG0000018	CENATAC	protein_codir	0.15513874	0.65707656	0.77210237
ENSG0000017	KCTD13	protein_codir	0.15500356	0.40834818	0.56131719
ENSG0000015	PAXIP1	protein_codir	0.15491927	0.1557832	0.28005285
ENSG0000017	ZBTB34	protein_codir	0.15487637	0.37736773	0.53102184
ENSG0000014	CPNE2	protein_codir	0.15483293	0.39777505	0.55078693
ENSG0000023	FAM215B	lncRNA	0.15471922	0.7562853	0.84551357
ENSG0000018	VWC2	protein_codir	0.15460798	0.85270224	0.91235989

ENSG0000017	WASHC2C	protein_codir	0.15455049	0.27277327	0.42070571
ENSG0000000	NFYA	protein_codir	0.15454927	0.16817464	0.29657666
ENSG0000028	RP1-102G20.8	lncRNA	0.15451068	0.78790254	0.86752514
ENSG0000006	IP6K2	protein_codir	0.15443939	0.38783682	0.54093082
ENSG0000023	DNAJC9-AS1	lncRNA	0.15432002	0.88384386	0.93115014
ENSG0000016	PYDC1	protein_codir	0.1542935	0.90909732	NA
ENSG0000018	AC092171.2	lncRNA	0.15425487	0.92779493	NA
ENSG0000006	GRIPAP1	protein_codir	0.15422865	0.36082312	0.5146896
ENSG0000014	SKP2	protein_codir	0.15420761	0.58610808	0.71651862
ENSG0000017	RNPEP	protein_codir	0.15393354	0.34952218	0.50300453
ENSG0000023	RP11-34P13.1	lncRNA	0.15379563	0.92963779	0.95826071
ENSG0000016	INTS8	protein_codir	0.15374993	0.30160771	0.45278862
ENSG0000025	RP11-849H4.2	protein_codir	0.15372193	0.53855418	0.67803516
ENSG0000018	ZKSCAN4	protein_codir	0.15368881	0.56203028	0.6967672
ENSG0000010	EDRF1	protein_codir	0.15365541	0.39896672	0.55219325
ENSG0000026	RP11-96O20.4	protein_codir	0.15358716	0.93676903	0.96241484
ENSG0000018	NUTM2A	protein_codir	0.15339679	0.68059239	0.79006269
ENSG0000018	CHRM2	protein_codir	0.15327574	0.92530835	0.95583657
ENSG0000023	FBXO30-DT	lncRNA	0.15326358	0.44693335	0.59781583
ENSG0000020	PSENEN	protein_codir	0.15316537	0.20903775	0.3479763
ENSG0000012	SYMPK	protein_codir	0.15312259	0.36777347	0.52134589
ENSG0000011	USP4	protein_codir	0.15309132	0.19781459	0.33401663
ENSG0000015	FCSK	protein_codir	0.15294702	0.43181523	0.58301494
ENSG0000014	NSD3	protein_codir	0.1529187	0.18456622	0.3175951
ENSG0000015	NAT2	protein_codir	0.15284593	0.87868142	0.92793308
ENSG0000010	ERI1	protein_codir	0.15283564	0.18686878	0.32035973
ENSG0000010	CRTC1	protein_codir	0.15272636	0.25025246	0.39619242
ENSG0000027	RP11-138H8.8	lncRNA	0.15260429	0.83308283	0.89942359
ENSG0000023	AC009312.1	lncRNA	0.15260096	0.9060462	0.94419621
ENSG0000013	ST6GALNAC4	protein_codir	0.15259497	0.38355727	0.5369413
ENSG0000023	AF129075.5	lncRNA	0.15251247	0.85414538	0.91289133
ENSG0000010	GALK1	protein_codir	0.15250977	0.38024873	0.53360786
ENSG0000000	GCFC2	protein_codir	0.15249272	0.32057274	0.47262833
ENSG0000016	SLBP	protein_codir	0.15245072	0.12023763	0.23132929
ENSG0000015	EEF1A1	protein_codir	0.15240566	0.24673738	0.39199171
ENSG0000010	TFAM	protein_codir	0.1520463	0.27162254	0.41962463
ENSG0000027	AC142391.3	protein_codir	0.15201316	0.93032506	0.95871868
ENSG0000010	PPM1F	protein_codir	0.15187023	0.21073936	0.35007043
ENSG0000010	UBE2R2	protein_codir	0.15171515	0.25349208	0.39983161
ENSG0000017	BANP	protein_codir	0.15169422	0.35719563	0.51073149
ENSG0000017	TMEM187	protein_codir	0.15162657	0.48729243	0.6344636
ENSG0000019	H2AC7	protein_codir	0.15130474	0.83399414	0.90001243
ENSG0000006	CCAR1	protein_codir	0.15088179	0.33704133	0.48994253
ENSG0000016	MIS12	protein_codir	0.15078097	0.17985941	0.31124096
ENSG0000011	HEATR1	protein_codir	0.15063956	0.42035541	0.57259756
ENSG0000006	EPN1	protein_codir	0.15053143	0.17830077	0.3092607
ENSG0000016	KIF27	protein_codir	0.15039634	0.34480847	0.49789376

ENSG0000015	ATAD2	protein_codir	0.15037442	0.38728029	0.54029101
ENSG0000015	SASS6	protein_codir	0.15030708	0.46708354	0.61664583
ENSG0000011	TPSG1	protein_codir	0.15027697	0.86466511	0.91935786
ENSG0000015	MYO1E	protein_codir	0.15015819	0.51637937	0.65953286
ENSG0000025	EID3	protein_codir	0.15010007	0.50070387	0.64585947
ENSG0000010	CMC2	protein_codir	0.14981455	0.36215223	0.51602957
ENSG0000022	RP4-756H11.3	lncRNA	0.14977851	0.70032981	0.80458838
ENSG0000023	RP11-324O2.3	lncRNA	0.14976087	0.78584629	0.86619342
ENSG0000008	PGS1	protein_codir	0.1497581	0.25953563	0.40660092
ENSG0000012	PTPN12	protein_codir	0.149732	0.24767856	0.39304686
ENSG0000025	RP11-377D9.3	lncRNA	0.14946077	0.87149832	0.92365991
ENSG0000010	SH2D4A	protein_codir	0.14935459	0.6149402	0.73897704
ENSG0000018	SLX4	protein_codir	0.14925047	0.45784391	0.60828557
ENSG0000010	TMEM33	protein_codir	0.14918017	0.26820065	0.41609291
ENSG0000008	SSH1	protein_codir	0.14900479	0.36260337	0.51645551
ENSG0000014	CEP78	protein_codir	0.14899353	0.129275	0.24413211
ENSG0000017	LVRN	protein_codir	0.14883262	0.69282868	0.79836869
ENSG0000013	RIPK1	protein_codir	0.14878445	0.10441444	0.20836115
ENSG0000013	TFCP2	protein_codir	0.14877099	0.2187864	0.35926757
ENSG0000012	WDR55	protein_codir	0.14875676	0.26665901	0.41425963
ENSG0000017	DPM3	protein_codir	0.14860727	0.30350437	0.45479551
ENSG0000007	CPSF1	protein_codir	0.1485685	0.47971724	0.62756549
ENSG0000010	PNN	protein_codir	0.14855752	0.37851453	0.53204827
ENSG0000026	RP11-686D22	lncRNA	0.14821086	0.64131293	0.75943506
ENSG0000023	LINC02802	lncRNA	0.14800547	0.65465877	0.77008839
ENSG0000011	HMGCR	protein_codir	0.14795155	0.49403002	0.64028479
ENSG0000028	RP11-501E24.	protein_codir	0.14791572	0.84358285	0.9064643
ENSG0000012	RAP2A	protein_codir	0.14784979	0.50231948	0.64705084
ENSG0000011	NEK4	protein_codir	0.14777105	0.18210192	0.31430344
ENSG0000014	TARS2	protein_codir	0.14770678	0.4195145	0.5720148
ENSG0000028	C2orf27A	lncRNA	0.14744599	0.63809736	0.7573807
ENSG0000016	SLC35G2	protein_codir	0.14740064	0.58327705	0.71435403
ENSG0000010	SGSM3	protein_codir	0.14736449	0.51533743	0.65856846
ENSG0000010	RAPGEF2	protein_codir	0.14731258	0.47926403	0.62703199
ENSG0000014	SLC25A37	protein_codir	0.14724842	0.47684064	0.62497817
ENSG0000025	RP11-286N22	protein_codir	0.14713933	0.58508188	0.71577098
ENSG0000016	ABRAXAS2	protein_codir	0.14709943	0.19629116	0.33216284
ENSG0000027	HCG17	lncRNA	0.14709714	0.83310923	0.89942359
ENSG0000010	DHX15	protein_codir	0.14687474	0.14519454	0.26614502
ENSG0000016	EXO5	protein_codir	0.14671995	0.38509186	0.53835285
ENSG0000005	TRAF1	protein_codir	0.14650271	0.61360007	0.73800879
ENSG0000016	NUDT5	protein_codir	0.14647729	0.37403211	0.52756443
ENSG0000012	UFM1	protein_codir	0.14641148	0.22692816	0.36910404
ENSG0000018	MED14	protein_codir	0.14631604	0.10697877	0.21222104
ENSG0000021	ADAT3	protein_codir	0.14626374	0.56838493	0.70212406
ENSG0000022	SCAMP4	protein_codir	0.14621413	0.14286792	0.2627892
ENSG0000016	ANTXR2	protein_codir	0.146205	0.64780988	0.76447378

ENSG0000018	YBEY	protein_codir	0.14590703	0.43764275	0.58889877
ENSG0000025	RP11-540D14	protein_codir	0.1458121	0.8976801	0.9391638
ENSG0000020	TP53TG3D	protein_codir	0.1457786	0.8416323	0.9054251
ENSG0000028	RP5-1022I14.:	lncRNA	0.14570778	0.76565525	0.85230526
ENSG0000017	PYM1	protein_codir	0.14562327	0.29595233	0.44643383
ENSG0000007	RASAL2	protein_codir	0.14560124	0.53495547	0.67536654
ENSG0000017	SMARCC1	protein_codir	0.14541518	0.34848034	0.50176221
ENSG0000013	KCNK1	protein_codir	0.14535193	0.64731365	0.76418205
ENSG0000011	TSNAX	protein_codir	0.14525441	0.27667293	0.42496421
ENSG0000024	AC093620.5	lncRNA	0.14512398	0.85890985	0.91587957
ENSG0000023	AC093702.1	lncRNA	0.14493217	0.89995535	0.94019089
ENSG0000026	RP11-375I20.(	lncRNA	0.14486668	0.7631279	0.85058889
ENSG0000016	DIS3L	protein_codir	0.14478621	0.28697652	0.43649384
ENSG0000008	GPATCH2L	protein_codir	0.14477481	0.22054888	0.36130295
ENSG0000027	GLIDR	lncRNA	0.14474743	0.50063401	0.64585947
ENSG0000010	PRX	protein_codir	0.14472729	0.6121132	0.73708709
ENSG0000026	LINC02864	lncRNA	0.1446794	0.91905966	0.95209035
ENSG0000017	BNIP3	protein_codir	0.14461455	0.47813901	0.62603458
ENSG0000015	PDE3B	protein_codir	0.1445198	0.68008066	0.7896678
ENSG0000019	HMGN2	protein_codir	0.1444751	0.35542681	0.5090691
ENSG0000011	CTSD	protein_codir	0.14445567	0.61415368	0.73844272
ENSG0000027	RP11-709D24	lncRNA	0.14442602	0.81537087	0.88625176
ENSG0000018	RASA3	protein_codir	0.14442281	0.10293336	0.2062135
ENSG0000017	ZBTB8OS	protein_codir	0.14441108	0.38267565	0.53608987
ENSG0000020	ADD3-AS1	lncRNA	0.14433541	0.73856054	0.83278392
ENSG0000016	TRIM44	protein_codir	0.14429847	0.10526165	0.20965028
ENSG0000004	HEXB	protein_codir	0.14421718	0.38846322	0.54144904
ENSG0000026	AC006547.15	lncRNA	0.144023	0.92428179	0.95541403
ENSG0000016	TPCN2	protein_codir	0.14398131	0.27862743	0.42737211
ENSG0000028	RP11-279N21	lncRNA	0.14393961	0.9412298	NA
ENSG0000026	RP11-690I21.:	lncRNA	0.14393485	0.78492861	0.86548364
ENSG0000010	EIF3L	protein_codir	0.14391991	0.3030534	0.45434158
ENSG0000026	AC010504.2	lncRNA	0.14373647	0.83408973	0.90004525
ENSG0000018	CNOT10	protein_codir	0.14352645	0.12209496	0.23418492
ENSG0000016	JCAD	protein_codir	0.14351387	0.68209238	0.79090644
ENSG0000016	YWHAZ	protein_codir	0.14341945	0.3414786	0.49471191
ENSG0000019	ZSCAN16	protein_codir	0.14328556	0.50121479	0.6461091
ENSG0000020	TDRKH-AS1	lncRNA	0.1432652	0.74869411	0.83966237
ENSG0000016	ABHD6	protein_codir	0.14304986	0.52812307	0.66963959
ENSG0000021	ZBTB9	protein_codir	0.14277923	0.54525806	0.68324234
ENSG0000012	RAB9A	protein_codir	0.14267596	0.22032046	0.36116393
ENSG0000027	RP11-474G23	protein_codir	0.14263587	0.70739535	0.80994319
ENSG0000014	SAMD1	protein_codir	0.1423567	0.30633031	0.45793696
ENSG0000007	CSNK2A2	protein_codir	0.14220601	0.08607701	0.18028532
ENSG0000015	SYNJ1	protein_codir	0.14197684	0.50690768	0.65135102
ENSG0000026	RP11-68I18.1(	lncRNA	0.14190184	0.78031908	0.86268315
ENSG0000025	RP11-1080G1	lncRNA	0.14181395	0.81925692	0.88907611

ENSG0000016	HELQ	protein_codir	0.14178216	0.30762262	0.45931966
ENSG0000012	PHF20L1	protein_codir	0.1416912	0.08272637	0.17476689
ENSG0000013	GUCD1	protein_codir	0.14165055	0.25711903	0.40386871
ENSG0000013	HRH4	protein_codir	0.14164028	0.86942656	0.92261024
ENSG0000028	RP11-680A11	lncRNA	0.14159858	0.68807614	0.79530729
ENSG0000027	RP11-493E12.	lncRNA	0.14154899	0.92246824	0.95428941
ENSG0000021	CSNK1E	protein_codir	0.14148772	0.33222763	0.48498276
ENSG0000017	CEP83	protein_codir	0.14144538	0.28022659	0.42934811
ENSG0000027	RP11-380B4.3	lncRNA	0.1413317	0.75483766	0.844347
ENSG0000010	HOXA1	protein_codir	0.14129012	0.61247024	0.73736411
ENSG0000006	COASY	protein_codir	0.14128788	0.30853302	0.46010918
ENSG0000014	ADPRH	protein_codir	0.14124667	0.37474466	0.52829969
ENSG0000011	C2CD5	protein_codir	0.14113854	0.27439593	0.42247704
ENSG0000027	PRH1-PRR4	protein_codir	0.14110502	0.62525283	0.74733786
ENSG0000026	RP11-459F6.3	lncRNA	0.14106211	0.75794753	0.84673049
ENSG0000019	LAMB3	protein_codir	0.14084255	0.67979088	0.78949728
ENSG0000017	NHLH2	protein_codir	0.14076829	0.90195591	0.94171098
ENSG0000018	ZNF17	protein_codir	0.14069101	0.35684519	0.51050091
ENSG0000027	RP11-43F13.4	lncRNA	0.14056336	0.81036831	0.8830035
ENSG0000016	ZNF382	protein_codir	0.14036031	0.48847426	0.63543986
ENSG0000012	TUBGCP3	protein_codir	0.1402815	0.15797084	0.28299097
ENSG0000027	RP1-178F15.5	lncRNA	0.14017521	0.77162694	0.85639473
ENSG0000011	OGFOD2	protein_codir	0.14009276	0.52213445	0.66463976
ENSG0000013	FAM155B	protein_codir	0.13995466	0.81837509	0.8884333
ENSG0000013	COX16	protein_codir	0.13985489	0.14667945	0.26815505
ENSG0000018	TRIM52	protein_codir	0.13954812	0.4830076	0.63046644
ENSG0000022	LINC02249	lncRNA	0.13947813	0.9317494	0.95966668
ENSG0000008	COL16A1	protein_codir	0.13947677	0.62705727	0.74858485
ENSG0000026	AC007292.4	lncRNA	0.13939864	0.94327398	0.96689145
ENSG0000018	SLC52A2	protein_codir	0.1393878	0.43549717	0.58681002
ENSG0000026	CTD-3105H18	lncRNA	0.1393764	0.80438186	0.87873381
ENSG0000026	RAB11B-AS1	lncRNA	0.13921002	0.3960947	0.54895557
ENSG0000011	ORC2	protein_codir	0.13908615	0.28144926	0.43082104
ENSG0000010	FBXO7	protein_codir	0.13881078	0.2351108	0.37855661
ENSG0000025	ZBED6	protein_codir	0.13868145	0.59112384	0.72041832
ENSG0000026	ZNF497-AS1	lncRNA	0.13861949	0.74835705	0.83965258
ENSG0000026	LINC01224	lncRNA	0.13845339	0.92044852	0.95300925
ENSG0000013	PRPF4	protein_codir	0.13820028	0.21438024	0.35428601
ENSG0000027	AP000692.10	lncRNA	0.13810526	0.75506863	0.84457116
ENSG0000010	KNOP1	protein_codir	0.13801043	0.44934611	0.60017117
ENSG0000026	MAFTRR	lncRNA	0.13800697	0.80034267	0.87620269
ENSG0000018	PTP4A2	protein_codir	0.13795612	0.27500053	0.42310131
ENSG0000012	TAF12	protein_codir	0.13779476	0.16908701	0.29761555
ENSG0000016	PIGO	protein_codir	0.13776737	0.20454194	0.34220416
ENSG0000016	TMEM79	protein_codir	0.13772596	0.63820944	0.75740533
ENSG0000017	GON7	protein_codir	0.13766633	0.40926906	0.56224876
ENSG0000010	MED31	protein_codir	0.1376515	0.28516594	0.43474046

ENSG0000013	CNTN6	protein_codir	0.13743431	0.87844218	0.92776278
ENSG0000000	SCYL3	protein_codir	0.13731986	0.2560064	0.4026876
ENSG0000017	PDZD3	protein_codir	0.13730385	0.86009858	0.91647401
ENSG0000014	SCD5	protein_codir	0.13718685	0.60865578	0.73436453
ENSG0000015	HNRNPU	protein_codir	0.13715816	0.18610597	0.31936657
ENSG0000016	SHROOM1	protein_codir	0.13694003	0.55017601	0.68731564
ENSG0000017	EXOSC10	protein_codir	0.13692493	0.08232494	0.17427875
ENSG0000012	MRPS2	protein_codir	0.1365998	0.2360539	0.37987582
ENSG0000018	CYB5D1	protein_codir	0.13655136	0.50560492	0.65019126
ENSG0000027	RP11-455J20.	lncRNA	0.13626921	0.87396165	0.92523044
ENSG0000017	BPTF	protein_codir	0.13622576	0.25414206	0.40055945
ENSG0000010	DRP2	protein_codir	0.13622165	0.82762892	0.89573167
ENSG0000007	TRIB2	protein_codir	0.1360768	0.51358936	0.65702682
ENSG0000017	ANAPC2	protein_codir	0.13605127	0.46027508	0.61081044
ENSG0000014	ESCO1	protein_codir	0.13584546	0.34187793	0.49506279
ENSG0000010	RAPGEFL1	protein_codir	0.13574967	0.70818523	0.81057795
ENSG0000010	CFAP69	protein_codir	0.13569345	0.71476553	0.81500326
ENSG0000028	RP11-27G22.2	lncRNA	0.13561126	0.92395065	0.9552505
ENSG0000019	STYX	protein_codir	0.13554176	0.36542248	0.51923906
ENSG0000013	ANKHD1	protein_codir	0.13537515	0.12003606	0.23105414
ENSG0000025	RP11-305E6.4	lncRNA	0.13520796	0.55005646	0.68725955
ENSG0000011	ATF6	protein_codir	0.13503347	0.40382978	0.55694677
ENSG0000018	DYNC2H1	protein_codir	0.13501647	0.55781847	0.693485
ENSG0000008	KIF9	protein_codir	0.13479039	0.56980544	0.70340691
ENSG0000022	CHROMR	lncRNA	0.13471328	0.53779894	0.67747461
ENSG0000027	LINC01138	lncRNA	0.13463097	0.60531522	0.73180454
ENSG0000014	TMEM91	protein_codir	0.13460754	0.61149435	0.73658271
ENSG0000007	STX7	protein_codir	0.13454203	0.29451513	0.4447992
ENSG0000016	MRNIP	protein_codir	0.13449058	0.66814831	0.78073598
ENSG0000008	NCOA1	protein_codir	0.13443838	0.40381317	0.55694677
ENSG0000016	SIK3	protein_codir	0.13443838	0.30968364	0.46139764
ENSG0000013	MDC1	protein_codir	0.13439148	0.24719771	0.39243596
ENSG0000015	CACUL1	protein_codir	0.13435632	0.15482619	0.27882241
ENSG0000028	LA16c-314G4.	lncRNA	0.13433049	0.94515908	NA
ENSG0000006	SMARCD1	protein_codir	0.13431056	0.21257904	0.35215217
ENSG0000014	GASK1A	protein_codir	0.13428933	0.73036146	0.82650811
ENSG0000015	GPATCH11	protein_codir	0.13427673	0.43665333	0.58790899
ENSG0000013	NDUFA10	protein_codir	0.13426013	0.16543264	0.29284429
ENSG0000026	STRADA	protein_codir	0.13413407	0.45379063	0.60423743
ENSG0000019	XPNPEP3	protein_codir	0.13403526	0.4061487	0.55915796
ENSG0000025	CENATAC-DT	lncRNA	0.13384864	0.71613266	0.81625883
ENSG0000023	NDUFA6-DT	lncRNA	0.13384753	0.43566401	0.58694439
ENSG0000009	PAPOLA	protein_codir	0.1338382	0.17275265	0.30235271
ENSG0000021	SCAF8	protein_codir	0.13372953	0.26939079	0.41723623
ENSG0000004	AP2S1	protein_codir	0.1335739	0.50333507	0.64805681
ENSG0000026	RBM8A	protein_codir	0.13350131	0.13074478	0.24621059
ENSG0000016	SMAD5-AS1	lncRNA	0.13347153	0.82374938	0.89283147

ENSG0000014	DUS3L	protein_codir	0.1334399	0.44738512	0.59821733
ENSG0000013	IDH1	protein_codir	0.13342166	0.57371369	0.70649481
ENSG0000015	PAFAH2	protein_codir	0.13330749	0.4095417	0.56256735
ENSG0000002	AKAP11	protein_codir	0.13324087	0.51102503	0.65486535
ENSG0000016	GBP2	protein_codir	0.13323556	0.48821708	0.63531476
ENSG0000014	CAPN10	protein_codir	0.13317169	0.49717447	0.64290616
ENSG0000020	SNHG32	lncRNA	0.13306038	0.38650168	0.5395318
ENSG0000014	DNAJB12	protein_codir	0.13301426	0.16478864	0.29205931
ENSG0000018	PPA1	protein_codir	0.13295291	0.37180661	0.52522261
ENSG0000014	ATP1B1	protein_codir	0.13294966	0.74238076	0.83552202
ENSG0000013	ARHGAP29	protein_codir	0.13293899	0.60315376	0.73001562
ENSG0000027	POM121C	protein_codir	0.13292831	0.30050934	0.45153237
ENSG0000012	RPAIN	protein_codir	0.13284069	0.51536562	0.65857307
ENSG0000014	NOM1	protein_codir	0.13266784	0.20842713	0.34714813
ENSG0000016	APEX2	protein_codir	0.13260464	0.32815942	0.48058129
ENSG0000011	BCAS2	protein_codir	0.13250329	0.41388334	0.56700899
ENSG0000010	CPEB3	protein_codir	0.13242153	0.38006082	0.53347968
ENSG0000027	RP1-170O19.2	lncRNA	0.13241912	0.94396823	0.96722075
ENSG0000006	ADAT1	protein_codir	0.13224242	0.43088112	0.58232307
ENSG0000022	TARID	lncRNA	0.13213862	0.86047334	0.91680259
ENSG0000014	RABL2A	protein_codir	0.13212161	0.48684347	0.634065
ENSG0000026	CTD-2278I10.4	lncRNA	0.13207162	0.86431369	0.91912155
ENSG0000020	TMEM256	protein_codir	0.13203448	0.43821617	0.58935148
ENSG0000020	SMN2	protein_codir	0.1319716	0.77389884	0.85778265
ENSG0000021	UMAD1	protein_codir	0.13189658	0.32499748	0.47731737
ENSG0000023	NPIPA9	protein_codir	0.13171772	0.61635502	0.74016198
ENSG0000008	ATXN7L3	protein_codir	0.13159962	0.21261764	0.35219498
ENSG0000009	MFSD11	protein_codir	0.13130446	0.21472298	0.35472509
ENSG0000014	KATNB1	protein_codir	0.13120658	0.31324443	0.46499203
ENSG0000019	QRICH1	protein_codir	0.13118818	0.10716921	0.21244078
ENSG0000028	LL0XNC01-235	lncRNA	0.13115182	0.8044317	0.8787535
ENSG0000014	CSGALNACT1	protein_codir	0.13114285	0.78311072	0.86431641
ENSG0000014	BDP1	protein_codir	0.13110641	0.46381931	0.61371512
ENSG0000025	RP11-315O6.1	lncRNA	0.13108127	0.83634083	0.90162895
ENSG0000015	NTAN1	protein_codir	0.13081826	0.26139055	0.40874183
ENSG0000016	ATP5MG	protein_codir	0.13078525	0.29413314	0.44446476
ENSG0000015	AASDH	protein_codir	0.13077202	0.41015406	0.56315645
ENSG0000014	ITGB3BP	protein_codir	0.1306624	0.57420617	0.70688066
ENSG0000027	RP11-467L19.	lncRNA	0.13058422	0.92525392	0.95583657
ENSG0000022	C6orf226	protein_codir	0.13057697	0.39020777	0.54327747
ENSG0000018	MED12	protein_codir	0.1305704	0.09829049	0.19917706
ENSG0000022	MUC12-AS1	lncRNA	0.13038982	0.83440011	0.90027466
ENSG0000015	PABIR2	protein_codir	0.13022709	0.28183476	0.43124787
ENSG0000022	LENG8-AS1	lncRNA	0.13016304	0.72545719	0.82310579
ENSG0000017	USP38	protein_codir	0.13015372	0.42945491	0.58102104
ENSG0000014	SH3BGRL3	protein_codir	0.1300589	0.58352736	0.71450218
ENSG0000026	RNFT1-DT	lncRNA	0.13003033	0.89756971	0.93912599

ENSG0000008	WBP11	protein_codir	0.13001705	0.41726874	0.56995018
ENSG0000026	CTC-471J1.8	lncRNA	0.12996887	0.85561045	0.91363027
ENSG0000027	RP11-4B16.3	lncRNA	0.12980529	0.86587468	0.92029113
ENSG0000011	DENND1A	protein_codir	0.12973383	0.21600628	0.35624849
ENSG0000017	NIPA1	protein_codir	0.12971031	0.38611159	0.53918817
ENSG0000028	RP4-714D9.5	protein_codir	0.12965586	0.69810884	0.80257091
ENSG0000017	HINFP	protein_codir	0.12961153	0.48086836	0.62850578
ENSG0000013	PNISR	protein_codir	0.12951108	0.61845904	0.7421401
ENSG0000010	MEST	protein_codir	0.12944023	0.77789766	0.86088527
ENSG0000017	EIF3F	protein_codir	0.12939805	0.30995597	0.46173259
ENSG0000008	RBM22	protein_codir	0.12937292	0.05056327	0.12155697
ENSG0000008	REST	protein_codir	0.12903097	0.36177818	0.51572882
ENSG0000016	UBTD2	protein_codir	0.12893857	0.46264059	0.61268379
ENSG0000000	ARSD	protein_codir	0.12878294	0.27652163	0.42475543
ENSG0000022	HNRNPA1P48	protein_codir	0.12857658	0.53376149	0.67442568
ENSG0000010	NUP93	protein_codir	0.12853461	0.18976891	0.32412451
ENSG0000028	GS1-590J6.3	lncRNA	0.12851485	0.92485582	NA
ENSG0000011	SET	protein_codir	0.12848	0.2410451	0.38563866
ENSG0000014	MYO10	protein_codir	0.1284715	0.67107283	0.78310369
ENSG0000011	TNNC1	protein_codir	0.12839903	0.78420326	0.86505563
ENSG0000017	BRMS1	protein_codir	0.12839099	0.40534344	0.55824412
ENSG0000024	RP5-1029K10.	lncRNA	0.12825351	0.91325624	0.94866815
ENSG0000025	ZBED3-AS1	lncRNA	0.12815726	0.68886829	0.79581878
ENSG0000003	EIPR1	protein_codir	0.12802602	0.51227802	0.65591769
ENSG0000023	DSCR9	lncRNA	0.12796081	0.84594409	0.90829482
ENSG0000020	PHACTR4	protein_codir	0.12786273	0.13385144	0.25055757
ENSG0000025	GLI4	protein_codir	0.12779064	0.61201697	0.73700334
ENSG0000019	TMEM239	protein_codir	0.12769417	0.9686811	NA
ENSG0000012	ZNF644	protein_codir	0.1274732	0.39984348	0.55315186
ENSG0000008	FUS	protein_codir	0.12740731	0.4041083	0.55720989
ENSG0000010	HOXB6	protein_codir	0.12737888	0.62569023	0.74756951
ENSG0000028	RP11-434E6.5	lncRNA	0.12735939	0.87811591	0.92765582
ENSG0000013	NARS2	protein_codir	0.12734792	0.29172582	0.44160184
ENSG0000011	ARMC2	protein_codir	0.12733981	0.49940687	0.64485162
ENSG0000011	STK16	protein_codir	0.12729999	0.36992675	0.52328982
ENSG0000027	RP11-449H3.3	protein_codir	0.12711053	0.7503291	0.84074649
ENSG0000019	TRIM33	protein_codir	0.127013	0.30232876	0.45367382
ENSG0000028	RP11-941F15.	lncRNA	0.12689813	0.91003298	0.9463877
ENSG0000026	LINC01960	lncRNA	0.1268762	0.85736274	0.91475872
ENSG0000019	ZNF480	protein_codir	0.1268738	0.48333153	0.63077003
ENSG0000013	TRIM21	protein_codir	0.12680009	0.38612095	0.53918817
ENSG0000027	ZNF595	protein_codir	0.1267948	0.5658986	0.70011733
ENSG0000016	C22orf15	protein_codir	0.12662433	0.81685221	0.88736527
ENSG0000026	PAN3-AS1	lncRNA	0.12650438	0.663605	0.77730153
ENSG0000028	RP11-7O11.8	lncRNA	0.12648498	0.88015648	0.92895877
ENSG0000024	ARHGAP42-AS	lncRNA	0.12646789	0.92659174	NA
ENSG0000016	ENTR1	protein_codir	0.12631486	0.32172573	0.47387291

ENSG0000001	KDM5D	protein_codir	0.12629593	0.82028988	0.88991737
ENSG0000028	RP13-726E6.5	lncRNA	0.12625504	0.92551626	NA
ENSG0000016	C1orf189	protein_codir	0.12624208	0.89184731	0.93556444
ENSG0000012	RNF6	protein_codir	0.12616429	0.35268009	0.5062376
ENSG0000005	PIK3CB	protein_codir	0.12586699	0.34992956	0.50346691
ENSG0000013	TGS1	protein_codir	0.12573863	0.35607935	0.50966013
ENSG0000016	TRAPPC11	protein_codir	0.12560529	0.48113233	0.62870203
ENSG0000026	CTD-2373J6.1	lncRNA	0.12542124	0.86111495	0.91722872
ENSG0000024	RBM12	protein_codir	0.12541258	0.42098336	0.57301205
ENSG0000014	SAT2	protein_codir	0.12538879	0.45275309	0.60327785
ENSG0000016	EIF4E3	protein_codir	0.12538809	0.59968823	0.72726652
ENSG0000013	RGS22	protein_codir	0.12536963	0.66196589	0.77598357
ENSG0000026	RP11-441F2.2	lncRNA	0.12534488	0.88978272	0.93436996
ENSG0000005	EIF2AK2	protein_codir	0.12530342	0.52740091	0.66903106
ENSG0000016	HEXIM2	protein_codir	0.12522085	0.61144896	0.73658271
ENSG0000019	NCOA6	protein_codir	0.12506748	0.26752593	0.41532779
ENSG0000016	HIPK1	protein_codir	0.12483641	0.5411202	0.67993903
ENSG0000011	DARS1	protein_codir	0.12475677	0.0827469	0.17478353
ENSG0000016	CCDC186	protein_codir	0.12475467	0.47247942	0.6210976
ENSG0000011	SCAMP3	protein_codir	0.12472252	0.22196079	0.36305561
ENSG0000015	GTF2E1	protein_codir	0.12466114	0.54198054	0.68079254
ENSG0000022	AC007383.3	lncRNA	0.1245667	0.43173261	0.58296038
ENSG0000024	CTC-325J23.2	lncRNA	0.12445714	0.92882738	0.95767553
ENSG0000013	DYNC2LI1	protein_codir	0.12442233	0.47456255	0.62282998
ENSG0000015	NPAS3	protein_codir	0.12438201	0.63072672	0.75140924
ENSG0000010	GPI	protein_codir	0.12427578	0.42554248	0.57743092
ENSG0000027	CTA-292E10.8	lncRNA	0.12427224	0.85162487	0.9117271
ENSG0000027	RP11-731D1.4	lncRNA	0.12427182	0.94934177	NA
ENSG0000026	GTF2I	protein_codir	0.12420895	0.28723515	0.43676695
ENSG0000016	MAP3K2	protein_codir	0.12415679	0.49355039	0.64002355
ENSG0000011	CRBN	protein_codir	0.12394123	0.38655121	0.5395439
ENSG0000003	TBC1D23	protein_codir	0.12392808	0.18606287	0.31931671
ENSG0000023	KIAA1614-AS1	lncRNA	0.12379481	0.70979841	0.81148359
ENSG0000016	PGGT1B	protein_codir	0.12374679	0.24059705	0.38507797
ENSG0000026	RP11-264L1.3	lncRNA	0.12367051	0.88900267	0.9340189
ENSG0000014	CCN1	protein_codir	0.12365089	0.75028951	0.84074649
ENSG0000019	HOXC4	protein_codir	0.12362196	0.80254926	0.87773629
ENSG0000011	STXBP3	protein_codir	0.12358385	0.09173154	0.18896369
ENSG0000023	AF127936.9	lncRNA	0.12357077	0.53569614	0.67592176
ENSG0000011	PITPNM1	protein_codir	0.12356989	0.57937785	0.71125011
ENSG0000016	ROBO1	protein_codir	0.12355543	0.34444505	0.49756721
ENSG0000017	GTF2IRD2B	protein_codir	0.12348728	0.58045648	0.71219439
ENSG0000026	RP11-46D6.1	lncRNA	0.1234691	0.49705255	0.64289904
ENSG0000016	CLPX	protein_codir	0.12326164	0.39789942	0.55090391
ENSG0000015	DBI	protein_codir	0.12313694	0.46462359	0.61457322
ENSG0000012	SPDEF	protein_codir	0.12303587	0.85921518	0.91602406
ENSG0000019	SLC9A8	protein_codir	0.12261374	0.42054535	0.57274417

ENSG0000002	AQR	protein_codir	0.12235135	0.37825959	0.5318433
ENSG0000010	RNF167	protein_codir	0.12233797	0.35552012	0.50914839
ENSG0000017	AKIRIN1	protein_codir	0.12233612	0.47355519	0.62200848
ENSG0000027	RP11-582E3.6	lncRNA	0.12223819	0.48839756	0.63539999
ENSG0000017	TIMM23B-AG	protein_codir	0.12210041	0.7937007	0.87182395
ENSG0000010	ARHGAP21	protein_codir	0.12207698	0.58545786	0.71613578
ENSG0000020	HSD3B2	protein_codir	0.12203804	0.94250998	NA
ENSG0000016	HPRT1	protein_codir	0.1220313	0.31230183	0.46410075
ENSG0000016	TSC1	protein_codir	0.12184305	0.42318049	0.57514208
ENSG0000022	LINC01534	lncRNA	0.12170633	0.60022726	0.72762803
ENSG0000013	USP8	protein_codir	0.12159034	0.09588656	0.19541184
ENSG0000017	ZDHHC14	protein_codir	0.12157945	0.33695494	0.48992921
ENSG0000014	SRPRB	protein_codir	0.12152418	0.49802476	0.64377048
ENSG0000002	PLEKHO1	protein_codir	0.12144711	0.64745055	0.76426078
ENSG0000028	RP11-454H13	lncRNA	0.12144664	0.67710092	0.78736643
ENSG0000019	LRIG2	protein_codir	0.1214191	0.50285439	0.64758886
ENSG0000026	AC008746.12	lncRNA	0.12138432	0.85145662	0.91162661
ENSG0000024	RP11-657O9.1	lncRNA	0.12124323	0.90628321	0.94423019
ENSG0000001	RUFY3	protein_codir	0.12116842	0.45777984	0.60822971
ENSG0000012	GZF1	protein_codir	0.12111422	0.4697439	0.61888601
ENSG0000015	LONRF1	protein_codir	0.12099267	0.6755835	0.78630194
ENSG0000008	PREP	protein_codir	0.12083431	0.35342328	0.50701454
ENSG0000024	CTD-2366F13.	lncRNA	0.12082884	0.63531969	0.75512221
ENSG0000025	TAS2R20	protein_codir	0.12077951	0.76412245	0.85113424
ENSG0000016	FUBP1	protein_codir	0.12077442	0.37564062	0.52916157
ENSG0000027	LINC00653	lncRNA	0.12074354	0.75977462	0.84808337
ENSG0000017	CCS	protein_codir	0.12070698	0.56677097	0.70074591
ENSG0000010	SQLE	protein_codir	0.1206815	0.65920555	0.7736247
ENSG0000025	RP11-522B15.	lncRNA	0.12047548	0.63941323	0.75816417
ENSG0000027	RP11-687F6.5	lncRNA	0.1204339	0.88192438	0.93000731
ENSG0000019	ZNF655	protein_codir	0.12035961	0.30575967	0.45738097
ENSG0000006	RFXANK	protein_codir	0.1201811	0.41034583	0.56331704
ENSG0000024	RP11-383G6.3	lncRNA	0.11986317	0.92179455	0.95394977
ENSG0000023	AC006547.13	lncRNA	0.11977659	0.95092736	NA
ENSG0000014	ZNF593	protein_codir	0.11977639	0.49966153	0.64501081
ENSG0000028	RP11-122G18	lncRNA	0.11975002	0.9426787	NA
ENSG0000015	UBN2	protein_codir	0.11967839	0.37493126	0.52848183
ENSG0000025	CTC-558O2.2	lncRNA	0.11965104	0.88490647	0.93169355
ENSG0000014	MAP1LC3B	protein_codir	0.11960333	0.50136465	0.6461091
ENSG0000015	CC2D1B	protein_codir	0.11948581	0.22025563	0.36110248
ENSG0000017	ADGRG2	protein_codir	0.11943212	0.80294856	0.87796672
ENSG0000026	LINC00683	lncRNA	0.11924399	0.85098711	0.91131047
ENSG0000013	OS9	protein_codir	0.11916853	0.19260063	0.32760641
ENSG0000015	CXXC1	protein_codir	0.11908562	0.52284744	0.6651182
ENSG0000027	XXbac-BPGBP	lncRNA	0.11876744	0.67594158	0.786515
ENSG0000018	GOLGA6L4	protein_codir	0.11874234	0.74975873	0.84051097
ENSG0000017	PSME3IP1	protein_codir	0.11870554	0.36631098	0.52020717

ENSG0000026	RP11-452L6.7	lncRNA	0.1186493	0.80687071	0.88047761
ENSG0000000	ZFX	protein_codir	0.1185054	0.52241533	0.66481346
ENSG0000017	GMPPB	protein_codir	0.11846901	0.48275638	0.63018905
ENSG0000012	HNRNPA2B1	protein_codir	0.1184503	0.35715448	0.51072258
ENSG0000028	RP11-686O6.3	lncRNA	0.11838329	0.94366938	NA
ENSG0000017	TMEM80	protein_codir	0.11834003	0.60040704	0.72767014
ENSG0000018	FAM174B	protein_codir	0.11833776	0.62620175	0.74795417
ENSG0000019	ZBTB14	protein_codir	0.11827816	0.47458652	0.62282998
ENSG0000016	PEX2	protein_codir	0.11824986	0.17399321	0.30399372
ENSG0000015	SPATA2	protein_codir	0.11823472	0.41865357	0.5713329
ENSG0000015	ZNF689	protein_codir	0.1182149	0.36127385	0.5151463
ENSG0000016	SDHAF2	protein_codir	0.11817993	0.20801904	0.34665657
ENSG0000000	CRAMP1	protein_codir	0.1181525	0.35246275	0.50603083
ENSG0000017	MYPOP	protein_codir	0.11810017	0.50999821	0.65413534
ENSG0000003	NSMAF	protein_codir	0.11804473	0.17584082	0.30607029
ENSG0000026	MAFG-DT	lncRNA	0.11796555	0.6946588	0.79976973
ENSG0000006	CTSA	protein_codir	0.11795358	0.39528768	0.5482773
ENSG0000017	ATOX1	protein_codir	0.11790268	0.56065511	0.69562437
ENSG0000000	DHX33	protein_codir	0.11781051	0.26990035	0.41772092
ENSG0000005	PUM2	protein_codir	0.11765355	0.30471956	0.45619569
ENSG0000028	RP11-2I7.1	protein_codir	0.11765294	0.82334786	0.89249874
ENSG0000013	PNPLA8	protein_codir	0.11757701	0.55608977	0.69194934
ENSG0000027	RP11-36I17.2	lncRNA	0.11734337	0.93431223	0.96135692
ENSG0000011	ANKRD13C	protein_codir	0.11731144	0.34844027	0.50174236
ENSG0000011	NSUN4	protein_codir	0.11727983	0.31576647	0.4675138
ENSG0000010	MLX	protein_codir	0.11718531	0.12155988	0.23335306
ENSG0000015	MFSD14A	protein_codir	0.11706444	0.43233773	0.58364894
ENSG0000021	PHB2	protein_codir	0.11696169	0.32962448	0.48211333
ENSG0000007	ING3	protein_codir	0.11682403	0.30048507	0.45152046
ENSG0000018	ZFP90	protein_codir	0.1167	0.35656192	0.51019221
ENSG0000019	RD3	protein_codir	0.11667523	0.89666456	0.93852794
ENSG0000012	BBS9	protein_codir	0.11664956	0.43546313	0.58680842
ENSG0000027	DGCR11	lncRNA	0.11660173	0.52791576	0.66940746
ENSG0000028	CTD-2168K21	lncRNA	0.11652571	0.96701071	NA
ENSG0000010	STK24	protein_codir	0.11645044	0.37121911	0.52464165
ENSG0000013	UBAC1	protein_codir	0.11640172	0.32164855	0.47379064
ENSG0000018	TRMT2B	protein_codir	0.11639413	0.52009762	0.66283382
ENSG0000019	SGMS1	protein_codir	0.11635262	0.51008993	0.65419223
ENSG0000003	AGA	protein_codir	0.11629266	0.55902567	0.69453866
ENSG0000014	POGZ	protein_codir	0.11625405	0.36752007	0.52114716
ENSG0000010	TIMM9	protein_codir	0.11616848	0.39367664	0.54669204
ENSG0000026	ADGRL1-AS1	lncRNA	0.11613395	0.58106408	0.71265497
ENSG0000018	MSL1	protein_codir	0.11611823	0.31779274	0.46975839
ENSG0000021	KCTD11	protein_codir	0.11608983	0.59231787	0.72123703
ENSG0000027	RP11-286H15	lncRNA	0.11589651	0.82828939	0.89622515
ENSG0000024	LINC02021	lncRNA	0.11574034	0.82652322	0.89506259
ENSG0000017	MBLAC2	protein_codir	0.11567246	0.59881735	0.72678012

ENSG0000010	KAT2A	protein_codir	0.11557891	0.72107323	0.81993512
ENSG0000017	NINJ2-AS1	lncRNA	0.11552344	0.55706768	0.69288523
ENSG0000017	CTD-2368P22	lncRNA	0.11545352	0.70343062	0.80687544
ENSG0000007	SREBF1	protein_codir	0.11544388	0.76157123	0.84950516
ENSG0000016	SPATA33	protein_codir	0.11514987	0.54360202	0.68200262
ENSG0000018	CCDC125	protein_codir	0.11511827	0.47456541	0.62282998
ENSG0000023	MMADHC-DT	lncRNA	0.11508678	0.83850001	0.90316843
ENSG0000014	GFOD2	protein_codir	0.11497808	0.27402023	0.42206328
ENSG0000010	POLI	protein_codir	0.11493985	0.51824938	0.66124857
ENSG0000025	RP11-326C3.7	lncRNA	0.11481527	0.72601973	0.82355189
ENSG0000013	AFDN	protein_codir	0.11472138	0.43822593	0.58935148
ENSG0000019	NPIPB6	protein_codir	0.11472016	0.85409011	0.91288916
ENSG0000024	NAA80	protein_codir	0.11467706	0.46857525	0.61799486
ENSG0000015	TAB3	protein_codir	0.11465569	0.40383682	0.55694677
ENSG0000017	XKR6	protein_codir	0.11445455	0.70225656	0.80607025
ENSG0000021	VPS16	protein_codir	0.11441163	0.43538788	0.58680842
ENSG0000016	PTPN9	protein_codir	0.11438739	0.4384515	0.58958279
ENSG0000008	RIF1	protein_codir	0.11433212	0.49529945	0.64126802
ENSG0000024	DDOST	protein_codir	0.11424038	0.42025347	0.57258292
ENSG0000025	RP11-805J14.	lncRNA	0.11406014	0.90053802	0.94052192
ENSG0000024	RP11-115J16.	lncRNA	0.11387919	0.82232569	0.89170568
ENSG0000017	SCAI	protein_codir	0.11359698	0.59316408	0.72191733
ENSG0000012	PCNX4	protein_codir	0.11351929	0.19607931	0.33192636
ENSG0000011	TIA1	protein_codir	0.11334794	0.54852069	0.68599237
ENSG0000017	EIF1	protein_codir	0.1133479	0.56655491	0.70055275
ENSG0000013	ZNF317	protein_codir	0.11330963	0.21486565	0.35487587
ENSG0000007	TP53INP2	protein_codir	0.11323589	0.75013096	0.84069688
ENSG0000016	HPS3	protein_codir	0.11293894	0.56926789	0.703031
ENSG0000003	CUL3	protein_codir	0.11276961	0.35632204	0.50995465
ENSG0000022	RP11-380G5.2	lncRNA	0.11269832	0.92381608	0.9551671
ENSG0000023	RP11-162G10	lncRNA	0.11262693	0.77626378	0.85957699
ENSG0000018	TEX38	protein_codir	0.11246272	0.9187747	0.95196598
ENSG0000026	RP11-589P10.	lncRNA	0.11245601	0.80533431	0.8793917
ENSG0000013	HACL1	protein_codir	0.11240213	0.31367978	0.46539727
ENSG0000016	SLC22A15	protein_codir	0.11229157	0.65335197	0.76900969
ENSG0000024	ZCCHC3	protein_codir	0.11227485	0.46149149	0.61166528
ENSG0000016	WDR26	protein_codir	0.11223066	0.4118457	0.56480528
ENSG0000026	CLASP1-AS1	lncRNA	0.11221781	0.89573709	0.93794815
ENSG0000004	SLC66A1	protein_codir	0.1120831	0.44432788	0.59539763
ENSG0000011	ACVR2B	protein_codir	0.11204308	0.60731276	0.73328867
ENSG0000016	TSACC	protein_codir	0.11190263	0.94965244	0.97048879
ENSG0000014	EFL1	protein_codir	0.11182721	0.37942726	0.532807
ENSG0000017	MED16	protein_codir	0.11181195	0.37024088	0.52366106
ENSG0000027	RP11-73M11.	lncRNA	0.11168937	0.91076826	0.94686714
ENSG0000027	RP11-61K9.3	lncRNA	0.1116198	0.72011657	0.81921083
ENSG0000016	SETD5	protein_codir	0.11157243	0.2959943	0.44644837
ENSG0000010	ZNF516	protein_codir	0.11147238	0.64474584	0.76238891

ENSG0000019	ZNF789	protein_codir	0.11146376	0.68701344	0.79474411
ENSG0000016	ZNF226	protein_codir	0.11143986	0.57257767	0.7056307
ENSG0000027	TSNAX-DISC1	protein_codir	0.11139523	0.92258947	0.95437907
ENSG0000016	ATP9B	protein_codir	0.11136745	0.17753564	0.3081658
ENSG0000011	GNAI2	protein_codir	0.11130483	0.30330841	0.45462518
ENSG0000027	RP13-131K19.	lncRNA	0.11129014	0.88030151	0.92904084
ENSG0000028	UCKL1-AS1	lncRNA	0.11128122	0.69547903	0.80051387
ENSG0000016	SLC25A32	protein_codir	0.11128087	0.53569126	0.67592176
ENSG0000023	RP1-125I3.2	lncRNA	0.11106857	0.94146865	NA
ENSG0000018	PP7080	lncRNA	0.1110431	0.42076104	0.57293531
ENSG0000025	CTD-3051D23	lncRNA	0.11092933	0.88448792	0.93139954
ENSG0000021	ZNF845	protein_codir	0.11091175	0.60034905	0.72767014
ENSG0000011	PARD3B	protein_codir	0.11089683	0.69212894	0.79792142
ENSG0000027	RP11-108L7.1	lncRNA	0.11082171	0.85451886	0.91313541
ENSG0000018	EP400	protein_codir	0.11073744	0.34967218	0.50317525
ENSG0000010	STS	protein_codir	0.11059433	0.70826509	0.81057795
ENSG0000016	BROX	protein_codir	0.11059027	0.47875327	0.62657579
ENSG0000019	RAD54B	protein_codir	0.11051421	0.80353646	0.8782271
ENSG0000016	ZBTB5	protein_codir	0.11038426	0.3745749	0.52814125
ENSG0000009	SPTLC1	protein_codir	0.11036609	0.29280905	0.442926
ENSG0000026	RP11-252A24	lncRNA	0.11031753	0.78439969	0.86518587
ENSG0000021	NUTM2D	protein_codir	0.11011364	0.63903134	0.75794224
ENSG0000006	DDX3Y	protein_codir	0.11007677	0.82831063	0.89622515
ENSG0000010	KIAA0930	protein_codir	0.11007237	0.4991883	0.64476154
ENSG0000018	CHP1	protein_codir	0.10998453	0.35601392	0.5095929
ENSG0000012	MAU2	protein_codir	0.10983576	0.33205612	0.48489955
ENSG0000019	SLC25A29	protein_codir	0.10977362	0.63447319	0.7545057
ENSG0000012	MAP2K2	protein_codir	0.10968916	0.35217487	0.50572267
ENSG0000002	UBR2	protein_codir	0.1096359	0.33778557	0.49058527
ENSG0000016	ZNF91	protein_codir	0.10949511	0.6153225	0.73931228
ENSG0000026	NBPF9	protein_codir	0.10935606	0.50497615	0.64958816
ENSG0000010	CBLL1	protein_codir	0.10934571	0.51385276	0.65718712
ENSG0000010	NKAP	protein_codir	0.10931638	0.25077385	0.39676771
ENSG0000009	SETD1A	protein_codir	0.10931369	0.24353704	0.38859
ENSG0000004	CTNS	protein_codir	0.10925646	0.4948159	0.6408823
ENSG0000015	CSNK1G3	protein_codir	0.10909071	0.4186712	0.5713329
ENSG0000019	ZSCAN25	protein_codir	0.10905822	0.45731476	0.60787496
ENSG0000014	INIP	protein_codir	0.10905683	0.46186516	0.61197965
ENSG0000023	RP11-96C23.9	lncRNA	0.10901364	0.90762242	0.9449625
ENSG0000010	SEC23IP	protein_codir	0.10889525	0.41167571	0.5647404
ENSG0000010	SNW1	protein_codir	0.10879747	0.11826275	0.22851666
ENSG0000010	MTMR4	protein_codir	0.10875706	0.55017279	0.68731564
ENSG0000016	BORCS7	protein_codir	0.10874246	0.45001719	0.60085779
ENSG0000014	AUH	protein_codir	0.1086534	0.36729076	0.52108007
ENSG0000019	PCNX3	protein_codir	0.108603	0.3919922	0.54499268
ENSG0000025	SMIM20	protein_codir	0.10851696	0.28685973	0.43641576
ENSG0000011	MESD	protein_codir	0.10849435	0.18706458	0.32054987

ENSG0000007	NDST1	protein_codir	0.10820284	0.59451487	0.72305155
ENSG0000013	DLG4	protein_codir	0.10809272	0.65661197	0.77176146
ENSG0000026	MPC1-DT	lncRNA	0.10804387	0.86388986	0.91909549
ENSG0000018	HMGN4	protein_codir	0.10801672	0.21408498	0.35390394
ENSG0000017	SMCO3	protein_codir	0.10801244	0.7970488	0.87386821
ENSG0000016	AKNAD1	protein_codir	0.1078373	0.87576638	0.92634296
ENSG0000017	CD34	protein_codir	0.10779087	0.73916031	0.83324012
ENSG0000011	NFE2L2	protein_codir	0.10772821	0.57857179	0.71067121
ENSG0000018	GPR89B	protein_codir	0.10771161	0.61035067	0.73573454
ENSG0000016	ARL13B	protein_codir	0.10767571	0.6367224	0.75633376
ENSG0000016	SPNS1	protein_codir	0.10760715	0.34525964	0.49812324
ENSG0000027	H3C10	protein_codir	0.10754123	0.80148997	0.87689258
ENSG0000023	LINC01503	lncRNA	0.10724235	0.70490038	0.8079838
ENSG0000023	ALKBH6	protein_codir	0.1072389	0.6483663	0.76470306
ENSG0000016	CFAP410	protein_codir	0.10720564	0.69563664	0.80065726
ENSG0000014	JTB	protein_codir	0.10694763	0.44214674	0.59321519
ENSG0000000	ST3GAL1	protein_codir	0.10693434	0.44864089	0.59949015
ENSG0000000	GGCT	protein_codir	0.10692322	0.51026178	0.65426072
ENSG0000019	SIPA1L1	protein_codir	0.10686033	0.47901928	0.62686025
ENSG0000012	GPSM2	protein_codir	0.106769	0.68232409	0.7911087
ENSG0000028	XXbac-BPG25	lncRNA	0.10668677	0.87845229	0.92776278
ENSG0000011	DNAJC6	protein_codir	0.10667464	0.69566159	0.80065726
ENSG0000021	ZBED1	protein_codir	0.10651512	0.42997826	0.58147276
ENSG0000017	PHF8	protein_codir	0.10642254	0.31946778	0.47157822
ENSG0000015	DNAI4	protein_codir	0.10630462	0.64156498	0.75960326
ENSG0000015	PLOD2	protein_codir	0.10628796	0.76837906	0.8540981
ENSG0000008	OTUB2	protein_codir	0.10628765	0.78409518	0.86505386
ENSG0000018	BTBD8	protein_codir	0.10605406	0.67998043	0.78958461
ENSG0000013	ADCK2	protein_codir	0.10597791	0.52649718	0.66834507
ENSG0000010	MLC1	protein_codir	0.10596213	0.88379654	0.93115014
ENSG0000010	OLFM4	protein_codir	0.10591544	0.8757292	0.92633911
ENSG0000016	TRAPPC2L	protein_codir	0.10571784	0.4018339	0.55490487
ENSG0000026	MIF4GD-DT	lncRNA	0.10559683	0.68744306	0.7949082
ENSG0000023	GTF3C2-AS1	lncRNA	0.10558979	0.93575535	0.96205333
ENSG0000027	RP11-196G11	lncRNA	0.10557947	0.54235642	0.68108958
ENSG0000014	PIP5K1A	protein_codir	0.1054217	0.41582901	0.56871489
ENSG0000015	FRRS1	protein_codir	0.10536466	0.70604061	0.8088622
ENSG0000007	ACADVL	protein_codir	0.10516873	0.58129452	0.71271606
ENSG0000012	TRMT13	protein_codir	0.1050174	0.6548882	0.77025985
ENSG0000026	CTC-444N24.8	lncRNA	0.10500862	0.91245587	0.94805068
ENSG0000018	ZNF573	protein_codir	0.10496477	0.61349227	0.7379434
ENSG0000025	CTC-467M3.3	lncRNA	0.10494903	0.94414137	NA
ENSG0000019	ABCA4	protein_codir	0.10488209	0.83204262	0.89885466
ENSG0000013	MKRN1	protein_codir	0.1048768	0.27492382	0.42305398
ENSG0000024	LRP4-AS1	lncRNA	0.10476641	0.92446823	0.95549948
ENSG0000010	JHY	protein_codir	0.10472059	0.58115693	0.71270555
ENSG0000023	RP11-443B7.1	lncRNA	0.10463211	0.82873634	0.89655658

ENSG0000028	RP11-416I2.3	lncRNA	0.10460181	0.88896411	0.9340189
ENSG0000010	C22orf31	protein_codir	0.10450401	0.92641657	0.95648785
ENSG0000015	AIFM1	protein_codir	0.10447051	0.42035332	0.57259756
ENSG0000026	RP11-17J14.2	lncRNA	0.10446279	0.90620009	0.94421027
ENSG0000026	RP11-893F2.1	lncRNA	0.10423909	0.89093825	0.93501461
ENSG0000016	ACAA2	protein_codir	0.1041472	0.5014021	0.6461102
ENSG0000010	RAB36	protein_codir	0.10413118	0.63901235	0.75794224
ENSG0000010	TRPC4AP	protein_codir	0.10390731	0.2523291	0.39847512
ENSG0000022	ZRANB2-AS2	lncRNA	0.10372675	0.74396805	0.83645679
ENSG0000018	AEN	protein_codir	0.10363259	0.50137087	0.6461091
ENSG0000007	CBFA2T2	protein_codir	0.10361015	0.44765506	0.59852032
ENSG0000022	SOS1-IT1	lncRNA	0.10341602	0.75964036	0.84800206
ENSG0000027	RP11-11N7.4	lncRNA	0.10335316	0.93486246	0.96157798
ENSG0000026	RP11-85A1.3	lncRNA	0.10334914	0.84261237	0.90611045
ENSG0000024	TNFSF12-TNF!	protein_codir	0.10334637	0.9141286	0.9491105
ENSG0000011	ABTB1	protein_codir	0.10328552	0.54625154	0.68420786
ENSG0000017	IMPDH2	protein_codir	0.10286353	0.48196527	0.62949263
ENSG0000019	EOLA2	protein_codir	0.10283194	0.423021	0.57504872
ENSG0000013	ATRAID	protein_codir	0.10273593	0.35291664	0.5065245
ENSG0000012	GPR83	protein_codir	0.10249822	0.86872331	0.92210667
ENSG0000012	USP45	protein_codir	0.10239878	0.52289593	0.66514924
ENSG0000016	RPL36AL	protein_codir	0.10239643	0.5039806	0.64871017
ENSG0000014	NOB1	protein_codir	0.10237192	0.41831088	0.57097833
ENSG0000018	TRIM69	protein_codir	0.10232914	0.46793192	0.61729377
ENSG0000013	TMEM254	protein_codir	0.10223275	0.60156714	0.72868747
ENSG0000018	AGRN	protein_codir	0.10220028	0.45920758	0.60965744
ENSG0000010	ALKBH1	protein_codir	0.10214546	0.396663	0.54952168
ENSG0000018	KIAA0408	protein_codir	0.10211945	0.79444306	0.87230935
ENSG0000017	WDR6	protein_codir	0.10199848	0.55841691	0.69396963
ENSG0000026	RP11-73K9.2	lncRNA	0.10195538	0.76340931	0.85066225
ENSG0000012	SNRPD2	protein_codir	0.10190365	0.47918117	0.62698298
ENSG0000015	AGPAT5	protein_codir	0.10188951	0.59890764	0.72680359
ENSG0000006	ACAA1	protein_codir	0.10185935	0.3709226	0.52430308
ENSG0000018	DMBT1	protein_codir	0.10181814	0.87588348	0.92639587
ENSG0000023	RP11-7O11.9	lncRNA	0.10176058	0.84967011	0.91056099
ENSG0000026	RP11-16E18.3	lncRNA	0.10175712	0.78773578	0.86743635
ENSG0000027	RP11-378J18.	lncRNA	0.10170166	0.68190759	0.79078108
ENSG0000010	ERCC2	protein_codir	0.10160355	0.53792996	0.67754479
ENSG0000027	RP11-33N14.3	lncRNA	0.10151051	0.86963601	0.92264997
ENSG0000013	GPNMB	protein_codir	0.1014962	0.71023855	0.81175158
ENSG0000012	PANK3	protein_codir	0.10143297	0.62619774	0.74795417
ENSG0000010	EIF5	protein_codir	0.10135774	0.55975435	0.69513129
ENSG0000020	PRAMEF20	protein_codir	0.10127266	0.97523981	NA
ENSG0000023	ZBTB40-IT1	lncRNA	0.10127266	0.97523981	NA
ENSG0000028	RP5-848E13.4	lncRNA	0.10127266	0.97523981	NA
ENSG0000022	RP4-533D7.4	lncRNA	0.10127266	0.97523981	NA
ENSG0000026	RP11-195M16	lncRNA	0.10127266	0.97523981	NA

ENSG0000016	LENEP	protein_codir	0.10127266	0.97523981	NA
ENSG0000028	RP5-879K22.2	lncRNA	0.10127266	0.97523981	NA
ENSG0000022	AC096669.1	lncRNA	0.10127266	0.97523981	NA
ENSG0000028	RP11-803D5.5	lncRNA	0.10127266	0.97523981	NA
ENSG0000022	AC073321.4	lncRNA	0.10127266	0.97523981	NA
ENSG0000023	LINC01937	lncRNA	0.10127266	0.97523981	NA
ENSG0000028	RP11-1406H1	lncRNA	0.10127266	0.97523981	NA
ENSG0000013	NDST4	protein_codir	0.10127266	0.97523981	NA
ENSG0000025	MGC32805	lncRNA	0.10127266	0.97523981	NA
ENSG0000027	AC005754.7	lncRNA	0.10127266	0.97523981	NA
ENSG0000028	CH17-140K24	lncRNA	0.10127266	0.97523981	NA
ENSG0000028	CTB-76P12.1	lncRNA	0.10127266	0.97523981	NA
ENSG0000023	ANKRD66	protein_codir	0.10127266	0.97523981	NA
ENSG0000022	RP11-173D14	lncRNA	0.10127266	0.97523981	NA
ENSG0000022	LAMA4-AS1	lncRNA	0.10127266	0.97523981	NA
ENSG0000028	RP3-425C14.8	lncRNA	0.10127266	0.97523981	NA
ENSG0000025	CTA-398F10.2	lncRNA	0.10127266	0.97523981	NA
ENSG0000028	RP11-685B14.	lncRNA	0.10127266	0.97523981	NA
ENSG0000022	RP11-403H13	lncRNA	0.10127266	0.97523981	NA
ENSG0000022	RP11-475I24.8	lncRNA	0.10127266	0.97523981	NA
ENSG0000019	LINC02692	lncRNA	0.10127266	0.97523981	NA
ENSG0000014	VAX1	protein_codir	0.10127266	0.97523981	NA
ENSG0000025	RP5-916O11.2	lncRNA	0.10127266	0.97523981	NA
ENSG0000028	RP11-613M5.	lncRNA	0.10127266	0.97523981	NA
ENSG0000022	LINC00355	lncRNA	0.10127266	0.97523981	NA
ENSG0000018	SIX6	protein_codir	0.10127266	0.97523981	NA
ENSG0000025	RP11-253M7.	lncRNA	0.10127266	0.97523981	NA
ENSG0000026	RP11-517A5.7	lncRNA	0.10127266	0.97523981	NA
ENSG0000024	RP11-303E16.	lncRNA	0.10127266	0.97523981	NA
ENSG0000018	SPEM2	protein_codir	0.10127266	0.97523981	NA
ENSG0000027	LHX1-DT	lncRNA	0.10127266	0.97523981	NA
ENSG0000026	LINC01905	lncRNA	0.10127266	0.97523981	NA
ENSG0000028	CTD-2189E23.	lncRNA	0.10127266	0.97523981	NA
ENSG0000026	CTB-60B18.18	lncRNA	0.10127266	0.97523981	NA
ENSG0000026	CTD-3099C6.7	lncRNA	0.10127266	0.97523981	NA
ENSG0000025	MTRNR2L3	protein_codir	0.10127266	0.97523981	NA
ENSG0000022	RP1-302D9.1	lncRNA	0.10127266	0.97523981	NA
ENSG0000027	RP5-1039K5.1	lncRNA	0.10127266	0.97523981	NA
ENSG0000017	FMR1NB	protein_codir	0.10127266	0.97523981	NA
ENSG0000020	DAZ4	protein_codir	0.10127266	0.97523981	NA
ENSG0000023	LRRC52-AS1	lncRNA	0.10127266	0.97523981	NA
ENSG0000022	ASIC4-AS1	lncRNA	0.10127266	0.97523981	NA
ENSG0000028	RP11-797H7.7	lncRNA	0.10127266	0.97523981	NA
ENSG0000028	RP11-370B11.	lncRNA	0.10127266	0.97523981	NA
ENSG0000022	LINC01509	lncRNA	0.10127266	0.97523981	NA
ENSG0000028	RP11-135A24	lncRNA	0.10127266	0.97523981	NA
ENSG0000012	SCGB1D2	protein_codir	0.10127266	0.97523981	NA

ENSG0000025	RP11-770G2.5	lncRNA	0.10127266	0.97523981	NA
ENSG0000025	RP11-753H16	lncRNA	0.10127266	0.97523981	NA
ENSG0000025	RP11-114H23	lncRNA	0.10127266	0.97523981	NA
ENSG0000028	RP11-21A20.1	lncRNA	0.10127266	0.97523981	NA
ENSG0000020	TMEM235	protein_codir	0.10127266	0.97523981	NA
ENSG0000026	RP11-17A19.2	lncRNA	0.10127266	0.97523981	NA
ENSG0000026	RP11-53I6.4	lncRNA	0.10127266	0.97523981	NA
ENSG0000012	RBPJL	protein_codir	0.10127266	0.97523981	NA
ENSG0000027	RP13-379O24	lncRNA	0.10127266	0.97523981	NA
ENSG0000022	LINC02816	lncRNA	0.10127261	0.97512348	NA
ENSG0000023	LINC02640	lncRNA	0.10127259	0.97507761	NA
ENSG0000025	CTC-441N14.4	protein_codir	0.10127256	0.97502872	NA
ENSG0000028	RP11-543L14.	lncRNA	0.10127256	0.97501771	NA
ENSG0000028	LA16c-60D12.	lncRNA	0.10127255	0.97499457	NA
ENSG0000028	CTB-113P19.6	lncRNA	0.10127253	0.9749463	NA
ENSG0000024	CTB-26E19.1	lncRNA	0.10127253	0.9749463	NA
ENSG0000027	CH507-216K1	lncRNA	0.10127253	0.9749463	NA
ENSG0000026	CTD-2369P2.5	lncRNA	0.1012725	0.97487373	NA
ENSG0000028	RP11-473C18.	lncRNA	0.10127249	0.97486297	NA
ENSG0000025	AC083843.2	lncRNA	0.10127248	0.97483909	NA
ENSG0000028	RP11-422P15.	lncRNA	0.10127247	0.97481687	NA
ENSG0000028	LINC02885	lncRNA	0.10127247	0.9748139	NA
ENSG0000028	CTA-385F2.1	lncRNA	0.10127244	0.97474892	NA
ENSG0000025	RP11-10H3.1	lncRNA	0.10127244	0.97474892	NA
ENSG0000028	RP4-610C12.6	lncRNA	0.10127244	0.97473397	NA
ENSG0000022	AC008073.9	lncRNA	0.10127244	0.974729	NA
ENSG0000028	TMEM275	protein_codir	0.10127242	0.97469375	NA
ENSG0000028	RP11-549K20.	lncRNA	0.10127239	0.97462704	NA
ENSG0000025	RP11-120K9.2	lncRNA	0.10127238	0.9746037	NA
ENSG0000017	C1orf167-AS1	lncRNA	0.10127237	0.97458136	NA
ENSG0000028	RP11-43A14.2	lncRNA	0.10127237	0.97456963	NA
ENSG0000026	RP11-770J1.7	lncRNA	0.10127235	0.97451591	NA
ENSG0000022	LINC02608	lncRNA	0.10127233	0.97447492	NA
ENSG0000023	RP11-13J8.1	lncRNA	0.10127232	0.97446248	NA
ENSG0000020	GDF5-AS1	lncRNA	0.10127232	0.97443892	NA
ENSG0000028	RP11-529E10.	lncRNA	0.10127231	0.97442883	NA
ENSG0000028	RP11-331M18	lncRNA	0.1012723	0.97440646	NA
ENSG0000011	PRMT8	protein_codir	0.1012723	0.97439643	NA
ENSG0000025	PKNOX2-AS1	lncRNA	0.10127227	0.97431638	NA
ENSG0000024	CTB-49A3.2	lncRNA	0.10127226	0.974312	NA
ENSG0000028	RP11-349L8.1	lncRNA	0.10127226	0.97429293	NA
ENSG0000014	DMRTC2	protein_codir	0.10127225	0.97426765	NA
ENSG0000023	AC009501.4	lncRNA	0.10127225	0.97426677	NA
ENSG0000028	RP4-736L20.4	lncRNA	0.10127224	0.97424256	NA
ENSG0000009	KCNK16	protein_codir	0.10127223	0.97421474	NA
ENSG0000023	LINC02525	lncRNA	0.10127222	0.9742012	NA
ENSG0000022	UBE2E1-AS1	lncRNA	0.10127222	0.97419435	NA

ENSG0000026	RP11-728E14.	lncRNA	0.1012722	0.97414707	NA
ENSG0000023	LINC02625	lncRNA	0.10127218	0.97410236	NA
ENSG0000028	CTB-92J24.5	lncRNA	0.10127217	0.97407675	NA
ENSG0000026	RP11-297C4.3	lncRNA	0.10127217	0.97406976	NA
ENSG0000028	RP13-126P21.	lncRNA	0.10127216	0.97403783	NA
ENSG0000025	EXOC1L	protein_codir	0.10127214	0.97400522	NA
ENSG0000010	GML	protein_codir	0.10127214	0.97398989	NA
ENSG0000026	RP11-149I9.2	lncRNA	0.10127213	0.97397153	NA
ENSG0000023	RP11-229P13.	lncRNA	0.10127208	0.9738413	NA
ENSG0000023	AC005162.5	lncRNA	0.10127208	0.97383332	NA
ENSG0000025	LINC02698	lncRNA	0.10127202	0.97368976	NA
ENSG0000025	LINC02159	lncRNA	0.10127202	0.97368688	NA
ENSG0000022	LINC02069	lncRNA	0.10127201	0.97366247	NA
ENSG0000027	WI2-80269A6	lncRNA	0.10127201	0.97365298	NA
ENSG0000028	RP11-141F7.1	lncRNA	0.10127198	0.9735625	NA
ENSG0000016	ALPP	protein_codir	0.10127197	0.9735565	NA
ENSG0000017	APOF	protein_codir	0.10127197	0.97354135	NA
ENSG0000024	FOXL3	protein_codir	0.10127196	0.97352274	NA
ENSG0000022	LAPTM4A-DT	lncRNA	0.10127192	0.97339899	NA
ENSG0000027	LINC01396	lncRNA	0.1012719	0.97336126	NA
ENSG0000024	RP11-286E11.	lncRNA	0.1012719	0.97335811	NA
ENSG0000026	AC010646.3	protein_codir	0.10127188	0.97330276	NA
ENSG0000022	LINC02612	lncRNA	0.10127183	0.9731602	NA
ENSG0000026	RP11-354P11.	lncRNA	0.10127179	0.97304753	NA
ENSG0000023	SLCO4A1-AS1	lncRNA	0.10127173	0.97286596	NA
ENSG0000016	SLC66A3	protein_codir	0.10126002	0.48460427	0.6319831
ENSG0000013	RBM17	protein_codir	0.10103217	0.38949786	0.54246119
ENSG0000025	RP11-359J14.	lncRNA	0.1009855	0.83607736	0.90150063
ENSG0000009	ZNF268	protein_codir	0.10093165	0.53155217	0.67259808
ENSG0000022	AC010894.3	lncRNA	0.10089039	0.86265629	0.91817213
ENSG0000025	RP11-44N21.4	lncRNA	0.10080513	0.90445646	0.94333105
ENSG0000016	NKX3-1	protein_codir	0.10069832	0.76049225	0.84861005
ENSG0000016	ZNF597	protein_codir	0.10069508	0.57718386	0.70951757
ENSG0000026	RP11-589C21.	lncRNA	0.10060133	0.92795235	0.95709488
ENSG0000012	CEP250	protein_codir	0.100516	0.53727595	0.67723019
ENSG0000017	LRRN3	protein_codir	0.10044012	0.77086926	0.85583282
ENSG0000013	TRA2B	protein_codir	0.10028495	0.3219368	0.47413322
ENSG0000026	RP11-287D1.3	protein_codir	0.10023247	0.90622991	0.94421027
ENSG0000018	ZDHHC9	protein_codir	0.10022995	0.67907856	0.78900175
ENSG0000019	ZNF775	protein_codir	0.10022831	0.6761332	0.78663854
ENSG0000016	CATSPER2	protein_codir	0.10021772	0.81698431	0.88744668
ENSG0000016	MIX23	protein_codir	0.10018165	0.65512358	0.77050389
ENSG0000014	AGBL3	protein_codir	0.10017535	0.66340483	0.77729229
ENSG0000013	RLIM	protein_codir	0.10016188	0.59885151	0.72678965
ENSG0000027	RP5-858L17.1	lncRNA	0.10014264	0.74352092	0.83615829
ENSG0000016	DHRS3	protein_codir	0.10006038	0.7690978	0.85464719
ENSG0000013	CTNNBL1	protein_codir	0.10002925	0.49282607	0.63926436

ENSG0000024	ZNF585B	protein_codir	0.1000029	0.49118907	0.63791989
ENSG0000020	CSNK2B	protein_codir	0.09992601	0.44975212	0.60059728
ENSG0000018	SLC36A4	protein_codir	0.09984074	0.68891416	0.79581878
ENSG0000012	ZNF780B	protein_codir	0.09945787	0.7217167	0.82035537
ENSG0000013	PAK6	protein_codir	0.09924305	0.78368321	0.86468419
ENSG0000016	MCM3AP	protein_codir	0.09916371	0.36311383	0.51702727
ENSG0000014	DOC2A	protein_codir	0.09910751	0.85030652	0.91088958
ENSG0000005	NRIP2	protein_codir	0.09906329	0.72547746	0.82310579
ENSG0000015	ZNF235	protein_codir	0.09897506	0.6430011	0.76087959
ENSG0000019	RP11-566K11.	protein_codir	0.09895385	0.95447147	0.97320388
ENSG0000025	RP11-894P9.2	lncRNA	0.09894211	0.94455307	0.96764051
ENSG0000010	ETV2	protein_codir	0.09891644	0.76135275	0.84936436
ENSG0000017	PDIK1L	protein_codir	0.09885877	0.68435436	0.79256436
ENSG0000018	RABIF	protein_codir	0.09882082	0.45440249	0.6048189
ENSG0000004	RFC2	protein_codir	0.09879372	0.40744422	0.56046662
ENSG0000014	MTDH	protein_codir	0.09848311	0.47291469	0.62131493
ENSG0000018	TMEM232	protein_codir	0.09840671	0.72224278	0.82077252
ENSG0000014	SHQ1	protein_codir	0.09823572	0.47501037	0.62329737
ENSG0000026	RP3-406A7.7	lncRNA	0.0982108	0.88942377	0.93419857
ENSG0000016	MAIP1	protein_codir	0.09807469	0.54441106	0.68263175
ENSG0000018	BRF1	protein_codir	0.09798646	0.48257983	0.6300239
ENSG0000023	HCG18	lncRNA	0.09794243	0.37560846	0.52916157
ENSG0000016	TYW5	protein_codir	0.09791422	0.63089613	0.75151383
ENSG0000018	AP2A2	protein_codir	0.09781674	0.46843809	0.61784347
ENSG0000024	ISY1	protein_codir	0.0978034	0.36437857	0.51836909
ENSG0000025	RP11-714G18	lncRNA	0.09774638	0.9036428	0.94281848
ENSG0000013	PUM1	protein_codir	0.09763104	0.26755711	0.41532779
ENSG0000011	SLC25A36	protein_codir	0.09762277	0.38771899	0.54079379
ENSG0000017	MGMT	protein_codir	0.09758957	0.57162074	0.70479733
ENSG0000001	HFE	protein_codir	0.09758639	0.68735212	0.79488823
ENSG0000015	PAGE5	protein_codir	0.09757499	0.94959509	0.97048154
ENSG0000010	TRADD	protein_codir	0.09756695	0.56596019	0.70015708
ENSG0000016	UBE2Q1	protein_codir	0.09751053	0.18526693	0.31842808
ENSG0000012	CENPL	protein_codir	0.09750951	0.48378124	0.63114994
ENSG0000028	AC140134.2	lncRNA	0.09748528	0.92499901	0.9557977
ENSG0000028	RP11-974F13.	lncRNA	0.09748528	0.92499901	0.9557977
ENSG0000023	LINC01063	lncRNA	0.09737636	0.91944227	0.95230043
ENSG0000026	RP11-55K13.1	lncRNA	0.09736282	0.90985876	0.94632476
ENSG0000010	TMEM97	protein_codir	0.09736089	0.7026523	0.80628402
ENSG0000022	XXbac-BPG55	lncRNA	0.09734819	0.8635121	0.91879976
ENSG0000016	LSM12	protein_codir	0.09717924	0.45029728	0.60105175
ENSG0000015	MARVELD2	protein_codir	0.09710449	0.76240324	0.84997014
ENSG0000024	JMJD7	protein_codir	0.09704479	0.64896836	0.76508904
ENSG0000017	CHD9	protein_codir	0.09702529	0.52429459	0.66631346
ENSG0000025	CTD-2555O16	lncRNA	0.09700878	0.70784831	0.81039463
ENSG0000012	SNRPB	protein_codir	0.09698488	0.62927784	0.75029804
ENSG0000022	AC093802.1	lncRNA	0.09695593	0.9604834	NA

ENSG0000028	RP11-153F10.	lncRNA	0.09690194	0.92659577	0.95648785
ENSG0000011	RBM24	protein_codir	0.09673859	0.86479804	0.91942422
ENSG0000013	ELP2	protein_codir	0.09670401	0.27117028	0.41916965
ENSG0000023	LINC00665	lncRNA	0.09662637	0.60355639	0.73012564
ENSG0000009	SCFD1	protein_codir	0.09658212	0.35964123	0.51340142
ENSG0000016	WBP1L	protein_codir	0.0965788	0.47887223	0.62669751
ENSG0000015	DDX19B	protein_codir	0.09652947	0.32249704	0.47462931
ENSG0000014	PARN	protein_codir	0.09646203	0.32201	0.47420085
ENSG0000006	SFSWAP	protein_codir	0.09639982	0.57657856	0.70903381
ENSG0000014	TP53	protein_codir	0.09632546	0.5329009	0.67374911
ENSG0000010	NRF1	protein_codir	0.09631812	0.3250838	0.47739806
ENSG0000024	RP11-613D13	lncRNA	0.09624224	0.9306758	0.95900857
ENSG0000009	CBARP	protein_codir	0.09616468	0.8512807	0.91150894
ENSG0000024	ADAMTS9-AS	lncRNA	0.0960743	0.87089994	0.92331633
ENSG0000010	PPP6R2	protein_codir	0.09590413	0.48708115	0.63431474
ENSG0000024	FGF10-AS1	lncRNA	0.09582137	0.89786695	0.93928813
ENSG0000026	RP11-160E2.6	lncRNA	0.09572107	0.89732063	0.93900113
ENSG0000017	RPL15	protein_codir	0.09557749	0.5190624	0.66192736
ENSG0000010	NBN	protein_codir	0.09556273	0.52542397	0.66732009
ENSG0000016	ANKRA2	protein_codir	0.09551492	0.50636007	0.65085933
ENSG0000013	SPTBN5	protein_codir	0.09533865	0.84288581	0.90620691
ENSG0000018	NKRF	protein_codir	0.09516313	0.40816828	0.56115765
ENSG0000011	HMGXB3	protein_codir	0.09489668	0.23511029	0.37855661
ENSG0000010	RBL2	protein_codir	0.0947897	0.47816547	0.62603955
ENSG0000011	GTF3C3	protein_codir	0.09478589	0.4615677	0.61170836
ENSG0000022	NPIPA3	protein_codir	0.0946475	0.87742948	0.92728533
ENSG0000011	GNB3	protein_codir	0.09452588	0.79432229	0.8722555
ENSG0000027	RP11-799M12	lncRNA	0.09452133	0.87366821	0.92508016
ENSG0000012	POLR3GL	protein_codir	0.09448695	0.35402307	0.50761134
ENSG0000028	RP11-1406H1	lncRNA	0.09447261	0.91387658	0.94906208
ENSG0000028	RP11-471M10	lncRNA	0.09437781	0.77969248	0.86216296
ENSG0000016	PPM1K	protein_codir	0.0943495	0.64605277	0.76348326
ENSG0000025	PCDHGA12	protein_codir	0.09429733	0.76204581	0.84979756
ENSG0000015	FZD7	protein_codir	0.0942829	0.74107881	0.83459867
ENSG0000016	RHOBTB3	protein_codir	0.0942134	0.72928035	0.82599538
ENSG0000012	PIK3CA	protein_codir	0.09413197	0.63578572	0.75554607
ENSG0000011	SRSF3	protein_codir	0.09390155	0.51551692	0.65873594
ENSG0000017	SIMC1	protein_codir	0.09386253	0.45588944	0.60659343
ENSG0000013	KIF23	protein_codir	0.09384986	0.81369682	0.88546023
ENSG0000019	ZMYM1	protein_codir	0.09377195	0.67223368	0.78383502
ENSG0000017	UBE2E1	protein_codir	0.09359454	0.24427211	0.38931308
ENSG0000026	RP11-61L19.1	lncRNA	0.09356584	0.91745249	0.95109552
ENSG0000021	ZNF888	protein_codir	0.09354247	0.64790067	0.76454826
ENSG0000016	MBD6	protein_codir	0.09352236	0.57847925	0.71058915
ENSG0000026	RP11-138I1.2	lncRNA	0.09351267	0.91646498	0.95047199
ENSG0000015	RABGEF1	protein_codir	0.09336739	0.58557292	0.71620924
ENSG0000010	AKAP10	protein_codir	0.09324514	0.39300864	0.54602346

ENSG0000010	SLC1A1	protein_codir	0.09313853	0.68841372	0.79555041
ENSG0000009	OSGEP	protein_codir	0.09306287	0.64831221	0.76469975
ENSG0000016	ZNF496	protein_codir	0.09304017	0.33356259	0.48645645
ENSG0000016	ELAPOR2	protein_codir	0.09299916	0.68426236	0.79250591
ENSG0000025	RP11-398J10.	lncRNA	0.09290356	0.90725768	0.94477051
ENSG0000014	PHIP	protein_codir	0.09289863	0.31959086	0.47163388
ENSG0000026	AC012531.25	lncRNA	0.09289227	0.86325821	0.91863578
ENSG0000013	UBL7	protein_codir	0.09287723	0.37144157	0.52484863
ENSG0000028	RP11-63L7.6	lncRNA	0.09285578	0.86945256	0.92261024
ENSG0000015	FBXO36	protein_codir	0.09274224	0.72460482	0.82234992
ENSG0000015	GUF1	protein_codir	0.09269576	0.5663876	0.70049032
ENSG0000000	AGK	protein_codir	0.09240831	0.3273741	0.47968555
ENSG0000028	RP4-646N3.2	lncRNA	0.09228659	0.88270199	0.93061413
ENSG0000013	LSM7	protein_codir	0.09226678	0.6756446	0.78630194
ENSG0000001	AGPS	protein_codir	0.09218153	0.58399826	0.71495199
ENSG0000016	ENGASE	protein_codir	0.0921724	0.71828745	0.81803972
ENSG0000023	MAPKAPK5-A	lncRNA	0.09216581	0.58327555	0.71435403
ENSG0000011	TBP	protein_codir	0.09212575	0.40453364	0.55751804
ENSG0000004	EPN3	protein_codir	0.09205588	0.95575832	NA
ENSG0000026	RP1-239B22.5	lncRNA	0.09195602	0.81509483	0.8861026
ENSG0000027	RP1-257A7.5	lncRNA	0.09192859	0.87362229	0.925067
ENSG0000008	ANKRD10	protein_codir	0.09168338	0.65695659	0.77206796
ENSG0000023	LINC00271	lncRNA	0.09159869	0.78828505	0.8678738
ENSG0000010	HOMER2	protein_codir	0.09155089	0.73264273	0.82834305
ENSG0000011	PRDM4	protein_codir	0.09143024	0.49622582	0.642139
ENSG0000021	NPEPL1	protein_codir	0.09137845	0.67549457	0.78630194
ENSG0000014	LYAR	protein_codir	0.09126459	0.57138383	0.70474433
ENSG0000026	RP11-498C9.1	lncRNA	0.0912502	0.7232579	0.82154484
ENSG0000016	SPIDR	protein_codir	0.09098769	0.24681853	0.39199171
ENSG0000013	KIAA1109	protein_codir	0.09096961	0.62858823	0.7497671
ENSG0000019	MT-ND1	protein_codir	0.09090515	0.7901985	0.86929074
ENSG0000020	BRD2	protein_codir	0.0908921	0.51229024	0.65591769
ENSG0000016	BMT2	protein_codir	0.09065252	0.58608326	0.71651862
ENSG0000026	AC006942.4	lncRNA	0.09055903	0.84932735	0.91035502
ENSG0000012	PARD6B	protein_codir	0.09053109	0.72616238	0.82367988
ENSG0000011	PUS3	protein_codir	0.09047465	0.46406115	0.61397629
ENSG0000010	RABGGTA	protein_codir	0.09042315	0.4963444	0.64222957
ENSG0000026	RP13-1032I1.7	lncRNA	0.09035289	0.68321204	0.79173939
ENSG0000018	PRPF39	protein_codir	0.09026354	0.64717953	0.76416225
ENSG0000014	NDUFB11	protein_codir	0.09023366	0.40074409	0.55392441
ENSG0000013	LDHA	protein_codir	0.09023237	0.70371707	0.80706995
ENSG0000026	RP11-31I22.3	lncRNA	0.09020102	0.92872787	0.95767553
ENSG0000024	TMED2-DT	lncRNA	0.09004161	0.88385901	0.93115014
ENSG0000010	KCNT1	protein_codir	0.09001845	0.84068228	0.90475022
ENSG0000011	ARSB	protein_codir	0.08998587	0.68940791	0.79608037
ENSG0000015	MSI2	protein_codir	0.08990856	0.61228992	0.73720347
ENSG0000026	ZNF460-AS1	lncRNA	0.08962837	0.84551919	0.90794449

ENSG000002З	RPS27AP5	protein_codir	0.08925262	0.76637718	0.8527652
ENSG000002З	RP11-76I23.7	lncRNA	0.08922039	0.92656499	0.95648785
ENSG000001З	LANCL2	protein_codir	0.0890457	0.53843828	0.67798267
ENSG0000014	PRPS1	protein_codir	0.08902045	0.54484853	0.68300924
ENSG0000012	TTPAL	protein_codir	0.08896183	0.59931542	0.72719289
ENSG000001З	CHSY1	protein_codir	0.08876987	0.74534617	0.83739266
ENSG000001З	CARF	protein_codir	0.08876616	0.65949138	0.77389438
ENSG000001C	SLC25A17	protein_codir	0.0886775	0.46571066	0.61535739
ENSG000001C	COPE	protein_codir	0.08852206	0.6058556	0.73220144
ENSG0000027	RP11-486I11.:	lncRNA	0.08849451	0.78401688	0.86500544
ENSG0000018	LITAF	protein_codir	0.08840704	0.59428858	0.72283999
ENSG000000З	LRRC7	protein_codir	0.0883916	NA	NA
ENSG0000027	RP11-127B20.	lncRNA	0.08813582	0.79569871	0.87311344
ENSG000001C	WSB1	protein_codir	0.08802247	0.68027581	0.78979706
ENSG0000015	DUSP23	protein_codir	0.088016	0.59191099	0.72099588
ENSG000001C	TM9SF4	protein_codir	0.08774313	0.28445175	0.43403996
ENSG0000015	NSMCE2	protein_codir	0.08769707	0.44002818	0.5909857
ENSG0000015	LPCAT1	protein_codir	0.08744808	0.62074227	0.7439749
ENSG0000025	RP11-381K20.	lncRNA	0.08737872	0.80915508	0.88209912
ENSG0000018	ERAS	protein_codir	0.08732475	0.95371888	0.97285767
ENSG000001З	TPMT	protein_codir	0.08718131	0.61266963	0.73743563
ENSG0000014	YIPF5	protein_codir	0.08717795	0.61580199	0.739723
ENSG0000012	PREX1	protein_codir	0.08691019	0.77182639	0.85643115
ENSG0000016	CDKN2AIP	protein_codir	0.08673285	0.5428063	0.68140669
ENSG0000007	MYDGF	protein_codir	0.08666181	0.64354639	0.76136175
ENSG0000016	RAET1E	protein_codir	0.08656104	0.87457039	0.92555894
ENSG0000019	CPLANE1	protein_codir	0.08653815	0.57398716	0.70671762
ENSG000001З	STXBP1	protein_codir	0.08644945	0.74585839	0.83765594
ENSG0000011	UCHL5	protein_codir	0.08642862	0.4851394	0.63247193
ENSG0000016	ABCF3	protein_codir	0.0862056	0.5172763	0.66040359
ENSG0000028	RP11-572M14	lncRNA	0.08618664	0.95261983	NA
ENSG0000027	RP11-214K3.2	lncRNA	0.08615235	0.87243765	0.92429161
ENSG0000018	ARID2	protein_codir	0.08613364	0.52693459	0.66867063
ENSG0000017	PITPNA	protein_codir	0.08588654	0.44290298	0.594007
ENSG0000018	VPS33B	protein_codir	0.08586065	0.53930121	0.67849001
ENSG0000011	NPRL2	protein_codir	0.08568289	0.66767855	0.78051727
ENSG0000017	CCDC14	protein_codir	0.08557712	0.73175402	0.8275415
ENSG0000014	C18orf21	protein_codir	0.08551996	0.5100336	0.65415036
ENSG0000019	ERI2	protein_codir	0.08551637	0.591729	0.72083778
ENSG0000011	RSRC2	protein_codir	0.08547568	0.56322309	0.69767135
ENSG000001C	LPIN2	protein_codir	0.08546138	0.575219	0.70778049
ENSG0000015	CCAR2	protein_codir	0.0854328	0.31673534	0.46844606
ENSG0000014	RRNAD1	protein_codir	0.08534236	0.59229526	0.72123703
ENSG0000018	ALYREF	protein_codir	0.08529278	0.60709257	0.73317765
ENSG0000017	WFIKKN2	protein_codir	0.08525513	0.90079853	0.94068038
ENSG000002C	NHSL2	protein_codir	0.08524799	0.74858995	0.83965258
ENSG0000014	DDX46	protein_codir	0.08522291	0.38642647	0.53948136

ENSG0000000	KMT2E	protein_codir	0.08509416	0.49719318	0.64290616
ENSG0000022	RP11-505P4.7	lncRNA	0.08487615	0.96016263	NA
ENSG0000019	TOPORS	protein_codir	0.0848186	0.56723867	0.70108435
ENSG0000011	ENDOU	protein_codir	0.08475732	0.87042194	0.92310639
ENSG0000008	ZNF510	protein_codir	0.08474586	0.62840143	0.74960903
ENSG0000013	PDGFRA	protein_codir	0.08474157	0.79724109	0.87397496
ENSG0000016	SERPINB8	protein_codir	0.0846919	0.68967675	0.79629094
ENSG0000013	GSE1	protein_codir	0.08453226	0.53906251	0.67834099
ENSG0000016	ZNF764	protein_codir	0.084328	0.57240077	0.70547565
ENSG0000027	RP4-751H13.7	lncRNA	0.08432265	0.86795625	0.92171754
ENSG0000007	XRCC1	protein_codir	0.08415299	0.46210334	0.61220713
ENSG0000026	CTB-174O21.2	lncRNA	0.08400299	0.97113885	NA
ENSG0000027	GGNBP2	protein_codir	0.08396799	0.4854942	0.63278513
ENSG0000013	ATP13A3	protein_codir	0.0839422	0.76625895	0.85268923
ENSG0000014	PSEN2	protein_codir	0.08366272	0.69085818	0.79712059
ENSG0000012	EPS15L1	protein_codir	0.08360954	0.31759897	0.46949708
ENSG0000016	GRIN2C	protein_codir	0.08347788	0.86695527	0.92097326
ENSG0000025	FLJ20021	lncRNA	0.08346506	0.66683644	0.7797914
ENSG0000014	ZC3H8	protein_codir	0.08328596	0.66315759	0.77714
ENSG0000010	ZNF14	protein_codir	0.08327434	0.76166784	0.84957752
ENSG0000011	GTF2H3	protein_codir	0.08326602	0.5827898	0.71394723
ENSG0000014	IDI2	protein_codir	0.08325452	0.95995467	NA
ENSG0000022	AC069513.4	lncRNA	0.08319593	0.91552497	0.94988166
ENSG0000011	CASP8AP2	protein_codir	0.08319199	0.62984484	0.75074721
ENSG0000028	CTB-88F18.5	lncRNA	0.08318559	0.85270305	0.91235989
ENSG0000014	MEPCE	protein_codir	0.08294264	0.65926147	0.77365745
ENSG0000018	SS18L1	protein_codir	0.08292426	0.53857398	0.67803516
ENSG0000012	FLRT3	protein_codir	0.08281361	0.87617311	0.9265248
ENSG0000027	AC004076.5	lncRNA	0.08279541	0.84892251	0.9100423
ENSG0000021	REPIN1	protein_codir	0.08275239	0.62722117	0.74869652
ENSG0000010	NSMCE4A	protein_codir	0.08258276	0.53524821	0.67560349
ENSG0000012	MYRF	protein_codir	0.08250209	0.90332195	0.94264019
ENSG0000016	LMBRD1	protein_codir	0.08237992	0.51533816	0.65856846
ENSG0000000	CYP26B1	protein_codir	0.08237761	0.81592972	0.88671961
ENSG0000022	ACVR2B-AS1	lncRNA	0.08236562	0.88227414	0.93026958
ENSG0000014	VPS53	protein_codir	0.08231204	0.36178777	0.51572882
ENSG0000018	HMGB1	protein_codir	0.08209217	0.52062133	0.66332508
ENSG0000019	MPZL1	protein_codir	0.08191994	0.62578528	0.74761838
ENSG0000010	KAT8	protein_codir	0.08183398	0.48865267	0.63563704
ENSG0000011	PHACTR2	protein_codir	0.08179002	0.70418965	0.80748703
ENSG0000017	DLGAP1-AS1	lncRNA	0.08175704	0.71424165	0.81467497
ENSG0000016	U2SURP	protein_codir	0.08173019	0.38511542	0.53835853
ENSG0000027	RP11-165D7.5	lncRNA	0.0816659	0.91842437	0.95174581
ENSG0000016	MEMO1	protein_codir	0.08104829	0.61251936	0.73736411
ENSG0000012	GPCPD1	protein_codir	0.08104744	0.72855233	0.8254416
ENSG0000024	CTC-241N9.1	lncRNA	0.08092829	0.80059773	0.8762635
ENSG0000016	LMBRD2	protein_codir	0.08090142	0.69586103	0.80072482

ENSG0000022	NUTM2E	protein_codir	0.08090036	0.79160712	0.87041011
ENSG000000€	SNRNP40	protein_codir	0.08088562	0.49471479	0.6407814
ENSG0000013	BUD13	protein_codir	0.08067954	0.36459355	0.5185414
ENSG0000018	BCOR	protein_codir	0.0806765	0.7422735	0.83552202
ENSG0000016	BLCAP	protein_codir	0.08063316	0.47803722	0.62593097
ENSG0000010	MFSD10	protein_codir	0.080566	0.64978064	0.76598128
ENSG0000011	CUTC	protein_codir	0.08011569	0.43084732	0.58230588
ENSG000000(	CDK11A	protein_codir	0.08010046	0.78845645	0.86796202
ENSG0000009	LAMB1	protein_codir	0.07980792	0.77580305	0.8593512
ENSG0000011	ALDH8A1	protein_codir	0.07975147	0.8463624	0.90846143
ENSG0000013	NXT1	protein_codir	0.0797048	0.76724669	0.85330194
ENSG0000013	NINJ1	protein_codir	0.07967583	0.60832433	0.73412496
ENSG0000014	WDR20	protein_codir	0.079614	0.33489033	0.48777467
ENSG0000010	PHEX	protein_codir	0.07937302	0.89350567	0.9365915
ENSG0000027	AC009120.11	lncRNA	0.07916005	0.92342725	0.95485233
ENSG0000012	ZNF484	protein_codir	0.07908596	0.63484348	0.75476654
ENSG0000018	CMC1	protein_codir	0.07904228	0.6483336	0.76469975
ENSG0000028	XXbac-BPG17	lncRNA	0.07893441	0.87562506	0.92626442
ENSG0000007	SENP1	protein_codir	0.07890206	0.61346983	0.7379434
ENSG0000015	UBC	protein_codir	0.07857761	0.7836944	0.86468419
ENSG0000010	TFPT	protein_codir	0.07856592	0.55289671	0.68937374
ENSG0000014	SEC11A	protein_codir	0.07848168	0.48971409	0.63656986
ENSG0000017	RBM4	protein_codir	0.07842091	0.4656267	0.61528129
ENSG0000018	S100A13	protein_codir	0.07841767	0.74587578	0.83765594
ENSG0000018	C15orf61	protein_codir	0.07836996	0.578789	0.71084318
ENSG000000€	SIRT2	protein_codir	0.07817283	0.35349855	0.50708543
ENSG0000010	SUPT20H	protein_codir	0.07813583	0.48676988	0.63399905
ENSG0000018	NPIPA1	protein_codir	0.07797697	0.72089112	0.81978808
ENSG0000008	ANAPC5	protein_codir	0.07796736	0.53179439	0.67281211
ENSG0000016	ZNF232	protein_codir	0.07790108	0.82679947	0.89525644
ENSG0000013	HERC3	protein_codir	0.07789809	0.65242512	0.76831167
ENSG0000010	ETHE1	protein_codir	0.07786137	0.40844099	0.56139041
ENSG0000016	CKB	protein_codir	0.07784583	0.91074302	0.94686714
ENSG0000019	ZNF841	protein_codir	0.07784241	0.70113748	0.80521485
ENSG0000011	GLE1	protein_codir	0.07783262	0.53577845	0.67596386
ENSG0000015	EYA3	protein_codir	0.07773794	0.56926368	0.703031
ENSG0000011	DARS2	protein_codir	0.07773555	0.66860493	0.78107128
ENSG0000027	CTD-3092A11	lncRNA	0.077495	0.80721674	0.88060737
ENSG0000014	ARMT1	protein_codir	0.07744175	0.63909984	0.75795172
ENSG0000013	GNS	protein_codir	0.07739976	0.70654227	0.80930193
ENSG0000024	ATP5MF	protein_codir	0.07739896	0.56210207	0.69682491
ENSG0000023	LINC00601	lncRNA	0.07732657	0.94875509	0.97014607
ENSG0000024	RP11-932O9.7	lncRNA	0.07719405	0.80422537	0.87863236
ENSG0000001	NUB1	protein_codir	0.07699562	0.60066256	0.72793207
ENSG0000027	RP11-295I5.3	lncRNA	0.07677657	0.8768404	0.92694653
ENSG0000013	EIF3G	protein_codir	0.07671687	0.52548861	0.66737151
ENSG0000010	CCNP	protein_codir	0.07661036	0.86872271	0.92210667

ENSG0000023	CT66	lncRNA	0.07646492	0.94352017	0.96698248
ENSG0000014	SCCPDH	protein_codir	0.07642301	0.62551487	0.74756951
ENSG0000011	HIPK3	protein_codir	0.07634741	0.74731676	0.83865142
ENSG0000010	LMF1	protein_codir	0.07630198	0.65351173	0.76906468
ENSG0000019	UCKL1	protein_codir	0.07600147	0.61548512	0.73940668
ENSG0000018	KIAA2026	protein_codir	0.07597917	0.56013153	0.69538084
ENSG0000018	ENTPD5	protein_codir	0.07584383	0.52744474	0.66905592
ENSG0000014	TCF25	protein_codir	0.07578441	0.64038191	0.75885311
ENSG0000018	TTC3	protein_codir	0.07576146	0.41927962	0.57187669
ENSG0000016	TRMT10B	protein_codir	0.07570155	0.67562881	0.78630194
ENSG0000026	ZNF236-DT	lncRNA	0.07568306	0.75712093	0.84624985
ENSG0000028	RP11-454P7.4	lncRNA	0.0755112	0.94921292	0.97039638
ENSG0000019	SULT1C4	protein_codir	0.07538331	0.8040788	0.87855437
ENSG0000006	IFT80	protein_codir	0.07528908	0.54063703	0.67957924
ENSG0000014	ZNF462	protein_codir	0.0750381	0.69081274	0.79712059
ENSG0000018	ZNF571	protein_codir	0.07481517	0.60165524	0.72868747
ENSG0000009	MZF1	protein_codir	0.07477699	0.78158016	0.86338752
ENSG0000017	NSUN3	protein_codir	0.07475665	0.62854545	0.74974845
ENSG0000024	C5orf34-AS1	lncRNA	0.07455831	0.9536142	0.97284569
ENSG0000012	POLR1B	protein_codir	0.07452071	0.7368103	0.83131947
ENSG0000024	BDNF-AS	lncRNA	0.07441739	0.69833905	0.80280215
ENSG0000016	LETM2	protein_codir	0.07434034	0.72980834	0.82618683
ENSG0000016	ZRSR2	protein_codir	0.07406113	0.76315988	0.85059021
ENSG0000013	DNAJC14	protein_codir	0.07400083	0.43107801	0.58245456
ENSG0000006	POLR1H	protein_codir	0.0739619	0.61298772	0.73762562
ENSG0000019	H2BC12	protein_codir	0.0739506	0.68156932	0.79059129
ENSG0000026	LINC02182	lncRNA	0.07384882	0.96452454	NA
ENSG0000017	CTNNBIP1	protein_codir	0.07383499	0.62683439	0.74843482
ENSG0000017	ZNF672	protein_codir	0.07361957	0.65963486	0.7739641
ENSG0000006	BTBD1	protein_codir	0.07337951	0.53254085	0.67347886
ENSG0000016	CALML6	protein_codir	0.07330119	0.92405728	0.95525347
ENSG0000013	SEPTIN11	protein_codir	0.07315059	0.72329536	0.82154484
ENSG0000014	GPC3	protein_codir	0.07304417	0.89858087	0.93967907
ENSG0000028	RP3-438D16.4	lncRNA	0.07302396	0.94952577	0.97046705
ENSG0000026	BEAN1-AS1	lncRNA	0.07285598	0.90331484	0.94264019
ENSG0000013	THOC6	protein_codir	0.07264671	0.75569957	0.84503733
ENSG0000014	PPRC1	protein_codir	0.07263863	0.81169376	0.88385499
ENSG0000014	INTS7	protein_codir	0.0725138	0.53559216	0.67592176
ENSG0000015	ZNF117	protein_codir	0.07247229	0.79960285	0.87559523
ENSG0000013	ITM2C	protein_codir	0.07245835	0.79147296	0.87031146
ENSG0000026	RP11-686D22	lncRNA	0.07243606	0.83780165	0.9026401
ENSG0000013	TAF4	protein_codir	0.07238694	0.57017961	0.70371155
ENSG0000024	RP11-521D12	lncRNA	0.07216021	0.9530726	0.97254461
ENSG0000027	RP11-397O8.7	lncRNA	0.07215936	0.90446389	0.94333105
ENSG0000010	RIOK3	protein_codir	0.07199485	0.35576074	0.50935261
ENSG0000013	SNX14	protein_codir	0.07192277	0.46347195	0.61337303
ENSG0000010	PABPN1	protein_codir	0.07190426	0.72363719	0.82169294

ENSG0000015	ST3GAL2	protein_codir	0.07184229	0.65258277	0.76846455
ENSG0000008	CADPS2	protein_codir	0.07178538	0.76819873	0.85400076
ENSG0000010	TM9SF1	protein_codir	0.07173546	0.5786258	0.71070595
ENSG0000010	XIAP	protein_codir	0.071729	0.37785858	0.53155702
ENSG0000017	TP53I11	protein_codir	0.07170576	0.71360002	0.81434014
ENSG0000027	CTD-2410N18	lncRNA	0.07165217	0.86963555	0.92264997
ENSG0000012	HAT1	protein_codir	0.07163075	0.55272453	0.68934673
ENSG0000011	BCL7A	protein_codir	0.0716257	0.84181237	0.90554829
ENSG0000017	EFCAB13	protein_codir	0.07161978	0.75327602	0.84305172
ENSG0000010	BFAR	protein_codir	0.07138476	0.35176657	0.50530639
ENSG0000014	CELSR2	protein_codir	0.07135343	0.77832184	0.86112999
ENSG0000010	HNRNPUL1	protein_codir	0.07130136	0.5376019	0.67736086
ENSG0000018	ERCC6L2	protein_codir	0.07128124	0.63195281	0.75241558
ENSG0000011	MED23	protein_codir	0.07125651	0.61420147	0.73844272
ENSG0000027	ATP6V1FNB	protein_codir	0.07122166	0.84160868	0.9054251
ENSG0000021	LINC02026	lncRNA	0.07102017	0.95313968	0.97257714
ENSG0000019	TRAPPC2	protein_codir	0.07094967	0.68527607	0.79329917
ENSG0000015	SRFBP1	protein_codir	0.07075882	0.71933178	0.8187227
ENSG0000026	CTD-3185P2.1	lncRNA	0.07068489	0.84079641	0.90477255
ENSG0000026	ZNF213-AS1	lncRNA	0.07065215	0.80601771	0.87979011
ENSG0000013	ACTR3B	protein_codir	0.07060004	0.660969	0.77520016
ENSG0000012	STEAP4	protein_codir	0.07051873	0.85371633	0.91285717
ENSG0000024	RP11-506M12	lncRNA	0.0705096	0.92074118	0.95315849
ENSG0000012	BMP4	protein_codir	0.0703489	0.81397568	0.88560657
ENSG0000010	DNAJC5	protein_codir	0.07028287	0.60907107	0.73473487
ENSG0000014	TCF12	protein_codir	0.07020736	0.65727997	0.77211947
ENSG0000012	PKN1	protein_codir	0.07019125	0.57280309	0.70584169
ENSG0000020	VARS1	protein_codir	0.0701761	0.67122188	0.78318378
ENSG0000015	TMBIM4	protein_codir	0.07015947	0.50108875	0.6461091
ENSG0000027	RP11-399C16.	lncRNA	0.07006349	0.94508153	0.96800237
ENSG0000017	ZFAND2A	protein_codir	0.069972	0.74024762	0.8339524
ENSG0000012	ICE2	protein_codir	0.06992467	0.53697281	0.67703775
ENSG0000024	ETV5	protein_codir	0.06986889	0.79001808	0.86912687
ENSG0000016	PTGER1	protein_codir	0.0696994	0.89580179	0.93798034
ENSG0000013	RELCH	protein_codir	0.06947713	0.63421109	0.75432393
ENSG0000017	ARV1	protein_codir	0.06934678	0.63544226	0.7552139
ENSG0000012	HOXA7	protein_codir	0.06915561	0.88794448	0.93348541
ENSG0000027	RP11-996F15.	lncRNA	0.06891629	0.79389596	0.87200376
ENSG0000013	PARP16	protein_codir	0.0688456	0.61287812	0.73760758
ENSG0000017	SEPHS2	protein_codir	0.06876522	0.65277821	0.76862916
ENSG0000026	CTC-459F4.3	lncRNA	0.06872519	0.7135488	0.81434014
ENSG0000016	GARRE1	protein_codir	0.06871884	0.6149701	0.73898082
ENSG0000019	ZNF830	protein_codir	0.0686849	0.67764361	0.78779848
ENSG0000026	LINC01415	lncRNA	0.06868466	0.87350158	0.92497464
ENSG0000020	RNPS1	protein_codir	0.06864852	0.48338029	0.63080386
ENSG0000013	DDC	protein_codir	0.06848532	0.95764986	0.97498209
ENSG0000018	JAKMIP3	protein_codir	0.06844874	0.8526468	0.91235989

ENSG0000013	GSTCD	protein_codir	0.0684116	0.61856858	0.74216799
ENSG0000024	AC069368.3	protein_codir	0.06829626	0.8892462	0.93416173
ENSG0000016	HIPK4	protein_codir	0.06825412	0.89747282	0.93908923
ENSG0000013	GCOM1	protein_codir	0.06823173	0.88544751	0.93202022
ENSG0000028	RP11-207D10	lncRNA	0.06806192	0.94575428	0.96849489
ENSG0000011	NUP155	protein_codir	0.06797458	0.67356385	0.784707
ENSG0000018	PAX8-AS1	lncRNA	0.06787719	0.90935427	0.94616514
ENSG0000028	RP11-233G1.8	lncRNA	0.06777963	0.96065843	0.97685685
ENSG0000027	RP11-817I4.1	lncRNA	0.06773381	0.78593649	0.86622017
ENSG0000026	ERVK13-1	lncRNA	0.06765343	0.81333767	0.88527866
ENSG0000014	EBAG9	protein_codir	0.06752932	0.51104155	0.65486535
ENSG0000007	FNDC3B	protein_codir	0.06747915	0.74973684	0.84051097
ENSG0000017	METTL7B	protein_codir	0.06747304	0.89844863	0.9396301
ENSG0000016	NXN	protein_codir	0.06728582	0.84304018	0.90626902
ENSG0000007	EXOSC5	protein_codir	0.0672707	0.72003318	0.81920049
ENSG0000013	ZNF740	protein_codir	0.06724821	0.60768684	0.73358
ENSG0000011	SAYSD1	protein_codir	0.06721865	0.51320179	0.65681079
ENSG0000018	EXD3	protein_codir	0.06718735	0.77892035	0.86165329
ENSG0000019	FAR1	protein_codir	0.06717921	0.59395877	0.7226376
ENSG0000015	FAM151B	protein_codir	0.06717805	0.7920875	0.87081387
ENSG0000017	TUFM	protein_codir	0.06708779	0.51844965	0.66143089
ENSG0000016	RIC3	protein_codir	0.06707769	0.86257731	0.91812345
ENSG0000010	PQBP1	protein_codir	0.06690901	0.6543008	0.76976564
ENSG0000017	CTD-2201E18.	lncRNA	0.06685705	0.77322445	0.85740963
ENSG0000021	CATSPERZ	protein_codir	0.06685314	0.93471802	0.96157798
ENSG0000022	RP11-269F19.	lncRNA	0.06679617	0.95067668	0.97106786
ENSG0000010	AP4S1	protein_codir	0.0667728	0.53167109	0.67271774
ENSG0000023	RP11-439A17	lncRNA	0.06665852	0.96863335	0.98160532
ENSG0000018	AP1S2	protein_codir	0.06662353	0.68371837	0.79218924
ENSG0000007	JMJD6	protein_codir	0.06649881	0.79054083	0.8695288
ENSG0000012	ATXN1	protein_codir	0.0664684	0.69880017	0.80316511
ENSG0000013	ANP32B	protein_codir	0.06646721	0.63479991	0.75476425
ENSG0000001	UBR7	protein_codir	0.06615652	0.53347085	0.6742228
ENSG0000010	MED13	protein_codir	0.06610531	0.75630913	0.84551357
ENSG0000018	SRPRA	protein_codir	0.0658411	0.63368338	0.75389107
ENSG0000017	KRCC1	protein_codir	0.06575973	0.58207702	0.7133588
ENSG0000018	CEP97	protein_codir	0.06560455	0.73003469	0.82634146
ENSG0000006	APPBP2	protein_codir	0.06558796	0.59728157	0.72545809
ENSG0000027	RP11-468E2.1	lncRNA	0.06556935	0.8272392	0.8953799
ENSG0000010	CHD8	protein_codir	0.06534675	0.55885665	0.69442238
ENSG0000013	DMTF1	protein_codir	0.06513954	0.77094715	0.85588489
ENSG0000021	TOMM6	protein_codir	0.06512923	0.58764383	0.71797875
ENSG0000005	ALX4	protein_codir	0.06499535	0.93150408	0.95953987
ENSG0000010	EIF3A	protein_codir	0.06496217	0.62327459	0.74578019
ENSG0000023	LINC00484	lncRNA	0.0649066	0.88941893	0.93419857
ENSG0000007	DAZAP1	protein_codir	0.06470082	0.66859215	0.78107128
ENSG0000017	RGMB	protein_codir	0.06465044	0.72418838	0.82198089

ENSG0000018	ZNF177	protein_codir	0.06442985	0.82849479	0.89635419
ENSG0000023	TRAF3IP2-AS1	lncRNA	0.06433599	0.70666066	0.80940397
ENSG0000027	CTD-2650P22	lncRNA	0.06430553	0.91855337	0.95180806
ENSG0000024	STIMATE-MUS	protein_codir	0.06430264	0.82711883	0.8953799
ENSG0000019	HTT	protein_codir	0.06430074	0.6501177	0.76634591
ENSG0000027	XXcos-LUCA1	protein_codir	0.06427938	0.98428281	NA
ENSG0000022	RP11-239E10.	lncRNA	0.06427938	0.98428281	NA
ENSG0000023	LMLN-AS1	lncRNA	0.06427938	0.98428281	NA
ENSG0000026	RP11-498C9.1	lncRNA	0.06427938	0.98428281	NA
ENSG0000017	TMEM270	protein_codir	0.06427938	0.98428281	NA
ENSG0000027	RP11-35J10.7	lncRNA	0.06427938	0.98428281	NA
ENSG0000016	LLCFC1	protein_codir	0.06427875	0.98304557	NA
ENSG0000028	RP11-512M8.	protein_codir	0.06427861	0.98270695	NA
ENSG0000014	S100A7	protein_codir	0.06427854	0.98256061	NA
ENSG0000028	CTD-3065B20	lncRNA	0.06427854	0.98255669	NA
ENSG0000023	TTLL1-AS1	lncRNA	0.06427851	0.98246935	NA
ENSG0000013	ANP32D	protein_codir	0.06427846	0.98235595	NA
ENSG0000028	CTC-205M6.5	lncRNA	0.06427845	0.98232065	NA
ENSG0000022	RP11-170M17	lncRNA	0.0642784	0.98219526	NA
ENSG0000025	RP11-50B3.4	lncRNA	0.06427838	0.98216018	NA
ENSG0000025	RP11-613D13	lncRNA	0.06427833	0.9820286	NA
ENSG0000025	RP11-434C1.2	lncRNA	0.06427833	0.98202477	NA
ENSG0000026	AF038458.3	lncRNA	0.06427831	0.9819642	NA
ENSG0000028	CTD-2159L10.	lncRNA	0.0642783	0.98193624	NA
ENSG0000013	ABCG5	protein_codir	0.06427825	0.98181068	NA
ENSG0000024	RP11-185E8.1	lncRNA	0.06427823	0.98174603	NA
ENSG0000022	RP11-128M1.	lncRNA	0.0642782	0.98165748	NA
ENSG0000025	RP11-103H7.2	lncRNA	0.0642781	0.98138018	NA
ENSG0000028	RP11-349F21.	lncRNA	0.06427808	0.9813202	NA
ENSG0000027	CTD-2012J19.	lncRNA	0.06427726	0.97825438	NA
ENSG0000022	FOCAD-AS1	lncRNA	0.06427723	0.97812397	NA
ENSG0000025	RP11-789C2.1	lncRNA	0.06427722	0.97807255	NA
ENSG0000023	RP11-107M16	lncRNA	0.0642772	0.97799531	NA
ENSG0000027	AC005618.9	lncRNA	0.06427717	0.97784661	NA
ENSG0000025	GSTCD-AS1	lncRNA	0.06427716	0.97779357	NA
ENSG0000018	TAS2R60	protein_codir	0.06427716	0.97778273	NA
ENSG0000022	LINC01690	lncRNA	0.06427714	0.97769025	NA
ENSG0000021	AC007277.3	lncRNA	0.06427714	0.97766517	NA
ENSG0000027	LINC02340	lncRNA	0.06427712	0.97760407	NA
ENSG0000026	CTD-2369P2.4	lncRNA	0.06427712	0.97760299	NA
ENSG0000025	LINC02421	lncRNA	0.06427712	0.97759495	NA
ENSG0000025	RP11-219J21.	lncRNA	0.06427712	0.97759469	NA
ENSG0000025	MTRNR2L10	protein_codir	0.06427712	0.9775883	NA
ENSG0000021	SPATA31D1	protein_codir	0.06427711	0.97752174	NA
ENSG0000021	IQCJ	protein_codir	0.06427709	0.97745702	NA
ENSG0000022	RP11-561I11.3	lncRNA	0.06427709	0.9774398	NA
ENSG0000023	TRIM31-AS1	lncRNA	0.06427709	0.97742376	NA

ENSG0000025	RP11-359K18.	lncRNA	0.06427709	0.97741849	NA
ENSG0000022	AC093382.1	lncRNA	0.06427707	0.97730163	NA
ENSG0000016	KLK6	protein_codir	0.06427704	0.97717993	NA
ENSG0000017	EXD1	protein_codir	0.06427704	0.97716711	NA
ENSG0000028	RP11-517I24.(	lncRNA	0.06427702	0.97704334	NA
ENSG0000024	LINC02056	lncRNA	0.06427699	0.97690839	NA
ENSG0000028	RP11-489G11	lncRNA	0.06427657	0.97405809	NA
ENSG0000027	RP11-33N14.5	lncRNA	0.06427651	0.97361275	NA
ENSG0000024	MEF2C-AS2	lncRNA	0.0642765	0.97355577	NA
ENSG0000013	NUTM2F	protein_codir	0.0642765	0.97354851	NA
ENSG0000028	RP11-1137N1	lncRNA	0.0642765	0.9735207	NA
ENSG0000028	RP11-446E24.	lncRNA	0.06427648	0.97334386	NA
ENSG0000023	MRPS9-AS1	lncRNA	0.06427647	0.9732257	NA
ENSG0000018	CSNK1A1L	protein_codir	0.06427646	0.9732077	NA
ENSG0000023	LINC02623	lncRNA	0.06427646	0.97319712	NA
ENSG0000025	SMAD1-AS1	lncRNA	0.06427646	0.97312741	NA
ENSG0000017	CATSPERD	protein_codir	0.06427645	0.97310448	NA
ENSG0000027	LHX1	protein_codir	0.06427643	0.97292458	NA
ENSG0000022	AC096558.1	lncRNA	0.06427641	0.97272098	NA
ENSG0000027	RP11-385J1.3	protein_codir	0.06427641	0.97268444	NA
ENSG0000026	CTC-482H14.5	lncRNA	0.06427639	0.97257529	NA
ENSG0000023	MCIDAS	protein_codir	0.06427607	0.96896534	NA
ENSG0000022	RP11-549L6.3	lncRNA	0.06427606	0.96880467	NA
ENSG0000022	GK3P	protein_codir	0.06427605	0.96865907	NA
ENSG0000017	LINC00303	lncRNA	0.06427605	0.96856121	NA
ENSG0000026	LINC01616	lncRNA	0.06427603	0.96831835	NA
ENSG0000016	SNTB2	protein_codir	0.0642482	0.72561874	0.82319846
ENSG0000012	SHLD2	protein_codir	0.06409344	0.57704738	0.70939817
ENSG0000028	CTD-2308N23	lncRNA	0.06389207	0.929458	0.9581469
ENSG0000008	YTHDC1	protein_codir	0.06386771	0.56911782	0.70293508
ENSG0000017	ZNF160	protein_codir	0.06379934	0.70986616	0.81149386
ENSG0000014	EIF3M	protein_codir	0.06359238	0.4913528	0.63807252
ENSG0000013	TERF2	protein_codir	0.06354952	0.57080787	0.7042252
ENSG0000000	FARP2	protein_codir	0.06354922	0.57348523	0.70633945
ENSG0000021	RNASEK	protein_codir	0.06350158	0.63136928	0.75194772
ENSG0000013	SBNO1	protein_codir	0.06348102	0.57455448	0.70721488
ENSG0000013	SPPL2A	protein_codir	0.06345138	0.66136711	0.77556828
ENSG0000007	MNT	protein_codir	0.06337956	0.62319759	0.74574829
ENSG0000018	TMPPE	protein_codir	0.06335909	0.80487422	0.87906307
ENSG0000019	MT-ND2	protein_codir	0.06329291	0.85494998	0.91324278
ENSG0000011	P3H1	protein_codir	0.06301038	0.75776731	0.84672143
ENSG0000016	FDPS	protein_codir	0.06298807	0.55955356	0.69497568
ENSG0000012	OPA3	protein_codir	0.06294113	0.49980408	0.64516468
ENSG0000011	CERT1	protein_codir	0.06292341	0.64264262	0.76061833
ENSG0000016	HDHD2	protein_codir	0.06286707	0.5077961	0.65228017
ENSG0000028	RP11-204M4.	lncRNA	0.06284843	0.87373775	0.92509736
ENSG0000026	CTC-277H1.7	lncRNA	0.06283135	0.92193051	0.95399179

ENSG0000019	ZNF84	protein_codir	0.06275355	0.71698904	0.81706636
ENSG0000019	KTI12	protein_codir	0.06268634	0.62411414	0.74652603
ENSG0000013	GNL2	protein_codir	0.0626188	0.69757868	0.80209496
ENSG0000011	YLPM1	protein_codir	0.06248459	0.52344988	0.66563931
ENSG0000009	ANGPT2	protein_codir	0.06245596	0.83985947	0.90418704
ENSG0000018	RGS7BP	protein_codir	0.06239694	0.89959151	0.94002412
ENSG0000012	CHURC1-FNTI	protein_codir	0.06227125	0.94368471	0.96701244
ENSG0000012	HNRNPH2	protein_codir	0.06224138	0.67576681	0.7863779
ENSG0000018	GRIN2A	protein_codir	0.06220465	0.90056651	0.94052192
ENSG0000025	RP11-20I23.3	protein_codir	0.06214044	0.97866573	0.98803814
ENSG0000025	RP11-234K19.	lncRNA	0.06189752	0.97096577	0.98331956
ENSG0000004	NOP16	protein_codir	0.0617183	0.83877139	0.90326662
ENSG0000025	MPV17L2	protein_codir	0.06171572	0.68082303	0.7902308
ENSG0000014	PFKL	protein_codir	0.0616102	0.60097616	0.72815238
ENSG0000017	MCMDC2	protein_codir	0.06148938	0.90021928	0.9403308
ENSG0000027	RP11-644N4.1	lncRNA	0.06141291	0.96902426	0.98192941
ENSG0000015	ANKAR	protein_codir	0.06140771	0.7633596	0.85066225
ENSG0000023	RASGRP3-AS1	lncRNA	0.06138121	0.93611716	0.96217461
ENSG0000028	RP11-531A21	lncRNA	0.06133204	0.8659979	0.9203456
ENSG0000014	KRBOX4	protein_codir	0.06129544	0.63727124	0.75679042
ENSG0000018	OR52H1	protein_codir	0.06121433	0.96751512	NA
ENSG0000002	BRD9	protein_codir	0.06119111	0.70967301	0.81148359
ENSG0000018	ZNF749	protein_codir	0.06118398	0.68460343	0.79271986
ENSG0000016	EEF2	protein_codir	0.06103145	0.67576261	0.7863779
ENSG0000014	ILF2	protein_codir	0.0609331	0.69226919	0.7980498
ENSG0000014	ZCCHC2	protein_codir	0.06091571	0.71657775	0.81666505
ENSG0000000	COX10	protein_codir	0.06089701	0.64824358	0.76469975
ENSG0000018	KRBA2	protein_codir	0.06085215	0.77187452	0.85643251
ENSG0000007	TTC38	protein_codir	0.06080234	0.54357045	0.68199399
ENSG0000013	RBMX2	protein_codir	0.06075333	0.67501437	0.78593276
ENSG0000012	SOCS2	protein_codir	0.06064941	0.84855496	0.90978953
ENSG0000028	RP3-451C13.1	lncRNA	0.06063133	0.89286681	0.93611377
ENSG0000011	ARTN	protein_codir	0.06062432	0.89690888	0.93874809
ENSG0000006	OTUD5	protein_codir	0.06055638	0.60912234	0.73473487
ENSG0000018	UBE2E2	protein_codir	0.06049377	0.71919865	0.81863864
ENSG0000026	CD2BP2-DT	lncRNA	0.06047534	0.86140161	0.91743772
ENSG0000028	RP11-732A19	lncRNA	0.06044873	0.91975562	0.9524821
ENSG0000012	ZMYM2	protein_codir	0.06033788	0.55474207	0.69086374
ENSG0000016	RCHY1	protein_codir	0.06031558	0.67385463	0.78491333
ENSG0000011	NUP107	protein_codir	0.06016424	0.53307446	0.67390366
ENSG0000013	MFSD9	protein_codir	0.0600699	0.66186403	0.77596278
ENSG0000015	C18orf25	protein_codir	0.05995357	0.5963801	0.72464996
ENSG0000025	ZFP91-CNTF	protein_codir	0.05990015	0.94324774	0.96689145
ENSG0000016	MTCL1	protein_codir	0.05969726	0.76569741	0.85231783
ENSG0000013	DOCK6	protein_codir	0.05961279	0.72662812	0.82389002
ENSG0000008	KIZ	protein_codir	0.05960697	0.74432636	0.83668935
ENSG0000013	EPO	protein_codir	0.05960174	0.96520602	0.97982176

ENSG0000010	USP10	protein_codir	0.05947415	0.56323953	0.69767135
ENSG0000012	INO80	protein_codir	0.05941204	0.59695508	0.72515721
ENSG0000014	ALDH9A1	protein_codir	0.0593857	0.69669691	0.80151485
ENSG0000017	ZNF24	protein_codir	0.05938168	0.66598416	0.77927283
ENSG0000005	FAM160A2	protein_codir	0.05936665	0.66896087	0.78132186
ENSG0000013	ANKRD16	protein_codir	0.05933403	0.72051551	0.81952964
ENSG0000016	SMARCAD1	protein_codir	0.05928198	0.73729733	0.83173306
ENSG0000010	DNAL4	protein_codir	0.05922952	0.62867356	0.74980412
ENSG0000028	RP11-208N14	lncRNA	0.05909402	0.93145308	0.95952312
ENSG0000017	ACOT4	protein_codir	0.0590187	0.828906	0.89669371
ENSG0000028	MATR3	protein_codir	0.05901452	0.85198989	0.91191478
ENSG0000016	MXRA8	protein_codir	0.0589638	0.8511316	0.91141995
ENSG0000017	MYADM	protein_codir	0.05893326	0.86812218	0.92182844
ENSG0000025	ZNF225	protein_codir	0.05892085	0.8110435	0.88337591
ENSG0000015	TMEM18	protein_codir	0.05885068	0.54668021	0.68446543
ENSG0000019	MAP3K3	protein_codir	0.05881249	0.62999556	0.75083168
ENSG0000018	BLOC1S4	protein_codir	0.05875534	0.68163885	0.79060514
ENSG0000013	URB2	protein_codir	0.05873495	0.78885473	0.86822639
ENSG0000024	RP11-440L14.	lncRNA	0.0586291	0.85003357	0.91070315
ENSG0000014	ADAM15	protein_codir	0.05847328	0.76171487	0.84957752
ENSG0000002	TBPL1	protein_codir	0.05828289	0.55037624	0.6874414
ENSG0000017	SLFN12	protein_codir	0.05821493	0.80683134	0.88046944
ENSG0000010	PLEKHA8	protein_codir	0.05820463	0.68592866	0.79378755
ENSG0000010	RAE1	protein_codir	0.05814204	0.68908348	0.79587222
ENSG0000007	CDC42	protein_codir	0.05808156	0.66890796	0.78132186
ENSG0000022	RP11-342M1.	lncRNA	0.05793051	0.75666367	0.84585034
ENSG0000012	GNG11	protein_codir	0.05791509	0.83280011	0.89928607
ENSG0000024	N4BP2L2	protein_codir	0.05772184	0.76418758	0.85115174
ENSG0000027	RP11-156E8.1	lncRNA	0.05769975	0.87890016	0.92809384
ENSG0000017	NOC3L	protein_codir	0.05766658	0.63659577	0.75630281
ENSG0000017	SYT12	protein_codir	0.05766518	0.9157409	0.94999863
ENSG0000017	ARIH2	protein_codir	0.05765767	0.534662	0.67514124
ENSG0000013	MED6	protein_codir	0.05756928	0.54334291	0.68180142
ENSG0000016	SMAD3	protein_codir	0.05753483	0.7079983	0.81049915
ENSG0000016	NOL6	protein_codir	0.05741989	0.70540323	0.80843308
ENSG0000016	MFSD8	protein_codir	0.05734534	0.69867872	0.80312578
ENSG0000011	CRIPT	protein_codir	0.05731292	0.59075289	0.72022274
ENSG0000026	RP11-304L19.	lncRNA	0.05723098	0.90704774	0.94467038
ENSG0000011	MPV17	protein_codir	0.05697382	0.67477798	0.78572377
ENSG0000026	CTD-2323K18	lncRNA	0.05684136	0.90808171	0.9453195
ENSG0000001	MAP4K5	protein_codir	0.05673998	0.75030853	0.84074649
ENSG0000016	FNTA	protein_codir	0.05664229	0.47694565	0.62503506
ENSG0000016	SNIP1	protein_codir	0.05659884	0.63719814	0.75676869
ENSG0000014	SEC16A	protein_codir	0.05655284	0.65412941	0.76966225
ENSG0000013	KIAA0513	protein_codir	0.05651273	0.71667499	0.81674217
ENSG0000012	NT5C	protein_codir	0.05649675	0.78543219	0.86587519
ENSG0000016	GLOD4	protein_codir	0.05642457	0.54975329	0.68700507

ENSG0000015	DYNLT5	protein_codir	0.05640916	0.90619448	0.94421027
ENSG0000014	VPS54	protein_codir	0.05626019	0.62331641	0.74579793
ENSG0000018	NIPSNAP1	protein_codir	0.05622433	0.71019355	0.81173374
ENSG0000010	YWHAE	protein_codir	0.05615866	0.6208368	0.74405591
ENSG0000013	COG3	protein_codir	0.0560689	0.3861579	0.53918817
ENSG0000014	IL17RD	protein_codir	0.05598765	0.90741425	0.94487395
ENSG0000016	CTD-2369P2.1	protein_codir	0.05588988	0.88152723	0.9298015
ENSG0000012	RBM48	protein_codir	0.0557589	0.78936671	0.86865709
ENSG0000022	PAPPA-AS2	lncRNA	0.05568391	0.963605	NA
ENSG0000027	TBC1D3H	protein_codir	0.05560054	0.9857378	0.99292772
ENSG0000026	RP11-152P23.	lncRNA	0.05554971	0.94903788	0.97032238
ENSG0000001	CAPN1	protein_codir	0.05549959	0.53665804	0.67676445
ENSG0000011	STX12	protein_codir	0.05542756	0.71260584	0.81378373
ENSG0000009	WASHC2A	protein_codir	0.05540752	0.62684803	0.74843482
ENSG0000016	POLL	protein_codir	0.05526099	0.69445775	0.79973465
ENSG0000007	EIF4G3	protein_codir	0.05520329	0.5945737	0.72309126
ENSG0000010	DKKL1	protein_codir	0.05488631	0.92593449	0.95626348
ENSG0000010	TASOR2	protein_codir	0.0548723	0.67954556	0.78937833
ENSG0000008	DNAJA1	protein_codir	0.05465054	0.80102466	0.87655709
ENSG0000026	LINC00662	lncRNA	0.05453349	0.79695557	0.87382745
ENSG0000021	MIF-AS1	lncRNA	0.05443059	0.9188844	0.95200244
ENSG0000017	ALK	protein_codir	0.05431556	0.9274875	0.95696805
ENSG0000023	KDM4A-AS1	lncRNA	0.05428909	0.92527749	0.95583657
ENSG0000012	CDK2	protein_codir	0.05428291	0.73585719	0.83068523
ENSG0000016	RPP25L	protein_codir	0.05422477	0.81560373	0.88643509
ENSG0000014	TXNL4B	protein_codir	0.05420272	0.75121057	0.84147411
ENSG0000021	UBXN2B	protein_codir	0.05402318	0.7650071	0.85189276
ENSG0000019	FAM118B	protein_codir	0.05399862	0.61393407	0.73824977
ENSG0000010	TBL2	protein_codir	0.0539484	0.64861632	0.76480459
ENSG0000024	SBF2-AS1	lncRNA	0.05392453	0.77619657	0.85957056
ENSG0000011	THADA	protein_codir	0.0536705	0.64825329	0.76469975
ENSG0000009	GPATCH2	protein_codir	0.05364478	0.73029063	0.82650811
ENSG0000016	IQUB	protein_codir	0.053487	0.8868036	0.93290861
ENSG0000012	NAPB	protein_codir	0.05329278	0.74646741	0.83800471
ENSG0000022	FAM66E	lncRNA	0.05320035	0.95612038	0.97410715
ENSG0000022	SGMS1-AS1	lncRNA	0.05319475	0.75559813	0.84499232
ENSG0000014	ATG3	protein_codir	0.05307746	0.60272308	0.72969097
ENSG0000003	NSUN2	protein_codir	0.0530456	0.60021815	0.72762803
ENSG0000014	INPP5E	protein_codir	0.05303782	0.73982857	0.83363313
ENSG0000026	RP11-106M3.	protein_codir	0.05303551	0.89651581	0.93847241
ENSG0000016	NEPRO	protein_codir	0.05302518	0.54263297	0.68125103
ENSG0000012	ATG101	protein_codir	0.05300594	0.80223114	0.87749492
ENSG0000011	BCL6	protein_codir	0.05281455	0.85362077	0.91285717
ENSG0000028	RP11-555E18.	protein_codir	0.05281178	0.74967759	0.84051097
ENSG0000016	IFT122	protein_codir	0.05272519	0.75320424	0.84303202
ENSG0000011	SLC11A2	protein_codir	0.05264915	0.69691011	0.80171483
ENSG0000001	ELOA	protein_codir	0.05255822	0.70323045	0.80671284

ENSG0000012	LPGAT1	protein_codir	0.05244408	0.76560966	0.85230526
ENSG0000021	ZNF407	protein_codir	0.05235135	0.70831282	0.81057795
ENSG0000018	C5orf47	protein_codir	0.05229235	0.96922993	0.98202974
ENSG0000012	MTERF1	protein_codir	0.05222699	0.79984721	0.87574536
ENSG0000007	FGF10	protein_codir	0.0518238	0.89352592	0.9365915
ENSG0000016	C1orf52	protein_codir	0.05160747	0.60088189	0.72810203
ENSG0000013	PELI2	protein_codir	0.05158809	0.84323068	0.90639867
ENSG0000017	LYSMD3	protein_codir	0.0515787	0.76883433	0.85446661
ENSG0000013	PNPT1	protein_codir	0.05155507	0.67760833	0.78779063
ENSG0000017	KNDC1	protein_codir	0.05155128	0.93074193	0.95904093
ENSG0000022	ZNF688	protein_codir	0.05150252	0.78294703	0.86427976
ENSG0000019	MAML3	protein_codir	0.05147951	0.79976601	0.8757349
ENSG0000027	CH507-9B2.5	protein_codir	0.05145086	0.82765995	0.89573167
ENSG0000010	KPNB1	protein_codir	0.0513156	0.64620851	0.76354265
ENSG0000017	GPR27	protein_codir	0.05114503	0.88178655	0.92993297
ENSG0000012	Aug-03	protein_codir	0.05092479	0.69066198	0.79699536
ENSG0000011	SNX4	protein_codir	0.05092018	0.6137105	0.73810947
ENSG0000008	DIS3	protein_codir	0.05088365	0.68043489	0.78994626
ENSG0000011	PPP6R3	protein_codir	0.05085933	0.51034898	0.65434216
ENSG0000019	GPATCH3	protein_codir	0.05069695	0.63240904	0.75282897
ENSG0000025	RP11-497E19.	lncRNA	0.05067677	0.95329501	0.97266377
ENSG0000007	PIK3C3	protein_codir	0.05066967	0.65118366	0.76714406
ENSG0000023	RP11-342D11	lncRNA	0.05061559	0.90728071	0.94477051
ENSG0000016	TMUB2	protein_codir	0.05046316	0.68911038	0.79587222
ENSG0000013	FBXO44	protein_codir	0.05043868	0.84909373	0.91019052
ENSG0000016	FTH1	protein_codir	0.0503153	0.74512252	0.83724356
ENSG0000019	RUNDC1	protein_codir	0.05027876	0.63001843	0.75083168
ENSG0000011	VPS4B	protein_codir	0.05020319	0.72248226	0.82092151
ENSG0000016	POLR3K	protein_codir	0.05018859	0.67650656	0.78690719
ENSG0000018	PRG2	protein_codir	0.05015066	0.94925871	0.97039638
ENSG0000011	MAN2A1	protein_codir	0.05003426	0.80340767	0.87816458
ENSG0000019	ENTPD6	protein_codir	0.05001397	0.74110101	0.83459867
ENSG0000013	ANKRD17	protein_codir	0.04997425	0.61457039	0.73866119
ENSG0000012	KHDRBS1	protein_codir	0.04971189	0.68256407	0.7913205
ENSG0000018	SLC26A11	protein_codir	0.0497116	0.72024816	0.81932678
ENSG0000026	AC003002.6	protein_codir	0.04967346	0.92747707	0.95696805
ENSG0000027	GAS2L2	protein_codir	0.0495794	0.95448924	0.97320388
ENSG0000024	ARHGAP31-A:	lncRNA	0.04957926	0.92877693	0.95767553
ENSG0000018	USP7	protein_codir	0.04954035	0.62422852	0.74659818
ENSG0000013	STK26	protein_codir	0.04947455	0.8559508	0.91388771
ENSG0000012	PRDX5	protein_codir	0.04939368	0.59257463	0.72138241
ENSG0000013	MBNL2	protein_codir	0.0492314	0.82072436	0.89024886
ENSG0000000	SNX11	protein_codir	0.04919067	0.6586498	0.77321347
ENSG0000018	POLR2A	protein_codir	0.04913531	0.64832701	0.76469975
ENSG0000027	NUDT18	protein_codir	0.04904089	0.83639398	0.90162901
ENSG0000021	MCM3AP-AS1	lncRNA	0.04900864	0.88343015	0.93102644
ENSG0000025	FAM66D	lncRNA	0.04898065	0.88955733	0.93423983

ENSG0000028	RP11-6G22.1	lncRNA	0.04892629	0.8606047	0.91687182
ENSG0000019	SND1	protein_codir	0.04866515	0.75383517	0.84346475
ENSG0000012	ATP5F1E	protein_codir	0.04847856	0.73779605	0.83215971
ENSG0000016	LARP4	protein_codir	0.04828923	0.75791616	0.84673049
ENSG0000021	SUPT4H1	protein_codir	0.04828019	0.4247454	0.57668328
ENSG0000023	LYRM9	protein_codir	0.04808011	0.79579811	0.87312083
ENSG0000015	ZNF18	protein_codir	0.04804607	0.76931913	0.85476465
ENSG0000014	CNGA3	protein_codir	0.04787638	0.96139407	0.97742497
ENSG0000011	CNOT2	protein_codir	0.04784461	0.64721326	0.76416225
ENSG0000001	ZNF207	protein_codir	0.04773312	0.65657172	0.77176146
ENSG0000021	CLIC1	protein_codir	0.04765768	0.76649357	0.85281488
ENSG0000019	AFAP1	protein_codir	0.04748771	0.84488921	0.90740911
ENSG0000012	IQSEC2	protein_codir	0.04742545	0.73461257	0.82975421
ENSG0000010	MICU1	protein_codir	0.04714724	0.78025644	0.86264842
ENSG0000026	AKAP1-DT	lncRNA	0.04705431	0.88363812	0.93115014
ENSG0000018	DDX41	protein_codir	0.04704238	0.63178962	0.75228614
ENSG0000028	RP5-934G17.7	lncRNA	0.04702122	0.95063669	0.97106348
ENSG0000016	LMNA	protein_codir	0.04689786	0.90972957	0.94632476
ENSG0000026	CTB-131K11.1	lncRNA	0.04685982	0.89485494	0.93733508
ENSG0000027	RP3-394A18.1	lncRNA	0.04679428	0.92193877	0.95399179
ENSG0000010	CIAO3	protein_codir	0.04663653	0.84837731	0.90970778
ENSG0000023	LINC00607	lncRNA	0.04662149	0.89050856	0.93474124
ENSG0000018	NHEJ1	protein_codir	0.04655914	0.66790899	0.78061038
ENSG0000027	ZNF8	protein_codir	0.04655435	0.74210562	0.83541704
ENSG0000010	CTCF	protein_codir	0.0464724	0.65875197	0.773288
ENSG0000014	TMC2	protein_codir	0.04637228	0.93186466	0.95973234
ENSG0000025	ZNF350	protein_codir	0.04634171	0.78045156	0.86272601
ENSG0000025	MC1R	protein_codir	0.04624013	0.88545296	0.93202022
ENSG0000022	LINC00863	lncRNA	0.04620701	0.85293891	0.91247086
ENSG0000025	GMDS-DT	lncRNA	0.0461872	0.83718142	0.90228863
ENSG0000021	ZC3H11B	protein_codir	0.04606702	0.89384724	0.93678609
ENSG0000013	PIWIL4	protein_codir	0.04598825	0.79987068	0.87574536
ENSG0000027	RP11-513M16	lncRNA	0.04596431	0.88103395	0.92938768
ENSG0000019	PDXDC2P-NPI	lncRNA	0.04595357	0.88514873	0.93187757
ENSG0000017	TADA2B	protein_codir	0.04590302	0.72870279	0.82551049
ENSG0000017	FARSA	protein_codir	0.04573278	0.68374364	0.79218924
ENSG0000028	RP11-400F1.2	lncRNA	0.04554832	0.96629278	0.98038447
ENSG0000000	SEC62	protein_codir	0.04553803	0.67109114	0.78310369
ENSG0000016	NXF1	protein_codir	0.0455305	0.8325141	0.89915909
ENSG0000013	PTGFRN	protein_codir	0.04539415	0.91064306	0.94686714
ENSG0000007	DERL2	protein_codir	0.04537685	0.59142909	0.72063136
ENSG0000018	PAQR9	protein_codir	0.04532413	0.96242861	0.97815272
ENSG0000024	RP3-508I15.1	lncRNA	0.04531645	0.94597216	0.9685386
ENSG0000014	YTHDF1	protein_codir	0.04526321	0.46207853	0.61220713
ENSG0000009	TRMT2A	protein_codir	0.04517067	0.8051488	0.87929341
ENSG0000015	RPP38	protein_codir	0.04510326	0.69789469	0.80239151
ENSG0000015	SLC45A3	protein_codir	0.04497372	0.87964571	0.92866809

ENSG0000016	E2F6	protein_codir	0.0449286	0.76018767	0.848373
ENSG0000014	ZSCAN29	protein_codir	0.04485374	0.72676506	0.82392362
ENSG0000010	MTMR3	protein_codir	0.04474177	0.60920029	0.73473873
ENSG0000023	HOXB-AS3	lncRNA	0.04450268	0.95585346	0.97399761
ENSG0000027	ARF4-AS1	lncRNA	0.04441312	0.89699444	0.93876649
ENSG0000010	AGR2	protein_codir	0.04440588	0.97398464	0.98521419
ENSG0000016	MARF1	protein_codir	0.04438233	0.74394531	0.83645679
ENSG0000027	RP4-621B10.8	lncRNA	0.04430325	0.94710203	0.969317
ENSG0000024	TMEM199	protein_codir	0.04422669	0.65268007	0.76854637
ENSG0000018	CCDC137	protein_codir	0.04419308	0.82587771	0.89450383
ENSG0000016	ZFPM2	protein_codir	0.04416116	0.89469732	0.93725024
ENSG0000014	SEMA6C	protein_codir	0.04415776	0.86079699	0.9170393
ENSG0000010	CSPP1	protein_codir	0.04398455	0.79798973	0.87441388
ENSG0000025	ZNF432	protein_codir	0.04395083	0.7587351	0.84727356
ENSG0000026	RP11-189E14.	lncRNA	0.04393101	0.97531971	0.98604274
ENSG0000014	PTGR2	protein_codir	0.04391708	0.83053229	0.89796061
ENSG0000011	ELL2	protein_codir	0.0435384	0.89024638	0.93462849
ENSG0000010	RAB5IF	protein_codir	0.04351017	0.72791684	0.82495848
ENSG0000008	C5orf22	protein_codir	0.04329319	0.77654656	0.85980336
ENSG0000014	LACTB2	protein_codir	0.04329291	0.76665648	0.85290418
ENSG0000011	RTKN	protein_codir	0.04321341	0.83230986	0.89903787
ENSG0000028	RP11-889I17.:	protein_codir	0.04317462	0.97643471	NA
ENSG0000014	COMMD10	protein_codir	0.043169	0.72182461	0.82041051
ENSG0000018	ZNF829	protein_codir	0.04313791	0.80043426	0.876244
ENSG0000008	CNOT3	protein_codir	0.04309204	0.72272265	0.82105954
ENSG0000014	RBAK	protein_codir	0.04303814	0.73192146	0.82766307
ENSG0000023	LINC01695	lncRNA	0.04301034	0.94766364	0.96964259
ENSG0000025	DNAAF4	protein_codir	0.04296586	0.91352163	0.94883678
ENSG0000027	MRPL20-DT	lncRNA	0.04281377	0.91592361	0.95001426
ENSG0000014	RARS2	protein_codir	0.0426992	0.65291077	0.76871984
ENSG0000016	IQCC	protein_codir	0.04266706	0.86408153	0.91911928
ENSG0000010	FZR1	protein_codir	0.04264154	0.67564389	0.78630194
ENSG0000023	WBP1	protein_codir	0.04261103	0.78301494	0.86427976
ENSG0000018	LINC02878	lncRNA	0.04249599	0.92071602	0.95315849
ENSG0000018	EWSR1	protein_codir	0.04247945	0.77266245	0.8569969
ENSG0000016	ZMYM6	protein_codir	0.04238138	0.80035184	0.87620269
ENSG0000018	ZNF669	protein_codir	0.0421009	0.88545136	0.93202022
ENSG0000011	LAMTOR2	protein_codir	0.04208451	0.75986232	0.84811272
ENSG0000014	CIAO1	protein_codir	0.04206741	0.61436407	0.73851363
ENSG0000016	TTC14	protein_codir	0.04201347	0.85730105	0.91475872
ENSG0000013	SMARCAL1	protein_codir	0.04194194	0.67111022	0.78310369
ENSG0000000	KDM1A	protein_codir	0.0418714	0.61887502	0.74241349
ENSG0000025	RP11-404E16.	lncRNA	0.04185734	0.95582221	0.97399761
ENSG0000014	POLE3	protein_codir	0.04176746	0.68166079	0.79060514
ENSG0000002	SLC39A9	protein_codir	0.04176188	0.77528598	0.85894099
ENSG0000012	CDKN2C	protein_codir	0.04169034	0.8195899	0.8893499
ENSG0000016	MAML1	protein_codir	0.04155043	0.68479966	0.79286568

ENSG0000017	CCDC168	protein_codir	0.04153772	0.93273741	0.96041631
ENSG000004	PHKA2	protein_codir	0.04147407	0.79892813	0.87502557
ENSG0000014	PPOX	protein_codir	0.04138519	0.84934607	0.91035502
ENSG0000010	WDR59	protein_codir	0.04135875	0.78455611	0.86525474
ENSG000000	OSBPL7	protein_codir	0.04134547	0.86648845	0.92065439
ENSG0000016	PMPCA	protein_codir	0.04134298	0.73069689	0.82674407
ENSG0000017	ZBTB42	protein_codir	0.04126401	0.82244373	0.89176366
ENSG0000018	CHM	protein_codir	0.04103139	0.82008951	0.88973494
ENSG0000016	ATP1A1	protein_codir	0.0410009	0.83609442	0.90150063
ENSG0000012	GTF2F1	protein_codir	0.04098488	0.64018913	0.75875488
ENSG0000016	TM2D2	protein_codir	0.04075556	0.81498782	0.8861026
ENSG0000007	PDE8A	protein_codir	0.04073118	0.74342793	0.83615829
ENSG0000018	PRMT3	protein_codir	0.04070889	0.7859181	0.86622017
ENSG0000014	TACC1	protein_codir	0.04064899	0.83847633	0.90316843
ENSG0000016	P2RY1	protein_codir	0.04032285	0.91104471	0.9470476
ENSG0000016	NEU3	protein_codir	0.0402964	0.87173153	0.92380865
ENSG0000021	QTRT1	protein_codir	0.04027225	0.8718321	0.92387978
ENSG0000013	UBE2D2	protein_codir	0.04022128	0.74624358	0.83788406
ENSG0000028	RP11-628A4.1	lncRNA	0.040097	0.98621917	NA
ENSG000000	TMEM132A	protein_codir	0.04006814	0.89980712	0.94012348
ENSG0000015	RNF111	protein_codir	0.04005051	0.78723334	0.86717825
ENSG0000026	RP11-401O9.3	lncRNA	0.04002221	0.96553764	0.97993203
ENSG0000010	NOP56	protein_codir	0.03987392	0.76915153	0.85464719
ENSG0000008	PCDHA6	protein_codir	0.03978325	0.91573415	0.94999863
ENSG0000007	MAP3K13	protein_codir	0.03977986	0.74891841	0.83980051
ENSG0000010	YY1	protein_codir	0.03968006	0.70849926	0.81064675
ENSG0000012	ZRANB3	protein_codir	0.03947567	0.83207826	0.898858
ENSG0000020	MIR29B2CHG	lncRNA	0.03940146	0.92076094	0.95315849
ENSG0000026	CTD-2521M24	protein_codir	0.03936752	0.98391536	NA
ENSG0000009	IFT74	protein_codir	0.03932685	0.73902585	0.83324012
ENSG0000023	LINC00412	lncRNA	0.03929105	0.97895686	NA
ENSG0000028	CH17-264B6.5	lncRNA	0.03926876	0.93636779	0.96225317
ENSG0000017	PCBP1-AS1	lncRNA	0.03925466	0.81668538	0.88726154
ENSG0000017	LPCAT4	protein_codir	0.03907382	0.82985148	0.89736401
ENSG0000021	AC110619.2	lncRNA	0.03903077	0.96233877	0.97811116
ENSG0000015	VPS26C	protein_codir	0.03902724	0.73629208	0.83094207
ENSG0000027	NUDT3	protein_codir	0.03884635	0.78311009	0.86431641
ENSG0000022	AC073636.1	lncRNA	0.03883462	0.97559776	0.98606534
ENSG0000026	LINC00907	lncRNA	0.03879079	0.9684493	0.98154462
ENSG0000018	HUS1B	protein_codir	0.03875662	0.94352563	0.96698248
ENSG0000010	NUBP1	protein_codir	0.03869334	0.74376391	0.83629536
ENSG0000021	CPNE1	protein_codir	0.03861978	0.84752739	0.90915075
ENSG0000010	BET1	protein_codir	0.03861248	0.78763432	0.86743635
ENSG0000016	PM20D1	protein_codir	0.03852491	0.95211276	0.97192425
ENSG0000026	RP11-271M24	lncRNA	0.03847673	0.97026174	0.98275072
ENSG0000023	PPP1R2B	protein_codir	0.03847399	0.92871632	0.95767553
ENSG0000023	XXbac-B476C2	lncRNA	0.03840484	0.97316316	0.98473573

ENSG0000027	JMJD1C-AS1	lncRNA	0.03834412	0.86743572	0.92133933
ENSG0000012	PSMB2	protein_codir	0.03824838	0.73074957	0.82674407
ENSG0000013	LSM4	protein_codir	0.03801497	0.79357843	0.87179366
ENSG0000014	DEF8	protein_codir	0.03798663	0.85382882	0.91285717
ENSG0000013	EXOSC2	protein_codir	0.03796321	0.77169848	0.85639473
ENSG0000005	DNAJC25	protein_codir	0.03795714	0.78300555	0.86427976
ENSG0000002	CD44	protein_codir	0.03787527	0.87841973	0.92776278
ENSG0000016	AFAP1L2	protein_codir	0.03785037	0.82642523	0.89499156
ENSG0000011	LDAH	protein_codir	0.03784557	0.76912811	0.85464719
ENSG0000014	COPS3	protein_codir	0.0377556	0.73162741	0.8274322
ENSG0000025	SHLD3	protein_codir	0.03772184	0.86428586	0.91912155
ENSG0000010	KLF8	protein_codir	0.03763133	0.85329289	0.91274327
ENSG0000014	RIT1	protein_codir	0.03752004	0.74681079	0.83825335
ENSG0000023	RP11-334A14	lncRNA	0.03739248	0.96736913	0.98085174
ENSG0000018	KTN1-AS1	lncRNA	0.03730161	0.87375144	0.92509736
ENSG0000027	RP11-2E11.9	lncRNA	0.03729793	0.94137957	0.96557129
ENSG0000010	SEC61B	protein_codir	0.03722563	0.8150941	0.8861026
ENSG0000014	LYPLAL1	protein_codir	0.03715875	0.84321734	0.90639867
ENSG0000018	EIF4EBP1	protein_codir	0.03711341	0.87841456	0.92776278
ENSG0000018	MTA1	protein_codir	0.03705326	0.77053578	0.85556576
ENSG0000016	PROSER3	protein_codir	0.03695457	0.79697346	0.87382745
ENSG0000017	THAP2	protein_codir	0.03689722	0.87787954	0.9274965
ENSG0000017	ZNF114	protein_codir	0.03684905	0.94595763	0.9685386
ENSG0000015	KAT6B	protein_codir	0.03682475	0.80946174	0.88225931
ENSG0000004	PSMA4	protein_codir	0.03678275	0.76399954	0.85104527
ENSG0000011	PECR	protein_codir	0.03677777	0.86449938	0.91926158
ENSG0000015	ARL14EP	protein_codir	0.03660698	0.81722124	0.88766913
ENSG0000005	KCNH2	protein_codir	0.03651668	NA	NA
ENSG0000025	RP4-607I7.1	lncRNA	0.03636026	0.94433656	0.96749048
ENSG0000016	PRELID1	protein_codir	0.03627756	0.83116695	0.89822487
ENSG0000013	C1RL	protein_codir	0.03616967	0.82789504	0.89590666
ENSG0000014	MCPH1	protein_codir	0.0360911	0.7499787	0.84061406
ENSG0000022	AC079305.8	lncRNA	0.03591946	0.93597828	0.96217461
ENSG0000010	SYTL4	protein_codir	0.03586453	0.91971884	0.95247973
ENSG0000014	TBC1D7	protein_codir	0.03585275	0.79788351	0.87433218
ENSG0000027	LA16c-358B7.	lncRNA	0.03575152	0.96064248	0.97685685
ENSG0000001	LARS2	protein_codir	0.03565999	0.8154137	0.88626344
ENSG0000024	PEDS1	protein_codir	0.03565403	0.74134683	0.83473296
ENSG0000008	AACS	protein_codir	0.03564436	0.80412218	0.87855437
ENSG0000012	GTF3C4	protein_codir	0.03562053	0.85487308	0.91319595
ENSG0000005	TRAF3IP2	protein_codir	0.03561787	0.86232887	0.91796514
ENSG0000012	SIL1	protein_codir	0.03555188	0.79139885	0.87026461
ENSG0000011	PPP2CA	protein_codir	0.03554543	0.81894867	0.8888813
ENSG0000013	CEP20	protein_codir	0.03551485	0.77632913	0.85961396
ENSG0000027	URGCP-MRPS	protein_codir	0.03541824	0.96822379	0.98147837
ENSG0000009	MAEA	protein_codir	0.03538596	0.77015348	0.85541638
ENSG0000014	TATDN1	protein_codir	0.03536566	0.81097332	0.88337591

ENSG0000019	SPATS2L	protein_codir	0.03530306	0.84005367	0.90432562
ENSG0000025	RP11-478C19.	lncRNA	0.03527403	0.95929789	0.97609759
ENSG0000026	LINC01686	lncRNA	0.03527054	0.8994009	0.93989606
ENSG0000019	CNOT7	protein_codir	0.03525053	0.75819913	0.84690662
ENSG0000016	PPP1R32	protein_codir	0.0351651	0.90836833	0.94540601
ENSG0000018	CENPP	protein_codir	0.03511275	0.79873297	0.87488121
ENSG0000024	RP11-1398P2.	lncRNA	0.0349814	0.91385829	0.94906208
ENSG0000018	MEX3D	protein_codir	0.03497325	0.87254594	0.92431706
ENSG0000021	ERV3-1	protein_codir	0.03496871	0.84666243	0.90864222
ENSG0000002	SNX1	protein_codir	0.03492609	0.82988302	0.89736401
ENSG0000010	MIEF1	protein_codir	0.03464456	0.82237153	0.89172038
ENSG0000013	VPS33A	protein_codir	0.03453585	0.83595815	0.90142751
ENSG0000017	TRAPPC1	protein_codir	0.03444354	0.82919568	0.89690169
ENSG0000013	REV1	protein_codir	0.03442302	0.77012578	0.85541638
ENSG0000016	RPUSD4	protein_codir	0.03432151	0.78474557	0.86532548
ENSG0000016	NEK10	protein_codir	0.03429906	0.90521314	0.94371842
ENSG0000025	MALAT1	lncRNA	0.03419043	0.93669831	0.96241419
ENSG0000018	ZFP82	protein_codir	0.03416165	0.88388249	0.93115014
ENSG0000025	RP11-140L24.	protein_codir	0.03415118	0.98039739	0.98924399
ENSG0000014	RSPO2	protein_codir	0.03409914	0.95966026	0.9762372
ENSG0000011	ABCC5	protein_codir	0.03407086	0.8984489	0.9396301
ENSG0000012	BBOX1	protein_codir	0.03373818	0.95435233	0.9732033
ENSG0000027	RP11-326G21	lncRNA	0.03363161	0.92778346	0.95705808
ENSG0000015	C7orf31	protein_codir	0.03361167	0.84751324	0.90915075
ENSG0000018	GREM2	protein_codir	0.03357858	NA	NA
ENSG0000017	EXOSC1	protein_codir	0.0335207	0.80928522	0.88216203
ENSG0000017	RUFY1	protein_codir	0.03326757	0.73000373	0.82634028
ENSG0000013	RTN3	protein_codir	0.03323009	0.81508202	0.8861026
ENSG0000025	CHCHD10	protein_codir	0.03314889	0.85407601	0.91288916
ENSG0000017	ZNF554	protein_codir	0.03292964	0.86248221	0.91809298
ENSG0000014	SIRT3	protein_codir	0.03285712	0.8387302	0.90326662
ENSG0000027	CTA-276F8.1	lncRNA	0.0327479	0.93493761	0.96157798
ENSG0000014	SHC3	protein_codir	0.03272573	0.94857839	0.97009297
ENSG0000010	INTS10	protein_codir	0.03271288	0.76858407	0.85424827
ENSG0000010	PHKB	protein_codir	0.03267023	0.76943599	0.85477388
ENSG0000028	PLD5P1	protein_codir	0.03246203	0.93401917	0.96112699
ENSG0000025	RP11-732A19	lncRNA	0.03224412	0.98485595	NA
ENSG0000018	C2orf88	protein_codir	0.03215595	0.9033237	0.94264019
ENSG0000019	ZXDB	protein_codir	0.0321503	0.88867508	0.93398804
ENSG0000013	GLCE	protein_codir	0.03211664	0.86511406	0.919654
ENSG0000015	GABPA	protein_codir	0.03207637	0.83450798	0.90028554
ENSG0000017	SMIM19	protein_codir	0.03202385	0.85319505	0.91267418
ENSG0000023	MED14OS	lncRNA	0.03200008	0.91812518	0.95156829
ENSG0000008	DDX18	protein_codir	0.03193697	0.74580197	0.83765594
ENSG0000017	PARL	protein_codir	0.03188818	0.8006637	0.87630098
ENSG0000021	NEURL4	protein_codir	0.03184257	0.85408943	0.91288916
ENSG0000017	MGA	protein_codir	0.03182122	0.84021134	0.90446009

ENSG0000013	ZNF304	protein_codir	0.03163076	0.84054679	0.90472185
ENSG0000016	YEATS2	protein_codir	0.03158089	0.79250024	0.87102425
ENSG0000013	IL6ST	protein_codir	0.03094938	0.87907428	0.92820673
ENSG0000012	AP5S1	protein_codir	0.03091084	0.83277378	0.89928607
ENSG0000016	PPIB	protein_codir	0.03085295	0.86643679	0.92063492
ENSG0000028	RP11-219B17.	lncRNA	0.0307072	0.98394901	0.99191564
ENSG0000016	NAA15	protein_codir	0.03065782	0.84984503	0.9106386
ENSG0000018	ANKDD1B	protein_codir	0.03055114	0.95542705	0.97390372
ENSG0000010	CCDC22	protein_codir	0.03035321	0.77787301	0.86088527
ENSG0000007	ATP6AP1	protein_codir	0.03033143	0.80704364	0.88056195
ENSG0000015	CETN3	protein_codir	0.03025762	0.8330811	0.89942359
ENSG0000010	L3MBTL2	protein_codir	0.0301269	0.79217624	0.8708421
ENSG0000007	SART3	protein_codir	0.0299701	0.73434058	0.82965136
ENSG0000013	RTN1	protein_codir	0.02990166	0.94545894	0.96824531
ENSG0000006	LRRC40	protein_codir	0.02988966	0.79586519	0.87315883
ENSG0000021	LINC01089	lncRNA	0.0298593	0.93124833	0.95938377
ENSG0000022	PET100	protein_codir	0.02985797	0.88011487	0.92895035
ENSG0000025	PRC1-AS1	lncRNA	0.0297544	0.94495568	0.96794526
ENSG0000013	NDUFA2	protein_codir	0.02974288	0.81502546	0.8861026
ENSG0000019	GPN1	protein_codir	0.02969973	0.79927019	0.87530441
ENSG0000017	IMP3	protein_codir	0.02967793	0.85368017	0.91285717
ENSG0000022	RP13-766D20	lncRNA	0.02957801	0.93914928	0.96392802
ENSG0000008	DOP1A	protein_codir	0.02949938	0.85945166	0.91610276
ENSG0000024	TM4SF1-AS1	lncRNA	0.02945298	0.97414455	0.98523829
ENSG0000013	CGREF1	protein_codir	0.02935801	0.94371984	0.96701244
ENSG0000008	ZFAND6	protein_codir	0.02933956	0.74871732	0.83966237
ENSG0000016	FXN	protein_codir	0.02932926	0.85084183	0.91121564
ENSG0000007	MAP2K7	protein_codir	0.02928963	0.82190273	0.89128203
ENSG0000017	SLCO3A1	protein_codir	0.02927132	0.83788806	0.90269798
ENSG0000018	TARS3	protein_codir	0.02924939	0.84876681	0.9099107
ENSG0000010	ARFGAP1	protein_codir	0.02916983	0.87802561	0.9275959
ENSG0000014	PDPK1	protein_codir	0.02900102	0.81242031	0.8845415
ENSG0000028	RP11-565O12	lncRNA	0.02896842	0.97118786	0.98350842
ENSG0000016	PBRM1	protein_codir	0.028892	0.85762634	0.91493393
ENSG0000017	SNHG11	lncRNA	0.02853333	0.88058594	0.92911872
ENSG0000004	HSPA5	protein_codir	0.02849159	0.91485599	0.94945858
ENSG0000017	FAM187B	protein_codir	0.02846379	0.98505985	NA
ENSG0000012	POT1	protein_codir	0.02838606	0.72672097	0.82390745
ENSG0000015	GPBP1L1	protein_codir	0.02833392	0.7480987	0.83942662
ENSG0000016	SCNM1	protein_codir	0.02824323	0.85480625	0.91319595
ENSG0000012	TM9SF2	protein_codir	0.02821125	0.86179082	0.91761439
ENSG0000012	ALDH1A2	protein_codir	0.02820994	0.96012728	0.97660438
ENSG0000008	RRN3	protein_codir	0.02809454	0.8384046	0.90316843
ENSG0000013	HIP1R	protein_codir	0.02806955	0.93090769	0.95917594
ENSG0000012	ACADS	protein_codir	0.02806045	0.86423899	0.91911928
ENSG0000006	AHRR	protein_codir	0.02792084	0.95590706	0.97399761
ENSG0000016	HGSNAT	protein_codir	0.02783787	0.79346254	0.8717357

ENSG0000010	DESI1	protein_codir	0.02783667	0.80237997	0.87762295
ENSG0000018	DGAT1	protein_codir	0.02779924	0.86626494	0.92055857
ENSG0000014	PSMA5	protein_codir	0.02768517	0.81975668	0.88947867
ENSG0000012	NUP214	protein_codir	0.02756293	0.6618565	0.77596278
ENSG0000028	RP11-1012A1	lncRNA	0.02755142	0.94577278	0.96849489
ENSG0000015	CDC123	protein_codir	0.0275442	0.82848679	0.89635419
ENSG0000010	NUP88	protein_codir	0.027505	0.84987441	0.9106386
ENSG0000024	FAM111A-DT	lncRNA	0.02728833	0.90487081	0.94357729
ENSG0000015	CTBP1	protein_codir	0.02723442	0.76737495	0.85331171
ENSG0000025	PRSS51	protein_codir	0.02714751	0.9834775	0.99155497
ENSG0000027	RP11-697E2.1	protein_codir	0.02710916	0.98683101	NA
ENSG0000015	RANBP2	protein_codir	0.02706419	0.90864394	0.94564063
ENSG0000021	FNIP1	protein_codir	0.02702188	0.87822468	0.92773525
ENSG0000012	SHC2	protein_codir	0.02695286	0.9539787	0.97297032
ENSG0000024	RP11-148K1.1	lncRNA	0.02659541	0.96287593	0.97828085
ENSG0000005	ZC3H11A	protein_codir	0.02637893	0.71734074	0.81736597
ENSG0000013	PI15	protein_codir	0.0263383	0.97142256	0.98363791
ENSG0000018	ZNF286A	protein_codir	0.02624243	0.85891067	0.91587957
ENSG0000007	SNX13	protein_codir	0.02623734	0.87755152	0.92735653
ENSG0000010	ATG4A	protein_codir	0.02622035	0.84769905	0.90918949
ENSG0000011	ASCC3	protein_codir	0.02606275	0.88314178	0.93082114
ENSG0000001	ABHD5	protein_codir	0.02591189	0.90351173	0.94272956
ENSG0000010	CRHR2	protein_codir	0.02587543	0.97242518	0.9843816
ENSG0000026	CTD-3220F14.	lncRNA	0.02584145	0.96675426	0.98060054
ENSG0000010	KIAA0586	protein_codir	0.02571566	0.8701607	0.92299391
ENSG0000010	GTPBP4	protein_codir	0.02560533	0.8888896	0.9340189
ENSG0000020	RANBP17	protein_codir	0.02556781	0.90714415	0.94473516
ENSG0000012	UTP20	protein_codir	0.02538398	0.90527722	0.94371842
ENSG0000008	GSK3B	protein_codir	0.02534684	0.87218013	0.92419376
ENSG0000013	NAA35	protein_codir	0.02531356	0.84107922	0.90494622
ENSG0000018	SNRPE	protein_codir	0.02524967	0.82911365	0.89684809
ENSG0000026	SLC25A53	protein_codir	0.02515853	0.90986967	0.94632476
ENSG0000014	AK3	protein_codir	0.02514873	0.88900792	0.9340189
ENSG0000010	SHOC2	protein_codir	0.0250079	0.84615018	0.90837484
ENSG0000016	C16orf46	protein_codir	0.02481578	0.96651274	0.98053561
ENSG0000015	TMEM87B	protein_codir	0.02478866	0.87703738	0.92711928
ENSG0000012	ENY2	protein_codir	0.02473326	0.87785082	0.9274965
ENSG0000015	TIAL1	protein_codir	0.0246748	0.80805757	0.88122486
ENSG0000013	FAM104A	protein_codir	0.02464258	0.74551861	0.83751811
ENSG0000016	PRRC1	protein_codir	0.02462934	0.89910474	0.93983424
ENSG0000014	FN3KRP	protein_codir	0.0246229	0.87625374	0.92653912
ENSG0000022	BTBD19	protein_codir	0.02446901	0.94716952	0.96935016
ENSG0000016	HNRNPK	protein_codir	0.02446045	0.83405462	0.90004254
ENSG0000016	PCMTD1	protein_codir	0.02444542	0.86935673	0.92260174
ENSG0000013	FGD4	protein_codir	0.02441425	0.92068933	0.95315849
ENSG0000017	PRDM10	protein_codir	0.02440257	0.83100297	0.89815309
ENSG0000013	TGFBRAP1	protein_codir	0.02437492	0.87493412	0.92578172

ENSG0000019	GTF2E2	protein_codir	0.02425818	0.8019395	0.87728014
ENSG0000013	TTC5	protein_codir	0.02417166	0.84658473	0.90859414
ENSG0000009	ECHDC1	protein_codir	0.0241591	0.853413	0.91280126
ENSG0000023	AC079354.5	lncRNA	0.02413992	0.98709574	NA
ENSG0000013	NEK3	protein_codir	0.02395909	0.90900048	0.94586001
ENSG0000022	NUTM2A-AS1	lncRNA	0.02392419	0.89764402	0.93916163
ENSG0000015	SLC16A12	protein_codir	0.0239059	0.95857361	0.97576567
ENSG0000016	STT3B	protein_codir	0.02387588	0.88931533	0.93419857
ENSG0000016	SGMS2	protein_codir	0.02385187	0.95009456	0.97065329
ENSG0000018	NPIPA5	protein_codir	0.02384979	0.94849195	0.97009297
ENSG0000014	USP53	protein_codir	0.0237672	0.94906803	0.97032238
ENSG0000017	MROH1	protein_codir	0.02362395	0.8940581	0.93686489
ENSG0000009	PDPR	protein_codir	0.02359946	0.85734959	0.91475872
ENSG0000010	GTPBP10	protein_codir	0.02347278	0.88060869	0.92911872
ENSG0000023	ADSL	protein_codir	0.02305671	0.81367456	0.88546023
ENSG0000011	RRAGC	protein_codir	0.02283478	0.88961405	0.93426389
ENSG0000027	FAM223A	lncRNA	0.02283039	0.96299283	0.97829228
ENSG0000027	FAM223B	lncRNA	0.02283039	0.96299283	0.97829228
ENSG0000026	NDUFV2-AS1	lncRNA	0.02271271	0.91543254	0.94982144
ENSG0000011	ATP5PB	protein_codir	0.02263587	0.84515224	0.9076232
ENSG0000023	LINC02580	lncRNA	0.02255664	0.95088196	0.97113444
ENSG0000014	IMMP1L	protein_codir	0.02255574	0.88970353	0.93432232
ENSG0000007	TOP2B	protein_codir	0.02247046	0.88676604	0.93290462
ENSG0000020	DAXX	protein_codir	0.02223374	0.87301952	0.92467689
ENSG0000017	LINC00476	lncRNA	0.02222305	0.94134471	0.96557129
ENSG0000013	UGGT1	protein_codir	0.02214058	0.89369101	0.93669345
ENSG0000013	RAP1GDS1	protein_codir	0.02212136	0.84809858	0.90951205
ENSG0000017	NFXL1	protein_codir	0.02203889	0.89283563	0.93611377
ENSG0000019	ZNF665	protein_codir	0.02197819	0.92644838	0.95648785
ENSG0000017	ZNF619	protein_codir	0.0219722	0.92546656	0.95583657
ENSG0000018	AC138969.4	protein_codir	0.0217907	0.88335459	0.93098232
ENSG0000016	RRP1	protein_codir	0.02171749	0.89412584	0.93690032
ENSG0000016	TK2	protein_codir	0.02156917	0.87411053	0.9253002
ENSG0000028	RP11-21M24.	lncRNA	0.02154334	0.96551446	0.97993203
ENSG0000025	TSPAN9-IT1	lncRNA	0.02118596	0.98844289	NA
ENSG0000024	LINC02067	lncRNA	0.02092355	0.98792558	NA
ENSG0000009	NANS	protein_codir	0.02088385	0.9196907	0.95247973
ENSG0000027	RP11-817I4.2	lncRNA	0.02086547	0.96156672	0.97756452
ENSG0000011	SRSF4	protein_codir	0.02082831	0.89279224	0.93610667
ENSG0000012	NCOA5	protein_codir	0.0207816	0.83482706	0.90052424
ENSG0000006	TAF11	protein_codir	0.02055173	0.84242124	0.9060621
ENSG0000017	TEX26	protein_codir	0.02052113	0.96450539	0.97932648
ENSG0000015	SPOCK1	protein_codir	0.02051196	0.95631925	0.97424897
ENSG0000007	RBM7	protein_codir	0.02045191	0.91883903	0.95199692
ENSG0000016	REPS2	protein_codir	0.02044208	0.92770869	0.95705808
ENSG0000013	EPC2	protein_codir	0.02039537	0.90897077	0.94586001
ENSG0000027	RP11-195B17.	lncRNA	0.02031001	0.96033662	0.97663748

ENSG0000019	ZNF81	protein_codir	0.02027849	0.91398816	0.9490715
ENSG0000003	EDC4	protein_codir	0.02027576	0.85911459	0.91602406
ENSG0000010	RBX1	protein_codir	0.02013356	0.87152429	0.92365991
ENSG0000010	DPH1	protein_codir	0.02012245	0.89026579	0.93462849
ENSG0000025	HMBS	protein_codir	0.02008457	0.93134114	0.9594436
ENSG0000027	GAS5-AS1	lncRNA	0.02007854	0.95658818	0.97434386
ENSG0000013	RMDN3	protein_codir	0.01992407	0.85402422	0.91288916
ENSG0000008	CHERP	protein_codir	0.01989442	0.8039497	0.87850494
ENSG0000018	ZNF816	protein_codir	0.01977388	0.94201175	0.96605665
ENSG0000011	WDR75	protein_codir	0.01958124	0.90065145	0.94056235
ENSG0000009	DPYSL2	protein_codir	0.01952639	0.91442696	0.94911132
ENSG0000027	ENSG0000027	protein_codir	0.01951189	0.96209527	0.97793033
ENSG0000004	MED17	protein_codir	0.01942124	0.85184756	0.91183311
ENSG0000017	TMED10	protein_codir	0.0193617	0.91589818	0.95001426
ENSG0000017	TNKS	protein_codir	0.01928145	0.88427826	0.93128621
ENSG0000026	RP11-817O13	lncRNA	0.01922273	0.93261218	0.96036153
ENSG0000025	RP11-234B24.	protein_codir	0.01918872	0.93610882	0.96217461
ENSG0000014	NVL	protein_codir	0.01914335	0.87871433	0.92793308
ENSG0000022	PRKAR2A-AS1	lncRNA	0.01902599	0.92686143	0.95661271
ENSG0000008	ATRN	protein_codir	0.01897679	0.92985709	0.95841522
ENSG0000026	LINC00543	lncRNA	0.01894754	0.98930986	0.99434486
ENSG0000011	FOXO3	protein_codir	0.01894325	0.9440401	0.96724419
ENSG0000015	NIFK	protein_codir	0.01892888	0.89112259	0.93510148
ENSG0000012	EMC6	protein_codir	0.01891587	0.88987539	0.93443174
ENSG0000016	TLCD3A	protein_codir	0.01890968	0.95078209	0.97106835
ENSG0000013	TMEM14B	protein_codir	0.01872252	0.85073213	0.91113348
ENSG0000028	RP11-61J19.8	lncRNA	0.01865953	0.97556492	0.98606534
ENSG0000023	RAMACL	protein_codir	0.01862445	0.97691705	0.98684986
ENSG0000025	LSM14A	protein_codir	0.01860037	0.87489303	0.92577981
ENSG0000014	SPATA5	protein_codir	0.01856763	0.84597699	0.90829483
ENSG0000017	ZNF417	protein_codir	0.01854901	0.92029524	0.95293374
ENSG0000019	CHAMP1	protein_codir	0.01854849	0.91507946	0.94958684
ENSG0000023	KMT2E-AS1	lncRNA	0.01851184	0.95556655	0.97394445
ENSG0000010	RIN3	protein_codir	0.01850786	0.91670042	0.95063094
ENSG0000014	COMMD4	protein_codir	0.01848263	0.90837019	0.94540601
ENSG0000016	SSU72	protein_codir	0.01845428	0.85819554	0.91543509
ENSG0000016	ZNF319	protein_codir	0.01838761	0.91286398	0.9484085
ENSG0000016	ZNF146	protein_codir	0.01821009	0.86207943	0.91777035
ENSG0000012	TENT4B	protein_codir	0.01811396	0.87992942	0.92883018
ENSG0000027	RP11-435O5.5	lncRNA	0.0180551	0.95178505	0.97173338
ENSG0000013	ELOF1	protein_codir	0.01804923	0.8516495	0.9117271
ENSG0000017	TUBAL3	protein_codir	0.01786798	0.98427662	0.99199854
ENSG0000014	AASDHPPT	protein_codir	0.01785643	0.90828343	0.94540601
ENSG0000012	BBS2	protein_codir	0.01785614	0.89061402	0.93478089
ENSG0000027	RP1-153G14.4	lncRNA	0.01782421	0.95833176	0.97564037
ENSG0000023	RP11-301L8.2	lncRNA	0.01779816	0.9853076	0.99260298
ENSG0000013	VPS13B	protein_codir	0.01772424	0.90664543	0.94447381

ENSG0000009	THPO	protein_codir	0.01763029	0.95234024	0.97200493
ENSG0000011	KLHL12	protein_codir	0.01742493	0.88902628	0.9340189
ENSG0000007	TNRC6C	protein_codir	0.01735713	0.89626275	0.93828515
ENSG0000017	ZNF654	protein_codir	0.01731315	0.9098482	0.94632476
ENSG0000025	RP11-631N16	lncRNA	0.01727453	0.95449384	0.97320388
ENSG0000025	RP11-946L16.	lncRNA	0.01726708	0.99404291	NA
ENSG0000022	LINC01277	lncRNA	0.01721915	0.97987429	0.98882455
ENSG0000026	RP11-384P7.7	lncRNA	0.01720043	0.97942442	0.98865954
ENSG0000026	RP4-806M20.	lncRNA	0.01706924	0.98716678	0.99352633
ENSG0000016	SLU7	protein_codir	0.01704624	0.84387881	0.90670837
ENSG0000013	GNL3L	protein_codir	0.01703184	0.89585621	0.93800176
ENSG0000006	SLC9A3	protein_codir	0.01698466	0.93757422	0.96286654
ENSG0000014	UBA3	protein_codir	0.0168975	0.8440716	0.90677782
ENSG0000017	TPRN	protein_codir	0.01669929	0.90501717	0.94369429
ENSG0000011	RNF130	protein_codir	0.01649242	0.91808679	0.95156829
ENSG0000014	FBXO38	protein_codir	0.01643659	0.86740202	0.92133933
ENSG0000028	RP11-506B6.7	protein_codir	0.01640345	0.9911838	NA
ENSG0000011	GNPDA1	protein_codir	0.01632944	0.92011047	0.95277814
ENSG0000013	MRPL15	protein_codir	0.01618545	0.90871174	0.94565459
ENSG0000025	SEC23A-AS1	lncRNA	0.01609253	0.99134085	NA
ENSG0000017	CHD3	protein_codir	0.01606837	0.90518564	0.94371842
ENSG0000013	PIGC	protein_codir	0.01573855	0.88097161	0.92938768
ENSG0000025	RP11-22A3.2	lncRNA	0.01566055	0.98673688	0.9934186
ENSG0000001	NISCH	protein_codir	0.01562217	0.90086284	0.94071197
ENSG0000015	BRAF	protein_codir	0.01556632	0.90258801	0.94215725
ENSG0000010	PMPCB	protein_codir	0.01534985	0.83731148	0.90232891
ENSG0000026	AF038458.5	lncRNA	0.01527741	0.99474247	NA
ENSG0000008	KHSRP	protein_codir	0.01517399	0.88777854	0.93348541
ENSG0000026	CTA-29F11.1	lncRNA	0.0150976	0.97193229	0.98404581
ENSG0000028	LA16c-306E5.	protein_codir	0.01489979	0.98372482	0.99176813
ENSG0000016	MPLKIP	protein_codir	0.01489914	0.89157603	0.93542747
ENSG0000013	MED4	protein_codir	0.01489892	0.86303718	0.9184981
ENSG0000016	ZNF606	protein_codir	0.01481357	0.95372186	0.97285767
ENSG0000016	SAP30	protein_codir	0.01461035	0.9557929	0.97399761
ENSG0000010	CDC37	protein_codir	0.01454799	0.92315889	0.95468676
ENSG0000014	WDR45B	protein_codir	0.01448823	0.9238354	0.9551671
ENSG0000016	SAP30BP	protein_codir	0.0144767	0.92599344	0.95627971
ENSG0000018	WASH6P	protein_codir	0.01432665	0.95947675	0.97619435
ENSG0000018	ZNF626	protein_codir	0.0142199	0.95589849	0.97399761
ENSG0000010	TOMM22	protein_codir	0.01408657	0.89624895	0.93828515
ENSG0000017	TMEM94	protein_codir	0.01401837	0.88739629	0.93335436
ENSG0000011	KCTD20	protein_codir	0.01398951	0.94325458	0.96689145
ENSG0000016	LZTFL1	protein_codir	0.01388577	0.93605109	0.96217461
ENSG0000010	RAI1	protein_codir	0.01366025	0.92735351	0.95690604
ENSG0000011	DNMT3A	protein_codir	0.01364126	0.923198	0.95468676
ENSG0000010	SRP54	protein_codir	0.01360635	0.89456253	0.93714459
ENSG0000015	VPS8	protein_codir	0.01360315	0.88451183	0.93139954

ENSG0000022	LINC00629	lncRNA	0.01356341	0.98775236	0.99372478
ENSG0000016	METTL27	protein_codir	0.01350797	0.97572268	0.9861323
ENSG0000011	NNT	protein_codir	0.01347413	0.92781271	0.95705808
ENSG0000014	ANP32A	protein_codir	0.01344675	0.91522749	0.94964436
ENSG0000026	LINC01775	lncRNA	0.01341248	0.99129883	0.99566973
ENSG0000000	AP2B1	protein_codir	0.01337745	0.91240659	0.94805017
ENSG0000013	ELP3	protein_codir	0.01334072	0.91184803	0.94756171
ENSG0000006	SEC61A2	protein_codir	0.01329954	0.95376684	0.97285767
ENSG0000010	LHB	protein_codir	0.01329401	0.98926336	0.99433945
ENSG0000015	TMEM164	protein_codir	0.01322764	0.93321476	0.96072875
ENSG0000011	MSH3	protein_codir	0.01321617	0.93439702	0.96137253
ENSG0000026	MYZAP	protein_codir	0.01317741	0.97844098	0.98788345
ENSG0000027	RP1-267D11.6	lncRNA	0.01314194	0.94666603	0.96908623
ENSG0000008	RBM41	protein_codir	0.01295233	0.93304226	0.96062277
ENSG0000013	HECW2	protein_codir	0.01286178	0.96643932	0.98049714
ENSG0000012	ATF1	protein_codir	0.01282872	0.93357111	0.96091653
ENSG0000025	AC002519.8	lncRNA	0.01282515	0.99022534	0.99487192
ENSG0000013	TBC1D5	protein_codir	0.01267266	0.93754291	0.96286654
ENSG0000020	VGLL3	protein_codir	0.01251695	0.97737116	0.98718951
ENSG0000010	CCNY	protein_codir	0.01244148	0.93887673	0.96371991
ENSG0000019	ZNF770	protein_codir	0.01243692	0.93024886	0.9586867
ENSG0000020	TCTN1	protein_codir	0.01243635	0.93513041	0.96159697
ENSG0000010	GRPEL1	protein_codir	0.0124179	0.94519183	0.96805259
ENSG0000011	FBRSL1	protein_codir	0.01241282	0.92874674	0.95767553
ENSG0000016	IRF2BP2	protein_codir	0.01239577	0.93251679	0.96033236
ENSG0000022	RP11-398K22.	lncRNA	0.01235809	0.94879729	0.97015331
ENSG0000015	PLEKHH2	protein_codir	0.01226359	0.97164821	0.98383033
ENSG0000015	NDUFS2	protein_codir	0.01201856	0.90963019	0.94632476
ENSG0000013	NPFF	protein_codir	0.01201441	0.98092596	0.98965329
ENSG0000015	AZIN1	protein_codir	0.0119948	0.9338998	0.96109918
ENSG0000011	ZNF346	protein_codir	0.01176279	0.93170022	0.95966668
ENSG0000013	IL13RA1	protein_codir	0.0117156	0.9507113	0.97106786
ENSG0000027	RP11-700J17.	lncRNA	0.0116338	0.98566744	0.99289305
ENSG0000025	RP11-893F2.1	lncRNA	0.01157121	0.98856164	0.99414063
ENSG0000019	SREBF2	protein_codir	0.01151442	0.95203047	0.97188741
ENSG0000026	RP11-285A1.1	lncRNA	0.01133922	0.98513887	0.99248652
ENSG0000026	PCF11-AS1	lncRNA	0.0113037	0.98453683	0.99211599
ENSG0000017	PRSS27	protein_codir	0.01121992	0.9587274	0.97579139
ENSG0000013	HADHB	protein_codir	0.01116256	0.93756522	0.96286654
ENSG0000028	AP000326.5	protein_codir	0.01108158	0.99112967	0.99559941
ENSG0000013	MAP2K5	protein_codir	0.01106202	0.93382175	0.96106706
ENSG0000023	RP11-38L15.3	lncRNA	0.0110437	0.97402401	0.98521419
ENSG0000018	ASB7	protein_codir	0.01061042	0.94672424	0.96908802
ENSG0000026	RP13-516M14	lncRNA	0.01059737	0.97244214	0.9843816
ENSG0000008	TRAF5	protein_codir	0.01049868	0.96605506	0.98030387
ENSG0000011	ACTR3	protein_codir	0.01043916	0.95301868	0.97252553
ENSG0000015	ZFYVE27	protein_codir	0.01039238	0.94752751	0.96957283

ENSG0000017	ZNF562	protein_codir	0.01037353	0.93448511	0.96142735
ENSG0000013	PTPRA	protein_codir	0.01030951	0.929694	0.95828289
ENSG0000001	NME1-NME2	protein_codir	0.01025565	0.97633511	0.98647848
ENSG0000006	ELAVL1	protein_codir	0.01024159	0.88102813	0.92938768
ENSG0000025	RP11-268P4.5	lncRNA	0.01018517	0.99584322	NA
ENSG0000011	ACCS	protein_codir	0.00996702	0.97831662	0.98783012
ENSG0000027	RP11-345P4.9	lncRNA	0.00993445	0.97043243	0.98281548
ENSG0000023	FAM200B	protein_codir	0.00978527	0.95401818	0.97297032
ENSG0000010	HMGXB4	protein_codir	0.00975813	0.91391036	0.94906208
ENSG0000012	METTL25	protein_codir	0.00972377	0.94988366	0.97062636
ENSG0000011	ESRRB	protein_codir	0.00972061	0.97940854	0.98865954
ENSG0000011	TCEANC2	protein_codir	0.00953166	0.95907853	0.9760049
ENSG0000016	PPP1R35	protein_codir	0.00950338	0.96506807	0.97974541
ENSG0000014	HERC4	protein_codir	0.009395	0.93507279	0.96159697
ENSG0000028	RP11-331G2.1	lncRNA	0.00935745	0.98649517	0.99329214
ENSG0000013	SULF1	protein_codir	0.00928305	0.98094635	0.98965329
ENSG0000016	MAGOH	protein_codir	0.00923756	0.93494484	0.96157798
ENSG0000014	BTF3	protein_codir	0.00921609	0.94747165	0.96955159
ENSG0000012	PAX1	protein_codir	0.00915799	0.99392174	0.99742464
ENSG0000016	OTUB1	protein_codir	0.00907206	0.94206085	0.96605665
ENSG0000014	MYLK4	protein_codir	0.00904126	0.96621939	0.98038205
ENSG0000022	FAM200A	protein_codir	0.00902056	0.97201677	0.98409527
ENSG0000028	CTC-250I14.7	lncRNA	0.00899173	0.99413277	0.99743626
ENSG0000016	CTNNB1	protein_codir	0.00894932	0.9603129	0.97663748
ENSG0000026	CTD-2349P21	lncRNA	0.00890508	0.99478761	0.99778602
ENSG0000011	TTLL5	protein_codir	0.00888368	0.94406114	0.96724419
ENSG0000023	NME1	protein_codir	0.00880386	0.97607589	0.98635364
ENSG0000016	CYB5A	protein_codir	0.0087941	0.94520066	0.96805259
ENSG0000014	COPS7B	protein_codir	0.00878302	0.94940665	0.97045304
ENSG0000027	RP11-323F24.	lncRNA	0.00872023	0.99545906	0.99809705
ENSG0000001	SLC6A13	protein_codir	0.00857119	0.98695347	0.99352633
ENSG0000010	UTP6	protein_codir	0.00853305	0.92671219	0.95650756
ENSG0000005	TAB2	protein_codir	0.00843303	0.96284459	0.97828085
ENSG0000019	TSC22D2	protein_codir	0.00836132	0.98447821	0.99209311
ENSG0000020	C4orf47	protein_codir	0.00826378	0.98030623	0.98920403
ENSG0000011	CEP164	protein_codir	0.00821114	0.95317919	0.97258153
ENSG0000027	RP11-585P4.6	lncRNA	0.00820677	0.98480659	0.99227923
ENSG0000015	TMEM218	protein_codir	0.00819121	0.95077091	0.97106835
ENSG0000013	PPFIA1	protein_codir	0.00816862	0.94357123	0.96699333
ENSG0000013	PLAAT2	protein_codir	0.00796517	0.9913582	0.99566973
ENSG0000014	PURB	protein_codir	0.00785459	0.9684161	0.98154462
ENSG0000004	TPR	protein_codir	0.00779368	0.94863291	0.97009297
ENSG0000006	NUP133	protein_codir	0.00776069	0.94997664	0.97062636
ENSG0000022	LINC01203	lncRNA	0.00774375	0.99551264	0.99811453
ENSG0000013	MYC	protein_codir	0.0077412	0.98937674	0.99434486
ENSG0000009	RANBP1	protein_codir	0.00771997	0.95719125	0.97473076
ENSG0000012	CSMD2	protein_codir	0.00771293	0.97769661	0.98738321

ENSG0000016	UBXN1	protein_codir	0.0076365	0.96118831	0.97728773
ENSG0000017	NDUFA3	protein_codir	0.00747946	0.9598379	0.97638195
ENSG0000013	MRPL50	protein_codir	0.00746074	0.94688068	0.96914689
ENSG0000016	AGL	protein_codir	0.00734397	0.97779339	0.98741009
ENSG0000000	PAF1	protein_codir	0.00722483	0.96607136	0.98030387
ENSG0000016	FLAD1	protein_codir	0.00705888	0.96846684	0.98154462
ENSG0000018	TRIM61	protein_codir	0.00698845	0.97892861	0.98826742
ENSG0000014	UBAP2L	protein_codir	0.00691657	0.92214797	0.95406529
ENSG0000011	NEK11	protein_codir	0.00688529	0.97950441	0.9886949
ENSG0000013	DKC1	protein_codir	0.00687252	0.9574978	0.97497348
ENSG0000017	GCC1	protein_codir	0.00668794	0.96658872	0.98054065
ENSG0000022	EXOSC6	protein_codir	0.00659144	0.95234648	0.97200493
ENSG0000011	CD58	protein_codir	0.0065844	0.97008595	0.98264473
ENSG0000007	PIAS2	protein_codir	0.00645215	0.95486049	0.97350584
ENSG0000015	RMND5A	protein_codir	0.00636781	0.96913205	0.98200261
ENSG0000013	ALDH1L2	protein_codir	0.00623736	0.98280903	0.99113427
ENSG0000008	SRCAP	protein_codir	0.00622998	0.96694223	0.98064715
ENSG0000018	ANKRD37	protein_codir	0.00619284	0.98194527	0.99033551
ENSG0000010	ATE1	protein_codir	0.00614582	0.97263814	0.98439962
ENSG0000014	EIF2A	protein_codir	0.00613563	0.96061335	0.97685685
ENSG0000010	TUBD1	protein_codir	0.00607484	0.97601272	0.98633315
ENSG0000012	GFER	protein_codir	0.00602884	0.95633013	0.97424897
ENSG0000006	ABCC9	protein_codir	0.00599826	0.98730857	0.99352633
ENSG0000028	RP11-8L8.3	lncRNA	0.00598481	0.99660473	0.99870902
ENSG0000015	SREK1	protein_codir	0.00592314	0.97542365	0.98606256
ENSG0000025	RP11-253I19.3	lncRNA	0.00571096	0.99555201	0.99811777
ENSG0000016	GNG3	protein_codir	0.00570745	0.99560484	0.99813451
ENSG0000013	FLOT2	protein_codir	0.00568329	0.96620553	0.98038205
ENSG0000018	ZDHHC17	protein_codir	0.00557955	0.9538903	0.97294767
ENSG0000014	UQCRC2	protein_codir	0.00553569	0.9609867	0.97711871
ENSG0000017	RUVBL1	protein_codir	0.00552372	0.97752673	0.98724909
ENSG0000019	GOLGA6L9	protein_codir	0.00550332	0.98900583	0.9943153
ENSG0000011	GALE	protein_codir	0.00537851	0.98133451	0.98990171
ENSG0000008	SLC25A24	protein_codir	0.00531122	0.97986075	0.98882455
ENSG0000013	MDH1B	protein_codir	0.00512139	0.98752753	0.99360717
ENSG0000011	SENP5	protein_codir	0.0050801	0.95659966	0.97434386
ENSG0000014	MAEL	protein_codir	0.00507983	0.99274976	0.99661064
ENSG0000004	USP28	protein_codir	0.00507412	0.97422225	0.9852808
ENSG0000023	FTX	lncRNA	0.00504859	0.98659824	0.99332349
ENSG0000024	RP11-46A10.5	lncRNA	0.00502385	0.98799493	0.99382402
ENSG0000016	SAAL1	protein_codir	0.00490819	0.965563	0.97993203
ENSG0000014	PRCC	protein_codir	0.00483262	0.95498486	0.9735967
ENSG0000010	PTPRS	protein_codir	0.00455915	0.98709036	0.99352633
ENSG0000019	PPIA	protein_codir	0.00455406	0.96859009	0.98160532
ENSG0000010	SUGP1	protein_codir	0.00446749	0.97318416	0.98473573
ENSG0000015	ZC3H18	protein_codir	0.00446201	0.96210358	0.97793033
ENSG0000016	BICDL2	protein_codir	0.00425636	0.99654651	0.99870902

ENSG0000013	ZFHX2	protein_codir	0.00424988	0.98515606	0.99248652
ENSG0000017	RXFP1	protein_codir	0.0042488	0.99398875	0.99743626
ENSG0000024	RN7SL832P	lncRNA	0.00421575	0.98998607	0.99477626
ENSG0000015	RGS12	protein_codir	0.00420634	0.9796436	0.98877238
ENSG0000019	ITGBL1	protein_codir	0.00412457	0.99272921	0.99661064
ENSG0000007	RAB7A	protein_codir	0.00410757	0.96770482	0.98109632
ENSG0000013	KANSL2	protein_codir	0.00404529	0.97538387	0.98605843
ENSG0000028	CTC-281F24.5	protein_codir	0.00385036	0.98439281	0.99207945
ENSG0000017	ALG14	protein_codir	0.00384584	0.98319452	0.99141443
ENSG0000015	CNOT11	protein_codir	0.00375961	0.97259524	0.98439304
ENSG0000027	RP11-274B21.	lncRNA	0.0036919	0.99391994	0.99742464
ENSG0000014	CCDC120	protein_codir	0.00367814	0.97388413	0.98519136
ENSG0000009	FMO2	protein_codir	0.00365409	0.99289498	0.99672019
ENSG0000019	IPO4	protein_codir	0.00352883	0.9889617	0.9943153
ENSG0000022	MT-ATP8	protein_codir	0.00335489	0.99008437	0.99480266
ENSG0000008	NFX1	protein_codir	0.0033257	0.97709421	0.98699272
ENSG0000020	E2F4	protein_codir	0.00330917	0.97953103	0.9886949
ENSG0000017	FGGY	protein_codir	0.00327253	0.98505523	0.99245734
ENSG0000010	KDELR1	protein_codir	0.00313412	0.97773102	0.98738321
ENSG0000012	FAM53C	protein_codir	0.00309998	0.98309401	0.99134928
ENSG0000018	LINC00265	lncRNA	0.0030731	0.98916286	0.99433945
ENSG0000010	SMURF2	protein_codir	0.00283563	0.98443169	0.99208244
ENSG0000018	TMEM45A	protein_codir	0.00276853	0.99302207	0.99681154
ENSG0000016	CCNDBP1	protein_codir	0.00276762	0.98032197	0.98920403
ENSG0000014	TLK2	protein_codir	0.00261191	0.97512651	0.9859787
ENSG0000027	AC008984.2	lncRNA	0.00258919	0.99843149	0.99962583
ENSG0000024	RP11-381N20	lncRNA	0.00257401	0.99809545	0.99959925
ENSG0000015	B4GALT5	protein_codir	0.00254032	0.99172686	0.99587333
ENSG0000002	SEC63	protein_codir	0.00243788	0.98739443	0.99354515
ENSG0000025	RP11-530C5.1	lncRNA	0.00240717	0.99775211	0.99943857
ENSG0000019	FUT11	protein_codir	0.0022365	0.98764083	0.99368496
ENSG0000027	RP11-1060G2	lncRNA	0.00211395	0.99827574	0.99959925
ENSG0000028	CH17-3B23.3	lncRNA	0.00192865	0.99787095	0.99949939
ENSG0000011	TTF2	protein_codir	0.00192856	0.99093168	0.99547294
ENSG0000010	SERPINE1	protein_codir	0.00178973	0.99760838	0.99938136
ENSG0000021	SRA1	protein_codir	0.00166685	0.9913389	0.99566973
ENSG0000010	CCDC130	protein_codir	0.0016266	0.99523056	0.99801285
ENSG0000015	TRMT44	protein_codir	0.00156353	0.9910869	0.99559265
ENSG0000023	LINC01366	lncRNA	0.00110825	0.99811119	0.99959925
ENSG0000005	SYNE2	protein_codir	0.00108551	0.99617308	0.99852297
ENSG0000023	RP4-715N11.2	lncRNA	0.00098605	0.9996068	NA
ENSG0000018	ZNF284	protein_codir	0.0009526	0.9974157	0.99924072
ENSG0000014	EMP3	protein_codir	0.00093319	0.99566075	0.99815433
ENSG0000018	NUDT17	protein_codir	0.00092421	0.99672511	0.99878633
ENSG0000016	FOXH1	protein_codir	0.00088175	0.9993202	0.99964593
ENSG0000022	RP11-173B14.	lncRNA	0.00074048	0.99923723	0.99963535
ENSG0000014	ZNF473	protein_codir	0.00072779	0.99592797	0.99835561

ENSG0000028	RP11-426C22.	lncRNA	0.00060078	0.99901749	0.99963535
ENSG0000021	GPX3	protein_codir	0.00051235	0.999199	0.99963535
ENSG0000018	MIR22HG	lncRNA	0.0004065	0.99912968	0.99963535
ENSG0000012	NDUFA1	protein_codir	0.00039782	0.99735181	0.99923306
ENSG0000017	CENPS	protein_codir	0.00031739	0.99872472	0.99963535
ENSG0000016	FLYWCH2	protein_codir	0.00026685	0.99829637	0.99959925
ENSG0000012	PRMT1	protein_codir	0.00025525	0.99861238	0.99963018
ENSG0000022	CPTP	protein_codir	0.00019066	0.9988982	0.99963535
ENSG0000024	RP11-712B9.2	lncRNA	0.00014914	0.99949643	0.9996774
ENSG0000012	ZNFX1	protein_codir	0.00011554	0.99942961	0.9996774
ENSG0000010	SAMM50	protein_codir	4.81E-05	0.99963742	0.99978222
ENSG0000012	PRR12	protein_codir	1.53E-06	0.99999131	0.99999131
ENSG0000027	SCARNA2	lncRNA	0	1	NA
ENSG0000024	PEX5L-AS2	lncRNA	0	1	NA
ENSG0000024	RP11-826N14	lncRNA	0	1	NA
ENSG0000013	ALDOB	protein_codir	0	1	NA
ENSG0000019	CYP2A13	protein_codir	0	1	NA
ENSG0000014	POLR3A	protein_codir	-3.32E-06	0.99997227	0.99999131
ENSG0000016	AZGP1	protein_codir	-2.70E-05	0.99996225	0.99999131
ENSG0000027	MRPL45	protein_codir	-0.00015931	0.99879465	0.99963535
ENSG0000010	TXN2	protein_codir	-0.00016737	0.99877812	0.99963535
ENSG0000019	NIF3L1	protein_codir	-0.00027474	0.99826826	0.99959925
ENSG0000013	FAAP24	protein_codir	-0.00028279	0.99893491	0.99963535
ENSG0000013	RPA1	protein_codir	-0.00032284	0.9983703	0.9996008
ENSG0000006	KARS1	protein_codir	-0.00043581	0.99645322	0.99869504
ENSG0000023	LINC01135	lncRNA	-0.00046977	0.99886507	0.99963535
ENSG0000019	MT-ATP6	protein_codir	-0.00053515	0.99861277	0.99963018
ENSG0000027	RP11-10E18.7	lncRNA	-0.00059846	0.99911981	0.99963535
ENSG0000015	CABP1	protein_codir	-0.00065164	0.99852202	0.99963018
ENSG0000022	RP11-66D17.5	lncRNA	-0.00067275	0.99930022	0.99964593
ENSG0000008	PHLPP1	protein_codir	-0.00070504	0.99699064	0.99897992
ENSG0000017	TMCC1	protein_codir	-0.00084561	0.99460137	0.99770791
ENSG0000014	SGSM2	protein_codir	-0.00086474	0.99593382	0.99835561
ENSG0000016	PAQR3	protein_codir	-0.00088277	0.99633775	0.99861555
ENSG0000027	RP11-351I21.	lncRNA	-0.00090567	0.99860276	0.99963018
ENSG0000013	YARS2	protein_codir	-0.00091857	0.99406014	0.99743626
ENSG0000025	RP11-283G6.3	lncRNA	-0.00100055	0.99796742	0.99955977
ENSG0000016	TTC9C	protein_codir	-0.00105808	0.99515666	0.99799241
ENSG0000016	KIAA1586	protein_codir	-0.00114114	0.99489956	0.99786208
ENSG0000025	FIGNL2-DT	lncRNA	-0.00115962	0.99922613	0.99963535
ENSG0000015	DHRS4	protein_codir	-0.0011712	0.9924839	0.99641617
ENSG0000003	ADSS2	protein_codir	-0.00137457	0.99191427	0.99602531
ENSG0000026	CTB-191K22.6	lncRNA	-0.00142371	0.99913064	0.99963535
ENSG0000026	CTD-2517M22	lncRNA	-0.00150394	0.99833846	0.9996008
ENSG0000012	PAICS	protein_codir	-0.00159894	0.99341556	0.99709367
ENSG0000016	AGGF1	protein_codir	-0.00176209	0.99067732	0.99528981
ENSG0000017	DHX36	protein_codir	-0.00179542	0.98676464	0.9934186

ENSG0000018	SLC38A3	protein_codir	-0.00180688	0.99777404	0.99943857
ENSG0000016	IFFO2	protein_codir	-0.00185995	0.99158078	0.99579905
ENSG0000012	MED1	protein_codir	-0.00199075	0.9888957	0.9943153
ENSG0000025	RP11-571M6.	protein_codir	-0.00199341	0.99861679	0.99963018
ENSG0000018	ZNF730	protein_codir	-0.00202908	0.99732129	0.99923306
ENSG0000011	NDUFB3	protein_codir	-0.0021198	0.98633314	0.9931652
ENSG0000025	RP11-867G23	lncRNA	-0.00220182	0.99411426	0.99743626
ENSG0000020	MBD5	protein_codir	-0.0022124	0.98838592	0.99407348
ENSG0000018	ACBD3	protein_codir	-0.00221861	0.98837858	0.99407348
ENSG0000013	GDPD2	protein_codir	-0.00222756	0.99866713	0.99963535
ENSG0000009	WDR7	protein_codir	-0.00234587	0.98853859	0.99414063
ENSG0000016	GHDC	protein_codir	-0.00245548	0.98108299	0.989755
ENSG0000019	ZGPAT	protein_codir	-0.00257989	0.98942134	0.9943535
ENSG0000016	TPST1	protein_codir	-0.00259625	0.99215942	0.99616283
ENSG0000011	CAND1	protein_codir	-0.00262299	0.98134022	0.98990171
ENSG0000015	PRR14	protein_codir	-0.00272743	0.98863459	0.99417779
ENSG0000019	FAM110D	protein_codir	-0.00277939	0.99314655	0.99690026
ENSG0000011	UBE4A	protein_codir	-0.00289902	0.97497318	0.98585976
ENSG0000016	TEF	protein_codir	-0.00297861	0.99135148	0.99566973
ENSG0000014	ZC3HAV1L	protein_codir	-0.00298403	0.99197582	0.9960509
ENSG0000014	SLC39A4	protein_codir	-0.00302474	0.98790235	0.9937944
ENSG0000011	CBL	protein_codir	-0.00307569	0.98774469	0.99372478
ENSG0000013	TBRG4	protein_codir	-0.0030758	0.98405968	0.99196089
ENSG0000011	C1orf54	protein_codir	-0.00324034	0.985891	0.9929734
ENSG0000013	DENND4C	protein_codir	-0.00334746	0.98497114	0.99240881
ENSG0000012	GPR108	protein_codir	-0.00343172	0.95888482	0.97587969
ENSG0000016	UTP15	protein_codir	-0.00346554	0.97627137	0.98645016
ENSG0000023	RP11-554F20.	lncRNA	-0.00346656	0.99829056	0.99959925
ENSG0000027	SMIM11B	protein_codir	-0.00347205	0.9941861	0.99743626
ENSG0000016	STK36	protein_codir	-0.00365991	0.9872065	0.99352633
ENSG0000018	ZKSCAN3	protein_codir	-0.0036801	0.98705853	0.99352633
ENSG0000020	NAP1L4	protein_codir	-0.00372815	0.96894314	0.98188324
ENSG0000018	C14orf180	protein_codir	-0.0037536	0.99629447	0.99860841
ENSG0000017	NAIF1	protein_codir	-0.00377861	0.97835534	0.9878331
ENSG0000000	MED24	protein_codir	-0.00381492	0.97133794	0.98358828
ENSG0000025	LINC02821	lncRNA	-0.00384306	0.9879295	0.9937944
ENSG0000011	PTP4A1	protein_codir	-0.00401237	0.98654328	0.99330437
ENSG0000020	PRR3	protein_codir	-0.0040612	0.9818463	0.99030802
ENSG0000014	TMEM219	protein_codir	-0.00409529	0.97968925	0.98878233
ENSG0000012	PSMG2	protein_codir	-0.0041631	0.9492341	0.97039638
ENSG0000016	DTYMK	protein_codir	-0.0043291	0.97978239	0.98882455
ENSG0000010	WNT3	protein_codir	-0.00439885	0.98742992	0.99354515
ENSG0000004	FAM184B	protein_codir	-0.00465108	0.99503122	0.99792165
ENSG0000018	ARL17A	protein_codir	-0.00483674	0.98713902	0.99352633
ENSG0000026	RP11-848P1.4	lncRNA	-0.00487128	0.99472806	0.99776253
ENSG0000026	RP11-65J3.14	lncRNA	-0.00518732	0.99517404	0.99799241
ENSG0000009	PPP2R3C	protein_codir	-0.00519326	0.96023464	0.97660569

ENSG0000017	KCNA2	protein_codir	-0.00528017	0.98733927	0.99352633
ENSG0000010	VWA8	protein_codir	-0.00569799	0.9683525	0.98153925
ENSG0000028	C8orf44	lncRNA	-0.00586545	0.98614193	0.99308127
ENSG0000011	BRD8	protein_codir	-0.00588616	0.96338159	0.97858113
ENSG0000026	DNAJA2-DT	lncRNA	-0.0058956	0.99541267	0.99809705
ENSG0000012	F10	protein_codir	-0.00617442	0.9920918	0.99613114
ENSG0000013	TMEM63B	protein_codir	-0.00618842	0.93632736	0.96225317
ENSG0000014	GMPPA	protein_codir	-0.00621614	0.97408505	0.98521419
ENSG0000019	ZNF485	protein_codir	-0.00632923	0.98080696	0.98962111
ENSG0000013	SQOR	protein_codir	-0.00633776	0.96426487	0.97919025
ENSG0000016	E4F1	protein_codir	-0.00635306	0.96931071	0.98207556
ENSG0000010	LMBR1	protein_codir	-0.00638108	0.96628962	0.98038447
ENSG0000028	NOTCH2NLC	protein_codir	-0.00639569	0.97813783	0.9876857
ENSG0000020	GPANK1	protein_codir	-0.00641028	0.95003306	0.97062636
ENSG0000018	ADIPOQ	protein_codir	-0.00655206	0.99466605	0.99773657
ENSG0000011	TMEM59	protein_codir	-0.00657195	0.96171505	0.97764334
ENSG0000025	LINC02757	lncRNA	-0.00661647	0.98711109	0.99352633
ENSG0000006	TAF2	protein_codir	-0.00664071	0.95751988	0.97497348
ENSG0000022	MELTF-AS1	lncRNA	-0.00677033	0.98729872	0.99352633
ENSG0000020	TRIQK	protein_codir	-0.00706844	0.96266799	0.97822339
ENSG0000005	ZNF280C	protein_codir	-0.00713923	0.95422473	0.97310911
ENSG0000013	HAUS2	protein_codir	-0.00730386	0.94099713	0.96525072
ENSG0000009	SPG21	protein_codir	-0.00766139	0.93788162	0.96309218
ENSG0000024	AC098820.4	lncRNA	-0.00778475	0.99415784	0.99743626
ENSG0000026	MANEA-DT	lncRNA	-0.00791048	0.98697457	0.99352633
ENSG0000012	EBPL	protein_codir	-0.00800306	0.95557279	0.97394445
ENSG0000014	CCZ1B	protein_codir	-0.00800499	0.96738478	0.98085174
ENSG0000017	FGG	protein_codir	-0.00811398	0.99542186	0.99809705
ENSG0000026	LINC00621	lncRNA	-0.00820803	0.98954479	0.99444138
ENSG0000019	NBR2	lncRNA	-0.00821236	0.97240693	0.9843816
ENSG0000022	C1QTNF5	protein_codir	-0.00832196	0.97180784	0.98395589
ENSG0000017	ZNF524	protein_codir	-0.00850787	0.94504741	0.96800237
ENSG0000009	MAST3	protein_codir	-0.00867567	0.97560491	0.98606534
ENSG0000019	H4-16	protein_codir	-0.00870992	0.97524505	0.98604274
ENSG0000023	GS1-124K5.4	lncRNA	-0.0088395	0.97259601	0.98439304
ENSG0000016	NUDT16L1	protein_codir	-0.00884756	0.93773752	0.96298001
ENSG0000013	PPIL4	protein_codir	-0.00891144	0.9593059	0.97609759
ENSG0000009	P2RX6	protein_codir	-0.00891242	0.97743199	0.98718951
ENSG0000018	GNB1L	protein_codir	-0.00891411	0.95243814	0.97200493
ENSG0000025	LINC01303	lncRNA	-0.008922	0.98892478	0.9943153
ENSG0000028	RP11-229A12	lncRNA	-0.00892273	0.99422795	0.99744201
ENSG0000015	AGAP1	protein_codir	-0.00895507	0.96813283	0.98142218
ENSG0000027	RP5-1024N4.4	lncRNA	-0.00910288	0.98852533	0.99414063
ENSG0000010	MTPN	protein_codir	-0.00930147	0.94859104	0.97009297
ENSG0000014	BIN3	protein_codir	-0.00933881	0.95763126	0.97498209
ENSG0000018	KLHL17	protein_codir	-0.00937816	0.97406261	0.98521419
ENSG0000010	OCIAD1	protein_codir	-0.00946693	0.89828917	0.9395781

ENSG0000014	SURF4	protein_codir	-0.00956506	0.95688219	0.97448789
ENSG000000(	RECQL	protein_codir	-0.00968574	0.9398889	0.96436462
ENSG000000(	ZNF263	protein_codir	-0.00968921	0.92371421	0.9551133
ENSG0000017	OXSR1	protein_codir	-0.0097323	0.95131622	0.97146987
ENSG0000013	DHX34	protein_codir	-0.0098546	0.95647298	0.97432373
ENSG0000016	DEPP1	protein_codir	-0.00987689	0.98420292	0.9919968
ENSG0000028	RP11-151A6.6	lncRNA	-0.00990881	0.99325667	0.99697457
ENSG0000027	CTD-2378E12.	lncRNA	-0.00991093	0.98268317	0.99104353
ENSG0000006	MED29	protein_codir	-0.00995477	0.9329025	0.96051469
ENSG0000016	LUZP1	protein_codir	-0.00998594	0.95876747	0.97579621
ENSG000000(	MAPK8IP2	protein_codir	-0.00999152	0.9774262	0.98718951
ENSG0000027	RP11-338I21.:	lncRNA	-0.01006715	0.99593552	NA
ENSG0000017	DRC3	protein_codir	-0.01008581	0.97322134	0.98473728
ENSG0000013	SLC35A5	protein_codir	-0.01012898	0.94890266	0.97022513
ENSG0000024	PDCD6	protein_codir	-0.01013441	0.91697454	0.95077846
ENSG0000013	DHX9	protein_codir	-0.01016407	0.94169131	0.96585518
ENSG0000015	AHCTF1	protein_codir	-0.01022674	0.94858171	0.97009297
ENSG0000019	SELENOT	protein_codir	-0.01022971	0.93649745	0.9623506
ENSG0000026	RP1-265C24.8	lncRNA	-0.01041623	0.98470436	0.99224861
ENSG0000017	SLC35A4	protein_codir	-0.01042127	0.90003319	0.94020108
ENSG0000015	VTI1A	protein_codir	-0.0104523	0.92527444	0.95583657
ENSG0000027	CH17-189H20	lncRNA	-0.01048722	0.96267463	0.97822339
ENSG0000026	RP5-1107A17	lncRNA	-0.01066872	0.9890248	0.9943153
ENSG0000014	STXBP5L	protein_codir	-0.01069728	NA	NA
ENSG0000017	ZNF680	protein_codir	-0.0106998	0.9641117	0.9790861
ENSG0000010	CUL2	protein_codir	-0.01079455	0.92896912	0.95778592
ENSG0000014	SNRNP200	protein_codir	-0.01088279	0.92103236	0.95335624
ENSG0000013	GPALPP1	protein_codir	-0.01098043	0.93801791	0.96312471
ENSG0000017	RBIS	protein_codir	-0.01099352	0.91443886	0.94911132
ENSG0000011	MEF2D	protein_codir	-0.01101945	0.9540095	0.97297032
ENSG0000014	STRIP1	protein_codir	-0.0110558	0.92445831	0.95549948
ENSG000000(	DNAJC11	protein_codir	-0.0110867	0.92108295	0.95335624
ENSG0000022	SNHG26	lncRNA	-0.01119607	0.9596497	0.9762372
ENSG0000016	GRPEL2	protein_codir	-0.01125427	0.94256289	0.96635439
ENSG0000004	ARID1B	protein_codir	-0.01134319	0.92047168	0.95300925
ENSG0000016	APLF	protein_codir	-0.01138504	0.94678494	0.96910021
ENSG0000016	ING5	protein_codir	-0.01147464	0.94220185	0.96610914
ENSG0000018	C15orf62	protein_codir	-0.01148731	0.97040286	0.98281548
ENSG0000016	CASKIN1	protein_codir	-0.01158238	0.97733064	0.98718951
ENSG0000014	NAPRT	protein_codir	-0.01181219	0.93950437	0.96411334
ENSG0000019	ZXDA	protein_codir	-0.01182682	0.95355672	0.97284569
ENSG0000017	MTX1	protein_codir	-0.01185595	0.93477564	0.96157798
ENSG0000009	HAUS4	protein_codir	-0.01193864	0.94595544	0.9685386
ENSG0000013	TOR1A	protein_codir	-0.01194796	0.91919965	0.95212054
ENSG0000023	NFE4	lncRNA	-0.0119807	0.98397888	0.99191564
ENSG0000026	CTC-425F1.4	lncRNA	-0.01208353	0.99004546	0.99479975
ENSG0000014	G6PC3	protein_codir	-0.01219114	0.94224214	0.96610914

ENSG0000020	THUMPD3-AS	lncRNA	-0.01222916	0.96353978	0.97866984
ENSG0000007	ELP1	protein_codir	-0.01229225	0.92548651	0.95583657
ENSG0000017	CTXN1	protein_codir	-0.01233357	0.97392051	0.98519208
ENSG0000014	WDR89	protein_codir	-0.01238938	0.94791862	0.96982932
ENSG0000026	EFCAB6-DT	lncRNA	-0.01239628	0.98789261	0.9937944
ENSG0000028	RP11-331F9.1	lncRNA	-0.0124379	0.98416121	0.9919968
ENSG0000013	RP11-84C10.2	lncRNA	-0.01247499	0.99320006	NA
ENSG0000017	WIPF2	protein_codir	-0.01248998	0.87596482	0.92642786
ENSG0000022	PPP2R2A	protein_codir	-0.01252063	0.89250983	0.93591714
ENSG0000023	ACBD6	protein_codir	-0.01254357	0.89223496	0.93577101
ENSG0000025	RP11-968O1.5	lncRNA	-0.01269876	0.96267524	0.97822339
ENSG0000025	RP1-240K6.3	lncRNA	-0.0127197	0.99357703	0.99718741
ENSG0000026	FBXL19-AS1	lncRNA	-0.01286655	0.95359022	0.97284569
ENSG0000010	FNDC3A	protein_codir	-0.01290095	0.94046493	0.96488397
ENSG0000022	AC007319.1	lncRNA	-0.01292931	0.96942618	0.98215652
ENSG0000008	ZNF324	protein_codir	-0.01296731	0.90745562	0.94488141
ENSG0000018	SLC35C1	protein_codir	-0.01306797	0.93843808	0.96336625
ENSG0000016	ZNF180	protein_codir	-0.01309166	0.94673795	0.96908802
ENSG0000019	GDAP2	protein_codir	-0.01321689	0.90477091	0.94350874
ENSG0000013	RPLP1	protein_codir	-0.01321916	0.94225373	0.96610914
ENSG0000016	GAPVD1	protein_codir	-0.01328569	0.88268349	0.93061413
ENSG0000012	GALR3	protein_codir	-0.01337303	0.99374018	NA
ENSG0000021	LINC01521	lncRNA	-0.01344265	0.95927171	0.97609759
ENSG0000013	AP3B1	protein_codir	-0.01348472	0.93342937	0.9608337
ENSG0000013	PTCD3	protein_codir	-0.01350613	0.93120062	0.95938377
ENSG0000015	DPY19L4	protein_codir	-0.01355767	0.93507651	0.96159697
ENSG0000014	NXPE3	protein_codir	-0.0135591	0.93797511	0.96312471
ENSG0000015	DHX37	protein_codir	-0.01364045	0.91710725	0.95086406
ENSG0000015	WHAMM	protein_codir	-0.01374305	0.91910156	0.95209035
ENSG0000011	ATP5F1B	protein_codir	-0.01409308	0.9220503	0.95403571
ENSG0000027	NATD1	protein_codir	-0.01413668	0.94080036	0.96515641
ENSG0000009	HNRNPM	protein_codir	-0.01415288	0.91503283	0.94958508
ENSG0000016	TM4SF1	protein_codir	-0.01436538	0.97289237	0.98451261
ENSG0000011	SYF2	protein_codir	-0.01436905	0.92140227	0.95361526
ENSG0000011	SUMO1	protein_codir	-0.01465932	0.81524842	0.88618821
ENSG0000014	NONO	protein_codir	-0.01471422	0.81913631	0.88900602
ENSG0000013	NDUFA9	protein_codir	-0.01483004	0.89831423	0.9395781
ENSG0000012	SOX4	protein_codir	-0.01484147	0.93900821	0.96381905
ENSG0000021	MYL5	protein_codir	-0.01523111	0.95990433	0.97641357
ENSG0000013	LGALS3	protein_codir	-0.01523756	0.92625393	0.95645033
ENSG0000016	TNIP2	protein_codir	-0.01528427	0.91427578	0.9491105
ENSG0000027	RP11-677M14	lncRNA	-0.01530097	0.96684604	0.98061418
ENSG0000017	CSTF3	protein_codir	-0.01532592	0.92404593	0.95525347
ENSG0000010	TELO2	protein_codir	-0.01533683	0.93722819	0.96270752
ENSG0000021	PPP1R3G	protein_codir	-0.01535555	0.95606436	0.97408601
ENSG0000017	ZNF664	protein_codir	-0.01545808	0.93485948	0.96157798
ENSG0000013	SPATA6	protein_codir	-0.01553638	0.94729847	0.96944621

ENSG0000011	HMGN3	protein_codir	-0.01555094	0.91117156	0.94705595
ENSG0000010	BLVRA	protein_codir	-0.01560899	0.88691537	0.93299065
ENSG0000016	POLR2J3	protein_codir	-0.01561689	0.95647406	0.97432373
ENSG0000007	TIGAR	protein_codir	-0.01572986	0.92461868	0.95558346
ENSG0000004	POLR2B	protein_codir	-0.01584672	0.88459968	0.93144155
ENSG0000025	AP002954.3	lncRNA	-0.01586259	0.96979395	0.98238498
ENSG0000014	TENM2	protein_codir	-0.01589907	0.97463864	0.98559364
ENSG0000007	TFRC	protein_codir	-0.01603897	0.94846545	0.97009297
ENSG0000024	ALG1L9P	lncRNA	-0.01622163	0.96351503	0.97866984
ENSG0000016	THAP9	protein_codir	-0.01624079	0.94098636	0.96525072
ENSG0000018	MRPL41	protein_codir	-0.0162801	0.93632448	0.96225317
ENSG0000027	RP11-167P11.	lncRNA	-0.01632408	0.98303077	0.99132169
ENSG0000006	ACSL4	protein_codir	-0.01635916	0.94603928	0.96855234
ENSG0000016	NEMF	protein_codir	-0.01640638	0.89737101	0.93901827
ENSG0000003	ZZZ3	protein_codir	-0.01657981	0.89493155	0.93735341
ENSG0000024	MALINC1	lncRNA	-0.01658966	0.93663989	0.96241419
ENSG0000013	DDX56	protein_codir	-0.01662957	0.93161374	0.95961704
ENSG0000013	GTPBP3	protein_codir	-0.01668676	0.92286001	0.95458743
ENSG0000021	SH3D21	protein_codir	-0.01678396	0.94861869	0.97009297
ENSG0000011	PCCB	protein_codir	-0.01686872	0.92751755	0.95696805
ENSG0000011	RXYLT1	protein_codir	-0.01691988	0.91071544	0.94686714
ENSG0000011	EDEM3	protein_codir	-0.01697268	0.92773141	0.95705808
ENSG0000026	RP1-228H13.5	lncRNA	-0.01708414	0.95135109	0.97146987
ENSG0000011	HPS5	protein_codir	-0.01713437	0.92312934	0.95468676
ENSG0000007	BUD23	protein_codir	-0.01720037	0.89389796	0.93680371
ENSG0000022	GAS1RR	lncRNA	-0.01725519	0.96412685	0.9790861
ENSG0000025	CTD-3074O7.5	lncRNA	-0.01730014	0.94333179	0.96689145
ENSG0000012	DCTD	protein_codir	-0.01737887	0.85761801	0.91493393
ENSG0000006	DHX8	protein_codir	-0.01738021	0.86178646	0.91761439
ENSG0000014	SNX27	protein_codir	-0.01739877	0.88762675	0.93341903
ENSG0000015	NUS1	protein_codir	-0.01741712	0.91935566	0.95224643
ENSG0000012	CRY2	protein_codir	-0.017464	0.92199099	0.95401009
ENSG0000004	SPATA7	protein_codir	-0.01748976	0.93824946	0.96325503
ENSG0000011	STAG1	protein_codir	-0.0174983	0.90558136	0.94381937
ENSG0000018	TMEM201	protein_codir	-0.01754817	0.88937212	0.93419857
ENSG0000017	FBXW8	protein_codir	-0.01771606	0.88894652	0.9340189
ENSG0000019	MYL6B	protein_codir	-0.01775782	0.91818521	0.95156829
ENSG0000013	BTF3L4	protein_codir	-0.01786588	0.85291623	0.91247086
ENSG0000011	CUTA	protein_codir	-0.01794252	0.90217054	0.94182826
ENSG0000022	LINC00571	lncRNA	-0.01798003	0.95852576	0.97576567
ENSG0000023	LINC01239	lncRNA	-0.01817654	0.9755997	0.98606534
ENSG0000019	CTR9	protein_codir	-0.01823421	0.87931118	0.9283504
ENSG0000010	MTPAP	protein_codir	-0.01835497	0.85871317	0.9158104
ENSG0000026	LINC00526	lncRNA	-0.01841479	0.92672492	0.95650756
ENSG0000012	SNRPB2	protein_codir	-0.0185511	0.86750003	0.92133933
ENSG0000010	NPDC1	protein_codir	-0.01858029	0.93176616	0.95966668
ENSG0000011	ATP6V0E1	protein_codir	-0.01866373	0.80365675	0.87828906

ENSG0000014	SSRP1	protein_codir	-0.0186747	0.81687724	0.88736527
ENSG0000010	ENO3	protein_codir	-0.01870234	0.95175479	0.97173338
ENSG0000012	ITPA	protein_codir	-0.01872532	0.92166944	0.95385604
ENSG0000012	KDM5C	protein_codir	-0.01879372	0.85600661	0.91391197
ENSG0000017	RALGAPB	protein_codir	-0.0189145	0.8763624	0.92661854
ENSG0000012	CDHR3	protein_codir	-0.01934625	0.93846243	0.96336625
ENSG0000026	RP11-49K24.6	protein_codir	-0.01935857	0.96572611	0.98006155
ENSG0000013	SRPK2	protein_codir	-0.01935967	0.83772767	0.90263082
ENSG0000010	DNTTIP1	protein_codir	-0.01940382	0.86347622	0.91879698
ENSG0000016	NFASC	protein_codir	-0.01954001	0.95440517	0.97320388
ENSG0000013	ZFC3H1	protein_codir	-0.01979598	0.90322934	0.94264019
ENSG0000011	ZNF410	protein_codir	-0.01991094	0.89901763	0.93983381
ENSG0000016	TMEM41A	protein_codir	-0.01992094	0.78300132	0.86427976
ENSG0000013	LATS1	protein_codir	-0.01998758	0.90028775	0.9403308
ENSG0000013	POMT1	protein_codir	-0.02006496	0.90901188	0.94586001
ENSG0000026	RP11-290F24.	lncRNA	-0.02007948	0.96776648	0.98110048
ENSG0000023	C9orf147	lncRNA	-0.02037678	0.97250528	0.98438787
ENSG0000011	NECTIN1	protein_codir	-0.02047821	0.91673983	0.95063094
ENSG0000015	DISP1	protein_codir	-0.02055636	0.91676342	0.95063094
ENSG0000000	KRIT1	protein_codir	-0.02096069	0.82370865	0.89283147
ENSG0000010	AMBP	protein_codir	-0.02106779	0.98581622	0.99293428
ENSG0000004	PREX2	protein_codir	-0.02108777	0.94320746	0.96689145
ENSG0000010	GARS1	protein_codir	-0.02109669	0.89311189	0.93629964
ENSG0000017	GABARAP	protein_codir	-0.02109758	0.84680702	0.90869148
ENSG0000028	RP11-90D4.7	lncRNA	-0.02109984	0.98327079	0.99145514
ENSG0000017	B3GALT6	protein_codir	-0.02111832	0.8965875	0.93848284
ENSG0000024	MICAL3	protein_codir	-0.021127	0.9310889	0.95932687
ENSG0000012	USP9X	protein_codir	-0.02114061	0.91337756	0.94872281
ENSG0000027	CCL15	protein_codir	-0.02115027	0.98934194	0.99434486
ENSG0000007	UFD1	protein_codir	-0.02118937	0.87160219	0.92370703
ENSG0000016	POGLUT1	protein_codir	-0.02126025	0.87587856	0.92639587
ENSG0000021	CLDN9	protein_codir	-0.0213722	0.97610442	0.98635364
ENSG0000015	MERTK	protein_codir	-0.02149981	0.94350571	0.96698248
ENSG0000018	IST1	protein_codir	-0.02153028	0.78069664	0.86289827
ENSG0000008	ZNF446	protein_codir	-0.02154537	0.89621208	0.93828515
ENSG0000011	SUB1	protein_codir	-0.02157213	0.87443146	0.92553351
ENSG0000014	ETFA	protein_codir	-0.02159448	0.79281336	0.87109178
ENSG0000018	BRCC3	protein_codir	-0.02161011	0.87458952	0.92555894
ENSG0000023	RP11-367N14	lncRNA	-0.02169692	0.95931097	0.97609759
ENSG0000016	MGAT4B	protein_codir	-0.02170127	0.90634932	0.94426345
ENSG0000028	EIF1B-AS1	lncRNA	-0.0218416	0.92544746	0.95583657
ENSG0000023	GORAB-AS1	lncRNA	-0.02185384	0.95955231	0.97623525
ENSG0000009	CRKL	protein_codir	-0.02190615	0.83272551	0.89928607
ENSG0000012	PSMA1	protein_codir	-0.02190774	0.8038834	0.87846724
ENSG0000014	PPP1R18	protein_codir	-0.02199166	0.86397124	0.91911928
ENSG0000028	RP11-539I5.5	lncRNA	-0.02204854	0.98091698	0.98965329
ENSG0000027	RP11-140I16.:	lncRNA	-0.02219498	0.97026109	0.98275072

ENSG0000011	UBE2B	protein_codir	-0.02224463	0.85529595	0.91336696
ENSG0000013	CASP6	protein_codir	-0.02229779	0.81672074	0.88726506
ENSG0000011	CAMKK2	protein_codir	-0.02231366	0.88452606	0.93139954
ENSG0000010	PIH1D1	protein_codir	-0.02246952	0.84730471	0.90911958
ENSG0000015	CDYL	protein_codir	-0.02252171	0.84612979	0.90837484
ENSG0000008	ERGIC2	protein_codir	-0.0225248	0.85988166	0.91631084
ENSG0000018	RNF220	protein_codir	-0.02255258	0.78775908	0.86743635
ENSG0000018	FAAP100	protein_codir	-0.02257775	0.89913758	0.93983424
ENSG0000011	SLF2	protein_codir	-0.02279398	0.8964027	0.93839609
ENSG0000007	ADGRL1	protein_codir	-0.02280189	0.9253649	0.95583657
ENSG0000019	SDHAF3	protein_codir	-0.02299018	0.84051327	0.90472185
ENSG0000024	NECTIN3-AS1	lncRNA	-0.02313734	0.96248311	0.97816075
ENSG0000017	FBXO45	protein_codir	-0.02319621	0.8723309	0.92423105
ENSG0000024	GNG10	protein_codir	-0.02335849	0.86704205	0.92103002
ENSG0000015	MMAA	protein_codir	-0.02340835	0.90549359	0.94376351
ENSG0000011	UTP25	protein_codir	-0.02345554	0.87869798	0.92793308
ENSG0000010	IFT20	protein_codir	-0.02349327	0.85991221	0.91631084
ENSG0000000	RPUSD1	protein_codir	-0.02358169	0.89024744	0.93462849
ENSG0000010	AAGAB	protein_codir	-0.02370279	0.82489422	0.89368394
ENSG0000003	FAM136A	protein_codir	-0.02373405	0.85940675	0.91609025
ENSG0000015	NAE1	protein_codir	-0.02380183	0.80831387	0.88139081
ENSG0000027	HEIH	lncRNA	-0.02380886	0.80928398	0.88216203
ENSG0000016	SMCO2	protein_codir	-0.02389797	0.96739255	0.98085174
ENSG0000026	AF001548.5	lncRNA	-0.02391411	0.96464771	0.97943498
ENSG0000010	EEA1	protein_codir	-0.02412596	0.92460665	0.95558346
ENSG0000016	ZBTB49	protein_codir	-0.02421971	0.88048295	0.92911872
ENSG0000004	DCUN1D1	protein_codir	-0.02424183	0.85204924	0.91194296
ENSG0000027	CTD-2653M23	lncRNA	-0.02428764	0.96680976	0.98061418
ENSG0000011	IRF2BPL	protein_codir	-0.02432726	0.91420405	0.9491105
ENSG0000018	GDPGP1	protein_codir	-0.02432825	0.92045592	0.95300925
ENSG0000023	H2BC15	protein_codir	-0.02439656	0.93673355	0.96241419
ENSG0000005	ANAPC4	protein_codir	-0.02442744	0.86409291	0.91911928
ENSG0000012	TAS2R4	protein_codir	-0.02452115	0.96952366	0.98218936
ENSG0000011	RHAG	protein_codir	-0.02454017	0.98425774	0.99199854
ENSG0000015	UBALD1	protein_codir	-0.02462472	0.85056244	0.91099105
ENSG0000009	KLHL22	protein_codir	-0.02472936	0.86372143	0.91898709
ENSG0000015	WASF2	protein_codir	-0.02493338	0.78009525	0.86253926
ENSG0000017	SRGAP2C	protein_codir	-0.02496303	0.88103135	0.92938768
ENSG0000010	ACD	protein_codir	-0.02496333	0.84839453	0.90970778
ENSG0000027	RP11-266K4.1	lncRNA	-0.02503878	0.92601945	0.95627971
ENSG0000015	ACSL1	protein_codir	-0.02513762	0.93513739	0.96159697
ENSG0000025	AC009120.6	lncRNA	-0.02519791	0.94656967	0.96902351
ENSG0000016	GATAD2A	protein_codir	-0.02536848	0.8495522	0.91049844
ENSG0000020	AGAP9	protein_codir	-0.02537586	0.9320878	0.95992636
ENSG0000012	GTDC1	protein_codir	-0.02547057	0.83172185	0.89861358
ENSG0000017	LSM3	protein_codir	-0.02547185	0.80300727	0.87799615
ENSG0000017	CDC26	protein_codir	-0.02558811	0.82716836	0.8953799

ENSG0000016	ZNF592	protein_codir	-0.02563324	0.79481584	0.87247504
ENSG0000016	ERCC3	protein_codir	-0.02565307	0.82587181	0.89450383
ENSG0000016	HCN1	protein_codir	-0.02567969	0.96674328	0.98060054
ENSG0000017	GEMIN4	protein_codir	-0.0258404	0.87532823	0.92605684
ENSG0000027	RP11-108M9.	lncRNA	-0.02595869	0.98815034	0.99394415
ENSG0000000	IBTK	protein_codir	-0.02596411	0.87219531	0.92419376
ENSG0000023	PRH1	protein_codir	-0.02602476	0.91891329	0.95200244
ENSG0000013	IL33	protein_codir	-0.02629405	0.94690073	0.96914689
ENSG0000018	MTF1	protein_codir	-0.02631057	0.8373624	0.90232891
ENSG0000017	SH2B1	protein_codir	-0.02647594	0.88651228	0.93273421
ENSG0000027	RP11-78J21.7	lncRNA	-0.02650598	0.88375996	0.93115014
ENSG0000016	ZNF3	protein_codir	-0.0266472	0.78783416	0.86748444
ENSG0000016	TMEM208	protein_codir	-0.02669058	0.86415467	0.91911928
ENSG0000018	ENTPD8	protein_codir	-0.02670816	0.97478768	0.98570827
ENSG0000013	EIF5A	protein_codir	-0.02673193	0.88999685	0.93452376
ENSG0000017	C18orf32	protein_codir	-0.02674478	0.84098448	0.90493966
ENSG0000009	CDC23	protein_codir	-0.02682204	0.85047379	0.91096278
ENSG0000018	SERINC4	protein_codir	-0.02684254	0.96509535	0.97974541
ENSG0000006	WDR18	protein_codir	-0.02686195	0.89869168	0.93975098
ENSG0000007	GBA2	protein_codir	-0.0269792	0.8690488	0.92233415
ENSG0000011	PDE10A	protein_codir	-0.02704497	0.93801487	0.96312471
ENSG0000009	BCL2L13	protein_codir	-0.02710799	0.81468139	0.88606025
ENSG0000006	ATXN3	protein_codir	-0.02711832	0.81009514	0.88274067
ENSG0000010	INTS2	protein_codir	-0.02712528	0.85861933	0.91574568
ENSG0000011	ACADM	protein_codir	-0.02720527	0.84744852	0.90915075
ENSG0000016	WDR82	protein_codir	-0.02720563	0.77898199	0.86165329
ENSG0000023	RP11-88I18.2	lncRNA	-0.02726394	0.90449959	0.94333265
ENSG0000014	POLR2D	protein_codir	-0.027417	0.77412182	0.85799537
ENSG0000022	TMEM185B	protein_codir	-0.02758531	0.85136768	0.91156673
ENSG0000017	ESRRA	protein_codir	-0.02777352	0.87524779	0.92601878
ENSG0000013	G3BP2	protein_codir	-0.02798954	0.86824318	0.92188047
ENSG0000015	SAMD8	protein_codir	-0.02802078	0.87401113	0.92523044
ENSG0000013	TRAF7	protein_codir	-0.02804649	0.80306459	0.87802407
ENSG0000027	bP-21264C1.2	lncRNA	-0.02810369	0.92315562	0.95468676
ENSG0000001	SPRTN	protein_codir	-0.02818448	0.7939792	0.87201947
ENSG0000016	DCAF13	protein_codir	-0.02831918	0.83276626	0.89928607
ENSG0000012	NR2C1	protein_codir	-0.02833328	0.87089079	0.92331633
ENSG0000016	LETM1	protein_codir	-0.02842687	0.81404535	0.88561697
ENSG0000024	LINC02101	lncRNA	-0.02848447	0.98690635	NA
ENSG0000021	MEG3	lncRNA	-0.02851343	0.95204148	0.97188741
ENSG0000008	BZW1	protein_codir	-0.0286055	0.88165132	0.9298659
ENSG0000010	TNFAIP1	protein_codir	-0.0286164	0.88819257	0.93365857
ENSG0000013	GALNT8	protein_codir	-0.02868223	0.94181908	0.96595035
ENSG0000012	MT2A	protein_codir	-0.02868274	0.96396255	0.97899124
ENSG0000016	HINT1	protein_codir	-0.02872601	0.83110763	0.89822487
ENSG0000028	RP11-413H22	protein_codir	-0.02878032	0.99141597	0.99566973
ENSG0000010	XPNPEP1	protein_codir	-0.02879687	0.76109946	0.84915039

ENSG0000018	DDX51	protein_codir	-0.02881414	0.86588	0.92029113
ENSG0000010	RAB5C	protein_codir	-0.02898448	0.84806346	0.9095097
ENSG0000018	PPIL6	protein_codir	-0.02900166	0.91907466	0.95209035
ENSG0000028	RP11-538D15	lncRNA	-0.02904025	0.99012849	NA
ENSG0000010	CLN5	protein_codir	-0.02918563	0.81793225	0.88809217
ENSG0000013	ANKRD52	protein_codir	-0.0293285	0.80656199	0.88027985
ENSG0000018	PLSCR3	protein_codir	-0.02935274	0.81319012	0.8851703
ENSG0000010	GRWD1	protein_codir	-0.02939857	0.85827204	0.91548133
ENSG0000016	RAVER1	protein_codir	-0.02940082	0.83333411	0.8996165
ENSG0000022	CTA-292E10.6	lncRNA	-0.02941456	0.93264943	0.96036153
ENSG0000010	ZNF423	protein_codir	-0.02948147	0.91778906	0.9513373
ENSG0000017	RSRC1	protein_codir	-0.02952466	0.74634031	0.83790558
ENSG0000027	CTD-3128G10	lncRNA	-0.02957013	0.95576808	0.97399761
ENSG0000014	FBXO25	protein_codir	-0.02961491	0.85394015	0.91288916
ENSG0000015	OBI1	protein_codir	-0.02967411	0.87127983	0.92357802
ENSG0000015	PANK4	protein_codir	-0.02967671	0.80452599	0.87878699
ENSG0000002	ALAS1	protein_codir	-0.02996973	0.88529864	0.93199986
ENSG0000010	AXIN1	protein_codir	-0.03000255	0.83212824	0.89887684
ENSG0000010	GDAP1	protein_codir	-0.03008288	0.90665382	0.94447381
ENSG0000007	DPP8	protein_codir	-0.0300829	0.86180004	0.91761439
ENSG0000008	PDS5B	protein_codir	-0.03014664	0.82461934	0.89356743
ENSG0000016	IFI27L1	protein_codir	-0.03021639	0.86089028	0.9170393
ENSG0000026	LINC02073	lncRNA	-0.03040011	0.96544765	0.97993203
ENSG0000007	CLCN4	protein_codir	-0.03049705	0.92482507	0.95572524
ENSG0000015	LARP1	protein_codir	-0.03056925	0.74524214	0.8373439
ENSG0000023	RAP2C-AS1	lncRNA	-0.03092911	0.91712594	0.95086406
ENSG0000011	ATL2	protein_codir	-0.031154	0.84484109	0.90739271
ENSG0000012	KDM3B	protein_codir	-0.03121537	0.79368404	0.87182395
ENSG0000013	FPGS	protein_codir	-0.03126384	0.84674969	0.90866526
ENSG0000016	PRKCI	protein_codir	-0.03141761	0.87101454	0.92336768
ENSG0000019	ASB13	protein_codir	-0.03174233	0.8802792	0.92904084
ENSG0000014	AMT	protein_codir	-0.03175273	0.92788848	0.95709488
ENSG0000007	PDCD2	protein_codir	-0.03177349	0.77735452	0.86054236
ENSG0000011	OSBPL9	protein_codir	-0.03195579	0.86683542	0.92088135
ENSG0000016	BTRC	protein_codir	-0.03200353	0.76659321	0.85286814
ENSG0000014	INTS4	protein_codir	-0.03202309	0.82267038	0.89183437
ENSG0000028	RP11-736K12.	protein_codir	-0.03205429	0.98908731	NA
ENSG0000025	RP11-619A14	lncRNA	-0.03212504	0.98696333	NA
ENSG0000019	GJC2	protein_codir	-0.03219059	0.92900383	0.95778595
ENSG0000022	PANK2-AS1	lncRNA	-0.03222234	0.96719707	0.98083357
ENSG0000010	TTI1	protein_codir	-0.03239186	0.77496627	0.85879346
ENSG0000027	PKD1P6-NPIP	lncRNA	-0.0326754	0.89380192	0.93677414
ENSG0000023	RP11-295G20	lncRNA	-0.0327056	0.89081151	0.93491713
ENSG0000019	TTC30B	protein_codir	-0.03274191	0.9254763	0.95583657
ENSG0000017	PHC3	protein_codir	-0.03281463	0.83437052	0.90027466
ENSG0000005	SEC61A1	protein_codir	-0.032829	0.84291679	0.90620691
ENSG0000012	MRS2	protein_codir	-0.0329398	0.79097567	0.8699378

ENSG0000016	TRAPPC9	protein_codir	-0.03299018	0.71214773	0.81342873
ENSG0000012	RCL1	protein_codir	-0.03307028	0.82474379	0.89356743
ENSG0000011	MAN1C1	protein_codir	-0.03312155	0.87447762	0.9255469
ENSG0000003	PNPLA6	protein_codir	-0.03317368	0.84755287	0.90915075
ENSG0000016	GTPBP8	protein_codir	-0.03318681	0.85670193	0.91437156
ENSG0000013	YME1L1	protein_codir	-0.03359068	0.78656071	0.86663502
ENSG0000013	ADCY3	protein_codir	-0.03365383	0.87725873	0.92717583
ENSG0000023	HOTAIRM1	lncRNA	-0.03382337	0.88414556	0.93128621
ENSG0000010	AVP	protein_codir	-0.03393505	0.98602114	NA
ENSG0000011	NCBP2	protein_codir	-0.03397569	0.6733452	0.78467407
ENSG0000016	COMMD8	protein_codir	-0.03402293	0.80701502	0.88056195
ENSG0000013	NUMA1	protein_codir	-0.03410681	0.79572554	0.87311344
ENSG0000011	RBBP5	protein_codir	-0.03416368	0.77357554	0.85756197
ENSG0000022	MIR181A2HG	lncRNA	-0.03420373	0.96969591	0.9823217
ENSG0000012	BMP2	protein_codir	-0.03423837	0.93921228	0.96394184
ENSG0000017	TRMT10C	protein_codir	-0.03423954	0.83612388	0.90150063
ENSG0000017	FRMD3	protein_codir	-0.03429775	0.89058483	0.93478089
ENSG0000010	UBE2W	protein_codir	-0.03470441	0.77236771	0.85677321
ENSG0000023	HRAT17	lncRNA	-0.0347169	0.96581502	0.98011576
ENSG0000012	HEATR5A	protein_codir	-0.03474865	0.84868375	0.90987435
ENSG0000008	MAPKAPK5	protein_codir	-0.03482146	0.76728228	0.85330194
ENSG0000002	SLC45A4	protein_codir	-0.03483721	0.83312285	0.89942359
ENSG0000018	ADGRB1	protein_codir	-0.03487289	0.91122418	0.94705595
ENSG0000010	CCDC47	protein_codir	-0.03491933	0.85653856	0.91433853
ENSG0000011	CILK1	protein_codir	-0.03493568	0.88728222	0.93326992
ENSG0000027	RP11-120K19.	lncRNA	-0.03498861	0.98164289	0.99013902
ENSG0000015	IGSF10	protein_codir	-0.03513439	0.95002251	0.97062636
ENSG0000017	SNHG29	lncRNA	-0.03519115	0.79268209	0.87106465
ENSG0000015	SLC35B2	protein_codir	-0.03532828	0.78865321	0.86807482
ENSG0000010	PDE4C	protein_codir	-0.03539662	0.95163759	0.97165466
ENSG0000014	HYOU1	protein_codir	-0.03542506	0.87474521	0.92568823
ENSG0000018	RAD51D	protein_codir	-0.03553766	0.80310997	0.87803207
ENSG0000026	AC015688.3	protein_codir	-0.03560185	0.98997571	0.99477626
ENSG0000026	ZNF790-AS1	lncRNA	-0.03561564	0.89920338	0.93984095
ENSG0000013	LRPPRC	protein_codir	-0.03567701	0.79982133	0.87574536
ENSG0000020	EFCAB7	protein_codir	-0.03581672	0.85932286	0.91604679
ENSG0000014	SLIT2	protein_codir	-0.0359442	0.91871769	0.95194262
ENSG0000026	MYL12-AS1	lncRNA	-0.03594435	0.96019911	0.97660552
ENSG0000028	RP11-592G13	lncRNA	-0.03602989	0.981921	0.99033551
ENSG0000012	EMG1	protein_codir	-0.03606989	0.72009817	0.81921083
ENSG0000014	CARM1	protein_codir	-0.03617331	0.73936192	0.83338133
ENSG0000011	USP9Y	protein_codir	-0.03620665	0.93626185	0.96225317
ENSG0000015	MMP14	protein_codir	-0.0362369	0.86175789	0.91761439
ENSG0000018	SUMO2	protein_codir	-0.03638008	0.64854701	0.76475551
ENSG0000017	SFN	protein_codir	-0.03657698	0.92724002	0.95682468
ENSG0000027	LINC01050	lncRNA	-0.03657966	0.9758086	0.98616295
ENSG0000026	CTD-2192J16.	lncRNA	-0.03666371	0.96777996	0.98110048

ENSG0000018	SMDT1	protein_codir	-0.03674505	0.85526659	0.91336696
ENSG0000011	NSL1	protein_codir	-0.03685517	0.78759988	0.86743396
ENSG0000026	CTB-92J24.2	lncRNA	-0.03691238	0.95143996	0.9715247
ENSG0000011	KRR1	protein_codir	-0.03703908	0.7604646	0.84861005
ENSG0000009	UPRT	protein_codir	-0.03714973	0.73184163	0.82760669
ENSG0000015	SLC2A13	protein_codir	-0.03724437	0.88861133	0.93395658
ENSG0000017	SLC22A13	protein_codir	-0.03724442	0.95851716	0.97576567
ENSG0000013	RCBTB1	protein_codir	-0.03727525	0.82513606	0.89391088
ENSG0000018	BLOC1S3	protein_codir	-0.03733113	0.81116174	0.88344981
ENSG0000016	DNAAF5	protein_codir	-0.03738208	0.76931827	0.85476465
ENSG0000023	LINC02535	lncRNA	-0.03748366	0.96302638	0.97829228
ENSG0000019	HNRNPAB	protein_codir	-0.03760267	0.84686353	0.90871681
ENSG0000019	MYO18A	protein_codir	-0.0377479	0.74850746	0.83965258
ENSG0000023	RP6-24A23.3	lncRNA	-0.03803026	0.9827576	NA
ENSG0000017	PTPRM	protein_codir	-0.03807773	0.8373793	0.90232891
ENSG0000023	NRAD1	lncRNA	-0.03815387	0.96919945	0.98202974
ENSG0000012	UBIAD1	protein_codir	-0.03815978	0.77949249	0.86204535
ENSG0000027	IER3-AS1	lncRNA	-0.038277	0.92650744	0.95648785
ENSG0000001	RGPD5	protein_codir	-0.03830646	0.88144184	0.92974694
ENSG0000016	LEMD2	protein_codir	-0.03839513	0.766984	0.85308353
ENSG0000026	ESRG	lncRNA	-0.03840974	0.97677953	0.98679069
ENSG0000026	RP11-467I17.1	lncRNA	-0.03848165	0.96674978	0.98060054
ENSG0000016	CLHC1	protein_codir	-0.03854878	0.88949776	0.9342128
ENSG0000013	LAMTOR5	protein_codir	-0.03855098	0.74351956	0.83615829
ENSG0000018	TCEA1	protein_codir	-0.03867475	0.76729207	0.85330194
ENSG0000021	NDUFS3	protein_codir	-0.03872001	0.67143696	0.78325594
ENSG0000027	RP11-474G23	lncRNA	-0.03878553	0.96078517	0.97694976
ENSG0000018	PTMA	protein_codir	-0.03881473	0.79418533	0.87218285
ENSG0000008	CDC14B	protein_codir	-0.03883655	0.86997981	0.92287292
ENSG0000019	C20orf96	protein_codir	-0.03905395	0.85917249	0.91602406
ENSG0000011	ACOT13	protein_codir	-0.03925872	0.77707217	0.86029875
ENSG0000028	RP11-692P14.	lncRNA	-0.03955024	0.95044212	0.97093654
ENSG0000011	ASF1A	protein_codir	-0.03958433	0.82247723	0.89176498
ENSG0000026	RP11-466A19	lncRNA	-0.03970884	0.96016756	0.97660552
ENSG0000017	NUDCD2	protein_codir	-0.03983352	0.70098289	0.80517278
ENSG0000018	TMEM64	protein_codir	-0.03989575	0.80054819	0.876244
ENSG0000018	SOWAHB	protein_codir	-0.04008561	0.94995677	0.97062636
ENSG0000007	ACACB	protein_codir	-0.04015446	0.90678555	0.94450416
ENSG0000019	FOXJ3	protein_codir	-0.04017287	0.65735543	0.77217527
ENSG0000006	HDHD5	protein_codir	-0.04025121	0.70906686	0.8110501
ENSG0000017	FAM53A	protein_codir	-0.04026336	0.87670772	0.9269127
ENSG0000014	ZNF687	protein_codir	-0.04029256	0.76003873	0.84827533
ENSG0000023	RNF103	protein_codir	-0.04031182	0.79168762	0.87044354
ENSG0000012	RBM19	protein_codir	-0.04037141	0.78036584	0.86270031
ENSG0000006	NFYC	protein_codir	-0.04038536	0.62180845	0.74470339
ENSG0000025	RP4-647C14.2	lncRNA	-0.04043574	0.89050395	0.93474124
ENSG0000026	RP11-138I1.4	lncRNA	-0.04054931	0.91094503	0.94697963

ENSG0000017	PARD6G	protein_codir	-0.04057939	0.92312603	0.95468676
ENSG0000001	ATP2C1	protein_codir	-0.04063689	0.71982928	0.81908642
ENSG0000013	IMPA1	protein_codir	-0.04064586	0.70818131	0.81057795
ENSG0000017	TMEM192	protein_codir	-0.04067576	0.76391457	0.85098493
ENSG0000018	ZFP3	protein_codir	-0.04068931	0.88428357	0.93128621
ENSG0000014	UTP23	protein_codir	-0.0407256	0.78507785	0.86558821
ENSG0000026	RP11-21L23.2	lncRNA	-0.04075644	0.95050493	0.97096479
ENSG0000028	ABC12-46663	lncRNA	-0.04077485	0.86907119	0.92233415
ENSG0000027	CTD-2376I4.1	lncRNA	-0.04079132	0.95580941	0.97399761
ENSG0000017	TBL1XR1	protein_codir	-0.04093824	0.73982725	0.83363313
ENSG0000025	SYNJ2BP-COX	protein_codir	-0.04097305	0.92781127	0.95705808
ENSG0000012	ARMH3	protein_codir	-0.04120403	0.63758634	0.75709686
ENSG0000010	GSPT1	protein_codir	-0.0412177	0.82111442	0.89056704
ENSG0000018	SMIM15	protein_codir	-0.04125694	0.75329981	0.84305172
ENSG0000027	CTC-366B18.4	lncRNA	-0.04126458	0.90407714	0.94310574
ENSG0000013	IRS4	protein_codir	-0.04129584	0.97530284	0.98604274
ENSG0000007	MLLT10	protein_codir	-0.04139263	0.72153425	0.8202155
ENSG0000010	DNASE2	protein_codir	-0.04157246	0.76218519	0.84984679
ENSG0000024	CTD-2203K17	lncRNA	-0.0415996	0.90535112	0.94372185
ENSG0000022	VPS52	protein_codir	-0.04161337	0.72530262	0.82300885
ENSG0000024	RP11-1000B6.	lncRNA	-0.04163681	0.91774395	0.95132625
ENSG0000013	C9orf40	protein_codir	-0.04165831	0.84103844	0.90494622
ENSG0000016	ADAMTS13	protein_codir	-0.04174078	0.89167461	0.93545901
ENSG0000001	FUZ	protein_codir	-0.04181147	0.77437993	0.858247
ENSG0000011	NCL	protein_codir	-0.04186942	0.77963711	0.86213624
ENSG0000028	LINC02246	lncRNA	-0.04192851	0.91737154	0.95104729
ENSG0000025	NPIPA2	protein_codir	-0.04200467	0.94947375	0.9704591
ENSG0000008	TRMT6	protein_codir	-0.04205864	0.8046806	0.87888636
ENSG0000027	ZNF516-DT	lncRNA	-0.04220964	0.93879368	0.96367047
ENSG0000010	PRORP	protein_codir	-0.04228629	0.81335378	0.88527866
ENSG0000016	ZNF528	protein_codir	-0.04234893	0.83350408	0.89965003
ENSG0000014	ENOPH1	protein_codir	-0.04249933	0.69085806	0.79712059
ENSG0000023	ERI3-IT1	lncRNA	-0.0425274	0.94770101	0.96964259
ENSG0000014	RASA1	protein_codir	-0.04259138	0.7445877	0.83681283
ENSG0000018	CCDC43	protein_codir	-0.0426061	0.67249104	0.78398626
ENSG0000015	UBE2Z	protein_codir	-0.04263145	0.62308961	0.74574829
ENSG0000025	RP11-138H8.6	lncRNA	-0.04275869	0.93701892	0.96259996
ENSG0000018	PPP1R2	protein_codir	-0.04282946	0.79674459	0.87377747
ENSG0000016	TAOK1	protein_codir	-0.04287216	0.8470634	0.90889597
ENSG0000021	ANKRD39	protein_codir	-0.04292617	0.76860694	0.85424827
ENSG0000024	MCPH1-AS1	lncRNA	-0.0431515	0.89757596	0.93912599
ENSG0000013	PHF21A	protein_codir	-0.04319535	0.73292594	0.82852759
ENSG0000027	RP11-422P24.	lncRNA	-0.04321027	0.94535069	0.96817034
ENSG0000026	ISY1-RAB43	protein_codir	-0.0433933	0.83559587	0.90124797
ENSG0000015	FRMD4A	protein_codir	-0.04349793	0.83287373	0.89933041
ENSG0000012	MRPS12	protein_codir	-0.04350868	0.78469808	0.86530766
ENSG0000015	BRPF1	protein_codir	-0.04369158	0.68065958	0.79007429

ENSG0000026	CTB-50L17.14	protein_codir	-0.04374072	0.94929345	0.97039638
ENSG0000020	LCMT1	protein_codir	-0.04374413	0.61857888	0.74216799
ENSG0000012	SMU1	protein_codir	-0.04388417	0.63089155	0.75151383
ENSG0000011	MCM3	protein_codir	-0.04413491	0.7705286	0.85556576
ENSG0000026	RP11-255C15.	lncRNA	-0.04414709	0.93923637	0.96394184
ENSG0000020	COLQ	protein_codir	-0.04416666	0.90057868	0.94052192
ENSG0000019	BORCS6	protein_codir	-0.04417319	0.78221695	0.86391907
ENSG0000006	MAP2K4	protein_codir	-0.04423256	0.68633344	0.79415709
ENSG0000000	CDC27	protein_codir	-0.04424743	0.74606573	0.83779214
ENSG0000017	THOP1	protein_codir	-0.04432584	0.80458074	0.87881204
ENSG0000010	TFIP11	protein_codir	-0.04436183	0.70315762	0.80666279
ENSG0000000	GDE1	protein_codir	-0.04441275	0.78296561	0.86427976
ENSG0000026	RP11-37C7.3	lncRNA	-0.04444874	0.94372992	0.96701244
ENSG0000009	ABCC6	protein_codir	-0.04449408	0.91108988	0.94705595
ENSG0000019	RNF216P1	lncRNA	-0.04450098	0.73536823	0.83023506
ENSG0000014	EIF4EBP2	protein_codir	-0.04450824	0.77256328	0.85695574
ENSG0000009	CERS4	protein_codir	-0.04454866	0.84274496	0.90612798
ENSG0000008	TXNDC16	protein_codir	-0.04458465	0.85971563	0.91624269
ENSG0000019	ZNF720	protein_codir	-0.04477444	0.72707676	0.82417549
ENSG0000017	ZNF354B	protein_codir	-0.04482391	0.7620584	0.84979756
ENSG0000006	MON2	protein_codir	-0.04484093	0.62306047	0.74574829
ENSG0000012	CDKN2D	protein_codir	-0.04496525	0.82874678	0.89655658
ENSG0000015	VBP1	protein_codir	-0.0449723	0.7455488	0.83751811
ENSG0000017	YPEL2	protein_codir	-0.04510939	0.82537945	0.89413949
ENSG0000008	SEH1L	protein_codir	-0.04516802	0.76613252	0.85264175
ENSG0000017	NDUFV2	protein_codir	-0.04519584	0.67161659	0.78339669
ENSG0000013	NUP42	protein_codir	-0.04522124	0.77283429	0.85709829
ENSG0000012	NGDN	protein_codir	-0.04540936	0.6676056	0.78046503
ENSG0000016	PDP1	protein_codir	-0.04542269	0.85382591	0.91285717
ENSG0000014	HSPD1	protein_codir	-0.04568362	0.841547	0.90540386
ENSG0000001	MATR3	protein_codir	-0.04596213	0.7345041	0.82970035
ENSG0000023	UQCRHL	protein_codir	-0.04601605	0.80619651	0.87991573
ENSG0000028	RP11-785J10.	lncRNA	-0.04606257	0.97286854	0.98451261
ENSG0000014	GATAD2B	protein_codir	-0.04635019	0.73197629	0.82769119
ENSG0000015	UQCRB	protein_codir	-0.04641973	0.77264392	0.8569969
ENSG0000027	RP11-620J15.	lncRNA	-0.04661614	0.94012018	0.9645661
ENSG0000025	DPP3	protein_codir	-0.04668961	0.82266474	0.89183437
ENSG0000024	RNF139-AS1	lncRNA	-0.04673867	0.88653651	0.93273421
ENSG0000015	CCDC174	protein_codir	-0.04678096	0.66616259	0.77927283
ENSG0000014	ACP1	protein_codir	-0.0469679	0.61568371	0.73961309
ENSG0000016	UBLCP1	protein_codir	-0.04699576	0.7064156	0.8091904
ENSG0000021	MYCBP	protein_codir	-0.04701516	0.72134574	0.82010247
ENSG0000012	FAM182A	lncRNA	-0.04715006	0.8840589	0.93126266
ENSG0000015	EMSY	protein_codir	-0.04730036	0.7158262	0.81601056
ENSG0000025	LINC02739	lncRNA	-0.04734711	0.96129458	0.9773598
ENSG0000025	LINC00638	lncRNA	-0.04735294	0.91821861	0.95156829
ENSG0000012	PPCS	protein_codir	-0.04736723	0.58439995	0.71528524

ENSG0000008	DDX43	protein_codir	-0.04745521	0.89350845	0.9365915
ENSG0000023	RP11-225H22	lncRNA	-0.04760968	0.91290327	0.9484085
ENSG0000010	BCKDK	protein_codir	-0.04767572	0.67227617	0.78383502
ENSG0000016	SLC45A2	protein_codir	-0.04775003	0.97852057	0.9879277
ENSG0000017	ELMOD2	protein_codir	-0.0477537	0.72286568	0.82118826
ENSG0000027	RP11-227G15	lncRNA	-0.04783054	0.93025932	0.9586867
ENSG0000013	UTP3	protein_codir	-0.04793446	0.70260995	0.80628402
ENSG0000020	PCDHA2	protein_codir	-0.04809441	0.95242113	0.97200493
ENSG0000011	PRKAB1	protein_codir	-0.04818025	0.66688822	0.7797914
ENSG0000011	IFT57	protein_codir	-0.04823731	0.77923548	0.86183013
ENSG0000012	XPO5	protein_codir	-0.04824931	0.64007352	0.75865041
ENSG0000014	ARL8A	protein_codir	-0.04825093	0.71796886	0.81784015
ENSG0000010	MON1B	protein_codir	-0.04825796	0.68272533	0.79135179
ENSG0000010	SNU13	protein_codir	-0.04828333	0.60680893	0.73300077
ENSG0000012	SIN3B	protein_codir	-0.04828529	0.73978113	0.83363313
ENSG0000022	PDE9A-AS1	lncRNA	-0.04832673	0.93531672	0.96170977
ENSG0000028	RP3-402L9.3	protein_codir	-0.04833209	0.92411064	0.95527287
ENSG0000017	ATG13	protein_codir	-0.04843768	0.7132727	0.81414774
ENSG0000024	ASPRV1	protein_codir	-0.04844671	0.93395735	0.96109918
ENSG0000010	ACOT8	protein_codir	-0.04847462	0.80054718	0.876244
ENSG0000016	YY1AP1	protein_codir	-0.04858189	0.65308722	0.76880868
ENSG0000013	TCHP	protein_codir	-0.04858698	0.73143045	0.82734214
ENSG0000015	SMARCA5	protein_codir	-0.04870653	0.70139703	0.80542693
ENSG0000014	SNAP47	protein_codir	-0.04872695	0.67293644	0.78430689
ENSG0000012	TICAM1	protein_codir	-0.04877946	0.78957988	0.86876482
ENSG0000025	LINC00641	lncRNA	-0.04886089	0.82084808	0.89034809
ENSG0000014	SLC30A5	protein_codir	-0.04896507	0.68276297	0.79135179
ENSG0000021	TAS2R19	protein_codir	-0.04921017	0.93672925	0.96241419
ENSG0000014	DCDC2	protein_codir	-0.04934553	0.94323481	0.96689145
ENSG0000010	IQCE	protein_codir	-0.04936169	0.63724278	0.75678916
ENSG0000016	TMEM67	protein_codir	-0.04938494	0.78675602	0.86681564
ENSG0000023	RP11-295P9.3	lncRNA	-0.04948609	0.84951138	0.91049687
ENSG0000016	CDK5	protein_codir	-0.04959218	0.78854371	0.86802348
ENSG0000016	USP49	protein_codir	-0.04964678	0.84393296	0.90670837
ENSG0000010	PHF5A	protein_codir	-0.04972121	0.63666896	0.75630281
ENSG0000010	DUS4L	protein_codir	-0.04979328	0.77760308	0.86071402
ENSG0000022	HYI-AS1	lncRNA	-0.04982558	0.97670649	NA
ENSG0000016	KDM1B	protein_codir	-0.04985514	0.80528209	0.87936944
ENSG0000016	FBXO33	protein_codir	-0.04988116	0.68939889	0.79608037
ENSG0000013	RTF1	protein_codir	-0.04990305	0.7246326	0.82234992
ENSG0000014	FIP1L1	protein_codir	-0.04991925	0.64393851	0.76170124
ENSG0000013	SEC24B	protein_codir	-0.04999098	0.69575198	0.80072482
ENSG0000013	ZNF341	protein_codir	-0.05000943	0.75092081	0.8412498
ENSG0000018	PLAC9	protein_codir	-0.05016306	0.8921657	0.9357339
ENSG0000000	CAMKK1	protein_codir	-0.05016947	0.79275642	0.87106465
ENSG0000012	NEURL2	protein_codir	-0.05029841	0.91585321	0.95001426
ENSG0000011	KPTN	protein_codir	-0.05049096	0.86758681	0.92136064

ENSG0000016	UBXN6	protein_codir	-0.0505616	0.68417065	0.79248451
ENSG0000014	MRPS5	protein_codir	-0.05060206	0.74626893	0.83788406
ENSG0000016	RAB31	protein_codir	-0.05086961	0.8499589	0.91065847
ENSG0000015	CCDC117	protein_codir	-0.05088121	0.76589227	0.85243168
ENSG0000010	ELP4	protein_codir	-0.05099553	0.62698729	0.74853637
ENSG0000027	RP11-464F9.2	lncRNA	-0.05105293	0.91818785	0.95156829
ENSG0000028	ARRDC3-AS1	lncRNA	-0.05110008	0.88841795	0.9338244
ENSG0000009	EFNB1	protein_codir	-0.05128795	0.79739984	0.87407959
ENSG0000018	PNPLA1	protein_codir	-0.05147185	0.88587452	0.93235736
ENSG0000027	CH17-353B19	lncRNA	-0.05161077	0.90937364	0.94616514
ENSG0000023	TMEM229A	protein_codir	-0.05162375	0.95940991	0.97616229
ENSG0000014	NPC1	protein_codir	-0.05162921	0.72379203	0.82176893
ENSG0000008	ATG16L1	protein_codir	-0.05172805	0.64103013	0.75926293
ENSG0000018	FOXD4L1	protein_codir	-0.05188605	0.94083551	0.96515662
ENSG0000015	ODR4	protein_codir	-0.05208116	0.61979665	0.74313177
ENSG0000010	CNIH1	protein_codir	-0.05208414	0.77867841	0.86148999
ENSG0000011	STK11	protein_codir	-0.05211123	0.65712973	0.77210237
ENSG0000008	GANAB	protein_codir	-0.05215989	0.62882728	0.74985794
ENSG0000002	RB1CC1	protein_codir	-0.05239293	0.75919217	0.84767303
ENSG0000010	GALNT16	protein_codir	-0.05240747	0.86842932	0.92191649
ENSG0000011	ACTR8	protein_codir	-0.05248632	0.68230654	0.7911087
ENSG0000012	HP1BP3	protein_codir	-0.05251641	0.66005398	0.77435717
ENSG0000025	RP11-158K1.3	lncRNA	-0.05251787	0.8050003	0.87916601
ENSG0000013	TOE1	protein_codir	-0.05253582	0.70198563	0.80582033
ENSG0000016	SLC25A46	protein_codir	-0.05255048	0.67418296	0.78521442
ENSG0000014	ABCA8	protein_codir	-0.05263785	0.91757741	0.95118932
ENSG0000026	KPNB1-DT	lncRNA	-0.05270926	0.9398343	0.96436462
ENSG0000024	ATP5PO	protein_codir	-0.05287943	0.6868984	0.79469217
ENSG0000017	MSRA	protein_codir	-0.05295131	0.78446081	0.86521874
ENSG0000004	ADRB1	protein_codir	-0.05355053	0.90029355	0.9403308
ENSG0000004	CUL7	protein_codir	-0.05355979	0.62588547	0.74770574
ENSG0000015	NRSN1	protein_codir	-0.05360899	0.9628664	0.97828085
ENSG0000011	KLHL18	protein_codir	-0.05370518	0.63171015	0.75222394
ENSG0000010	CACTIN	protein_codir	-0.05376002	0.58731673	0.71761505
ENSG0000008	COQ9	protein_codir	-0.05387782	0.78869907	0.86809071
ENSG0000013	PSAT1	protein_codir	-0.05388713	0.89654356	0.93847241
ENSG0000014	ANKRD31	protein_codir	-0.05397939	0.8884852	0.93385955
ENSG0000007	UBE2A	protein_codir	-0.05403972	0.55359139	0.68984198
ENSG0000015	HNRNPDL	protein_codir	-0.05411554	0.61418795	0.73844272
ENSG0000008	TMEM230	protein_codir	-0.05416284	0.65633262	0.77166299
ENSG0000013	MED10	protein_codir	-0.0541843	0.64613182	0.76350479
ENSG0000015	SLC25A28	protein_codir	-0.05418762	0.71533304	0.81554936
ENSG0000010	FMR1	protein_codir	-0.05423895	0.62738281	0.7487173
ENSG0000027	DDX52	protein_codir	-0.05434888	0.69495092	0.80000602
ENSG0000012	SLC25A51	protein_codir	-0.05436498	0.71335038	0.81419635
ENSG0000023	CYP21A2	protein_codir	-0.05436958	0.84930258	0.91035502
ENSG0000014	TAF1	protein_codir	-0.05445983	0.6561669	0.77154122

ENSG0000007	ANO8	protein_codir	-0.05461044	0.72338778	0.82154484
ENSG0000014	ZNF7	protein_codir	-0.05471057	0.66611764	0.77927283
ENSG0000022	RP4-781K5.2	lncRNA	-0.05476079	0.87072466	0.92327301
ENSG0000013	KIF21A	protein_codir	-0.05484166	0.737237	0.83169897
ENSG0000018	PSMD13	protein_codir	-0.05485587	0.59612593	0.72446861
ENSG0000013	ECPAS	protein_codir	-0.05488327	0.62626752	0.74800039
ENSG0000015	UPF2	protein_codir	-0.05496947	0.61824694	0.74191782
ENSG0000013	OSER1	protein_codir	-0.05504197	0.67244625	0.78398626
ENSG0000011	TTL	protein_codir	-0.05505685	0.63500297	0.75486982
ENSG0000026	RBFADN	lncRNA	-0.05509493	0.88600627	0.93242496
ENSG0000006	TRMT11	protein_codir	-0.05510045	0.75818365	0.84690662
ENSG0000010	PSMA2	protein_codir	-0.05510135	0.68153893	0.79059129
ENSG0000014	BNIP2	protein_codir	-0.05550085	0.72651209	0.82389002
ENSG0000017	HNRNPA0	protein_codir	-0.05552382	0.73995659	0.83371139
ENSG0000024	NR2F2-AS1	lncRNA	-0.05557639	0.83180556	0.89863371
ENSG0000013	FBXW9	protein_codir	-0.05560959	0.77083211	0.85582598
ENSG0000018	FOXO4	protein_codir	-0.05561328	0.80728333	0.88061476
ENSG0000012	MKLN1	protein_codir	-0.05563568	0.70978003	0.81148359
ENSG0000011	GTF2H1	protein_codir	-0.05570965	0.64629775	0.76357019
ENSG0000013	TRPC3	protein_codir	-0.05572149	0.8861177	0.9325067
ENSG0000019	EIF1AY	protein_codir	-0.0557968	0.91178498	0.94753184
ENSG0000025	RP11-37B2.1	lncRNA	-0.05579835	0.83076909	0.89807602
ENSG0000015	KIN	protein_codir	-0.0558199	0.63372239	0.753905
ENSG0000016	ANAPC16	protein_codir	-0.0558547	0.66356216	0.77730153
ENSG0000008	TNPO1	protein_codir	-0.05590312	0.71878365	0.81843609
ENSG0000012	DGCR8	protein_codir	-0.05600342	0.70998126	0.81159185
ENSG0000007	FGF22	protein_codir	-0.05606575	0.93124018	0.95938377
ENSG0000010	ARMC6	protein_codir	-0.05611635	0.69996544	0.80423668
ENSG0000016	ARPC2	protein_codir	-0.0561346	0.73973071	0.83362693
ENSG0000013	STX17	protein_codir	-0.05639198	0.63167037	0.752209
ENSG0000018	PGBD2	protein_codir	-0.05652377	0.81372696	0.88546023
ENSG0000016	TMEM42	protein_codir	-0.05655305	0.72541568	0.82310332
ENSG0000016	ATG4B	protein_codir	-0.05659201	0.74988253	0.84054038
ENSG0000009	DDT	protein_codir	-0.0566584	0.68393155	0.79230725
ENSG0000019	TBC1D9B	protein_codir	-0.05672627	0.58483217	0.71562398
ENSG0000016	TMEM223	protein_codir	-0.05676569	0.76367082	0.85083972
ENSG0000016	SENP2	protein_codir	-0.05688094	0.62708211	0.74858485
ENSG0000013	COG2	protein_codir	-0.05697992	0.54595257	0.68389542
ENSG0000011	NRDE2	protein_codir	-0.05708689	0.64746336	0.76426078
ENSG0000010	CENPT	protein_codir	-0.05714192	0.84171012	0.90547355
ENSG0000009	PUS7	protein_codir	-0.05734321	0.78126289	0.86317659
ENSG0000011	KLF9	protein_codir	-0.0574276	0.88595866	0.93241039
ENSG0000009	HECTD1	protein_codir	-0.05754455	0.69334093	0.79875189
ENSG0000026	CTD-2313J17.	lncRNA	-0.05760412	0.91018407	0.94647355
ENSG0000014	MFSD14B	protein_codir	-0.0576602	0.71465533	0.81494489
ENSG0000015	RSPRY1	protein_codir	-0.05768193	0.65675722	0.7718665
ENSG0000000	DPM1	protein_codir	-0.05781781	0.5200092	0.66275918

ENSG0000014	RP11-977G19	protein_codir	-0.05789699	0.85641494	0.91426956
ENSG0000017	NAA20	protein_codir	-0.05793635	0.58642549	0.71681148
ENSG0000014	UCN2	protein_codir	-0.05798607	0.91419751	0.9491105
ENSG0000017	CBWD1	protein_codir	-0.05801937	0.77032149	0.8554879
ENSG0000021	AC025171.1	lncRNA	-0.0581081	0.82265546	0.89183437
ENSG0000004	ERCC8	protein_codir	-0.0581306	0.64730052	0.76418205
ENSG0000016	GPX4	protein_codir	-0.05814933	0.7394382	0.83339929
ENSG0000017	LRRC34	protein_codir	-0.05817802	0.81008551	0.88274067
ENSG0000022	ZFAND2A-DT	lncRNA	-0.05821405	0.85425926	0.91296397
ENSG0000008	ERO1B	protein_codir	-0.05846952	0.67022189	0.78243733
ENSG0000012	CHCHD5	protein_codir	-0.05847869	0.6261289	0.74793185
ENSG0000013	ODF2	protein_codir	-0.05854646	0.59891563	0.72680359
ENSG0000027	RP11-166O4.6	lncRNA	-0.05856555	0.90542951	0.94374117
ENSG0000024	ZNF436-AS1	lncRNA	-0.05856948	0.88790667	0.93348541
ENSG0000013	POGLUT2	protein_codir	-0.05872185	0.80724468	0.88060737
ENSG0000014	ZMYM4	protein_codir	-0.0587548	0.55890919	0.69444605
ENSG0000012	DTD1	protein_codir	-0.05878521	0.65173244	0.76765961
ENSG0000007	BCS1L	protein_codir	-0.05895719	0.74057334	0.83427016
ENSG0000028	TFAP2A-AS2	lncRNA	-0.059014	0.91796655	0.95148557
ENSG0000010	IFT52	protein_codir	-0.05904245	0.72253088	0.82094298
ENSG0000015	RADIL	protein_codir	-0.05913744	0.85238544	0.91219675
ENSG0000013	UNK	protein_codir	-0.05914657	0.67658966	0.78696777
ENSG0000018	MOSMO	protein_codir	-0.05914833	0.67247106	0.78398626
ENSG0000010	POU6F2	protein_codir	-0.05920444	0.92482406	0.95572524
ENSG0000026	RP11-1072C15	lncRNA	-0.05923948	0.87430361	0.92546912
ENSG0000025	RP11-210M15	lncRNA	-0.05930264	0.91728352	0.95099173
ENSG0000017	MRPL57	protein_codir	-0.05935347	0.46513379	0.61491735
ENSG0000011	CFAP94	protein_codir	-0.0595145	0.89247587	0.93591714
ENSG0000028	RP11-791J18.	lncRNA	-0.0595488	0.94931608	0.97039638
ENSG0000019	SRGAP2B	protein_codir	-0.05956853	0.74457166	0.83681283
ENSG0000015	ACAD8	protein_codir	-0.05957855	0.75847859	0.84708174
ENSG0000018	NELFA	protein_codir	-0.05958743	0.54563115	0.6836168
ENSG0000017	MRM3	protein_codir	-0.05960449	0.61346758	0.7379434
ENSG0000027	RP11-313P13.	lncRNA	-0.05964276	0.93540576	0.96176552
ENSG0000017	EVC2	protein_codir	-0.05969361	0.73741049	0.83179277
ENSG0000010	ACTR5	protein_codir	-0.05973379	0.64071211	0.75908156
ENSG0000025	RP5-991G20.1	lncRNA	-0.05974677	0.76384612	0.850943
ENSG0000010	BRAT1	protein_codir	-0.05986334	0.69953154	0.8038719
ENSG0000027	NOL12	protein_codir	-0.05986557	0.73832068	0.83258144
ENSG0000009	TIMM13	protein_codir	-0.0599207	0.68739701	0.79488823
ENSG0000012	C22orf23	protein_codir	-0.05992237	0.91671436	0.95063094
ENSG0000014	MIGA2	protein_codir	-0.05994132	0.74653341	0.83802061
ENSG0000010	C1QBP	protein_codir	-0.05996749	0.74925903	0.84011425
ENSG0000000	ADIPOR2	protein_codir	-0.06015653	0.6366582	0.75630281
ENSG0000018	INTS5	protein_codir	-0.06018164	0.60920876	0.73473873
ENSG0000023	SNTG2-AS1	lncRNA	-0.06024806	0.9575355	0.97497348
ENSG0000010	MTCH2	protein_codir	-0.06028629	0.69299322	0.79848453

ENSG000001З	POMP	protein_codir	-0.06050125	0.66626812	0.77936327
ENSG0000027	H3C8	protein_codir	-0.06051795	0.93319375	0.96072875
ENSG0000023	HOXB-AS1	lncRNA	-0.06058556	0.83362593	0.89972053
ENSG000001З	SLC9A5	protein_codir	-0.06064036	0.88563501	0.93217632
ENSG000001(	LUC7L3	protein_codir	-0.06068197	0.84284883	0.90620439
ENSG0000017	C11orf45	protein_codir	-0.06068991	0.85379213	0.91285717
ENSG0000027	NBPF14	protein_codir	-0.06077729	0.7409854	0.83457897
ENSG000001€	C1orf50	protein_codir	-0.06078386	0.7122227	0.81344709
ENSG000001‡	PPP2R5E	protein_codir	-0.06082829	0.61147448	0.73658271
ENSG000001€	TGFBR2	protein_codir	-0.06093942	0.80268905	0.87773629
ENSG000000⁄	CNTLN	protein_codir	-0.06096051	0.72912955	0.82585844
ENSG0000027	RP11-22N19.2	lncRNA	-0.06096401	0.87146138	0.92365991
ENSG0000008	RFX2	protein_codir	-0.06106935	0.88643781	0.93270142
ENSG000001€	ICE1	protein_codir	-0.06115329	0.70254906	0.80628402
ENSG000001З	NAT10	protein_codir	-0.06117274	0.62835012	0.7495802
ENSG000001€	TMEM92	protein_codir	-0.06136621	0.89265348	0.93603224
ENSG0000011	PDCD10	protein_codir	-0.06136751	0.5841647	0.71510012
ENSG0000011	MED28	protein_codir	-0.06140137	0.61783318	0.74151795
ENSG000001З	PRRG1	protein_codir	-0.06145464	0.81404937	0.88561697
ENSG0000024	LINC02511	lncRNA	-0.06149245	0.92444647	0.95549948
ENSG0000001	VSIG2	protein_codir	-0.06152198	0.90416705	0.94312829
ENSG000001€	USP1	protein_codir	-0.06169108	0.67940123	0.78927705
ENSG000001€	SNRNP48	protein_codir	-0.06170597	0.67790997	0.78797548
ENSG0000017	CHRNB1	protein_codir	-0.06187731	0.69306108	0.7984961
ENSG000002€	RP11-242D8.1	lncRNA	-0.06201784	0.85021909	0.91086658
ENSG000002€	CTD-3126B10	lncRNA	-0.06211609	0.92696409	0.95664518
ENSG0000027	RP5-1116H23	lncRNA	-0.06213674	0.95539727	0.97390372
ENSG000001G	LTN1	protein_codir	-0.06229841	0.7308248	0.82678794
ENSG000001(	ZDHHC2	protein_codir	-0.06234692	0.79839939	0.87468926
ENSG0000018	SPATA8	lncRNA	-0.06237094	0.97456971	NA
ENSG0000012	HS3ST2	protein_codir	-0.06247041	0.91288445	0.9484085
ENSG0000012	TTC21B	protein_codir	-0.06250599	0.61622738	0.74004088
ENSG0000017	ULK1	protein_codir	-0.06267069	0.68062398	0.79006617
ENSG000001З	PAK4	protein_codir	-0.06267988	0.6485023	0.76473544
ENSG0000011	WDR48	protein_codir	-0.06279267	0.65707619	0.77210237
ENSG000001‡	CWF19L2	protein_codir	-0.06282503	0.710414	0.81190694
ENSG0000017	STX19	protein_codir	-0.06289339	0.92660194	0.95648785
ENSG0000028	CTD-2544D21	lncRNA	-0.06309276	0.94450318	0.96762529
ENSG0000011	COQ10B	protein_codir	-0.06326443	0.75176243	0.84190423
ENSG0000012	RBCK1	protein_codir	-0.06329907	0.45775676	0.60822971
ENSG000001G	ZNF512B	protein_codir	-0.06338905	0.43544606	0.58680842
ENSG0000022	RP5-971N18.3	lncRNA	-0.06344352	0.90024135	0.9403308
ENSG0000007	ZXDC	protein_codir	-0.06348521	0.63700682	0.75660654
ENSG0000012	ZNF45	protein_codir	-0.0635102	0.70109949	0.8052047
ENSG0000023	RP5-997D16.2	lncRNA	-0.06366965	0.97815068	NA
ENSG000001€	ABRAXAS1	protein_codir	-0.06367813	0.67277759	0.78418792
ENSG0000017	ACBD7	protein_codir	-0.06370355	0.85956106	0.91613624

ENSG0000025	AP006621.5	lncRNA	-0.0637686	0.85219502	0.9120283
ENSG0000017	RP11-97O12.7	protein_codir	-0.06386956	0.81867954	0.88869395
ENSG0000012	RIOK1	protein_codir	-0.06388207	0.59133417	0.72057927
ENSG0000025	RP11-144G6.1	lncRNA	-0.06395327	0.8198075	0.88949886
ENSG0000026	POLG-DT	lncRNA	-0.06398035	0.89871768	0.93975098
ENSG0000017	GPR137	protein_codir	-0.06416945	0.56393342	0.69829111
ENSG0000012	COX6B1	protein_codir	-0.06418739	0.62046242	0.74373632
ENSG0000018	SRSF10	protein_codir	-0.06430245	0.57328141	0.70624587
ENSG0000027	RP11-31H5.3	lncRNA	-0.06432762	0.8665733	0.92070913
ENSG0000013	FBXL8	protein_codir	-0.06445402	0.74419583	0.83657667
ENSG0000028	RP1-138B7.7	lncRNA	-0.06449141	0.96137203	NA
ENSG0000010	PCIF1	protein_codir	-0.06451041	0.60828571	0.73412496
ENSG0000010	CHCHD3	protein_codir	-0.06467279	0.68178406	0.79068171
ENSG0000015	UBR1	protein_codir	-0.06480298	0.69378516	0.79913033
ENSG0000018	TOP1MT	protein_codir	-0.06493477	0.60310575	0.72999609
ENSG0000026	RP11-626G11	lncRNA	-0.06499508	0.94206263	0.96605665
ENSG0000008	HADHA	protein_codir	-0.06502484	0.59249076	0.72135214
ENSG0000014	ANP32E	protein_codir	-0.06507153	0.64820845	0.76469975
ENSG0000012	NUP43	protein_codir	-0.06517947	0.71588853	0.81604792
ENSG0000014	TMEM138	protein_codir	-0.0651975	0.74654225	0.83802061
ENSG0000017	METAP1D	protein_codir	-0.06524035	0.77523634	0.85892045
ENSG0000010	SH3PXD2A	protein_codir	-0.06524875	0.79033377	0.86937029
ENSG0000011	FANCL	protein_codir	-0.06528617	0.75856223	0.8471409
ENSG0000014	KAT14	protein_codir	-0.06534274	0.69119768	0.79731373
ENSG0000002	TOMM34	protein_codir	-0.0654262	0.65879679	0.773288
ENSG0000013	ATP5IF1	protein_codir	-0.06562814	0.61194784	0.73695223
ENSG0000027	RPS10-NUDT3	protein_codir	-0.0658686	0.88704243	0.93308877
ENSG0000013	RCBTB2	protein_codir	-0.06608508	0.7338081	0.82926604
ENSG0000016	VPS11	protein_codir	-0.06613223	0.46115525	0.61139096
ENSG0000015	DHRS1	protein_codir	-0.06622446	0.69782656	0.80234658
ENSG0000014	EIF2D	protein_codir	-0.06624889	0.49936724	0.64484189
ENSG0000020	PCDHA9	protein_codir	-0.06633065	0.95869198	0.97579129
ENSG0000023	UBE2R2-AS1	lncRNA	-0.0663374	0.94593566	0.9685386
ENSG0000017	CHID1	protein_codir	-0.06641187	0.50098041	0.6461091
ENSG0000016	RNF169	protein_codir	-0.06655029	0.56927185	0.703031
ENSG0000017	RFESD	protein_codir	-0.06660762	0.82614512	0.89475838
ENSG0000008	MYNN	protein_codir	-0.06669996	0.63805451	0.7573624
ENSG0000015	BTG3	protein_codir	-0.06672603	0.80268746	0.87773629
ENSG0000026	RP11-616M22	lncRNA	-0.06680791	0.96785235	NA
ENSG0000028	RP1-137D17.4	protein_codir	-0.06681962	0.90191308	0.94170186
ENSG0000021	LYRM4	protein_codir	-0.06682563	0.47605021	0.62425104
ENSG0000008	IPO11	protein_codir	-0.06689631	0.69664769	0.80149161
ENSG0000025	CNPY2	protein_codir	-0.0669416	0.55989917	0.69524862
ENSG0000024	RP1-27K12.4	lncRNA	-0.06694325	0.98140002	NA
ENSG0000006	INTS13	protein_codir	-0.06695962	0.67221059	0.78383502
ENSG0000018	YTHDF3	protein_codir	-0.06700908	0.70693675	0.80956786
ENSG0000026	CTD-3138B18	lncRNA	-0.06701014	0.91242105	0.94805017

ENSG0000018	POLR3C	protein_codir	-0.06701921	0.3330115	0.48572969
ENSG0000013	ARHGEF4	protein_codir	-0.06702627	0.86466905	0.91935786
ENSG0000018	SGSH	protein_codir	-0.06703915	0.63508934	0.7549134
ENSG0000016	BMI1	protein_codir	-0.06709385	0.60493986	0.73138276
ENSG0000027	H2AC4	protein_codir	-0.06709749	0.95962334	0.9762372
ENSG0000010	GID8	protein_codir	-0.06711779	0.54916832	0.68663467
ENSG0000012	RASL11B	protein_codir	-0.06713766	0.86836404	0.92190248
ENSG0000018	OAZ2	protein_codir	-0.06716443	0.59406775	0.72266199
ENSG0000010	GLRX3	protein_codir	-0.06717348	0.593089	0.72185777
ENSG0000016	ATP23	protein_codir	-0.06717533	0.75830717	0.84695879
ENSG0000017	NMNAT1	protein_codir	-0.0671803	0.66911226	0.78139954
ENSG0000013	TJAP1	protein_codir	-0.06721346	0.62379757	0.74624958
ENSG0000011	ABHD14A-AC	protein_codir	-0.06742281	0.91565604	0.94998196
ENSG0000013	CMTR1	protein_codir	-0.06757716	0.50551656	0.6501079
ENSG0000015	TWF1	protein_codir	-0.06770819	0.71150171	0.81291061
ENSG0000027	RP11-458J1.1	lncRNA	-0.06774231	0.75139345	0.84158504
ENSG0000015	MPPE1	protein_codir	-0.0678469	0.46789504	0.61728943
ENSG0000016	ZNF202	protein_codir	-0.06791194	0.64815161	0.76469975
ENSG0000017	TOMM20	protein_codir	-0.06793304	0.71416025	0.81467497
ENSG0000009	BRPF3	protein_codir	-0.0679686	0.69724374	0.80188618
ENSG0000013	CBWD2	protein_codir	-0.06800038	0.61109619	0.73637611
ENSG0000011	CCNG1	protein_codir	-0.06802586	0.71073727	0.81211995
ENSG0000005	SIKE1	protein_codir	-0.06808457	0.62591722	0.74771133
ENSG0000019	OPA1	protein_codir	-0.06813008	0.67143921	0.78325594
ENSG0000017	TVP23C	protein_codir	-0.06842939	0.75619205	0.84548534
ENSG0000027	PSMB3	protein_codir	-0.06862659	0.64053506	0.75900204
ENSG0000019	MAK16	protein_codir	-0.06870223	0.67733388	0.78753785
ENSG0000019	ZNF667	protein_codir	-0.06871522	0.78629911	0.86641592
ENSG0000013	ADAM20	protein_codir	-0.06874906	0.90439188	0.94332718
ENSG0000020	ATP1A1-AS1	lncRNA	-0.06887549	0.77615287	0.85956672
ENSG0000013	CALCOCO2	protein_codir	-0.06889528	0.67143047	0.78325594
ENSG0000011	AIP	protein_codir	-0.0689743	0.59440943	0.72295515
ENSG0000010	ADNP2	protein_codir	-0.06911942	0.68794633	0.79522381
ENSG0000025	CTB-160O22.1	lncRNA	-0.06914881	0.96862354	0.98160532
ENSG0000015	RBMS1	protein_codir	-0.06916927	0.65910256	0.7735367
ENSG0000026	RSL1D1-DT	lncRNA	-0.06917312	0.92944976	0.9581469
ENSG0000016	PRPF18	protein_codir	-0.06929568	0.46534057	0.61510919
ENSG0000007	EXOSC7	protein_codir	-0.06930198	0.49366425	0.64005164
ENSG0000015	NKAPD1	protein_codir	-0.0693533	0.45639893	0.60697893
ENSG0000010	HTATIP2	protein_codir	-0.06937929	0.67452887	0.78553076
ENSG0000010	STX10	protein_codir	-0.06976827	0.53735763	0.67723019
ENSG0000015	TRIP12	protein_codir	-0.06993589	0.64844622	0.76470912
ENSG0000012	ARMCX5	protein_codir	-0.06993931	0.51786156	0.66099803
ENSG0000014	TRIM65	protein_codir	-0.07007255	0.71151752	0.81291061
ENSG0000011	PRPF19	protein_codir	-0.07013634	0.40757169	0.56058612
ENSG0000012	SMARCA4	protein_codir	-0.0701452	0.56503358	0.69932907
ENSG0000006	DNAJA2	protein_codir	-0.07025055	0.54242635	0.68114643

ENSG0000008	RTRAF	protein_codir	-0.07025132	0.49891119	0.64452608
ENSG0000013	ABCB10	protein_codir	-0.07063846	0.65420624	0.76968718
ENSG0000026	RP11-932O9.9	lncRNA	-0.07065897	0.90640781	0.94428876
ENSG0000007	GTF3C1	protein_codir	-0.07081041	0.30762695	0.45931966
ENSG0000020	DXO	protein_codir	-0.07086086	0.67579713	0.78638005
ENSG0000010	RAB11A	protein_codir	-0.07090279	0.58996796	0.71974006
ENSG0000012	TRIR	protein_codir	-0.07090651	0.62120896	0.74430815
ENSG0000010	UBE2I	protein_codir	-0.07101758	0.41600341	0.56881259
ENSG0000017	STAT5B	protein_codir	-0.07104313	0.52927327	0.67069773
ENSG0000017	OR2T33	protein_codir	-0.07111416	0.97610909	NA
ENSG0000010	PIAS4	protein_codir	-0.07114868	0.54332063	0.68180142
ENSG0000017	PRPF8	protein_codir	-0.07126442	0.60195129	0.72895013
ENSG0000014	BRD4	protein_codir	-0.07127825	0.66165107	0.77580246
ENSG0000026	RP6-201G10.2	lncRNA	-0.07134467	0.8632168	0.91862711
ENSG0000016	RDH13	protein_codir	-0.07141909	0.71657082	0.81666505
ENSG0000017	MPI	protein_codir	-0.07158901	0.53262943	0.67352921
ENSG0000028	RP11-342L8.3	lncRNA	-0.07159843	0.8972499	0.93896269
ENSG0000005	THRAP3	protein_codir	-0.07184026	0.4883976	0.63539999
ENSG0000015	ATP5F1A	protein_codir	-0.07206148	0.55829979	0.69391775
ENSG0000027	XXbac-BPG18	lncRNA	-0.07219554	0.8715161	0.92365991
ENSG0000011	AMBRA1	protein_codir	-0.07245329	0.39084397	0.54380689
ENSG0000013	CFAP300	protein_codir	-0.07247012	0.75778809	0.84672143
ENSG0000025	CTD-2292M16	lncRNA	-0.07253646	0.7812967	0.86317659
ENSG0000007	TM9SF3	protein_codir	-0.07255073	0.7069503	0.80956786
ENSG0000017	PAAF1	protein_codir	-0.07271784	0.70881858	0.81086684
ENSG0000010	EPHX3	protein_codir	-0.07271911	0.79614186	0.87335921
ENSG0000013	PPIG	protein_codir	-0.07279187	0.60466273	0.73114594
ENSG0000015	PFKFB1	protein_codir	-0.07290745	0.87789793	0.9274965
ENSG0000007	FDFT1	protein_codir	-0.07305482	0.62561175	0.74756951
ENSG0000023	RP11-135A24	lncRNA	-0.07314687	0.9623523	0.97811116
ENSG0000018	SETD4	protein_codir	-0.07315672	0.72086992	0.81978808
ENSG0000026	RAET1E-AS1	lncRNA	-0.07326539	0.86411209	0.91911928
ENSG0000026	GAN	protein_codir	-0.07334424	0.63315077	0.75345215
ENSG0000016	PGM2	protein_codir	-0.07334782	0.65389554	0.76941997
ENSG0000018	UQCR10	protein_codir	-0.07337227	0.4012775	0.55433053
ENSG0000011	MSH6	protein_codir	-0.0733861	0.50832745	0.65272971
ENSG0000020	PCDHA5	protein_codir	-0.0734936	0.8982904	0.9395781
ENSG0000011	TCTN3	protein_codir	-0.07371948	0.60222395	0.72921638
ENSG0000008	ALG9	protein_codir	-0.07372127	0.4088433	0.56181542
ENSG0000016	ATMIN	protein_codir	-0.07395414	0.63921474	0.75802289
ENSG0000019	ZNF763	protein_codir	-0.07402601	0.86746874	0.92133933
ENSG0000026	AP001063.1	lncRNA	-0.0741522	0.97855671	NA
ENSG0000016	CPT1C	protein_codir	-0.07422773	0.81792728	0.88809217
ENSG0000017	CALR	protein_codir	-0.07422816	0.72003782	0.81920049
ENSG0000013	RAN	protein_codir	-0.07423182	0.63999466	0.7585895
ENSG0000025	RP11-680H20	lncRNA	-0.07427764	0.89393494	0.93680692
ENSG0000024	PEG10	protein_codir	-0.0745064	0.81756164	0.88794163

ENSG0000026	RP1-193H18.3	lncRNA	-0.07454358	0.92648356	0.95648785
ENSG0000021	TMEM167B	protein_codir	-0.0746224	0.57157462	0.70479733
ENSG0000022	GRTP1-AS1	lncRNA	-0.07467682	0.9459908	0.9685386
ENSG0000027	CTB-13F3.1	lncRNA	-0.07499453	0.9269968	0.95664518
ENSG0000024	ATP5MF-PTCI	protein_codir	-0.07500651	0.69249416	0.79824251
ENSG0000018	PTAR1	protein_codir	-0.07510233	0.61780202	0.74151276
ENSG0000010	ANTKMT	protein_codir	-0.07512785	0.71018875	0.81173374
ENSG0000018	NF2	protein_codir	-0.07531679	0.5207081	0.66339669
ENSG0000025	RP11-770J1.3	lncRNA	-0.07541204	0.84670066	0.90864795
ENSG0000017	RAB43	protein_codir	-0.07567398	0.76908004	0.85464719
ENSG0000007	USP33	protein_codir	-0.07577641	0.53247163	0.67344823
ENSG0000026	RP11-42I10.1	lncRNA	-0.07578777	0.8804199	0.92909478
ENSG0000011	PPP1R8	protein_codir	-0.07592545	0.37825223	0.5318433
ENSG0000017	GVQW3	protein_codir	-0.07603221	0.80971347	0.88242921
ENSG0000020	MZT1	protein_codir	-0.0762145	0.62998933	0.75083168
ENSG0000014	DPP9	protein_codir	-0.07634759	0.56819861	0.70198809
ENSG0000017	RIOX1	protein_codir	-0.07637089	0.70458569	0.80774498
ENSG0000015	GART	protein_codir	-0.0763729	0.60998303	0.73541976
ENSG0000014	ELAC1	protein_codir	-0.07638563	0.66919085	0.78143015
ENSG0000028	RP11-122G18	lncRNA	-0.07646136	0.75607558	0.84538934
ENSG0000016	DNASE1L2	protein_codir	-0.07650177	0.87077779	0.92329389
ENSG0000014	GABPB2	protein_codir	-0.0765189	0.63023636	0.75098703
ENSG0000009	SNAP29	protein_codir	-0.07657225	0.50282081	0.64758886
ENSG0000025	RP11-644F5.1	protein_codir	-0.07670001	0.82721801	0.8953799
ENSG0000016	API5	protein_codir	-0.07674506	0.58095213	0.71261259
ENSG0000013	PRKAA1	protein_codir	-0.07676347	0.59521227	0.72367667
ENSG0000023	GNG12-AS1	lncRNA	-0.07677434	0.90415812	0.94312829
ENSG0000026	RP11-424G14	lncRNA	-0.0767863	0.88061079	0.92911872
ENSG0000026	TMEM202-AS	lncRNA	-0.07684412	0.69711476	0.80182858
ENSG0000009	TMEM101	protein_codir	-0.07685524	0.50836144	0.65273316
ENSG0000008	UBA5	protein_codir	-0.07685536	0.36741592	0.52110981
ENSG0000027	LLNLR-268E12	lncRNA	-0.07689671	0.90607341	0.94419621
ENSG0000017	PDZK1	protein_codir	-0.07702195	0.87714634	0.92714723
ENSG0000010	PSMA3	protein_codir	-0.0770349	0.47419094	0.62266586
ENSG0000015	DLAT	protein_codir	-0.07721551	0.63997408	0.7585895
ENSG0000015	MIER3	protein_codir	-0.07724039	0.6359065	0.75562457
ENSG0000000	ARF5	protein_codir	-0.07724252	0.54043168	0.67946774
ENSG0000000	TSPAN6	protein_codir	-0.07750773	0.73940292	0.83339353
ENSG0000011	BRIX1	protein_codir	-0.07754379	0.5663988	0.70049032
ENSG0000011	PTPMT1	protein_codir	-0.07759046	0.38844993	0.54144904
ENSG0000016	NDUFV1	protein_codir	-0.07763163	0.58240618	0.7136672
ENSG0000026	RP5-1142A6.9	lncRNA	-0.07772011	0.85888274	0.91587957
ENSG0000022	RP11-536C5.7	lncRNA	-0.077805	0.86132925	0.91739603
ENSG0000018	ZNF707	protein_codir	-0.07786092	0.6718598	0.7835811
ENSG0000014	ITPRIP	protein_codir	-0.07791471	0.85251517	0.91226488
ENSG0000016	HSP90B1	protein_codir	-0.07796043	0.67968962	0.7894701
ENSG0000027	RP11-1017G2	lncRNA	-0.07802874	0.74349184	0.83615829

ENSG0000022	LINC00630	lncRNA	-0.07803077	0.67359902	0.78471487
ENSG0000022	RTCA-AS1	lncRNA	-0.07805434	0.78227899	0.86392907
ENSG0000015	ZNF230	protein_codir	-0.07808798	0.7136164	0.81434014
ENSG0000011	MAGOHB	protein_codir	-0.07817982	0.52156945	0.66413481
ENSG0000010	UBTF	protein_codir	-0.07818621	0.4779219	0.62583932
ENSG0000028	RP11-286O18	lncRNA	-0.07829512	0.94370798	0.96701244
ENSG0000025	ATXN7L3B	protein_codir	-0.07835768	0.56062688	0.6956206
ENSG0000016	RGPD8	protein_codir	-0.07836003	0.74351277	0.83615829
ENSG0000010	TRIM37	protein_codir	-0.07842951	0.54484947	0.68300924
ENSG0000017	TSNARE1	protein_codir	-0.07843513	0.52225996	0.66473825
ENSG0000016	HOOK3	protein_codir	-0.07844432	0.67697085	0.78728147
ENSG0000007	MCCC1	protein_codir	-0.07849372	0.54989037	0.68714527
ENSG0000023	SHROOM3-AS	lncRNA	-0.07853203	0.89022866	0.93462849
ENSG0000018	RBM34	protein_codir	-0.07856364	0.42234518	0.5743279
ENSG0000011	ATF2	protein_codir	-0.07859905	0.47775654	0.62574145
ENSG0000014	TPRKB	protein_codir	-0.07861273	0.49660076	0.64247098
ENSG0000001	IFFO1	protein_codir	-0.0786967	0.71056049	0.81197363
ENSG0000026	CTB-25B13.12	lncRNA	-0.07877083	0.60925197	0.73473873
ENSG0000018	BCAP31	protein_codir	-0.07888446	0.44216224	0.59321519
ENSG0000011	PPP6C	protein_codir	-0.07891039	0.58161617	0.71295259
ENSG0000014	NAF1	protein_codir	-0.07900185	0.80341567	0.87816458
ENSG0000007	ZNF638	protein_codir	-0.07906583	0.50695521	0.65135149
ENSG0000018	ANGPTL5	protein_codir	-0.07930566	0.88388467	0.93115014
ENSG0000016	GDF9	protein_codir	-0.07931778	0.86979063	0.92270768
ENSG0000018	MFSD5	protein_codir	-0.07937647	0.63941622	0.75816417
ENSG0000013	APC	protein_codir	-0.07940391	0.6886907	0.79565137
ENSG0000021	DDX47	protein_codir	-0.07944095	0.39458867	0.54758429
ENSG0000019	FAN1	protein_codir	-0.07946938	0.65051989	0.76662368
ENSG0000011	PANX1	protein_codir	-0.07950449	0.78775819	0.86743635
ENSG0000015	CCL28	protein_codir	-0.07952429	0.72133379	0.82010247
ENSG0000024	FAM138D	lncRNA	-0.07959972	0.97249546	NA
ENSG0000011	GHR	protein_codir	-0.07960729	0.82930378	0.89698349
ENSG0000016	SLC9B2	protein_codir	-0.07968506	0.61901512	0.74248012
ENSG0000016	BRD3	protein_codir	-0.07970569	0.53868111	0.67810821
ENSG0000017	ARL10	protein_codir	-0.07979364	0.52970569	0.67104565
ENSG0000027	AL133243.1	lncRNA	-0.07989793	0.80331809	0.87813322
ENSG0000014	PI4KB	protein_codir	-0.08004085	0.18487459	0.31800006
ENSG0000013	ISCA1	protein_codir	-0.08015457	0.60755403	0.73351586
ENSG0000027	RP11-575F12.	lncRNA	-0.08022633	0.91127078	0.94706874
ENSG0000016	CAMLG	protein_codir	-0.08026582	0.62671166	0.74833669
ENSG0000004	MRPS10	protein_codir	-0.08031901	0.51898926	0.66191762
ENSG0000010	GSKIP	protein_codir	-0.0803398	0.41098468	0.56393248
ENSG0000028	RP11-196B3.6	lncRNA	-0.0804145	0.90382511	0.94294967
ENSG0000015	MIA3	protein_codir	-0.08045542	0.51319003	0.65681079
ENSG0000008	SMARCA2	protein_codir	-0.08051914	0.57964051	0.71144607
ENSG0000016	ANKRD11	protein_codir	-0.0805919	0.3948838	0.54785466
ENSG0000011	RAB29	protein_codir	-0.08062876	0.59221035	0.72117496

ENSG0000011	YIPF4	protein_codir	-0.08092527	0.43124293	0.582527
ENSG0000013	LLPH	protein_codir	-0.08094835	0.34823718	0.50158067
ENSG0000004	VPS13D	protein_codir	-0.08098202	0.71915783	0.81863864
ENSG0000011	IDE	protein_codir	-0.08105199	0.50912086	0.65343486
ENSG0000014	LAMTOR1	protein_codir	-0.08116276	0.49669561	0.6425335
ENSG0000022	ARIH2OS	lncRNA	-0.08121036	0.75529347	0.84478843
ENSG0000019	CTNND1	protein_codir	-0.08126668	0.58417103	0.71510012
ENSG0000010	DHX35	protein_codir	-0.08136232	0.59130854	0.72057927
ENSG0000024	ARPC4	protein_codir	-0.08144506	0.55501215	0.69109356
ENSG0000028	RP11-483F11.	protein_codir	-0.08153766	0.92651906	0.95648785
ENSG0000011	E2F3	protein_codir	-0.08162186	0.63425156	0.75433958
ENSG0000011	STRN	protein_codir	-0.08164205	0.64755083	0.7642987
ENSG0000013	CDC16	protein_codir	-0.08167523	0.22812564	0.37046273
ENSG0000014	NICN1	protein_codir	-0.08171867	0.57722157	0.70951757
ENSG0000003	PEX3	protein_codir	-0.08186515	0.61900878	0.74248012
ENSG0000015	DEDD	protein_codir	-0.08205759	0.57627401	0.70874804
ENSG0000021	TM6SF2	protein_codir	-0.08207218	0.85181391	0.91183311
ENSG0000016	CCNYL1	protein_codir	-0.08217263	0.72128588	0.82010192
ENSG0000014	CCNB3	protein_codir	-0.08222906	0.83386909	0.89991264
ENSG0000014	CYP39A1	protein_codir	-0.08227165	0.78412341	0.86505386
ENSG0000019	SYNGAP1	protein_codir	-0.08236751	0.56197178	0.6967672
ENSG0000016	UQCRFS1	protein_codir	-0.08252712	0.53560544	0.67592176
ENSG0000020	CCHCR1	protein_codir	-0.08252754	0.62880743	0.74985794
ENSG0000016	BNIPL	protein_codir	-0.08257446	0.89099604	0.93503972
ENSG0000011	OGG1	protein_codir	-0.08262955	0.69288852	0.7983972
ENSG0000017	NUDT6	protein_codir	-0.08267713	0.74152846	0.83490343
ENSG0000025	SLC10A5	protein_codir	-0.08277032	0.877564	0.92735653
ENSG0000014	TMEM168	protein_codir	-0.08277072	0.44059558	0.59154433
ENSG0000013	GYPC	protein_codir	-0.08278561	0.52538239	0.66729797
ENSG0000017	ZNF440	protein_codir	-0.08279828	0.68372993	0.79218924
ENSG0000020	SARNP	protein_codir	-0.0829136	0.48777845	0.6348337
ENSG0000025	CLSTN2-AS1	lncRNA	-0.08312308	0.95470758	0.97338587
ENSG0000018	ZNF383	protein_codir	-0.08325518	0.64062891	0.75901555
ENSG0000020	METTL6	protein_codir	-0.08328246	0.47011061	0.6191754
ENSG0000025	AC009133.15	lncRNA	-0.08336704	0.76776555	0.85358791
ENSG0000027	RP11-358L22.	lncRNA	-0.0833834	0.79839438	0.87468926
ENSG0000020	INPP5B	protein_codir	-0.08348305	0.5882413	0.71839515
ENSG0000016	NACC1	protein_codir	-0.08367412	0.59825457	0.72635242
ENSG0000013	IER3IP1	protein_codir	-0.08367765	0.47875654	0.62657579
ENSG0000011	UMPS	protein_codir	-0.08374389	0.60742024	0.73338638
ENSG0000014	ARNT	protein_codir	-0.0838331	0.55248519	0.6891931
ENSG0000015	CWC15	protein_codir	-0.08398818	0.31722336	0.46909248
ENSG0000010	ZNF500	protein_codir	-0.08399393	0.620312	0.74358827
ENSG0000022	LINC01985	lncRNA	-0.08401227	0.89425901	0.93700431
ENSG0000010	TBC1D17	protein_codir	-0.08402438	0.65329919	0.76899189
ENSG0000014	TSPAN3	protein_codir	-0.08405538	0.32430922	0.47666138
ENSG0000007	GLI2	protein_codir	-0.08411028	0.71947795	0.81878785

ENSG0000017	UQCRH	protein_codir	-0.08411713	0.5534963	0.68980993
ENSG0000025	CTD-2152M2(	lncRNA	-0.08412278	0.89929798	0.93985962
ENSG0000016	TRUB2	protein_codir	-0.08429322	0.59557581	0.72399119
ENSG0000028	RP11-284E5.1	lncRNA	-0.08431877	0.71430744	0.81471637
ENSG0000020	DDR1	protein_codir	-0.08443437	0.66340092	0.77729229
ENSG0000005	MRTO4	protein_codir	-0.08444534	0.6195522	0.74296769
ENSG0000013	KLHL36	protein_codir	-0.08446126	0.5553332	0.69128528
ENSG0000014	USP21	protein_codir	-0.08457489	0.64633002	0.7635735
ENSG0000010	AP1S1	protein_codir	-0.08474367	0.62947569	0.75043675
ENSG0000000	SPATA20	protein_codir	-0.08478479	0.65445737	0.76991705
ENSG0000013	MOSPD2	protein_codir	-0.08478548	0.62130641	0.74436032
ENSG0000009	BLVRB	protein_codir	-0.0852268	0.5901916	0.71988928
ENSG0000010	SBF1	protein_codir	-0.0853283	0.52091785	0.66346262
ENSG0000011	CREB1	protein_codir	-0.0853644	0.5664537	0.70049032
ENSG0000010	SDR39U1	protein_codir	-0.08541639	0.7097709	0.81148359
ENSG0000016	PSMD6	protein_codir	-0.08545216	0.30852891	0.46010918
ENSG0000010	HLF	protein_codir	-0.08545327	0.84199324	0.9056723
ENSG0000011	SCP2	protein_codir	-0.08548443	0.53944744	0.67860896
ENSG0000007	RUNX1T1	protein_codir	-0.08579158	0.78944191	0.86870062
ENSG0000017	LRRC37A3	protein_codir	-0.08580799	0.72882765	0.82561807
ENSG0000017	HECTD4	protein_codir	-0.08581311	0.40926665	0.56224876
ENSG0000015	COPG2	protein_codir	-0.0858841	0.57155213	0.70479733
ENSG0000028	RP11-28H5.3	lncRNA	-0.08592825	0.95550422	0.97394445
ENSG0000015	DCP1B	protein_codir	-0.08593524	0.63652765	0.75629758
ENSG0000021	RP11-248G5.8	lncRNA	-0.08597993	0.97288753	0.98451261
ENSG0000018	UTP11	protein_codir	-0.08599159	0.62362006	0.74612217
ENSG0000026	RP11-20I23.6	lncRNA	-0.08617046	0.81528032	0.88618821
ENSG0000018	KCNA4	protein_codir	-0.08618025	0.89247852	0.93591714
ENSG0000006	FGFR2	protein_codir	-0.08621051	0.87835777	0.92776278
ENSG0000000	RPAP3	protein_codir	-0.08621869	0.48492962	0.63234767
ENSG0000011	MAPK14	protein_codir	-0.08625062	0.44580959	0.59674618
ENSG0000011	CNPPD1	protein_codir	-0.0862611	0.43453517	0.58594255
ENSG0000012	Aug-02	protein_codir	-0.0863152	0.70143661	0.80542693
ENSG0000014	RHBDL3	protein_codir	-0.0863189	0.85510047	0.91336696
ENSG0000001	HIVEP2	protein_codir	-0.08648202	0.70976275	0.81148359
ENSG0000008	DHX32	protein_codir	-0.08650421	0.4895977	0.63650818
ENSG0000000	NDUFAF7	protein_codir	-0.08656523	0.48502681	0.63239317
ENSG0000013	NSUN5	protein_codir	-0.08661794	0.65039643	0.76654361
ENSG0000017	ORMDL3	protein_codir	-0.08663966	0.56294738	0.69756084
ENSG0000026	LINC02080	lncRNA	-0.08677346	0.90266735	0.94216885
ENSG0000025	RP11-182J1.1	lncRNA	-0.08682413	0.84071015	0.90475022
ENSG0000018	BTBD9	protein_codir	-0.08688608	0.57773497	0.70999066
ENSG0000024	PGAM5	protein_codir	-0.08689575	0.4531704	0.6036735
ENSG0000016	KCTD6	protein_codir	-0.08691196	0.55202442	0.6887949
ENSG0000017	USP19	protein_codir	-0.08704104	0.38034668	0.53366441
ENSG0000016	MLST8	protein_codir	-0.08704898	0.43467647	0.58607586
ENSG0000014	ADAMTSL4	protein_codir	-0.08707704	0.72938512	0.82603402

ENSG0000014	CPSF7	protein_codir	-0.08712422	0.42121543	0.57318668
ENSG0000001	PIK3C2A	protein_codir	-0.08715395	0.68913489	0.79587222
ENSG0000013	TOMM40	protein_codir	-0.08715538	0.62364143	0.74612217
ENSG0000021	PAXIP1-AS2	lncRNA	-0.08718899	0.61818468	0.74187533
ENSG0000010	DNAJC3	protein_codir	-0.08726462	0.6859292	0.79378755
ENSG0000015	EXOG	protein_codir	-0.08743728	0.62575389	0.74761322
ENSG0000016	MAPK1IP1L	protein_codir	-0.08749229	0.56142176	0.69629408
ENSG0000016	RAD54L2	protein_codir	-0.0874993	0.43307683	0.58440405
ENSG0000010	MAZ	protein_codir	-0.08756021	0.49058628	0.63734837
ENSG0000026	MRPS21	protein_codir	-0.08763982	0.4894668	0.63640131
ENSG0000015	RNF207	protein_codir	-0.08764887	0.79606448	0.873309
ENSG0000018	COMMD6	protein_codir	-0.08766119	0.51920998	0.66201549
ENSG0000020	LSM2	protein_codir	-0.08775588	0.45695673	0.60748675
ENSG0000027	MRM1	protein_codir	-0.08799599	0.71276492	0.8138308
ENSG0000013	HUS1	protein_codir	-0.08802704	0.36577344	0.51965754
ENSG0000009	ZDHHC8	protein_codir	-0.08806284	0.44591465	0.59685579
ENSG0000014	ARFGAP2	protein_codir	-0.08812495	0.38514632	0.53837448
ENSG0000027	RP11-679B19.	lncRNA	-0.08834942	0.77581181	0.8593512
ENSG0000011	KMT2A	protein_codir	-0.08852595	0.63112896	0.75169391
ENSG0000025	SNHG21	lncRNA	-0.08853031	0.80316728	0.87803207
ENSG0000011	GOLPH3	protein_codir	-0.08858781	0.50398345	0.64871017
ENSG0000008	PUM3	protein_codir	-0.08859381	0.51175787	0.65547915
ENSG0000015	NMT2	protein_codir	-0.08862158	0.40392389	0.55702379
ENSG0000013	MFSD13A	protein_codir	-0.08867593	0.78596465	0.86622017
ENSG0000017	IP6K1	protein_codir	-0.08902954	0.35870031	0.51234951
ENSG0000018	THAP7	protein_codir	-0.08904998	0.39099305	0.54384993
ENSG0000019	NOL8	protein_codir	-0.08909085	0.58235248	0.71363306
ENSG0000011	ING4	protein_codir	-0.08913553	0.54863932	0.68609822
ENSG0000016	UBE2J2	protein_codir	-0.08919732	0.41164938	0.56473232
ENSG0000024	SPTY2D1OS	protein_codir	-0.08931418	0.85521609	0.91336696
ENSG0000011	PHF13	protein_codir	-0.08936604	0.74444842	0.83675845
ENSG0000018	CEP57L1	protein_codir	-0.08937501	0.52241205	0.66481346
ENSG0000024	LINC00942	lncRNA	-0.08938543	0.88743952	0.93336429
ENSG0000012	COPB1	protein_codir	-0.08945763	0.52909219	0.6706221
ENSG0000015	PTPDC1	protein_codir	-0.08946168	0.67045316	0.78256841
ENSG0000016	SERPINF2	protein_codir	-0.08963171	0.7975434	0.87413289
ENSG0000000	SPAG9	protein_codir	-0.08966231	0.6734331	0.78467625
ENSG0000018	TMED9	protein_codir	-0.08967423	0.61614709	0.74004088
ENSG0000018	IRS2	protein_codir	-0.08970466	0.79685167	0.87382745
ENSG0000010	EIF3B	protein_codir	-0.08970852	0.41038239	0.56332994
ENSG0000026	ZNF865	protein_codir	-0.08978084	0.5067857	0.65123729
ENSG0000025	LINC02254	lncRNA	-0.08998111	0.9633233	NA
ENSG0000013	MTHFS	protein_codir	-0.09012702	0.64705892	0.76415754
ENSG0000013	CAPRIN1	protein_codir	-0.0901298	0.57927793	0.71122227
ENSG0000028	RP11-247I13.1	lncRNA	-0.0905596	0.8539805	0.91288916
ENSG0000028	CTD-2547L16.	lncRNA	-0.09065899	0.95672188	NA
ENSG0000023	HCG25	lncRNA	-0.0908808	0.87776385	0.9274965

ENSG0000015	GNE	protein_codir	-0.09095952	0.56073534	0.69569266
ENSG0000013	FNBP1L	protein_codir	-0.09097392	0.67912442	0.78902185
ENSG0000014	PDCD11	protein_codir	-0.09107849	0.54077519	0.67963752
ENSG0000026	LINC00663	lncRNA	-0.09112993	0.67848841	0.78838241
ENSG0000005	RIOK2	protein_codir	-0.09118614	0.48045705	0.62820601
ENSG0000009	SEC22C	protein_codir	-0.09132859	0.4217773	0.57380992
ENSG0000015	SPPL3	protein_codir	-0.09133479	0.38340007	0.53677564
ENSG0000008	EIF3I	protein_codir	-0.09139253	0.4724756	0.6210976
ENSG0000015	SLC16A1	protein_codir	-0.0915992	0.73145792	0.82734214
ENSG0000022	ZNF717	protein_codir	-0.09167502	0.6125515	0.73736411
ENSG0000023	GET4	protein_codir	-0.09176121	0.5270115	0.66872135
ENSG0000009	SNAP23	protein_codir	-0.09179232	0.47278995	0.62131493
ENSG0000023	RP11-230B22.	lncRNA	-0.0919063	0.90832784	0.94540601
ENSG0000012	EIF2S2	protein_codir	-0.09195812	0.37566257	0.52916157
ENSG0000010	RANGAP1	protein_codir	-0.09198504	0.44641184	0.59740758
ENSG0000003	ATP6V0A1	protein_codir	-0.09203905	0.49364385	0.64005164
ENSG0000018	OVCH1	protein_codir	-0.09238221	0.86496176	0.91956288
ENSG0000006	FTSJ1	protein_codir	-0.09251293	0.50133207	0.6461091
ENSG0000016	ARFIP1	protein_codir	-0.09251888	0.39098907	0.54384993
ENSG0000024	MIF	protein_codir	-0.09278974	0.5905118	0.72005368
ENSG0000028	RP11-450B15.	lncRNA	-0.09280726	0.90290733	0.94234812
ENSG0000016	ITIH3	protein_codir	-0.09284294	0.8269555	0.895285
ENSG0000026	HOMER3-AS1	lncRNA	-0.0928572	0.77341233	0.85744987
ENSG0000008	MOK	protein_codir	-0.09286304	0.7266203	0.82389002
ENSG0000012	KNSTRN	protein_codir	-0.09289017	0.72973953	0.8261428
ENSG0000011	RAD23B	protein_codir	-0.092891	0.51708242	0.66024763
ENSG0000010	GFUS	protein_codir	-0.09290237	0.54702609	0.68477432
ENSG0000025	HOXA10-AS	lncRNA	-0.09304643	NA	NA
ENSG0000010	PLOD3	protein_codir	-0.09314876	0.54676044	0.68450382
ENSG0000017	RARG	protein_codir	-0.09320025	0.62879538	0.74985794
ENSG0000016	MBL2	protein_codir	-0.09328393	0.96852272	NA
ENSG0000011	TENT4A	protein_codir	-0.09334078	0.56965231	0.70328075
ENSG0000007	XAB2	protein_codir	-0.09347668	0.50033235	0.64564215
ENSG0000013	DPM2	protein_codir	-0.09350494	0.57409827	0.70681083
ENSG0000011	RSRP1	protein_codir	-0.09352219	0.7776732	0.86075714
ENSG0000025	RP11-202H2.1	lncRNA	-0.09352699	0.93980697	0.96436462
ENSG0000010	LHPP	protein_codir	-0.09359011	0.54847022	0.68597239
ENSG0000011	DR1	protein_codir	-0.09370591	0.49929433	0.64483818
ENSG0000011	CHST10	protein_codir	-0.09376566	0.48971911	0.63656986
ENSG0000018	TLCD2	protein_codir	-0.09378845	0.74311252	0.8359897
ENSG0000016	ARIH1	protein_codir	-0.09383799	0.52826384	0.66975658
ENSG0000014	TMEM50B	protein_codir	-0.09388893	0.51207157	0.65572891
ENSG0000026	RP11-73M18.	lncRNA	-0.09389712	0.74370413	0.83629536
ENSG0000011	LNPEP	protein_codir	-0.09427995	0.66190018	0.77596278
ENSG0000016	MED11	protein_codir	-0.09428015	0.53998111	0.67903288
ENSG0000014	DET1	protein_codir	-0.09435567	0.63967304	0.75838039
ENSG0000006	BORCS8-MEF2	protein_codir	-0.09447577	0.77942573	0.86200603

ENSG0000019	MAN1A2	protein_codir	-0.09454698	0.58366767	0.71464231
ENSG0000013	EPRS1	protein_codir	-0.09457086	0.56304929	0.69756084
ENSG0000016	TAPT1	protein_codir	-0.09462109	0.56678724	0.70074591
ENSG0000017	ZDHHC21	protein_codir	-0.0946242	0.45848118	0.60881011
ENSG0000016	WRN	protein_codir	-0.09462729	0.48975777	0.63656986
ENSG0000014	EIF2B5	protein_codir	-0.0946909	0.51218541	0.65584427
ENSG0000018	TRNAU1AP	protein_codir	-0.09470215	0.49643605	0.64228798
ENSG0000017	MFN1	protein_codir	-0.09479404	0.46934164	0.61863643
ENSG0000014	PIGK	protein_codir	-0.09484434	0.5963435	0.72463919
ENSG0000026	MAN1B1-DT	lncRNA	-0.09492594	0.69030912	0.79685451
ENSG0000028	AC116366.7	protein_codir	-0.09505709	0.71468759	0.81494804
ENSG0000015	UEVLD	protein_codir	-0.09518309	0.59131698	0.72057927
ENSG0000020	CHIC1	protein_codir	-0.0951942	0.51646928	0.6596172
ENSG0000026	RP11-1055B8.	lncRNA	-0.09520969	0.90336897	0.94265182
ENSG0000025	RP11-849H4.4	lncRNA	-0.09532914	0.61311548	0.7376508
ENSG0000012	RAB2B	protein_codir	-0.09534248	0.42861156	0.58024955
ENSG0000019	WDR5B	protein_codir	-0.09552126	0.70830285	0.81057795
ENSG0000028	RP6-91H8.6	lncRNA	-0.09552352	0.92318129	0.95468676
ENSG0000016	ZNF23	protein_codir	-0.09558628	0.52595734	0.66781325
ENSG0000013	RP11-809H16	lncRNA	-0.09567945	0.93988553	0.96436462
ENSG0000010	NELFCD	protein_codir	-0.09568002	0.46770743	0.61723302
ENSG0000028	RP11-147I11.!	lncRNA	-0.09573055	0.96059058	NA
ENSG0000013	CDK7	protein_codir	-0.09591644	0.22161306	0.36265865
ENSG0000024	RP11-499E18.	lncRNA	-0.09594597	0.78041671	0.86272201
ENSG0000011	UCHL3	protein_codir	-0.09596609	0.63044052	0.75110068
ENSG0000014	CHST7	protein_codir	-0.09611229	0.66609537	0.77927283
ENSG0000016	TIGD6	protein_codir	-0.09611907	0.43774088	0.58899764
ENSG0000000	HCCS	protein_codir	-0.09628696	0.49212464	0.63871447
ENSG0000012	PLCG1	protein_codir	-0.09637969	0.51334893	0.65685234
ENSG0000012	ASH2L	protein_codir	-0.0964009	0.32681884	0.47904986
ENSG0000015	MTERF3	protein_codir	-0.09655342	0.49377033	0.64010262
ENSG0000013	GPR12	protein_codir	-0.0966146	0.92666692	0.95650756
ENSG0000027	ENSG0000027	protein_codir	-0.09661587	0.74272638	0.83567307
ENSG0000010	CYB5B	protein_codir	-0.09664531	0.2853834	0.43493294
ENSG0000016	ZNF354A	protein_codir	-0.09672562	0.51336953	0.65685234
ENSG0000000	PNPLA4	protein_codir	-0.09676023	0.59898279	0.72682122
ENSG0000013	MAP3K12	protein_codir	-0.09681044	0.57923911	0.71121207
ENSG0000013	GPS2	protein_codir	-0.09681607	0.57160605	0.70479733
ENSG0000013	MYBBP1A	protein_codir	-0.09681726	0.51919882	0.66201549
ENSG0000026	RP11-264B17.	lncRNA	-0.09690062	0.8143168	0.88579941
ENSG0000004	FAM120A	protein_codir	-0.0969217	0.52356959	0.66567468
ENSG0000027	RP11-347I19.7	lncRNA	-0.09696344	0.83338717	0.89963862
ENSG0000025	WAC-AS1	lncRNA	-0.09711132	0.3875208	0.54054465
ENSG0000006	FUNDC1	protein_codir	-0.09711514	0.35456834	0.50814491
ENSG0000016	POP5	protein_codir	-0.09724545	0.5705112	0.70399497
ENSG0000022	RP1-310O13.7	lncRNA	-0.09724705	0.91586721	0.95001426
ENSG0000018	TXNRD2	protein_codir	-0.09725685	0.51814548	0.66114654

ENSG0000019	ZNF782	protein_codir	-0.09730255	0.57480711	0.70739974
ENSG0000018	ARHGEF37	protein_codir	-0.09735551	0.82379273	0.89284081
ENSG0000010	SMC3	protein_codir	-0.09735612	0.44303653	0.59409958
ENSG0000009	POLRMT	protein_codir	-0.09738402	0.43776044	0.58899764
ENSG0000010	DHRS7	protein_codir	-0.09742015	0.45160222	0.60219443
ENSG0000025	RP11-87C12.5	lncRNA	-0.09751467	0.86476091	0.91942014
ENSG0000024	LINC02288	lncRNA	-0.0975432	0.85991227	0.91631084
ENSG0000011	LDHB	protein_codir	-0.09756521	0.59167193	0.72080005
ENSG0000026	RP5-1085F17.	lncRNA	-0.09770594	0.79445843	0.87230935
ENSG0000011	IFI27L2	protein_codir	-0.09778986	0.52094547	0.66346262
ENSG0000027	RP11-63G10.3	lncRNA	-0.09779167	0.93452401	NA
ENSG0000026	CTD-2616J11.	lncRNA	-0.09780767	0.91441912	0.94911132
ENSG0000028	PTENP1-AS	lncRNA	-0.09786417	0.90200249	0.94172402
ENSG0000010	RPRD1B	protein_codir	-0.09791312	0.3398458	0.49278355
ENSG0000013	AP1AR	protein_codir	-0.09795397	0.46920006	0.61857898
ENSG0000008	COBLL1	protein_codir	-0.09796143	0.72963027	0.82612743
ENSG0000012	VPS26A	protein_codir	-0.0979661	0.49070462	0.63744058
ENSG0000013	ZC3H4	protein_codir	-0.098053	0.49266033	0.63919947
ENSG0000010	BLMH	protein_codir	-0.09807141	0.24339812	0.38848267
ENSG0000000	FAM76A	protein_codir	-0.09809842	0.55667274	0.69254994
ENSG0000011	RO60	protein_codir	-0.098112	0.42969572	0.58124962
ENSG0000025	RP11-459E5.1	lncRNA	-0.09821134	0.91466951	0.94931504
ENSG0000016	ZNF326	protein_codir	-0.09829457	0.45141989	0.60206757
ENSG0000017	PLD6	protein_codir	-0.09834017	0.60924863	0.73473873
ENSG0000027	RP11-403P17.	lncRNA	-0.09874821	0.95231839	NA
ENSG0000005	AP5M1	protein_codir	-0.09881455	0.47735848	0.62536836
ENSG0000009	DHPS	protein_codir	-0.09883993	0.47631015	0.62449787
ENSG0000016	THAP8	protein_codir	-0.09894073	0.55924866	0.69469071
ENSG0000016	SLC3A2	protein_codir	-0.09902553	0.49774702	0.64347169
ENSG0000013	SAA2	protein_codir	-0.09914384	0.88945039	0.93419857
ENSG0000017	ZNF212	protein_codir	-0.09930009	0.53749227	0.67730602
ENSG0000028	RP5-994D16.1	protein_codir	-0.09932395	0.59839612	0.72644745
ENSG0000010	RNF40	protein_codir	-0.0993322	0.11023165	0.21699858
ENSG0000011	AMPD2	protein_codir	-0.09939117	0.49856046	0.64425189
ENSG0000016	SMIM4	protein_codir	-0.09941829	0.51730454	0.66040912
ENSG0000028	RP11-453A3.1	lncRNA	-0.09959012	0.84759031	0.90915075
ENSG0000015	CCDC50	protein_codir	-0.09961262	0.64297337	0.76087937
ENSG0000016	TIGD4	protein_codir	-0.09966111	0.82467755	0.89356743
ENSG0000013	ANKRD42	protein_codir	-0.0997005	0.53878595	0.67814747
ENSG0000015	MR1	protein_codir	-0.09970645	0.53352601	0.67426165
ENSG0000023	OBI1-AS1	lncRNA	-0.09978573	0.9595628	NA
ENSG0000015	TMEM251	protein_codir	-0.09997911	0.50650973	0.6509911
ENSG0000004	GOPC	protein_codir	-0.1001681	0.42888255	0.58045491
ENSG0000018	PGP	protein_codir	-0.10022344	0.557851	0.693485
ENSG0000012	SNRNP27	protein_codir	-0.10026703	0.37652293	0.53012519
ENSG0000017	PACS1	protein_codir	-0.10033687	0.36659272	0.52050023
ENSG0000013	EXOC4	protein_codir	-0.1003563	0.39374194	0.54670986

ENSG0000016	USP39	protein_codir	-0.10036323	0.28422628	0.4338043
ENSG0000012	ILF3	protein_codir	-0.10039113	0.48112173	0.62870203
ENSG0000016	TMEM184C	protein_codir	-0.10040466	0.53402309	0.67462122
ENSG0000018	ZNF875	protein_codir	-0.10043027	0.53793916	0.67754479
ENSG0000012	NAA60	protein_codir	-0.10047345	0.18271524	0.31511676
ENSG0000018	RBM10	protein_codir	-0.1005331	0.36516585	0.51900791
ENSG0000013	ACTL6A	protein_codir	-0.10067546	0.36402992	0.5180599
ENSG0000014	RPTOR	protein_codir	-0.10073394	0.57009843	0.70367425
ENSG0000026	TMEM185A	protein_codir	-0.10076807	0.45289142	0.60333097
ENSG0000008	BRAP	protein_codir	-0.10096136	0.35087971	0.50446606
ENSG0000012	CAAP1	protein_codir	-0.10126766	0.31063326	0.46241731
ENSG0000015	BICD1	protein_codir	-0.10131051	0.52458214	0.66654788
ENSG0000016	TERF2IP	protein_codir	-0.1013825	0.20720056	0.34564713
ENSG0000014	RETREG2	protein_codir	-0.10152207	0.25145859	0.39760054
ENSG0000023	AC074117.10	lncRNA	-0.10161298	0.74333305	0.83615829
ENSG0000015	XPC	protein_codir	-0.10164991	0.50123039	0.6461091
ENSG0000011	TFG	protein_codir	-0.10166662	0.3922811	0.54522967
ENSG0000018	BCDIN3D	protein_codir	-0.10171308	0.62933256	0.7502985
ENSG0000014	NBPF3	protein_codir	-0.10173438	0.79655443	0.87360361
ENSG0000019	MARCHF5	protein_codir	-0.10175087	0.45351031	0.60400978
ENSG0000006	WNK1	protein_codir	-0.10192148	0.55535587	0.69128528
ENSG0000027	ILRUN-AS1	lncRNA	-0.10194277	0.6838227	0.79224761
ENSG0000015	JAZF1	protein_codir	-0.10201384	0.60626325	0.73256588
ENSG0000016	C1orf115	protein_codir	-0.10211616	0.74439527	0.83673276
ENSG0000010	EIF3J	protein_codir	-0.1021457	0.44359266	0.59458552
ENSG0000011	RCOR3	protein_codir	-0.10219081	0.52581853	0.66769839
ENSG0000017	ZNF622	protein_codir	-0.10220707	0.15743585	0.28232555
ENSG0000028	AC013271.5	lncRNA	-0.10225542	0.93041448	0.95877506
ENSG0000016	ZNF598	protein_codir	-0.10229951	0.45751768	0.60811541
ENSG0000018	ZSCAN22	protein_codir	-0.10235918	0.5465897	0.68440356
ENSG0000010	GMPR2	protein_codir	-0.1025487	0.23002427	0.37238557
ENSG0000010	KDELR3	protein_codir	-0.10261792	0.69035755	0.79687711
ENSG0000012	GOT1	protein_codir	-0.10263959	0.27499332	0.42310131
ENSG0000016	GSTM4	protein_codir	-0.10275242	0.70628257	0.80907642
ENSG0000016	TBC1D10B	protein_codir	-0.10276981	0.35532132	0.50897079
ENSG0000018	WDR53	protein_codir	-0.10309337	0.40361264	0.55677661
ENSG0000016	SP2	protein_codir	-0.10322715	0.42938428	0.58101086
ENSG0000015	ATP5MC3	protein_codir	-0.10328051	0.43581495	0.58703774
ENSG0000018	ADO	protein_codir	-0.10340919	0.38534369	0.5385686
ENSG0000013	CHRNA1	protein_codir	-0.10341782	0.8999927	0.94019435
ENSG0000015	NBAS	protein_codir	-0.10344773	0.38034419	0.53366441
ENSG0000015	PCGF6	protein_codir	-0.10347973	0.43644865	0.58771936
ENSG0000007	PTPN23	protein_codir	-0.10353432	0.15650005	0.28099404
ENSG0000016	COA6	protein_codir	-0.10358646	0.56848337	0.70221425
ENSG0000015	PPP1R1C	protein_codir	-0.10363649	0.87980651	0.92880235
ENSG0000028	RP11-421M1.	lncRNA	-0.10372354	0.92401803	0.95525347
ENSG0000022	SRRM5	protein_codir	-0.10389514	0.82954362	0.89714894

ENSG0000027	RP11-54O7.16	lncRNA	-0.1039131	0.95081896	NA
ENSG0000017	AMER3	protein_codir	-0.10396316	0.97095697	NA
ENSG0000013	BEX1	protein_codir	-0.10404849	0.84332193	0.90639867
ENSG0000021	LINC00887	lncRNA	-0.10406899	0.93337547	0.9608337
ENSG0000028	RP11-554A11	protein_codir	-0.10412623	0.75034037	0.84074649
ENSG0000009	EXOC1	protein_codir	-0.10416653	0.34700498	0.50019715
ENSG0000027	RP11-722E23.	lncRNA	-0.10419469	0.80598382	0.87979011
ENSG0000016	TMX3	protein_codir	-0.10423923	0.36000519	0.51381476
ENSG0000012	ETF1	protein_codir	-0.10426459	0.63658001	0.75630281
ENSG0000010	PROCR	protein_codir	-0.10428913	0.78695814	0.86700374
ENSG0000018	C16orf72	protein_codir	-0.10434955	0.54373715	0.68204823
ENSG0000018	LAMTOR4	protein_codir	-0.10435675	0.54218987	0.68094235
ENSG0000016	VMA21	protein_codir	-0.10441241	0.39071729	0.54369174
ENSG0000017	JUN	protein_codir	-0.10444015	0.84756177	0.90915075
ENSG0000013	DPH2	protein_codir	-0.10448849	0.51433997	0.65757111
ENSG0000027	TUBGCP5	protein_codir	-0.10450917	0.42064378	0.57283213
ENSG0000027	RP11-843B15.	lncRNA	-0.10460979	0.83140896	0.89838095
ENSG0000011	SRM	protein_codir	-0.10461137	0.5994368	0.72726652
ENSG0000010	ACO2	protein_codir	-0.10465909	0.2838424	0.43340182
ENSG0000019	ACADSB	protein_codir	-0.104724	0.63930831	0.75810131
ENSG0000005	GYG2	protein_codir	-0.10478057	0.82397017	0.89299809
ENSG0000013	MCCC2	protein_codir	-0.10485718	0.46917883	0.61857898
ENSG0000013	NUP54	protein_codir	-0.10487818	0.25039018	0.39632729
ENSG0000016	NUP35	protein_codir	-0.10493881	0.41748354	0.5701308
ENSG0000007	TCOF1	protein_codir	-0.1049541	0.46304375	0.61298257
ENSG0000018	EPOR	protein_codir	-0.10517503	0.64872896	0.76490476
ENSG0000013	POLN	protein_codir	-0.1054455	0.72357444	0.82165548
ENSG0000008	ZNF416	protein_codir	-0.10556252	0.56567832	0.69993886
ENSG0000016	COG5	protein_codir	-0.10562879	0.54370866	0.68204823
ENSG0000007	CARMIL1	protein_codir	-0.10563773	0.69730426	0.80188618
ENSG0000014	PTGES2	protein_codir	-0.10566674	0.4767149	0.62485125
ENSG0000013	TMEM128	protein_codir	-0.10570393	0.45010477	0.6008591
ENSG0000011	KLHL29	protein_codir	-0.10570757	0.58068676	0.712382
ENSG0000013	CYP27A1	protein_codir	-0.10577901	0.61174528	0.73674042
ENSG0000027	RP11-535A5.1	lncRNA	-0.10593595	0.86816081	0.92182844
ENSG0000024	TMEM141	protein_codir	-0.10599854	0.41605569	0.56882775
ENSG0000026	RP5-1148A21	lncRNA	-0.10603157	0.67209953	0.78379451
ENSG0000027	PPP4R3B	protein_codir	-0.10654548	0.30646048	0.45810676
ENSG0000016	TAF6L	protein_codir	-0.10657965	0.43508957	0.58649319
ENSG0000018	RNLS	protein_codir	-0.10661089	0.52581019	0.66769839
ENSG0000003	MAP2K3	protein_codir	-0.10664715	0.70465362	0.80777508
ENSG0000014	SLX4IP	protein_codir	-0.10667673	0.72963623	0.82612743
ENSG0000027	CTD-3234P18	lncRNA	-0.10667969	0.83671467	0.90185597
ENSG0000014	UCK2	protein_codir	-0.10669803	0.65825367	0.77296741
ENSG0000017	CHCHD1	protein_codir	-0.10676439	0.29133388	0.44120198
ENSG0000013	THUMPD3	protein_codir	-0.1067963	0.42907311	0.58067516
ENSG0000018	ZNF546	protein_codir	-0.106881	0.70274496	0.80635233

ENSG0000024	DDX11-AS1	lncRNA	-0.10692876	0.85381805	0.91285717
ENSG0000014	KLHL8	protein_codir	-0.10694341	0.3663878	0.52028926
ENSG0000007	RHOBTB1	protein_codir	-0.10694382	0.60455454	0.73110891
ENSG0000012	UBR4	protein_codir	-0.106996	0.39668223	0.54952168
ENSG0000023	EPCAM-DT	lncRNA	-0.10716266	0.93655999	0.96237907
ENSG0000016	ASPSCR1	protein_codir	-0.10718653	0.59843185	0.72644745
ENSG0000018	TRAPPC5	protein_codir	-0.10719766	0.47913274	0.62697903
ENSG0000013	ZNF189	protein_codir	-0.10720481	0.40206427	0.55505648
ENSG0000016	SLC35A1	protein_codir	-0.10725369	0.5085832	0.65295718
ENSG0000012	NCLN	protein_codir	-0.10734256	0.5569662	0.69279021
ENSG0000014	FARS2	protein_codir	-0.10735	0.29175497	0.44162176
ENSG0000011	MAPKAP1	protein_codir	-0.10739433	0.27286622	0.42075812
ENSG0000017	RAPH1	protein_codir	-0.10739486	0.72422012	0.82198314
ENSG0000016	CLPB	protein_codir	-0.1074785	0.48918432	0.63615382
ENSG0000005	CUL1	protein_codir	-0.10748452	0.40381383	0.55694677
ENSG0000016	CHRNA5	protein_codir	-0.10755362	0.80717802	0.88060737
ENSG0000025	ZNF252P-AS1	lncRNA	-0.10756658	0.86758666	0.92136064
ENSG0000017	LIG4	protein_codir	-0.10757481	0.47456782	0.62282998
ENSG0000022	LINC00685	lncRNA	-0.10758032	0.88749985	0.93339219
ENSG0000010	VPS35L	protein_codir	-0.10764228	0.37640207	0.529987
ENSG0000025	AP006621.6	lncRNA	-0.10766389	0.93685872	NA
ENSG0000010	DHRS7B	protein_codir	-0.10767914	0.33534147	0.48820005
ENSG0000028	LINC01232	lncRNA	-0.10768251	0.74863448	0.83965258
ENSG0000011	UBE3A	protein_codir	-0.10772648	0.39301037	0.54602346
ENSG0000023	PDYN-AS1	lncRNA	-0.10773043	0.95554492	NA
ENSG0000027	RP11-801F7.1	lncRNA	-0.10774106	0.87057768	0.92318803
ENSG0000022	AC096574.4	lncRNA	-0.10776978	0.90701099	0.94466773
ENSG0000014	CCDC82	protein_codir	-0.10790174	0.31296639	0.46468829
ENSG0000016	TBX10	protein_codir	-0.10791227	0.96963556	NA
ENSG0000024	LINC00636	lncRNA	-0.10793136	0.93339135	0.9608337
ENSG0000017	ADORA2A-AS	lncRNA	-0.10807768	0.92963668	0.95826071
ENSG0000025	PLBD1-AS1	lncRNA	-0.10807907	0.67336513	0.78467407
ENSG0000024	LINC02432	lncRNA	-0.10809513	0.85332573	0.91274327
ENSG0000019	ZNF583	protein_codir	-0.10812163	0.54406548	0.68234361
ENSG0000017	ZNF282	protein_codir	-0.10812864	0.25813577	0.40512018
ENSG0000006	FECH	protein_codir	-0.10814136	0.59843805	0.72644745
ENSG0000017	PLAC8L1	protein_codir	-0.10816237	0.84432788	0.90698258
ENSG0000013	UQCC2	protein_codir	-0.1081686	0.5655269	0.69978285
ENSG0000010	FZD3	protein_codir	-0.1082093	0.7092654	0.8112436
ENSG0000006	HEATR6	protein_codir	-0.10827235	0.56686496	0.70077926
ENSG0000012	TTF1	protein_codir	-0.10831244	0.38813818	0.54115985
ENSG0000024	STMP1	protein_codir	-0.1083324	0.39284735	0.54585188
ENSG0000010	PIN4	protein_codir	-0.10834157	0.5369528	0.67703775
ENSG0000014	NTMT1	protein_codir	-0.10845956	0.4339334	0.58533115
ENSG0000010	HSDL1	protein_codir	-0.10853619	0.42584184	0.57768918
ENSG0000006	WDR3	protein_codir	-0.10876579	0.58969538	0.71952682
ENSG0000016	PPP1R21	protein_codir	-0.10898928	0.3881624	0.5411663

ENSG0000014	MED9	protein_codir	-0.10904753	0.51511031	0.65833819
ENSG000001(	HTR2A	protein_codir	-0.1091629	0.81097989	0.88337591
ENSG000001(	TRIP4	protein_codir	-0.10917225	0.33692124	0.48992921
ENSG0000013	GOLGA1	protein_codir	-0.10917632	0.3001263	0.45107953
ENSG0000011	SF3B6	protein_codir	-0.10936746	0.28501883	0.43463155
ENSG0000011	PCDHB3	protein_codir	-0.10948048	0.75380971	0.84346475
ENSG000002(	FAM205A	protein_codir	-0.10948738	0.95566497	NA
ENSG0000027	RP3-412A9.16	lncRNA	-0.10971255	0.75234421	0.84235858
ENSG0000014	LSM14B	protein_codir	-0.10973064	0.26431859	0.41180408
ENSG0000018	GTF2F2	protein_codir	-0.10974131	0.34714756	0.50027213
ENSG0000018	PSMG4	protein_codir	-0.10975936	0.46297665	0.61298257
ENSG0000006	TNFRSF1A	protein_codir	-0.10999379	0.56598143	0.70015708
ENSG0000025	ZNF260	protein_codir	-0.11005018	0.47617525	0.62438096
ENSG0000027	LL21NC02-1C1	lncRNA	-0.11005582	0.90543799	0.94374117
ENSG000001(	CCP110	protein_codir	-0.1100938	0.37342227	0.52700032
ENSG0000027	VPS11-DT	lncRNA	-0.11010398	0.88754078	0.9333997
ENSG0000018	GPR88	protein_codir	-0.11010774	0.83838029	0.90316843
ENSG0000024	AC108676.1	lncRNA	-0.11015326	0.85816568	0.91543509
ENSG0000014	SYBU	protein_codir	-0.11016672	0.61544029	0.73938498
ENSG0000015	ANO4	protein_codir	-0.1102074	0.85407387	0.91288916
ENSG0000012	USP22	protein_codir	-0.11024125	0.34508634	0.49797726
ENSG0000019	SUPT5H	protein_codir	-0.11059414	0.3410872	0.49422036
ENSG0000014	PRKACB	protein_codir	-0.11061196	0.56551087	0.69978285
ENSG0000008	MGST2	protein_codir	-0.11066097	0.38864257	0.54156122
ENSG0000011	MLEC	protein_codir	-0.11066631	0.48206131	0.62952877
ENSG0000016	ASB11	protein_codir	-0.110727	0.92134588	0.95359264
ENSG0000023	RP11-89N17.4	lncRNA	-0.11081832	0.94829676	NA
ENSG0000019	CD47	protein_codir	-0.11086511	0.36814143	0.52165331
ENSG0000013	TMEM241	protein_codir	-0.11088421	0.44241329	0.59346551
ENSG000001(	MAGT1	protein_codir	-0.1110413	0.59478196	0.72331268
ENSG0000016	FAM204A	protein_codir	-0.11126613	0.47214472	0.62083491
ENSG0000013	INPP5K	protein_codir	-0.1113346	0.27648864	0.42472838
ENSG000001(	EZH1	protein_codir	-0.11138401	0.60355643	0.73012564
ENSG0000017	NR2C2	protein_codir	-0.11152999	0.50433742	0.64904481
ENSG0000021	ALOX12-AS1	lncRNA	-0.11154968	0.69911545	0.80346257
ENSG0000028	RP11-20J15.6	lncRNA	-0.11161151	0.86089472	0.9170393
ENSG0000026	DM1-AS	lncRNA	-0.11171503	0.83569215	0.90127871
ENSG0000015	ZDHHC5	protein_codir	-0.1118859	0.34604435	0.49907289
ENSG0000017	PIFO	protein_codir	-0.11189517	0.84420778	0.90688884
ENSG0000017	NFRKB	protein_codir	-0.11199225	0.3503721	0.50394612
ENSG0000013	CAB39	protein_codir	-0.1120572	0.50535934	0.64993597
ENSG0000002	SLC7A9	protein_codir	-0.11209302	0.85903767	0.91597964
ENSG0000013	SNRPF	protein_codir	-0.11212664	0.54504256	0.6830872
ENSG0000005	AKR7A2	protein_codir	-0.11246458	0.33906991	0.49196842
ENSG0000011	HSPE1	protein_codir	-0.11256726	0.61015981	0.73560078
ENSG0000013	GIPC2	protein_codir	-0.11263564	0.66349362	0.77730153
ENSG0000015	CIART	protein_codir	-0.11271744	0.7902491	0.86931178

ENSG0000018	GOLGA8R	protein_codir	-0.11277619	0.55995605	0.69528235
ENSG0000016	TNXB	protein_codir	-0.11278812	0.8087603	0.88173836
ENSG0000016	SPATC1L	protein_codir	-0.11291503	0.73604552	0.83086387
ENSG0000025	RP11-561B11.	protein_codir	-0.11297401	0.66552928	0.77905992
ENSG0000017	APLN	protein_codir	-0.11316729	0.81937636	0.8891708
ENSG0000026	ZNF350-AS1	lncRNA	-0.11324435	0.88275295	0.93063233
ENSG0000012	FAM126A	protein_codir	-0.11329168	0.49962285	0.64501081
ENSG0000016	DOLPP1	protein_codir	-0.11330404	0.42459702	0.57656685
ENSG0000011	DLST	protein_codir	-0.11333679	0.12897932	0.24376686
ENSG0000016	THAP11	protein_codir	-0.1135162	0.33156284	0.48428627
ENSG0000018	FANCF	protein_codir	-0.11354235	0.74096448	0.83457897
ENSG0000016	POLR2H	protein_codir	-0.11363785	0.50358293	0.64831549
ENSG0000001	SLC30A9	protein_codir	-0.11370716	0.45339351	0.60390177
ENSG0000023	EGOT	lncRNA	-0.1137579	0.88797453	0.93348541
ENSG0000010	SYNGR1	protein_codir	-0.11376356	0.70692207	0.80956786
ENSG0000018	OR2A4	protein_codir	-0.11380879	0.92239081	0.95428079
ENSG0000019	HHLA3	lncRNA	-0.11381292	0.58234324	0.71363306
ENSG0000023	AP000695.4	lncRNA	-0.11381468	0.82690002	0.89526003
ENSG0000012	HNRNPR	protein_codir	-0.11391691	0.44412795	0.59518748
ENSG0000012	WASHC3	protein_codir	-0.11392923	0.35598213	0.5095738
ENSG0000009	MTAP	protein_codir	-0.1139502	0.38419931	0.53748594
ENSG0000013	PEX11B	protein_codir	-0.11403465	0.51597076	0.6591876
ENSG0000026	C15orf65	protein_codir	-0.11408749	0.71871886	0.81839607
ENSG0000008	NUFIP1	protein_codir	-0.11410077	0.50928976	0.65353016
ENSG0000022	RP4-565E6.1	lncRNA	-0.11410719	0.7779057	0.86088527
ENSG0000009	AAAS	protein_codir	-0.11414105	0.52660256	0.6684174
ENSG0000010	EHD4	protein_codir	-0.1143724	0.6379217	0.75730238
ENSG0000011	FIG4	protein_codir	-0.11449518	0.41985563	0.5723232
ENSG0000015	ETS2	protein_codir	-0.11453907	0.69105154	0.79721174
ENSG0000014	HDAC8	protein_codir	-0.1145893	0.42791161	0.57972819
ENSG0000006	ATP1B3	protein_codir	-0.11463717	0.7131135	0.81406054
ENSG0000010	MRPL4	protein_codir	-0.11492879	0.40988196	0.56285277
ENSG0000010	CRNKL1	protein_codir	-0.11493242	0.4609367	0.61125249
ENSG0000022	CCNT2-AS1	lncRNA	-0.11494733	0.53876864	0.67814747
ENSG0000023	TMSB15B-AS1	lncRNA	-0.11496947	0.75825377	0.8469334
ENSG0000025	RP11-463D19	protein_codir	-0.11498545	0.82672842	0.89521461
ENSG0000010	AHCY	protein_codir	-0.11510683	0.33987889	0.49280565
ENSG0000019	C1D	protein_codir	-0.11525546	0.28173776	0.43116118
ENSG0000007	SNRPA	protein_codir	-0.11530537	0.36912648	0.5227273
ENSG0000017	CTSF	protein_codir	-0.11533053	0.75435409	0.84387444
ENSG0000027	LYRM4-AS1	lncRNA	-0.11533165	0.64707156	0.76415754
ENSG0000014	GNA12	protein_codir	-0.11559231	0.40622574	0.55919841
ENSG0000011	PCDHB2	protein_codir	-0.11562265	0.70157295	0.805514
ENSG0000017	PFAS	protein_codir	-0.1156511	0.56202762	0.6967672
ENSG0000016	CCDC38	protein_codir	-0.11566513	0.89209836	0.93569881
ENSG0000028	RP11-346C20.	lncRNA	-0.11579194	0.90788023	0.94514537
ENSG0000012	TST	protein_codir	-0.11587525	0.49551164	0.64145255

ENSG0000011	LPCAT3	protein_codir	-0.11589705	0.4563976	0.60697893
ENSG0000010	DNAJC17	protein_codir	-0.11595482	0.42437935	0.57632795
ENSG0000022	AC009506.1	lncRNA	-0.11597215	0.61171822	0.73674042
ENSG0000013	ANXA1	protein_codir	-0.11600449	0.67998043	0.78958461
ENSG0000028	RP11-317B17.	lncRNA	-0.11602014	0.69698684	0.80171483
ENSG0000018	CMTM4	protein_codir	-0.11624569	0.62734113	0.7487173
ENSG0000016	PDSS2	protein_codir	-0.11630196	0.28490801	0.43453841
ENSG0000019	MT-ND4	protein_codir	-0.11643507	0.70101339	0.80517278
ENSG0000009	PLEKHG2	protein_codir	-0.11644731	0.53568838	0.67592176
ENSG0000016	RPRD2	protein_codir	-0.11648828	0.40915342	0.56214582
ENSG0000012	PARP2	protein_codir	-0.11653752	0.32095924	0.47309714
ENSG0000019	TMA16	protein_codir	-0.11658237	0.47633225	0.62449787
ENSG0000010	MOSPD1	protein_codir	-0.11658301	0.3577197	0.51121451
ENSG0000013	DBR1	protein_codir	-0.11662086	0.59070179	0.72022175
ENSG0000024	RAB30-DT	lncRNA	-0.11670041	0.67971805	0.7894701
ENSG0000020	DHX16	protein_codir	-0.11670684	0.25839589	0.4054593
ENSG0000017	EID2	protein_codir	-0.11673824	0.23452297	0.37787449
ENSG0000010	DIDO1	protein_codir	-0.11679496	0.37083695	0.52427512
ENSG0000013	SLC7A1	protein_codir	-0.1169544	0.7051177	0.80817291
ENSG0000017	CTDSP2	protein_codir	-0.11696433	0.47539662	0.62367402
ENSG0000014	TXNL4A	protein_codir	-0.11702656	0.41681263	0.56949616
ENSG0000003	RANBP3	protein_codir	-0.11707872	0.28416295	0.43374305
ENSG0000013	THEM6	protein_codir	-0.1170882	0.45058343	0.60130039
ENSG0000014	TBCK	protein_codir	-0.11713698	0.45406799	0.60446105
ENSG0000012	MRM2	protein_codir	-0.1172059	0.44139528	0.59237532
ENSG0000023	AC002310.12	lncRNA	-0.117258	0.73917302	0.83324012
ENSG0000025	CTC-260F20.3	protein_codir	-0.11729842	0.73966576	0.83362175
ENSG0000019	FICD	protein_codir	-0.11737491	0.54746313	0.68519719
ENSG0000006	VPS35	protein_codir	-0.11740788	0.35116347	0.50472257
ENSG0000014	SLC12A6	protein_codir	-0.11743179	0.63416294	0.75431215
ENSG0000013	SCYL2	protein_codir	-0.11748112	0.47780473	0.62577084
ENSG0000010	ARL2BP	protein_codir	-0.11755801	0.31143774	0.46322328
ENSG0000020	C12orf73	protein_codir	-0.11769981	0.54305355	0.68162414
ENSG0000014	CCDC40	protein_codir	-0.11776258	0.69168074	0.79757116
ENSG0000027	MIOS-DT	lncRNA	-0.11813824	0.77339303	0.85744987
ENSG0000017	GALNT11	protein_codir	-0.11838691	0.41514858	0.56820633
ENSG0000015	BEND6	protein_codir	-0.11842815	0.65415718	0.76966225
ENSG0000013	ENOSF1	protein_codir	-0.11852179	0.67535888	0.78620131
ENSG0000017	MAGEF1	protein_codir	-0.11853782	0.508652	0.65301517
ENSG0000022	EIPR1-IT1	lncRNA	-0.11877203	0.93380229	0.96106706
ENSG0000015	CENPJ	protein_codir	-0.11878593	0.42029967	0.57258936
ENSG0000017	MROH2B	protein_codir	-0.11885822	0.92850703	0.95759547
ENSG0000010	VTI1B	protein_codir	-0.11887814	0.33260941	0.48536252
ENSG0000013	CEP162	protein_codir	-0.11894145	0.43091903	0.58234581
ENSG0000016	NDRG2	protein_codir	-0.1189887	0.69915696	0.80347488
ENSG0000016	KIAA1958	protein_codir	-0.11902479	0.59257218	0.72138241
ENSG0000013	YIPF3	protein_codir	-0.11910182	0.18181634	0.31386046

ENSG0000010	SH3D19	protein_codir	-0.11911732	0.70338266	0.80685394
ENSG0000008	TMED2	protein_codir	-0.11916507	0.4915734	0.63826961
ENSG0000013	PPA2	protein_codir	-0.11920365	0.34839319	0.50170073
ENSG0000020	SYCE1L	protein_codir	-0.11925615	0.74863175	0.83965258
ENSG0000010	SNX16	protein_codir	-0.1194129	0.56191959	0.69675507
ENSG0000028	TBCEL-TECTA	protein_codir	-0.11951324	0.81481819	0.8861026
ENSG0000007	EPHA8	protein_codir	-0.11953266	0.88533841	0.93200621
ENSG0000005	POLR3E	protein_codir	-0.11958103	0.23396549	0.37724243
ENSG0000010	PSMD11	protein_codir	-0.11959362	0.28377527	0.43340182
ENSG0000026	SETBP1-DT	lncRNA	-0.11971552	0.6657902	0.77926627
ENSG0000013	HINT2	protein_codir	-0.11979187	0.3641757	0.51818726
ENSG0000018	DCAF4L1	protein_codir	-0.11985536	0.81776109	0.88804597
ENSG0000018	CC2D2B	protein_codir	-0.1199298	NA	NA
ENSG0000014	CBR4	protein_codir	-0.11995798	0.56661642	0.70059744
ENSG0000012	PRXL2A	protein_codir	-0.11998806	0.60585212	0.73220144
ENSG0000018	HARBI1	protein_codir	-0.12004626	0.47286667	0.62131493
ENSG0000006	PKN2	protein_codir	-0.12013295	0.36215486	0.51602957
ENSG0000010	PHF14	protein_codir	-0.12018464	0.11659497	0.22616427
ENSG0000010	NFAT5	protein_codir	-0.12030045	0.55764313	0.69344424
ENSG0000016	ATP5F1C	protein_codir	-0.12035408	0.3245634	0.47695883
ENSG0000013	HSD17B4	protein_codir	-0.12043965	0.35296473	0.5065672
ENSG0000010	SCN1B	protein_codir	-0.12046154	0.67051619	0.78257584
ENSG0000009	CHRD	protein_codir	-0.12050243	0.64185376	0.75981488
ENSG0000027	RP11-54O7.17	lncRNA	-0.12051135	0.81167544	0.88385499
ENSG0000017	ZNF713	protein_codir	-0.12057031	0.51314481	0.65679872
ENSG0000022	AC007403.3	lncRNA	-0.12067732	0.97049755	NA
ENSG0000028	CPS1-IT1	lncRNA	-0.12067732	0.97049755	NA
ENSG0000022	RP11-556N4.1	lncRNA	-0.12067732	0.97049755	NA
ENSG0000025	RP11-76G10.1	lncRNA	-0.12067732	0.97049755	NA
ENSG0000025	LINC01262	lncRNA	-0.12067732	0.97049755	NA
ENSG0000028	CTD-2537M22	lncRNA	-0.12067732	0.97049755	NA
ENSG0000023	AC004543.2	lncRNA	-0.12067732	0.97049755	NA
ENSG0000021	TMEM213	protein_codir	-0.12067732	0.97049755	NA
ENSG0000028	RP11-107M13	lncRNA	-0.12067732	0.97049755	NA
ENSG0000025	RP11-557C18.	lncRNA	-0.12067732	0.97049755	NA
ENSG0000028	RP11-154I21.1	lncRNA	-0.12067732	0.97049755	NA
ENSG0000019	OR11G2	protein_codir	-0.12067732	0.97049755	NA
ENSG0000028	RP3-514A23.4	lncRNA	-0.12067732	0.97049755	NA
ENSG0000015	PDZD9	protein_codir	-0.12067732	0.97049755	NA
ENSG0000027	RP1-59D14.10	lncRNA	-0.12067732	0.97049755	NA
ENSG0000026	CTD-2561B21	lncRNA	-0.12067732	0.97049755	NA
ENSG0000026	LINC01444	lncRNA	-0.12067732	0.97049755	NA
ENSG0000023	AC002511.2	lncRNA	-0.12067732	0.97049755	NA
ENSG0000021	IGSF23	protein_codir	-0.12067732	0.97049755	NA
ENSG0000022	RP5-1031J8.1	lncRNA	-0.12067732	0.97049755	NA
ENSG0000022	RP11-46E17.6	lncRNA	-0.12067732	0.97049755	NA
ENSG0000028	INE2	lncRNA	-0.12067732	0.97049755	NA

ENSG0000023	LINC01777	lncRNA	-0.12067732	0.97049755	NA
ENSG0000028	RP11-179G8.2	lncRNA	-0.12067732	0.97049755	NA
ENSG0000028	RP11-565D17	lncRNA	-0.12067732	0.97049755	NA
ENSG0000027	RP4-547N15.3	lncRNA	-0.12067732	0.97049755	NA
ENSG0000028	RP11-275A6.1	lncRNA	-0.12067732	0.97049755	NA
ENSG0000028	RP11-427B20.	lncRNA	-0.12067732	0.97049755	NA
ENSG0000023	RP11-148B18.	lncRNA	-0.12067732	0.97049755	NA
ENSG0000027	RP11-433J22.	lncRNA	-0.12067732	0.97049755	NA
ENSG0000023	LINC01731	lncRNA	-0.12067732	0.97049755	NA
ENSG0000026	RP11-98D18.1	lncRNA	-0.12067732	0.97049755	NA
ENSG0000023	RP11-9L18.3	lncRNA	-0.12067732	0.97049755	NA
ENSG0000020	MAPRE3-AS1	lncRNA	-0.12067732	0.97049755	NA
ENSG0000028	RP11-1173K8.	lncRNA	-0.12067732	0.97049755	NA
ENSG0000027	RP11-761B3.1	protein_codir	-0.12067732	0.97049755	NA
ENSG0000028	RP11-260E12.	lncRNA	-0.12067732	0.97049755	NA
ENSG0000028	RP11-570C16.	lncRNA	-0.12067732	0.97049755	NA
ENSG0000027	RP11-796E10.	lncRNA	-0.12067732	0.97049755	NA
ENSG0000027	RP11-337N6.1	lncRNA	-0.12067732	0.97049755	NA
ENSG0000028	RP11-387P12.	lncRNA	-0.12067732	0.97049755	NA
ENSG0000017	AGXT	protein_codir	-0.12067732	0.97049755	NA
ENSG0000028	FAM240A	protein_codir	-0.12067732	0.97049755	NA
ENSG0000023	RUVBL1-AS1	lncRNA	-0.12067732	0.97049755	NA
ENSG0000027	RP11-368I23.3	lncRNA	-0.12067732	0.97049755	NA
ENSG0000023	TMEM212-AS	lncRNA	-0.12067732	0.97049755	NA
ENSG0000024	RP11-225N10	lncRNA	-0.12067732	0.97049755	NA
ENSG0000016	CCKAR	protein_codir	-0.12067732	0.97049755	NA
ENSG0000027	UGT2A2	protein_codir	-0.12067732	0.97049755	NA
ENSG0000017	GYPA	protein_codir	-0.12067732	0.97049755	NA
ENSG0000024	RP11-310P5.1	lncRNA	-0.12067732	0.97049755	NA
ENSG0000024	RP11-5N11.7	lncRNA	-0.12067732	0.97049755	NA
ENSG0000024	CTD-2066L21.	lncRNA	-0.12067732	0.97049755	NA
ENSG0000024	LINC02224	lncRNA	-0.12067732	0.97049755	NA
ENSG0000024	LINC02057	lncRNA	-0.12067732	0.97049755	NA
ENSG0000025	RP11-428C6.2	lncRNA	-0.12067732	0.97049755	NA
ENSG0000028	CTD-2503O16	lncRNA	-0.12067732	0.97049755	NA
ENSG0000025	SLC25A48-AS1	lncRNA	-0.12067732	0.97049755	NA
ENSG0000025	CTD-2532K18	lncRNA	-0.12067732	0.97049755	NA
ENSG0000028	CTD-2537M22	protein_codir	-0.12067732	0.97049755	NA
ENSG0000028	RP11-299J5.2	lncRNA	-0.12067732	0.97049755	NA
ENSG0000028	LINC00581	lncRNA	-0.12067732	0.97049755	NA
ENSG0000022	LINC01623	lncRNA	-0.12067732	0.97049755	NA
ENSG0000022	HCG14	lncRNA	-0.12067732	0.97049755	NA
ENSG0000027	XXbac-BPG25.	lncRNA	-0.12067732	0.97049755	NA
ENSG0000020	TRIM40	protein_codir	-0.12067732	0.97049755	NA
ENSG0000020	PSORS1C2	protein_codir	-0.12067732	0.97049755	NA
ENSG0000028	RP11-642N5.2	lncRNA	-0.12067732	0.97049755	NA
ENSG0000028	RP11-34M7.1	lncRNA	-0.12067732	0.97049755	NA

ENSG0000027	RP5-855F16.1	lncRNA	-0.12067732	0.97049755	NA
ENSG0000028	CTA-271G13.2	lncRNA	-0.12067732	0.97049755	NA
ENSG0000021	OR2F1	protein_codir	-0.12067732	0.97049755	NA
ENSG0000020	FABP9	protein_codir	-0.12067732	0.97049755	NA
ENSG0000027	RP11-642C5.1	lncRNA	-0.12067732	0.97049755	NA
ENSG0000025	LINC01030	lncRNA	-0.12067732	0.97049755	NA
ENSG0000025	RP11-10N23.4	lncRNA	-0.12067732	0.97049755	NA
ENSG0000027	KB-1043D8.8	lncRNA	-0.12067732	0.97049755	NA
ENSG0000025	KB-1410C5.3	lncRNA	-0.12067732	0.97049755	NA
ENSG0000026	RP11-354P17.	lncRNA	-0.12067732	0.97049755	NA
ENSG0000027	RP11-392E22.	lncRNA	-0.12067732	0.97049755	NA
ENSG0000023	RP11-575L7.4	lncRNA	-0.12067732	0.97049755	NA
ENSG0000022	TMEM246-AS	lncRNA	-0.12067732	0.97049755	NA
ENSG0000023	RP11-410K21.	lncRNA	-0.12067732	0.97049755	NA
ENSG0000023	RP1-251M9.2	lncRNA	-0.12067732	0.97049755	NA
ENSG0000023	RP3-323N1.2	lncRNA	-0.12067732	0.97049755	NA
ENSG0000023	RP11-402L1.4	lncRNA	-0.12067732	0.97049755	NA
ENSG0000023	RP11-20J15.2	lncRNA	-0.12067732	0.97049755	NA
ENSG0000022	RP11-574K11.	lncRNA	-0.12067732	0.97049755	NA
ENSG0000027	RP11-399K21.	lncRNA	-0.12067732	0.97049755	NA
ENSG0000028	RP11-7F3.1	lncRNA	-0.12067732	0.97049755	NA
ENSG0000022	LBX1-AS1	lncRNA	-0.12067732	0.97049755	NA
ENSG0000028	RP11-481L19.	protein_codir	-0.12067732	0.97049755	NA
ENSG0000023	RP11-140A10	lncRNA	-0.12067732	0.97049755	NA
ENSG0000017	MRGPRX3	protein_codir	-0.12067732	0.97049755	NA
ENSG0000025	RP11-428C19.	lncRNA	-0.12067732	0.97049755	NA
ENSG0000023	BTBD18	protein_codir	-0.12067732	0.97049755	NA
ENSG0000025	CTD-2531D15	lncRNA	-0.12067732	0.97049755	NA
ENSG0000026	CTD-2007L18.	lncRNA	-0.12067732	0.97049755	NA
ENSG0000028	RP11-817J15.	lncRNA	-0.12067732	0.97049755	NA
ENSG0000025	LINC02764	lncRNA	-0.12067732	0.97049755	NA
ENSG0000025	OR8G5	protein_codir	-0.12067732	0.97049755	NA
ENSG0000025	RP5-1154L15.	lncRNA	-0.12067732	0.97049755	NA
ENSG0000025	RP11-153K16.	lncRNA	-0.12067732	0.97049755	NA
ENSG0000024	RP11-709A23	lncRNA	-0.12067732	0.97049755	NA
ENSG0000025	RP11-776A13	lncRNA	-0.12067732	0.97049755	NA
ENSG0000025	LINC02874	lncRNA	-0.12067732	0.97049755	NA
ENSG0000017	GLIPR1L1	protein_codir	-0.12067732	0.97049755	NA
ENSG0000023	TSPAN19	protein_codir	-0.12067732	0.97049755	NA
ENSG0000025	RP11-202G11	lncRNA	-0.12067732	0.97049755	NA
ENSG0000028	RP11-463C23.	lncRNA	-0.12067732	0.97049755	NA
ENSG0000025	RP11-417L19.	lncRNA	-0.12067732	0.97049755	NA
ENSG0000026	RP11-629E24.	lncRNA	-0.12067732	0.97049755	NA
ENSG0000026	RP11-307O13	lncRNA	-0.12067732	0.97049755	NA
ENSG0000028	RP11-165D7.6	lncRNA	-0.12067732	0.97049755	NA
ENSG0000027	RP11-195L15.	lncRNA	-0.12067732	0.97049755	NA
ENSG0000018	FSCB	protein_codir	-0.12067732	0.97049755	NA

ENSG0000027	LINC00216	lncRNA	-0.12067732	0.97049755	NA
ENSG0000028	RP11-488C13.	lncRNA	-0.12067732	0.97049755	NA
ENSG0000019	LINC02291	lncRNA	-0.12067732	0.97049755	NA
ENSG0000023	LINC02250	lncRNA	-0.12067732	0.97049755	NA
ENSG0000025	RP11-265N7.2	lncRNA	-0.12067732	0.97049755	NA
ENSG0000025	RP11-37C7.1	lncRNA	-0.12067732	0.97049755	NA
ENSG0000026	RP11-48G14.1	lncRNA	-0.12067732	0.97049755	NA
ENSG0000025	RP11-300G22	lncRNA	-0.12067732	0.97049755	NA
ENSG0000017	RNF151	protein_codir	-0.12067732	0.97049755	NA
ENSG0000026	AC106782.20	lncRNA	-0.12067732	0.97049755	NA
ENSG0000026	RP11-510J16.	lncRNA	-0.12067732	0.97049755	NA
ENSG0000016	KCNG4	protein_codir	-0.12067732	0.97049755	NA
ENSG0000026	RP11-1113L8.	lncRNA	-0.12067732	0.97049755	NA
ENSG0000026	RP1-41C23.1	lncRNA	-0.12067732	0.97049755	NA
ENSG0000026	RP11-212E8.1	lncRNA	-0.12067732	0.97049755	NA
ENSG0000007	FNDC8	protein_codir	-0.12067732	0.97049755	NA
ENSG0000016	SLC35G3	protein_codir	-0.12067732	0.97049755	NA
ENSG0000017	NEUROD2	protein_codir	-0.12067732	0.97049755	NA
ENSG0000026	RP11-6N17.6	lncRNA	-0.12067732	0.97049755	NA
ENSG0000028	RP11-178C3.9	lncRNA	-0.12067732	0.97049755	NA
ENSG0000026	LINC01999	lncRNA	-0.12067732	0.97049755	NA
ENSG0000017	EFCAB3	protein_codir	-0.12067732	0.97049755	NA
ENSG0000026	GACAT2	lncRNA	-0.12067732	0.97049755	NA
ENSG0000026	RP11-419J16.	lncRNA	-0.12067732	0.97049755	NA
ENSG0000028	RP11-116O18	lncRNA	-0.12067732	0.97049755	NA
ENSG0000022	LINC01630	lncRNA	-0.12067732	0.97049755	NA
ENSG0000027	RP11-734B5.2	lncRNA	-0.12067732	0.97049755	NA
ENSG0000026	CTB-66B24.1	lncRNA	-0.12067732	0.97049755	NA
ENSG0000026	CTC-499B15.6	lncRNA	-0.12067732	0.97049755	NA
ENSG0000026	CTD-2293H3.2	lncRNA	-0.12067732	0.97049755	NA
ENSG0000026	ZNF649-AS1	lncRNA	-0.12067732	0.97049755	NA
ENSG0000026	ZNF628-DT	lncRNA	-0.12067732	0.97049755	NA
ENSG0000022	LINC01713	lncRNA	-0.12067732	0.97049755	NA
ENSG0000023	RP5-905G11.3	lncRNA	-0.12067732	0.97049755	NA
ENSG0000028	RP5-970A17.1	lncRNA	-0.12067732	0.97049755	NA
ENSG0000028	RP5-998C11.1	lncRNA	-0.12067732	0.97049755	NA
ENSG0000023	RP5-897D18.1	lncRNA	-0.12067732	0.97049755	NA
ENSG0000023	RP1-293L6.1	lncRNA	-0.12067732	0.97049755	NA
ENSG0000022	MGAT3-AS1	lncRNA	-0.12067732	0.97049755	NA
ENSG0000028	RP6-113J7.2	lncRNA	-0.12067732	0.97049755	NA
ENSG0000028	RP13-111A12	lncRNA	-0.12067732	0.97049755	NA
ENSG0000027	ENSG0000027	protein_codir	-0.12067732	0.97049755	NA
ENSG0000016	MRPL16	protein_codir	-0.12076481	0.2746498	0.42270297
ENSG0000005	PITHD1	protein_codir	-0.12077975	0.20641367	0.34456338
ENSG0000015	OTULIN	protein_codir	-0.1207857	0.52256646	0.66494451
ENSG0000010	PIK3R2	protein_codir	-0.12094814	0.37864207	0.53216436
ENSG0000023	AC123023.1	lncRNA	-0.12113515	0.91422836	0.9491105

ENSG0000028	RP11-482M8.	lncRNA	-0.12121559	0.89310038	0.93629964
ENSG0000027	ZKSCAN2-DT	lncRNA	-0.12123912	0.80779593	0.88100614
ENSG0000007	NUCB2	protein_codir	-0.12130447	0.44002972	0.5909857
ENSG0000025	RP11-809N8.2	lncRNA	-0.12132342	0.94970984	NA
ENSG0000016	PRICKLE2	protein_codir	-0.12138035	0.65856664	0.77320342
ENSG0000007	PCM1	protein_codir	-0.12141223	0.47662541	0.6247636
ENSG0000014	SNRPG	protein_codir	-0.12143397	0.40681573	0.55986671
ENSG0000016	LIMS1	protein_codir	-0.12146159	0.48074252	0.62840597
ENSG0000018	ZNF443	protein_codir	-0.12160131	0.65996903	0.7742904
ENSG0000010	PPP1R37	protein_codir	-0.12167147	0.36670035	0.52059911
ENSG0000013	C9orf78	protein_codir	-0.12168875	0.37347595	0.52704457
ENSG0000023	RP11-767N6.7	lncRNA	-0.12173639	0.68457635	0.79271986
ENSG0000010	HPS1	protein_codir	-0.12175972	0.26911114	0.41688725
ENSG0000008	DDHD2	protein_codir	-0.12179428	0.51892206	0.66191762
ENSG0000010	YJU2	protein_codir	-0.12183035	0.19410536	0.32959487
ENSG0000019	NF1	protein_codir	-0.12192548	0.497255	0.642956
ENSG0000004	LSG1	protein_codir	-0.12203709	0.24892303	0.39458529
ENSG0000015	FER	protein_codir	-0.12205211	0.6250715	0.74721812
ENSG0000025	MTRNR2L1	protein_codir	-0.12209021	0.93362249	0.9609336
ENSG0000017	PPP2R2D	protein_codir	-0.12212108	0.26825598	0.41613199
ENSG0000018	GTF2H2C	protein_codir	-0.12231366	0.33299158	0.48572969
ENSG0000026	CTD-2265O21	lncRNA	-0.12250808	0.90563964	0.9438445
ENSG0000018	TOB2	protein_codir	-0.12254235	0.61248949	0.73736411
ENSG0000021	VDAC1	protein_codir	-0.12255593	0.35772059	0.51121451
ENSG0000015	RAB28	protein_codir	-0.12260091	0.15231756	0.27541807
ENSG0000020	AC022007.5	lncRNA	-0.12278497	0.68424364	0.79250591
ENSG0000027	NAMA	lncRNA	-0.12279444	0.92245514	0.95428941
ENSG0000025	CHMP4A	protein_codir	-0.1228454	0.35961127	0.51340142
ENSG0000027	TAF15	protein_codir	-0.12287754	0.18951354	0.32378845
ENSG0000023	LINC00460	lncRNA	-0.12294309	0.89954021	0.94000608
ENSG0000014	ABHD10	protein_codir	-0.1233319	0.47166314	0.62043797
ENSG0000008	SEPHS1	protein_codir	-0.12343039	0.22424232	0.36592303
ENSG0000019	STKLD1	protein_codir	-0.12345414	0.74206499	0.83541704
ENSG0000014	FBXO28	protein_codir	-0.12359786	0.41940457	0.57196239
ENSG0000017	DEAF1	protein_codir	-0.12363538	0.18936101	0.32354785
ENSG0000011	REEP6	protein_codir	-0.12365076	0.66890251	0.78132186
ENSG0000012	FASTKD3	protein_codir	-0.12365848	0.56039056	0.69554609
ENSG0000010	ZNHIT1	protein_codir	-0.12367283	0.33540052	0.48824563
ENSG0000016	KLHDC2	protein_codir	-0.1237594	0.42975013	0.58124962
ENSG0000010	ZNF574	protein_codir	-0.1238404	0.40213152	0.55509384
ENSG0000023	POU6F2-AS2	lncRNA	-0.12392993	0.89271178	0.93605783
ENSG0000017	HNRNPA3	protein_codir	-0.12394271	0.30883485	0.4604407
ENSG0000017	TPT1-AS1	lncRNA	-0.12400523	0.65237335	0.76828346
ENSG0000019	ZNF699	protein_codir	-0.12407194	0.5581813	0.6938017
ENSG0000014	NIPSNAP2	protein_codir	-0.12418604	0.54510952	0.6830872
ENSG0000007	NUAK1	protein_codir	-0.12426484	0.72941104	0.82603402
ENSG0000017	SMN1	protein_codir	-0.12426814	0.49521691	0.64119121

ENSG0000009	RNF215	protein_codir	-0.12430566	0.24579385	0.39101637
ENSG0000028	ERICD	lncRNA	-0.12444613	0.68849314	0.79558951
ENSG0000016	BSDC1	protein_codir	-0.12445811	0.25382016	0.40022885
ENSG0000011	TRAPPC13	protein_codir	-0.12449281	0.27396976	0.42201983
ENSG0000005	JKAMP	protein_codir	-0.12450161	0.30259541	0.45399996
ENSG0000025	NANOGP8	protein_codir	-0.12467463	0.87110725	0.92343052
ENSG0000014	CYP1A2	protein_codir	-0.12478882	0.94115739	NA
ENSG0000017	XXYLT1	protein_codir	-0.12491031	0.41817034	0.57087113
ENSG0000012	TMEM39B	protein_codir	-0.12499682	0.22724041	0.36941613
ENSG0000009	CBX5	protein_codir	-0.12499819	0.45095482	0.60167973
ENSG0000022	NRSN2-AS1	lncRNA	-0.12503667	0.48156724	0.62915554
ENSG0000019	MPHOSPH8	protein_codir	-0.12504777	0.45185803	0.60244829
ENSG0000026	RP11-22P6.2	lncRNA	-0.12505051	0.90522004	0.94371842
ENSG0000014	TRPT1	protein_codir	-0.12509446	0.5832004	0.71432351
ENSG0000018	ZSCAN30	protein_codir	-0.1252401	0.41604811	0.56882775
ENSG0000014	ARMC5	protein_codir	-0.1253528	0.44611932	0.59707398
ENSG0000016	CGGBP1	protein_codir	-0.12549417	0.30969771	0.46139764
ENSG0000017	EIF1AD	protein_codir	-0.12555366	0.20159945	0.33861752
ENSG0000026	IKBKG	protein_codir	-0.12591478	0.272261	0.42018778
ENSG0000027	RP4-665N4.8	lncRNA	-0.12595994	0.95632761	NA
ENSG0000013	ARF3	protein_codir	-0.12598131	0.26229222	0.40975741
ENSG0000027	RP11-506K6.4	lncRNA	-0.12600504	0.87085954	0.92331633
ENSG0000016	NUDT2	protein_codir	-0.12607114	0.36800462	0.52153972
ENSG0000015	RER1	protein_codir	-0.12607802	0.06846466	0.15248722
ENSG0000016	TSR1	protein_codir	-0.1261057	0.46180404	0.61192802
ENSG0000015	IFIT5	protein_codir	-0.12618632	0.55460528	0.69078571
ENSG0000028	RP11-575F12.	lncRNA	-0.12622698	0.89326729	0.93642702
ENSG0000018	NOXA1	protein_codir	-0.12635344	0.68704218	0.79474411
ENSG0000014	SERPING1	protein_codir	-0.12652635	0.64034524	0.75885311
ENSG0000014	SERBP1	protein_codir	-0.12653476	0.39638722	0.54925485
ENSG0000016	UPF3A	protein_codir	-0.12673035	0.56246618	0.69721371
ENSG0000012	ENKD1	protein_codir	-0.12708228	0.52313359	0.66535963
ENSG0000006	ZC3H15	protein_codir	-0.12712937	0.30979858	0.46152302
ENSG0000028	RP11-281A20	protein_codir	-0.12727945	0.84640537	0.90847225
ENSG0000006	CS	protein_codir	-0.1272846	0.27559875	0.42370801
ENSG0000024	LINC01184	lncRNA	-0.12730646	0.38099679	0.53437137
ENSG0000014	DCAF1	protein_codir	-0.12735821	0.26807701	0.41594782
ENSG0000009	CD200	protein_codir	-0.12744079	0.56055923	0.69560453
ENSG0000028	RP11-416N2.5	lncRNA	-0.12745964	0.83379023	0.8998627
ENSG0000010	PSMD3	protein_codir	-0.12746199	0.31176611	0.46348563
ENSG0000020	GP1BB	protein_codir	-0.1275242	0.74588379	0.83765594
ENSG0000027	RP3-465N24.6	lncRNA	-0.12762393	0.85586302	0.91382931
ENSG0000017	TMEM11	protein_codir	-0.12763587	0.15160889	0.27438814
ENSG0000012	KLK10	protein_codir	-0.12766385	0.89112087	0.93510148
ENSG0000016	AZI2	protein_codir	-0.12768543	0.34641266	0.49949225
ENSG0000012	VAMP7	protein_codir	-0.12772725	0.39722107	0.55013017
ENSG0000017	BCLAF3	protein_codir	-0.12779917	0.52013976	0.66283382

ENSG0000012	SEC22A	protein_codir	-0.12780643	0.11528864	0.22419715
ENSG0000017	GAP43	protein_codir	-0.12786968	0.81751632	0.88794163
ENSG0000016	DNAJB14	protein_codir	-0.12787335	0.34620375	0.49925064
ENSG0000011	UPK2	protein_codir	-0.12788469	0.9479829	NA
ENSG0000010	CEP41	protein_codir	-0.1280584	0.50869006	0.65303367
ENSG0000011	INVS	protein_codir	-0.12811112	0.30460078	0.45604259
ENSG0000013	SYNCRIP	protein_codir	-0.12812977	0.46725853	0.61680289
ENSG0000027	RP11-504P24.	lncRNA	-0.12818731	0.79751792	0.87413289
ENSG0000013	CALHM2	protein_codir	-0.12825255	0.46761354	0.61716854
ENSG0000026	NDUFA7	protein_codir	-0.12839333	0.54962912	0.68691205
ENSG0000011	DROSHA	protein_codir	-0.12842672	0.11590926	0.22512394
ENSG0000027	RP11-54G14.1	lncRNA	-0.12843594	0.89441304	0.9370276
ENSG0000021	BBIP1	protein_codir	-0.12849777	0.15921938	0.28461843
ENSG0000019	SUPT3H	protein_codir	-0.12867277	0.41614095	0.56888799
ENSG0000026	RP11-677O4.6	lncRNA	-0.12873523	0.81621076	0.88692033
ENSG0000023	BOK-AS1	lncRNA	-0.12879612	0.9231105	0.95468676
ENSG0000016	COG7	protein_codir	-0.12887958	0.23414381	0.37748378
ENSG0000006	PDE4A	protein_codir	-0.12888443	0.45283469	0.60330285
ENSG0000011	LTBR	protein_codir	-0.1289264	0.53543443	0.67580403
ENSG0000018	FOCAD	protein_codir	-0.12916307	0.45128511	0.60200409
ENSG0000012	SNRPC	protein_codir	-0.12924172	0.21541202	0.35560816
ENSG0000023	PAXBP1-AS1	lncRNA	-0.12926493	0.53866437	0.67810821
ENSG0000011	BNIP1	protein_codir	-0.12937497	0.44593469	0.59685579
ENSG0000018	TMLHE	protein_codir	-0.12938176	0.51813755	0.66114654
ENSG0000026	KDSR-DT	lncRNA	-0.1293953	0.6196885	0.74303435
ENSG0000016	BMS1	protein_codir	-0.12943986	0.25969311	0.40680148
ENSG0000013	MAP1S	protein_codir	-0.12948264	0.22148538	0.36247118
ENSG0000012	SLC25A16	protein_codir	-0.12962836	0.25898917	0.40606727
ENSG0000028	GATD3B	protein_codir	-0.12966535	0.55729188	0.69307046
ENSG0000027	RP11-493E12.	lncRNA	-0.12967529	0.90763613	0.9449625
ENSG0000011	PLEKHM2	protein_codir	-0.12969013	0.15275225	0.27602347
ENSG0000028	RP11-2C24.9	protein_codir	-0.12971644	0.51201322	0.65568458
ENSG0000019	GAL3ST4	protein_codir	-0.12974592	0.72177781	0.82039107
ENSG0000019	KLHDC1	protein_codir	-0.1297928	0.57530494	0.70785471
ENSG0000023	LINC02015	lncRNA	-0.12981731	0.75835428	0.84697716
ENSG0000027	H2AC15	protein_codir	-0.12982841	0.83878332	0.90326662
ENSG0000016	CUL5	protein_codir	-0.12985677	0.36795627	0.52149796
ENSG0000013	POLR1E	protein_codir	-0.12988946	0.56957303	0.70324574
ENSG0000004	KATNIP	protein_codir	-0.12996012	0.11460708	0.22316703
ENSG0000017	KCMF1	protein_codir	-0.13007932	0.3093991	0.46107711
ENSG0000010	SLC35A2	protein_codir	-0.13011479	0.419198	0.57182034
ENSG0000023	ANKRD34C	protein_codir	-0.13014186	0.84764709	0.90916907
ENSG0000016	BAG5	protein_codir	-0.13026022	0.42304944	0.57505908
ENSG0000019	PSMD12	protein_codir	-0.13029489	0.42199329	0.57399067
ENSG0000011	HSPA9	protein_codir	-0.13035447	0.3737337	0.52730511
ENSG0000027	RP11-301O19	lncRNA	-0.13039321	0.83585923	0.90135603
ENSG0000017	TVP23B	protein_codir	-0.1305693	0.34461163	0.49768227

ENSG0000018	SHMT2	protein_codir	-0.1306099	0.47990478	0.62766745
ENSG0000013	KIF3A	protein_codir	-0.13061737	0.31156714	0.46332521
ENSG0000013	BPHL	protein_codir	-0.13077317	0.39593174	0.54883988
ENSG0000021	CHUK	protein_codir	-0.13080008	0.38018547	0.5335733
ENSG0000016	WASHC5	protein_codir	-0.13088643	0.4081688	0.56115765
ENSG0000007	ZNF37A	protein_codir	-0.13095602	0.21248095	0.35207124
ENSG0000025	SNHG9	lncRNA	-0.13101198	0.69911716	0.80346257
ENSG0000012	PEX16	protein_codir	-0.13101329	0.40139205	0.55440558
ENSG0000006	TP53BP1	protein_codir	-0.13103635	0.21228949	0.35188357
ENSG0000014	GTF3C5	protein_codir	-0.13115152	0.2167604	0.35700192
ENSG0000014	UFC1	protein_codir	-0.13116615	0.17945705	0.31077834
ENSG0000015	BABAM2	protein_codir	-0.13118859	0.09659693	0.19655275
ENSG0000010	CSNK2A1	protein_codir	-0.13119465	0.13547074	0.25256172
ENSG0000016	CPSF2	protein_codir	-0.13126602	0.33708077	0.48994253
ENSG0000010	PRPF6	protein_codir	-0.13137246	0.27443554	0.42249374
ENSG0000018	BICD2	protein_codir	-0.13146515	0.28167797	0.43109356
ENSG0000010	TSC22D1	protein_codir	-0.1314937	0.66334907	0.77729229
ENSG0000014	TRIM41	protein_codir	-0.13153061	0.42369771	0.57569797
ENSG0000013	NDUFAF1	protein_codir	-0.13170028	0.2528769	0.39906639
ENSG0000020	PLPP6	protein_codir	-0.13176048	0.48510119	0.63245196
ENSG0000000	SYPL1	protein_codir	-0.13177024	0.4366034	0.58787042
ENSG0000010	UBXN8	protein_codir	-0.13185952	0.57082526	0.7042252
ENSG0000016	GEM	protein_codir	-0.13200596	0.78105834	0.86308587
ENSG0000012	TIMM8A	protein_codir	-0.13235396	0.52819286	0.66969734
ENSG0000028	NIPBL-DT	lncRNA	-0.13249042	0.52222522	0.66472466
ENSG0000008	UIMC1	protein_codir	-0.1325108	0.37504194	0.52858388
ENSG0000011	GNB4	protein_codir	-0.13259758	0.60859308	0.73432095
ENSG0000025	LINC01603	lncRNA	-0.13262374	0.94581167	NA
ENSG0000011	PPM1G	protein_codir	-0.13271257	0.22623832	0.36827374
ENSG0000016	PRIMPOL	protein_codir	-0.13305089	0.26755162	0.41532779
ENSG0000010	GBF1	protein_codir	-0.13311116	0.11566381	0.22476849
ENSG0000012	WDR24	protein_codir	-0.13313551	0.40142196	0.55441917
ENSG0000021	TMX2	protein_codir	-0.133164	0.18793603	0.32176997
ENSG0000016	ACOX1	protein_codir	-0.13323726	0.3625501	0.51645551
ENSG0000016	INPPL1	protein_codir	-0.13341124	0.3946673	0.54766433
ENSG0000019	ZNF677	protein_codir	-0.13353828	0.40382043	0.55694677
ENSG0000010	TMEM248	protein_codir	-0.13354393	0.23449679	0.37785434
ENSG0000021	HOMEZ	protein_codir	-0.13359491	0.4480379	0.59891621
ENSG0000011	FAHD2A	protein_codir	-0.13369507	0.30369852	0.45499756
ENSG0000012	ATG14	protein_codir	-0.13373446	0.44700785	0.59782863
ENSG0000022	PIGBOS1	protein_codir	-0.13375213	0.3318414	0.48463724
ENSG0000023	KLF3-AS1	lncRNA	-0.13386485	0.64556901	0.76310092
ENSG0000017	ZBTB38	protein_codir	-0.1338672	0.49448731	0.64057687
ENSG0000010	MAPRE1	protein_codir	-0.13394347	0.40191592	0.55496264
ENSG0000001	UFL1	protein_codir	-0.13398991	0.36932013	0.52286749
ENSG0000019	DCLRE1A	protein_codir	-0.13400348	0.56231841	0.69706183
ENSG0000010	DRG2	protein_codir	-0.13417668	0.28576162	0.4353412

ENSG0000009	OCEL1	protein_codir	-0.13431192	0.4200503	0.57250379
ENSG0000028	RP11-155O18	lncRNA	-0.13433113	0.91122372	0.94705595
ENSG0000008	CHMP5	protein_codir	-0.13461845	0.15003999	0.27235177
ENSG0000010	SRPX	protein_codir	-0.13462708	0.74846399	0.83965258
ENSG0000015	USP12	protein_codir	-0.13468459	0.51363369	0.65702682
ENSG0000027	MAFIP	protein_codir	-0.13475409	0.73160728	0.8274322
ENSG0000023	SMIM26	protein_codir	-0.13480078	0.31817217	0.47017421
ENSG0000015	ZNF19	protein_codir	-0.13481375	0.63502535	0.75486982
ENSG0000028	RP11-307I14.!	lncRNA	-0.13481923	0.90401345	0.94307491
ENSG0000021	SACM1L	protein_codir	-0.13493179	0.22891122	0.37138909
ENSG0000016	CCT2	protein_codir	-0.1349691	0.47854308	0.62638548
ENSG0000022	LINC00092	lncRNA	-0.134973	0.77364845	0.85757395
ENSG0000023	TWIST2	protein_codir	-0.13497829	0.64471562	0.76238581
ENSG0000007	XRCC5	protein_codir	-0.13519868	0.33766193	0.4904666
ENSG0000013	DDB2	protein_codir	-0.13523922	0.56029666	0.69549205
ENSG0000022	TRAPPC12-AS	lncRNA	-0.13533921	0.93716986	0.96268341
ENSG0000016	MRPL55	protein_codir	-0.13551323	0.40352923	0.55668934
ENSG0000026	LINC01355	lncRNA	-0.13568135	0.79589597	0.87315883
ENSG0000016	RPS6KA4	protein_codir	-0.13575763	0.44500511	0.59614054
ENSG0000006	ELOVL1	protein_codir	-0.13587635	0.30560473	0.45724825
ENSG0000010	POP4	protein_codir	-0.13595731	0.22085719	0.36165741
ENSG0000008	DUSP12	protein_codir	-0.13597143	0.21131448	0.35073049
ENSG0000018	ZNF329	protein_codir	-0.13616993	0.36408868	0.51811682
ENSG0000018	THBS2	protein_codir	-0.13631185	0.72941033	0.82603402
ENSG0000006	PIGB	protein_codir	-0.13635782	0.28867765	0.43826069
ENSG0000000	KIAA0100	protein_codir	-0.13644388	0.35733187	0.51081757
ENSG0000016	NMD3	protein_codir	-0.13648083	0.39084291	0.54380689
ENSG0000011	COMMD2	protein_codir	-0.13648705	0.43409008	0.5854853
ENSG0000014	HNRNPLL	protein_codir	-0.13664068	0.40862386	0.56161381
ENSG0000014	TAF1A	protein_codir	-0.1366938	0.3573237	0.51081757
ENSG0000010	PIGU	protein_codir	-0.13670403	0.13352254	0.2501114
ENSG0000010	TWNK	protein_codir	-0.13679794	0.52336739	0.66559569
ENSG0000017	UBB	protein_codir	-0.13685581	0.32511643	0.47741591
ENSG0000020	MRPL53	protein_codir	-0.13686439	0.28332018	0.43288728
ENSG0000014	FOXK2	protein_codir	-0.13700004	0.29423038	0.4445404
ENSG0000007	FKBP6	protein_codir	-0.13715542	0.96262799	NA
ENSG0000016	MVD	protein_codir	-0.13728172	0.40684395	0.55986671
ENSG0000024	CRCP	protein_codir	-0.13733505	0.26421964	0.41172054
ENSG0000019	ZNF44	protein_codir	-0.13734028	0.54628833	0.68422291
ENSG0000014	CRTC3	protein_codir	-0.13735073	0.42171588	0.57375463
ENSG0000011	SUPT7L	protein_codir	-0.13735436	0.16919995	0.29773845
ENSG0000017	EIF1AX	protein_codir	-0.13743517	0.46697361	0.61658911
ENSG0000019	XRCC6	protein_codir	-0.13750985	0.32477427	0.47716716
ENSG0000011	RAB3GAP2	protein_codir	-0.13757193	0.14022162	0.2593532
ENSG0000010	GOSR1	protein_codir	-0.137699	0.18967474	0.32400374
ENSG0000027	RP4-569M23.	lncRNA	-0.13788191	0.86451193	0.91926158
ENSG0000020	ABCF1	protein_codir	-0.1380929	0.20521094	0.34309481

ENSG0000017	ELP5	protein_codir	-0.13816998	0.222816	0.36410945
ENSG0000025	TUG1	protein_codir	-0.13825543	0.24437882	0.38939328
ENSG0000012	PEX1	protein_codir	-0.1382816	0.24091521	0.38545317
ENSG0000020	PJVK	protein_codir	-0.13843611	0.67219287	0.78383502
ENSG0000013	H3-3B	protein_codir	-0.13861394	0.40066688	0.55384712
ENSG0000018	ZNF320	protein_codir	-0.13861616	0.39331249	0.54636076
ENSG0000013	KCTD3	protein_codir	-0.13868611	0.46312703	0.61306344
ENSG0000027	CTD-2588E21.	lncRNA	-0.13879656	0.7656453	0.85230526
ENSG0000015	KCNA6	protein_codir	-0.13880343	0.73427593	0.82964619
ENSG0000016	RAMAC	protein_codir	-0.13881668	0.1983109	0.33457621
ENSG0000008	DELE1	protein_codir	-0.13888785	0.13001337	0.24511736
ENSG0000015	NTAQ1	protein_codir	-0.13890549	0.41813829	0.57087113
ENSG0000022	HAR1A	lncRNA	-0.13900892	0.62974533	0.75071473
ENSG0000017	SGF29	protein_codir	-0.13902692	0.41595581	0.56880381
ENSG0000016	CHTOP	protein_codir	-0.13906304	0.17141596	0.30066101
ENSG0000021	COG8	protein_codir	-0.13909577	0.28150319	0.43087382
ENSG0000006	TBC1D25	protein_codir	-0.13909796	0.31629144	0.46801508
ENSG0000027	GOLPH3-DT	lncRNA	-0.13910345	0.60436336	0.73093582
ENSG0000025	TRIM6-TRIM3	protein_codir	-0.13918345	0.9368342	0.962446
ENSG0000009	SUPT16H	protein_codir	-0.13919442	0.33261918	0.48536252
ENSG0000016	C19orf33	protein_codir	-0.139206	0.85479276	0.91319595
ENSG0000011	SMAP1	protein_codir	-0.13922655	0.20295019	0.34009734
ENSG0000000	VPS50	protein_codir	-0.13928909	0.32655505	0.47884108
ENSG0000015	ZSCAN12	protein_codir	-0.13937442	0.43133566	0.58262378
ENSG0000017	IRAG1-AS1	lncRNA	-0.13941056	0.87346738	0.92497464
ENSG0000013	ARFIP2	protein_codir	-0.13953769	0.40890909	0.56186602
ENSG0000013	ZNF414	protein_codir	-0.13955087	0.38281408	0.53617309
ENSG0000016	YWHAB	protein_codir	-0.13994022	0.2446662	0.38964888
ENSG0000023	TTC28-AS1	lncRNA	-0.14000293	0.50127976	0.6461091
ENSG0000026	RP11-265N6.1	lncRNA	-0.14010291	0.81353048	0.88540121
ENSG0000020	CDPF1	protein_codir	-0.1402079	0.42416033	0.57622884
ENSG0000023	SBK3	protein_codir	-0.14021375	0.79529354	0.8728793
ENSG0000007	SMARCE1	protein_codir	-0.14024417	0.24191977	0.38674678
ENSG0000013	RGS8	protein_codir	-0.14029837	0.798547	0.87474689
ENSG0000010	KATNAL1	protein_codir	-0.14030262	0.56748234	0.70130274
ENSG0000016	PSCA	protein_codir	-0.14031517	0.85470334	0.91319595
ENSG0000014	ZNF514	protein_codir	-0.14036058	0.56093504	0.6958154
ENSG0000011	SMNDC1	protein_codir	-0.14038004	0.21645127	0.35678811
ENSG0000017	CKS1B	protein_codir	-0.14038241	0.5078718	0.65234708
ENSG0000014	GOLPH3L	protein_codir	-0.1404382	0.31474781	0.4665834
ENSG0000009	SH3GLB1	protein_codir	-0.14064304	0.41937849	0.57196154
ENSG0000016	TMEM234	protein_codir	-0.14079796	0.49159126	0.63826961
ENSG0000012	MRPS7	protein_codir	-0.14086611	0.18372996	0.31643501
ENSG0000026	LMF1-AS1	lncRNA	-0.14095117	0.79256269	0.87102425
ENSG0000024	AC037459.4	protein_codir	-0.14119528	0.78615681	0.86630031
ENSG0000015	NMRAL1	protein_codir	-0.14120182	0.33761054	0.49044354
ENSG0000026	MIR762HG	lncRNA	-0.14129407	0.58792266	0.71816478

ENSG0000025	RP11-649G15	lncRNA	-0.14135262	0.93407194	NA
ENSG0000014	HDGF	protein_codir	-0.14153094	0.35178274	0.50530639
ENSG0000011	RAB14	protein_codir	-0.14156567	0.18415198	0.31702054
ENSG0000022	ENTPD3-AS1	lncRNA	-0.14156875	0.5658679	0.70011733
ENSG0000023	FAM27C	lncRNA	-0.14161591	0.54751259	0.68522805
ENSG0000014	TMCO1	protein_codir	-0.14166646	0.16135262	0.28742724
ENSG0000020	ZNF316	protein_codir	-0.14183629	0.38911746	0.54208737
ENSG0000026	DCXR-DT	lncRNA	-0.14188779	0.9086641	0.94564063
ENSG0000009	MISP	protein_codir	-0.14201112	0.8789932	0.9281566
ENSG0000011	CTBS	protein_codir	-0.14202189	0.46114535	0.61139096
ENSG0000016	ZSWIM1	protein_codir	-0.14202929	0.47366167	0.62208917
ENSG0000010	B9D1	protein_codir	-0.14208149	0.35227767	0.505844
ENSG0000015	UTP14A	protein_codir	-0.14235345	0.25256169	0.39877402
ENSG0000013	EMC3	protein_codir	-0.14237676	0.15907796	0.28447817
ENSG0000013	ASCC1	protein_codir	-0.14250437	0.12629385	0.24016737
ENSG0000023	LINC00884	lncRNA	-0.1425226	0.59812954	0.7262964
ENSG0000021	MT-ND4L	protein_codir	-0.14254105	0.60486029	0.73131857
ENSG0000020	ZNF155	protein_codir	-0.14267095	0.65617318	0.77154122
ENSG0000019	AC005276.1	lncRNA	-0.14268209	0.91213033	0.9478194
ENSG0000017	CFL1	protein_codir	-0.142805	0.42935184	0.58099543
ENSG0000017	TMEM139	protein_codir	-0.14282146	0.76030453	0.84846914
ENSG0000012	TIMM17B	protein_codir	-0.14305546	0.3664843	0.52037304
ENSG0000006	MSANTD3	protein_codir	-0.14315175	0.47847115	0.62635069
ENSG0000015	PROM2	protein_codir	-0.14330179	0.7138219	0.81446527
ENSG0000023	RBM14	protein_codir	-0.14345505	0.41564412	0.56857464
ENSG0000016	ENDOG	protein_codir	-0.14354314	0.46481087	0.61476207
ENSG0000015	KLHL40	protein_codir	-0.14360629	0.88619719	0.93253824
ENSG0000016	VPS39	protein_codir	-0.14376761	0.10680257	0.21197807
ENSG0000017	GNG5	protein_codir	-0.14387967	0.36597115	0.51988495
ENSG0000013	LYVE1	protein_codir	-0.14388584	0.80853045	0.88155736
ENSG0000010	CCNB1IP1	protein_codir	-0.14400398	0.43557893	0.58687903
ENSG0000011	RARS1	protein_codir	-0.14402052	0.22455261	0.36623789
ENSG0000027	RP11-9E17.1	lncRNA	-0.14414531	0.65804268	0.77275251
ENSG0000019	AP2A1	protein_codir	-0.14420808	0.23794511	0.38205115
ENSG0000017	TOMM5	protein_codir	-0.14428705	0.3080164	0.45973818
ENSG0000014	FEM1A	protein_codir	-0.14434585	0.26650933	0.41409885
ENSG0000025	LINC02235	lncRNA	-0.14439694	0.88484963	0.93166922
ENSG0000011	SASH1	protein_codir	-0.14443028	0.62562222	0.74756951
ENSG0000016	ZNF208	protein_codir	-0.14449716	0.55838245	0.69395803
ENSG0000025	RP11-603J24.	lncRNA	-0.14452531	0.89364336	0.93667905
ENSG0000015	C16orf87	protein_codir	-0.14455125	0.45340735	0.60390177
ENSG0000013	PSRC1	protein_codir	-0.14476002	0.66647916	0.77954411
ENSG0000019	ZNF420	protein_codir	-0.14490507	0.47424293	0.62267493
ENSG0000016	PKNOX1	protein_codir	-0.14495587	0.21000302	0.34916654
ENSG0000018	DENND5A	protein_codir	-0.145035	0.41675331	0.56949616
ENSG0000028	RP11-167N5.6	lncRNA	-0.1450675	0.93945253	NA
ENSG0000009	ALKBH5	protein_codir	-0.14510885	0.3278082	0.48022387

ENSG0000012	TBC1D20	protein_codir	-0.14530658	0.11841902	0.2287706
ENSG0000019	ZNF682	protein_codir	-0.1453745	0.68534051	0.79330193
ENSG0000027	CTD-2358C21	lncRNA	-0.14547504	0.88424698	0.93128621
ENSG0000022	PGA4	protein_codir	-0.14550307	0.85644085	0.91426956
ENSG0000014	SLC25A26	protein_codir	-0.14553655	0.32615299	0.47840391
ENSG0000010	CCDC61	protein_codir	-0.14554493	0.32361301	0.47591669
ENSG0000011	POLR1G	protein_codir	-0.14555969	0.40461538	0.55754115
ENSG0000028	AL022345.12	lncRNA	-0.14560845	0.815014	0.8861026
ENSG0000003	DNAH5	protein_codir	-0.14561402	0.71605274	0.81620143
ENSG0000018	NCR3LG1	protein_codir	-0.14565445	0.62976664	0.75071473
ENSG0000023	DLEU2	lncRNA	-0.14565925	0.59007303	0.71983645
ENSG0000016	SMG8	protein_codir	-0.14569241	0.51367639	0.65702682
ENSG0000012	TSFM	protein_codir	-0.14569859	0.28475912	0.43436499
ENSG0000022	EPHA1-AS1	lncRNA	-0.1457402	NA	NA
ENSG0000020	ITSN1	protein_codir	-0.14581793	0.54579426	0.68372811
ENSG0000013	PEX5	protein_codir	-0.14583527	0.44570826	0.59674618
ENSG0000017	ARNT2	protein_codir	-0.14590324	0.71408489	0.81463073
ENSG0000007	NCBP3	protein_codir	-0.14600201	0.1687168	0.29709937
ENSG0000007	FRYL	protein_codir	-0.14600314	0.46789898	0.61728943
ENSG0000022	PARG	protein_codir	-0.14607994	0.21659463	0.35690192
ENSG0000017	JAGN1	protein_codir	-0.14614196	0.09750576	0.19799114
ENSG0000000	PRKAR2B	protein_codir	-0.1462123	0.72578473	0.82335295
ENSG0000009	SPAG7	protein_codir	-0.14624032	0.31681762	0.46853058
ENSG0000024	ARFGAP3	protein_codir	-0.14633208	0.37792172	0.53158474
ENSG0000016	SLC33A1	protein_codir	-0.14648532	0.23669107	0.3806621
ENSG0000016	KLKB1	protein_codir	-0.14671317	0.70098854	0.80517278
ENSG0000026	FAM156A	protein_codir	-0.14672669	0.67511112	0.78597914
ENSG0000012	PSPN	protein_codir	-0.14681465	0.45943125	0.60989576
ENSG0000016	TMUB1	protein_codir	-0.14692251	0.43544568	0.58680842
ENSG0000012	KBTBD4	protein_codir	-0.14723972	0.30613148	0.45770526
ENSG0000012	POR	protein_codir	-0.1473381	0.45884666	0.60926613
ENSG0000027	RP5-1159O4.2	lncRNA	-0.14736621	0.76538153	0.85221557
ENSG0000028	RP11-322M19	lncRNA	-0.14749529	0.86680932	0.92088135
ENSG0000026	CTD-2528L19.	lncRNA	-0.14757053	0.58088526	0.71256222
ENSG0000017	SMAD2	protein_codir	-0.1477316	0.28892967	0.43849868
ENSG0000017	PWWP2A	protein_codir	-0.1478061	0.15514528	0.27923048
ENSG0000011	NPHP3	protein_codir	-0.14781776	0.3847606	0.53799867
ENSG0000022	ZNF816-ZNF3	protein_codir	-0.14788061	0.91592794	0.95001426
ENSG0000019	CD55	protein_codir	-0.14790933	0.62688112	0.74844198
ENSG0000014	CIZ1	protein_codir	-0.14790957	0.19200208	0.32674661
ENSG0000016	ZCCHC4	protein_codir	-0.14797832	0.31440415	0.46633741
ENSG0000010	FKTN	protein_codir	-0.1480258	0.28826694	0.43790193
ENSG0000028	RP11-936I5.5	lncRNA	-0.14807555	0.93844728	NA
ENSG0000026	PGLS-DT	lncRNA	-0.148223	0.67038566	0.7825227
ENSG0000014	ABHD1	protein_codir	-0.14824198	0.73020301	0.82649812
ENSG0000011	MRPL3	protein_codir	-0.14828099	0.26968296	0.41751145
ENSG0000020	AC074391.1	lncRNA	-0.14830716	0.79463645	0.87243546

ENSG0000017	TRMT61B	protein_codir	-0.14833855	0.25696009	0.40371015
ENSG0000014	SUMF1	protein_codir	-0.14836108	0.41967928	0.57219575
ENSG0000027	AC000403.4	lncRNA	-0.14846625	0.56099787	0.69586209
ENSG0000014	GNRH1	protein_codir	-0.14847661	0.78088237	0.86296047
ENSG0000012	DYNC2I1	protein_codir	-0.14857842	0.51088664	0.65475797
ENSG0000017	FAXDC2	protein_codir	-0.14859622	0.69101157	0.79721174
ENSG0000010	CYP3A5	protein_codir	-0.14874157	0.81510532	0.8861026
ENSG0000014	GLUD1	protein_codir	-0.14877639	0.33239404	0.48511088
ENSG0000016	DHX57	protein_codir	-0.1487848	0.27104576	0.41902406
ENSG0000026	RP13-192B19.	lncRNA	-0.14880699	0.89856116	0.93967907
ENSG0000016	NOL9	protein_codir	-0.14889898	0.2888267	0.43843305
ENSG0000028	RP11-79P21.2	protein_codir	-0.14897728	0.7525006	0.84244929
ENSG0000012	TARDBP	protein_codir	-0.14903766	0.0955877	0.19497322
ENSG0000013	PSME3	protein_codir	-0.14905278	0.28344565	0.43300684
ENSG0000027	RP11-9N20.3	lncRNA	-0.14913953	0.70833439	0.81057795
ENSG0000026	CTC-429P9.3	lncRNA	-0.14917571	0.5179576	0.66105952
ENSG0000028	RP11-428J1.6	lncRNA	-0.14918267	0.76698036	0.85308353
ENSG0000027	SUGT1-DT	lncRNA	-0.1492456	0.71974777	0.81902741
ENSG0000016	THOC7	protein_codir	-0.14942245	0.21632561	0.35666866
ENSG0000012	ALKBH7	protein_codir	-0.14944555	0.27443737	0.42249374
ENSG0000027	RP11-131L12.	lncRNA	-0.14952296	0.79712114	0.8738951
ENSG0000006	SPEN	protein_codir	-0.14968174	0.33629543	0.48927938
ENSG0000026	RP11-119F7.5	lncRNA	-0.14971215	0.69745045	0.80199448
ENSG0000028	PDCD6-AHRR	protein_codir	-0.14983248	0.94070651	0.96509598
ENSG0000013	LRCH1	protein_codir	-0.1498342	0.33796582	0.49076247
ENSG0000017	PTEN	protein_codir	-0.15013347	0.27918888	0.42804313
ENSG0000026	EGLN2	protein_codir	-0.15017751	0.08960229	0.18560187
ENSG0000020	C1QTNF9B	protein_codir	-0.15022401	0.86640036	0.92063163
ENSG0000014	NPEPPS	protein_codir	-0.15024861	0.24549571	0.39063207
ENSG0000013	TMEM165	protein_codir	-0.15025992	0.36581175	0.51968525
ENSG0000017	HOXD4	protein_codir	-0.15042849	0.70191666	0.80580815
ENSG0000006	ZFY	protein_codir	-0.15056686	0.72399867	0.82190067
ENSG0000027	RP11-1246C1!	lncRNA	-0.15058364	0.57095635	0.704324
ENSG0000010	GMEB2	protein_codir	-0.15062483	0.31328752	0.46501511
ENSG0000017	SUCLG2	protein_codir	-0.15072218	0.33950478	0.49241832
ENSG0000028	RP11-478C6.7	lncRNA	-0.15077896	0.80113605	0.8765748
ENSG0000022	ATXN1L	protein_codir	-0.1507802	0.35804455	0.51162453
ENSG0000027	RP11-350J20.	lncRNA	-0.15093063	0.7062868	0.80907642
ENSG0000023	RP11-27K13.3	lncRNA	-0.15101217	0.85617851	0.91406016
ENSG0000014	GLYR1	protein_codir	-0.15128667	0.13271103	0.24906426
ENSG0000016	DPY30	protein_codir	-0.15140557	0.18850125	0.32239795
ENSG0000023	LINC02609	lncRNA	-0.15161905	0.64026036	0.75880674
ENSG0000012	EFNB2	protein_codir	-0.15174435	0.53479428	0.67521568
ENSG0000023	RP11-553A21	lncRNA	-0.1517535	0.80214281	0.87743305
ENSG0000011	KANSL3	protein_codir	-0.15177988	0.41938324	0.57196154
ENSG0000013	DENR	protein_codir	-0.15186581	0.14180994	0.26134586
ENSG0000022	TMCO1-AS1	lncRNA	-0.15188017	0.77810657	0.86102979

ENSG0000010	SGTA	protein_codir	-0.15206525	0.17491667	0.30501916
ENSG0000017	UBXN2A	protein_codir	-0.15210795	0.33039808	0.48296349
ENSG0000017	SSNA1	protein_codir	-0.15212674	0.27249909	0.4204002
ENSG0000018	TMEM216	protein_codir	-0.15249093	0.18044014	0.31199178
ENSG0000013	ARHGEF11	protein_codir	-0.15250674	0.23544068	0.37895522
ENSG0000010	UQCC1	protein_codir	-0.15260127	0.25725073	0.4039837
ENSG0000018	DIABLO	protein_codir	-0.15260461	0.21212507	0.35167433
ENSG0000010	MTHFSD	protein_codir	-0.15266069	0.3288357	0.4811854
ENSG0000016	PLIN1	protein_codir	-0.15282022	0.85471595	0.91319595
ENSG0000027	RP11-147L13.	lncRNA	-0.15283101	0.24592923	0.39116414
ENSG0000022	LINC02051	lncRNA	-0.15292484	0.93542088	NA
ENSG0000017	C1GALT1C1	protein_codir	-0.15304637	0.17272631	0.3023407
ENSG0000013	FAM149B1	protein_codir	-0.15305099	0.18736322	0.32094841
ENSG0000014	ATG12	protein_codir	-0.15307415	0.13890232	0.25740898
ENSG0000017	OTP	protein_codir	-0.15313898	0.9381464	NA
ENSG0000020	TRIM27	protein_codir	-0.1531647	0.17863904	0.3096724
ENSG0000027	CTA-363E19.2	lncRNA	-0.1531898	0.70934215	0.81126705
ENSG0000010	ZNF821	protein_codir	-0.15336926	0.41644043	0.56924105
ENSG0000021	RTL10	protein_codir	-0.15348047	0.43970886	0.59072712
ENSG0000011	ARMC8	protein_codir	-0.15353686	0.09286463	0.19082814
ENSG0000009	XXbac-B461K1	lncRNA	-0.1538295	0.76627804	0.85268923
ENSG0000011	NIT2	protein_codir	-0.15402573	0.27545486	0.42361155
ENSG0000015	PDE6D	protein_codir	-0.1540304	0.12379416	0.23652427
ENSG0000009	TINF2	protein_codir	-0.15415804	0.18127176	0.31315524
ENSG0000016	THBS3	protein_codir	-0.15429354	0.49237367	0.63891759
ENSG0000027	RP11-423P10.	lncRNA	-0.15433643	0.63770103	0.75710551
ENSG0000013	SRSF1	protein_codir	-0.15440172	0.21116156	0.35056094
ENSG0000007	MLH1	protein_codir	-0.15448775	0.08582893	0.17987431
ENSG0000012	FAM199X	protein_codir	-0.15457327	0.29383692	0.44409847
ENSG0000016	GPS1	protein_codir	-0.15483069	0.1505981	0.27307769
ENSG0000018	ACTG1	protein_codir	-0.15493288	0.35382235	0.50745526
ENSG0000023	RPL37A-DT	lncRNA	-0.15502069	0.76902582	0.85464503
ENSG0000027	RP11-171I2.5	lncRNA	-0.15506534	0.77171645	0.85639473
ENSG0000015	EDNRA	protein_codir	-0.15511735	0.68815003	0.79535942
ENSG0000014	TAOK2	protein_codir	-0.15516971	0.11242817	0.22013797
ENSG0000016	RNF123	protein_codir	-0.15517581	0.02041681	0.06021488
ENSG0000020	RUFY2	protein_codir	-0.15518809	0.25480965	0.40129106
ENSG0000014	SRD5A1	protein_codir	-0.15520425	0.42185172	0.57382638
ENSG0000027	RP1-161P9.5	lncRNA	-0.15537991	0.92690388	NA
ENSG0000002	NCAPH2	protein_codir	-0.15543547	0.32027514	0.4723913
ENSG0000016	TOR1AIP2	protein_codir	-0.15544416	0.18785896	0.32169785
ENSG0000017	FLJ37453	lncRNA	-0.15547903	0.48535114	0.63267034
ENSG0000017	RALGAPA1	protein_codir	-0.15560083	0.27950848	0.42846177
ENSG0000025	RP11-394O4.3	lncRNA	-0.15578636	0.86413125	0.91911928
ENSG0000011	MLH3	protein_codir	-0.15581137	0.35005391	0.50359336
ENSG0000012	CLCC1	protein_codir	-0.15584785	0.16711335	0.29523288
ENSG0000028	RP11-107C16.	protein_codir	-0.15606157	0.94581029	NA

ENSG0000014	C7orf26	protein_codir	-0.15632541	0.22242055	0.36361382
ENSG0000018	ZNF527	protein_codir	-0.15635336	0.41816149	0.57087113
ENSG0000013	ERAL1	protein_codir	-0.15643164	0.1557362	0.28002303
ENSG0000014	TBX19	protein_codir	-0.15643367	0.53767051	0.67736086
ENSG0000025	CTD-2373J6.2	lncRNA	-0.15646709	0.89969627	0.94009758
ENSG0000013	ZNF337	protein_codir	-0.15650837	0.49631772	0.64222514
ENSG0000025	GATC	protein_codir	-0.15654475	0.19044094	0.32509139
ENSG0000025	PINX1	protein_codir	-0.15660703	0.21078875	0.35013142
ENSG0000018	CCR10	protein_codir	-0.15664662	0.55729132	0.69307046
ENSG0000019	WDR45	protein_codir	-0.15680381	0.38831853	0.54132932
ENSG0000001	MVP	protein_codir	-0.15682905	0.20431498	0.34190729
ENSG0000014	PKP4	protein_codir	-0.15696678	0.34350196	0.496677
ENSG0000016	ZNF148	protein_codir	-0.15704204	0.42850763	0.58019417
ENSG0000016	SDC3	protein_codir	-0.15710357	0.51077323	0.65467338
ENSG0000017	LARP7	protein_codir	-0.15738009	0.25981885	0.40695228
ENSG0000016	COA7	protein_codir	-0.15756356	0.40463115	0.55754115
ENSG0000018	XPOT	protein_codir	-0.15766065	0.21799128	0.35842318
ENSG0000017	TRIAP1	protein_codir	-0.15766295	0.22753447	0.36978535
ENSG0000026	CTD-2525I3.3	lncRNA	-0.1576706	0.96145987	NA
ENSG0000023	LINC01090	lncRNA	-0.1576706	0.96145987	NA
ENSG0000024	LINC02504	lncRNA	-0.1576706	0.96145987	NA
ENSG0000027	RP11-573G6.8	lncRNA	-0.1576706	0.96145987	NA
ENSG0000025	NAV2-AS1	lncRNA	-0.1576706	0.96145987	NA
ENSG0000028	RP11-124N19	lncRNA	-0.1576706	0.96145987	NA
ENSG0000025	LINC02279	lncRNA	-0.1576706	0.96145987	NA
ENSG0000026	RP11-387H17	lncRNA	-0.1576706	0.96145987	NA
ENSG0000028	RP4-800J21.4	lncRNA	-0.1576706	0.96145987	NA
ENSG0000025	RP11-613C6.2	lncRNA	-0.15767177	0.95806548	NA
ENSG0000025	RP11-196H14	lncRNA	-0.15767188	0.95770207	NA
ENSG0000026	RP11-480I12.1	lncRNA	-0.15767196	0.95742375	NA
ENSG0000025	RP3-377H17.2	lncRNA	-0.15767204	0.95713531	NA
ENSG0000010	CABP5	protein_codir	-0.15767206	0.95708872	NA
ENSG0000022	LINC00343	lncRNA	-0.15767213	0.95684479	NA
ENSG0000018	MYADML2	protein_codir	-0.15767217	0.95668537	NA
ENSG0000011	ACTL8	protein_codir	-0.15767217	0.95667712	NA
ENSG0000025	RASSF10-DT	lncRNA	-0.15767226	0.95636713	NA
ENSG0000024	CASC16	lncRNA	-0.15767229	0.95623321	NA
ENSG0000023	DDX39B-AS1	lncRNA	-0.15767236	0.95598519	NA
ENSG0000026	RP11-53B2.1	lncRNA	-0.1576725	0.95545186	NA
ENSG0000025	RP11-7M10.2	lncRNA	-0.15767252	0.95537375	NA
ENSG0000023	LINC01827	lncRNA	-0.15767267	0.95474691	NA
ENSG0000013	PLA2G12B	protein_codir	-0.15767413	0.94717468	NA
ENSG0000027	RP11-19E18.2	lncRNA	-0.15767418	0.94684099	NA
ENSG0000026	LINC01926	lncRNA	-0.15767419	0.94680986	NA
ENSG0000018	C12orf40	protein_codir	-0.15767419	0.94676273	NA
ENSG0000027	CTD-3028N15	lncRNA	-0.15767424	0.94645941	NA
ENSG0000007	ACTL6B	protein_codir	-0.15767427	0.94624755	NA

ENSG0000025	RP11-90P5.2	lncRNA	-0.15767427	0.94622983	NA
ENSG0000025	RP11-804A23	lncRNA	-0.15767429	0.94608161	NA
ENSG0000028	RP11-509A17	lncRNA	-0.1576743	0.94604141	NA
ENSG0000028	CH17-322D16	lncRNA	-0.1576743	0.94600351	NA
ENSG0000025	RP11-186F10.	lncRNA	-0.1576743	0.94599912	NA
ENSG0000023	RP11-205K6.1	lncRNA	-0.15767431	0.94598144	NA
ENSG0000022	AC006372.5	lncRNA	-0.15767431	0.94591932	NA
ENSG0000027	RP11-370A5.2	lncRNA	-0.15767433	0.94582712	NA
ENSG0000025	RP11-219E7.3	lncRNA	-0.15767434	0.94574141	NA
ENSG0000016	C1orf147	lncRNA	-0.15767437	0.9455325	NA
ENSG0000026	MIR1539	lncRNA	-0.15767439	0.94540201	NA
ENSG0000015	APOA2	protein_codir	-0.15767442	0.94519832	NA
ENSG0000025	RP11-482D24	lncRNA	-0.15767443	0.94510794	NA
ENSG0000027	RP5-1057I20.5	lncRNA	-0.15767444	0.94498367	NA
ENSG0000028	RP11-568J23.	lncRNA	-0.15767445	0.94497277	NA
ENSG0000028	PANDAR	lncRNA	-0.15767448	0.94475247	NA
ENSG0000023	RAI1-AS1	lncRNA	-0.15767448	0.94468939	NA
ENSG0000027	RP11-307C12.	lncRNA	-0.1576745	0.94456613	NA
ENSG0000023	LINC02653	lncRNA	-0.1576745	0.94454573	NA
ENSG0000013	GAD2	protein_codir	-0.15767459	0.94387673	NA
ENSG0000025	AC078852.1	lncRNA	-0.15767543	0.93580668	NA
ENSG0000015	NODAL	protein_codir	-0.15767546	0.93547945	NA
ENSG0000021	SMCO1	protein_codir	-0.15767548	0.93513858	NA
ENSG0000024	GMCL2	protein_codir	-0.1576755	0.93497392	NA
ENSG0000025	AC025442.3	lncRNA	-0.15767552	0.93472243	NA
ENSG0000028	CTD-2381F24.	lncRNA	-0.15767561	0.93361891	NA
ENSG0000014	CDR2	protein_codir	-0.15782987	0.50514144	0.64971625
ENSG0000015	ELOC	protein_codir	-0.15793467	0.45278602	0.60327785
ENSG0000010	ELF2	protein_codir	-0.1580008	0.30529112	0.45687803
ENSG0000020	GIGYF2	protein_codir	-0.1580048	0.18674418	0.32020576
ENSG0000022	RAB6C	protein_codir	-0.15808759	0.81102318	0.88337591
ENSG0000015	H2BC5	protein_codir	-0.15815426	0.71422194	0.81467497
ENSG0000016	C9orf64	protein_codir	-0.15817032	0.29926355	0.45029741
ENSG0000017	SFT2D3	protein_codir	-0.15836893	0.426611	0.5784201
ENSG0000017	METTL18	protein_codir	-0.15839197	0.44342228	0.59441484
ENSG0000009	RBM27	protein_codir	-0.15842211	0.38114624	0.53446003
ENSG0000028	CTB-193M12.	lncRNA	-0.15848482	0.51021287	0.65422838
ENSG0000014	RPP30	protein_codir	-0.15850079	0.10430191	0.20825697
ENSG0000022	MRPL20-AS1	lncRNA	-0.15858912	0.3662928	0.52020717
ENSG0000017	TP53RK	protein_codir	-0.15865325	0.24872263	0.39442608
ENSG0000001	MAMLD1	protein_codir	-0.15867321	0.57050167	0.70399497
ENSG0000010	URI1	protein_codir	-0.15882886	0.17269743	0.30231425
ENSG0000011	STEAP3	protein_codir	-0.15882905	0.61445107	0.73854992
ENSG0000017	ERCC4	protein_codir	-0.15884343	0.38808326	0.54113791
ENSG0000026	RP11-29G8.3	lncRNA	-0.15888286	0.56751693	0.70130274
ENSG0000013	ZNF426	protein_codir	-0.15893976	0.23901645	0.38328115
ENSG0000019	ABCB8	protein_codir	-0.15905318	0.33752677	0.4903882

ENSG0000006	TM7SF3	protein_codir	-0.15906082	0.31109661	0.46290746
ENSG0000018	COPG1	protein_codir	-0.15912617	0.21191124	0.3514681
ENSG0000011	SLC17A5	protein_codir	-0.15920977	0.3390029	0.49189704
ENSG0000026	RP11-556I13.1	lncRNA	-0.15932776	0.89197313	0.93560299
ENSG0000010	ZNF175	protein_codir	-0.15938303	0.39060905	0.54358955
ENSG0000013	SESN2	protein_codir	-0.15944245	0.39618484	0.54905295
ENSG0000026	CTC-429P9.5	lncRNA	-0.15950731	0.61034318	0.73573454
ENSG0000015	SLC24A2	protein_codir	-0.1596857	0.84461828	0.90718868
ENSG0000010	FRG1	protein_codir	-0.15981268	0.15026681	0.2726918
ENSG0000013	BLOC1S1	protein_codir	-0.15986798	0.31155337	0.46332521
ENSG0000013	STT3A	protein_codir	-0.15995747	0.26003151	0.40716993
ENSG0000022	LINC01465	lncRNA	-0.16008813	0.60331736	0.73006026
ENSG0000025	HOXA-AS3	lncRNA	-0.16012092	0.72734251	0.8244429
ENSG0000011	TDP2	protein_codir	-0.16019555	0.1462475	0.26752474
ENSG0000025	AL157871.2	lncRNA	-0.16021797	0.94457174	NA
ENSG0000009	FBXL19	protein_codir	-0.1602221	0.20355389	0.34089288
ENSG0000028	RP4-568C11.5	lncRNA	-0.16030217	0.92321633	NA
ENSG0000010	CYTH2	protein_codir	-0.16051694	0.41294455	0.56600309
ENSG0000011	ART4	protein_codir	-0.16062092	0.74489168	0.83708634
ENSG0000011	ABITRAM	protein_codir	-0.16069606	0.20908343	0.34801039
ENSG0000024	C8orf58	protein_codir	-0.16071857	0.33049745	0.48307332
ENSG0000025	ARPIN-AP3S2	protein_codir	-0.16086575	0.46486057	0.61479535
ENSG0000014	ALDOA	protein_codir	-0.16098766	0.33578765	0.48869507
ENSG0000010	PES1	protein_codir	-0.16105075	0.22452754	0.36623789
ENSG0000011	GOLT1B	protein_codir	-0.16108174	0.28492001	0.43453841
ENSG0000017	SPSB1	protein_codir	-0.16129854	0.59608561	0.7244515
ENSG0000014	STOM	protein_codir	-0.16129938	0.55744379	0.69322816
ENSG0000001	MBTPS2	protein_codir	-0.1613163	0.4268254	0.57865399
ENSG0000018	GJA4	protein_codir	-0.16134866	0.62314301	0.74574829
ENSG0000010	MGRN1	protein_codir	-0.16137867	0.13479531	0.25177835
ENSG0000016	TMEM68	protein_codir	-0.16137918	0.29577532	0.44621555
ENSG0000015	FAM160B1	protein_codir	-0.16154384	0.32525112	0.4775883
ENSG0000017	CMKLR1	protein_codir	-0.16157067	0.55972235	0.6951228
ENSG0000025	AP000662.4	lncRNA	-0.16164411	0.70304158	0.80661773
ENSG0000012	GLO1	protein_codir	-0.16169083	0.15503148	0.27908736
ENSG0000012	FYTTD1	protein_codir	-0.16173963	0.37791885	0.53158474
ENSG0000014	SLC39A3	protein_codir	-0.16184828	0.22168214	0.36269005
ENSG0000008	ATRX	protein_codir	-0.16207917	0.23352961	0.37676161
ENSG0000000	BAZ1B	protein_codir	-0.1620958	0.11580281	0.22497528
ENSG0000027	RAB33B-AS1	lncRNA	-0.16215696	0.50497598	0.64958816
ENSG0000010	SRPX2	protein_codir	-0.16238026	0.65172996	0.76765961
ENSG0000013	UBQLN1	protein_codir	-0.16238271	0.26598564	0.41354638
ENSG0000010	GYS1	protein_codir	-0.16265206	0.29973054	0.45072998
ENSG0000007	MRPS34	protein_codir	-0.16275475	0.22790644	0.37019026
ENSG0000016	PRDX3	protein_codir	-0.16281666	0.22993348	0.37232589
ENSG0000013	RANBP6	protein_codir	-0.1630714	0.43381285	0.5852257
ENSG0000019	VPS13A	protein_codir	-0.16316877	0.52012803	0.66283382

ENSG0000015	RPUSD3	protein_codir	-0.16318922	0.23286441	0.37590385
ENSG0000017	KCNE5	protein_codir	-0.16336655	0.67258965	0.78406813
ENSG0000027	AC005740.6	lncRNA	-0.16348674	0.75285749	0.84275201
ENSG0000020	SKIV2L	protein_codir	-0.16352136	0.28423448	0.4338043
ENSG0000016	ANAPC10	protein_codir	-0.16363064	0.16963699	0.29829845
ENSG0000019	MTOR	protein_codir	-0.16374083	0.14686219	0.26831152
ENSG0000014	MBTPS1	protein_codir	-0.16385227	0.12152393	0.23334893
ENSG0000028	CH17-264B6.6	lncRNA	-0.16391813	0.62567467	0.74756951
ENSG0000016	S100A11	protein_codir	-0.16402039	0.49957232	0.64498609
ENSG0000026	RP11-73M18.	lncRNA	-0.16410508	0.32394231	0.47623345
ENSG0000027	RP11-182L21.	lncRNA	-0.16413115	0.42511501	0.57707164
ENSG0000022	OSER1-DT	lncRNA	-0.16423065	0.46647205	0.61607144
ENSG0000016	MED30	protein_codir	-0.16425804	0.24709404	0.39236158
ENSG0000017	C12orf76	protein_codir	-0.16425844	0.13618348	0.25351403
ENSG0000015	RILPL2	protein_codir	-0.16427448	0.38153369	0.534949
ENSG0000019	ZNF777	protein_codir	-0.16436015	0.13523537	0.25222505
ENSG0000014	MINDY1	protein_codir	-0.16437694	0.22069463	0.36143407
ENSG0000017	PDE12	protein_codir	-0.16443781	0.32558733	0.47783711
ENSG0000014	ADCY10	protein_codir	-0.16457766	0.815028	0.8861026
ENSG0000010	TCFL5	protein_codir	-0.16460693	0.43513186	0.58651808
ENSG0000011	C10orf88	protein_codir	-0.16468873	0.19830448	0.33457621
ENSG0000027	TAGAP-AS1	lncRNA	-0.16496851	0.52926733	0.67069773
ENSG0000013	PSMC3IP	protein_codir	-0.16500995	0.41891997	0.57152727
ENSG0000026	ILF3-DT	lncRNA	-0.16511518	0.47179462	0.62056292
ENSG0000021	ALG3	protein_codir	-0.16515019	0.3083769	0.45997569
ENSG0000010	ANXA13	protein_codir	-0.16516445	0.88212634	0.93018477
ENSG0000012	CLPP	protein_codir	-0.16532959	0.1439057	0.26411558
ENSG0000003	OTC	protein_codir	-0.16535144	0.83251974	0.89915909
ENSG0000017	DPY19L1	protein_codir	-0.16543724	0.33515358	0.48800368
ENSG0000022	LINC01839	lncRNA	-0.16545471	0.91337652	0.94872281
ENSG0000019	ZNF585A	protein_codir	-0.16552647	0.39745883	0.55037666
ENSG0000016	VPS28	protein_codir	-0.1655388	0.25202794	0.39813611
ENSG0000013	CDK4	protein_codir	-0.16554084	0.11011722	0.21687376
ENSG0000013	CAMSAP1	protein_codir	-0.16557261	0.341642	0.49484253
ENSG0000005	MCUR1	protein_codir	-0.16559388	0.31590243	0.46763989
ENSG0000019	MRPL42	protein_codir	-0.16567858	0.08146693	0.17305927
ENSG0000017	FAM222B	protein_codir	-0.16571917	0.03071032	0.08289888
ENSG0000012	IKZF4	protein_codir	-0.16584643	0.55688848	0.69272516
ENSG0000028	RP11-357L2.2	lncRNA	-0.16588082	0.57946092	0.71131468
ENSG0000021	GANC	protein_codir	-0.16591716	0.19807748	0.33432535
ENSG0000005	F7	protein_codir	-0.1659371	0.72865889	0.82549461
ENSG0000017	SPTLC3	protein_codir	-0.16596675	0.52933043	0.67070862
ENSG0000015	GGPS1	protein_codir	-0.16622639	0.1666995	0.29463365
ENSG0000011	ANAPC15	protein_codir	-0.16623894	0.44826675	0.59913514
ENSG0000022	SMIM13	protein_codir	-0.16626701	0.27950226	0.42846177
ENSG0000018	ZBTB6	protein_codir	-0.16631848	0.51895833	0.66191762
ENSG0000012	LRP1	protein_codir	-0.16632315	0.58501728	0.71572366

ENSG0000028	RP11-9J18.1	protein_codir	-0.16643102	0.78126253	0.86317659
ENSG0000027	RP11-173P15.	lncRNA	-0.16644713	0.76294969	0.85042457
ENSG0000027	CTD-2649C14	lncRNA	-0.16647425	0.78648328	0.86658428
ENSG0000012	CISD1	protein_codir	-0.16648338	0.43947223	0.59046492
ENSG0000013	ARL8B	protein_codir	-0.16654939	0.21072464	0.35006925
ENSG0000018	LRCH3	protein_codir	-0.16654972	0.25318975	0.39949163
ENSG0000028	RP5-900K19.2	protein_codir	-0.16658369	0.77880842	0.86156875
ENSG0000027	GPR179	protein_codir	-0.16667915	0.77352196	0.85753699
ENSG0000018	PRELID2	protein_codir	-0.16673185	0.32829978	0.48069651
ENSG0000015	SPRR2G	protein_codir	-0.16679399	0.9435252	NA
ENSG0000018	SF3A3	protein_codir	-0.16680762	0.01628131	0.0507593
ENSG0000004	C12orf4	protein_codir	-0.16684527	0.30883404	0.4604407
ENSG0000022	RASAL2-AS1	lncRNA	-0.16696664	0.63694351	0.75656388
ENSG0000013	MTMR6	protein_codir	-0.16696681	0.30748979	0.45924645
ENSG0000021	PAM16	protein_codir	-0.16696992	0.40422507	0.55726948
ENSG0000010	SMAD7	protein_codir	-0.16697959	0.61089327	0.73619582
ENSG0000027	RP11-348P10.	lncRNA	-0.16698825	0.41829654	0.57097833
ENSG0000013	TXN	protein_codir	-0.16709116	0.42527039	0.57721511
ENSG0000018	PNRC2	protein_codir	-0.16714566	0.24539138	0.39052626
ENSG0000014	AMMECR1L	protein_codir	-0.16731593	0.277294	0.42574787
ENSG0000014	RPL7L1	protein_codir	-0.16742434	0.09303084	0.1910548
ENSG0000019	ZNF705A	protein_codir	-0.1674619	0.93390218	NA
ENSG0000022	MRLN	protein_codir	-0.1675775	0.91149139	NA
ENSG0000007	EXOC5	protein_codir	-0.16765805	0.34108224	0.49422036
ENSG0000011	PCGF1	protein_codir	-0.16772313	0.31340233	0.46513553
ENSG0000018	RXRA	protein_codir	-0.16793052	0.29656574	0.44706613
ENSG0000010	FKBP3	protein_codir	-0.16800433	0.14672278	0.26817601
ENSG0000006	ST3GAL6	protein_codir	-0.1681095	0.50474854	0.64939233
ENSG0000012	DDX54	protein_codir	-0.16813198	0.16221956	0.28852497
ENSG0000020	SDHAF1	protein_codir	-0.16813552	0.34832858	0.50163479
ENSG0000012	RP11-298J23.	protein_codir	-0.16815787	0.53884383	0.6781585
ENSG0000017	CYB561D1	protein_codir	-0.16818812	0.38596194	0.53913259
ENSG0000026	RP11-566K11.	lncRNA	-0.16821764	0.73041888	0.82653922
ENSG0000013	ETFBKMT	protein_codir	-0.16822263	0.39671638	0.54952382
ENSG0000025	CRYZL2P-SEC1	lncRNA	-0.16828975	0.61884723	0.74241239
ENSG0000018	MCRS1	protein_codir	-0.16843159	0.15447914	0.27837893
ENSG0000011	PAK1IP1	protein_codir	-0.16846783	0.30225749	0.45359152
ENSG0000018	EPGN	protein_codir	-0.16861065	0.85529772	0.91336696
ENSG0000017	SLC35G1	protein_codir	-0.16881127	0.48221189	0.62966587
ENSG0000012	SERPINB6	protein_codir	-0.16882445	0.1497815	0.27206136
ENSG0000004	H6PD	protein_codir	-0.16882824	0.32327016	0.47551376
ENSG0000015	CA10	protein_codir	-0.16887608	0.91037878	0.94664038
ENSG0000017	DCTPP1	protein_codir	-0.16887946	0.2627032	0.41015076
ENSG0000023	CLCA4-AS1	lncRNA	-0.16887971	0.89695039	0.93875596
ENSG0000010	ERH	protein_codir	-0.16889233	0.13779469	0.2557034
ENSG0000019	TOMM7	protein_codir	-0.16898509	0.28141855	0.43082104
ENSG0000012	ZC4H2	protein_codir	-0.1691037	0.15052612	0.27298302

ENSG0000010	KIF3B	protein_codir	-0.16915294	0.12672585	0.24066383
ENSG0000016	H2AZ1	protein_codir	-0.16929029	0.19757051	0.33375956
ENSG0000006	ISOC1	protein_codir	-0.16931925	0.29649666	0.4470352
ENSG0000018	TMEM222	protein_codir	-0.16941936	0.15976606	0.28541095
ENSG0000018	MRPL54	protein_codir	-0.16953765	0.26651054	0.41409885
ENSG0000012	OSGEPL1	protein_codir	-0.16959074	0.5012902	0.6461091
ENSG0000010	TLE5	protein_codir	-0.16963536	0.29482495	0.44519519
ENSG0000011	PDHX	protein_codir	-0.16971244	0.19327322	0.32854545
ENSG0000016	ZDHHC3	protein_codir	-0.16973674	0.0751342	0.16316577
ENSG0000005	FAM114A2	protein_codir	-0.16978751	0.16503576	0.29232911
ENSG0000014	MBD1	protein_codir	-0.16984874	0.06544047	0.14777743
ENSG0000016	ALKBH3	protein_codir	-0.16990564	0.22401548	0.3656153
ENSG0000008	L2HGDH	protein_codir	-0.16998807	0.31541997	0.4672262
ENSG0000008	HSD17B14	protein_codir	-0.17002091	0.63107862	0.75166638
ENSG0000009	CDC5L	protein_codir	-0.17006686	0.16263513	0.28907795
ENSG0000016	SHISA5	protein_codir	-0.17013617	0.04414763	0.10952833
ENSG0000020	AGPAT1	protein_codir	-0.17037442	0.11707399	0.22677492
ENSG0000007	TULP3	protein_codir	-0.17044473	0.41267819	0.56575031
ENSG0000000	FBXL3	protein_codir	-0.17048675	0.39939103	0.55263666
ENSG0000021	RPL17-C18orf	protein_codir	-0.17061184	0.19624107	0.33213912
ENSG0000028	LLfos-22E10.1	lncRNA	-0.1707036	0.90994117	NA
ENSG0000011	NOL10	protein_codir	-0.17075878	0.22000849	0.36086128
ENSG0000016	C4orf3	protein_codir	-0.17076706	0.35988235	0.51366598
ENSG0000010	LRRC59	protein_codir	-0.17079947	0.46782561	0.61728744
ENSG0000016	GTF2A1	protein_codir	-0.17110488	0.31848726	0.47048287
ENSG0000010	FTSJ3	protein_codir	-0.17121545	0.09614238	0.19582983
ENSG0000010	TGFB1	protein_codir	-0.17123356	0.38543395	0.53864782
ENSG0000016	ALDH1A1	protein_codir	-0.17124399	0.69873687	0.80315919
ENSG0000015	GEMIN6	protein_codir	-0.17127119	0.40028647	0.55352103
ENSG0000012	ZNF384	protein_codir	-0.17128958	0.13278181	0.24915492
ENSG0000022	RP11-429E11.	lncRNA	-0.17138744	0.91775355	NA
ENSG0000005	USP13	protein_codir	-0.17140138	0.38008477	0.5334862
ENSG0000012	MTHFD1L	protein_codir	-0.17141844	0.54139873	0.68019618
ENSG0000016	SPTSSA	protein_codir	-0.1714814	0.41793039	0.57065638
ENSG0000027	RP11-334C17.	lncRNA	-0.17158226	0.91695861	NA
ENSG0000016	HDGFL2	protein_codir	-0.17165213	0.19104247	0.32564933
ENSG0000011	PFDN1	protein_codir	-0.17220972	0.06077787	0.13934375
ENSG0000026	RP3-426I6.5	lncRNA	-0.17224153	0.90958824	0.94632476
ENSG0000020	EHMT2	protein_codir	-0.17228529	0.18664754	0.32007979
ENSG0000002	B4GALT7	protein_codir	-0.1724486	0.30136019	0.45251617
ENSG0000019	GET3	protein_codir	-0.17271735	0.15636452	0.28087852
ENSG0000007	SMC1A	protein_codir	-0.17283821	0.2907071	0.44049171
ENSG0000019	TTC37	protein_codir	-0.17292166	0.17893588	0.31003131
ENSG0000021	CRPPA	protein_codir	-0.17300932	0.54215073	0.68092416
ENSG0000018	PIGP	protein_codir	-0.17313964	0.36771348	0.5212876
ENSG0000013	SBF2	protein_codir	-0.17325465	0.43842766	0.58957944
ENSG0000016	USP41	protein_codir	-0.17334336	0.8315661	0.89848045

ENSG000001(	CLPTM1	protein_codir	-0.1733916	0.01072253	0.0368044
ENSG000001	ELMOD3	protein_codir	-0.17345093	0.3939097	0.54688281
ENSG000001(	NAPA	protein_codir	-0.17347591	0.18421521	0.31708987
ENSG000001(	NDUFAF5	protein_codir	-0.1736958	0.29250358	0.44258513
ENSG000001	MSTN	protein_codir	-0.17374721	0.76150055	0.84948732
ENSG000002	AC099850.1	lncRNA	-0.17383055	0.534786	0.67521568
ENSG000001	CYP4V2	protein_codir	-0.17390227	0.36793142	0.52148949
ENSG000001	UBAP1	protein_codir	-0.17406486	0.40500995	0.5578961
ENSG000002	RP11-342K6.2	lncRNA	-0.17418943	0.58184518	0.7131731
ENSG000000	GOLGA5	protein_codir	-0.17433592	0.33486589	0.48777467
ENSG000001	NDUFS7	protein_codir	-0.17449838	0.14662453	0.26807239
ENSG000001	TRIM4	protein_codir	-0.17454284	0.16836914	0.29676808
ENSG000001(	BABAM1	protein_codir	-0.17463451	0.13123228	0.24697708
ENSG000001	ZNF577	protein_codir	-0.17463988	0.46296051	0.61298257
ENSG000002	RP11-57H14.2	lncRNA	-0.17478923	0.82160114	0.89098997
ENSG000001	ZSWIM3	protein_codir	-0.17479024	0.47772848	0.62573437
ENSG000001	TUBA1A	protein_codir	-0.17497393	0.43907858	0.59013871
ENSG000001	TMEM267	protein_codir	-0.17505363	0.1815401	0.31352076
ENSG000001	CFAP126	protein_codir	-0.17506914	0.8344933	0.90028554
ENSG000001	MICALL2	protein_codir	-0.1752384	0.46961778	0.61883794
ENSG000001	AMFR	protein_codir	-0.17525587	0.17227151	0.30172093
ENSG000001	SLC35E1	protein_codir	-0.17538127	0.08670822	0.18132049
ENSG000001	ISCU	protein_codir	-0.17539044	0.17216215	0.30165219
ENSG000002	RP11-54O7.1	lncRNA	-0.17544266	0.8058471	0.87970819
ENSG000000	ARAF	protein_codir	-0.17551306	0.06858166	0.15267392
ENSG000000	C20orf194	protein_codir	-0.17554901	0.31027457	0.46205767
ENSG000001	FAM120B	protein_codir	-0.17555413	0.14059481	0.25985203
ENSG000001	ANGPTL2	protein_codir	-0.17569014	0.59017102	0.71988928
ENSG000002	XXbac-BPG18	lncRNA	-0.17569053	0.87034441	0.92310639
ENSG000000	MUL1	protein_codir	-0.17605806	0.22958835	0.37205058
ENSG000001	MAN2C1	protein_codir	-0.17608202	0.47253448	0.62112289
ENSG000001	COPS2	protein_codir	-0.17608371	0.37440063	0.52797639
ENSG000001	CWC22	protein_codir	-0.17611676	0.08524358	0.17897589
ENSG000001	MRPS30	protein_codir	-0.1761872	0.20114802	0.33806185
ENSG000001(	BUD31	protein_codir	-0.1762432	0.13963928	0.25844917
ENSG000002	POTEJ	protein_codir	-0.17648516	0.91118365	0.94705595
ENSG000001	CYHR1	protein_codir	-0.17650092	0.29089275	0.44063057
ENSG000001	KIAA1328	protein_codir	-0.17653659	0.28551137	0.43500794
ENSG000001	FKBP9	protein_codir	-0.17654354	0.51076313	0.65467338
ENSG000001	PSMB4	protein_codir	-0.17659455	0.14759393	0.26920323
ENSG000001	SSBP4	protein_codir	-0.17665978	0.22449439	0.36623789
ENSG000001	TBCA	protein_codir	-0.17674955	0.18618436	0.319464
ENSG000000	Aug-01	protein_codir	-0.17677456	0.1805203	0.31207177
ENSG000002	C17orf100	protein_codir	-0.17680851	0.38267709	0.53608987
ENSG000001	PEX10	protein_codir	-0.17681156	0.26623092	0.41375749
ENSG000001	CLTA	protein_codir	-0.17699627	0.10302506	0.20634896
ENSG000000	ZFHX4	protein_codir	-0.17715746	0.62793219	0.74921107

ENSG0000000	LAS1L	protein_codir	-0.17721705	0.16085781	0.28676775
ENSG0000020	BAG6	protein_codir	-0.17734963	0.10793701	0.21363097
ENSG0000012	COIL	protein_codir	-0.17751462	0.13652346	0.2539546
ENSG0000015	GPR180	protein_codir	-0.17753909	0.31571911	0.4674938
ENSG0000025	RNASE4	protein_codir	-0.177795	0.6630283	0.77702145
ENSG0000012	RWDD3	protein_codir	-0.17786749	0.36702585	0.52090098
ENSG0000013	HERC5	protein_codir	-0.1779107	0.51805233	0.66111931
ENSG0000026	RARA-AS1	lncRNA	-0.17800068	0.48376047	0.63114994
ENSG0000011	MFN2	protein_codir	-0.17804816	0.26562735	0.41319145
ENSG0000022	LINC01886	lncRNA	-0.17806912	0.92609335	NA
ENSG0000014	MOB3C	protein_codir	-0.17841025	0.20144484	0.33847832
ENSG0000021	GALT	protein_codir	-0.17853291	0.39143132	0.54434989
ENSG0000013	USP44	protein_codir	-0.17861178	0.59823958	0.72635242
ENSG0000001	MAP4K3	protein_codir	-0.17876646	0.42042353	0.57264513
ENSG0000014	LRP12	protein_codir	-0.17922183	0.48037299	0.62812583
ENSG0000010	SLC7A6OS	protein_codir	-0.17923148	0.16152028	0.28767025
ENSG0000017	PGAM1	protein_codir	-0.17932626	0.21218304	0.35174933
ENSG0000019	CTC-499B15.5	lncRNA	-0.17948077	0.75307704	0.8429238
ENSG0000010	PORCN	protein_codir	-0.17971382	0.05683118	0.13237283
ENSG0000016	VCP	protein_codir	-0.1797644	0.10922991	0.21560401
ENSG0000012	SEM1	protein_codir	-0.1797881	0.39276274	0.54578923
ENSG0000011	HEMK1	protein_codir	-0.17979665	0.43262265	0.58393382
ENSG0000011	MTR	protein_codir	-0.17990353	0.27163174	0.41962463
ENSG0000025	RP1-102E24.8	lncRNA	-0.17992746	0.6066878	0.7328865
ENSG0000028	AC006427.4	lncRNA	-0.1800484	0.91529094	NA
ENSG0000009	POLR2E	protein_codir	-0.18009474	0.14231669	0.26191277
ENSG0000028	RP11-67M24.	lncRNA	-0.18018366	0.91484072	NA
ENSG0000012	AAMP	protein_codir	-0.18020609	0.12217373	0.23428719
ENSG0000020	NFKBIL1	protein_codir	-0.1802692	0.2586684	0.40579469
ENSG0000013	MMP27	protein_codir	-0.18029809	0.89569212	0.93793662
ENSG0000015	RCAN1	protein_codir	-0.18041206	0.69345462	0.79881622
ENSG0000016	BSN	protein_codir	-0.18050092	0.49293007	0.63933921
ENSG0000028	RP11-379B18.	lncRNA	-0.1805088	0.78616283	0.86630031
ENSG0000009	WAC	protein_codir	-0.18057276	0.134945	0.25201914
ENSG0000009	TNRC6A	protein_codir	-0.18061097	0.18229778	0.31455392
ENSG0000016	GFM1	protein_codir	-0.18063672	0.24669753	0.39198317
ENSG0000010	ARFRP1	protein_codir	-0.18080678	0.23946053	0.38381262
ENSG0000017	P4HTM	protein_codir	-0.1810497	0.13380221	0.25049936
ENSG0000013	FCHSD2	protein_codir	-0.18117282	0.25226067	0.39842273
ENSG0000012	MPHOSPH10	protein_codir	-0.18120792	0.07178708	0.15792912
ENSG0000019	ZNF461	protein_codir	-0.18133374	0.23260456	0.37561612
ENSG0000001	PSMC4	protein_codir	-0.18133791	0.20580505	0.34383835
ENSG0000010	ZC3H14	protein_codir	-0.18135608	0.14771191	0.26936505
ENSG0000018	C1QTNF12	protein_codir	-0.18150081	0.5967801	0.72500842
ENSG0000009	ATP5F1D	protein_codir	-0.18157497	0.25744748	0.40422371
ENSG0000018	CCIN	protein_codir	-0.18177888	0.66432715	0.77785051
ENSG0000026	SMIM17	protein_codir	-0.18190503	0.58467383	0.71549363

ENSG0000027	CTD-2002J20.	lncRNA	-0.18198228	0.87926671	0.92833894
ENSG0000014	PYCR2	protein_codir	-0.18204452	0.06964955	0.15445368
ENSG0000014	BBS4	protein_codir	-0.18206419	0.06773367	0.1515546
ENSG0000018	ATP6AP2	protein_codir	-0.18212682	0.26060599	0.40788449
ENSG0000019	WDR5	protein_codir	-0.1821578	0.04887153	0.11838553
ENSG0000013	TNS3	protein_codir	-0.18216169	0.54047855	0.67947281
ENSG0000010	NFKBIB	protein_codir	-0.18228624	0.31550444	0.4673031
ENSG0000025	HTT-AS	lncRNA	-0.18240308	0.87038756	0.92310639
ENSG0000024	RP11-677M14	lncRNA	-0.18240847	0.45042009	0.60114052
ENSG0000025	CTD-2270L9.2	lncRNA	-0.18243194	0.83748311	0.90240251
ENSG0000010	RTCB	protein_codir	-0.1827853	0.13672425	0.25424653
ENSG0000012	STAU1	protein_codir	-0.18280334	0.25347999	0.39983161
ENSG0000017	FOXA3	protein_codir	-0.18288047	0.79902584	0.87509789
ENSG0000011	COQ6	protein_codir	-0.18299919	0.12897703	0.24376686
ENSG0000016	SNUPN	protein_codir	-0.18300183	0.23538672	0.37890036
ENSG0000007	RDH11	protein_codir	-0.1830052	0.02581487	0.07237911
ENSG0000013	CCDC115	protein_codir	-0.18305833	0.16320579	0.28986842
ENSG0000011	PSMD14	protein_codir	-0.18306762	0.27353162	0.42154183
ENSG0000023	RP11-411K7.1	lncRNA	-0.18312363	0.784652	0.86530766
ENSG0000016	PDIA3	protein_codir	-0.18338097	0.34292083	0.49612243
ENSG0000027	RP11-710E1.2	lncRNA	-0.18350237	0.87643937	0.92666445
ENSG0000028	CTA-150C2.23	lncRNA	-0.18367444	0.92511112	NA
ENSG0000023	RP11-196G18	lncRNA	-0.18385564	0.8620363	0.9177598
ENSG0000027	RP1-136B1.1	lncRNA	-0.1839737	0.8368719	0.90199024
ENSG0000017	UBE2N	protein_codir	-0.18400672	0.19243852	0.32738847
ENSG0000027	RP5-965G21.3	lncRNA	-0.18421866	0.6859566	0.79378755
ENSG0000015	GATAD1	protein_codir	-0.18430879	0.18727299	0.32083364
ENSG0000006	HDAC4	protein_codir	-0.18431511	0.27711367	0.4255314
ENSG0000018	PAQR7	protein_codir	-0.18433033	0.06291359	0.14327781
ENSG0000002	RNH1	protein_codir	-0.18433216	0.28885297	0.43843305
ENSG0000010	RBM28	protein_codir	-0.18473288	0.17358989	0.30348583
ENSG0000017	TMEM31	protein_codir	-0.18479202	0.85218977	0.9120283
ENSG0000016	FRS2	protein_codir	-0.18483567	0.38100562	0.53437137
ENSG0000016	CEP44	protein_codir	-0.18488054	0.24628252	0.39156823
ENSG0000008	GMCL1	protein_codir	-0.18497937	0.17218244	0.30165219
ENSG0000027	RP11-121C6.5	lncRNA	-0.18499368	0.9028637	0.94233819
ENSG0000028	EEF1AKMT4	protein_codir	-0.18514435	0.53987498	0.67893034
ENSG0000028	RP11-793J2.1	lncRNA	-0.18522078	0.74472667	0.83693496
ENSG0000012	ZCCHC17	protein_codir	-0.18529836	0.07076171	0.15630506
ENSG0000026	AC007292.6	lncRNA	-0.18547034	0.79692874	0.87382745
ENSG0000017	C14orf119	protein_codir	-0.18566227	0.01389994	0.0449168
ENSG0000011	CNN3	protein_codir	-0.18584739	0.53545603	0.67580403
ENSG0000008	MAVS	protein_codir	-0.18589971	0.03801539	0.09748261
ENSG0000026	RP11-1055B8.	lncRNA	-0.186092	0.71241013	0.81359387
ENSG0000015	SREK1IP1	protein_codir	-0.18613941	0.10795402	0.21364934
ENSG0000015	MRPL39	protein_codir	-0.18618791	0.22453227	0.36623789
ENSG0000017	TCAIM	protein_codir	-0.18623634	0.26074141	0.40801758

ENSG0000025	TOLLIP-AS1	lncRNA	-0.18624834	0.55460328	0.69078571
ENSG0000012	TTI2	protein_codir	-0.18630642	0.14382833	0.26400062
ENSG0000012	DNAJC8	protein_codir	-0.18633469	0.08422042	0.17721835
ENSG0000016	DTWD2	protein_codir	-0.18637876	0.37084587	0.52427512
ENSG0000007	TSG101	protein_codir	-0.1864167	0.11504373	0.22387853
ENSG0000012	ECD	protein_codir	-0.18649791	0.14088545	0.26016112
ENSG0000006	AP3D1	protein_codir	-0.18654825	0.07723703	0.16633815
ENSG0000015	CCDC175	protein_codir	-0.18660692	0.88821948	NA
ENSG0000017	PA2G4	protein_codir	-0.18669155	0.14812308	0.26995443
ENSG0000016	ULK4	protein_codir	-0.18673769	0.31802488	0.47000092
ENSG0000024	RP11-314A20	lncRNA	-0.1868786	0.92493152	0.9557977
ENSG0000017	TRAPPC12	protein_codir	-0.18691407	0.08000246	0.17063072
ENSG0000022	RP11-57H12.3	lncRNA	-0.18699851	0.61052684	0.73587737
ENSG0000005	PHF21B	protein_codir	-0.18714725	0.71619574	0.81629705
ENSG0000016	TMEM150A	protein_codir	-0.18729029	0.13945364	0.25822671
ENSG0000013	HSD17B3	protein_codir	-0.1873408	0.68146939	0.79058235
ENSG0000012	DNPEP	protein_codir	-0.18740586	0.14815816	0.26998488
ENSG0000016	ARF4	protein_codir	-0.187768	0.37706284	0.53067391
ENSG0000016	SRR	protein_codir	-0.18777873	0.26178823	0.40922468
ENSG0000026	RP11-13N13.5	lncRNA	-0.18779315	0.87489879	0.92577981
ENSG0000027	RP11-218C14.	lncRNA	-0.18780181	0.71758448	0.81753527
ENSG0000020	ZNF616	protein_codir	-0.1878516	0.3372623	0.49011811
ENSG0000010	RANGRF	protein_codir	-0.18788902	0.17505652	0.3051667
ENSG0000020	TRIM39	protein_codir	-0.18789106	0.14378152	0.26395784
ENSG0000013	ECHDC3	protein_codir	-0.18789365	0.55285778	0.68937374
ENSG0000013	PHF10	protein_codir	-0.18790301	0.31644632	0.46816902
ENSG0000012	PCSK2	protein_codir	-0.18793499	0.76093803	0.84900457
ENSG0000012	PSMF1	protein_codir	-0.18808065	0.06899512	0.15329782
ENSG0000010	ODAD1	protein_codir	-0.18808234	0.66792778	0.78061038
ENSG0000027	CTD-2201E18.	lncRNA	-0.18809582	0.76329522	0.85066225
ENSG0000028	RP11-302L19.	lncRNA	-0.18824903	0.85582874	0.91382804
ENSG0000027	AC011330.13	lncRNA	-0.18824922	0.59046808	0.72005368
ENSG0000017	GBA	protein_codir	-0.18839299	0.16824501	0.29666287
ENSG0000000	VTA1	protein_codir	-0.1885604	0.14068022	0.2599403
ENSG0000010	USP14	protein_codir	-0.18862911	0.08897702	0.1847012
ENSG0000011	FARSB	protein_codir	-0.18869651	0.19437512	0.3298384
ENSG0000014	ZFP14	protein_codir	-0.18883707	0.48713414	0.63435384
ENSG0000011	FXR1	protein_codir	-0.18884205	0.08291411	0.17506977
ENSG0000011	FASTKD2	protein_codir	-0.1889415	0.09859499	0.19961837
ENSG0000024	ARPIN	protein_codir	-0.18917393	0.18828075	0.32214052
ENSG0000016	LRRC58	protein_codir	-0.18922732	0.18713421	0.32063566
ENSG0000008	LYRM2	protein_codir	-0.18933079	0.17194628	0.30130417
ENSG0000013	FAM189A2	protein_codir	-0.18946625	0.64084789	0.75916406
ENSG0000012	TMEM115	protein_codir	-0.18951193	0.1002642	0.20212025
ENSG0000007	RABL2B	protein_codir	-0.18962151	0.11103175	0.21825471
ENSG0000014	RGR	protein_codir	-0.18969648	0.87924448	0.92833894
ENSG0000011	PRRX1	protein_codir	-0.18974926	0.49905684	0.64465203

ENSG0000019	PIK3R4	protein_codir	-0.18976415	0.23761882	0.38170478
ENSG0000018	NDUFA6	protein_codir	-0.18986515	0.04304478	0.10740779
ENSG0000017	HCFC1	protein_codir	-0.18987844	0.1605137	0.2863317
ENSG0000011	NT5C1A	protein_codir	-0.18991289	0.76241891	0.84997014
ENSG0000026	MRPL12	protein_codir	-0.19016612	0.27224717	0.42018778
ENSG0000019	MAFG	protein_codir	-0.19028244	0.29686898	0.44740116
ENSG0000018	LYPD6	protein_codir	-0.19037277	0.70561108	0.80857063
ENSG0000023	RP3-428L16.1	lncRNA	-0.19055253	0.89972988	0.94009758
ENSG0000018	NR2C2AP	protein_codir	-0.19066578	0.15637954	0.28088723
ENSG0000011	RPF1	protein_codir	-0.19067582	0.01063063	0.03653223
ENSG0000011	DGUOK	protein_codir	-0.19093011	0.22373703	0.36521577
ENSG0000027	RP5-894D12.5	lncRNA	-0.19095348	0.85483031	0.91319595
ENSG0000012	CYREN	protein_codir	-0.19103652	0.26749082	0.41531825
ENSG0000025	RP11-798K3.2	lncRNA	-0.19109904	0.76945123	0.85477388
ENSG0000024	CPEB2-DT	lncRNA	-0.19116166	0.8727467	0.92449428
ENSG0000012	MSTO1	protein_codir	-0.19150225	0.35145839	0.50500888
ENSG0000013	STAM	protein_codir	-0.19157168	0.11863123	0.22906516
ENSG0000018	NOC4L	protein_codir	-0.19172067	0.21304629	0.35273579
ENSG0000018	LINC02724	lncRNA	-0.19174847	0.64845229	0.76470912
ENSG0000016	OTUD3	protein_codir	-0.19182491	0.24292777	0.38790917
ENSG0000028	RP11-934B9.8	lncRNA	-0.19184692	0.32473818	0.47716489
ENSG0000024	RP11-61A14.4	lncRNA	-0.19189012	0.84397791	0.90671243
ENSG0000012	PDCD2L	protein_codir	-0.19203133	0.2605577	0.40783201
ENSG0000019	FAM217B	protein_codir	-0.19210342	0.40671508	0.55977031
ENSG0000028	RP11-460N20	lncRNA	-0.19220524	0.90757915	NA
ENSG0000023	AC005540.3	lncRNA	-0.19220996	0.62189804	0.74477839
ENSG0000012	SUMF2	protein_codir	-0.19226086	0.01815156	0.05500836
ENSG0000024	UBA6-AS1	lncRNA	-0.19244313	0.27254961	0.42045466
ENSG0000014	THNSL2	protein_codir	-0.19246251	0.59562099	0.72399753
ENSG0000022	RP13-131K19.	lncRNA	-0.19248041	0.67554975	0.78630194
ENSG0000016	RHO	protein_codir	-0.19248214	0.88792222	0.93348541
ENSG0000028	RP11-14N9.3	lncRNA	-0.19261897	0.8960052	NA
ENSG0000009	PGC	protein_codir	-0.19285099	0.85443204	0.91311329
ENSG0000028	RP5-1087E8.6	protein_codir	-0.19292559	0.52271506	0.66498042
ENSG0000017	RPP25	protein_codir	-0.19303421	0.26527826	0.4128577
ENSG0000014	VPS51	protein_codir	-0.19304352	0.19350701	0.32884178
ENSG0000010	SSBP1	protein_codir	-0.19311241	0.06702634	0.15037507
ENSG0000014	NSDHL	protein_codir	-0.19316607	0.18033243	0.31187579
ENSG0000018	UBOX5	protein_codir	-0.19316979	0.13233169	0.24861473
ENSG0000017	RELA	protein_codir	-0.19320131	0.23804813	0.38217213
ENSG0000027	ZNHIT3	protein_codir	-0.19323341	0.07501874	0.1629329
ENSG0000008	EXD2	protein_codir	-0.19328542	0.12819295	0.24258087
ENSG0000027	RP4-605O3.4	lncRNA	-0.19342493	0.6189128	0.74242655
ENSG0000027	AP000350.5	lncRNA	-0.19358971	0.78556557	0.86598767
ENSG0000026	MIR4453HG	lncRNA	-0.19360685	0.40691227	0.55993009
ENSG0000026	CTC-513N18.7	lncRNA	-0.19388541	0.84841284	0.90970778
ENSG0000025	RBM15B	protein_codir	-0.19388593	0.20750891	0.34603612

ENSG0000018	UBE2G2	protein_codir	-0.1939437	0.17449019	0.3045639
ENSG0000010	MICALL1	protein_codir	-0.19394417	0.3543618	0.50796517
ENSG0000006	GNB5	protein_codir	-0.19418039	0.19946517	0.33596951
ENSG0000023	ST3GAL5-AS1	lncRNA	-0.19421633	0.69043054	0.79692806
ENSG0000018	TRMT12	protein_codir	-0.19428378	0.05260463	0.12502709
ENSG0000013	MED18	protein_codir	-0.19435275	0.29779448	0.44862153
ENSG0000011	NDUFA8	protein_codir	-0.19451977	0.08346589	0.17596581
ENSG0000010	GOSR2	protein_codir	-0.19471107	0.03132113	0.08409543
ENSG0000013	CUL4A	protein_codir	-0.19477309	0.04579649	0.11256555
ENSG0000019	ZNF836	protein_codir	-0.19478208	0.38653787	0.5395439
ENSG0000001	XYLT2	protein_codir	-0.19485727	0.11658979	0.22616427
ENSG0000017	TSEN34	protein_codir	-0.19486297	0.19757309	0.33375956
ENSG0000016	ZNF513	protein_codir	-0.19486606	0.37086858	0.52428039
ENSG0000018	H2AC6	protein_codir	-0.19487148	0.50964066	0.65382856
ENSG0000012	MXD4	protein_codir	-0.19489317	0.15032396	0.27276403
ENSG0000017	THBD	protein_codir	-0.19502077	0.59960133	0.72726652
ENSG0000014	RAB13	protein_codir	-0.19507852	0.18862159	0.32254386
ENSG0000027	RP11-214K3.1	lncRNA	-0.19510822	0.84341133	0.90639867
ENSG0000009	DDTL	protein_codir	-0.19513581	0.73431595	0.82965136
ENSG0000018	SMIM29	protein_codir	-0.19514548	0.10776682	0.21335528
ENSG0000020	RP11-1148L6.	lncRNA	-0.19530975	0.71760771	0.81753527
ENSG0000012	TRAP1	protein_codir	-0.1953727	0.11190809	0.21946191
ENSG0000023	NPIPB7	protein_codir	-0.19538629	0.7893709	0.86865709
ENSG0000013	RSL24D1	protein_codir	-0.19538891	0.15255679	0.27574232
ENSG0000017	AC093323.3	protein_codir	-0.19539219	0.06036356	0.13864796
ENSG0000027	CTB-161C1.1	lncRNA	-0.1954989	0.90719713	NA
ENSG0000014	SDHC	protein_codir	-0.19558628	0.12760799	0.24183701
ENSG0000017	ETV4	protein_codir	-0.19559773	0.55103434	0.68801449
ENSG0000010	CDK16	protein_codir	-0.19577786	0.06290327	0.14327781
ENSG0000007	UBE2K	protein_codir	-0.1958909	0.17618752	0.30651924
ENSG0000023	ZNF593OS	protein_codir	-0.19591737	0.83999527	0.904298
ENSG0000010	ZFAND1	protein_codir	-0.19599442	0.2969837	0.44750081
ENSG0000016	SCG5	protein_codir	-0.19602017	0.52436475	0.66631346
ENSG0000020	LINC00501	lncRNA	-0.19609545	0.91882617	NA
ENSG0000014	PFDN2	protein_codir	-0.19612684	0.28141412	0.43082104
ENSG0000022	OST4	protein_codir	-0.19613422	0.12322885	0.23578669
ENSG0000007	HACD3	protein_codir	-0.19623886	0.24664747	0.39194541
ENSG0000026	SRSF8	protein_codir	-0.19625589	0.10283877	0.20611036
ENSG0000019	S100A5	protein_codir	-0.19631979	0.8105224	0.88306689
ENSG0000000	CD99	protein_codir	-0.19635367	0.22467912	0.3663795
ENSG0000019	DZIP3	protein_codir	-0.19635964	0.08442536	0.17759547
ENSG0000017	HTR1A	protein_codir	-0.19643948	0.93369812	NA
ENSG0000011	SLC35F5	protein_codir	-0.1965045	0.26435117	0.41180933
ENSG0000015	CEP112	protein_codir	-0.19653474	0.30763774	0.45931966
ENSG0000017	WDR73	protein_codir	-0.19656031	0.27685097	0.42521402
ENSG0000018	C2orf76	protein_codir	-0.19672405	0.1956414	0.3314085
ENSG0000028	RP4-541C22.8	protein_codir	-0.19673701	0.43046313	0.58195749

ENSG0000012	EEF1AKMT3	protein_codir	-0.19681672	0.29872104	0.44967708
ENSG0000015	GDPD5	protein_codir	-0.19692282	0.35246084	0.50603083
ENSG0000022	AC090587.4	lncRNA	-0.19702779	0.58336553	0.71437876
ENSG0000027	RP5-1042K10.	lncRNA	-0.19712854	0.86306231	0.9184981
ENSG0000026	RP11-552F3.9	lncRNA	-0.19715913	0.5297179	0.67104565
ENSG0000014	DYM	protein_codir	-0.19726533	0.26389155	0.41139496
ENSG0000013	CAPN7	protein_codir	-0.19731587	0.1534048	0.27691267
ENSG0000010	PPM1A	protein_codir	-0.19733629	0.33326454	0.48604744
ENSG0000009	POU4F3	protein_codir	-0.1973421	0.71373521	0.81440001
ENSG0000022	AC015987.1	lncRNA	-0.19745962	0.74714643	0.83852842
ENSG0000018	MRPL40	protein_codir	-0.19752076	0.10459477	0.2086358
ENSG0000026	LINC01003	lncRNA	-0.19757537	0.4937928	0.64010262
ENSG0000018	ZP3	protein_codir	-0.1976513	0.6620303	0.77598357
ENSG0000008	AK6	protein_codir	-0.19767473	0.19396649	0.32941363
ENSG0000027	AATF	protein_codir	-0.19767602	0.0390061	0.09951316
ENSG0000010	EDF1	protein_codir	-0.19768573	0.12617176	0.23997135
ENSG0000028	RP11-83M8.1	lncRNA	-0.19787169	0.68904813	0.79587222
ENSG0000028	KB-1042C11.5	lncRNA	-0.19811577	0.63033165	0.75102532
ENSG0000023	RP11-399E6.1	lncRNA	-0.19813699	0.91995378	0.9526516
ENSG0000017	MRPS22	protein_codir	-0.19817069	0.09413751	0.19284121
ENSG0000018	SPATA21	protein_codir	-0.198242	0.79269715	0.87106465
ENSG0000003	GABARAPL2	protein_codir	-0.19826739	0.1396243	0.25843876
ENSG0000017	BET1L	protein_codir	-0.19827262	0.013589	0.04413549
ENSG0000013	MAP3K7	protein_codir	-0.19834154	0.06688694	0.1501721
ENSG0000018	DYNLT2	protein_codir	-0.19842242	0.58290191	0.7140529
ENSG0000022	DNAJC9-AS1	lncRNA	-0.19844647	0.57219877	0.70531434
ENSG0000021	CDC14C	protein_codir	-0.19846465	0.87340652	NA
ENSG0000026	CTC-459F4.1	lncRNA	-0.19854365	0.55096408	0.68795786
ENSG0000018	ZFP69	protein_codir	-0.19856552	0.26568038	0.41320412
ENSG0000027	TADA2A	protein_codir	-0.19861652	0.20044305	0.33720533
ENSG0000026	EEF1E1-BLOC1	protein_codir	-0.19862673	0.93261661	0.96036153
ENSG0000013	AMHR2	protein_codir	-0.1986302	0.86408309	0.91911928
ENSG0000020	FKBPL	protein_codir	-0.19871057	0.23388392	0.37719689
ENSG0000002	RABEP1	protein_codir	-0.1987153	0.13713901	0.25485663
ENSG0000010	CORO2B	protein_codir	-0.19880587	0.52851398	0.66995072
ENSG0000021	CTC-297N7.7	lncRNA	-0.19888801	0.88510932	0.9318716
ENSG0000017	SART1	protein_codir	-0.19893804	0.09588145	0.19541184
ENSG0000019	RABL6	protein_codir	-0.19896244	0.10459739	0.2086358
ENSG0000023	AC023481.1	lncRNA	-0.1990919	0.90383053	NA
ENSG0000014	RAB40B	protein_codir	-0.1991198	0.15431724	0.2781259
ENSG0000024	ACAD11	protein_codir	-0.19914625	0.48667481	0.63393502
ENSG0000014	PEX14	protein_codir	-0.1991991	0.0261507	0.0731203
ENSG0000023	UBE2L5	protein_codir	-0.19922485	0.78235315	0.86392907
ENSG0000025	LINC01018	lncRNA	-0.19929141	0.67062892	0.782648
ENSG0000011	COX7A2L	protein_codir	-0.19936732	0.06607045	0.14880935
ENSG0000016	ZNF507	protein_codir	-0.1994163	0.26277897	0.41015076
ENSG0000019	MIER1	protein_codir	-0.1994499	0.19063977	0.32526651

ENSG0000013	DMGDH	protein_codir	-0.19945294	0.55270599	0.68934673
ENSG0000018	TSPYL1	protein_codir	-0.19950726	0.24398824	0.38911429
ENSG0000028	PLAC4	lncRNA	-0.19956204	0.91793883	NA
ENSG0000026	RP11-274H2.5	lncRNA	-0.19969229	0.66604493	0.77927283
ENSG0000000	ANKIB1	protein_codir	-0.19975937	0.1719179	0.30128694
ENSG0000017	C16orf91	protein_codir	-0.20019968	0.15811804	0.28318119
ENSG0000015	TSEN2	protein_codir	-0.20027523	0.27582357	0.42396611
ENSG0000017	CCDC184	protein_codir	-0.20027871	0.63378124	0.75394253
ENSG0000011	KMT5B	protein_codir	-0.20043229	0.09507686	0.1942181
ENSG0000013	NAA30	protein_codir	-0.20046919	0.22245226	0.36364414
ENSG0000017	ZNF77	protein_codir	-0.20058668	0.39089483	0.54382286
ENSG0000014	SMAD4	protein_codir	-0.2006188	0.19762891	0.33383343
ENSG0000014	TIAM2	protein_codir	-0.20066242	0.55243742	0.6891931
ENSG0000015	CACNA2D1	protein_codir	-0.20080341	0.46946582	0.61872625
ENSG0000008	DIMT1	protein_codir	-0.20082491	0.30278653	0.45416336
ENSG0000013	EMC7	protein_codir	-0.20082928	0.10060175	0.20259718
ENSG0000020	ARRDC1-AS1	lncRNA	-0.20088286	0.36551827	0.51934846
ENSG0000008	DZANK1	protein_codir	-0.20095829	0.29520076	0.44556779
ENSG0000018	CCDC30	protein_codir	-0.201239	0.24938188	0.39522192
ENSG0000005	HOMER3	protein_codir	-0.20127649	0.31851201	0.47049426
ENSG0000014	UBR3	protein_codir	-0.20130288	0.3247158	0.47715739
ENSG0000011	EIF2B1	protein_codir	-0.20133585	0.01511482	0.04796866
ENSG0000006	CA11	protein_codir	-0.20145416	0.50423251	0.64894002
ENSG0000016	GOLM2	protein_codir	-0.20146835	0.33694693	0.48992921
ENSG0000014	TBX6	protein_codir	-0.20149442	0.55892599	0.69444605
ENSG0000005	ATG5	protein_codir	-0.20163906	0.1223148	0.23447631
ENSG0000001	RALBP1	protein_codir	-0.2016658	0.03149663	0.08445989
ENSG0000025	LINC01618	lncRNA	-0.20178619	0.89234645	0.9358524
ENSG0000013	GSTM5	protein_codir	-0.20185451	0.70899667	0.8110034
ENSG0000017	GXYLT2	protein_codir	-0.20197889	0.5890624	0.71901642
ENSG0000010	ESRP2	protein_codir	-0.2019897	0.65077213	0.76683576
ENSG0000019	ZNF569	protein_codir	-0.20200546	0.26391156	0.41140295
ENSG0000026	ZNF793-AS1	lncRNA	-0.20209952	0.5604672	0.6955787
ENSG0000027	RP11-212P7.2	lncRNA	-0.20211993	0.41292166	0.56599981
ENSG0000012	NPPB	protein_codir	-0.20212607	0.93068307	NA
ENSG0000024	NFS1	protein_codir	-0.20213363	0.13714461	0.25485663
ENSG0000028	RP11-422P24.	lncRNA	-0.20222709	0.59513156	0.72364225
ENSG0000013	DCAF5	protein_codir	-0.20223617	0.0132922	0.0433653
ENSG0000021	LINC02449	lncRNA	-0.20226583	0.54847988	0.68597239
ENSG0000024	DNM1P35	lncRNA	-0.20234702	0.64547038	0.76301698
ENSG0000011	MYL12B	protein_codir	-0.20246129	0.2414587	0.38612142
ENSG0000018	BBS12	protein_codir	-0.20274094	0.3440335	0.4971558
ENSG0000018	UBE2F	protein_codir	-0.20278781	0.08331064	0.17574581
ENSG0000016	EVA1C	protein_codir	-0.20279084	0.47715022	0.62524381
ENSG0000013	WBP2	protein_codir	-0.20284022	0.09093772	0.18763633
ENSG0000023	GSN-AS1	lncRNA	-0.20287281	0.85362676	0.91285717
ENSG0000023	MAP4K3-DT	lncRNA	-0.20322055	0.21964953	0.36044918

ENSG0000018	KRT16	protein_codir	-0.20325846	0.78804592	0.86764842
ENSG0000010	ARHGAP5	protein_codir	-0.20330633	0.38110728	0.53445966
ENSG0000021	VSTM5	protein_codir	-0.20332876	0.62737443	0.7487173
ENSG0000010	FAM32A	protein_codir	-0.2033895	0.01284927	0.04223454
ENSG0000019	PHF2	protein_codir	-0.2035375	0.1994801	0.33597417
ENSG0000025	LINC00930	lncRNA	-0.20371905	0.71913492	0.81863864
ENSG0000020	ZDBF2	protein_codir	-0.20376683	0.41479143	0.56788635
ENSG0000016	TMEM135	protein_codir	-0.20379711	0.32774767	0.48018197
ENSG0000019	TCAF1	protein_codir	-0.20384116	0.20181616	0.33887545
ENSG0000017	WSCD1	protein_codir	-0.2038558	0.49918509	0.64476154
ENSG0000019	COL4A6	protein_codir	-0.20400249	0.74290375	0.8358386
ENSG0000016	RNF181	protein_codir	-0.20405744	0.13729366	0.25506497
ENSG0000017	SH3PXD2B	protein_codir	-0.2040771	0.41662354	0.56937863
ENSG0000000	DVL2	protein_codir	-0.20426355	0.15350168	0.27705132
ENSG0000007	SRI	protein_codir	-0.2043032	0.16212511	0.28844754
ENSG0000026	U91319.1	lncRNA	-0.20434711	0.94994559	NA
ENSG0000027	NFYC-AS1	lncRNA	-0.20436241	0.63812585	0.75738197
ENSG0000019	MYO6	protein_codir	-0.20437405	0.08829272	0.18361908
ENSG0000010	TSC2	protein_codir	-0.20460681	0.06382786	0.1449655
ENSG0000018	ZBTB20	protein_codir	-0.20462272	0.26565042	0.41320407
ENSG0000017	TMEM167A	protein_codir	-0.2046598	0.24447472	0.38950115
ENSG0000028	RP13-46H24.3	lncRNA	-0.2047826	0.73526588	0.83015344
ENSG0000013	TET1	protein_codir	-0.20485847	0.48396304	0.63135553
ENSG0000013	FUT5	protein_codir	-0.20487815	0.90524754	NA
ENSG0000013	NEK1	protein_codir	-0.20493363	0.12410176	0.23698082
ENSG0000009	ZC3HC1	protein_codir	-0.20498996	0.11519587	0.22407987
ENSG0000013	LARP1B	protein_codir	-0.20510588	0.34759163	0.50078143
ENSG0000018	OGFOD3	protein_codir	-0.20517427	0.01506405	0.04786255
ENSG0000016	NDUFS4	protein_codir	-0.20522034	0.36127204	0.5151463
ENSG0000016	FADD	protein_codir	-0.20525865	0.17375894	0.30369057
ENSG0000018	MT1X	protein_codir	-0.20529406	0.78182211	0.86361879
ENSG0000016	C4orf36	protein_codir	-0.20529718	0.58819702	0.71837284
ENSG0000001	RNF216	protein_codir	-0.2053247	0.16856371	0.29697836
ENSG0000012	SEPTIN7	protein_codir	-0.20534698	0.12918653	0.24404733
ENSG0000014	GPN2	protein_codir	-0.20535526	0.03078302	0.08301376
ENSG0000019	ZNF568	protein_codir	-0.20547559	0.22278021	0.36407251
ENSG0000016	METTL15	protein_codir	-0.20547734	0.05794045	0.13427396
ENSG0000014	C1orf35	protein_codir	-0.20549702	0.31569845	0.46748828
ENSG0000016	TRUB1	protein_codir	-0.20568791	0.32487738	0.4772171
ENSG0000016	NAXE	protein_codir	-0.20571578	0.13734402	0.25508704
ENSG0000013	CDC73	protein_codir	-0.20597769	0.305793	0.45740605
ENSG0000021	ARL16	protein_codir	-0.20607819	0.40515313	0.55803767
ENSG0000009	SLC9A1	protein_codir	-0.20612169	0.0379062	0.09727007
ENSG0000022	AP000320.6	lncRNA	-0.20612844	0.79365138	0.87182395
ENSG0000023	RP11-342M3.	lncRNA	-0.20616275	0.83664964	0.90182107
ENSG0000025	RP11-531A24	lncRNA	-0.20619033	0.50579847	0.6503796
ENSG0000027	XXbac-B444P2	lncRNA	-0.20622966	0.85279306	0.91242085

ENSG0000010	SNRPD3	protein_codir	-0.20624923	0.16344146	0.29017503
ENSG0000016	SF3B5	protein_codir	-0.20635325	0.07469559	0.16244819
ENSG0000013	APTX	protein_codir	-0.20640613	0.02360208	0.06740662
ENSG0000019	SHPK	protein_codir	-0.20649421	0.16981101	0.29849042
ENSG0000014	ZNF614	protein_codir	-0.20668446	0.26833366	0.41618237
ENSG0000011	PNO1	protein_codir	-0.20677063	0.36961121	0.52307485
ENSG0000024	PI4KA	protein_codir	-0.20688523	0.12719386	0.24130052
ENSG0000025	BORCS8	protein_codir	-0.20689383	0.29018371	0.43987916
ENSG0000010	GAR1	protein_codir	-0.20692967	0.16092633	0.28683813
ENSG0000011	CNRIP1	protein_codir	-0.20694792	0.22336283	0.36487352
ENSG0000007	GNB1	protein_codir	-0.20696403	0.19491661	0.33046447
ENSG0000011	TMEM14C	protein_codir	-0.20696433	0.05769628	0.13381644
ENSG0000026	HSD17B1-AS1	lncRNA	-0.20698494	0.15735519	0.28223588
ENSG0000023	AP000695.6	lncRNA	-0.20703165	0.65660911	0.77176146
ENSG0000024	CTB-46B19.2	lncRNA	-0.20707319	0.85686325	0.9145084
ENSG0000014	FRA10AC1	protein_codir	-0.20716886	0.32374346	0.47605784
ENSG0000013	CD164	protein_codir	-0.20717151	0.17476327	0.30486714
ENSG0000010	DCAF11	protein_codir	-0.20721137	0.07362011	0.16083433
ENSG0000016	CNBP	protein_codir	-0.20726154	0.16922481	0.29776323
ENSG0000023	U73166.2	lncRNA	-0.20745712	0.5759357	0.70847299
ENSG0000013	EFR3A	protein_codir	-0.20766068	0.22783418	0.3701853
ENSG0000026	FMC1-LUC7L2	protein_codir	-0.20801957	0.2484723	0.39407436
ENSG0000014	SCRN2	protein_codir	-0.20809507	0.11223399	0.21991365
ENSG0000016	PSMC2	protein_codir	-0.20810105	0.04812777	0.11696339
ENSG0000015	MED8	protein_codir	-0.20811811	0.02031901	0.06001615
ENSG0000017	MRPL48	protein_codir	-0.208131	0.15062224	0.27310354
ENSG0000020	SLCO6A1	protein_codir	-0.20817944	0.91628665	NA
ENSG0000019	SDAD1	protein_codir	-0.20828462	0.13620518	0.25353734
ENSG0000023	HNRNPUL2-BS	protein_codir	-0.2083237	0.14977681	0.27206136
ENSG0000003	MTREX	protein_codir	-0.20833111	0.12645015	0.2402692
ENSG0000011	TIMMDC1	protein_codir	-0.20835083	0.04090598	0.10325564
ENSG0000013	RSPH3	protein_codir	-0.20837089	0.17008371	0.29891271
ENSG0000011	ITGB1BP1	protein_codir	-0.20849388	0.14960274	0.27180817
ENSG0000027	RP11-365H22	lncRNA	-0.20860681	0.76488571	0.85182626
ENSG0000019	MT-ND5	protein_codir	-0.20865296	0.31682641	0.46853058
ENSG0000026	SEPTIN4-AS1	lncRNA	-0.20866675	0.58063682	0.71235238
ENSG0000027	XXyac-YX155E	lncRNA	-0.20882859	0.79960675	0.87559523
ENSG0000013	CCDC142	protein_codir	-0.20888069	0.46367016	0.61357655
ENSG0000022	LINC01770	lncRNA	-0.20902497	0.6741984	0.78521442
ENSG0000022	ITGB1-DT	lncRNA	-0.2091149	0.75459379	0.8441084
ENSG0000016	PCNT	protein_codir	-0.2091306	0.01734581	0.05316147
ENSG0000006	SPHK2	protein_codir	-0.2091852	0.14937114	0.27158542
ENSG0000014	APIP	protein_codir	-0.20918944	0.20794841	0.34658069
ENSG0000007	TIPIN	protein_codir	-0.2092206	0.32238939	0.47452145
ENSG0000017	LRFN4	protein_codir	-0.20926164	0.27170136	0.41967878
ENSG0000016	INO80E	protein_codir	-0.2092805	0.33316826	0.48593268
ENSG0000017	EDC3	protein_codir	-0.20936084	0.00861021	0.03092511

ENSG0000023	KB-318B8.7	lncRNA	-0.20937144	0.74373009	0.83629536
ENSG0000010	ZPR1	protein_codir	-0.20950606	0.19272387	0.32773262
ENSG0000003	RIPOR1	protein_codir	-0.20965321	0.05279979	0.12532635
ENSG0000013	ARNTL	protein_codir	-0.20966398	0.418441	0.57112771
ENSG0000009	STRN4	protein_codir	-0.20967139	0.14526549	0.26622207
ENSG0000028	RP11-492O8.3	lncRNA	-0.20967927	0.91411741	NA
ENSG0000009	NUP188	protein_codir	-0.20974366	0.15043938	0.27286155
ENSG0000010	DHX40	protein_codir	-0.20982261	0.19419789	0.32967091
ENSG0000013	ATIC	protein_codir	-0.20988345	0.08213741	0.17400178
ENSG0000014	PSPH	protein_codir	-0.20990712	0.29507791	0.44550162
ENSG0000023	RP11-477J21.	lncRNA	-0.20994241	0.83004914	0.8975085
ENSG0000010	PSMD7	protein_codir	-0.21012021	0.13059428	0.24599427
ENSG0000012	ABHD8	protein_codir	-0.2102012	0.09428568	0.19310177
ENSG0000012	TRMT1L	protein_codir	-0.2102479	0.32821062	0.48063079
ENSG0000025	RP11-582J16.	lncRNA	-0.21030205	0.38550933	0.53866381
ENSG0000012	MTRR	protein_codir	-0.21040819	0.15471303	0.27867313
ENSG0000015	DCUN1D2	protein_codir	-0.21049007	0.3175446	0.46944183
ENSG0000023	RGL2	protein_codir	-0.21061778	0.07050349	0.15587181
ENSG0000024	SMARCA5-AS	lncRNA	-0.2106669	0.65760563	0.77243631
ENSG0000015	VIPAS39	protein_codir	-0.21069974	0.17664507	0.30709631
ENSG0000012	AP1M2	protein_codir	-0.21074661	0.87251416	0.92431706
ENSG0000016	LDLRAD4	protein_codir	-0.21090387	0.25990364	0.40701877
ENSG0000025	TVP23C-CDRT	protein_codir	-0.21102349	0.67623492	0.78669061
ENSG0000017	RBM4B	protein_codir	-0.21105018	0.30664149	0.45830292
ENSG0000010	POLA1	protein_codir	-0.21108984	0.23854131	0.3827859
ENSG0000021	GCC2-AS1	lncRNA	-0.2114957	0.73637304	0.83094207
ENSG0000012	KLHDC3	protein_codir	-0.21150258	0.07796375	0.16744701
ENSG0000013	NPAS1	protein_codir	-0.21154077	0.74862376	0.83965258
ENSG0000025	AC008592.5	lncRNA	-0.21159884	0.90097328	NA
ENSG0000006	EVI5	protein_codir	-0.21179791	0.2933035	0.44345538
ENSG0000014	SH3GL1	protein_codir	-0.21190398	0.19351245	0.32884178
ENSG0000026	DLGAP1-AS5	lncRNA	-0.21192265	0.85161946	0.9117271
ENSG0000023	RP3-523C21.2	lncRNA	-0.21208202	0.77881199	0.86156875
ENSG0000028	RP11-867O8.1	lncRNA	-0.21218661	0.66391983	0.7775384
ENSG0000016	CCDC112	protein_codir	-0.21226156	0.20608027	0.34413163
ENSG0000016	APEH	protein_codir	-0.21238715	0.07410554	0.16155126
ENSG0000025	RP11-33B1.3	lncRNA	-0.21240447	0.73720851	0.83169897
ENSG0000017	ZSCAN2	protein_codir	-0.21247811	0.32543444	0.47770511
ENSG0000013	TMEM127	protein_codir	-0.21256153	0.12854223	0.24314043
ENSG0000006	TNPO3	protein_codir	-0.21258891	0.02988532	0.08121937
ENSG0000028	RP11-24I5.1	protein_codir	-0.21261554	0.70604123	0.8088622
ENSG0000012	ZNF639	protein_codir	-0.21262753	0.25555604	0.402247
ENSG0000024	ZBTB20-AS4	lncRNA	-0.21262764	0.8446157	0.90718868
ENSG0000018	MUC1	protein_codir	-0.21270756	0.49712675	0.64289904
ENSG0000008	IMPG2	protein_codir	-0.21276202	0.54199664	0.68079254
ENSG0000014	CASK	protein_codir	-0.21281493	0.39344167	0.54650894
ENSG0000011	IK	protein_codir	-0.21284223	0.01809404	0.05489427

ENSG0000016	MRPS18C	protein_codir	-0.21290551	0.00956643	0.03359929
ENSG0000016	BPNT1	protein_codir	-0.21306747	0.25113651	0.39722772
ENSG0000027	RP11-1017G2	lncRNA	-0.21314453	0.72399673	0.82190067
ENSG0000014	OSBPL1A	protein_codir	-0.21327383	0.4066043	0.55967362
ENSG0000017	FOXD4	protein_codir	-0.21332754	0.66233066	0.77623681
ENSG0000011	FAM172A	protein_codir	-0.2134721	0.27216014	0.42015062
ENSG0000027	RP11-335L23.	lncRNA	-0.21363406	0.7917232	0.870448
ENSG0000016	IWS1	protein_codir	-0.2136988	0.02531402	0.07130784
ENSG0000013	ATAD1	protein_codir	-0.2137024	0.22192406	0.36303853
ENSG0000013	COX7B	protein_codir	-0.21372583	0.15155188	0.27433446
ENSG0000013	FAM98C	protein_codir	-0.21377181	0.14902957	0.27120694
ENSG0000000	ETV1	protein_codir	-0.21383759	0.4316163	0.58286033
ENSG0000016	KIAA1143	protein_codir	-0.21410879	0.23151856	0.3742975
ENSG0000016	SPATA17	protein_codir	-0.21412341	0.63804081	0.7573624
ENSG0000027	RP11-70D24.2	lncRNA	-0.21416767	0.64761677	0.76431121
ENSG0000028	RP11-503C24.	lncRNA	-0.21419881	0.84344737	0.90639867
ENSG0000014	LIMD1	protein_codir	-0.21422151	0.22516275	0.36694567
ENSG0000016	STX3	protein_codir	-0.21438087	0.15551655	0.27970835
ENSG0000010	SLC25A15	protein_codir	-0.21440034	0.38571596	0.53887076
ENSG0000005	USE1	protein_codir	-0.21456194	0.11243845	0.22014249
ENSG0000014	ARHGDIA	protein_codir	-0.21457935	0.18046428	0.31201399
ENSG0000016	PLEKHF1	protein_codir	-0.21480715	0.25627591	0.40297983
ENSG0000007	RBMS2	protein_codir	-0.21490468	0.26370874	0.4111796
ENSG0000016	WNK2	protein_codir	-0.21503541	NA	NA
ENSG0000011	FBXL4	protein_codir	-0.21507744	0.16010303	0.2857911
ENSG0000000	MARK4	protein_codir	-0.21515539	0.18489366	0.31800006
ENSG0000005	CROCC	protein_codir	-0.21542679	0.231587	0.37432309
ENSG0000012	GLIS2	protein_codir	-0.21544499	0.44477062	0.59590417
ENSG0000010	MROH8	protein_codir	-0.21548992	0.34714582	0.50027213
ENSG0000011	C11orf58	protein_codir	-0.21554781	0.0763915	0.16514392
ENSG0000017	TRMT112	protein_codir	-0.21559075	0.04418053	0.10958835
ENSG0000010	DGKH	protein_codir	-0.2157067	0.38991772	0.54292839
ENSG0000012	HOXB3	protein_codir	-0.21570757	0.48372355	0.63114994
ENSG0000023	ZNF529-AS1	lncRNA	-0.21577205	0.32366588	0.4759691
ENSG0000028	RP4-769N13.9	lncRNA	-0.21583727	0.70761274	0.8101585
ENSG0000027	USP34-DT	lncRNA	-0.21583998	0.30833787	0.45997569
ENSG0000016	PDHB	protein_codir	-0.21584641	0.09828371	0.19917706
ENSG0000028	RP11-264E23.	lncRNA	-0.21585085	0.73627128	0.83094207
ENSG0000025	LINC02682	lncRNA	-0.21588985	0.8529162	0.91247086
ENSG0000027	RP11-379F4.9	lncRNA	-0.21598052	0.67127453	0.78319615
ENSG0000005	ALDH18A1	protein_codir	-0.2161383	0.22353243	0.36503274
ENSG0000013	ACE2	protein_codir	-0.21627124	0.78185329	0.86361879
ENSG0000010	DTWD1	protein_codir	-0.21644255	0.21980665	0.36059276
ENSG0000015	ADAMTS1	protein_codir	-0.2164821	0.6682742	0.78085005
ENSG0000008	PIGS	protein_codir	-0.21663528	0.07935709	0.16963086
ENSG0000016	SAP30L	protein_codir	-0.21665779	0.27026443	0.41810033
ENSG0000017	SCAND1	protein_codir	-0.21666159	0.17062935	0.29964284

ENSG0000027	RP11-527J8.1	lncRNA	-0.21666433	0.64759345	0.76431121
ENSG0000014	GSTO1	protein_codir	-0.21680061	0.15795052	0.28297292
ENSG0000012	CANX	protein_codir	-0.21696942	0.27518468	0.42329031
ENSG0000007	KIFAP3	protein_codir	-0.21701962	0.1280796	0.24244798
ENSG0000013	RDX	protein_codir	-0.21704005	0.18136301	0.3132737
ENSG0000015	TBRG1	protein_codir	-0.21728633	0.08697312	0.18164657
ENSG0000015	PLBD2	protein_codir	-0.21745607	0.16959903	0.29827486
ENSG0000014	RAB5A	protein_codir	-0.21767124	0.22990056	0.37232589
ENSG0000014	B3GAT3	protein_codir	-0.2177035	0.08433128	0.17742459
ENSG0000027	H4C9	protein_codir	-0.21770969	0.72004817	0.81920049
ENSG0000024	TRIM52-AS1	lncRNA	-0.21788749	0.27271845	0.42064465
ENSG0000012	TCP1	protein_codir	-0.21803099	0.15079297	0.27330545
ENSG0000019	ATG9A	protein_codir	-0.21810129	0.02047239	0.06034658
ENSG0000016	METTL2B	protein_codir	-0.21818126	0.09903567	0.20030501
ENSG0000018	ANXA2	protein_codir	-0.21825085	0.27600988	0.42416439
ENSG0000017	ZNF454	protein_codir	-0.21832666	0.45153579	0.60213492
ENSG0000012	NDUFA5	protein_codir	-0.21835341	0.11809549	0.22824137
ENSG0000015	MRPL17	protein_codir	-0.21854483	0.13670777	0.25423557
ENSG0000023	AC092168.2	lncRNA	-0.21860685	0.8104395	0.88303526
ENSG0000019	RAB40C	protein_codir	-0.21862475	0.18277673	0.31518345
ENSG0000027	LL0XNC01-7P3	lncRNA	-0.21869997	0.64276996	0.7607208
ENSG0000000	PSMB1	protein_codir	-0.21873617	0.1000084	0.20174057
ENSG0000017	NDUFAF3	protein_codir	-0.21878859	0.08699681	0.18167638
ENSG0000026	CTD-2583A14	protein_codir	-0.21888431	0.4192176	0.57182034
ENSG0000023	GOLGA8N	protein_codir	-0.21898462	0.42118588	0.57317471
ENSG0000015	USP16	protein_codir	-0.21903103	0.16643489	0.2942978
ENSG0000016	FASTK	protein_codir	-0.21905155	0.2714242	0.41944477
ENSG0000011	ZNF142	protein_codir	-0.21911353	0.03742475	0.09635376
ENSG0000019	ZNF766	protein_codir	-0.21929403	0.25391588	0.40031718
ENSG0000020	LIPJ	protein_codir	-0.21933211	0.76351069	0.85074089
ENSG0000016	STIM1	protein_codir	-0.21948454	0.15343547	0.27694992
ENSG0000013	TRIM28	protein_codir	-0.21957652	0.11295908	0.22080182
ENSG0000014	MRPL9	protein_codir	-0.21961086	0.00915619	0.03251601
ENSG0000013	ARHGAP24	protein_codir	-0.21962577	0.3361986	0.48919004
ENSG0000018	MRPS16	protein_codir	-0.21971334	0.07158667	0.15762306
ENSG0000018	C9orf153	protein_codir	-0.21990362	0.68721277	0.79480829
ENSG0000009	PDCD7	protein_codir	-0.22006695	0.08745394	0.18231353
ENSG0000016	URM1	protein_codir	-0.22011629	0.07988595	0.17043488
ENSG0000013	DCAF8	protein_codir	-0.22011764	0.15612881	0.28054633
ENSG0000027	RP11-440L14.	lncRNA	-0.22013626	0.61439414	0.73851363
ENSG0000025	LINC02236	lncRNA	-0.22019728	0.69160338	0.79754855
ENSG0000017	GOLGB1	protein_codir	-0.22055159	0.14725675	0.26878348
ENSG0000010	TAB1	protein_codir	-0.22063719	0.06545219	0.14779181
ENSG0000013	EIF2S1	protein_codir	-0.22065815	0.17641568	0.30681953
ENSG0000011	HARS2	protein_codir	-0.22066093	0.12416216	0.23705789
ENSG0000010	USB1	protein_codir	-0.22066885	0.08250573	0.17454107
ENSG0000013	CKAP4	protein_codir	-0.2206716	0.38866135	0.54156122

ENSG0000013	GLTP	protein_codir	-0.22070759	0.08102812	0.17229937
ENSG0000010	MTG2	protein_codir	-0.22073141	0.22003131	0.36086128
ENSG0000013	SLC15A4	protein_codir	-0.22087224	0.11643061	0.22594066
ENSG0000015	GOLGA6A	protein_codir	-0.22101818	0.8385907	0.90320835
ENSG0000019	NEK5	protein_codir	-0.22110029	0.51125798	0.65505151
ENSG0000018	DRG1	protein_codir	-0.22125315	0.01074326	0.03686987
ENSG0000027	ENSG0000027	protein_codir	-0.22126377	0.6167221	0.74050621
ENSG0000013	AHI1	protein_codir	-0.22126648	0.2459567	0.39118531
ENSG0000013	PDCL	protein_codir	-0.22139378	0.10671586	0.21189735
ENSG0000028	RP1-130E4.3	lncRNA	-0.22139784	0.83097701	0.89815309
ENSG0000013	CCNJL	protein_codir	-0.22151335	0.30706578	0.4588002
ENSG0000017	TMEM182	protein_codir	-0.22158964	0.2213014	0.36225595
ENSG0000013	SAP130	protein_codir	-0.22178066	0.06790014	0.15175611
ENSG0000010	PIGH	protein_codir	-0.22184849	0.02455911	0.0696298
ENSG0000014	LMO4	protein_codir	-0.22200546	0.15643216	0.28096347
ENSG0000026	TFIP11-DT	lncRNA	-0.22214418	0.48013734	0.62790688
ENSG0000004	DTNBP1	protein_codir	-0.22222889	0.12243851	0.23464832
ENSG0000018	KPNA4	protein_codir	-0.22225726	0.26320646	0.41060505
ENSG0000022	INTS6L-AS1	lncRNA	-0.2222584	0.88029541	NA
ENSG0000013	MYCBPAP	protein_codir	-0.22227455	0.5022742	0.64702268
ENSG0000015	TGOLN2	protein_codir	-0.22232975	0.14458052	0.2651603
ENSG0000013	ERG28	protein_codir	-0.22235113	0.06038385	0.13865995
ENSG0000013	WDR44	protein_codir	-0.22235996	0.21271116	0.35232877
ENSG0000021	NBPF1	protein_codir	-0.22244554	0.36640646	0.52028926
ENSG0000011	FOXP1	protein_codir	-0.22256159	0.25848486	0.40554107
ENSG0000011	DNAL1	protein_codir	-0.22260032	0.30375906	0.45502903
ENSG0000012	MMP24	protein_codir	-0.22262994	0.63894762	0.75794224
ENSG0000012	H2BC11	protein_codir	-0.22268342	0.55033627	0.68742257
ENSG0000025	AGAP2-AS1	lncRNA	-0.22276669	0.19745917	0.33364875
ENSG0000016	SERPINI1	protein_codir	-0.22283157	0.41439064	0.56747828
ENSG0000008	EFR3B	protein_codir	-0.22286129	0.39362859	0.54668988
ENSG0000016	CMYA5	protein_codir	-0.22288966	0.63353437	0.75374625
ENSG0000008	JMJD4	protein_codir	-0.22289001	0.07802118	0.16754672
ENSG0000014	BAHD1	protein_codir	-0.22292373	0.02552084	0.0717806
ENSG0000025	RP11-563N12	lncRNA	-0.22295643	0.90756429	NA
ENSG0000024	NSMCE1-DT	lncRNA	-0.22299024	0.71557325	0.81575587
ENSG0000026	ZNF723	protein_codir	-0.22299187	0.89441958	NA
ENSG0000028	RP11-783K16.	protein_codir	-0.22299543	0.27182259	0.4198025
ENSG0000013	NUDT15	protein_codir	-0.22309382	0.19697063	0.33302699
ENSG0000023	RP11-297A16	lncRNA	-0.22313105	0.83566243	0.90127871
ENSG0000010	CD276	protein_codir	-0.22326238	0.33214886	0.48496527
ENSG0000027	CTB-152G17.6	lncRNA	-0.22329819	0.55305204	0.68950516
ENSG0000015	ATP5MJ	protein_codir	-0.2233193	0.05954936	0.13714279
ENSG0000007	TRNT1	protein_codir	-0.22334976	0.09844033	0.19942216
ENSG0000011	SLC35A3	protein_codir	-0.22335435	0.09549695	0.19482239
ENSG0000016	ADAL	protein_codir	-0.22341221	0.12440275	0.23739145
ENSG0000012	NQO2	protein_codir	-0.22348416	0.31526818	0.46712661

ENSG0000026	CTD-2270L9.4	lncRNA	-0.22374368	0.36034014	0.51421311
ENSG0000027	RP11-136C24.	protein_codir	-0.22381604	0.57142467	0.70474433
ENSG0000013	VPS25	protein_codir	-0.22396167	0.05825028	0.13484811
ENSG0000008	CNOT4	protein_codir	-0.22396215	0.04989735	0.12036373
ENSG0000014	ATP6V1C2	protein_codir	-0.22403945	0.62747997	0.7487362
ENSG0000007	CLASP1	protein_codir	-0.22406165	0.0781788	0.16776463
ENSG0000027	CITF22-92A6.:	lncRNA	-0.22431509	0.28093047	0.43021863
ENSG0000025	OTUD6B-AS1	lncRNA	-0.22432817	0.06854044	0.15260676
ENSG0000025	RP11-212D19	protein_codir	-0.22436643	0.62738021	0.7487173
ENSG0000016	EBF1	protein_codir	-0.22437946	0.38379775	0.5371146
ENSG0000014	SLC25A38	protein_codir	-0.22438033	0.04999015	0.12048237
ENSG0000010	RHEB	protein_codir	-0.22452923	0.23799368	0.38210692
ENSG0000011	ECE1	protein_codir	-0.22454597	0.11716928	0.22692766
ENSG0000016	LEO1	protein_codir	-0.22470714	0.17250297	0.30208801
ENSG0000027	RP4-742C19.1	lncRNA	-0.2247336	0.6310564	0.75166638
ENSG0000026	LINC00622	lncRNA	-0.22475609	0.57533913	0.70786524
ENSG0000023	HM13-AS1	lncRNA	-0.22511662	0.84571984	0.90812464
ENSG0000026	CTB-129P6.4	lncRNA	-0.22520515	0.89162367	NA
ENSG0000011	ODC1	protein_codir	-0.22530643	0.4002907	0.55352103
ENSG0000010	FKBP14	protein_codir	-0.22537229	0.37545467	0.52897664
ENSG0000028	LA16c-407A1(	lncRNA	-0.22538475	0.82664324	0.89515746
ENSG0000011	UNC50	protein_codir	-0.22543246	0.01418679	0.04567953
ENSG0000023	AC073130.1	lncRNA	-0.22548661	0.75171681	0.84190423
ENSG0000028	RP11-809H16	lncRNA	-0.22550049	0.89357095	NA
ENSG0000013	RNF38	protein_codir	-0.22555968	0.26328355	0.41068809
ENSG0000019	HYLS1	protein_codir	-0.22558896	0.23864805	0.38290015
ENSG0000014	ARSG	protein_codir	-0.22574063	0.18143261	0.31337431
ENSG0000026	CTC-471J1.2	lncRNA	-0.22578115	0.54493921	0.68305972
ENSG0000003	IFT88	protein_codir	-0.22621623	0.08820773	0.18353906
ENSG0000000	SCIN	protein_codir	-0.22625809	0.59531407	0.72376858
ENSG0000013	ASNSD1	protein_codir	-0.22644465	0.04595914	0.112895
ENSG0000010	DDX49	protein_codir	-0.22648803	0.08724884	0.18203964
ENSG0000026	RP11-355O1.:	lncRNA	-0.22664295	0.47148528	0.62032217
ENSG0000012	CLYBL	protein_codir	-0.22676117	0.14417974	0.26453065
ENSG0000026	SEC22B	protein_codir	-0.22683367	0.03352326	0.08862102
ENSG0000027	C11orf98	protein_codir	-0.22690894	0.14734218	0.26890386
ENSG0000013	RAC1	protein_codir	-0.2270202	0.10488228	0.20908326
ENSG0000013	RPS6KC1	protein_codir	-0.22710365	0.21696859	0.3572383
ENSG0000010	HIRA	protein_codir	-0.2274243	0.06290884	0.14327781
ENSG0000008	ESF1	protein_codir	-0.22757724	0.22508657	0.36685409
ENSG0000017	GPHN	protein_codir	-0.2276161	0.1548216	0.27882241
ENSG0000001	BTBD7	protein_codir	-0.22762656	0.11914368	0.22980087
ENSG0000027	HK2-DT	lncRNA	-0.22763402	0.62637444	0.74806339
ENSG0000007	TIMM21	protein_codir	-0.22769139	0.0792765	0.16955373
ENSG0000022	AC017104.6	lncRNA	-0.22782295	0.37889736	0.53228193
ENSG0000028	TMEM265	protein_codir	-0.22785953	0.94365662	NA
ENSG0000006	RAB27A	protein_codir	-0.22788689	0.11352899	0.22168051

ENSG0000011	VPS29	protein_codir	-0.2279177	0.07627823	0.16496786
ENSG0000014	WDPCP	protein_codir	-0.22822591	0.11833335	0.22863708
ENSG0000010	DNAJC12	protein_codir	-0.22830131	0.55803278	0.69364831
ENSG0000012	ORMDL2	protein_codir	-0.22833955	0.11150867	0.21888553
ENSG0000020	GNL1	protein_codir	-0.22843805	0.26539261	0.41298912
ENSG0000019	BCO2	protein_codir	-0.2284775	0.40827191	0.5612418
ENSG0000012	MED20	protein_codir	-0.22850779	0.09777932	0.19840323
ENSG0000010	POLR1F	protein_codir	-0.2286399	0.13913619	0.25774257
ENSG0000027	NCBP2AS2	protein_codir	-0.22869898	0.12140997	0.23319496
ENSG0000012	BECN1	protein_codir	-0.22872419	0.07197954	0.15824842
ENSG0000017	SVBP	protein_codir	-0.2288219	0.14576066	0.26684626
ENSG0000016	MAMDC2	protein_codir	-0.22885876	0.66856395	0.78107128
ENSG0000018	TMEM198	protein_codir	-0.22895223	0.42604459	0.57787908
ENSG0000018	P4HB	protein_codir	-0.22903953	0.2398569	0.38429419
ENSG0000025	RP11-723D22	lncRNA	-0.22906373	0.89094009	NA
ENSG0000015	EIF4E	protein_codir	-0.22918059	0.21453859	0.35450529
ENSG0000013	BRME1	protein_codir	-0.2291944	0.42841514	0.58015194
ENSG0000020	PRRC2A	protein_codir	-0.22923618	0.04574333	0.11250496
ENSG0000023	LINC02777	lncRNA	-0.22930834	0.54250483	0.68118139
ENSG0000004	RTN4R	protein_codir	-0.22935149	0.59165559	0.72080005
ENSG0000017	DDX23	protein_codir	-0.229414	0.01262633	0.04166539
ENSG0000019	COPS8	protein_codir	-0.2297876	0.17300127	0.30271112
ENSG0000022	LINC02679	lncRNA	-0.22988262	0.880174	NA
ENSG0000011	RBM18	protein_codir	-0.23034179	0.03697619	0.09550049
ENSG0000018	ZNF197	protein_codir	-0.23036828	0.17524335	0.30535747
ENSG0000017	ZBTB41	protein_codir	-0.2304628	0.37161255	0.52503989
ENSG0000022	RP11-98I9.4	lncRNA	-0.23067418	0.37849091	0.53204827
ENSG0000014	CDAN1	protein_codir	-0.23070185	0.15293865	0.27626983
ENSG0000016	TCTN2	protein_codir	-0.23071061	0.18460706	0.31764559
ENSG0000023	ZFY-AS1	lncRNA	-0.23072094	0.71943052	0.81878785
ENSG0000024	CTB-129O4.1	lncRNA	-0.2307811	0.79653908	0.87360361
ENSG0000018	NPB	protein_codir	-0.23078731	0.61621539	0.74004088
ENSG0000017	FAM174A	protein_codir	-0.23090801	0.21186354	0.3514306
ENSG0000028	LLNLR-245B6.	lncRNA	-0.231018	0.22939418	0.3718761
ENSG0000026	AC084219.2	protein_codir	-0.23108899	0.74122686	0.8346319
ENSG0000016	CCDC127	protein_codir	-0.23113263	0.16960198	0.29827486
ENSG0000012	AMD1	protein_codir	-0.23115959	0.25436763	0.40084635
ENSG0000013	EEFSEC	protein_codir	-0.23158009	0.01988433	0.05904574
ENSG0000019	KIAA0895L	protein_codir	-0.23158875	0.50048524	0.64574216
ENSG0000015	CUL4B	protein_codir	-0.23163579	0.05924488	0.13656681
ENSG0000012	LRP11	protein_codir	-0.23164077	0.2644864	0.41197352
ENSG0000010	TGFBR1	protein_codir	-0.23165287	0.50997778	0.65413534
ENSG0000018	FAM120AOS	protein_codir	-0.23181835	0.02286065	0.06577886
ENSG0000025	MIR3180-2	lncRNA	-0.23186827	0.73920647	0.83324012
ENSG0000025	MIR3180-3	lncRNA	-0.23186827	0.73920647	0.83324012
ENSG0000011	SLC1A4	protein_codir	-0.23190626	0.44501787	0.59614054
ENSG0000013	PWP1	protein_codir	-0.23191307	0.08367557	0.17634054

ENSG0000022	XXbac-B476C2	lncRNA	-0.23196636	0.70445925	0.80768637
ENSG0000016	SHC1	protein_codir	-0.23198813	0.14895896	0.27110216
ENSG0000007	GPATCH1	protein_codir	-0.23199168	0.13994974	0.25895437
ENSG0000012	C4BPB	protein_codir	-0.23210843	0.72651217	0.82389002
ENSG0000010	LYRM1	protein_codir	-0.23217954	0.10621169	0.21112393
ENSG0000006	RHOA	protein_codir	-0.23229327	0.12541905	0.23890166
ENSG0000013	LINC00470	lncRNA	-0.23235208	0.82684852	0.89526003
ENSG0000017	CANT1	protein_codir	-0.23246931	0.11237443	0.22012636
ENSG0000027	AC005754.8	lncRNA	-0.23249902	0.58244649	0.71368493
ENSG0000016	SEMA4C	protein_codir	-0.23252931	0.3255961	0.47783711
ENSG0000027	H4C5	protein_codir	-0.23253312	0.49887966	0.64452608
ENSG0000016	MARS1	protein_codir	-0.23253417	0.1374892	0.25524383
ENSG0000026	KCNJ2-AS1	lncRNA	-0.23260074	0.43888714	0.58996752
ENSG0000025	RP11-190A12	protein_codir	-0.23271122	0.56685047	0.70077926
ENSG0000010	EIF4H	protein_codir	-0.23284889	0.08267798	0.17470615
ENSG0000016	GMPS	protein_codir	-0.23294885	0.11140823	0.21873012
ENSG0000013	COG6	protein_codir	-0.23297	0.16235339	0.28869092
ENSG0000018	MCFD2	protein_codir	-0.23300275	0.33915002	0.49203296
ENSG0000000	MPND	protein_codir	-0.23300944	0.0982838	0.19917706
ENSG0000010	COG4	protein_codir	-0.23315234	0.04602383	0.11301054
ENSG0000011	UHRF1BP1L	protein_codir	-0.23353159	0.39542909	0.54839081
ENSG0000019	ATAD3A	protein_codir	-0.2335418	0.21406834	0.35390394
ENSG0000027	EPB41L4A-DT	lncRNA	-0.23378487	0.35385528	0.50747613
ENSG0000011	ERRFI1	protein_codir	-0.23380461	0.63411363	0.75430546
ENSG0000013	SMARCC2	protein_codir	-0.23382144	0.0596758	0.13741106
ENSG0000019	NOP9	protein_codir	-0.23383423	0.00598904	0.02329642
ENSG0000016	GALNT10	protein_codir	-0.23386256	0.3904608	0.54346594
ENSG0000028	RP11-429A24	lncRNA	-0.23411388	0.87298439	NA
ENSG0000000	UPF1	protein_codir	-0.234143	3.50E-05	0.0003641
ENSG0000013	BIVM	protein_codir	-0.23415379	0.1029018	0.20620676
ENSG0000015	TEX12	protein_codir	-0.23452553	0.71832339	0.81804693
ENSG0000014	SPOPL	protein_codir	-0.23458946	0.30851209	0.46010918
ENSG0000013	SECISBP2L	protein_codir	-0.23463477	0.22455607	0.36623789
ENSG0000018	ZFP69B	protein_codir	-0.23464224	0.31887114	0.47089903
ENSG0000016	AHCYL1	protein_codir	-0.23466712	0.07011273	0.15529921
ENSG0000010	MAPK3	protein_codir	-0.23467655	0.07213425	0.15848762
ENSG0000018	ABAT	protein_codir	-0.23472951	0.23786884	0.3819731
ENSG0000022	HDAC4-AS1	lncRNA	-0.23478023	0.65865918	0.77321347
ENSG0000015	TIRAP	protein_codir	-0.2347869	0.12630091	0.24016737
ENSG0000024	NME2	protein_codir	-0.23490082	0.0923432	0.18999696
ENSG0000023	ZNF879	protein_codir	-0.23508043	0.26903813	0.41680689
ENSG0000023	MKLN1-AS	lncRNA	-0.23512389	0.19197735	0.32672466
ENSG0000011	MRPS27	protein_codir	-0.23514573	0.02832993	0.07792777
ENSG0000016	BRI3	protein_codir	-0.23521121	0.11853478	0.22893019
ENSG0000026	ZNF234	protein_codir	-0.23532591	0.19635373	0.33220765
ENSG0000018	ZNF566	protein_codir	-0.23538185	0.16634467	0.29417593
ENSG0000025	RP11-793H13	lncRNA	-0.23550304	0.48834623	0.63539302

ENSG0000017	SOCS5	protein_codir	-0.23573411	0.26132171	0.40868047
ENSG0000017	CHMP6	protein_codir	-0.23573569	0.07339222	0.16044746
ENSG0000019	C17orf102	lncRNA	-0.23588243	0.90598767	NA
ENSG0000011	GGCX	protein_codir	-0.23606317	0.01830439	0.05538036
ENSG0000019	SGTB	protein_codir	-0.23608058	0.21202452	0.35157095
ENSG0000028	RP1-81D8.7	lncRNA	-0.23614227	0.91884252	NA
ENSG0000025	RP11-624J12.	lncRNA	-0.23618538	0.88741702	NA
ENSG0000012	RECK	protein_codir	-0.23620195	0.32019583	0.47232776
ENSG0000011	PPP1R7	protein_codir	-0.23628196	0.03187168	0.08519266
ENSG0000027	SOCS7	protein_codir	-0.23635503	0.03942887	0.10040802
ENSG0000015	BAG4	protein_codir	-0.23639779	0.10140679	0.20389135
ENSG0000015	PRSS23	protein_codir	-0.23644368	0.5577129	0.69344424
ENSG0000027	RP11-214K3.2	lncRNA	-0.2365833	0.78533984	0.86580794
ENSG0000010	CGRRF1	protein_codir	-0.23661588	0.06466507	0.14640245
ENSG0000010	GALNT7	protein_codir	-0.2366926	0.23146224	0.37423502
ENSG0000028	RP11-118B23.	lncRNA	-0.23671704	0.5762876	0.70874804
ENSG0000010	PDCD5	protein_codir	-0.236734	0.07859852	0.16842975
ENSG0000025	HLX-AS1	lncRNA	-0.23674093	0.60726266	0.73328867
ENSG0000019	TXNRD3	protein_codir	-0.2367605	0.40098765	0.55406868
ENSG0000027	RP11-803P9.1	lncRNA	-0.23692096	0.72205555	0.82063922
ENSG0000014	IQGAP1	protein_codir	-0.23694861	0.28274775	0.43224153
ENSG0000012	COPA	protein_codir	-0.23704319	0.08738042	0.18221572
ENSG0000016	RPUSD2	protein_codir	-0.23712298	0.28346114	0.43300684
ENSG0000011	CARS1	protein_codir	-0.23714029	0.02451354	0.06952802
ENSG0000025	RP11-121C2.2	lncRNA	-0.23742314	0.30776472	0.45948441
ENSG0000014	SLC16A2	protein_codir	-0.23745466	0.39650467	0.54938593
ENSG0000019	ZNF649	protein_codir	-0.23746314	0.19256925	0.3275907
ENSG0000017	MRPL36	protein_codir	-0.23749024	0.10432894	0.20828083
ENSG0000018	TMEM262	protein_codir	-0.23749127	0.78838895	0.86792231
ENSG0000015	TEX29	protein_codir	-0.23749145	0.6571658	0.77210237
ENSG0000012	TAF1L	protein_codir	-0.23788339	0.70361655	0.80698818
ENSG0000023	ZBED5	protein_codir	-0.23790496	0.17859381	0.30962576
ENSG0000016	STOML2	protein_codir	-0.23791901	0.0833889	0.17585718
ENSG0000020	HACD2	protein_codir	-0.23796594	0.18345238	0.31604149
ENSG0000025	TMED7-TICAN	protein_codir	-0.23803574	0.33737966	0.49026287
ENSG0000015	PTPRK	protein_codir	-0.23817504	0.28654624	0.43612757
ENSG0000014	MVB12A	protein_codir	-0.23823809	0.02089968	0.06135726
ENSG0000021	SFT2D2	protein_codir	-0.23831339	0.32035683	0.47244067
ENSG0000009	MARCHF2	protein_codir	-0.23831445	0.21349392	0.35325784
ENSG0000009	XYLB	protein_codir	-0.23836432	0.31478331	0.4665834
ENSG0000023	RP4-630A11.3	lncRNA	-0.23837113	0.85832174	0.915499
ENSG0000019	KIF13B	protein_codir	-0.23842313	0.09387474	0.19241709
ENSG0000014	FTO	protein_codir	-0.23847047	0.19124739	0.32584374
ENSG0000010	PSMC6	protein_codir	-0.23847584	0.01748569	0.05350114
ENSG0000020	TIMM23B	protein_codir	-0.23847798	0.2287456	0.37122942
ENSG0000026	RP11-90P13.1	lncRNA	-0.23852936	0.72304719	0.82136067
ENSG0000007	SIRT6	protein_codir	-0.23855246	0.17590667	0.30614019

ENSG0000026	AC026150.8	lncRNA	-0.23860196	0.36227473	0.51612049
ENSG0000026	RP11-111J6.2	lncRNA	-0.23878376	0.63761152	0.75709686
ENSG0000022	SNED1-AS1	lncRNA	-0.23884728	0.84273383	0.90612798
ENSG0000013	TBC1D8B	protein_codir	-0.23920947	0.27301869	0.42091969
ENSG0000022	ATXN1-AS1	lncRNA	-0.23923956	NA	NA
ENSG0000010	FUBP3	protein_codir	-0.23929323	0.15725536	0.2821301
ENSG0000018	RBM12B	protein_codir	-0.23980153	0.2789082	0.42770776
ENSG0000017	ZNF570	protein_codir	-0.23980947	0.15886023	0.28419713
ENSG0000015	MTMR12	protein_codir	-0.23989775	0.1438335	0.26400062
ENSG0000013	LTV1	protein_codir	-0.24027044	0.08193038	0.17376311
ENSG0000013	RRP8	protein_codir	-0.24028755	0.08320273	0.17557299
ENSG0000016	STAT3	protein_codir	-0.24031393	0.19149352	0.32606197
ENSG0000011	C19orf25	protein_codir	-0.24038793	0.10870465	0.21479629
ENSG0000018	NDUFB1	protein_codir	-0.2405124	0.05919751	0.13653738
ENSG0000013	IMMT	protein_codir	-0.24052346	0.03300219	0.08757883
ENSG0000025	GIHCG	lncRNA	-0.2405951	0.25914072	0.4062381
ENSG0000012	METTL16	protein_codir	-0.24064589	0.04811281	0.11694761
ENSG0000024	LINC02469	lncRNA	-0.24065153	0.89954643	NA
ENSG0000027	RP11-440D17	lncRNA	-0.24072377	0.40755993	0.56058612
ENSG0000012	TSPAN8	protein_codir	-0.24082494	0.71276008	0.8138308
ENSG0000013	OLA1	protein_codir	-0.24089454	0.10255505	0.20567605
ENSG0000018	NOC2L	protein_codir	-0.24090249	0.08560998	0.17958136
ENSG0000011	SDHB	protein_codir	-0.24098047	0.04611828	0.11319032
ENSG0000011	SUDS3	protein_codir	-0.24100181	0.0435375	0.10834361
ENSG0000026	RP11-676J12.	lncRNA	-0.24104387	0.90038332	NA
ENSG0000018	TEAD1	protein_codir	-0.2411586	0.41533631	0.56831677
ENSG0000013	RFK	protein_codir	-0.24115904	0.13243138	0.24870842
ENSG0000009	NUBP2	protein_codir	-0.24128977	0.10693954	0.21215845
ENSG0000011	GNPAT	protein_codir	-0.24147652	0.00411168	0.01742323
ENSG0000011	NRBP1	protein_codir	-0.24154982	0.03595398	0.09340252
ENSG0000010	IDH3B	protein_codir	-0.2417142	0.05161407	0.12324016
ENSG0000006	ELMO2	protein_codir	-0.24182257	0.00831542	0.03009327
ENSG0000026	CTD-2291D10	lncRNA	-0.24184311	0.77117241	0.85608432
ENSG0000013	ADRM1	protein_codir	-0.24229305	0.05697986	0.13265203
ENSG0000027	RP4-539M6.2	lncRNA	-0.2423299	0.78144146	0.86330199
ENSG0000010	SLC25A11	protein_codir	-0.24250857	0.00928014	0.03287387
ENSG0000024	RP11-469J4.3	lncRNA	-0.24255182	0.80578667	0.87967699
ENSG0000014	SURF1	protein_codir	-0.24263454	0.04428406	0.109786
ENSG0000023	LINC02569	lncRNA	-0.24265932	0.72404129	0.82191527
ENSG0000013	TTPA	protein_codir	-0.24269313	0.64392925	0.76170124
ENSG0000001	ERCC1	protein_codir	-0.24278869	0.101507	0.20403343
ENSG0000014	ARF1	protein_codir	-0.24287406	0.07237718	0.15889498
ENSG0000018	BLOC1S5	protein_codir	-0.242958	0.17134165	0.30056885
ENSG0000027	RP11-660M5.	lncRNA	-0.24305943	0.87093267	0.92331633
ENSG0000016	LMTK2	protein_codir	-0.24308324	0.08684851	0.1814902
ENSG0000019	ZNF79	protein_codir	-0.24308648	0.22368894	0.36516866
ENSG0000012	TMX4	protein_codir	-0.24309948	0.31113555	0.46294047

ENSG0000009	DLD	protein_codir	-0.24325318	0.07038922	0.15566901
ENSG0000017	PACS2	protein_codir	-0.24326826	0.0360337	0.09355749
ENSG0000016	OXER1	protein_codir	-0.24352801	0.40057628	0.5538314
ENSG0000010	TRIP11	protein_codir	-0.24353821	0.13737383	0.25511096
ENSG0000024	RP1-130H16.1	protein_codir	-0.24354572	0.67406267	0.78512255
ENSG0000003	RFC1	protein_codir	-0.24357113	0.12868188	0.2433764
ENSG0000014	ZC3H12C	protein_codir	-0.24366774	0.41032393	0.56331704
ENSG0000025	RP11-661A12	lncRNA	-0.24371665	0.82141141	0.89085417
ENSG0000025	RP11-100L22.	lncRNA	-0.24372927	0.5614656	0.69631717
ENSG0000019	SPOUT1	protein_codir	-0.24384988	0.14706404	0.26855596
ENSG0000026	RP11-927P21.	lncRNA	-0.24416947	0.60760065	0.73352016
ENSG0000015	IMPACT	protein_codir	-0.24418234	0.19549419	0.33124039
ENSG0000011	MRPL37	protein_codir	-0.2442094	0.04383789	0.10888322
ENSG0000019	ZNF511	protein_codir	-0.24434897	0.03226318	0.08596459
ENSG0000016	WDFY3	protein_codir	-0.24436788	0.41538115	0.56831677
ENSG0000025	FNTB	protein_codir	-0.24454082	0.10149418	0.20402251
ENSG0000012	POLK	protein_codir	-0.24454562	0.14230777	0.26191277
ENSG0000019	MT-CYB	protein_codir	-0.24461005	0.46934576	0.61863643
ENSG0000010	TMEM104	protein_codir	-0.24461013	0.12143913	0.23321853
ENSG0000020	C6orf47	protein_codir	-0.24464245	0.0116503	0.03923049
ENSG0000017	DMAP1	protein_codir	-0.24465989	0.20750722	0.34603612
ENSG0000010	MTFMT	protein_codir	-0.24484587	0.06636893	0.14933685
ENSG0000010	TMEM87A	protein_codir	-0.24514391	0.10714271	0.21242422
ENSG0000027	CD2AP-DT	lncRNA	-0.24515282	0.8043481	0.87873169
ENSG0000015	C12orf43	protein_codir	-0.2452502	0.08063164	0.17172072
ENSG0000016	SLC4A1AP	protein_codir	-0.24534808	0.00021875	0.00166078
ENSG0000022	INKA2-AS1	lncRNA	-0.24563023	0.57582668	0.70837043
ENSG0000013	DCTN4	protein_codir	-0.24564296	0.09447496	0.19334606
ENSG0000021	RP11-323J4.1	lncRNA	-0.24576625	0.8993041	NA
ENSG0000010	EMC2	protein_codir	-0.24587075	0.17069308	0.29971664
ENSG0000011	INO80B	protein_codir	-0.24596264	0.12537962	0.23884302
ENSG0000015	EEF1AKMT1	protein_codir	-0.24604165	0.21042879	0.34965968
ENSG0000011	ZBTB17	protein_codir	-0.24607244	0.25323775	0.39954454
ENSG0000017	DNAJC30	protein_codir	-0.24614568	0.01228138	0.04079514
ENSG0000012	B4GALT4	protein_codir	-0.24628904	0.11432966	0.22280288
ENSG0000023	CBR3-AS1	lncRNA	-0.24631643	0.33224934	0.48498276
ENSG0000018	SCFD2	protein_codir	-0.24643742	0.24457339	0.38961351
ENSG0000010	DNAJA3	protein_codir	-0.24651892	0.03123256	0.08396373
ENSG0000018	NDUFA12	protein_codir	-0.24654334	0.04399698	0.10920171
ENSG0000012	COX7C	protein_codir	-0.24654941	0.08583358	0.17987431
ENSG0000016	DCTN5	protein_codir	-0.24661167	0.09375351	0.19219714
ENSG0000025	RP11-507B12.	lncRNA	-0.24678409	0.91438999	NA
ENSG0000012	SPATA1	protein_codir	-0.24711233	0.58796544	0.71818528
ENSG0000010	PARD6A	protein_codir	-0.24716173	0.2125047	0.35207124
ENSG0000024	RP11-834C11.	lncRNA	-0.24734866	0.75434839	0.84387444
ENSG0000020	SAMD13	protein_codir	-0.24748371	0.54255284	0.68118139
ENSG0000016	MEAF6	protein_codir	-0.2475177	0.04293935	0.10725584

ENSG0000027	CTD-2095E4.5	lncRNA	-0.24757298	0.61057573	0.73587737
ENSG0000022	MEG8	lncRNA	-0.24757969	0.5904071	0.72005317
ENSG0000025	LINC02381	lncRNA	-0.247647	0.46741342	0.61696337
ENSG0000025	RP11-3D4.2	lncRNA	-0.24793263	0.69507456	0.800115
ENSG0000019	DDRGK1	protein_codir	-0.2480293	0.00579894	0.02272513
ENSG0000017	ZNF57	protein_codir	-0.24814038	0.37439795	0.52797639
ENSG0000024	RPP21	protein_codir	-0.24828937	0.05336429	0.12636503
ENSG0000004	STAU2	protein_codir	-0.24829101	0.04757087	0.11586485
ENSG0000011	RRP9	protein_codir	-0.24842542	0.11565898	0.22476849
ENSG0000027	RP1-225E12.3	lncRNA	-0.24844011	0.70583092	0.80874768
ENSG0000025	RP4-773N10.4	lncRNA	-0.24858948	0.09020613	0.18649731
ENSG0000013	HILPDA	protein_codir	-0.24881563	0.6433869	0.76127088
ENSG0000025	ST20-MTHFS	protein_codir	-0.24895477	0.37841512	0.53200782
ENSG0000012	CCDC91	protein_codir	-0.24899159	0.02322193	0.06659636
ENSG0000025	RP11-410E4.1	lncRNA	-0.24911144	0.66744832	0.78031951
ENSG0000022	WDR46	protein_codir	-0.24940298	0.05032828	0.12117728
ENSG0000026	SMC5-DT	lncRNA	-0.2494226	0.53848391	0.67798357
ENSG0000008	CLDND1	protein_codir	-0.24951673	0.10988003	0.21651468
ENSG0000014	C1orf43	protein_codir	-0.24980669	0.02169956	0.06312848
ENSG0000023	LL0XNC01-237	lncRNA	-0.24981187	0.3579312	0.51148902
ENSG0000019	ZNF790	protein_codir	-0.25000672	0.26676341	0.41435189
ENSG0000011	PDCL3	protein_codir	-0.25004051	0.21761289	0.3579364
ENSG0000010	TNPO2	protein_codir	-0.25014301	0.16221854	0.28852497
ENSG0000024	INMT	protein_codir	-0.25017764	0.42805516	0.57978577
ENSG0000028	RP11-551M15	lncRNA	-0.25019079	0.85858537	0.91574482
ENSG0000027	ZNF658	protein_codir	-0.25019905	0.46303916	0.61298257
ENSG0000026	RP11-490O6.2	lncRNA	-0.25020377	0.56887394	0.70266528
ENSG0000005	FAM168A	protein_codir	-0.25021952	0.12739755	0.24155354
ENSG0000016	COX6C	protein_codir	-0.2503184	0.13733702	0.25508704
ENSG0000015	SAR1B	protein_codir	-0.25033257	0.07245126	0.15900705
ENSG0000028	RP11-249L21.	lncRNA	-0.25039476	0.44100932	0.59201348
ENSG0000000	GTF2IRD1	protein_codir	-0.25051053	0.24917405	0.3949152
ENSG0000014	SRP9	protein_codir	-0.25055203	0.02322487	0.06659789
ENSG0000024	CCDC169	protein_codir	-0.25065679	0.65348635	0.76906468
ENSG0000028	RP11-384P7.1	lncRNA	-0.25069576	0.81812821	0.88823511
ENSG0000019	MIB2	protein_codir	-0.25076775	0.16936639	0.29793641
ENSG0000021	STARD10	protein_codir	-0.25079258	0.1562613	0.28076615
ENSG0000015	NUBPL	protein_codir	-0.25093085	0.07086388	0.15646818
ENSG0000016	CHTF8	protein_codir	-0.25095501	0.05769356	0.13381644
ENSG0000008	RRAGB	protein_codir	-0.25108293	0.1670538	0.29516543
ENSG0000015	ZNF599	protein_codir	-0.25113753	0.14131996	0.26071186
ENSG0000017	ETFDH	protein_codir	-0.25116314	0.02480599	0.07017017
ENSG0000014	LIPT1	protein_codir	-0.25127994	0.22144248	0.36242246
ENSG0000023	RP11-65J3.3	lncRNA	-0.25130725	0.89545671	NA
ENSG0000016	NIPAL1	protein_codir	-0.25135284	0.52462255	0.66654788
ENSG0000011	CD46	protein_codir	-0.25141169	0.03668345	0.09491305
ENSG0000012	SNX19	protein_codir	-0.25151557	0.19815866	0.33440106

ENSG0000016	IDH3A	protein_codir	-0.25153689	0.07316178	0.16013379
ENSG0000017	TRIM8	protein_codir	-0.25158953	0.04330788	0.10790832
ENSG0000021	GTF2H4	protein_codir	-0.25159462	0.091842	0.18914836
ENSG0000017	TMEM126A	protein_codir	-0.25178398	0.08959157	0.18559358
ENSG0000026	LINC01311	lncRNA	-0.25182277	0.46362223	0.61354252
ENSG0000028	RP11-698N11	lncRNA	-0.25203614	0.92866873	NA
ENSG0000017	CORO1B	protein_codir	-0.25228492	0.04439074	0.1099715
ENSG0000014	CTU1	protein_codir	-0.25236412	0.16659288	0.29452061
ENSG0000016	MFF	protein_codir	-0.2523827	0.02558668	0.07191453
ENSG0000018	ZNF594	protein_codir	-0.25247506	0.38352829	0.53692795
ENSG0000011	PGF	protein_codir	-0.25278204	0.42430986	0.57629025
ENSG0000017	POLR2L	protein_codir	-0.25291723	0.09812534	0.19897378
ENSG0000016	ZNF641	protein_codir	-0.25299121	0.1192731	0.22995414
ENSG0000017	EPM2AIP1	protein_codir	-0.25309013	0.05250008	0.12483232
ENSG0000012	MSANTD2	protein_codir	-0.25312484	0.23737659	0.3814461
ENSG0000016	POLR2G	protein_codir	-0.253224	0.02037024	0.06012888
ENSG0000024	RP11-325L7.1	lncRNA	-0.25334964	0.82091033	0.89038066
ENSG0000018	RNF41	protein_codir	-0.25339999	0.1763842	0.30680204
ENSG0000011	WDR77	protein_codir	-0.25366389	0.08839087	0.18375401
ENSG0000027	AP001505.10	lncRNA	-0.25370565	0.47689782	0.62500203
ENSG0000017	SPATA5L1	protein_codir	-0.25375335	0.07911378	0.16929748
ENSG0000013	LARS1	protein_codir	-0.2537549	0.0318616	0.08517396
ENSG0000013	TOR1B	protein_codir	-0.25380255	0.03192045	0.08522405
ENSG0000017	COMMD5	protein_codir	-0.25382134	0.00446543	0.01856619
ENSG0000000	ALS2	protein_codir	-0.25391489	0.04322407	0.107787
ENSG0000013	VPS4A	protein_codir	-0.25392613	0.02896258	0.07932042
ENSG0000016	BOLA3	protein_codir	-0.2539448	0.17306218	0.30276017
ENSG0000011	TNNI3K	protein_codir	-0.25398146	0.73498816	0.83000952
ENSG0000011	HMGCS1	protein_codir	-0.2540033	0.36510704	0.51897774
ENSG0000014	RUSF1	protein_codir	-0.25400993	0.01765706	0.05388963
ENSG0000016	FOXK1	protein_codir	-0.25410861	0.11720387	0.22697874
ENSG0000019	ZNF786	protein_codir	-0.25424358	0.37473104	0.52829969
ENSG0000027	RP11-74E22.8	lncRNA	-0.25432684	0.72986488	0.82621698
ENSG0000016	ATP5PD	protein_codir	-0.25435869	0.03794918	0.09735801
ENSG0000022	AC073326.3	lncRNA	-0.25439095	0.85522079	0.91336696
ENSG0000016	CENPX	protein_codir	-0.25441763	0.23764179	0.38171946
ENSG0000016	JOSD2	protein_codir	-0.25442141	0.09610975	0.1957778
ENSG0000014	LIN7C	protein_codir	-0.25444718	0.06446516	0.14606461
ENSG0000016	CHCHD4	protein_codir	-0.25451702	0.11286408	0.22074111
ENSG0000025	FPGT-TNNI3K	protein_codir	-0.25464072	0.48407744	0.63147494
ENSG0000013	CDK8	protein_codir	-0.25466124	0.04531169	0.11184171
ENSG0000022	DIP2A-IT1	lncRNA	-0.25479744	0.86419063	0.91911928
ENSG0000018	MRPL30	protein_codir	-0.25489287	0.093194	0.19131992
ENSG0000018	LHFPL6	protein_codir	-0.25498706	0.49935482	0.64484189
ENSG0000013	EGLN1	protein_codir	-0.25503929	0.1766186	0.30707577
ENSG0000010	ARHGEF7	protein_codir	-0.25504482	0.06071555	0.13925652
ENSG0000016	ANKRD33	protein_codir	-0.25512879	0.83062998	0.89799593

ENSG0000011	SLC25A12	protein_codir	-0.25514746	0.24067791	0.38518506
ENSG0000018	TRAPPC6B	protein_codir	-0.25527782	0.05811814	0.13462118
ENSG0000017	TAF7	protein_codir	-0.255515	0.05367317	0.12694407
ENSG0000014	SURF2	protein_codir	-0.25558666	0.03556509	0.0926793
ENSG0000018	CYP2R1	protein_codir	-0.25562432	0.32407888	0.47637353
ENSG0000019	LINC01560	lncRNA	-0.25565636	0.31618709	0.46792476
ENSG0000017	PTDSS2	protein_codir	-0.25566223	0.01524639	0.04831407
ENSG0000017	SAMD12	protein_codir	-0.25570549	0.47728816	0.62533556
ENSG0000017	DDX10	protein_codir	-0.25592671	0.01947869	0.0581246
ENSG0000011	FBXO3	protein_codir	-0.25604403	0.04445038	0.11010936
ENSG0000017	TMEM126B	protein_codir	-0.25617742	0.00553736	0.02194931
ENSG0000026	AP000654.4	lncRNA	-0.25628678	0.7791579	0.86177884
ENSG0000027	RP11-840I19.!	lncRNA	-0.25638918	0.77587188	0.85938327
ENSG0000009	NAT14	protein_codir	-0.25646231	0.10187049	0.20461918
ENSG0000011	PYROXD2	protein_codir	-0.25653517	0.34482577	0.49789376
ENSG0000017	ZMAT3	protein_codir	-0.2566947	0.24815233	0.39365731
ENSG0000020	AKT1S1	protein_codir	-0.25671415	0.04627393	0.1134463
ENSG0000013	ATXN10	protein_codir	-0.25677218	0.06403025	0.14531761
ENSG0000006	ROGDI	protein_codir	-0.25686467	0.23430943	0.37768465
ENSG0000010	VAT1	protein_codir	-0.25694877	0.31956324	0.47162946
ENSG0000012	KLHDC10	protein_codir	-0.25704126	0.06686092	0.15013809
ENSG0000016	PEX13	protein_codir	-0.25721737	0.09482544	0.19387657
ENSG0000003	RPL26L1	protein_codir	-0.25723884	0.07172312	0.15784819
ENSG0000027	RP11-700J17.	lncRNA	-0.25729118	0.52922339	0.67069606
ENSG0000024	CTD-2631K10	lncRNA	-0.25745056	0.8142684	0.88579941
ENSG0000017	FUT10	protein_codir	-0.25771236	0.26430868	0.41180408
ENSG0000011	TRIM23	protein_codir	-0.25773257	0.25406862	0.40046654
ENSG0000010	PON2	protein_codir	-0.25776035	0.14710713	0.26858136
ENSG0000010	TRIM35	protein_codir	-0.25779857	0.0654944	0.14783879
ENSG0000024	LINC00893	lncRNA	-0.25790462	0.46254004	0.61260938
ENSG0000016	PCYT1A	protein_codir	-0.25796492	0.06881792	0.15301489
ENSG0000007	FGF20	protein_codir	-0.25849681	0.77512234	0.85892045
ENSG0000019	AKR1B10	protein_codir	-0.25867905	0.67836942	0.78829979
ENSG0000023	CTD-2619J13.	lncRNA	-0.25869555	0.28535202	0.4349091
ENSG0000012	PCK1	protein_codir	-0.25872871	0.76563077	0.85230526
ENSG0000016	POLR3D	protein_codir	-0.25873711	0.05663215	0.13207633
ENSG0000015	CCT8	protein_codir	-0.25891741	0.08738706	0.18221581
ENSG0000018	SETD3	protein_codir	-0.25902286	0.15559742	0.27980993
ENSG0000002	RNF10	protein_codir	-0.25921106	0.0142461	0.04580644
ENSG0000010	YKT6	protein_codir	-0.25923705	0.12575902	0.23938415
ENSG0000007	RPS6KA2	protein_codir	-0.25929168	0.32678501	0.4790269
ENSG0000020	EIF3CL	protein_codir	-0.25930925	0.3135368	0.46521012
ENSG0000011	RHOQ	protein_codir	-0.25947542	0.3243648	0.47669234
ENSG0000027	AC002550.6	lncRNA	-0.25972209	0.70821074	0.81057795
ENSG0000016	ZFYVE1	protein_codir	-0.25973701	0.07574806	0.16407823
ENSG0000013	C19orf12	protein_codir	-0.25992806	0.01757799	0.05370027
ENSG0000027	RP11-73M18.	lncRNA	-0.25993403	0.48991064	0.63666014

ENSG0000008	ULK2	protein_codir	-0.25993581	0.22054456	0.36130295
ENSG0000013	ZCCHC9	protein_codir	-0.2599883	0.01739111	0.05326485
ENSG0000017	ZNF621	protein_codir	-0.26005907	0.01937945	0.05792477
ENSG0000017	FABP4	protein_codir	-0.26005943	0.6910231	0.79721174
ENSG0000021	DHRS4-AS1	lncRNA	-0.26009118	0.13781807	0.25572959
ENSG0000025	RP11-770J1.5	lncRNA	-0.26016829	0.87487026	NA
ENSG0000026	ANXA8	protein_codir	-0.26020139	0.80599008	0.87979011
ENSG0000017	VEGFB	protein_codir	-0.26030489	0.21137617	0.35079072
ENSG0000013	MRPL34	protein_codir	-0.26049624	0.10631953	0.21127813
ENSG0000018	WWOX	protein_codir	-0.26051163	0.04002329	0.10159391
ENSG0000013	NCBP1	protein_codir	-0.26052977	0.19366004	0.32906123
ENSG0000010	DMAC2	protein_codir	-0.26062659	0.00094741	0.00548581
ENSG0000016	IQCD	protein_codir	-0.26068356	0.41080543	0.56374253
ENSG0000012	HECTD3	protein_codir	-0.26095157	0.01255767	0.04148941
ENSG0000014	EML3	protein_codir	-0.26100669	0.08864088	0.18420444
ENSG0000020	COL11A2	protein_codir	-0.26100748	0.35704598	0.51065916
ENSG0000014	CLBA1	protein_codir	-0.26101663	0.18703639	0.32052772
ENSG0000007	FAM3A	protein_codir	-0.26103228	0.07304452	0.15994052
ENSG0000010	TRPS1	protein_codir	-0.26111539	0.19909039	0.3355224
ENSG0000017	B4GAT1	protein_codir	-0.261618	0.20066858	0.33744086
ENSG0000027	PPP2CA-DT	lncRNA	-0.26164967	0.5513038	0.68828871
ENSG0000015	MRPL10	protein_codir	-0.261673	0.0284565	0.07821361
ENSG0000016	SUCLG1	protein_codir	-0.26170901	0.01876169	0.05646703
ENSG0000010	AIMP2	protein_codir	-0.26171476	0.10957256	0.21609383
ENSG0000027	RP5-1126H10	lncRNA	-0.26194317	0.46052831	0.61097036
ENSG0000016	DDB1	protein_codir	-0.26195424	0.06798277	0.15188614
ENSG0000013	PRRG4	protein_codir	-0.26205908	0.32201706	0.47420085
ENSG0000026	SNHG30	lncRNA	-0.26214688	0.32265193	0.4747726
ENSG0000025	CTD-2651B20	lncRNA	-0.26236137	0.53314352	0.67393245
ENSG0000017	DDIT3	protein_codir	-0.262485	0.35750483	0.51101193
ENSG0000020	RP11-176H8.1	protein_codir	-0.26277192	0.545656	0.68361692
ENSG0000017	FRMD5	protein_codir	-0.26289515	0.59796404	0.72619122
ENSG0000026	CTD-2619J13.	lncRNA	-0.26292875	0.44580574	0.59674618
ENSG0000021	LEPROT	protein_codir	-0.26299173	0.18869079	0.32260542
ENSG0000022	HCG11	lncRNA	-0.26306084	0.20150369	0.3385566
ENSG0000017	GTPBP6	protein_codir	-0.26323283	0.0667682	0.14997795
ENSG0000022	FGD5-AS1	lncRNA	-0.2634083	0.1746142	0.30468022
ENSG0000025	RP11-707G14	lncRNA	-0.26345398	0.57477688	0.70739406
ENSG0000026	ZNF132-DT	lncRNA	-0.26359595	0.66564375	0.77912787
ENSG0000023	IPO9-AS1	lncRNA	-0.26361526	0.55834209	0.69393909
ENSG0000010	PSMD10	protein_codir	-0.26367119	0.01535279	0.04853429
ENSG0000009	AARS1	protein_codir	-0.26372086	0.04211461	0.10567871
ENSG0000013	EIF4E2	protein_codir	-0.26375942	0.01141981	0.03864906
ENSG0000014	PYURF	protein_codir	-0.26397217	0.04395635	0.10911066
ENSG0000025	PIGY	protein_codir	-0.26397217	0.04395635	0.10911066
ENSG0000012	RHOJ	protein_codir	-0.26408244	0.29611603	0.4465832
ENSG0000008	MID2	protein_codir	-0.26412928	0.33926129	0.49211684

ENSG0000018	KCNK4	protein_codir	-0.26414789	0.70838511	0.81057795
ENSG0000014	GOLGA7	protein_codir	-0.26435823	0.01092885	0.03737214
ENSG0000014	MDH2	protein_codir	-0.26439971	0.0688948	0.15316118
ENSG0000013	EMC8	protein_codir	-0.26448121	0.02148339	0.06265137
ENSG0000013	PGPEP1	protein_codir	-0.26459968	0.1251062	0.23843729
ENSG0000027	RP4-539M6.2	lncRNA	-0.26462801	0.85540799	0.9134494
ENSG0000012	DCAF10	protein_codir	-0.26465316	0.07851024	0.16830586
ENSG0000016	NNMT	protein_codir	-0.26472042	0.39983275	0.55315186
ENSG0000026	CAPN10-DT	lncRNA	-0.26480758	0.49819883	0.64393522
ENSG0000012	NEDD8	protein_codir	-0.26484578	0.03475828	0.09107049
ENSG0000024	DENND6A-DT	lncRNA	-0.26488108	0.79852117	0.87474689
ENSG0000013	IGHMBP2	protein_codir	-0.26493108	0.10639382	0.21137946
ENSG0000005	CHRDL2	protein_codir	-0.26513084	0.62111616	0.74429383
ENSG0000025	RP11-977G19	lncRNA	-0.26519765	0.7391928	0.83324012
ENSG0000022	UBXN7-AS1	lncRNA	-0.26529953	0.86597287	0.9203456
ENSG0000026	AC005253.4	lncRNA	-0.26536597	0.77308165	0.85732414
ENSG0000028	CTC-325H20.8	lncRNA	-0.26539386	0.83961376	0.90399299
ENSG0000026	LINC00667	lncRNA	-0.26564773	0.10968811	0.21622909
ENSG0000013	SEC61G	protein_codir	-0.2656516	0.12263367	0.23492453
ENSG0000023	LINC00941	lncRNA	-0.2656873	0.88168929	0.9298659
ENSG0000015	TIMM8B	protein_codir	-0.2657854	0.07474352	0.16248848
ENSG0000013	HTR2B	protein_codir	-0.26581457	0.53063313	0.67195851
ENSG0000011	ADPRS	protein_codir	-0.26590029	0.03549493	0.09255757
ENSG0000004	PTCD2	protein_codir	-0.26598646	0.20366679	0.34100852
ENSG0000018	KRT10	protein_codir	-0.26601273	0.03476311	0.09107058
ENSG0000012	RAB22A	protein_codir	-0.26604985	0.10987597	0.21651468
ENSG0000009	MYO9B	protein_codir	-0.2660986	0.14945461	0.271682
ENSG0000023	LINC01160	lncRNA	-0.26634184	0.59712878	0.7253044
ENSG0000012	ZFP30	protein_codir	-0.26639975	0.26968942	0.41751145
ENSG0000015	VPS26B	protein_codir	-0.26650641	0.02881607	0.0790132
ENSG0000015	ZKSCAN2	protein_codir	-0.26659126	0.1277284	0.24193673
ENSG0000027	RP11-545P7.9	lncRNA	-0.2666888	0.72652763	0.82389002
ENSG0000012	CEP89	protein_codir	-0.26670549	0.02291184	0.06588495
ENSG0000022	PPM1F-AS1	lncRNA	-0.26676814	0.21590491	0.3561451
ENSG0000016	BDH2	protein_codir	-0.26687392	0.26689651	0.4144887
ENSG0000027	RP11-530C5.4	lncRNA	-0.26731292	0.61427282	0.73849635
ENSG0000006	SNCAIP	protein_codir	-0.26737222	0.54895791	0.68644592
ENSG0000016	PGRMC2	protein_codir	-0.26759073	0.1583396	0.28348608
ENSG0000010	AKT2	protein_codir	-0.26769052	0.00011417	0.00097443
ENSG0000019	ZNF780A	protein_codir	-0.26769166	0.2114142	0.35083275
ENSG0000016	RRAD	protein_codir	-0.26771873	0.54643567	0.68432919
ENSG0000016	SMIM14	protein_codir	-0.26774837	0.17704281	0.30751063
ENSG0000026	LINC02175	lncRNA	-0.26783377	0.46044206	0.61095168
ENSG0000008	NSFL1C	protein_codir	-0.26788693	0.07977318	0.17026367
ENSG0000000	ST7L	protein_codir	-0.26792737	0.19332706	0.32861674
ENSG0000014	PLK2	protein_codir	-0.26793618	0.31448551	0.46639228
ENSG0000019	CAPZA2	protein_codir	-0.26797089	0.15781489	0.28285836

ENSG0000017	PC	protein_codir	-0.26800471	0.36125698	0.5151463
ENSG0000010	MOSPD3	protein_codir	-0.26816221	0.06071796	0.13925652
ENSG0000011	ORC4	protein_codir	-0.26817145	0.10253461	0.20565705
ENSG0000008	ZW10	protein_codir	-0.26821784	0.03449308	0.0906124
ENSG0000017	RRS1	protein_codir	-0.26828167	0.14341702	0.26351638
ENSG0000015	SRP19	protein_codir	-0.26834222	0.01861161	0.05612543
ENSG0000017	GAL3ST3	protein_codir	-0.26835804	0.73275777	0.82840529
ENSG0000016	ATP5ME	protein_codir	-0.26840367	0.13305381	0.24950413
ENSG0000026	AC002985.3	protein_codir	-0.26846839	0.872019	NA
ENSG0000001	NR1H4	protein_codir	-0.26848291	0.88885092	NA
ENSG0000014	DEGS1	protein_codir	-0.26850194	0.14132433	0.26071186
ENSG0000025	COA8	protein_codir	-0.26853344	0.06637503	0.14933842
ENSG0000011	HDLBP	protein_codir	-0.26856039	0.07024864	0.15543279
ENSG0000013	SH3BGRL	protein_codir	-0.2686123	0.19442659	0.32989695
ENSG0000011	SENP6	protein_codir	-0.26867054	0.05005995	0.12061903
ENSG0000015	PPP4R1	protein_codir	-0.26867385	0.16100518	0.28693864
ENSG0000025	RP11-282K24.	lncRNA	-0.26873102	0.73515154	0.83009221
ENSG0000022	LINC01736	lncRNA	-0.26873719	0.78067855	0.86289827
ENSG0000012	COPS5	protein_codir	-0.26875168	0.0097213	0.03403391
ENSG0000015	KCTD18	protein_codir	-0.26897346	0.10102781	0.2033366
ENSG0000015	UBE3B	protein_codir	-0.26900692	0.01092418	0.03736079
ENSG0000018	CMC4	protein_codir	-0.26904717	0.12030383	0.23139218
ENSG0000027	CTD-2515H24	lncRNA	-0.26946874	0.68360011	0.79215586
ENSG0000010	CHRAC1	protein_codir	-0.26950352	0.14490245	0.26566251
ENSG0000011	KAT2B	protein_codir	-0.26951322	0.11088461	0.21801202
ENSG0000012	CRYGN	protein_codir	-0.26957664	0.61382167	0.73820293
ENSG0000023	CPB2-AS1	lncRNA	-0.2695869	0.60178206	0.72880911
ENSG0000016	NDUFS5	protein_codir	-0.26963497	0.2621293	0.40964192
ENSG0000014	UTP4	protein_codir	-0.26976356	0.14697841	0.26848834
ENSG0000011	TXNDC15	protein_codir	-0.26993267	0.06850032	0.15252973
ENSG0000010	TBC1D19	protein_codir	-0.26993669	0.20303915	0.34020513
ENSG0000016	VIRMA	protein_codir	-0.27001023	0.06878601	0.15296443
ENSG0000028	CNTNAP3C	protein_codir	-0.27023132	0.70306929	0.80661773
ENSG0000010	ZKSCAN1	protein_codir	-0.27027193	0.13513586	0.25213841
ENSG0000027	RP11-96C23.1	lncRNA	-0.27029097	0.83844299	0.90316843
ENSG0000018	RPS19BP1	protein_codir	-0.27030666	0.03458971	0.09077129
ENSG0000019	ZNF69	protein_codir	-0.27040879	0.11159526	0.21901947
ENSG0000013	CERS5	protein_codir	-0.27050601	0.00501035	0.02030013
ENSG0000014	GSN	protein_codir	-0.2705526	0.56399204	0.6983216
ENSG0000015	CFAP298	protein_codir	-0.27056403	0.00701159	0.02638061
ENSG0000009	TXNL1	protein_codir	-0.27061949	0.03246239	0.0864037
ENSG0000026	TIMM23	protein_codir	-0.2707079	0.04500646	0.11121753
ENSG0000016	FUNDC2	protein_codir	-0.27072262	0.12515204	0.23849175
ENSG0000028	RP11-589M4.	lncRNA	-0.27075982	0.54387681	0.68216146
ENSG0000020	ATXN2	protein_codir	-0.27084142	0.05602434	0.13100171
ENSG0000024	CDK11B	protein_codir	-0.27085648	0.17462174	0.30468022
ENSG0000019	STK39	protein_codir	-0.27086106	0.07067475	0.15617542

ENSG0000010	POP1	protein_codir	-0.2710294	0.30902123	0.46063826
ENSG0000018	IFT140	protein_codir	-0.27104026	0.01903445	0.05709509
ENSG0000026	CTD-2196E14.	lncRNA	-0.27105846	0.5220266	0.66456373
ENSG0000013	RAB15	protein_codir	-0.27107639	0.28626449	0.43580412
ENSG0000018	ALG12	protein_codir	-0.27111351	0.00929006	0.0328837
ENSG0000008	MAP3K4	protein_codir	-0.27126608	0.005692	0.02240139
ENSG0000011	METAP2	protein_codir	-0.2712667	0.04119052	0.10383136
ENSG0000018	COL4A1	protein_codir	-0.27143413	0.47524233	0.62354249
ENSG0000013	SCARB2	protein_codir	-0.27147556	0.26314462	0.41053177
ENSG0000016	RANBP3L	protein_codir	-0.27158727	0.48636105	0.63358608
ENSG0000016	VDAC2	protein_codir	-0.27169165	0.10194066	0.20469655
ENSG0000017	PDCD6IP	protein_codir	-0.27204692	0.03194652	0.08527718
ENSG0000028	RP5-1120P11.	lncRNA	-0.27212089	0.40991251	0.56285277
ENSG0000011	B4GALT6	protein_codir	-0.27224417	0.24680278	0.39199171
ENSG0000015	STEAP2	protein_codir	-0.27253357	0.52686757	0.66866159
ENSG0000012	KRT17	protein_codir	-0.27271006	0.64813579	0.76469975
ENSG0000028	RP11-15H20.6	lncRNA	-0.27271833	0.27961468	0.42860078
ENSG0000022	AC079305.10	lncRNA	-0.27275651	0.49558014	0.64151115
ENSG0000010	MAP1LC3A	protein_codir	-0.27276456	0.23195504	0.37474253
ENSG0000016	COG1	protein_codir	-0.27276909	0.0303875	0.08226039
ENSG0000011	KPNA1	protein_codir	-0.27281456	0.13998557	0.25898676
ENSG0000008	MRPL22	protein_codir	-0.27287133	0.05491418	0.1290614
ENSG0000023	HSBP1	protein_codir	-0.27292503	0.00626223	0.02414331
ENSG0000017	TADA3	protein_codir	-0.27308281	0.00549485	0.02181824
ENSG0000013	CSAD	protein_codir	-0.27309238	0.40246813	0.55550296
ENSG0000026	RP11-310J24.	lncRNA	-0.27310049	0.76618207	0.85265114
ENSG0000010	PRMT5	protein_codir	-0.27316465	0.06417131	0.14555042
ENSG0000018	TBL3	protein_codir	-0.2734583	0.02255305	0.06511762
ENSG0000020	SAMD5	protein_codir	-0.27351349	0.33225357	0.48498276
ENSG0000024	MGC16275	lncRNA	-0.27352962	0.37918091	0.53259645
ENSG0000011	RHOU	protein_codir	-0.27354263	0.33372705	0.48661921
ENSG0000019	TDRD7	protein_codir	-0.27372526	0.07268181	0.15937374
ENSG0000010	SUPT6H	protein_codir	-0.27376828	0.05159915	0.12324016
ENSG0000016	LRRC14	protein_codir	-0.27381036	0.06043128	0.13869991
ENSG0000016	CD2BP2	protein_codir	-0.27387401	0.01137953	0.03853166
ENSG0000017	STARD6	protein_codir	-0.2739297	0.86931235	NA
ENSG0000025	RP11-692D12	lncRNA	-0.27399922	0.72972828	0.8261428
ENSG0000016	SPINDOC	protein_codir	-0.27408068	0.02861505	0.07856338
ENSG0000028	RP11-477E3.3	lncRNA	-0.27410461	0.47712456	0.62523985
ENSG0000020	GOLGA8O	protein_codir	-0.27416531	0.34644363	0.49949225
ENSG0000026	RP11-1260E13	lncRNA	-0.27435321	0.87041466	NA
ENSG0000018	AIDA	protein_codir	-0.27440393	0.04532203	0.11185399
ENSG0000016	RNF214	protein_codir	-0.274434	0.05141792	0.12293655
ENSG0000016	USP54	protein_codir	-0.27450537	0.23353218	0.37676161
ENSG0000025	RP11-429A20	lncRNA	-0.27452146	0.80748878	0.88073452
ENSG0000008	GCN1	protein_codir	-0.27465912	0.00279878	0.01277089
ENSG0000019	ANKRD13B	protein_codir	-0.27469891	0.29136131	0.44121033

ENSG0000013	RAB1A	protein_codir	-0.27475345	0.07712331	0.16618395
ENSG0000016	ELP6	protein_codir	-0.27477296	0.02926412	0.07996197
ENSG0000018	FZD9	protein_codir	-0.2748325	0.56617238	0.70033057
ENSG0000014	PELP1	protein_codir	-0.27495542	0.02685796	0.07462195
ENSG0000005	ARHGEF5	protein_codir	-0.2753437	0.26958649	0.41742229
ENSG0000015	HOMER1	protein_codir	-0.27538743	0.49209856	0.63871063
ENSG0000027	AC009133.21	lncRNA	-0.27542888	0.74312886	0.8359897
ENSG0000025	HTD2	protein_codir	-0.27547953	0.27053559	0.41842272
ENSG0000017	ARL4D	protein_codir	-0.27579473	0.47083872	0.61986207
ENSG0000010	DDX50	protein_codir	-0.27583226	0.0366893	0.09491931
ENSG0000013	FLOT1	protein_codir	-0.27595119	0.03182889	0.08511123
ENSG0000024	RP11-367J11.	lncRNA	-0.27606957	0.64691209	0.76413257
ENSG0000019	ZNF250	protein_codir	-0.27608187	0.12883805	0.24361655
ENSG0000022	RP11-433J20.	lncRNA	-0.27617583	0.84980634	0.9106386
ENSG0000026	CCNQ	protein_codir	-0.27620427	0.1343599	0.25127094
ENSG0000014	UBE2Q2	protein_codir	-0.27622107	0.09727375	0.19762419
ENSG0000027	CTB-113P19.5	lncRNA	-0.27628999	0.48731031	0.6344636
ENSG0000020	BMPR2	protein_codir	-0.27631569	0.36769933	0.5212876
ENSG0000012	PEPD	protein_codir	-0.27640345	0.02710953	0.0751395
ENSG0000025	RP11-203J24.	protein_codir	-0.27648427	0.31249223	0.4642588
ENSG0000013	CCDC90B	protein_codir	-0.27670189	0.00596833	0.02323401
ENSG0000015	GTF3C6	protein_codir	-0.27676026	0.03708387	0.09570703
ENSG0000010	PFDN4	protein_codir	-0.27681053	0.03047788	0.08245655
ENSG0000013	RABEPK	protein_codir	-0.27682336	0.00418099	0.01766005
ENSG0000017	NME6	protein_codir	-0.27692007	0.06775351	0.15155599
ENSG0000028	RP11-397E7.5	lncRNA	-0.27694516	0.09187177	0.189182
ENSG0000016	OR51E2	protein_codir	-0.27694587	NA	NA
ENSG0000023	RP11-80H5.9	lncRNA	-0.27702987	0.77129076	0.85612871
ENSG0000012	MIF4GD	protein_codir	-0.27705738	0.10524961	0.20965028
ENSG0000022	RP11-4C20.4	lncRNA	-0.27712622	0.82253714	0.89179493
ENSG0000012	SCPEP1	protein_codir	-0.2772234	0.11114489	0.21839939
ENSG0000017	TMEM217	protein_codir	-0.27727342	0.6414699	0.75953322
ENSG0000018	TMEM120B	protein_codir	-0.27736218	0.04334082	0.10797254
ENSG0000023	ZBTB22	protein_codir	-0.277366	0.05824843	0.13484811
ENSG0000010	SETD6	protein_codir	-0.27760628	0.06329917	0.14395415
ENSG0000012	EMC4	protein_codir	-0.27761463	0.00987538	0.03446962
ENSG0000028	RP11-177J6.2	protein_codir	-0.27766913	0.41131327	0.56432728
ENSG0000023	COX10-AS1	lncRNA	-0.27770148	0.17192197	0.30128694
ENSG0000014	MED22	protein_codir	-0.2777515	0.08684281	0.1814902
ENSG0000016	TMEM100	protein_codir	-0.27792951	0.47458322	0.62282998
ENSG0000021	MIR99AHG	lncRNA	-0.27803346	0.49192258	0.63857224
ENSG0000023	CCDC144NL-A	lncRNA	-0.27805621	0.61215171	0.73710132
ENSG0000001	ZNF582	protein_codir	-0.27810374	0.15210682	0.27510666
ENSG0000017	VSIG10	protein_codir	-0.27811599	0.17143661	0.30067812
ENSG0000027	SAP30-DT	lncRNA	-0.2781319	0.30551154	0.45718311
ENSG0000020	RAB12	protein_codir	-0.27814451	0.06178003	0.14118521
ENSG0000016	PCBP1	protein_codir	-0.27821368	0.02689782	0.07471514

ENSG0000000	POMT2	protein_codir	-0.2782422	0.05872848	0.13567085
ENSG0000013	ALKBH8	protein_codir	-0.2783001	0.11909314	0.22971944
ENSG0000018	PLA2G2A	protein_codir	-0.27830223	0.61874527	0.74232232
ENSG0000008	SF3B2	protein_codir	-0.27836174	0.01192036	0.03988374
ENSG0000013	FRS3	protein_codir	-0.27855678	0.16087195	0.28677444
ENSG0000018	MRPS11	protein_codir	-0.27867803	0.0194531	0.0580749
ENSG0000014	GTF2A2	protein_codir	-0.27868803	0.03956413	0.1006504
ENSG0000006	TMEM161A	protein_codir	-0.27897905	0.04327232	0.10786119
ENSG0000022	JPX	lncRNA	-0.27914955	0.21980837	0.36059276
ENSG0000012	GOT2	protein_codir	-0.27916774	0.01256184	0.04149718
ENSG0000012	HERC2	protein_codir	-0.27926985	0.12327791	0.23586137
ENSG0000014	TERF1	protein_codir	-0.27950809	0.02577401	0.07230124
ENSG0000024	SUCLG2-AS1	lncRNA	-0.27989921	0.1595004	0.28508387
ENSG0000013	C2orf49	protein_codir	-0.27992922	0.16539244	0.2927919
ENSG0000019	ZFP62	protein_codir	-0.28003149	0.18763298	0.32137065
ENSG0000016	TAF3	protein_codir	-0.28003908	0.01583704	0.04972933
ENSG0000002	AGPAT4	protein_codir	-0.28005811	0.20325233	0.34054167
ENSG0000014	TIPRL	protein_codir	-0.28006958	0.04175362	0.10499535
ENSG0000005	MAPK9	protein_codir	-0.28010719	0.12167702	0.23349679
ENSG0000024	KCTD7	protein_codir	-0.28018799	0.0692009	0.15368085
ENSG0000011	ASH1L	protein_codir	-0.2803952	0.17495324	0.30506367
ENSG0000018	RBM43	protein_codir	-0.28041668	0.29379103	0.44407094
ENSG0000010	RPAP1	protein_codir	-0.28046111	0.03201689	0.08540723
ENSG0000020	ASPDH	protein_codir	-0.28055627	0.86874157	NA
ENSG0000015	ATP5PF	protein_codir	-0.28083887	0.04968252	0.11995029
ENSG0000017	THAP5	protein_codir	-0.28094388	0.09146006	0.18848879
ENSG0000023	ZBED9	protein_codir	-0.2809478	0.60438495	0.73093582
ENSG0000013	NABP2	protein_codir	-0.28098912	0.01659286	0.05145909
ENSG0000000	CYP51A1	protein_codir	-0.28099048	0.28407647	0.43370692
ENSG0000012	INSIG2	protein_codir	-0.28101616	0.11674445	0.22635884
ENSG0000022	LINC00184	lncRNA	-0.2810347	0.83270867	0.89928607
ENSG0000022	RP11-483F11.	lncRNA	-0.28113793	0.85138836	NA
ENSG0000027	BACE1-AS	lncRNA	-0.28116718	0.17481417	0.30491742
ENSG0000010	GCDH	protein_codir	-0.28117171	0.0329526	0.08746404
ENSG0000018	GLRX5	protein_codir	-0.28126116	0.04701829	0.11478215
ENSG0000016	B3GALNT1	protein_codir	-0.28142829	0.28977782	0.43940375
ENSG0000028	NPTN-IT1	lncRNA	-0.28144213	0.39598072	0.54888024
ENSG0000019	SPRED2	protein_codir	-0.28167132	0.16167364	0.2878274
ENSG0000019	TBKBP1	protein_codir	-0.28172517	0.09290372	0.19088188
ENSG0000016	NSMCE1	protein_codir	-0.28172887	0.0038946	0.0166515
ENSG0000026	RP11-798G7.6	lncRNA	-0.28177541	0.44053698	0.59153237
ENSG0000006	MAST4	protein_codir	-0.281936	0.16402838	0.29102999
ENSG0000016	RRM1	protein_codir	-0.28207718	0.04365869	0.10857673
ENSG0000007	SMG6	protein_codir	-0.28212011	0.08046253	0.17142665
ENSG0000026	RP11-517B11.	lncRNA	-0.28216523	0.26281996	0.41015076
ENSG0000014	EPHX1	protein_codir	-0.28233874	0.27801044	0.42659881
ENSG0000027	RP11-170N16	lncRNA	-0.28236073	0.62425841	0.74660159

ENSG0000016	ATP7A	protein_codir	-0.28239298	0.14619942	0.26747221
ENSG0000010	CERK	protein_codir	-0.2824305	0.06370365	0.14470721
ENSG0000026	LINC02256	lncRNA	-0.28279354	0.33243909	0.48515099
ENSG0000016	ANKRD2	protein_codir	-0.2829802	0.59492223	0.72341956
ENSG0000015	SDHAF4	protein_codir	-0.28298215	0.03698513	0.09551465
ENSG0000007	AP1M1	protein_codir	-0.28298509	0.00080927	0.00483572
ENSG0000010	BCCIP	protein_codir	-0.28304866	0.04463235	0.11045118
ENSG0000010	TJP1	protein_codir	-0.28309059	0.29211425	0.44214136
ENSG0000019	ZFP2	protein_codir	-0.28309861	0.4083674	0.56131719
ENSG0000013	ZNF280D	protein_codir	-0.28310015	0.09909405	0.20039374
ENSG0000011	HTRA2	protein_codir	-0.28324385	0.02121691	0.06202487
ENSG0000026	RP11-432I5.8	lncRNA	-0.28336557	0.74340042	0.83615829
ENSG0000010	EXOSC3	protein_codir	-0.28337496	0.01642469	0.05106369
ENSG0000014	DCAF6	protein_codir	-0.28346802	0.04889275	0.11841616
ENSG0000012	SEC16B	protein_codir	-0.28347414	0.27758635	0.42608287
ENSG0000019	TSEN15	protein_codir	-0.28360562	0.02848989	0.07828979
ENSG0000016	RNF187	protein_codir	-0.28375079	0.00371872	0.01602605
ENSG0000025	ALG11	protein_codir	-0.2838667	0.19444172	0.32990235
ENSG0000009	PITPNM2	protein_codir	-0.28390346	0.09904875	0.20031679
ENSG0000019	ZKSCAN5	protein_codir	-0.28405392	0.00466925	0.01925135
ENSG0000019	ZNF605	protein_codir	-0.28414563	0.1006761	0.20271736
ENSG0000012	DUT	protein_codir	-0.28425387	0.00015294	0.0012446
ENSG0000014	WDR61	protein_codir	-0.28445147	0.03587669	0.09328731
ENSG0000010	MYL12A	protein_codir	-0.28448885	0.25116784	0.39725452
ENSG0000019	ZKSCAN7	protein_codir	-0.28471465	0.31460295	0.46650828
ENSG0000013	MYG1	protein_codir	-0.28471634	0.01383298	0.04473328
ENSG0000026	RP11-57H14.4	lncRNA	-0.28474015	0.06987278	0.1548741
ENSG0000011	FCF1	protein_codir	-0.28480336	0.02471138	0.06995985
ENSG0000028	RP1-166D18.1	lncRNA	-0.28494098	0.83538833	0.90109449
ENSG0000016	UBTD1	protein_codir	-0.28507102	0.13002717	0.24512663
ENSG0000023	AC113189.5	lncRNA	-0.28516313	0.16620176	0.29400635
ENSG0000023	AF064858.10	lncRNA	-0.28534114	0.8110619	0.88337591
ENSG0000027	GS1-279B7.2	lncRNA	-0.28540861	0.68898164	0.79585433
ENSG0000017	FIBP	protein_codir	-0.28542561	0.08222125	0.17412597
ENSG0000028	RP11-260L6.2	lncRNA	-0.28543413	0.69577868	0.80072482
ENSG0000018	GET1	protein_codir	-0.28544193	0.0110775	0.03774276
ENSG0000010	CCDC9	protein_codir	-0.28548037	0.19994156	0.33655781
ENSG0000026	RNF213-AS1	lncRNA	-0.28549357	0.37817705	0.5317814
ENSG0000010	CYP2C18	protein_codir	-0.28549507	0.86734066	NA
ENSG0000018	NDUFA13	protein_codir	-0.28566183	0.02476977	0.07008923
ENSG0000028	XXyac-YM960	lncRNA	-0.28572902	0.6896266	0.79628497
ENSG0000015	RMND1	protein_codir	-0.28573926	0.05368419	0.12694839
ENSG0000010	LENG1	protein_codir	-0.28592271	0.01035768	0.03579569
ENSG0000017	NANP	protein_codir	-0.28613555	0.15787326	0.28288235
ENSG0000015	PANK1	protein_codir	-0.28635827	0.17867226	0.30970301
ENSG0000015	CFAP251	protein_codir	-0.28636031	0.44853578	0.59940769
ENSG0000017	YWHAG	protein_codir	-0.28636166	0.19813465	0.33438097

ENSG0000020	RNF5	protein_codir	-0.28639116	0.04684993	0.11450269
ENSG0000028	RP1-191N21.5	lncRNA	-0.28640543	0.88354752	NA
ENSG0000028	PRRC2B	protein_codir	-0.2865288	0.02113589	0.06186006
ENSG0000016	KLHL26	protein_codir	-0.2866159	0.1557732	0.28005285
ENSG0000013	GRSF1	protein_codir	-0.28685846	0.0306711	0.08284946
ENSG0000016	NUDT8	protein_codir	-0.28694177	0.25176241	0.39792147
ENSG0000024	TIPARP-AS1	lncRNA	-0.28707204	0.57720363	0.70951757
ENSG0000017	ASB8	protein_codir	-0.28712704	0.02184976	0.06349188
ENSG0000014	SS18	protein_codir	-0.28725559	0.01156392	0.0390173
ENSG0000015	CCT5	protein_codir	-0.28735196	0.09784945	0.19851636
ENSG0000010	PLEKHA1	protein_codir	-0.28736947	0.03370104	0.08902282
ENSG0000018	NOP10	protein_codir	-0.2874759	0.03532459	0.09220263
ENSG0000013	TSPAN31	protein_codir	-0.28760446	0.00470291	0.01934969
ENSG0000014	METTL14	protein_codir	-0.28782955	0.02004868	0.05940827
ENSG0000017	USP47	protein_codir	-0.28791073	0.04810601	0.11694138
ENSG0000000	ARHGAP44	protein_codir	-0.28807038	0.18326801	0.31585314
ENSG0000007	TUBE1	protein_codir	-0.28814892	0.09734609	0.19775662
ENSG0000027	RP11-15A1.9	lncRNA	-0.28824364	0.76079746	0.84891633
ENSG0000022	AC003986.5	lncRNA	-0.2886764	0.8093086	0.88216203
ENSG0000026	CTB-31O20.4	lncRNA	-0.28869202	0.77517867	0.85892045
ENSG0000011	CHORDC1	protein_codir	-0.28880306	0.14662311	0.26807239
ENSG0000016	DEPDC4	protein_codir	-0.28896018	0.42853491	0.58020266
ENSG0000015	CCDC74B	protein_codir	-0.28896451	0.42579128	0.57767733
ENSG0000012	MTUS1	protein_codir	-0.28902194	0.28262383	0.4321341
ENSG0000014	PIGM	protein_codir	-0.28913618	0.052038	0.12394702
ENSG0000027	RP11-884K10.	lncRNA	-0.2891686	0.03333961	0.08822841
ENSG0000008	METTL2A	protein_codir	-0.28921352	0.00922046	0.03268761
ENSG0000019	ANXA6	protein_codir	-0.28924978	0.10269724	0.20590141
ENSG0000015	EFCAB14	protein_codir	-0.28932984	0.09527275	0.19451755
ENSG0000021	SLC35F6	protein_codir	-0.28960633	0.05762419	0.13371252
ENSG0000016	WDR41	protein_codir	-0.28963865	0.03308199	0.08772317
ENSG0000020	MRPL38	protein_codir	-0.2899109	0.04905248	0.11873013
ENSG0000006	LAPTM4A	protein_codir	-0.28994226	0.12704045	0.24107573
ENSG0000016	VKORC1	protein_codir	-0.28995576	0.05454623	0.12844803
ENSG0000008	GEMIN5	protein_codir	-0.28999528	0.10969902	0.21623516
ENSG0000019	TRAPPC4	protein_codir	-0.29006696	0.05574888	0.1305123
ENSG0000016	TM2D1	protein_codir	-0.29012649	0.04960769	0.11980104
ENSG0000001	MDH1	protein_codir	-0.29016908	0.02063106	0.06074945
ENSG0000026	RP11-799B12.	lncRNA	-0.29043599	0.63709004	0.75667284
ENSG0000027	RP11-402D21	lncRNA	-0.29055516	0.68534332	0.79330193
ENSG0000013	RNASEL	protein_codir	-0.29060143	0.25387594	0.40027705
ENSG0000019	OGA	protein_codir	-0.2906697	0.1155853	0.22464753
ENSG0000017	UBE2E3	protein_codir	-0.29088524	0.04993287	0.12040737
ENSG0000021	TSN	protein_codir	-0.29089176	0.0662779	0.14916175
ENSG0000018	KATNA1	protein_codir	-0.29105072	0.01587436	0.04982385
ENSG0000016	HPS6	protein_codir	-0.29108676	0.14958639	0.27179633
ENSG0000015	CDK20	protein_codir	-0.2911002	0.15163601	0.27441908

ENSG0000025	CTA-204B4.2	lncRNA	-0.29110761	0.31309042	0.46479749
ENSG0000006	NUCKS1	protein_codir	-0.29125625	0.08625489	0.18056389
ENSG0000016	ZNF143	protein_codir	-0.29129604	0.05391895	0.12736178
ENSG0000023	RP11-403I13.!	lncRNA	-0.29141309	0.38850873	0.54148507
ENSG0000007	NLE1	protein_codir	-0.29142506	0.01730704	0.05307209
ENSG0000013	SERINC3	protein_codir	-0.29142651	0.1213772	0.23315717
ENSG0000026	RP11-452L6.1	lncRNA	-0.29157685	0.52734874	0.66901646
ENSG0000010	TTC26	protein_codir	-0.29158109	0.15416418	0.27797459
ENSG0000025	LINC02718	lncRNA	-0.2916264	0.69163872	0.797556
ENSG0000010	RAB3D	protein_codir	-0.29166199	0.02979256	0.08103906
ENSG0000013	PLAA	protein_codir	-0.29169764	0.06430039	0.14572716
ENSG0000021	FAM66B	lncRNA	-0.29203901	0.29519021	0.44556779
ENSG0000025	NAV2-IT1	lncRNA	-0.29223523	0.89594496	NA
ENSG0000017	SCG2	protein_codir	-0.29240363	0.653406	0.76904051
ENSG0000025	CTD-2651B20	lncRNA	-0.29247453	0.70548784	0.8084965
ENSG0000006	THUMPD1	protein_codir	-0.29255239	0.00654962	0.02499901
ENSG0000010	PFN1	protein_codir	-0.29271497	0.14395367	0.26416853
ENSG0000023	FOXP4-AS1	lncRNA	-0.29273464	0.7955995	0.87311344
ENSG0000001	EEF1AKNMT	protein_codir	-0.29279076	0.04573721	0.11249994
ENSG0000011	PRDX1	protein_codir	-0.2929131	0.03127856	0.08403832
ENSG0000009	TBC1D10A	protein_codir	-0.29291505	0.12228558	0.23443658
ENSG0000014	TEX261	protein_codir	-0.29292011	0.02952438	0.08048396
ENSG0000010	MRPL32	protein_codir	-0.29309444	0.09498982	0.19412644
ENSG0000019	ASPH	protein_codir	-0.29320154	0.26523139	0.41283127
ENSG0000014	SRP14	protein_codir	-0.2932668	0.02328237	0.06670737
ENSG0000006	MRPS35	protein_codir	-0.29327309	0.02347456	0.06711877
ENSG0000017	THAP4	protein_codir	-0.29331263	0.04723337	0.11522572
ENSG0000013	NRM	protein_codir	-0.2933621	0.04303995	0.10740545
ENSG0000017	HARS1	protein_codir	-0.29341916	0.00963462	0.03379581
ENSG0000020	LINC00963	lncRNA	-0.29351288	0.08507125	0.17868197
ENSG0000012	MYO1B	protein_codir	-0.29351589	0.22763647	0.36990759
ENSG0000011	PINK1-AS	lncRNA	-0.29357067	0.09745508	0.19794892
ENSG0000013	RIDA	protein_codir	-0.29360643	0.07161259	0.15766243
ENSG0000016	A2ML1	protein_codir	-0.29363469	0.82300396	0.89216097
ENSG0000014	CLTC	protein_codir	-0.29368722	0.22610997	0.36812009
ENSG0000013	DDA1	protein_codir	-0.29373269	0.04839368	0.11744429
ENSG0000013	OSBPL2	protein_codir	-0.29376606	0.07726596	0.16637451
ENSG0000022	RP11-69I8.3	lncRNA	-0.29381713	0.52043836	0.66314803
ENSG0000021	AC005562.1	lncRNA	-0.29397855	0.04833516	0.11733481
ENSG0000013	PSMB7	protein_codir	-0.29417325	0.02613288	0.07308528
ENSG0000021	GSTM2	protein_codir	-0.2943225	0.45769948	0.60821071
ENSG0000026	MIRLET7A1HC	lncRNA	-0.29448777	0.36735486	0.52108007
ENSG0000027	CTD-3025N20	lncRNA	-0.2946281	0.38622592	0.53925588
ENSG0000010	C19orf53	protein_codir	-0.29470442	0.00956501	0.03359856
ENSG0000010	NOL4	protein_codir	-0.29480672	0.60348003	0.73012564
ENSG0000026	ZSCAN16-AS1	lncRNA	-0.29487425	0.08641936	0.18086707
ENSG0000025	RP1-261D10.2	lncRNA	-0.29487963	0.64799363	0.76462529

ENSG0000010	CPPED1	protein_codir	-0.29496146	0.15783337	0.28287019
ENSG0000028	SEPSECS-AS1	lncRNA	-0.2949658	0.35203185	0.50559615
ENSG0000007	DGKD	protein_codir	-0.29501332	0.29219415	0.44222114
ENSG0000016	TSPAN5	protein_codir	-0.29501576	0.06813261	0.15207063
ENSG0000015	AP3S2	protein_codir	-0.29504108	0.0406127	0.10267587
ENSG0000017	ZNHIT2	protein_codir	-0.29518622	0.06100433	0.13971307
ENSG0000026	LINC02516	lncRNA	-0.29522931	0.76651446	0.85281488
ENSG0000019	RASSF9	protein_codir	-0.29529792	0.46688207	0.61649772
ENSG0000018	ALKBH2	protein_codir	-0.29534898	0.04772465	0.11613698
ENSG0000019	NUDT16	protein_codir	-0.29535374	0.0108316	0.0371039
ENSG0000010	TANGO6	protein_codir	-0.29540426	0.01700503	0.05240756
ENSG0000010	ABCA2	protein_codir	-0.29543257	0.06491378	0.14687626
ENSG0000027	BTF3-DT	lncRNA	-0.29545302	0.56052806	0.69560453
ENSG0000027	KB-226F1.2	lncRNA	-0.29547385	0.81760071	0.88794163
ENSG0000021	MRPL23	protein_codir	-0.29550371	0.1796414	0.31100009
ENSG0000016	SNRPD1	protein_codir	-0.29550981	0.04438894	0.1099715
ENSG0000018	RRP7A	protein_codir	-0.29566585	0.1240723	0.23695733
ENSG0000014	EGFR	protein_codir	-0.2956863	0.47195917	0.62070912
ENSG0000013	IMP4	protein_codir	-0.29581311	0.04679786	0.11441595
ENSG0000025	RP11-1081L13	lncRNA	-0.29584775	0.44227311	0.59330629
ENSG0000014	IQSEC1	protein_codir	-0.29586032	0.12657848	0.24044687
ENSG0000011	SNX3	protein_codir	-0.29612565	0.01179475	0.03959297
ENSG0000026	RP11-6O2.4	lncRNA	-0.29627829	0.64394366	0.76170124
ENSG0000011	EIF4G2	protein_codir	-0.29631282	0.07963142	0.17006263
ENSG0000012	TOX2	protein_codir	-0.29636118	0.29828688	0.44912144
ENSG0000014	SAA4	protein_codir	-0.29640212	0.824653	0.89356743
ENSG0000015	SUV39H2	protein_codir	-0.29652993	0.12785454	0.24213813
ENSG0000016	FAM207A	protein_codir	-0.29656989	0.07377438	0.16107892
ENSG0000012	NFYB	protein_codir	-0.29658181	0.23527313	0.37877383
ENSG0000018	NELFB	protein_codir	-0.29677266	0.00729368	0.02717542
ENSG0000024	DECR2	protein_codir	-0.29681404	0.09587532	0.19541184
ENSG0000011	SLC39A7	protein_codir	-0.29691932	0.1052543	0.20965028
ENSG0000010	EARS2	protein_codir	-0.29697417	0.06323324	0.14385158
ENSG0000006	PLPP1	protein_codir	-0.29733221	0.351292	0.50484859
ENSG0000025	ARMS2	protein_codir	-0.29739614	0.85860039	NA
ENSG0000007	SDF4	protein_codir	-0.29739901	0.01330409	0.04339896
ENSG0000013	NREP	protein_codir	-0.29741488	0.1362188	0.25354561
ENSG0000009	TOX4	protein_codir	-0.29747644	0.02802438	0.07724885
ENSG0000009	PSMD5	protein_codir	-0.2975135	0.02319511	0.0665402
ENSG0000014	GLB1L2	protein_codir	-0.29754644	0.46017828	0.61071132
ENSG0000011	TTC1	protein_codir	-0.29766346	0.00782724	0.02870648
ENSG0000010	SLC39A14	protein_codir	-0.2977712	0.38309743	0.53647514
ENSG0000017	KCNMB3	protein_codir	-0.29796975	0.30628138	0.45788859
ENSG0000026	RP5-1139B12.	lncRNA	-0.29806794	0.14308968	0.26305491
ENSG0000010	GABRE	protein_codir	-0.29813648	0.38960272	0.54257183
ENSG0000015	RBM45	protein_codir	-0.29825233	0.12368857	0.23638793
ENSG0000011	SPTBN1	protein_codir	-0.29827348	0.24768214	0.39304686

ENSG0000014	SLC39A1	protein_codir	-0.29829566	0.04655964	0.11401499
ENSG0000027	H3-3A-DT	lncRNA	-0.29833722	0.70880457	0.81086684
ENSG0000016	PIGX	protein_codir	-0.29843727	0.07981557	0.17033736
ENSG0000010	ABHD11	protein_codir	-0.29845326	0.05620988	0.13132438
ENSG0000013	MRPL47	protein_codir	-0.29848168	0.03614453	0.09379986
ENSG0000013	DSTYK	protein_codir	-0.29854046	0.07447624	0.16211158
ENSG0000023	AC073257.1	lncRNA	-0.2985954	0.5876841	0.71797875
ENSG0000015	ZDHHC1	protein_codir	-0.29867234	0.25594737	0.40268158
ENSG0000019	CTD-2192J16.	protein_codir	-0.29927592	0.8072443	0.88060737
ENSG0000016	CCDC126	protein_codir	-0.2993509	0.00887608	0.03169865
ENSG0000012	INTS11	protein_codir	-0.29943876	0.08939783	0.18535907
ENSG0000011	RAD1	protein_codir	-0.29961175	0.06035944	0.13864796
ENSG0000013	CC2D1A	protein_codir	-0.29961649	0.03553781	0.09264316
ENSG0000010	SIX4	protein_codir	-0.29971011	0.32795542	0.48033348
ENSG0000006	IDH3G	protein_codir	-0.29979896	0.0612019	0.14007263
ENSG0000015	VPS37A	protein_codir	-0.29980253	0.10657842	0.21165488
ENSG0000012	MTX2	protein_codir	-0.29984836	0.0098934	0.03451506
ENSG0000012	MRPS14	protein_codir	-0.2998955	0.02137004	0.06239327
ENSG0000024	CKMT2-AS1	lncRNA	-0.29991232	0.1292537	0.24411838
ENSG0000023	AC123886.2	lncRNA	-0.29992004	0.7498083	0.84051097
ENSG0000010	DHODH	protein_codir	-0.30017346	0.05177565	0.12351385
ENSG0000022	PPT2	protein_codir	-0.3001799	0.09593615	0.195453
ENSG0000011	CCT4	protein_codir	-0.3004543	0.02576062	0.07227837
ENSG0000017	GOLIM4	protein_codir	-0.30053878	0.18620022	0.31947137
ENSG0000019	DDX42	protein_codir	-0.30055195	0.04246634	0.10639699
ENSG0000006	YBX1	protein_codir	-0.30056574	0.0431013	0.10751967
ENSG0000025	RP11-46H11.3	lncRNA	-0.30064577	0.33483469	0.48777467
ENSG0000016	RCCD1	protein_codir	-0.3006548	0.10748273	0.21286914
ENSG0000017	MRFAP1L1	protein_codir	-0.30079445	0.00358824	0.01558046
ENSG0000020	ETFRF1	protein_codir	-0.30082301	0.02109483	0.0617857
ENSG0000012	ABCC11	protein_codir	-0.30083104	0.63010245	0.75085985
ENSG0000017	SOX12	protein_codir	-0.30084561	0.15002983	0.27235122
ENSG0000011	FBXW2	protein_codir	-0.30095187	0.02149471	0.06267778
ENSG0000012	FBXL12	protein_codir	-0.30098407	0.00707999	0.02654034
ENSG0000025	RAD21-AS1	lncRNA	-0.30101591	0.65642215	0.77170557
ENSG0000017	NBEA	protein_codir	-0.30115816	0.31656052	0.46828781
ENSG0000016	SEPTIN2	protein_codir	-0.30119406	0.07104305	0.15670093
ENSG0000014	LRSAM1	protein_codir	-0.30125305	0.06542658	0.14775816
ENSG0000014	LNPK	protein_codir	-0.30127147	0.06279938	0.1431003
ENSG0000026	CTD-2350C19	lncRNA	-0.30128474	0.53845865	0.67798267
ENSG0000018	MIGA1	protein_codir	-0.30131213	0.07219617	0.15858583
ENSG0000013	IPO8	protein_codir	-0.30135457	0.04742165	0.11560336
ENSG0000017	DENND2C	protein_codir	-0.30141262	0.17514678	0.30526749
ENSG0000016	ASB5	protein_codir	-0.30147842	0.77340833	0.85744987
ENSG0000009	ESR1	protein_codir	-0.30164181	0.23375048	0.37706565
ENSG0000010	C19orf44	protein_codir	-0.30181539	0.15292394	0.27626132
ENSG0000018	ZNF501	protein_codir	-0.30187928	0.28902745	0.43858483

ENSG0000011	FAM162A	protein_codir	-0.30190523	0.04458849	0.11036241
ENSG0000026	CTD-2207O23	protein_codir	-0.30195794	0.81434535	0.88579941
ENSG0000012	WDR83	protein_codir	-0.30206326	0.01943041	0.05803069
ENSG0000001	IDS	protein_codir	-0.30209533	0.23396679	0.37724243
ENSG0000011	NEK9	protein_codir	-0.30230363	0.03045133	0.08240088
ENSG0000026	RP11-678G15	lncRNA	-0.30240944	0.76339902	0.85066225
ENSG0000024	ZNF709	protein_codir	-0.30259785	0.60281231	0.72970487
ENSG0000026	CTC-344H19.4	lncRNA	-0.30265209	0.61274444	0.73749353
ENSG0000010	PRKAR1A	protein_codir	-0.30277684	0.14056007	0.2598052
ENSG0000013	DMAC1	protein_codir	-0.30279123	0.03084587	0.0831589
ENSG0000015	SEC13	protein_codir	-0.30280346	0.0364835	0.0944931
ENSG0000014	ZSCAN32	protein_codir	-0.30282202	0.09165517	0.18884863
ENSG0000026	ST3GAL1-DT	lncRNA	-0.30283459	0.57362358	0.70644684
ENSG0000012	ZNF133	protein_codir	-0.30289252	0.1004212	0.20233685
ENSG0000027	RP1-86C11.7	lncRNA	-0.30292907	0.65208159	0.76797261
ENSG0000028	BLACAT1	protein_codir	-0.30297814	0.72243103	0.82089707
ENSG0000022	AC078842.3	lncRNA	-0.30320812	0.83815241	NA
ENSG0000017	FAM220A	protein_codir	-0.30326439	0.04815583	0.11700069
ENSG0000008	GNAS	protein_codir	-0.3032644	0.0243868	0.06923253
ENSG0000017	ZDHHC16	protein_codir	-0.30333947	0.0186723	0.05625317
ENSG0000013	SPOCD1	protein_codir	-0.30344046	0.56004822	0.69533992
ENSG0000024	ZNF324B	protein_codir	-0.30347572	0.07429453	0.16185637
ENSG0000022	LINC00377	lncRNA	-0.30349465	0.65718156	0.77210237
ENSG0000001	DNASE1L1	protein_codir	-0.30354028	0.04952382	0.1196508
ENSG0000018	VSIG10L	protein_codir	-0.30365735	0.05605942	0.13103938
ENSG0000023	AF064858.8	lncRNA	-0.30367736	0.48342153	0.63082787
ENSG0000017	SEC24C	protein_codir	-0.30369568	0.00213603	0.01033578
ENSG0000027	RP11-330O11	lncRNA	-0.30378588	0.34895132	0.50229494
ENSG0000004	CTPS2	protein_codir	-0.30396923	0.01127327	0.03823731
ENSG0000018	S100A16	protein_codir	-0.30397759	0.19780178	0.33401663
ENSG0000013	NARS1	protein_codir	-0.30398853	0.0126305	0.04167416
ENSG0000016	HSPA12A	protein_codir	-0.30404908	0.2070398	0.34546241
ENSG0000016	INTS1	protein_codir	-0.30405082	0.02051936	0.06045923
ENSG0000005	PLEKHA5	protein_codir	-0.30409541	0.28276614	0.43224153
ENSG0000018	AP3M1	protein_codir	-0.30418169	0.04185801	0.1051973
ENSG0000017	CYC1	protein_codir	-0.30419948	0.03240677	0.08629724
ENSG0000019	CACNA1H	protein_codir	-0.30424516	0.59944197	0.72726652
ENSG0000016	MYL3	protein_codir	-0.30425258	0.47865606	0.62650366
ENSG0000006	SLK	protein_codir	-0.30438629	0.28163675	0.43105436
ENSG0000020	C5orf51	protein_codir	-0.30443369	0.09815841	0.19899701
ENSG0000027	RP11-440D17	lncRNA	-0.30460853	0.6667066	0.77977711
ENSG0000019	PHETA1	protein_codir	-0.30463346	0.05939726	0.13686096
ENSG0000016	EFHB	protein_codir	-0.30468648	0.71195412	0.81327486
ENSG0000016	COX11	protein_codir	-0.30468896	0.00507678	0.02051808
ENSG0000017	CKAP5	protein_codir	-0.30487478	0.06182601	0.14124355
ENSG0000013	BARD1	protein_codir	-0.30512681	0.21599929	0.35624849
ENSG0000027	RP11-367N14	lncRNA	-0.30522166	0.68311534	0.79166054

ENSG0000018	UBE2L3	protein_codir	-0.30533427	0.00058405	0.00371006
ENSG0000012	ELK1	protein_codir	-0.30541965	0.04350952	0.10829629
ENSG0000026	RP11-156P1.3	lncRNA	-0.30557483	0.25641599	0.40311666
ENSG0000013	SDF2	protein_codir	-0.30561064	0.00077412	0.00465921
ENSG0000023	RP11-154D17	lncRNA	-0.30584792	0.79864913	0.87482408
ENSG0000016	MMADHC	protein_codir	-0.30587817	0.01287428	0.04230165
ENSG0000014	HIRIP3	protein_codir	-0.30597488	0.03129516	0.08406252
ENSG0000006	SPA17	protein_codir	-0.3060853	0.26361288	0.41105334
ENSG0000014	ZMYM3	protein_codir	-0.30613864	0.079584	0.16997449
ENSG0000015	SLC37A3	protein_codir	-0.30615311	0.13324376	0.24977552
ENSG0000007	AFF4	protein_codir	-0.30615376	0.21486241	0.35487587
ENSG0000010	ADNP	protein_codir	-0.30626012	0.02081747	0.06115492
ENSG0000012	CCZ1	protein_codir	-0.30632645	0.20144126	0.33847832
ENSG0000013	COX5B	protein_codir	-0.30635817	0.00571476	0.02246857
ENSG0000016	RFNG	protein_codir	-0.30641186	0.04052379	0.10251078
ENSG0000025	AC007040.11	protein_codir	-0.30647381	0.58010263	0.71182348
ENSG0000018	POFUT2	protein_codir	-0.3065198	0.08494511	0.17847131
ENSG0000016	ZNF558	protein_codir	-0.30655022	0.03081874	0.08309388
ENSG0000022	RP5-858B6.3	lncRNA	-0.30690141	0.49905188	0.64465203
ENSG0000018	NAA38	protein_codir	-0.30698584	0.04943786	0.11949537
ENSG0000011	ALMS1	protein_codir	-0.30701513	0.11304083	0.22091862
ENSG0000005	MTA3	protein_codir	-0.3071087	0.07527091	0.16337781
ENSG0000006	HMG20B	protein_codir	-0.30711829	0.17969915	0.31106108
ENSG0000018	LDLRAD2	protein_codir	-0.30728209	0.21951051	0.36028678
ENSG0000024	CD302	protein_codir	-0.30728548	0.26849101	0.41626279
ENSG0000028	RP11-1109F1	lncRNA	-0.30740958	0.83476618	NA
ENSG0000024	MRPL20	protein_codir	-0.30744374	0.02157867	0.06285624
ENSG0000026	RP11-834C11	lncRNA	-0.3074923	0.72948653	0.82605953
ENSG0000004	RSF1	protein_codir	-0.30750423	0.08787531	0.18294386
ENSG0000013	ILKAP	protein_codir	-0.3075705	0.01184577	0.0397015
ENSG0000014	RADX	protein_codir	-0.30758082	0.41503847	0.56816825
ENSG0000016	PRPSAP1	protein_codir	-0.30768248	0.08345123	0.17594833
ENSG0000011	CAPRIN2	protein_codir	-0.30769596	0.12640549	0.24025941
ENSG0000007	CIC	protein_codir	-0.30785615	0.07858955	0.16842359
ENSG0000023	MAGI2-AS3	lncRNA	-0.30803553	0.16031161	0.28607098
ENSG0000014	LUC7L2	protein_codir	-0.30805831	0.00606095	0.02351173
ENSG0000013	NMT1	protein_codir	-0.3081361	0.00456322	0.01889032
ENSG0000014	GPR107	protein_codir	-0.30827711	0.06041081	0.13868727
ENSG0000010	SRRD	protein_codir	-0.30832325	0.02243515	0.06485858
ENSG0000018	FAM104B	protein_codir	-0.30834022	0.11105796	0.21829069
ENSG0000020	LINC01597	lncRNA	-0.30840692	0.42673024	0.57855336
ENSG0000008	HUWE1	protein_codir	-0.30840694	0.06806597	0.15198158
ENSG0000020	ST8SIA6-AS1	lncRNA	-0.30852638	0.78749907	0.86735751
ENSG0000013	MIP	protein_codir	-0.30901571	0.83593706	NA
ENSG0000010	PLA2G4C	protein_codir	-0.3093001	0.19195434	0.32670608
ENSG0000013	ESYT1	protein_codir	-0.30947118	0.04558864	0.1122945
ENSG0000014	SH3GLB2	protein_codir	-0.30960868	0.11729885	0.22711492

ENSG0000018	SOCS4	protein_codir	-0.30963178	0.0713084	0.15719835
ENSG0000013	YARS1	protein_codir	-0.30973429	0.02601704	0.07283506
ENSG0000010	HIBADH	protein_codir	-0.30980033	0.08676432	0.18137568
ENSG0000015	TADA1	protein_codir	-0.31010122	0.10959254	0.21611216
ENSG0000013	CHMP1A	protein_codir	-0.31020119	0.00816093	0.02968152
ENSG0000018	B3GLCT	protein_codir	-0.31039615	0.14774702	0.26940721
ENSG0000026	CTC-398G3.6	protein_codir	-0.31068709	0.86154895	0.91755927
ENSG0000027	RP11-234G16	lncRNA	-0.31073795	0.84763036	NA
ENSG0000009	COMT	protein_codir	-0.31082344	0.07325514	0.16028734
ENSG0000014	INTS3	protein_codir	-0.31088468	0.04374693	0.10872211
ENSG0000027	CYFIP1	protein_codir	-0.31099185	0.16303472	0.28962047
ENSG0000011	MRPL2	protein_codir	-0.31102525	0.00017378	0.00138123
ENSG0000016	COMTD1	protein_codir	-0.3110675	0.190921	0.32550852
ENSG0000023	LINC01750	lncRNA	-0.31119986	0.39223153	0.54522967
ENSG0000028	RP11-268J15.	lncRNA	-0.31126057	0.42582401	0.57768918
ENSG0000012	DAD1	protein_codir	-0.31130915	0.02542958	0.07154579
ENSG0000013	BTBD2	protein_codir	-0.31136321	0.00055184	0.00354336
ENSG0000025	SMILR	lncRNA	-0.31165944	0.7567824	0.84593994
ENSG0000026	RP11-95O2.5	lncRNA	-0.31174501	0.58783392	0.7181199
ENSG0000013	TMEM160	protein_codir	-0.31176253	0.04992071	0.12040258
ENSG0000018	SPDYE16	protein_codir	-0.31200713	0.68859344	0.79560556
ENSG0000018	ZNF445	protein_codir	-0.31216215	0.052118	0.12407895
ENSG0000013	ANXA7	protein_codir	-0.31218392	0.05227103	0.12435021
ENSG0000015	CWC27	protein_codir	-0.31238688	0.00994538	0.03466139
ENSG0000015	SEC24D	protein_codir	-0.31255525	0.17260153	0.30222228
ENSG0000013	KIDINS220	protein_codir	-0.312581	0.13648244	0.25389944
ENSG0000023	RNASEH1-AS1	lncRNA	-0.31258516	0.13080957	0.24629902
ENSG0000028	RENO1	lncRNA	-0.31262552	0.44215133	0.59321519
ENSG0000023	LINC01807	lncRNA	-0.31284643	0.7853112	0.86580794
ENSG0000013	XPO7	protein_codir	-0.31285545	0.00545555	0.02172465
ENSG0000026	RP4-798A10.7	lncRNA	-0.31291656	0.5801011	0.71182348
ENSG0000027	RP4-548D19.3	lncRNA	-0.3129922	0.43986748	0.590854
ENSG0000028	RP13-512J5.1	protein_codir	-0.3130347	0.30566178	0.45729083
ENSG0000011	WDR35	protein_codir	-0.31317112	0.19518266	0.33087492
ENSG0000012	TXNDC17	protein_codir	-0.31326664	0.08077931	0.17195602
ENSG0000013	KAT7	protein_codir	-0.31328003	0.00972345	0.03403391
ENSG0000016	ZNF333	protein_codir	-0.31333921	0.05266166	0.12511815
ENSG0000015	MED19	protein_codir	-0.31342358	0.00656049	0.02502207
ENSG0000016	GHITM	protein_codir	-0.31347201	0.02104295	0.061673
ENSG0000017	RMDN1	protein_codir	-0.31349443	0.01621887	0.05064616
ENSG0000017	AURKAIP1	protein_codir	-0.31374812	0.03189565	0.08522016
ENSG0000012	CDKN1A	protein_codir	-0.31385671	0.48979663	0.63659042
ENSG0000028	RP11-358F13.	lncRNA	-0.3138802	0.74808227	0.83942662
ENSG0000006	MYO9A	protein_codir	-0.31391008	0.10358388	0.20718276
ENSG0000022	RP11-276H7.2	lncRNA	-0.31407049	0.49852126	0.64423138
ENSG0000026	UXT-AS1	lncRNA	-0.31423929	0.33430143	0.48722524
ENSG0000011	SNX17	protein_codir	-0.3142538	0.00523715	0.0210339

ENSG0000018	RUVBL2	protein_codir	-0.31429297	0.0290234	0.07946335
ENSG0000012	PGAP6	protein_codir	-0.31440704	0.04470855	0.11060594
ENSG0000014	CTTNBP2NL	protein_codir	-0.31452987	0.15420744	0.27801629
ENSG0000013	WDR12	protein_codir	-0.31467864	0.11353763	0.22168171
ENSG0000007	ACTR6	protein_codir	-0.31472847	0.00331829	0.01465245
ENSG0000018	OSTN	protein_codir	-0.31475478	0.79254786	0.87102425
ENSG0000008	SLC23A2	protein_codir	-0.31488058	0.05427092	0.12797428
ENSG0000017	STX8	protein_codir	-0.31511608	0.02363677	0.06749175
ENSG0000028	RP4-760C5.1	lncRNA	-0.31513633	0.58807152	0.71825132
ENSG0000014	CCDC25	protein_codir	-0.31518157	0.02390223	0.06810188
ENSG0000019	ZKSCAN8	protein_codir	-0.31520518	0.05572774	0.13048494
ENSG0000013	SMPD4	protein_codir	-0.31530164	0.00031919	0.00226869
ENSG0000012	PCMT1	protein_codir	-0.31535737	0.00837393	0.03025256
ENSG0000025	CTD-2054N24	lncRNA	-0.31539886	0.37870557	0.53217236
ENSG0000017	HOXC5	protein_codir	-0.31564408	0.57651604	0.70899742
ENSG0000011	SOS1	protein_codir	-0.31567689	0.01355134	0.04405188
ENSG0000010	TLDC2	protein_codir	-0.31582061	0.67176227	0.78353353
ENSG0000010	WDR83OS	protein_codir	-0.3158665	0.01270629	0.0418688
ENSG0000012	PCID2	protein_codir	-0.3158947	0.00364105	0.01575772
ENSG0000012	ZC3H7A	protein_codir	-0.31590563	0.00741738	0.02755453
ENSG0000010	RHBDL1	protein_codir	-0.31593655	0.37603859	0.5295562
ENSG0000007	SAR1A	protein_codir	-0.31603362	0.00553788	0.02194931
ENSG0000012	MMP24OS	protein_codir	-0.31649301	0.14081684	0.26005356
ENSG0000010	PRPF31	protein_codir	-0.3165409	0.00846466	0.03050172
ENSG0000028	SEPT5-GP1BB	protein_codir	-0.31660075	0.33084889	0.48346894
ENSG0000010	ARMC1	protein_codir	-0.316755	0.03323592	0.08802992
ENSG0000007	MKRN2	protein_codir	-0.3168231	0.01453475	0.0465449
ENSG0000011	TXNDC9	protein_codir	-0.31714506	0.03815745	0.09776633
ENSG0000010	TMEM147	protein_codir	-0.31714577	0.01165591	0.03923181
ENSG0000027	RNU12	lncRNA	-0.31729916	NA	NA
ENSG0000013	DNAJC13	protein_codir	-0.31757418	0.13849676	0.25681642
ENSG0000028	RP11-188P8.4	lncRNA	-0.31757449	0.57930501	0.71122392
ENSG0000017	FLII	protein_codir	-0.31762336	0.02502431	0.07065748
ENSG0000010	PICK1	protein_codir	-0.31786679	0.12635635	0.24022749
ENSG0000027	CTC-1337H24	lncRNA	-0.31788199	0.65108266	0.76714065
ENSG0000021	ZSWIM8	protein_codir	-0.31793478	0.01395386	0.0450556
ENSG0000013	TMTC3	protein_codir	-0.31797394	0.19978617	0.3363871
ENSG0000010	CUEDC2	protein_codir	-0.31805736	0.00055847	0.00357556
ENSG0000018	POU3F2	protein_codir	-0.31805982	0.78864213	0.86807482
ENSG0000020	RING1	protein_codir	-0.31809218	0.00854878	0.03073243
ENSG0000010	BAG1	protein_codir	-0.31825787	0.07648659	0.16523737
ENSG0000012	ATF4	protein_codir	-0.31834155	0.20480711	0.34255977
ENSG0000025	RP11-752G15	lncRNA	-0.31838243	0.55858478	0.69414702
ENSG0000027	ZNF670	protein_codir	-0.31839993	0.11863784	0.22906516
ENSG0000011	ID3	protein_codir	-0.31852797	0.29780868	0.44862153
ENSG0000012	ADGRB2	protein_codir	-0.31863584	0.28462881	0.43423817
ENSG0000014	CAMK2D	protein_codir	-0.31863628	0.03800731	0.09748085

ENSG0000011	SERINC1	protein_codir	-0.31891921	0.17740306	0.30799375
ENSG0000010	RAD51C	protein_codir	-0.31892085	0.00695814	0.02622596
ENSG0000019	LONP1	protein_codir	-0.31904226	0.00721976	0.02695821
ENSG0000019	C6orf89	protein_codir	-0.31908692	0.05457989	0.12851448
ENSG0000019	TOR4A	protein_codir	-0.31909617	0.27235274	0.4202918
ENSG0000026	NORAD	lncRNA	-0.31915822	0.154578	0.27852073
ENSG0000018	FAF1	protein_codir	-0.31941951	0.07013204	0.15529921
ENSG0000027	RP11-236L14.	lncRNA	-0.31950226	0.55298283	0.68944999
ENSG0000011	GORASP2	protein_codir	-0.31974982	0.03991233	0.10134018
ENSG0000010	USP31	protein_codir	-0.31975586	0.12966898	0.24466236
ENSG0000013	SSB	protein_codir	-0.31976038	0.00446075	0.01855002
ENSG0000018	ZDHHC11	protein_codir	-0.31977163	0.48770507	0.63482793
ENSG0000028	RP11-118H4.4	lncRNA	-0.31979374	0.87051872	NA
ENSG0000017	PSMD2	protein_codir	-0.31982563	0.01726521	0.05299089
ENSG0000013	ACLY	protein_codir	-0.31985638	0.13235449	0.24861473
ENSG0000021	MDP1	protein_codir	-0.31987646	0.02203675	0.06392093
ENSG0000014	FAHD2B	protein_codir	-0.31988887	0.07585583	0.16427301
ENSG0000007	KLHL20	protein_codir	-0.32000072	0.03439106	0.09044763
ENSG0000016	ALDH7A1	protein_codir	-0.32008829	0.07536449	0.16352951
ENSG0000027	EIF2B5-DT	lncRNA	-0.32015493	0.84340816	NA
ENSG0000026	KC6	lncRNA	-0.32019453	0.80315868	0.87803207
ENSG0000010	POLDIP3	protein_codir	-0.32025587	0.0001427	0.00117616
ENSG0000013	DHX30	protein_codir	-0.32032145	0.0028936	0.01310615
ENSG0000014	GRIP2	protein_codir	-0.32034812	0.27355053	0.42154183
ENSG0000004	RRM2B	protein_codir	-0.32036541	0.07178856	0.15792912
ENSG0000028	RP11-661A12	lncRNA	-0.32054822	0.45520527	0.60577064
ENSG0000028	RP1-199L16.2	lncRNA	-0.32066819	0.80267669	0.87773629
ENSG0000013	UBA1	protein_codir	-0.32074362	0.00447605	0.01860196
ENSG0000021	APTR	lncRNA	-0.32087942	0.05101915	0.12233256
ENSG0000007	FBXW11	protein_codir	-0.32097586	0.08669198	0.18130025
ENSG0000027	RP11-307N16	protein_codir	-0.32098633	0.82981531	0.89736401
ENSG0000014	HTR7	protein_codir	-0.32102546	0.40577498	0.558727
ENSG0000016	UBQLN4	protein_codir	-0.32103028	0.00143124	0.00759874
ENSG0000012	AIF1L	protein_codir	-0.32103045	0.53236336	0.67340857
ENSG0000013	IFT172	protein_codir	-0.3211911	0.11742971	0.22728862
ENSG0000018	MYT1L	protein_codir	-0.32134192	0.7139195	0.81454299
ENSG0000027	RP11-395A13	lncRNA	-0.32135725	0.05707765	0.13277897
ENSG0000013	LRRC41	protein_codir	-0.3214109	0.02290273	0.06587247
ENSG0000005	KIF1B	protein_codir	-0.32147649	0.20525201	0.3431427
ENSG0000028	CTD-2252G20	protein_codir	-0.32172729	0.58203219	0.71333551
ENSG0000023	HOXD-AS2	lncRNA	-0.32177508	0.43430601	0.58574793
ENSG0000018	L3MBTL1	protein_codir	-0.32178579	0.25182167	0.39799236
ENSG0000014	SCAPER	protein_codir	-0.32197324	0.02474896	0.07003752
ENSG0000020	TMEM231	protein_codir	-0.32201916	0.14757742	0.26919088
ENSG0000025	RP11-2E11.5	lncRNA	-0.32207668	0.75749423	0.84652512
ENSG0000018	MORN2	protein_codir	-0.32215893	0.08379458	0.17653748
ENSG0000017	TALDO1	protein_codir	-0.32223579	0.02607134	0.0729427

ENSG0000018	TM2D3	protein_codir	-0.32232278	0.00567428	0.02234758
ENSG0000018	SBK2	protein_codir	-0.32256853	0.81430453	0.88579941
ENSG0000005	FLYWCH1	protein_codir	-0.32263187	0.08984588	0.18595304
ENSG0000016	ZNF32	protein_codir	-0.32267162	0.00987939	0.03447489
ENSG0000011	C1orf109	protein_codir	-0.32272352	0.04615064	0.11322236
ENSG0000022	RP11-134G8.1	lncRNA	-0.32274715	0.89174302	NA
ENSG0000020	LINC02731	lncRNA	-0.32280644	0.42515474	0.57709721
ENSG0000016	AP2M1	protein_codir	-0.32298433	0.03007193	0.08161411
ENSG0000024	FRG1-DT	lncRNA	-0.32306078	0.47916198	0.62698298
ENSG0000010	PSMA7	protein_codir	-0.32330264	0.02180434	0.06336657
ENSG0000027	DHRS11	protein_codir	-0.32331206	0.01027632	0.03557236
ENSG0000014	CERS2	protein_codir	-0.32347008	0.03574385	0.09302421
ENSG0000013	ZDHHC4	protein_codir	-0.32348489	0.00174399	0.00884973
ENSG0000022	FAM66A	lncRNA	-0.32356726	0.58971501	0.71952682
ENSG0000011	PTBP2	protein_codir	-0.32372741	0.1276578	0.24190405
ENSG0000007	TRHDE	protein_codir	-0.32378041	0.59672388	0.724972
ENSG0000008	PPIE	protein_codir	-0.3238009	0.01779898	0.05422543
ENSG0000011	CYP20A1	protein_codir	-0.32385278	0.0015318	0.00800382
ENSG0000015	CPB1	protein_codir	-0.32397188	0.77608934	0.85955521
ENSG0000019	WDSUB1	protein_codir	-0.3240058	0.04775548	0.11619155
ENSG0000018	EXOC3	protein_codir	-0.32406233	0.02896868	0.07932141
ENSG0000015	ME3	protein_codir	-0.3241352	0.22656105	0.36864815
ENSG0000018	ZAR1L	protein_codir	-0.32416665	0.70672048	0.80943892
ENSG0000020	LINC01291	lncRNA	-0.32442059	0.8280264	NA
ENSG0000008	RAB10	protein_codir	-0.32447004	0.0324287	0.08634051
ENSG0000012	PLA2G5	protein_codir	-0.32447674	0.1882126	0.32208377
ENSG0000006	NDUFB4	protein_codir	-0.32459845	0.00945811	0.03332054
ENSG0000026	LIN37	protein_codir	-0.32463889	0.07029662	0.15550157
ENSG0000017	COMMD1	protein_codir	-0.3248682	0.01678466	0.0519254
ENSG0000018	LYRM7	protein_codir	-0.32496387	0.06733556	0.15088497
ENSG0000017	ZNF354C	protein_codir	-0.32516569	0.12839395	0.24290985
ENSG0000010	PRKAG2	protein_codir	-0.32519144	0.10583668	0.21053
ENSG0000027	NBPF26	protein_codir	-0.32522147	0.08916638	0.18496247
ENSG0000013	METTL26	protein_codir	-0.32528921	0.06644622	0.14944988
ENSG0000009	FH	protein_codir	-0.32538061	0.02283404	0.06571452
ENSG0000008	GNAO1	protein_codir	-0.32544396	0.4793582	0.6271255
ENSG0000019	ZNF627	protein_codir	-0.32548985	0.18999721	0.32445424
ENSG0000027	RP11-347C12.	lncRNA	-0.3255518	0.52498497	0.66691587
ENSG0000011	HBS1L	protein_codir	-0.32566423	0.02642832	0.07368769
ENSG0000015	PTS	protein_codir	-0.32569618	0.11894473	0.22949728
ENSG0000007	CRMP1	protein_codir	-0.32575655	0.49469604	0.6407814
ENSG0000014	SERPINH1	protein_codir	-0.32578718	0.27895496	0.42775572
ENSG0000013	GRHPR	protein_codir	-0.32591823	0.01777179	0.05417248
ENSG0000014	DHX38	protein_codir	-0.32616553	0.00994832	0.03466725
ENSG0000019	ZNF517	protein_codir	-0.32647979	0.03891917	0.09933901
ENSG0000013	SH3BP4	protein_codir	-0.32680438	0.24819383	0.39367792
ENSG0000012	MOCS3	protein_codir	-0.32680511	0.19130618	0.3258836

ENSG0000018	MACIR	protein_codir	-0.32682522	0.16535052	0.29275521
ENSG0000023	ALMS1-IT1	lncRNA	-0.3268799	0.33013344	0.4826998
ENSG0000027	RP11-697E2.1	lncRNA	-0.32696262	0.72707618	0.82417549
ENSG0000024	AC226118.1	lncRNA	-0.32708313	0.52930802	0.67070862
ENSG0000010	GSK3A	protein_codir	-0.32719636	0.01849649	0.05584533
ENSG0000013	PRPF38A	protein_codir	-0.32728894	0.01736962	0.05321674
ENSG0000016	WDR4	protein_codir	-0.32732765	0.10583359	0.21053
ENSG0000007	SLC25A3	protein_codir	-0.32735811	0.03447288	0.0905716
ENSG0000001	HGF	protein_codir	-0.32744585	0.36259559	0.51645551
ENSG0000012	COQ8B	protein_codir	-0.32758715	0.01356103	0.04406537
ENSG0000027	LLfos-48D6.2	protein_codir	-0.32783637	0.81472053	0.88606793
ENSG0000016	C9orf24	protein_codir	-0.32791145	0.66807896	0.7807197
ENSG0000012	UPF3B	protein_codir	-0.32793471	0.17683735	0.30726268
ENSG0000013	DCUN1D5	protein_codir	-0.32797775	0.14747369	0.26907275
ENSG0000008	AGBL5	protein_codir	-0.32804865	0.16475554	0.29205931
ENSG0000018	PLK5	protein_codir	-0.32808219	0.83880831	0.90326662
ENSG0000008	PDRG1	protein_codir	-0.32815308	0.01064126	0.03656072
ENSG0000016	PPIP5K1	protein_codir	-0.3282346	0.01376307	0.04456981
ENSG0000017	MRPL11	protein_codir	-0.32828381	0.00873664	0.03126949
ENSG0000017	ATP6V0E2	protein_codir	-0.3283345	0.04535683	0.11190245
ENSG0000022	AC105053.3	lncRNA	-0.32836271	0.50129945	0.6461091
ENSG0000014	MRPS28	protein_codir	-0.32838578	0.02125632	0.06210721
ENSG0000016	OSGIN2	protein_codir	-0.32841757	0.08334534	0.17579216
ENSG0000013	ZCRB1	protein_codir	-0.32854175	0.04214407	0.10573342
ENSG0000012	STAMBP	protein_codir	-0.32867629	0.00937614	0.03311664
ENSG0000002	STRAP	protein_codir	-0.32869867	0.12274531	0.23510578
ENSG0000019	ZNF347	protein_codir	-0.32890451	0.09809388	0.1989392
ENSG0000011	RAP1A	protein_codir	-0.32901166	0.200294	0.33699563
ENSG0000025	ZNF10	protein_codir	-0.32919623	0.16079276	0.28667028
ENSG0000026	RP13-890H12	lncRNA	-0.32926823	0.47599266	0.6242413
ENSG0000018	NRG3	protein_codir	-0.32932222	0.54542273	0.68341767
ENSG0000010	LONP2	protein_codir	-0.32934451	0.00181926	0.00911942
ENSG0000008	CRLS1	protein_codir	-0.32937281	0.01046696	0.03610108
ENSG0000017	KB-1552D7.2	lncRNA	-0.32954459	0.88961005	NA
ENSG0000017	SWI5	protein_codir	-0.32959598	0.03934791	0.1002573
ENSG0000019	ZNF71	protein_codir	-0.32966506	0.22859015	0.37102075
ENSG0000025	CTD-2298J14.	lncRNA	-0.3298052	0.4983323	0.64407759
ENSG0000026	RP11-666A8.8	lncRNA	-0.32981473	0.58662989	0.71702959
ENSG0000028	RP11-582H21	lncRNA	-0.3298566	0.86507021	NA
ENSG0000022	KIF9-AS1	lncRNA	-0.33004575	0.11692345	0.22656276
ENSG0000015	PSMD4	protein_codir	-0.33009328	0.00258119	0.01197186
ENSG0000016	GFM2	protein_codir	-0.33014556	0.00130462	0.00707793
ENSG0000013	CAP1	protein_codir	-0.33019295	0.07369523	0.16096973
ENSG0000010	ILVBL	protein_codir	-0.33027657	0.00549152	0.02181443
ENSG0000014	SORD	protein_codir	-0.33060976	0.06897191	0.15327091
ENSG0000022	TMEM30A-DT	lncRNA	-0.33070432	0.24086088	0.38540013
ENSG0000012	ARPP19	protein_codir	-0.33071243	0.10357068	0.20717136

ENSG0000016	HNF4G	protein_codir	-0.3307572	0.72777628	0.82486688
ENSG0000021	ZNF891	protein_codir	-0.33103414	0.14134084	0.26072491
ENSG0000015	MAGI1	protein_codir	-0.33110597	0.26083609	0.40807038
ENSG0000011	DCAF17	protein_codir	-0.33119786	0.03565807	0.0928515
ENSG0000016	TRIM56	protein_codir	-0.33121468	0.06163626	0.14093828
ENSG0000008	MRPL28	protein_codir	-0.33127399	0.01783566	0.05430313
ENSG0000013	FRMD6	protein_codir	-0.33129692	0.28184125	0.43124787
ENSG0000025	RP11-783K16.	lncRNA	-0.33139695	0.42110398	0.57314435
ENSG0000011	CSRNP2	protein_codir	-0.33144691	0.08167687	0.1733719
ENSG0000019	ZNF398	protein_codir	-0.33148019	0.02938363	0.08019525
ENSG0000022	GRK5-IT1	lncRNA	-0.33153899	0.703597	0.80698818
ENSG0000010	H2AZ2	protein_codir	-0.33161463	0.04540565	0.11196357
ENSG0000017	PDXDC1	protein_codir	-0.33170237	0.00038144	0.00263053
ENSG0000018	PRKAG1	protein_codir	-0.33173585	0.0089016	0.03177744
ENSG0000013	TACO1	protein_codir	-0.33180237	0.01858703	0.05606353
ENSG0000011	ADAM23	protein_codir	-0.33192511	0.35051814	0.50408407
ENSG0000017	KRT4	protein_codir	-0.33200813	0.78971743	0.8688316
ENSG0000016	ZNF30	protein_codir	-0.33206152	0.27560333	0.42370801
ENSG0000017	TEFM	protein_codir	-0.33207091	0.07778849	0.16718684
ENSG0000008	CHMP2B	protein_codir	-0.3321697	0.08612881	0.18035463
ENSG0000025	LRRC24	protein_codir	-0.33288665	0.13598269	0.25322558
ENSG0000025	C8orf37-AS1	lncRNA	-0.33304167	0.55512678	0.69109356
ENSG0000011	ZNHIT6	protein_codir	-0.33320206	0.01753916	0.05361722
ENSG0000019	CCDC152	protein_codir	-0.33327673	0.12870776	0.24340351
ENSG0000027	ZNF559-ZNF1	protein_codir	-0.3333026	0.30084979	0.45192153
ENSG0000011	PHF1	protein_codir	-0.33336966	0.15234418	0.27543873
ENSG0000014	SERF2	protein_codir	-0.33341868	0.01381253	0.04469525
ENSG0000011	RWDD1	protein_codir	-0.33343188	0.13472126	0.25173498
ENSG0000010	PTOV1	protein_codir	-0.33347065	0.00551938	0.0218936
ENSG0000007	PICALM	protein_codir	-0.33355467	0.15806199	0.28311753
ENSG0000008	TXLNA	protein_codir	-0.33388198	0.00290771	0.01315065
ENSG0000025	BRK1	protein_codir	-0.33389836	0.0058741	0.02295393
ENSG0000018	RGPD6	protein_codir	-0.33399631	0.59088976	0.72026017
ENSG0000006	IARS2	protein_codir	-0.33419385	0.05470726	0.12869555
ENSG0000008	ARG2	protein_codir	-0.33419756	0.35075976	0.50437237
ENSG0000016	FAAP20	protein_codir	-0.334266	0.01754781	0.05363562
ENSG0000010	PLIN3	protein_codir	-0.33434609	0.03249439	0.08646388
ENSG0000026	TNRC6B-DT	lncRNA	-0.33453652	0.72151486	0.8202155
ENSG0000017	SRRM3	protein_codir	-0.33455464	0.30293677	0.4542052
ENSG0000012	IVD	protein_codir	-0.33483042	0.04421833	0.10966225
ENSG0000010	ZC3H7B	protein_codir	-0.33491087	0.00094747	0.00548581
ENSG0000018	ZBTB3	protein_codir	-0.33492099	0.14037047	0.25955897
ENSG0000024	NEAT1	lncRNA	-0.33497576	0.47003066	0.61914568
ENSG0000017	RIC8A	protein_codir	-0.33509426	0.00108153	0.00609256
ENSG0000026	AC139099.4	lncRNA	-0.33511714	0.47118517	0.62015284
ENSG0000013	BTBD3	protein_codir	-0.33524789	0.12356539	0.23620155
ENSG0000010	PYCR3	protein_codir	-0.33546095	0.14486014	0.26561783

ENSG0000012	CAPNS1	protein_codir	-0.33553691	0.08156392	0.17323866
ENSG0000014	MEIS1	protein_codir	-0.33556984	0.36891638	0.52248335
ENSG0000010	ST13	protein_codir	-0.33572907	0.11651007	0.22604722
ENSG0000015	XRCC4	protein_codir	-0.3358555	0.13848724	0.256816
ENSG0000011	RAD50	protein_codir	-0.33598635	0.0119952	0.04005653
ENSG0000017	ACAD9	protein_codir	-0.33604042	0.00264081	0.01220534
ENSG0000017	UFSP1	protein_codir	-0.33619737	0.14700787	0.26849226
ENSG0000027	RP3-508I15.2	lncRNA	-0.33635948	0.81810247	0.88823511
ENSG0000010	ZNF330	protein_codir	-0.33646738	0.00917139	0.0325594
ENSG0000012	SMUG1	protein_codir	-0.33648846	0.02144712	0.06257865
ENSG0000028	RP13-968A2.1	lncRNA	-0.3365434	0.66589107	0.77927283
ENSG0000018	ZNF438	protein_codir	-0.33660172	0.05067461	0.12178133
ENSG0000011	EIF4G1	protein_codir	-0.33670017	0.04172325	0.10494456
ENSG0000016	CACNG2	protein_codir	-0.33673217	0.88505806	NA
ENSG0000016	PDZD8	protein_codir	-0.33686796	0.18667516	0.32010728
ENSG0000019	UBL5	protein_codir	-0.33688102	0.0123444	0.04094538
ENSG0000027	RP11-114F3.4	lncRNA	-0.33691017	0.56023665	0.69544882
ENSG0000010	PBDC1	protein_codir	-0.33696137	0.02771486	0.07653314
ENSG0000013	ECSIT	protein_codir	-0.33697061	0.00866341	0.03108383
ENSG0000026	LINC00565	lncRNA	-0.33722758	0.15911889	0.28451545
ENSG0000018	C2orf66	protein_codir	-0.33734237	0.73563836	0.83047214
ENSG0000011	MRPL19	protein_codir	-0.33736884	0.1416773	0.26119017
ENSG0000015	C9orf85	protein_codir	-0.33738349	0.04823601	0.1171749
ENSG0000023	RP13-131K19.	lncRNA	-0.337402	0.72842751	0.82540175
ENSG0000018	HGS	protein_codir	-0.33746773	0.01786058	0.05434118
ENSG0000017	PSMD1	protein_codir	-0.33774	0.0161651	0.05052114
ENSG0000017	ZNF223	protein_codir	-0.33780804	0.25657802	0.40323747
ENSG0000017	COX8A	protein_codir	-0.33788192	0.03724978	0.09603649
ENSG0000018	GSPT2	protein_codir	-0.33790399	0.18682316	0.32032129
ENSG0000023	LINC01786	lncRNA	-0.33794345	0.49170212	0.63834606
ENSG0000014	HAX1	protein_codir	-0.33800574	0.00051802	0.00336415
ENSG0000010	KPNA3	protein_codir	-0.33803213	0.06733506	0.15088497
ENSG0000008	DNM1L	protein_codir	-0.33814904	0.04301121	0.10736513
ENSG0000009	NDUFB2	protein_codir	-0.33822711	0.01181566	0.03964872
ENSG0000014	LPAR4	protein_codir	-0.33835932	0.28035022	0.42946606
ENSG0000015	SAP18	protein_codir	-0.33836518	0.03167822	0.08483096
ENSG0000017	FAM87B	lncRNA	-0.33850924	0.435006	0.58646292
ENSG0000012	UQCR11	protein_codir	-0.3385444	0.01059076	0.03642344
ENSG0000009	TMED1	protein_codir	-0.3385793	0.00849936	0.03061055
ENSG0000027	CTD-2619J13.	lncRNA	-0.3386959	0.65194761	0.76784755
ENSG0000014	ETAA1	protein_codir	-0.33873352	0.15359166	0.27717816
ENSG0000027	KB-1125A3.11	lncRNA	-0.33887996	0.64111857	0.75933512
ENSG0000025	FPGT	protein_codir	-0.33896021	0.07473549	0.16248381
ENSG0000011	MRPS15	protein_codir	-0.33914271	0.00310234	0.01387187
ENSG0000011	PSMD9	protein_codir	-0.33928267	0.01624302	0.05069291
ENSG0000024	MEF2C-AS1	lncRNA	-0.33932616	0.25916064	0.4062381
ENSG0000016	CIAO2B	protein_codir	-0.33934989	0.00292076	0.01320316

ENSG0000007	KEAP1	protein_codir	-0.33958987	0.00067625	0.00418688
ENSG0000011	EXPH5	protein_codir	-0.33958989	0.22633729	0.36835523
ENSG0000016	CHST14	protein_codir	-0.33960276	0.02073843	0.0609876
ENSG0000013	TMEM117	protein_codir	-0.33974005	0.1225799	0.23487696
ENSG0000012	KCNIP2	protein_codir	-0.33976554	0.53979989	0.67889773
ENSG0000021	SMIM7	protein_codir	-0.3399302	0.00152958	0.00799981
ENSG0000024	KB-1208A12.3	lncRNA	-0.34012257	0.68087908	0.79026264
ENSG0000015	ZFAND3	protein_codir	-0.34014154	0.03451822	0.0906612
ENSG0000027	RP11-251G23	lncRNA	-0.34015402	0.08922065	0.18503337
ENSG0000000	VPS41	protein_codir	-0.34022309	0.03941028	0.10036993
ENSG0000007	VDAC3	protein_codir	-0.340291	0.00556075	0.02201573
ENSG0000015	NDUFC2	protein_codir	-0.34031683	0.00149658	0.00787044
ENSG0000013	THAP1	protein_codir	-0.34031728	0.02007177	0.05945155
ENSG0000013	ZRANB2	protein_codir	-0.34035581	0.02533356	0.07134831
ENSG0000027	RP11-378A13	lncRNA	-0.34036529	0.53619185	0.67636184
ENSG0000016	TTC7B	protein_codir	-0.34040266	0.16001605	0.28569122
ENSG0000001	YAF2	protein_codir	-0.34040751	0.1264102	0.24025941
ENSG0000016	AIMP1	protein_codir	-0.34045473	0.03263688	0.08675943
ENSG0000016	TPRA1	protein_codir	-0.3404726	0.00891499	0.03181703
ENSG0000014	SOGA1	protein_codir	-0.34048727	0.0700236	0.15513369
ENSG0000017	DPY19L3	protein_codir	-0.34054353	0.03695985	0.09546723
ENSG0000015	CAST	protein_codir	-0.34070366	0.07195405	0.15821757
ENSG0000015	GNAQ	protein_codir	-0.34071445	0.06866226	0.1528041
ENSG0000011	STK25	protein_codir	-0.34081454	0.00753235	0.02787666
ENSG0000013	MRPS9	protein_codir	-0.34087023	0.00750006	0.0277832
ENSG0000027	AC142472.6	lncRNA	-0.34099041	0.26622037	0.41375749
ENSG0000021	CYB5RL	protein_codir	-0.34100743	0.04746266	0.11566249
ENSG0000025	AP001372.2	lncRNA	-0.34109153	0.17460503	0.30468022
ENSG0000019	IARS1	protein_codir	-0.34116064	0.0224229	0.064836
ENSG0000010	VAPA	protein_codir	-0.34125717	0.07641105	0.16515889
ENSG0000017	ZNF784	protein_codir	-0.34144519	0.19448105	0.32994882
ENSG0000010	PSMC1	protein_codir	-0.34144898	0.01498929	0.04772383
ENSG0000021	RP4-761J14.8	lncRNA	-0.34152077	0.50693417	0.65135149
ENSG0000026	AC004156.3	lncRNA	-0.34158235	0.53752889	0.67730602
ENSG0000025	RP11-531H8.2	lncRNA	-0.34161253	0.83851832	NA
ENSG0000016	PHAX	protein_codir	-0.34162453	0.01154253	0.03894988
ENSG0000028	KB-218C10.1	protein_codir	-0.34180626	0.8826699	NA
ENSG0000003	MRI1	protein_codir	-0.34186803	0.04196634	0.10540241
ENSG0000017	PUF60	protein_codir	-0.34196596	0.01788036	0.05438938
ENSG0000027	RP3-402G11.2	lncRNA	-0.34199381	0.47974217	0.62756837
ENSG0000010	ARMCX3	protein_codir	-0.34208021	0.01671936	0.05177583
ENSG0000016	MESP1	protein_codir	-0.34213535	0.38044727	0.53372377
ENSG0000021	SYT3	protein_codir	-0.34215204	0.55439878	0.69059392
ENSG0000027	RP5-965G21.6	lncRNA	-0.34221406	0.67474824	0.78572227
ENSG0000013	XPA	protein_codir	-0.34243247	0.07104251	0.15670093
ENSG0000019	RPF2	protein_codir	-0.34253407	0.04977398	0.1200976
ENSG0000020	C1orf195	lncRNA	-0.34262729	0.91637119	NA

ENSG0000028	RP3-406M12.	lncRNA	-0.34262729	0.91637119	NA
ENSG0000028	RP11-348A7.7	lncRNA	-0.34262729	0.91637119	NA
ENSG0000013	RHBG	protein_codir	-0.34262729	0.91637119	NA
ENSG0000023	RP11-25K21.1	lncRNA	-0.34262729	0.91637119	NA
ENSG0000016	LRRC52	protein_codir	-0.34262729	0.91637119	NA
ENSG0000028	RP1-127C7.8	lncRNA	-0.34262729	0.91637119	NA
ENSG0000023	RP11-25B7.1	lncRNA	-0.34262729	0.91637119	NA
ENSG0000023	LINC01641	lncRNA	-0.34262729	0.91637119	NA
ENSG0000022	AC074363.1	lncRNA	-0.34262729	0.91637119	NA
ENSG0000026	RP11-95H11.1	lncRNA	-0.34262729	0.91637119	NA
ENSG0000017	IQCF1	protein_codir	-0.34262729	0.91637119	NA
ENSG0000024	RP11-769N22	lncRNA	-0.34262729	0.91637119	NA
ENSG0000028	RP11-638O6.2	lncRNA	-0.34262729	0.91637119	NA
ENSG0000025	LINC02260	lncRNA	-0.34262729	0.91637119	NA
ENSG0000024	RP11-567N4.2	lncRNA	-0.34262729	0.91637119	NA
ENSG0000024	LINC00498	lncRNA	-0.34262729	0.91637119	NA
ENSG0000028	RP11-364L4.4	lncRNA	-0.34262729	0.91637119	NA
ENSG0000025	RP11-563E2.2	lncRNA	-0.34262729	0.91637119	NA
ENSG0000018	CLDN24	protein_codir	-0.34262729	0.91637119	NA
ENSG0000025	RP11-332J15.	lncRNA	-0.34262729	0.91637119	NA
ENSG0000024	LINC02226	lncRNA	-0.34262729	0.91637119	NA
ENSG0000024	CTD-2139B15	lncRNA	-0.34262729	0.91637119	NA
ENSG0000024	CTD-2175A23	lncRNA	-0.34262729	0.91637119	NA
ENSG0000024	LINC02198	lncRNA	-0.34262729	0.91637119	NA
ENSG0000025	RP11-136K7.3	lncRNA	-0.34262729	0.91637119	NA
ENSG0000025	CTC-480C2.1	lncRNA	-0.34262729	0.91637119	NA
ENSG0000023	AC004237.1	lncRNA	-0.34262729	0.91637119	NA
ENSG0000025	CTC-535M15.	lncRNA	-0.34262729	0.91637119	NA
ENSG0000028	RP1-33B19.1	lncRNA	-0.34262729	0.91637119	NA
ENSG0000026	RP1-232P20.1	lncRNA	-0.34262729	0.91637119	NA
ENSG0000022	RP11-552E20.	lncRNA	-0.34262729	0.91637119	NA
ENSG0000023	RP3-390M24.	lncRNA	-0.34262729	0.91637119	NA
ENSG0000025	SMLR1	protein_codir	-0.34262729	0.91637119	NA
ENSG0000007	NOX3	protein_codir	-0.34262729	0.91637119	NA
ENSG0000023	AC003092.2	lncRNA	-0.34262729	0.91637119	NA
ENSG0000023	AC003084.2	lncRNA	-0.34262729	0.91637119	NA
ENSG0000028	FAM90A7P	protein_codir	-0.34262729	0.91637119	NA
ENSG0000025	RP11-521M14	lncRNA	-0.34262729	0.91637119	NA
ENSG0000021	LINC00588	lncRNA	-0.34262729	0.91637119	NA
ENSG0000028	RP11-326E22.	lncRNA	-0.34262729	0.91637119	NA
ENSG0000025	RP11-48B3.3	lncRNA	-0.34262729	0.91637119	NA
ENSG0000028	RP11-932B15.	lncRNA	-0.34262729	0.91637119	NA
ENSG0000025	RP11-1C8.4	lncRNA	-0.34262729	0.91637119	NA
ENSG0000028	RP11-659A24	lncRNA	-0.34262729	0.91637119	NA
ENSG0000023	IFNA13	protein_codir	-0.34262729	0.91637119	NA
ENSG0000020	FOXB2	protein_codir	-0.34262729	0.91637119	NA
ENSG0000014	OR13C4	protein_codir	-0.34262729	0.91637119	NA

ENSG0000017	OR2K2	protein_codir	-0.34262729	0.91637119	NA
ENSG0000022	RP11-787B4.2	lncRNA	-0.34262729	0.91637119	NA
ENSG0000028	RP11-305F14.	lncRNA	-0.34262729	0.91637119	NA
ENSG0000022	ZNF32-AS3	lncRNA	-0.34262729	0.91637119	NA
ENSG0000028	RP11-790G19	lncRNA	-0.34262729	0.91637119	NA
ENSG0000022	RP11-348J12.	lncRNA	-0.34262729	0.91637119	NA
ENSG0000015	GPR26	protein_codir	-0.34262729	0.91637119	NA
ENSG0000025	RP11-304M2.	lncRNA	-0.34262729	0.91637119	NA
ENSG0000011	SCGB2A2	protein_codir	-0.34262729	0.91637119	NA
ENSG0000016	TRIM49	protein_codir	-0.34262729	0.91637119	NA
ENSG0000028	CTD-2564P9.4	lncRNA	-0.34262729	0.91637119	NA
ENSG0000028	RP11-2I22.1	lncRNA	-0.34262729	0.91637119	NA
ENSG0000016	SPATA19	protein_codir	-0.34262729	0.91637119	NA
ENSG0000025	RP11-1022B3.	lncRNA	-0.34262729	0.91637119	NA
ENSG0000025	RP11-71J4.2	lncRNA	-0.34262729	0.91637119	NA
ENSG0000025	RP11-1060G2	lncRNA	-0.34262729	0.91637119	NA
ENSG0000013	ZIC5	protein_codir	-0.34262729	0.91637119	NA
ENSG0000024	CTD-2251F13.	lncRNA	-0.34262729	0.91637119	NA
ENSG0000025	SFTA3	protein_codir	-0.34262729	0.91637119	NA
ENSG0000025	LINC02305	lncRNA	-0.34262729	0.91637119	NA
ENSG0000016	SERPINA12	protein_codir	-0.34262729	0.91637119	NA
ENSG0000028	RP11-433J8.3	lncRNA	-0.34262729	0.91637119	NA
ENSG0000025	CTD-3051D23	lncRNA	-0.34262729	0.91637119	NA
ENSG0000028	RP11-385H1.1	lncRNA	-0.34262729	0.91637119	NA
ENSG0000025	RP11-386M24	lncRNA	-0.34262729	0.91637119	NA
ENSG0000027	CTD-3037G24	lncRNA	-0.34262729	0.91637119	NA
ENSG0000026	RP11-538I12.	lncRNA	-0.34262729	0.91637119	NA
ENSG0000028	RP11-568J23.	lncRNA	-0.34262729	0.91637119	NA
ENSG0000028	RP1-168P16.4	lncRNA	-0.34262729	0.91637119	NA
ENSG0000026	RP1-253P7.1	lncRNA	-0.34262729	0.91637119	NA
ENSG0000027	RP11-697E22.	lncRNA	-0.34262729	0.91637119	NA
ENSG0000026	MIR3976HG	lncRNA	-0.34262729	0.91637119	NA
ENSG0000028	RP11-677O4.8	lncRNA	-0.34262729	0.91637119	NA
ENSG0000026	RP11-403A21	lncRNA	-0.34262729	0.91637119	NA
ENSG0000026	LINC02582	lncRNA	-0.34262729	0.91637119	NA
ENSG0000028	FAM138F	lncRNA	-0.34262729	0.91637119	NA
ENSG0000014	CBLC	protein_codir	-0.34262729	0.91637119	NA
ENSG0000010	FGF21	protein_codir	-0.34262729	0.91637119	NA
ENSG0000026	AC010525.4	lncRNA	-0.34262729	0.91637119	NA
ENSG0000023	RP11-410N8.3	lncRNA	-0.34262729	0.91637119	NA
ENSG0000012	ANKRD60	protein_codir	-0.34262729	0.91637119	NA
ENSG0000022	RP13-30A9.2	lncRNA	-0.34262729	0.91637119	NA
ENSG0000019	DSCR8	lncRNA	-0.34262729	0.91637119	NA
ENSG0000023	RP1-76B20.11	lncRNA	-0.34262729	0.91637119	NA
ENSG0000022	SGSM3-AS1	lncRNA	-0.34262729	0.91637119	NA
ENSG0000018	MAGEB16	protein_codir	-0.34262729	0.91637119	NA
ENSG0000026	SSX4	protein_codir	-0.34262729	0.91637119	NA

ENSG0000022	PABPC1L2B-A	lncRNA	-0.34262729	0.91637119	NA
ENSG0000023	RP11-493K23.	lncRNA	-0.34262729	0.91637119	NA
ENSG0000023	RP11-308B5.2	lncRNA	-0.34262729	0.91637119	NA
ENSG0000012	VCY	protein_codir	-0.34262729	0.91637119	NA
ENSG0000020	RP5-1113E3.3	lncRNA	-0.34262729	0.91637119	NA
ENSG0000011	PRAMEF12	protein_codir	-0.34262729	0.91637119	NA
ENSG0000023	PRAMEF6	protein_codir	-0.34262729	0.91637119	NA
ENSG0000027	PRAMEF13	protein_codir	-0.34262729	0.91637119	NA
ENSG0000023	RP11-91K11.2	lncRNA	-0.34262729	0.91637119	NA
ENSG0000028	RP11-373M8.	lncRNA	-0.34262729	0.91637119	NA
ENSG0000022	RP5-994D16.9	lncRNA	-0.34262729	0.91637119	NA
ENSG0000026	MKNK1-AS1	lncRNA	-0.34262729	0.91637119	NA
ENSG0000028	LINC02808	lncRNA	-0.34262729	0.91637119	NA
ENSG0000022	RP11-550H2.1	lncRNA	-0.34262729	0.91637119	NA
ENSG0000022	LINC01763	lncRNA	-0.34262729	0.91637119	NA
ENSG0000023	RP5-1073O3.2	lncRNA	-0.34262729	0.91637119	NA
ENSG0000028	RP4-590F24.2	lncRNA	-0.34262729	0.91637119	NA
ENSG0000028	CTXND2	protein_codir	-0.34262729	0.91637119	NA
ENSG0000016	CRCT1	protein_codir	-0.34262729	0.91637119	NA
ENSG0000019	SPRR2B	protein_codir	-0.34262729	0.91637119	NA
ENSG0000018	S100A7A	protein_codir	-0.34262729	0.91637119	NA
ENSG0000022	LINC02776	lncRNA	-0.34262729	0.91637119	NA
ENSG0000011	PDC	protein_codir	-0.34262729	0.91637119	NA
ENSG0000022	RP11-354K1.2	lncRNA	-0.34262729	0.91637119	NA
ENSG0000028	RP11-61J19.6	lncRNA	-0.34262729	0.91637119	NA
ENSG0000016	OR6F1	protein_codir	-0.34262729	0.91637119	NA
ENSG0000026	RP11-116D2.1	lncRNA	-0.34262729	0.91637119	NA
ENSG0000022	AGBL5-IT1	lncRNA	-0.34262729	0.91637119	NA
ENSG0000023	AC007391.2	lncRNA	-0.34262729	0.91637119	NA
ENSG0000023	LINC01794	lncRNA	-0.34262729	0.91637119	NA
ENSG0000023	AC013402.5	lncRNA	-0.34262729	0.91637119	NA
ENSG0000022	LINC01159	lncRNA	-0.34262729	0.91637119	NA
ENSG0000028	RP11-56O18.2	lncRNA	-0.34262729	0.91637119	NA
ENSG0000023	AC062031.1	lncRNA	-0.34262729	0.91637119	NA
ENSG0000028	RP11-81F8.1	lncRNA	-0.34262729	0.91637119	NA
ENSG0000022	AC009487.4	lncRNA	-0.34262729	0.91637119	NA
ENSG0000028	RP11-434M17	lncRNA	-0.34262729	0.91637119	NA
ENSG0000028	RP11-270G18	lncRNA	-0.34262729	0.91637119	NA
ENSG0000023	AC016903.2	lncRNA	-0.34262729	0.91637119	NA
ENSG0000022	AC007879.1	lncRNA	-0.34262729	0.91637119	NA
ENSG0000016	CRYGC	protein_codir	-0.34262729	0.91637119	NA
ENSG0000023	LINC01953	lncRNA	-0.34262729	0.91637119	NA
ENSG0000023	AC007563.1	lncRNA	-0.34262729	0.91637119	NA
ENSG0000026	RP11-256I23.	lncRNA	-0.34262729	0.91637119	NA
ENSG0000023	AC010149.4	lncRNA	-0.34262729	0.91637119	NA
ENSG0000025	RP11-315F22.	lncRNA	-0.34262729	0.91637119	NA
ENSG0000017	OTOS	protein_codir	-0.34262729	0.91637119	NA

ENSG0000023	LINC02022	lncRNA	-0.34262729	0.91637119	NA
ENSG0000028	RP11-224E16.	lncRNA	-0.34262729	0.91637119	NA
ENSG0000028	RP11-256B12.	lncRNA	-0.34262729	0.91637119	NA
ENSG0000023	HHATL-AS1	lncRNA	-0.34262729	0.91637119	NA
ENSG0000028	RP11-509I21.3	lncRNA	-0.34262729	0.91637119	NA
ENSG0000028	RP11-253K11.	lncRNA	-0.34262729	0.91637119	NA
ENSG0000017	FRG2C	protein_codir	-0.34262729	0.91637119	NA
ENSG0000024	CD200R1L-AS	lncRNA	-0.34262729	0.91637119	NA
ENSG0000024	LSAMP-AS1	lncRNA	-0.34262729	0.91637119	NA
ENSG0000023	PLCH1-AS1	lncRNA	-0.34262729	0.91637119	NA
ENSG0000021	RP11-674E16.	lncRNA	-0.34262729	0.91637119	NA
ENSG0000027	RP11-368I23.4	lncRNA	-0.34262729	0.91637119	NA
ENSG0000024	MECOM-AS1	lncRNA	-0.34262729	0.91637119	NA
ENSG0000023	TBL1XR1-AS1	lncRNA	-0.34262729	0.91637119	NA
ENSG0000028	RP11-513G11	lncRNA	-0.34262729	0.91637119	NA
ENSG0000027	RP11-534C12.	lncRNA	-0.34262729	0.91637119	NA
ENSG0000017	PSAPL1	protein_codir	-0.34262729	0.91637119	NA
ENSG0000020	AC024132.1	lncRNA	-0.34262729	0.91637119	NA
ENSG0000025	RP11-180C1.1	lncRNA	-0.34262729	0.91637119	NA
ENSG0000025	RP11-123O22	lncRNA	-0.34262729	0.91637119	NA
ENSG0000025	RP11-315A17	lncRNA	-0.34262729	0.91637119	NA
ENSG0000025	RP11-632F7.3	lncRNA	-0.34262729	0.91637119	NA
ENSG0000024	LNX1-AS2	lncRNA	-0.34262729	0.91637119	NA
ENSG0000028	RP11-25H12.2	lncRNA	-0.34262729	0.91637119	NA
ENSG0000019	TMPRSS11F	protein_codir	-0.34262729	0.91637119	NA
ENSG0000018	TMPRSS11B	protein_codir	-0.34262729	0.91637119	NA
ENSG0000012	STATH	protein_codir	-0.34262729	0.91637119	NA
ENSG0000018	AMTN	protein_codir	-0.34262729	0.91637119	NA
ENSG0000023	LINC00575	lncRNA	-0.34262729	0.91637119	NA
ENSG0000025	RP11-427M20	lncRNA	-0.34262729	0.91637119	NA
ENSG0000024	RP11-733C7.1	lncRNA	-0.34262729	0.91637119	NA
ENSG0000025	LINC02272	lncRNA	-0.34262729	0.91637119	NA
ENSG0000024	LINC02275	lncRNA	-0.34262729	0.91637119	NA
ENSG0000028	RP11-40L7.2	lncRNA	-0.34262729	0.91637119	NA
ENSG0000027	RP11-215A19	protein_codir	-0.34262729	0.91637119	NA
ENSG0000024	RP11-366H4.3	lncRNA	-0.34262729	0.91637119	NA
ENSG0000020	FRG2	protein_codir	-0.34262729	0.91637119	NA
ENSG0000022	LINC02142	lncRNA	-0.34262729	0.91637119	NA
ENSG0000025	ANKRD33B-AS	lncRNA	-0.34262729	0.91637119	NA
ENSG0000024	TAF11L12	protein_codir	-0.34262729	0.91637119	NA
ENSG0000028	TAF11L13	protein_codir	-0.34262729	0.91637119	NA
ENSG0000024	RP11-5N11.3	lncRNA	-0.34262729	0.91637119	NA
ENSG0000024	RP11-479O16	lncRNA	-0.34262729	0.91637119	NA
ENSG0000025	LINC02229	lncRNA	-0.34262729	0.91637119	NA
ENSG0000018	MINAR2	protein_codir	-0.34262729	0.91637119	NA
ENSG0000024	CTC-276P9.2	lncRNA	-0.34262729	0.91637119	NA
ENSG0000025	CTB-35F21.4	lncRNA	-0.34262729	0.91637119	NA

ENSG0000027	CTB-108O6.2	lncRNA	-0.34262729	0.91637119	NA
ENSG0000028	CTB-43E15.5	lncRNA	-0.34262729	0.91637119	NA
ENSG0000028	MSANTD5	protein_codir	-0.34262729	0.91637119	NA
ENSG0000023	RP3-400B16.3	lncRNA	-0.34262729	0.91637119	NA
ENSG0000023	LINC02522	lncRNA	-0.34262729	0.91637119	NA
ENSG0000027	RP11-239L20.	lncRNA	-0.34262729	0.91637119	NA
ENSG0000020	C6orf15	protein_codir	-0.34262729	0.91637119	NA
ENSG0000022	BTBD9-AS1	lncRNA	-0.34262729	0.91637119	NA
ENSG0000022	FAM135A-AS1	lncRNA	-0.34262729	0.91637119	NA
ENSG0000022	LINC01526	lncRNA	-0.34262729	0.91637119	NA
ENSG0000026	RP1-149C7.1	lncRNA	-0.34262729	0.91637119	NA
ENSG0000028	RP11-64I5.2	lncRNA	-0.34262729	0.91637119	NA
ENSG0000023	RP3-355L5.4	lncRNA	-0.34262729	0.91637119	NA
ENSG0000022	REV3L-IT1	lncRNA	-0.34262729	0.91637119	NA
ENSG0000027	RP1-142L7.8	lncRNA	-0.34262729	0.91637119	NA
ENSG0000022	RP11-497D6.3	lncRNA	-0.34262729	0.91637119	NA
ENSG0000022	RP1-111C20.5	lncRNA	-0.34262729	0.91637119	NA
ENSG0000028	RP11-459F1.4	lncRNA	-0.34262729	0.91637119	NA
ENSG0000022	RP11-503C24.	lncRNA	-0.34262729	0.91637119	NA
ENSG0000023	RP1-125N5.2	lncRNA	-0.34262729	0.91637119	NA
ENSG0000024	AC093627.12	lncRNA	-0.34262729	0.91637119	NA
ENSG0000022	AC024028.1	lncRNA	-0.34262729	0.91637119	NA
ENSG0000022	AC005013.5	lncRNA	-0.34262729	0.91637119	NA
ENSG0000026	RP11-196O2.1	lncRNA	-0.34262729	0.91637119	NA
ENSG0000023	AC010132.10	lncRNA	-0.34262729	0.91637119	NA
ENSG0000022	ZNF735	protein_codir	-0.34262729	0.91637119	NA
ENSG0000027	CH17-264B6.1	lncRNA	-0.34262729	0.91637119	NA
ENSG0000027	CTB-13L3.1	lncRNA	-0.34262729	0.91637119	NA
ENSG0000021	SPDYE3	protein_codir	-0.34262729	0.91637119	NA
ENSG0000028	AC002057.1	lncRNA	-0.34262729	0.91637119	NA
ENSG0000025	OR9A4	protein_codir	-0.34262729	0.91637119	NA
ENSG0000021	AC008060.7	lncRNA	-0.34262729	0.91637119	NA
ENSG0000028	FAM90A10P	protein_codir	-0.34262729	0.91637119	NA
ENSG0000025	RP11-211C9.1	lncRNA	-0.34262729	0.91637119	NA
ENSG0000025	RP11-981G7.3	lncRNA	-0.34262729	0.91637119	NA
ENSG0000014	CRH	protein_codir	-0.34262729	0.91637119	NA
ENSG0000028	RP11-62E9.2	lncRNA	-0.34262729	0.91637119	NA
ENSG0000025	RP11-157I4.4	lncRNA	-0.34262729	0.91637119	NA
ENSG0000025	RP11-219B4.3	lncRNA	-0.34262729	0.91637119	NA
ENSG0000025	RP11-587H10	lncRNA	-0.34262729	0.91637119	NA
ENSG0000025	LINC01609	lncRNA	-0.34262729	0.91637119	NA
ENSG0000026	RP11-973F15.	lncRNA	-0.34262729	0.91637119	NA
ENSG0000025	HPYR1	lncRNA	-0.34262729	0.91637119	NA
ENSG0000026	AP006547.3	lncRNA	-0.34262729	0.91637119	NA
ENSG0000013	DMRT1	protein_codir	-0.34262729	0.91637119	NA
ENSG0000017	TPD52L3	protein_codir	-0.34262729	0.91637119	NA
ENSG0000023	RP11-462B18.	lncRNA	-0.34262729	0.91637119	NA

ENSG0000023	LINC01400	lncRNA	-0.34262729	0.91637119	NA
ENSG0000028	RP11-159H20	lncRNA	-0.34262729	0.91637119	NA
ENSG0000028	RP11-533K9.5	lncRNA	-0.34262729	0.91637119	NA
ENSG0000023	LINC02643	lncRNA	-0.34262729	0.91637119	NA
ENSG0000022	RP11-166N17	lncRNA	-0.34262729	0.91637119	NA
ENSG0000022	RP11-563N6.6	lncRNA	-0.34262729	0.91637119	NA
ENSG0000023	RP11-135D11	lncRNA	-0.34262729	0.91637119	NA
ENSG0000023	RP11-399K21.	lncRNA	-0.34262729	0.91637119	NA
ENSG0000017	LIPM	protein_codir	-0.34262729	0.91637119	NA
ENSG0000028	RP11-30G13.1	lncRNA	-0.34262729	0.91637119	NA
ENSG0000025	INS	protein_codir	-0.34262729	0.91637119	NA
ENSG0000018	OR52L1	protein_codir	-0.34262729	0.91637119	NA
ENSG0000018	OR56A4	protein_codir	-0.34262729	0.91637119	NA
ENSG0000025	OR52B2	protein_codir	-0.34262729	0.91637119	NA
ENSG0000025	RP11-406D1.2	lncRNA	-0.34262729	0.91637119	NA
ENSG0000025	RP11-624D11	lncRNA	-0.34262729	0.91637119	NA
ENSG0000025	LINC02687	lncRNA	-0.34262729	0.91637119	NA
ENSG0000018	TRIM49B	protein_codir	-0.34262729	0.91637119	NA
ENSG0000028	OOSP3	protein_codir	-0.34262729	0.91637119	NA
ENSG0000025	RP11-881M11	lncRNA	-0.34262729	0.91637119	NA
ENSG0000025	RP11-655M14	lncRNA	-0.34262729	0.91637119	NA
ENSG0000026	RP11-211G23	lncRNA	-0.34262729	0.91637119	NA
ENSG0000020	KRTAP5-11	protein_codir	-0.34262729	0.91637119	NA
ENSG0000025	RP11-313I2.1	lncRNA	-0.34262729	0.91637119	NA
ENSG0000022	TRIM49D1	protein_codir	-0.34262729	0.91637119	NA
ENSG0000025	RP11-338H14	lncRNA	-0.34262729	0.91637119	NA
ENSG0000025	LINC02552	lncRNA	-0.34262729	0.91637119	NA
ENSG0000025	RP11-977P2.1	lncRNA	-0.34262729	0.91637119	NA
ENSG0000028	RP11-624J6.2	protein_codir	-0.34262729	0.91637119	NA
ENSG0000025	RP11-644F5.1	lncRNA	-0.34262729	0.91637119	NA
ENSG0000011	MYF5	protein_codir	-0.34262729	0.91637119	NA
ENSG0000025	RP11-397H6.1	lncRNA	-0.34262729	0.91637119	NA
ENSG0000025	RP11-173P15.	lncRNA	-0.34262729	0.91637119	NA
ENSG0000028	RP11-483I24.1	lncRNA	-0.34262729	0.91637119	NA
ENSG0000028	RP11-749H20	lncRNA	-0.34262729	0.91637119	NA
ENSG0000025	RP13-895J2.7	lncRNA	-0.34262729	0.91637119	NA
ENSG0000022	ANHX	protein_codir	-0.34262729	0.91637119	NA
ENSG0000016	GSX1	protein_codir	-0.34262729	0.91637119	NA
ENSG0000028	RP11-179A7.4	lncRNA	-0.34262729	0.91637119	NA
ENSG0000022	RP11-279N8.1	lncRNA	-0.34262729	0.91637119	NA
ENSG0000027	RP11-52L5.6	lncRNA	-0.34262729	0.91637119	NA
ENSG0000028	RP11-470M1.	lncRNA	-0.34262729	0.91637119	NA
ENSG0000028	RP11-66G15.1	lncRNA	-0.34262729	0.91637119	NA
ENSG0000022	RP11-120E13.	lncRNA	-0.34262729	0.91637119	NA
ENSG0000025	LINC02297	lncRNA	-0.34262729	0.91637119	NA
ENSG0000025	RP11-516J2.1	lncRNA	-0.34262729	0.91637119	NA
ENSG0000028	RP11-242P2.3	lncRNA	-0.34262729	0.91637119	NA

ENSG0000019	LINC00523	lncRNA	-0.34262729	0.91637119	NA
ENSG0000025	RP11-463I20.1	lncRNA	-0.34262729	0.91637119	NA
ENSG0000025	RP11-325E5.1	lncRNA	-0.34262729	0.91637119	NA
ENSG0000025	RP11-313P18.	lncRNA	-0.34262729	0.91637119	NA
ENSG0000026	NTRK3-AS1	lncRNA	-0.34262729	0.91637119	NA
ENSG0000025	RP11-255M2.	lncRNA	-0.34262729	0.91637119	NA
ENSG0000026	RP11-473M20	lncRNA	-0.34262729	0.91637119	NA
ENSG0000026	RP11-82O18.2	lncRNA	-0.34262729	0.91637119	NA
ENSG0000026	RP11-719K4.6	lncRNA	-0.34262729	0.91637119	NA
ENSG0000026	COQ7-DT	lncRNA	-0.34262729	0.91637119	NA
ENSG0000026	LCMT1-AS2	lncRNA	-0.34262729	0.91637119	NA
ENSG0000025	CTD-3032H12	lncRNA	-0.34262729	0.91637119	NA
ENSG0000026	RP11-407G23	lncRNA	-0.34262729	0.91637119	NA
ENSG0000026	RP11-405F3.4	lncRNA	-0.34262729	0.91637119	NA
ENSG0000026	RP11-96D1.6	lncRNA	-0.34262729	0.91637119	NA
ENSG0000028	CPHXL	protein_codir	-0.34262729	0.91637119	NA
ENSG0000028	RP11-150D5.3	lncRNA	-0.34262729	0.91637119	NA
ENSG0000028	RP11-22H5.3	lncRNA	-0.34262729	0.91637119	NA
ENSG0000023	AC130689.5	lncRNA	-0.34262729	0.91637119	NA
ENSG0000012	OR1E2	protein_codir	-0.34262729	0.91637119	NA
ENSG0000028	RP11-663N22	lncRNA	-0.34262729	0.91637119	NA
ENSG0000026	RP11-848P1.3	lncRNA	-0.34262729	0.91637119	NA
ENSG0000026	RP11-805L22.	lncRNA	-0.34262729	0.91637119	NA
ENSG0000024	LINC02075	lncRNA	-0.34262729	0.91637119	NA
ENSG0000026	RP11-893F2.1	lncRNA	-0.34262729	0.91637119	NA
ENSG0000022	AC015923.1	lncRNA	-0.34262729	0.91637119	NA
ENSG0000026	RP11-28G8.1	lncRNA	-0.34262729	0.91637119	NA
ENSG0000026	RP11-53B2.5	lncRNA	-0.34262729	0.91637119	NA
ENSG0000026	RP11-296E23.	lncRNA	-0.34262729	0.91637119	NA
ENSG0000027	ST8SIA5-DT	lncRNA	-0.34262729	0.91637119	NA
ENSG0000026	RP11-110H1.9	lncRNA	-0.34262729	0.91637119	NA
ENSG0000026	RP11-795H16	lncRNA	-0.34262729	0.91637119	NA
ENSG0000027	LINC01879	lncRNA	-0.34262729	0.91637119	NA
ENSG0000026	RP11-63N3.1	lncRNA	-0.34262729	0.91637119	NA
ENSG0000026	CBARP-DT	lncRNA	-0.34262729	0.91637119	NA
ENSG0000017	RAX2	protein_codir	-0.34262729	0.91637119	NA
ENSG0000019	MBD3L2B	protein_codir	-0.34262729	0.91637119	NA
ENSG0000020	MBD3L4	protein_codir	-0.34262729	0.91637119	NA
ENSG0000013	TSPAN16	protein_codir	-0.34262729	0.91637119	NA
ENSG0000018	OR7A17	protein_codir	-0.34262729	0.91637119	NA
ENSG0000026	RP11-157B13.	lncRNA	-0.34262729	0.91637119	NA
ENSG0000026	L34079.2	protein_codir	-0.34262729	0.91637119	NA
ENSG0000028	CTD-2224J9.1	lncRNA	-0.34262729	0.91637119	NA
ENSG0000026	AC006116.12	lncRNA	-0.34262729	0.91637119	NA
ENSG0000028	RP5-1103G7.1	lncRNA	-0.34262729	0.91637119	NA
ENSG0000028	RP5-1128N12	lncRNA	-0.34262729	0.91637119	NA
ENSG0000018	DEFB119	protein_codir	-0.34262729	0.91637119	NA

ENSG0000020	CTD-2308N23	lncRNA	-0.34262729	0.91637119	NA
ENSG0000022	RP1-138B7.5	lncRNA	-0.34262729	0.91637119	NA
ENSG0000023	RIPOR3-AS1	lncRNA	-0.34262729	0.91637119	NA
ENSG0000027	CH507-210P1	lncRNA	-0.34262729	0.91637119	NA
ENSG0000022	KRTAP10-10	protein_codir	-0.34262729	0.91637119	NA
ENSG0000027	CTA-243E7.3	lncRNA	-0.34262729	0.91637119	NA
ENSG0000027	RP3-510H16.3	lncRNA	-0.34262729	0.91637119	NA
ENSG0000023	RP6-109B7.2	lncRNA	-0.34262729	0.91637119	NA
ENSG0000021	RP1-29C18.10	lncRNA	-0.34262729	0.91637119	NA
ENSG0000023	ZFX-AS1	lncRNA	-0.34262729	0.91637119	NA
ENSG0000028	RP11-486N10	lncRNA	-0.34262729	0.91637119	NA
ENSG0000014	CPXCR1	protein_codir	-0.34262729	0.91637119	NA
ENSG0000010	LUZP4	protein_codir	-0.34262729	0.91637119	NA
ENSG0000022	RP5-1139I1.2	lncRNA	-0.34262729	0.91637119	NA
ENSG0000028	RP13-63I15.1	lncRNA	-0.34262729	0.91637119	NA
ENSG0000014	GPR119	protein_codir	-0.34262729	0.91637119	NA
ENSG0000020	SPANXA2	protein_codir	-0.34262729	0.91637119	NA
ENSG0000022	RP1-171K16.5	lncRNA	-0.34262729	0.91637119	NA
ENSG0000023	RP11-329E24.	lncRNA	-0.34262729	0.91637119	NA
ENSG0000027	ENSG0000027	protein_codir	-0.34262729	0.91637119	NA
ENSG0000002	TFB1M	protein_codir	-0.34263808	0.0440294	0.10927235
ENSG0000011	ST6GALNAC5	protein_codir	-0.34277139	0.48426861	0.631605
ENSG0000017	ZNF415	protein_codir	-0.34278912	0.20580244	0.34383835
ENSG0000000	POLR2J	protein_codir	-0.34291716	0.00483667	0.01977043
ENSG0000015	NEK7	protein_codir	-0.34293357	0.22012645	0.360953
ENSG0000018	COPB2	protein_codir	-0.34302258	0.01885039	0.05667354
ENSG0000027	RP11-104O19	lncRNA	-0.34307861	0.79162571	0.87041011
ENSG0000013	DPH6	protein_codir	-0.34312676	0.12900311	0.24377846
ENSG0000028	RP11-678N14	lncRNA	-0.34328603	0.5741435	0.70683502
ENSG0000017	MTX3	protein_codir	-0.34333657	0.06992531	0.15497809
ENSG0000027	RP11-325L12.	lncRNA	-0.34338157	0.72625048	0.82374598
ENSG0000013	UBE4B	protein_codir	-0.34370722	0.02943169	0.08030259
ENSG0000019	ZNF607	protein_codir	-0.34375734	0.23729608	0.38140816
ENSG0000012	CSE1L	protein_codir	-0.34381215	0.01264312	0.04170085
ENSG0000020	GMNC	protein_codir	-0.34392886	0.81438392	0.88580648
ENSG0000006	HAGH	protein_codir	-0.34394034	0.04050748	0.10248388
ENSG0000011	OSBP	protein_codir	-0.3439505	0.03652497	0.09456504
ENSG0000025	THTPA	protein_codir	-0.34399751	0.00187989	0.00935372
ENSG0000019	DYNC1H1	protein_codir	-0.34438407	0.0816062	0.17326184
ENSG0000006	PKM	protein_codir	-0.3444539	0.02976818	0.08100465
ENSG0000007	CYBRD1	protein_codir	-0.34468137	0.40004885	0.5533261
ENSG0000000	PHTF2	protein_codir	-0.34470591	0.12550589	0.23902355
ENSG0000016	ZNF22-AS1	lncRNA	-0.34473275	0.10886045	0.21505797
ENSG0000023	TRHDE-AS1	lncRNA	-0.3448578	0.57809414	0.71030563
ENSG0000012	HIP1	protein_codir	-0.34487672	0.11597716	0.22521895
ENSG0000027	RP11-507M3.	protein_codir	-0.3450675	0.50882948	0.65318229
ENSG0000016	ZNF747	protein_codir	-0.34507835	0.00268334	0.01236466

ENSG0000027	RP11-15A1.8	lncRNA	-0.34514273	0.45003422	0.60085779
ENSG0000013	TMPRSS4	protein_codir	-0.34527302	0.83640618	0.90162901
ENSG0000026	ZNF594-DT	lncRNA	-0.34542718	0.20467744	0.34236864
ENSG0000016	FZD6	protein_codir	-0.3454576	0.1354103	0.25246607
ENSG0000011	SCAP	protein_codir	-0.34549082	0.00010741	0.00092365
ENSG0000022	LINC01132	lncRNA	-0.34565235	0.49353543	0.64002355
ENSG0000010	OGDH	protein_codir	-0.34566949	0.03829235	0.09803807
ENSG0000016	GOLGA6C	protein_codir	-0.34580714	0.78323055	0.86437958
ENSG0000018	RGMA	protein_codir	-0.34590363	0.4208521	0.57295797
ENSG0000027	RP11-543P15.	lncRNA	-0.34598972	0.74729537	0.83865142
ENSG0000025	RP11-503E24.	lncRNA	-0.34600003	0.56283238	0.69747982
ENSG0000016	FAM110B	protein_codir	-0.34613745	0.22250565	0.36370989
ENSG0000002	NDUFS1	protein_codir	-0.34617046	0.07901252	0.16912249
ENSG0000013	CPEB2	protein_codir	-0.34620863	0.24117604	0.38580345
ENSG0000011	CPT1A	protein_codir	-0.34637263	0.14797805	0.26970792
ENSG0000027	TP53RK-DT	lncRNA	-0.34643005	0.59368926	0.722461
ENSG0000016	ZNF12	protein_codir	-0.34647578	0.03246889	0.08641115
ENSG0000020	SPIN3	protein_codir	-0.34656106	0.06071927	0.13925652
ENSG0000018	KCNJ11	protein_codir	-0.34671371	0.4578914	0.60831941
ENSG0000012	MINDY2	protein_codir	-0.3467286	0.1396926	0.25853053
ENSG0000014	GOLGA4	protein_codir	-0.34682851	0.07642037	0.16515889
ENSG0000024	WDR92	protein_codir	-0.347106	0.02000406	0.05931425
ENSG0000009	HNRNPH3	protein_codir	-0.3474475	0.00281762	0.01283565
ENSG0000017	C9orf16	protein_codir	-0.34750204	0.02484455	0.07025764
ENSG0000026	AC024592.12	protein_codir	-0.34753458	0.42589475	0.57773258
ENSG0000022	SMIM10L2B-A	lncRNA	-0.34769624	0.79854373	NA
ENSG0000010	CPQ	protein_codir	-0.34775336	0.19582069	0.33161052
ENSG0000008	MPP4	protein_codir	-0.34780979	0.70453513	0.80773983
ENSG0000015	PTPRR	protein_codir	-0.34781721	0.50137785	0.6461091
ENSG0000015	ZCCHC10	protein_codir	-0.34810904	0.00703866	0.02643696
ENSG0000012	RALY	protein_codir	-0.34822624	0.02163109	0.06298237
ENSG0000013	LNX2	protein_codir	-0.34830739	0.08264338	0.17469976
ENSG0000016	DPCD	protein_codir	-0.34838013	0.09660895	0.19656274
ENSG0000003	METTL1	protein_codir	-0.34842928	0.07991932	0.17046657
ENSG0000010	STN1	protein_codir	-0.34851737	0.13342103	0.250023
ENSG0000018	TMEM203	protein_codir	-0.34857401	0.0016979	0.00868304
ENSG0000011	PLEKHA3	protein_codir	-0.34862925	0.04110001	0.10366962
ENSG0000010	ZBTB16	protein_codir	-0.34868999	0.4230078	0.57504872
ENSG0000016	LRRC28	protein_codir	-0.34880973	0.02218688	0.06423498
ENSG0000024	NOP14-AS1	lncRNA	-0.34883482	0.06191576	0.14141349
ENSG0000015	PALM2AKAP2	protein_codir	-0.34894864	0.17316597	0.302865
ENSG0000016	MRPL1	protein_codir	-0.34917348	0.00239087	0.01129204
ENSG0000019	NHLRC2	protein_codir	-0.34939086	0.03389139	0.08936936
ENSG0000012	DTD2	protein_codir	-0.34945012	0.0789133	0.16897319
ENSG0000010	TIMM44	protein_codir	-0.34966089	0.03268622	0.08688225
ENSG0000020	NBDY	protein_codir	-0.34978194	0.00384487	0.01648738
ENSG0000011	EPHA4	protein_codir	-0.34978561	0.16799792	0.29637856

ENSG0000013	TUBGCP4	protein_codir	-0.34985467	0.00043636	0.00292451
ENSG0000014	ZMAT2	protein_codir	-0.34994124	0.00014554	0.00119568
ENSG0000011	MRPL51	protein_codir	-0.35012339	0.00277053	0.01267342
ENSG0000015	NR3C2	protein_codir	-0.35023393	0.1937722	0.32919106
ENSG0000027	LINC01971	lncRNA	-0.35031496	0.78234315	0.86392907
ENSG0000017	GNB2	protein_codir	-0.35032981	0.02075056	0.06101026
ENSG0000014	CLVS2	protein_codir	-0.35036505	0.79833513	0.87468826
ENSG0000023	JRK	protein_codir	-0.3505177	0.1492041	0.27140236
ENSG0000020	FAM216A	protein_codir	-0.35053569	0.00991073	0.03456961
ENSG0000012	NR1D1	protein_codir	-0.35057365	0.41696237	0.56964439
ENSG0000018	MRPL14	protein_codir	-0.35058282	0.01761209	0.05378561
ENSG0000008	PCNP	protein_codir	-0.35071223	0.07012761	0.15529921
ENSG0000016	ADAM9	protein_codir	-0.35074957	0.2423748	0.38725006
ENSG0000011	GDA	protein_codir	-0.35083095	0.76372322	0.85084041
ENSG0000024	LINC00882	lncRNA	-0.35085586	0.35568631	0.50928232
ENSG0000023	MRAP-AS1	lncRNA	-0.35086534	0.79064766	0.86961168
ENSG0000013	CHMP2A	protein_codir	-0.35090435	0.01156765	0.0390251
ENSG0000010	SAMD15	protein_codir	-0.35091507	0.47540454	0.62367402
ENSG0000015	ATP5MC1	protein_codir	-0.35118717	0.03858081	0.09864858
ENSG0000002	ZFP64	protein_codir	-0.35134892	0.0375015	0.09646037
ENSG0000000	RAD52	protein_codir	-0.35175568	0.21721209	0.35746874
ENSG0000016	NPY1R	protein_codir	-0.35175808	0.55348815	0.68980993
ENSG0000023	PHKA2-AS1	lncRNA	-0.35178633	0.4269669	0.57876059
ENSG0000011	STX2	protein_codir	-0.35182018	0.06105048	0.13980718
ENSG0000014	MBOAT2	protein_codir	-0.35182486	0.08942259	0.18539641
ENSG0000008	PTHLH	protein_codir	-0.35189667	0.39491218	0.54786651
ENSG0000015	CBR3	protein_codir	-0.3519037	0.11288848	0.22076028
ENSG0000018	ZFP91	protein_codir	-0.35219614	0.06775573	0.15155599
ENSG0000010	CSTF2	protein_codir	-0.3522652	0.02811041	0.07741645
ENSG0000024	OSMR-AS1	lncRNA	-0.3523274	0.22921169	0.37173045
ENSG0000010	TMEM106B	protein_codir	-0.35238992	0.12894605	0.24373732
ENSG0000015	SAV1	protein_codir	-0.35253527	0.10892393	0.2151526
ENSG0000017	CEP295NL	protein_codir	-0.3526256	0.2498184	0.39573206
ENSG0000013	SCLY	protein_codir	-0.35281592	0.03435993	0.09037438
ENSG0000027	RP11-44N11.3	lncRNA	-0.35284579	0.83365777	NA
ENSG0000023	RP11-108M9.	lncRNA	-0.35285896	0.18690974	0.32041008
ENSG0000015	C8orf37	protein_codir	-0.35295591	0.1124212	0.22013797
ENSG0000012	HOXB5	protein_codir	-0.35302838	0.24022161	0.38469999
ENSG0000018	MAPT	protein_codir	-0.35314742	0.56366817	0.69810838
ENSG0000015	SRSF12	protein_codir	-0.35319201	0.26555959	0.41310932
ENSG0000028	RP5-1184F4.8	lncRNA	-0.35319893	0.68466345	0.79275612
ENSG0000022	RP1-18D14.7	lncRNA	-0.35335803	0.8790911	NA
ENSG0000003	CYP46A1	protein_codir	-0.35364714	0.23340278	0.37664085
ENSG0000014	MSN	protein_codir	-0.35371002	0.06189346	0.14137425
ENSG0000027	RP5-1074L1.4	lncRNA	-0.35379941	0.47086626	0.61986207
ENSG0000023	LINC00384	lncRNA	-0.35416797	0.71770405	0.8176113
ENSG0000024	RP11-115C21.	lncRNA	-0.35440706	0.19126905	0.32585391

ENSG0000012	LRRFIP1	protein_codir	-0.35445787	0.15319455	0.27664873
ENSG0000007	DLG1	protein_codir	-0.35446197	0.01445567	0.04631853
ENSG0000011	HMGCL	protein_codir	-0.35446292	0.00442543	0.01844152
ENSG0000011	MOB4	protein_codir	-0.35455207	0.04365615	0.10857673
ENSG0000014	PTPRF	protein_codir	-0.35460772	0.12432037	0.23725065
ENSG0000028	LINC01230	lncRNA	-0.3550062	0.63874001	0.75789475
ENSG0000016	SRP68	protein_codir	-0.35501171	0.00049515	0.00324463
ENSG0000010	CINP	protein_codir	-0.35520113	0.0085063	0.03063155
ENSG0000028	RP11-725L2.2	lncRNA	-0.35524397	0.88033665	NA
ENSG0000025	RP11-804A23	lncRNA	-0.35537579	0.87943332	NA
ENSG0000015	ANKH	protein_codir	-0.35541507	0.22503775	0.36680688
ENSG0000011	SNX15	protein_codir	-0.35545238	0.01061162	0.0364775
ENSG0000016	DNALI1	protein_codir	-0.35556874	0.16929964	0.29785693
ENSG0000025	PRR23D2	protein_codir	-0.35570221	0.87805682	NA
ENSG0000003	CHPF2	protein_codir	-0.35583089	0.06612872	0.14891767
ENSG0000027	RP11-227D13	lncRNA	-0.35589847	0.87591114	NA
ENSG0000003	ABCF2	protein_codir	-0.35597752	0.01646892	0.05116665
ENSG0000017	PFKFB3	protein_codir	-0.35603711	0.31241984	0.46421223
ENSG0000027	RP1-41C23.4	lncRNA	-0.35604159	0.6737234	0.78479356
ENSG0000017	MORN4	protein_codir	-0.35611072	0.05160087	0.12324016
ENSG0000016	PACRGL	protein_codir	-0.35620566	0.03615654	0.09381553
ENSG0000026	RP11-923I11.:	lncRNA	-0.35633726	0.83289821	NA
ENSG0000015	SLC26A2	protein_codir	-0.35640883	0.16866983	0.29709937
ENSG0000011	CD81	protein_codir	-0.35644908	0.02578984	0.07233096
ENSG0000017	CTU2	protein_codir	-0.35651429	0.03598994	0.09348653
ENSG0000017	IRF2BP1	protein_codir	-0.35664029	0.02347871	0.06712369
ENSG0000016	TMEM183A	protein_codir	-0.35670511	0.00062284	0.00390975
ENSG0000011	FAM184A	protein_codir	-0.35693232	0.23539288	0.37890036
ENSG0000023	MTRNR2L4	protein_codir	-0.35694779	0.85543869	NA
ENSG0000026	RP11-104H15	lncRNA	-0.35707214	0.85469619	NA
ENSG0000013	FBH1	protein_codir	-0.35713941	0.00027845	0.00202978
ENSG0000027	RP11-394I13.:	lncRNA	-0.35718516	0.59158709	0.72076028
ENSG0000025	RP4-791M13.	lncRNA	-0.35723936	0.41404081	0.56716794
ENSG0000014	TMEFF2	protein_codir	-0.35726772	0.68095201	0.79028087
ENSG0000019	SLC9A6	protein_codir	-0.35729039	0.03723912	0.09602692
ENSG0000027	CTD-2036P10	lncRNA	-0.35735199	0.64117523	0.75936966
ENSG0000016	NDUFV3	protein_codir	-0.35763528	0.01108599	0.03775525
ENSG0000018	MANEAL	protein_codir	-0.35803172	0.05828097	0.13490784
ENSG0000011	TMEM214	protein_codir	-0.35812011	0.00996548	0.03470952
ENSG0000013	LMO7	protein_codir	-0.35814344	0.33644021	0.4893869
ENSG0000010	GRB10	protein_codir	-0.35826427	0.11870218	0.22910575
ENSG0000028	RP11-472I20.4	lncRNA	-0.35827575	0.5438354	0.68214049
ENSG0000016	NDEL1	protein_codir	-0.35837415	0.11696704	0.22661544
ENSG0000018	SLC25A41	protein_codir	-0.35844491	0.64573257	0.76319627
ENSG0000017	KLHL11	protein_codir	-0.35849255	0.19401421	0.32946034
ENSG0000023	CDKN2AIPNL	protein_codir	-0.35857321	0.00050215	0.00328192
ENSG0000026	ZNRD2-AS1	lncRNA	-0.35870823	0.27003997	0.41786678

ENSG0000017	ZNF449	protein_codir	-0.35873225	0.10102081	0.2033366
ENSG0000011	ST3GAL4	protein_codir	-0.35905347	0.03682628	0.09519343
ENSG0000021	SMIM30	protein_codir	-0.3590856	0.00211902	0.01027179
ENSG0000023	RP11-307E17.	lncRNA	-0.35927685	0.33821139	0.49105811
ENSG0000013	IAH1	protein_codir	-0.35927773	0.02563193	0.07200853
ENSG0000011	PPP2R5D	protein_codir	-0.35939795	0.00111902	0.006254
ENSG0000024	RP11-304F15.	lncRNA	-0.3596298	0.85322503	NA
ENSG0000025	RTEL1	protein_codir	-0.35966309	0.06436442	0.1458603
ENSG0000020	ZCWPW2	protein_codir	-0.35974367	0.12637472	0.24024158
ENSG0000016	TMED3	protein_codir	-0.3597453	0.11151116	0.21888553
ENSG0000017	PCCA	protein_codir	-0.35997499	0.04298797	0.1073339
ENSG0000016	PYGO2	protein_codir	-0.36006793	0.04256247	0.10657983
ENSG0000016	BBS5	protein_codir	-0.36006808	0.07232026	0.15880789
ENSG0000015	LCA5L	protein_codir	-0.36017804	0.17390881	0.30391404
ENSG0000026	RP11-697E2.6	protein_codir	-0.36020737	0.61664161	0.74044176
ENSG0000024	FLJ46284	lncRNA	-0.36047872	0.39727109	0.55017185
ENSG0000008	SCAMP1	protein_codir	-0.360539	0.07810719	0.1676761
ENSG0000000	CDKL3	protein_codir	-0.36056295	0.21703725	0.35728745
ENSG0000027	RP11-554D15	lncRNA	-0.36061838	0.84778405	NA
ENSG0000024	CTD-2288O8.1	lncRNA	-0.36073513	0.48432492	0.63164862
ENSG0000026	STAM-AS1	lncRNA	-0.36077526	0.58880795	0.71881521
ENSG0000015	CFDP1	protein_codir	-0.36087203	0.00919335	0.03260674
ENSG0000006	MRPS24	protein_codir	-0.36106707	0.00841017	0.03034227
ENSG0000019	IPO9	protein_codir	-0.36123568	0.00626387	0.02414612
ENSG0000018	TSPYL2	protein_codir	-0.36125523	0.26268116	0.41015076
ENSG0000013	FBXO21	protein_codir	-0.36126587	0.04499981	0.11121105
ENSG0000016	CAMK2N1	protein_codir	-0.36128029	0.31075832	0.46257854
ENSG0000010	BCAT2	protein_codir	-0.36132222	0.01577362	0.04954759
ENSG0000026	RP5-1050D4.5	lncRNA	-0.36137952	0.18584317	0.31909663
ENSG0000027	CTD-2287O16	lncRNA	-0.361394	0.20163822	0.33863843
ENSG0000003	FBXO42	protein_codir	-0.36145031	0.06175841	0.14115774
ENSG0000010	DAAM1	protein_codir	-0.36154909	0.1125928	0.22038219
ENSG0000012	PIGT	protein_codir	-0.36155252	0.04586649	0.11268456
ENSG0000017	CES2	protein_codir	-0.36159114	0.00309759	0.01385739
ENSG0000014	COMMD7	protein_codir	-0.36161334	0.00742561	0.02758141
ENSG0000012	PYROXD1	protein_codir	-0.36166273	0.02072213	0.06095911
ENSG0000013	LARGE1	protein_codir	-0.36167046	0.28093495	0.43021863
ENSG0000025	TAS2R43	protein_codir	-0.36168409	0.66116543	0.77539762
ENSG0000012	ACAT2	protein_codir	-0.36179822	0.10494104	0.20915511
ENSG0000014	ADHFE1	protein_codir	-0.36186008	0.34113407	0.49426234
ENSG0000016	NRG4	protein_codir	-0.36198457	0.75183084	0.84190423
ENSG0000026	RP11-727F15.	lncRNA	-0.36215986	0.11214278	0.21981291
ENSG0000016	ANKS6	protein_codir	-0.36218529	0.03593671	0.0933746
ENSG0000019	ZNF311	protein_codir	-0.36230575	0.20849845	0.34724597
ENSG0000027	RP11-57A19.5	lncRNA	-0.36238751	0.76538955	0.85221557
ENSG0000018	CLCNKB	protein_codir	-0.36239962	0.47344521	0.6218936
ENSG0000023	LINC01783	lncRNA	-0.3626	0.78348894	0.86452657

ENSG0000015	STARD9	protein_codir	-0.36267839	0.16178897	0.28798166
ENSG0000007	JADE1	protein_codir	-0.36271098	0.04599939	0.11297378
ENSG0000011	PRDX6	protein_codir	-0.36294146	0.00957897	0.03363478
ENSG0000017	BBS10	protein_codir	-0.36314347	0.09500679	0.1941324
ENSG0000013	TSTD2	protein_codir	-0.36318256	0.00816587	0.02969133
ENSG0000010	LAMTOR3	protein_codir	-0.36319831	0.01226982	0.04078086
ENSG0000013	PDHA1	protein_codir	-0.36327425	0.00419955	0.01770846
ENSG0000012	RRP36	protein_codir	-0.36327877	0.00690258	0.02604854
ENSG0000021	CEBPZOS	protein_codir	-0.36332648	0.00258772	0.0119961
ENSG0000025	INMT-MINDY4	protein_codir	-0.36337469	0.55255562	0.68922848
ENSG0000016	AGPAT2	protein_codir	-0.36342595	0.03802003	0.09748544
ENSG0000011	EIF1B	protein_codir	-0.36344237	0.07254687	0.15914101
ENSG0000011	SLC35F2	protein_codir	-0.36347477	0.17560214	0.30581371
ENSG0000016	ZCCHC24	protein_codir	-0.36350551	0.15038141	0.27279222
ENSG0000016	STXBP4	protein_codir	-0.36351127	0.00995497	0.03468605
ENSG0000025	RP11-48B3.5	lncRNA	-0.36359964	0.13929077	0.25799431
ENSG0000014	NDUFB9	protein_codir	-0.36374854	0.01478899	0.04720581
ENSG0000028	CTD-2010I16.	protein_codir	-0.36391223	0.75337436	0.84305172
ENSG0000010	ATP6V1D	protein_codir	-0.36406608	0.00992985	0.03462473
ENSG0000016	ZHX1	protein_codir	-0.36422711	0.05516791	0.12949245
ENSG0000026	CLCA4-AS1	lncRNA	-0.36429239	0.16411453	0.29110865
ENSG0000014	ENSA	protein_codir	-0.36433418	0.0162271	0.05065467
ENSG0000019	MRPL21	protein_codir	-0.36433643	0.04044388	0.10236986
ENSG0000010	MTHFD1	protein_codir	-0.36440134	0.02272798	0.06549262
ENSG0000028	RP11-151A10	lncRNA	-0.36450826	0.76505127	0.8519076
ENSG0000013	MRPS36	protein_codir	-0.36452936	0.00198285	0.00976225
ENSG0000007	FRY	protein_codir	-0.364539	0.32404565	0.47635004
ENSG0000014	POGK	protein_codir	-0.36455126	0.04261322	0.10668756
ENSG0000016	MOGAT2	protein_codir	-0.36458284	0.61302945	0.7376437
ENSG0000016	SENP3	protein_codir	-0.3646412	0.00206904	0.01008773
ENSG0000023	AC002310.7	lncRNA	-0.36473841	0.64093827	0.75921924
ENSG0000027	RP11-454E5.4	lncRNA	-0.36484264	0.26858893	0.41633357
ENSG0000018	SCRIB	protein_codir	-0.36492018	4.57E-05	0.00045257
ENSG0000011	MRPL18	protein_codir	-0.36500949	0.01668029	0.05166643
ENSG0000026	TAPT1-AS1	lncRNA	-0.36528503	0.36823933	0.52171173
ENSG0000018	SERPINA11	protein_codir	-0.36549747	0.82339493	NA
ENSG0000015	ZFAND2B	protein_codir	-0.36557466	0.06006662	0.13809552
ENSG0000023	LINC02888	lncRNA	-0.36574639	0.68693767	0.79469217
ENSG0000014	FBXL17	protein_codir	-0.36577112	0.09243155	0.19012519
ENSG0000019	TMEM184B	protein_codir	-0.36593775	0.03773604	0.09692824
ENSG0000010	HSF4	protein_codir	-0.36609611	0.40428615	0.55728819
ENSG0000017	ENDOV	protein_codir	-0.36621611	0.02376631	0.06780555
ENSG0000013	NDFIP1	protein_codir	-0.3662527	0.05669216	0.1321828
ENSG0000016	PRDX2	protein_codir	-0.36631122	0.03062384	0.08273815
ENSG0000008	DDX24	protein_codir	-0.36633452	0.00479795	0.01965122
ENSG0000009	RAB18	protein_codir	-0.36656526	0.00516819	0.02082961
ENSG0000010	DCTN6	protein_codir	-0.36660593	0.04816238	0.11700633

ENSG0000014	DIAPH2	protein_codir	-0.3667345	0.22063811	0.36138437
ENSG0000012	KLC1	protein_codir	-0.36674245	0.00180624	0.00907719
ENSG0000023	RP1-149A16.1	lncRNA	-0.36695689	0.80447395	NA
ENSG0000017	MRPL52	protein_codir	-0.36728769	0.01140956	0.03861913
ENSG0000013	KHDC4	protein_codir	-0.36740513	0.14176299	0.26129421
ENSG0000010	NAA10	protein_codir	-0.36750043	0.0471451	0.11504087
ENSG0000013	CHD1L	protein_codir	-0.36760806	0.01685765	0.05207927
ENSG0000011	TTC33	protein_codir	-0.36776783	0.06540506	0.14772162
ENSG0000014	GREB1L	protein_codir	-0.36778513	0.4853739	0.63267034
ENSG0000016	MECP2	protein_codir	-0.36778879	0.03591051	0.09333286
ENSG0000027	PPM1B-DT	lncRNA	-0.36791037	0.876636	NA
ENSG0000008	PLOD1	protein_codir	-0.36800282	0.10271482	0.2059217
ENSG0000028	HYMAI	lncRNA	-0.36804221	0.38612845	0.53918817
ENSG0000013	LCA5	protein_codir	-0.36815545	0.14910503	0.2712986
ENSG0000027	RP11-66H6.4	lncRNA	-0.36818467	0.76769866	0.8535479
ENSG0000027	RP11-381O6.1	lncRNA	-0.36830265	0.78724212	0.86717825
ENSG0000016	PBXIP1	protein_codir	-0.36854305	0.18541481	0.31861862
ENSG0000021	HNRNPUL2	protein_codir	-0.36862881	0.01374048	0.04452275
ENSG0000025	RP13-672B3.2	lncRNA	-0.36883775	0.82611517	NA
ENSG0000019	ECI2	protein_codir	-0.36898857	0.0013127	0.00711057
ENSG0000010	PPP3CB	protein_codir	-0.36903258	0.05425802	0.12797428
ENSG0000015	LGI2	protein_codir	-0.36910776	0.26333782	0.41069508
ENSG0000014	CETN2	protein_codir	-0.3692551	0.06402526	0.14531761
ENSG0000027	RP11-250B2.6	lncRNA	-0.36926195	0.23877788	0.38300977
ENSG0000013	RSAD1	protein_codir	-0.36928281	0.00273753	0.01255153
ENSG0000017	FEZ2	protein_codir	-0.36937771	0.0221597	0.06418318
ENSG0000016	NKIRAS2	protein_codir	-0.36952844	0.00598691	0.02329322
ENSG0000026	RP11-323N12	lncRNA	-0.36953796	0.36424358	0.51825714
ENSG0000023	RP11-141M1.	lncRNA	-0.36957684	0.48560186	0.63281793
ENSG0000028	CDC42-AS1	lncRNA	-0.36965666	0.70938107	0.81127511
ENSG0000027	RP11-327J17.	lncRNA	-0.36967124	0.4103526	0.56331704
ENSG0000014	SCYL1	protein_codir	-0.36970515	0.00964122	0.03381038
ENSG0000024	LINC01033	lncRNA	-0.36974421	0.76266464	NA
ENSG0000020	PPP1R11	protein_codir	-0.36978132	7.27E-05	0.00067047
ENSG0000010	C5	protein_codir	-0.36989024	0.0293679	0.08016024
ENSG0000026	RP11-303E16.	protein_codir	-0.36989624	0.11893955	0.22949728
ENSG0000011	CLIP4	protein_codir	-0.36991317	0.08403187	0.1769564
ENSG0000018	FAM120C	protein_codir	-0.36997451	0.01145494	0.03873001
ENSG0000018	TAF9B	protein_codir	-0.37012151	0.07688398	0.16584938
ENSG0000027	H4C1	protein_codir	-0.37020199	0.56304249	0.69756084
ENSG0000016	PGAP3	protein_codir	-0.37063871	0.04282914	0.10704153
ENSG0000006	ROCK1	protein_codir	-0.37071066	0.14009691	0.25917458
ENSG0000023	LINC00894	lncRNA	-0.37078758	0.41887764	0.57152598
ENSG0000010	CDC37L1	protein_codir	-0.3709223	0.17903903	0.31019056
ENSG0000026	RP11-573D15	lncRNA	-0.37099915	0.53969099	0.67882259
ENSG0000003	TRIO	protein_codir	-0.37102752	0.10040874	0.2023265
ENSG0000002	RTF2	protein_codir	-0.37108109	0.00090067	0.00526596

ENSG0000005	FNIP2	protein_codir	-0.37109293	0.19893035	0.33527314
ENSG0000026	RP11-53I6.3	lncRNA	-0.37123745	0.74099855	0.83457897
ENSG0000017	PDP2	protein_codir	-0.37125422	0.0560489	0.13103636
ENSG0000011	TFDP2	protein_codir	-0.37125994	0.09858628	0.19961837
ENSG0000010	GLIS3	protein_codir	-0.3712911	0.22729366	0.36945922
ENSG0000017	CASC2	lncRNA	-0.37157621	0.28688523	0.43641576
ENSG0000026	LINC01028	lncRNA	-0.37162758	0.74619451	0.83786862
ENSG0000011	CCDC86	protein_codir	-0.37168933	0.07787313	0.16731668
ENSG0000025	AB015752.3	lncRNA	-0.37186056	0.68027777	0.78979706
ENSG0000014	BHMT	protein_codir	-0.37188424	0.57825852	0.71044456
ENSG0000014	XPR1	protein_codir	-0.37193871	0.05884205	0.13587639
ENSG0000019	HRNR	protein_codir	-0.37204089	0.79813038	NA
ENSG0000027	RP11-265E18.	lncRNA	-0.37207585	0.79230728	0.87091683
ENSG0000025	RP11-128L5.1	lncRNA	-0.37254025	0.84909897	NA
ENSG0000026	GEMIN7-AS1	lncRNA	-0.37254397	0.13068561	0.24611596
ENSG0000017	ZNF596	protein_codir	-0.37266785	0.08075863	0.17193814
ENSG0000013	UBE2M	protein_codir	-0.37268991	0.01715517	0.05275283
ENSG0000022	RP11-474I16.8	lncRNA	-0.37285421	0.72212968	0.82068969
ENSG0000024	FOXO3B	protein_codir	-0.37295596	0.01920511	0.05751213
ENSG0000014	KIFC3	protein_codir	-0.37302485	0.09936479	0.2008236
ENSG0000008	RAB21	protein_codir	-0.37305325	0.07095956	0.15660432
ENSG0000020	RXRB	protein_codir	-0.37320861	0.00950084	0.03342416
ENSG0000026	AARSD1	protein_codir	-0.37321203	0.0273876	0.07576577
ENSG0000015	SSBP3	protein_codir	-0.37333417	0.08276278	0.17480369
ENSG0000013	ZFP37	protein_codir	-0.37339198	0.18761357	0.32135731
ENSG0000012	MTERF2	protein_codir	-0.37339305	0.15186629	0.27472798
ENSG0000018	ZNF662	protein_codir	-0.37345885	0.20338614	0.34066253
ENSG0000016	DVL3	protein_codir	-0.37348004	0.03177735	0.08503103
ENSG0000011	POLE4	protein_codir	-0.37349951	0.02340791	0.06698368
ENSG0000012	AHNAK	protein_codir	-0.37365341	0.21126361	0.35066712
ENSG0000016	ECI1	protein_codir	-0.37390922	0.00815449	0.02967028
ENSG0000026	CTD-2349P21	lncRNA	-0.37394736	0.3093002	0.4609546
ENSG0000013	LLGL1	protein_codir	-0.37429267	0.00104174	0.00591545
ENSG0000027	RP11-53O19.3	lncRNA	-0.37450826	0.07601818	0.16456008
ENSG0000016	PTH1R	protein_codir	-0.37451737	0.26050669	0.40777527
ENSG0000001	DYRK4	protein_codir	-0.37455549	0.0023836	0.01126586
ENSG0000012	DESI2	protein_codir	-0.37468258	0.07677155	0.16565861
ENSG0000001	PPP5C	protein_codir	-0.37523746	0.00013272	0.00110514
ENSG0000018	POU6F1	protein_codir	-0.37534123	0.07569421	0.16402589
ENSG0000016	NPY5R	protein_codir	-0.37547381	0.47277779	0.62131493
ENSG0000010	TIMM50	protein_codir	-0.37550496	0.01777629	0.05418021
ENSG0000025	STRA6LP	lncRNA	-0.37563315	0.2282539	0.37059219
ENSG0000013	ZSCAN9	protein_codir	-0.37564379	0.01510869	0.04796023
ENSG0000024	RP11-119K6.6	lncRNA	-0.37565781	0.79481771	0.87247504
ENSG0000013	RP11-178L8.4	protein_codir	-0.37588275	0.63961665	0.75836925
ENSG0000016	ZBTB8A	protein_codir	-0.37609088	0.05871648	0.13565446
ENSG0000012	RAB38	protein_codir	-0.3761829	0.28964336	0.43924393

ENSG0000015	LATS2	protein_codir	-0.37620737	0.27819445	0.42682322
ENSG0000022	LINC01823	lncRNA	-0.37634952	0.82268716	NA
ENSG0000025	CTD-2517M22	lncRNA	-0.37654816	0.82142527	NA
ENSG0000010	MTMR8	protein_codir	-0.37657138	0.16734355	0.29556394
ENSG0000011	RNF146	protein_codir	-0.37667607	0.03289209	0.08732022
ENSG0000013	VEZF1	protein_codir	-0.37673998	0.07083121	0.15642105
ENSG0000014	NCOR1	protein_codir	-0.37679041	0.00076926	0.00463806
ENSG0000027	RP11-278C7.5	lncRNA	-0.3768024	0.23842595	0.38266749
ENSG0000017	VWA3A	protein_codir	-0.37684284	0.6001148	0.72761111
ENSG0000014	POMGNT2	protein_codir	-0.37687723	0.18346567	0.31604149
ENSG0000011	C2orf42	protein_codir	-0.37698978	0.02799552	0.07718469
ENSG0000006	IPO5	protein_codir	-0.37703057	0.104323	0.20828083
ENSG0000025	HIF1A-AS3	lncRNA	-0.37728788	0.46555038	0.61525604
ENSG0000022	SLC25A5-AS1	lncRNA	-0.3772978	0.11005154	0.21677011
ENSG0000017	ZNF16	protein_codir	-0.37749263	0.02201459	0.06387005
ENSG0000024	EIF6	protein_codir	-0.37758042	0.01402994	0.04524837
ENSG0000018	GPR173	protein_codir	-0.37788251	0.15046239	0.27288536
ENSG0000017	DMRTA1	protein_codir	-0.37790649	0.42875887	0.58036363
ENSG0000016	ZYG11B	protein_codir	-0.37794992	0.10473651	0.20888312
ENSG0000013	BCAR3	protein_codir	-0.37800258	0.02563764	0.0720138
ENSG0000024	ZNF564	protein_codir	-0.3780457	0.1391227	0.25773486
ENSG0000017	TYMSOS	lncRNA	-0.37808534	0.34484495	0.49789376
ENSG0000022	AC105760.2	lncRNA	-0.37818674	0.04564993	0.11238533
ENSG0000014	ASB6	protein_codir	-0.37820349	0.00153562	0.00802078
ENSG0000013	VPS45	protein_codir	-0.37830142	0.01561788	0.04916412
ENSG0000010	MAPK1	protein_codir	-0.37831656	0.05641883	0.1316789
ENSG0000011	FBXO30	protein_codir	-0.37852624	0.07985993	0.17039253
ENSG0000017	FZD4	protein_codir	-0.37867324	0.21641072	0.3567451
ENSG0000018	PTTG1IP	protein_codir	-0.37875304	0.08130317	0.17280442
ENSG0000018	SIVA1	protein_codir	-0.37889199	0.00683945	0.02585267
ENSG0000019	HDAC2	protein_codir	-0.37910124	0.0158856	0.04985217
ENSG0000007	PLD1	protein_codir	-0.3791104	0.08942927	0.18539641
ENSG0000017	ZNF843	protein_codir	-0.37913106	0.11385672	0.22214769
ENSG0000013	DZIP1	protein_codir	-0.37928328	0.02968832	0.08086701
ENSG0000016	ZNF526	protein_codir	-0.37930061	0.0112047	0.03807034
ENSG0000010	TBC1D13	protein_codir	-0.37940191	0.00672775	0.02550026
ENSG0000018	CTD-3088G3.8	protein_codir	-0.37945152	0.25655581	0.40323747
ENSG0000013	DPF2	protein_codir	-0.37949344	0.0074838	0.02774252
ENSG0000018	ACBD4	protein_codir	-0.37951182	0.09020284	0.18649731
ENSG0000010	MEGF9	protein_codir	-0.37955303	0.11781824	0.22788094
ENSG0000010	RAB11FIP2	protein_codir	-0.37957901	0.05104447	0.12236143
ENSG0000022	AC106869.2	lncRNA	-0.37962057	0.90738052	NA
ENSG0000018	C2orf80	protein_codir	-0.37962057	0.90738052	NA
ENSG0000013	C4orf17	protein_codir	-0.37962057	0.90738052	NA
ENSG0000023	RP11-203H2.2	lncRNA	-0.37962057	0.90738052	NA
ENSG0000024	RP11-716O23	lncRNA	-0.37962057	0.90738052	NA
ENSG0000028	RP1-261K5.3	lncRNA	-0.37962057	0.90738052	NA

ENSG0000022	RP1-283K11.3	lncRNA	-0.37962057	0.90738052	NA
ENSG0000026	RP11-616M22	lncRNA	-0.37962057	0.90738052	NA
ENSG0000026	LLNLR-249E10	lncRNA	-0.37962057	0.90738052	NA
ENSG0000023	AF064860.7	lncRNA	-0.37962057	0.90738052	NA
ENSG0000022	RP5-1024N4.2	lncRNA	-0.37962057	0.90738052	NA
ENSG0000027	RP11-25O10.2	lncRNA	-0.37962057	0.90738052	NA
ENSG0000016	FBXW12	protein_codir	-0.37962057	0.90738052	NA
ENSG0000028	CTC-431G16.4	lncRNA	-0.37962057	0.90738052	NA
ENSG0000023	NCOA7-AS1	lncRNA	-0.37962057	0.90738052	NA
ENSG0000022	AC005281.2	lncRNA	-0.37962057	0.90738052	NA
ENSG0000028	CTB-114A6.1	lncRNA	-0.37962057	0.90738052	NA
ENSG0000025	CTD-3080F16.	lncRNA	-0.37962057	0.90738052	NA
ENSG0000028	KB-1303B4.4	lncRNA	-0.37962057	0.90738052	NA
ENSG0000026	RP11-732A21	lncRNA	-0.37962057	0.90738052	NA
ENSG0000028	RP11-542M13	lncRNA	-0.37962057	0.90738052	NA
ENSG0000026	RP11-296E23.	lncRNA	-0.37962057	0.90738052	NA
ENSG0000026	LIVAR	lncRNA	-0.37962057	0.90738052	NA
ENSG0000013	USP29	protein_codir	-0.37962057	0.90738052	NA
ENSG0000025	RNF185-AS1	lncRNA	-0.37962057	0.90738052	NA
ENSG0000024	CTD-2236F14.	lncRNA	-0.37962121	0.90448214	NA
ENSG0000027	RP11-44B19.1	lncRNA	-0.37962138	0.90367826	NA
ENSG0000026	KB-7G2.9	lncRNA	-0.3796215	0.90306915	NA
ENSG0000018	SRARP	protein_codir	-0.37962151	0.90303233	NA
ENSG0000023	AC133680.1	lncRNA	-0.37962168	0.90217016	NA
ENSG0000022	AC006042.6	lncRNA	-0.37962169	0.90210907	NA
ENSG0000018	DGAT2L6	protein_codir	-0.3796217	0.90204958	NA
ENSG0000025	RP11-10N23.5	lncRNA	-0.37962181	0.90151811	NA
ENSG0000028	RP11-306M1.	lncRNA	-0.37962193	0.90084789	NA
ENSG0000027	RP5-851M4.1	lncRNA	-0.37962194	0.9008042	NA
ENSG0000020	SPRR2E	protein_codir	-0.37962195	0.90076835	NA
ENSG0000026	CTD-3137H5.1	lncRNA	-0.37962196	0.90070756	NA
ENSG0000028	RP11-605B16.	lncRNA	-0.37962199	0.90057502	NA
ENSG0000018	NT5C1B	protein_codir	-0.37962202	0.90038428	NA
ENSG0000026	AC018755.17	lncRNA	-0.37962202	0.90038428	NA
ENSG0000022	UPK1A-AS1	lncRNA	-0.37962205	0.90020744	NA
ENSG0000013	BTG4	protein_codir	-0.3796221	0.89997236	NA
ENSG0000022	AC010745.2	lncRNA	-0.37962213	0.89980991	NA
ENSG0000018	PIWIL3	protein_codir	-0.37962213	0.89977405	NA
ENSG0000028	CTD-2285E13.	lncRNA	-0.37962215	0.89967676	NA
ENSG0000021	TSSK1B	protein_codir	-0.37962217	0.89959683	NA
ENSG0000027	RP11-338K13.	lncRNA	-0.37962221	0.89936179	NA
ENSG0000018	TBC1D28	protein_codir	-0.37962224	0.89919238	NA
ENSG0000027	CTD-2140B24	lncRNA	-0.37962224	0.8991756	NA
ENSG0000018	TMCO2	protein_codir	-0.37962226	0.89908414	NA
ENSG0000025	RP11-700H6.2	lncRNA	-0.37962227	0.89901283	NA
ENSG0000024	LINC02058	lncRNA	-0.37962229	0.89890494	NA
ENSG0000020	ARSH	protein_codir	-0.37962231	0.89879205	NA

ENSG0000023	AC114730.11	lncRNA	-0.37962233	0.89866768	NA
ENSG0000025	RP11-60I3.4	lncRNA	-0.37962235	0.8985871	NA
ENSG0000022	AC093585.6	lncRNA	-0.37962238	0.89840542	NA
ENSG0000025	LINC01629	lncRNA	-0.37962243	0.89811544	NA
ENSG0000024	LINC02120	lncRNA	-0.37962249	0.89777102	NA
ENSG0000026	L34079.3	lncRNA	-0.3796225	0.8977257	NA
ENSG0000028	RP11-47L11.1	lncRNA	-0.37962256	0.89737112	NA
ENSG0000016	FABP1	protein_codir	-0.37962266	0.89673335	NA
ENSG0000022	RP11-90J7.2	lncRNA	-0.37962268	0.89663755	NA
ENSG0000020	FBXO47	protein_codir	-0.37962272	0.8964258	NA
ENSG0000025	AC007126.1	lncRNA	-0.37962275	0.89619752	NA
ENSG0000028	RP3-466I7.2	lncRNA	-0.37962284	0.89564791	NA
ENSG0000022	AC096649.2	lncRNA	-0.37962291	0.89523005	NA
ENSG0000022	RP5-1177I5.3	lncRNA	-0.37962526	0.87647599	NA
ENSG0000028	RP11-452G18	lncRNA	-0.37962535	0.87549753	NA
ENSG0000011	HRG	protein_codir	-0.37962542	0.87479561	NA
ENSG0000028	RP11-527D7.3	lncRNA	-0.37962542	0.87475129	NA
ENSG0000025	LINC02373	lncRNA	-0.37962547	0.87420796	NA
ENSG0000017	MUCL1	protein_codir	-0.37962553	0.87359264	NA
ENSG0000026	RP11-166B2.7	lncRNA	-0.37962555	0.87341604	NA
ENSG0000023	RNF216-IT1	lncRNA	-0.37962556	0.87327706	NA
ENSG0000011	TECTB	protein_codir	-0.37962557	0.87309272	NA
ENSG0000023	C4A-AS1	lncRNA	-0.37962558	0.87301227	NA
ENSG0000022	C4B-AS1	lncRNA	-0.37962558	0.87301227	NA
ENSG0000026	RP11-134D3.2	lncRNA	-0.37962559	0.87293623	NA
ENSG0000028	RP11-12C17.3	lncRNA	-0.37962567	0.87204706	NA
ENSG0000026	RP11-515O17	lncRNA	-0.37962568	0.87187053	NA
ENSG0000023	LINC01648	lncRNA	-0.37962569	0.87178487	NA
ENSG0000026	CTB-180A7.3	lncRNA	-0.37962572	0.87145449	NA
ENSG0000028	RP11-57A19.7	lncRNA	-0.37962579	0.87057767	NA
ENSG0000023	LINC00330	lncRNA	-0.37962594	0.86879508	NA
ENSG0000027	RP11-631N16	lncRNA	-0.37965549	0.4102005	0.56319222
ENSG0000020	PCOTH	lncRNA	-0.37976597	0.2305001	0.37300286
ENSG0000027	NKILA	lncRNA	-0.37982928	0.03257175	0.086628
ENSG0000016	ZBTB7B	protein_codir	-0.38015588	0.01890806	0.05680851
ENSG0000017	LONRF3	protein_codir	-0.38030262	0.34071226	0.49380662
ENSG0000008	ZNF302	protein_codir	-0.38042447	0.05348429	0.12658407
ENSG0000010	KCTD17	protein_codir	-0.38042987	0.00037244	0.0025788
ENSG0000027	RP11-932O9.8	lncRNA	-0.38079056	0.57067236	0.70413091
ENSG0000013	PPP1R3D	protein_codir	-0.38102786	0.06812387	0.15207063
ENSG0000017	ACYP2	protein_codir	-0.38114579	0.01700114	0.05240752
ENSG0000027	SLC30A6-DT	lncRNA	-0.38133668	0.49380408	0.64010262
ENSG0000019	PGAP1	protein_codir	-0.38141609	0.20234044	0.3393844
ENSG0000028	AC006115.7	lncRNA	-0.38156238	0.43274221	0.58400957
ENSG0000025	CTD-3065J16.	lncRNA	-0.3816666	0.17815615	0.30906808
ENSG0000014	ARL6IP5	protein_codir	-0.3817441	0.01899872	0.05702507
ENSG0000026	RP11-116O18	lncRNA	-0.38176307	0.66413167	0.7776546

ENSG0000017	CDC42EP4	protein_codir	-0.38177831	0.22210604	0.36322866
ENSG0000025	LINC02259	lncRNA	-0.3819898	0.79114415	NA
ENSG0000027	RP11-54C4.3	lncRNA	-0.38204139	0.09662015	0.19656378
ENSG0000014	NBEAL1	protein_codir	-0.38211881	0.37239618	0.52584778
ENSG0000015	ATG10	protein_codir	-0.38268021	0.03615736	0.09381553
ENSG0000010	POLR2F	protein_codir	-0.38292436	0.00587756	0.02296155
ENSG0000016	STAT6	protein_codir	-0.38298483	0.00525009	0.02107088
ENSG0000023	AC004231.2	lncRNA	-0.38306701	0.86889725	NA
ENSG0000028	RP11-111H3.4	lncRNA	-0.38317315	0.86735516	NA
ENSG0000011	GORASP1	protein_codir	-0.38321905	0.00022488	0.0016989
ENSG0000024	RP11-99J16__	lncRNA	-0.38332857	0.65833559	0.77303074
ENSG0000013	RNFT2	protein_codir	-0.38345964	0.2859296	0.43547701
ENSG0000008	MAGI3	protein_codir	-0.3834694	0.04112931	0.10372457
ENSG0000023	RP3-413H6.2	lncRNA	-0.38370084	0.73657397	0.83108677
ENSG0000012	ASL	protein_codir	-0.38391516	0.00939015	0.03314877
ENSG0000026	RP11-343C2.1	protein_codir	-0.38393987	0.01718812	0.05281297
ENSG0000001	RWDD2A	protein_codir	-0.38401939	0.05851106	0.13528172
ENSG0000027	RP11-930O11	lncRNA	-0.38409126	0.55411358	0.69036118
ENSG0000023	TMEM250	protein_codir	-0.38416771	0.00140229	0.00747706
ENSG0000027	RP1-20N2.8	lncRNA	-0.3841979	0.5654193	0.6997124
ENSG0000015	MALSU1	protein_codir	-0.38427046	0.00408869	0.01734445
ENSG0000023	CD81-AS1	lncRNA	-0.38434161	0.41521046	0.56823543
ENSG0000013	CLUH	protein_codir	-0.38441509	0.0015775	0.00820171
ENSG0000012	ITFG1	protein_codir	-0.38447283	0.05117944	0.1225316
ENSG0000019	ANAPC7	protein_codir	-0.38461717	0.00344728	0.01508935
ENSG0000017	VANGL1	protein_codir	-0.3846434	0.17846776	0.30947261
ENSG0000014	SNX12	protein_codir	-0.38465218	0.00455182	0.0188516
ENSG0000012	ARHGAP22	protein_codir	-0.38467961	0.14948221	0.27170797
ENSG0000002	GLRX2	protein_codir	-0.38468634	0.02251848	0.06502462
ENSG0000022	MIS18A-AS1	lncRNA	-0.38479325	0.81480607	NA
ENSG0000015	AMN1	protein_codir	-0.38491468	0.0052998	0.02123609
ENSG0000010	GSDME	protein_codir	-0.38501816	0.1393135	0.25801911
ENSG0000008	MAPRE3	protein_codir	-0.38513972	0.0429928	0.10733627
ENSG0000021	C17orf67	protein_codir	-0.38521176	0.04294117	0.10725584
ENSG0000022	NECTIN4-AS1	lncRNA	-0.38523551	0.84074592	NA
ENSG0000026	CTD-2540B15	lncRNA	-0.38540358	0.78288658	0.86427976
ENSG0000028	RP1-193N13.1	lncRNA	-0.38557998	0.58303665	0.71418629
ENSG0000014	PRELID3A	protein_codir	-0.38566171	0.11177688	0.21928244
ENSG0000014	NPHP1	protein_codir	-0.38590213	0.1633549	0.29007001
ENSG0000007	OPHN1	protein_codir	-0.38594794	0.09239916	0.19009793
ENSG0000016	COQ7	protein_codir	-0.38609342	0.0763955	0.16514392
ENSG0000010	SLC25A14	protein_codir	-0.38619744	0.00804021	0.02934725
ENSG0000026	RP11-466A19	lncRNA	-0.3862575	0.77516607	0.85892045
ENSG0000023	MRPS17	protein_codir	-0.38627858	0.0046905	0.01932166
ENSG0000017	FSHR	protein_codir	-0.38633477	0.70838995	0.81057795
ENSG0000023	NALCN-AS1	lncRNA	-0.38635936	0.84301852	NA
ENSG0000000	UBE3C	protein_codir	-0.38637483	0.01877724	0.05650767

ENSG0000017	MICOS10	protein_codir	-0.38640714	0.00362451	0.01570582
ENSG0000026	CTD-2132N18	protein_codir	-0.38650552	0.86976747	0.92270768
ENSG0000019	ZNF534	protein_codir	-0.38654905	0.43267954	0.58395352
ENSG0000010	TTLL1	protein_codir	-0.38669698	0.00545794	0.02173103
ENSG0000014	SSC4D	protein_codir	-0.38700818	0.16990452	0.29863579
ENSG0000025	PPT2-EGFL8	protein_codir	-0.3870251	0.2627879	0.41015076
ENSG0000007	DNAJC10	protein_codir	-0.38712195	0.07746987	0.16672261
ENSG0000017	ZNF613	protein_codir	-0.38713414	0.04261725	0.10668798
ENSG0000018	CAPS2	protein_codir	-0.3871407	0.06556829	0.14795721
ENSG0000018	IFITM2	protein_codir	-0.38720041	0.02038578	0.06015802
ENSG0000017	KAT5	protein_codir	-0.38721123	0.00838859	0.03028664
ENSG0000022	NCKAP5-AS2	lncRNA	-0.38722267	0.53838386	0.67798267
ENSG0000016	VPS72	protein_codir	-0.38724928	0.00108113	0.00609154
ENSG0000027	RP3-368A4.6	lncRNA	-0.38772011	0.34003477	0.4930058
ENSG0000010	ZNF106	protein_codir	-0.38782786	0.08991191	0.18606182
ENSG0000016	AKIP1	protein_codir	-0.38800474	0.00209684	0.01018551
ENSG0000020	DPF3	protein_codir	-0.38802919	0.27248039	0.42039483
ENSG0000018	SPATA41	lncRNA	-0.38805686	0.23929761	0.38359837
ENSG0000025	CTD-2555K7.2	lncRNA	-0.38818944	0.37195478	0.52533193
ENSG0000016	TMEM99	lncRNA	-0.38836494	0.0250138	0.07063502
ENSG0000013	ZNF428	protein_codir	-0.38840101	0.01700874	0.05240756
ENSG0000016	SLC16A14	protein_codir	-0.38843062	0.24281656	0.38777644
ENSG0000000	MTMR7	protein_codir	-0.38854747	0.51426875	0.65753648
ENSG0000026	TMEM256-PLS	protein_codir	-0.38883522	0.56988059	0.70346825
ENSG0000010	FBXO31	protein_codir	-0.38893702	0.05849358	0.13527527
ENSG0000020	ASAH2B	protein_codir	-0.38903675	0.05699514	0.13266034
ENSG0000015	GPD1L	protein_codir	-0.38936833	0.24376178	0.38883629
ENSG0000008	HSP90AA1	protein_codir	-0.38937892	0.06885666	0.15308871
ENSG0000011	GPN3	protein_codir	-0.38950725	0.01959146	0.05836025
ENSG0000016	MRPL58	protein_codir	-0.38982089	0.00323607	0.01436287
ENSG0000015	ATPSCKMT	protein_codir	-0.39000087	0.02071521	0.06094526
ENSG0000010	ZMAT5	protein_codir	-0.39023186	0.00573473	0.02254065
ENSG0000018	PDIA2	protein_codir	-0.39027272	0.41853159	0.57122314
ENSG0000017	NDUFA11	protein_codir	-0.39039763	0.00243339	0.01144785
ENSG0000022	PLCG1-AS1	lncRNA	-0.39070071	0.17871745	0.30973057
ENSG0000018	CMSS1	protein_codir	-0.39076412	0.01744168	0.05339607
ENSG0000010	RBFA	protein_codir	-0.390904	0.00688004	0.02597411
ENSG0000018	PRSS45P	protein_codir	-0.39095934	0.7441924	0.83657667
ENSG0000025	LINC01833	lncRNA	-0.39099019	0.86630082	0.92056128
ENSG0000011	PRKAR2A	protein_codir	-0.39101325	0.03886415	0.09924442
ENSG0000027	RP11-34F20.7	lncRNA	-0.39104215	0.34250364	0.49575257
ENSG0000004	ATP6V1H	protein_codir	-0.39112191	0.00210816	0.01023676
ENSG0000028	RP11-523H20	lncRNA	-0.39113279	0.24255166	0.38746541
ENSG0000025	RP1-39G22.7	lncRNA	-0.39115467	0.14106565	0.26040858
ENSG0000016	CRK	protein_codir	-0.39117096	0.01761906	0.05379598
ENSG0000027	TSTD3	protein_codir	-0.39127006	0.08746033	0.18231353
ENSG0000027	RP11-707G18	lncRNA	-0.39133742	0.37338504	0.52699274

ENSG0000011	RERGL	protein_codir	-0.3914621	0.38649015	0.5395318
ENSG0000028	RP11-155D18	lncRNA	-0.39151925	0.60118088	0.72828012
ENSG0000018	FSCN2	protein_codir	-0.39170373	0.29666782	0.4471468
ENSG0000027	RP11-56D16.8	lncRNA	-0.39192768	0.45230754	0.602873
ENSG0000017	SNX32	protein_codir	-0.39194465	0.54445757	0.68264188
ENSG0000025	AP002884.2	protein_codir	-0.39194911	0.6398954	0.75856951
ENSG0000021	IFRD2	protein_codir	-0.39201088	0.03946854	0.10046535
ENSG0000022	RP11-258F22.	lncRNA	-0.39210625	0.57924386	0.71121207
ENSG0000004	RETSAT	protein_codir	-0.39233728	0.01690483	0.05219221
ENSG0000000	PAFAH1B1	protein_codir	-0.39234435	0.0107695	0.03694219
ENSG0000012	PANK2	protein_codir	-0.39243724	8.45E-06	0.00011204
ENSG0000018	CLDN6	protein_codir	-0.39254278	0.81137486	NA
ENSG0000013	SDSL	protein_codir	-0.39297482	0.31836953	0.47040959
ENSG0000020	ITPRIPL2	protein_codir	-0.39299825	0.11442432	0.22288749
ENSG0000006	PPP2R5A	protein_codir	-0.3931239	0.00466491	0.01924731
ENSG0000020	NYNRIN	protein_codir	-0.39314403	0.12095267	0.23249445
ENSG0000013	AAR2	protein_codir	-0.39316009	0.00064886	0.00404609
ENSG0000013	SLC2A8	protein_codir	-0.39318699	0.01516826	0.0481051
ENSG0000014	VGLL4	protein_codir	-0.39332991	0.03131205	0.08407923
ENSG0000018	ZNF48	protein_codir	-0.3935009	0.01808568	0.05488366
ENSG0000008	PSMC5	protein_codir	-0.39350381	0.00039374	0.00270153
ENSG0000011	CCN2	protein_codir	-0.39351552	0.34744178	0.50064388
ENSG0000024	XXbac-BPG11	protein_codir	-0.39360309	0.83113655	0.89822487
ENSG0000008	HACE1	protein_codir	-0.39388304	0.01678646	0.0519254
ENSG0000017	BOLA1	protein_codir	-0.39395035	0.04463148	0.11045118
ENSG0000013	POLR3F	protein_codir	-0.39401832	0.00554593	0.02197055
ENSG0000018	LAMP1	protein_codir	-0.39408548	0.01530088	0.04842562
ENSG0000017	CSTF2T	protein_codir	-0.39414334	0.0476013	0.11590474
ENSG0000005	PLEKHH1	protein_codir	-0.39431481	0.30076735	0.45185003
ENSG0000020	CTB-158D10.3	lncRNA	-0.39444301	0.1436624	0.26380928
ENSG0000020	HSPA1L	protein_codir	-0.39445861	0.03549837	0.09255781
ENSG0000010	USP46	protein_codir	-0.39453804	0.05715632	0.1329172
ENSG0000005	TRAPPC3	protein_codir	-0.39457683	0.00552344	0.02190343
ENSG0000017	C3orf33	protein_codir	-0.39460504	0.04896754	0.1185557
ENSG0000013	SCO1	protein_codir	-0.39469663	0.01809832	0.05489973
ENSG0000024	RP11-34F13.2	lncRNA	-0.3947985	0.76492639	0.85183723
ENSG0000012	RAB3IP	protein_codir	-0.39510399	0.06385223	0.14500893
ENSG0000018	ZBTB40	protein_codir	-0.39512273	0.08208523	0.17391792
ENSG0000018	POTEE	protein_codir	-0.39518112	0.18833091	0.32220638
ENSG0000023	RP5-1184F4.5	lncRNA	-0.39518799	0.78733035	NA
ENSG0000011	BTN2A1	protein_codir	-0.39534443	0.00779774	0.02863248
ENSG0000020	KIAA1671-AS1	lncRNA	-0.39543646	0.77118877	0.85608432
ENSG0000016	IRGQ	protein_codir	-0.39561603	1.01E-05	0.00012975
ENSG0000020	GGTA1	protein_codir	-0.39569204	0.10311238	0.20647893
ENSG0000010	TAX1BP1	protein_codir	-0.39574105	0.02482241	0.07020685
ENSG0000027	RP11-996F15.	lncRNA	-0.3958261	0.23947295	0.38381262
ENSG0000013	DPP6	protein_codir	-0.39600993	NA	NA

ENSG0000007	ASIC4	protein_codir	-0.3963775	0.45370309	0.60420026
ENSG0000017	EXOSC4	protein_codir	-0.3965321	0.01032021	0.03568406
ENSG0000027	RP11-314N13	protein_codir	-0.39654997	0.29285547	0.44297196
ENSG0000013	CD63	protein_codir	-0.39660694	0.02215089	0.06418022
ENSG0000011	CHD4	protein_codir	-0.39661049	0.00022764	0.00171412
ENSG0000016	MED27	protein_codir	-0.39666556	0.02078381	0.06109502
ENSG0000016	PEX19	protein_codir	-0.39667837	0.03429792	0.09025423
ENSG0000002	PLEKHB1	protein_codir	-0.39705279	0.24046187	0.38488392
ENSG0000017	EID2B	protein_codir	-0.39705494	0.02617823	0.07317507
ENSG0000019	ASB12	protein_codir	-0.39744075	0.50833512	0.65272971
ENSG0000016	ZER1	protein_codir	-0.39745506	0.00903086	0.03215577
ENSG0000026	LINC01963	lncRNA	-0.3974837	0.14658742	0.26805776
ENSG0000019	IPP	protein_codir	-0.39763568	0.08179849	0.17356337
ENSG0000014	SF3B4	protein_codir	-0.39765946	0.00373681	0.01608895
ENSG0000008	WDR47	protein_codir	-0.39767292	0.04031154	0.10211911
ENSG0000017	DNAJC24	protein_codir	-0.39769492	0.00173938	0.00883119
ENSG0000017	SAMD4B	protein_codir	-0.39779401	0.02494333	0.07047927
ENSG0000001	HEBP1	protein_codir	-0.39779693	0.01089916	0.03727985
ENSG0000018	TSPYL5	protein_codir	-0.39786734	0.01619278	0.05058756
ENSG0000017	TIMM22	protein_codir	-0.39804362	0.00091658	0.00534203
ENSG0000026	CMB9-55A18.	lncRNA	-0.39826355	0.80891582	NA
ENSG0000016	CCT3	protein_codir	-0.39835502	0.01040609	0.03594501
ENSG0000022	CTD-2330K9.3	lncRNA	-0.39847447	0.71723127	0.81727495
ENSG0000018	COA5	protein_codir	-0.39853001	0.01865737	0.05622659
ENSG0000016	VEGFD	protein_codir	-0.39858868	0.53388718	0.67450592
ENSG0000017	ZNRD2	protein_codir	-0.39859379	0.00748405	0.02774252
ENSG0000019	SIGLEC15	protein_codir	-0.39868354	0.44914469	0.60002634
ENSG0000008	OSTM1	protein_codir	-0.3989365	0.03622128	0.09396373
ENSG0000023	SHISA9	protein_codir	-0.39900978	0.63005163	0.75083168
ENSG0000000	POLDIP2	protein_codir	-0.39901286	0.00423298	0.01780364
ENSG0000027	CTB-96E2.10	lncRNA	-0.39934209	0.60925855	0.73473873
ENSG0000027	PPP1R21-DT	lncRNA	-0.3994304	0.67348064	0.78467625
ENSG0000013	GSTM3	protein_codir	-0.39950275	0.32204107	0.47421092
ENSG0000024	LINC01011	lncRNA	-0.39989038	0.0874213	0.18227345
ENSG0000014	ANAPC11	protein_codir	-0.39991133	0.00235468	0.01116311
ENSG0000014	TCF7L2	protein_codir	-0.40025437	0.08009973	0.17080486
ENSG0000025	LINC02356	lncRNA	-0.40033472	0.68502663	0.79304365
ENSG0000006	FOXJ2	protein_codir	-0.40043298	0.03474434	0.09105412
ENSG0000027	RP11-355F16.	lncRNA	-0.40043892	0.45637684	0.60697893
ENSG0000013	SMYD5	protein_codir	-0.40045962	0.00282831	0.01287373
ENSG0000013	KRT7	protein_codir	-0.40047651	0.49276033	0.63923913
ENSG0000004	KITLG	protein_codir	-0.40048268	0.1977089	0.3339277
ENSG0000011	CSNK1A1	protein_codir	-0.40052054	0.01931351	0.05778155
ENSG0000010	NAT9	protein_codir	-0.40059201	0.02209422	0.06404055
ENSG0000007	PRKACA	protein_codir	-0.40059694	0.02406626	0.06847034
ENSG0000028	AC009133.23	protein_codir	-0.40086595	0.1033007	0.20675112
ENSG0000027	RP11-503P10.	lncRNA	-0.40093782	0.55024784	0.68734319

ENSG0000011	ZNF684	protein_codir	-0.40098378	0.10194939	0.20469921
ENSG0000016	CMPK1	protein_codir	-0.40101447	0.02222721	0.0643315
ENSG0000008	CD59	protein_codir	-0.40101987	0.0568732	0.13245955
ENSG0000019	SMURF1	protein_codir	-0.40132572	0.04118816	0.10383136
ENSG0000024	RP11-298J20.	lncRNA	-0.40141429	0.25630484	0.40300238
ENSG0000010	RBBP7	protein_codir	-0.40165611	0.01928742	0.0577326
ENSG0000014	TMEM181	protein_codir	-0.40170802	0.00501861	0.0203276
ENSG0000013	SNX9	protein_codir	-0.40214689	0.0929046	0.19088188
ENSG0000027	LINC02712	lncRNA	-0.40239401	0.65972388	0.77403566
ENSG0000017	THAP6	protein_codir	-0.40248072	0.00278215	0.01270972
ENSG0000011	PEX7	protein_codir	-0.40249308	0.07110178	0.15681701
ENSG0000010	HTATSF1	protein_codir	-0.40249372	0.03812226	0.09771128
ENSG0000009	ACOT7	protein_codir	-0.40269955	0.02648515	0.07378654
ENSG0000017	SHARPIN	protein_codir	-0.40270671	0.00522325	0.02099637
ENSG0000011	CTNNAL1	protein_codir	-0.40301054	0.2236315	0.36509647
ENSG0000017	PNMA1	protein_codir	-0.40335911	0.0889526	0.1847012
ENSG0000017	RNF26	protein_codir	-0.40356756	0.00349623	0.01526975
ENSG0000014	MYLK3	protein_codir	-0.40370718	0.22999848	0.37238557
ENSG0000005	DCBLD2	protein_codir	-0.4037223	0.09784854	0.19851636
ENSG0000017	MSC	protein_codir	-0.40376101	0.21136703	0.35079072
ENSG0000024	LINC02614	lncRNA	-0.40384623	0.25202218	0.39813611
ENSG0000028	CTD-2510L9.2	lncRNA	-0.40392721	0.31215929	0.46393886
ENSG0000016	SUGT1	protein_codir	-0.40401836	0.00181562	0.00911274
ENSG0000018	NBR1	protein_codir	-0.40424899	0.05389365	0.1273247
ENSG0000024	LNCSRLR	lncRNA	-0.40425036	0.51374059	0.6570741
ENSG0000017	GK5	protein_codir	-0.40433137	0.09477371	0.19378516
ENSG0000013	TIMM10	protein_codir	-0.40464631	0.0195704	0.05831638
ENSG0000017	SNAPC5	protein_codir	-0.40477603	0.0008897	0.00521951
ENSG0000011	SMAD5	protein_codir	-0.40479266	0.04855298	0.11777915
ENSG0000020	COX20	protein_codir	-0.40483666	0.04933623	0.11930831
ENSG0000013	MMACHC	protein_codir	-0.40493134	0.11941437	0.23010874
ENSG0000026	RP4-545K15.5	lncRNA	-0.40497984	0.25697619	0.40371015
ENSG0000009	SUSD2	protein_codir	-0.40500471	0.06334782	0.14404106
ENSG0000014	RABL3	protein_codir	-0.4051289	0.00648877	0.02486057
ENSG0000011	ZC2HC1C	protein_codir	-0.40518161	0.07310052	0.16002507
ENSG0000013	SFTPD	protein_codir	-0.40526837	0.5209331	0.66346262
ENSG0000017	MAMDC4	protein_codir	-0.40540028	0.30865843	0.4602666
ENSG0000026	RP13-20L14.1	lncRNA	-0.40554959	0.78129566	NA
ENSG0000011	SLC35D1	protein_codir	-0.40557882	0.01866891	0.05624907
ENSG0000025	URB1-AS1	lncRNA	-0.40565621	0.02419233	0.06874406
ENSG0000027	RP11-571M6.	lncRNA	-0.40587774	0.34272309	0.49586676
ENSG0000010	MTMR9	protein_codir	-0.40615307	0.00203502	0.00995348
ENSG0000023	TMEM44-AS1	lncRNA	-0.40628079	0.10712083	0.21239608
ENSG0000020	PFDN6	protein_codir	-0.40629792	0.00142959	0.00759359
ENSG0000028	ABCF2-H2BE1	protein_codir	-0.4063534	0.02082762	0.06117822
ENSG0000010	ZC2HC1A	protein_codir	-0.40637521	0.04728139	0.1153225
ENSG0000023	GNAS-AS1	lncRNA	-0.40641891	0.45762194	0.60813693

ENSG0000024	AC002467.7	lncRNA	-0.40647605	0.1139464	0.22227556
ENSG0000026	RP11-1007O2	lncRNA	-0.40669335	0.50499482	0.64958816
ENSG0000017	MRPL13	protein_codir	-0.40670148	0.00482492	0.019737
ENSG0000008	CPNE3	protein_codir	-0.40673092	0.02091403	0.06139286
ENSG0000027	RP11-42O15.3	lncRNA	-0.40674179	0.64096788	0.75922175
ENSG0000001	CD9	protein_codir	-0.40681114	0.17423329	0.30419239
ENSG0000008	CFAP61	protein_codir	-0.40685519	0.54832113	0.68592697
ENSG0000016	OTUD7A	protein_codir	-0.40688618	0.06667939	0.14985394
ENSG0000013	DCTN3	protein_codir	-0.40693639	0.00064846	0.00404609
ENSG0000008	OGFOD1	protein_codir	-0.40702279	0.00872426	0.03126001
ENSG0000002	HSF2	protein_codir	-0.40705733	0.00953188	0.03349499
ENSG0000014	TTC12	protein_codir	-0.40709505	0.07559629	0.16387799
ENSG0000000	ELAC2	protein_codir	-0.40741127	0.00035448	0.00247432
ENSG0000010	PDLIM1	protein_codir	-0.40757636	0.16764202	0.29593969
ENSG0000010	STK3	protein_codir	-0.40770255	0.08964556	0.18566364
ENSG0000011	BYSL	protein_codir	-0.40770515	0.14346993	0.26356098
ENSG0000000	AASS	protein_codir	-0.40779635	0.14979357	0.27206539
ENSG0000026	RP11-452L6.5	lncRNA	-0.40786924	0.01326032	0.04329709
ENSG0000017	B3GNT8	protein_codir	-0.40792943	0.14019517	0.259339
ENSG0000019	RPE	protein_codir	-0.40810892	0.00299186	0.01347605
ENSG0000012	UROD	protein_codir	-0.40835623	0.00060958	0.00384167
ENSG0000011	ALDH6A1	protein_codir	-0.40843861	0.05569486	0.13041899
ENSG0000016	FMC1	protein_codir	-0.40858384	0.1120787	0.21971849
ENSG0000016	SLITRK5	protein_codir	-0.40892752	0.41984399	0.5723232
ENSG0000018	CREB3L2	protein_codir	-0.40897193	0.07012913	0.15529921
ENSG0000014	PRUNE1	protein_codir	-0.40917105	0.01184551	0.0397015
ENSG0000028	RP11-294J22.	lncRNA	-0.40955341	0.78282569	0.86427811
ENSG0000012	KLHL7	protein_codir	-0.40969745	0.02154967	0.06279825
ENSG0000004	THAP3	protein_codir	-0.4097512	0.02973816	0.08096285
ENSG0000017	PLRG1	protein_codir	-0.40987322	0.00025733	0.00189986
ENSG0000024	RDH14	protein_codir	-0.41043349	0.01025044	0.03550948
ENSG0000005	PHPT1	protein_codir	-0.4106237	0.01034465	0.03575515
ENSG0000019	TEX45	protein_codir	-0.41068774	0.62179693	0.74470339
ENSG0000026	RP11-250B2.5	lncRNA	-0.41070337	0.48832662	0.63539302
ENSG0000014	ATP10D	protein_codir	-0.41079846	0.09934242	0.20080942
ENSG0000013	TIMM17A	protein_codir	-0.41092903	0.01331304	0.0434179
ENSG0000024	TTC4	protein_codir	-0.41095715	3.68E-06	5.49E-05
ENSG0000012	TMEM53	protein_codir	-0.41136586	1.54E-05	0.00018417
ENSG0000011	KIAA2013	protein_codir	-0.41136913	0.00019652	0.00152271
ENSG0000011	MECR	protein_codir	-0.41141529	0.0164974	0.05124938
ENSG0000011	PTGES3	protein_codir	-0.41143709	0.00272003	0.01248999
ENSG0000018	ZNF623	protein_codir	-0.41151461	0.05078561	0.12197152
ENSG0000018	BCR	protein_codir	-0.41186156	0.01631318	0.05080924
ENSG0000000	COPZ2	protein_codir	-0.41187147	0.14741044	0.26899289
ENSG0000010	FBXL15	protein_codir	-0.4118841	0.00619777	0.02394493
ENSG0000010	PBLD	protein_codir	-0.41228889	0.17666397	0.30709631
ENSG0000020	TRIM13	protein_codir	-0.4123686	0.00338185	0.01486772

ENSG0000018	COA4	protein_codir	-0.41243678	0.00020313	0.00156456
ENSG0000010	LAPTM4B	protein_codir	-0.41311815	0.13441849	0.25132945
ENSG0000017	AP3S1	protein_codir	-0.41313133	0.03952226	0.10057165
ENSG0000012	ANAPC13	protein_codir	-0.41344337	0.00017891	0.0014131
ENSG0000016	CFAP36	protein_codir	-0.41345927	0.02947015	0.08037577
ENSG0000012	WRNIP1	protein_codir	-0.41372383	0.00360447	0.0156337
ENSG0000018	HEATR4	protein_codir	-0.413846	0.53618063	0.67636184
ENSG0000019	CIPC	protein_codir	-0.41397821	0.03882283	0.09915726
ENSG0000016	RFTN2	protein_codir	-0.41405178	0.0961997	0.19591462
ENSG0000012	EPHX2	protein_codir	-0.41422479	0.18563655	0.3189203
ENSG0000025	SLIT3-AS2	lncRNA	-0.41441851	0.66365251	0.77732421
ENSG0000007	MPP5	protein_codir	-0.41447785	0.02365355	0.06752568
ENSG0000005	MSMO1	protein_codir	-0.41451136	0.13596835	0.25322558
ENSG0000026	CTD-3149D2.4	lncRNA	-0.41452574	0.82763263	NA
ENSG0000024	IFITM10	protein_codir	-0.41461968	0.40028241	0.55352103
ENSG0000014	MRPL24	protein_codir	-0.41464702	0.00207349	0.01010482
ENSG0000012	PLXDC2	protein_codir	-0.41475976	0.25004647	0.39595709
ENSG0000019	ARMCX6	protein_codir	-0.41477937	0.0041958	0.01770092
ENSG0000023	RP11-390F4.6	lncRNA	-0.41485731	0.77162142	NA
ENSG0000018	EIF3C	protein_codir	-0.41504925	0.08164307	0.17332679
ENSG0000026	RP11-77K12.1	protein_codir	-0.41511771	0.53573929	0.67594532
ENSG0000012	MPST	protein_codir	-0.41512169	0.02726603	0.07550509
ENSG0000026	NOTCH2NLA	protein_codir	-0.41515712	0.18252887	0.31488945
ENSG0000015	MED7	protein_codir	-0.41516094	0.00037786	0.00261043
ENSG0000010	C17orf75	protein_codir	-0.41516419	0.04027619	0.10204956
ENSG0000014	ANKRD53	protein_codir	-0.41578602	0.11506001	0.22387864
ENSG0000025	RP11-582J16.4	lncRNA	-0.41581015	0.27632943	0.42455465
ENSG0000027	HERPUD2-AS1	lncRNA	-0.41603513	0.00538988	0.02150968
ENSG0000018	NDUFA4L2	protein_codir	-0.41620367	0.32598507	0.47823379
ENSG0000011	HCFC2	protein_codir	-0.41631552	0.05160966	0.12324016
ENSG0000023	GLIS3-AS1	lncRNA	-0.41656915	0.82640947	NA
ENSG0000024	RP11-727A23	lncRNA	-0.41661874	0.24486646	0.38991489
ENSG0000013	FAM8A1	protein_codir	-0.41672473	0.09358126	0.19197226
ENSG0000016	MTHFD2L	protein_codir	-0.4167658	0.02215788	0.06418318
ENSG0000025	RP11-110G21	lncRNA	-0.41687714	0.73107174	NA
ENSG0000010	TSPO	protein_codir	-0.4168957	0.02674729	0.07437432
ENSG0000016	GPER1	protein_codir	-0.41702981	0.2234522	0.36497633
ENSG0000013	ACTR1A	protein_codir	-0.41709352	0.01245105	0.041215
ENSG0000009	GLG1	protein_codir	-0.41713637	0.00643531	0.02469341
ENSG0000007	SYNJ2	protein_codir	-0.41713709	0.04833582	0.11733481
ENSG0000018	NPLOC4	protein_codir	-0.41715949	0.00950586	0.03343758
ENSG0000013	TBX3	protein_codir	-0.41717826	0.07335985	0.16038938
ENSG0000019	VKORC1L1	protein_codir	-0.41721266	0.04656809	0.11401499
ENSG0000011	SLIRP	protein_codir	-0.41721642	0.02528817	0.07124229
ENSG0000003	GAB2	protein_codir	-0.41725336	0.08159271	0.17325595
ENSG0000024	EXTL3-AS1	lncRNA	-0.41737758	0.35115771	0.50472257
ENSG0000016	PRTG	protein_codir	-0.41741003	0.22924275	0.37173045

ENSG0000012	GORAB	protein_codir	-0.41759947	0.03790566	0.09727007
ENSG0000016	ENOX2	protein_codir	-0.41773447	0.07911169	0.16929748
ENSG0000016	ASMTL	protein_codir	-0.41778741	0.01232278	0.0408998
ENSG0000022	WEE2-AS1	lncRNA	-0.41790485	0.14768859	0.26934029
ENSG0000027	C1QTNF3-AM	protein_codir	-0.41792991	0.55575102	0.69168363
ENSG0000009	CABIN1	protein_codir	-0.4179451	0.0001289	0.00107949
ENSG0000021	RP11-701H24	protein_codir	-0.4180517	0.45149175	0.60210526
ENSG0000012	BCAS4	protein_codir	-0.41820111	0.262841	0.41015076
ENSG0000028	RP11-269P11.	lncRNA	-0.41822855	0.55054692	0.68754254
ENSG0000016	ANKS3	protein_codir	-0.41854068	0.06215593	0.14187991
ENSG0000018	WDFY3-AS2	lncRNA	-0.41872219	0.24016723	0.38467983
ENSG0000019	TAF13	protein_codir	-0.41876518	0.08343678	0.17593127
ENSG0000014	C11orf49	protein_codir	-0.41883242	0.00015488	0.00125743
ENSG0000019	ZNF781	protein_codir	-0.41896212	0.0911627	0.18800222
ENSG0000015	ANKRD61	protein_codir	-0.41898144	0.57110638	0.70444615
ENSG0000017	DES	protein_codir	-0.41921776	0.56294995	0.69756084
ENSG0000027	RP11-2H3.6	lncRNA	-0.41932182	0.62229552	0.74518978
ENSG0000019	ILRUN	protein_codir	-0.41947217	0.00250821	0.01172392
ENSG0000022	XKR9	protein_codir	-0.41953882	0.41674392	0.56949616
ENSG0000007	FAM50A	protein_codir	-0.41998163	0.01789589	0.05441864
ENSG0000016	ZNF35	protein_codir	-0.42004741	0.02876676	0.07890149
ENSG0000017	C14orf28	protein_codir	-0.42011479	0.05667505	0.13215406
ENSG0000026	ARHGEF18-AS	lncRNA	-0.42012563	0.798528	NA
ENSG0000028	RP11-807H22	protein_codir	-0.42021458	0.36671891	0.52059911
ENSG0000018	EIF4ENIF1	protein_codir	-0.42039281	0.01606168	0.05029177
ENSG0000028	RP5-1140G11	lncRNA	-0.42056969	0.48416325	0.63149741
ENSG0000002	KPNA6	protein_codir	-0.42073745	9.00E-05	0.00079666
ENSG0000026	OTULIN-DT	lncRNA	-0.42082545	0.49514806	0.64116218
ENSG0000022	RP13-216E22.	lncRNA	-0.42092947	0.39095727	0.54384993
ENSG0000026	LINC01987	lncRNA	-0.42093877	0.79536794	NA
ENSG0000027	RP11-761I4.4	lncRNA	-0.42096901	0.79593045	NA
ENSG0000012	SCAF1	protein_codir	-0.42109828	0.00286461	0.01300254
ENSG0000015	ANKRD9	protein_codir	-0.42120902	0.08777789	0.18279242
ENSG0000006	RRP15	protein_codir	-0.42137289	0.02583537	0.07241476
ENSG0000017	DNAJC28	protein_codir	-0.42138719	0.19168391	0.3263258
ENSG0000017	ZNF491	protein_codir	-0.42152727	0.12933662	0.2441915
ENSG0000028	RP5-940J5.10	protein_codir	-0.4217813	0.14447275	0.26498024
ENSG0000018	H1-2	protein_codir	-0.42193985	0.29139077	0.44121033
ENSG0000025	C6orf3	lncRNA	-0.42206975	0.08010265	0.17080486
ENSG0000006	TFE3	protein_codir	-0.42211279	0.00672921	0.02550232
ENSG0000025	LINC01485	lncRNA	-0.42222833	0.60640393	0.73267177
ENSG0000016	LMO1	protein_codir	-0.42229916	0.73798801	0.83227426
ENSG0000011	TUSC2	protein_codir	-0.42232992	3.76E-05	0.0003859
ENSG0000018	ZFP41	protein_codir	-0.42236247	0.00216351	0.01044324
ENSG0000014	SPATA25	protein_codir	-0.42280916	0.34260736	0.49585073
ENSG0000015	ANKRD40	protein_codir	-0.42313024	0.10305437	0.20637774
ENSG0000011	DYNC2I2	protein_codir	-0.42331963	0.01663732	0.05157163

ENSG0000016	SPRY3	protein_codir	-0.4233442	0.01838993	0.05560261
ENSG0000012	KTN1	protein_codir	-0.42339355	0.03723354	0.09602151
ENSG0000014	NDUFB10	protein_codir	-0.42349511	0.00380624	0.01634968
ENSG0000012	FXR2	protein_codir	-0.42358821	0.01352046	0.04397494
ENSG0000016	ANKDD1A	protein_codir	-0.42370151	0.15863745	0.28390892
ENSG0000024	USP51	protein_codir	-0.42384063	0.08368974	0.17635695
ENSG0000013	YWHAQ	protein_codir	-0.42401065	0.0298255	0.08109671
ENSG0000024	CCDC153	protein_codir	-0.42405059	0.24024757	0.38471927
ENSG0000023	PET117	protein_codir	-0.42418385	0.02047529	0.06034869
ENSG0000023	AC079922.3	lncRNA	-0.42419765	0.11078819	0.21785347
ENSG0000027	RP11-182J1.3	lncRNA	-0.42435207	0.06806706	0.15198158
ENSG0000017	GPR156	protein_codir	-0.42460376	0.34518231	0.49803768
ENSG0000018	HOXB4	protein_codir	-0.42468485	0.09527035	0.19451755
ENSG0000022	RP11-775D22	lncRNA	-0.42480541	0.53430881	0.6748993
ENSG0000016	AQP7	protein_codir	-0.42482876	0.4877782	0.6348337
ENSG0000008	KIF16B	protein_codir	-0.42486359	0.01999441	0.05929395
ENSG0000022	LINC00205	lncRNA	-0.42544159	0.00021067	0.00161136
ENSG0000013	ITGAV	protein_codir	-0.42563673	0.20188583	0.33895122
ENSG0000011	BCL9	protein_codir	-0.42589781	0.05977013	0.13757719
ENSG0000018	SKA2	protein_codir	-0.42592582	0.01382613	0.0447216
ENSG0000015	MED21	protein_codir	-0.42597471	0.01382478	0.0447216
ENSG0000028	RP11-352J13.	lncRNA	-0.42603967	0.7769814	NA
ENSG0000017	ZNF408	protein_codir	-0.42610241	0.00112533	0.00627783
ENSG0000022	VAMP2	protein_codir	-0.42614193	0.00038327	0.00263985
ENSG0000025	AP000640.10	lncRNA	-0.42621925	0.35157365	0.50512195
ENSG0000026	LINC02846	lncRNA	-0.42625306	0.23915042	0.38342919
ENSG0000010	DNAH11	protein_codir	-0.42626375	0.51941115	0.66218029
ENSG0000013	USP30	protein_codir	-0.42643671	0.00279998	0.01277428
ENSG0000027	RP1-191J18.6	lncRNA	-0.42671452	0.21659631	0.35690192
ENSG0000013	NOTCH2	protein_codir	-0.42677109	0.12698154	0.24098337
ENSG0000017	RNASEH1	protein_codir	-0.42693124	0.03592419	0.09335962
ENSG0000011	TREH	protein_codir	-0.42700223	0.3811393	0.53446003
ENSG0000013	PXDN	protein_codir	-0.42709482	0.18330784	0.31588236
ENSG0000010	POLR2I	protein_codir	-0.42714171	0.00461213	0.01907277
ENSG0000018	TMEM121	protein_codir	-0.42728204	0.04814409	0.11698596
ENSG0000022	RP11-91A18.4	lncRNA	-0.42741172	0.73871169	0.83292035
ENSG0000025	RP11-887P2.6	lncRNA	-0.42750885	0.60562031	0.73201317
ENSG0000025	RP11-769N19	lncRNA	-0.42759532	0.76917354	NA
ENSG0000018	SERHL2	protein_codir	-0.42759835	0.32093702	0.47308963
ENSG0000021	LINC00671	lncRNA	-0.42764837	0.55155536	0.68844717
ENSG0000010	URGCP	protein_codir	-0.42765086	0.00479968	0.01965122
ENSG0000027	RP11-674N23	lncRNA	-0.42781565	0.69122745	0.79731477
ENSG0000020	STK19	protein_codir	-0.42786468	0.00619981	0.0239476
ENSG0000025	ATP6V1G2-DD	protein_codir	-0.42786839	0.61767222	0.74142141
ENSG0000008	ADD1	protein_codir	-0.42788376	0.01381214	0.04469525
ENSG0000013	MORC4	protein_codir	-0.42796479	0.05148339	0.12305047
ENSG0000014	MEAK7	protein_codir	-0.42813108	0.00112703	0.00628606

ENSG0000000	BAD	protein_codir	-0.42814145	0.01392561	0.04499067
ENSG0000006	METTL22	protein_codir	-0.42822371	0.00614517	0.0237883
ENSG0000027	RP11-676J12.!	lncRNA	-0.42828417	0.52373094	0.66584343
ENSG0000010	CDADC1	protein_codir	-0.42839879	0.00473735	0.01944793
ENSG0000017	ZBTB7A	protein_codir	-0.42849616	0.00236715	0.01120877
ENSG0000007	CAPN6	protein_codir	-0.42853828	0.30554956	0.45721523
ENSG0000014	CTDSP1	protein_codir	-0.42855451	0.00423995	0.01782457
ENSG0000027	RP1-101A2.1	lncRNA	-0.42857083	0.16210291	0.28844751
ENSG0000010	CCSER2	protein_codir	-0.42864119	0.10700218	0.21223701
ENSG0000028	TBCE	protein_codir	-0.42867953	0.01530621	0.04843692
ENSG0000006	POLR1A	protein_codir	-0.42873339	0.00869933	0.03119651
ENSG0000001	ZMYND11	protein_codir	-0.42880414	0.07520091	0.1632772
ENSG0000005	GINM1	protein_codir	-0.42892785	0.05640487	0.13165745
ENSG0000016	SUN1	protein_codir	-0.42896364	0.07302479	0.15990999
ENSG0000023	HGH1	protein_codir	-0.42935256	0.00740592	0.02752677
ENSG0000008	BCKDHB	protein_codir	-0.42956709	0.02935809	0.08014138
ENSG0000014	PHLDB2	protein_codir	-0.42958001	0.19433003	0.32981414
ENSG0000014	TIMM29	protein_codir	-0.42968678	0.00577015	0.02264444
ENSG0000015	ADAMTS3	protein_codir	-0.4297912	0.18238191	0.31467944
ENSG0000028	GS1-238N13.1	lncRNA	-0.43000075	0.68112638	0.79034734
ENSG0000001	STEEP1	protein_codir	-0.43010398	0.00967481	0.03391093
ENSG0000025	RP11-449P15.	lncRNA	-0.43018073	0.462538	0.61260938
ENSG0000027	CTA-390C10.1	lncRNA	-0.43025086	0.3224538	0.47459096
ENSG0000017	POP7	protein_codir	-0.43029095	0.00116274	0.00644359
ENSG0000000	CRY1	protein_codir	-0.43060305	0.21357339	0.35329084
ENSG0000017	KCND3	protein_codir	-0.4308894	0.25546954	0.40214685
ENSG0000015	MZT2B	protein_codir	-0.4310768	0.00624989	0.02410922
ENSG0000013	NAB1	protein_codir	-0.43110109	0.01439155	0.04614519
ENSG0000017	VWC2L	protein_codir	-0.43119145	0.79371469	NA
ENSG0000023	SNRK-AS1	lncRNA	-0.43138644	0.16261701	0.28907795
ENSG0000026	AC005943.2	protein_codir	-0.43146206	0.01912586	0.05731948
ENSG0000027	TMEM167B-D	lncRNA	-0.43176771	0.0900925	0.1863378
ENSG0000022	AC005076.5	lncRNA	-0.43188431	0.22667803	0.36876234
ENSG0000028	ABALON	lncRNA	-0.43192229	0.12275473	0.23510752
ENSG0000010	NAGLU	protein_codir	-0.43215707	0.00765622	0.02823294
ENSG0000010	CNTNAP1	protein_codir	-0.43215755	0.11541441	0.22440179
ENSG0000016	SAC3D1	protein_codir	-0.4321803	0.05746156	0.13344726
ENSG0000014	NFIB	protein_codir	-0.43224019	0.12797118	0.24229258
ENSG0000017	ATP5MK	protein_codir	-0.43235123	0.00451159	0.0187074
ENSG0000013	UCK1	protein_codir	-0.43240127	0.02107779	0.06175545
ENSG0000011	LOXL3	protein_codir	-0.43250173	0.06348952	0.14431573
ENSG0000000	IFRD1	protein_codir	-0.43276192	0.14045059	0.25967235
ENSG0000010	ABCC3	protein_codir	-0.43285096	0.25719703	0.40392233
ENSG0000016	CCDC51	protein_codir	-0.43313844	0.01641348	0.05103461
ENSG0000024	LINC02268	lncRNA	-0.4331571	0.6660385	0.77927283
ENSG0000010	PDYN	protein_codir	-0.43330291	0.74511288	0.83724356
ENSG0000002	RTEL1-TNFRSF	protein_codir	-0.43339025	0.17487546	0.30500506

ENSG0000028	RP11-205A8.4	lncRNA	-0.43371505	0.71307342	0.81404843
ENSG0000014	C17orf80	protein_codir	-0.43378305	0.0092318	0.03271941
ENSG0000014	CCT6A	protein_codir	-0.43393375	0.00565274	0.02228498
ENSG0000017	STOX2	protein_codir	-0.43401447	0.10631987	0.21127813
ENSG0000011	TMEM109	protein_codir	-0.43402632	0.00339906	0.01492321
ENSG0000014	MIEN1	protein_codir	-0.43406481	0.00234863	0.01114972
ENSG0000027	RP11-378A12	lncRNA	-0.43406895	0.81597703	NA
ENSG0000025	AP000439.2	lncRNA	-0.4342517	0.81749117	NA
ENSG0000005	SZRD1	protein_codir	-0.43429815	0.02269302	0.06545185
ENSG0000019	ZNF823	protein_codir	-0.43430601	0.11316209	0.22108913
ENSG0000015	SCN3A	protein_codir	-0.4345032	0.28850104	0.43811339
ENSG0000018	ZFP1	protein_codir	-0.43450844	0.02177973	0.06331099
ENSG0000028	RP11-331G2.7	lncRNA	-0.43462517	0.36498647	0.51888647
ENSG0000010	ZC3H12B	protein_codir	-0.43536758	0.18475896	0.31782775
ENSG0000020	ZBTB10	protein_codir	-0.43539274	0.12692208	0.24090384
ENSG0000023	B3GALT4	protein_codir	-0.43560123	0.01225204	0.04074797
ENSG0000022	LINC00323	lncRNA	-0.43569949	0.24648347	0.39175241
ENSG0000018	LRRC57	protein_codir	-0.43590791	0.00043066	0.00289341
ENSG0000017	NAALADL2	protein_codir	-0.43600809	0.19938912	0.33592177
ENSG0000016	AXIN2	protein_codir	-0.43636338	0.14128062	0.26070088
ENSG0000020	TRAF3IP1	protein_codir	-0.43640839	0.04482168	0.11084025
ENSG0000010	TNNC2	protein_codir	-0.43645894	0.55024024	0.68734319
ENSG0000010	KXD1	protein_codir	-0.43659679	0.00142428	0.00757531
ENSG0000006	SLC9A3R2	protein_codir	-0.43671072	0.06025416	0.13847727
ENSG0000019	ZNF181	protein_codir	-0.43674143	0.07024245	0.15543279
ENSG0000007	WSCD2	protein_codir	-0.43680531	0.43920323	0.59027434
ENSG0000013	TRIM29	protein_codir	-0.43690801	0.43280719	0.58406872
ENSG0000018	RTL6	protein_codir	-0.43697506	0.06896609	0.15327091
ENSG0000010	BCL7B	protein_codir	-0.4371806	0.03061186	0.08272182
ENSG0000022	ERCC6	protein_codir	-0.43719429	0.04006256	0.10165012
ENSG0000013	METTL5	protein_codir	-0.43748784	0.0296089	0.0806825
ENSG0000027	AF129408.17	lncRNA	-0.43748879	0.70789985	0.81042003
ENSG0000016	BAP1	protein_codir	-0.43752942	0.00016884	0.00135049
ENSG0000018	TSSC4	protein_codir	-0.43757586	0.01139985	0.03859572
ENSG0000022	PLXNA4	protein_codir	-0.43778204	0.27011337	0.41795693
ENSG0000013	GNG5P2	protein_codir	-0.43780166	0.42090648	0.5729858
ENSG0000007	EIF2B3	protein_codir	-0.43804325	0.00169015	0.0086528
ENSG0000018	HSF1	protein_codir	-0.43805118	0.00395409	0.0168563
ENSG0000016	GPATCH4	protein_codir	-0.43829702	0.02716814	0.0752793
ENSG0000016	FAM219A	protein_codir	-0.43838231	0.00756798	0.02797854
ENSG0000023	PINLYP	protein_codir	-0.43841037	0.09006388	0.1863065
ENSG0000023	MAP3K2-DT	lncRNA	-0.43860259	0.15135333	0.27408727
ENSG0000012	IFT22	protein_codir	-0.43861498	0.03746132	0.09641227
ENSG0000024	HYPK	protein_codir	-0.43865438	0.0081567	0.0296744
ENSG0000016	MOAP1	protein_codir	-0.43866636	0.02974546	0.08096676
ENSG0000007	CA12	protein_codir	-0.43869776	0.3342549	0.48719102
ENSG0000016	PHB	protein_codir	-0.43871246	0.00138612	0.00740167

ENSG0000027	RP11-358N2.2	lncRNA	-0.43874563	0.50790334	0.65235725
ENSG0000026	RP4-576H24.4	protein_codir	-0.43875162	0.68589364	0.79378755
ENSG0000013	ATP8A2	protein_codir	-0.43875605	0.34711397	0.50027213
ENSG0000027	RP11-736N17	lncRNA	-0.43877793	0.58924036	0.71910899
ENSG0000027	CTD-3247H4.2	lncRNA	-0.43888232	0.48829466	0.63538577
ENSG0000018	FAHD1	protein_codir	-0.4390084	0.00130799	0.00709199
ENSG0000009	MRPS18A	protein_codir	-0.4393362	0.0001501	0.00122727
ENSG0000025	RP11-505E24.	lncRNA	-0.43942755	0.55328489	0.68963982
ENSG0000028	RP11-627H22	lncRNA	-0.43949139	0.79349817	0.87174016
ENSG0000016	DENND2B	protein_codir	-0.43965896	0.20156773	0.33860519
ENSG0000013	RAF1	protein_codir	-0.43979475	0.00393062	0.01677696
ENSG0000015	CCDC24	protein_codir	-0.44048959	0.07557595	0.16384675
ENSG0000012	MACF1	protein_codir	-0.44049991	0.07901364	0.16912249
ENSG0000028	F8A1	protein_codir	-0.44065515	0.07115338	0.15688165
ENSG0000013	BEX2	protein_codir	-0.440848	0.21567323	0.35582669
ENSG0000024	SNHG10	lncRNA	-0.44097063	0.11852977	0.22893019
ENSG0000016	GNPDA2	protein_codir	-0.44100481	0.02166737	0.06305475
ENSG0000007	NT5C2	protein_codir	-0.44110747	0.01735483	0.05318323
ENSG0000008	FAT1	protein_codir	-0.44175924	0.2448975	0.3899273
ENSG0000016	DDX19A	protein_codir	-0.44177215	0.00067765	0.00419091
ENSG0000000	PIGQ	protein_codir	-0.44181956	0.00010421	0.00090006
ENSG0000012	ZC3H13	protein_codir	-0.44188128	0.01508468	0.04790605
ENSG0000011	KDSR	protein_codir	-0.44190629	0.10639253	0.21137946
ENSG0000020	PCDHA4	protein_codir	-0.44191519	0.21386344	0.35370708
ENSG0000027	RP11-508N22	lncRNA	-0.44207749	0.31263519	0.46439625
ENSG0000023	AC142528.1	lncRNA	-0.44240151	0.2967856	0.44729992
ENSG0000017	TRAF6	protein_codir	-0.44252323	0.01200597	0.04008763
ENSG0000017	RAB6A	protein_codir	-0.44262309	0.0240511	0.06843425
ENSG0000006	MPC1	protein_codir	-0.44267712	0.07613726	0.1647404
ENSG0000015	LSM11	protein_codir	-0.44274603	0.04301214	0.10736513
ENSG0000022	PROSER2-AS1	lncRNA	-0.44303058	0.30526491	0.45686354
ENSG0000010	FKBP8	protein_codir	-0.44303289	0.0100318	0.03488773
ENSG0000004	VAMP3	protein_codir	-0.44314706	0.01989578	0.05906291
ENSG0000027	RP1-40E16.12	lncRNA	-0.44316185	0.1602464	0.28599157
ENSG0000011	NDUFS8	protein_codir	-0.4432202	0.00163498	0.00844392
ENSG0000011	HDHD3	protein_codir	-0.44324606	0.05744517	0.13342041
ENSG0000025	RP4-559A3.7	protein_codir	-0.44325925	0.63345104	0.75368708
ENSG0000011	NRXN2	protein_codir	-0.44354287	0.06501808	0.14705204
ENSG0000011	PTPA	protein_codir	-0.44364842	0.00021497	0.00163521
ENSG0000023	RP11-446H18	lncRNA	-0.44384151	0.55362192	0.68984198
ENSG0000025	AP003068.9	lncRNA	-0.44390975	0.62257141	0.74535857
ENSG0000027	RP11-97C16.1	lncRNA	-0.44394634	0.19413707	0.32962017
ENSG0000010	MRPL27	protein_codir	-0.44409671	0.00124474	0.00680651
ENSG0000016	TRIM68	protein_codir	-0.44410869	0.00632522	0.02435219
ENSG0000025	RP11-386G11	lncRNA	-0.44415234	0.11870863	0.22910575
ENSG0000025	LINC00567	lncRNA	-0.44425547	0.55504209	0.69109356
ENSG0000021	ST7-OT4	lncRNA	-0.44429954	0.46556819	0.61525604

ENSG0000026	RP11-332H18	lncRNA	-0.44447627	0.62234847	0.74522088
ENSG0000016	NFU1	protein_codir	-0.44459484	0.00944464	0.03328157
ENSG0000010	NFATC4	protein_codir	-0.44474706	0.08078523	0.17195602
ENSG0000000	ARHGAP33	protein_codir	-0.4447849	0.11005674	0.21677011
ENSG0000023	LINC00278	lncRNA	-0.44486546	0.2087249	0.3475812
ENSG0000019	MT-ND6	protein_codir	-0.44501443	0.0896941	0.18575025
ENSG0000027	RP11-1000B6.	lncRNA	-0.44520488	0.09065298	0.18718865
ENSG0000027	PRICKLE4	protein_codir	-0.44528183	0.41952584	0.5720148
ENSG0000016	C1orf74	protein_codir	-0.4453353	0.06085518	0.13948714
ENSG0000015	FLCN	protein_codir	-0.44557495	0.02710314	0.0751395
ENSG0000010	SPAG1	protein_codir	-0.44559912	0.05391724	0.12736178
ENSG0000026	RP11-178L8.5	lncRNA	-0.44561615	0.84561274	NA
ENSG0000027	CTD-2366F13.	lncRNA	-0.44573516	0.51499444	0.65825103
ENSG0000025	RP11-1079K1(	lncRNA	-0.44577617	0.45008997	0.6008591
ENSG0000014	POLR2K	protein_codir	-0.44578105	0.00415908	0.01758491
ENSG0000028	RP11-157K17.	lncRNA	-0.4458447	0.79059405	NA
ENSG0000015	MARVELD1	protein_codir	-0.44587787	0.16747593	0.29575993
ENSG0000025	FOXD1	protein_codir	-0.44597859	0.34855342	0.5018268
ENSG0000011	FBXL5	protein_codir	-0.44599987	0.01545845	0.04878826
ENSG0000015	ZIC1	protein_codir	-0.44601882	0.57160513	0.70479733
ENSG0000025	CTB-193M12.	lncRNA	-0.44603006	0.72894735	0.8257198
ENSG0000010	UBFD1	protein_codir	-0.44611317	0.0034409	0.01506632
ENSG0000013	MAP7	protein_codir	-0.44614936	0.03647562	0.09448153
ENSG0000013	MAP3K10	protein_codir	-0.44621086	0.01922713	0.05756052
ENSG0000011	MAP7D1	protein_codir	-0.44622266	0.01103003	0.03763891
ENSG0000016	NMNAT3	protein_codir	-0.44628758	0.05389403	0.1273247
ENSG0000012	DOHH	protein_codir	-0.44641886	0.01260506	0.04161509
ENSG0000022	RP11-439K3.1	lncRNA	-0.44642335	0.5557887	0.69169936
ENSG0000011	EHHADH	protein_codir	-0.4465667	0.13140435	0.24725037
ENSG0000004	GEMIN8	protein_codir	-0.4466658	0.02138907	0.06244225
ENSG0000024	CTD-2547L16.	lncRNA	-0.44678564	0.19530769	0.33104342
ENSG0000013	SETD1B	protein_codir	-0.44689357	0.04653824	0.11397445
ENSG0000026	ZNF230-DT	lncRNA	-0.44689454	0.19995466	0.33655781
ENSG0000001	MRC2	protein_codir	-0.44689551	0.1158199	0.22499266
ENSG0000027	RP11-533E19.	lncRNA	-0.44705615	0.14996837	0.27226847
ENSG0000027	RP11-468N14	lncRNA	-0.44717797	0.87372688	NA
ENSG0000018	IZUMO1R	protein_codir	-0.44720198	0.50927167	0.65353016
ENSG0000025	LINC02232	lncRNA	-0.44725427	0.87535351	NA
ENSG0000025	ATP6V1E2	protein_codir	-0.44741927	0.00883637	0.03157316
ENSG0000011	EIF2B4	protein_codir	-0.44756552	0.00426835	0.01792487
ENSG0000020	NELFE	protein_codir	-0.44760399	0.00016522	0.00132771
ENSG0000027	ADIRF-AS1	lncRNA	-0.44776669	0.07702839	0.16601827
ENSG0000010	USP11	protein_codir	-0.44784264	0.00063818	0.00399334
ENSG0000013	ZNF132	protein_codir	-0.44801201	0.04685803	0.11451236
ENSG0000026	ZNNT1	lncRNA	-0.44803904	0.1610158	0.28693864
ENSG0000018	YIPF6	protein_codir	-0.44812946	0.04107084	0.1036055
ENSG0000009	CDC34	protein_codir	-0.44831865	0.0036408	0.01575772

ENSG0000012	GNAI1	protein_codir	-0.44834369	0.12989694	0.24497718
ENSG0000019	ZNF560	protein_codir	-0.44840293	0.69430902	0.79963368
ENSG0000015	THYN1	protein_codir	-0.44840777	0.00255253	0.01185886
ENSG0000016	IRS1	protein_codir	-0.44853245	0.17140115	0.30065411
ENSG0000004	RCN1	protein_codir	-0.44863292	0.02552892	0.07178407
ENSG0000023	FSIP2-AS1	lncRNA	-0.44868137	0.7340424	0.8294502
ENSG0000018	LINC00839	lncRNA	-0.44896139	0.26221471	0.40974393
ENSG0000016	WDCP	protein_codir	-0.44899724	0.02653439	0.07389223
ENSG0000016	CDC42EP5	protein_codir	-0.44916503	0.02019155	0.0597291
ENSG0000027	H2BC6	protein_codir	-0.44918988	0.26223908	0.40974393
ENSG0000016	SMIM12	protein_codir	-0.44933165	0.02013756	0.0596141
ENSG0000025	RP3-454B23.1	lncRNA	-0.44938223	0.76028847	NA
ENSG0000028	RP11-422P24.	protein_codir	-0.44945477	0.02630434	0.0734161
ENSG0000027	RP11-528I4.2	lncRNA	-0.44962565	0.62273596	0.74549095
ENSG0000027	GS1-259H13.1	protein_codir	-0.44971058	0.78714946	0.86717825
ENSG0000025	ZNF578	protein_codir	-0.44986969	0.02751621	0.07603778
ENSG0000012	TTBK2	protein_codir	-0.4500182	0.04099469	0.10343229
ENSG0000019	MAP3K5	protein_codir	-0.45009895	0.04204037	0.10554996
ENSG0000018	ZNF696	protein_codir	-0.45048281	0.02098552	0.06153731
ENSG0000017	GATD1	protein_codir	-0.45060288	0.00336701	0.01481549
ENSG0000018	PRKN	protein_codir	-0.45077021	0.23973594	0.38416725
ENSG0000010	MLYCD	protein_codir	-0.45079796	0.00665253	0.02527874
ENSG0000027	CTD-2382E5.6	lncRNA	-0.45082362	0.48606315	0.63325774
ENSG0000008	SIRT4	protein_codir	-0.45084693	0.26859696	0.41633357
ENSG0000016	ARL5A	protein_codir	-0.45092163	0.02313374	0.06640485
ENSG0000014	NDUFS6	protein_codir	-0.45123532	0.00750976	0.0278117
ENSG0000015	RIBC1	protein_codir	-0.45132524	0.2391651	0.38343046
ENSG0000012	PDZD11	protein_codir	-0.45133073	0.00257601	0.01195385
ENSG0000015	EDA	protein_codir	-0.45134272	0.21202288	0.35157095
ENSG0000013	ARL3	protein_codir	-0.45139487	0.00609781	0.02365137
ENSG0000000	TMEM98	protein_codir	-0.45141651	0.09944881	0.20093584
ENSG0000020	TMEM240	protein_codir	-0.45147498	0.21968584	0.36046418
ENSG0000007	ENO1	protein_codir	-0.4515115	0.0158888	0.04985217
ENSG0000001	ZRANB1	protein_codir	-0.45164189	0.03972004	0.10099121
ENSG0000010	GCAT	protein_codir	-0.4516823	0.06736073	0.15092914
ENSG0000027	CTD-2541M15	lncRNA	-0.45176692	0.4436969	0.59469639
ENSG0000021	ARHGEF33	protein_codir	-0.45190388	0.27843622	0.42712627
ENSG0000016	GPX8	protein_codir	-0.45205628	0.10329094	0.20674656
ENSG0000002	VEZT	protein_codir	-0.45232874	0.00743609	0.02760479
ENSG0000010	HSPA8	protein_codir	-0.45238118	0.01421884	0.04574425
ENSG0000018	PTRH1	protein_codir	-0.45238913	0.0401932	0.1019128
ENSG0000009	ABL1	protein_codir	-0.4525842	0.09588764	0.19541184
ENSG0000014	HIKESHI	protein_codir	-0.4533112	0.00269987	0.01242219
ENSG0000011	HINT3	protein_codir	-0.453375	0.03654035	0.09459597
ENSG0000010	ANKRD54	protein_codir	-0.45353165	0.00232518	0.01106883
ENSG0000026	RP11-727F15.	lncRNA	-0.45362829	0.51251546	0.65614522
ENSG0000016	RYK	protein_codir	-0.4538372	0.01739783	0.05327409

ENSG0000013	YIPF2	protein_codir	-0.4538632	0.00519639	0.02092189
ENSG0000027	CTD-2649C14	lncRNA	-0.45392157	0.48650412	0.63374257
ENSG0000019	ZNF615	protein_codir	-0.45421741	0.0439212	0.10904302
ENSG0000016	RAB4A	protein_codir	-0.45425621	0.03894036	0.09938392
ENSG0000012	RBM42	protein_codir	-0.45437395	0.00052032	0.00337747
ENSG0000012	DGCR6L	protein_codir	-0.45463511	0.00080092	0.00479132
ENSG0000015	NEIL2	protein_codir	-0.45467027	0.00046494	0.00308098
ENSG0000026	FSBP	protein_codir	-0.45471992	0.14208397	0.26168046
ENSG0000011	ABCD3	protein_codir	-0.45477848	0.00134593	0.00725216
ENSG0000011	CACYBP	protein_codir	-0.45524192	0.00062205	0.00390787
ENSG0000011	LRRC42	protein_codir	-0.45530952	0.01411918	0.0455096
ENSG0000027	FAM27E3	lncRNA	-0.455331	0.17420312	0.30418852
ENSG0000016	PTK2	protein_codir	-0.45533352	0.0737631	0.16106703
ENSG0000018	ZNF555	protein_codir	-0.45551623	0.07924818	0.16950629
ENSG0000012	CYSTM1	protein_codir	-0.45558506	0.03314668	0.08784411
ENSG0000020	HCG9	lncRNA	-0.45565423	0.68091377	0.7902697
ENSG0000017	LCLAT1	protein_codir	-0.45592302	0.00988554	0.03449198
ENSG0000027	RP5-1136G13	lncRNA	-0.45613274	0.04534223	0.11187711
ENSG0000028	RP11-152N13	lncRNA	-0.45613533	0.25027397	0.39620376
ENSG0000019	ZNF34	protein_codir	-0.45622705	0.06474731	0.14654763
ENSG0000011	CLCN2	protein_codir	-0.45629572	0.02261831	0.06526513
ENSG0000006	GPKOW	protein_codir	-0.45629951	0.00070979	0.0043517
ENSG0000019	CDC42BPB	protein_codir	-0.45637896	0.01650442	0.05125965
ENSG0000027	RP11-514P8.7	protein_codir	-0.45649334	0.77149181	0.85631746
ENSG0000020	SLC35B4	protein_codir	-0.45649536	0.00997154	0.03472186
ENSG0000017	CLSTN1	protein_codir	-0.45676166	8.06E-05	0.00072882
ENSG0000000	CSDE1	protein_codir	-0.45679768	0.03182273	0.08511123
ENSG0000025	RP11-1379J22	lncRNA	-0.45686481	0.24742804	0.3927339
ENSG0000025	CPNE8-AS1	lncRNA	-0.45690282	0.35539537	0.50905047
ENSG0000013	ATAT1	protein_codir	-0.45735088	0.03051863	0.08253948
ENSG0000008	BCORL1	protein_codir	-0.45739358	0.00746748	0.02770517
ENSG0000011	NEDD9	protein_codir	-0.45746248	0.23751241	0.38157822
ENSG0000013	SLC39A5	protein_codir	-0.45775922	0.50453713	0.64922167
ENSG0000016	FBXO8	protein_codir	-0.45788777	0.00205937	0.01005069
ENSG0000027	RP11-78I14.1	lncRNA	-0.45814692	0.68818607	0.79536778
ENSG0000016	NDUFB8	protein_codir	-0.45818923	0.00171366	0.00873879
ENSG0000014	RNF217	protein_codir	-0.45831398	0.12250305	0.23475573
ENSG0000009	PSMD8	protein_codir	-0.45833175	0.00702755	0.0264119
ENSG0000015	TOMM40L	protein_codir	-0.45837982	0.00224296	0.0107553
ENSG0000015	UBP1	protein_codir	-0.45839339	0.00018977	0.00147853
ENSG0000016	TMEM129	protein_codir	-0.4584407	0.00066372	0.00412325
ENSG0000017	INSR	protein_codir	-0.45867164	0.10675339	0.21191093
ENSG0000012	OPTN	protein_codir	-0.45884411	0.0209397	0.06145428
ENSG0000011	SMIM8	protein_codir	-0.45896619	0.01285263	0.04224054
ENSG0000020	LINC00449	lncRNA	-0.45905518	0.62311393	0.74574829
ENSG0000026	RP5-940J5.9	lncRNA	-0.45912134	0.09489914	0.19400843
ENSG0000004	ARHGAP6	protein_codir	-0.45916541	0.15005733	0.27236534

ENSG0000017	MAGED1	protein_codir	-0.45927867	0.01818687	0.05509118
ENSG0000012	ACKR4	protein_codir	-0.45928488	0.35691287	0.51053568
ENSG0000016	CYB5D2	protein_codir	-0.45934962	0.00069143	0.00426471
ENSG0000010	TTC28	protein_codir	-0.45950973	0.03772299	0.09690857
ENSG0000019	SPTAN1	protein_codir	-0.45965897	0.00619468	0.02393635
ENSG0000005	MRPL43	protein_codir	-0.45971235	0.00029056	0.00210033
ENSG0000013	NIPSNAP3A	protein_codir	-0.45992387	0.06030006	0.13855678
ENSG0000014	DYNC1LI1	protein_codir	-0.45997004	0.00365462	0.01579913
ENSG0000006	GUCY1B1	protein_codir	-0.46023717	0.01883147	0.05664619
ENSG0000010	AGFG2	protein_codir	-0.4603635	0.01861418	0.05612704
ENSG0000013	NT5E	protein_codir	-0.46052271	0.0846726	0.17802064
ENSG0000022	LRRK2-DT	lncRNA	-0.46054048	0.07715245	0.16623377
ENSG0000012	SPART-AS1	lncRNA	-0.46067219	0.57662264	0.70903381
ENSG0000025	ZNF674	protein_codir	-0.46077482	0.00595548	0.02319377
ENSG0000021	ZNF580	protein_codir	-0.46078048	0.04528073	0.11180975
ENSG0000022	RP11-153F1.1	lncRNA	-0.46081126	0.65048516	0.76661546
ENSG0000018	BACE1	protein_codir	-0.46098141	0.09091406	0.18761554
ENSG0000022	EXOC3-AS1	lncRNA	-0.46110547	0.0768148	0.16572604
ENSG0000027	CTC-203F4.2	lncRNA	-0.46115752	0.14776823	0.26941438
ENSG0000024	NPHP3-AS1	lncRNA	-0.46122944	0.50111832	0.6461091
ENSG0000018	AGBL4	protein_codir	-0.46136248	0.47622956	0.62442252
ENSG0000018	RAB11B	protein_codir	-0.46150292	0.00041299	0.00280367
ENSG0000026	DDX19A-DT	lncRNA	-0.46169022	0.30477129	0.45622367
ENSG0000015	SAXO1	protein_codir	-0.46187178	0.46817804	0.61755945
ENSG0000014	SLC24A4	protein_codir	-0.4619318	0.18266065	0.31507755
ENSG0000025	YJEFN3	protein_codir	-0.46224973	0.26649773	0.41409885
ENSG0000015	TOMM70	protein_codir	-0.46227226	0.00856893	0.03079287
ENSG0000028	METTL14-DT	lncRNA	-0.46232379	0.47285417	0.62131493
ENSG0000021	HLA-F-AS1	lncRNA	-0.46235957	0.03037362	0.08224699
ENSG0000010	VPS18	protein_codir	-0.46238813	0.00027574	0.00201211
ENSG0000026	RP11-311C24.	lncRNA	-0.46244758	0.17018388	0.29901265
ENSG0000022	NDUFAF8	protein_codir	-0.46257577	0.00854205	0.03071831
ENSG0000025	AC007036.5	lncRNA	-0.46270368	0.38668555	0.53967938
ENSG0000013	SEC31A	protein_codir	-0.46278276	0.02442913	0.06933845
ENSG0000024	RP11-379F4.4	lncRNA	-0.4631302	0.17609211	0.30640431
ENSG0000011	TGFB3	protein_codir	-0.46359753	0.23348065	0.37672249
ENSG0000024	ARPC1A	protein_codir	-0.46360576	0.00160384	0.00830953
ENSG0000011	SLC16A7	protein_codir	-0.4636501	0.12400744	0.23684984
ENSG0000010	ANKMY2	protein_codir	-0.46395839	0.00981796	0.03429952
ENSG0000022	LINC02344	lncRNA	-0.46396299	0.70352477	0.80694992
ENSG0000015	FBXL18	protein_codir	-0.46401993	0.00201605	0.00989013
ENSG0000018	RWDD4	protein_codir	-0.46420235	0.00343805	0.01506327
ENSG0000015	EPS8	protein_codir	-0.46438476	0.11736738	0.22721574
ENSG0000012	INHA	protein_codir	-0.46443366	0.41521099	0.56823543
ENSG0000010	KCND1	protein_codir	-0.46443611	0.01226335	0.04077161
ENSG0000013	ITPRID2	protein_codir	-0.46451595	0.03725911	0.09605158
ENSG0000025	RP11-267M23	lncRNA	-0.46474783	0.29569783	0.44612406

ENSG0000010	POFUT1	protein_codir	-0.46478104	0.01596271	0.05003293
ENSG0000014	TOR1AIP1	protein_codir	-0.46481131	0.06285276	0.14318651
ENSG0000022	RP11-228B15.	lncRNA	-0.4648602	0.19414786	0.32962017
ENSG0000016	ZNF444	protein_codir	-0.46486341	0.01049726	0.03617472
ENSG0000018	MAGI2	protein_codir	-0.4649408	0.14754483	0.26918476
ENSG0000022	LINC02054	lncRNA	-0.46513272	0.76850567	0.85420446
ENSG0000027	RP11-245J9.5	lncRNA	-0.46545748	0.48715763	0.63435452
ENSG0000028	RP11-399L7.4	lncRNA	-0.46567866	0.44008071	0.59102544
ENSG0000018	TSPYL4	protein_codir	-0.46577247	0.00428913	0.01799572
ENSG0000018	LRIT3	protein_codir	-0.46590864	0.46051144	0.61097036
ENSG0000027	PFKP-DT	lncRNA	-0.46594605	0.42496676	0.5769271
ENSG0000016	ZSCAN21	protein_codir	-0.46595623	0.00510372	0.02060888
ENSG0000027	RP11-791G15	lncRNA	-0.4660372	0.34188369	0.49506279
ENSG0000014	ADAMTS10	protein_codir	-0.46607218	0.06995069	0.15502192
ENSG0000015	MBIP	protein_codir	-0.46614367	0.00205652	0.0100407
ENSG0000016	TKT	protein_codir	-0.4661987	0.00110148	0.00617992
ENSG0000025	RP11-400D2.2	lncRNA	-0.46626294	0.67505369	0.78594541
ENSG0000018	SKIDA1	protein_codir	-0.46645151	0.23514491	0.37858948
ENSG0000026	RP11-930P14.	lncRNA	-0.46660378	0.35647438	0.51011981
ENSG0000013	ACTR10	protein_codir	-0.46682106	0.00816188	0.02968152
ENSG0000009	ARCN1	protein_codir	-0.46685969	0.0101579	0.03524197
ENSG0000012	TRMT5	protein_codir	-0.46704117	0.00638588	0.02452421
ENSG0000010	ELOB	protein_codir	-0.4670902	0.01079336	0.03700055
ENSG0000011	ACVR1	protein_codir	-0.46734074	0.03412752	0.08987434
ENSG0000011	COX6A1	protein_codir	-0.46751849	0.00215426	0.01040586
ENSG0000026	ARL14EPL	protein_codir	-0.46759925	0.62556331	0.74756951
ENSG0000010	STUB1	protein_codir	-0.46811036	0.00119953	0.00660903
ENSG0000017	CCDC85B	protein_codir	-0.46843955	0.01638757	0.0509655
ENSG0000012	CGNL1	protein_codir	-0.4685573	0.20074975	0.3375568
ENSG0000011	CCND2	protein_codir	-0.46876479	0.00196311	0.00968405
ENSG0000025	HOXA-AS2	lncRNA	-0.46882124	0.15382044	0.27746347
ENSG0000028	RP11-439L12.	lncRNA	-0.46896623	0.7729088	NA
ENSG0000006	DHX29	protein_codir	-0.46906801	0.01113106	0.03786673
ENSG0000016	TTC8	protein_codir	-0.46907739	0.0374178	0.09635283
ENSG0000014	PROK1	protein_codir	-0.46908456	NA	NA
ENSG0000013	ASS1	protein_codir	-0.46921261	0.0936479	0.19205189
ENSG0000016	PPIC	protein_codir	-0.46927406	0.07276604	0.15950779
ENSG0000014	TP53BP2	protein_codir	-0.46940668	0.18702093	0.3205211
ENSG0000026	RP11-484L8.1	lncRNA	-0.46944086	0.62722986	0.74869652
ENSG0000012	MEA1	protein_codir	-0.46944765	0.0006036	0.00381235
ENSG0000012	MORF4L2	protein_codir	-0.46948994	0.02625041	0.07329522
ENSG0000018	UBQLN2	protein_codir	-0.46949088	0.0009476	0.00548581
ENSG0000012	WBP4	protein_codir	-0.46950747	0.05044216	0.12135997
ENSG0000016	FBXO40	protein_codir	-0.46961065	0.50042966	0.64573078
ENSG0000000	FAM214B	protein_codir	-0.46964142	0.0348316	0.09120675
ENSG0000016	DNAJC21	protein_codir	-0.46965151	0.01204696	0.04019533
ENSG0000022	LINC01128	lncRNA	-0.469829	0.03613976	0.09379631

ENSG0000007	MYH7B	protein_codir	-0.46991575	0.17844645	0.30945507
ENSG0000017	FAM161A	protein_codir	-0.46995003	0.00266224	0.01228591
ENSG0000028	GSEC	lncRNA	-0.46999137	0.03317711	0.08791631
ENSG0000026	CTD-2620I22.	lncRNA	-0.47001578	0.77558899	NA
ENSG0000016	NT5DC2	protein_codir	-0.47009139	0.03352804	0.08862518
ENSG0000020	IPO7	protein_codir	-0.47025226	0.06700107	0.1503428
ENSG0000027	RP11-352M15	lncRNA	-0.47056157	0.02290599	0.065875
ENSG0000010	ANKRD27	protein_codir	-0.47083474	0.00025458	0.00188156
ENSG0000014	NHP2	protein_codir	-0.47099752	0.00649759	0.02487802
ENSG0000021	RNF208	protein_codir	-0.47101229	0.12564341	0.23921356
ENSG0000022	RORB-AS1	lncRNA	-0.47120769	0.69147803	0.7975372
ENSG0000026	RP11-30L15.6	lncRNA	-0.47130582	0.77539733	0.85902989
ENSG0000023	FAM224A	lncRNA	-0.47131963	0.64326119	0.76115474
ENSG0000001	PRICKLE3	protein_codir	-0.47133723	2.51E-05	0.00027733
ENSG0000011	SPARC	protein_codir	-0.4714141	0.13986921	0.25884003
ENSG0000027	CTB-1144G6.6	lncRNA	-0.47146774	0.71456517	NA
ENSG0000022	RP4-633O19_	lncRNA	-0.471665	0.56394212	0.69829111
ENSG0000016	ALKBH4	protein_codir	-0.4718388	0.00945478	0.03331304
ENSG0000014	GUK1	protein_codir	-0.47212502	0.00239376	0.01130256
ENSG0000020	LIN52	protein_codir	-0.4722486	0.00653411	0.02496913
ENSG0000001	TMEM159	protein_codir	-0.4723099	0.15547821	0.27968661
ENSG0000002	RRAGD	protein_codir	-0.47270104	0.02382464	0.06792986
ENSG0000021	LINC02693	lncRNA	-0.47277017	0.01343792	0.04374252
ENSG0000022	FLJ31356	lncRNA	-0.47284909	0.51367984	0.65702682
ENSG0000025	RP11-685N10	lncRNA	-0.47285922	0.4726523	0.62123616
ENSG0000013	CLOCK	protein_codir	-0.4729247	0.02053111	0.06048738
ENSG0000016	MMGT1	protein_codir	-0.4730634	0.03558037	0.09271035
ENSG0000013	HSPBP1	protein_codir	-0.4732362	0.02508932	0.07079038
ENSG0000027	CH507-39O4.	lncRNA	-0.47358035	0.75017434	0.84069688
ENSG0000026	RP11-552F3.1	protein_codir	-0.47362813	0.37796378	0.53161682
ENSG0000017	DRAP1	protein_codir	-0.47366621	0.0148943	0.04746517
ENSG0000016	GPT2	protein_codir	-0.47370225	0.02960558	0.08068142
ENSG0000011	CHPT1	protein_codir	-0.47372406	0.00850978	0.03064009
ENSG0000025	LINC02883	lncRNA	-0.4739793	0.68128076	0.79042991
ENSG0000017	ZNF561	protein_codir	-0.47401055	0.00903938	0.03218196
ENSG0000027	ARHGAP23	protein_codir	-0.47430302	0.11807459	0.22821695
ENSG0000011	HSDL2	protein_codir	-0.47442519	0.06948683	0.15415179
ENSG0000014	DDIT4L	protein_codir	-0.47453656	0.20481651	0.34255977
ENSG0000026	BOP1	protein_codir	-0.47457636	0.03816364	0.09777198
ENSG0000015	AK5	protein_codir	-0.47461801	0.18506133	0.31818345
ENSG0000023	TCF7L1-IT1	lncRNA	-0.47462899	0.77269866	NA
ENSG0000027	RP1-137D17.2	lncRNA	-0.47472909	0.11641793	0.22593193
ENSG0000016	CZIB	protein_codir	-0.47480441	0.00057553	0.00366608
ENSG0000014	FAM71E1	protein_codir	-0.47485499	0.30086749	0.451923
ENSG0000023	RP11-90C4.1	lncRNA	-0.47512691	0.54098554	0.67983169
ENSG0000014	BOD1	protein_codir	-0.47516677	0.0183002	0.05537373
ENSG0000007	ASNS	protein_codir	-0.47529931	0.01810914	0.05491594

ENSG0000011	DHDDS	protein_codir	-0.47538865	0.00039127	0.00268963
ENSG0000027	CTB-113I20.2	lncRNA	-0.47539961	0.1298332	0.24491142
ENSG0000028	RP11-1E4.2	lncRNA	-0.47539966	0.74451722	NA
ENSG0000019	PLXNB2	protein_codir	-0.47540056	0.00184826	0.00922793
ENSG0000007	HSD17B10	protein_codir	-0.47541361	0.01193323	0.03990264
ENSG0000010	PPP2R1A	protein_codir	-0.47542939	0.00472816	0.0194275
ENSG0000023	RP11-536K7.3	lncRNA	-0.47547952	0.02506323	0.070724
ENSG0000016	NLGN2	protein_codir	-0.47563984	0.02440477	0.06927644
ENSG0000025	RP11-21A7A.3	lncRNA	-0.47577039	0.75097801	0.84125619
ENSG0000027	RP5-1165K10.	lncRNA	-0.47587621	0.20938683	0.34841041
ENSG0000028	RP5-1141E15.	lncRNA	-0.47588208	0.7411067	0.83459867
ENSG0000023	B3GNT9	protein_codir	-0.47588445	0.02215637	0.06418318
ENSG0000008	ACOX3	protein_codir	-0.47608545	0.01529705	0.04842458
ENSG0000017	PRNP	protein_codir	-0.47609735	0.05813268	0.13464356
ENSG0000017	DCAKD	protein_codir	-0.47634222	0.00823267	0.02987603
ENSG0000023	AC092835.2	protein_codir	-0.47647485	0.09150881	0.1885752
ENSG0000016	HDGFL3	protein_codir	-0.4765277	0.06429926	0.14572716
ENSG0000007	DGCR2	protein_codir	-0.47684253	0.00020132	0.00155275
ENSG0000016	RAG1	protein_codir	-0.4772494	0.10863532	0.21469
ENSG0000018	AHNAK2	protein_codir	-0.47725245	0.31042236	0.46222792
ENSG0000027	RP3-402G11.2	lncRNA	-0.47727254	0.24417738	0.38923928
ENSG0000015	TMED4	protein_codir	-0.4773451	0.00018009	0.00142039
ENSG0000012	REEP5	protein_codir	-0.47741382	0.01063042	0.03653223
ENSG0000026	RP11-110H1.4	lncRNA	-0.47767356	0.71824765	0.81802813
ENSG0000016	FSTL1	protein_codir	-0.47768096	0.1094677	0.21593328
ENSG0000028	LINC00941	lncRNA	-0.47770932	0.15965157	0.28528022
ENSG0000010	CHMP4B	protein_codir	-0.47781464	0.00149918	0.0078781
ENSG0000014	TPGS1	protein_codir	-0.47782178	0.00364519	0.0157707
ENSG0000025	RP11-20G13.1	lncRNA	-0.47829466	0.44549333	0.59655414
ENSG0000026	RP11-1096G2	lncRNA	-0.47831224	0.56770558	0.70144172
ENSG0000028	RP11-492E3.5	lncRNA	-0.47833532	0.40490886	0.55784032
ENSG0000011	MFAP2	protein_codir	-0.47870342	0.12415083	0.23705789
ENSG0000012	RHOT1	protein_codir	-0.47870584	0.01601567	0.05017044
ENSG0000013	KDELR2	protein_codir	-0.47888784	0.0230877	0.06629402
ENSG0000025	VPS13B-DT	lncRNA	-0.4791389	0.19620921	0.33210555
ENSG0000016	FEM1B	protein_codir	-0.47942982	0.05130313	0.12274709
ENSG0000025	RP11-540O11	lncRNA	-0.47957458	0.80302689	NA
ENSG0000018	SMTNL2	protein_codir	-0.47963706	0.3474671	0.50065425
ENSG0000009	TYRO3	protein_codir	-0.47965082	0.07137083	0.15728575
ENSG0000026	AC002398.11	lncRNA	-0.47989073	0.57086043	0.70423713
ENSG0000012	DNAJC15	protein_codir	-0.48023624	0.00928208	0.03287455
ENSG0000021	FIS1	protein_codir	-0.48030175	0.0078988	0.02892282
ENSG0000022	RP11-314N13	lncRNA	-0.48050129	0.62281155	0.74554912
ENSG0000010	RGP1	protein_codir	-0.48055973	0.0003709	0.00257005
ENSG0000028	CTB-163K11.1	protein_codir	-0.48058613	0.22972161	0.37217919
ENSG0000017	COX14	protein_codir	-0.48093616	0.00110065	0.00617758
ENSG0000008	RBBP9	protein_codir	-0.48119479	0.01904042	0.05710058

ENSG0000010	FBXW4	protein_codir	-0.48131415	0.02845011	0.07820383
ENSG0000011	MDGA1	protein_codir	-0.4815453	0.18715792	0.3206564
ENSG0000006	TBC1D22B	protein_codir	-0.48165626	0.02580817	0.07236769
ENSG0000015	EIF5B	protein_codir	-0.4816878	0.01698225	0.05236668
ENSG0000010	CAMTA2	protein_codir	-0.48172937	1.59E-05	0.00018934
ENSG0000015	SNF8	protein_codir	-0.4817684	0.00218323	0.01052005
ENSG0000015	ABCB9	protein_codir	-0.48198717	0.00934053	0.03302424
ENSG0000027	RP11-680G24	lncRNA	-0.48216118	0.61619076	0.74004088
ENSG0000013	PHC2	protein_codir	-0.48238376	0.02099806	0.06156102
ENSG0000015	MCU	protein_codir	-0.48255423	0.02863626	0.07859817
ENSG0000010	ZNF174	protein_codir	-0.48263913	0.03978855	0.10109095
ENSG0000021	RBMXL1	protein_codir	-0.4829319	0.02397755	0.06828126
ENSG0000024	NNT-AS1	lncRNA	-0.48296043	0.00698843	0.02631727
ENSG0000023	MFF-DT	lncRNA	-0.48297301	0.00786493	0.02882173
ENSG0000022	RP5-956O18.3	lncRNA	-0.48305275	0.51674012	0.65987157
ENSG0000017	DCTN2	protein_codir	-0.48307949	0.00549243	0.02181491
ENSG0000010	TSNAXIP1	protein_codir	-0.48311414	0.2685419	0.41629496
ENSG0000012	QRICH2	protein_codir	-0.48315704	0.04150439	0.10447971
ENSG0000028	RP11-122G18	lncRNA	-0.48323001	0.05875928	0.13573065
ENSG0000027	APP-DT	lncRNA	-0.48324375	0.6927502	0.79836869
ENSG0000015	GRAMD2B	protein_codir	-0.48382032	0.09044504	0.18688501
ENSG0000018	CEP63	protein_codir	-0.48451641	0.00051519	0.00334811
ENSG0000013	KHK	protein_codir	-0.4845199	0.08264202	0.17469976
ENSG0000026	CCPG1	protein_codir	-0.48453252	0.01783065	0.05430164
ENSG0000011	STAM2	protein_codir	-0.48461055	0.01171413	0.03939935
ENSG0000014	MRPL49	protein_codir	-0.4848728	0.00640437	0.02458493
ENSG0000013	CCT7	protein_codir	-0.48496137	0.00113115	0.00630143
ENSG0000020	UQCC3	protein_codir	-0.4849912	0.00106887	0.0060385
ENSG0000016	STPG2	protein_codir	-0.48512602	0.59692254	0.72514957
ENSG0000024	RP11-111H13	protein_codir	-0.48520399	0.17689031	0.30733536
ENSG0000014	CALM2	protein_codir	-0.48530733	0.01749809	0.05353314
ENSG0000014	SAE1	protein_codir	-0.48534926	0.00918019	0.03258247
ENSG0000020	MICA	protein_codir	-0.48535068	0.06197966	0.14152432
ENSG0000016	UBE2V2	protein_codir	-0.48545647	0.00714165	0.0267207
ENSG0000022	FKBP14-AS1	lncRNA	-0.48546778	0.27310788	0.4209867
ENSG0000000	LIG3	protein_codir	-0.48551927	0.00018614	0.0014552
ENSG0000011	FANCE	protein_codir	-0.48553939	0.06817758	0.15214226
ENSG0000011	ZBTB45	protein_codir	-0.48575122	1.27E-05	0.00015719
ENSG0000017	SOCS6	protein_codir	-0.48590027	0.07019448	0.15538453
ENSG0000010	NME3	protein_codir	-0.48592087	0.00777815	0.02858336
ENSG0000013	USP20	protein_codir	-0.48596746	0.02623525	0.07327585
ENSG0000021	NAXD	protein_codir	-0.48601168	0.00029161	0.00210681
ENSG0000024	LINC02044	lncRNA	-0.48612139	0.83163471	NA
ENSG0000012	ECHDC2	protein_codir	-0.48622814	0.13426625	0.25114681
ENSG0000022	LINC00240	lncRNA	-0.48665893	0.16642396	0.29429732
ENSG0000026	RP5-968P14.2	lncRNA	-0.48666935	0.15552068	0.27970835
ENSG0000015	TPRG1L	protein_codir	-0.48681383	0.00593329	0.02313345

ENSG0000011	FN1	protein_codir	-0.48693447	0.27404862	0.42208347
ENSG0000018	CIBAR1	protein_codir	-0.48708427	0.01808657	0.05488366
ENSG0000014	C1orf56	protein_codir	-0.48718444	0.07178179	0.15792912
ENSG0000026	MIR378D2HG	lncRNA	-0.48728956	0.74571395	0.83763549
ENSG0000027	RP11-467L13.	lncRNA	-0.48730725	0.01976955	0.05878297
ENSG0000011	ALDH2	protein_codir	-0.48740246	0.1201993	0.23127167
ENSG0000018	NOMO2	protein_codir	-0.48752836	0.00611821	0.02370819
ENSG0000026	RP11-1C8.5	lncRNA	-0.48752863	0.58098863	0.71261555
ENSG0000016	SNED1	protein_codir	-0.48772249	0.05876704	0.13573721
ENSG0000012	ROMO1	protein_codir	-0.48798797	0.00614516	0.0237883
ENSG0000010	LIN7B	protein_codir	-0.48822653	0.08872331	0.1843064
ENSG0000018	MROH2A	protein_codir	-0.48832573	0.80085775	NA
ENSG0000016	ZNF653	protein_codir	-0.48841856	0.02825917	0.07777185
ENSG0000016	PPDPFL	protein_codir	-0.48844558	0.73259665	0.82832485
ENSG0000014	APP	protein_codir	-0.48852163	0.10721093	0.21248048
ENSG0000021	DDAH2	protein_codir	-0.48860594	0.04120021	0.10383701
ENSG0000015	BOLL	protein_codir	-0.48866524	0.83490036	NA
ENSG0000012	ACOT9	protein_codir	-0.48870161	0.04074183	0.10288274
ENSG0000019	GPAA1	protein_codir	-0.48883244	0.00079578	0.00476777
ENSG0000011	ARHGEF2	protein_codir	-0.4890453	0.01045336	0.03605866
ENSG0000028	COPG2IT1	lncRNA	-0.48918395	0.30003387	0.45098968
ENSG0000027	RP11-152N13	lncRNA	-0.48923656	0.18210709	0.31430344
ENSG0000025	GALNT4	protein_codir	-0.48935833	0.02588359	0.0725129
ENSG0000020	DNAJC19	protein_codir	-0.48937602	0.00111072	0.00621645
ENSG0000016	PMVK	protein_codir	-0.48951649	0.00016405	0.00132235
ENSG0000026	CTC-524C5.2	lncRNA	-0.4895287	0.28676513	0.43637177
ENSG0000028	AC006116.28	lncRNA	-0.48964176	0.46970465	0.61888601
ENSG0000017	COPS9	protein_codir	-0.48965042	0.00026151	0.00192509
ENSG0000008	ZNF343	protein_codir	-0.48988259	0.00586496	0.0229318
ENSG0000018	ZNF396	protein_codir	-0.4899558	0.10560362	0.21018751
ENSG0000016	IER2	protein_codir	-0.48998344	0.22202206	0.36313431
ENSG0000020	FAM229B	protein_codir	-0.49027149	0.06557483	0.14795987
ENSG0000023	MYCBP2-AS1	lncRNA	-0.49031407	0.45382668	0.60425631
ENSG0000027	CTA-212A2.3	lncRNA	-0.49031533	0.76953946	0.85483752
ENSG0000010	RAB2A	protein_codir	-0.49047972	0.00814871	0.02965706
ENSG0000017	MUS81	protein_codir	-0.49050031	0.00646788	0.02479428
ENSG0000016	RNF25	protein_codir	-0.49077674	3.34E-05	0.00034985
ENSG0000022	FAM66C	lncRNA	-0.49078195	0.04577099	0.11252289
ENSG0000015	PRSS53	protein_codir	-0.49082721	0.18054264	0.31209086
ENSG0000027	NPHP3-ACAD	protein_codir	-0.49088403	0.26615176	0.41372757
ENSG0000021	MTCP1	protein_codir	-0.49093688	0.00613277	0.02375361
ENSG0000028	RP11-707K3.1	lncRNA	-0.49114065	0.68266054	0.7913327
ENSG0000003	SARS1	protein_codir	-0.4915267	0.00015774	0.00127652
ENSG0000016	UGP2	protein_codir	-0.49153914	0.0171032	0.05263988
ENSG0000010	EPHB6	protein_codir	-0.49181176	0.08560698	0.17958136
ENSG0000017	C11orf24	protein_codir	-0.49209105	0.01160113	0.03909985
ENSG0000018	GPC6	protein_codir	-0.49235153	0.24640007	0.3916424

ENSG0000011	FBXO9	protein_codir	-0.49242722	0.00561588	0.02219355
ENSG0000016	MAPRE2	protein_codir	-0.49250871	0.00972108	0.03403391
ENSG0000010	SALL1	protein_codir	-0.49251512	0.69303758	0.7984961
ENSG0000017	MAF1	protein_codir	-0.49260241	0.00984985	0.03440223
ENSG0000020	CTD-2126E3.1	lncRNA	-0.4932459	0.57232258	0.70541075
ENSG0000000	NDUFAB1	protein_codir	-0.49332823	3.03E-05	0.00032308
ENSG0000026	CIRBP-AS1	lncRNA	-0.4938277	0.15824934	0.28337164
ENSG0000023	RP11-374P20.	lncRNA	-0.4939658	0.57282554	0.70584169
ENSG0000023	RP11-70C1.3	lncRNA	-0.49397423	0.31794593	0.46990938
ENSG0000025	BLOC1S5-TXN	protein_codir	-0.49424708	NA	NA
ENSG0000023	RP5-1024G6.2	lncRNA	-0.49431469	0.20754997	0.34606279
ENSG0000016	PDZRN4	protein_codir	-0.4944052	0.4820228	0.62950823
ENSG0000026	CTC-457E21.1	lncRNA	-0.49458103	0.53982834	0.6789026
ENSG0000007	ACAT1	protein_codir	-0.49459778	0.04297793	0.10732506
ENSG0000027	LIX1L	protein_codir	-0.49464322	0.00500372	0.02027624
ENSG0000010	TPD52L2	protein_codir	-0.49472349	0.00951856	0.0334567
ENSG0000013	RAI2	protein_codir	-0.49489901	0.21401085	0.3538848
ENSG0000013	CLIP1	protein_codir	-0.49511829	0.06428915	0.14572559
ENSG0000011	LANCL1	protein_codir	-0.49530651	0.02280667	0.06565771
ENSG0000018	S100A3	protein_codir	-0.49568946	0.18840035	0.32227552
ENSG0000015	QDPR	protein_codir	-0.49570624	0.00327978	0.01451723
ENSG0000028	RP11-181L23.	lncRNA	-0.49588419	0.26461854	0.41213286
ENSG0000016	BORCS5	protein_codir	-0.49596386	0.01625969	0.050722
ENSG0000018	BACE2	protein_codir	-0.49606628	0.04071493	0.10283343
ENSG0000026	LINC02185	lncRNA	-0.49611204	0.13744213	0.25518631
ENSG0000016	BMP1	protein_codir	-0.49615009	0.01774656	0.0541135
ENSG0000024	LINC01948	lncRNA	-0.49617098	0.16354907	0.29034743
ENSG0000013	SPART	protein_codir	-0.49627312	0.06861265	0.15271829
ENSG0000028	RP11-259O18	lncRNA	-0.49653696	0.59392251	0.7226376
ENSG0000013	INTS12	protein_codir	-0.49663658	0.00054911	0.00353177
ENSG0000018	TNRC18	protein_codir	-0.49675184	0.0180337	0.05477738
ENSG0000015	TMSB4Y	protein_codir	-0.49677505	0.22876073	0.37123216
ENSG0000014	SNAPIN	protein_codir	-0.49691055	0.00023632	0.0017679
ENSG0000017	FAM98B	protein_codir	-0.49713387	0.00144943	0.00767803
ENSG0000017	PLEC	protein_codir	-0.4971957	0.02287942	0.06581915
ENSG0000025	RP3-414A15.2	lncRNA	-0.49721147	0.56939095	0.70311521
ENSG0000016	PARS2	protein_codir	-0.49745011	0.07099483	0.1566446
ENSG0000013	PRKAB2	protein_codir	-0.49762126	0.03378767	0.08918342
ENSG0000027	CTD-2006H14	lncRNA	-0.49766632	0.19858893	0.33488471
ENSG0000018	TPCN1	protein_codir	-0.49767952	0.00247781	0.01161132
ENSG0000025	AP000640.2	lncRNA	-0.49769725	0.71372224	NA
ENSG0000026	RP11-284F21.	lncRNA	-0.49814654	0.56692152	0.70081782
ENSG0000015	FZD1	protein_codir	-0.49820442	0.14203258	0.26165142
ENSG0000007	SLC46A1	protein_codir	-0.49824375	0.0094312	0.0332427
ENSG0000012	MKKS	protein_codir	-0.49824706	0.00251345	0.01173575
ENSG0000012	GLT8D2	protein_codir	-0.49826874	0.06495812	0.14695251
ENSG0000018	TCEAL8	protein_codir	-0.49834633	0.00089141	0.00522511

ENSG0000019	ZNF358	protein_codir	-0.49836115	0.04785929	0.11639285
ENSG0000010	CLUAP1	protein_codir	-0.4984568	0.0066168	0.02517388
ENSG0000012	SLC44A2	protein_codir	-0.49846098	0.00033387	0.00235541
ENSG0000010	RSPH14	protein_codir	-0.49850187	0.33893218	0.49182025
ENSG0000014	PSMB6	protein_codir	-0.49856299	0.00191598	0.00949395
ENSG0000021	ZNF625-ZNF2	protein_codir	-0.49857913	0.08624781	0.18056389
ENSG0000017	PAIP1	protein_codir	-0.4991178	0.00666557	0.02531322
ENSG0000014	ALAD	protein_codir	-0.49912681	0.00051788	0.003364
ENSG0000009	LZTR1	protein_codir	-0.49917406	0.00040181	0.00274433
ENSG0000014	LRMDA	protein_codir	-0.49933241	0.05676086	0.13227599
ENSG0000016	IFTAP	protein_codir	-0.49937162	0.03471914	0.09100719
ENSG0000026	DNAAF4-CCP(	lncRNA	-0.49953302	0.53969039	0.67882259
ENSG0000015	OTUD6B	protein_codir	-0.49958825	0.00304325	0.01365918
ENSG0000013	PPIL1	protein_codir	-0.49963441	0.01486597	0.0473913
ENSG0000017	ZNF135	protein_codir	-0.49975614	0.02147817	0.06265137
ENSG0000024	AP000304.12	protein_codir	-0.49977483	0.61968679	0.74303435
ENSG0000013	COX17	protein_codir	-0.50005978	0.00112886	0.00629371
ENSG0000028	RP11-571L19.	lncRNA	-0.5001416	0.76470029	0.85165411
ENSG0000017	MICOS13	protein_codir	-0.50043947	0.00313039	0.01397919
ENSG0000025	CTB-79E8.2	lncRNA	-0.50054495	0.79759364	NA
ENSG0000023	MANCR	lncRNA	-0.50060314	0.43568189	0.58694439
ENSG0000018	PJA1	protein_codir	-0.50066684	0.001014	0.00579129
ENSG0000011	ERGIC1	protein_codir	-0.50093716	0.000917	0.00534338
ENSG0000014	SURF6	protein_codir	-0.50099012	0.00160028	0.00829574
ENSG0000023	LINC01719	lncRNA	-0.5010336	0.07532689	0.16346939
ENSG0000018	MRPS23	protein_codir	-0.50126278	0.00091156	0.00531903
ENSG0000016	FBXO22	protein_codir	-0.50132829	0.00225946	0.01082313
ENSG0000028	RP11-545E17.	protein_codir	-0.50145834	0.09860971	0.1996189
ENSG0000011	WLS	protein_codir	-0.50159464	0.03011186	0.08168234
ENSG0000027	RP11-11C20.3	lncRNA	-0.50163772	0.49206718	0.63869992
ENSG0000010	BRF2	protein_codir	-0.50168748	0.00106468	0.00601852
ENSG0000010	TMEM245	protein_codir	-0.50173531	0.05162723	0.12324494
ENSG0000027	RP11-134K13.	lncRNA	-0.50212046	0.46641089	0.61605225
ENSG0000024	AMACR	protein_codir	-0.50261982	0.09958606	0.20113835
ENSG0000011	RNF7	protein_codir	-0.5027013	0.00294677	0.01329465
ENSG0000018	ARL6IP4	protein_codir	-0.50287389	0.0085688	0.03079287
ENSG0000016	ZNF667-AS1	lncRNA	-0.50309254	0.04437244	0.10996561
ENSG0000013	PRADC1	protein_codir	-0.50333302	0.0008458	0.00501092
ENSG0000014	MYOM3	protein_codir	-0.50338091	0.3395995	0.49250398
ENSG0000016	AXL	protein_codir	-0.50341412	0.07810518	0.1676761
ENSG0000011	CAMSAP2	protein_codir	-0.50356731	0.11249362	0.22021929
ENSG0000016	LRPAP1	protein_codir	-0.50378987	0.0005098	0.00331933
ENSG0000011	LMAN2L	protein_codir	-0.5038075	0.00575783	0.02260253
ENSG0000018	BCL9L	protein_codir	-0.50388133	0.00351713	0.01533437
ENSG0000012	FAM210B	protein_codir	-0.50449759	0.00920332	0.03263521
ENSG0000007	DYNC1I2	protein_codir	-0.50452725	0.00585907	0.022912
ENSG0000018	HIGD1A	protein_codir	-0.50457132	0.03398082	0.08956486

ENSG0000027	SUV39H2-DT	lncRNA	-0.50468116	0.17528546	0.30539232
ENSG0000013	MORC2	protein_codir	-0.50477497	0.00070527	0.00433014
ENSG0000008	NFE2L1	protein_codir	-0.50480553	0.00422246	0.01778301
ENSG0000013	DNASE2B	protein_codir	-0.50497472	0.5332113	0.67398728
ENSG0000017	PPID	protein_codir	-0.50504553	0.00244543	0.01148688
ENSG0000016	TPM4	protein_codir	-0.50505176	0.06936992	0.15396397
ENSG0000028	RP4-687F11.2	lncRNA	-0.50531682	0.3845541	0.53776437
ENSG0000025	LINC00640	lncRNA	-0.50549585	0.60893715	0.73460773
ENSG0000018	MMP23B	protein_codir	-0.50566707	0.1348634	0.2518885
ENSG0000028	CTA-215D11.6	lncRNA	-0.50567595	0.33230216	0.48502804
ENSG0000006	EML1	protein_codir	-0.50574886	0.23040114	0.37286457
ENSG0000028	RP11-465L10.	lncRNA	-0.50584212	0.12529951	0.23870689
ENSG0000027	CRYAA2	protein_codir	-0.50587673	0.53067817	0.67198473
ENSG0000013	DNAJB2	protein_codir	-0.50594323	0.00079151	0.00474514
ENSG0000027	LINC01023	lncRNA	-0.50626424	0.17088465	0.29995767
ENSG0000027	MYHAS	lncRNA	-0.50632972	0.71408433	0.81463073
ENSG0000013	MYOF	protein_codir	-0.50645601	0.05208174	0.12404051
ENSG0000011	RNF8	protein_codir	-0.50647539	0.00039887	0.00272761
ENSG0000017	UBQLNL	protein_codir	-0.50655008	0.2938414	0.44409847
ENSG0000009	NUDC	protein_codir	-0.50692137	0.00181042	0.00909367
ENSG0000019	ZSCAN26	protein_codir	-0.50706882	0.03067691	0.08285706
ENSG0000013	RITA1	protein_codir	-0.50709184	6.74E-05	0.00062907
ENSG0000012	VASP	protein_codir	-0.5071521	0.01332446	0.04345
ENSG0000014	TPBG	protein_codir	-0.50720631	0.02746229	0.07591917
ENSG0000023	AC016907.3	lncRNA	-0.50735627	0.5745822	0.70721749
ENSG0000024	ZNF512	protein_codir	-0.50747898	0.00507365	0.02051144
ENSG0000027	RP11-230F18.	lncRNA	-0.50761703	0.55543798	0.69135633
ENSG0000012	KCNJ8	protein_codir	-0.50762705	0.10737947	0.21271038
ENSG0000027	LA16c-380H5.	lncRNA	-0.50783049	0.67819453	0.7881404
ENSG0000015	LDLRAP1	protein_codir	-0.50786603	0.01503045	0.04779427
ENSG0000028	RP11-396M20	lncRNA	-0.50791299	0.6407802	0.75912967
ENSG0000012	PIN1	protein_codir	-0.50795059	0.00017997	0.00141979
ENSG0000011	MVK	protein_codir	-0.50818779	0.01259178	0.04157622
ENSG0000024	BMPR1B-DT	lncRNA	-0.50833727	0.76501716	NA
ENSG0000025	TGFB3-AS1	lncRNA	-0.5086331	0.7646117	NA
ENSG0000021	KLHL23	protein_codir	-0.50866356	0.15734138	0.28222944
ENSG0000025	PCDHGB7	protein_codir	-0.50871004	0.08950238	0.18547838
ENSG0000011	PAPPA2	protein_codir	-0.50872733	0.20562406	0.3436191
ENSG0000018	MOB2	protein_codir	-0.50886547	0.0321587	0.08574413
ENSG0000019	SMOC1	protein_codir	-0.50895663	0.42312125	0.57510008
ENSG0000018	KLHL32	protein_codir	-0.50905738	0.23893609	0.38319075
ENSG0000022	AC097721.2	lncRNA	-0.50914036	0.2434954	0.38856847
ENSG0000013	GDF11	protein_codir	-0.5091986	0.01102062	0.0376161
ENSG0000017	SPRYD4	protein_codir	-0.509237	0.00049419	0.00324188
ENSG0000017	TCEA2	protein_codir	-0.50924479	0.01307117	0.04282121
ENSG0000016	ZNF691	protein_codir	-0.50936712	0.01760731	0.05377793
ENSG0000013	ATP6V1E1	protein_codir	-0.50937194	0.00052453	0.00340002

ENSG0000023	LINC01376	lncRNA	-0.50939375	0.12351742	0.23614253
ENSG0000025	RP11-903H12	protein_codir	-0.50940242	0.23812965	0.38223635
ENSG0000014	IRAK1BP1	protein_codir	-0.50943033	0.03874191	0.09901467
ENSG0000011	AKT3	protein_codir	-0.50944776	0.07455861	0.16226528
ENSG0000014	RERE	protein_codir	-0.50947164	0.0084258	0.03038524
ENSG0000010	PPP1R13L	protein_codir	-0.509553	0.12162756	0.23344421
ENSG0000022	TIAF1	protein_codir	-0.50963775	0.15321713	0.27666429
ENSG0000022	NOL7	protein_codir	-0.50966762	0.00016509	0.00132709
ENSG0000017	SPATA24	protein_codir	-0.50994535	0.06367883	0.14466272
ENSG0000023	LINC01554	lncRNA	-0.50996024	0.44218375	0.59321523
ENSG0000013	CPNE8	protein_codir	-0.51000185	0.00137113	0.00734942
ENSG0000016	TBC1D16	protein_codir	-0.51001124	0.02479506	0.07014642
ENSG0000016	PLAAT5	protein_codir	-0.51028393	0.16786586	0.29622126
ENSG0000010	MAPK10	protein_codir	-0.51039884	0.14847584	0.2704724
ENSG0000017	ZBTB26	protein_codir	-0.51067516	0.00627759	0.02418904
ENSG0000011	RAB5B	protein_codir	-0.51071554	0.00724833	0.02703564
ENSG0000024	RP11-666A20	lncRNA	-0.51074208	0.34323408	0.49639361
ENSG0000012	LRAT	protein_codir	-0.51075215	0.52509129	0.66698958
ENSG0000016	DFFA	protein_codir	-0.51108379	0.00013801	0.00114332
ENSG0000010	PTCD1	protein_codir	-0.5113305	0.00161259	0.00834548
ENSG0000017	LGALS7B	protein_codir	-0.51134159	0.81970137	NA
ENSG0000012	MAPK8IP1	protein_codir	-0.5114398	0.10673271	0.21191093
ENSG0000011	DOCK7	protein_codir	-0.51145155	0.0374013	0.09632824
ENSG0000027	RP11-792A8.4	lncRNA	-0.51152638	0.02036493	0.06011963
ENSG0000023	CTA-150C2.13	lncRNA	-0.5115848	0.82083446	NA
ENSG0000025	ST20-AS1	lncRNA	-0.51189252	0.01106843	0.03773265
ENSG0000013	MMAB	protein_codir	-0.5119102	0.00292951	0.01323839
ENSG0000014	MAP3K6	protein_codir	-0.51211854	0.04040864	0.1023088
ENSG0000028	RP1-158P9.3	protein_codir	-0.51218788	0.36515448	0.51900791
ENSG0000014	OCIAD2	protein_codir	-0.51222273	0.07158227	0.15762306
ENSG0000010	KLHL2	protein_codir	-0.51229909	0.0005492	0.00353177
ENSG0000013	CPLANE2	protein_codir	-0.51257948	0.00042923	0.00288605
ENSG0000013	MTRES1	protein_codir	-0.51295262	0.0008477	0.00501788
ENSG0000009	JAK2	protein_codir	-0.51300909	0.11309535	0.22097436
ENSG0000012	MRPS26	protein_codir	-0.51301563	0.0012624	0.00688401
ENSG0000010	PTPRZ1	protein_codir	-0.51329568	0.3593562	0.51310062
ENSG0000012	APOL5	protein_codir	-0.51342693	0.63516458	0.75497033
ENSG0000010	PSMB5	protein_codir	-0.51346756	0.00191212	0.00947996
ENSG0000019	ARHGEF12	protein_codir	-0.51363941	0.05115699	0.12249295
ENSG0000022	PCOLCE-AS1	lncRNA	-0.51373675	0.27464657	0.42270297
ENSG0000021	ZNF579	protein_codir	-0.51402823	0.0001687	0.00134981
ENSG0000013	PPM1B	protein_codir	-0.51404331	0.05112013	0.12247058
ENSG0000010	PEX12	protein_codir	-0.51410278	0.02663483	0.0741213
ENSG0000014	TRMT10A	protein_codir	-0.51413672	0.02957772	0.08061345
ENSG0000025	FAM242C	lncRNA	-0.51414311	0.71158157	0.81295015
ENSG0000001	RABGAP1	protein_codir	-0.5143424	0.03010993	0.08168234
ENSG0000027	RP1-292L20.3	lncRNA	-0.51440342	0.0549034	0.12904704

ENSG0000017	TCEAL1	protein_codir	-0.51448982	0.16235467	0.28869092
ENSG0000017	CXXC5	protein_codir	-0.51476879	0.00283064	0.0128762
ENSG0000019	KLHL9	protein_codir	-0.5147754	0.0184725	0.05579729
ENSG0000028	RP11-523H20	lncRNA	-0.5149119	0.36450508	0.51845235
ENSG0000017	LSM1	protein_codir	-0.51505653	0.00100723	0.00576333
ENSG0000027	RP11-646I6.6	lncRNA	-0.51512693	0.61723073	0.74095588
ENSG0000016	CCDC171	protein_codir	-0.51513618	0.01473763	0.04706908
ENSG0000023	AC012360.4	lncRNA	-0.51516167	0.67043495	NA
ENSG0000015	RRAGA	protein_codir	-0.5155329	0.00242202	0.01141183
ENSG0000012	BFSP1	protein_codir	-0.5155844	0.1633613	0.29007001
ENSG0000010	TSPAN12	protein_codir	-0.51569447	0.06606054	0.14880052
ENSG0000028	RP11-503P21.	protein_codir	-0.5157198	0.08767638	0.18268117
ENSG0000011	NENF	protein_codir	-0.51572898	0.0108028	0.03702823
ENSG0000026	RP11-388C12.	lncRNA	-0.51588735	0.4314788	0.58273162
ENSG0000010	PDAP1	protein_codir	-0.51593377	0.00235334	0.01115956
ENSG0000027	RP11-395N3.2	lncRNA	-0.51622858	0.31303017	0.46473303
ENSG0000011	ADGRL2	protein_codir	-0.51638554	0.06591385	0.1485428
ENSG0000014	NCAM1	protein_codir	-0.51650248	0.39411686	0.54709292
ENSG0000021	SYCE3	protein_codir	-0.51652001	0.6872121	NA
ENSG0000017	CASKIN2	protein_codir	-0.51653116	0.0828313	0.17493505
ENSG0000014	IGFBP3	protein_codir	-0.51664401	0.07694853	0.16591901
ENSG0000026	GOLGA8M	lncRNA	-0.51691945	0.47981102	0.62761781
ENSG0000017	BBS1	protein_codir	-0.51711078	0.02571132	0.07218354
ENSG0000022	AC008269.2	lncRNA	-0.51720636	0.23937696	0.3837033
ENSG0000013	LRRC1	protein_codir	-0.51733911	0.01116793	0.0379781
ENSG0000010	ENG	protein_codir	-0.51739483	0.08198247	0.17379352
ENSG0000016	UQCRQ	protein_codir	-0.51745694	0.00150819	0.00791641
ENSG0000011	BBX	protein_codir	-0.51747174	0.005227	0.02100536
ENSG0000020	GDI1	protein_codir	-0.51750111	0.00122941	0.00674273
ENSG0000020	KRTAP5-2	protein_codir	-0.51757787	0.79467017	0.8724378
ENSG0000019	TXNRD1	protein_codir	-0.51764931	0.05188722	0.12367271
ENSG0000016	MAPK7	protein_codir	-0.51768816	0.00064961	0.00404742
ENSG0000015	MBNL1	protein_codir	-0.51774013	0.07174513	0.15787129
ENSG0000004	CDH10	protein_codir	-0.51790052	0.60984496	0.73528539
ENSG0000013	HBZ	protein_codir	-0.51795167	0.68598668	0.79378911
ENSG0000010	MCAT	protein_codir	-0.51797163	0.00109006	0.00612936
ENSG0000010	CHCHD2	protein_codir	-0.5181222	0.00134884	0.00726357
ENSG0000016	EMC10	protein_codir	-0.51815739	1.37E-05	0.00016742
ENSG0000018	RTN4RL1	protein_codir	-0.51841799	0.21297925	0.35266709
ENSG0000018	GAS2L1	protein_codir	-0.51862271	0.00668756	0.02537287
ENSG0000026	RP11-120K24.	lncRNA	-0.51867873	0.76172837	0.84957752
ENSG0000015	INPP1	protein_codir	-0.51874429	0.01840307	0.05561798
ENSG0000026	RP11-196G18	lncRNA	-0.51898092	0.19438015	0.3298384
ENSG0000015	ZSCAN1	protein_codir	-0.51952706	0.58805392	0.71825132
ENSG0000014	TCTA	protein_codir	-0.51961651	0.00423133	0.01780364
ENSG0000014	MFAP1	protein_codir	-0.51966858	0.00022503	0.00169957
ENSG0000016	GLYATL1	protein_codir	-0.52018376	0.71532559	NA

ENSG0000028	RP11-181G12	lncRNA	-0.52022192	0.11540767	0.22440179
ENSG0000024	RP11-1334A2	lncRNA	-0.52034172	0.75632841	NA
ENSG0000015	RGPD3	protein_codir	-0.52052793	0.04901652	0.11866388
ENSG0000014	ZNF300	protein_codir	-0.52063508	0.01338041	0.04360672
ENSG0000028	RP4-733M16.	lncRNA	-0.52069346	0.00173293	0.00880945
ENSG0000022	RP11-316M21	lncRNA	-0.52073206	0.71646113	NA
ENSG0000010	SLC35E4	protein_codir	-0.52084701	0.00678556	0.02568413
ENSG0000013	RNF121	protein_codir	-0.52091863	0.0003522	0.0024621
ENSG0000010	TBCB	protein_codir	-0.5210566	0.00067438	0.00417725
ENSG0000018	DYNLT4	protein_codir	-0.52110548	0.23102664	0.37367978
ENSG0000027	H3-2	protein_codir	-0.52118889	0.05581694	0.13061627
ENSG0000024	MIR4458HG	lncRNA	-0.52125578	0.08018525	0.17093121
ENSG0000017	KIF5B	protein_codir	-0.52149731	0.09501522	0.19413525
ENSG0000027	CAHM	lncRNA	-0.5215046	0.15749496	0.28239488
ENSG0000018	COA3	protein_codir	-0.52160011	0.00041785	0.00282504
ENSG0000023	BRD3OS	protein_codir	-0.52161027	0.00206059	0.01005069
ENSG0000028	RP11-429O22	lncRNA	-0.52170346	0.48538322	0.63267034
ENSG0000018	EXOC7	protein_codir	-0.52188299	4.90E-05	0.00048259
ENSG0000023	NIFK-AS1	lncRNA	-0.52201622	0.01016484	0.03526159
ENSG0000025	RP11-783K16.	lncRNA	-0.52206235	0.09473994	0.19374479
ENSG0000025	NDUFC2-KCTI	protein_codir	-0.52214511	0.35105305	0.50463647
ENSG0000012	NECAB3	protein_codir	-0.52214639	0.01314296	0.04301048
ENSG0000013	MLLT1	protein_codir	-0.52218021	0.00021245	0.00162049
ENSG0000025	RP1-117B12.4	lncRNA	-0.52239443	0.23604904	0.37987582
ENSG0000012	ATPAF1	protein_codir	-0.52241939	0.00081637	0.00486789
ENSG0000017	ECEL1	protein_codir	-0.52257056	0.36974869	0.52312564
ENSG0000016	BEND7	protein_codir	-0.52265803	0.08096554	0.17222044
ENSG0000026	RP11-1021N1	protein_codir	-0.52268585	0.53218615	0.67321524
ENSG0000026	RP11-464F9.2	lncRNA	-0.52274216	0.38595319	0.53913259
ENSG0000016	NAALADL1	protein_codir	-0.52289116	0.00016835	0.00134781
ENSG0000017	RAD23A	protein_codir	-0.52331747	0.00260577	0.01207242
ENSG0000013	SLC3A1	protein_codir	-0.52338514	0.33526984	0.48812151
ENSG0000025	RP11-950C14.	lncRNA	-0.52341235	0.72693865	0.82408658
ENSG0000027	PIP4K2B	protein_codir	-0.52351292	0.00011564	0.00098335
ENSG0000025	RP11-806O11	lncRNA	-0.52363358	0.3129897	0.46469792
ENSG0000013	TMED7	protein_codir	-0.5238864	0.0546707	0.12865338
ENSG0000022	PDC-AS1	lncRNA	-0.52395966	0.25661495	0.40323747
ENSG0000025	CTD-2014B16	lncRNA	-0.52399104	0.78375202	NA
ENSG0000017	BPGM	protein_codir	-0.52406633	0.00319088	0.01419884
ENSG0000027	CTD-2026K11	lncRNA	-0.52408004	0.52470703	0.66662411
ENSG0000017	RAB1B	protein_codir	-0.52443983	0.0002116	0.00161667
ENSG0000024	C22orf39	protein_codir	-0.52448486	0.01277334	0.04205502
ENSG0000016	CRADD	protein_codir	-0.5245151	0.00010355	0.00089481
ENSG0000023	AC068580.6	lncRNA	-0.52463318	0.37204074	0.52542646
ENSG0000002	MNAT1	protein_codir	-0.52469123	0.00221429	0.01064557
ENSG0000025	RP11-715J22.	lncRNA	-0.52475813	0.60697897	0.7331741
ENSG0000027	RP11-415J8.7	lncRNA	-0.52479893	0.60973891	0.73525318

ENSG0000013	FRAS1	protein_codir	-0.52493214	0.31136292	0.46317897
ENSG0000016	GOLGA2	protein_codir	-0.52500857	0.00413227	0.01749974
ENSG0000019	SLC6A17	protein_codir	-0.52547275	0.38791753	0.54096144
ENSG0000017	BCL2L1	protein_codir	-0.5255244	0.00425854	0.01789139
ENSG0000020	CRYZL1	protein_codir	-0.52554773	0.00273434	0.01254418
ENSG0000014	PARD3	protein_codir	-0.52558918	0.09497721	0.19411504
ENSG0000025	RP11-644F5.1	lncRNA	-0.52570328	0.00202474	0.009913
ENSG0000027	RP11-961A15	lncRNA	-0.52578847	0.69448355	0.79973465
ENSG0000023	RP11-440G5.2	lncRNA	-0.52579777	0.45254247	0.60307233
ENSG0000010	TRIOBP	protein_codir	-0.52600162	0.00709904	0.02660094
ENSG0000012	KIAA1191	protein_codir	-0.52607646	0.00650384	0.02489081
ENSG0000017	SMIM10L2A	protein_codir	-0.52608035	0.05219008	0.12419019
ENSG0000018	ANKRD46	protein_codir	-0.52615031	0.01744462	0.05339723
ENSG0000010	DMC1	protein_codir	-0.52631727	0.28257361	0.43208123
ENSG0000008	MLF2	protein_codir	-0.52661132	0.00338455	0.01487133
ENSG0000017	PPP1R3B	protein_codir	-0.5266568	0.18334483	0.31590668
ENSG0000026	CTD-2526A2.2	lncRNA	-0.52667136	0.60116687	0.72828012
ENSG0000017	ATOH7	protein_codir	-0.52681791	0.6327809	0.75317425
ENSG0000010	EMC9	protein_codir	-0.52685175	0.00506079	0.02047591
ENSG0000013	GALNT5	protein_codir	-0.52693459	0.28545793	0.43495052
ENSG0000013	USO1	protein_codir	-0.52696053	0.00699869	0.02633927
ENSG0000006	YBX3	protein_codir	-0.52701604	0.08193949	0.1737691
ENSG0000010	DNAJB6	protein_codir	-0.52702354	0.00562588	0.02222022
ENSG0000010	TTLL12	protein_codir	-0.5271023	6.15E-05	0.00058486
ENSG0000010	DHDH	protein_codir	-0.52719668	0.40416482	0.55723313
ENSG0000012	SLC10A3	protein_codir	-0.52726359	0.001592	0.00826367
ENSG0000016	NLRX1	protein_codir	-0.52742526	0.00070863	0.00434597
ENSG0000016	FAM71A	protein_codir	-0.52751641	0.52390991	0.66600965
ENSG0000010	CAV1	protein_codir	-0.52759649	0.13348591	0.25007671
ENSG0000014	PLCB3	protein_codir	-0.52807798	0.01087167	0.0372227
ENSG0000013	MAPKBP1	protein_codir	-0.52826932	0.00036008	0.00250639
ENSG0000012	FOXP2	protein_codir	-0.52833086	0.14330739	0.26337347
ENSG0000025	CLDN23	protein_codir	-0.52854978	0.08322485	0.17560506
ENSG0000014	BEST4	protein_codir	-0.52862074	0.06678417	0.14997795
ENSG0000025	MIR583HG	lncRNA	-0.52879908	0.66376034	0.77741755
ENSG0000015	PINK1	protein_codir	-0.52883449	0.0373844	0.09630266
ENSG0000021	C1orf167	protein_codir	-0.52887451	0.06325286	0.14388437
ENSG0000019	NMB	protein_codir	-0.52892168	0.01594131	0.04998284
ENSG0000028	RP11-333O1.2	lncRNA	-0.52914609	0.41318727	0.56622339
ENSG0000014	BCAS3	protein_codir	-0.52925934	0.00028602	0.00207458
ENSG0000015	FAM168B	protein_codir	-0.52940408	0.00978224	0.03418773
ENSG0000016	AQP2	protein_codir	-0.52943331	0.75285665	0.84275201
ENSG0000010	WDR13	protein_codir	-0.5295085	0.00064866	0.00404609
ENSG0000027	WASL-DT	lncRNA	-0.52958402	0.05323696	0.12611759
ENSG0000013	TIMM10B	protein_codir	-0.52959031	0.02161604	0.06295182
ENSG0000011	ACTR1B	protein_codir	-0.52964361	0.00486337	0.01984724
ENSG0000011	VAMP4	protein_codir	-0.52980244	0.02399042	0.06831086

ENSG0000011	CCNI	protein_codir	-0.52983851	0.02135032	0.0623423
ENSG0000028	HULC	lncRNA	-0.52984449	0.07340544	0.16046366
ENSG0000014	SLC39A6	protein_codir	-0.52999457	0.01689912	0.0521804
ENSG0000024	AP001432.14	lncRNA	-0.53002297	0.6813303	0.79045419
ENSG0000010	NUCB1	protein_codir	-0.53048139	0.00036396	0.00252961
ENSG0000006	ANKS1A	protein_codir	-0.53059612	0.01190608	0.03985046
ENSG0000001	POLR3B	protein_codir	-0.5306738	0.01631356	0.05080924
ENSG0000012	UBA2	protein_codir	-0.5306876	0.03451514	0.0906612
ENSG0000013	NR1H2	protein_codir	-0.53071825	0.00076628	0.00462615
ENSG0000010	SPIN1	protein_codir	-0.53082051	0.01839782	0.05561429
ENSG0000013	NTPCR	protein_codir	-0.53087198	0.00087014	0.00512547
ENSG0000026	ZNF488	protein_codir	-0.53088379	0.32234395	0.47452145
ENSG0000013	TMTC1	protein_codir	-0.53096006	0.15971313	0.2853533
ENSG0000017	AQP11	protein_codir	-0.53096063	0.19243803	0.32738847
ENSG0000026	RP11-214K3.2	lncRNA	-0.53101865	0.36259531	0.51645551
ENSG0000017	SLFNL1	protein_codir	-0.53109484	0.31054833	0.46234071
ENSG0000006	CCN5	protein_codir	-0.53124684	0.33812219	0.49095442
ENSG0000018	TANGO2	protein_codir	-0.53126902	7.42E-05	0.00068042
ENSG0000018	PITPNB	protein_codir	-0.53137428	0.01186831	0.0397608
ENSG0000024	GATA2-AS1	lncRNA	-0.5314257	0.163072	0.28966806
ENSG0000005	ZFR	protein_codir	-0.5317963	0.0101362	0.0351932
ENSG0000018	PCDH18	protein_codir	-0.53199976	0.22055054	0.36130295
ENSG0000014	GID4	protein_codir	-0.53215109	0.05142917	0.12294215
ENSG0000011	PHC1	protein_codir	-0.53235987	0.00163161	0.00843128
ENSG0000022	TRAM2-AS1	lncRNA	-0.53262465	0.02342398	0.06702184
ENSG0000015	WWC2	protein_codir	-0.53268644	0.03836124	0.09818714
ENSG0000027	CTD-2036P10	lncRNA	-0.533246	0.41254577	0.56562492
ENSG0000016	VPS37C	protein_codir	-0.53327105	0.00835572	0.0302114
ENSG0000028	RP3-395C13.2	lncRNA	-0.53343009	0.26017555	0.40737237
ENSG0000014	TMEM177	protein_codir	-0.5336113	0.05677756	0.13228142
ENSG0000028	RP11-178F10.	lncRNA	-0.53384276	0.86991501	NA
ENSG0000025	FAM218A	lncRNA	-0.53404244	0.10252601	0.20565705
ENSG0000014	SCRN3	protein_codir	-0.53418308	0.00177792	0.00897737
ENSG0000010	POLR2C	protein_codir	-0.53421177	0.00122568	0.00672632
ENSG0000025	RP11-638I2.4	lncRNA	-0.53426414	0.58055668	0.7122857
ENSG0000012	TP53TG5	protein_codir	-0.53427187	0.35350941	0.50708543
ENSG0000027	RP11-171I2.3	lncRNA	-0.534482	0.52493455	0.66688249
ENSG0000010	SPECC1L	protein_codir	-0.53448483	0.00056945	0.00363235
ENSG0000013	RDH5	protein_codir	-0.53461182	0.19050502	0.32514049
ENSG0000011	OSCP1	protein_codir	-0.53463426	0.04875376	0.11818316
ENSG0000023	TNFSF12	protein_codir	-0.53468218	0.00084196	0.00499141
ENSG0000016	PPM1L	protein_codir	-0.53475135	0.07776029	0.16715224
ENSG0000015	NIT1	protein_codir	-0.53485942	0.00046284	0.00307017
ENSG0000026	AC138430.4	lncRNA	-0.53499461	0.63815752	0.75738702
ENSG0000027	RP11-15L13.4	lncRNA	-0.53510915	0.54204732	0.68082524
ENSG0000018	SNHG28	lncRNA	-0.53523505	0.08365478	0.17632365
ENSG0000024	TCP10L	protein_codir	-0.53552414	0.46514098	0.61491735

ENSG0000019	ANXA4	protein_codir	-0.5356289	0.00816673	0.02969133
ENSG0000016	HIF1AN	protein_codir	-0.53617194	0.00023618	0.00176748
ENSG0000011	VWA5A	protein_codir	-0.53625275	0.01430715	0.04593856
ENSG0000027	RP11-307C18.	lncRNA	-0.53638631	0.32388314	0.47621253
ENSG0000016	ZNF768	protein_codir	-0.53666488	0.00211496	0.01026088
ENSG0000014	NR1I2	protein_codir	-0.53707253	0.35041095	0.50397575
ENSG0000012	NSRP1	protein_codir	-0.53710251	0.01470832	0.04699177
ENSG0000018	LINC01547	lncRNA	-0.53714824	0.03975946	0.10104663
ENSG0000010	AHSA1	protein_codir	-0.53717289	0.00011526	0.000981
ENSG0000011	PHACTR1	protein_codir	-0.53722059	0.08778957	0.18279291
ENSG0000027	PCGF2	protein_codir	-0.53762612	0.02295146	0.06597143
ENSG0000013	LRRC47	protein_codir	-0.53773897	6.47E-05	0.00060841
ENSG0000019	ARC	protein_codir	-0.5380308	NA	NA
ENSG0000023	RP11-477D19	lncRNA	-0.53808805	0.07021766	0.15540766
ENSG0000016	FBXO27	protein_codir	-0.53813963	0.05563783	0.1303407
ENSG0000025	ODC1-DT	lncRNA	-0.53840202	0.01971403	0.05863682
ENSG0000015	CNDP1	protein_codir	-0.53846517	0.50237153	0.6470877
ENSG0000015	NADK2	protein_codir	-0.53868	0.0069828	0.02630395
ENSG0000014	WDR31	protein_codir	-0.53919856	0.11262942	0.22040701
ENSG0000023	AC104135.2	lncRNA	-0.53923089	0.58128969	0.71271606
ENSG0000028	RP11-105F10.	lncRNA	-0.53924445	0.46798976	0.61734059
ENSG0000015	SETD9	protein_codir	-0.5392799	0.01338981	0.04363221
ENSG0000007	ACTB	protein_codir	-0.53930819	0.0195166	0.05820628
ENSG0000007	HLTF	protein_codir	-0.53937	0.00861893	0.03094449
ENSG0000017	ZNF575	protein_codir	-0.53949947	0.00110601	0.0062014
ENSG0000025	RP11-253E3.3	lncRNA	-0.53994743	0.15522286	0.27930002
ENSG0000010	ZNF629	protein_codir	-0.54019369	0.00073853	0.00449302
ENSG0000001	RNF14	protein_codir	-0.54029044	0.00952369	0.03347047
ENSG0000028	THSD8	protein_codir	-0.54052292	0.42456002	0.57654495
ENSG0000012	L3HYPDH	protein_codir	-0.5405483	0.00650646	0.02489727
ENSG0000021	COLCA2	protein_codir	-0.5407224	0.17182131	0.30121896
ENSG0000014	ZNF185	protein_codir	-0.54081147	0.03384823	0.0892921
ENSG0000011	GBP3	protein_codir	-0.5414886	0.08196475	0.17378263
ENSG0000022	LINC01004	lncRNA	-0.54150161	0.3398171	0.4927678
ENSG0000023	RP11-195F19.	lncRNA	-0.54180736	0.14227962	0.26188876
ENSG0000020	CHML	protein_codir	-0.54200663	0.03032152	0.08212201
ENSG0000010	CDIPT	protein_codir	-0.54203868	5.75E-05	0.00055332
ENSG0000010	THOC5	protein_codir	-0.54204208	3.20E-06	4.88E-05
ENSG0000016	INTU	protein_codir	-0.54205559	0.02751532	0.07603778
ENSG0000027	RP11-401O9.4	lncRNA	-0.54207308	0.61292329	0.73760758
ENSG0000021	ANG	protein_codir	-0.54247712	0.0677824	0.1515771
ENSG0000009	KCNK6	protein_codir	-0.54254547	0.01550022	0.04888844
ENSG0000020	INF2	protein_codir	-0.54277663	6.17E-06	8.53E-05
ENSG0000027	RP11-339B21.	lncRNA	-0.54288772	0.14816911	0.26998488
ENSG0000010	MIB1	protein_codir	-0.54292791	0.07171598	0.15784819
ENSG0000019	MAN2A2	protein_codir	-0.54311722	5.49E-07	1.09E-05
ENSG0000016	PKIG	protein_codir	-0.54336581	0.0902236	0.18651118

ENSG0000028	BCL6-AS1	protein_codir	-0.54336777	0.48020447	0.62796494
ENSG0000011	TSPAN1	protein_codir	-0.54339306	0.06172375	0.14109162
ENSG0000018	PGBD4	protein_codir	-0.54365423	0.03008856	0.08164319
ENSG0000028	RP11-141H1.1	lncRNA	-0.54382019	0.30614218	0.45770526
ENSG0000011	COX7A2	protein_codir	-0.54386772	0.00098934	0.00568215
ENSG0000001	MAN2B2	protein_codir	-0.54391779	0.02577316	0.07230124
ENSG0000026	RP11-248J18.	lncRNA	-0.54399873	0.05190939	0.12369403
ENSG0000017	UNC119B	protein_codir	-0.54409795	0.00245497	0.01151992
ENSG0000016	LZIC	protein_codir	-0.54436071	0.00294358	0.01328675
ENSG0000016	ZNF362	protein_codir	-0.54443725	0.00947629	0.03336547
ENSG0000022	RP13-228J13.	lncRNA	-0.54450753	0.40196758	0.5549771
ENSG0000015	ZNF221	protein_codir	-0.54462793	0.05315808	0.12599555
ENSG0000028	RP11-331G2.9	lncRNA	-0.54463306	0.43123679	0.582527
ENSG0000017	DHFR2	protein_codir	-0.54465893	0.000889	0.00521653
ENSG0000011	PDGFRB	protein_codir	-0.54472699	0.03897325	0.09944029
ENSG0000015	DOCK1	protein_codir	-0.54514774	0.03239458	0.08628141
ENSG0000023	ZNF341-AS1	lncRNA	-0.54549758	0.62128559	0.74436032
ENSG0000019	SERPINA3	protein_codir	-0.54559963	0.21842951	0.35887354
ENSG0000017	ARMC10	protein_codir	-0.54578817	0.00034514	0.0024189
ENSG0000023	MIR3936HG	lncRNA	-0.54585161	0.08182746	0.17358483
ENSG0000027	ZNF454-DT	lncRNA	-0.54598223	0.70388569	NA
ENSG0000016	CFAP97	protein_codir	-0.54601485	0.05609657	0.13110401
ENSG0000013	PGBD1	protein_codir	-0.54606798	0.01026338	0.03554094
ENSG0000023	BNC2-AS1	lncRNA	-0.54616359	0.38377224	0.53710612
ENSG0000014	RMND5B	protein_codir	-0.5462286	0.00219345	0.01056007
ENSG0000026	AC145343.2	lncRNA	-0.54628871	0.6885871	0.79560556
ENSG0000021	TMEM242	protein_codir	-0.54637365	0.00384749	0.01649095
ENSG0000015	PSKH1	protein_codir	-0.5464002	0.00123076	0.00674745
ENSG0000024	PTPRG-AS1	lncRNA	-0.54675443	0.16788285	0.29623231
ENSG0000027	MAPT-IT1	lncRNA	-0.54675486	0.73798775	NA
ENSG0000017	PDE4DIP	protein_codir	-0.54682103	0.01538387	0.04860473
ENSG0000025	CHURC1	protein_codir	-0.54696892	0.00506671	0.0204954
ENSG0000026	LCMT1-AS1	lncRNA	-0.54710807	0.26384613	0.41134738
ENSG0000028	WI2-933P11.2	lncRNA	-0.54715979	0.58257137	0.71377462
ENSG0000011	PARK7	protein_codir	-0.54736304	0.00017927	0.00141513
ENSG0000027	RP11-38C17.1	protein_codir	-0.54748857	0.54019766	0.67927427
ENSG0000016	CALHM4	protein_codir	-0.54748974	0.80801679	NA
ENSG0000018	LRRC66	protein_codir	-0.5475285	0.10416112	0.20802099
ENSG0000013	C12orf29	protein_codir	-0.54768896	0.00458158	0.01895495
ENSG0000025	CTA-481E9.4	lncRNA	-0.54774284	0.85184996	NA
ENSG0000027	RCC1L	protein_codir	-0.548229	6.28E-06	8.64E-05
ENSG0000022	LMCD1-AS1	lncRNA	-0.54856919	0.03146686	0.08438826
ENSG0000028	AP000223.43	lncRNA	-0.54869773	0.46421064	0.61413727
ENSG0000013	DBT	protein_codir	-0.54874239	0.00747288	0.02771599
ENSG0000013	MRPL35	protein_codir	-0.54880986	0.00104415	0.00592308
ENSG0000023	LINC01725	lncRNA	-0.5490778	0.32930731	0.48170045
ENSG0000008	MEF2C	protein_codir	-0.54949903	0.02594261	0.07263404

ENSG0000014	HSPG2	protein_codir	-0.54953601	0.10049004	0.20244601
ENSG0000011	MTARC2	protein_codir	-0.54959587	0.00313911	0.0140106
ENSG0000010	TULP2	protein_codir	-0.54968307	0.42212504	0.57411333
ENSG0000017	BSG	protein_codir	-0.5499442	0.0012243	0.0067227
ENSG0000028	RP11-242G5.1	lncRNA	-0.55027663	0.35312971	0.50669867
ENSG0000026	CTD-2286N8.2	lncRNA	-0.55057409	0.09858911	0.19961837
ENSG0000027	RP13-1032I1.1	lncRNA	-0.5507102	0.34541136	0.4983161
ENSG0000007	PKD2L2	protein_codir	-0.55101675	0.71046267	0.81190694
ENSG0000023	RP11-400L8.2	lncRNA	-0.55103612	0.64567311	0.76315865
ENSG0000026	RP11-120K24.	lncRNA	-0.55115718	0.68536461	0.79330193
ENSG0000020	ODAD4	protein_codir	-0.55140976	0.08260615	0.17469976
ENSG0000022	FAM185A	protein_codir	-0.55157465	0.00022266	0.00168582
ENSG0000010	ABCC1	protein_codir	-0.55170559	0.00108774	0.00611879
ENSG0000016	PSMC3	protein_codir	-0.55179993	0.00338551	0.01487318
ENSG0000028	LMLN2	protein_codir	-0.55198992	0.80950937	NA
ENSG0000025	RP13-46H24.1	lncRNA	-0.55254856	0.47213925	0.62083491
ENSG0000016	PSIP1	protein_codir	-0.55260568	0.00198994	0.00978828
ENSG0000017	ATF7	protein_codir	-0.55261098	0.01053844	0.03628871
ENSG0000020	ZNF425	protein_codir	-0.55265284	0.06211812	0.14180531
ENSG0000027	RP11-334G22	lncRNA	-0.55267277	0.80857234	NA
ENSG0000000	MGST1	protein_codir	-0.55294078	0.26953014	0.41738184
ENSG0000027	RP11-338N10	lncRNA	-0.55296268	0.6515556	NA
ENSG0000012	CFAP58	protein_codir	-0.5531762	0.11825586	0.22851666
ENSG0000017	ZNF25	protein_codir	-0.55322253	0.0219843	0.06379559
ENSG0000010	ITPK1	protein_codir	-0.55333518	0.00155262	0.00808966
ENSG0000007	MBD3	protein_codir	-0.5536075	0.00169161	0.00865549
ENSG0000016	GABRB3	protein_codir	-0.55373389	0.13570934	0.25281883
ENSG0000011	TRIM32	protein_codir	-0.55378029	0.00600382	0.02333598
ENSG0000013	SPRY2	protein_codir	-0.55417916	0.04949355	0.1195986
ENSG0000024	GPRC5D-AS1	lncRNA	-0.55419377	0.21659596	0.35690192
ENSG0000011	ERI3	protein_codir	-0.55437149	0.00251885	0.01174784
ENSG0000016	PDZK1IP1	protein_codir	-0.55457353	0.23377972	0.37706906
ENSG0000028	RP11-107E5.5	lncRNA	-0.55460848	0.15519041	0.27925982
ENSG0000001	UQCRC1	protein_codir	-0.55462092	0.00286365	0.01300032
ENSG0000028	RP11-397E7.6	protein_codir	-0.55477927	0.03504761	0.09163983
ENSG0000027	RP11-474G23	lncRNA	-0.5548338	0.19481874	0.33033909
ENSG0000022	RP11-38L15.2	lncRNA	-0.55491857	0.7137196	0.81440001
ENSG0000028	CTD-3193O13	lncRNA	-0.55492624	0.57074224	0.70418568
ENSG0000013	KCTD1	protein_codir	-0.55514835	0.00255441	0.01186359
ENSG0000010	RS1	protein_codir	-0.55521913	0.84662209	NA
ENSG0000006	PDZD4	protein_codir	-0.55529484	0.11113956	0.21839939
ENSG0000028	RP11-597D13	lncRNA	-0.55557573	0.71488412	0.81510484
ENSG0000011	ECRG4	protein_codir	-0.55592321	0.28531223	0.43487245
ENSG0000008	CDIP1	protein_codir	-0.55604314	0.00032516	0.00230162
ENSG0000013	RBSN	protein_codir	-0.556622	0.00792105	0.02897622
ENSG0000010	ECM2	protein_codir	-0.55676121	0.18508093	0.31818345
ENSG0000014	PLPBP	protein_codir	-0.55683025	4.39E-05	0.00043753

ENSG0000016	NDUFB6	protein_codir	-0.55703667	0.00037283	0.00258084
ENSG0000025	RP11-304L19.	lncRNA	-0.55761269	0.75627089	0.84551357
ENSG0000013	SLC6A8	protein_codir	-0.55769782	0.12733492	0.24146862
ENSG0000013	COPS4	protein_codir	-0.55781492	0.00122123	0.00670848
ENSG0000019	ZNF565	protein_codir	-0.55848554	0.00072289	0.00441634
ENSG0000015	FBXO32	protein_codir	-0.55861702	0.19734834	0.33350228
ENSG0000025	LINC02761	lncRNA	-0.55864367	0.86358733	NA
ENSG0000022	RP5-1077I2.3	lncRNA	-0.55917538	0.77351508	NA
ENSG0000015	B3GNT7	protein_codir	-0.55922893	0.05361899	0.12682679
ENSG0000023	OLMALINC	lncRNA	-0.55932544	0.00278201	0.01270972
ENSG0000025	RP11-860B13.	lncRNA	-0.55964211	0.5497285	0.68700507
ENSG0000013	RRAS2	protein_codir	-0.55971249	0.01783628	0.05430313
ENSG0000018	ZNF74	protein_codir	-0.55972075	0.00373338	0.01607671
ENSG0000017	COL8A2	protein_codir	-0.55974596	0.13709473	0.25479823
ENSG0000028	XXbac-BPG11	lncRNA	-0.55986018	0.1787602	0.30977317
ENSG0000018	PPARA	protein_codir	-0.55988298	0.00269302	0.01240309
ENSG0000013	KLC4	protein_codir	-0.56001045	0.00229747	0.01096412
ENSG0000028	RP11-582J16.	lncRNA	-0.56013798	0.54162898	0.68042356
ENSG0000011	EBNA1BP2	protein_codir	-0.56029172	0.00256679	0.01191708
ENSG0000013	KRBA1	protein_codir	-0.56031933	0.00245085	0.01150452
ENSG0000013	DSCC1	protein_codir	-0.56041481	0.02389756	0.06810136
ENSG0000015	BAG3	protein_codir	-0.56043023	0.20091614	0.33775434
ENSG0000022	PPP1R26-AS1	lncRNA	-0.56051118	0.14539966	0.26636192
ENSG0000017	DOLK	protein_codir	-0.56051164	0.00152698	0.00799226
ENSG0000013	GATA4	protein_codir	-0.56056702	0.59849062	0.72647934
ENSG0000017	PNPLA2	protein_codir	-0.56070086	0.00649538	0.02487771
ENSG0000014	MEP1B	protein_codir	-0.56119737	0.69511931	NA
ENSG0000011	RFX4	protein_codir	-0.56120782	0.66677774	0.7797914
ENSG0000024	GTF2A1L	protein_codir	-0.56123235	0.51874881	0.66173297
ENSG0000024	PNMA2	protein_codir	-0.56129305	0.09648813	0.196349
ENSG0000028	ABC10-43608	lncRNA	-0.56161953	0.62102224	0.74421356
ENSG0000017	EFEMP2	protein_codir	-0.56167867	0.02948503	0.08039255
ENSG0000022	LINC00393	lncRNA	-0.56182257	0.77268992	NA
ENSG0000016	HECTD2	protein_codir	-0.56191509	0.00813423	0.02961607
ENSG0000016	TXNDC2	protein_codir	-0.56193778	0.16576752	0.29332429
ENSG0000006	MTHFD2	protein_codir	-0.56218665	0.00506091	0.02047591
ENSG0000018	MAP6D1	protein_codir	-0.56228198	0.00986931	0.03445477
ENSG0000006	NEDD4	protein_codir	-0.56244642	0.05560789	0.1302816
ENSG0000018	LSMEM1	protein_codir	-0.56259921	0.4134022	0.56647835
ENSG0000010	TMEM59L	protein_codir	-0.56275486	0.38234536	0.53581505
ENSG0000025	MIR3179-3	lncRNA	-0.56283574	0.34582066	0.49882449
ENSG0000015	FLACC1	protein_codir	-0.56285942	0.08608422	0.18028532
ENSG0000027	IRF2-DT	lncRNA	-0.56345993	0.00861486	0.03093778
ENSG0000010	TRIM2	protein_codir	-0.56355827	0.05886848	0.1359147
ENSG0000011	CEP70	protein_codir	-0.56361551	0.02777962	0.0766813
ENSG0000018	CTNNA3	protein_codir	-0.5636731	0.34362433	0.49677592
ENSG0000025	TRIM36-IT1	lncRNA	-0.56379001	0.807374	NA

ENSG0000016	PCBD1	protein_codir	-0.56396541	0.00111355	0.00622975
ENSG0000025	LINC02301	lncRNA	-0.56414997	0.53602477	0.67621285
ENSG0000011	CRYZ	protein_codir	-0.56432868	0.00154701	0.00806413
ENSG0000013	GLS2	protein_codir	-0.5646309	0.14050895	0.2597281
ENSG0000024	RP11-259O2.1	lncRNA	-0.5646673	0.07724459	0.16634147
ENSG0000027	RP5-1021I20.8	lncRNA	-0.56536944	0.24587561	0.39111478
ENSG0000010	POLR3H	protein_codir	-0.56548905	0.00010447	0.00090127
ENSG0000018	MORF4L1	protein_codir	-0.56569328	0.00278114	0.01270972
ENSG0000001	SYT13	protein_codir	-0.56597233	0.51616738	0.65935358
ENSG0000027	RP11-365P13.	lncRNA	-0.5662834	0.29931335	0.45032328
ENSG0000024	AC108488.4	lncRNA	-0.56629277	0.0829092	0.17506977
ENSG0000011	TARS1	protein_codir	-0.56660044	0.00020027	0.00154726
ENSG0000011	TENT5A	protein_codir	-0.5668586	0.05384951	0.12725552
ENSG0000025	KB-1991G8.1	lncRNA	-0.56689065	0.73528296	NA
ENSG0000009	PRTFDC1	protein_codir	-0.56698536	0.10425979	0.20818793
ENSG0000017	GSTA4	protein_codir	-0.56716395	0.00168866	0.00864841
ENSG0000014	SETD7	protein_codir	-0.56717431	0.04614066	0.11322006
ENSG0000016	COPS6	protein_codir	-0.56719935	5.61E-05	0.00054197
ENSG0000013	KIAA1614	protein_codir	-0.5673472	0.03091352	0.08330877
ENSG0000017	FIZ1	protein_codir	-0.5673946	1.56E-05	0.00018575
ENSG0000017	LAMB2	protein_codir	-0.56769221	0.05228739	0.12436943
ENSG0000016	TMCO4	protein_codir	-0.56773608	0.00806229	0.02941618
ENSG0000015	PIH1D2	protein_codir	-0.56791301	0.04092743	0.10330033
ENSG0000013	MTCH1	protein_codir	-0.56796265	0.00017243	0.00137363
ENSG0000025	LINC01397	lncRNA	-0.56810848	0.76828713	NA
ENSG0000013	MPRIP	protein_codir	-0.56815061	0.01519821	0.04818234
ENSG0000022	LINC00840	lncRNA	-0.56817824	0.17014803	0.29898897
ENSG0000007	CTTNBP2	protein_codir	-0.56818793	0.22425636	0.36592303
ENSG0000026	SP2-DT	lncRNA	-0.56893135	0.01214283	0.04046633
ENSG0000026	CTD-3099C6.1	protein_codir	-0.56908738	0.02200198	0.06384019
ENSG0000024	GOLGA8K	protein_codir	-0.56913903	0.04530875	0.11184171
ENSG0000013	LRRCC1	protein_codir	-0.56921588	0.00681388	0.0257666
ENSG0000013	BBS7	protein_codir	-0.56930139	0.00655025	0.02499901
ENSG0000017	ZNF584	protein_codir	-0.56932934	0.00550059	0.02183475
ENSG0000023	RP1-266L20.2	lncRNA	-0.56943771	0.60236248	0.72932017
ENSG0000007	REXO2	protein_codir	-0.569609	0.00377374	0.01622773
ENSG0000006	PTPRU	protein_codir	-0.5697088	0.04251984	0.10651169
ENSG0000017	ANO6	protein_codir	-0.56988468	0.10721725	0.21248048
ENSG0000009	HSP90AB1	protein_codir	-0.5699017	0.0035292	0.01537628
ENSG0000027	RP11-307C12.	lncRNA	-0.56998609	0.03431011	0.09027143
ENSG0000025	RP11-867G23	lncRNA	-0.57048883	0.44274391	0.59384906
ENSG0000025	RP11-227D13	lncRNA	-0.57060688	0.25519	0.40184424
ENSG0000027	CBSL	protein_codir	-0.57079906	0.55771126	0.69344424
ENSG0000011	CHMP3	protein_codir	-0.57082	0.02206868	0.06399337
ENSG0000020	GABBR1	protein_codir	-0.57084225	0.05794899	0.13427441
ENSG0000016	U2AF1	protein_codir	-0.57116922	0.41375987	0.56692369
ENSG0000023	AP000253.1	lncRNA	-0.57120603	0.38866005	0.54156122

ENSG0000028	RP11-3N2.15	lncRNA	-0.57120963	0.86086397	NA
ENSG0000012	ANXA11	protein_codir	-0.57132943	0.00784861	0.02876573
ENSG0000011	RBP1	protein_codir	-0.57147585	0.14228453	0.26188876
ENSG0000018	ZNF572	protein_codir	-0.57159455	0.08085921	0.1720655
ENSG0000005	HPF1	protein_codir	-0.57167696	0.00036199	0.00251716
ENSG0000026	RP11-629B11.	lncRNA	-0.57179099	0.30671387	0.4583863
ENSG0000028	RP11-205M20	protein_codir	-0.57207764	0.29966212	0.45072521
ENSG0000015	SKI	protein_codir	-0.57238835	0.00872966	0.03126467
ENSG0000020	JPT2	protein_codir	-0.57262342	0.00360942	0.01565026
ENSG0000022	RP11-441O15	lncRNA	-0.57300469	0.1543013	0.2781259
ENSG0000016	CLASP2	protein_codir	-0.57313814	0.01270768	0.0418688
ENSG0000028	RP11-499P9.1	lncRNA	-0.57317278	0.11046246	0.21735222
ENSG0000008	MTMR2	protein_codir	-0.57317994	0.00970399	0.03399165
ENSG0000026	RP11-64C12.6	lncRNA	-0.57320926	0.6630854	NA
ENSG0000013	PEMT	protein_codir	-0.57336657	0.01515245	0.04806599
ENSG0000012	ZNF576	protein_codir	-0.57337708	3.49E-07	7.38E-06
ENSG0000010	DVL1	protein_codir	-0.57339479	0.0009011	0.00526656
ENSG0000001	CALCOCO1	protein_codir	-0.57350728	0.03030582	0.08210365
ENSG0000013	LGR5	protein_codir	-0.57354914	0.25359506	0.39994481
ENSG0000027	RP11-295D4.4	lncRNA	-0.57362905	0.41390424	0.56700899
ENSG0000027	CASC15	lncRNA	-0.57376202	0.05449542	0.12834898
ENSG0000011	BLZF1	protein_codir	-0.5739823	0.0002597	0.00191432
ENSG0000018	PABIR1	protein_codir	-0.57400722	0.00101262	0.00578535
ENSG0000016	ACP6	protein_codir	-0.57407583	0.0059986	0.02331898
ENSG0000014	MMUT	protein_codir	-0.5742401	0.00841807	0.03036135
ENSG0000026	CTC-250I14.6	lncRNA	-0.57494021	NA	NA
ENSG0000023	MKX-AS1	lncRNA	-0.57507985	0.57023977	0.70375435
ENSG0000023	FAM41C	lncRNA	-0.57521817	0.38543937	0.53864782
ENSG0000028	RP5-973N23.5	protein_codir	-0.57566927	0.09884729	0.19999723
ENSG0000010	MET	protein_codir	-0.57590133	0.14399172	0.26421976
ENSG0000013	SLAIN1	protein_codir	-0.57596876	0.02555003	0.07183346
ENSG0000015	EIF4A2	protein_codir	-0.57610357	3.54E-05	0.00036683
ENSG0000006	OAT	protein_codir	-0.57614725	0.0234261	0.06702184
ENSG0000016	STIP1	protein_codir	-0.57631397	0.00034446	0.00241654
ENSG0000018	KBTBD3	protein_codir	-0.57676235	0.02225627	0.06440212
ENSG0000002	NRXN3	protein_codir	-0.57683808	0.22101672	0.36185428
ENSG0000023	LINC00957	lncRNA	-0.57687964	0.00113524	0.00631847
ENSG0000025	CTD-2308G16	lncRNA	-0.57743687	0.80418547	NA
ENSG0000025	RP11-474P2.2	lncRNA	-0.5775732	0.80274591	NA
ENSG0000027	RP11-321N4.5	protein_codir	-0.57771859	0.56652733	0.70055
ENSG0000026	RP11-143I21.1	lncRNA	-0.5777379	0.80101856	NA
ENSG0000012	RASSF8	protein_codir	-0.57788378	0.12187179	0.23382181
ENSG0000010	GAPDHS	protein_codir	-0.57790532	0.79928187	NA
ENSG0000015	PTPRO	protein_codir	-0.57806206	0.13022588	0.24546775
ENSG0000012	ECHS1	protein_codir	-0.5781146	0.00050715	0.00330598
ENSG0000013	NAV1	protein_codir	-0.57860616	0.02461562	0.06974593
ENSG0000019	STRN3	protein_codir	-0.57877686	0.008495	0.03059885

ENSG0000018	TSKU	protein_codir	-0.57884238	0.07933352	0.16963086
ENSG0000024	UBE2D3-AS1	lncRNA	-0.57885944	0.00978959	0.03420906
ENSG0000023	LINC00322	lncRNA	-0.57938982	0.7440407	0.83650442
ENSG0000025	SPESP1	protein_codir	-0.57969143	0.14123023	0.26062539
ENSG0000006	NTHL1	protein_codir	-0.57981394	8.95E-05	0.0007943
ENSG0000010	SFXN3	protein_codir	-0.57998595	0.00970182	0.03398837
ENSG0000022	RP3-323P24.3	lncRNA	-0.58021123	0.34272616	0.49586676
ENSG0000025	RP3-405J10.3	lncRNA	-0.58025142	0.20954184	0.34862632
ENSG0000023	RP11-469A15	lncRNA	-0.58030382	0.31004903	0.46180385
ENSG0000015	FAIM	protein_codir	-0.58047978	0.00056604	0.00361482
ENSG0000025	MRPL46	protein_codir	-0.58080633	2.67E-05	0.00029185
ENSG0000024	RP11-89B16.1	lncRNA	-0.58082749	0.36191705	0.51582377
ENSG0000017	SH3BP5L	protein_codir	-0.58113939	0.0003216	0.00227933
ENSG0000012	A4GALT	protein_codir	-0.58116632	0.02038008	0.06015151
ENSG0000014	EI24	protein_codir	-0.58151246	0.00088615	0.00520312
ENSG0000026	AP000223.42	lncRNA	-0.58160079	0.39366822	0.54669204
ENSG0000025	RP11-950C14.	lncRNA	-0.58174996	0.5527253	0.68934673
ENSG0000026	CCDC177	protein_codir	-0.58223962	0.75923712	NA
ENSG0000019	PPP1R26	protein_codir	-0.58223977	0.00207365	0.01010482
ENSG0000015	GRHL3	protein_codir	-0.58225202	0.45148227	0.60210526
ENSG0000025	CUX1	protein_codir	-0.5822763	0.00680597	0.02574729
ENSG0000014	CTDSPL	protein_codir	-0.58249734	0.05106965	0.12240053
ENSG0000016	CDC42EP3	protein_codir	-0.58257336	0.04539011	0.11193524
ENSG0000011	DAP	protein_codir	-0.58294591	0.00994531	0.03466139
ENSG0000022	TRIM16	protein_codir	-0.5830733	0.00111063	0.00621645
ENSG0000025	SMIM10L1	protein_codir	-0.58342476	0.00163973	0.00845583
ENSG0000024	YAE1	protein_codir	-0.58355863	1.26E-05	0.00015611
ENSG0000008	SLC27A5	protein_codir	-0.58359452	0.01627561	0.05075446
ENSG0000027	RP1-79C4.4	lncRNA	-0.58367305	0.33925621	0.49211684
ENSG0000020	ZBTB12	protein_codir	-0.58370022	0.00133823	0.00722052
ENSG0000026	ZNF285	protein_codir	-0.58393427	0.11591998	0.22512394
ENSG0000028	RP11-120K9.3	protein_codir	-0.5839897	0.68460106	0.79271986
ENSG0000015	CNTNAP5	protein_codir	-0.58415718	0.72971249	0.8261428
ENSG0000021	CRIP1	protein_codir	-0.58416051	0.10314971	0.20653871
ENSG0000023	CTC-338M12.	lncRNA	-0.58449362	0.01111692	0.03783869
ENSG0000025	PDF	protein_codir	-0.58455093	0.01631914	0.05080924
ENSG0000019	LAMA2	protein_codir	-0.58483512	0.22545358	0.36720297
ENSG0000016	DCST1	protein_codir	-0.58488371	0.57539974	0.70790828
ENSG0000016	MST1R	protein_codir	-0.58490583	0.14368176	0.2638273
ENSG0000017	NUDT4	protein_codir	-0.5849138	0.01314085	0.0430087
ENSG0000015	ANKRD50	protein_codir	-0.58502392	0.057844	0.13409865
ENSG0000017	WSB2	protein_codir	-0.58512239	0.0080032	0.02922762
ENSG0000027	RP11-138E2.1	lncRNA	-0.58512551	0.65033758	0.7665095
ENSG0000018	PRKD1	protein_codir	-0.58515606	0.07846043	0.16825133
ENSG0000013	ABI2	protein_codir	-0.58602548	0.00915675	0.03251601
ENSG0000028	RP11-274G22	lncRNA	-0.58606054	0.62776786	0.74904736
ENSG0000017	PLAAT3	protein_codir	-0.58609976	0.00951245	0.03344799

ENSG0000023	ELN-AS1	lncRNA	-0.58619311	0.3471298	0.50027213
ENSG0000015	FAM160B2	protein_codir	-0.58627418	0.00021902	0.00166235
ENSG0000017	PXT1	protein_codir	-0.58628297	0.51186229	0.65558249
ENSG0000025	RP11-298I3.4	lncRNA	-0.58632486	0.3842291	0.53750039
ENSG0000016	DDR2	protein_codir	-0.58633094	0.08573559	0.17976293
ENSG0000024	RNF103-CHM	protein_codir	-0.58643586	0.57735984	0.70965595
ENSG0000016	CAPN2	protein_codir	-0.58663609	0.0124546	0.0412218
ENSG0000007	RGS11	protein_codir	-0.58686684	0.15727789	0.28215221
ENSG0000009	TMEM38B	protein_codir	-0.58696109	0.00679934	0.02572926
ENSG0000012	CITED1	protein_codir	-0.58697436	0.28207357	0.43150772
ENSG0000019	TEAD4	protein_codir	-0.5871216	0.06015604	0.13826329
ENSG0000013	ADAMTS7	protein_codir	-0.58727143	0.05846577	0.13525343
ENSG0000026	CTD-2371O3.3	lncRNA	-0.5872917	0.06909636	0.15347339
ENSG0000028	RP11-705O3.3	lncRNA	-0.58744371	0.18864353	0.3225614
ENSG0000010	R3HCC1	protein_codir	-0.58748431	0.0001779	0.00140706
ENSG0000017	TCP11L1	protein_codir	-0.58759946	0.00665202	0.02527874
ENSG0000018	DIPK2A	protein_codir	-0.58765931	0.02292447	0.06591006
ENSG0000019	H2AC11	protein_codir	-0.58771439	0.0937426	0.19218902
ENSG0000024	FGF14-IT1	lncRNA	-0.58802503	0.44356464	0.59457682
ENSG0000016	S100A1	protein_codir	-0.58810783	0.15517161	0.27924419
ENSG0000010	CDR2L	protein_codir	-0.5881779	0.02636462	0.07353234
ENSG0000027	LINC02881	lncRNA	-0.58821827	0.73464191	0.82975421
ENSG0000016	SLC25A34	protein_codir	-0.58854568	0.04698794	0.11472832
ENSG0000013	TSSK4	protein_codir	-0.58864941	0.61943939	0.74289691
ENSG0000017	FBXW10	protein_codir	-0.58874109	0.47727521	0.62533556
ENSG0000011	TMEM9	protein_codir	-0.58877104	5.68E-05	0.00054762
ENSG0000017	RAB33B	protein_codir	-0.58877132	0.01680812	0.05196914
ENSG0000004	PPP1R3F	protein_codir	-0.58877537	0.0038952	0.0166515
ENSG0000026	RP11-573N10	lncRNA	-0.58915614	0.66697672	0.77986186
ENSG0000028	RP4-660H19.2	lncRNA	-0.58925476	0.2724692	0.42039483
ENSG0000027	RP11-1060J15	lncRNA	-0.58942767	0.23720483	0.38130801
ENSG0000018	B3GALT5-AS1	lncRNA	-0.58964319	NA	NA
ENSG0000026	AC139100.3	lncRNA	-0.58966636	0.44682658	0.59773091
ENSG0000013	SLCO5A1	protein_codir	-0.58977781	0.11855166	0.22894677
ENSG0000011	RAB32	protein_codir	-0.59005494	0.0494698	0.11955166
ENSG0000010	EEF2K	protein_codir	-0.59016097	1.93E-05	0.00022224
ENSG0000019	TUBB	protein_codir	-0.59034392	0.00435996	0.01821635
ENSG0000001	STMN4	protein_codir	-0.59035689	0.45670339	0.6072588
ENSG0000016	TUBA1C	protein_codir	-0.59039697	0.0521661	0.12416647
ENSG0000016	PTCHD1	protein_codir	-0.59051348	0.55798961	0.69362586
ENSG0000010	NOMO1	protein_codir	-0.59082754	0.01006508	0.03496825
ENSG0000015	WDR19	protein_codir	-0.59092594	0.00768491	0.02830473
ENSG0000013	ZC3H10	protein_codir	-0.59112719	0.00055263	0.00354721
ENSG0000009	BCL7C	protein_codir	-0.59114094	0.00224683	0.01076825
ENSG0000013	SUOX	protein_codir	-0.59114362	0.00023182	0.00174036
ENSG0000018	C12orf60	protein_codir	-0.59117428	0.07064582	0.15612397
ENSG0000017	DHCR7	protein_codir	-0.59120087	0.01843138	0.05567933

ENSG0000027	AL133243.3	lncRNA	-0.59175644	0.6845295	0.79271986
ENSG0000012	TWIST1	protein_codir	-0.59178329	0.10526539	0.20965028
ENSG0000013	CTIF	protein_codir	-0.59229675	0.0222596	0.06440501
ENSG0000011	CEP104	protein_codir	-0.59235641	0.00319161	0.01419978
ENSG0000013	C7orf25	protein_codir	-0.59242116	0.03505672	0.09163983
ENSG0000011	IPO13	protein_codir	-0.59263883	2.20E-05	0.00024785
ENSG0000010	SPEF1	protein_codir	-0.59294632	0.28145311	0.43082104
ENSG0000012	PLA2G12A	protein_codir	-0.59302593	0.01025401	0.03551741
ENSG0000027	RP11-108L7.1	lncRNA	-0.59318838	0.72196958	NA
ENSG0000019	KIFBP	protein_codir	-0.59341484	0.00189053	0.00939019
ENSG0000027	TERC	lncRNA	-0.59343974	0.75602797	0.84537034
ENSG0000010	SLC16A8	protein_codir	-0.59345037	0.20289255	0.34004201
ENSG0000016	PPP1R16A	protein_codir	-0.59370533	1.98E-05	0.00022706
ENSG0000025	MUC5B-AS1	lncRNA	-0.59377423	0.65520394	0.77056559
ENSG0000015	NPM2	protein_codir	-0.59420561	0.14728319	0.26881396
ENSG0000022	AC009961.3	lncRNA	-0.59422367	0.01755699	0.05365393
ENSG0000027	AC005753.1	lncRNA	-0.59429737	0.41648808	0.56927801
ENSG0000027	RP11-307L14.	lncRNA	-0.59432262	0.51426317	0.65753648
ENSG0000019	LINC00477	lncRNA	-0.59455466	0.76069495	NA
ENSG0000023	LINC00899	lncRNA	-0.5947451	0.05090128	0.1221536
ENSG0000008	PEBP1	protein_codir	-0.59484695	0.00482486	0.019737
ENSG0000025	RP11-977G19	lncRNA	-0.59501727	0.01941146	0.0579944
ENSG0000016	BRD7	protein_codir	-0.59502481	0.00144393	0.00765477
ENSG0000023	LINC00159	lncRNA	-0.59509712	0.7545505	NA
ENSG0000019	MIRLET7BHG	lncRNA	-0.59510059	0.10355347	0.20715194
ENSG0000010	SLC25A1	protein_codir	-0.59512863	2.01E-05	0.00023051
ENSG0000018	PBX1	protein_codir	-0.59519407	0.11900838	0.22959298
ENSG0000019	ZFP28	protein_codir	-0.59576424	0.00961948	0.03374701
ENSG0000017	UBQLN3	protein_codir	-0.59581826	0.62820533	0.74947221
ENSG0000007	KCNQ2	protein_codir	-0.59591727	0.74961276	0.84047676
ENSG0000000	PDK2	protein_codir	-0.59604707	0.01534174	0.04851047
ENSG0000018	H2AC21	protein_codir	-0.59605268	0.34169517	0.49489358
ENSG0000026	ATF7-NPFF	protein_codir	-0.59615929	0.49434748	0.64047535
ENSG0000028	RP11-960B9.3	protein_codir	-0.59625041	0.83564585	NA
ENSG0000010	ESS2	protein_codir	-0.59629088	2.28E-06	3.69E-05
ENSG0000010	SNAPC2	protein_codir	-0.59652343	5.05E-05	0.00049526
ENSG0000027	RP11-408A13	lncRNA	-0.59653098	0.55192984	0.68872785
ENSG0000027	H3C1	protein_codir	-0.59672727	0.40459599	0.55754115
ENSG0000017	SNX33	protein_codir	-0.59707616	0.02023122	0.05981585
ENSG0000011	ACSS3	protein_codir	-0.59732054	0.02695542	0.07481747
ENSG0000019	NLGN3	protein_codir	-0.59735817	0.04833469	0.11733481
ENSG0000027	RP11-12D24.1	lncRNA	-0.59757731	0.65309769	0.76880868
ENSG0000017	YIF1A	protein_codir	-0.59785513	0.00232946	0.01108384
ENSG0000018	TMEM186	protein_codir	-0.59798542	0.00059453	0.00376798
ENSG0000026	PCDHGA9	protein_codir	-0.59812068	0.03706537	0.09566822
ENSG0000007	TOLLIP	protein_codir	-0.59838101	0.00029762	0.00213792
ENSG0000017	FAM182B	lncRNA	-0.59857588	0.11761954	0.22757629

ENSG0000023	RP11-360O19	lncRNA	-0.59869024	0.71800175	NA
ENSG0000028	RP11-642C5.2	lncRNA	-0.59874707	0.71386695	NA
ENSG0000023	SDK1-AS1	lncRNA	-0.59882665	0.47598766	0.6242413
ENSG0000024	RASSF8-AS1	lncRNA	-0.59889639	0.00099941	0.00572688
ENSG0000012	ARL1	protein_codir	-0.59909849	0.00234606	0.01114268
ENSG0000023	RP4-604G5.1	lncRNA	-0.59916633	0.71360685	NA
ENSG0000018	RPS27L	protein_codir	-0.59921588	0.00025195	0.00186712
ENSG0000011	PLCD4	protein_codir	-0.59928895	0.06798582	0.15188614
ENSG0000016	KIAA1522	protein_codir	-0.59934115	0.00021226	0.00161998
ENSG0000024	KRBOX1	protein_codir	-0.5994885	0.20275559	0.33989252
ENSG0000023	RP11-517P14.	lncRNA	-0.59951325	0.03544011	0.09244083
ENSG0000001	PHLDB1	protein_codir	-0.59955749	0.00501551	0.02031802
ENSG0000025	RP11-363J20.	lncRNA	-0.59962982	0.53146554	0.67254853
ENSG0000026	ANXA8L1	protein_codir	-0.59968254	0.58458647	0.71541842
ENSG0000026	RP11-264B17.	lncRNA	-0.59971921	0.53471933	0.67518277
ENSG0000014	ZNF287	protein_codir	-0.59995343	0.01384651	0.04476656
ENSG0000005	HEBP2	protein_codir	-0.60015521	0.00152761	0.00799403
ENSG0000018	DMWD	protein_codir	-0.60044912	0.00176669	0.00893206
ENSG0000028	RP1-179E13.2	lncRNA	-0.60061883	0.77030165	0.8554879
ENSG0000026	ZNF8-DT	lncRNA	-0.60081472	0.00653207	0.02496759
ENSG0000008	KIF3C	protein_codir	-0.60098486	0.00109827	0.00616677
ENSG0000028	RP11-121C6.6	protein_codir	-0.6010197	0.71093343	NA
ENSG0000017	MRGPRD	protein_codir	-0.60113983	0.61483275	0.73891222
ENSG0000014	CBLN2	protein_codir	-0.6012582	NA	NA
ENSG0000025	RP11-45P15.4	lncRNA	-0.60133081	0.19454829	0.33002234
ENSG0000011	RIC8B	protein_codir	-0.60137991	0.00042797	0.00287881
ENSG0000022	VIM-AS1	lncRNA	-0.60153723	0.03008535	0.0816425
ENSG0000020	PRAMEF14	protein_codir	-0.60157054	0.8537273	NA
ENSG0000022	RP1-15D23.2	lncRNA	-0.60157054	0.8537273	NA
ENSG0000018	LEMD1	protein_codir	-0.60157054	0.8537273	NA
ENSG0000014	TRIM43B	protein_codir	-0.60157054	0.8537273	NA
ENSG0000028	RP11-813C7.1	lncRNA	-0.60157054	0.8537273	NA
ENSG0000022	AC078883.4	lncRNA	-0.60157054	0.8537273	NA
ENSG0000022	RP11-136C24.	lncRNA	-0.60157054	0.8537273	NA
ENSG0000025	RP11-79E3.2	lncRNA	-0.60157054	0.8537273	NA
ENSG0000028	RP11-472A1.2	lncRNA	-0.60157054	0.8537273	NA
ENSG0000028	RP11-169I2.3	lncRNA	-0.60157054	0.8537273	NA
ENSG0000016	KCNV1	protein_codir	-0.60157054	0.8537273	NA
ENSG0000022	RP11-179B15.	lncRNA	-0.60157054	0.8537273	NA
ENSG0000020	RP11-25I9.3	lncRNA	-0.60157054	0.8537273	NA
ENSG0000028	RP11-89C3.5	lncRNA	-0.60157054	0.8537273	NA
ENSG0000025	LINC02293	lncRNA	-0.60157054	0.8537273	NA
ENSG0000025	RP11-275I4.1	lncRNA	-0.60157054	0.8537273	NA
ENSG0000025	RP11-352D13	lncRNA	-0.60157054	0.8537273	NA
ENSG0000023	AC004448.5	lncRNA	-0.60157054	0.8537273	NA
ENSG0000027	ELOA3CP	protein_codir	-0.60157054	0.8537273	NA
ENSG0000022	LINC00658	lncRNA	-0.60157054	0.8537273	NA

ENSG0000018	WFDC11	protein_codir	-0.60157054	0.8537273	NA
ENSG0000028	XX-15A10.1	lncRNA	-0.60157054	0.8537273	NA
ENSG0000010	ITIH6	protein_codir	-0.60157054	0.8537273	NA
ENSG0000028	ABC14-11888	lncRNA	-0.60157054	0.8537273	NA
ENSG0000014	C1orf94	protein_codir	-0.60157054	0.8537273	NA
ENSG0000024	RP11-315I20.1	lncRNA	-0.60157054	0.8537273	NA
ENSG0000028	RP11-414K7.2	lncRNA	-0.60157054	0.8537273	NA
ENSG0000023	FBXO36-IT1	lncRNA	-0.60157054	0.8537273	NA
ENSG0000028	RP11-190C21.	lncRNA	-0.60157054	0.8537273	NA
ENSG0000018	TMEM212	protein_codir	-0.60157054	0.8537273	NA
ENSG0000024	RP11-265O12	lncRNA	-0.60157054	0.8537273	NA
ENSG0000024	LINC01258	lncRNA	-0.60157054	0.8537273	NA
ENSG0000028	RP11-10L12.8	lncRNA	-0.60157054	0.8537273	NA
ENSG0000025	SLC7A11-AS1	lncRNA	-0.60157054	0.8537273	NA
ENSG0000028	CTD-2350M8.	lncRNA	-0.60157054	0.8537273	NA
ENSG0000025	LINC02199	lncRNA	-0.60157054	0.8537273	NA
ENSG0000025	CTD-2201E9.2	lncRNA	-0.60157054	0.8537273	NA
ENSG0000028	CTC-250P22.2	lncRNA	-0.60157054	0.8537273	NA
ENSG0000025	LINC01170	lncRNA	-0.60157054	0.8537273	NA
ENSG0000028	RP11-274H24	lncRNA	-0.60157054	0.8537273	NA
ENSG0000016	ADGRF2	protein_codir	-0.60157054	0.8537273	NA
ENSG0000027	RP11-95G17.2	lncRNA	-0.60157054	0.8537273	NA
ENSG0000028	RP11-346I3.6	protein_codir	-0.60157054	0.8537273	NA
ENSG0000025	RP11-219B4.5	lncRNA	-0.60157054	0.8537273	NA
ENSG0000025	LINC01298	lncRNA	-0.60157054	0.8537273	NA
ENSG0000028	RP11-290L7.6	lncRNA	-0.60157054	0.8537273	NA
ENSG0000028	RP11-157J13.	lncRNA	-0.60157054	0.8537273	NA
ENSG0000023	RP11-383C5.5	lncRNA	-0.60157054	0.8537273	NA
ENSG0000025	LINC00958	lncRNA	-0.60157054	0.8537273	NA
ENSG0000025	NAV2-AS4	lncRNA	-0.60157054	0.8537273	NA
ENSG0000025	RP11-91P24.5	lncRNA	-0.60157054	0.8537273	NA
ENSG0000025	SLC6A12-AS1	lncRNA	-0.60157054	0.8537273	NA
ENSG0000027	RP11-713N11	lncRNA	-0.60157054	0.8537273	NA
ENSG0000025	LINC02412	lncRNA	-0.60157054	0.8537273	NA
ENSG0000025	LINC02419	lncRNA	-0.60157054	0.8537273	NA
ENSG0000025	RP11-325D8.1	lncRNA	-0.60157054	0.8537273	NA
ENSG0000025	RP11-475A13	lncRNA	-0.60157054	0.8537273	NA
ENSG0000025	RP11-707P17.	lncRNA	-0.60157054	0.8537273	NA
ENSG0000026	RP11-326A19	lncRNA	-0.60157054	0.8537273	NA
ENSG0000026	RP11-626K17.	lncRNA	-0.60157054	0.8537273	NA
ENSG0000026	RP11-21B23.3	lncRNA	-0.60157054	0.8537273	NA
ENSG0000026	RP5-1142A6.7	lncRNA	-0.60157054	0.8537273	NA
ENSG0000026	RP11-19P22.8	lncRNA	-0.60157054	0.8537273	NA
ENSG0000028	RP11-443G13	lncRNA	-0.60157054	0.8537273	NA
ENSG0000017	DYNAP	protein_codir	-0.60157054	0.8537273	NA
ENSG0000023	LINC01722	lncRNA	-0.60157054	0.8537273	NA
ENSG0000023	ITCH-IT1	lncRNA	-0.60157054	0.8537273	NA

ENSG0000028	LINC01668	lncRNA	-0.60157054	0.8537273	NA
ENSG0000022	BX322557.13	lncRNA	-0.60157054	0.8537273	NA
ENSG0000014	AKAP4	protein_codir	-0.60157054	0.8537273	NA
ENSG0000026	OPN1MW	protein_codir	-0.60157054	0.8537273	NA
ENSG0000018	ATP4B	protein_codir	-0.60157081	0.85232558	NA
ENSG0000028	RP11-73G16.4	lncRNA	-0.60157082	0.85225737	NA
ENSG0000025	RP11-319E12.	lncRNA	-0.60157087	0.85199319	NA
ENSG0000027	GS1-304P7.3	lncRNA	-0.60157089	0.85189826	NA
ENSG0000027	RP11-255E6.6	lncRNA	-0.6015709	0.85184208	NA
ENSG0000022	KCNQ5-AS1	lncRNA	-0.60157091	0.85177879	NA
ENSG0000027	RP11-785G17	lncRNA	-0.60157092	0.8516943	NA
ENSG0000023	RP3-333A15.1	lncRNA	-0.60157103	0.85108942	NA
ENSG0000028	AC006538.9	lncRNA	-0.60157111	0.85066178	NA
ENSG0000018	ADGRD2	protein_codir	-0.60157122	0.85004958	NA
ENSG0000025	LINC01944	lncRNA	-0.60157123	0.85000741	NA
ENSG0000015	CDH12	protein_codir	-0.6015713	0.84961095	NA
ENSG0000027	RP11-681H18	lncRNA	-0.60157143	0.84885296	NA
ENSG0000013	GPR45	protein_codir	-0.60157145	0.84870697	NA
ENSG0000013	TTC6	protein_codir	-0.60157148	0.84856967	NA
ENSG0000028	RP4-592P3.1	lncRNA	-0.60157152	0.84830888	NA
ENSG0000014	A1CF	protein_codir	-0.60157164	0.84759006	NA
ENSG0000025	RP11-616M22	lncRNA	-0.60157164	0.84759006	NA
ENSG0000026	RP11-344B2.3	lncRNA	-0.60157164	0.84759006	NA
ENSG0000025	RP11-221N13	lncRNA	-0.60157165	0.84756716	NA
ENSG0000025	LINC01299	lncRNA	-0.60157171	0.84718219	NA
ENSG0000028	RP1-294H2.1	lncRNA	-0.60157171	0.84718051	NA
ENSG0000023	AC004012.1	lncRNA	-0.60157174	0.84698419	NA
ENSG0000022	RP11-342D14	lncRNA	-0.6015719	0.84603456	NA
ENSG0000023	RP11-80H5.2	lncRNA	-0.60157192	0.84590339	NA
ENSG0000024	RP11-266N13	lncRNA	-0.60157194	0.84576348	NA
ENSG0000023	LINC00479	lncRNA	-0.60157194	0.84576348	NA
ENSG0000025	RP11-265D17	lncRNA	-0.60157218	0.84427751	NA
ENSG0000023	VPS13A-AS1	lncRNA	-0.60157218	0.84427532	NA
ENSG0000016	PWWP3A	protein_codir	-0.60172303	0.00053868	0.00347788
ENSG0000013	PLXNA3	protein_codir	-0.60182175	0.00120439	0.00663165
ENSG0000014	HAPLN3	protein_codir	-0.60198269	0.01241704	0.04113692
ENSG0000015	RNF20	protein_codir	-0.60200047	0.00017419	0.00138259
ENSG0000026	RP11-599B13.	protein_codir	-0.60201351	0.02381528	0.06791965
ENSG0000015	SCOC	protein_codir	-0.60231342	0.00202007	0.00989789
ENSG0000028	RP11-380J17.	lncRNA	-0.60233299	0.40983735	0.56285277
ENSG0000005	ATP2B4	protein_codir	-0.60240943	0.05987254	0.13772304
ENSG0000014	EXOC6B	protein_codir	-0.60241445	0.00853865	0.03071831
ENSG0000027	RP11-546J1.1	lncRNA	-0.60286749	0.51979989	0.66261472
ENSG0000025	MTRNR2L8	protein_codir	-0.60304091	0.40498922	0.55789538
ENSG0000013	MMD2	protein_codir	-0.60309185	0.79025147	NA
ENSG0000013	TPGS2	protein_codir	-0.60340116	0.00271322	0.01246908
ENSG0000025	RP11-173P15.	lncRNA	-0.60362573	0.22344147	0.36497633

ENSG000001З	MEIS2	protein_codir	-0.60365417	0.11196037	0.21951768
ENSG0000015	CNKSR3	protein_codir	-0.60384521	0.13030834	0.2455729
ENSG0000019	RP11-544M22	lncRNA	-0.60393932	0.83864279	NA
ENSG0000012	DYNLRB1	protein_codir	-0.60416874	0.0005299	0.00342762
ENSG0000015	DZIP1L	protein_codir	-0.6045241	0.00529354	0.02121411
ENSG0000010	FSCN3	protein_codir	-0.60454295	0.27456802	0.42264776
ENSG0000028	RP11-803J6.1	protein_codir	-0.60455825	0.17417867	0.30417394
ENSG0000010	CRYBB3	protein_codir	-0.60472942	0.42096867	0.57301205
ENSG0000014	COL6A1	protein_codir	-0.60478145	0.03410672	0.08984527
ENSG0000027	RP11-547D13	lncRNA	-0.60480974	0.02097986	0.0615338
ENSG0000015	MKX	protein_codir	-0.60490793	0.32481832	0.4771811
ENSG0000025	RP11-46C24.7	lncRNA	-0.60506058	0.01657437	0.05142485
ENSG0000015	GAREM2	protein_codir	-0.60514227	0.00086227	0.00508669
ENSG0000010	MID1	protein_codir	-0.60526713	0.14918146	0.27138193
ENSG0000022	AC003088.1	lncRNA	-0.60529055	0.7536822	NA
ENSG0000012	C4BPA	protein_codir	-0.60553295	0.56122737	0.69608424
ENSG0000018	FKRP	protein_codir	-0.60563612	0.00010485	0.00090333
ENSG0000022	SLC26A6	protein_codir	-0.60573593	0.05264818	0.12510908
ENSG0000003	FAM13B	protein_codir	-0.60578076	0.0262859	0.07337949
ENSG0000028	RP11-472B18.	lncRNA	-0.60578994	0.11901096	0.22959298
ENSG0000020	SNHG5	lncRNA	-0.60579472	0.01787054	0.05436549
ENSG0000018	ZNF322	protein_codir	-0.60582006	0.00417801	0.01765013
ENSG0000025	RP11-161H23	lncRNA	-0.6060043	0.22963428	0.372099
ENSG0000017	HOXB2	protein_codir	-0.60609075	0.01185437	0.0397207
ENSG0000019	FAM47C	protein_codir	-0.60611887	0.79258561	NA
ENSG0000025	LINGO1-AS1	lncRNA	-0.60618928	0.82888523	NA
ENSG0000027	CTD-2574D22	lncRNA	-0.60634295	0.14563038	0.26667846
ENSG0000016	LYSMD1	protein_codir	-0.6063882	0.02556864	0.07187115
ENSG0000023	RP11-97N19.2	lncRNA	-0.60641851	0.78706189	NA
ENSG0000020	SERF1B	protein_codir	-0.60660604	0.00485858	0.01983946
ENSG0000016	CNPY4	protein_codir	-0.60687244	0.00092327	0.00537422
ENSG0000027	RP11-672L10.	lncRNA	-0.60703609	0.48073117	0.62840597
ENSG0000010	APBA1	protein_codir	-0.60747179	0.00471245	0.01937686
ENSG0000027	GTF2H5	protein_codir	-0.60749115	0.02855801	0.07843017
ENSG0000016	LCMT2	protein_codir	-0.60755552	0.00065399	0.00407009
ENSG0000008	NOX4	protein_codir	-0.60763728	0.14565444	0.26670081
ENSG0000008	COL4A4	protein_codir	-0.60819793	0.06283476	0.14315731
ENSG0000018	PSMG1	protein_codir	-0.60839831	5.97E-05	0.00057006
ENSG0000017	ZBTB4	protein_codir	-0.60840718	0.00409342	0.01736184
ENSG0000027	H4C2	protein_codir	-0.60854201	0.29988648	0.45091122
ENSG0000027	RP11-370I10.1	lncRNA	-0.60854556	0.7515546	NA
ENSG0000020	RNASE13	protein_codir	-0.60882306	0.53299266	0.67383427
ENSG0000026	LA16c-313D11	lncRNA	-0.60883239	0.01384887	0.04476893
ENSG0000012	NRL	protein_codir	-0.60894989	0.0001742	0.00138259
ENSG0000014	GAS8	protein_codir	-0.60898878	0.00276428	0.0126511
ENSG0000014	CGN	protein_codir	-0.60900568	0.12008608	0.2311021
ENSG0000018	LRRC75A	protein_codir	-0.60900689	0.00507243	0.02051144

ENSG0000027	CTD-3064M3.	lncRNA	-0.60918171	0.09863753	0.19964594
ENSG0000023	TTLL11-IT1	lncRNA	-0.60943726	0.48927576	0.63621743
ENSG0000028	RP11-734J23.	lncRNA	-0.6095874	0.65479774	0.77018626
ENSG0000017	NAP1L5	protein_codir	-0.60973382	0.08478949	0.17822571
ENSG0000021	C8orf82	protein_codir	-0.60975118	0.01933998	0.05784193
ENSG0000000	TSR3	protein_codir	-0.60978345	2.59E-06	4.07E-05
ENSG0000017	GADD45GIP1	protein_codir	-0.60997359	8.89E-06	0.00011657
ENSG0000019	PJA2	protein_codir	-0.61010894	0.03607388	0.0936429
ENSG0000014	MPC2	protein_codir	-0.610705	0.00016896	0.0013511
ENSG0000022	RP3-508I15.9	lncRNA	-0.61073816	0.03056181	0.0826425
ENSG0000019	ARMCX4	protein_codir	-0.61102698	0.00368054	0.01588382
ENSG0000027	RP11-248G5.9	lncRNA	-0.61129847	0.71395378	NA
ENSG0000018	PHLDA2	protein_codir	-0.61169597	0.09159005	0.18872854
ENSG0000023	C1orf226	protein_codir	-0.61181295	0.15878792	0.28410457
ENSG0000013	LRRC32	protein_codir	-0.61191739	0.06727003	0.15079432
ENSG0000018	CUEDC1	protein_codir	-0.61194693	0.01045057	0.03605356
ENSG0000005	FOXN3	protein_codir	-0.61195272	0.05432557	0.12808129
ENSG0000014	MINDY3	protein_codir	-0.61209443	0.00135206	0.00726931
ENSG0000026	CTC-429L19.3	lncRNA	-0.61212699	0.64886578	0.76500077
ENSG0000025	DCHS1-AS1	lncRNA	-0.61238552	0.37900822	0.53240805
ENSG0000023	UNC5B-AS1	lncRNA	-0.61255386	0.17835419	0.30933392
ENSG0000017	FAM89B	protein_codir	-0.61286103	0.00022418	0.001695
ENSG0000010	ST8SIA5	protein_codir	-0.61289431	0.25849206	0.40554107
ENSG0000027	CTD-2270P14	lncRNA	-0.61293461	0.08895772	0.1847012
ENSG0000017	ZDHHC22	protein_codir	-0.61313153	0.63457355	0.75459256
ENSG0000015	HLCS	protein_codir	-0.61353772	0.00158845	0.00825147
ENSG0000014	BTBD10	protein_codir	-0.61363265	0.00057609	0.00366798
ENSG0000010	MSLN	protein_codir	-0.6136406	0.46322561	0.61316454
ENSG0000028	RP11-606E8.2	protein_codir	-0.61376109	0.51337695	0.65685234
ENSG0000017	MAMSTR	protein_codir	-0.61379719	0.02889358	0.07917857
ENSG0000015	DGKI	protein_codir	-0.61380487	0.31204374	0.46379208
ENSG0000008	ZNF213	protein_codir	-0.6138899	0.00015071	0.00123082
ENSG0000027	RP11-295D4.5	lncRNA	-0.61411123	0.52413345	0.66617117
ENSG0000016	PRRT3	protein_codir	-0.61419944	5.90E-06	8.22E-05
ENSG0000027	RP11-823E8.3	lncRNA	-0.61433429	0.02850612	0.07831882
ENSG0000025	RP11-571M6.	lncRNA	-0.61452923	0.34650813	0.49955917
ENSG0000028	RP11-1228E1.	lncRNA	-0.61488397	0.3926749	0.54570341
ENSG0000027	RP11-158I9.8	lncRNA	-0.61502696	0.0868843	0.18153751
ENSG0000012	TRIP10	protein_codir	-0.61544155	0.02731286	0.07561148
ENSG0000019	INPP5F	protein_codir	-0.61556967	0.00018585	0.00145436
ENSG0000000	ST7	protein_codir	-0.61593614	0.01751557	0.05356864
ENSG0000019	SLC2A10	protein_codir	-0.61606153	0.02649044	0.07379384
ENSG0000013	SHF	protein_codir	-0.6163997	0.01024668	0.03550091
ENSG0000018	SNRNP35	protein_codir	-0.61642874	0.00025184	0.00186682
ENSG0000016	SNX7	protein_codir	-0.61665704	0.0514121	0.12293327
ENSG0000026	RP1-223E5.4	lncRNA	-0.61675156	0.1043657	0.20829942
ENSG0000027	XXbac-B444P2	lncRNA	-0.61683389	0.2343843	0.37776123

ENSG0000023	RP1-102D24.5	lncRNA	-0.61708377	0.69475025	0.79984168
ENSG0000014	ZNF157	protein_codir	-0.61728366	0.20222725	0.33929757
ENSG0000026	RP13-582O9.6	protein_codir	-0.61734734	0.30000103	0.45096487
ENSG0000012	CHPF	protein_codir	-0.61775933	0.04828031	0.11725156
ENSG0000027	CTD-3220F14.	lncRNA	-0.61785711	0.27469987	0.42273292
ENSG0000016	CA5B	protein_codir	-0.61791725	0.04309931	0.10751967
ENSG0000010	CDS2	protein_codir	-0.61812784	0.02018412	0.05971991
ENSG0000010	MEDAG	protein_codir	-0.61846263	0.25917167	0.4062381
ENSG0000023	ITGA9-AS1	lncRNA	-0.6184811	0.0857064	0.17972901
ENSG0000016	SEPTIN8	protein_codir	-0.61854737	0.00361795	0.01567989
ENSG0000014	ERLIN2	protein_codir	-0.61927998	0.00266992	0.01231515
ENSG0000028	RP4-613B23.5	protein_codir	-0.61929102	0.04720428	0.11517512
ENSG0000018	C9orf152	protein_codir	-0.61948574	0.53922733	0.67849001
ENSG0000022	AP4M1	protein_codir	-0.61949541	7.31E-05	0.00067268
ENSG0000015	CATSPER3	protein_codir	-0.61959938	0.09354808	0.19194695
ENSG0000016	ARHGAP35	protein_codir	-0.61968391	0.00403845	0.01716029
ENSG0000023	AC002066.1	lncRNA	-0.61974026	0.32663532	0.47893334
ENSG0000015	EXT2	protein_codir	-0.61982659	0.00081248	0.00485202
ENSG0000022	P3H2-AS1	lncRNA	-0.62006885	0.74236813	NA
ENSG0000011	C3orf14	protein_codir	-0.62025788	0.00115655	0.0064209
ENSG0000019	ATL1	protein_codir	-0.6203137	0.00754647	0.02790984
ENSG0000016	SMPD1	protein_codir	-0.62036678	4.40E-06	6.36E-05
ENSG0000016	PRMT2	protein_codir	-0.62038428	0.00021037	0.00161042
ENSG0000018	HS6ST3	protein_codir	-0.62038471	0.49386629	0.64010262
ENSG0000014	CAMKMT	protein_codir	-0.62098799	0.00206072	0.01005069
ENSG0000017	ADAMTSL1	protein_codir	-0.6210237	0.06391551	0.14514071
ENSG0000016	CARD19	protein_codir	-0.62109775	0.00038956	0.00267989
ENSG0000028	CTD-2297M2.	lncRNA	-0.62129938	0.6842752	0.79250591
ENSG0000027	CTC-336P14.1	lncRNA	-0.62130309	0.58712362	0.71744257
ENSG0000026	RP11-5A19.5	protein_codir	-0.62139382	0.07394536	0.16129923
ENSG0000000	SARM1	protein_codir	-0.62143692	1.61E-08	5.24E-07
ENSG0000003	VCAN	protein_codir	-0.62188269	0.19812359	0.33438097
ENSG0000027	RP11-350N15	lncRNA	-0.62204588	0.14674451	0.26817601
ENSG0000028	RP11-251I17.	lncRNA	-0.6224626	0.70548885	NA
ENSG0000016	IGSF8	protein_codir	-0.62269606	1.06E-05	0.00013417
ENSG0000011	EIF2B2	protein_codir	-0.6227745	4.22E-06	6.15E-05
ENSG0000012	PEX6	protein_codir	-0.62291895	0.00022561	0.00170258
ENSG0000003	TIMP2	protein_codir	-0.62295423	0.06876062	0.15292441
ENSG0000012	EMC1	protein_codir	-0.62316076	0.00052795	0.0034182
ENSG0000027	PCCA-DT	lncRNA	-0.62322827	0.01000907	0.03482623
ENSG0000019	ZNF502	protein_codir	-0.62333916	0.0013757	0.00736538
ENSG0000028	RP4-621F18.2	lncRNA	-0.62345377	0.1819469	0.3140662
ENSG0000017	TTTY14	lncRNA	-0.62362319	0.21168723	0.35120142
ENSG0000012	MAP7D3	protein_codir	-0.62364423	0.03485969	0.09125436
ENSG0000017	WDR25	protein_codir	-0.62380402	0.00013969	0.0011552
ENSG0000027	USP27X	protein_codir	-0.62381972	0.00708802	0.02656325
ENSG0000010	EMD	protein_codir	-0.6242648	0.00060928	0.00384152

ENSG0000017	LRRC75B	protein_codir	-0.62447719	0.03796781	0.09739676
ENSG0000010	TTC23	protein_codir	-0.62454397	0.000161	0.00130059
ENSG0000005	DGKG	protein_codir	-0.62458383	0.06838274	0.15239296
ENSG0000016	REEP3	protein_codir	-0.62469771	0.03910355	0.09972502
ENSG0000022	DNAJC27-AS1	lncRNA	-0.62498675	0.05893031	0.13601195
ENSG0000016	KLHDC9	protein_codir	-0.62516111	0.0584889	0.13527527
ENSG0000010	BRMS1L	protein_codir	-0.62589329	0.01684409	0.05204893
ENSG0000012	WFIKKN1	protein_codir	-0.62603429	0.0831746	0.1755527
ENSG0000016	SLC4A2	protein_codir	-0.62622244	0.00012359	0.00104197
ENSG0000016	SPTBN4	protein_codir	-0.62626554	0.08334081	0.17579216
ENSG0000007	UBE2D4	protein_codir	-0.62652736	0.00189029	0.00939019
ENSG0000014	SSBP2	protein_codir	-0.6266241	0.00107383	0.00606033
ENSG0000010	UFSP2	protein_codir	-0.62679655	0.00482901	0.01975006
ENSG0000011	TNS2	protein_codir	-0.62686527	0.05931935	0.13671387
ENSG0000012	TUBA1B	protein_codir	-0.62713652	0.01294918	0.04249738
ENSG0000012	HIF3A	protein_codir	-0.62745069	0.21725904	0.35750341
ENSG0000018	ZNF793	protein_codir	-0.62765143	0.00712116	0.02666213
ENSG0000011	DCAF4	protein_codir	-0.62770042	0.00201982	0.00989789
ENSG0000016	FAM234A	protein_codir	-0.62788605	0.00046425	0.00307715
ENSG0000018	CCDC149	protein_codir	-0.62811459	0.02424461	0.06887842
ENSG0000026	CTD-2207O23	protein_codir	-0.62816165	0.63545152	0.7552139
ENSG0000018	LRTOMT	protein_codir	-0.62843161	0.00022912	0.00172483
ENSG0000012	RASL11A	protein_codir	-0.62870994	0.05839293	0.13511039
ENSG0000002	CPS1	protein_codir	-0.6287255	0.04265527	0.10674776
ENSG0000026	ZNF582-DT	lncRNA	-0.62894281	0.00841581	0.03035714
ENSG0000027	GOLGA4-AS1	lncRNA	-0.62897378	0.01343508	0.04373941
ENSG0000001	NUDCD3	protein_codir	-0.62917127	0.00012608	0.00105909
ENSG0000014	TNKS1BP1	protein_codir	-0.62926502	0.0035047	0.01529708
ENSG0000025	INAFM1	protein_codir	-0.62939959	0.01427743	0.04586978
ENSG0000026	ZNF561-AS1	lncRNA	-0.6294404	0.00056552	0.00361234
ENSG0000026	ZNF470-DT	lncRNA	-0.62958039	0.05295471	0.12556741
ENSG0000018	KLHDC8B	protein_codir	-0.62981569	0.00171422	0.00873879
ENSG0000028	RP11-481H12	lncRNA	-0.63037045	0.29796476	0.44876388
ENSG0000014	CDC42BPA	protein_codir	-0.63065911	0.01614685	0.05047832
ENSG0000026	PTOV1-AS1	lncRNA	-0.63125882	0.06039379	0.13867126
ENSG0000022	TMLHE-AS1	lncRNA	-0.63172911	0.43028211	0.58177786
ENSG0000018	C8orf33	protein_codir	-0.63188072	0.00064076	0.00400855
ENSG0000006	TLE2	protein_codir	-0.63210566	0.08865867	0.18422753
ENSG0000026	CFAP298-TCP	protein_codir	-0.63230039	0.00828919	0.03002197
ENSG0000027	RP11-120E11.	lncRNA	-0.63237886	0.34185088	0.49506279
ENSG0000011	C3orf52	protein_codir	-0.63259326	0.1277171	0.24193673
ENSG0000026	RP11-1055B8.	lncRNA	-0.63289394	0.7446308	NA
ENSG0000024	CRYBB2	protein_codir	-0.63310884	0.35544897	0.50907444
ENSG0000015	CCNO	protein_codir	-0.63325036	0.22992663	0.37232589
ENSG0000027	AP001437.1	lncRNA	-0.63328771	0.27286819	0.42075812
ENSG0000027	RP11-90L1.8	lncRNA	-0.63329129	0.19391602	0.32935435
ENSG0000018	EZHIP	protein_codir	-0.63340838	0.52676045	0.66855637

ENSG0000018	TRDN	protein_codir	-0.63354263	NA	NA
ENSG0000026	RP11-477H21	lncRNA	-0.63361451	0.14948857	0.27170797
ENSG0000010	TAF6	protein_codir	-0.63374747	2.00E-07	4.52E-06
ENSG0000018	PRKRA	protein_codir	-0.63377277	0.00244529	0.01148688
ENSG0000016	MOCS2	protein_codir	-0.63382501	0.00146973	0.00776769
ENSG0000016	SPACA9	protein_codir	-0.6338603	0.00168403	0.00863108
ENSG0000007	PLXNA2	protein_codir	-0.63407889	0.00788869	0.02889345
ENSG0000013	PDLIM4	protein_codir	-0.63412732	0.07832371	0.16799726
ENSG0000016	LDHD	protein_codir	-0.63420728	0.00623045	0.02404092
ENSG0000011	GAPDH	protein_codir	-0.63447929	0.0002083	0.00159721
ENSG0000017	KLF17	protein_codir	-0.63472118	0.30265676	0.45404268
ENSG0000017	PHETA2	protein_codir	-0.63499684	8.91E-05	0.00079169
ENSG0000016	ICA1L	protein_codir	-0.63575127	0.02748248	0.0759674
ENSG0000021	SYNJ2BP	protein_codir	-0.63590456	0.00231604	0.01103486
ENSG0000027	RP11-139H15	lncRNA	-0.63606172	NA	NA
ENSG0000011	COPS7A	protein_codir	-0.63633796	4.15E-05	0.00041803
ENSG0000024	RP11-434D9.1	lncRNA	-0.63664429	0.3147598	0.4665834
ENSG0000023	SPDYE21	protein_codir	-0.63666319	0.7860254	NA
ENSG0000026	LA16c-390H2.	lncRNA	-0.63671453	0.31290628	0.46462402
ENSG0000010	NDUFC1	protein_codir	-0.63672729	1.09E-05	0.00013764
ENSG0000025	PPFIA2-AS1	lncRNA	-0.63673501	0.81681463	NA
ENSG0000012	AMOT	protein_codir	-0.63678181	0.10963397	0.2161532
ENSG0000023	PKN2-AS1	lncRNA	-0.63686025	0.40436562	0.55736992
ENSG0000014	SIGMAR1	protein_codir	-0.63695799	0.0008039	0.00480807
ENSG0000013	HRC	protein_codir	-0.6370336	0.21588864	0.35613953
ENSG0000018	SHOX	protein_codir	-0.63729309	0.69378082	NA
ENSG0000028	RP11-77I22.5	lncRNA	-0.63735934	0.6960005	NA
ENSG0000027	ANKH-DT	lncRNA	-0.63756117	0.67522253	0.78607572
ENSG0000013	ACSBG2	protein_codir	-0.63788097	0.57048895	0.70399497
ENSG0000016	SLC38A8	protein_codir	-0.63857582	0.73878809	NA
ENSG0000015	NRGN	protein_codir	-0.63863208	0.13528114	0.25229339
ENSG0000011	IFT46	protein_codir	-0.63869352	5.37E-06	7.56E-05
ENSG0000017	FAM20C	protein_codir	-0.63874776	0.01801767	0.05474073
ENSG0000022	SMCR5	lncRNA	-0.63925403	0.52161745	0.66416531
ENSG0000020	LOH12CR2	lncRNA	-0.63939907	0.10538713	0.20983222
ENSG0000000	PON1	protein_codir	-0.63965424	0.78147191	NA
ENSG0000022	C19orf73	protein_codir	-0.63969846	0.15852651	0.28374715
ENSG0000015	TSR2	protein_codir	-0.63984698	0.00175819	0.00890049
ENSG0000025	AP006621.8	lncRNA	-0.6400565	0.18018183	0.31170126
ENSG0000017	TMEM81	protein_codir	-0.64016668	0.01525592	0.04832761
ENSG0000013	TRIM5	protein_codir	-0.64022509	2.09E-05	0.00023805
ENSG0000026	RP11-541N10	lncRNA	-0.64030128	0.13531881	0.25234661
ENSG0000017	NUDT4B	protein_codir	-0.64044635	0.00151176	0.00793364
ENSG0000028	RP11-47G4.3	lncRNA	-0.64051486	0.8212434	NA
ENSG0000025	RP11-867G2.8	lncRNA	-0.64057231	0.62813535	NA
ENSG0000013	TUBB2A	protein_codir	-0.64108464	0.0946567	0.19361757
ENSG0000027	RP11-1293J14	lncRNA	-0.64208367	0.59098433	0.72031187

ENSG0000028	RP13-512J5.2	lncRNA	-0.64213971	0.0124264	0.04115806
ENSG0000013	TAF5L	protein_codir	-0.64225573	0.00577367	0.02265182
ENSG0000016	ANXA5	protein_codir	-0.64251673	0.00698359	0.02630395
ENSG0000023	RP11-269F20.	lncRNA	-0.64253417	0.69174877	NA
ENSG0000022	STEAP3-AS1	lncRNA	-0.6427152	0.21016094	0.34933831
ENSG0000014	NME7	protein_codir	-0.64293065	9.42E-05	0.00082716
ENSG0000021	SLC23A3	protein_codir	-0.64320475	0.1994469	0.33595922
ENSG0000018	MAGEH1	protein_codir	-0.64374495	1.01E-05	0.00012965
ENSG0000027	RP11-793H13	lncRNA	-0.64415389	0.05112556	0.12247058
ENSG0000026	L34079.4	lncRNA	-0.64426895	0.73417885	NA
ENSG0000025	RP11-111K18.	protein_codir	-0.64482761	0.00041131	0.00279536
ENSG0000019	ZNF846	protein_codir	-0.64487702	0.00047134	0.00311667
ENSG0000021	LCAT	protein_codir	-0.64489327	0.05761055	0.1336921
ENSG0000010	PITRM1	protein_codir	-0.64513688	0.00021211	0.00161926
ENSG0000025	EID1	protein_codir	-0.64527036	0.01072285	0.0368044
ENSG0000018	PEAR1	protein_codir	-0.64531359	0.078562	0.16838557
ENSG0000017	KIAA0232	protein_codir	-0.6453297	0.00244979	0.01150147
ENSG0000016	CFAP221	protein_codir	-0.64535349	0.06815384	0.15210157
ENSG0000010	MINDY4	protein_codir	-0.64616038	0.03652258	0.09456504
ENSG0000012	ZNF205	protein_codir	-0.64628375	0.00564913	0.02228031
ENSG0000016	APBB2	protein_codir	-0.64647377	0.02746054	0.07591917
ENSG0000025	CTD-2184D3.6	lncRNA	-0.64648871	0.67050878	0.78257584
ENSG0000010	SMS	protein_codir	-0.6468592	0.00034357	0.00241277
ENSG0000022	RP11-374M1.	lncRNA	-0.64695277	0.67192336	0.78362214
ENSG0000016	MON1A	protein_codir	-0.64716456	3.09E-05	0.00032803
ENSG0000019	ZNF785	protein_codir	-0.64719195	0.00046144	0.00306338
ENSG0000027	CTD-2540B15	lncRNA	-0.64723047	0.03085923	0.0831868
ENSG0000023	GRM3-AS1	lncRNA	-0.64726069	0.24657473	0.39185237
ENSG0000027	RP11-573G6.9	lncRNA	-0.64742174	0.48074649	0.62840597
ENSG0000017	IL17D	protein_codir	-0.64743311	0.14954884	0.27175105
ENSG0000013	DYNC1LI2	protein_codir	-0.64774367	0.0009663	0.00557417
ENSG0000007	DDX1	protein_codir	-0.64781775	0.00085955	0.0050739
ENSG0000027	HMGN3-AS1	lncRNA	-0.64787014	0.04564693	0.11238533
ENSG0000016	TRIM46	protein_codir	-0.64788479	0.05082836	0.12203857
ENSG0000022	RP11-310H4.1	lncRNA	-0.64816538	0.69847435	NA
ENSG0000025	RP11-793H13	lncRNA	-0.64821178	0.36617363	0.52009235
ENSG0000006	MTFR1	protein_codir	-0.64824044	0.00592697	0.0231186
ENSG0000023	RP4-613B23.1	lncRNA	-0.64828532	0.19459354	0.33005939
ENSG0000026	CTD-2288F12.	lncRNA	-0.64864139	0.74704445	0.83844803
ENSG0000016	SPATA18	protein_codir	-0.64865833	0.04476493	0.11070982
ENSG0000010	HDAC5	protein_codir	-0.64870961	0.00864354	0.03102059
ENSG0000017	CHRNA7	protein_codir	-0.64891998	0.05675327	0.13227599
ENSG0000014	GCSH	protein_codir	-0.64910921	0.00654319	0.0249928
ENSG0000018	PCDH9	protein_codir	-0.64942546	0.07436681	0.16194996
ENSG0000028	CTC-483I11.5	lncRNA	-0.64948784	0.45847931	0.60881011
ENSG0000022	LINC02607	lncRNA	-0.65004943	0.53088396	0.67215288
ENSG0000016	XIRP2	protein_codir	-0.65016291	0.66385163	0.7774915

ENSG0000015	CYB5R1	protein_codir	-0.65020483	0.0003287	0.00232309
ENSG0000006	SNAP91	protein_codir	-0.65043197	0.20911348	0.34803944
ENSG0000008	PIR	protein_codir	-0.65064552	0.00673257	0.02551155
ENSG0000004	AIFM2	protein_codir	-0.65067384	1.50E-05	0.00018081
ENSG0000018	KCTD21	protein_codir	-0.65067757	0.00264282	0.01220852
ENSG0000003	EFCAB1	protein_codir	-0.65069816	0.38487975	0.53811079
ENSG0000004	CTNNA1	protein_codir	-0.65076063	0.01179641	0.03959373
ENSG0000024	RP11-159H10	lncRNA	-0.65101699	0.19999978	0.33658261
ENSG0000014	GPR161	protein_codir	-0.65118626	0.00864874	0.03103525
ENSG0000001	LRRC23	protein_codir	-0.65136485	1.41E-05	0.00017163
ENSG0000010	MEGF8	protein_codir	-0.65140385	1.49E-06	2.60E-05
ENSG0000010	WASL	protein_codir	-0.65149009	0.03709336	0.09572258
ENSG0000027	RP11-817O13	lncRNA	-0.65170425	0.21623829	0.35656726
ENSG0000025	AC083843.1	lncRNA	-0.65231088	0.09443877	0.19331497
ENSG0000016	PDLIM5	protein_codir	-0.65232487	0.0743338	0.16191027
ENSG0000028	KB-1683C8.2	lncRNA	-0.65241705	0.64746741	NA
ENSG0000009	GOLGA3	protein_codir	-0.65265977	8.52E-05	0.00076394
ENSG0000018	ZBTB7C	protein_codir	-0.65296205	0.02435003	0.06914237
ENSG0000011	FAM20B	protein_codir	-0.65318402	0.01025542	0.03551783
ENSG0000016	PGM2L1	protein_codir	-0.65321923	0.05558209	0.13024327
ENSG0000016	BSND	protein_codir	-0.65333214	0.73131541	NA
ENSG0000023	SMAD9-IT1	lncRNA	-0.65347676	0.59086	0.72026017
ENSG0000010	NUTF2	protein_codir	-0.65376084	0.00062141	0.00390606
ENSG0000017	LRRC20	protein_codir	-0.65377334	7.18E-08	1.91E-06
ENSG0000007	TEAD2	protein_codir	-0.65399835	0.01374285	0.04452523
ENSG0000011	SKP1	protein_codir	-0.65414481	0.00329562	0.014571
ENSG0000028	RP5-1104E15.	protein_codir	-0.65424612	0.5095348	0.65375348
ENSG0000027	CTD-3001H11	lncRNA	-0.65436389	0.21315761	0.35285664
ENSG0000026	AC006116.15	lncRNA	-0.65465253	0.57925213	NA
ENSG0000014	TIGD7	protein_codir	-0.6547921	0.00547736	0.02178279
ENSG0000011	PPFIBP1	protein_codir	-0.65501871	0.05377052	0.12710902
ENSG0000018	C11orf54	protein_codir	-0.65508545	0.00056229	0.00359667
ENSG0000023	RP4-650F12.2	lncRNA	-0.65511024	0.63878524	0.75790405
ENSG0000025	RP11-754B17.	lncRNA	-0.6551798	0.26086395	0.4080801
ENSG0000024	PRICKLE2-AS1	lncRNA	-0.65550138	0.73741806	NA
ENSG0000026	AC010761.8	lncRNA	-0.65580811	0.16506509	0.2923623
ENSG0000022	RP13-43E11.1	lncRNA	-0.65624592	0.68805929	NA
ENSG0000019	SMG5	protein_codir	-0.65637206	3.23E-08	9.50E-07
ENSG0000027	CISD3	protein_codir	-0.65646188	8.82E-05	0.00078469
ENSG0000026	CTC-273B12.5	lncRNA	-0.65662203	0.58672475	0.71705034
ENSG0000018	TMEM220	protein_codir	-0.65674411	0.01991013	0.05909917
ENSG0000018	PURA	protein_codir	-0.65681524	0.00235219	0.01115956
ENSG0000018	IMMP2L	protein_codir	-0.6569259	0.00327076	0.01448425
ENSG0000023	VLDLR-AS1	lncRNA	-0.6569587	0.29638254	0.44688752
ENSG0000023	RP11-111F5.3	lncRNA	-0.65715338	0.56252009	0.69724924
ENSG0000015	SEMA3D	protein_codir	-0.657643	0.20276644	0.33989252
ENSG0000023	CTD-2643I7.5	protein_codir	-0.65838255	0.18157804	0.31356668

ENSG0000027	RP3-453C12.1	lncRNA	-0.65843037	0.40565164	0.55861286
ENSG0000008	MAST2	protein_codir	-0.65851147	0.0014984	0.0078781
ENSG0000011	ATN1	protein_codir	-0.65862319	0.00972883	0.03404413
ENSG0000026	FRRS1L	protein_codir	-0.65907656	NA	NA
ENSG0000016	PIP5KL1	protein_codir	-0.65909173	0.05285685	0.12541073
ENSG0000012	TUBA4A	protein_codir	-0.65913447	0.00820819	0.02981851
ENSG0000018	HDHD5-AS1	lncRNA	-0.65917374	0.40605718	0.55905983
ENSG0000027	LINC00624	lncRNA	-0.65922069	0.76787399	NA
ENSG0000023	CLEC12A-AS1	lncRNA	-0.65922785	0.76797941	NA
ENSG0000013	RNF185	protein_codir	-0.65925628	0.00224145	0.01075178
ENSG0000017	SETMAR	protein_codir	-0.65992252	0.01263222	0.04167484
ENSG0000012	GIPC1	protein_codir	-0.65994145	3.30E-05	0.00034594
ENSG0000016	ANTXR1	protein_codir	-0.66000188	0.08545494	0.17937874
ENSG0000027	RP11-188P17.	lncRNA	-0.66042337	0.29853794	0.44945044
ENSG0000020	LRRC73	protein_codir	-0.6605449	0.01782881	0.05430164
ENSG0000015	HK1	protein_codir	-0.66058427	0.00173448	0.0088144
ENSG0000018	CDIN1	protein_codir	-0.66072641	0.04553232	0.11221581
ENSG0000018	KAZN	protein_codir	-0.66073413	0.01751939	0.05356864
ENSG0000017	MRFAP1	protein_codir	-0.66140941	0.00173852	0.00882847
ENSG0000025	RP11-630C16.	lncRNA	-0.66160558	0.58143182	0.71283489
ENSG0000012	OCRL	protein_codir	-0.66162946	0.00837406	0.03025256
ENSG0000027	RP11-225B17.	lncRNA	-0.66166172	0.03528259	0.09212566
ENSG0000018	PCYT2	protein_codir	-0.66182998	0.00330616	0.01460823
ENSG0000014	SPAG16	protein_codir	-0.66218781	0.00152476	0.00798367
ENSG0000017	GOLGA6L2	protein_codir	-0.66228408	0.81896396	NA
ENSG0000027	RP11-354E23.	lncRNA	-0.66234026	0.55122928	0.68822678
ENSG0000027	RP3-329A5.8	lncRNA	-0.6624653	0.01974331	0.05871758
ENSG0000013	TCEAL4	protein_codir	-0.66258255	0.09016192	0.18643948
ENSG0000010	SHB	protein_codir	-0.66259903	0.0866389	0.18124414
ENSG0000018	TP53TG1	lncRNA	-0.66304662	2.38E-08	7.30E-07
ENSG0000019	SLC6A9	protein_codir	-0.66325735	0.04448126	0.11017598
ENSG0000011	RTN4	protein_codir	-0.66332226	0.00932311	0.03297531
ENSG0000004	COL9A2	protein_codir	-0.66338156	0.09452506	0.19342326
ENSG0000016	JMJD8	protein_codir	-0.66357151	0.0012046	0.00663165
ENSG0000027	LINC02018	lncRNA	-0.66363991	0.05518186	0.12950319
ENSG0000010	SNX25	protein_codir	-0.66374161	0.0037027	0.01596713
ENSG0000009	LRRFIP2	protein_codir	-0.66386504	0.00196631	0.00969465
ENSG0000014	AGTR1	protein_codir	-0.66399131	0.11059159	0.21754431
ENSG0000009	MYO15A	protein_codir	-0.66410362	0.03566696	0.09285992
ENSG0000017	PRIMA1	protein_codir	-0.66412791	0.21902973	0.35962437
ENSG0000024	AC093627.8	lncRNA	-0.66439462	0.29140329	0.44121033
ENSG0000027	RP11-521C20.	lncRNA	-0.66450798	0.730129	NA
ENSG0000013	SPAG8	protein_codir	-0.66452464	0.02519305	0.07105401
ENSG0000017	PCSK1	protein_codir	-0.66472351	0.21836442	0.35883063
ENSG0000019	ADAM32	protein_codir	-0.66477581	0.00873666	0.03126949
ENSG0000018	CFAP91	protein_codir	-0.6648168	0.079348	0.16963086
ENSG0000012	ZNF391	protein_codir	-0.66494554	0.02011122	0.05954886

ENSG0000010	TRDMT1	protein_codir	-0.66497275	0.00358125	0.01556478
ENSG0000028	CTC-359D24.5	protein_codir	-0.6652596	0.03951812	0.1005704
ENSG0000009	NDUFB7	protein_codir	-0.66548653	0.00024242	0.0018096
ENSG0000018	C11orf95	protein_codir	-0.66549601	0.01568437	0.04932844
ENSG0000016	PRKAA2	protein_codir	-0.66550818	0.27002795	0.41786678
ENSG0000020	LRRC10B	protein_codir	-0.66555602	0.15156929	0.27433446
ENSG0000018	SPIN2B	protein_codir	-0.66573269	0.00036825	0.00255584
ENSG0000016	CMTM5	protein_codir	-0.66596571	0.1113647	0.2186602
ENSG0000027	GTSE1-DT	lncRNA	-0.66606658	0.12395671	0.23680207
ENSG0000028	RP11-246E12.	lncRNA	-0.66636112	0.47722878	0.62531709
ENSG0000015	CYP2U1	protein_codir	-0.66636501	0.00424364	0.01783739
ENSG0000025	RP11-286N22	lncRNA	-0.66658923	0.2603861	0.40760962
ENSG0000027	U47924.32	lncRNA	-0.66686888	0.36391279	0.51791988
ENSG0000024	LINC01095	lncRNA	-0.66717044	0.67274064	0.78417795
ENSG0000011	ENO2	protein_codir	-0.66736474	0.00649595	0.02487771
ENSG0000010	NDRG3	protein_codir	-0.66748133	0.00198165	0.00975978
ENSG0000027	RP11-154H23	lncRNA	-0.66750969	0.07256219	0.15916199
ENSG0000008	ERC1	protein_codir	-0.66760133	0.00487528	0.01988277
ENSG0000025	LINC02323	lncRNA	-0.66765462	0.77251026	NA
ENSG0000023	RP4-803J11.2	lncRNA	-0.66771222	0.4946311	0.64073309
ENSG0000026	ERVV-1	protein_codir	-0.66782762	0.77658642	NA
ENSG0000019	DCAF12L1	protein_codir	-0.66787181	0.67037562	0.7825227
ENSG0000028	ACTL10	protein_codir	-0.66788194	0.01266176	0.04175237
ENSG0000018	NDN	protein_codir	-0.66808025	0.01245847	0.04122967
ENSG0000020	ZDHHC11B	protein_codir	-0.6681269	0.19147751	0.32605479
ENSG0000011	CMAS	protein_codir	-0.66832257	1.92E-05	0.00022202
ENSG0000025	RP11-407N17	lncRNA	-0.66833852	0.00867327	0.03111518
ENSG0000019	TECPR2	protein_codir	-0.66835862	0.00492274	0.02002419
ENSG0000025	RP5-877J2.1	protein_codir	-0.66848347	0.38745858	0.54048515
ENSG0000020	DCTN1	protein_codir	-0.66861233	0.00039028	0.00268417
ENSG0000010	PCYT1B	protein_codir	-0.66862741	0.13694714	0.25460958
ENSG0000021	ITGA1	protein_codir	-0.66865844	0.10491326	0.20911483
ENSG0000026	RP5-1057I20.4	lncRNA	-0.66876733	0.08075797	0.17193814
ENSG0000018	BTBD6	protein_codir	-0.66884081	0.00037816	0.00261186
ENSG0000018	SPACA6	protein_codir	-0.66889903	0.05654419	0.13190936
ENSG0000022	KLLN	protein_codir	-0.66915957	0.02217839	0.06421711
ENSG0000016	ZNF283	protein_codir	-0.66971699	0.00223738	0.010736
ENSG0000027	RP11-373D23	lncRNA	-0.66977188	0.19368232	0.32907884
ENSG0000026	TMSB15B	protein_codir	-0.66981173	0.05069744	0.12182558
ENSG0000019	TTC30A	protein_codir	-0.66982817	0.02809714	0.07738763
ENSG0000016	FABP7	protein_codir	-0.66985341	0.83700265	NA
ENSG0000028	RP11-609L3.3	lncRNA	-0.66985341	0.83700265	NA
ENSG0000010	GAB1	protein_codir	-0.66988518	0.0734294	0.16050333
ENSG0000017	SSH3	protein_codir	-0.66995957	0.02195505	0.06373078
ENSG0000017	HYI	protein_codir	-0.66998294	0.00394327	0.01681798
ENSG0000027	AP001412.1	lncRNA	-0.67021199	0.49641819	0.64228798
ENSG0000012	NDUFAF4	protein_codir	-0.67033118	0.01107034	0.03773451

ENSG0000008	XPO1	protein_codir	-0.67044594	0.01015454	0.03523471
ENSG0000018	MTURN	protein_codir	-0.67071551	0.07444292	0.16207073
ENSG0000016	TRMT61A	protein_codir	-0.67097046	0.00272422	0.01250713
ENSG0000019	MAFK	protein_codir	-0.6710271	0.0656009	0.14798244
ENSG0000025	STX17-AS1	lncRNA	-0.67137841	0.10582519	0.21053
ENSG0000026	RP11-235E17.	lncRNA	-0.67142527	0.08265032	0.17469976
ENSG0000016	PLPP3	protein_codir	-0.67143654	0.045869	0.11268456
ENSG0000016	MPP3	protein_codir	-0.67224783	0.01189385	0.03981917
ENSG0000018	GPR21	protein_codir	-0.67230115	0.19721864	0.33332387
ENSG0000019	COLGALT2	protein_codir	-0.67258473	0.09390851	0.19247204
ENSG0000025	RP5-972B16.2	protein_codir	-0.6726017	0.40416551	0.55723313
ENSG0000016	TYW3	protein_codir	-0.67272153	0.00070656	0.00433671
ENSG0000015	PELO	protein_codir	-0.67282567	0.00417529	0.01764136
ENSG0000016	SQSTM1	protein_codir	-0.6732539	9.53E-05	0.00083532
ENSG0000025	SMIM15-AS1	lncRNA	-0.67343498	0.32127562	0.47343715
ENSG0000001	RANBP9	protein_codir	-0.67350647	0.0028656	0.01300493
ENSG0000000	CCDC124	protein_codir	-0.6735802	3.05E-05	0.00032454
ENSG0000008	TASP1	protein_codir	-0.67360212	0.00972856	0.03404413
ENSG0000026	RP11-192H23	lncRNA	-0.6738432	0.60111327	NA
ENSG0000027	RP11-725P16.	lncRNA	-0.67384387	0.30827324	0.45994527
ENSG0000027	RP11-88E10.4	lncRNA	-0.6739599	0.02739213	0.07577073
ENSG0000023	EP300-AS1	lncRNA	-0.67398017	0.77416133	NA
ENSG0000017	CTBP2	protein_codir	-0.67469748	0.0074682	0.02770517
ENSG0000007	AP3M2	protein_codir	-0.6747426	0.00358769	0.01558046
ENSG0000015	TMSB15B	protein_codir	-0.67478308	0.08868831	0.18427527
ENSG0000025	RP11-933H2.4	lncRNA	-0.67480586	0.14284518	0.26278034
ENSG0000027	CTD-3035K23	lncRNA	-0.67483663	0.02623944	0.0732794
ENSG0000027	WI2-85898F1(	lncRNA	-0.67496356	0.20121922	0.33814035
ENSG0000020	RTL8A	protein_codir	-0.67525944	0.00110806	0.00620737
ENSG0000026	LINC02166	lncRNA	-0.67532158	0.32874254	0.48107815
ENSG0000022	RP11-70P17.1	lncRNA	-0.67551882	0.14195801	0.2615664
ENSG0000013	LAMC1	protein_codir	-0.67556429	0.03919631	0.09992634
ENSG0000011	SLC29A1	protein_codir	-0.67557174	0.02165161	0.06302218
ENSG0000012	ACO1	protein_codir	-0.67559687	0.00509475	0.02057866
ENSG0000023	RP11-432J22.	lncRNA	-0.67559737	0.05004658	0.12060786
ENSG0000016	GPSM1	protein_codir	-0.67560609	0.00112369	0.00627251
ENSG0000011	ARL6	protein_codir	-0.67626276	0.00172242	0.00877088
ENSG0000013	ROCK2	protein_codir	-0.67633834	0.04213451	0.10571903
ENSG0000014	SLC25A52	protein_codir	-0.67653381	0.57454247	0.70721488
ENSG0000028	RP11-245P10.	lncRNA	-0.67670949	0.35457917	0.50814491
ENSG0000004	WWC3	protein_codir	-0.67692491	0.00300547	0.01352414
ENSG0000027	RP11-179B2.2	lncRNA	-0.67740439	0.14617961	0.26745369
ENSG0000014	NFIC	protein_codir	-0.67745533	0.00524714	0.02106484
ENSG0000011	CCDC92	protein_codir	-0.6784646	0.00753501	0.02787905
ENSG0000027	RP11-616M22	lncRNA	-0.67858022	0.49333478	0.63980404
ENSG0000028	SAMD12-AS1	lncRNA	-0.67879681	0.50499417	0.64958816
ENSG0000012	GNRH2	protein_codir	-0.67888876	0.48254767	0.63001497

ENSG0000015	MICU3	protein_codir	-0.67902316	0.02862441	0.07858127
ENSG0000018	TUBB4B	protein_codir	-0.67903134	0.00595779	0.0231995
ENSG0000014	PRDM14	protein_codir	-0.67924945	0.72079872	NA
ENSG0000027	ENSG0000027	protein_codir	-0.67933241	0.02391165	0.06811971
ENSG0000010	ABCD1	protein_codir	-0.6794279	0.00044781	0.0029881
ENSG0000019	FAT4	protein_codir	-0.67966232	0.05748387	0.13348785
ENSG0000017	NT5DC1	protein_codir	-0.67988489	0.00466755	0.01925004
ENSG0000019	POTEF	protein_codir	-0.67995034	0.48606004	0.63325774
ENSG0000014	RPRD1A	protein_codir	-0.68006177	0.00025009	0.00185635
ENSG0000000	MLXIPL	protein_codir	-0.68026161	0.19545011	0.33119507
ENSG0000011	HDDC2	protein_codir	-0.68028191	0.00214421	0.01036455
ENSG0000025	RP11-671J11.4	lncRNA	-0.68043891	0.68614529	NA
ENSG0000015	TMEM263	protein_codir	-0.68049377	0.0003393	0.00238826
ENSG0000013	GGT7	protein_codir	-0.680637	8.98E-05	0.00079519
ENSG0000016	EIF5A2	protein_codir	-0.68089268	0.01054835	0.03630924
ENSG0000017	MIEF2	protein_codir	-0.68101321	0.00533171	0.02132684
ENSG0000012	TP53AIP1	protein_codir	-0.68117129	0.41299777	0.56601987
ENSG0000016	ZFPL1	protein_codir	-0.6812657	6.27E-05	0.0005926
ENSG0000027	PCDHB16	protein_codir	-0.68136903	0.14156059	0.2610431
ENSG0000027	RP3-426I6.6	lncRNA	-0.68137933	0.50135817	0.6461091
ENSG0000021	PEX26	protein_codir	-0.68148052	1.96E-08	6.16E-07
ENSG0000010	SEPTIN4	protein_codir	-0.68184395	0.00873473	0.03126949
ENSG0000008	POMGNT1	protein_codir	-0.68185057	9.94E-07	1.83E-05
ENSG0000025	RYR3-DT	lncRNA	-0.68190055	0.35148491	0.50502071
ENSG0000028	RP11-424G14	lncRNA	-0.68204718	0.2032806	0.34056595
ENSG0000010	POLD2	protein_codir	-0.68235194	1.40E-05	0.00017023
ENSG0000028	AC008079.12	protein_codir	-0.68241836	0.26730245	0.41504912
ENSG0000016	OXR1	protein_codir	-0.68246128	0.00637908	0.02451846
ENSG0000014	FADS1	protein_codir	-0.68250602	0.00135843	0.00729673
ENSG0000017	SLC26A9	protein_codir	-0.6826952	0.29077046	0.44049589
ENSG0000012	SPRYD7	protein_codir	-0.68273671	0.00918966	0.03260023
ENSG0000011	PLAGL1	protein_codir	-0.68301883	0.00193718	0.00957153
ENSG0000013	NES	protein_codir	-0.68305615	0.10257113	0.20569193
ENSG0000027	RP11-632L2.2	lncRNA	-0.68348682	0.05175812	0.12350403
ENSG0000028	RP11-363H12	lncRNA	-0.68364174	0.60002569	0.72754333
ENSG0000016	STEAP1	protein_codir	-0.68375153	0.14673673	0.26817601
ENSG0000019	MSRB1	protein_codir	-0.68375789	0.0023627	0.01119344
ENSG0000015	PTPN14	protein_codir	-0.68383066	0.01680375	0.05196725
ENSG0000027	RP11-216L13.	protein_codir	-0.68395664	0.25934397	0.40643898
ENSG0000028	RP11-788A4.3	lncRNA	-0.68400885	0.52843525	0.6699124
ENSG0000010	DCUN1D4	protein_codir	-0.68406122	0.00077622	0.0046688
ENSG0000015	FAM177A1	protein_codir	-0.6843298	0.01095232	0.03743645
ENSG0000014	GIN1	protein_codir	-0.6844227	0.00466824	0.01925004
ENSG0000025	RP11-736K20.	lncRNA	-0.68444624	0.102805	0.20605762
ENSG0000014	SOD1	protein_codir	-0.68487504	5.87E-05	0.00056302
ENSG0000000	YBX2	protein_codir	-0.68488903	NA	NA
ENSG0000026	RP11-678G14	lncRNA	-0.68492461	0.50065318	0.64585947

ENSG0000027	RP11-567M16	lncRNA	-0.68503799	0.01454744	0.04658014
ENSG0000012	POPDC2	protein_codir	-0.68521573	0.09944942	0.20093584
ENSG0000028	RP11-932A10	lncRNA	-0.68576951	0.58078914	0.71247595
ENSG0000018	JRKL	protein_codir	-0.68599663	0.00042316	0.00285482
ENSG0000001	TSPAN9	protein_codir	-0.68657729	0.00214399	0.01036455
ENSG0000022	ENTPD1-AS1	lncRNA	-0.68663369	0.01665398	0.0516081
ENSG0000017	CLDN20	protein_codir	-0.68678029	0.80995975	NA
ENSG0000020	SMIM11A	protein_codir	-0.68679144	0.01525788	0.04832827
ENSG0000025	CTD-2330J20.	lncRNA	-0.6869035	0.608591	0.73432095
ENSG0000027	RP11-513M16	lncRNA	-0.68722972	0.0796513	0.17009193
ENSG0000017	NECTIN3	protein_codir	-0.68727654	0.05713827	0.1328864
ENSG0000026	CTD-2196E14.	lncRNA	-0.6874813	0.37195183	0.52533193
ENSG0000013	MACROD1	protein_codir	-0.68763422	0.00784157	0.02874699
ENSG0000017	ARHGAP1	protein_codir	-0.68766645	0.01730611	0.05307209
ENSG0000025	RP11-154J22.	lncRNA	-0.68798987	0.02271153	0.06548559
ENSG0000014	TRPC1	protein_codir	-0.6885013	0.0252092	0.0710923
ENSG0000014	IFITM3	protein_codir	-0.68854795	0.00374597	0.01611837
ENSG0000026	RP11-360L9.8	lncRNA	-0.68864421	0.49165336	0.63831276
ENSG0000028	RP11-16N10.2	protein_codir	-0.68865801	0.04033347	0.1021653
ENSG0000018	NRBP2	protein_codir	-0.68867082	0.02089872	0.06135726
ENSG0000020	PSORS1C3	lncRNA	-0.68867486	0.52775635	0.66932824
ENSG0000010	PXMP4	protein_codir	-0.68885269	0.00282513	0.01286142
ENSG0000012	SORBS3	protein_codir	-0.68890667	0.01095308	0.03743645
ENSG0000028	RP11-7O11.3	lncRNA	-0.6889402	0.21805641	0.35848476
ENSG0000027	RP11-422P24.	lncRNA	-0.68930339	0.03376051	0.08912878
ENSG0000011	SPSB2	protein_codir	-0.68931501	0.00233449	0.01110167
ENSG0000015	ZNF827	protein_codir	-0.6893702	0.00040439	0.0027592
ENSG0000028	RP5-994D16.1	lncRNA	-0.6894679	0.2430629	0.38805765
ENSG0000028	CTC-337B15.3	protein_codir	-0.68999276	NA	NA
ENSG0000013	TUBG1	protein_codir	-0.69007468	0.00107187	0.00605297
ENSG0000009	MAP3K20	protein_codir	-0.69026427	0.12560364	0.23917081
ENSG0000008	DYNLL1	protein_codir	-0.69027938	2.45E-06	3.91E-05
ENSG0000021	PPP1CB	protein_codir	-0.69029157	0.0065607	0.02502207
ENSG0000019	GYPE	protein_codir	-0.69030164	0.01706973	0.05255443
ENSG0000019	SLC22A5	protein_codir	-0.6904648	0.00166941	0.00856886
ENSG0000025	RP11-539G18	lncRNA	-0.69071961	0.11920048	0.22987832
ENSG0000013	SLC44A5	protein_codir	-0.69105746	0.12964155	0.24463343
ENSG0000026	RP11-552F3.1	lncRNA	-0.69107611	0.36928486	0.52286749
ENSG0000027	CEP83-DT	lncRNA	-0.69114681	0.06392883	0.14514707
ENSG0000015	KCTD15	protein_codir	-0.6911692	0.05177413	0.12351385
ENSG0000027	RP11-74E22.6	lncRNA	-0.69126383	0.55988341	0.69524862
ENSG0000023	LL0XNC01-25(	lncRNA	-0.69153557	0.13735172	0.25508704
ENSG0000016	G6PD	protein_codir	-0.69169902	3.83E-05	0.00039179
ENSG0000013	PDE6H	protein_codir	-0.69174758	0.76475079	NA
ENSG0000025	RPPH1	lncRNA	-0.6919598	0.7612791	NA
ENSG0000026	CTD-3193O13	lncRNA	-0.69196604	0.59056712	NA
ENSG0000011	C1orf198	protein_codir	-0.69202907	0.06849475	0.15252964

ENSG0000018	FAM131C	protein_codir	-0.69218688	0.26137815	0.40874183
ENSG0000015	AK9	protein_codir	-0.69224351	0.00164127	0.00845901
ENSG0000015	STK32B	protein_codir	-0.69234768	0.00187526	0.00933572
ENSG0000014	ESAM	protein_codir	-0.69236713	NA	NA
ENSG0000013	POU2F3	protein_codir	-0.69257327	0.3951844	0.54818909
ENSG0000021	FAM228B	protein_codir	-0.69299042	0.00254985	0.01185239
ENSG0000010	BPNT2	protein_codir	-0.69301325	0.02663103	0.0741213
ENSG0000024	RP11-18H7.1	lncRNA	-0.69309701	0.02779288	0.07671025
ENSG0000021	PPME1	protein_codir	-0.69316735	1.47E-05	0.00017761
ENSG0000015	NRG2	protein_codir	-0.69321376	0.03730317	0.09613826
ENSG0000017	ALS2CL	protein_codir	-0.69363261	0.02188384	0.0635535
ENSG0000025	LINC02298	lncRNA	-0.69389035	0.07322081	0.1602249
ENSG0000018	ASAH2	protein_codir	-0.69405287	0.05210075	0.12406437
ENSG0000014	LMTK3	protein_codir	-0.69407942	0.11305522	0.2209272
ENSG0000011	FKBP1B	protein_codir	-0.69466881	0.00055418	0.00355385
ENSG0000018	GLIPR1L2	protein_codir	-0.69479344	0.06317782	0.14374919
ENSG0000018	TDRD6	protein_codir	-0.69487285	0.20958049	0.34866963
ENSG0000012	EEF1E1	protein_codir	-0.69499673	0.00016981	0.00135671
ENSG0000014	CNIH4	protein_codir	-0.69505103	0.00091462	0.00533174
ENSG0000026	AC006538.4	protein_codir	-0.69559724	0.03706041	0.09566435
ENSG0000016	CDK2AP2	protein_codir	-0.69600207	0.00181081	0.00909367
ENSG0000018	PAPPA	protein_codir	-0.69617522	0.04474959	0.11068181
ENSG0000017	ZNF778	protein_codir	-0.69635715	0.00094116	0.00545998
ENSG0000010	CPE	protein_codir	-0.69640177	0.08690992	0.18154985
ENSG0000023	LNCTAM34A	lncRNA	-0.69669991	0.01857599	0.05604248
ENSG0000028	RP11-47B24.1	lncRNA	-0.69684721	0.76345593	NA
ENSG0000015	CEBPG	protein_codir	-0.69689238	0.00258096	0.01197186
ENSG0000010	CCDC113	protein_codir	-0.69727449	0.04350381	0.10829629
ENSG0000014	URB1	protein_codir	-0.69732486	0.0097337	0.03405684
ENSG0000028	PCAT14	lncRNA	-0.69742873	0.75983371	NA
ENSG0000027	RP11-393I2.4	lncRNA	-0.69743076	0.25692518	0.40367901
ENSG0000015	DMP1	protein_codir	-0.69763931	0.28831633	0.43792878
ENSG0000009	HOOK2	protein_codir	-0.69771981	0.00074368	0.00451515
ENSG0000018	TCEAL9	protein_codir	-0.69802586	0.01585798	0.04978374
ENSG0000028	RP11-596C23.	lncRNA	-0.69823632	0.02263624	0.06531003
ENSG0000015	SH3RF1	protein_codir	-0.69832526	0.00465434	0.01921569
ENSG0000017	EIF3J-DT	lncRNA	-0.69832817	0.00308275	0.01380441
ENSG0000023	RP11-213G2.2	lncRNA	-0.69842612	0.14700048	0.26849226
ENSG0000027	RP11-414C23.	lncRNA	-0.69847957	0.51473396	0.65794854
ENSG0000010	CBX7	protein_codir	-0.69871058	0.01030255	0.03563639
ENSG0000015	ZYX	protein_codir	-0.69894815	0.00938	0.03312103
ENSG0000015	CHCHD6	protein_codir	-0.69895145	0.00071231	0.0043652
ENSG0000017	HSPA4	protein_codir	-0.69899441	0.00229141	0.01094014
ENSG0000015	SYN2	protein_codir	-0.69915731	0.24418708	0.38923928
ENSG0000001	WIZ	protein_codir	-0.69979094	0.00021459	0.0016332
ENSG0000015	DPH3	protein_codir	-0.69990473	0.00100547	0.00575685
ENSG0000012	PPP1R12C	protein_codir	-0.6999204	0.01130252	0.03831784

ENSG0000014	HSD17B12	protein_codir	-0.70004623	0.00358465	0.01557219
ENSG0000028	RP11-373D17	lncRNA	-0.70010608	0.22872081	0.37121101
ENSG0000027	LPP-AS2	lncRNA	-0.70055277	0.00102259	0.00582953
ENSG0000023	BAALC-AS2	lncRNA	-0.70069756	0.60844938	0.7342438
ENSG0000028	CTA-286B10.7	lncRNA	-0.70073867	0.7115375	NA
ENSG0000010	NME4	protein_codir	-0.70093249	0.00973608	0.03406086
ENSG0000027	RP11-449G16	lncRNA	-0.70106315	0.21520102	0.35532353
ENSG0000010	PTGR1	protein_codir	-0.70113541	0.01199268	0.04005295
ENSG0000013	SUCLA2	protein_codir	-0.70116206	0.00027255	0.00199254
ENSG0000011	MTFR1L	protein_codir	-0.7011685	0.00046617	0.00308767
ENSG0000011	CORO1C	protein_codir	-0.70123079	0.01355209	0.04405188
ENSG0000023	RPEL1	protein_codir	-0.70124433	0.71388525	NA
ENSG0000013	WNT2B	protein_codir	-0.70168694	0.05982597	0.13766501
ENSG0000016	MID1IP1	protein_codir	-0.70171606	0.00492555	0.02002971
ENSG0000007	GPC4	protein_codir	-0.70186326	0.05112256	0.12247058
ENSG0000016	C16orf71	protein_codir	-0.70192325	0.01767165	0.0539208
ENSG0000024	RP11-67L3.5	lncRNA	-0.70204536	0.52729036	0.668983
ENSG0000026	CTD-2380F24.	lncRNA	-0.70235287	0.62284094	0.745552
ENSG0000027	ARMCX5-GPR	lncRNA	-0.7024309	0.00016062	0.00129794
ENSG0000012	PEDS1-UBE2V	protein_codir	-0.70252517	0.77387099	0.85778265
ENSG0000010	ZDHHC15	protein_codir	-0.70264869	0.00898903	0.03202747
ENSG0000013	SCRN1	protein_codir	-0.7026646	0.0073158	0.02723948
ENSG0000027	RP11-143K11.	lncRNA	-0.70303817	0.34319048	0.49635656
ENSG0000016	FBXL13	protein_codir	-0.70308798	0.00723005	0.02698932
ENSG0000025	LINC01269	lncRNA	-0.70316233	0.54813741	0.6857925
ENSG0000017	ATP2A2	protein_codir	-0.70327588	0.01375018	0.04453852
ENSG0000021	LBH	protein_codir	-0.70353812	0.00863539	0.03099539
ENSG0000023	AGBL5-AS1	lncRNA	-0.70380524	0.54462411	0.68281969
ENSG0000018	ZNF563	protein_codir	-0.70402148	0.00591741	0.02309111
ENSG0000015	DDAH1	protein_codir	-0.70442448	0.05418813	0.12787731
ENSG0000026	AC003002.4	protein_codir	-0.70448136	0.79854742	NA
ENSG0000024	RP11-468H14	lncRNA	-0.70450849	0.25452744	0.40101381
ENSG0000025	ACTN1-AS1	lncRNA	-0.70465066	0.29057944	0.44037333
ENSG0000015	RWDD2B	protein_codir	-0.70469019	0.00095855	0.00554223
ENSG0000015	LURAP1L	protein_codir	-0.70470955	0.0122658	0.04077282
ENSG0000013	PATJ	protein_codir	-0.70481208	0.02030854	0.0600109
ENSG0000025	CTC-338M12.	lncRNA	-0.70484359	0.0687536	0.15292111
ENSG0000016	NAB2	protein_codir	-0.7049866	0.00098267	0.00564976
ENSG0000014	C7orf50	protein_codir	-0.70500882	0.00024664	0.0018357
ENSG0000025	PCDHGB6	protein_codir	-0.70522996	0.01709735	0.05262774
ENSG0000011	CEP126	protein_codir	-0.70551157	0.01946416	0.05809456
ENSG0000012	SLC2A4RG	protein_codir	-0.70616254	0.00017047	0.00136157
ENSG0000026	C16orf95	protein_codir	-0.70620065	0.00071823	0.00439569
ENSG0000016	FN3K	protein_codir	-0.70638439	0.00334553	0.01474918
ENSG0000017	CALB2	protein_codir	-0.70653969	0.30283614	0.45418843
ENSG0000016	RNF183	protein_codir	-0.70666263	0.4469929	0.59782863
ENSG0000011	C12orf57	protein_codir	-0.70668835	0.00074138	0.00450538

ENSG0000015	ZFYVE9	protein_codir	-0.70674571	0.01232621	0.0408998
ENSG0000025	PAPPA-AS1	lncRNA	-0.70723022	0.1993143	0.33581779
ENSG0000011	DBN1	protein_codir	-0.70727573	0.03978416	0.1010891
ENSG0000023	AC108868.5	lncRNA	-0.70734643	0.71204195	NA
ENSG0000028	LINC01144	lncRNA	-0.70738728	0.0160662	0.05030022
ENSG0000027	RP11-302B13.	protein_codir	-0.70780584	0.22498907	0.36674918
ENSG0000011	DPYSL3	protein_codir	-0.70790766	0.03040777	0.08230331
ENSG0000014	SELENBP1	protein_codir	-0.7079253	0.15867252	0.28395329
ENSG0000016	NDUFAF2	protein_codir	-0.70821412	0.0031619	0.01408804
ENSG0000027	KB-68A7.1	lncRNA	-0.70827459	0.2504421	0.39636778
ENSG0000013	CAMK1	protein_codir	-0.70843735	3.93E-05	0.0003995
ENSG0000023	ZNF197-AS1	lncRNA	-0.70863989	0.48187413	0.62949022
ENSG0000018	CDHR4	protein_codir	-0.709006	0.25611583	0.4027969
ENSG0000019	LYPD2	protein_codir	-0.70901019	0.74785982	NA
ENSG0000015	DST	protein_codir	-0.70903596	0.04257169	0.10659326
ENSG0000017	RPRM	protein_codir	-0.7091292	0.36956619	0.523055
ENSG0000027	RP11-500M8.	protein_codir	-0.70919042	0.11931713	0.23000693
ENSG0000015	PIP4P2	protein_codir	-0.70934172	0.00528331	0.02118541
ENSG0000013	LPIN3	protein_codir	-0.70946248	0.01112719	0.03785987
ENSG0000016	FCN2	protein_codir	-0.70965447	0.36870889	0.52224426
ENSG0000015	ALDH4A1	protein_codir	-0.70981165	0.00562307	0.02221489
ENSG0000025	LINC02208	lncRNA	-0.7098784	0.54834014	0.68592697
ENSG0000027	RP11-165A20	lncRNA	-0.71039866	0.41174502	0.56475133
ENSG0000023	RP11-656D10	lncRNA	-0.71057438	0.26109412	0.4083939
ENSG0000028	CTD-3018O17	lncRNA	-0.71057799	0.12891916	0.24370958
ENSG0000018	PCGF5	protein_codir	-0.71067298	0.08080038	0.17196074
ENSG0000020	DDO	protein_codir	-0.71070073	0.00289177	0.01310214
ENSG0000025	CTC-554D6.1	protein_codir	-0.71085552	0.80205377	0.8773704
ENSG0000014	C11orf52	protein_codir	-0.71090164	0.116833	0.22645992
ENSG0000012	THAP10	protein_codir	-0.71106381	0.0029053	0.0131456
ENSG0000025	RP11-540O11	lncRNA	-0.71116263	0.18809486	0.32192217
ENSG0000011	CDK2AP1	protein_codir	-0.71131285	0.00052114	0.00338204
ENSG0000017	NUDT9	protein_codir	-0.71135653	1.12E-05	0.00014026
ENSG0000011	NUDT12	protein_codir	-0.71145031	0.00730007	0.0271919
ENSG0000014	ZNF660	protein_codir	-0.71157423	0.00029879	0.00214294
ENSG0000011	RAB3GAP1	protein_codir	-0.711665	0.00397502	0.01693149
ENSG0000010	SLAIN2	protein_codir	-0.71218097	0.00340274	0.0149299
ENSG0000019	PARVA	protein_codir	-0.71218979	0.03255083	0.08658069
ENSG0000013	NAPG	protein_codir	-0.71236326	7.14E-06	9.67E-05
ENSG0000028	RP4-684F13.1	lncRNA	-0.71243665	0.65712212	NA
ENSG0000028	RP11-311H10	lncRNA	-0.71247124	0.47122185	0.62015284
ENSG0000027	HSPE1-MOB4	protein_codir	-0.71253191	0.17673076	0.30715478
ENSG0000011	PHF19	protein_codir	-0.7125668	2.17E-08	6.72E-07
ENSG0000015	ASAP2	protein_codir	-0.71298765	0.04104042	0.10353822
ENSG0000026	CTD-3252C9.2	lncRNA	-0.7132639	0.71125336	NA
ENSG0000011	FAM98A	protein_codir	-0.71342452	2.66E-05	0.00029122
ENSG0000027	LENG9	protein_codir	-0.71345045	6.31E-05	0.00059582

ENSG0000011	RNF141	protein_codir	-0.71394857	0.06078253	0.13934375
ENSG0000015	PTPRD	protein_codir	-0.71427455	0.05071475	0.12185658
ENSG0000016	ST6GALNAC6	protein_codir	-0.71512153	0.00428242	0.0179703
ENSG0000011	USP5	protein_codir	-0.71526682	0.00038071	0.00262747
ENSG0000017	PHYHD1	protein_codir	-0.71531054	0.03990715	0.10133636
ENSG0000026	DOCK9-DT	lncRNA	-0.71544025	0.07390243	0.16125042
ENSG0000027	CTC-471J1.11	lncRNA	-0.71585713	0.03659819	0.09473684
ENSG0000025	RP11-359B12.	lncRNA	-0.71592047	0.01564774	0.04922444
ENSG0000019	ZNF441	protein_codir	-0.71627721	0.05495315	0.12912004
ENSG0000018	PALM3	protein_codir	-0.7164371	0.00670535	0.02542582
ENSG0000005	PPP1R12A	protein_codir	-0.71683046	0.00875331	0.03132095
ENSG0000015	PDE1C	protein_codir	-0.71687886	0.08415746	0.17713984
ENSG0000026	RP11-45M22.	protein_codir	-0.71695258	0.46953405	0.61875712
ENSG0000019	ZNF471	protein_codir	-0.7169529	0.00426518	0.01791426
ENSG0000028	LINC00294	lncRNA	-0.7170739	0.00076012	0.004597
ENSG0000022	ZNF630	protein_codir	-0.71710687	5.12E-05	0.00050129
ENSG0000014	C20orf144	protein_codir	-0.71725168	0.24182552	0.38664086
ENSG0000010	SCML2	protein_codir	-0.71750369	0.01185352	0.0397207
ENSG0000018	ZNF397	protein_codir	-0.71788009	0.00037166	0.0025747
ENSG0000016	SENP8	protein_codir	-0.71824577	0.00485075	0.01981626
ENSG0000027	RP11-305L7.7	lncRNA	-0.71871333	0.23180179	0.37456066
ENSG0000024	MRPS6	protein_codir	-0.71902238	0.00447045	0.01858425
ENSG0000022	PSPC1-AS2	lncRNA	-0.71949032	0.66013624	NA
ENSG0000000	CYTH3	protein_codir	-0.71949631	0.03584288	0.09322726
ENSG0000022	NGF-AS1	lncRNA	-0.71957682	0.10292154	0.2062135
ENSG0000022	RP3-495K2.2	lncRNA	-0.71962058	0.32600364	0.47823563
ENSG0000001	ANOS1	protein_codir	-0.71992231	0.10415648	0.20802099
ENSG0000020	RP13-259N13	lncRNA	-0.72039307	0.58121691	0.71271606
ENSG0000010	NINL	protein_codir	-0.72077868	0.03862201	0.09871736
ENSG0000013	DTNA	protein_codir	-0.72079748	0.18974451	0.32410287
ENSG0000025	LINC02284	lncRNA	-0.72083362	0.23440478	0.3777722
ENSG0000017	POGLUT3	protein_codir	-0.7208507	0.01457464	0.04666182
ENSG0000023	RP11-34A14.3	lncRNA	-0.72086364	0.49497554	0.64096885
ENSG0000010	CLIP3	protein_codir	-0.72092973	0.00720616	0.02691609
ENSG0000016	DNAJB4	protein_codir	-0.72115187	0.05861891	0.13550839
ENSG0000013	CNGA4	protein_codir	-0.72142688	0.22361006	0.36508304
ENSG0000026	RP11-19N8.4	lncRNA	-0.72142788	0.71236749	NA
ENSG0000016	ZSWIM5	protein_codir	-0.72146378	0.00762304	0.02813689
ENSG0000016	CALM3	protein_codir	-0.72153879	5.34E-08	1.47E-06
ENSG0000013	FOXP4	protein_codir	-0.7218154	0.00103225	0.00587732
ENSG0000026	CTB-25B13.5	lncRNA	-0.72183011	0.11133078	0.21862467
ENSG0000025	RP11-627D16	lncRNA	-0.7218911	0.5729804	0.70600101
ENSG0000022	LINC02601	lncRNA	-0.72192359	0.65629692	NA
ENSG0000014	ABHD2	protein_codir	-0.72195683	0.01053843	0.03628871
ENSG0000014	AKT1	protein_codir	-0.722228	1.99E-05	0.00022776
ENSG0000022	MIR4500HG	lncRNA	-0.72282258	0.42721367	0.57897871
ENSG0000023	LINC01474	lncRNA	-0.72312875	0.25604624	0.40271038

ENSG0000024	DUBR	lncRNA	-0.72354146	0.02038882	0.06015802
ENSG0000016	CA3	protein_codir	-0.72365671	0.11012703	0.21687762
ENSG0000017	TRAM1L1	protein_codir	-0.7237315	0.0223602	0.06468252
ENSG0000017	C15orf56	lncRNA	-0.72377145	0.3391966	0.4920747
ENSG0000015	GDF6	protein_codir	-0.72396243	0.16576672	0.29332429
ENSG0000025	RP11-613M1(	protein_codir	-0.7240924	0.28587789	0.43544628
ENSG0000016	SOAT2	protein_codir	-0.72415178	0.12155715	0.23335306
ENSG0000014	GPR17	protein_codir	-0.72424236	0.17732622	0.30787969
ENSG0000025	RP11-589M4.	lncRNA	-0.72448561	0.20894101	0.34787363
ENSG0000026	LINC02177	lncRNA	-0.72451931	0.45426188	0.60469001
ENSG0000016	CCDC110	protein_codir	-0.72455637	0.02298454	0.06605274
ENSG0000012	ZNF835	protein_codir	-0.72461407	0.10142648	0.2039161
ENSG0000015	CLDN12	protein_codir	-0.72509916	0.01276305	0.04202617
ENSG0000016	KLHL21	protein_codir	-0.72536407	0.01987661	0.05903141
ENSG0000017	FGFBP3	protein_codir	-0.72575656	0.00687536	0.02596355
ENSG0000011	MTRF1L	protein_codir	-0.72575968	8.20E-06	0.00010901
ENSG0000013	NIBAN2	protein_codir	-0.72616389	0.00668771	0.02537287
ENSG0000028	RP11-323F24.	lncRNA	-0.72623926	6.61E-06	9.01E-05
ENSG0000010	CASC3	protein_codir	-0.72625922	0.00091244	0.00532014
ENSG0000014	ANKRD30A	protein_codir	-0.72633501	0.6755321	0.78630194
ENSG0000021	TAX1BP3	protein_codir	-0.72646414	0.00421103	0.01774352
ENSG0000014	PHOSPHO2	protein_codir	-0.72648468	0.00155782	0.00811523
ENSG0000002	NCDN	protein_codir	-0.72651598	4.21E-05	0.0004228
ENSG0000012	YWHAH	protein_codir	-0.72662022	0.02326096	0.06665986
ENSG0000017	C2orf73	protein_codir	-0.72663243	0.2776115	0.42609779
ENSG0000010	MPG	protein_codir	-0.72674848	3.78E-05	0.00038709
ENSG0000027	RP11-670E13.	lncRNA	-0.72701398	NA	NA
ENSG0000012	KBTBD7	protein_codir	-0.72762636	0.0083973	0.03031414
ENSG0000028	RP11-2A1.2	lncRNA	-0.72766028	0.56301283	0.69756084
ENSG0000022	MCRIP1	protein_codir	-0.72830405	0.00063135	0.00395508
ENSG0000027	RP3-337O18.9	lncRNA	-0.72833098	0.05590364	0.13077353
ENSG0000024	RP11-256P1.1	lncRNA	-0.72835979	0.69885548	NA
ENSG0000017	MSANTD4	protein_codir	-0.72900619	0.0001978	0.00153164
ENSG0000017	RNASEH2C	protein_codir	-0.72915414	8.52E-06	0.00011264
ENSG0000011	EEF2KMT	protein_codir	-0.72992228	0.0003283	0.00232089
ENSG0000018	SESTD1	protein_codir	-0.73035697	0.02067693	0.06085855
ENSG0000019	STK40	protein_codir	-0.73039577	0.00215251	0.01039923
ENSG0000019	GFPT1	protein_codir	-0.7308875	0.01765793	0.05388963
ENSG0000024	OXCT1-AS1	lncRNA	-0.73101338	0.14795837	0.26968985
ENSG0000008	OXCT1	protein_codir	-0.73102345	0.00178854	0.00901145
ENSG0000019	LRP10	protein_codir	-0.73106786	0.00154453	0.00805663
ENSG0000028	RP11-506F3.1	lncRNA	-0.73124872	0.41949943	0.5720148
ENSG0000010	NUMBL	protein_codir	-0.73125447	0.00474303	0.01946546
ENSG0000012	PDLIM2	protein_codir	-0.73133892	0.00029877	0.00214294
ENSG0000016	DIRAS3	protein_codir	-0.73207474	0.30803457	0.45973818
ENSG0000026	CTD-2622I13.	lncRNA	-0.73211702	0.16164565	0.28780074
ENSG0000026	RP11-715J22.	lncRNA	-0.73217261	0.52266677	0.66494962

ENSG0000010	GNPNAT1	protein_codir	-0.73220081	0.00200807	0.0098626
ENSG0000012	MOCS1	protein_codir	-0.7328731	0.0040677	0.01727397
ENSG0000011	TMEM30A	protein_codir	-0.73314551	0.00171035	0.0087239
ENSG0000014	EPHA5	protein_codir	-0.7331503	0.31484266	0.46664029
ENSG0000028	RP11-806L2.4	lncRNA	-0.73345954	0.67838876	0.78829979
ENSG0000021	STIMATE	protein_codir	-0.73372904	0.00232054	0.01105123
ENSG0000022	AP000704.5	lncRNA	-0.73387475	0.06454627	0.14620043
ENSG0000017	CLTB	protein_codir	-0.73458534	0.00182565	0.00914673
ENSG0000023	AC093388.3	lncRNA	-0.73485316	0.01769429	0.0539839
ENSG0000028	RP1-64K7.5	lncRNA	-0.73487318	0.05371254	0.1269937
ENSG0000027	RP11-468E2.1	lncRNA	-0.7359599	0.07270338	0.15940839
ENSG0000023	LINC01517	lncRNA	-0.73619281	0.7030007	NA
ENSG0000017	DNAJC18	protein_codir	-0.73642684	0.00701855	0.02638884
ENSG0000007	WDR1	protein_codir	-0.73645401	0.00760656	0.0280986
ENSG0000015	NPTN	protein_codir	-0.73658759	0.01217731	0.04056541
ENSG0000028	RP3-465N24.7	lncRNA	-0.73670416	0.26827261	0.41613441
ENSG0000027	RP11-500G22	lncRNA	-0.73678598	0.42688748	0.57868133
ENSG0000025	RP11-673E1.1	lncRNA	-0.73831198	0.02157581	0.06285453
ENSG0000016	CCDC116	protein_codir	-0.73858617	0.02458352	0.06968359
ENSG0000023	SYNE1-AS1	lncRNA	-0.73896399	0.36361525	0.51765071
ENSG0000026	RP11-322E11.	protein_codir	-0.73897301	0.58867328	0.7187033
ENSG0000018	ROR1	protein_codir	-0.73901549	0.04886693	0.11838476
ENSG0000017	HSFX1	protein_codir	-0.73914182	0.21495431	0.35500109
ENSG0000013	AEBP2	protein_codir	-0.73959426	0.00508851	0.02055648
ENSG0000013	TRIM47	protein_codir	-0.74005036	5.28E-06	7.46E-05
ENSG0000014	AIG1	protein_codir	-0.74043994	0.00159776	0.00828579
ENSG0000016	MT1E	protein_codir	-0.74048991	0.30697406	0.45872057
ENSG0000018	KCTD2	protein_codir	-0.74061887	2.85E-06	4.41E-05
ENSG0000024	LIFR-AS1	lncRNA	-0.74064883	0.05345062	0.12653122
ENSG0000023	ARHGAP15-AS	lncRNA	-0.74065482	0.7508891	NA
ENSG0000023	RP11-327P2.5	lncRNA	-0.74065827	0.00539918	0.02154367
ENSG0000026	RP11-24M17.	lncRNA	-0.74116758	0.64483375	NA
ENSG0000028	RP11-749H20	lncRNA	-0.74174224	0.6878873	NA
ENSG0000022	ST7-AS1	lncRNA	-0.74218274	0.0496963	0.11997307
ENSG0000028	CH17-270A2.2	protein_codir	-0.74222631	0.8195354	NA
ENSG0000000	RHOBTB2	protein_codir	-0.74232305	0.0104705	0.03610878
ENSG0000014	EVA1B	protein_codir	-0.74241815	1.58E-05	0.00018855
ENSG0000027	RP11-317L10.	lncRNA	-0.74245225	0.61004402	NA
ENSG0000019	ARID5A	protein_codir	-0.74287033	0.09950974	0.20102831
ENSG0000013	TFAP2A	protein_codir	-0.74289442	0.11182575	0.219316
ENSG0000028	CTA-270D13.1	lncRNA	-0.74325496	0.43545947	0.58680842
ENSG0000028	RP11-136C24.	lncRNA	-0.74341171	0.52504462	0.66696097
ENSG0000022	HCG20	lncRNA	-0.74399751	0.25210965	0.39824242
ENSG0000011	PPL	protein_codir	-0.74413854	0.10434932	0.20829942
ENSG0000016	HR	protein_codir	-0.74469927	0.02289146	0.06584691
ENSG0000011	PLSCR4	protein_codir	-0.74481629	0.0214396	0.06256332
ENSG0000011	RCN2	protein_codir	-0.74491513	0.00085325	0.00504423

ENSG0000011	ANGPTL1	protein_codir	-0.74502762	0.10332949	0.20677876
ENSG0000023	CFAP97D1	protein_codir	-0.74507499	0.26414179	0.41162245
ENSG0000010	FGD1	protein_codir	-0.74528718	0.00076895	0.00463806
ENSG0000017	TMEM43	protein_codir	-0.74542366	0.01366818	0.04435093
ENSG0000007	PGM1	protein_codir	-0.74543496	0.01017457	0.03528651
ENSG0000016	ARHGEF40	protein_codir	-0.7454897	0.00117895	0.00651722
ENSG0000010	PLCB4	protein_codir	-0.74558744	0.02582634	0.07240394
ENSG0000024	SCARF2	protein_codir	-0.7456592	0.04551016	0.11218124
ENSG0000018	SEPTIN10	protein_codir	-0.7459418	0.022998	0.06607768
ENSG0000028	COL1A2-AS1	lncRNA	-0.74594245	0.13451174	0.25145275
ENSG0000021	CD27-AS1	lncRNA	-0.74596988	0.00013756	0.00114026
ENSG0000017	LONRF2	protein_codir	-0.74603678	0.19351898	0.32884178
ENSG0000026	RP11-483I13.5	lncRNA	-0.74606755	0.00333646	0.0147139
ENSG0000011	ESYT2	protein_codir	-0.74624558	0.01293825	0.04247908
ENSG0000010	ARHGEF10	protein_codir	-0.74638077	0.00420455	0.01772161
ENSG0000027	CTA-268H5.14	lncRNA	-0.7464885	0.39373425	0.54670986
ENSG0000026	RP11-420A6.2	lncRNA	-0.74656634	0.46878676	0.61821478
ENSG0000016	NLGN4Y	protein_codir	-0.74695776	0.14249699	0.26219014
ENSG0000023	RP11-1114A5	lncRNA	-0.74700405	0.05271311	0.12519873
ENSG0000022	RP11-3B7.1	lncRNA	-0.74706424	0.06255803	0.14260918
ENSG0000003	RAI14	protein_codir	-0.74725443	0.01368348	0.04439013
ENSG0000028	LINC00842	lncRNA	-0.74725709	0.21106157	0.35041599
ENSG0000012	ACSL3	protein_codir	-0.74744339	0.00058499	0.00371503
ENSG0000025	RP11-467D6.1	lncRNA	-0.74747142	0.00018532	0.00145244
ENSG0000027	RP11-136L23.	lncRNA	-0.74752722	0.56534057	NA
ENSG0000028	RP11-792A8.5	protein_codir	-0.74797762	0.31533169	0.46714553
ENSG0000017	PDGFD	protein_codir	-0.74851795	0.12153762	0.23335306
ENSG0000016	MAP1A	protein_codir	-0.74874684	0.01522964	0.04826653
ENSG0000011	HS1BP3	protein_codir	-0.74876427	0.00131258	0.00711057
ENSG0000028	RP11-564C24.	lncRNA	-0.74909566	0.36937829	0.52289624
ENSG0000017	KYAT1	protein_codir	-0.74930236	0.00172903	0.00879645
ENSG0000014	ZCCHC14	protein_codir	-0.74942843	0.00233158	0.01109166
ENSG0000010	RABAC1	protein_codir	-0.74966852	6.37E-05	0.0005995
ENSG0000018	FIGN	protein_codir	-0.74971	0.06805466	0.15197847
ENSG0000015	ANK3	protein_codir	-0.74988146	0.03499802	0.09153839
ENSG0000014	RSU1	protein_codir	-0.75000066	0.01204559	0.04019533
ENSG0000018	FBXL7	protein_codir	-0.75009917	0.01130873	0.03832481
ENSG0000028	CH17-174L20.	lncRNA	-0.75011087	0.74369221	NA
ENSG0000027	RP11-565F19.	lncRNA	-0.75017449	0.00088308	0.00518732
ENSG0000015	DIP2C	protein_codir	-0.75034052	0.03702052	0.09557925
ENSG0000020	LINC01545	lncRNA	-0.75049886	0.34294565	0.49612341
ENSG0000011	ACOT2	protein_codir	-0.75057779	0.00076856	0.00463788
ENSG0000014	TUFT1	protein_codir	-0.75102279	0.00467479	0.01927129
ENSG0000008	ZMPSTE24	protein_codir	-0.75130033	0.01100817	0.03758756
ENSG0000019	ADARB1	protein_codir	-0.75134472	0.03181438	0.08510539
ENSG0000017	SNCG	protein_codir	-0.75158012	0.09648968	0.196349
ENSG0000028	TMX2-CTNND	protein_codir	-0.75166428	0.2702042	0.41807406

ENSG0000024	USP46-DT	lncRNA	-0.75174041	0.01552859	0.04895555
ENSG0000016	CD320	protein_codir	-0.75204301	1.52E-05	0.00018221
ENSG0000019	TCEAL3	protein_codir	-0.75229967	0.03134889	0.08413723
ENSG0000014	P3H4	protein_codir	-0.75259724	0.00129225	0.007022
ENSG0000023	RP11-483I13.1	lncRNA	-0.75295973	0.31089629	0.46268096
ENSG0000022	RP11-131J3.1	lncRNA	-0.75295973	0.31089629	0.46268096
ENSG0000026	PIN1-DT	lncRNA	-0.75318379	0.40504175	0.55791209
ENSG0000016	CORO6	protein_codir	-0.7534312	0.13934036	0.25805155
ENSG0000014	FKBP10	protein_codir	-0.75375254	0.00356107	0.01549903
ENSG0000022	KCNIP2-AS1	lncRNA	-0.753788	0.31564825	0.467439
ENSG0000009	GADD45B	protein_codir	-0.75413481	NA	NA
ENSG0000017	RP5-1061H20	lncRNA	-0.75418	0.00045069	0.00300172
ENSG0000000	SEMA3F	protein_codir	-0.75478747	0.00286818	0.01301447
ENSG0000022	XPC-AS1	lncRNA	-0.75488772	0.05682496	0.13236951
ENSG0000015	PLA2R1	protein_codir	-0.75549792	0.00398942	0.01697498
ENSG0000015	SCHIP1	protein_codir	-0.75555132	0.04193564	0.10533811
ENSG0000013	MYPN	protein_codir	-0.75573073	0.51925811	0.66202711
ENSG0000011	A4GNT	protein_codir	-0.75579329	0.81665141	NA
ENSG0000024	CTD-2201E9.4	lncRNA	-0.75579329	0.81665141	NA
ENSG0000028	BTG3-AS1	lncRNA	-0.75585704	0.04974101	0.12007051
ENSG0000010	FAM149A	protein_codir	-0.75587748	0.06708226	0.15046912
ENSG0000009	TF	protein_codir	-0.75591982	0.16588027	0.2934862
ENSG0000013	PDZD2	protein_codir	-0.7562985	0.04208828	0.10563183
ENSG0000010	CD99L2	protein_codir	-0.75630058	5.57E-05	0.00053884
ENSG0000027	RP11-787I22.1	lncRNA	-0.75635001	0.34419668	0.49731702
ENSG0000027	RP11-15E1.5	lncRNA	-0.75670167	0.42086066	0.57295797
ENSG0000026	AC007292.7	lncRNA	-0.75682655	0.80237794	NA
ENSG0000012	CENPB	protein_codir	-0.75688229	2.57E-05	0.00028335
ENSG0000019	GPRASP1	protein_codir	-0.75711183	0.00991154	0.03456961
ENSG0000007	LRP6	protein_codir	-0.75719949	0.01805721	0.05483072
ENSG0000022	TCEAL3-AS1	lncRNA	-0.75741413	0.79477078	NA
ENSG0000007	MGLL	protein_codir	-0.75779653	0.00241197	0.0113742
ENSG0000019	DCAF12L2	protein_codir	-0.7580904	0.25188439	0.39806007
ENSG0000028	AC009133.24	lncRNA	-0.75824165	0.24898458	0.3946602
ENSG0000012	SDC4	protein_codir	-0.75834676	0.0781349	0.16769647
ENSG0000015	SLC25A27	protein_codir	-0.75875033	0.08078571	0.17195602
ENSG0000021	DNLZ	protein_codir	-0.75907311	0.0008331	0.0049495
ENSG0000017	PYGO1	protein_codir	-0.75920843	0.0237515	0.06777031
ENSG0000026	LINC01929	lncRNA	-0.75966422	0.09989597	0.20162673
ENSG0000027	FCHO2-DT	lncRNA	-0.75967507	0.08812679	0.18341209
ENSG0000011	TRAK2	protein_codir	-0.76009655	0.00732723	0.02727835
ENSG0000010	SEC23A	protein_codir	-0.76020792	0.01460274	0.04671931
ENSG0000027	ZNF2	protein_codir	-0.76028257	0.00027477	0.0020061
ENSG0000012	CALU	protein_codir	-0.76045878	0.01169787	0.03935696
ENSG0000027	MESTIT1	lncRNA	-0.76046594	0.250634	0.39661458
ENSG0000016	IL17RC	protein_codir	-0.76053568	0.00231305	0.01102252
ENSG0000017	C11orf68	protein_codir	-0.76055621	0.00147224	0.00777285

ENSG000001C	PMM1	protein_codir	-0.76072781	9.16E-06	0.00011972
ENSG000006	MEF2A	protein_codir	-0.76117685	0.01529515	0.04842458
ENSG000002З	SEMA3B-AS1	lncRNA	-0.76167704	0.34053987	0.49358266
ENSG000001Ş	SOWAHC	protein_codir	-0.76179362	0.01595728	0.05002726
ENSG0000024	DNAJC3-DT	lncRNA	-0.76180361	0.01400633	0.04518805
ENSG0000017	RTP1	protein_codir	-0.7619655	0.36965184	0.52307485
ENSG000002Ş	CTD-2245O6.4	lncRNA	-0.76275488	0.1604187	0.28620661
ENSG000001З	TES	protein_codir	-0.76410385	0.09512892	0.19429571
ENSG000001Ş	EIF2S3B	protein_codir	-0.76425605	0.28019811	0.42932829
ENSG0000027	RP11-159N11	lncRNA	-0.76427225	0.02792701	0.07701888
ENSG000001Ş	TTC39B	protein_codir	-0.76466678	2.43E-06	3.87E-05
ENSG000001€	SDC2	protein_codir	-0.76478699	0.03604651	0.09358065
ENSG000002Ş	RP13-465B17.	lncRNA	-0.76479342	0.27281223	0.4207188
ENSG000001€	SLC27A4	protein_codir	-0.76510237	3.25E-05	0.000342
ENSG000002€	CTD-3105H18	protein_codir	-0.76523019	0.36473353	0.51866036
ENSG000000Z	OSBPL5	protein_codir	-0.7654964	0.0004053	0.00276291
ENSG000002Ş	RP11-13N12.1	lncRNA	-0.76550429	0.56263426	0.69730681
ENSG000000Ş	AAMDC	protein_codir	-0.76587015	0.01165483	0.03923181
ENSG000002€	RP11-48B3.4	lncRNA	-0.76596513	0.00674285	0.02554697
ENSG000001C	CCDC6	protein_codir	-0.7660486	0.00331787	0.01465245
ENSG000001€	DAPL1	protein_codir	-0.7661137	0.23194209	0.37474253
ENSG0000024	RP11-967K21.	lncRNA	-0.76619684	0.0080611	0.02941571
ENSG0000017	HRAS	protein_codir	-0.76685357	0.00011325	0.00096807
ENSG000001Ş	FITM2	protein_codir	-0.76693455	0.0001848	0.00144921
ENSG000001З	TMBIM1	protein_codir	-0.76694212	0.00891452	0.03181703
ENSG000000Ş	CTTN	protein_codir	-0.76712546	0.00852726	0.03069502
ENSG0000017	CAMTA1	protein_codir	-0.76776763	0.00116695	0.00646302
ENSG0000004	GPM6B	protein_codir	-0.76816548	0.11472206	0.22334696
ENSG000001Ş	FXYD4	protein_codir	-0.76830235	0.7385362	NA
ENSG000002Z	THORLNC	lncRNA	-0.76941912	0.23812299	0.38223635
ENSG000002€	RP11-473M2C	lncRNA	-0.76964571	0.13605488	0.25334295
ENSG000002€	CTA-305I2.1	lncRNA	-0.76971428	0.79268463	NA
ENSG000001З	FAIM2	protein_codir	-0.76987174	0.0947134	0.19370486
ENSG000002€	PMF1-BGLAP	protein_codir	-0.77012119	0.00132183	0.00714742
ENSG000002З	RP1-60N8.1	lncRNA	-0.7703526	0.44319493	0.59421815
ENSG000002Z	RP5-1142A6.2	lncRNA	-0.77072009	0.01784995	0.05433277
ENSG0000011	UST	protein_codir	-0.77081858	0.0204297	0.06024646
ENSG000001C	CBY1	protein_codir	-0.77112946	0.00014607	0.00119934
ENSG000001Z	SSPN	protein_codir	-0.77241724	0.01293862	0.04247908
ENSG000002Ş	RP11-156E6.1	lncRNA	-0.77250652	0.02946212	0.08036181
ENSG000001€	PRYP3	protein_codir	-0.77277645	0.73879584	NA
ENSG000002Ş	RP11-196G11	protein_codir	-0.77286406	0.05335017	0.12634244
ENSG000002Ş	RP11-317N8.5	lncRNA	-0.77303418	0.00334811	0.0147582
ENSG0000007	P4HA2	protein_codir	-0.77318746	0.00169864	0.008685
ENSG000001Z	ST3GAL3	protein_codir	-0.77321178	0.00062247	0.00390921
ENSG000001З	CDK5RAP2	protein_codir	-0.77336694	4.37E-05	0.00043598
ENSG000002Ş	LLNLR-252D1Z	lncRNA	-0.77342489	0.32099881	0.47310498

ENSG0000024	CTD-2001C12	lncRNA	-0.77367278	0.47136307	0.62025003
ENSG0000028	RP11-421K1.1	lncRNA	-0.77370911	0.01444923	0.04630326
ENSG0000027	RP11-74E22.5	lncRNA	-0.77390391	0.7394885	NA
ENSG0000017	ZHX3	protein_codir	-0.77407367	0.01473482	0.04706553
ENSG0000025	RP11-732A19	lncRNA	-0.77475011	0.17561591	0.30581371
ENSG0000028	RP11-51J9.7	lncRNA	-0.77487699	0.00110597	0.0062014
ENSG0000015	C2CD6	protein_codir	-0.77497852	0.10596209	0.21071877
ENSG0000027	RIMBP3	protein_codir	-0.77510995	0.1136992	0.22188736
ENSG0000023	RP11-310H4.3	lncRNA	-0.77513206	0.62284729	NA
ENSG0000016	EOGT	protein_codir	-0.77538072	0.02248097	0.06493667
ENSG0000023	DIRC3	lncRNA	-0.77577013	0.04433228	0.10987596
ENSG0000011	CDK18	protein_codir	-0.77581251	0.00469736	0.01934053
ENSG0000010	CADM4	protein_codir	-0.77623389	0.02657009	0.07397842
ENSG0000019	TLE1	protein_codir	-0.77635088	0.00081652	0.00486789
ENSG0000018	SLC25A21	protein_codir	-0.77645913	0.22897667	0.37145878
ENSG0000024	RP5-966M1.6	protein_codir	-0.7768431	0.10480068	0.20898092
ENSG0000015	AUTS2	protein_codir	-0.7776206	0.02540037	0.07148544
ENSG0000023	YEATS2-AS1	lncRNA	-0.77796279	0.0412085	0.10384843
ENSG0000014	ATP8B2	protein_codir	-0.77826587	0.00463932	0.01916512
ENSG0000026	RP11-1299A1	lncRNA	-0.77829359	0.19648241	0.33238921
ENSG0000027	RP11-634H22	lncRNA	-0.77847497	0.31742777	0.46929424
ENSG0000017	CTD-2530N21	lncRNA	-0.77867069	0.22522107	0.3669974
ENSG0000000	PLEKHG6	protein_codir	-0.77868012	0.24860859	0.39426788
ENSG0000028	RP11-1396O1	lncRNA	-0.7790601	0.11541824	0.22440179
ENSG0000025	PCDHA3	protein_codir	-0.77953395	0.074581	0.16228845
ENSG0000015	RAB6B	protein_codir	-0.77962033	0.00052741	0.00341552
ENSG0000025	FAM85B	lncRNA	-0.7797461	0.24518207	0.39026791
ENSG0000016	ACOX2	protein_codir	-0.77994918	0.0042225	0.01778301
ENSG0000024	RP11-362A9.3	lncRNA	-0.78059384	0.81027625	NA
ENSG0000026	CTD-2278I10.	protein_codir	-0.78059384	0.81027625	NA
ENSG0000011	IFT43	protein_codir	-0.7806391	0.00085905	0.00507313
ENSG0000017	RNF150	protein_codir	-0.78147189	0.03490217	0.09133959
ENSG0000028	KB-1836B5.5	lncRNA	-0.78153852	0.45133413	0.60203544
ENSG0000028	SSU72P2	protein_codir	-0.78189422	0.79108735	NA
ENSG0000014	NUDT7	protein_codir	-0.78211694	0.00316581	0.01410213
ENSG0000012	XG	protein_codir	-0.78278908	0.14264189	0.26242383
ENSG0000024	CTD-2574D22	lncRNA	-0.78297463	0.01532351	0.04846687
ENSG0000026	RP11-370I10.	lncRNA	-0.78303766	0.09772859	0.19832039
ENSG0000018	TNFAIP8L3	protein_codir	-0.78308495	0.01278622	0.0420874
ENSG0000007	EVC	protein_codir	-0.78332465	0.01052883	0.03626466
ENSG0000019	RYR3	protein_codir	-0.78370657	0.15994467	0.28564098
ENSG0000010	CBX1	protein_codir	-0.7837585	0.00200072	0.00983447
ENSG0000028	RP11-23N11.	lncRNA	-0.78402509	0.26501467	0.41256369
ENSG0000028	PARTICL	lncRNA	-0.78433332	0.04740443	0.11557156
ENSG0000008	APLP2	protein_codir	-0.78452494	0.00611846	0.02370819
ENSG0000011	PCYOX1	protein_codir	-0.784691	0.01729449	0.05304539
ENSG0000018	UNC5C	protein_codir	-0.78510027	0.08956203	0.1855463

ENSG0000022	LINC01703	lncRNA	-0.78532573	0.10419844	0.20808047
ENSG0000013	NOVA1	protein_codir	-0.78549789	0.03447423	0.0905716
ENSG0000012	GNAZ	protein_codir	-0.78557294	0.117704	0.22769187
ENSG0000020	LINC00222	lncRNA	-0.78565453	0.10055608	0.20253474
ENSG0000027	KB-1125A3.12	lncRNA	-0.7856922	0.63090956	NA
ENSG0000011	GLS	protein_codir	-0.78573192	0.00223137	0.01071276
ENSG0000027	PDCD6IP-DT	lncRNA	-0.78581666	0.03700123	0.0955473
ENSG0000016	GKAP1	protein_codir	-0.78586778	0.01651679	0.05128909
ENSG0000027	RP11-477N3.1	lncRNA	-0.78607824	0.01591572	0.04991961
ENSG0000017	NEGR1	protein_codir	-0.78638112	0.06075994	0.13933343
ENSG0000028	LINC00506	lncRNA	-0.78652691	0.02123333	0.06205975
ENSG0000017	TTC36	protein_codir	-0.78673054	0.11599051	0.22522905
ENSG0000006	CACNB1	protein_codir	-0.78699288	0.00166026	0.00853144
ENSG0000019	CYSRT1	protein_codir	-0.78705727	0.14292905	0.26281533
ENSG0000025	RP11-356M20	lncRNA	-0.7871967	0.33865704	0.49149848
ENSG0000017	TUBB6	protein_codir	-0.78724306	0.01602665	0.05019388
ENSG0000022	AC012462.2	lncRNA	-0.78727651	0.31485548	0.46664029
ENSG0000025	MIR2052HG	lncRNA	-0.78763989	0.5472744	0.68502306
ENSG0000027	H4C14	protein_codir	-0.78770504	0.00805724	0.02940554
ENSG0000026	RP13-1032I1.1	protein_codir	-0.78820893	0.80113171	0.8765748
ENSG0000027	RP11-626G11	lncRNA	-0.78826687	0.17269784	0.30231425
ENSG0000012	SMO	protein_codir	-0.78841072	7.11E-05	0.0006576
ENSG0000023	ANKRD18B	protein_codir	-0.78863472	0.56943014	0.70313217
ENSG0000010	BEX4	protein_codir	-0.78949185	0.00025443	0.00188148
ENSG0000012	ADGRE5	protein_codir	-0.7896992	0.01554311	0.04897581
ENSG0000014	EVI5L	protein_codir	-0.78978907	3.84E-05	0.0003924
ENSG0000026	RP11-167N5.5	lncRNA	-0.78996459	0.23231505	0.3752408
ENSG0000010	ALDOC	protein_codir	-0.79022795	4.44E-06	6.41E-05
ENSG0000000	ENPP4	protein_codir	-0.79056095	2.34E-06	3.75E-05
ENSG0000027	CMP21-97G8.	lncRNA	-0.79107053	0.5528119	0.68937374
ENSG0000023	AP000688.14	lncRNA	-0.79110018	0.26328943	0.41068809
ENSG0000028	RP11-747B2.1	lncRNA	-0.79132802	0.10934371	0.21575033
ENSG0000026	LA16c-385E7.	lncRNA	-0.79143202	0.53762751	0.67736086
ENSG0000013	SIX3	protein_codir	-0.79174341	0.33289226	0.48563271
ENSG0000023	MID1IP1-AS1	lncRNA	-0.79184561	0.51290081	0.65651684
ENSG0000010	PHYH	protein_codir	-0.79187084	0.00376398	0.01618828
ENSG0000011	PLXNA1	protein_codir	-0.79190456	0.00252015	0.01175191
ENSG0000017	RNF152	protein_codir	-0.79205968	0.01874163	0.05641278
ENSG0000027	RP11-463O9.9	lncRNA	-0.79217268	0.35443374	0.50801557
ENSG0000017	MTLN	protein_codir	-0.79238946	0.00228616	0.01093019
ENSG0000018	TH	protein_codir	-0.79269149	NA	NA
ENSG0000028	CTB-53D8.2	lncRNA	-0.79316017	0.8077114	NA
ENSG0000026	LINC00514	protein_codir	-0.79316017	0.8077114	NA
ENSG0000025	RP11-94C24.8	lncRNA	-0.79372012	0.25265172	0.39887056
ENSG0000014	KIAA1755	protein_codir	-0.79378832	0.0022827	0.01091553
ENSG0000012	LRFN3	protein_codir	-0.79398406	0.00018369	0.00144175
ENSG0000025	RP11-328C8.5	lncRNA	-0.79401318	0.78899601	NA

ENSG0000028	SOD2-OT1	lncRNA	-0.79404238	0.12216577	0.23428719
ENSG0000026	ARHGAP44-AS	lncRNA	-0.79404285	0.59799112	0.72619217
ENSG0000025	RP11-115C10.	lncRNA	-0.79413543	0.7864723	NA
ENSG0000020	RP11-514P8.6	protein_codir	-0.79414131	0.31857699	0.47056508
ENSG0000016	LY6K	protein_codir	-0.79424237	0.054464	0.12830785
ENSG0000022	RP3-441A12.1	lncRNA	-0.79456441	0.679988	NA
ENSG0000019	HMGN5	protein_codir	-0.79491993	0.01089267	0.03726393
ENSG0000006	PPP2R5B	protein_codir	-0.79496441	3.68E-05	0.00037919
ENSG0000016	BEND5	protein_codir	-0.79514829	0.02352364	0.06721734
ENSG0000027	RP11-362K14.	lncRNA	-0.79524271	0.22051684	0.36130295
ENSG0000010	UBL4A	protein_codir	-0.79529774	3.09E-05	0.00032803
ENSG0000016	KIF5C	protein_codir	-0.79551894	0.00499564	0.02024942
ENSG0000023	LINC00486	lncRNA	-0.79628161	0.68182123	NA
ENSG0000014	SLC27A3	protein_codir	-0.79628653	0.000174	0.00138255
ENSG0000013	ZBED3	protein_codir	-0.79630053	0.00078715	0.00472424
ENSG0000025	POLR2M	protein_codir	-0.79657713	0.00245795	0.01152999
ENSG0000010	KCTD9	protein_codir	-0.7967367	0.02334261	0.06683144
ENSG0000028	RP11-418H16	lncRNA	-0.79698217	0.24134743	0.3860329
ENSG0000013	ADGRB3	protein_codir	-0.79733127	0.10106025	0.20338707
ENSG0000017	CRTAP	protein_codir	-0.79763512	0.00946161	0.03332321
ENSG0000019	CALM1	protein_codir	-0.79813111	0.00277253	0.01268047
ENSG0000010	UGGT2	protein_codir	-0.79842561	0.00116752	0.00646491
ENSG0000025	ZNF625	protein_codir	-0.79879918	0.01858011	0.05604879
ENSG0000025	CTC-461F20.1	lncRNA	-0.79889677	0.77325457	NA
ENSG0000006	ASB1	protein_codir	-0.79893576	0.00034486	0.00241784
ENSG0000018	C11orf96	protein_codir	-0.79893825	0.19648511	0.33238921
ENSG0000016	C19orf47	protein_codir	-0.79908781	0.00054427	0.00350918
ENSG0000018	THNSL1	protein_codir	-0.79910723	0.00652912	0.02496322
ENSG0000026	RP11-20I23.7	lncRNA	-0.79927328	0.73306369	NA
ENSG0000016	LSS	protein_codir	-0.800477	0.00036005	0.00250639
ENSG0000024	RAD21L1	protein_codir	-0.80050785	0.68507508	NA
ENSG0000025	RP11-27M24.	lncRNA	-0.80060163	0.64462598	0.76234509
ENSG0000011	B4GALT2	protein_codir	-0.80084409	0.00067989	0.00420198
ENSG0000012	HSPA2	protein_codir	-0.80084561	0.05050923	0.12148958
ENSG0000027	RP11-50D16.4	lncRNA	-0.80084561	0.37438071	0.52797639
ENSG0000026	DDX59-AS1	lncRNA	-0.80087355	0.38683257	0.53985728
ENSG0000027	MROH7-TTC4	protein_codir	-0.80109292	0.25157078	0.39769813
ENSG0000026	CTD-3214H19	protein_codir	-0.80145917	0.73051946	0.82661918
ENSG0000017	DPY19L2	protein_codir	-0.80151714	0.04074335	0.10288274
ENSG0000017	C9orf106	lncRNA	-0.80163896	0.00128705	0.0069977
[truncated: 313,585 more chars]
