# Supplementary material for: Identification of endoplasmic reticulum stress-associated lncRNAs influencing inflammation and VSMC function in abdominal aortic aneurysm
Source: Clin Sci (Lond). 2025 Mar 25;139(6):357–72. doi: 10.1042/CS20242476 (PMC12204013; doi:10.1042/CS20242476)
Supplement: Supplementary Table S4 [file CS-139-06-CS20242476-s005.pdf]

Table S4. RNAseq analysis of aorta from AAA patients and donors. lncRNA expressed in aortic samples including in GO term response to mitochondrion (GO:00005739).

ID		
GO00005739(7634 elements)	lncRNA upregulated in AAA (165 elements)	lncRNA downregulated in AAA (112 elements)
H19	H19	LOXL1-AS1
UCA1	RP11-326C3.2	CRNDE
AC005082.12	CTC-281F24.1	RP11-527N22.1
LOXL1-AS1	IL21-AS1	RP11-416N4.4
RP11-158I13.2	LINC00582	BVES-AS1
RP11-57H14.2	RP5-1120P11.3	ALDH1L1-AS2
CTD-3199J23.4	PRKAG2-AS1	RP11-893F2.5
CRNDE	AC124789.1	AC007563.5
C11orf95	RP3-439F8.1	ARMCX3-AS1
NEFL	RP11-553L6.2	KB-1410C5.5
RP11-680F20.5	LBX2-AS1	ZNF503-AS2
XKR5	RP11-142C4.6	RP11-346C20.4
RP11-834C11.4	RP1-8B1.4	RP11-554A11.4
HOXC-AS3	RP11-452K12.4	AP000318.2
AF131216.5	RP11-615I2.2	RP11-316O14.1
SNHG1	RP11-390P2.4	RP11-795J1.1
RP11-334A14.8	RP11-380I10.4	RP11-145A3.1
HOTAIR	RP3-455J7.4	LINC00310
GS1-358P8.4	RP11-293M10.6	RP11-735G4.1
SNHG7	CTD-2006K23.1	LINC00472
CTD-2540L5.5	CACNA1C-AS1	MEIS1-AS2
CTD-2516F10.2	IGF2-AS	LINC00327
ENTPD1-AS1	RAMP2-AS1	CTD-2207P18.2
RP11-480I12.7	AC132217.4	CTC-308K20.1
RP11-94L15.2	LEF1-AS1	DACT3-AS1
RP5-1148A21.3	RP11-66B24.2	RP11-367F23.1
AP000688.14	RFPL1S	RP11-613D13.8
RP13-238F13.5	AC002456.2	RP1-140K8.5
RP11-527N22.1	AP001057.1	CACTIN-AS1
RASAL2-AS1	RP11-383J24.1	RP4-584D14.7
PTCHD3P1	AE000661.37	RP11-21L23.3
TPTEP1	GS1-72M22.1	RP11-1134I14.8
ZNF518A	AC021188.4	CTB-140J7.2
RP11-217B1.2	FENDRR	RP11-626H12.1
RP11-384O8.1	CTD-2154I11.2	RP11-332H18.5
AC009531.2	DBH-AS1	RP11-545A16.4
RP1-59M18.2	CTD-2561B21.11	RP11-92A5.2
RP11-473M20.16	RP11-284F21.10	AC079779.4
EPB41L4A-AS1	CTD-2369P2.8	AC005618.6
LINC00667	CTB-41I6.2	RP11-158I9.5
EHHADH-AS1	CHL1-AS2	AF186192.1
RP11-236P13.1	AC093642.4	RP11-999E24.3
CYP1B1-AS1	CTC-378H22.1	CTD-3064M3.3
CTD-2269F5.1	RP11-527N22.2	LINC00454
AC005562.1	AC004947.2	RP11-680F8.3
LINC00116	AC013463.2	LINC00691
AC005307.3	RP1-212P9.3	RP11-389G6.3
CBR3-AS1	RP11-543C4.1	JAZF1-AS1
MAGI2-AS3	RP11-834C11.10	LINC00652
MIR17HG	AC058791.1	RP11-543D5.2
ANKRD62P1-PARP4P3	FAM225A	CALML3-AS1
DANCR	RP11-297B17.3	KB-1448A5.1
RP11-622K12.1	AC131056.3	AC010907.5
SEC22B	RP11-134G8.7	CTD-2523D13.2
AC105344.2	LINC00494	SSTR5-AS1
RP11-384P7.7	RP11-164H13.1	CTD-2541J13.1
SPON1	KB-1471A8.1	RP11-9G1.3
RP11-1094M14.11	LINC00861	SMIM2-AS1
RP11-220I1.1	RP11-266O8.1	ARHGEF7-AS2
SNHG5	RP11-96K19.4	RP11-420L9.5
RP6-24A23.7	RP11-863P13.3	DLG5-AS1
ZEB1-AS1	CTD-2547L24.4	RP11-16N11.2
RP11-436H22.1	RP11-645C24.5	RP11-284H19.1
RP11-206L10.11	PVT1	RP1-223B1.1
HOXD-AS1	RP11-15A1.3	RP11-307B6.3
RP11-38P22.2	AC008697.1	RP11-867G23.10
RP11-64P12.8	RP11-430C7.5	LINC00592
LINC00839	PRKCQ-AS1	AC106786.1
AC108051.2	RP11-21C4.1	SERTAD4-AS1
TDRG1	RP11-16E12.2	RP11-800A3.7
AC009299.3	RP11-730K11.1	FOXD2-AS1
AC011752.1	AC007278.3	AL022344.7
VTRNA2-1	KB-173C10.1	RP11-311F12.1
NEAT1	WT1-AS	PCAT7
RP11-224O19.2	AC022182.3	FGF14-AS2
RP11-1252I4.2	RP11-44F14.2	RP11-71H17.7

RP4-604G5.1
RP11-245A18.1
CTB-58E17.2
AC079630.4
CTD-2265O21.3
RP11-475O6.1
linc-NSMCE4A-2
RP11-329B9.1
PRSS51
RP5-881L22.6
CTD-2555C10.3
RP11-386D6.1
RP13-122B23.8
RP1-102K2.6
linc-SYT12
CYP17A1-AS1
linc-CSTB-9
AC007950.1
linc-MRPL33
RP11-632K5.3
RP11-37N22.1
linc-FAM92B-1
RP11-375B1.2
linc-CSNK1E
linc-ARHGAP5
AC079988.3
AC090627.1
RP11-135A1.2
RP5-1029K10.2
RP11-462G12.1
linc-LDOC1L-1
AC010987.6
RP11-624M8.1
linc-OTUD7B-1
RP11-304F15.4
RP11-416N4.4
CTD-3025N20.2
LINC00543
RP4-640H8.2
AP000696.2
linc-CARS2-2
linc-TMEM72-2
RP11-1055B8.3
PADI6
GS1-594A7.3
linc-CD2AP-2
linc-HDDC2-4
linc-C17orf108-5
AC011523.2
RP11-60L3.2
linc-DHX30
linc-ANP32A-2
RP1-153P14.8
RP11-326C3.2
GATA3-AS1
CTC-347C20.2
linc-PHF17-1
CYP4F8
RP11-445H22.3
AC067969.1
RP11-115K3.1
linc-TMEM206-2
VPS13A-AS1
SNORA40
RP11-673F18.1
RP11-269F19.2
linc-PGS1-3
RP11-70F11.11
LINC00941
RP11-305L7.3
RP11-148O21.6
RP11-173B14.5
CTB-41I6.1
BZRAP1-AS1
linc-SECTM1-2
RP11-702H23.4
CTC-281F24.1
RP11-433J20.2
linc-CORO1C
RP11-373D23.2
RP11-902B17.1
RP11-96K19.2
LINC00626
RP5-1028K7.2
RP11-326C3.15
RP1-244F24.1
MIAT
FAM201A
RP11-519G16.3
AC004540.5
RP11-1094M14.5
AC009495.2
RP11-758N13.1
CTB-114C7.4
TRBV11-2
LINC00892
RP11-439L18.1
RP11-379K17.4
RP11-363D14.1
RP11-945C19.4
RP11-160E2.19
CTD-2342N23.3
RP11-482H16.1
PCAT1
RP5-968J1.1
WDR86-AS1
RP11-161M6.3
LINC00299
RP11-390F4.3
RP11-138I18.2
LINC00900
RP11-25K19.1
TCL6
AC112721.2
RP3-467K16.4
U62631.5
RP11-799O21.2
HOXA11-AS
RP11-495K9.6
RP11-33A14.1
RP11-1036E20.9
RP11-13P5.2
RP11-1151B14.4
RP11-598F7.5
RP11-981P6.1
LINC00841
RP11-202G18.1
RP11-214O1.2
RP13-452N2.1
DIO3OS
RP11-147L13.8
BX255923.3
LINC00937
RP4-555D20.2
RP11-624C23.1
RP11-43F13.3
RP11-87G24.6
NR2F1-AS1
AC007255.8
RP1-153P14.5
RP3-395M20.9
CTC-378H22.2
RP11-53I6.2
RP11-491F9.1
AC104024.1
RP11-408O19.5
LINC00544
LINC00920
AC022182.1
RP11-60A8.1
RP11-16K12.1
RP11-13P5.1
RP11-598F7.6
CACNA1C-AS2
LINC00877
ITGB2-AS1
RP11-598F7.3
LY86-AS1
RP11-760H22.2
RP11-203E8.1
RP3-325F22.5
SLC26A4-AS1
RBPMS-AS1
RP11-157J24.2
SH3RF3-AS1
RP11-71E19.1
AC093390.1
LINC00865
AC067959.1
RP11-879F14.2
RP11-857B24.5
RP11-483L5.1
AC007405.4
AC003986.6
ARHGEF26-AS1
RP11-400K9.4
AC007405.6
RP11-680F8.1
ADAMTS9-AS2
RP11-731J8.2
HAND2-AS1
GATA6-AS1
RP11-344B5.4
FGF13-AS1
AC006262.6
THRB-AS1
BX470102.3
RP11-92C4.6
AC016738.4
MAST4-AS1
SLC2A1-AS1

LINC00272	RP11-119J18.1
RP3-434P1.6	RP11-203B7.2
AC133680.1	RP11-532F6.3
IL21-AS1	LINC00426
RP11-230G5.2	RP11-536O18.1
linc-GPR157-2	MIR137HG
RP11-418J17.3	CTD-2540L5.6
RP1-90K10.4	RP11-567J20.3
linc-P2RX7	CTC-523E23.1
linc-NEURL1B-3	
RP11-429K17.1	
RP11-469H8.8	
RP11-1090M7.1	
OSTM1-AS1	
RP11-204E9.1	
RP11-488C13.4	
linc-CNNM1	
linc-KLF6-7	
RP11-554A11.6	
BVES-AS1	
LINC00582	
RP11-154H12.3	
RP1-76B20.11	
CTA-150C2.13	
ALDH1L1-AS2	
RP5-1120P11.3	
linc-GPRC5C	
RP11-422J8.1	
RP11-613D13.5	
BSN-AS2	
linc-FAM20A-2	
RP11-432I5.1	
RP11-463O9.9	
linc-PCBD1-2	
RP11-404O13.1	
RP11-867G23.4	
AC017116.11	
AC002064.5	
RP11-805I24.4	
RP5-827C21.2	
AC004463.6	
LINC00671	
RP11-893F2.5	
AC053503.6	
RP11-57A19.2	
linc-TLE3-1	
RP11-298D21.3	
RP11-66B24.5	
RP11-2N1.2	
RP11-213G2.2	
AC018731.3	
RP11-893F2.13	
RP1-13P20.6	
PRKAG2-AS1	
RP11-88I21.2	
linc-SSTR1-3	
RP11-720L2.4	
AC124789.1	
RHPN1-AS1	
linc-SHPRH-9	
RP11-82L18.2	
AC034228.2	
CTC-277H1.7	
CTC-525D6.1	
ADARB2-AS1	
RP3-512B11.3	
PCAT6	
AC012462.1	
RP1-293L8.2	
RP11-62F24.1	
RP4-781K5.2	
RP11-399K21.11	
linc-HELT-4	
linc-MAP1LC3B2-13	
RP11-64C12.6	
RP11-19E11.1	
RP5-1170K4.7	
linc-SOX17-1	
RP11-701P16.4	
RP11-84C10.3	

MIR143HG
AC007563.5
AP000438.2
RP11-523L20.2
RP11-108M9.6
AC006994.2
RP5-834N19.1
TINCR
linc-FOXF1-6
RP11-16C18.3
CTC-246B18.8
RP11-297D21.2
AL163195.3
RP11-103B5.2
linc-ZFHX3-1
linc-TANC2-1
AC013275.2
AC073342.12
AC002480.4
RP11-305O6.3
linc-C1QTNF3-1
RP11-66B24.4
RP11-21A7A.2
linc-ALDH6A1
linc-CPEB4-5
AC067969.2
RP11-543F8.2
linc-PSORS1C1-2
AC005625.1
ST3GAL6-AS1
CACNA1C-IT2
RP11-420L9.4
GS1-122H1.2
CTA-250D10.23
LINC00917
RP11-806H10.4
linc-CABP2
RP11-466C23.4
linc-HPDL
RP11-503C24.6
RP11-101E7.2
AC003958.2
RP13-392I16.1
AC144836.1
RP11-444D3.1
AC139100.3
CTC-255N20.1
linc-GINS2
AC005597.1
linc-GRHL2-4
linc-RIPK4
CTC-281M20.1
FER1L6-AS2
linc-HOXB9
RP11-208K4.1
RP3-393E18.2
RP11-419J16.1
linc-WISP3-2
CTB-60E11.9
RP11-13G14.4
AC128709.4
FLG-AS1
FER1L6-AS1
RP1-293L6.1
linc-SSBP3-1
linc-DIRAS2-5
RP11-6N17.2
RP1-118J21.5
RP5-1029F21.3
linc-TRIM52
RP11-99E15.2
RP11-5P18.5
AE000661.50
RP11-331K21.1
CITF22-24E5.1
linc-PDE6B
linc-KIF16B-4
CTD-2134A5.4
RP11-156K13.3
CTD-2033C11.1

linc-CMC1-2
LINC00229
RP3-439F8.1
linc-COL6A1
RP3-510D11.1
RP11-281J9.2
CTB-58E17.1
CCDC13-AS1
linc-HOXA13-1
AC002480.5
RP1-90G24.6
RP11-96D1.6
SAA2-SAA4
RP11-62F24.2
RP3-437I16.1
linc-FAM43A-5
RP11-68I18.10
RP1-166H1.2
linc-HLF
AP001625.5
RP5-1070G24.2
RP11-329J18.2
CTD-2194A8.2
AC140912.1
RP5-881L22.5
HLA-AS1
RP1-179N16.6
linc-CDR2L
CTC-439O9.3
linc-C22orf26
RP11-442N1.1
ARMCX3-AS1
AF038458.5
RP11-343J3.2
FEZF1-AS1
linc-LHFP
CTD-2024P10.1
RP11-395N3.1
LINC00340
RP11-64C12.8
RP11-589C21.5
GS1-122H1.1
RP11-59J16.2
linc-CD5L-2
AC103563.8
linc-FOXC1-1
RP11-805I24.3
HOXA-AS3
LINC00967
DGUOK-AS1
DNM3-IT1
RP11-252A24.5
RP11-798K23.4
RP11-59N23.3
LINC00577
RP11-125I23.3
RP11-35J10.5
RP11-244F12.3
linc-C11orf35
RP11-553L6.2
RP11-386B13.3
RP3-468B3.3
linc-FOXF1-5
XXcos-LUCA11.4
KB-1410C5.5
linc-PSMG4
RP3-468B3.2
AC098617.1
CTD-2521M24.5
RP11-620J15.2
linc-NMS-2
CTC-359M8.1
RP11-893F2.6
linc-OR2AG1-2
linc-MCM2-1
KIRREL3-AS1
RP11-469H8.6
RP11-680F20.4
RP1-40E16.11
LINC00887

RP11-221N13.3
linc-DIRAS2-4
HCG21
RP11-222K16.2
RP11-383C5.3
RP11-498C9.17
linc-TRYX3-1
RP3-414A15.10
TBC1D3P1-DHX40P1
RP5-1119A7.10
linc-CNOT6-1
RP11-442N1.2
RP3-425P12.2
RP11-128M1.1
AC140542.2
RP11-412B14.1
RP3-323P13.2
linc-KLK15
linc-VSTM2B-4
RP11-429B14.1
linc-LRP12-2
linc-THPO-2
AC131025.8
RP11-538P18.2
RP11-420G6.4
linc-ZP2
linc-GPR157-3
RP11-386B13.4
ZNF503-AS2
RP11-225H22.5
linc-GRHL2-8
RP11-326A19.4
linc-ANKRD50-2
RP11-557H15.5
linc-SPR-1
RP5-1109J22.2
RP3-340B19.3
RP11-390F4.6
CTD-2353F22.1
RP11-429J17.7
AC013472.4
RP11-255G12.2
RP11-494M8.4
linc-CTNNBL1-2
RP11-493K19.3
MIR1470
CTD-3193O13.1
linc-IDH3A
linc-KCNE2-1
RP11-1082L8.2
AC008703.1
linc-TAGAP-1
CTB-147C22.9
linc-COX4NB-3
RP11-212E8.1
AC114765.1
linc-LHX1-2
RP11-674N23.1
CTD-2534I21.8
RP11-698N11.2
linc-ESR2
linc-CAPRIN2
AC073934.6
RP11-66D17.3
LBX2-AS1
linc-MMP2
linc-SUPT3H
CTC-273B12.10
linc-CCDC140-8
linc-ADC
CTD-2189E23.1
RP11-605F22.1
RP1-4G17.2
RP1-128O3.4
CTD-3035D6.2
RP1-140K8.1
CTA-342B11.2
LINC00087
CTD-2568A17.5
RP11-820L6.1

RP3-510D11.2
RP11-728G15.1
RP11-688I9.4
AC009236.1
RP11-346C20.4
CTD-2555A7.1
RP5-905G11.3
AC099754.1
RP11-554A11.4
CTD-2521M24.11
SOCS2-AS1
RP11-416I2.1
linc-BBC3
RP11-142C4.6
linc-ARSI
RP1-127L4.7
KB-1930G5.4
RP11-431K24.3
RP11-112J1.2
RP11-367J11.3
RP1-8B1.4
hsa-mir-6080
RP11-27N21.3
KCP
CTD-2587H24.10
RP4-594I10.3
linc-SVIL-1
AC078883.4
RP1-7G5.6
RP5-1027G4.3
CTD-2105E13.15
linc-CLN5-4
RP11-770J1.3
linc-C19orf12-1
RP11-885B4.2
RP11-324O2.3
RP11-299P2.1
linc-F13A1-1
linc-NCOA1-1
RP11-506H20.1
ATP13A5-AS1
RP3-467K16.2
RP11-973N13.3
RP11-141O11.1
RP11-138M12.1
RP11-697E22.2
RP11-662I13.2
linc-PXN
PXN-AS1
AP000318.2
RP11-452K12.4
RP11-1079K10.5
LL09NC01-254D11.1
RP11-1069G10.2
linc-ADARB2-5
linc-ONECUT1
linc-IL6-1
linc-KIAA1737-3
AC000124.1
CTB-75G16.3
PPP2R2B-IT1
RP11-404O13.5
TMEM51-AS1
linc-ZCCHC17-9
linc-AKR1E2-17
linc-ALDH1A3-1
linc-SPTBN1-1
CTD-2308N23.2
RP5-1160K1.8
CTC-529G1.1
RP4-535B20.1
RP11-58B17.2
AC073072.5
APOBEC3B-AS1
CTD-2377D24.4
RP1-40E16.2
RP1-76B20.12
RP11-1094H24.4
HCCAT5
AL161668.5

AC007392.3
AC074389.7
linc-BLK
linc-CDC25A
RP11-358M14.2
RP11-375B1.1
RP11-536G4.2
linc-CLPB
RP3-496C20.1
linc-PPP1R16A
AP000662.4
AC053503.11
RP11-605F22.2
RP11-105C19.1
CTD-2311M21.3
RP11-243E13.1
RP11-148G20.1
linc-MRPS18A-4
RP11-2I17.4
RP11-511P7.2
linc-CYP11B2
CTD-2616J11.16
LINC00851
BPESC1
AC051649.16
linc-C15orf42-1
RP11-780O24.2
AC012531.23
RP11-98D18.9
linc-PPARGC1A
RP11-323C15.2
RP11-356I2.4
linc-TATDN3
linc-TTC24-1
linc-ADARB2-7
linc-PPP1R3B-1
linc-CREBBP
CTD-2168K21.2
RP11-196G18.21
RP3-473L9.4
linc-CCR8-3
RP11-317P15.4
RP11-567L7.6
RP11-346C20.3
RP11-316O14.1
RP11-131N11.4
SLC38A3
RP11-363J20.2
CROCCP2
RP11-136K14.2
CTD-2337A12.1
CTC-349C3.1
CTD-2131I18.1
RP11-209D14.2
RP11-964E11.2
linc-C17orf72
RP11-757G1.6
AC073128.10
RP11-432I5.2
linc-KCNG2
RP11-510M2.5
RP11-551L14.4
RP11-231E6.1
RP11-567J24.4
RP11-597M17.1
RP11-118B18.1
linc-SPTBN1-3
RP1-310O13.12
RP11-480I12.10
RP11-615I2.2
linc-SDSL
RP11-327L3.3
linc-C2orf60-2
linc-CLRN2-3
ARHGAP5-AS1
RP11-96C21.1
RP11-390P2.4
RP11-699L21.1
linc-COX4NB-7
RP11-673E11.2

RP11-82C23.2
HTR3E-AS1
SMCR2
CTD-2135J3.3
linc-NOP14-1
CTB-158E9.1
MAFG-AS1
RP11-114H24.3
RP5-1119D9.4
AC104695.3
linc-PLEKHO2-1
linc-SATB2-2
AC005041.17
RP11-501C14.8
linc-MARK1-1
RP11-129J12.2
linc-EPHB3-2
RP11-795J1.1
CTD-2325P2.4
RP11-307C19.2
RP4-550H1.4
RP11-284F21.9
RP11-440G5.2
CTC-228N24.2
linc-NKX2-5-1
RP11-885B4.1
RP11-145A3.1
RP11-317P15.3
RP11-696D21.2
linc-PRDM1-2
RP11-589C21.6
linc-FHL2-2
AP000697.6
SEMA3B
linc-FLJ44606-2
RP11-514F3.4
linc-MED1
RP11-293P20.4
RP11-380I10.4
LINC00598
RP11-7F17.4
linc-KIAA0232
AC003102.3
linc-COIL-4
RP11-70F11.8
RP11-560I19.1
CTA-384D8.31
RP11-4M23.3
CACNA1G-AS1
RP1-287H17.1
RP11-83J21.3
linc-IFITM5
RP11-138H8.6
HOXB-AS2
RP11-219G17.4
RAPGEF4-AS1
RP11-893F2.14
RP11-834C11.5
CTD-2377D24.8
RP11-104H15.8
LINC00505
RP11-903H12.3
linc-MIB2
linc-FGF9-1
linc-ABHD6
RP11-426L16.9
linc-TNFRSF19-1
linc-ZKSCAN1-1
linc-MECR-5
AC011526.1
linc-CCDC37-5
linc-NQO2-2
RP11-830F9.7
RP11-1070A24.2
RP11-186F10.2
RP11-132A1.4
RP4-550H1.5
linc-GRHL2-1
linc-HSD17B12-1
linc-NUDCD2-5

RP11-809M12.1
CTD-3194G12.2
linc-ZNF366-5
RP11-449J10.1
RP11-727A23.5
RP11-542M13.3
RP1-71H24.1
RP11-445H22.4
RP3-455J7.4
RP11-10N16.3
RP5-930J4.2
RP11-94C24.11
STEAP2-AS1
RP11-293M10.6
AC005606.14
linc-TPCN2
linc-KRAS
linc-OSR1-1
linc-TCF7L2-1
SNAP25-AS1
RP1-37N7.1
ZNF503-AS1
linc-BSPRY
RP11-104H15.9
linc-CHRD
LINC00928
RP11-452J13.1
linc-CPT2-1
LINC00310
AP001059.5
linc-PPP1R3B-2
linc-CPEB4-8
linc-CD5L-1
VIPR1-AS1
linc-MTHFSD-1
RP11-256L11.1
RP1-28O10.1
linc-MTMR9
linc-ZFAND3-2
RP11-319G6.3
AP006285.6
RP11-571M6.13
linc-ARL16-1
RP11-720L2.2
RP11-326C3.7
RP11-735G4.1
DLG1-AS1
RP11-394O4.3
RP11-309N17.4
RP11-377D9.3
CTD-2006K23.1
RP11-5N11.6
linc-CLP1
LA16c-444G7.1
linc-RREB1-3
LINC00472
LINC00601
FAM222A-AS1
linc-HRCT1
MEIS1-AS2
linc-CSTB-6
RP1-144F13.3
RP11-716D16.1
AF001550.7
RP11-386G11.5
RP11-214C8.2
RP11-541G9.2
linc-WDR5
AC024560.2
RP11-305L7.6
RP11-132A1.3
RP11-306I1.2
linc-PTK2B
RP11-624G17.3
linc-COX4NB-4
CTD-2201G3.1
linc-SARS
RP11-770G2.2
AC074011.2
RP11-867G23.3

linc-DHRS4-2
RP11-106M3.3
RP11-375B1.3
DLX6-AS1
linc-NEDD4L-1
AC006482.1
XX-DJ76P10__A.2
RP11-100E13.1
CTD-2231H16.1
MEIS1-AS1
CACNA1C-AS1
linc-ATP13A4-8
RP5-881L22.4
AC025165.8
RP3-449H6.1
CRYM-AS1
RP11-514D23.2
RP11-469L4.1
KB-1410C5.3
LINC00640
LINC00487
linc-TCP10-2
RP4-799P18.2
RASSF8-AS1
linc-KLK3-2
RP3-414A15.11
RP1-60O19.1
RP11-762H8.3
RP11-554A11.5
AC007796.1
RP11-256I23.1
CTB-111F10.1
CACNA1C-IT3
DLX6-AS2
linc-SH2D7-2
AC034228.3
linc-AKR1E2-5
UPK1A-AS1
CTC-558O2.1
RP11-688I9.2
RP11-379F12.3
AC104801.1
AC092431.3
AD000091.2
RP11-523H20.3
RP11-534L6.2
linc-DHRS2-2
RP11-298E9.6
linc-TP53
AP001625.4
RP11-433J22.2
RP11-501C14.5
RP11-65M17.3
Z83851.1
XXbac-BPG181B23.4
AC092614.2
AC004775.5
CTD-2315E11.1
RP11-486L19.2
linc-EXOC7
linc-ELK3-1
LL22NC01-81G9.3
AC004051.2
RP11-893F2.15
linc-SGTB-3
RP11-456D7.1
AC114730.5
IGF2-AS
GFOD1-AS1
RP11-807H22.7
linc-EMP1-1
RP11-26L20.4
LINC00327
RP5-858B6.3
KB-1562D12.1
RP5-1059M17.1
RAMP2-AS1
RP11-229C3.2
RP11-69H7.2
linc-FOXF1-4

RP11-196G18.3
linc-ATG5-2
RP11-251M1.1
RP13-238F13.3
linc-NGFR-3
AC051649.12
AC132217.4
linc-CYP24A1-3
RP11-321G12.1
RP11-1112C15.1
linc-COX4NB-1
linc-ELFN1
RP11-481J13.1
RP11-148O21.3
RP11-706C16.7
AC005682.5
RP11-542K23.10
RP6-65G23.3
RP11-74C1.4
CTB-189B5.3
linc-STXBP1-1
SERHL
RP11-139I14.2
linc-CTNNA1-1
RP11-227H15.5
linc-CD1D-2
RP11-863K10.2
RP5-1119A7.17
AC008992.2
linc-ADRA1B-2
linc-ZCCHC17-5
RP11-373D23.3
SATB2-AS1
AC019185.4
LGALS8-AS1
linc-HES1-1
CTB-32O4.2
LINC00954
linc-CETN1-2
AC000067.1
RP4-537K23.4
AC012613.2
RP11-359I18.5
CTC-518B2.9
linc-RNFT2-1
LINC00840
CTB-52I2.5
linc-UBR2-2
CTD-2207P18.2
linc-GRHL2-3
LEF1-AS1
RP11-276H7.2
RP11-959I15.4
linc-HOXC13-3
RP11-736K20.6
RP11-202I11.2
linc-TRAK1
AL450992.2
AC004112.4
AP001626.1
RP11-221N13.4
RP5-866L20.1
AC005740.6
RP11-169D4.1
AC137723.5
linc-PICK1
AC097468.7
RP11-632K5.2
linc-NDUFV3-3
HOXB-AS1
linc-EFHA1-5
AC006126.4
RP11-783K16.10
RP4-726N1.2
linc-SUCLG1
linc-TGM3-1
LINC00925
RP11-415J8.3
RP11-280K24.4
linc-MPPE1-3

RP11-513G11.4
RP11-1055B8.4
RP11-61J19.2
RP11-44N21.4
RP11-485O10.3
RP11-64D24.4
CTD-3194G12.1
linc-PITRM1-3
linc-ZBTB32
linc-MEGF10
AC093642.6
linc-CHRNB4
linc-ZNF716-8
linc-SLC25A26-1
MIR10A
RP11-770G2.4
linc-BTRC-2
RP11-209K10.2
RP11-944L7.4
RP11-219E7.4
RP11-506B6.6
RP11-1094H24.3
linc-KIAA1486-4
RP11-509E16.1
GS1-24F4.2
linc-C6orf223
linc-RLN2
RP11-445F12.1
AC087491.2
RP11-61A14.4
RP5-1029K10.4
linc-MYST3-1
RP11-66B24.2
RP1-67M12.2
RP11-505E24.2
CTC-308K20.1
RP11-285A1.1
HOXB-AS4
DACT3-AS1
RP11-371A19.2
RP11-386G11.3
RP5-1182A14.5
CECR3
ATP13A4-AS1
linc-JAKMIP3-1
RP1-159G19.1
RP11-367F23.1
RP11-399K21.10
RP11-150O12.6
RP11-59J16.1
linc-TMEM179
RP11-680F20.9
RP11-438N16.1
RP11-685G9.2
RP11-417L19.2
linc-COL1A1-1
RP11-284F21.8
linc-MFSD2A
RP11-98D18.17
CTD-2081C10.1
RP11-579D7.4
RP3-416H24.4
FGF12-AS2
RP11-113O24.3
RP11-116N8.1
RFPL1S
RP5-1101C3.1
EVX1-AS
linc-ZBTB25
AP000251.2
RP11-60A24.3
RNF157-AS1
RP11-706C16.5
AC002456.2
AP001057.1
RP1-90J20.2
RP11-973N13.4
AC002115.5
RP11-613D13.8
CTB-174D11.1

linc-DIRAS2-2
AC010136.2
RP11-501C14.6
linc-EML6-3
linc-ENTPD1-3
RP11-431K24.1
CTD-2024P10.2
ZNF32-AS3
linc-CCDC37-4
OOEP-AS1
LINC00519
linc-RASL12
RP11-379F12.4
RP4-705F19.1
linc-NAE1-2
RP11-99J16__A.2
HOTTIP
linc-GPR33
RP11-341N2.1
linc-C1orf151-1
linc-SAFB-2
linc-SERPINB6-2
RP11-57H14.3
RP11-521M14.1
RP11-117D22.1
CTB-109A12.1
AC002451.3
AC138430.4
RP11-285G1.2
linc-GSDMD-1
RP11-383J24.1
linc-SH3BGRL2-1
RP1-97J1.2
RP5-921G16.1
AE000661.37
linc-COL13A1
CTC-525D6.2
RP11-1E4.1
linc-NCAM1-2
RP1-38C16.2
linc-C6orf203-1
RP11-286H14.6
RP1-140K8.5
RP11-35J10.4
linc-GPATCH4
AC009955.8
CACTIN-AS1
RP11-680F20.11
linc-PDZD7
HOXB-AS5
RP11-154H12.2
RP11-395G23.3
linc-ATOH8
linc-MTIF3
RP11-405F3.5
RP11-61E11.2
LINC00534
KB-1460A1.2
linc-GNB4-2
RP11-715G15.1
FAM99B
LINC00208
KB-1460A1.1
RP11-706C16.8
linc-PDLIM3
RP11-177H2.2
AC128709.2
GS1-72M22.1
AC005895.4
linc-FLJ44606-1
RP11-566K11.4
linc-KIAA1257
SOX2-OT
RP11-475N22.4
linc-AIM1-2
linc-SLAMF9
linc-WDR26-2
RP11-532F12.5
RP4-681L3.2
RP11-304C12.3

RP11-982M15.8
AC144450.2
linc-C17orf76
AC113607.1
RP11-579O24.3
AC021188.4
CTC-459I6.1
RP11-439E19.7
RP11-1069G10.1
RP11-416N4.1
LINC00599
RP11-26L20.3
FENDRR
CTD-2154I11.2
linc-ASB4-2
linc-EML6-2
AC005592.2
BAIAP2-AS1
RP11-805I24.2
linc-GTF2H2-5
linc-JPH4-1
CTD-2587H24.5
RP11-6N17.10
RP11-60H5.1
RP11-50B3.2
RP11-542M13.2
RP11-298I3.4
linc-CD1D-1
RP4-749H3.1
linc-REPIN1
AATK-AS1
linc-GRAMD4-1
RP11-403P17.2
RP11-7K24.3
RP4-584D14.7
DBH-AS1
NBPF18P
RP11-430B1.2
RP11-10L7.1
CTC-264K15.6
RP5-1120P11.1
RP11-392O1.4
RP11-159D12.10
linc-ALDH1B1-3
RP11-95M5.1
PEX5L-AS1
RNF144A-AS1
linc-C20orf72
CTC-239J10.1
RP11-739P1.2
RP11-325N19.3
RP11-687M24.5
CTC-276P9.2
linc-ADRA1A
CLYBL-AS2
CTA-85E5.10
RP11-1082L8.3
RP11-89M22.3
linc-CHAC1
RP11-514D23.1
linc-B4GALNT4-1
RP11-459F6.1
RP11-1020M18.10
RP11-473L15.2
CTD-2561B21.11
CTD-2547L16.1
RP11-486A14.2
RP11-156K13.1
CTD-2517O10.6
RP11-344J7.2
linc-SPR-2
RP3-370M22.8
RP11-548O1.3
RP11-348J12.2
RP5-884C9.2
CTB-22K21.2
linc-C1orf198
linc-TLN2-1
LINC00957
RP11-28F1.2

AC005330.2
CTD-2278I10.1
RP5-1007G16.1
linc-CENPP-6
CTB-107G13.1
CTD-2083E4.6
CTD-2213F21.4
LINC00567
RP11-809C9.2
RP11-284F21.10
AC128709.3
CTD-2369P2.8
RP11-956E11.1
linc-LRRC4-1
RP11-463P17.1
linc-LZTS1-1
LINC00854
linc-EML4
linc-STK39-1
RP11-946L16.1
linc-DNAJB11-3
RP11-571O6.1
RP3-333A15.2
AC012361.1
RP11-476M19.3
RP11-145H9.3
SOX21-AS1
CTA-293F17.1
RP11-403N16.3
AC073316.2
RP11-457I16.2
RP11-205K6.2
BTBD9-AS1
RP11-1085N6.6
AC018866.1
linc-SRBD1-5
RP5-857K21.5
KIRREL3-AS3
RAB30-AS1
RP11-744K17.9
RP11-577B7.1
RP11-1C8.5
RP11-371M22.1
linc-ADAM18-2
RP13-870H17.3
RP11-356C4.3
linc-EPT1
AC090505.1
RP11-13A1.1
RP11-48B3.4
linc-PTPRS-1
RP11-227D2.3
linc-C7orf65-2
RP11-503G7.2
RP11-402C9.1
linc-FLI1-5
AC010745.1
linc-KIF2A-2
linc-IRF6
linc-SLC35F5-3
RP11-309E23.2
linc-ALDH1A1-4
AC006026.13
RP11-67H24.2
linc-RNF2-2
RP11-385N23.1
LINC00326
linc-NR2E3-2
RP11-731D1.4
RP11-264C15.2
HTR2A-AS1
linc-OTUD7A-1
RP11-167H9.4
linc-SLC44A1
linc-C14orf102-1
linc-AKIRIN1-3
linc-CENPQ-2
AC005042.4
AC003986.7
RP11-69E9.1

RP11-1018N14.4
RP11-524P6.1
linc-CCDC40-3
RP11-554A11.8
RP11-366L5.1
CTD-3098H1.2
linc-PCTP
RP11-561O23.5
RP11-234B24.4
RP11-343B18.2
linc-ANKRD55-3
RP11-21L23.3
linc-ASAP1-1
linc-PTP4A3-3
RP11-148B3.1
CTB-41I6.2
RP4-535B20.4
RP11-1070N10.7
CTB-33O18.3
RP11-879F14.3
AC015977.6
linc-ANKRD55-6
NCAM1-AS1
CTD-2265M8.2
linc-CNTNAP5-8
RP11-338N10.3
CTC-340A15.2
AC003084.2
AC093627.8
GRIK1-AS1
RP11-834C11.8
linc-SVIL-2
MIR503HG
RP11-122D10.1
AC115522.3
AC010731.3
RP11-514D23.3
linc-HUS1B-5
RP11-129B22.2
AC096579.7
linc-NGFR-4
CTC-503K11.2
LPP-AS2
LINC00635
linc-DYNLL2-2
MME-AS1
RP11-216M21.7
linc-CRH-2
RP11-352D13.5
RP11-495K9.3
linc-ADCY1-4
RP11-204P2.3
AC079630.2
RP11-338N10.2
AC092687.3
RP11-223I10.1
RP11-540K16.1
RP11-383G10.5
RP13-395E19.3
linc-ZBTB44
XXbac-B33L19.4
CTB-35F21.4
AC105402.4
RP11-87C12.5
RP11-556H2.4
linc-KCNK13
linc-ECHDC3-1
RP11-482D24.2
linc-RGMA-8
AC005235.1
RP11-1030E3.1
CTD-2089N3.2
linc-FMN1-4
linc-RNF32-1
AC116609.1
RP6-1O2.1
SORCS3-AS1
RP11-469N6.2
RP11-677M14.2
LINC00352

RP11-423G4.7
RP11-438E5.1
linc-PPP1R1A
linc-C2CD4A-6
RP11-266E14.1
CTD-2256P15.5
RP11-20J15.3
RP11-338N10.1
RP11-195B3.1
linc-ANO5-1
RP3-527G5.1
linc-C5orf43-1
RP11-319E16.2
RP11-150O12.2
linc-WSCD2
RP11-369E15.3
AC016907.3
linc-GBP5-4
linc-C18orf21
RP11-150O12.3
RP1-118J21.25
linc-WFDC12
AC002463.3
linc-DNAJB11-5
AC097713.3
C12orf80
RP11-805J14.3
linc-ATP1B1-2
linc-LAMA4-1
RP11-497G19.1
linc-MTERFD1
RP3-428L16.1
AC073987.1
RP11-704J17.5
RP11-164C1.2
RP11-676J15.1
RP1-90J4.1
linc-PRPF18-3
RP11-1018N14.5
RP1-37J18.2
CTD-2008P7.9
CTD-3032H12.1
linc-FLJ44606-4
CHL1-AS2
linc-CCDC90A-2
RP11-1134I14.8
linc-SLC19A1-4
RP11-223E19.2
RP11-298D21.1
linc-POTED-7
RP11-21B23.3
RP11-1137G4.3
AC093585.6
linc-IMPACT-1
linc-CCDC146-2
linc-SLC1A4-1
CTB-140J7.2
LINC00473
RP11-169D4.2
RP11-15M15.1
RP11-626H12.1
RP1-63G5.5
CTD-2621I17.6
RP11-359B12.2
RP11-332H18.5
CTB-27N1.1
linc-SYK-1
RP11-542A14.2
RP13-977J11.2
LINC00910
RP11-755O11.2
RP11-545A16.4
linc-PM20D2
RP11-1C1.7
RP11-168O16.2
RP11-55L4.1
RP11-328K2.1
NKX2-2-AS1
linc-CST11
linc-MLPH-2

linc-LRRC33
CTD-2055G21.1
linc-RSPH1-2
linc-ROPN1B-2
RP1-272L16.1
MYO16-AS2
linc-SMC1B-2
linc-PAXIP1-6
RP1-111C20.3
linc-IDI1
AC018755.17
RP5-903G2.2
RP11-554D20.1
RP11-92A5.2
RP11-386P4.1
linc-CPEB4-4
RP11-626I20.3
linc-TSHZ3-2
RP11-348B17.1
linc-DCTN1
RP11-554D14.6
RP11-547D24.3
RP5-1180C18.1
CTA-221G9.7
linc-MTRNR2L1-2
RP11-446F17.3
linc-CACNA1S
RP11-573D15.9
AC093642.4
linc-ZNF599-2
CTC-391G2.1
RP11-403A21.2
linc-TMEM188
RP11-402G3.3
linc-PRKAA2-4
CTD-2530H12.1
RP11-461L18.1
RP4-555D20.3
RP11-175K6.1
RP11-844P9.1
TMEM5-AS1
RP5-1024C24.1
CTD-2210P24.2
linc-NMT2
RP13-476E20.1
AC108142.1
linc-SAAL1
RP11-728F11.4
AC012485.2
RP11-956J14.1
AC024084.1
linc-EHD3-2
CTD-2020K17.1
AC108025.2
RP11-863P13.2
RP11-483I13.2
RP11-365P13.3
RP11-95L3.2
RP11-809H16.4
RP11-501J20.2
AC083843.3
RP11-380P13.2
AC079779.4
linc-PRSS12-1
RP11-573D15.2
RP1-102D24.5
RP11-143I21.1
linc-BHLHE23-1
RP11-521O16.2
AC108448.3
linc-PCSK6-1
linc-TPK1-2
RP11-290L1.3
RP5-894D12.5
linc-DYRK1A-1
TMEM191A
RP11-15A1.2
RP11-279F6.3
XX-C2158C6.1
UBE2E1-AS1

AC005618.6
RP11-713P17.3
linc-LRP8-3
linc-PCDH8-5
CTC-378H22.1
linc-RTL1-6
RP11-167N24.4
RP11-637O19.2
LINC00614
RP11-527N22.2
linc-PROP1-2
RP11-158I9.5
UPP2-IT1
RP11-702F3.3
linc-WDR60-2
AC009487.4
CTD-2246P4.1
RP11-299H22.7
AC004947.2
RP11-143K11.5
RP11-285E9.5
AP002856.7
AC079154.1
RP11-677M14.7
linc-ZNF131-5
AC090957.2
linc-ZBTB40
RP11-38L15.2
linc-G2E3-1
RP11-557J10.5
LINC00870
RP11-370I10.2
linc-HNRNPA1-1
RP11-567M16.1
linc-CPEB2-7
AC012506.1
linc-ATP2B2-3
linc-ZNF131-4
RP11-444A22.1
RP11-770E5.3
CTD-3118D7.1
GS1-39E22.1
RP11-720L2.3
RP11-627G18.2
CTD-2194L12.2
RP11-635N19.3
linc-C21orf2-2
AC096574.4
linc-CHST8
RP11-89C3.4
CTD-2308G16.1
AC007557.2
RP11-542B15.1
linc-C1orf65-2
RP11-1259L22.1
CTA-407F11.8
RP11-402F9.3
RP11-538I12.3
AC016745.3
linc-CTNNB1-3
linc-NPVF-3
AF127577.12
linc-EPB41L4A
CTD-2015A6.2
linc-TMEM14A-1
RP11-561P12.5
RP11-676F20.1
linc-KLKB1
RP11-413G15.1
linc-SPAM1
RP5-860P4.2
AC073325.1
RP11-138P22.1
linc-TMEM18-3
linc-PPP2R5C
linc-ZMIZ1-1
AC068858.1
RP11-75L1.1
AC005703.3
LINC00334

linc-PTPRG-1
AC118345.1
RP11-549L6.3
linc-SSTR4-5
LINC00642
RP11-349A8.3
RP11-685G9.4
RP11-629E24.2
CTD-2195B23.3
AC027269.2
AC006262.5
XXbac-BPG157A10.21
linc-PRDM10
RP11-552D4.1
RP11-310H4.1
CTD-2023N9.2
AC013463.2
RP11-573G6.9
AC145123.2
CTD-2001E22.2
RP11-116D17.2
RP5-955M13.3
AC073321.3
RP11-966I7.2
linc-SLC30A5-5
LINC00942
ZEB2-AS1
PRKG1-AS1
CTA-125H2.1
RP11-909N17.3
RP11-338C15.3
RP11-148M9.1
RP11-434D2.2
RP4-806M20.5
CTC-369A16.3
AC018643.4
RP11-236J17.6
NDP-AS1
CTD-2553C6.1
AF186192.1
RP11-90D11.1
RP11-22D3.1
linc-DYRK2-2
RP11-335K5.2
CTA-250D10.19
linc-MRPS18A-2
linc-FICD
linc-PTBP2-7
AF186192.6
RP11-430L16.1
linc-GRIP1-7
RP11-13K12.1
AC104782.3
LINC00514
linc-HAS2-3
LINC00856
AC124861.1
linc-ARFIP1-1
linc-ASB7-3
RP11-13A1.3
linc-BICC1
linc-RHOU
linc-PRSS22
linc-GJA5
RP11-284A20.2
MRVI1-AS1
linc-SMCHD1
RP1-230L10.1
AC005324.6
RP11-116G8.5
AC116609.2
RP4-565E6.1
RP11-763E3.1
AC093415.2
linc-KIN-7
RP11-108M9.4
RP11-573G6.10
RP1-167F1.2
linc-NOL4-3
RPS6KA2-AS1

AC008992.1
RP5-1103B4.3
RP11-254F7.2
RP3-503A6.2
AC011343.1
RP1-80N2.2
RP4-644L1.2
RP11-290H9.4
RP11-16B9.1
GS1-39E22.2
RP11-711K1.7
RP13-137A17.4
linc-ZNF322A-4
RP4-713A8.1
linc-MALT1-2
linc-DLX6-1
linc-RAN-2
linc-RFX8
linc-EPB42
RP11-661C8.2
LEPREL1-AS1
linc-MMAA
RP11-264E20.2
RP1-212P9.3
RP11-415C15.3
RP11-217B7.3
AC007040.7
linc-COLEC10-1
RP11-844P9.2
RP11-401N18.1
linc-GNAZ
RP3-377H17.2
RP1-17K7.3
linc-C13orf23-3
LINC00570
RP11-557L19.1
RP11-543C4.1
linc-NGLY1-1
GNAS-AS1
RP11-262H14.3
linc-NOX3-4
linc-IRS2-1
RP11-23E19.1
MIR4454
RP11-834C11.10
linc-YOD1-5
linc-CD5
linc-FAM72B-3
AC005863.1
linc-SSTR4-3
linc-CBWD3-5
CTD-2008P7.1
CTB-12O2.1
RP11-248G5.8
RP11-5P4.2
GCSAML-AS1
AC004837.4
linc-SRD5A2-2
LINC00331
RP11-267C16.1
linc-FAM75A1-4
linc-POTED-8
linc-SH3BGRL2-3
RP11-661P17.1
RP11-361D14.2
HOXC-AS1
linc-ATP4B-1
RP11-439C15.2
RP11-106M7.1
RP11-202A13.1
RP11-91I20.1
linc-CHD9-8
RP13-137A17.6
linc-PHF20L1
linc-NUDT6-2
linc-FAM155B
RP11-259P20.1
CTD-2021H9.1
RP11-25I15.2
RP1-56K13.5

RP11-324F11.1
CTD-2560E9.5
AP001065.15
RP11-144G6.10
CTB-174D11.2
AC093822.1
LINC00703
linc-CLLU1-2
linc-ULBP3-2
linc-ALDH1L1-2
RP11-552E20.4
linc-OPRK1-5
AC006380.3
linc-EFCAB9-2
linc-LYZL1-6
linc-VIPR2-3
RP11-217L21.1
AC010096.2
AC009229.5
linc-COL5A2
RP11-543N12.1
linc-GPR137B-1
RP11-23J9.4
linc-VLDLR-2
RP11-551M15.1
POU6F2-AS1
RP11-388P9.2
linc-C14orf102-2
RP11-641A6.2
linc-ANO1-3
AC008278.3
RP6-74O6.3
AC011513.4
RP11-630C16.2
RP11-179A10.1
RP11-999E24.3
CTD-2536I1.1
linc-SNRK
linc-CLDN24-3
LINC00529
SRGAP3-AS4
linc-LRRC38-1
CTD-2227E11.1
MAPT-AS1
AC007163.3
AC092071.1
linc-ZNF322B-3
AC058791.1
linc-SLC9A3-1
RP11-275F13.1
SNORD45
linc-C9orf170-2
AC010982.2
FAM225A
linc-ZMIZ1-2
linc-NHLH2-6
linc-ARAP3
linc-ALK-2
linc-JPH4-2
linc-GRIP1-4
linc-CD180-7
AC002539.1
linc-DIRAS2-1
RP11-761I4.3
linc-CALCOCO1-6
RP11-1406H17.1
linc-CACNA1A-3
AC140481.4
RP11-32D16.1
RP11-441F2.5
RP11-770E5.2
linc-RNF19A
LINC00277
RP11-64C12.4
AC095067.1
linc-CD247
AC084149.1
linc-C2CD4B-5
CTB-79E8.2
CLRN1-AS1

RP11-297B17.3
RP5-1177M21.1
LINC00305
RP11-547D24.1
CTD-2588J6.2
RP11-619L12.3
RP11-227F19.2
linc-GBP5-6
RP11-27G24.3
LINC00297
RP11-569G13.2
AC104623.2
LZTS1-AS1
RP11-1299A16.3
RP11-680B3.2
CTD-2319I12.4
linc-MYEOV-2
linc-CDKN2C-5
RP11-88H9.2
RP11-700H6.2
LINC00951
linc-SORBS2-3
linc-LAMA1-7
linc-ERICH1-1
CTD-2377D24.6
linc-C6orf120-6
CTD-2339F6.1
RP11-21L23.4
linc-LRRC8D-2
RP11-763B22.6
CTD-2532D12.4
AC131056.3
linc-SLC6A6
DLGAP1-AS1
RP11-493K23.1
linc-GCNT7-1
RP11-209E8.1
AC108051.1
RP11-883G14.2
RP11-463J10.4
RP11-334E6.12
ZNF582-AS1
AC073869.19
RP11-526K17.2
AC009133.14
RP11-473C19.1
linc-GCNT2-9
linc-THSD7B-2
linc-HIST3H2A-2
HOXD-AS2
linc-TRIM38
linc-AMOTL1-1
linc-ANKRD56-1
CTD-2320G14.2
RP11-108B14.5
AP000688.8
RP11-332E19.2
linc-OPLAH
LA16c-380H5.5
RP11-161M6.2
RP11-25E2.1
linc-UBE2F
RP11-325D15.2
AC007743.1
RP11-778J16.3
RP11-1008C21.1
LINC00322
RP11-96A1.5
RP11-843B15.2
CTD-3247F14.2
RP11-120C12.3
linc-CDYL-1
linc-TUBA1A-3
RP11-321E2.3
RP11-532F6.4
RP11-22D3.2
linc-QSOX2
linc-IGFL4
LINC00051
RP11-153M7.5

RP11-566K19.3
RP11-927P21.11
RP11-530C5.1
CTD-2514K5.2
LA16c-321D4.2
RP11-576I22.2
RP11-807H7.1
linc-THSD4
RP11-469N6.3
linc-NTM-6
linc-GBP5-3
linc-KCNMB1-4
AC006960.7
RP11-863P13.4
PPEF1-AS1
RP11-558A11.3
RP11-435B5.6
A2M-AS1
LINC00434
CTC-782O7.1
linc-ZNF608-5
KCND3-IT1
AP003774.6
AP001046.5
linc-ITIH2-10
linc-AMD1-2
RP11-452F19.3
RP11-440G9.1
linc-SLCO4A1-2
KRBOX1-AS1
WI2-1959D15.1
RP11-85G21.2
RP11-753D20.1
RP11-128A17.1
MLIP-AS1
RP1-34H18.1
linc-GALC-3
CTD-2545H1.1
linc-MUC20-8
linc-ADAMTS14
RP11-2E11.5
RP11-327I22.8
RP11-332E3.2
linc-PRDM11-1
linc-PPARGC1B
RP11-466A19.3
AC109642.1
RP11-15E18.1
RP11-157E21.1
linc-RXRA-1
linc-TP53TG3B-8
RP11-308N19.3
RP11-63A1.2
linc-PRSS3-3
linc-SLC46A2
RP11-121E16.1
RP11-110L15.1
linc-PRICKLE2-2
RP11-134G8.7
AC118754.4
linc-CIDEA-3
CTD-2588J1.1
ERICH1-AS1
RP11-104J23.2
RP3-467K16.7
linc-TSPO2-1
linc-CD9-2
RP4-669L17.2
linc-RALGAPA1-3
LINC-ROR
AC073257.1
MIR212
RP11-214F16.4
RP11-475O23.2
AC008940.1
linc-ANKRD20A4-4
linc-MAP3K8-1
CDRT8
RP11-190J1.3
AC008691.1

linc-CDC7
linc-FAM113B-2
RP11-3K16.2
RP11-785G17.1
RP11-310H4.2
RP11-730G20.1
CTB-178M22.1
RP11-214K3.19
linc-PDE10A-2
AL132709.8
RP5-1178H5.2
linc-ACP6
RP1-187B23.1
RP11-679B19.2
RP11-624J12.1
AC141928.1
RP11-58A12.3
RP11-355I22.6
linc-GLI3-3
linc-OR14J1-2
linc-GOLGA8B-2
LINC00453
RP11-277L2.4
RP11-470L19.2
linc-NIPAL2-2
AP001044.2
RP11-73M7.1
LINC00494
linc-TCF7-3
RP11-344E13.3
RP11-643A5.2
RP11-364C11.2
LINC00977
SSPO
CTC-431G16.2
AC017074.1
linc-TTC15-5
RP11-13B9.5
RP3-380B8.4
AC007277.3
RP11-63E5.8
RP11-463H12.2
RP11-136H19.1
linc-IRS2-2
linc-TPK1-3
linc-FAM75A7-8
linc-GALNT10
RP11-90J7.2
RP11-429A20.3
linc-RHOBTB2-1
linc-C11orf58-4
linc-IL10
LINC00302
RP11-101P17.9
linc-ARFGEF2-14
RP11-189B4.6
linc-MBNL2
linc-CTU1-1
CTC-349C3.2
linc-C20orf70
RP11-474D1.4
linc-RAB2A-1
RP11-814P5.1
RP11-10J21.6
FAM138F
RP11-98J9.3
RP3-514A23.2
linc-U2AF1-1
linc-ADAM11
RP11-73M14.1
linc-SLMO2-3
linc-NBPF3-1
RP11-319E12.2
linc-ZNF673-2
RP11-167N24.3
RP11-261C10.4
RP1-29G21.1
linc-ARL6IP5
linc-PTBP2-1
RP11-525K10.2

CTD-2223O18.1
CTD-2526A2.2
linc-GGTLC1-3
CTD-3064M3.3
AC005592.1
linc-FAM120B-5
linc-CWC15
RP4-594L9.2
linc-IGFL1-1
RP1-122P22.2
CTA-398F10.2
RP4-651E10.4
LINC00681
CTC-304I17.4
RP11-254F19.3
RP11-518I13.1
AC013402.2
linc-CR2-1
RP5-1172A22.1
RP11-646J21.2
linc-PAK1-1
RP11-463P17.3
linc-LCTL-2
LINC00846
linc-GLT25D2-1
linc-MPPED2-1
RP11-21B23.2
RP11-483H20.6
RP11-171A24.3
RP13-614K11.2
linc-TTC23
RP11-97N19.2
RP11-74K11.2
RP11-280K24.1
linc-NBPF15-1
RP11-110L15.2
RP3-495K2.1
RP11-883G14.1
RP11-168G16.2
linc-RPRM-7
AC226118.1
RP11-39M21.1
RP11-324D17.2
RP11-278I4.2
linc-TGS1
RP4-562J12.2
AC108051.3
linc-CBWD3-4
RP11-44F21.2
RP11-218C14.5
linc-KIAA0182-2
linc-OR4M2-2
AC073133.1
RP11-160N1.9
RP11-538D16.2
AF064858.7
RP11-379K22.2
RP11-1038A11.1
AC019181.2
linc-DUSP22-4
linc-ASCC3-2
RP11-736E3.1
LINC00454
linc-ST8SIA3-1
RP11-164H13.1
linc-PARP11-3
RP11-488P3.1
RP11-269C23.3
linc-FRG1-2
XXyac-YM21GA2.3
AC009508.1
linc-RGSL1-3
RP11-74C13.3
linc-OSBPL8
AC091633.3
linc-INADL
RP11-791O21.5
linc-AKT3-2
RP11-572M11.3
RP11-1070N10.6

linc-ZNF599-1
RP11-215E13.2
RP11-1113L8.1
RP11-702F3.4
RP11-281N10.1
CTC-304I17.3
linc-ZNF236-6
AC007128.1
linc-ENPP4-1
KB-1471A8.1
linc-LOC100132288-2
AC108868.5
RP11-535M15.1
RP11-680F8.3
linc-IL1R2-1
RP11-469A15.2
SYNPR-AS1
RP11-3L21.2
LINC00691
RP11-439H13.2
RP11-466A19.6
linc-TMC3-4
RP11-753H16.4
RP11-1085N6.4
ENOX1-AS2
linc-WNT4
linc-EIF5AL1-1
RP11-298O21.5
AC006378.3
linc-ENPEP-1
linc-PRKAR2B
RP11-638F5.1
RP11-1258F18.1
RP11-510M2.6
RP11-389G6.3
linc-PRDM4-1
MAST4-IT1
RP11-159N11.3
linc-OSBPL2
RP5-965F6.2
RP1-90G24.10
linc-THBS2-2
HECW1-IT1
RP11-473E2.2
RP11-646E18.2
C4A-AS1
linc-AMZ2-2
RP3-461P17.10
linc-RAB11FIP5
RP11-381N20.1
linc-CNKSR2
RP11-472N13.2
RP11-145M4.3
RP1-212P9.2
CTD-2308B18.2
RP1-276E15.1
linc-TMEM132E-2
linc-CHRNA7
linc-PECI-4
AC093901.1
RP5-858B6.1
RP11-5N11.5
AC079354.5
RP11-761N21.1
linc-COL5A1-1
AC002472.11
RP11-354K1.2
linc-PROC-4
RP11-524G24.2
RP11-435M3.2
RP11-692C24.1
RP11-102F4.2
RP1-155D22.1
AC008069.1
CTB-17P3.4
RP4-568F9.6
AL121578.2
RP11-344B23.2
RP11-143M1.3
AC093850.2

XXyac-YX60D10.1
TBX5-AS1
RP11-527L4.5
LINC00861
RP11-794P6.1
AC116614.1
CTD-2116F7.1
RP11-359D14.3
RP11-266O8.1
RP11-96K19.4
linc-AGBL4-1
CTA-929C8.6
RP1-63G5.7
RP11-71E19.2
RP11-326C3.11
RP11-1041F24.1
AC013271.3
RP11-416N2.3
CTC-321K16.1
TPTE2P6
linc-AP3S1-2
AC023137.2
RP11-648L3.2
CTD-2275D24.4
RP3-434O14.8
RP11-883G14.3
CTD-2587M23.1
RMDN2-AS1
AC139099.6
RP11-255A11.21
linc-COL18A1-1
AC109826.1
RP11-169N13.4
linc-SLCO2A1-2
linc-C1orf185
RP11-863P13.3
RP11-626H12.3
RP11-1094M14.12
linc-ELOVL5-1
RP11-18F14.1
linc-ODF2L
RP11-217B7.2
CTD-2008P7.8
AC009965.2
MIR146A
linc-ODF1-1
RP11-372H2.1
CTD-2547L24.4
RP11-752G15.3
RP11-63N8.3
RP11-326K13.4
CTD-3074O7.2
linc-C6orf145-2
linc-TP53I11-1
linc-CCND2
AP002856.5
linc-POLR3A-4
RP11-234K24.3
RP11-264J4.5
XXbac-BPGBPG55C20.1
linc-PIGF-2
RP11-107I14.5
RP3-368B9.2
linc-GAS1-2
RP11-65L19.4
RP11-651P23.5
AC003986.5
RP5-1096D14.3
ATP2B2-IT2
RP4-683M8.2
linc-THBS1-2
AC139099.4
linc-CADM1-1
linc-PRSS42
RP3-359N14.2
RP11-779P15.2
linc-SNX9
CEBPA-AS1
linc-APOC3-3
RP4-676L2.1

RP11-483P21.3
RP11-60L3.1
RP11-85M11.2
LINC00588
RP11-137J7.2
linc-HMGB1-2
linc-EXOC4-4
RP11-102C16.3
RP11-646J21.6
RP11-826N14.1
linc-FABP5-2
BDNF-AS
RP11-116D17.3
RP4-749H3.2
linc-FAM78A-2
RP11-328D5.1
linc-LOC100129520
RP11-530N7.3
GS1-166A23.1
RP1-90G24.8
KCNJ6-IT1
RP11-382D12.2
RP5-1100I6.1
RP11-543C4.3
DLGAP1-AS3
linc-LYRM7-2
CTB-131B5.4
linc-MFHAS1-2
RP13-379L11.2
RP11-162J8.2
MIR205HG
RP4-614C15.2
RP11-142J21.2
linc-GDF10-2
RP11-811P12.3
RP11-895M11.3
AC009518.5
RP11-737F9.1
RP11-348F1.3
linc-FAM92B-4
AC113607.3
RP11-21L23.2
AC005029.1
linc-PLGLB2
RP11-978I15.10
linc-TFAP2A-2
linc-PPAPDC1A
linc-DNAH14
CTB-104H12.6
linc-MTOR-2
AC007381.3
TMPRSS4-AS1
RP11-645C24.5
RP11-1049A21.2
PVT1
linc-NKX2-5-2
AC010096.1
RP11-85G21.3
NEBL-AS1
RP11-445N18.7
GPR158-AS1
RP11-64B16.5
CTD-2154H6.1
NAV2-AS4
RP11-168E14.1
RP11-570K4.1
RP11-397C18.2
RP11-324J3.1
RP4-806M20.3
AC114776.3
RP11-734K21.5
linc-COX19
linc-PSD2-1
LA16c-380H5.4
linc-IQCG-5
RP11-178F10.3
linc-ENKUR-4
linc-ALG2-2
linc-NR2F2-6
RP11-15A1.3

RP11-410N8.1
RP1-79C4.4
linc-EOMES-2
CTD-2193G5.1
linc-DACT2-4
linc-PLCH2
ADAMTS9-AS1
RP5-982E9.1
RP11-125D12.2
linc-USP28-2
linc-KLF6-3
RP11-875H7.2
RP1-140J1.1
linc-LYPD6
LRCOL1
linc-THBD
linc-C17orf97-2
CTD-2218K11.2
RP11-10A14.6
RP5-1002M8.4
linc-KLRD1
linc-CACNA1A-1
linc-ENPP4-2
JAZF1-AS1
RP11-58A17.4
linc-CBX7
CTC-254B4.1
RP11-415C15.1
linc-SRBD1-1
linc-PZP-3
RP11-543E8.1
AC006445.8
CTC-321K16.4
RP11-168P8.3
linc-ADAR
linc-APPL2-2
RP11-69I8.3
HCG9
RP11-160H22.5
RP11-536C5.2
LINC00652
linc-IVNS1ABP
linc-FNDC1-2
linc-RANBP3L-1
RP11-536I6.2
RP11-466P24.6
linc-BMP4-2
RP11-449J1.1
RP11-462G22.1
AC012363.13
RP11-831A10.2
RP11-108K3.2
AC026150.5
RP11-543D5.2
RP11-160H12.2
RP11-57H12.5
AP001610.9
linc-APOB-1
HOTAIRM1
RP1-93I3.1
RP4-591L5.2
linc-TCP10-4
RP11-1260E13.4
linc-FAM118B
RP11-568A7.3
RP4-610C12.1
linc-PZP-4
RP11-1C8.7
RP3-453D15.1
RP11-669I1.1
RP11-61J19.4
linc-FRG1-5
AC008088.4
ATP2B2-IT1
RP11-260G13.1
AP000439.3
RP11-554I8.2
linc-PEX3
RP11-767C1.1
RP11-433A10.2

FAM66B
MORC1-AS1
RP11-293A10.3
linc-GGTLC1-2
RP11-284P20.3
linc-MTRNR2L1-4
AC009134.1
RP11-260M19.2
SNORA11
PARD3-AS1
RP6-74O6.2
RP11-551L14.6
linc-DLX2-3
linc-SPACA1
HTR4-IT1
CALML3-AS1
RP11-19O2.2
linc-TNFAIP3-2
C1QTNF9-AS1
RP11-412H9.2
RP11-165J3.5
RP11-272J7.4
linc-ZNF354A
linc-SSPN-1
AC092619.1
linc-ADAMTS8-1
linc-COL5A1-5
AC003985.1
RP11-378A12.1
CTC-505O3.2
RP11-701B16.2
linc-SALL3-2
linc-SIK1-1
RP11-643M14.1
RP11-406H4.1
RP4-663N10.1
linc-TAF1L-2
AC004448.5
RP11-316M20.1
linc-OR5AC2-1
linc-TWIST2-2
RP11-697B24.1
AC006262.4
RP1-302D9.3
LINC00853
RP11-53A1.2
RP11-542G1.3
linc-RAP1B-4
linc-CDR2-1
RP11-515E23.1
linc-FAR2-1
linc-PTAR1-5
RP11-738B7.1
RP11-90L20.2
linc-CTNNB1-1
linc-SBDS-8
RP4-683L5.1
linc-CD300C
CTD-2228K2.7
linc-RPL38-1
CTD-2196P11.2
RP11-354P11.4
RP13-379O24.3
linc-POU4F2-1
linc-IL1R1
RP11-1006G14.1
RP13-30A9.2
F10-AS1
RP11-1018N14.1
RP11-414H17.5
linc-SNX20-1
RP11-536K17.1
RP11-318G21.4
linc-ZBED1-2
linc-LYZL1-3
linc-C18orf62-4
CTD-2270F17.1
linc-MARK1-2
RP11-545A16.1
RP11-402L1.11

AC008697.1
RP1-17K7.2
linc-SORCS1-2
linc-ADRB1-2
LINC00658
RP11-713P17.4
linc-FAM120B-4
CTD-2083E4.7
RP3-443C4.2
RP11-43D2.2
RP11-53B2.4
RP11-183E24.2
CTC-523E23.5
linc-DAAM1-2
RP11-797E24.3
RP11-372M18.2
AC145343.2
linc-PDIA6
AP001092.4
linc-SEP15-6
RP11-430H10.3
FLI1-AS1
CTD-2562J17.9
RP11-749H17.1
CCDC148-AS1
RP11-1151B14.3
RP11-94B19.6
AC073636.1
C20orf166-AS1
CTD-2009A10.1
linc-LINS-2
RP11-597A11.2
linc-PAFAH1B1
linc-UBASH3B-3
linc-TMEM169-1
RP11-539E17.5
RP13-43E11.1
linc-RALGPS2-2
linc-RPP25-2
linc-GALNT9-1
RP11-430C7.5
linc-RGPD1
linc-RAB23-3
CTD-2176I21.2
linc-CITED4
RP11-583F24.8
linc-ZNF673-3
LINC00319
linc-PPP1R3D-1
RP11-386G11.8
linc-STK32B-2
RP11-358D14.2
CTC-229L21.1
CTC-347C20.1
RP11-158I9.7
linc-PFAS-1
RP11-317B7.3
RP11-90K6.1
RP11-54O7.2
GS1-204I12.1
linc-EXOC6B-1
RP5-859D4.3
RP4-745E8.2
RP11-648F7.1
linc-FAM55D
AC002480.3
AC104134.2
RP11-510C10.4
RP11-63K6.7
RP11-351M16.3
RP11-75C23.1
RP11-415A20.1
AP000343.2
RP11-436H11.2
RP11-139B1.1
linc-FAIM3
linc-SHISA6-2
ZIC4-AS1
linc-UNC5B-1
CTD-3064M3.1

RP11-373E16.3
RP1-37N7.3
RP11-31I22.3
RP11-426C22.6
CTD-2015A6.1
linc-HIST1H2AG-4
linc-USP24-2
AP001043.1
AC017101.10
linc-HABP4-2
RP11-63E5.6
RP11-165F24.5
RP11-899L11.3
LINC00693
linc-GPR31-2
LINC00643
RP11-298O21.6
linc-C17orf108-4
RP11-136K7.1
AC007682.1
KB-1448A5.1
RP1-302D9.4
RP11-347D21.3
RP11-558A11.1
RP11-84A19.2
RP13-977J11.8
linc-GDF10-1
PRKCQ-AS1
RP11-166D18.1
RP11-587D21.4
CTA-992D9.7
AC012506.3
AC079776.3
RP11-567E21.3
RP1-207H1.3
RP11-318M2.3
RP11-308B5.2
RP11-496B10.3
AC004593.3
AC010907.5
FAM99A
RP11-71G7.1
RP11-260A9.6
RP11-498E2.9
CTD-2015G9.1
linc-NFIL3
RP11-626H12.2
linc-ALX4-2
RP11-254F7.1
AC079586.1
FAM170B-AS1
linc-EFR3A-1
RP5-1125M8.2
RP11-856M7.6
linc-ACSM5-6
RP11-353N14.5
LINC00032
linc-ARMC3-1
linc-FNBP1-2
RP11-44N12.5
linc-MDGA2-2
RP11-24H2.2
linc-SCUBE1
LA16c-83F12.6
RP11-278H7.3
RP11-254F7.3
linc-TG
RP5-857K21.15
CTD-2530N21.4
RP11-333O1.1
linc-SLC25A32-3
RP11-160E2.11
linc-PARD6G-2
RP4-601P9.2
linc-SLC28A3
linc-TBC1D21
RP11-290K4.1
AC107057.2
AC005042.5
CTC-551A13.2

RP11-129J12.1
linc-PAX1-1
AC019330.1
AY269186.2
RP11-774O3.2
NALCN-AS1
linc-COX5B-3
linc-CRYBB1-7
RP11-470E16.1
linc-GLT25D2-2
RP11-282I1.1
linc-RPP30-1
RP1-290I10.6
linc-GREB1-2
ZNF883
linc-DYX1C1
RP11-272D12.1
linc-GREB1-4
RP11-481E4.2
RP5-897D18.1
AP001271.3
linc-RCC2-2
RP11-96L7.2
linc-RABL3-1
RP11-569A11.2
linc-FBXL7-2
RP11-89N17.2
DGKK
CTD-2523D13.2
RP11-39H13.1
SSTR5-AS1
U1
CEACAM20
CTD-2383M3.1
RP11-24P4.1
HPYR1
GS1-279B7.1
RP11-404P21.5
linc-PVALB-1
linc-ERGIC2
UCHL1-AS1
RP11-132N15.3
linc-MRGPRF-4
RP11-756K15.2
RP11-734K21.2
linc-CLVS1
CTD-2210P24.3
linc-C14orf101-2
RP4-597A16.2
RP11-744J10.3
RP11-24J19.1
RP11-394B5.2
RP11-256I23.2
linc-C10orf57-2
RP11-505C13.1
RP11-479J7.2
RP11-377G16.2
AC017002.4
LINC00086
linc-SERP2-4
RP11-129I19.2
linc-PHF21A-1
RP11-1042B17.5
AC022201.4
HAS2-AS1
linc-AUTS2-1
RP11-474O21.5
RP11-21C4.1
RP11-73G16.3
RP11-436H11.4
RP11-475D8.1
SRGAP3-AS3
linc-OR10Q1
LINC00704
RP4-813D12.3
ADORA2A-AS1
linc-FTSJD1
linc-BNC2-2
RP11-342D11.3
RP11-265D19.6

RP4-665J23.2
CTD-2537O9.1
RP11-431J24.2
RP11-260E18.1
linc-CALB2-2
linc-PCDH8-9
RP3-406A7.7
RP11-685A21.1
AL845154.2
linc-ZNF366-6
CTD-2247C11.5
RP11-778O17.4
RP11-278H7.1
AP001046.6
AC006129.1
linc-SHOX-2
RP11-53B5.1
linc-CARD11-3
linc-CTTNBP2NL
RP11-138E9.2
RP11-34F13.3
RP11-290L1.2
linc-S100P
RP13-16H11.2
CTD-2023N9.3
KRTAP5-AS1
RP11-515O17.3
RP11-5N11.7
RP11-272B17.1
CTD-2330J20.2
RP1-45N11.1
RP13-467H17.1
PDX1-AS1
CTB-35F21.2
RP11-168L22.2
CTD-2034I21.2
AC007182.6
AC093171.1
linc-C15orf59-1
RP11-856M7.1
TTLL11-IT1
LINC00654
CTA-134P22.2
linc-MARK1-3
linc-ADCY1-2
RP11-17E2.2
linc-MGMT-4
linc-ACCN1-3
RP11-16E12.2
RP11-288G11.3
linc-NR2F2-2
linc-CLDN10-1
RP11-495P10.6
RP11-324P9.1
RP11-10A14.7
TRIL
RP11-396J6.1
AC011625.1
linc-APOH
RP11-730K11.1
RP11-778D12.2
linc-GTPBP4-1
linc-SCAMP1-1
MIR3179-1
linc-MIER3
linc-MYH10
RP11-770E5.1
LINC00989
RP11-509J21.4
linc-TFAP2C-3
RP11-597A11.6
RP11-465M18.1
RP11-175B12.2
linc-CRH-4
linc-ARHGAP28-2
RP11-94B19.1
RP11-47L3.1
RP11-45A12.2
RP1-111D6.2
RP13-726E6.2

linc-RABL2A-2
linc-TNKS2-2
AC139099.5
RP11-58O15.1
linc-CD33
RP11-21B23.1
RP11-49I11.1
RP11-1028N23.2
RP11-491F9.8
RP11-20G6.2
RP11-78J21.4
RP11-614O9.1
linc-CLMN-2
linc-IDH3B-1
RP11-428C6.2
RP11-342M3.1
CTD-2313F11.3
linc-SGPP1
RP11-354A14.1
linc-SDK2-1
RP11-62I21.1
CTD-2034I4.2
LINC00845
linc-ALS2-2
CTD-2309H9.3
RP11-546O6.4
AP001505.9
linc-CDH11-4
RP11-269F21.2
RP11-114H24.7
AC011518.2
KB-1184D12.1
RP11-220I1.2
linc-SFTPB
AL022476.2
RP11-21M24.2
RP11-706O15.7
RP11-499F3.1
DSCR10
AC122136.2
linc-OAF-3
DNAJB8-AS1
LINC00556
linc-DMRTB1-1
linc-TTLL7-6
DENND5B-AS1
AC007278.3
linc-TAS2R38
AC007386.2
RP11-635L1.3
linc-BRD9-2
AC009501.4
linc-C20orf85
linc-MFAP3L
linc-IRAK4-2
RP11-451H23.3
CTD-2540B15.9
linc-STAB2-1
linc-LCLAT1-1
CTA-221G9.11
linc-RIMBP2
RP11-189E14.5
AC096579.13
RP11-265P11.1
linc-SORCS3-2
RP11-711G10.1
linc-AQPEP
RP11-1223D19.1
linc-ARHGEF11
RP4-724E13.2
SYN2
PABPC5-AS1
RP11-397O4.1
KB-173C10.1
linc-ZCCHC17-7
linc-ZRANB1
RP5-1139I1.1
RP4-646N3.1
linc-GGTLC1-7
linc-PTK2-1

linc-COL15A1
linc-XPC-2
RP11-805I24.1
linc-C20orf196-1
linc-ZBTB16
linc-CDKN2C-4
CTD-2130F23.2
RP11-2E17.1
RP1-85F18.5
linc-C5orf38-5
DISC1-IT1
CTD-2541J13.1
ARPP21-AS1
linc-RHOB-5
linc-NCOA2
linc-SULF2-6
CTD-3037G24.3
linc-ISOC2-1
RP11-465K1.2
RP13-895J2.2
CECR7
linc-PGA3
AC012354.6
AC003005.2
RP1-122K4.3
RP11-9G1.3
RP11-435O5.7
RP11-430N14.4
linc-ATP4B-2
RP11-267A15.3
AC026202.5
WI2-87327B8.1
RP11-157F20.3
WT1-AS
RP11-403B2.6
RP11-149B9.2
linc-CPEB2-15
RP11-396N11.1
AC022182.3
RP11-51M24.1
RP6-191P20.4
RP1-23K20.2
linc-DCT-1
linc-FLI1-1
CTA-109P11.4
AC123886.2
SMIM2-AS1
LINC00566
RP5-1031D4.2
RP11-18H21.1
RP4-782G3.1
RP11-114J13.1
RP1-93H18.6
RP11-932A10.1
linc-ARHGEF10
RP11-337A23.6
RP11-764K9.1
LINC00970
AC007278.2
RP11-78B10.2
CTD-2314G24.2
FRMPD3-AS1
RP11-642M2.1
AC007009.1
RP11-10A14.5
RP4-710M16.2
RP11-231L11.1
linc-C2CD4B-1
RP11-398B16.2
AP000688.29
linc-FAM75A7-3
RP11-363E6.3
linc-ARMC3-2
RP11-44F14.2
linc-ASCL1-2
AP001891.1
RP11-77A13.1
RP11-344E13.4
RP11-628E19.3
RP4-669B10.3

U73167.7
HOXA-AS4
linc-ERG-11
AC013733.4
linc-PARP11-1
LINC00617
GPHB5
RP11-205K6.1
LINC00222
linc-SCN11A-2
linc-CPEB4-7
RP5-998N21.7
RP11-923I11.1
RP11-902B17.1
RP11-200A1.1
RP11-478H13.1
RP11-431M7.3
HCG24
LBX1-AS1
RP11-317M11.1
RP11-905K4.1
RP11-717I24.1
OTX2-AS1
GS1-519E5.1
RP11-341G5.1
AC018685.1
linc-KCNA5
Z99756.1
linc-INO80
RP11-323H21.3
linc-ZIC2
linc-PPID
linc-EML6-1
linc-ARFGEF2-10
KCNC4-AS1
AC073321.4
linc-CHL1-2
RP11-262A12.1
IQCF5-AS1
RP11-309G3.3
linc-MAP1LC3B-6
RP5-828H9.1
linc-ENKUR-2
RP11-159J2.2
linc-CREM-4
CTD-2329C7.2
RP5-896L10.1
AC011286.1
CTD-2216M2.1
linc-PML
RP13-635I23.3
linc-WTAP-2
linc-FOXM1-3
RP11-691H4.3
RP11-622A1.2
linc-NTRK2-3
GS1-542M4.4
linc-ANXA8L2-2
linc-C20orf196-2
RP11-463O9.6
linc-PDSS1-1
RP11-165E7.1
RP11-219B17.2
RP11-213G21.1
RP11-474D1.3
linc-TRPM5
EGFLAM-AS2
CTD-2540B15.7
linc-PSMA8-4
RP11-1124B17.1
RP11-2L4.1
RP11-243E13.2
RP11-522M21.2
RP11-20G13.1
linc-SEPSECS-2
linc-NBAS-9
RP11-165I9.6
RP4-625H18.2
linc-C1orf174-1
linc-C3orf79-4

RP11-542A14.1
RP13-436F16.1
RP11-94H18.1
RP11-113I22.1
linc-HSCB-11
linc-NTRK2-6
linc-GATA3-2
RP1-137H15.2
RP11-12M5.1
CTD-2130O13.1
linc-FOXO4
linc-EIF5
RP11-439A17.4
linc-HTR2A-4
AC132807.1
CTC-459M5.2
linc-RUFY4-3
AC017048.1
RP11-556H2.2
linc-KLF12-2
linc-NID2-2
AC005487.2
RP5-1186N24.3
BX322559.3
AJ003147.9
RP11-430H10.2
RP11-96K19.2
RP11-91I8.1
RP11-231G15.1
AF131215.8
linc-JAKMIP1
AC078882.1
CTC-297N7.9
LINC00535
linc-HAS2-2
RP11-141E13.1
AC013402.5
linc-PITX2-1
RP11-567J20.1
RP11-317N12.1
RP5-1077H22.1
C4B-AS1
linc-PTCHD2-2
RP4-799D16.1
RP11-561O23.8
RP11-578F21.6
linc-C1orf201-1
linc-GATA4-1
RP11-292E2.1
linc-RASGRP1-1
RP11-1049H7.2
ARHGEF7-AS2
RP11-89N17.4
linc-LRRC10B
RP11-489N22.3
IL21R-AS1
linc-DIAPH3-1
RP11-401O9.3
linc-MAP3K8-2
RP11-180I22.2
RP11-677I18.4
RP11-46I8.3
RP1-27K12.2
RP11-435O5.4
RP11-831A10.1
AC023490.1
linc-KIAA0564-2
linc-POGK-1
U66059.58
AC004152.6
RP1-17K7.1
linc-IPCEF1
linc-UFM1-2
RP11-14D22.1
CTA-363E6.2
linc-VSX1-3
DNM1P35
FAM66E
LINC00626
linc-RBM7

RP11-179A7.2
CTD-2195M15.3
RP11-14N7.2
ACVR2B-AS1
PRSS29P
RP11-195M16.1
linc-ARHGAP28-6
linc-WNT3
AC012506.2
RP11-531H8.1
RP11-555J4.4
AC007403.2
RP11-507B12.2
AP006285.7
RP11-328K22.1
linc-RBBP9-2
RP11-420L9.5
CTA-929C8.5
linc-TNFAIP3-3
RP11-1042B17.3
CTC-359M8.3
RP4-614N24.1
RP11-5N11.2
RP11-408H20.1
AC104076.3
RP11-25I9.2
NOVA1-AS1
RP11-930O11.2
AP000260.4
RP11-488L4.1
RP11-103H7.3
RP11-214D15.2
linc-ZNF280D
RP11-327J17.2
RP11-473A10.2
linc-ARHGAP20-1
LINC00202-1
CTD-2171N6.1
RP11-728E14.2
RP11-17A4.3
linc-STOX2
linc-SLC45A4
RP11-598F7.4
LINC00388
RP11-162K11.4
RP11-751H17.1
AC004448.2
RP11-310J24.3
RP11-364C11.3
CTD-2562J15.6
AC068538.4
RP11-907D1.1
linc-TACR2
DLG5-AS1
LINC00284
linc-CCDC93-1
RP11-286N22.10
RP11-18H21.2
linc-USP47-1
RP11-359N11.1
AC093326.3
AC011747.3
AP000439.5
linc-CARTPT
RP11-439A17.10
linc-CD9-1
AC006552.1
LINC00517
ITPR1-AS1
RP11-236J17.5
linc-SEMG1
linc-HES1-3
linc-GNG8
RP11-973F15.2
linc-RGS8-1
RP11-150C16.1
RP11-372E1.4
RP11-231K24.2
GRIP2
CTD-2313F11.1

AC009518.4
AC009365.4
RP11-245G13.2
RP11-503C24.4
RP11-480A16.1
linc-ADRB1-1
RP5-1028K7.2
XXbac-BPG154L12.4
linc-PROM2-1
linc-VWA5A
RP11-701I24.3
AC005789.11
CTA-714B7.5
RP11-930P14.2
RP11-87N24.2
RP11-326C3.15
RP11-366L20.4
RP11-72M17.1
linc-DEFB104B
CTD-2193P3.2
RP1-34M23.5
CTD-2215E18.3
linc-RCC2-1
linc-ARHGAP26-2
RP11-164D18.2
RP1-251I12.1
CTD-2072I24.1
AC069155.1
RP11-174J11.1
RP11-434E6.2
linc-C2CD4A-7
RP11-268G13.1
AC135178.7
linc-GOLGA8B-1
CTD-2050B12.1
RP11-16N11.2
RP11-398G24.2
RP11-161I6.2
SLC39A12-AS1
CTC-304I17.2
RP11-58A18.1
linc-SCEL-1
RP1-244F24.1
AC007557.4
RP11-446H18.5
MIR1302-2
MRGPRG-AS1
linc-AKAP3-1
linc-SOX1-1
linc-GJA8-1
linc-CPXM2-1
RP11-2C15.1
CTD-2540B15.6
linc-APPL2-3
linc-MUDENG
linc-RYK
SRGAP3-AS2
RP11-500K19.1
RP5-1119O21.2
linc-TSHZ2-4
RP11-181B18.1
LINC00637
RP11-343J18.2
RP11-310H4.3
linc-PLEKHH2-5
linc-VPS33B-3
linc-PAH-1
RP11-313F23.4
RP11-703H8.9
AC011648.1
RP11-136K7.2
RP11-54O7.1
RP13-30A9.1
AC016768.1
linc-RAB7A-2
RP11-210M15.1
linc-C9orf102-3
RP11-86H7.7
RP3-448I9.1
CTD-2526M8.3

RP11-61A14.1
linc-CHL1-1
RP11-172E9.2
linc-AGBL4-3
ROPN1L-AS1
RP11-889D3.1
RP11-12A1.1
linc-CLVS2
RP11-373I8.1
FRY-AS1
linc-PET112L-5
RP11-79C6.3
RP11-351I24.1
CTC-461F20.1
CTA-360L10.1
RP11-486M23.2
CTD-2044J15.1
RP11-1055B8.2
MIAT
UNC5B-AS1
linc-MICAL3-2
RP11-74H8.1
RP4-781K5.8
RP11-284H19.1
linc-MTHFSD-5
linc-NUP35
RP11-65J21.3
linc-KCNMB1-2
AC017076.5
AC008268.1
linc-KIAA0649-4
LHFPL3-AS1
linc-HUS1B-2
RP11-1101H11.1
RP11-14C22.3
RP3-340N1.6
linc-TRMT112-3
AP001189.4
AP002954.4
RP11-159K7.2
RP11-316P17.2
RP3-512E2.2
CTC-523E23.4
CTD-2540M10.1
linc-EHD3-1
linc-RGMA-6
linc-POU3F1-1
RP11-23F23.2
RP1-166H4.2
AC018737.3
RP11-495O11.1
MIR519A2
linc-PTBP2-6
RP11-355F16.1
ANO1-AS2
RP11-168O10.6
RP11-57C13.4
linc-MAP1LC3B2-11
RP11-107N7.1
AC113618.1
RP11-199B17.1
CTD-3187F8.14
GS1-421I3.2
AC006050.2
linc-ERCC4-1
linc-SULF2-9
CTB-187M2.3
RP11-567J20.2
linc-ZNF366-2
linc-E2F7-3
RP11-679B17.2
linc-ANKFN1
RP11-239E10.3
linc-FAM92B-2
RP11-44F21.3
RP11-1057B8.2
linc-MUC20-3
RP11-357K6.1
AC140481.8
CTD-2319I12.5

linc-CDH11-5
RP11-755E23.3
RP11-368D24__A.1
RP11-204E4.3
PVRL3-AS1
RP11-231I13.2
linc-GAP43-1
RP11-379P15.1
RP11-554D14.7
linc-GATA5-3
linc-GRPEL1-2
RP11-482E14.1
linc-KIAA1217
RP5-984P4.4
linc-SMC6
CTD-2201G16.1
AC106874.1
XXbac-BPG55C20.7
RP11-722P11.4
linc-FOXF1-1
linc-KHDRBS3-5
CACNA1C-AS3
linc-TNKS-3
RP11-85O21.4
linc-TCTE3-2
linc-HIST3H3
FAM201A
linc-HSPB8
RP11-646E18.4
linc-FAM134A
RP1-223B1.1
linc-OTUD4-1
AC000111.3
linc-PDSS1-3
RP11-557C18.4
linc-CMPK1-3
AC012531.25
RP11-105C19.2
RP11-123K19.1
linc-ATL2-1
FAM138C
AC026471.6
linc-CLCN6
WI2-2373I1.2
RP11-353B9.1
LINC00311
linc-OLIG3-3
RP11-143M1.4
RP5-827L5.2
CTD-2553L13.4
RP11-148B6.2
linc-MAP1LC3B2-8
linc-TRPS1-1
linc-HIST1H2AG-1
RP11-325I22.2
linc-SSTR4-2
RP11-307B6.3
RP11-395L14.4
RP11-867G23.10
AC092667.2
LRRC3-AS1
MIR3150B
linc-RYR2-2
RP11-748L13.6
CTB-167B5.2
linc-LSM14B-1
CTD-2544H17.1
linc-RYBP-3
linc-RTL1-8
RP11-257P3.3
RP11-557C18.3
RP11-397A16.2
RP11-265N7.2
linc-DET1-2
RP11-366M4.13
RP11-416O18.2
RP11-733D4.1
CTC-345K18.2
LINC00882
linc-TMEM215-1

RP11-465B22.8
RP11-168A11.1
TMEM220-AS1
AC019118.2
RP11-164N3.2
RP11-140I16.3
linc-PPDPF-1
RP11-732A21.2
CTD-2210P24.1
linc-HAO2-3
RP11-373E16.4
RP11-380D23.1
linc-CD276
RP11-38J22.6
RP11-396O20.2
linc-CDS2
CTD-3051D23.3
MIR1302-10
RP11-319G9.3
RP11-342C24.8
AC016723.4
linc-SLC35C1-3
CTA-363E6.1
RP11-278H7.4
RP11-973H7.4
RP11-923I11.6
RP11-110A12.2
LINC00592
linc-MLPH-5
RP11-434D9.1
TM4SF1-AS1
linc-TMEM232
linc-LASS6
AC096559.1
linc-HNRNPA2B1-2
RP11-152K4.2
TGFA-IT1
linc-CDCA4-2
CTD-2530H12.8
linc-KCNB2-6
AL109767.1
linc-MASTL
linc-SLC44A3-2
CACNA1C-AS4
RP11-766F14.1
linc-PCSK6-2
linc-TCF7L2-2
RP11-428C19.5
RP11-328K15.1
linc-SHPRH-4
LINC00864
KB-1410C5.2
RP11-465B22.5
linc-SEPP1-2
RP13-57D9.3
linc-C2orf29
CTD-2124B20.3
RP11-111F5.4
RP11-343H19.2
RP11-465L8.1
AC026188.1
RP11-478B9.1
linc-PHB-2
RP11-277L2.3
linc-SESN3-1
linc-DNAJB11-1
AC104654.1
RP11-72L22.1
KB-1047C11.2
AC114803.3
RP11-139K4.1
linc-NTRK2-4
linc-SSTR1-4
CTC-756D1.3
linc-UBE2QL1
RP11-354P11.2
RP5-893G23.1
linc-CXorf49B-2
AC006481.1
linc-ALG2-1

RP5-919F19.5
RP11-923I11.4
linc-F3-4
RP11-598F7.1
RP3-326L13.2
linc-ABCA5-8
CTD-3060P21.1
AC106875.1
AC008073.7
RP11-208N14.4
RP11-353N14.3
RP11-299H22.6
CTD-2194D22.4
linc-GRID1
CTD-2126E3.4
RP11-108P20.4
LINC00868
RP11-963H4.3
RP5-963E22.4
DOCK9-AS2
RP11-672L10.2
RP11-403I13.5
RP11-389K14.3
linc-FAM69C-2
RP11-431I8.1
RP11-443G13.2
RP5-1022J11.2
RP11-560A15.4
RP11-414H23.3
CTD-2034I21.1
CTD-2012M11.3
linc-NKX2-2-5
RP11-472K17.1
linc-PAX9-3
RP11-302I18.3
CTD-2015G9.2
AC009014.3
RP11-292F22.5
RP11-68L18.1
RP11-120K24.3
linc-MARCO
RP11-162I7.1
CTC-304I17.5
RP11-300J18.1
AC005013.5
CTA-929C8.8
RP11-40A13.1
AC006262.10
CTB-35F21.5
RP11-313C15.1
linc-XPO7-4
CTB-114C7.3
LINC00565
linc-CCDC39-1
AC105760.3
linc-RNF170-4
AC007040.5
RP11-3P17.5
RP11-432I5.8
linc-MSI2
linc-PTCHD2-1
RP5-1051D14.1
AP004550.1
LINC00028
CTD-2520I13.1
RP4-773N10.6
WNT5A-AS1
RP11-519G16.3
RP11-214K3.24
RP5-908M14.5
RP11-67L3.2
U1
RP11-91I8.3
AC007099.1
linc-NUDT10
RP11-782C8.1
RP11-710F7.3
RP3-326L13.3
linc-PTPN20B-1
LINC00533

linc-ANKRD1-1
RP1-111D6.3
linc-PTCD3-2
MIR4313
RP3-340N1.5
LINC00323
LEMD1-AS1
linc-RGPD4-8
RP11-59N23.1
AC087393.1
linc-CSGALNACT1-2
AC004540.5
RP11-78O9.1
CTD-2357A8.3
RP11-510C10.3
linc-WRN-1
KCNH1-IT1
PDZRN3-AS1
MIR296
linc-CDHR4
RP5-827L5.1
MIR181A1HG
CTD-2611O12.7
RP11-222A5.1
RP11-243A14.2
LINC00620
LINC00415
RP4-655J12.5
RP11-196E1.3
RP11-369E15.2
RP11-244O19.1
NBPF5P
CTC-523E23.6
RP4-669H2.1
RP11-344B2.3
RP11-423O2.5
RP11-787D18.2
linc-ARHGAP32-2
RP11-182J23.1
RP11-345F18.1
linc-COL4A2-2
linc-ZIC5
RP11-795H16.3
CTC-235G5.3
WWTR1-AS1
CTD-2532K18.2
RP11-18D7.2
RP11-503L23.1
RP5-842K16.2
linc-EVX2-2
RP11-95P13.2
KB-1458E12.1
linc-TMEM18-8
RP11-421L10.1
RP11-79P5.2
RP4-543J13.1
AC106786.1
AL589986.2
linc-ANKRD20A2-2
linc-PRKACG-1
RP11-57H12.2
RP11-131J3.1
RP11-214L19.1
linc-CD300LB
RP11-285E9.6
RP11-3P17.4
RP11-216C10.1
linc-GTPBP4-4
RP11-565N2.1
AC113144.2
linc-FIGNL2-1
SHANK2-AS2
RP11-27G22.1
RP11-26M5.2
linc-PPP4R1-5
linc-GRIP1-3
RP11-78H24.1
CTB-60B18.12
linc-TMEM100-2
linc-ULBP2-2

linc-C20orf46
RP11-991C1.1
RP11-540K16.2
RP11-152O14.1
CTD-2194L12.3
linc-CDKN2C-3
CTB-187M2.2
linc-ST3GAL5-2
RP11-68I3.10
linc-IGSF3
linc-CMPK2-11
CTC-490E21.10
LINC00608
RP11-698F20.3
AC009236.2
linc-GP9
RP11-442J21.1
RP11-1094M14.5
CTD-2140G10.4
RP11-771K4.1
RP11-700H6.1
GDNF-AS1
AC091705.1
RP11-1151B14.2
CTD-2124B20.2
linc-FLI1-4
RP11-168F9.2
linc-MRGPRF-1
RP11-536C10.7
RP5-1096J16.1
BRWD1-IT2
linc-LYZL1-1
RP11-756J15.3
AC093702.1
RP11-25K21.1
RP11-17M24.2
AC093590.1
RP11-547C5.2
CTD-2007H18.1
RP11-355I22.2
linc-SH3RF3
RP11-465K16.1
RP11-433M22.1
AC013269.3
linc-FAM120B-8
TLR8-AS1
SERTAD4-AS1
linc-LRRC6
AE000658.31
linc-RNF32-2
linc-FERMT2
linc-CDH7-2
RP11-538I12.2
RP11-881M11.4
linc-WFDC8
CTD-2587H19.1
RP11-436H11.3
RP11-800A3.7
FOXD2-AS1
CTC-527H23.2
RP11-381K20.5
linc-VWA5B1-2
RP11-85L21.4
CTD-2275D24.1
linc-SPTBN1-2
AC009495.2
RP11-89N17.3
KCNQ1-AS1
linc-IARS2-2
AJ009632.3
linc-HEXDC-3
PLCH1-AS1
RP11-274B18.2
RP11-422P22.1
RP11-131K5.1
linc-DTL-1
AC079612.2
linc-SLC25A38
linc-TMCC1-1
linc-FAM75A6-4

linc-HS6ST1-5
linc-PHACTR2-2
RP11-78C3.1
RP11-758N13.1
AC087499.10
C10orf71-AS1
RP11-99H8.1
AC008074.4
CTD-3088G3.6
RP11-638L3.1
AC017048.4
linc-ZNF32-5
linc-WDR48
RP11-554K11.2
CTD-2251F13.1
RP11-124O11.1
linc-ZNF184
linc-CD9-4
RP11-703M24.5
CTB-114C7.4
linc-STX18-2
RP11-641A6.3
linc-NTSR2-4
CTD-2560E9.3
linc-PROK2-1
RP11-94C24.8
RP11-510C10.2
linc-LOC642587-4
RP11-705O24.1
RP11-118G23.1
linc-LCP1-2
RP11-94B19.5
linc-RREB1-4
AC013268.5
linc-MUC20-9
RP11-343J18.1
RP11-23J9.5
RP1-167G20.2
RP5-952N6.1
linc-WHSC2-1
linc-DNAJC19-5
CTC-430J12.2
KCNJ2-AS1
RP11-61G19.1
linc-EIF2C4-2
RP11-183M13.1
AC009487.5
RP11-120J1.1
RP13-735L24.1
LINC00966
RP11-809H16.2
linc-TSSK3-2
RP11-881M11.2
TTLL10-AS1
linc-ASB2
linc-RXFP2-2
RP11-161D15.3
RP11-513O17.2
AC092168.3
KB-1592A4.14
linc-CELF2-9
RP11-167H9.5
AC084809.2
linc-DUSP4-4
XXbac-BPG254F23.7
linc-FIGNL1
AC098828.2
AC012074.2
TRBV11-2
linc-LRRC38-7
linc-ATG2B-3
AL592528.1
linc-ADARB2-8
AC092168.4
linc-CDYL-3
RP11-218M11.6
RP11-543D5.1
linc-ANKRD20A1-2
RP11-401P9.4
AC079776.1

CTD-2382E5.2
RP5-1121E10.2
linc-SOD1-2
RP11-486O13.2
RP11-423J7.1
RP11-293E1.1
RP13-126C7.1
RP11-883G14.4
RP11-350D17.2
linc-FBXL5-2
LINC00892
CTD-2061E19.6
RP11-507B12.1
linc-TSEN2
AP001471.1
AC104781.1
LINC00269
linc-C6orf192-1
linc-PPAP2B
CTD-2140G10.1
linc-TCTE3-8
RP1-149L1.1
RP11-482M8.1
FAM157C
AC005152.2
RP11-586D19.1
linc-SULF2-5
FAM230B
AC004988.1
linc-KLHL29-9
linc-GAS7-2
RP11-1112C15.2
linc-SNRPB2-1
RP11-525K10.1
RP11-71E19.5
linc-TSPO2-3
RP11-212D3.3
linc-UTP23-2
NAV2-AS5
RP11-157D23.2
LINC00303
RP11-506K6.4
linc-NTN1-2
RP11-776A13.1
RP11-439L18.1
CTC-248O19.1
AC005550.5
CTC-512J12.4
AP002856.6
AC064865.1
RP5-998N21.4
RP11-753D20.3
AC016292.1
RP11-188C12.3
linc-BEND7-1
AC104654.2
RP11-23E19.2
linc-PSMB2
RP11-481G8.2
DGCR5
linc-TMEM98
linc-FRG2C-4
LINC00485
linc-C10orf107-1
linc-CCDC122-2
linc-FAM116B-1
LINC00623
RP11-402L6.1
CTC-297N7.5
RP4-639F20.1
linc-PPAPDC3
RP3-416J7.5
linc-YPEL5-1
CTD-2113L7.1
RP11-25O3.1
RP11-379K17.4
RP11-395B7.4
AP000281.2
RP11-675M1.2
RP11-258O13.1

RP11-770G2.5
linc-HEXB-2
linc-VPS33B-5
RP11-385M4.1
linc-CEBPG
linc-NEURL1B-1
RP11-279F6.2
linc-ZNF507-3
RP11-363D14.1
RP11-497G19.2
FAM157B
CTB-25J19.1
linc-ACTL7A-6
RP11-81F13.1
RP1-74B13.2
RP11-301L8.2
RP11-314O13.1
RP11-212I21.2
RP1-170O19.14
linc-BRI3BP-1
linc-CDH5-2
RP11-495P10.3
RP11-809O17.1
linc-NOX3-5
AC105053.4
PEX5L-AS2
RP11-916L7.1
RP13-539F13.3
RP11-644F5.16
RP11-981G7.3
MTUS2-AS1
AJ003147.8
RP11-1260E13.2
linc-ALK-1
KB-1517D11.4
linc-C17orf102
linc-SUSD4
AC106053.1
linc-SYK-2
AC069363.1
RP11-445F12.2
RP11-1085N6.5
LINC00636
RP13-895J2.7
linc-PFKFB3
RP11-196O2.1
linc-GSDMD-2
RP11-962G15.1
RP11-346L1.2
RP11-44D19.1
linc-C15orf41-1
RP11-680F20.6
RP11-680F20.12
linc-PEX26-1
RP11-441F2.2
RP11-476H24.1
AC002398.5
AC104135.3
linc-PMEPA1-1
GNG12-AS1
linc-EMB-3
linc-MRPS33-1
linc-ARFGEF2-3
RP11-335O13.8
linc-PITRM1-1
RP11-973H7.5
linc-JARID2-1
RP1-127D3.4
RP11-458K10.2
linc-ZDHHC6-2
XXbac-BPG308K3.6
linc-TMEM132E-3
RP11-47I22.2
linc-LYPLAL1
linc-TACC2-4
CTA-254O6.1
AC013480.2
RP11-445P17.3
linc-MAP1LC3B2-7
RP11-334N17.1

RP6-24A23.3
RP11-3P22.2
AC142119.1
AC093627.11
linc-GLDC-2
RP11-264F23.4
RP11-118K6.2
AP003025.2
linc-KIAA1524-1
RP11-260M19.1
linc-MYEOV-1
AC009498.1
RP1-225E12.2
RP11-958F21.3
RP5-857K21.2
AC002454.1
FAM66A
RP11-587H10.2
RP11-22M7.2
RP11-90C4.3
RP11-15I11.3
AC008060.5
CTD-2653M23.3
RP5-1092A11.5
AC020601.1
linc-CTCFL-1
linc-NKX2-2-3
RP1-241P17.1
LINC00574
RP11-65M17.1
RP11-855O10.3
RP11-231E4.2
linc-BET3L
linc-OAF-2
RP11-347D21.4
RP11-945C19.4
RP11-963H4.5
RP11-160E2.19
C20orf203
RP4-728D4.2
linc-PITRM1-4
RP11-718B12.2
RP11-35P15.1
RP11-299G20.3
ADAMTS19-AS1
linc-C13orf28-4
linc-NTM-8
RP11-529H2.1
RP11-69I8.2
linc-ADCY2-5
linc-GPSM1
RP11-419L4.1
AC023481.1
AC068134.6
RP4-712E4.1
linc-TIMP4
RP11-267A15.1
RP11-15K2.2
RP3-462C17.1
RP11-513H8.1
AC114814.4
RP11-473O4.5
AC004920.3
linc-ADCYAP1R1
LINC00609
RP11-216M21.5
RP11-73G16.1
RP11-697E22.3
RP11-958J22.1
RP4-639J15.1
RP11-404P21.1
RP11-626K17.3
linc-GALNT1
RP11-96B5.4
LINC00982
AC023115.2
linc-CDC16-2
CTD-2527I21.4
RP11-45A12.1
RP11-805F19.1

linc-FRG1-4
linc-DTNBP1-2
RP11-680F20.10
EGFLAM-AS4
FTX
linc-TP53BP2-4
AC112229.7
AC007966.1
RP11-273B19.2
CTD-2342N23.3
AC016751.2
RP11-686F15.2
linc-CTDSP2-4
RP11-541G9.1
RP1-149A16.12
RP11-121P10.1
linc-SLC25A46
RP11-616L12.1
linc-ODZ3-6
RP11-402J6.1
RERG-AS1
MIR202
HOXC-AS5
RP11-249C24.11
RP11-107M16.2
RP11-696P8.2
linc-RELL1-4
linc-FOXG1-8
LINC00112
LINC00466
RP11-855A2.5
SIGLECP3
linc-CLDN5-2
AC064875.2
AC062028.1
RP11-424N24.2
linc-TAF5L-3
RP11-544L8__B.4
RP11-482H16.1
RP5-1077I2.3
linc-NRG2-1
linc-MARCH6
linc-LMAN1-1
RP11-118B22.4
linc-ADCY1-3
RP11-341G23.2
SRGAP3-AS1
RP5-1077H22.2
linc-MAF-3
TSPEAR-AS1
RP11-31I22.1
linc-PIP4K2A-1
CTD-2540B15.11
CTC-523E23.15
RP11-146I2.1
RP11-335E6.4
linc-THBS2-3
RP11-44N11.3
RP11-542K23.9
linc-ADRA2C-4
RP11-588P8.1
RP4-794H19.2
RP11-87E22.2
RP11-285C1.2
AC016903.1
linc-AP3S1-3
linc-ZNF133
CTD-2049J23.2
RP11-417J8.3
LINC00943
RP11-231E4.3
RP11-158I3.3
linc-FER-2
AC100802.3
RP1-65J11.1
CTD-2561B21.5
RP5-986I17.2
RP11-358H9.1
linc-AKIRIN1-4
PCAT1

linc-LGALS9B-2
linc-AGAP9-2
RP11-14J7.6
RP11-90J7.4
RP11-647F2.2
RP11-12L8.1
7SK
RP11-19P22.5
linc-BAT2L1
RP11-14I4.3
AC108868.6
linc-KIF26B
linc-CA5A-1
AC012594.1
linc-VANGL1-1
U3
RP11-1038A11.2
RP11-552E20.1
C16orf82
linc-ZNF227
linc-ZMAT4-2
RP11-37E23.5
linc-POLG
RP11-189E14.4
RP11-283G6.3
RP11-1012E15.1
RP5-968J1.1
linc-CLINT1-2
RP11-98J9.1
RP11-462B18.2
RP11-62C7.2
linc-IRX3-1
linc-GUCY1A3-2
RP11-433J8.2
RP11-57H12.3
FAM138D
RP11-315H15.1
RP11-476M19.2
linc-C1orf57-1
linc-BRD3-5
linc-SORCS3-3
AC007464.1
linc-SAA1
linc-C6
AF064858.10
RP11-17M16.2
linc-MFSD9-11
RP11-95I16.2
RP11-587H10.1
linc-C13orf33-1
CTC-436K13.5
RP11-461L13.4
linc-DENND3-1
RP11-424I19.1
RP11-457P14.5
RP3-400B16.3
linc-DMRT2
linc-SEPP1-3
AC009336.24
RP11-849I19.1
RP11-481J2.1
KB-1958F4.2
CTC-436K13.1
XXyac-YX155B6.5
-
linc-RALGPS2-1
linc-RBM15
linc-CTDSP2-3
CTD-2089N3.3
linc-SIM2
AP001063.1
RP4-790G17.7
linc-KLF3-5
RP11-526P5.2
RP13-81N3.2
RP11-560A15.3
LA16c-444G7.2
RP4-655C5.4
CTC-575N7.1
AC058791.2

linc-TMEM132D-2
RP5-856G1.1
RP13-401N8.1
RP11-359E10.1
RP11-242F24.1
RP11-549B18.1
CTA-796E4.4
RP11-159H22.2
RP13-895J2.3
CTD-3032H12.2
RP11-503G7.1
linc-IL28RA-1
RP11-310P5.1
linc-PAQR8-2
RP11-451B8.1
AC009133.21
CTD-3224I3.3
linc-AJAP1-1
PCOLCE-AS1
linc-FBXO32
SPATA41
RP4-614C15.3
NTRK3-AS1
linc-HOXC13-2
RP11-834C11.11
RP4-694A7.4
AC083843.2
RP11-231C18.1
RP11-417F21.2
RP11-109I13.2
linc-REST
RP11-154H17.1
linc-NAV3-1
linc-SGCG-4
LL22NC03-102D1.18
AC105053.3
RP11-13K12.5
linc-CRP
AC002066.1
AC106869.2
LA16c-23H5.4
AC010731.4
RP11-94H18.2
linc-NKX2-2-6
RP11-103H7.1
LIFR-AS1
linc-RUNX2-3
RP11-885N19.6
CTD-2562J17.7
RP11-496D24.2
linc-COG2-2
RP3-441A12.1
RP11-445K13.2
linc-ALOX5-1
WDR86-AS1
linc-COL5A1-4
linc-BTN3A2
linc-IRX3-6
AP001626.2
linc-OSBPL1A-1
RP11-2N1.1
RP11-408H20.3
RP5-1024N4.2
AC079779.5
RP4-655J12.4
RP11-1081L13.4
RP11-547I7.2
RP11-161M6.3
RP1-305B16.2
RP1-206D15.3
linc-ROPN1B-1
linc-ALX4-1
linc-GAS6-2
linc-TNFAIP3-5
linc-E2F3
LINC00551
linc-SULF2-8
AC068286.1
linc-C4orf27-2
RP11-548B3.3

AC009120.10
LINC00622
linc-RNF152
MEF2C-AS1
linc-DACT2-7
AP000345.2
LINC00299
RP1-46F2.3
WWC2-AS2
RP11-485M7.3
RP11-634B22.4
AP000432.2
RP11-435O5.5
linc-FAM72B-1
HAR1B
MYCNOS
linc-ZNFX1
RP11-276H19.1
RP11-403I13.7
CTC-565M22.1
AC002465.2
AC023115.1
RP4-612J11.1
RP11-390F4.3
RP3-416J7.4
linc-PATE4
RP11-1140I5.1
AC018890.6
ATP1A1OS
linc-TRIML2-5
RP11-152L7.1
linc-PARP11-5
RP11-148O21.4
CTD-2207A17.1
linc-SEMA4D-1
CTD-2532D12.5
RP11-1152H14.1
RP11-328M4.2
RP11-340F16.1
linc-CDH20-1
RP1-67A8.3
RP11-618M23.2
RP11-374M1.3
RP11-338O1.2
LINC00502
AL132709.1
RP11-187C18.5
RP5-1004I9.1
AC113608.1
linc-ZNF16
CTD-2089N3.1
RP11-517A5.7
LINC00883
linc-POTEB-1
RP11-388K12.2
RP11-849N15.4
DLGAP1-AS4
linc-WSCD1-1
RP11-132N15.2
linc-PTDSS1-1
RP11-157P23.2
CTD-2298J14.2
AC006042.7
RP11-728K20.2
RP11-1096D5.1
RP11-138I18.2
RP11-742D12.2
CTD-2201E9.2
linc-GPR27-3
CTC-467M3.2
linc-JPH3-1
linc-MLLT4-1
RP11-350O14.18
linc-CNTNAP4
linc-MANEA-8
linc-MMD-2
CTD-2194D22.3
RP5-1121A15.1
RP11-514F8.2
AP000997.2

A1BG-AS1
RP11-531A24.3
RP11-835E18.5
linc-ANKRD1-2
RP11-109E24.2
RP6-91H8.1
RP11-115J16.3
RP11-656E20.5
linc-ACSM5-5
RP11-252M21.6
RP11-66A2.1
linc-PPIAL4A-5
APCDD1L-AS1
CTB-40H15.4
AC034110.1
CTB-32H22.1
RP11-261N11.8
linc-XPO7-2
RP11-782C8.5
linc-NDST3-8
RP11-308D13.3
linc-ELF5
linc-GOLGA7B
RP11-141J13.5
linc-TMEM14A-2
RP4-666F24.3
RP11-161I10.1
LINC00200
linc-VAX1-2
linc-SLC30A5-2
linc-ETV3-1
linc-THBS4-2
CTD-2244C20.2
linc-COL6A6
AC010149.4
RP11-62L18.3
linc-IFT88-2
RP11-686F15.3
ARHGEF7-AS1
RP11-92B11.3
AC005237.4
RP11-27M9.1
linc-SRD5A2-3
linc-RHOXF2
RP4-771M4.3
RP11-27P7.1
linc-ARFGEF2-2
linc-PKN2-1
RP11-114M1.1
CTD-3224K15.3
RP11-439A17.9
RP11-252M21.7
RP1-170O19.21
RP11-672L10.1
RP11-863P13.1
AL022344.7
linc-SFSWAP-2
linc-NSMCE4A-1
RP11-311F12.1
RP11-264F23.3
linc-SLC12A7-1
LINC00686
AC004540.4
CTD-2296D1.2
linc-USP24-1
RP11-923I11.3
RP5-894D12.3
RP11-28H5.2
RP11-89K21.1
linc-LOC647589-1
RP11-459K23.2
linc-BCL2A1-3
LINC00211
linc-SECTM1-3
RP11-846C15.2
RP11-2A4.3
LINC00900
RP11-173L6.1
PCAT7
RP11-93K22.6

AC092580.4
RP11-662M24.2
linc-MRPS33-3
FOXI3
linc-MTRNR2L9-3
RP11-185P18.2
RP11-45I20.1
RP3-393K13.1
RP11-627G18.1
RP11-25K19.1
linc-C11orf82-6
RP11-619A14.3
linc-PPP4R1-3
linc-PAX9-2
RP4-678D15.1
linc-ZNF467-2
AC010987.5
RP11-449O16.2
AC002368.4
FAM226A
RP11-266L9.5
linc-ATP5G3-1
RP11-219B17.1
RP1-62O9.3
linc-FBXL14
linc-LOX-3
linc-FXR1
RP1-228P16.3
RP11-665J16.1
linc-TMEM99-1
CTB-54I1.1
RP11-370F5.4
RP11-867G2.2
RP11-622O11.4
AOAH-IT1
linc-ZNF236-5
linc-COL5A1-2
RP11-25J23.3
FGF14-AS2
RP11-834C11.6
RP11-932O9.4
AP000462.3
linc-RELL1-3
7SK
linc-MAP3K9-2
linc-FAF1-1
RP11-966I7.1
AC007563.3
CTC-344H19.4
CTC-505O3.3
RP11-508N12.2
AC010468.2
EGFLAM-AS3
AC017048.2
RP11-1O10.1
RP11-796E10.1
linc-MUC20-5
WI2-85898F10.1
RP11-275I4.1
HHATL-AS1
AC004231.2
linc-VSNL1-1
AC004485.3
RP3-365O12.2
AC133633.1
RP11-148O21.2
linc-SIP1-1
CTC-537E7.2
linc-CALCB-1
SRD5A2
linc-TBC1D8-1
linc-NR2F2-3
linc-IRX4-1
CTD-2126E3.3
linc-SLC25A15-1
RP11-734K21.4
RP11-43F13.4
RP11-236B18.2
DSCR4-IT1
RP11-274B18.4

TCL6
RP11-503C24.2
linc-USPL1-1
linc-HMX1-3
linc-ABHD3-3
linc-ASAP1-2
linc-ANKRD28
AC005336.1
CTC-518B2.12
linc-DGCR6-1
linc-KCNN2
MIR1302-9
RP5-1063M23.2
RP11-558A11.2
LINC00521
linc-TMC7-2
AP001257.1
RP11-321M21.1
CTA-398F10.1
linc-ITIH2-8
RP11-293F5.1
RP4-672N11.1
linc-FGFR1OP-9
linc-COL18A1-2
linc-HSPH1
RP11-31L22.3
linc-C10orf96
RP11-124D2.3
AC067968.3
RP11-258B16.1
CACNA1C-IT1
FAM41C
AC018464.3
UBE2E2-AS1
RP11-356I2.1
RP11-809H16.5
linc-ATP6AP2-4
RP11-428J1.4
CTD-2308B18.1
linc-DLGAP2-8
linc-C5orf32-2
LINC00619
RP11-5P4.1
linc-TRIM7-1
RP5-1112F19.2
linc-FGD3-1
linc-DKK3-1
linc-OAF-4
RP11-725G5.2
RP1-10C16.1
RP11-650J17.2
linc-EPHA7-5
linc-CALB2-1
linc-UMODL1-2
linc-C1QTNF9B
RP11-75N4.2
linc-ANXA8L2-1
CTD-2544M6.1
RP11-203F8.1
linc-IQCA1-2
linc-TNK2-1
linc-FABP1
linc-CCDC140-9
PGM5-AS1
linc-BTN1A1-1
RP11-960B9.2
linc-RUNX1
RP4-630C24.3
RP11-266N13.2
RP11-719J20.1
RP11-175E9.1
linc-NPVF-1
linc-ZNF25-1
CTC-527H23.3
RP11-154C3.2
linc-C2CD4B-7
linc-TEKT4-1
FAM157A
AC073133.2
LINC00624

RP11-737F9.2
linc-FOXQ1-1
RP11-401P9.6
RP11-179A16.2
linc-NAV2
RP11-503N18.1
RP11-5P4.3
AC011718.3
linc-ADRA2C-1
RP11-481J2.2
RP11-158L12.5
AC068492.1
linc-PELI2
RP11-700H6.4
LL22NC03-23C6.13
RP11-1078H9.1
AC018730.4
RP13-726E6.1
linc-RBM16-1
RP11-556I14.1
linc-C8orf79-1
linc-LRIG1-1
RP11-447M4.1
linc-HNRNPA2B1-1
FAM230C
RP11-68L1.1
RP11-107I14.2
RP11-1105O14.1
linc-TPBG-1
CTB-191K22.5
linc-BMP7
linc-MGAT5B-1
linc-KY-2
RP11-168P13.1
linc-TRIM43B-3
CTD-2047H16.4
RP11-434D2.3
RP11-36D19.8
linc-DUSP22-2
linc-PDCD2-4
KIRREL3-AS2
RP11-474D1.1
FAM225B
RP11-367J7.3
RP11-876N24.5
MYLK-AS1
RP4-529N6.1
RP11-84O12.4
AC112721.2
AC006372.5
RP11-153K11.3
linc-HACE1-2
linc-CD207-1
linc-APOB-3
RP3-380E11.2
linc-DPPA3
RP11-896J10.3
RP11-752L20.5
RP11-17M24.1
CTD-2066L21.3
RP11-822E23.7
linc-LRCH1-4
RP11-134P9.1
RP4-723E3.1
linc-DTNBP1-3
RP11-127O4.3
CTB-164N12.1
RP11-483I13.5
linc-MTDH
linc-TTLL7-3
linc-ABCA5-6
CCDC39-AS1
U91324.1
AC005281.2
RP11-794M8.2
RP11-283I3.1
linc-RXRA-2
linc-TNFAIP3-4
RP11-330A16.1
RP11-44L9.1

linc-MSX1-1
AC093326.2
linc-B4GALNT4-2
linc-C16orf72
MCF2L-AS1
RP4-799P18.3
AF146191.4
RP11-1007I4.1
RP11-458D21.2
RP11-134D3.2
RP11-498J9.2
RP11-403A21.1
CTD-2562J17.2
RP11-71H17.7
linc-MBOAT1-5
CTD-2535L24.3
RP11-328M4.3
CTC-537E7.1
RP11-498P14.3
RP3-325F22.5
RP11-239E10.2
RP11-104L21.2
USP2-AS1
RP4-765H13.1
linc-RAB23-2
RP11-20I20.2
CDH23-AS1
TEX26-AS1
linc-ARFIP1-7
AC016735.1
RP11-855O10.2
RP11-470L19.5
RP11-384P7.5
RP11-355I22.7
RP3-467K16.4
linc-C10orf25
linc-KIAA1712-5
RP11-38C18.3
RP11-410C4.5
RP11-194G10.3
RP11-867G2.8
RP11-619L12.4
RP11-392B6.1
RP11-666A1.5
AC034187.2
linc-WDR26-1
U62631.5
RP1-191J18.66
CERS6-AS1
RP11-675F6.3
AC010745.2
AC138655.4
AC019048.1
RP11-162N7.1
RP11-178F10.2
CTB-43E15.1
RP11-103H7.5
RP1-45C12.1
RP11-214K3.18
RP11-157D23.1
AC000036.4
RP11-369E15.1
RP11-404P21.3
POTEH-AS1
linc-ANKRD10-7
linc-TNRC6C-1
LINC00264
RP11-628E19.2
linc-FMOD-2
linc-COL1A2-1
linc-VSNL1-2
RP11-92C4.3
RP1-292B18.4
LINC00668
RP4-704D23.1
linc-DEFA4
linc-ANKRD10-5
linc-ADRA1D-1
RP11-271F18.4
linc-TMEM132B-1

linc-CRH-1
CTD-2526M8.2
linc-PRR5-3
RP1-60O19.2
RP11-281O15.8
RP11-219J21.1
RP11-797H7.5
linc-ANKRD55-2
RP11-355N15.1
RP11-2N5.1
RP11-168L7.3
RP11-973F15.1
RP13-582L3.4
RP1-161P9.5
RP11-712C7.1
AC091736.10
RP11-7M8.2
RP11-799O21.2
linc-KIAA0427-3
linc-COX10
GS1-410F4.4
RP4-755D9.1
ISM1-AS1
SLC26A4-AS1
linc-COL4A2-3
RP11-292D4.3
CTC-497E21.3
RP11-335O13.7
RP11-38L15.3
RP11-80K21.3
CTA-363E6.5
PAPPA-AS2
RP11-136K14.1
RP11-372E1.6
RP11-124N19.3
CTD-2532K18.1
linc-CYP4B1-1
RP11-531H8.2
RP11-75C9.1
RP11-94B19.2
RP5-1073O3.7
linc-RHD-2
linc-ZC3H12B-3
RP5-1110E20.1
RP11-70J12.1
AC011306.1
linc-MORF4L1-2
CTC-523E23.14
linc-JAG1-2
RP11-79P5.7
RP11-672A2.5
AC138472.6
AC074363.1
RP11-231G15.3
RP11-100F15.1
RP13-210D15.4
linc-IQCG-1
RP11-399H11.2
RP11-133K1.6
RBPMS-AS1
linc-ZIC4-4
AC109309.4
RP11-1070N10.5
linc-LOC642587-6
LA16c-381G6.1
CTB-147C13.1
AC008278.2
CTD-2384B11.2
RP11-439C15.4
LA16c-325D7.1
RP11-495O10.1
linc-EGLN3
RP5-968D22.1
RP11-109M19.1
linc-SHH-5
linc-SULF2-7
linc-EVI2A
linc-SGCG-3
linc-HMX1-5
AC004125.3

LINC00092
linc-HDDC2-3
linc-ITIH2-1
RP11-256I23.3
EMCN-IT2
AC064853.2
linc-LZTS1-3
RP11-534L20.5
RP11-473M14.3
RP11-101P17.10
linc-ATPBD4-2
linc-GDF10-3
linc-RIMBP3C-5
linc-KCNB2-4
FGF12-AS1
RP5-933K21.3
RP11-17A19.2
RP11-465L10.10
RP3-436N22.3
RP11-1018N14.2
linc-FBXW4-1
RP11-70C1.3
RP11-301L7.1
linc-NLRP3-3
RP11-355I22.5
RP11-360A18.2
AC012317.1
AF003625.3
AC007652.1
AC011537.3
C9orf135-AS1
linc-KIAA1524-2
RP11-283C24.1
RP11-355F22.1
linc-ANP32D-1
RP5-1121H13.4
RP11-794P6.3
linc-MT1B
RP11-265N7.1
AC005009.1
RP11-31E13.2
linc-ASB15-1
linc-TMEM170A
linc-MPHOSPH6-2
linc-SUZ12-2
AC125421.1
RP11-516J2.1
-
HOXA11-AS
RP11-637O11.2
linc-MKRN3-3
RP11-174I12.2
linc-CD93-2
RP11-134D3.1
RP11-167J8.3
CTD-2314B22.1
LINC00621
RP13-20L14.1
RP11-359E19.2
RP11-575L7.4
linc-INPP4B-2
linc-LEP
linc-C9orf106-3
RP11-83C7.1
RP11-813F20.2
linc-CDC16-5
linc-MYL2-4
RP11-332H18.4
RP11-205M3.3
RP11-378I13.1
linc-DIO3-4
AC018470.4
RP3-483K16.4
linc-ARFGEF2-13
RP11-398J5.1
linc-ULBP2-1
linc-QRFPR
RP11-895K13.2
NAV2-IT1
RP11-64B16.3

linc-C8orf86-1
linc-NADSYN1-2
linc-N4BP3
AC108463.1
linc-LOC100129636-3
linc-ZMAT4-1
RP11-310P5.2
linc-PUM2-2
RP11-404H14.1
linc-XRCC4-2
linc-C9orf79-1
RP11-343N15.5
AC023115.4
RP11-423C15.3
RP11-881M11.8
RP3-518E13.2
FAM66D
FAM183CP
linc-ARL1-1
linc-EN2-2
RP3-388N13.3
linc-FLI1-6
AC091199.1
RP11-100F15.2
linc-FAM72A-3
AC005592.3
linc-UTP23-1
AC003003.5
RP4-659J6.2
RP11-42I10.1
RP11-766N7.3
AC011718.2
AC079790.2
linc-DHRS7B-1
linc-CLMN-1
linc-CCR8-1
linc-PITRM1-5
CTC-265N9.1
RP11-473E2.4
AC007362.3
RP11-395D3.1
RP5-842K16.1
RP11-27M24.2
RP11-1028N23.4
LINC00605
linc-RPP30-5
RP11-498P14.4
AC109589.1
RP11-358H18.3
RP11-506E9.3
RP11-101O21.1
LINC00595
RP11-143N13.2
RP1-90L6.2
linc-HUS1B-1
AF131215.6
linc-DACT2-5
linc-MCTP2-6
AC005027.3
RP11-384P7.6
RP11-461O7.1
linc-TMEM56-1
RP11-586K2.1
RP11-168K9.1
AC004562.1
RP11-497E19.1
linc-TCEAL2
linc-TBX3-1
RP1-137D17.1
RP11-204M4.2
linc-SYT4-2
CTD-3049M7.1
RP11-66B24.1
TMEM212-AS1
RP11-90C4.1
RP11-550H2.2
linc-IL9-2
RP11-278L15.2
linc-SEP15-1
RP11-214O1.1

LINC00974
RP13-225O21.2
RP11-296L22.8
linc-FCGR1B-7
RP11-490M8.1
AP001627.1
RP11-244K5.8
RP11-307O10.1
ELOVL2-AS1
RP5-943J3.1
linc-TMEM90B-5
FGF12-AS3
RP11-117D22.2
linc-TRRAP
linc-LHX2-2
linc-RDH10
RP11-1008C21.2
RP11-159H10.3
RP11-15G16.1
RP11-396O20.1
CTD-2175A23.1
linc-MBOAT1-4
RP11-483E7.1
RP11-383H17.4
CTC-448D22.1
linc-RBFOX2-1
AC010894.3
CTD-2515H24.4
PTCSC3
RP11-403B2.5
RP11-27G24.1
RP11-434C1.2
RP11-327I22.6
linc-FAM20A-1
RP11-54O7.3
RP11-545H22.1
linc-CREB3L1-1
CTA-481E9.4
CTD-2194D22.2
RP11-44N21.1
HCG16
RP11-173E2.2
RP11-222N13.1
RP4-706G24.1
RP1-251M9.2
EIF2B5-AS1
linc-NANOS1-5
linc-PRKAA2-7
RP11-2O17.2
CTD-3076O17.2
RP11-513G11.3
RP11-248N22.2
CTD-2331C18.5
RP11-157J24.2
RP11-495K9.6
RP11-629G13.1
linc-PBX3-4
NKX2-1-AS1
RP11-449L23.3
-
linc-KLHL31-1
RP11-33A14.1
linc-ENOPH1-1
RP5-942I16.1
RP11-552M11.8
linc-ALDH3B1-2
linc-ID3-1
RP11-282I1.2
AC073626.2
linc-FAM75D4-2
RP11-796I2.1
linc-INSIG2-1
linc-KBTBD12-1
RP11-174G17.2
RP11-90D4.3
RP11-463J17.1
RP11-80K6.2
RP11-536K7.5
RP1-309F20.4
linc-HNRNPA3-1

RP11-300M6.1
AC090952.5
RP1-249F5.3
RP11-65D17.1
RP11-231C18.2
RP11-227D13.1
RP11-1022B3.1
LA16c-306E5.2
RP3-495K2.3
RP11-6E9.4
MKNK1-AS1
linc-OAF-5
RP11-111K18.2
linc-WDR11-2
CTC-436K13.2
RP11-586L23.1
RP11-716O23.2
CTA-929C8.7
RP11-100G15.10
linc-OXNAD1
RP11-554A11.7
linc-CLEC2D-2
linc-JARID2-3
RP11-96B5.3
linc-SGMS2-1
RP11-91K11.2
AC159540.2
AP006216.5
RP11-473E2.3
linc-C20orf71
AC010148.1
RP11-478H13.3
RP11-791G15.2
linc-ANKRD33B-1
linc-CIDEA-1
linc-SDC2
linc-LOC642587-7
RP11-650J17.1
AC006159.3
linc-FAM75A6-3
linc-GPR26-2
CTD-2292M14.1
RP11-234B24.2
linc-MKI67-4
RP11-116N8.4
RP11-472N19.3
linc-TTPA-1
CTD-2201I18.1
RP11-521D12.1
RP11-123K19.2
AC007389.3
RP11-517B11.4
linc-ADRA2C-2
CTD-2561F5.1
CHODL-AS1
RP5-912I13.1
AC003090.1
linc-C17orf108-1
linc-DSC1-3
RP11-218M11.3
linc-TTC7A-3
RP11-1036E20.9
linc-GCNT2-3
linc-GMPS-3
RP1-37N7.4
linc-UMOD
AL353997.3
RP11-268I9.3
AP001628.7
linc-LYPD5-2
RP11-981G7.6
linc-ERG-10
linc-PGK2
RP11-120A1.1
linc-C20orf166
RP11-616M22.1
RP5-945I17.2
SYP-AS1
RP5-1028L10.2
RP11-91I20.2

linc-CHAC2-5
linc-SAMD11-8
RP11-108E14.1
linc-SULF2-2
RP11-360I20.2
AC006372.4
CTD-2297D10.2
linc-NPVF-5
SH3RF3-AS1
RP11-13P5.2
AC144831.1
linc-GLRX5-2
linc-IL1F9
linc-APOB-4
RP11-461C13.1
linc-FAM18B2
linc-ERG-3
RP11-1151B14.4
linc-SLC22A16
linc-SPAST-2
RP11-90K17.2
linc-LMOD2
RP11-393I23.4
AC084809.3
linc-NEURL1B-2
linc-MLLT4-2
linc-MAST4-1
RP11-89K10.1
RP11-862G15.2
linc-KCND3-2
RP11-85G21.1
AC007395.3
RP11-107I14.4
linc-SLC10A2-2
linc-CARD11-7
ZFHX4-AS1
RP11-95P13.1
RP11-626G11.4
RP11-345M22.2
RP11-114L10.2
RP3-462D8.2
RP11-845M18.6
LINC00644
linc-LMAN1-2
CTD-2534I21.9
linc-KHDRBS3-4
RP11-503C24.3
RP11-958J22.3
RP11-553E24.2
RP11-145E17.2
AC007193.10
linc-SULF2-10
RP11-522B15.7
RP4-662A9.2
linc-LRRC38-3
RP5-1164C1.2
linc-ITGB2-4
linc-SPANXB1-2
linc-KCNS2
linc-DNAH6-1
AC092669.6
CNTFR-AS1
RP11-855C21.1
RP11-438C19.2
RP11-399E6.4
RP4-740C4.6
RP11-19D2.2
RP11-850F7.7
RP11-353N4.5
RP1-32B1.4
RP11-64P14.7
RP11-232L2.2
RP1-16A9.1
linc-SERTAD2-4
LINC00683
CTD-2206N4.2
AC004603.4
linc-FRMD4B-2
AC073115.7
CTD-2363C16.2

AC007556.3
linc-SAA2
RP11-335O4.1
RP11-101P17.11
RP11-556E13.1
RP4-620F22.2
linc-C14orf159-2
RP11-165I9.4
RP1-136B1.1
linc-SOD1-1
RP11-383M4.2
RP11-187O7.3
RP11-87E22.1
linc-TES-1
linc-VCAM1
linc-FAM84A-5
linc-ZMYM2
RP11-265P11.2
linc-GARNL3
RP11-58K22.1
RP11-24D15.1
RP11-67L3.4
RP11-463J10.3
RP4-564M11.2
linc-FOXG1-7
RP1-310O13.7
RP11-103H7.2
linc-SSTR4-4
RP11-1029J19.4
linc-TBX20-2
CTD-2358C21.3
RP11-254F19.2
RP11-192P3.4
CTB-83J4.1
linc-UTRN
RP11-490N5.1
linc-PRR5-4
AC090505.4
RP11-108K3.4
linc-RHOXF1-4
linc-GAS1-3
RP11-360I2.1
linc-SH3D19
RP11-35O15.1
RP11-109E10.1
linc-GAS2-1
RP11-434D2.7
RP11-527L4.6
linc-NDUFB4-4
RP11-218I7.2
linc-SCRG1
RP11-263K4.4
RP11-429H9.4
RP11-458D21.1
LINC00572
RP11-472G23.10
LINC00948
linc-HAS2-1
CTD-3010D24.3
RP1-46F2.2
CTC-501O10.1
RP11-89K22.1
CTD-2135D7.4
SPATA42
RP11-563N12.2
CTC-276P9.3
RP11-373N22.3
linc-ANKRD20A1-10
RP11-313I2.11
RP11-272P10.2
AC097532.2
RP11-71E19.1
AC007392.4
RP1-15D23.2
RP5-1125N11.1
linc-PTPRU-2
AL133247.2
CASC8
AC074391.1
linc-ALOX15

RP6-206I17.1
AC092580.3
linc-COL4A2-1
linc-DENND1A
ANO1-AS1
CTC-518B2.10
CTD-2275D24.2
FRMPD4-AS1
linc-FAM38B-3
linc-GCM1
LINC00469
linc-CMPK2-5
RP11-728E14.3
linc-NHLH2-3
RP11-359P5.1
linc-GNAQ-7
AC092159.3
RP11-479J7.1
FGF14-AS1
HCG22
LINC00554
AC004053.1
RP11-257I8.2
RP11-363D24.1
linc-INSIG1-2
linc-CCDC67
CTD-2022H16.1
RP11-384L8.1
linc-NOL6-4
linc-ALG2-3
linc-DLEU7-1
RP11-866E20.3
linc-PDIK1L-2
AC114752.3
RP11-319E16.1
AC020956.3
AC093390.1
linc-FBXL7-1
RP4-735C1.4
linc-NLRP3-1
AC091962.3
RP11-598F7.5
linc-LYRM4
linc-TMED7
RP11-136K7.3
linc-PRR20A-5
RP11-545I10.2
LINC00865
LINC00987
RP11-1078H9.6
RP11-366O17.4
RP11-610P16.1
linc-USP14-2
linc-UMODL1-3
linc-CDK20-1
linc-MCTP2-7
RP11-321E2.4
RP11-379L18.1
RP11-506M13.3
RP11-111E14.1
RP11-660M5.1
LINC00709
RP11-168O16.1
linc-HNRNPA2B1-3
linc-ISLR2-2
RP11-66N11.7
linc-KCNAB2-1
AC068057.1
AC005703.2
linc-TNFRSF19-2
RP11-219A15.2
CTD-2378E21.1
RP11-567G11.1
CTD-2227C6.3
linc-TAGAP-6
RP11-981P6.1
linc-HABP2
linc-C22orf9
RP11-813F20.4
AC067959.1

linc-PBX1
MIR132
AC005162.4
RP11-335I12.2
linc-PECI-2
linc-TTC7A-1
RP11-386M24.3
linc-AGGF1-2
AC073316.1
RP11-354M1.2
RP11-439K3.1
RP11-53B2.5
linc-CLDN24-1
RP5-1185H19.2
linc-ROBO4
RP11-500M8.4
RP11-24M17.4
LINC00841
U66061.31
RP11-430H10.4
RP11-817J15.2
RP11-419C23.1
RP11-498B4.5
RP11-554L12.1
AC092835.2
RP1-41P2.7
linc-CLEC2D-3
RP11-273B19.1
linc-GPM6B
BOK-AS1
linc-ARFGEF2-6
RP11-1094M14.14
CTC-458A3.8
RP1-18D14.7
linc-MTHFSD-4
AC005538.5
CTC-338M12.9
AC017048.3
RP11-350G8.5
RP11-114G22.1
AC091493.2
linc-KHDRBS3-6
CTD-2308B18.3
linc-NSDHL
linc-LEPROTL1-2
RP11-34N19.1
RP11-543B16.3
linc-PCDH8-4
AC024028.1
RP11-298E9.5
RP4-555L14.4
RP11-68L1.2
RP5-1050E16.1
CTB-60B18.10
RP11-634B7.4
EIF2B5-IT1
linc-SLC22A15-1
RP13-463N16.6
AC073834.3
linc-C9orf66-1
XXyac-YX155B6.6
RP11-436F23.1
linc-TMEM90B-6
linc-TLE3-2
linc-CD93-3
RP4-798A10.4
RP11-76G10.1
KB-1639H6.4
linc-FAM110B-6
CTD-3118D11.3
RP11-25L3.3
linc-AIDA
RP11-202G18.1
AC078941.1
RP11-713P17.5
RP11-223A3.1
RP11-264E20.1
linc-AGGF1-1
LINC00633
RP11-378I6.1

RP11-573G6.8
CTA-992D9.6
AC007349.4
CTD-2252P21.1
MIR1302-11
RP5-983L19.2
linc-HFM1-1
RP11-429P3.5
linc-ST3GAL5-3
linc-C20orf79
AC010746.3
linc-SHISA3
linc-CCDC37-2
RP11-761E20.1
RP11-490N5.2
RP11-654A16.3
AP000688.15
linc-VPS8-1
RP11-104J23.1
linc-RFC2-2
linc-TRIM36
RP11-142A23.1
linc-KIAA0564-3
linc-MAN2A1-2
linc-PRELP
RP11-57C13.6
linc-FBXO17
linc-FAM69C-1
AC018890.4
linc-OVOL2
RP11-443N24.3
RP11-214O1.2
linc-MIXL1-1
linc-ROPN1B-3
RP11-465L10.7
RP13-452N2.1
RP11-57P19.1
RP11-530I17.1
linc-TECTB-2
AC093627.7
linc-PDCD2-1
AC003051.1
linc-PRKAA2-2
RP11-61G23.1
DIO3OS
RP11-410C4.4
AC005019.3
LINC00606
RP5-1028L10.1
CTD-3064H18.1
linc-IPO5
RP1-50J22.4
RP11-147L13.8
RP11-305L7.1
RP11-20D14.3
linc-S100B-2
RP5-1013A22.2
RP11-510M2.9
RP11-108M9.1
BX255923.3
RP11-234K19.1
linc-CHD9-6
linc-KCNG1
linc-FGFRL1
linc-TEAD1
RP11-403I13.8
CTD-3051D23.1
CTC-490G23.4
LINC00596
NDFIP2-AS1
LINC00561
LINC00937
linc-EVX1-1
AC012065.4
AC083864.3
RP11-290K4.2
RP5-1022P6.7
RP3-448I9.2
linc-RGMA-7
AL157902.3

RP11-423H2.5
RP11-453A12.1
AC106870.2
AC010132.10
AC004692.4
RP11-909N17.2
RP11-35J23.1
linc-MARCH11-2
TRPC7-AS1
RP11-252P19.2
RP11-388E23.2
RP11-2A4.4
linc-TMEM156
RP5-955M13.4
RP11-213G6.2
AC005256.1
RP11-353N14.1
CTD-2245E15.3
ARHGEF38-IT1
AC099668.5
RP4-555D20.2
AC013269.4
linc-SLC30A5-3
linc-RHOBTB1
linc-SERPINA12
RP11-203P2.2
linc-FAM84B-3
RP11-61G23.2
linc-CDKL5-1
AC010729.1
linc-DHX37-23
linc-NFE2L3-2
RP11-166N6.2
linc-NKIRAS1-1
RP11-458K10.3
RP11-356N1.2
RP11-88L24.4
linc-OR2D2
RP11-624C23.1
AC012456.3
AC005324.7
linc-RGS5-2
CTB-35F21.3
LINC00242
RP11-876N24.4
linc-HRH1
PTPRG-AS1
linc-CALML5-2
linc-TNRC6C-3
linc-GGTLC1-1
linc-HEG1
linc-MEST-1
CTD-2218G20.2
AC012499.1
TRHDE-AS1
RP11-128P17.1
AC023469.2
RP11-426L16.8
CTD-2170G1.2
RP11-298O21.3
RP11-286M16.1
RP11-262I2.2
linc-PHOX2B-2
RP11-599B13.3
RP11-189E14.3
RP11-395L14.3
RP11-42A4.1
FAM138E
RP11-443N24.4
RP11-363E6.4
linc-C10orf57-3
linc-IQCA1-1
CTC-276P9.1
AC013248.2
RP11-168J19.2
AC116609.3
RP11-692C24.2
RP11-461F16.3
RP11-616M22.7
linc-PPDPF-4

AC007405.8
PROX1-AS1
RP4-718J7.4
RP11-77K12.4
linc-KIAA1712-3
RP11-879F14.2
AC008060.8
LINC00684
CTD-3076O17.1
AC003092.2
AF064858.11
AC008154.5
RP11-259O2.1
RP11-129B22.1
ZRANB2-AS1
linc-HNRNPA3-2
linc-ARHGAP28-1
RP11-321A17.5
TSPAN9-IT1
RP11-199F6.4
linc-RGS5-1
RP11-857B24.5
LINC00593
RP11-277J6.3
CTB-50E14.4
linc-MTOR-1
linc-LGALS12
RP11-305P14.1
RP11-799D4.4
RP4-791M13.5
linc-ACBD5-3
linc-CCDC60
RP11-67M1.1
RP5-1030M6.3
JRKL-AS1
linc-NOX3-1
linc-FAM150B-2
RP4-798A10.7
CTD-2313F11.2
RP1-121G13.2
linc-BIRC7-3
RP11-475C16.2
RP11-348F1.2
linc-CCDC99
RP11-53B2.3
linc-ARID1B-2
RP11-405F3.4
AC114814.3
RP4-697K14.3
RP11-421P23.2
linc-C7orf65-11
CTD-2561B21.10
CTD-2034I4.1
RP11-763K15.1
LINC00273
RP11-695J4.2
RP11-483L5.1
linc-JARID2-4
linc-KIAA1712-4
RP11-340E6.1
RP11-575H3.1
RP11-97O12.2
RP11-809C18.1
linc-BSND
AC104389.28
RP11-143J24.1
linc-CDH22
FZD10-AS1
LINC00629
RP11-386M24.6
linc-FAM75A6-1
RP11-140A10.3
CTD-2544H17.2
linc-WRB-5
RP11-386M24.9
linc-PCGF5-1
linc-CYP2J2
RP11-673P17.2
linc-TTLL7-1
RP11-930O11.1

RP11-195M16.3
linc-RPS6KA3-1
AC007092.1
MEG9
RP5-1048B16.1
linc-TYR-5
AC011193.1
linc-IQCA1-6
linc-TGM3-2
CTC-281M20.4
RP11-90P13.1
linc-LHFPL4-2
PCA3
linc-PLEKHG6-2
RP11-81F13.2
RP11-875H7.1
RP11-93B14.4
AC008074.3
RP11-542G1.2
RP11-834C11.3
linc-SETD7-1
KB-1047C11.1
linc-C2orf27A-2
RP11-219E7.2
linc-DEGS2
linc-SESN3-2
RP11-874J12.3
RP11-573G6.6
linc-SLCO3A1-1
RP11-353N14.2
RP6-91H8.2
RP11-13L2.2
CTD-2071N1.1
AC018730.1
RP11-161M6.5
linc-FMOD-3
linc-BCL2A1-1
AC005537.2
RP11-473M20.5
LINC00479
linc-CLIC6
XX-C2158C6.3
RP11-219E7.3
linc-INF2
RP11-43F13.3
RP11-13J10.1
CTC-527H23.4
CTD-2308B18.4
RP11-157I4.4
linc-SEL1L-7
linc-DUSP26-4
RP11-4N23.1
RP11-395B7.2
RP11-17A4.2
RP11-528N21.1
RP11-259K15.2
linc-CCDC140-10
linc-NAP1L2-1
RP11-235C23.5
RP11-945C19.1
CTD-2154B17.1
RP11-297J22.1
RP11-87G24.6
RP11-534L6.5
RP11-744I24.3
linc-CDH11-1
RP11-49G10.3
RP11-227F8.2
linc-KIAA1383-3
KB-1507C5.4
LINC00867
LINC00461
RP11-17L5.4
linc-TYRP1-5
linc-C1orf192
RP1-149A16.3
RP11-752D24.3
RP11-153K16.1
RP11-44H4.1
RP11-783L4.1

Z82214.2
RP11-640N11.2
HAR1A
linc-HUS1B-7
RP11-535C7.1
RP11-23I7.1
AP000997.1
RP11-7O14.1
CTC-497E21.5
RP11-113E21.1
RP11-275O4.3
RP11-794G24.1
RP11-142M10.2
RP5-1158E12.3
RP11-108K3.3
RP11-504A18.1
RP11-291B21.2
SIX3-AS1
MIR4500HG
RP11-122K13.14
linc-C9orf37-4
RP11-229P13.15
CTB-118N6.2
HAO2-IT1
RP11-527D7.1
RP11-366O17.3
ARHGEF7-IT1
RP11-742B18.1
RP11-565N2.2
RP11-789C17.3
RP5-1024N4.4
linc-ZC3H12A-1
KB-1043D8.8
CTA-331P3.1
RP11-522B15.5
RP11-180P8.1
RP11-54O7.17
RP11-10K17.6
linc-ARFGEF2-11
RP11-521O16.1
CTD-2568P8.1
MEIS1-AS3
RP5-1063M23.1
RP11-794M8.1
RP5-865N13.1
AC007003.1
linc-NIPAL2-1
linc-ANO1-1
linc-PDRG1
AC012451.1
linc-ISLR2-3
RP11-408H20.2
RP11-470P21.2
linc-AKR1E2-13
RP11-54O7.16
RP11-672A2.6
linc-PGBD5-2
linc-ARL1-2
RP4-797C5.2
AC092570.2
RP11-146G7.2
RP11-619A14.2
AC005550.4
linc-TMC7-1
RP11-154D17.1
RP11-84D1.1
RP11-495K9.9
RP5-827O9.1
RP5-1173A5.1
LNX1-AS2
RP11-707P17.1
AC133785.1
linc-SRBD1-2
linc-LRPPRC-1
linc-CENPP-9
CTD-2140G10.2
RP11-90E5.1
CTD-2127H9.1
LINC00682
RP11-349I1.2

AC092198.1
RP11-1O2.1
RP11-457M11.5
RP11-760L24.1
RP4-586O15.1
RGMB-AS1
linc-NFE2L3-1
NR2F1-AS1
linc-CARD11-10
RP11-13K12.2
RP11-293E1.2
linc-PHACTR2-1
CTD-3065J16.6
RP11-299L17.3
AC008063.2
RP11-347D21.1
RP11-710C12.1
linc-LYZL2-2
RP11-125D12.1
linc-TSHZ3-1
RP11-1026M7.3
linc-KLHL29-8
RP11-382A20.5
RP11-196I18.4
RP11-421P23.1
linc-MRPL32-2
LINC00207
linc-GJB5
linc-PRKAA2-5
RP11-297M9.2
linc-LOC100288255-1
linc-RABL3-2
RP11-145M4.1
RP1-228P16.4
AC007405.4
linc-GTF2H2-4
AC024619.2
linc-SEC22C-1
RP11-849F2.4
linc-TNRC6C-2
linc-PAFAH2-2
RP11-181B11.1
AC073094.4
linc-GAD1-1
AC003986.6
RP11-550C4.6
RP11-575L7.2
RP11-136I14.2
RP11-71J4.2
AP000282.2
CTD-2125J1.1
linc-ITIH2-5
RP11-629O1.2
linc-ZNF131-6
CTD-2529O21.1
linc-WWC3
RP3-461P17.9
RP11-522B15.3
AC068535.3
PKD1L3
RP11-298J23.8
KCND3-AS1
RP11-815J4.1
AC131056.5
RP11-244H18.1
linc-SCTR-7
CDRT7
linc-HAUS5
RP11-317J10.4
ADIPOQ-AS1
RP11-240L7.4
AC159540.3
RP11-92B11.4
RP11-958F21.1
AF064858.8
linc-KCNA4-2
RP5-1052I5.1
RP11-193P11.3
linc-FOXP2-5
RP11-542G1.1

linc-TRA2A-2
linc-LRIG1-2
linc-C20orf202
RP11-168K9.2
linc-CABLES1
linc-ALDH1B1-2
XXbac-BPG13B8.10
CTD-2554C21.2
AC092669.3
RP11-380B22.1
CTD-2143L24.1
linc-CERK-2
AC007255.8
linc-INTS10
RP11-14P20.1
RP11-660L16.2
RP11-85O21.5
XXbac-BPG308K3.5
AC012506.4
GPR50-AS1
linc-TSN-3
linc-PAXIP1-1
RP11-749H17.2
linc-ANKRA2-5
CTD-2562J17.4
AC008269.2
RP11-470M17.2
RP11-391M7.3
RP11-508P1.2
linc-PET112L-3
RP11-458K10.1
RP11-227F19.1
RP11-1C8.4
linc-GPR39-2
AC092669.2
linc-TSPO2-4
OXCT1-AS1
RP11-78A18.2
AP000797.3
RP11-14O19.1
RP11-98L5.2
RP11-529E10.7
NTM-IT
RP1-153P14.5
RP11-475O23.3
RP3-395M20.9
CTC-508F8.1
linc-ATF7IP2-1
RP11-467I17.1
AC019172.2
RP3-329A5.8
linc-GADD45G-2
RP11-168C9.1
AC004009.3
CTC-378H22.2
RP11-776A13.3
RP11-327I22.5
AC007349.5
RP11-174G6.1
CTC-436P18.3
LINC00960
DYX1C1-CCPG1
RP11-148L24.1
linc-COG6-1
LINC00261
RP11-347D21.2
RP4-704D21.2
RP1-58B11.1
linc-PRSS38
RP5-1065P14.2
linc-ANKRD55-4
RP4-760C5.5
AC092162.1
linc-NKX2-2-4
RP11-432J24.5
AC009492.1
linc-SCAMP1-2
RP11-305P22.9
RP11-569A11.1
RP11-111F5.3

RP11-233G1.4
RP4-773A18.4
RP11-280H21.1
RP11-466P24.7
AC073871.2
linc-POTEB-2
RP11-454C18.1
RP11-553A21.3
CTB-60B18.18
linc-NFIA-4
linc-RAD23B-2
RP11-115J16.1
linc-STXBP6-1
RP11-94B19.7
linc-TREM2
RP11-134O21.1
linc-DAAM1-1
FAM138B
linc-PRKACG-2
linc-C3orf77
RP4-668G5.1
AC007036.6
linc-GPR39-1
linc-FITM2
AC007557.3
CTD-2194D22.1
AC034228.4
CTD-2258A20.5
RP4-791M13.4
RP3-333B15.4
linc-NUDT15-4
linc-BLCAP-2
RP11-439L18.3
RP6-99M1.2
CTD-2240H23.2
RP11-435B5.3
RP11-561O23.7
linc-PAM-1
RP11-53I6.2
RP11-402P6.7
linc-FAM98A-3
linc-UNC13C-4
RP11-710F7.2
linc-HERPUD2-2
RP11-467K18.2
LA16c-306A4.1
linc-THUMPD3
RP11-353N14.4
RP11-366M4.3
RP4-788L20.3
linc-EGR4-3
RP11-3B12.1
LINC00511
RP11-673E1.1
RP11-115F18.1
AC068134.8
RP11-407B7.1
RP11-148B18.1
AC018647.3
RP1-66N13.1
linc-CHMP4C-2
RP11-332M4.1
RP11-175D17.3
linc-RGPD4-6
RP11-543D5.4
AC090505.6
RP11-491F9.1
linc-TGDS-1
RP11-63P12.7
linc-OPCML
AC010974.3
linc-LAMA1-2
CTD-2540B15.12
AC068535.2
linc-PLCL2-2
AC104024.1
AP000439.1
RP5-1018K9.1
RP11-235G24.3
linc-RALYL-2

RP11-134J21.1
linc-ZPBP-1
RP11-134P9.3
AC079145.4
RP11-122C5.1
RP11-424M24.5
LINC00404
linc-RPGRIP1L
SCEL-AS1
CTD-2185K10.1
linc-ITIH2-13
RP11-1029J19.2
CTD-2275D24.3
linc-PCBD1-4
linc-SEP15-7
ARHGEF26-AS1
CTD-2503O16.4
RP11-715J22.2
AC020743.4
linc-HNRNPA1-3
AL592494.5
linc-STK39-3
RP11-400K9.4
linc-VIPR2-2
RP11-299H22.1
RP11-246K15.1
RP11-631F7.1
linc-CDR2-2
MMP12
linc-SMPD1
-
RP11-493K23.4
linc-CX3CR1-3
RP1-91G5.3
RP11-95O2.5
RP4-549F15.1
RP3-470L22.1
RP11-25O10.2
RP11-138E16.1
RP11-34P13.8
linc-EEF1B2-2
linc-ACTL7A-4
RP11-164N3.3
CTD-3064M3.4
linc-FAM113B-1
AC004901.1
TCF7L1-IT1
RP11-449D8.1
RP11-94A24.1
AF015720.3
RP11-699A5.2
RP11-410K21.2
RP11-593F23.1
LL0XNC01-116E7.2
RP11-38L15.8
RP11-296E23.1
linc-MAF-5
CTD-3073N11.9
AP000282.3
linc-PLP1
RP11-521D12.5
linc-SOX6
linc-ADAM2-2
RP4-798C17.6
linc-LRRC8D-3
linc-POLE4
RP4-564O4.1
RP1-12G14.5
linc-ARHGAP20-3
linc-UMODL1-5
linc-CST7-1
linc-DLGAP5-1
RP11-177B4.2
CTC-436K13.6
RP11-70P17.1
linc-MYT1L-4
FAM138A
RP11-252C15.1
RP5-963E22.5
linc-TSKU-1

linc-ACBD5-1
linc-PROM1
RP11-26M5.3
RP5-894D12.4
AC007405.6
linc-ATF3-1
RP11-218M11.1
RP4-791M13.3
linc-EXOSC9-1
AP000289.6
AC066593.1
CTB-138E5.1
linc-SNTG2-7
linc-IYD
linc-SAMD12-1
AC097468.4
RP11-435B5.4
linc-C13orf31-1
linc-FCHSD2-1
CTC-348L5.1
linc-SUMF1-4
RP4-668J24.2
linc-ARHGAP26-5
RP1-140C12.2
linc-ALDH1L1-3
RP11-60I3.5
RP11-43N5.1
linc-ZNF322B-2
RP11-64J4.2
FNDC1-IT1
linc-TTC35-1
RP11-418I22.2
linc-C10orf93-1
RP11-122C5.3
linc-MAP3K8-3
RP11-495P10.5
RP3-523C21.1
LINC00603
linc-RBPJ
RP11-680F8.1
AY269186.1
RP11-24F11.2
linc-KIAA1383-2
ADAMTS9-AS2
linc-SULF2-3
RP11-432J9.6
linc-SPATA8-2
linc-HELT-2
AC002511.1
RP11-456O19.5
RP11-576D8.4
RP11-451G4.1
RP11-53M11.3
linc-MTHFSD-3
RP11-439L8.3
linc-FGFR1OP-8
linc-COX7A2L-1
linc-ADRA1D-3
linc-C16orf78-6
PLCE1-AS1
RP11-675F6.4
CTD-2643K12.3
CSAG2
linc-STX2-7
linc-NID2-4
RP11-731J8.2
RP11-862G15.1
RP11-649A16.1
RP11-923I11.5
RP11-84D1.2
linc-TYRP1-6
RP5-1198O20.4
RP11-434D2.12
RP11-686D22.9
linc-FABP3-2
linc-NPBWR2
BX322557.13
linc-C1orf227-2
RP11-752G15.6
RP11-495P10.2

RP3-430A16.1
linc-HIST1H2AG-3
linc-GLI2-1
linc-TCTE3-7
RP3-525N10.2
linc-RXFP2-1
KCNQ1DN
linc-SPIN1-1
AC141930.2
AC019068.2
LINC00111
RP11-118G23.2
RP11-449P1.1
LINC00475
AC009502.4
AC099344.3
RP13-578N3.3
AC013733.3
linc-CEP110-8
RP11-174G17
linc-GATA6
linc-USP47-2
RP11-588G21.2
RP11-687M24.8
RP11-140I24.1
linc-PUM2-3
AC022431.3
RP11-46C24.3
RP11-150O12.4
GRIFIN
linc-DBT-2
linc-BTC-2
LINC00350
RP11-330M19.1
RP11-134F2.2
RP4-710M3.2
linc-C18orf62-7
linc-OAF-1
RP13-539J13.1
RP11-672A2.4
RP5-1121A15.4
RP11-555F9.2
AC012370.3
RP11-30O15.1
RP11-300A12.2
AC010969.1
linc-EDAR
linc-TRUB2-1
AP005530.2
RP11-677M14.3
RP11-647P12.1
RP11-369F10.2
RP11-88I18.2
RP11-55L4.2
HAND2-AS1
linc-ITIH2-3
RP4-753M9.1
RP11-110H1.8
AC008154.4
RP11-158D2.2
linc-RBFOX2-2
RP11-355E10.1
linc-KLHL13-1
RP1-302D9.1
RP11-329N22.1
TNR-IT1
RP11-95M15.1
linc-BEST3-1
RP11-309M7.1
linc-CXorf49B-1
RP11-503N18.4
RP1-296L11.1
RP11-4C20.4
RP11-555H7.2
linc-SEP15-5
RP11-116O18.1
RP11-46E17.6
linc-OR8D4-1
RP11-381O7.3
TMEM212-IT1

CTD-2582D11.1
RP11-164O23.7
RP11-122M14.3
linc-ARID1B-1
linc-OR7C2-1
linc-DNAJC1
linc-KCNMB1-1
RP11-295M18.2
RP11-449D8.2
CTA-503F6.2
LINC00162
AF001548.6
RP11-646J21.5
RP11-403I13.4
CTD-2560K21.6
RP11-14D22.2
RP4-650F12.2
AC093063.3
linc-MGLL
RP11-568A7.2
linc-HEATR2-5
linc-WDR63
RP11-753D20.4
linc-ZNF536
RP11-146N18.1
linc-CCDC73
RP11-374M1.4
RP11-15M15.2
KB-1083B1.1
RP11-459E5.1
linc-SLC30A4-2
RP11-128B16.3
linc-WDR91
AC079135.1
AC107072.2
CTC-340D7.1
RP11-597D13.9
RP11-1072C15.2
linc-ZEB2-7
LINC00424
RP11-65J3.2
CTD-3229J4.1
AC114812.8
RP5-1121A15.3
RP4-738P15.1
linc-PRDM4-3
CTA-941F9.9
RP11-22P6.3
AP000769.7
RP11-453E2.2
RP11-429A20.4
CTC-573N18.1
RP11-322N21.2
RP11-476D10.1
linc-PTHLH-3
linc-GNE
RP1-251M9.3
RP11-168L7.1
AC011747.7
RP11-118H17.1
RP11-646J21.4
linc-PRICKLE2-1
CTC-218H9.1
linc-OTUD4-3
AQP4-AS1
linc-ZNF236-4
linc-GALNTL4
RP11-145M4.2
RP1-56L9.7
AC002511.2
RP11-484O2.1
linc-MUC2-1
CTD-2050B12.2
linc-LRFN2-1
linc-MAP1LC3B-5
CTD-2313J23.1
AC099552.2
RP11-424M21.1
RP11-135D11.2
linc-ALB-3

linc-ANGEL2-1
AC090939.1
RP11-277J6.2
AC002064.4
SMIM2-IT1
RP11-408O19.5
RP11-388G22.1
linc-TCN2-2
ARMC2-AS1
AC073343.13
RP4-575N6.5
RP4-545C24.1
RP11-262H14.1
RP11-443O13.3
RP1-231P7P.1
linc-SDCBP
RP11-343P9.1
RP5-1043L13.1
RP11-6L6.3
linc-BCL6-5
AC011738.4
linc-ACO1-2
LINC00489
RP11-556G22.2
RP11-849N15.1
RP11-23B15.1
linc-ERG-8
RP11-491F9.5
linc-LMO2-1
RP3-337D23.3
AGAP11
RP11-89F3.2
RP1-117P20.3
RP11-221N13.2
CTC-467M3.3
AC007563.1
RP11-826N14.4
AC007193.6
CTD-2008P7.3
AC011747.6
RP13-439H18.4
RP11-799D4.3
linc-GLTSCR1-2
linc-MC4R-1
RP1-182D15.2
AC011893.3
LINC00544
linc-RAB19
RP11-203H2.2
LINC00927
AC007099.2
RP11-703H8.7
RP11-322F10.2
RP11-5K23.5
linc-LRRIQ1
RP11-406A20.4
RP11-556H2.3
linc-ANKRD20A1-4
LINC00452
linc-PRDM8-3
RP11-139K4.2
RP4-753D10.5
LINC00545
linc-TSHZ1-2
AC096559.2
RP11-334E15.2
linc-GRIK2-1
linc-KIF13B
RP11-87G24.3
linc-UGGT2
RP11-319G9.5
ALDH1L1-AS1
RP11-665G4.1
RP1-35C21.1
linc-HDAC4-2
CTC-467M3.1
linc-GNPDA1
RP11-640M9.1
AC103563.9
linc-FOXB2-3

linc-ZNF404
LINC00920
CTD-2337J16.1
RP1-309F20.3
linc-CHCHD6-1
RP11-280O1.2
RP1-158P9.1
RP11-31I22.4
HOXC-AS2
AF064858.6
AC026904.1
RP11-687M24.4
linc-UBE2U
linc-PARK2-2
EMX2OS
linc-GSX1
RP11-495P10.7
linc-ACTL7A-7
linc-TMEM207-3
linc-SOCS5
RP11-719N22.2
linc-DLEU7-2
RP11-79P5.5
CTD-2023N9.1
linc-TBX3-2
linc-EPHB2
RP11-402G3.5
RP11-397C12.1
RP11-259A24.1
RP11-359G22.2
linc-CDC42EP5-1
AC016912.3
RP11-434I12.4
RP11-127O4.2
linc-H1FNT
linc-GXYLT2-3
CTA-796E4.3
CTA-109P11.1
RP11-116D2.1
AP006748.1
AP001476.4
AP001422.3
linc-BIRC7-2
linc-NPHP1-1
RP11-311H10.4
RP4-647C14.2
RP11-436H11.5
linc-C9orf66-6
linc-BDH1-2
linc-TUBA1A-4
CTD-2385L22.1
linc-ABLIM2
RP11-432J9.3
RP11-495P10.8
LINC00029
RP1-170O19.17
AP005273.1
RP11-332J15.1
RNF219-AS1
linc-TOR1AIP2-3
RP4-705O1.1
RP11-766N7.4
linc-PTGDR
RP11-490O24.2
AC004870.3
RP11-317O24.2
linc-CCDC93-3
CTD-2199O4.1
RP11-899L11.1
RP11-646J21.3
CTB-99A3.1
linc-CBWD3-2
CTC-297N7.7
AC096670.3
VAV3-AS1
CTD-2632K10.1
linc-LCLAT1-2
RP13-379O24.2
RP11-220I1.5
linc-GNB2

RP11-111M22.5
linc-VIM-2
GATA6-AS1
RP11-21G20.3
MLLT4-AS1
RP11-805L22.3
RP11-496I9.1
RP11-406O23.2
RP11-229P13.22
CTD-2515H24.3
linc-IGF1R
RP11-522N14.2
LINC00930
RP11-57C13.3
linc-USPL1-7
linc-CCND1-1
RP11-179A16.1
AC130710.1
CTD-2540B15.10
LINC00163
XXbac-B476C20.17
RP11-198M11.2
linc-SCUBE3
RP11-436H11.6
RP11-705O24.3
RP11-432J24.6
RP11-447M12.2
RP5-978I12.1
RP11-989E6.8
RP11-713M15.2
AC093662.5
linc-CDH4-1
GS1-421I3.4
AC092675.4
LINC00945
RP11-662G23.1
LINC00237
RP11-982M15.7
LINC00612
linc-NOTCH2-3
RP11-167B3.1
AC010649.1
linc-SGCG-2
RP11-503C24.1
RP4-575N6.4
RP11-38C18.2
linc-ZNRF2
LINC00159
RP11-690C23.2
linc-NUBPL-1
AC011518.1
AL121656.5
RP11-89M16.1
linc-METAP1-2
PDXDC2P
linc-C15orf41-5
RP13-137A17.5
AC008753.4
RP11-38J22.3
linc-ZNF311
linc-DYNC1I1-1
CTD-2277K2.1
GS1-24F4.3
RP11-523L1.2
RP11-179B15.6
RP11-417J8.2
LINC00365
AC010145.3
linc-NUDT9-1
linc-TRAPPC1
linc-ARFGEF2-7
AC113331.9
RP11-411H5.1
RP5-907D15.3
RP11-532F6.5
RP11-343H5.6
linc-NLRP1-2
RP11-335E6.3
RP11-136I14.5
RP11-327F22.5

RP11-82L7.4
linc-FAM154B-3
FAM66C
linc-LRRC32-2
RP11-777N19.1
RP11-746B8.1
linc-ARFGEF2-5
linc-FRG2C-6
linc-COG6-2
RP11-677O4.2
CTB-49A3.4
linc-MLPH-4
RP11-339N8.1
linc-CNOT2
AC114788.2
RP11-128L5.1
linc-KCNIP1
RP3-522D1.1
RP11-445P17.8
RP11-574O16.1
linc-TMEM132D-6
AC105339.1
RP11-654G14.1
AC011298.2
linc-C2orf51
linc-PELI1-4
RP11-559N14.5
RP11-283I3.4
RP11-342D14.1
RP11-525K10.3
RP11-244K5.1
CTD-2377O17.1
linc-ZBED5-3
linc-GBP6-4
RP11-109D9.4
RP11-607P23.1
RP11-96O20.2
linc-DYNC1LI1-2
AC009166.5
RP11-482M8.3
RP11-554I8.1
linc-RFTN1
AC104655.3
RP11-338C15.5
RP11-666A8.7
RP11-358H18.2
RP11-523L20.1
RP11-89C3.3
RP11-152L20.3
LINC00858
linc-CDC42BPB-4
RP11-460N16.1
AP002856.4
linc-MYL2-3
KB-1183D5.14
RP11-61I13.3
RP11-284J1.1
RP11-295G24.4
linc-RPS23-2
linc-IQCJ
linc-RBL2
linc-CD9-3
RP11-536O18.2
AC003092.1
RP11-7I15.4
CTB-33O18.2
RP11-300G22.2
CTD-2643I7.1
linc-MAP3K5
RP11-672L10.3
RP11-771D21.2
AP000345.1
RP11-435B5.7
CTD-2139B15.2
linc-LOC642587-5
RP11-528A4.2
linc-CNTN1
RP11-565P22.2
LINC00866
linc-FAM196B

linc-AGBL4-2
linc-SLC25A48-2
RP11-689K5.3
RP13-494C23.1
linc-CSMD2
RP11-108M9.2
RP11-94I2.4
MIR7-3HG
linc-TUBGCP3-4
linc-VAT1L
NR2F2-AS1
AP000290.7
RP11-351J23.1
AC105398.3
CTD-2313N18.5
HCG23
RP11-295G24.5
RP11-498P14.5
AC005265.1
RP11-483K5.3
linc-TUSC1
RP11-804A23.1
RP11-168P8.5
linc-ITGA11
RP5-905N1.2
linc-ERI1-1
RP11-12M5.3
RP11-8P11.3
linc-ZNF25-4
RP11-369E15.4
AC004528.4
linc-TNPO1-2
AC022182.1
linc-ISL1-2
linc-ADRA1D-2
linc-C9orf135-1
AC144835.1
CXorf49
AC004941.3
RP11-360A18.1
RP11-36B6.1
linc-CHRNA4
linc-CHD9-11
linc-GBP5-1
RP11-595B24.1
AC007391.2
linc-NAV1-1
AC092168.2
RP11-448P19.1
RP11-490D19.6
RP11-382B18.4
LINC00676
linc-CPO-1
linc-OLIG2-2
AC092535.3
CTD-2189E23.2
RP11-326N17.1
RP11-1081M5.1
RP11-522B15.4
linc-ATXN10
linc-RNASE1
XXbac-BPG308J9.3
AC106870.1
linc-C6orf89
RP11-500B12.1
linc-TSPAN2
HS6ST2-AS1
linc-MEOX1
RP11-218E20.2
linc-DLGAP2-2
AC000099.1
RP11-884K10.6
linc-POTED-9
linc-CA8-4
linc-LARS2-1
RP11-267D19.1
linc-HARS-1
RP11-456K23.1
RP3-470B24.5
RP11-60A8.1

linc-NUPL1-2
RP11-16K12.1
linc-GLI2-2
RP11-337C18.9
RP11-111I12.1
RP11-215E13.1
linc-MAN2B2-2
linc-WASF3
linc-NOS1AP
linc-GNA13
linc-C14orf101-3
RP4-712E4.2
RP11-62H20.1
LINC00557
linc-NDC80-1
RP11-344F13.1
RP11-554A11.9
linc-CDH11-2
RP13-379L11.1
linc-RUNX1T1-2
RP11-630C16.1
CTD-2571L23.6
RP11-664I21.5
linc-ARFGEF2-8
RP11-31I22.2
RP11-9L18.3
RP11-6I2.4
linc-HAS2-6
RP4-591L5.1
CTD-2116N24.1
RP11-430C7.4
CTC-369A16.2
RP11-13P5.1
LINC00359
LINC00520
RP5-1139B12.4
RP11-307E17.8
linc-QSOX1
linc-NLRP1-1
RP11-344B5.4
FGF13-AS1
CTC-441N14.1
linc-HDAC11-2
RP11-445O3.1
LINC00083
linc-NUBPL-2
RP11-977P2.1
RP1-269M15.3
RP3-522J7.6
linc-SLC48A1-3
linc-CARD11-1
RP11-539I5.1
linc-KCTD15
CTC-756D1.2
RP11-259P1.1
AP003039.3
RP11-779O18.1
linc-GNAL-3
RP11-317J10.2
linc-PRSS3-1
RP11-393K12.2
AF064860.7
linc-STIM2-1
linc-RPP25-1
LINC00160
RP11-345M22.3
RP5-1050E16.2
RP11-598F7.6
linc-P2RY1-2
linc-DPP6-1
RP11-324L3.1
linc-SMOC1
linc-APPL2-1
linc-TP53I11-2
linc-FOXA1-2
AC098872.3
RP11-479O16.1
RP11-90C4.2
RP11-283I3.2
linc-ADAMTS8-2

linc-NCKAP5
AC006262.6
linc-BET1-1
linc-C10orf119-1
AL133493.2
RP11-491F9.6
RP11-218D6.4
linc-CCL23
RP11-510M2.1
RP11-359E8.3
AC007403.3
linc-MBOAT2-2
linc-NPVF-2
linc-TRIP10-6
CACNA1C-AS2
RP11-856M7.4
RP11-650P15.1
RP11-669E14.4
linc-RGS8-3
RP11-128P17.2
THRB-AS1
linc-FAM154B-1
AC124944.5
RP11-305P22.5
AC107079.1
CTD-2240J17.1
linc-ZNF831
RP11-430H10.1
LINC00877
linc-MAP1LC3B-4
ITGB2-AS1
ABO
RP11-605F20.1
RP11-598F7.3
RP11-567N4.3
linc-UNC13C-5
GS1-57L11.1
RP11-344B5.3
RP11-439L8.4
linc-ASAP2-4
RP11-88I18.3
CCDC147-AS1
linc-ADM-1
RP11-401P9.1
CTD-2611O12.8
linc-SMC1B-6
AC026150.8
CTD-2644I21.1
AP006547.3
RP11-34F13.2
RP11-180I4.2
RP11-341G23.3
RP11-179B15.5
RP11-318M2.2
CTD-2288O8.1
ZNF252P-AS1
RP11-167N24.5
linc-CES5A-1
linc-TCF24
linc-RBM45-2
linc-MORF4-3
AC020743.3
linc-ANO1-2
RP11-433J22.3
BX470102.3
linc-NMNAT2-2
LY86-AS1
RP11-292E2.4
LINC00421
RP11-85B7.2
linc-ABHD3-1
AP003900.6
AC096649.3
RP11-1C1.6
SLC6A1-AS1
EPHA1-AS1
RP11-760H22.2
RP11-524D16__A.3
linc-JARID2-2
RP5-837I24.4

LA16c-380H5.3
linc-TMCC3
RP4-607I7.1
AC079354.3
RP11-109P11.1
RP11-352M15.1
linc-C18orf62-6
linc-MRPS33-2
linc-SCNN1G-2
KCNAB1-AS1
RP5-998H6.2
linc-HUS1B-4
RP11-214L13.1
RP11-991C1.2
RP11-536I6.1
RP11-2C7.1
linc-HABP4-3
linc-ZNF521
linc-PCDH8-6
RP11-380D23.2
RP11-543G18.1
RP11-203E8.1
AC147651.3
RP11-609L23.1
RP11-463O9.5
RP11-1018N14.3
RP11-98O2.1
RP11-845M18.7
CTB-49A3.2
RP4-779E11.3
RP13-188A5.1
RP11-449L23.2
linc-PROKR2-4
linc-AKAP9-3
RP11-433J8.1
GS1-115G20.1
RP4-737E23.2
RP5-1185I7.1
RP13-1039J1.2
RP11-879F14.1
linc-RGL4-4
RP4-539M6.14
linc-DDX26B-1
linc-ERG-9
RP11-385F7.1
linc-EN2-1
CTB-187M2.1
linc-RYR3
RP1-65J11.5
RP11-359N5.1
RP11-23E10.2
linc-ARHGAP32-1
linc-POTEB-3
RP11-20G13.3
RP1-290I10.3
RP11-119J18.1
RP1-202O8.2
LL22NC01-116C6.1
RP11-687M24.7
AC106706.1
AC073115.6
RP11-526J3.3
linc-WFDC2-1
RP11-165I9.8
RP11-305A4.3
CTD-2528A14.1
AC007463.2
linc-GADD45G-1
RP11-292F22.7
RP11-449D8.5
AE000658.22
EGFLAM-AS1
RP11-130L8.2
CTB-30L5.1
AC006369.3
linc-ARFGEF2-12
RP11-423O2.7
linc-GLI2-3
linc-C10orf137-3
AC144525.1

AC020743.2
AC018730.3
AC091814.2
linc-GPHN-2
CTD-2263F21.1
PCYT1B-AS1
RP11-495K9.7
linc-SLC35B4-3
RP11-245P10.6
LINC00526
RP11-231N3.1
RP11-798G7.5
AC004791.2
linc-TNP1-3
RP5-1097F14.3
linc-U2AF1-3
linc-KIAA1383-1
RP11-470F18.1
RP11-793A3.2
RP11-533N14.3
AC073283.4
RP11-48F14.1
RP11-91I20.3
linc-IL6-2
AP003774.5
RP11-586D19.2
RP11-875H7.5
linc-APIP-2
CYP4A22-AS1
linc-ANKRD20A1-1
linc-TOX2
RP11-71G12.1
RP11-102F4.3
RP11-85O21.2
RP5-820B18.3
RP11-342K6.4
linc-BRI3BP-2
XXbac-B33L19.3
linc-TREM1-2
LINC00403
linc-PRKCQ-1
RP11-203B7.2
linc-COX7A2L-2
RP11-566H8.3
AC099684.1
linc-LYZL2-1
linc-ITGA6-1
RP3-395M20.7
RP11-13E1.5
RP11-709D24.5
LINC00919
CTD-2195M18.1
linc-DARS
RP11-353N4.4
RP11-532F6.3
RP11-561E1.1
linc-PRKCE-1
AC015922.6
AC006116.21
RP11-116N8.2
linc-LRIT1-1
linc-ARL15
linc-XPO7-1
RP11-52L5.6
AC104984.4
CAMTA1-IT1
RP13-444H2.1
RP11-150O12.5
linc-RPL7
RP11-85L21.6
RP11-61J19.3
TRIM31-AS1
linc-DTNBP1-4
RP11-1058N17.1
linc-AKR1E2-3
linc-ARFGEF2-4
RP11-307C19.3
AC013402.3
RP11-403I13.9
RP11-39M21.2

AP000770.1
linc-HDAC4-1
LINC00391
RP11-227B21.2
RP11-625I7.1
CTD-2004A9.1
KCNAB1-AS2
linc-QPCT
linc-SLC24A3
linc-GPR78-1
linc-ZNF703-1
CTD-2126E3.1
linc-AKR1E2-12
linc-C3orf67-1
RP11-22C11.2
linc-MNAT1
RP1-60N8.1
linc-CPEB2-6
RP11-420N3.2
RP11-92C4.6
AC018495.3
RP4-665J23.1
linc-CNTNAP2-3
linc-PLCE1-1
RP11-60A8.2
AC016582.2
AC008592.8
RP11-12C17.2
RP11-433M22.2
RP11-239L20.6
AC016738.4
linc-KAZ
RP11-93H12.2
AC114752.1
linc-SLC9A8
AL132709.5
RP11-367B6.2
RP11-236J17.3
RP1-122K4.2
LINC00710
RP11-84C10.2
RP5-857K21.1
RP11-227F19.5
AC144521.1
CTD-3187F8.11
RP11-672A2.3
linc-MYCN-2
RP11-589B3.6
AC114730.2
linc-IGSF5-2
KB-1930G5.3
LINC00265
RP11-123O10.4
linc-AGAP1-1
RP4-781K5.6
RP11-304F15.6
XXbac-BPG248L24.13
linc-ASCL1-1
FGF14-IT1
MIR378D2
RP11-798K3.4
AC018804.3
CTD-3187F8.2
AC016910.1
RP5-1044H5.1
RP11-326J18.1
linc-ATAD5-2
linc-OR7C2-2
RP11-708H21.4
RP11-182J1.1
RP4-753D10.3
LINC00426
linc-RAB6C-3
linc-SULF2-4
linc-C9orf170-4
RP11-536O18.1
AC068057.2
RP11-335E6.2
linc-FAM120B-6
linc-PFKP-2

linc-RAD21-1
linc-TTLL7-7
RP11-439C15.5
RP11-763B22.4
CELF2-AS2
RP4-684O24.5
LINC00283
linc-SHOX-3
linc-TMEM90B-2
linc-NTN1-3
RP11-515O17.2
RP11-343N15.1
linc-CXXC1
linc-WRB-4
SNORA59B
linc-SYNGR1-2
AC002076.10
linc-OR11A1
CTD-2311B13.2
RP11-10N16.2
RP11-631F7.2
RP4-792G4.2
linc-KHDRBS3-3
linc-LRRC3B
RP11-313P18.2
linc-MAFB-1
linc-VIPR2-5
LINC00115
RP4-813D12.2
AC006129.4
RP11-44N11.2
linc-ATP6AP2-5
linc-DLK1-3
RP11-567N4.2
RP11-245J24.1
RP11-508M1.7
linc-ACTL7A-5
linc-ABCA1-1
RP11-143M1.2
RP11-132N15.1
RP11-119D9.1
DMD-AS1
RP11-384F7.2
RP11-691G17.1
RP11-5N11.4
AC011747.4
RP11-308N19.4
linc-KCNQ2
linc-TLR3-1
linc-ST6GAL2-8
RP11-55K22.5
AC091878.1
linc-WDR60-3
RP11-352D13.6
RP5-1086L22.1
linc-BTF3-3
RP11-19J5.2
linc-GJA1-1
linc-RAD23B-1
linc-TBPL2-2
MIR137HG
linc-ATP13A4-3
linc-GPC5-1
RP11-144L1.4
AP000344.3
linc-TNFAIP8L1-1
RP11-31L23.3
RP11-32K4.1
LINC00379
linc-TRA2A-3
RP11-49K4.2
CTD-2540L5.6
linc-KIAA0649-2
CTB-33O18.1
linc-FOXP2-6
CTD-2530H12.7
linc-CPXM2-2
RP4-555D20.4
linc-STIL-4
RP11-489D6.2

linc-MZF1-2
RP11-382D12.1
RP11-299D14.2
linc-LYZL1-4
RP11-567C20.2
linc-PAPPA-5
linc-SMAD2
AP001048.4
AC015849.16
RP11-444P10.1
linc-ST6GAL2-9
CACNA2D3-AS1
RP11-471L13.2
AC079922.3
linc-FTH1-2
linc-TRIML2-6
linc-C2orf15
linc-CCR8-2
RP11-94B19.3
RP11-123O10.3
linc-EBF3-2
LINC00890
linc-SLC35B4-2
linc-LAMA1-1
RP11-32F11.2
RP5-1061H20.5
linc-SEZ6-1
RP11-64K12.4
linc-NTN1-4
RP11-406A20.1
linc-C7orf65-6
RP11-544M22.1
RP1-81D8.3
XXbac-B33L19.6
RP11-417J8.1
RP11-567J20.3
linc-ARHGAP11B-3
linc-PARP4
LINC00575
linc-ZNF366-4
GHc-362H12.3
CTD-2200A16.1
RP11-511I2.2
linc-UBR2-1
linc-GPR27-2
CTD-3157E16.1
linc-TMEM206-3
linc-C17orf103
MAST4-AS1
AC067960.1
RP11-84C13.1
RP11-309I15.1
RP11-403N16.2
linc-PARM1-2
LINC00362
RP11-86H7.6
linc-MYCN-3
RP11-188D8.1
RP11-1070N10.4
RP11-272D20.2
linc-BMPER
RP11-774O3.3
AC016751.3
CTD-2545H1.2
AC061961.2
CTD-2014E2.2
linc-ZNF337-10
linc-FBRSL1-2
CTD-2026D20.2
RP11-336A10.5
RP11-907D1.2
linc-DYNC2H1-4
LINC00446
AC013460.1
RP11-848D3.5
RP11-718O11.1
RP1-276N6.2
RP1-290I10.5
linc-CNTN3-1
RP3-513G18.2

linc-DOCK3
RP11-182I10.3
linc-LRCH2-2
LA16c-380A1.1
linc-SLC10A2-3
linc-ZCCHC17-1
LINC00594
AC009505.4
linc-SCAND3-2
linc-NTM-4
RP11-115J16.2
linc-RRP15-1
RP11-813I20.2
RP11-62G11.2
MIR4435-1HG
LINC00690
RP11-86H7.1
CTB-11I22.2
LINC00400
linc-FGD6-3
linc-COTL1-2
linc-PLXNA2-4
linc-ACTL7A-2
CTC-523E23.1
RP11-292D4.2
RP11-146E13.4
RP11-89B16.1
linc-FANCB
AC016994.2
RP1-69M21.2
linc-TMEM132B-2
linc-ADAM29-1
RP11-482D24.3
linc-CERK-4
RP11-981G7.1
AC007271.3
DAQB-12N14.5
linc-C17orf97-3
RP4-718D20.3
CTB-1H10.1
linc-TBX3-4
AC002056.5
RP11-319F12.2
RP11-402P6.11
RP11-203F10.5
CTC-428G20.2
linc-MLPH-3
linc-MSX2-5
linc-SLC15A3
RP11-298O21.7
RP11-119H12.6
LINC00316
AC002511.3
linc-HMGCS1
CTB-3M24.3
RP1-90L6.3
RP11-616M22.5
LINC00524
linc-SEC11A-5
RP11-739N20.2
RP5-968D22.3
RP11-889D3.2
RP1-302D9.2
RP3-454G6.2
AC007349.7
RP11-359J6.1
RP11-111H3.3
CTB-113P19.4
RP11-380L11.4
CTB-1I21.1
RP11-132M7.3
AC008281.1
RP11-699L21.2
RP11-415D17.4
RP11-171I2.1
RP11-799D4.2
RP11-466A17.1
RP11-292D4.1
RP11-718G2.5
Metazoa_SRP

RP11-5P22.3
linc-GUCA2B
RP11-438C19.1
RP1-142L7.8
RP11-125M16.1
linc-OLA1
RP11-180P8.3
RP11-45A16.4
AC073257.2
linc-BTNL8-1
RP11-501J20.5
RP1-191L6.2
RP11-369K17.1
linc-UGT8-1
SLC2A1-AS1
linc-FAT3-1
RP11-48O20.4
RP11-321C24.1
CTB-83J4.2
linc-OR51F2-2
RP11-279N8.1
linc-DYDC1-3
RP11-666A20.4
linc-MFAP4-2
RP11-690J15.1
linc-TMC5
RP3-416H24.1
LINC00518
CTD-2006K23.2
